# Dietary calcium and zinc deficiency risks are decreasing but remain prevalent

**DOI:** 10.1038/srep10974

**Published:** 2015-06-22

**Authors:** Diriba B. Kumssa, Edward J. M. Joy, E. Louise Ander, Michael J. Watts, Scott D. Young, Sue Walker, Martin R. Broadley

**Affiliations:** 1 School of Biosciences, University of Nottingham, Sutton Bonington Campus, Loughborough, LE12 5RD, UK; 2 Centre for Environmental Geochemistry, British Geological Survey, Keyworth, Nottingham, NG12 5GG, UK; 3 Crops For the Future, The University of Nottingham Malaysia Campus, Jalan Broga, 43500 Semenyih, Selangor Darul Ehsan, Malaysia

## Abstract

Globally, more than 800 million people are undernourished while >2 billion people have one or more chronic micronutrient deficiencies (MNDs). More than 6% of global mortality and morbidity burdens are associated with undernourishment and MNDs. Here we show that, in 2011, 3.5 and 1.1 billion people were at risk of calcium (Ca) and zinc (Zn) deficiency respectively due to inadequate dietary supply. The global mean dietary supply of Ca and Zn in 2011 was 684 ± 211 and 16 ± 3 mg *capita*^−1^ d^−1^ (±SD) respectively. Between 1992 and 2011, global risk of deficiency of Ca and Zn decreased from 76 to 51%, and 22 to 16%, respectively. Approximately 90% of those at risk of Ca and Zn deficiency in 2011 were in Africa and Asia. To our knowledge, these are the first global estimates of dietary Ca deficiency risks based on food supply. We conclude that continuing to reduce Ca and Zn deficiency risks through dietary diversification and food and agricultural interventions including fortification, crop breeding and use of micronutrient fertilisers will remain a significant challenge.

Food security is essential to human wellbeing^1^,^2^,^3^,^4^ and a central component of the Millennium Development Goals (MDGs) of the United Nations (UN)^5^. Food security exists when “all people, at all times, have physical, social and economic access to sufficient safe and nutritious food that meets their dietary needs and food preferences for an active and healthy life”^4^ and therefore includes consideration of the macro and micro-nutrient components of the diet. For example, >800 million people are undernourished^6^, and >2 billion people are likely to be at risk of one or more micronutrient deficiencies (MNDs), notably the minerals Ca, iodine (I), iron (Fe), selenium (Se), Zn, and vitamins (e.g. vitamin A)^1^,^3^,^7^,^8^,^9^,^10^. Micronutrients have pivotal roles in human health and MNDs can retard growth and cognitive development, impair immunological functioning and increase the risks of non-communicable diseases including skeletal, cardiovascular and metabolic disorders^10^,^11^. It has been estimated that Fe and Zn deficiency reduce the Gross Domestic Product (GDP) of developing countries by 2–5%^1^. Although the estimated prevalence of inadequate dietary energy supply decreased from 18.7% to 11.3% at global and 23.4% to 13.5% for developing countries between 1990/2 and 2011/14^6^, trends in MNDs for all essential mineral nutrients at various geospatial scales have not been quantified over this period.

Determining the prevalence of MNDs is challenging^12^. For some micronutrients (e.g. Fe, I, Se, Zn), body tissues or urine can be analysed directly for micronutrients and micronutrient-responsive enzymes^9^,^10^,^13^. Where reliable tissue biomarkers are lacking and for larger population sizes, MND risks can be quantified from dietary analyses or surveys incorporating food composition data^14^. However, many countries lack nationally-representative surveys and food composition tables. Data from surveys are also affected by behavioural change and systematic misreporting^15^. The Food and Agriculture Organization (FAO) of the United Nations (UN) uses national food balance sheets (FBSs) as a proxy for food consumption to estimate the global prevalence of undernourishment^16^. The FBSs of the FAO are currently available from 1961–2011^17^ and represent net *per capita* food supply calculated from national production, trade, transport losses, storage, non-food uses, livestock feed, etc., but with no adjustment for household waste or inter- and intra-household variation in access to food^18^. *Per capita* micronutrient supply can be estimated by multiplying the edible portion of each food item (derived from energy values provided by the FAO in kcal *capita*^−1^ d^−1^)^7^ by its micronutrient concentration compiled in food composition tables^19^,^20^. The *per capita* supply of a mineral is then compared to a demographically-weighted requirement threshold for a population, termed the WtdEAR, which represents the daily intake that meets the requirements of half the healthy individuals in that population^21^,^22^. FBS and food composition data were used to estimate a global prevalence of dietary Zn deficiency of 17% in 2003–07^7^, however, the prevalence of dietary Ca deficiency has not previously been reported.

Here, the prevalence of Ca and Zn deficiency risks and their recent temporal trends were determined for 145 countries with populations >1 million. Food supply data (see Supplementary Table S1 online) for 94 food items (see Supplementary Table S2 online) between 1992 and 2011 were integrated with food composition data for Ca and Zn from the United States Department of Agriculture^19^, and phytate data from Tanzania^20^ (see Supplementary Table S3 online). Food composition data were assumed to remain constant over time and location. Intakes of Ca and Zn were based on *per capita* supply adjusted for edible portions using *per capita* energy supply data (see Supplementary Table S4 online). An inter-individual coefficient of variation of 25% was applied to net food supply^7^,^8^,^22^. Demographic (age and gender distributions) data were obtained from the UN Department of Economic and Social Affairs Population Division^23^ (see Supplementary Table S5 online). A single WtdEAR per country was estimated for each element and year from World Health Organization (WHO) vitamin and mineral requirements data^21^ (see Supplementary Table S6 online). Thus, the WtdEAR changes slightly between countries and years due to the difference in the population composition and size of the various countries although the EAR for each age/gender group did not change with time. Those with intakes of Ca and Zn less than the WtdEAR were classed as being at risk of dietary deficiency. This approach assumes (1) minimal correlation between requirement and intake, (2) that the distribution of requirement is symmetrical around the WtdEAR, (3) that the variability in intake is greater than the variability in requirement^8^,^12^. Data were analysed and visualised using Microsoft Excel 2013, Microsoft Access 2013, IBM SPSS Statistics 21, Tableau Software 8.2, and ArcGIS 10.2.1. For regional-, continental- and global-level analyses, national-level data were weighted by the population size of each country.

## Results and discussion

The supplies and deficiency risks of Ca and Zn are presented at various spatial (global, continental, regional and country), and temporal (from 1992 to 2011) scales (means  ± SD unless stated).

### Calcium supply and deficiency risk

**Figure 1:**
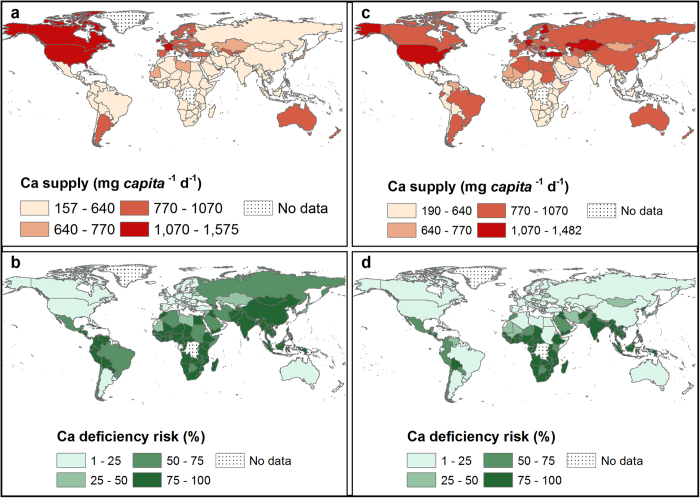
Ca supply (mg *capita*^−1^ d^−1^) and deficiency risk (%). Calcium supply in 1992 (**a**) and 2011 (**c**). Calcium deficiency risk in 1992 (**b**) and 2011 (**d**). Country boundaries were downloaded from the GADM Global Administrative Areas database (http://gadm.org/, Version 2, January 2012). Thematic mapping of Ca supply and deficiency risk was carried out in ArcGIS 10.2.1.

**Figure 2:**
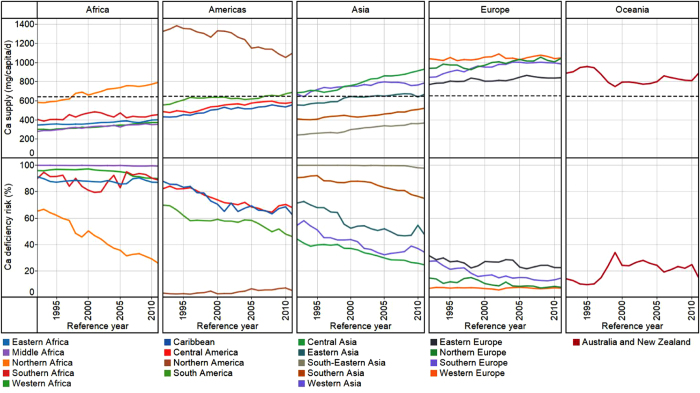
Regional temporal trends in population-weighted mean Ca supply (mg *capita*^−1^ d^−1^) and deficiency risk (%) between 1992 and 2011. The horizontal broken lines represent the average WtdEAR for Ca. Line graphs were drawn using Tableau Software for Desktop version 8.2.

At a global scale, in 2011, Ca supply was 684 ± 211 mg *capita*^−1^ d^−1^ and Ca deficiency risk was 51 ± 32% (3.5 billion people). In 1992, Ca supply was 547 ± 230 mg *capita*^−1^ d^−1^ and Ca deficiency risk was 76 ± 23% (4.1 billion people) (see Supplementary Table S7 online). These reflect an overall increase in global food supply between 1992 and 2011^24^,^25^. In 2011, the WtdEAR for Ca at a global level was 644 ± 3 mg d^−1^. At a continental scale, Ca supply in 2011 was 474 ± 188, 858 ± 234, 639 ± 223, 982 ± 130, and 936 ± 50 mg *capita*^−1^ d^−1^ for Africa, Americas, Asia, Europe, and Oceania respectively (see Supplementary Table S8 online). The mean Ca deficiency risk in 2011 was 80 ± 31, 29 ± 27, 57 ± 36, 11 ± 7 and 11 ± 4% for Africa, Americas, Asia, Europe and Oceania respectively (see Supplementary Table S8 online). Regionally, Ca supply in 2011 ranged from 356 ± 295 mg *capita*^−1^ d^−1^ in South-Eastern Asia to 1126 ± 269 mg *capita*^−1^ d^−1^ in Northern America, representing Ca deficiency risks of 98 ± 3 and 4 ± 1% (Figs. 1 and 2 and see Supplementary Table S9 online). At a country level, Ca supply ranged from 157 mg *capita*^−1^ d^−1^ in Mozambique in 1992 to 1640 mg *capita*^−1^ d^−1^ in the United States of America in 1994 (Supplementary Table S6). In 1992, 86 out of 137 countries had Ca deficiency risk >50%, which decreased to 69 out of 145 countries in 2011 (Fig. 1; and see Supplementary Table S6 online).

To our knowledge, these are the first global estimates of dietary Ca deficiency risks based on data for food supply, food composition, demography and EAR. In a study conducted in Africa by Joy *et al*.^12^, the deficiency risk of Ca was estimated to be 54%, compared to our estimate of 82% in 2009. This disparity can be attributed to the different food composition table used by Joy *et al*.^12^ because similar food supply, demographic and EAR data were used. However, there is general agreement about the existence of high Ca deficiency risk in Africa between the two studies. Interestingly, in a recent analysis of all age- sex- and country-specific groups from 187 countries^26^ (4862 observations), the median Ca intake was 611 (third-quintile range 553-658) mg *capita* d^−1^. These data are based on dietary recall surveys of milk consumption, as a proxy for Ca intake, but are remarkably consistent with our estimates of mean Ca intake based on food supply^26^.

Animal products were the major sources of dietary Ca, but there is variation between regions (see Supplementary Fig. S1 online). For example, in Central, North and South America, Central Asia, Europe, and Oceania, 50–70% of dietary Ca supply was from animal products. Fruits and vegetables contributed 10–40% of dietary Ca supply, but had a higher contribution in some regions (e.g., >50% in Eastern Asia, see Supplementary Fig. S1 online). Cereals contributed little Ca to the diet. The changes in the proportional dietary sources of Ca between 1992 and 2011 was small in most of the regions, reflecting food production and supply systems that are generally consistent over the time period.

Given the high prevalence of Ca deficiency risks, an important question is whether these translate into detrimental health outcomes such as rickets, which can be caused by vitamin D and/or Ca deficiency, or osteoporosis. A review by Pettifor^27^ indicated that nutritional rickets was more prevalent in infants and children in developing countries where the diet is based on cereal staples and the phytate content is high. In mid-latitude countries, where there is adequate sunlight to enable the production of vitamin D in the skin, rickets is mainly attributed to Ca deficiency unless exposure to sun is limited due to religious and cultural reasons^28^. Norhaizan and Nor Faizadatul^29^ reported 2.4 million cases of osteoporosis due to Ca deficiency in Malaysia in 2009, where Ca deficiency is likely to be prevalent based on Ca supply. It is noteworthy that when Ca intakes are low, the efficiency of Ca homeostasis can increase through reduced Ca excretion, thereby enabling populations with low intake of Ca to maintain a healthy skeleton and teeth^30^. Conversely, in populations with high intakes of animal products with high calcium and protein, and high sodium intakes, urinary Ca excretion increases balancing the plasma Ca^30^,^31^. Hence the manifestations of Ca deficiency risk in the form of rickets and osteoporosis may not be as conspicuous as the observed Ca deficiency risks. Clearly, there are many complex issues surrounding dietary Ca deficiency risk, which warrant much further investigation.

### Zinc supply and deficiency risk

**Figure 3:**
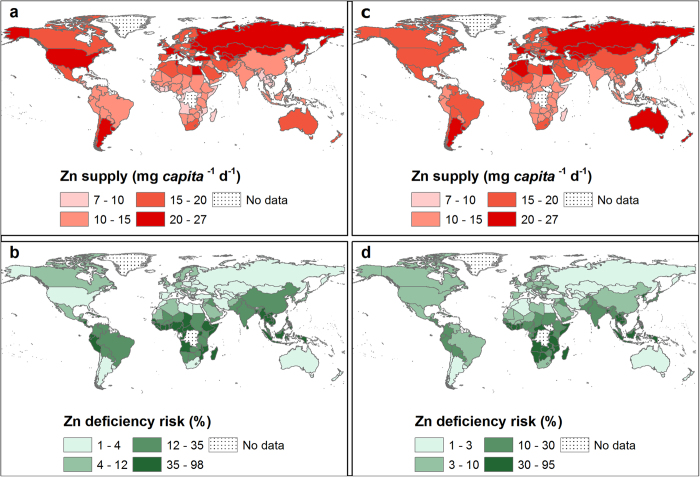
Zn supply (mg *capita*^−1^ d^−1^) and deficiency risk (%). Zinc supply in 1992 (**a**) and 2011 (**c**). Zinc deficiency risk in 1992 (**b**) and 2011 (**d**). Country boundaries were downloaded from the GADM Global Administrative Areas database (http://gadm.org/, Version 2, January 2012). Thematic mapping of Zn supply and deficiency risk was carried out in ArcGIS 10.2.1.

**Figure 4:**
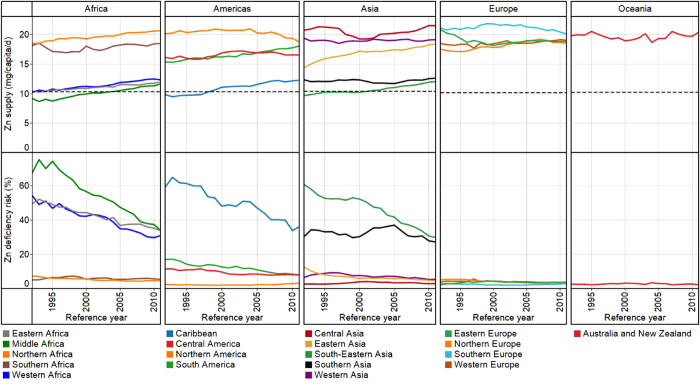
Regional temporal trends in population-weighted mean Zn supply (mg *capita*^−1^ d^−1^) and deficiency risk (%) between 1992 and 2011. The horizontal broken lines represent the average WtdEAR for Zn. Line graphs were drawn using Tableau Software for Desktop version 8.2.

At a global scale, in 2011, Zn supply was 16 ± 3 mg *capita*^−1^ d^−1^ and Zn deficiency risk was 16 ± 14% (1.1 billion people). In 1992, Zn supply was 15 ± 3 mg *capita*^−1^ d^−1^ and Zn deficiency risk was 22 ± 19% (1.2 billion people) (see Supplementary Table S7 online). As seen for Ca, these data reflect an overall increase in global food supply between 1992 and 2011^24^,^25^. In 2011, the WtdEAR for Zn at a global level was 10.3 ± 0.1 mg *capita*^−1^ d^−1^. At a continental scale, Zn supply in 2011 was 14 ± 4, 18 ± 3, 15 ± 4, 19 ± 2, and 20 ± 0 mg *capita*^−1^ d^−1^ for Africa, Americas, Asia, Europe, and Oceania respectively (see Supplementary Table S8 online). The mean Zn deficiency risk in 2011 was 25 ± 20%, 7 ± 10%, 17 ± 14%, 3 ± 2%, and 2 ± 0% for Africa, Americas, Asia, Europe and Oceania respectively. Regionally, Zn supply in 2011 ranged from 12 ± 6 mg *capita*^−1^ d^−1^ in Caribbean to 22 ± 7 mg *capita*^−1^d^−1^ in Central Asia, Zn deficiency risks ranged from 2 ± 0% in Australia and New Zealand to 36 ± 38% in Caribbean (Figs. 3 and 4 and see Supplementary Table S9 online). At country level, Zn supply ranged from 6 mg *capita*^−1^ d^−1^ in Rwanda in 1998 to 28 mg *capita*^−1^ d^−1^ in Uruguay in 1994, representing Zn deficiency risks of ~100% and 1% respectively (see Supplementary Table S6 online). In 1992, 48 out of 137 countries had Zn deficiency risk >25%, which decreased to 39 out of 145 countries in 2011 (Fig. 3 and see Supplementary Table S6 online).

Our estimate of Zn deficiency risk (20%) is comparable to Wessells *et al*.^7^,^32^ global estimate of 17.3% in 2003-07 where they accounted for the impact of various factors to Zn absorption in their model. Joy *et al*.^12^ estimated the Zn deficiency risk for Africa in 2009 to be 40% as compared to our estimate of 26%. This discrepancy, as for Ca, is due to the different food composition data used.

**Figure 5:**
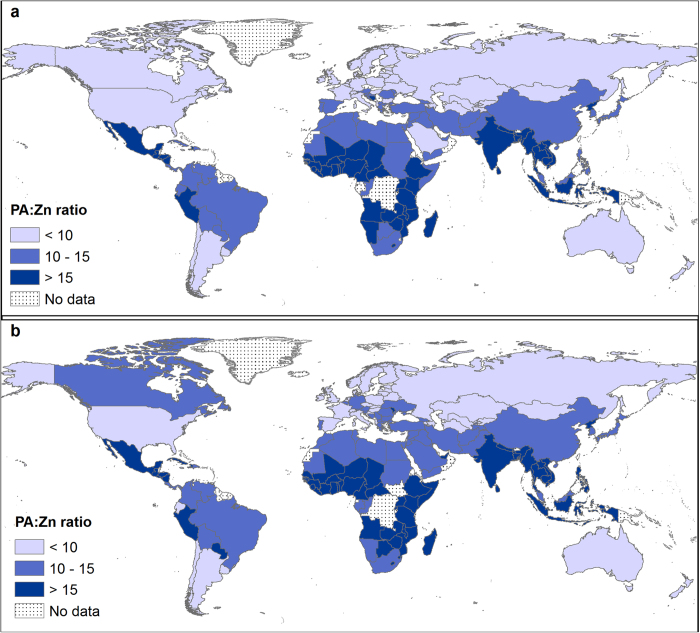
Phytate:Zn (PA:Zn) molar ratio in 1992 **(a) and 2011 (b), based on *per capita* PA and Zn supplies**. Country boundaries were downloaded from the GADM Global Administrative Areas database (http://gadm.org/, Version 2, January 2012). Thematic mapping of PA:Zn ratio was carried out in ArcGIS 10.2.1.

Dietary sources of Zn varied across regions. Animal products were the major sources of Zn (>40%) in North Africa; North and South America; North, South and West Europe; and Australia and New Zealand. In other regions, the major source of Zn was cereals (see Supplementary Fig. S1 online). The prevalence of deficiency risk and utilization of Zn is influenced by the quantity of Zn intake, and the overall dietary composition that either promotes or antagonises Zn bioavailability^7^,^32^,^33^. Among the anti-nutrient factors that reduce the absorption of Zn is the phytate (myoinositol hexakisphosphate). Phytate chelates with mono and divalent cations such as Zn. Due to the absence of intestinal digestive phytases, humans cannot digest phytate and hence chelated Zn cannot be utilized^34^,^35^. In 2011, dietary phytate supply ranged from 997 in Ecuador to 4179 mg *capita*^−1^ d^−1^ in Niger and 25-80% of the Zn supply in all regions originated from cereals which have high phytate content (see Supplementary Fig. S1, and Supplementary Table S6 online). Phytate:Zn (PA:Zn) ratios ranged between 5 and 27 (Fig. 5). Phytate:Zn ratio for most countries in this study were >15, the critical threshold level beyond which Zn absorption is considered to be inhibited^35^,^36^,^37^,^38^. This will compound the Zn deficiency risk of the population due to lower bioavailability^13^,^33^. Populations relying on plant-sourced foods with low Ca and Zn are also vulnerable to toxic metals that have similar properties to Ca and Zn, for example cadmium (Cd)^39^. Deficiencies in Ca and Zn may lead to organ accumulation and retention of dietary Cd^40^.

### *Per capita* income, Ca and Zn supplies, and deficiency risks

Calcium and Zn supplies were highly positively correlated with *per capita* Gross National Income based on purchasing power parity (GNI-PPP), across all countries and years (Table 1). Countries with lower GNI-PPP had higher Ca and Zn deficiency risks than those with high *per capita* GNI-PPP (see Supplementary Table S10 online), with strong positive correlations observed between GNI-PPP and Ca and Zn supplies (Table 1). These relationships imply that those living in countries with higher GNI-PPP purchase or produce more animal products, fruits, legumes and vegetables than those with lower GNI-PPP, which is consistent with observations from household surveys^14^. The green revolution of the 1960s was supported by high rates of investment in research on staple foods (mainly wheat, rice and maize)^41^,^42^. Lower-yielding cereal and legume landraces that were potentially better sources of essential micronutrients (e.g., Fe, Zn, Ca, and potassium) have generally been replaced with higher yielding cereal varieties^43^,^44^. This general decline in agro-biodiversity might therefore have compounded MNDs^42^.

### Calcium and Zn deficiency risks and Millennium Development Goal 1

At a global scale, if the Millennium Development Goal 1 (MDG1)^5^ target to halve hunger by 2015 is framed in terms of Ca deficiency risks, the 76% risk observed in 1992 should have decreased to 46% in 2011. However, the Ca deficiency risk was 51% in 2011. For Zn, the 22% risk in 1992 should have decreased to 13% in 2011 but was 16%. National level reductions in Ca and Zn deficiency risks were uneven. By 2011, 101 and 86 countries were not on target to halve Ca and Zn deficiency risks, respectively, by 2015 (see Supplementary Table S11 online). Out of these; 34, 19, 26, 20, and 2 countries fell short of halving Ca deficiency risks and 22, 14, 22, 26, and 2 countries fell short of halving Zn deficiency risks, from Africa, Americas, Asia, Europe and Oceania respectively.

## Conclusion and limitations

In 2011, 3.5 and 1.1 billion people were at risk of Ca and Zn deficiency respectively. To our knowledge, these are the first global estimates of dietary Ca deficiency risks based on food supply. Our estimates of Zn deficiency risks are consistent with recent studies^7^,^32^. Supplies and deficiency risks of Ca and Zn differed geographically with countries in Asia and Africa accounting for >90% of the estimated Ca and Zn deficiency risks. At higher spatial resolution, differences in Ca and Zn supplies and deficiency risks between individual countries become pronounced.

This study uses data from several sources, each of which has its limitations. For example, food supply data are aggregated at national level with a fixed coefficient of variation in intake. Such data do not capture community and household level socio-economic factors which systematically affect intake, nor food wastage at a household level, nor seasonal variation in the type and quantity of food supply. All of these factors will, of course, affect deficiency risks for Ca and Zn. In addition, the rise in awareness of the roles that mineral micronutrients play in human health has led to increased research and development to biofortify food crops with micronutrients^45^,^46^,^47^,^48^,^49^,^50^,^51^,^52^,^53^. This may lead to change in the food composition in addition to future increases in food supply due to improvement in crop yields. However, the impact of changes in food composition on the reduction of deficiency risk of Ca and Zn due to fortification either through agronomic interventions or breeding could not be captured by this study mainly due to a lack of temporal, spatial, and varietal food composition data. Similarly, the effect of Ca fortification in cereals and milk is not addressed in this study due to lack of data. Therefore, whilst the information presented here can inform policy in a general sense, such analyses would become more useful at higher geospatial resolution (for example, at subnational levels). Further research could include assessing the health, and nutritional status of various age/gender/socioeconomic groups through biochemical, clinical and anthropometric measurements in countries with high deficiency risks of Ca and Zn^21^. Development of localized food composition tables and updating existing ones with information on new/under-utilised food crop varieties is crucial to improve the accuracy of estimating deficiency risks of Ca and Zn.

Possible solutions to Ca and Zn deficiency include: supplementation, direct fortification^9^,^50^, fertiliser application^51^,^52^,^53^ and plant breeding^46^. Supplementation is crucial in situations that require short term actions with high impact, for example, for pregnant women. However, supplementation and direct fortification of foodstuffs with Ca and Zn may not be economically feasible and cannot reach the majority of the population in developing countries who produce their own food^42^. Under such circumstances, agronomic intervention by applying Zn fertiliser either through foliar routes or to the soil can help increase the composition of Zn in food crops^53^. Similarly, breeding interventions through developing food crop varieties with the ability to absorb and accumulate more Zn and Ca from the soil and translocate them to the edible parts, or with lower phytate composition^54^, can potentially be pursued to increase the bioavailability of these nutrients. In addition, the production and provision of affordable animal products, and education on how to reduce the impact of phytate in plant source foods on Zn bioavailability (for example, soaking, germination, and fermentation) are essential^22^. We conclude that continuing to reduce Ca and Zn deficiency risks through dietary diversification^42^ and food and agricultural interventions including fortification^9^, crop breeding^46^ and use of micronutrient fertilisers^49^,^53^ will remain a significant challenge in the post-Millennium Development Goals (MDGs) era.

## Materials and Methods

Food supply, food composition, Estimated Average Requirements (EARs) for Ca and Zn, and demographic data were compiled to assess global dietary Ca and Zn supplies and deficiency risks between 1992 and 2011. A total of 145 countries with population >1 million were included in this study. The EAR “cut-point” (EAR-CP) method was used to assess the prevalence of Ca and Zn deficiency risks.

### Data sources

The four major datasets required for this research were food supply, food composition, EARs for Ca and Zn, and demographic data for each country. Food supply data from 1992 to 2011 were obtained from FAOSTAT^17^. Food composition data for Ca and Zn were obtained from the United States Department of Agriculture National Nutrient Database for Standard Reference 26 (USDA SR26) which was released in 2013^19^, and the phytate composition of foods were obtained from the Tanzanian food composition table (TFC)^20^. The EAR for Ca and Zn were obtained from the FAO/WHO Human Vitamin and Mineral Requirements^21^. Demographic data were obtained from the United Nations, Department of Economic and Social Affairs Population Division, Population Estimates and Projection web page^23^. These data were compiled and integrated in to MS Access relational database management system to assess global dietary Ca and Zn supplies and deficiency risks.

### Calcium and zinc supply

The 94 food items from FAOSTAT^17^ food supply data, reported in g *capita*^−1^ d^−1^, were matched^55^ with the food commodities in the nutrient composition data in the USDA SR26 food composition database (see Supplementary Table S2 online). Nutrient composition data were assumed to not change between 1992 and 2011. *Per capita* Ca and Zn supply was estimated by multiplying the edible portion of each food item (derived from energy values provided by the FAO in kcal *capita*^−1^ d^−1^)^7^,^18^. Calcium and Zn supply from each food commodity was summed to obtain the *per capita* nutrient supply (PCNS) per day for every reference year and country.

### Calcium and zinc intakes and requirements

Calcium and Zn intakes were estimated to be the *per capita* Ca and Zn supply with a coefficient of variation of 25%^7^,^32^,^56^. The EAR was estimated and available for a given age and gender groups^21^. As supply data were available for the whole population at national level, the EAR was converted to single WtdEAR (Equation 1) based on the population size in each age and gender group for each country and year^23^. The EAR for a given age/gender group was assumed to remain unchanged while the WtdEAR varied with the population composition and size. The WtdEAR for Ca and Zn is a *per capita* intake level that fulfils the Ca and Zn needs of half of the apparently healthy individuals of the population in a given country in a specific year.

### Estimated average requirement “cut-point” (EAR-CP)

Historical Ca and Zn deficiency risks were assessed using the EAR-CP as described and used by Carriquiry^57^, Joy *et al*.^55^ and Wuehler *et al*.^8^. The EAR-CP method provides an estimate of the number of people in a given country and year with intakes of Ca and Zn below the WtdEAR, which is termed as the deficiency risk in this paper. The EAR-CP method has been applied with the following underlying assumptions: little correlation between requirement and intake, the distribution of requirement is symmetrical around the EAR, and variability in intake is greater than the variability in requirement^22^,^57^,^58^.

### Calculation of molar ratio of phytate:Zn

The dietary PA composition of the food items was obtained from the TFC^20^ and the PA supply for each country across the years was estimated by applying similar methods as for Ca and Zn supplies. Thence, the daily molar ratio of PA:Zn was calculated by dividing the molar intake of PA (molecular weight = 660 g mol^−1^) by the molar intake of Zn (molecular weight = 65.4 g mol^−1^).

### Data analyses and visualisation

Descriptive statistics calculations were conducted in MS Excel. Visualisations were carried out in Tableau Software for desktop version 8.2, and ArcGIS 10.2.1. Correlation analyses was carried out using IBM SPSS Statistics version 21. The EAR, and aggregations of results (mean and standard deviations) at global, regional, and continental levels were weighted by the population sizes of the member countries (Equation 1) and (Equation 2).

**Equation (1):** Derivation of the weighted estimated average requirement (WtdEAR) for Ca and Zn.


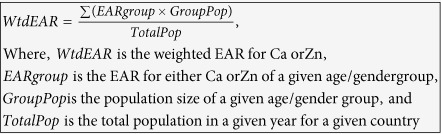


**Equation (2):** Aggregation of mean (a) and standard deviation (b) of supply, WtdEAR, and deficiency risk of Ca at regional level as an example.

a) Derivation of the mean of aggregated information at regional level.


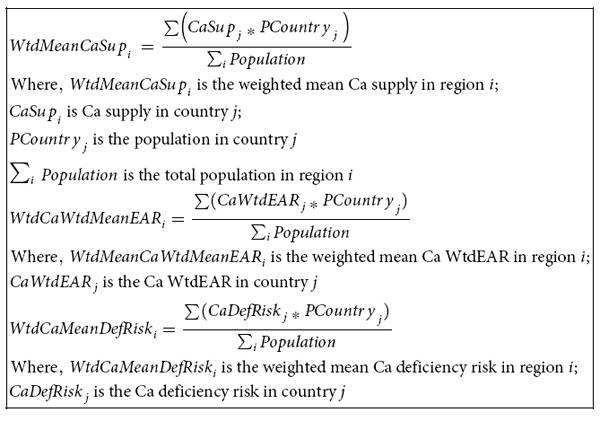


b) Derivation of the standard deviation of aggregated information at regional level.


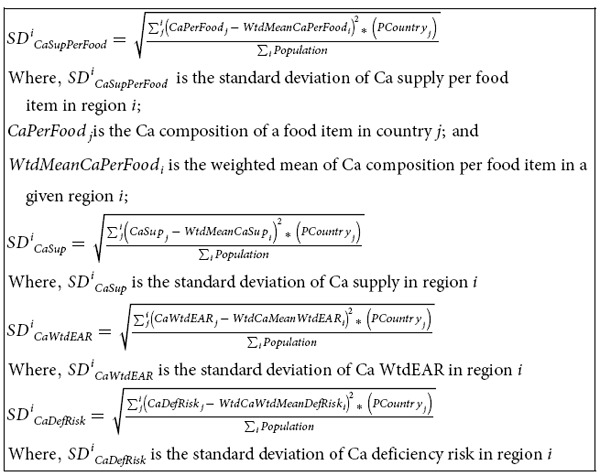


## Additional Information

**How to cite this article**: Kumssa, D. B. *et al*. Dietary calcium and zinc deficiency risks are decreasing but remain prevalent. *Sci. Rep*. **5**, 10974; doi: 10.1038/srep10974 (2015).

## Supplementary Material

Supplementary Information

## Figures and Tables

**Table 1:** Relationship between Ca, Zn and energy supply, phytate:Zn molar ratio, and GNI-PPP.

	Ca supply	Zn supply	Phytate: Zn	GNI-PPP
Zn supply	0.817**			
Phytate:Zn	−0.775**	−0.714**		
GNI-PPP	0.755**	0.604**	−0.682**	
Energy supply	0.832**	0.770**	−0.641**	0.825**

Based on 2655 data points from 1992-2011 (see Supplementary Table S10 online).

^**^Spearman’s Rank Correlation is significant at *P* ≤ 0.01. GNI-PPP is gross national income converted to international dollars using purchasing power parity rates. An international dollar has the same purchasing power as a US Dollar in the US.
