# Supplementary material for: Dietary calcium and zinc deficiency risks are decreasing but remain prevalent
Source: Sci Rep. 2015 Jun 22;5:10974. doi: 10.1038/srep10974 (PMC4476434; doi:10.1038/srep10974)
Supplement: Supplementary Information [file srep10974-s1.pdf]

Dietary calcium and zinc deficiency risks are decreasing but remain prevalent

Diriba B. Kumssa[1,2,3], Edward J. M. Joy[1,2], E. Louise Ander[2], Michael J. Watts[2], Scott D. Young[1], Sue Walker[3] & Martin R. Broadley[1]

[1]School of Biosciences, University of Nottingham, Sutton Bonington Campus, Loughborough, LE12 5RD, UK, [2]Centre for Environmental Geochemistry, British Geological Survey, Keyworth, Nottingham, NG12 5GG, UK, [3]Crops For the Future, The University of Nottingham Malaysia Campus, Jalan Broga, 43500 Semenyih, Selangor Darul Ehsan, Malaysia.

Correspondence and requests for materials should be addressed to M.R.B. (martin.broadley@nottingham.ac.uk)

Supplementary figures

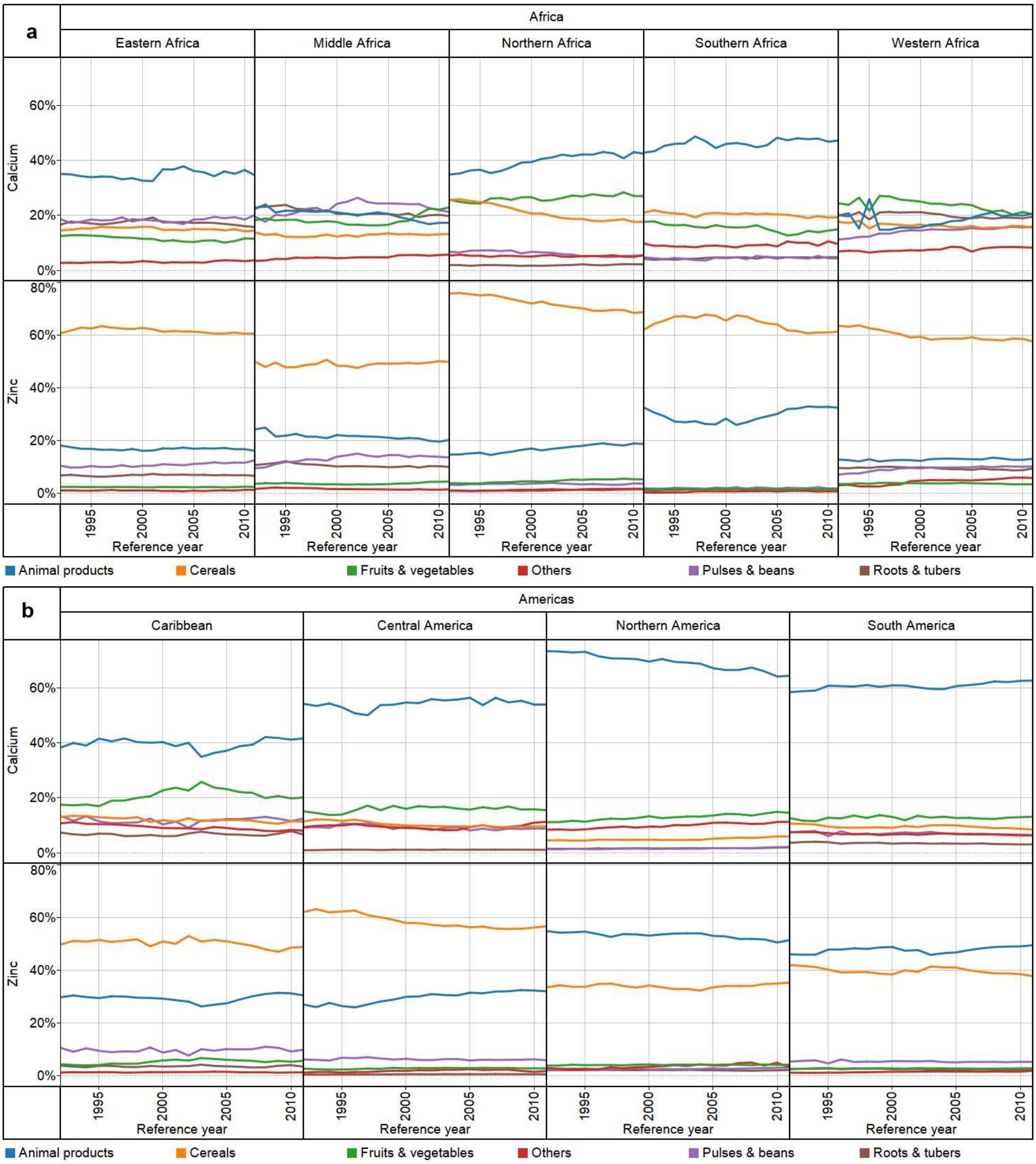

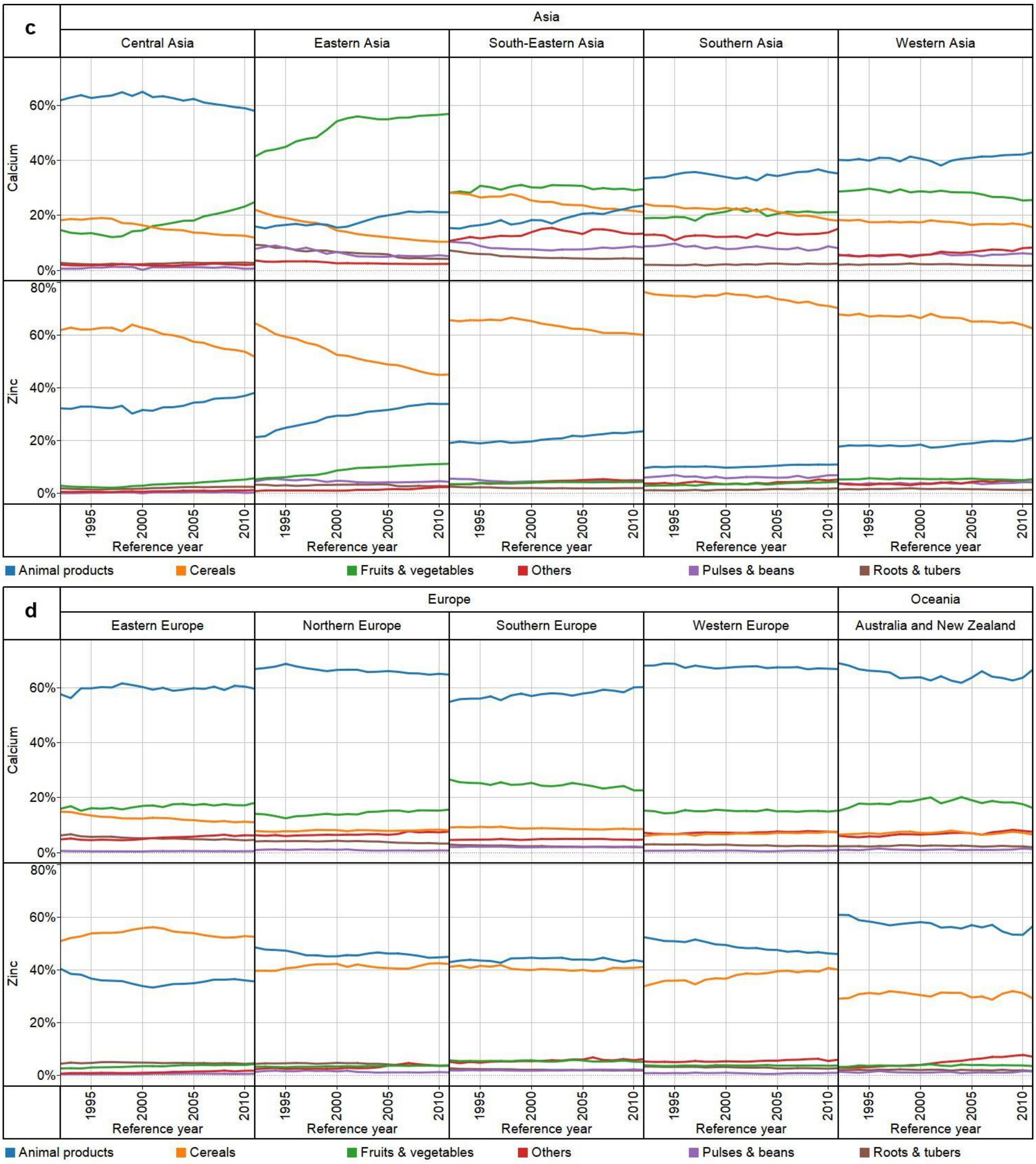

Supplementary Figure S1: Trends in the percentage contribution of food groups to Ca and Zn supply between 1992 and 2011. Africa (a), Americas (b), Asia (c), and Europe and Oceania (d). The line graphs were drawn using Tableau Software for Desktop version 8.2.

Dietary calcium and zinc deficiency risks are decreasing but remain prevalent

Diriba B. Kumssa[1,2,3], Edward J. M. Joy[1,2], E. Louise Ander[2], Michael J. Watts[2], Scott D. Young[1], Sue Walker[3] & Martin R. Broadley[1]

[1]School of Biosciences, University of Nottingham, Sutton Bonington Campus, Loughborough, LE12 5RD, UK, [2]Centre for Environmental Geochemistry, British Geological Survey, Keyworth, Nottingham, NG12 5GG, UK, [3]Crops For the Future, The University of Nottingham Malaysia Campus, Jalan Broga, 43500 Semenyih, Selangor Darul Ehsan, Malaysia.

Correspondence and requests for materials should be addressed to M.R.B. (martin.broadley@nottingham.ac.uk)

Supplementary information

Supplementary Figure S1: Trends in the percentage contribution of food groups to Ca and Zn supply between 1992 and 2011. Africa (a), Americas (b), Asia (c), and Europe and Oceania (d). The line graphs were drawn using Tableau Software for Desktop version 8.2.

Supplementary Table S1: Food supply by FBS food item, reference year, and country.

Supplementary Table S2: Food Balance Sheet (FBS) food items obtained from FAOSTAT.

Supplementary Table S3: Nutrient and phytate composition of food commodities (Ca and Zn composition data from USDA nutrient database SR 26 [USDA SR 26]; phytate composition from the Tanzanian food composition table [Tanzanian FCT]).

Supplementary Table S4: Dietary energy supply (kcal *capita*$^{-1}$ d^{-1}) by Food Balance Sheet (FBS) food item, reference year, and country.

Supplementary Table S5: Population size by age/gender group, reference year, and country.

Supplementary Table S6: Country-level Ca, Zn and phytate supplies, WtdEAR, phytate:zinc molar ratio and population between 1992 and 2011.

Supplementary Table S7: Global-level Ca and Zn supply, WtdEAR and deficiency risks between 1992 and 2011.

Supplementary Table S8: Continental-level Ca and Zn supply, WtdEAR and deficiency risks between 1992 and 2011 (mean±SD; national-level data were weighted by population size).

Supplementary Table S9: Regional-level Ca and Zn supply, WtdEAR and deficiency risks between 1992 and 2011 (mean±SD; national-level data were weighted by population size).

Supplementary Table S10: Gross national income based on purchasing power parity (GNI-PPP) by country for comparison with the supplies and deficiency risks of Ca and Zn, and energy between 1992 and 2011. GNI-PPP obtained from the World Bank (http://data.worldbank.org/indicator/NY.GNP.PCAP.PP.CD, Accessed on 09 July 2014).

Supplementary Table S11: Estimated Ca and Zn deficiency risks in 1992 and 2011; target Ca and Zn deficiency risks in 2011, consistent with MDG1 to halve hunger in the period 1990-2015; and national MDG1 Ca and Zn deficiency risk reduction target status (Yes/No).

Dietary calcium and zinc deficiency risks are decreasing but remain prevalent

Diriba B. Kumssa[1,2,3], Edward J. M. Joy[1,2], E. Louise Ander[2], Michael J. Watts[2], Scott D. Young[1], Sue Walker3 & Martin R. Broadley[1]
[1]School of Biosciences, University of Nottingham, Sutton Bonington Campus, Loughborough, LE12 5RD, UK, [2]Centre for Environmental Geochemistry, British Geological Survey, Keyworth, Nottingham, NG12 5GG, UK, [3]Crops For the Future, The University of Nottingham Malaysia Campus, Jalan Broga, 43500 Semenyih, Selangor Darul Ehsan, Malaysia.
Correspondence and requests for materials should be addressed to M.R.B. (martin.broadley@nottingham.ac.uk)

Supplementary Table S1 : Food supply by FBS food item, reference year, and country.

FAO country code	Country	Reference year	Food item	Gross food supply (g *capita*$^{-1}$ d^{-1})
2	Afghanistan	1992	Apples	3
2	Afghanistan	1992	Bananas	1
2	Afghanistan	1992	Barley	24
2	Afghanistan	1992	Bovine Meat	17
2	Afghanistan	1992	Butter, Ghee	3
2	Afghanistan	1992	Cheese	3
2	Afghanistan	1992	Citrus, Other	1
2	Afghanistan	1992	Cottonseed Oil	1
2	Afghanistan	1992	Eggs	2
2	Afghanistan	1992	Fats, Animals, Raw	3
2	Afghanistan	1992	Fruits, Other	44
2	Afghanistan	1992	Grapes	38
2	Afghanistan	1992	Honey	1
2	Afghanistan	1992	Maize	34
2	Afghanistan	1992	Meat, Other	3
2	Afghanistan	1992	Milk, Whole	87
2	Afghanistan	1992	Millet	4
2	Afghanistan	1992	Mutton & Goat Meat	26
2	Afghanistan	1992	Nuts	3
2	Afghanistan	1992	Offals, Edible	8
2	Afghanistan	1992	Oilcrops Oil, Other	6
2	Afghanistan	1992	Oranges, Mandarines	3
2	Afghanistan	1992	Palm Oil	1
2	Afghanistan	1992	Potatoes	42
2	Afghanistan	1992	Poultry Meat	3
2	Afghanistan	1992	Pulses, Other	6
2	Afghanistan	1992	Rice (Milled Equivalent)	49
2	Afghanistan	1992	Sesameseed	3
2	Afghanistan	1992	Sesameseed Oil	1
2	Afghanistan	1992	Sugar, Refined Equivalent	9
2	Afghanistan	1992	Sunflowerseed Oil	1
2	Afghanistan	1992	Tea	2
2	Afghanistan	1992	Vegetables, Other	86
2	Afghanistan	1992	Wheat	314
2	Afghanistan	1993	Apples	3
2	Afghanistan	1993	Bananas	1
2	Afghanistan	1993	Barley	28
2	Afghanistan	1993	Bovine Meat	18
2	Afghanistan	1993	Butter, Ghee	4
2	Afghanistan	1993	Cheese	3
2	Afghanistan	1993	Citrus, Other	1
2	Afghanistan	1993	Cottonseed Oil	1
2	Afghanistan	1993	Eggs	2
2	Afghanistan	1993	Fats, Animals, Raw	2
2	Afghanistan	1993	Fruits, Other	44
2	Afghanistan	1993	Grapes	36
2	Afghanistan	1993	Honey	1
2	Afghanistan	1993	Maize	49
2	Afghanistan	1993	Meat, Other	3
2	Afghanistan	1993	Milk, Whole	92
2	Afghanistan	1993	Millet	4
2	Afghanistan	1993	Mutton & Goat Meat	24
2	Afghanistan	1993	Nuts	3
2	Afghanistan	1993	Offals, Edible	8
2	Afghanistan	1993	Oilcrops Oil, Other	2
2	Afghanistan	1993	Oranges, Mandarines	2
2	Afghanistan	1993	Potatoes	38
2	Afghanistan	1993	Poultry Meat	2
2	Afghanistan	1993	Pulses, Other	6
2	Afghanistan	1993	Rice (Milled Equivalent)	39
2	Afghanistan	1993	Sesameseed	2
2	Afghanistan	1993	Sesameseed Oil	1
2	Afghanistan	1993	Sugar, Refined Equivalent	8
2	Afghanistan	1993	Sunflowerseed Oil	1
2	Afghanistan	1993	Tea	2
2	Afghanistan	1993	Vegetables, Other	82
2	Afghanistan	1993	Wheat	332
2	Afghanistan	1994	Apples	2
2	Afghanistan	1994	Barley	24
2	Afghanistan	1994	Bovine Meat	19
2	Afghanistan	1994	Butter, Ghee	4
2	Afghanistan	1994	Cheese	3
2	Afghanistan	1994	Citrus, Other	1
2	Afghanistan	1994	Cottonseed Oil	1
2	Afghanistan	1994	Eggs	2
2	Afghanistan	1994	Fats, Animals, Raw	2
2	Afghanistan	1994	Fruits, Other	44
2	Afghanistan	1994	Grapes	34
2	Afghanistan	1994	Honey	1
2	Afghanistan	1994	Maize	48
2	Afghanistan	1994	Meat, Other	2
2	Afghanistan	1994	Milk, Whole	102
2	Afghanistan	1994	Millet	3
2	Afghanistan	1994	Mutton & Goat Meat	22
2	Afghanistan	1994	Nuts	3

FAO country code	Country	Reference year	Food item	Gross food supply (g $capita^{-1}$ d^{-1})
2	Afghanistan	1994	Offals, Edible	8
2	Afghanistan	1994	Oilcrops Oil, Other	1
2	Afghanistan	1994	Oranges, Mandarines	2
2	Afghanistan	1994	Potatoes	35
2	Afghanistan	1994	Poultry Meat	2
2	Afghanistan	1994	Pulses, Other	7
2	Afghanistan	1994	Rice (Milled Equivalent)	43
2	Afghanistan	1994	Sesameseed	2
2	Afghanistan	1994	Sesameseed Oil	1
2	Afghanistan	1994	Sugar, Refined Equivalent	7
2	Afghanistan	1994	Sunflowerseed Oil	1
2	Afghanistan	1994	Tea	2
2	Afghanistan	1994	Vegetables, Other	74
2	Afghanistan	1994	Wheat	308
2	Afghanistan	1995	Apples	2
2	Afghanistan	1995	Barley	19
2	Afghanistan	1995	Bovine Meat	20
2	Afghanistan	1995	Butter, Ghee	4
2	Afghanistan	1995	Cheese	3
2	Afghanistan	1995	Citrus, Other	1
2	Afghanistan	1995	Cottonseed Oil	1
2	Afghanistan	1995	Eggs	2
2	Afghanistan	1995	Fats, Animals, Raw	4
2	Afghanistan	1995	Fruits, Other	40
2	Afghanistan	1995	Grapes	35
2	Afghanistan	1995	Honey	1
2	Afghanistan	1995	Maize	48
2	Afghanistan	1995	Meat, Other	2
2	Afghanistan	1995	Milk, Whole	109
2	Afghanistan	1995	Millet	3
2	Afghanistan	1995	Mutton & Goat Meat	21
2	Afghanistan	1995	Nuts	2
2	Afghanistan	1995	Offals, Edible	8
2	Afghanistan	1995	Oilcrops Oil, Other	2
2	Afghanistan	1995	Oranges, Mandarines	2
2	Afghanistan	1995	Potatoes	33
2	Afghanistan	1995	Poultry Meat	2
2	Afghanistan	1995	Pulses, Other	5
2	Afghanistan	1995	Rice (Milled Equivalent)	50
2	Afghanistan	1995	Sesameseed	2
2	Afghanistan	1995	Sesameseed Oil	1
2	Afghanistan	1995	Soyabean Oil	5
2	Afghanistan	1995	Sugar, Refined Equivalent	7
2	Afghanistan	1995	Sunflowerseed Oil	3
2	Afghanistan	1995	Tea	3
2	Afghanistan	1995	Vegetables, Other	75
2	Afghanistan	1995	Wheat	289
2	Afghanistan	1996	Apples	2
2	Afghanistan	1996	Barley	21
2	Afghanistan	1996	Bovine Meat	21
2	Afghanistan	1996	Butter, Ghee	4
2	Afghanistan	1996	Cheese	3
2	Afghanistan	1996	Citrus, Other	1
2	Afghanistan	1996	Cottonseed Oil	1
2	Afghanistan	1996	Eggs	2
2	Afghanistan	1996	Fats, Animals, Raw	3
2	Afghanistan	1996	Fruits, Other	40
2	Afghanistan	1996	Grapes	31
2	Afghanistan	1996	Honey	1
2	Afghanistan	1996	Maize	31
2	Afghanistan	1996	Meat, Other	1
2	Afghanistan	1996	Milk, Whole	119
2	Afghanistan	1996	Millet	3
2	Afghanistan	1996	Mutton & Goat Meat	22
2	Afghanistan	1996	Nuts	2
2	Afghanistan	1996	Offals, Edible	8
2	Afghanistan	1996	Oilcrops Oil, Other	1
2	Afghanistan	1996	Oranges, Mandarines	2
2	Afghanistan	1996	Potatoes	32
2	Afghanistan	1996	Poultry Meat	2
2	Afghanistan	1996	Pulses, Other	5
2	Afghanistan	1996	Rice (Milled Equivalent)	38
2	Afghanistan	1996	Sesameseed	2
2	Afghanistan	1996	Sesameseed Oil	1
2	Afghanistan	1996	Soyabean Oil	5
2	Afghanistan	1996	Sugar, Refined Equivalent	6
2	Afghanistan	1996	Sunflowerseed Oil	2
2	Afghanistan	1996	Tea	2
2	Afghanistan	1996	Vegetables, Other	74
2	Afghanistan	1996	Wheat	324
2	Afghanistan	1997	Apples	2
2	Afghanistan	1997	Barley	23
2	Afghanistan	1997	Bovine Meat	22
2	Afghanistan	1997	Butter, Ghee	5
2	Afghanistan	1997	Cheese	3
2	Afghanistan	1997	Citrus, Other	1
2	Afghanistan	1997	Cottonseed Oil	1
2	Afghanistan	1997	Eggs	2
2	Afghanistan	1997	Fats, Animals, Raw	3
2	Afghanistan	1997	Fruits, Other	40
2	Afghanistan	1997	Grapes	31
2	Afghanistan	1997	Honey	1
2	Afghanistan	1997	Maize	25
2	Afghanistan	1997	Meat, Other	1
2	Afghanistan	1997	Milk, Whole	127
2	Afghanistan	1997	Millet	3
2	Afghanistan	1997	Mutton & Goat Meat	23

FAO country code	Country	Reference year	Food item	Gross food supply (g $capita^{-1}$ d^{-1})
2	Afghanistan	1997	Nuts	2
2	Afghanistan	1997	Offals, Edible	8
2	Afghanistan	1997	Oranges, Mandarines	3
2	Afghanistan	1997	Potatoes	31
2	Afghanistan	1997	Poultry Meat	2
2	Afghanistan	1997	Pulses, Other	4
2	Afghanistan	1997	Rice (Milled Equivalent)	47
2	Afghanistan	1997	Sesameseed	2
2	Afghanistan	1997	Sesameseed Oil	1
2	Afghanistan	1997	Soyabean Oil	1
2	Afghanistan	1997	Sugar, Refined Equivalent	7
2	Afghanistan	1997	Sunflowerseed Oil	1
2	Afghanistan	1997	Tea	4
2	Afghanistan	1997	Vegetables, Other	76
2	Afghanistan	1997	Wheat	337
2	Afghanistan	1998	Apples	2
2	Afghanistan	1998	Barley	22
2	Afghanistan	1998	Bovine Meat	24
2	Afghanistan	1998	Butter, Ghee	5
2	Afghanistan	1998	Cheese	4
2	Afghanistan	1998	Citrus, Other	1
2	Afghanistan	1998	Cottonseed Oil	1
2	Afghanistan	1998	Eggs	2
2	Afghanistan	1998	Fats, Animals, Raw	3
2	Afghanistan	1998	Fruits, Other	42
2	Afghanistan	1998	Grapes	30
2	Afghanistan	1998	Honey	1
2	Afghanistan	1998	Maize	27
2	Afghanistan	1998	Meat, Other	1
2	Afghanistan	1998	Milk, Whole	129
2	Afghanistan	1998	Millet	3
2	Afghanistan	1998	Mutton & Goat Meat	24
2	Afghanistan	1998	Nuts	2
2	Afghanistan	1998	Offals, Edible	9
2	Afghanistan	1998	Oranges, Mandarines	2
2	Afghanistan	1998	Potatoes	30
2	Afghanistan	1998	Poultry Meat	2
2	Afghanistan	1998	Pulses, Other	5
2	Afghanistan	1998	Rice (Milled Equivalent)	49
2	Afghanistan	1998	Sesameseed	2
2	Afghanistan	1998	Sesameseed Oil	1
2	Afghanistan	1998	Soyabean Oil	2
2	Afghanistan	1998	Sugar, Refined Equivalent	7
2	Afghanistan	1998	Sunflowerseed Oil	1
2	Afghanistan	1998	Tea	3
2	Afghanistan	1998	Vegetables, Other	74
2	Afghanistan	1998	Wheat	338
2	Afghanistan	1999	Apples	1
2	Afghanistan	1999	Barley	19
2	Afghanistan	1999	Bovine Meat	20
2	Afghanistan	1999	Butter, Ghee	5
2	Afghanistan	1999	Cheese	4
2	Afghanistan	1999	Citrus, Other	1
2	Afghanistan	1999	Cottonseed Oil	1
2	Afghanistan	1999	Eggs	2
2	Afghanistan	1999	Fats, Animals, Raw	3
2	Afghanistan	1999	Fruits, Other	38
2	Afghanistan	1999	Grapes	27
2	Afghanistan	1999	Honey	1
2	Afghanistan	1999	Maize	19
2	Afghanistan	1999	Meat, Other	1
2	Afghanistan	1999	Milk, Whole	145
2	Afghanistan	1999	Millet	2
2	Afghanistan	1999	Mutton & Goat Meat	26
2	Afghanistan	1999	Nuts	2
2	Afghanistan	1999	Offals, Edible	8
2	Afghanistan	1999	Oranges, Mandarines	2
2	Afghanistan	1999	Potatoes	30
2	Afghanistan	1999	Poultry Meat	2
2	Afghanistan	1999	Pulses, Other	3
2	Afghanistan	1999	Rice (Milled Equivalent)	37
2	Afghanistan	1999	Sesameseed	2
2	Afghanistan	1999	Sesameseed Oil	1
2	Afghanistan	1999	Soyabean Oil	4
2	Afghanistan	1999	Sugar, Refined Equivalent	8
2	Afghanistan	1999	Sunflowerseed Oil	1
2	Afghanistan	1999	Tea	2
2	Afghanistan	1999	Vegetables, Other	79
2	Afghanistan	1999	Wheat	338
2	Afghanistan	2000	Apples	1
2	Afghanistan	2000	Barley	11
2	Afghanistan	2000	Bovine Meat	17
2	Afghanistan	2000	Butter, Ghee	4
2	Afghanistan	2000	Cheese	3
2	Afghanistan	2000	Citrus, Other	1
2	Afghanistan	2000	Cottonseed Oil	1
2	Afghanistan	2000	Eggs	2
2	Afghanistan	2000	Fats, Animals, Raw	2
2	Afghanistan	2000	Fruits, Other	38
2	Afghanistan	2000	Grapes	25
2	Afghanistan	2000	Honey	1
2	Afghanistan	2000	Maize	9
2	Afghanistan	2000	Meat, Other	1
2	Afghanistan	2000	Milk, Whole	113
2	Afghanistan	2000	Millet	3
2	Afghanistan	2000	Mutton & Goat Meat	23
2	Afghanistan	2000	Nuts	2

FAO country code	Country	Reference year	Food item	Gross food supply (g $capita^{-1}$ d^{-1})
2	Afghanistan	2000	Offals, Edible	7
2	Afghanistan	2000	Oranges, Mandarines	2
2	Afghanistan	2000	Potatoes	29
2	Afghanistan	2000	Poultry Meat	2
2	Afghanistan	2000	Pulses, Other	3
2	Afghanistan	2000	Rice (Milled Equivalent)	49
2	Afghanistan	2000	Sesameseed	2
2	Afghanistan	2000	Sesameseed Oil	1
2	Afghanistan	2000	Soyabean Oil	3
2	Afghanistan	2000	Sugar, Refined Equivalent	7
2	Afghanistan	2000	Sunflowerseed Oil	1
2	Afghanistan	2000	Tea	3
2	Afghanistan	2000	Vegetables, Other	80
2	Afghanistan	2000	Wheat	346
2	Afghanistan	2001	Apples	2
2	Afghanistan	2001	Barley	11
2	Afghanistan	2001	Bovine Meat	14
2	Afghanistan	2001	Butter, Ghee	3
2	Afghanistan	2001	Cheese	3
2	Afghanistan	2001	Citrus, Other	1
2	Afghanistan	2001	Cottonseed Oil	1
2	Afghanistan	2001	Eggs	1
2	Afghanistan	2001	Fats, Animals, Raw	2
2	Afghanistan	2001	Fruits, Other	38
2	Afghanistan	2001	Grapes	32
2	Afghanistan	2001	Honey	1
2	Afghanistan	2001	Maize	12
2	Afghanistan	2001	Meat, Other	1
2	Afghanistan	2001	Milk, Whole	70
2	Afghanistan	2001	Millet	3
2	Afghanistan	2001	Mutton & Goat Meat	19
2	Afghanistan	2001	Nuts	1
2	Afghanistan	2001	Offals, Edible	5
2	Afghanistan	2001	Oranges, Mandarines	3
2	Afghanistan	2001	Potatoes	28
2	Afghanistan	2001	Poultry Meat	2
2	Afghanistan	2001	Pulses, Other	5
2	Afghanistan	2001	Rice (Milled Equivalent)	53
2	Afghanistan	2001	Sesameseed	1
2	Afghanistan	2001	Sesameseed Oil	1
2	Afghanistan	2001	Soyabean Oil	3
2	Afghanistan	2001	Sugar, Refined Equivalent	7
2	Afghanistan	2001	Sunflowerseed Oil	1
2	Afghanistan	2001	Tea	3
2	Afghanistan	2001	Vegetables, Other	93
2	Afghanistan	2001	Wheat	342
2	Afghanistan	2002	Apples	2
2	Afghanistan	2002	Barley	15
2	Afghanistan	2002	Bovine Meat	19
2	Afghanistan	2002	Butter, Ghee	4
2	Afghanistan	2002	Cheese	2
2	Afghanistan	2002	Citrus, Other	1
2	Afghanistan	2002	Cottonseed Oil	1
2	Afghanistan	2002	Eggs	2
2	Afghanistan	2002	Fats, Animals, Raw	1
2	Afghanistan	2002	Fruits, Other	36
2	Afghanistan	2002	Grapes	27
2	Afghanistan	2002	Honey	1
2	Afghanistan	2002	Maize	21
2	Afghanistan	2002	Meat, Other	1
2	Afghanistan	2002	Milk, Whole	122
2	Afghanistan	2002	Millet	2
2	Afghanistan	2002	Mutton & Goat Meat	15
2	Afghanistan	2002	Nuts	2
2	Afghanistan	2002	Offals, Edible	6
2	Afghanistan	2002	Oranges, Mandarines	3
2	Afghanistan	2002	Palm Oil	2
2	Afghanistan	2002	Potatoes	27
2	Afghanistan	2002	Poultry Meat	3
2	Afghanistan	2002	Pulses, Other	3
2	Afghanistan	2002	Rice (Milled Equivalent)	55
2	Afghanistan	2002	Sesameseed	1
2	Afghanistan	2002	Soyabean Oil	1
2	Afghanistan	2002	Sugar, Refined Equivalent	8
2	Afghanistan	2002	Sunflowerseed Oil	1
2	Afghanistan	2002	Tea	2
2	Afghanistan	2002	Vegetables, Other	74
2	Afghanistan	2002	Wheat	343
2	Afghanistan	2003	Apples	2
2	Afghanistan	2003	Bananas	1
2	Afghanistan	2003	Barley	17
2	Afghanistan	2003	Bovine Meat	16
2	Afghanistan	2003	Butter, Ghee	4
2	Afghanistan	2003	Cheese	2
2	Afghanistan	2003	Citrus, Other	1
2	Afghanistan	2003	Eggs	2
2	Afghanistan	2003	Fats, Animals, Raw	1
2	Afghanistan	2003	Fruits, Other	29
2	Afghanistan	2003	Grapes	20
2	Afghanistan	2003	Honey	1
2	Afghanistan	2003	Maize	14
2	Afghanistan	2003	Meat, Other	1
2	Afghanistan	2003	Milk, Whole	109
2	Afghanistan	2003	Millet	2
2	Afghanistan	2003	Mutton & Goat Meat	14
2	Afghanistan	2003	Nuts	1
2	Afghanistan	2003	Offals, Edible	5

FAO countrycode	Country	Reference year	Food item	Gross food supply (g *capita*$^{-1}$ d^{-1})
2	Afghanistan	2003	Oranges, Mandarines	3
2	Afghanistan	2003	Palm Oil	4
2	Afghanistan	2003	Potatoes	41
2	Afghanistan	2003	Poultry Meat	3
2	Afghanistan	2003	Pulses, Other	3
2	Afghanistan	2003	Rice (Milled Equivalent)	55
2	Afghanistan	2003	Sesameseed	2
2	Afghanistan	2003	Soyabean Oil	1
2	Afghanistan	2003	Sugar, Refined Equivalent	10
2	Afghanistan	2003	Sunflowerseed Oil	1
2	Afghanistan	2003	Sweeteners, Other	1
2	Afghanistan	2003	Tea	6
2	Afghanistan	2003	Vegetables, Other	95
2	Afghanistan	2003	Wheat	367
2	Afghanistan	2004	Apples	2
2	Afghanistan	2004	Bananas	1
2	Afghanistan	2004	Barley	21
2	Afghanistan	2004	Beer	1
2	Afghanistan	2004	Bovine Meat	17
2	Afghanistan	2004	Butter, Ghee	4
2	Afghanistan	2004	Cheese	2
2	Afghanistan	2004	Citrus, Other	1
2	Afghanistan	2004	Eggs	2
2	Afghanistan	2004	Fats, Animals, Raw	1
2	Afghanistan	2004	Fruits, Other	28
2	Afghanistan	2004	Grapes	23
2	Afghanistan	2004	Maize	26
2	Afghanistan	2004	Meat, Other	1
2	Afghanistan	2004	Milk, Whole	108
2	Afghanistan	2004	Millet	2
2	Afghanistan	2004	Mutton & Goat Meat	15
2	Afghanistan	2004	Nuts	1
2	Afghanistan	2004	Offals, Edible	5
2	Afghanistan	2004	Oranges, Mandarines	4
2	Afghanistan	2004	Palm Oil	8
2	Afghanistan	2004	Potatoes	32
2	Afghanistan	2004	Poultry Meat	4
2	Afghanistan	2004	Pulses, Other	2
2	Afghanistan	2004	Rice (Milled Equivalent)	48
2	Afghanistan	2004	Sesameseed	2
2	Afghanistan	2004	Soyabean Oil	1
2	Afghanistan	2004	Sugar, Refined Equivalent	13
2	Afghanistan	2004	Sunflowerseed Oil	1
2	Afghanistan	2004	Sweeteners, Other	1
2	Afghanistan	2004	Tea	3
2	Afghanistan	2004	Vegetables, Other	72
2	Afghanistan	2004	Wheat	371
2	Afghanistan	2005	Apples	2
2	Afghanistan	2005	Barley	5
2	Afghanistan	2005	Bovine Meat	16
2	Afghanistan	2005	Butter, Ghee	4
2	Afghanistan	2005	Cheese	2
2	Afghanistan	2005	Citrus, Other	1
2	Afghanistan	2005	Dates	1
2	Afghanistan	2005	Eggs	2
2	Afghanistan	2005	Fats, Animals, Raw	1
2	Afghanistan	2005	Fruits, Other	30
2	Afghanistan	2005	Grapes	25
2	Afghanistan	2005	Maize	7
2	Afghanistan	2005	Meat, Other	2
2	Afghanistan	2005	Milk, Whole	101
2	Afghanistan	2005	Millet	2
2	Afghanistan	2005	Mutton & Goat Meat	15
2	Afghanistan	2005	Nuts	1
2	Afghanistan	2005	Offals, Edible	6
2	Afghanistan	2005	Oranges, Mandarines	1
2	Afghanistan	2005	Palm Oil	8
2	Afghanistan	2005	Potatoes	32
2	Afghanistan	2005	Poultry Meat	6
2	Afghanistan	2005	Pulses, Other	4
2	Afghanistan	2005	Rice (Milled Equivalent)	49
2	Afghanistan	2005	Sesameseed	2
2	Afghanistan	2005	Soyabean Oil	4
2	Afghanistan	2005	Sugar, Refined Equivalent	15
2	Afghanistan	2005	Sunflowerseed Oil	1
2	Afghanistan	2005	Sweeteners, Other	2
2	Afghanistan	2005	Tea	1
2	Afghanistan	2005	Vegetables, Other	101
2	Afghanistan	2005	Wheat	384
2	Afghanistan	2006	Apples	5
2	Afghanistan	2006	Bananas	1
2	Afghanistan	2006	Barley	5
2	Afghanistan	2006	Bovine Meat	13
2	Afghanistan	2006	Butter, Ghee	3
2	Afghanistan	2006	Cheese	2
2	Afghanistan	2006	Citrus, Other	1
2	Afghanistan	2006	Dates	1
2	Afghanistan	2006	Eggs	2
2	Afghanistan	2006	Fats, Animals, Raw	1
2	Afghanistan	2006	Fruits, Other	28
2	Afghanistan	2006	Grapes	21
2	Afghanistan	2006	Maize	9
2	Afghanistan	2006	Meat, Other	1
2	Afghanistan	2006	Milk, Whole	93
2	Afghanistan	2006	Millet	1
2	Afghanistan	2006	Mutton & Goat Meat	13
2	Afghanistan	2006	Nuts	3

FAO country code	Country	Reference year	Food item	Gross food supply (g *capita*$^{-1}$ d^{-1})
2	Afghanistan	2006	Offals, Edible	5
2	Afghanistan	2006	Oranges, Mandarines	5
2	Afghanistan	2006	Palm Oil	6
2	Afghanistan	2006	Potatoes	31
2	Afghanistan	2006	Poultry Meat	4
2	Afghanistan	2006	Pulses, Other	4
2	Afghanistan	2006	Rice (Milled Equivalent)	58
2	Afghanistan	2006	Sesameseed	1
2	Afghanistan	2006	Soyabean Oil	2
2	Afghanistan	2006	Sugar, Refined Equivalent	17
2	Afghanistan	2006	Sunflowerseed Oil	1
2	Afghanistan	2006	Sweeteners, Other	1
2	Afghanistan	2006	Tea	2
2	Afghanistan	2006	Tomatoes	1
2	Afghanistan	2006	Vegetables, Other	101
2	Afghanistan	2006	Wheat	396
2	Afghanistan	2007	Apples	5
2	Afghanistan	2007	Bananas	1
2	Afghanistan	2007	Barley	5
2	Afghanistan	2007	Bovine Meat	15
2	Afghanistan	2007	Butter, Ghee	3
2	Afghanistan	2007	Cheese	2
2	Afghanistan	2007	Eggs	3
2	Afghanistan	2007	Fats, Animals, Raw	1
2	Afghanistan	2007	Fruits, Other	24
2	Afghanistan	2007	Grapes	24
2	Afghanistan	2007	Maize	7
2	Afghanistan	2007	Meat, Other	1
2	Afghanistan	2007	Milk, Whole	94
2	Afghanistan	2007	Millet	2
2	Afghanistan	2007	Mutton & Goat Meat	10
2	Afghanistan	2007	Nuts	4
2	Afghanistan	2007	Offals, Edible	5
2	Afghanistan	2007	Oranges, Mandarines	1
2	Afghanistan	2007	Palm Oil	5
2	Afghanistan	2007	Potatoes	27
2	Afghanistan	2007	Poultry Meat	3
2	Afghanistan	2007	Pulses, Other	4
2	Afghanistan	2007	Rape and Mustard Oil	1
2	Afghanistan	2007	Rice (Milled Equivalent)	47
2	Afghanistan	2007	Sesameseed	2
2	Afghanistan	2007	Soyabean Oil	2
2	Afghanistan	2007	Sugar, Refined Equivalent	18
2	Afghanistan	2007	Sunflowerseed Oil	1
2	Afghanistan	2007	Sweeteners, Other	1
2	Afghanistan	2007	Tea	3
2	Afghanistan	2007	Vegetables, Other	99
2	Afghanistan	2007	Wheat	433
2	Afghanistan	2008	Apples	5
2	Afghanistan	2008	Bananas	1
2	Afghanistan	2008	Barley	6
2	Afghanistan	2008	Bovine Meat	14
2	Afghanistan	2008	Butter, Ghee	3
2	Afghanistan	2008	Cheese	2
2	Afghanistan	2008	Eggs	3
2	Afghanistan	2008	Fats, Animals, Raw	1
2	Afghanistan	2008	Fruits, Other	24
2	Afghanistan	2008	Grapes	20
2	Afghanistan	2008	Maize	7
2	Afghanistan	2008	Meat, Other	1
2	Afghanistan	2008	Milk, Whole	90
2	Afghanistan	2008	Millet	2
2	Afghanistan	2008	Mutton & Goat Meat	13
2	Afghanistan	2008	Nuts	4
2	Afghanistan	2008	Offals, Edible	5
2	Afghanistan	2008	Oranges, Mandarines	1
2	Afghanistan	2008	Palm Oil	4
2	Afghanistan	2008	Potatoes	25
2	Afghanistan	2008	Poultry Meat	5
2	Afghanistan	2008	Pulses, Other	5
2	Afghanistan	2008	Rape and Mustard Oil	1
2	Afghanistan	2008	Rice (Milled Equivalent)	50
2	Afghanistan	2008	Sesameseed	2
2	Afghanistan	2008	Soyabean Oil	1
2	Afghanistan	2008	Spices, Other	1
2	Afghanistan	2008	Sugar, Refined Equivalent	20
2	Afghanistan	2008	Sweeteners, Other	1
2	Afghanistan	2008	Tea	4
2	Afghanistan	2008	Vegetables, Other	91
2	Afghanistan	2008	Wheat	431
2	Afghanistan	2009	Apples	6
2	Afghanistan	2009	Bananas	4
2	Afghanistan	2009	Barley	5
2	Afghanistan	2009	Bovine Meat	13
2	Afghanistan	2009	Butter, Ghee	3
2	Afghanistan	2009	Cheese	2
2	Afghanistan	2009	Eggs	3
2	Afghanistan	2009	Fats, Animals, Raw	1
2	Afghanistan	2009	Fruits, Other	22
2	Afghanistan	2009	Grapes	21
2	Afghanistan	2009	Maize	7
2	Afghanistan	2009	Meat, Other	1
2	Afghanistan	2009	Milk, Whole	89
2	Afghanistan	2009	Millet	2
2	Afghanistan	2009	Mutton & Goat Meat	14
2	Afghanistan	2009	Nuts	3
2	Afghanistan	2009	Offals, Edible	5

FAO country code	Country	Reference year	Food item	Gross food supply (g *capita*$^{-1}$ d^{-1})
2	Afghanistan	2009	Oranges, Mandarines	4
2	Afghanistan	2009	Palm Oil	5
2	Afghanistan	2009	Potatoes	25
2	Afghanistan	2009	Poultry Meat	4
2	Afghanistan	2009	Pulses, Other	8
2	Afghanistan	2009	Rape and Mustard Oil	1
2	Afghanistan	2009	Rice (Milled Equivalent)	41
2	Afghanistan	2009	Sesameseed	2
2	Afghanistan	2009	Soyabean Oil	1
2	Afghanistan	2009	Sugar, Refined Equivalent	21
2	Afghanistan	2009	Sunflowerseed Oil	1
2	Afghanistan	2009	Tea	1
2	Afghanistan	2009	Vegetables, Other	104
2	Afghanistan	2009	Wheat	449
2	Afghanistan	2010	Apples	6
2	Afghanistan	2010	Bananas	2
2	Afghanistan	2010	Barley	6
2	Afghanistan	2010	Bovine Meat	13
2	Afghanistan	2010	Butter, Ghee	3
2	Afghanistan	2010	Cheese	2
2	Afghanistan	2010	Dates	1
2	Afghanistan	2010	Eggs	2
2	Afghanistan	2010	Fats, Animals, Raw	2
2	Afghanistan	2010	Fruits, Other	24
2	Afghanistan	2010	Grapes	25
2	Afghanistan	2010	Maize	8
2	Afghanistan	2010	Meat, Other	1
2	Afghanistan	2010	Milk, Whole	87
2	Afghanistan	2010	Millet	1
2	Afghanistan	2010	Mutton & Goat Meat	15
2	Afghanistan	2010	Nuts	6
2	Afghanistan	2010	Offals, Edible	5
2	Afghanistan	2010	Oranges, Mandarines	12
2	Afghanistan	2010	Palm Oil	6
2	Afghanistan	2010	Potatoes	19
2	Afghanistan	2010	Poultry Meat	6
2	Afghanistan	2010	Pulses, Other	6
2	Afghanistan	2010	Rice (Milled Equivalent)	43
2	Afghanistan	2010	Sesameseed	2
2	Afghanistan	2010	Soyabean Oil	1
2	Afghanistan	2010	Sugar, Refined Equivalent	21
2	Afghanistan	2010	Sunflowerseed Oil	1
2	Afghanistan	2010	Sweeteners, Other	1
2	Afghanistan	2010	Tea	3
2	Afghanistan	2010	Vegetables, Other	94
2	Afghanistan	2010	Wheat	444
2	Afghanistan	2011	Apples	5
2	Afghanistan	2011	Bananas	3
2	Afghanistan	2011	Barley	7
2	Afghanistan	2011	Bovine Meat	13
2	Afghanistan	2011	Butter, Ghee	3
2	Afghanistan	2011	Cheese	2
2	Afghanistan	2011	Citrus, Other	3
2	Afghanistan	2011	Dates	1
2	Afghanistan	2011	Eggs	3
2	Afghanistan	2011	Fats, Animals, Raw	2
2	Afghanistan	2011	Fruits, Other	19
2	Afghanistan	2011	Grapes	31
2	Afghanistan	2011	Maize	7
2	Afghanistan	2011	Meat, Other	1
2	Afghanistan	2011	Milk, Whole	85
2	Afghanistan	2011	Millet	1
2	Afghanistan	2011	Mutton & Goat Meat	15
2	Afghanistan	2011	Nuts	7
2	Afghanistan	2011	Offals, Edible	5
2	Afghanistan	2011	Oranges, Mandarines	10
2	Afghanistan	2011	Palm Oil	5
2	Afghanistan	2011	Potatoes	16
2	Afghanistan	2011	Poultry Meat	7
2	Afghanistan	2011	Pulses, Other	8
2	Afghanistan	2011	Rice (Milled Equivalent)	45
2	Afghanistan	2011	Sesameseed	2
2	Afghanistan	2011	Soyabean Oil	2
2	Afghanistan	2011	Spices, Other	1
2	Afghanistan	2011	Sugar, Refined Equivalent	21
2	Afghanistan	2011	Sunflowerseed Oil	1
2	Afghanistan	2011	Sweeteners, Other	2
2	Afghanistan	2011	Tea	6
2	Afghanistan	2011	Vegetables, Other	81
2	Afghanistan	2011	Wheat	443
3	Albania	1992	Apples	8
3	Albania	1992	Barley	1
3	Albania	1992	Beans	11
3	Albania	1992	Beer	3
3	Albania	1992	Beverages, Alcoholic	3
3	Albania	1992	Bovine Meat	31
3	Albania	1992	Butter, Ghee	8
3	Albania	1992	Cheese	8
3	Albania	1992	Cocoa Beans	1
3	Albania	1992	Coffee	2
3	Albania	1992	Demersal Fish	1
3	Albania	1992	Eggs	10
3	Albania	1992	Fats, Animals, Raw	3
3	Albania	1992	Freshwater Fish	1
3	Albania	1992	Fruits, Other	18
3	Albania	1992	Grapes	18
3	Albania	1992	Maize	16

FAO country code	Country	Reference year	Food item	Gross food supply (g *capita*$^{-1}$ d^{-1})
3	Albania	1992	Milk, Whole	393
3	Albania	1992	Mutton & Goat Meat	12
3	Albania	1992	Nuts	3
3	Albania	1992	Offals, Edible	6
3	Albania	1992	Olive Oil	2
3	Albania	1992	Olives	6
3	Albania	1992	Onions	37
3	Albania	1992	Oranges, Mandarines	10
3	Albania	1992	Pigmeat	10
3	Albania	1992	Potatoes	40
3	Albania	1992	Poultry Meat	2
3	Albania	1992	Rice (Milled Equivalent)	13
3	Albania	1992	Soyabean Oil	4
3	Albania	1992	Sugar, Refined Equivalent	37
3	Albania	1992	Sunflowerseed Oil	17
3	Albania	1992	Sweeteners, Other	11
3	Albania	1992	Tomatoes	79
3	Albania	1992	Vegetables, Other	239
3	Albania	1992	Wheat	537
3	Albania	1992	Wine	10
3	Albania	1993	Apples	21
3	Albania	1993	Bananas	1
3	Albania	1993	Beans	11
3	Albania	1993	Beer	18
3	Albania	1993	Beverages, Alcoholic	1
3	Albania	1993	Bovine Meat	24
3	Albania	1993	Butter, Ghee	9
3	Albania	1993	Cheese	8
3	Albania	1993	Cocoa Beans	1
3	Albania	1993	Coffee	3
3	Albania	1993	Eggs	13
3	Albania	1993	Fats, Animals, Raw	2
3	Albania	1993	Freshwater Fish	1
3	Albania	1993	Fruits, Other	25
3	Albania	1993	Grapes	28
3	Albania	1993	Maize	16
3	Albania	1993	Milk, Whole	442
3	Albania	1993	Molluscs, Other	1
3	Albania	1993	Mutton & Goat Meat	12
3	Albania	1993	Nuts	3
3	Albania	1993	Offals, Edible	7
3	Albania	1993	Olive Oil	2
3	Albania	1993	Olives	3
3	Albania	1993	Onions	42
3	Albania	1993	Oranges, Mandarines	20
3	Albania	1993	Pigmeat	13
3	Albania	1993	Potatoes	40
3	Albania	1993	Poultry Meat	11
3	Albania	1993	Rice (Milled Equivalent)	17
3	Albania	1993	Rye	1
3	Albania	1993	Soyabean Oil	2
3	Albania	1993	Sugar, Refined Equivalent	40
3	Albania	1993	Sunflowerseed Oil	18
3	Albania	1993	Sweeteners, Other	32
3	Albania	1993	Tomatoes	90
3	Albania	1993	Vegetables, Other	273
3	Albania	1993	Wheat	545
3	Albania	1993	Wine	10
3	Albania	1994	Apples	16
3	Albania	1994	Bananas	4
3	Albania	1994	Beans	10
3	Albania	1994	Beer	44
3	Albania	1994	Beverages, Alcoholic	4
3	Albania	1994	Bovine Meat	27
3	Albania	1994	Butter, Ghee	5
3	Albania	1994	Cheese	11
3	Albania	1994	Cocoa Beans	1
3	Albania	1994	Coffee	2
3	Albania	1994	Eggs	13
3	Albania	1994	Fats, Animals, Raw	2
3	Albania	1994	Freshwater Fish	1
3	Albania	1994	Fruits, Other	29
3	Albania	1994	Grapes	30
3	Albania	1994	Lemons, Limes	2
3	Albania	1994	Maize	16
3	Albania	1994	Milk, Whole	543
3	Albania	1994	Molluscs, Other	2
3	Albania	1994	Mutton & Goat Meat	15
3	Albania	1994	Nuts	4
3	Albania	1994	Offals, Edible	9
3	Albania	1994	Olive Oil	2
3	Albania	1994	Olives	6
3	Albania	1994	Onions	46
3	Albania	1994	Oranges, Mandarines	25
3	Albania	1994	Pigmeat	14
3	Albania	1994	Potatoes	40
3	Albania	1994	Poultry Meat	12
3	Albania	1994	Rice (Milled Equivalent)	8
3	Albania	1994	Rye	2
3	Albania	1994	Sugar, Refined Equivalent	45
3	Albania	1994	Sunflowerseed Oil	17
3	Albania	1994	Sweeteners, Other	49
3	Albania	1994	Tomatoes	95
3	Albania	1994	Vegetables, Other	287
3	Albania	1994	Wheat	550
3	Albania	1994	Wine	10
3	Albania	1995	Apples	13

FAO country code	Country	Reference year	Food item	Gross food supply (g $capita^{-1}$ d^{-1})
3	Albania	1995	Bananas	18
3	Albania	1995	Beans	11
3	Albania	1995	Beer	35
3	Albania	1995	Beverages, Alcoholic	3
3	Albania	1995	Bovine Meat	28
3	Albania	1995	Butter, Ghee	4
3	Albania	1995	Cheese	11
3	Albania	1995	Cocoa Beans	1
3	Albania	1995	Coffee	2
3	Albania	1995	Eggs	14
3	Albania	1995	Fats, Animals, Raw	4
3	Albania	1995	Fruits, Other	29
3	Albania	1995	Grapes	31
3	Albania	1995	Lemons, Limes	1
3	Albania	1995	Maize	19
3	Albania	1995	Milk, Whole	644
3	Albania	1995	Molluscs, Other	3
3	Albania	1995	Mutton & Goat Meat	15
3	Albania	1995	Nuts	3
3	Albania	1995	Offals, Edible	10
3	Albania	1995	Oilcrops Oil, Other	1
3	Albania	1995	Olive Oil	2
3	Albania	1995	Olives	7
3	Albania	1995	Onions	33
3	Albania	1995	Oranges, Mandarines	18
3	Albania	1995	Pelagic Fish	1
3	Albania	1995	Pigmeat	14
3	Albania	1995	Potatoes	61
3	Albania	1995	Poultry Meat	14
3	Albania	1995	Rice (Milled Equivalent)	15
3	Albania	1995	Rye	3
3	Albania	1995	Sugar, Refined Equivalent	50
3	Albania	1995	Sunflowerseed Oil	15
3	Albania	1995	Tomatoes	115
3	Albania	1995	Vegetables, Other	245
3	Albania	1995	Wheat	444
3	Albania	1995	Wine	15
3	Albania	1996	Apples	25
3	Albania	1996	Bananas	10
3	Albania	1996	Beans	12
3	Albania	1996	Beer	20
3	Albania	1996	Beverages, Alcoholic	5
3	Albania	1996	Bovine Meat	33
3	Albania	1996	Butter, Ghee	2
3	Albania	1996	Cereals, Other	2
3	Albania	1996	Cheese	11
3	Albania	1996	Cocoa Beans	1
3	Albania	1996	Coffee	1
3	Albania	1996	Eggs	16
3	Albania	1996	Fats, Animals, Raw	4
3	Albania	1996	Fruits, Other	45
3	Albania	1996	Grapes	26
3	Albania	1996	Honey	1
3	Albania	1996	Lemons, Limes	4
3	Albania	1996	Maize	8
3	Albania	1996	Marine Fish, Other	1
3	Albania	1996	Meat, Other	1
3	Albania	1996	Milk, Whole	709
3	Albania	1996	Mutton & Goat Meat	14
3	Albania	1996	Nuts	7
3	Albania	1996	Offals, Edible	9
3	Albania	1996	Oilcrops Oil, Other	1
3	Albania	1996	Olive Oil	2
3	Albania	1996	Olives	9
3	Albania	1996	Onions	32
3	Albania	1996	Oranges, Mandarines	16
3	Albania	1996	Pelagic Fish	1
3	Albania	1996	Pigmeat	9
3	Albania	1996	Potatoes	72
3	Albania	1996	Poultry Meat	18
3	Albania	1996	Rice (Milled Equivalent)	24
3	Albania	1996	Rye	4
3	Albania	1996	Sugar, Refined Equivalent	46
3	Albania	1996	Sunflowerseed Oil	17
3	Albania	1996	Sweeteners, Other	12
3	Albania	1996	Tomatoes	138
3	Albania	1996	Vegetables, Other	271
3	Albania	1996	Wheat	460
3	Albania	1996	Wine	20
3	Albania	1997	Apples	19
3	Albania	1997	Bananas	3
3	Albania	1997	Beans	12
3	Albania	1997	Beer	16
3	Albania	1997	Beverages, Alcoholic	3
3	Albania	1997	Bovine Meat	30
3	Albania	1997	Butter, Ghee	1
3	Albania	1997	Cheese	10
3	Albania	1997	Eggs	13
3	Albania	1997	Fats, Animals, Raw	4
3	Albania	1997	Fruits, Other	36
3	Albania	1997	Grapes	41
3	Albania	1997	Groundnut Oil	1
3	Albania	1997	Honey	1
3	Albania	1997	Lemons, Limes	2
3	Albania	1997	Maize	7
3	Albania	1997	Meat, Other	1
3	Albania	1997	Milk, Whole	591

FAO country code	Country	Reference year	Food item	Gross food supply (g $capita^{-1}$ d^{-1})
3	Albania	1997	Molluscs, Other	1
3	Albania	1997	Mutton & Goat Meat	13
3	Albania	1997	Nuts	6
3	Albania	1997	Offals, Edible	11
3	Albania	1997	Oilcrops Oil, Other	1
3	Albania	1997	Olive Oil	2
3	Albania	1997	Olives	11
3	Albania	1997	Onions	36
3	Albania	1997	Oranges, Mandarines	12
3	Albania	1997	Pelagic Fish	2
3	Albania	1997	Pigmeat	8
3	Albania	1997	Potatoes	69
3	Albania	1997	Poultry Meat	9
3	Albania	1997	Rice (Milled Equivalent)	15
3	Albania	1997	Rye	2
3	Albania	1997	Soyabean Oil	1
3	Albania	1997	Sugar, Refined Equivalent	54
3	Albania	1997	Sunflowerseed Oil	16
3	Albania	1997	Sweeteners, Other	3
3	Albania	1997	Tomatoes	111
3	Albania	1997	Vegetables, Other	261
3	Albania	1997	Wheat	482
3	Albania	1997	Wine	14
3	Albania	1998	Apples	20
3	Albania	1998	Bananas	3
3	Albania	1998	Barley	1
3	Albania	1998	Beans	12
3	Albania	1998	Beer	16
3	Albania	1998	Beverages, Alcoholic	2
3	Albania	1998	Beverages, Fermented	1
3	Albania	1998	Bovine Meat	29
3	Albania	1998	Butter, Ghee	1
3	Albania	1998	Cheese	9
3	Albania	1998	Demersal Fish	1
3	Albania	1998	Eggs	14
3	Albania	1998	Fats, Animals, Raw	4
3	Albania	1998	Fruits, Other	48
3	Albania	1998	Grapes	48
3	Albania	1998	Groundnut Oil	1
3	Albania	1998	Honey	1
3	Albania	1998	Lemons, Limes	1
3	Albania	1998	Maize	7
3	Albania	1998	Marine Fish, Other	1
3	Albania	1998	Meat, Other	1
3	Albania	1998	Milk, Whole	563
3	Albania	1998	Mutton & Goat Meat	14
3	Albania	1998	Nuts	7
3	Albania	1998	Offals, Edible	11
3	Albania	1998	Oilcrops Oil, Other	1
3	Albania	1998	Olive Oil	1
3	Albania	1998	Olives	30
3	Albania	1998	Onions	41
3	Albania	1998	Oranges, Mandarines	11
3	Albania	1998	Pelagic Fish	3
3	Albania	1998	Pigmeat	14
3	Albania	1998	Potatoes	79
3	Albania	1998	Poultry Meat	7
3	Albania	1998	Pulses, Other	1
3	Albania	1998	Rape and Mustard Oil	1
3	Albania	1998	Rice (Milled Equivalent)	21
3	Albania	1998	Rye	2
3	Albania	1998	Soyabean Oil	1
3	Albania	1998	Sugar, Refined Equivalent	48
3	Albania	1998	Sunflowerseed Oil	16
3	Albania	1998	Sweeteners, Other	4
3	Albania	1998	Tomatoes	117
3	Albania	1998	Vegetables, Other	288
3	Albania	1998	Wheat	468
3	Albania	1998	Wine	9
3	Albania	1999	Apples	23
3	Albania	1999	Bananas	7
3	Albania	1999	Beans	13
3	Albania	1999	Beer	29
3	Albania	1999	Beverages, Alcoholic	3
3	Albania	1999	Bovine Meat	30
3	Albania	1999	Butter, Ghee	1
3	Albania	1999	Cheese	9
3	Albania	1999	Cocoa Beans	1
3	Albania	1999	Coffee	2
3	Albania	1999	Dates	1
3	Albania	1999	Demersal Fish	1
3	Albania	1999	Eggs	15
3	Albania	1999	Fats, Animals, Raw	3
3	Albania	1999	Freshwater Fish	1
3	Albania	1999	Fruits, Other	60
3	Albania	1999	Grapes	48
3	Albania	1999	Honey	1
3	Albania	1999	Lemons, Limes	2
3	Albania	1999	Maize	8
3	Albania	1999	Marine Fish, Other	3
3	Albania	1999	Milk, Whole	561
3	Albania	1999	Molluscs, Other	1
3	Albania	1999	Mutton & Goat Meat	15
3	Albania	1999	Nuts	7
3	Albania	1999	Offals, Edible	9
3	Albania	1999	Oilcrops Oil, Other	1
3	Albania	1999	Olive Oil	1

FAO country code	Country	Reference year	Food item	Gross food supply (g *capita*$^{-1}$ d^{-1})
3	Albania	1999	Olives	28
3	Albania	1999	Onions	32
3	Albania	1999	Oranges, Mandarines	17
3	Albania	1999	Pelagic Fish	2
3	Albania	1999	Pigmeat	17
3	Albania	1999	Potatoes	85
3	Albania	1999	Poultry Meat	24
3	Albania	1999	Pulses, Other	1
3	Albania	1999	Rice (Milled Equivalent)	16
3	Albania	1999	Rye	2
3	Albania	1999	Sugar, Refined Equivalent	47
3	Albania	1999	Sunflowerseed Oil	17
3	Albania	1999	Sweeteners, Other	5
3	Albania	1999	Tomatoes	121
3	Albania	1999	Vegetables, Other	270
3	Albania	1999	Wheat	476
3	Albania	1999	Wine	11
3	Albania	2000	Apples	30
3	Albania	2000	Bananas	15
3	Albania	2000	Beans	13
3	Albania	2000	Beer	34
3	Albania	2000	Beverages, Alcoholic	3
3	Albania	2000	Bovine Meat	31
3	Albania	2000	Butter, Ghee	1
3	Albania	2000	Cheese	9
3	Albania	2000	Cocoa Beans	1
3	Albania	2000	Coffee	3
3	Albania	2000	Demersal Fish	1
3	Albania	2000	Eggs	17
3	Albania	2000	Fats, Animals, Raw	4
3	Albania	2000	Freshwater Fish	1
3	Albania	2000	Fruits, Other	62
3	Albania	2000	Grapes	61
3	Albania	2000	Groundnuts (Shelled Eq)	1
3	Albania	2000	Honey	1
3	Albania	2000	Lemons, Limes	1
3	Albania	2000	Maize	14
3	Albania	2000	Marine Fish, Other	1
3	Albania	2000	Milk, Whole	593
3	Albania	2000	Molluscs, Other	1
3	Albania	2000	Mutton & Goat Meat	16
3	Albania	2000	Nuts	6
3	Albania	2000	Offals, Edible	11
3	Albania	2000	Oilcrops Oil, Other	1
3	Albania	2000	Olive Oil	2
3	Albania	2000	Olives	20
3	Albania	2000	Onions	30
3	Albania	2000	Oranges, Mandarines	24
3	Albania	2000	Pelagic Fish	5
3	Albania	2000	Pigmeat	11
3	Albania	2000	Potatoes	80
3	Albania	2000	Poultry Meat	15
3	Albania	2000	Rice (Milled Equivalent)	15
3	Albania	2000	Rye	1
3	Albania	2000	Soyabean Oil	3
3	Albania	2000	Sugar, Refined Equivalent	47
3	Albania	2000	Sunflowerseed Oil	12
3	Albania	2000	Sweeteners, Other	12
3	Albania	2000	Tomatoes	120
3	Albania	2000	Vegetables, Other	280
3	Albania	2000	Wheat	443
3	Albania	2000	Wine	6
3	Albania	2001	Apples	37
3	Albania	2001	Bananas	7
3	Albania	2001	Beans	13
3	Albania	2001	Beer	40
3	Albania	2001	Beverages, Alcoholic	3
3	Albania	2001	Bovine Meat	31
3	Albania	2001	Butter, Ghee	1
3	Albania	2001	Cheese	9
3	Albania	2001	Citrus, Other	1
3	Albania	2001	Cocoa Beans	2
3	Albania	2001	Coffee	4
3	Albania	2001	Demersal Fish	1
3	Albania	2001	Eggs	16
3	Albania	2001	Fats, Animals, Raw	5
3	Albania	2001	Freshwater Fish	1
3	Albania	2001	Fruits, Other	95
3	Albania	2001	Grapes	58
3	Albania	2001	Groundnuts (Shelled Eq)	1
3	Albania	2001	Honey	1
3	Albania	2001	Lemons, Limes	2
3	Albania	2001	Maize	8
3	Albania	2001	Maize Germ Oil	1
3	Albania	2001	Marine Fish, Other	1
3	Albania	2001	Milk, Whole	615
3	Albania	2001	Mutton & Goat Meat	16
3	Albania	2001	Nuts	7
3	Albania	2001	Offals, Edible	10
3	Albania	2001	Oilcrops Oil, Other	1
3	Albania	2001	Olive Oil	2
3	Albania	2001	Olives	22
3	Albania	2001	Onions	30
3	Albania	2001	Oranges, Mandarines	29
3	Albania	2001	Pelagic Fish	5
3	Albania	2001	Pigmeat	18
3	Albania	2001	Potatoes	81

FAO country code	Country	Reference year	Food item	Gross food supply (g $capita^{-1}$ d^{-1})
3	Albania	2001	Poultry Meat	21
3	Albania	2001	Rape and Mustard Oil	1
3	Albania	2001	Rice (Milled Equivalent)	16
3	Albania	2001	Rye	2
3	Albania	2001	Soyabean Oil	1
3	Albania	2001	Sugar, Refined Equivalent	46
3	Albania	2001	Sunflowerseed Oil	13
3	Albania	2001	Sweet Potatoes	1
3	Albania	2001	Sweeteners, Other	15
3	Albania	2001	Tomatoes	130
3	Albania	2001	Vegetables, Other	286
3	Albania	2001	Wheat	445
3	Albania	2001	Wine	8
3	Albania	2002	Apples	34
3	Albania	2002	Bananas	16
3	Albania	2002	Beans	13
3	Albania	2002	Beer	36
3	Albania	2002	Beverages, Alcoholic	2
3	Albania	2002	Bovine Meat	36
3	Albania	2002	Butter, Ghee	1
3	Albania	2002	Cheese	12
3	Albania	2002	Cocoa Beans	1
3	Albania	2002	Coffee	3
3	Albania	2002	Demersal Fish	1
3	Albania	2002	Eggs	14
3	Albania	2002	Fats, Animals, Raw	5
3	Albania	2002	Freshwater Fish	1
3	Albania	2002	Fruits, Other	99
3	Albania	2002	Grapes	67
3	Albania	2002	Groundnuts (Shelled Eq)	1
3	Albania	2002	Honey	1
3	Albania	2002	Lemons, Limes	2
3	Albania	2002	Maize	9
3	Albania	2002	Marine Fish, Other	1
3	Albania	2002	Milk, Whole	624
3	Albania	2002	Mutton & Goat Meat	17
3	Albania	2002	Nuts	7
3	Albania	2002	Offals, Edible	11
3	Albania	2002	Oilcrops Oil, Other	1
3	Albania	2002	Olive Oil	2
3	Albania	2002	Olives	14
3	Albania	2002	Onions	32
3	Albania	2002	Oranges, Mandarines	21
3	Albania	2002	Pelagic Fish	8
3	Albania	2002	Pigmeat	20
3	Albania	2002	Potatoes	81
3	Albania	2002	Poultry Meat	32
3	Albania	2002	Rice (Milled Equivalent)	21
3	Albania	2002	Rye	1
3	Albania	2002	Soyabean Oil	1
3	Albania	2002	Sugar, Refined Equivalent	47
3	Albania	2002	Sunflowerseed Oil	12
3	Albania	2002	Sweet Potatoes	3
3	Albania	2002	Sweeteners, Other	19
3	Albania	2002	Tomatoes	131
3	Albania	2002	Vegetables, Other	308
3	Albania	2002	Wheat	455
3	Albania	2002	Wine	9
3	Albania	2003	Apples	34
3	Albania	2003	Bananas	19
3	Albania	2003	Beans	11
3	Albania	2003	Beer	34
3	Albania	2003	Beverages, Alcoholic	2
3	Albania	2003	Bovine Meat	38
3	Albania	2003	Butter, Ghee	2
3	Albania	2003	Cheese	13
3	Albania	2003	Cocoa Beans	1
3	Albania	2003	Coffee	4
3	Albania	2003	Demersal Fish	1
3	Albania	2003	Eggs	16
3	Albania	2003	Fats, Animals, Raw	5
3	Albania	2003	Freshwater Fish	2
3	Albania	2003	Fruits, Other	93
3	Albania	2003	Grapes	83
3	Albania	2003	Groundnuts (Shelled Eq)	1
3	Albania	2003	Honey	1
3	Albania	2003	Lemons, Limes	1
3	Albania	2003	Maize	9
3	Albania	2003	Milk, Whole	672
3	Albania	2003	Molluscs, Other	1
3	Albania	2003	Mutton & Goat Meat	17
3	Albania	2003	Nuts	7
3	Albania	2003	Offals, Edible	11
3	Albania	2003	Oilcrops Oil, Other	2
3	Albania	2003	Olive Oil	2
3	Albania	2003	Olives	15
3	Albania	2003	Onions	49
3	Albania	2003	Oranges, Mandarines	21
3	Albania	2003	Pelagic Fish	8
3	Albania	2003	Pigmeat	22
3	Albania	2003	Potatoes	82
3	Albania	2003	Poultry Meat	27
3	Albania	2003	Rice (Milled Equivalent)	21
3	Albania	2003	Rye	1
3	Albania	2003	Sugar, Refined Equivalent	47
3	Albania	2003	Sunflowerseed Oil	13
3	Albania	2003	Sweeteners, Other	15

FAO country code	Country	Reference year	Food item	Gross food supply (g *capita*$^{-1}$ d^{-1})
3	Albania	2003	Tomatoes	121
3	Albania	2003	Vegetables, Other	338
3	Albania	2003	Wheat	404
3	Albania	2003	Wine	11
3	Albania	2004	Apples	38
3	Albania	2004	Bananas	14
3	Albania	2004	Beans	13
3	Albania	2004	Beer	44
3	Albania	2004	Beverages, Alcoholic	3
3	Albania	2004	Bovine Meat	41
3	Albania	2004	Butter, Ghee	2
3	Albania	2004	Cheese	13
3	Albania	2004	Cocoa Beans	1
3	Albania	2004	Coffee	3
3	Albania	2004	Dates	5
3	Albania	2004	Demersal Fish	2
3	Albania	2004	Eggs	16
3	Albania	2004	Fats, Animals, Raw	5
3	Albania	2004	Freshwater Fish	2
3	Albania	2004	Fruits, Other	95
3	Albania	2004	Grapefruit	1
3	Albania	2004	Grapes	77
3	Albania	2004	Groundnuts (Shelled Eq)	1
3	Albania	2004	Honey	2
3	Albania	2004	Lemons, Limes	3
3	Albania	2004	Maize	7
3	Albania	2004	Marine Fish, Other	1
3	Albania	2004	Milk, Whole	670
3	Albania	2004	Molluscs, Other	1
3	Albania	2004	Mutton & Goat Meat	18
3	Albania	2004	Nuts	8
3	Albania	2004	Offals, Edible	11
3	Albania	2004	Oilcrops Oil, Other	2
3	Albania	2004	Olive Oil	2
3	Albania	2004	Olives	43
3	Albania	2004	Onions	48
3	Albania	2004	Oranges, Mandarines	25
3	Albania	2004	Pelagic Fish	7
3	Albania	2004	Pigmeat	24
3	Albania	2004	Potatoes	83
3	Albania	2004	Poultry Meat	25
3	Albania	2004	Rice (Milled Equivalent)	20
3	Albania	2004	Rye	1
3	Albania	2004	Sugar, Refined Equivalent	47
3	Albania	2004	Sunflowerseed Oil	14
3	Albania	2004	Sweet Potatoes	2
3	Albania	2004	Sweeteners, Other	2
3	Albania	2004	Tomatoes	123
3	Albania	2004	Vegetables, Other	334
3	Albania	2004	Wheat	383
3	Albania	2004	Wine	13
3	Albania	2005	Apples	41
3	Albania	2005	Bananas	13
3	Albania	2005	Beans	13
3	Albania	2005	Beer	42
3	Albania	2005	Beverages, Alcoholic	3
3	Albania	2005	Bovine Meat	41
3	Albania	2005	Butter, Ghee	2
3	Albania	2005	Cheese	14
3	Albania	2005	Cocoa Beans	1
3	Albania	2005	Coffee	3
3	Albania	2005	Dates	7
3	Albania	2005	Demersal Fish	2
3	Albania	2005	Eggs	16
3	Albania	2005	Fats, Animals, Raw	6
3	Albania	2005	Freshwater Fish	2
3	Albania	2005	Fruits, Other	99
3	Albania	2005	Grapes	89
3	Albania	2005	Groundnuts (Shelled Eq)	1
3	Albania	2005	Honey	2
3	Albania	2005	Lemons, Limes	3
3	Albania	2005	Maize	7
3	Albania	2005	Maize Germ Oil	1
3	Albania	2005	Marine Fish, Other	2
3	Albania	2005	Milk, Whole	674
3	Albania	2005	Molluscs, Other	1
3	Albania	2005	Mutton & Goat Meat	19
3	Albania	2005	Nuts	9
3	Albania	2005	Offals, Edible	11
3	Albania	2005	Oilcrops Oil, Other	2
3	Albania	2005	Olive Oil	1
3	Albania	2005	Olives	19
3	Albania	2005	Onions	48
3	Albania	2005	Oranges, Mandarines	29
3	Albania	2005	Pelagic Fish	7
3	Albania	2005	Pigmeat	25
3	Albania	2005	Potatoes	84
3	Albania	2005	Poultry Meat	26
3	Albania	2005	Pulses, Other	1
3	Albania	2005	Rice (Milled Equivalent)	21
3	Albania	2005	Rye	2
3	Albania	2005	Sugar, Refined Equivalent	48
3	Albania	2005	Sunflowerseed Oil	14
3	Albania	2005	Sweet Potatoes	2
3	Albania	2005	Sweeteners, Other	5
3	Albania	2005	Tomatoes	125
3	Albania	2005	Vegetables, Other	336

FAO countrycode	Country	Reference year	Food item	Gross food supply (g *capita*$^{-1}$ d^{-1})
3	Albania	2005	Wheat	401
3	Albania	2005	Wine	17
3	Albania	2006	Apples	51
3	Albania	2006	Bananas	13
3	Albania	2006	Beans	13
3	Albania	2006	Beer	49
3	Albania	2006	Beverages, Alcoholic	3
3	Albania	2006	Bovine Meat	50
3	Albania	2006	Butter, Ghee	3
3	Albania	2006	Cheese	14
3	Albania	2006	Cocoa Beans	1
3	Albania	2006	Coconuts - Incl Copra	1
3	Albania	2006	Coffee	5
3	Albania	2006	Dates	7
3	Albania	2006	Demersal Fish	2
3	Albania	2006	Eggs	16
3	Albania	2006	Fats, Animals, Raw	6
3	Albania	2006	Freshwater Fish	2
3	Albania	2006	Fruits, Other	102
3	Albania	2006	Grapes	97
3	Albania	2006	Groundnuts (Shelled Eq)	1
3	Albania	2006	Honey	2
3	Albania	2006	Lemons, Limes	4
3	Albania	2006	Maize	8
3	Albania	2006	Marine Fish, Other	2
3	Albania	2006	Milk, Whole	680
3	Albania	2006	Molluscs, Other	1
3	Albania	2006	Mutton & Goat Meat	19
3	Albania	2006	Nuts	11
3	Albania	2006	Offals, Edible	12
3	Albania	2006	Oilcrops Oil, Other	2
3	Albania	2006	Olive Oil	1
3	Albania	2006	Olives	29
3	Albania	2006	Onions	57
3	Albania	2006	Oranges, Mandarines	30
3	Albania	2006	Pelagic Fish	6
3	Albania	2006	Pigmeat	33
3	Albania	2006	Potatoes	86
3	Albania	2006	Poultry Meat	20
3	Albania	2006	Pulses, Other	1
3	Albania	2006	Rice (Milled Equivalent)	26
3	Albania	2006	Rye	2
3	Albania	2006	Sugar, Refined Equivalent	48
3	Albania	2006	Sunflowerseed Oil	14
3	Albania	2006	Sweet Potatoes	7
3	Albania	2006	Sweeteners, Other	13
3	Albania	2006	Tomatoes	137
3	Albania	2006	Vegetables, Other	327
3	Albania	2006	Wheat	364
3	Albania	2006	Wine	16
3	Albania	2007	Apples	49
3	Albania	2007	Bananas	18
3	Albania	2007	Beans	13
3	Albania	2007	Beer	57
3	Albania	2007	Beverages, Alcoholic	3
3	Albania	2007	Bovine Meat	50
3	Albania	2007	Butter, Ghee	4
3	Albania	2007	Cephalopods	1
3	Albania	2007	Cheese	15
3	Albania	2007	Citrus, Other	1
3	Albania	2007	Cocoa Beans	1
3	Albania	2007	Coffee	5
3	Albania	2007	Dates	7
3	Albania	2007	Demersal Fish	2
3	Albania	2007	Eggs	16
3	Albania	2007	Fats, Animals, Raw	7
3	Albania	2007	Freshwater Fish	2
3	Albania	2007	Fruits, Other	105
3	Albania	2007	Grapes	109
3	Albania	2007	Groundnuts (Shelled Eq)	1
3	Albania	2007	Honey	2
3	Albania	2007	Lemons, Limes	4
3	Albania	2007	Maize	8
3	Albania	2007	Maize Germ Oil	1
3	Albania	2007	Marine Fish, Other	1
3	Albania	2007	Milk, Whole	585
3	Albania	2007	Molluscs, Other	1
3	Albania	2007	Mutton & Goat Meat	24
3	Albania	2007	Nuts	11
3	Albania	2007	Offals, Edible	15
3	Albania	2007	Oilcrops Oil, Other	2
3	Albania	2007	Olive Oil	1
3	Albania	2007	Olives	19
3	Albania	2007	Onions	56
3	Albania	2007	Oranges, Mandarines	32
3	Albania	2007	Pelagic Fish	7
3	Albania	2007	Pigmeat	32
3	Albania	2007	Potatoes	86
3	Albania	2007	Poultry Meat	27
3	Albania	2007	Pulses, Other	1
3	Albania	2007	Rice (Milled Equivalent)	23
3	Albania	2007	Rye	2
3	Albania	2007	Sugar, Refined Equivalent	48
3	Albania	2007	Sunflowerseed Oil	14
3	Albania	2007	Sweet Potatoes	11
3	Albania	2007	Sweeteners, Other	21
3	Albania	2007	Tomatoes	132

FAO country code	Country	Reference year	Food item	Gross food supply (g *capita*$^{-1}$ d^{-1})
3	Albania	2007	Vegetables, Other	320
3	Albania	2007	Wheat	368
3	Albania	2007	Wine	18
3	Albania	2008	Apples	50
3	Albania	2008	Bananas	14
3	Albania	2008	Beans	13
3	Albania	2008	Beer	57
3	Albania	2008	Beverages, Alcoholic	3
3	Albania	2008	Bovine Meat	40
3	Albania	2008	Butter, Ghee	3
3	Albania	2008	Cheese	13
3	Albania	2008	Citrus, Other	2
3	Albania	2008	Cocoa Beans	1
3	Albania	2008	Coffee	6
3	Albania	2008	Dates	8
3	Albania	2008	Demersal Fish	2
3	Albania	2008	Eggs	16
3	Albania	2008	Fats, Animals, Raw	7
3	Albania	2008	Freshwater Fish	2
3	Albania	2008	Fruits, Other	101
3	Albania	2008	Grapes	116
3	Albania	2008	Honey	2
3	Albania	2008	Lemons, Limes	4
3	Albania	2008	Maize	7
3	Albania	2008	Maize Germ Oil	1
3	Albania	2008	Marine Fish, Other	1
3	Albania	2008	Milk, Whole	635
3	Albania	2008	Molluscs, Other	1
3	Albania	2008	Mutton & Goat Meat	18
3	Albania	2008	Nuts	9
3	Albania	2008	Offals, Edible	13
3	Albania	2008	Oilcrops Oil, Other	2
3	Albania	2008	Olive Oil	2
3	Albania	2008	Olives	42
3	Albania	2008	Onions	58
3	Albania	2008	Oranges, Mandarines	31
3	Albania	2008	Pelagic Fish	8
3	Albania	2008	Pigmeat	27
3	Albania	2008	Potatoes	86
3	Albania	2008	Poultry Meat	35
3	Albania	2008	Pulses, Other	1
3	Albania	2008	Rice (Milled Equivalent)	17
3	Albania	2008	Rye	2
3	Albania	2008	Sugar, Refined Equivalent	49
3	Albania	2008	Sunflowerseed Oil	12
3	Albania	2008	Sweet Potatoes	10
3	Albania	2008	Sweeteners, Other	47
3	Albania	2008	Tomatoes	132
3	Albania	2008	Vegetables, Other	374
3	Albania	2008	Wheat	377
3	Albania	2008	Wine	16
3	Albania	2009	Apples	49
3	Albania	2009	Bananas	15
3	Albania	2009	Beans	13
3	Albania	2009	Beer	53
3	Albania	2009	Beverages, Alcoholic	3
3	Albania	2009	Bovine Meat	37
3	Albania	2009	Butter, Ghee	3
3	Albania	2009	Cereals, Other	1
3	Albania	2009	Cheese	14
3	Albania	2009	Citrus, Other	2
3	Albania	2009	Cocoa Beans	1
3	Albania	2009	Coffee	6
3	Albania	2009	Dates	8
3	Albania	2009	Demersal Fish	2
3	Albania	2009	Eggs	16
3	Albania	2009	Fats, Animals, Raw	9
3	Albania	2009	Freshwater Fish	2
3	Albania	2009	Fruits, Other	111
3	Albania	2009	Grapes	121
3	Albania	2009	Honey	2
3	Albania	2009	Lemons, Limes	4
3	Albania	2009	Maize	8
3	Albania	2009	Marine Fish, Other	2
3	Albania	2009	Milk, Whole	630
3	Albania	2009	Molluscs, Other	1
3	Albania	2009	Mutton & Goat Meat	19
3	Albania	2009	Nuts	11
3	Albania	2009	Offals, Edible	12
3	Albania	2009	Oilcrops Oil, Other	3
3	Albania	2009	Olive Oil	2
3	Albania	2009	Olives	62
3	Albania	2009	Onions	58
3	Albania	2009	Oranges, Mandarines	36
3	Albania	2009	Pelagic Fish	8
3	Albania	2009	Pigmeat	28
3	Albania	2009	Potatoes	87
3	Albania	2009	Poultry Meat	38
3	Albania	2009	Pulses, Other	1
3	Albania	2009	Rice (Milled Equivalent)	20
3	Albania	2009	Rye	2
3	Albania	2009	Sugar, Refined Equivalent	49
3	Albania	2009	Sunflowerseed Oil	14
3	Albania	2009	Sweet Potatoes	13
3	Albania	2009	Sweeteners, Other	62
3	Albania	2009	Tomatoes	131
3	Albania	2009	Vegetables, Other	365

FAO country code	Country	Reference year	Food item	Gross food supply (g *capita*$^{-1}$ d^{-1})
3	Albania	2009	Wheat	361
3	Albania	2009	Wine	18
3	Albania	2010	Apples	59
3	Albania	2010	Bananas	15
3	Albania	2010	Beans	13
3	Albania	2010	Beer	68
3	Albania	2010	Beverages, Alcoholic	3
3	Albania	2010	Bovine Meat	38
3	Albania	2010	Butter, Ghee	3
3	Albania	2010	Cephalopods	1
3	Albania	2010	Cheese	15
3	Albania	2010	Citrus, Other	2
3	Albania	2010	Cocoa Beans	1
3	Albania	2010	Coffee	6
3	Albania	2010	Crustaceans	1
3	Albania	2010	Dates	10
3	Albania	2010	Demersal Fish	2
3	Albania	2010	Eggs	16
3	Albania	2010	Fats, Animals, Raw	7
3	Albania	2010	Freshwater Fish	3
3	Albania	2010	Fruits, Other	105
3	Albania	2010	Grapefruit	3
3	Albania	2010	Grapes	140
3	Albania	2010	Groundnuts (Shelled Eq)	1
3	Albania	2010	Honey	3
3	Albania	2010	Lemons, Limes	4
3	Albania	2010	Maize	10
3	Albania	2010	Maize Germ Oil	1
3	Albania	2010	Marine Fish, Other	1
3	Albania	2010	Milk, Whole	636
3	Albania	2010	Molluscs, Other	1
3	Albania	2010	Mutton & Goat Meat	19
3	Albania	2010	Nuts	11
3	Albania	2010	Offals, Edible	14
3	Albania	2010	Oilcrops Oil, Other	3
3	Albania	2010	Olive Oil	2
3	Albania	2010	Olives	54
3	Albania	2010	Onions	66
3	Albania	2010	Oranges, Mandarines	41
3	Albania	2010	Pelagic Fish	7
3	Albania	2010	Pigmeat	27
3	Albania	2010	Potatoes	91
3	Albania	2010	Poultry Meat	39
3	Albania	2010	Pulses, Other	1
3	Albania	2010	Rice (Milled Equivalent)	21
3	Albania	2010	Rye	2
3	Albania	2010	Sugar, Refined Equivalent	42
3	Albania	2010	Sunflowerseed Oil	11
3	Albania	2010	Sweet Potatoes	15
3	Albania	2010	Sweeteners, Other	65
3	Albania	2010	Tomatoes	157
3	Albania	2010	Vegetables, Other	427
3	Albania	2010	Wheat	380
3	Albania	2010	Wine	18
3	Albania	2011	Apples	61
3	Albania	2011	Bananas	15
3	Albania	2011	Beans	13
3	Albania	2011	Beer	77
3	Albania	2011	Beverages, Alcoholic	3
3	Albania	2011	Bovine Meat	38
3	Albania	2011	Butter, Ghee	3
3	Albania	2011	Cephalopods	1
3	Albania	2011	Cheese	16
3	Albania	2011	Citrus, Other	1
3	Albania	2011	Cocoa Beans	1
3	Albania	2011	Coffee	6
3	Albania	2011	Cream	1
3	Albania	2011	Crustaceans	1
3	Albania	2011	Dates	10
3	Albania	2011	Demersal Fish	2
3	Albania	2011	Eggs	16
3	Albania	2011	Fats, Animals, Raw	6
3	Albania	2011	Freshwater Fish	2
3	Albania	2011	Fruits, Other	106
3	Albania	2011	Grapefruit	3
3	Albania	2011	Grapes	152
3	Albania	2011	Honey	3
3	Albania	2011	Lemons, Limes	4
3	Albania	2011	Maize	9
3	Albania	2011	Maize Germ Oil	1
3	Albania	2011	Marine Fish, Other	2
3	Albania	2011	Milk, Whole	648
3	Albania	2011	Molluscs, Other	1
3	Albania	2011	Mutton & Goat Meat	19
3	Albania	2011	Nuts	11
3	Albania	2011	Offals, Edible	13
3	Albania	2011	Oilcrops Oil, Other	3
3	Albania	2011	Olive Oil	2
3	Albania	2011	Olives	51
3	Albania	2011	Onions	91
3	Albania	2011	Oranges, Mandarines	37
3	Albania	2011	Pelagic Fish	7
3	Albania	2011	Pigmeat	26
3	Albania	2011	Potatoes	108
3	Albania	2011	Poultry Meat	37
3	Albania	2011	Pulses, Other	1
3	Albania	2011	Rice (Milled Equivalent)	18

FAO country code	Country	Reference year	Food item	Gross food supply (g $capita^{-1}$ d^{-1})
3	Albania	2011	Rye	2
3	Albania	2011	Sugar, Refined Equivalent	42
3	Albania	2011	Sunflowerseed Oil	8
3	Albania	2011	Sweet Potatoes	6
3	Albania	2011	Sweeteners, Other	72
3	Albania	2011	Tomatoes	153
3	Albania	2011	Vegetables, Other	444
3	Albania	2011	Wheat	386
3	Albania	2011	Wine	18
4	Algeria	1992	Apples	6
4	Algeria	1992	Bananas	1
4	Algeria	1992	Barley	59
4	Algeria	1992	Beans	3
4	Algeria	1992	Beer	6
4	Algeria	1992	Bovine Meat	12
4	Algeria	1992	Butter, Ghee	3
4	Algeria	1992	Cheese	1
4	Algeria	1992	Cocoa Beans	1
4	Algeria	1992	Coffee	5
4	Algeria	1992	Dates	23
4	Algeria	1992	Demersal Fish	1
4	Algeria	1992	Eggs	9
4	Algeria	1992	Fats, Animals, Raw	1
4	Algeria	1992	Fruits, Other	26
4	Algeria	1992	Grapes	17
4	Algeria	1992	Groundnut Oil	1
4	Algeria	1992	Lemons, Limes	1
4	Algeria	1992	Maize	5
4	Algeria	1992	Meat, Other	1
4	Algeria	1992	Milk, Whole	183
4	Algeria	1992	Mutton & Goat Meat	16
4	Algeria	1992	Nuts	2
4	Algeria	1992	Offals, Edible	4
4	Algeria	1992	Oilcrops Oil, Other	1
4	Algeria	1992	Olive Oil	5
4	Algeria	1992	Olives	2
4	Algeria	1992	Onions	24
4	Algeria	1992	Oranges, Mandarines	32
4	Algeria	1992	Palm Oil	5
4	Algeria	1992	Peas	2
4	Algeria	1992	Pelagic Fish	8
4	Algeria	1992	Pimento	1
4	Algeria	1992	Potatoes	100
4	Algeria	1992	Poultry Meat	20
4	Algeria	1992	Pulses, Other	11
4	Algeria	1992	Rape and Mustard Oil	8
4	Algeria	1992	Rice (Milled Equivalent)	4
4	Algeria	1992	Soyabean Oil	6
4	Algeria	1992	Sugar, Refined Equivalent	66
4	Algeria	1992	Sunflowerseed Oil	20
4	Algeria	1992	Tea	2
4	Algeria	1992	Tomatoes	66
4	Algeria	1992	Vegetables, Other	126
4	Algeria	1992	Wheat	516
4	Algeria	1993	Apples	6
4	Algeria	1993	Barley	58
4	Algeria	1993	Beans	2
4	Algeria	1993	Beer	7
4	Algeria	1993	Bovine Meat	12
4	Algeria	1993	Butter, Ghee	4
4	Algeria	1993	Coffee	9
4	Algeria	1993	Dates	22
4	Algeria	1993	Demersal Fish	1
4	Algeria	1993	Eggs	9
4	Algeria	1993	Fats, Animals, Raw	1
4	Algeria	1993	Fruits, Other	29
4	Algeria	1993	Grapes	12
4	Algeria	1993	Groundnut Oil	1
4	Algeria	1993	Lemons, Limes	2
4	Algeria	1993	Maize	7
4	Algeria	1993	Meat, Other	1
4	Algeria	1993	Milk, Whole	184
4	Algeria	1993	Mutton & Goat Meat	17
4	Algeria	1993	Nuts	2
4	Algeria	1993	Offals, Edible	4
4	Algeria	1993	Oilcrops Oil, Other	1
4	Algeria	1993	Olive Oil	3
4	Algeria	1993	Olives	2
4	Algeria	1993	Onions	23
4	Algeria	1993	Oranges, Mandarines	31
4	Algeria	1993	Palm Oil	7
4	Algeria	1993	Peas	2
4	Algeria	1993	Pelagic Fish	8
4	Algeria	1993	Pimento	1
4	Algeria	1993	Potatoes	90
4	Algeria	1993	Poultry Meat	20
4	Algeria	1993	Pulses, Other	9
4	Algeria	1993	Rape and Mustard Oil	8
4	Algeria	1993	Rice (Milled Equivalent)	4
4	Algeria	1993	Soyabean Oil	17
4	Algeria	1993	Sugar, Refined Equivalent	68
4	Algeria	1993	Sunflowerseed Oil	8
4	Algeria	1993	Tomatoes	74
4	Algeria	1993	Vegetables, Other	120
4	Algeria	1993	Wheat	517
4	Algeria	1994	Apples	4
4	Algeria	1994	Barley	53

FAO country code	Country	Reference year	Food item	Gross food supply (g *capita*$^{-1}$ d^{-1})
4	Algeria	1994	Beans	4
4	Algeria	1994	Beer	8
4	Algeria	1994	Bovine Meat	12
4	Algeria	1994	Butter, Ghee	2
4	Algeria	1994	Cereals, Other	1
4	Algeria	1994	Cheese	1
4	Algeria	1994	Coffee	10
4	Algeria	1994	Dates	29
4	Algeria	1994	Demersal Fish	1
4	Algeria	1994	Eggs	9
4	Algeria	1994	Fats, Animals, Raw	1
4	Algeria	1994	Fruits, Other	22
4	Algeria	1994	Grapes	7
4	Algeria	1994	Groundnut Oil	1
4	Algeria	1994	Lemons, Limes	2
4	Algeria	1994	Maize	8
4	Algeria	1994	Meat, Other	1
4	Algeria	1994	Milk, Whole	176
4	Algeria	1994	Mutton & Goat Meat	17
4	Algeria	1994	Nuts	2
4	Algeria	1994	Offals, Edible	4
4	Algeria	1994	Oilcrops Oil, Other	1
4	Algeria	1994	Olive Oil	2
4	Algeria	1994	Olives	2
4	Algeria	1994	Onions	22
4	Algeria	1994	Oranges, Mandarines	32
4	Algeria	1994	Palm Oil	2
4	Algeria	1994	Peas	2
4	Algeria	1994	Pelagic Fish	11
4	Algeria	1994	Pimento	1
4	Algeria	1994	Potatoes	66
4	Algeria	1994	Poultry Meat	20
4	Algeria	1994	Pulses, Other	8
4	Algeria	1994	Rape and Mustard Oil	9
4	Algeria	1994	Rice (Milled Equivalent)	4
4	Algeria	1994	Soyabean Oil	17
4	Algeria	1994	Sugar, Refined Equivalent	65
4	Algeria	1994	Sunflowerseed Oil	10
4	Algeria	1994	Sweeteners, Other	1
4	Algeria	1994	Tea	1
4	Algeria	1994	Tomatoes	61
4	Algeria	1994	Vegetables, Other	119
4	Algeria	1994	Wheat	508
4	Algeria	1995	Apples	6
4	Algeria	1995	Bananas	3
4	Algeria	1995	Barley	51
4	Algeria	1995	Beans	6
4	Algeria	1995	Beer	9
4	Algeria	1995	Bovine Meat	12
4	Algeria	1995	Butter, Ghee	2
4	Algeria	1995	Cheese	1
4	Algeria	1995	Coconut Oil	2
4	Algeria	1995	Coffee	5
4	Algeria	1995	Dates	24
4	Algeria	1995	Demersal Fish	1
4	Algeria	1995	Eggs	11
4	Algeria	1995	Fats, Animals, Raw	1
4	Algeria	1995	Fruits, Other	25
4	Algeria	1995	Grapes	12
4	Algeria	1995	Groundnut Oil	1
4	Algeria	1995	Lemons, Limes	1
4	Algeria	1995	Maize	9
4	Algeria	1995	Meat, Other	1
4	Algeria	1995	Milk, Whole	155
4	Algeria	1995	Mutton & Goat Meat	17
4	Algeria	1995	Nuts	2
4	Algeria	1995	Offals, Edible	3
4	Algeria	1995	Olive Oil	2
4	Algeria	1995	Olives	1
4	Algeria	1995	Onions	28
4	Algeria	1995	Oranges, Mandarines	28
4	Algeria	1995	Palm Oil	1
4	Algeria	1995	Peas	1
4	Algeria	1995	Pelagic Fish	9
4	Algeria	1995	Pimento	1
4	Algeria	1995	Potatoes	101
4	Algeria	1995	Poultry Meat	19
4	Algeria	1995	Pulses, Other	8
4	Algeria	1995	Rape and Mustard Oil	8
4	Algeria	1995	Rice (Milled Equivalent)	3
4	Algeria	1995	Soyabean Oil	11
4	Algeria	1995	Sugar, Refined Equivalent	65
4	Algeria	1995	Sunflowerseed Oil	20
4	Algeria	1995	Tomatoes	84
4	Algeria	1995	Vegetables, Other	118
4	Algeria	1995	Wheat	508
4	Algeria	1996	Apples	7
4	Algeria	1996	Bananas	1
4	Algeria	1996	Barley	59
4	Algeria	1996	Beans	4
4	Algeria	1996	Beer	7
4	Algeria	1996	Bovine Meat	12
4	Algeria	1996	Butter, Ghee	1
4	Algeria	1996	Cereals, Other	3
4	Algeria	1996	Cheese	1
4	Algeria	1996	Cocoa Beans	1
4	Algeria	1996	Coffee	7

FAO country code	Country	Reference year	Food item	Gross food supply (g $capita^{-1}$ d^{-1})
4	Algeria	1996	Dates	30
4	Algeria	1996	Demersal Fish	1
4	Algeria	1996	Eggs	6
4	Algeria	1996	Fats, Animals, Raw	1
4	Algeria	1996	Fruits, Other	28
4	Algeria	1996	Grapes	13
4	Algeria	1996	Groundnut Oil	1
4	Algeria	1996	Lemons, Limes	1
4	Algeria	1996	Maize	18
4	Algeria	1996	Meat, Other	1
4	Algeria	1996	Milk, Whole	153
4	Algeria	1996	Mutton & Goat Meat	18
4	Algeria	1996	Nuts	3
4	Algeria	1996	Offals, Edible	3
4	Algeria	1996	Oilcrops Oil, Other	1
4	Algeria	1996	Olive Oil	5
4	Algeria	1996	Olives	3
4	Algeria	1996	Onions	27
4	Algeria	1996	Oranges, Mandarines	28
4	Algeria	1996	Peas	2
4	Algeria	1996	Pelagic Fish	7
4	Algeria	1996	Pimento	1
4	Algeria	1996	Potatoes	93
4	Algeria	1996	Poultry Meat	20
4	Algeria	1996	Pulses, Other	11
4	Algeria	1996	Rape and Mustard Oil	7
4	Algeria	1996	Rice (Milled Equivalent)	4
4	Algeria	1996	Sugar, Refined Equivalent	66
4	Algeria	1996	Sunflowerseed Oil	24
4	Algeria	1996	Tomatoes	62
4	Algeria	1996	Vegetables, Other	118
4	Algeria	1996	Wheat	513
4	Algeria	1997	Apples	6
4	Algeria	1997	Barley	53
4	Algeria	1997	Beans	6
4	Algeria	1997	Beer	6
4	Algeria	1997	Bovine Meat	10
4	Algeria	1997	Butter, Ghee	1
4	Algeria	1997	Cheese	1
4	Algeria	1997	Cocoa Beans	1
4	Algeria	1997	Coffee	8
4	Algeria	1997	Dates	25
4	Algeria	1997	Demersal Fish	1
4	Algeria	1997	Eggs	7
4	Algeria	1997	Fats, Animals, Raw	1
4	Algeria	1997	Fruits, Other	22
4	Algeria	1997	Grapes	13
4	Algeria	1997	Groundnut Oil	1
4	Algeria	1997	Lemons, Limes	1
4	Algeria	1997	Maize	12
4	Algeria	1997	Meat, Other	1
4	Algeria	1997	Milk, Whole	162
4	Algeria	1997	Mutton & Goat Meat	16
4	Algeria	1997	Nuts	2
4	Algeria	1997	Offals, Edible	3
4	Algeria	1997	Oilcrops Oil, Other	1
4	Algeria	1997	Olive Oil	4
4	Algeria	1997	Olives	4
4	Algeria	1997	Onions	30
4	Algeria	1997	Oranges, Mandarines	29
4	Algeria	1997	Palm Oil	1
4	Algeria	1997	Peas	2
4	Algeria	1997	Pelagic Fish	7
4	Algeria	1997	Pimento	1
4	Algeria	1997	Potatoes	89
4	Algeria	1997	Poultry Meat	20
4	Algeria	1997	Pulses, Other	10
4	Algeria	1997	Rape and Mustard Oil	9
4	Algeria	1997	Rice (Milled Equivalent)	5
4	Algeria	1997	Sorghum	1
4	Algeria	1997	Soyabean Oil	1
4	Algeria	1997	Sugar, Refined Equivalent	70
4	Algeria	1997	Sunflowerseed Oil	25
4	Algeria	1997	Tomatoes	59
4	Algeria	1997	Vegetables, Other	115
4	Algeria	1997	Wheat	490
4	Algeria	1998	Apples	6
4	Algeria	1998	Barley	61
4	Algeria	1998	Beans	5
4	Algeria	1998	Beer	6
4	Algeria	1998	Bovine Meat	11
4	Algeria	1998	Butter, Ghee	1
4	Algeria	1998	Cheese	1
4	Algeria	1998	Cocoa Beans	1
4	Algeria	1998	Coffee	8
4	Algeria	1998	Dates	32
4	Algeria	1998	Demersal Fish	1
4	Algeria	1998	Eggs	8
4	Algeria	1998	Fats, Animals, Raw	1
4	Algeria	1998	Fruits, Other	23
4	Algeria	1998	Grapes	8
4	Algeria	1998	Lemons, Limes	2
4	Algeria	1998	Maize	3
4	Algeria	1998	Meat, Other	1
4	Algeria	1998	Milk, Whole	181
4	Algeria	1998	Mutton & Goat Meat	16
4	Algeria	1998	Nuts	2

FAO country code	Country	Reference year	Food item	Gross food supply (g $capita^{-1}$ d^{-1})
4	Algeria	1998	Offals, Edible	3
4	Algeria	1998	Oilcrops Oil, Other	1
4	Algeria	1998	Olive Oil	1
4	Algeria	1998	Olives	1
4	Algeria	1998	Onions	33
4	Algeria	1998	Oranges, Mandarines	33
4	Algeria	1998	Palm Oil	1
4	Algeria	1998	Peas	2
4	Algeria	1998	Pelagic Fish	7
4	Algeria	1998	Pimento	1
4	Algeria	1998	Potatoes	91
4	Algeria	1998	Poultry Meat	22
4	Algeria	1998	Pulses, Other	11
4	Algeria	1998	Rape and Mustard Oil	8
4	Algeria	1998	Rice (Milled Equivalent)	2
4	Algeria	1998	Soyabean Oil	6
4	Algeria	1998	Sugar, Refined Equivalent	48
4	Algeria	1998	Sunflowerseed Oil	23
4	Algeria	1998	Sweeteners, Other	1
4	Algeria	1998	Tea	1
4	Algeria	1998	Tomatoes	63
4	Algeria	1998	Vegetables, Other	120
4	Algeria	1998	Wheat	541
4	Algeria	1999	Apples	7
4	Algeria	1999	Barley	52
4	Algeria	1999	Beans	8
4	Algeria	1999	Beer	6
4	Algeria	1999	Bovine Meat	12
4	Algeria	1999	Butter, Ghee	1
4	Algeria	1999	Cheese	2
4	Algeria	1999	Cocoa Beans	1
4	Algeria	1999	Coffee	8
4	Algeria	1999	Dates	35
4	Algeria	1999	Demersal Fish	1
4	Algeria	1999	Eggs	7
4	Algeria	1999	Fats, Animals, Raw	1
4	Algeria	1999	Fruits, Other	28
4	Algeria	1999	Grapes	11
4	Algeria	1999	Lemons, Limes	2
4	Algeria	1999	Maize	9
4	Algeria	1999	Meat, Other	1
4	Algeria	1999	Milk, Whole	184
4	Algeria	1999	Mutton & Goat Meat	16
4	Algeria	1999	Nuts	2
4	Algeria	1999	Offals, Edible	3
4	Algeria	1999	Oilcrops Oil, Other	2
4	Algeria	1999	Olive Oil	5
4	Algeria	1999	Olives	4
4	Algeria	1999	Onions	32
4	Algeria	1999	Oranges, Mandarines	35
4	Algeria	1999	Palm Oil	1
4	Algeria	1999	Peas	3
4	Algeria	1999	Pelagic Fish	8
4	Algeria	1999	Pimento	1
4	Algeria	1999	Potatoes	79
4	Algeria	1999	Poultry Meat	21
4	Algeria	1999	Pulses, Other	11
4	Algeria	1999	Rape and Mustard Oil	7
4	Algeria	1999	Rice (Milled Equivalent)	4
4	Algeria	1999	Soyabean Oil	1
4	Algeria	1999	Sugar, Refined Equivalent	54
4	Algeria	1999	Sunflowerseed Oil	27
4	Algeria	1999	Sweeteners, Other	1
4	Algeria	1999	Tomatoes	78
4	Algeria	1999	Vegetables, Other	126
4	Algeria	1999	Wheat	539
4	Algeria	2000	Apples	8
4	Algeria	2000	Barley	25
4	Algeria	2000	Beans	3
4	Algeria	2000	Beer	9
4	Algeria	2000	Bovine Meat	12
4	Algeria	2000	Butter, Ghee	1
4	Algeria	2000	Cheese	2
4	Algeria	2000	Cocoa Beans	1
4	Algeria	2000	Coffee	9
4	Algeria	2000	Dates	30
4	Algeria	2000	Demersal Fish	1
4	Algeria	2000	Eggs	7
4	Algeria	2000	Fats, Animals, Raw	1
4	Algeria	2000	Fruits, Other	27
4	Algeria	2000	Grapes	13
4	Algeria	2000	Lemons, Limes	2
4	Algeria	2000	Maize	49
4	Algeria	2000	Marine Fish, Other	1
4	Algeria	2000	Meat, Other	1
4	Algeria	2000	Milk, Whole	185
4	Algeria	2000	Mutton & Goat Meat	16
4	Algeria	2000	Nuts	2
4	Algeria	2000	Offals, Edible	3
4	Algeria	2000	Oilcrops Oil, Other	2
4	Algeria	2000	Olive Oil	3
4	Algeria	2000	Olives	2
4	Algeria	2000	Onions	26
4	Algeria	2000	Oranges, Mandarines	33
4	Algeria	2000	Palm Oil	2
4	Algeria	2000	Peas	1
4	Algeria	2000	Pelagic Fish	8

FAO country code	Country	Reference year	Food item	Gross food supply (g $capita^{-1}$ d^{-1})
4	Algeria	2000	Pimento	1
4	Algeria	2000	Potatoes	102
4	Algeria	2000	Poultry Meat	21
4	Algeria	2000	Pulses, Other	11
4	Algeria	2000	Rape and Mustard Oil	7
4	Algeria	2000	Rice (Milled Equivalent)	5
4	Algeria	2000	Soyabean Oil	1
4	Algeria	2000	Sugar, Refined Equivalent	72
4	Algeria	2000	Sunflowerseed Oil	25
4	Algeria	2000	Sweeteners, Other	1
4	Algeria	2000	Tea	1
4	Algeria	2000	Tomatoes	66
4	Algeria	2000	Vegetables, Other	116
4	Algeria	2000	Wheat	494
4	Algeria	2001	Apples	9
4	Algeria	2001	Bananas	7
4	Algeria	2001	Barley	34
4	Algeria	2001	Beans	4
4	Algeria	2001	Beer	9
4	Algeria	2001	Bovine Meat	9
4	Algeria	2001	Butter, Ghee	1
4	Algeria	2001	Cheese	1
4	Algeria	2001	Cocoa Beans	1
4	Algeria	2001	Coffee	7
4	Algeria	2001	Dates	35
4	Algeria	2001	Demersal Fish	1
4	Algeria	2001	Eggs	8
4	Algeria	2001	Fats, Animals, Raw	1
4	Algeria	2001	Fruits, Other	27
4	Algeria	2001	Grapes	13
4	Algeria	2001	Groundnut Oil	1
4	Algeria	2001	Lemons, Limes	2
4	Algeria	2001	Maize	40
4	Algeria	2001	Marine Fish, Other	1
4	Algeria	2001	Meat, Other	1
4	Algeria	2001	Milk, Whole	211
4	Algeria	2001	Mutton & Goat Meat	15
4	Algeria	2001	Nuts	3
4	Algeria	2001	Offals, Edible	3
4	Algeria	2001	Oilcrops Oil, Other	2
4	Algeria	2001	Olive Oil	2
4	Algeria	2001	Olives	2
4	Algeria	2001	Onions	35
4	Algeria	2001	Oranges, Mandarines	35
4	Algeria	2001	Palm Oil	5
4	Algeria	2001	Peas	1
4	Algeria	2001	Pelagic Fish	10
4	Algeria	2001	Pimento	1
4	Algeria	2001	Potatoes	76
4	Algeria	2001	Poultry Meat	21
4	Algeria	2001	Pulses, Other	10
4	Algeria	2001	Rape and Mustard Oil	6
4	Algeria	2001	Rice (Milled Equivalent)	2
4	Algeria	2001	Rye	4
4	Algeria	2001	Soyabean Oil	1
4	Algeria	2001	Sugar, Refined Equivalent	70
4	Algeria	2001	Sunflowerseed Oil	27
4	Algeria	2001	Sweeteners, Other	1
4	Algeria	2001	Tea	1
4	Algeria	2001	Tomatoes	65
4	Algeria	2001	Vegetables, Other	124
4	Algeria	2001	Wheat	505
4	Algeria	2001	Whey	1
4	Algeria	2002	Apples	13
4	Algeria	2002	Bananas	18
4	Algeria	2002	Barley	41
4	Algeria	2002	Beans	4
4	Algeria	2002	Beer	9
4	Algeria	2002	Bovine Meat	11
4	Algeria	2002	Butter, Ghee	1
4	Algeria	2002	Cheese	2
4	Algeria	2002	Cocoa Beans	1
4	Algeria	2002	Coffee	9
4	Algeria	2002	Dates	33
4	Algeria	2002	Demersal Fish	1
4	Algeria	2002	Eggs	12
4	Algeria	2002	Fats, Animals, Raw	1
4	Algeria	2002	Fruits, Other	34
4	Algeria	2002	Grapes	17
4	Algeria	2002	Groundnut Oil	2
4	Algeria	2002	Lemons, Limes	3
4	Algeria	2002	Maize	37
4	Algeria	2002	Marine Fish, Other	1
4	Algeria	2002	Meat, Other	1
4	Algeria	2002	Milk, Whole	194
4	Algeria	2002	Mutton & Goat Meat	15
4	Algeria	2002	Nuts	3
4	Algeria	2002	Offals, Edible	3
4	Algeria	2002	Oilcrops Oil, Other	2
4	Algeria	2002	Olive Oil	2
4	Algeria	2002	Olives	2
4	Algeria	2002	Onions	36
4	Algeria	2002	Oranges, Mandarines	39
4	Algeria	2002	Palm Oil	7
4	Algeria	2002	Peas	1
4	Algeria	2002	Pelagic Fish	10
4	Algeria	2002	Pimento	1

FAO countrycode	Country	Reference year	Food item	Gross food supply (g *capita*$^{-1}$ d^{-1})
4	Algeria	2002	Potatoes	107
4	Algeria	2002	Poultry Meat	21
4	Algeria	2002	Pulses, Other	10
4	Algeria	2002	Rape and Mustard Oil	13
4	Algeria	2002	Rice (Milled Equivalent)	8
4	Algeria	2002	Soyabean Oil	2
4	Algeria	2002	Spices, Other	1
4	Algeria	2002	Sugar, Refined Equivalent	73
4	Algeria	2002	Sunflowerseed Oil	12
4	Algeria	2002	Sweeteners, Other	1
4	Algeria	2002	Tea	1
4	Algeria	2002	Tomatoes	64
4	Algeria	2002	Vegetables, Other	128
4	Algeria	2002	Wheat	507
4	Algeria	2003	Apples	15
4	Algeria	2003	Bananas	17
4	Algeria	2003	Barley	41
4	Algeria	2003	Beans	4
4	Algeria	2003	Beer	10
4	Algeria	2003	Bovine Meat	14
4	Algeria	2003	Butter, Ghee	1
4	Algeria	2003	Cheese	2
4	Algeria	2003	Cocoa Beans	1
4	Algeria	2003	Coffee	9
4	Algeria	2003	Dates	39
4	Algeria	2003	Demersal Fish	1
4	Algeria	2003	Eggs	12
4	Algeria	2003	Fats, Animals, Raw	1
4	Algeria	2003	Fruits, Other	38
4	Algeria	2003	Grapes	17
4	Algeria	2003	Groundnut Oil	2
4	Algeria	2003	Lemons, Limes	3
4	Algeria	2003	Maize	29
4	Algeria	2003	Marine Fish, Other	1
4	Algeria	2003	Meat, Other	1
4	Algeria	2003	Milk, Whole	199
4	Algeria	2003	Mutton & Goat Meat	15
4	Algeria	2003	Nuts	4
4	Algeria	2003	Offals, Edible	3
4	Algeria	2003	Oilcrops Oil, Other	2
4	Algeria	2003	Olive Oil	2
4	Algeria	2003	Olives	1
4	Algeria	2003	Onions	44
4	Algeria	2003	Oranges, Mandarines	41
4	Algeria	2003	Palm Oil	3
4	Algeria	2003	Peas	1
4	Algeria	2003	Pelagic Fish	11
4	Algeria	2003	Pimento	1
4	Algeria	2003	Potatoes	140
4	Algeria	2003	Poultry Meat	22
4	Algeria	2003	Pulses, Other	12
4	Algeria	2003	Rape and Mustard Oil	4
4	Algeria	2003	Rice (Milled Equivalent)	5
4	Algeria	2003	Soyabean Oil	1
4	Algeria	2003	Sugar, Refined Equivalent	75
4	Algeria	2003	Sunflowerseed Oil	20
4	Algeria	2003	Sweeteners, Other	1
4	Algeria	2003	Tea	1
4	Algeria	2003	Tomatoes	71
4	Algeria	2003	Vegetables, Other	153
4	Algeria	2003	Wheat	530
4	Algeria	2004	Apples	18
4	Algeria	2004	Bananas	15
4	Algeria	2004	Barley	41
4	Algeria	2004	Beans	4
4	Algeria	2004	Beer	9
4	Algeria	2004	Bovine Meat	18
4	Algeria	2004	Butter, Ghee	1
4	Algeria	2004	Cheese	2
4	Algeria	2004	Cocoa Beans	1
4	Algeria	2004	Coffee	11
4	Algeria	2004	Dates	35
4	Algeria	2004	Demersal Fish	1
4	Algeria	2004	Eggs	13
4	Algeria	2004	Fats, Animals, Raw	1
4	Algeria	2004	Fruits, Other	39
4	Algeria	2004	Grapes	16
4	Algeria	2004	Groundnut Oil	2
4	Algeria	2004	Lemons, Limes	3
4	Algeria	2004	Maize	26
4	Algeria	2004	Meat, Other	1
4	Algeria	2004	Milk, Whole	228
4	Algeria	2004	Mutton & Goat Meat	16
4	Algeria	2004	Nuts	4
4	Algeria	2004	Offals, Edible	3
4	Algeria	2004	Oilcrops Oil, Other	4
4	Algeria	2004	Olive Oil	5
4	Algeria	2004	Olives	7
4	Algeria	2004	Onions	51
4	Algeria	2004	Oranges, Mandarines	44
4	Algeria	2004	Palm Oil	3
4	Algeria	2004	Peas	2
4	Algeria	2004	Pelagic Fish	10
4	Algeria	2004	Pimento	1
4	Algeria	2004	Potatoes	139
4	Algeria	2004	Poultry Meat	22
4	Algeria	2004	Pulses, Other	10

FAO country code	Country	Reference year	Food item	Gross food supply (g $capita^{-1}$ d^{-1})
4	Algeria	2004	Rape and Mustard Oil	5
4	Algeria	2004	Rice (Milled Equivalent)	5
4	Algeria	2004	Soyabean Oil	4
4	Algeria	2004	Sugar, Refined Equivalent	76
4	Algeria	2004	Sunflowerseed Oil	12
4	Algeria	2004	Sweeteners, Other	1
4	Algeria	2004	Tea	1
4	Algeria	2004	Tomatoes	92
4	Algeria	2004	Vegetables, Other	176
4	Algeria	2004	Wheat	521
4	Algeria	2004	Whey	2
4	Algeria	2005	Apples	21
4	Algeria	2005	Bananas	11
4	Algeria	2005	Barley	40
4	Algeria	2005	Beans	4
4	Algeria	2005	Beer	10
4	Algeria	2005	Bovine Meat	18
4	Algeria	2005	Butter, Ghee	1
4	Algeria	2005	Cheese	2
4	Algeria	2005	Cocoa Beans	1
4	Algeria	2005	Coconuts - Incl Copra	1
4	Algeria	2005	Coffee	9
4	Algeria	2005	Dates	39
4	Algeria	2005	Demersal Fish	1
4	Algeria	2005	Eggs	12
4	Algeria	2005	Fats, Animals, Raw	1
4	Algeria	2005	Fruits, Other	48
4	Algeria	2005	Grapes	19
4	Algeria	2005	Groundnut Oil	2
4	Algeria	2005	Lemons, Limes	4
4	Algeria	2005	Maize	26
4	Algeria	2005	Meat, Other	1
4	Algeria	2005	Milk, Whole	236
4	Algeria	2005	Mutton & Goat Meat	17
4	Algeria	2005	Nuts	4
4	Algeria	2005	Offals, Edible	3
4	Algeria	2005	Oilcrops Oil, Other	4
4	Algeria	2005	Olive Oil	3
4	Algeria	2005	Olives	4
4	Algeria	2005	Onions	53
4	Algeria	2005	Oranges, Mandarines	45
4	Algeria	2005	Peas	1
4	Algeria	2005	Pelagic Fish	11
4	Algeria	2005	Pimento	1
4	Algeria	2005	Potatoes	154
4	Algeria	2005	Poultry Meat	22
4	Algeria	2005	Pulses, Other	13
4	Algeria	2005	Rape and Mustard Oil	3
4	Algeria	2005	Rice (Milled Equivalent)	4
4	Algeria	2005	Soyabean Oil	14
4	Algeria	2005	Sugar, Refined Equivalent	68
4	Algeria	2005	Sunflowerseed Oil	6
4	Algeria	2005	Sweeteners, Other	1
4	Algeria	2005	Tea	1
4	Algeria	2005	Tomatoes	81
4	Algeria	2005	Vegetables, Other	187
4	Algeria	2005	Wheat	520
4	Algeria	2005	Whey	2
4	Algeria	2006	Apples	28
4	Algeria	2006	Bananas	11
4	Algeria	2006	Barley	47
4	Algeria	2006	Beans	4
4	Algeria	2006	Beer	11
4	Algeria	2006	Bovine Meat	15
4	Algeria	2006	Butter, Ghee	1
4	Algeria	2006	Cheese	2
4	Algeria	2006	Cocoa Beans	1
4	Algeria	2006	Coffee	9
4	Algeria	2006	Dates	37
4	Algeria	2006	Demersal Fish	1
4	Algeria	2006	Eggs	12
4	Algeria	2006	Fats, Animals, Raw	1
4	Algeria	2006	Fruits, Other	57
4	Algeria	2006	Grapes	22
4	Algeria	2006	Groundnut Oil	1
4	Algeria	2006	Lemons, Limes	3
4	Algeria	2006	Maize	52
4	Algeria	2006	Meat, Other	1
4	Algeria	2006	Milk, Whole	259
4	Algeria	2006	Mutton & Goat Meat	16
4	Algeria	2006	Nuts	5
4	Algeria	2006	Offals, Edible	3
4	Algeria	2006	Oilcrops Oil, Other	5
4	Algeria	2006	Olive Oil	3
4	Algeria	2006	Olives	4
4	Algeria	2006	Onions	53
4	Algeria	2006	Oranges, Mandarines	48
4	Algeria	2006	Palm Oil	2
4	Algeria	2006	Peas	1
4	Algeria	2006	Pelagic Fish	13
4	Algeria	2006	Pimento	1
4	Algeria	2006	Potatoes	157
4	Algeria	2006	Poultry Meat	21
4	Algeria	2006	Pulses, Other	10
4	Algeria	2006	Rape and Mustard Oil	4
4	Algeria	2006	Rice (Milled Equivalent)	5
4	Algeria	2006	Soyabean Oil	16

FAO country code	Country	Reference year	Food item	Gross food supply (g *capita*$^{-1}$ d^{-1})
4	Algeria	2006	Spices, Other	1
4	Algeria	2006	Sugar, Refined Equivalent	71
4	Algeria	2006	Sunflowerseed Oil	6
4	Algeria	2006	Sweeteners, Other	1
4	Algeria	2006	Tea	1
4	Algeria	2006	Tomatoes	70
4	Algeria	2006	Vegetables, Other	179
4	Algeria	2006	Wheat	492
4	Algeria	2006	Whey	2
4	Algeria	2007	Apples	21
4	Algeria	2007	Bananas	11
4	Algeria	2007	Barley	50
4	Algeria	2007	Beans	4
4	Algeria	2007	Beer	11
4	Algeria	2007	Bovine Meat	15
4	Algeria	2007	Butter, Ghee	1
4	Algeria	2007	Cheese	2
4	Algeria	2007	Cocoa Beans	2
4	Algeria	2007	Coconuts - Incl Copra	1
4	Algeria	2007	Coffee	9
4	Algeria	2007	Dates	39
4	Algeria	2007	Demersal Fish	1
4	Algeria	2007	Eggs	13
4	Algeria	2007	Fats, Animals, Raw	1
4	Algeria	2007	Fruits, Other	42
4	Algeria	2007	Grapes	17
4	Algeria	2007	Groundnut Oil	2
4	Algeria	2007	Lemons, Limes	3
4	Algeria	2007	Maize	58
4	Algeria	2007	Marine Fish, Other	1
4	Algeria	2007	Meat, Other	1
4	Algeria	2007	Milk, Whole	241
4	Algeria	2007	Mutton & Goat Meat	16
4	Algeria	2007	Nuts	4
4	Algeria	2007	Offals, Edible	3
4	Algeria	2007	Oilcrops Oil, Other	4
4	Algeria	2007	Olive Oil	2
4	Algeria	2007	Olives	2
4	Algeria	2007	Onions	61
4	Algeria	2007	Oranges, Mandarines	49
4	Algeria	2007	Palm Oil	1
4	Algeria	2007	Peas	1
4	Algeria	2007	Pelagic Fish	12
4	Algeria	2007	Pimento	1
4	Algeria	2007	Potatoes	114
4	Algeria	2007	Poultry Meat	21
4	Algeria	2007	Pulses, Other	12
4	Algeria	2007	Rape and Mustard Oil	4
4	Algeria	2007	Rice (Milled Equivalent)	6
4	Algeria	2007	Soyabean Oil	21
4	Algeria	2007	Spices, Other	1
4	Algeria	2007	Sugar, Refined Equivalent	79
4	Algeria	2007	Sunflowerseed Oil	3
4	Algeria	2007	Sweeteners, Other	1
4	Algeria	2007	Tea	1
4	Algeria	2007	Tomatoes	53
4	Algeria	2007	Vegetables, Other	181
4	Algeria	2007	Wheat	485
4	Algeria	2007	Whey	3
4	Algeria	2008	Apples	26
4	Algeria	2008	Bananas	11
4	Algeria	2008	Barley	38
4	Algeria	2008	Beans	5
4	Algeria	2008	Beer	12
4	Algeria	2008	Bovine Meat	15
4	Algeria	2008	Butter, Ghee	1
4	Algeria	2008	Cheese	1
4	Algeria	2008	Cocoa Beans	2
4	Algeria	2008	Coffee	10
4	Algeria	2008	Dates	41
4	Algeria	2008	Demersal Fish	1
4	Algeria	2008	Eggs	12
4	Algeria	2008	Fats, Animals, Raw	1
4	Algeria	2008	Fruits, Other	54
4	Algeria	2008	Grapes	26
4	Algeria	2008	Groundnut Oil	2
4	Algeria	2008	Lemons, Limes	3
4	Algeria	2008	Maize	52
4	Algeria	2008	Marine Fish, Other	1
4	Algeria	2008	Meat, Other	1
4	Algeria	2008	Milk, Whole	232
4	Algeria	2008	Mutton & Goat Meat	15
4	Algeria	2008	Nuts	5
4	Algeria	2008	Offals, Edible	3
4	Algeria	2008	Oilcrops Oil, Other	5
4	Algeria	2008	Olive Oil	3
4	Algeria	2008	Olives	1
4	Algeria	2008	Onions	55
4	Algeria	2008	Oranges, Mandarines	48
4	Algeria	2008	Palm Oil	2
4	Algeria	2008	Peas	1
4	Algeria	2008	Pelagic Fish	10
4	Algeria	2008	Pimento	1
4	Algeria	2008	Potatoes	150
4	Algeria	2008	Poultry Meat	21
4	Algeria	2008	Pulses, Other	10
4	Algeria	2008	Rape and Mustard Oil	2

FAO country code	Country	Reference year	Food item	Gross food supply (g $capita^{-1}$ d^{-1})
4	Algeria	2008	Rice (Milled Equivalent)	7
4	Algeria	2008	Soyabean Oil	20
4	Algeria	2008	Spices, Other	1
4	Algeria	2008	Sugar, Refined Equivalent	70
4	Algeria	2008	Sunflowerseed Oil	3
4	Algeria	2008	Sweeteners, Other	2
4	Algeria	2008	Tea	1
4	Algeria	2008	Tomatoes	47
4	Algeria	2008	Vegetables, Other	182
4	Algeria	2008	Wheat	503
4	Algeria	2008	Whey	3
4	Algeria	2009	Apples	28
4	Algeria	2009	Bananas	12
4	Algeria	2009	Barley	38
4	Algeria	2009	Beans	4
4	Algeria	2009	Beer	11
4	Algeria	2009	Bovine Meat	16
4	Algeria	2009	Butter, Ghee	1
4	Algeria	2009	Cheese	2
4	Algeria	2009	Citrus, Other	1
4	Algeria	2009	Cocoa Beans	2
4	Algeria	2009	Coconuts - Incl Copra	1
4	Algeria	2009	Coffee	9
4	Algeria	2009	Dates	43
4	Algeria	2009	Demersal Fish	1
4	Algeria	2009	Eggs	13
4	Algeria	2009	Fats, Animals, Raw	1
4	Algeria	2009	Fruits, Other	60
4	Algeria	2009	Grapes	35
4	Algeria	2009	Groundnut Oil	2
4	Algeria	2009	Groundnuts (Shelled Eq)	1
4	Algeria	2009	Lemons, Limes	4
4	Algeria	2009	Maize	36
4	Algeria	2009	Marine Fish, Other	1
4	Algeria	2009	Meat, Other	1
4	Algeria	2009	Milk, Whole	274
4	Algeria	2009	Mutton & Goat Meat	16
4	Algeria	2009	Nuts	6
4	Algeria	2009	Offals, Edible	3
4	Algeria	2009	Oilcrops Oil, Other	5
4	Algeria	2009	Olive Oil	4
4	Algeria	2009	Olives	6
4	Algeria	2009	Onions	70
4	Algeria	2009	Oranges, Mandarines	57
4	Algeria	2009	Palm Oil	2
4	Algeria	2009	Peas	1
4	Algeria	2009	Pelagic Fish	10
4	Algeria	2009	Pimento	1
4	Algeria	2009	Potatoes	178
4	Algeria	2009	Poultry Meat	20
4	Algeria	2009	Pulses, Other	11
4	Algeria	2009	Rape and Mustard Oil	2
4	Algeria	2009	Rice (Milled Equivalent)	6
4	Algeria	2009	Soyabean Oil	16
4	Algeria	2009	Spices, Other	1
4	Algeria	2009	Sugar, Refined Equivalent	77
4	Algeria	2009	Sunflowerseed Oil	3
4	Algeria	2009	Sweeteners, Other	1
4	Algeria	2009	Tea	1
4	Algeria	2009	Tomatoes	57
4	Algeria	2009	Vegetables, Other	218
4	Algeria	2009	Wheat	518
4	Algeria	2009	Whey	2
4	Algeria	2010	Apples	34
4	Algeria	2010	Bananas	4
4	Algeria	2010	Barley	37
4	Algeria	2010	Beans	4
4	Algeria	2010	Beer	11
4	Algeria	2010	Bovine Meat	13
4	Algeria	2010	Butter, Ghee	1
4	Algeria	2010	Cheese	2
4	Algeria	2010	Cocoa Beans	2
4	Algeria	2010	Coconuts - Incl Copra	1
4	Algeria	2010	Coffee	5
4	Algeria	2010	Dates	46
4	Algeria	2010	Demersal Fish	1
4	Algeria	2010	Eggs	17
4	Algeria	2010	Fats, Animals, Raw	1
4	Algeria	2010	Fruits, Other	68
4	Algeria	2010	Grapes	37
4	Algeria	2010	Groundnut Oil	1
4	Algeria	2010	Groundnuts (Shelled Eq)	1
4	Algeria	2010	Lemons, Limes	4
4	Algeria	2010	Maize	51
4	Algeria	2010	Marine Fish, Other	1
4	Algeria	2010	Meat, Other	1
4	Algeria	2010	Milk, Whole	267
4	Algeria	2010	Mutton & Goat Meat	16
4	Algeria	2010	Nuts	6
4	Algeria	2010	Offals, Edible	3
4	Algeria	2010	Oilcrops Oil, Other	2
4	Algeria	2010	Olive Oil	3
4	Algeria	2010	Olives	3
4	Algeria	2010	Onions	70
4	Algeria	2010	Oranges, Mandarines	53
4	Algeria	2010	Palm Oil	2
4	Algeria	2010	Peas	1

FAO country code	Country	Reference year	Food item	Gross food supply (g *capita*$^{-1}$ d^{-1})
4	Algeria	2010	Pelagic Fish	8
4	Algeria	2010	Pimento	1
4	Algeria	2010	Potatoes	180
4	Algeria	2010	Poultry Meat	21
4	Algeria	2010	Pulses, Other	14
4	Algeria	2010	Rape and Mustard Oil	1
4	Algeria	2010	Rice (Milled Equivalent)	8
4	Algeria	2010	Soyabean Oil	22
4	Algeria	2010	Spices, Other	1
4	Algeria	2010	Sugar, Refined Equivalent	77
4	Algeria	2010	Sunflowerseed Oil	6
4	Algeria	2010	Sweeteners, Other	2
4	Algeria	2010	Tea	1
4	Algeria	2010	Tomatoes	59
4	Algeria	2010	Vegetables, Other	239
4	Algeria	2010	Wheat	494
4	Algeria	2010	Whey	4
4	Algeria	2011	Apples	37
4	Algeria	2011	Bananas	16
4	Algeria	2011	Barley	37
4	Algeria	2011	Beans	6
4	Algeria	2011	Beer	11
4	Algeria	2011	Bovine Meat	13
4	Algeria	2011	Butter, Ghee	2
4	Algeria	2011	Cheese	1
4	Algeria	2011	Citrus, Other	1
4	Algeria	2011	Cocoa Beans	2
4	Algeria	2011	Coffee	9
4	Algeria	2011	Dates	50
4	Algeria	2011	Demersal Fish	1
4	Algeria	2011	Eggs	17
4	Algeria	2011	Fats, Animals, Raw	1
4	Algeria	2011	Fruits, Other	76
4	Algeria	2011	Grapes	25
4	Algeria	2011	Groundnut Oil	1
4	Algeria	2011	Groundnuts (Shelled Eq)	1
4	Algeria	2011	Lemons, Limes	5
4	Algeria	2011	Maize	40
4	Algeria	2011	Marine Fish, Other	1
4	Algeria	2011	Meat, Other	1
4	Algeria	2011	Milk, Whole	313
4	Algeria	2011	Mutton & Goat Meat	20
4	Algeria	2011	Nuts	4
4	Algeria	2011	Offals, Edible	4
4	Algeria	2011	Oilcrops Oil, Other	5
4	Algeria	2011	Olive Oil	4
4	Algeria	2011	Olives	6
4	Algeria	2011	Onions	79
4	Algeria	2011	Oranges, Mandarines	73
4	Algeria	2011	Palm Oil	2
4	Algeria	2011	Peas	1
4	Algeria	2011	Pelagic Fish	8
4	Algeria	2011	Pimento	1
4	Algeria	2011	Potatoes	177
4	Algeria	2011	Poultry Meat	20
4	Algeria	2011	Pulses, Other	15
4	Algeria	2011	Rape and Mustard Oil	1
4	Algeria	2011	Rice (Milled Equivalent)	6
4	Algeria	2011	Soyabean Oil	21
4	Algeria	2011	Spices, Other	1
4	Algeria	2011	Sugar, Refined Equivalent	67
4	Algeria	2011	Sunflowerseed Oil	6
4	Algeria	2011	Sweeteners, Other	1
4	Algeria	2011	Tea	1
4	Algeria	2011	Tomatoes	64
4	Algeria	2011	Vegetables, Other	247
4	Algeria	2011	Wheat	507
4	Algeria	2011	Whey	3
7	Angola	1992	Bananas	61
7	Angola	1992	Beans	15
7	Angola	1992	Beer	17
7	Angola	1992	Beverages, Alcoholic	2
7	Angola	1992	Beverages, Fermented	12
7	Angola	1992	Bovine Meat	21
7	Angola	1992	Cassava	444
7	Angola	1992	Citrus, Other	18
7	Angola	1992	Demersal Fish	2
7	Angola	1992	Eggs	1
7	Angola	1992	Fats, Animals, Raw	2
7	Angola	1992	Freshwater Fish	2
7	Angola	1992	Fruits, Other	7
7	Angola	1992	Groundnut Oil	1
7	Angola	1992	Groundnuts (Shelled Eq)	2
7	Angola	1992	Honey	4
7	Angola	1992	Maize	70
7	Angola	1992	Marine Fish, Other	13
7	Angola	1992	Meat, Other	2
7	Angola	1992	Milk, Whole	35
7	Angola	1992	Millet	15
7	Angola	1992	Mutton & Goat Meat	1
7	Angola	1992	Offals, Edible	3
7	Angola	1992	Oilcrops Oil, Other	1
7	Angola	1992	Olive Oil	1
7	Angola	1992	Palm Oil	9
7	Angola	1992	Palmkernel Oil	1
7	Angola	1992	Pelagic Fish	14
7	Angola	1992	Pigmeat	8

FAO country code	Country	Reference year	Food item	Gross food supply (g *capita*$^{-1}$ d^{-1})
7	Angola	1992	Pineapples	8
7	Angola	1992	Potatoes	9
7	Angola	1992	Poultry Meat	8
7	Angola	1992	Rape and Mustard Oil	1
7	Angola	1992	Rice (Milled Equivalent)	23
7	Angola	1992	Sesameseed	1
7	Angola	1992	Soyabean Oil	9
7	Angola	1992	Soyabeans	2
7	Angola	1992	Sugar, Refined Equivalent	21
7	Angola	1992	Sunflowerseed Oil	2
7	Angola	1992	Sweet Potatoes	40
7	Angola	1992	Sweeteners, Other	1
7	Angola	1992	Tomatoes	4
7	Angola	1992	Vegetables, Other	54
7	Angola	1992	Wheat	38
7	Angola	1992	Wine	15
7	Angola	1993	Bananas	58
7	Angola	1993	Beans	16
7	Angola	1993	Beer	16
7	Angola	1993	Beverages, Alcoholic	1
7	Angola	1993	Beverages, Fermented	8
7	Angola	1993	Bovine Meat	19
7	Angola	1993	Cassava	427
7	Angola	1993	Citrus, Other	17
7	Angola	1993	Demersal Fish	2
7	Angola	1993	Eggs	1
7	Angola	1993	Fats, Animals, Raw	2
7	Angola	1993	Freshwater Fish	2
7	Angola	1993	Fruits, Other	7
7	Angola	1993	Groundnut Oil	1
7	Angola	1993	Groundnuts (Shelled Eq)	2
7	Angola	1993	Honey	5
7	Angola	1993	Maize	82
7	Angola	1993	Marine Fish, Other	13
7	Angola	1993	Meat, Other	2
7	Angola	1993	Milk, Whole	34
7	Angola	1993	Millet	8
7	Angola	1993	Mutton & Goat Meat	1
7	Angola	1993	Offals, Edible	3
7	Angola	1993	Palm Oil	9
7	Angola	1993	Palmkernel Oil	1
7	Angola	1993	Pelagic Fish	15
7	Angola	1993	Pigmeat	7
7	Angola	1993	Pineapples	7
7	Angola	1993	Potatoes	4
7	Angola	1993	Poultry Meat	5
7	Angola	1993	Rape and Mustard Oil	1
7	Angola	1993	Rice (Milled Equivalent)	22
7	Angola	1993	Soyabean Oil	7
7	Angola	1993	Sugar, Refined Equivalent	18
7	Angola	1993	Sunflowerseed Oil	2
7	Angola	1993	Sweet Potatoes	40
7	Angola	1993	Sweeteners, Other	1
7	Angola	1993	Tomatoes	5
7	Angola	1993	Vegetables, Other	51
7	Angola	1993	Wheat	42
7	Angola	1993	Wine	8
7	Angola	1994	Bananas	58
7	Angola	1994	Beans	27
7	Angola	1994	Beer	18
7	Angola	1994	Beverages, Alcoholic	1
7	Angola	1994	Beverages, Fermented	10
7	Angola	1994	Bovine Meat	17
7	Angola	1994	Cassava	527
7	Angola	1994	Citrus, Other	17
7	Angola	1994	Demersal Fish	2
7	Angola	1994	Eggs	1
7	Angola	1994	Fats, Animals, Raw	2
7	Angola	1994	Freshwater Fish	2
7	Angola	1994	Fruits, Other	6
7	Angola	1994	Groundnut Oil	1
7	Angola	1994	Groundnuts (Shelled Eq)	2
7	Angola	1994	Honey	5
7	Angola	1994	Maize	76
7	Angola	1994	Marine Fish, Other	15
7	Angola	1994	Meat, Other	2
7	Angola	1994	Milk, Whole	30
7	Angola	1994	Millet	11
7	Angola	1994	Mutton & Goat Meat	1
7	Angola	1994	Offals, Edible	3
7	Angola	1994	Palm Oil	9
7	Angola	1994	Palmkernel Oil	1
7	Angola	1994	Pelagic Fish	13
7	Angola	1994	Pigmeat	7
7	Angola	1994	Pineapples	8
7	Angola	1994	Potatoes	7
7	Angola	1994	Poultry Meat	3
7	Angola	1994	Rape and Mustard Oil	1
7	Angola	1994	Rice (Milled Equivalent)	14
7	Angola	1994	Soyabean Oil	5
7	Angola	1994	Sugar, Refined Equivalent	14
7	Angola	1994	Sunflowerseed Oil	2
7	Angola	1994	Sweet Potatoes	38
7	Angola	1994	Sweeteners, Other	1
7	Angola	1994	Tomatoes	4
7	Angola	1994	Vegetables, Other	50
7	Angola	1994	Wheat	48

FAO country code	Country	Reference year	Food item	Gross food supply (g *capita*$^{-1}$ d^{-1})
7	Angola	1994	Wine	9
7	Angola	1995	Bananas	58
7	Angola	1995	Beans	25
7	Angola	1995	Beer	19
7	Angola	1995	Beverages, Alcoholic	2
7	Angola	1995	Beverages, Fermented	10
7	Angola	1995	Bovine Meat	15
7	Angola	1995	Cassava	549
7	Angola	1995	Citrus, Other	16
7	Angola	1995	Demersal Fish	2
7	Angola	1995	Eggs	1
7	Angola	1995	Fats, Animals, Raw	2
7	Angola	1995	Freshwater Fish	1
7	Angola	1995	Fruits, Other	7
7	Angola	1995	Groundnuts (Shelled Eq)	2
7	Angola	1995	Honey	5
7	Angola	1995	Maize	75
7	Angola	1995	Marine Fish, Other	10
7	Angola	1995	Meat, Other	2
7	Angola	1995	Milk, Whole	31
7	Angola	1995	Millet	11
7	Angola	1995	Mutton & Goat Meat	1
7	Angola	1995	Offals, Edible	3
7	Angola	1995	Palm Oil	9
7	Angola	1995	Palmkernel Oil	1
7	Angola	1995	Pelagic Fish	14
7	Angola	1995	Pigmeat	8
7	Angola	1995	Pineapples	7
7	Angola	1995	Potatoes	5
7	Angola	1995	Poultry Meat	4
7	Angola	1995	Rape and Mustard Oil	1
7	Angola	1995	Rice (Milled Equivalent)	10
7	Angola	1995	Soyabean Oil	6
7	Angola	1995	Sugar, Refined Equivalent	16
7	Angola	1995	Sunflowerseed Oil	2
7	Angola	1995	Sweet Potatoes	38
7	Angola	1995	Sweeteners, Other	1
7	Angola	1995	Tomatoes	5
7	Angola	1995	Vegetables, Other	49
7	Angola	1995	Wheat	58
7	Angola	1995	Wine	7
7	Angola	1996	Bananas	57
7	Angola	1996	Beans	24
7	Angola	1996	Beer	23
7	Angola	1996	Beverages, Alcoholic	2
7	Angola	1996	Beverages, Fermented	11
7	Angola	1996	Bovine Meat	18
7	Angola	1996	Cassava	523
7	Angola	1996	Citrus, Other	16
7	Angola	1996	Demersal Fish	3
7	Angola	1996	Eggs	1
7	Angola	1996	Fats, Animals, Raw	1
7	Angola	1996	Freshwater Fish	1
7	Angola	1996	Fruits, Other	7
7	Angola	1996	Groundnut Oil	1
7	Angola	1996	Groundnuts (Shelled Eq)	2
7	Angola	1996	Honey	5
7	Angola	1996	Maize	90
7	Angola	1996	Marine Fish, Other	18
7	Angola	1996	Meat, Other	2
7	Angola	1996	Milk, Whole	32
7	Angola	1996	Millet	12
7	Angola	1996	Mutton & Goat Meat	2
7	Angola	1996	Offals, Edible	3
7	Angola	1996	Oilcrops Oil, Other	1
7	Angola	1996	Palm Oil	10
7	Angola	1996	Palmkernel Oil	1
7	Angola	1996	Pelagic Fish	9
7	Angola	1996	Pigmeat	8
7	Angola	1996	Pineapples	8
7	Angola	1996	Potatoes	5
7	Angola	1996	Poultry Meat	3
7	Angola	1996	Rape and Mustard Oil	1
7	Angola	1996	Rice (Milled Equivalent)	9
7	Angola	1996	Soyabean Oil	6
7	Angola	1996	Sugar, Refined Equivalent	19
7	Angola	1996	Sunflowerseed Oil	2
7	Angola	1996	Sweet Potatoes	37
7	Angola	1996	Sweeteners, Other	1
7	Angola	1996	Tomatoes	7
7	Angola	1996	Vegetables, Other	49
7	Angola	1996	Wheat	61
7	Angola	1996	Wine	6
7	Angola	1997	Apples	1
7	Angola	1997	Bananas	55
7	Angola	1997	Beans	27
7	Angola	1997	Beer	28
7	Angola	1997	Beverages, Alcoholic	2
7	Angola	1997	Beverages, Fermented	14
7	Angola	1997	Bovine Meat	19
7	Angola	1997	Cassava	473
7	Angola	1997	Citrus, Other	15
7	Angola	1997	Demersal Fish	2
7	Angola	1997	Eggs	3
7	Angola	1997	Fats, Animals, Raw	1
7	Angola	1997	Freshwater Fish	1
7	Angola	1997	Fruits, Other	7

FAO country code	Country	Reference year	Food item	Gross food supply (g $capita^{-1}$ d^{-1})
7	Angola	1997	Groundnuts (Shelled Eq)	2
7	Angola	1997	Honey	6
7	Angola	1997	Maize	96
7	Angola	1997	Marine Fish, Other	16
7	Angola	1997	Meat, Other	2
7	Angola	1997	Milk, Whole	35
7	Angola	1997	Millet	16
7	Angola	1997	Mutton & Goat Meat	2
7	Angola	1997	Offals, Edible	3
7	Angola	1997	Oilcrops Oil, Other	1
7	Angola	1997	Palm Oil	10
7	Angola	1997	Palmkernel Oil	1
7	Angola	1997	Pelagic Fish	9
7	Angola	1997	Pigmeat	8
7	Angola	1997	Pineapples	7
7	Angola	1997	Potatoes	6
7	Angola	1997	Poultry Meat	7
7	Angola	1997	Rice (Milled Equivalent)	9
7	Angola	1997	Soyabean Oil	6
7	Angola	1997	Sugar, Refined Equivalent	22
7	Angola	1997	Sunflowerseed Oil	2
7	Angola	1997	Sweet Potatoes	36
7	Angola	1997	Sweeteners, Other	2
7	Angola	1997	Tomatoes	6
7	Angola	1997	Vegetables, Other	47
7	Angola	1997	Wheat	63
7	Angola	1997	Wine	10
7	Angola	1998	Apples	1
7	Angola	1998	Bananas	55
7	Angola	1998	Beans	19
7	Angola	1998	Beer	24
7	Angola	1998	Beverages, Alcoholic	2
7	Angola	1998	Beverages, Fermented	13
7	Angola	1998	Bovine Meat	20
7	Angola	1998	Cassava	561
7	Angola	1998	Citrus, Other	15
7	Angola	1998	Demersal Fish	5
7	Angola	1998	Eggs	3
7	Angola	1998	Fats, Animals, Raw	1
7	Angola	1998	Freshwater Fish	1
7	Angola	1998	Fruits, Other	7
7	Angola	1998	Groundnut Oil	1
7	Angola	1998	Groundnuts (Shelled Eq)	2
7	Angola	1998	Honey	5
7	Angola	1998	Maize	95
7	Angola	1998	Marine Fish, Other	9
7	Angola	1998	Meat, Other	2
7	Angola	1998	Milk, Whole	37
7	Angola	1998	Millet	15
7	Angola	1998	Mutton & Goat Meat	2
7	Angola	1998	Offals, Edible	4
7	Angola	1998	Oilcrops Oil, Other	1
7	Angola	1998	Palm Oil	10
7	Angola	1998	Palmkernel Oil	1
7	Angola	1998	Pelagic Fish	8
7	Angola	1998	Pigmeat	9
7	Angola	1998	Pineapples	7
7	Angola	1998	Potatoes	7
7	Angola	1998	Poultry Meat	9
7	Angola	1998	Rape and Mustard Oil	2
7	Angola	1998	Rice (Milled Equivalent)	9
7	Angola	1998	Soyabean Oil	5
7	Angola	1998	Sugar, Refined Equivalent	20
7	Angola	1998	Sunflowerseed Oil	2
7	Angola	1998	Sweet Potatoes	36
7	Angola	1998	Sweeteners, Other	2
7	Angola	1998	Tomatoes	6
7	Angola	1998	Vegetables, Other	47
7	Angola	1998	Wheat	62
7	Angola	1998	Wine	11
7	Angola	1999	Bananas	50
7	Angola	1999	Beans	15
7	Angola	1999	Beer	23
7	Angola	1999	Beverages, Alcoholic	2
7	Angola	1999	Beverages, Fermented	14
7	Angola	1999	Bovine Meat	19
7	Angola	1999	Cassava	564
7	Angola	1999	Citrus, Other	14
7	Angola	1999	Demersal Fish	6
7	Angola	1999	Eggs	2
7	Angola	1999	Fats, Animals, Raw	1
7	Angola	1999	Freshwater Fish	1
7	Angola	1999	Fruits, Other	6
7	Angola	1999	Groundnuts (Shelled Eq)	1
7	Angola	1999	Honey	5
7	Angola	1999	Maize	109
7	Angola	1999	Marine Fish, Other	6
7	Angola	1999	Meat, Other	2
7	Angola	1999	Milk, Whole	35
7	Angola	1999	Millet	17
7	Angola	1999	Mutton & Goat Meat	2
7	Angola	1999	Offals, Edible	4
7	Angola	1999	Oilcrops Oil, Other	1
7	Angola	1999	Onions	1
7	Angola	1999	Palm Oil	9
7	Angola	1999	Palmkernel Oil	1
7	Angola	1999	Pelagic Fish	19

FAO countrycode	Country	Reference year	Food item	Gross food supply (g *capita*$^{-1}$ d^{-1})
7	Angola	1999	Pigmeat	8
7	Angola	1999	Pineapples	6
7	Angola	1999	Potatoes	4
7	Angola	1999	Poultry Meat	7
7	Angola	1999	Rape and Mustard Oil	1
7	Angola	1999	Rice (Milled Equivalent)	10
7	Angola	1999	Soyabean Oil	6
7	Angola	1999	Sugar, Refined Equivalent	25
7	Angola	1999	Sunflowerseed Oil	1
7	Angola	1999	Sweet Potatoes	33
7	Angola	1999	Sweeteners, Other	3
7	Angola	1999	Tomatoes	6
7	Angola	1999	Vegetables, Other	42
7	Angola	1999	Wheat	63
7	Angola	1999	Wine	5
7	Angola	2000	Apples	1
7	Angola	2000	Bananas	50
7	Angola	2000	Beans	19
7	Angola	2000	Beer	24
7	Angola	2000	Beverages, Alcoholic	1
7	Angola	2000	Beverages, Fermented	14
7	Angola	2000	Bovine Meat	24
7	Angola	2000	Cassava	534
7	Angola	2000	Citrus, Other	14
7	Angola	2000	Demersal Fish	7
7	Angola	2000	Eggs	2
7	Angola	2000	Fats, Animals, Raw	1
7	Angola	2000	Freshwater Fish	1
7	Angola	2000	Fruits, Other	6
7	Angola	2000	Groundnuts (Shelled Eq)	1
7	Angola	2000	Honey	4
7	Angola	2000	Maize	100
7	Angola	2000	Marine Fish, Other	6
7	Angola	2000	Meat, Other	1
7	Angola	2000	Milk, Whole	35
7	Angola	2000	Millet	17
7	Angola	2000	Mutton & Goat Meat	2
7	Angola	2000	Offals, Edible	3
7	Angola	2000	Oilcrops Oil, Other	1
7	Angola	2000	Onions	1
7	Angola	2000	Palm Oil	9
7	Angola	2000	Palmkernel Oil	1
7	Angola	2000	Pelagic Fish	18
7	Angola	2000	Pigmeat	8
7	Angola	2000	Pineapples	7
7	Angola	2000	Potatoes	1
7	Angola	2000	Poultry Meat	11
7	Angola	2000	Rape and Mustard Oil	1
7	Angola	2000	Rice (Milled Equivalent)	13
7	Angola	2000	Soyabean Oil	8
7	Angola	2000	Sugar, Refined Equivalent	20
7	Angola	2000	Sunflowerseed Oil	1
7	Angola	2000	Sweet Potatoes	40
7	Angola	2000	Sweeteners, Other	1
7	Angola	2000	Tomatoes	9
7	Angola	2000	Vegetables, Other	45
7	Angola	2000	Wheat	71
7	Angola	2000	Wine	5
7	Angola	2001	Apples	1
7	Angola	2001	Bananas	57
7	Angola	2001	Beans	18
7	Angola	2001	Beer	41
7	Angola	2001	Beverages, Alcoholic	2
7	Angola	2001	Beverages, Fermented	14
7	Angola	2001	Bovine Meat	24
7	Angola	2001	Cassava	529
7	Angola	2001	Citrus, Other	14
7	Angola	2001	Crustaceans	1
7	Angola	2001	Demersal Fish	11
7	Angola	2001	Eggs	2
7	Angola	2001	Fats, Animals, Raw	1
7	Angola	2001	Freshwater Fish	2
7	Angola	2001	Fruits, Other	6
7	Angola	2001	Groundnut Oil	1
7	Angola	2001	Groundnuts (Shelled Eq)	2
7	Angola	2001	Honey	5
7	Angola	2001	Maize	104
7	Angola	2001	Marine Fish, Other	15
7	Angola	2001	Meat, Other	1
7	Angola	2001	Milk, Whole	36
7	Angola	2001	Millet	17
7	Angola	2001	Mutton & Goat Meat	2
7	Angola	2001	Offals, Edible	3
7	Angola	2001	Oilcrops Oil, Other	2
7	Angola	2001	Onions	2
7	Angola	2001	Palm Oil	9
7	Angola	2001	Palmkernel Oil	1
7	Angola	2001	Pelagic Fish	14
7	Angola	2001	Pigmeat	9
7	Angola	2001	Pineapples	7
7	Angola	2001	Potatoes	15
7	Angola	2001	Poultry Meat	8
7	Angola	2001	Rice (Milled Equivalent)	14
7	Angola	2001	Soyabean Oil	8
7	Angola	2001	Sugar, Refined Equivalent	20
7	Angola	2001	Sunflowerseed Oil	1
7	Angola	2001	Sweet Potatoes	61

FAO country code	Country	Reference year	Food item	Gross food supply (g *capita*$^{-1}$ d^{-1})
7	Angola	2001	Sweeteners, Other	3
7	Angola	2001	Tomatoes	9
7	Angola	2001	Vegetables, Other	44
7	Angola	2001	Wheat	66
7	Angola	2001	Wine	7
7	Angola	2002	Apples	2
7	Angola	2002	Bananas	73
7	Angola	2002	Beans	21
7	Angola	2002	Beer	51
7	Angola	2002	Beverages, Alcoholic	2
7	Angola	2002	Beverages, Fermented	14
7	Angola	2002	Bovine Meat	25
7	Angola	2002	Cassava	528
7	Angola	2002	Citrus, Other	13
7	Angola	2002	Crustaceans	1
7	Angola	2002	Demersal Fish	16
7	Angola	2002	Eggs	3
7	Angola	2002	Fats, Animals, Raw	1
7	Angola	2002	Freshwater Fish	2
7	Angola	2002	Fruits, Other	7
7	Angola	2002	Groundnut Oil	1
7	Angola	2002	Groundnuts (Shelled Eq)	2
7	Angola	2002	Honey	5
7	Angola	2002	Maize	108
7	Angola	2002	Marine Fish, Other	14
7	Angola	2002	Meat, Other	1
7	Angola	2002	Milk, Whole	36
7	Angola	2002	Millet	17
7	Angola	2002	Mutton & Goat Meat	3
7	Angola	2002	Offals, Edible	3
7	Angola	2002	Oilcrops Oil, Other	2
7	Angola	2002	Onions	2
7	Angola	2002	Palm Oil	7
7	Angola	2002	Palmkernel Oil	1
7	Angola	2002	Pelagic Fish	11
7	Angola	2002	Pigmeat	10
7	Angola	2002	Pineapples	7
7	Angola	2002	Potatoes	17
7	Angola	2002	Poultry Meat	16
7	Angola	2002	Rice (Milled Equivalent)	15
7	Angola	2002	Soyabean Oil	8
7	Angola	2002	Sugar, Refined Equivalent	25
7	Angola	2002	Sunflowerseed Oil	1
7	Angola	2002	Sweet Potatoes	70
7	Angola	2002	Sweeteners, Other	5
7	Angola	2002	Tomatoes	10
7	Angola	2002	Vegetables, Other	46
7	Angola	2002	Wheat	67
7	Angola	2002	Wine	7
7	Angola	2003	Apples	1
7	Angola	2003	Bananas	96
7	Angola	2003	Beans	21
7	Angola	2003	Beer	51
7	Angola	2003	Beverages, Alcoholic	2
7	Angola	2003	Beverages, Fermented	12
7	Angola	2003	Bovine Meat	26
7	Angola	2003	Cassava	530
7	Angola	2003	Citrus, Other	13
7	Angola	2003	Crustaceans	1
7	Angola	2003	Demersal Fish	16
7	Angola	2003	Eggs	2
7	Angola	2003	Fats, Animals, Raw	1
7	Angola	2003	Freshwater Fish	2
7	Angola	2003	Fruits, Other	6
7	Angola	2003	Groundnut Oil	1
7	Angola	2003	Groundnuts (Shelled Eq)	3
7	Angola	2003	Honey	4
7	Angola	2003	Maize	104
7	Angola	2003	Marine Fish, Other	4
7	Angola	2003	Meat, Other	1
7	Angola	2003	Milk, Whole	36
7	Angola	2003	Millet	14
7	Angola	2003	Mutton & Goat Meat	3
7	Angola	2003	Offals, Edible	3
7	Angola	2003	Oilcrops Oil, Other	3
7	Angola	2003	Onions	2
7	Angola	2003	Palm Oil	8
7	Angola	2003	Palmkernel Oil	1
7	Angola	2003	Pelagic Fish	14
7	Angola	2003	Pigmeat	10
7	Angola	2003	Pineapples	7
7	Angola	2003	Potatoes	16
7	Angola	2003	Poultry Meat	19
7	Angola	2003	Rice (Milled Equivalent)	16
7	Angola	2003	Soyabean Oil	9
7	Angola	2003	Sugar, Refined Equivalent	34
7	Angola	2003	Sunflowerseed Oil	1
7	Angola	2003	Sweet Potatoes	86
7	Angola	2003	Tomatoes	11
7	Angola	2003	Vegetables, Other	45
7	Angola	2003	Wheat	74
7	Angola	2003	Wine	9
7	Angola	2004	Apples	1
7	Angola	2004	Bananas	108
7	Angola	2004	Beans	17
7	Angola	2004	Beer	58
7	Angola	2004	Beverages, Alcoholic	3

FAO countrycode	Country	Reference year	Food item	Gross food supply (g *capita*$^{-1}$ d^{-1})
7	Angola	2004	Beverages, Fermented	16
7	Angola	2004	Bovine Meat	22
7	Angola	2004	Cassava	530
7	Angola	2004	Citrus, Other	13
7	Angola	2004	Crustaceans	1
7	Angola	2004	Demersal Fish	16
7	Angola	2004	Eggs	3
7	Angola	2004	Fats, Animals, Raw	1
7	Angola	2004	Freshwater Fish	2
7	Angola	2004	Fruits, Other	6
7	Angola	2004	Groundnut Oil	1
7	Angola	2004	Groundnuts (Shelled Eq)	3
7	Angola	2004	Honey	4
7	Angola	2004	Maize	104
7	Angola	2004	Marine Fish, Other	6
7	Angola	2004	Meat, Other	1
7	Angola	2004	Milk, Whole	36
7	Angola	2004	Millet	19
7	Angola	2004	Mutton & Goat Meat	3
7	Angola	2004	Offals, Edible	3
7	Angola	2004	Oilcrops Oil, Other	3
7	Angola	2004	Onions	2
7	Angola	2004	Palm Oil	9
7	Angola	2004	Palmkernel Oil	1
7	Angola	2004	Pelagic Fish	12
7	Angola	2004	Pigmeat	11
7	Angola	2004	Pineapples	6
7	Angola	2004	Potatoes	7
7	Angola	2004	Poultry Meat	16
7	Angola	2004	Rice (Milled Equivalent)	20
7	Angola	2004	Soyabean Oil	8
7	Angola	2004	Sugar, Refined Equivalent	33
7	Angola	2004	Sunflowerseed Oil	1
7	Angola	2004	Sweet Potatoes	96
7	Angola	2004	Tomatoes	18
7	Angola	2004	Vegetables, Other	44
7	Angola	2004	Wheat	82
7	Angola	2004	Wine	10
7	Angola	2005	Apples	1
7	Angola	2005	Bananas	125
7	Angola	2005	Beans	20
7	Angola	2005	Beer	63
7	Angola	2005	Beverages, Alcoholic	3
7	Angola	2005	Beverages, Fermented	16
7	Angola	2005	Bovine Meat	26
7	Angola	2005	Cassava	531
7	Angola	2005	Cheese	1
7	Angola	2005	Citrus, Other	13
7	Angola	2005	Coffee	1
7	Angola	2005	Demersal Fish	14
7	Angola	2005	Eggs	3
7	Angola	2005	Fats, Animals, Raw	1
7	Angola	2005	Freshwater Fish	2
7	Angola	2005	Fruits, Other	6
7	Angola	2005	Groundnut Oil	1
7	Angola	2005	Groundnuts (Shelled Eq)	4
7	Angola	2005	Honey	4
7	Angola	2005	Maize	101
7	Angola	2005	Marine Fish, Other	3
7	Angola	2005	Meat, Other	1
7	Angola	2005	Milk, Whole	36
7	Angola	2005	Millet	19
7	Angola	2005	Mutton & Goat Meat	3
7	Angola	2005	Offals, Edible	3
7	Angola	2005	Oilcrops Oil, Other	3
7	Angola	2005	Onions	2
7	Angola	2005	Palm Oil	9
7	Angola	2005	Palmkernel Oil	1
7	Angola	2005	Pelagic Fish	13
7	Angola	2005	Pigmeat	12
7	Angola	2005	Pineapples	7
7	Angola	2005	Potatoes	8
7	Angola	2005	Poultry Meat	18
7	Angola	2005	Rice (Milled Equivalent)	19
7	Angola	2005	Soyabean Oil	9
7	Angola	2005	Sugar, Refined Equivalent	30
7	Angola	2005	Sunflowerseed Oil	1
7	Angola	2005	Sweet Potatoes	98
7	Angola	2005	Tomatoes	17
7	Angola	2005	Vegetables, Other	40
7	Angola	2005	Wheat	86
7	Angola	2005	Wine	12
7	Angola	2006	Apples	1
7	Angola	2006	Bananas	129
7	Angola	2006	Beans	18
7	Angola	2006	Beer	83
7	Angola	2006	Beverages, Alcoholic	4
7	Angola	2006	Beverages, Fermented	19
7	Angola	2006	Bovine Meat	24
7	Angola	2006	Cassava	527
7	Angola	2006	Citrus, Other	13
7	Angola	2006	Coffee	1
7	Angola	2006	Demersal Fish	16
7	Angola	2006	Eggs	4
7	Angola	2006	Fats, Animals, Raw	1
7	Angola	2006	Freshwater Fish	2
7	Angola	2006	Fruits, Other	6

FAO country code	Country	Reference year	Food item	Gross food supply (g *capita*$^{-1}$ d^{-1})
7	Angola	2006	Groundnut Oil	1
7	Angola	2006	Groundnuts (Shelled Eq)	3
7	Angola	2006	Honey	4
7	Angola	2006	Maize	101
7	Angola	2006	Marine Fish, Other	4
7	Angola	2006	Meat, Other	1
7	Angola	2006	Milk, Whole	28
7	Angola	2006	Millet	19
7	Angola	2006	Mutton & Goat Meat	3
7	Angola	2006	Offals, Edible	3
7	Angola	2006	Oilcrops Oil, Other	3
7	Angola	2006	Onions	2
7	Angola	2006	Palm Oil	9
7	Angola	2006	Palmkernel Oil	1
7	Angola	2006	Pelagic Fish	16
7	Angola	2006	Pigmeat	13
7	Angola	2006	Pineapples	11
7	Angola	2006	Potatoes	28
7	Angola	2006	Poultry Meat	22
7	Angola	2006	Rice (Milled Equivalent)	19
7	Angola	2006	Soyabean Oil	9
7	Angola	2006	Sugar, Refined Equivalent	32
7	Angola	2006	Sunflowerseed Oil	1
7	Angola	2006	Sweet Potatoes	98
7	Angola	2006	Tomatoes	17
7	Angola	2006	Vegetables, Other	46
7	Angola	2006	Wheat	91
7	Angola	2006	Wine	14
7	Angola	2007	Apples	1
7	Angola	2007	Bananas	140
7	Angola	2007	Beans	19
7	Angola	2007	Beer	83
7	Angola	2007	Beverages, Alcoholic	4
7	Angola	2007	Beverages, Fermented	19
7	Angola	2007	Bovine Meat	25
7	Angola	2007	Cassava	531
7	Angola	2007	Cheese	1
7	Angola	2007	Citrus, Other	13
7	Angola	2007	Coffee	2
7	Angola	2007	Demersal Fish	20
7	Angola	2007	Eggs	3
7	Angola	2007	Fats, Animals, Raw	1
7	Angola	2007	Freshwater Fish	4
7	Angola	2007	Fruits, Other	6
7	Angola	2007	Groundnut Oil	1
7	Angola	2007	Groundnuts (Shelled Eq)	4
7	Angola	2007	Honey	4
7	Angola	2007	Maize	100
7	Angola	2007	Marine Fish, Other	4
7	Angola	2007	Meat, Other	1
7	Angola	2007	Milk, Whole	28
7	Angola	2007	Millet	8
7	Angola	2007	Mutton & Goat Meat	3
7	Angola	2007	Offals, Edible	3
7	Angola	2007	Oilcrops Oil, Other	3
7	Angola	2007	Onions	1
7	Angola	2007	Palm Oil	9
7	Angola	2007	Palmkernel Oil	1
7	Angola	2007	Pelagic Fish	18
7	Angola	2007	Pigmeat	16
7	Angola	2007	Pineapples	11
7	Angola	2007	Potatoes	47
7	Angola	2007	Poultry Meat	23
7	Angola	2007	Rice (Milled Equivalent)	19
7	Angola	2007	Soyabean Oil	9
7	Angola	2007	Sugar, Refined Equivalent	33
7	Angola	2007	Sunflowerseed Oil	1
7	Angola	2007	Sweet Potatoes	131
7	Angola	2007	Tomatoes	11
7	Angola	2007	Vegetables, Other	49
7	Angola	2007	Wheat	94
7	Angola	2007	Wine	14
7	Angola	2008	Apples	1
7	Angola	2008	Bananas	141
7	Angola	2008	Beans	22
7	Angola	2008	Beer	104
7	Angola	2008	Beverages, Alcoholic	5
7	Angola	2008	Beverages, Fermented	27
7	Angola	2008	Bovine Meat	23
7	Angola	2008	Cassava	525
7	Angola	2008	Cheese	1
7	Angola	2008	Citrus, Other	14
7	Angola	2008	Coffee	1
7	Angola	2008	Demersal Fish	20
7	Angola	2008	Eggs	4
7	Angola	2008	Fats, Animals, Raw	1
7	Angola	2008	Freshwater Fish	4
7	Angola	2008	Fruits, Other	7
7	Angola	2008	Groundnut Oil	2
7	Angola	2008	Groundnuts (Shelled Eq)	5
7	Angola	2008	Honey	4
7	Angola	2008	Maize	106
7	Angola	2008	Marine Fish, Other	4
7	Angola	2008	Meat, Other	1
7	Angola	2008	Milk, Whole	27
7	Angola	2008	Millet	7
7	Angola	2008	Mutton & Goat Meat	4

FAO country code	Country	Reference year	Food item	Gross food supply (g $capita^{-1}$ d^{-1})
7	Angola	2008	Offals, Edible	3
7	Angola	2008	Oilcrops Oil, Other	2
7	Angola	2008	Palm Oil	9
7	Angola	2008	Palmkernel Oil	1
7	Angola	2008	Pelagic Fish	19
7	Angola	2008	Pigmeat	18
7	Angola	2008	Pineapples	13
7	Angola	2008	Potatoes	41
7	Angola	2008	Poultry Meat	27
7	Angola	2008	Rice (Milled Equivalent)	20
7	Angola	2008	Soyabean Oil	9
7	Angola	2008	Sugar, Refined Equivalent	37
7	Angola	2008	Sunflowerseed Oil	1
7	Angola	2008	Sweet Potatoes	109
7	Angola	2008	Tomatoes	12
7	Angola	2008	Vegetables, Other	121
7	Angola	2008	Wheat	96
7	Angola	2008	Wine	17
7	Angola	2009	Apples	1
7	Angola	2009	Bananas	142
7	Angola	2009	Beans	24
7	Angola	2009	Beer	119
7	Angola	2009	Beverages, Alcoholic	4
7	Angola	2009	Beverages, Fermented	32
7	Angola	2009	Bovine Meat	20
7	Angola	2009	Cassava	525
7	Angola	2009	Cheese	1
7	Angola	2009	Citrus, Other	30
7	Angola	2009	Cocoa Beans	1
7	Angola	2009	Coconuts - Incl Copra	1
7	Angola	2009	Coffee	2
7	Angola	2009	Demersal Fish	19
7	Angola	2009	Eggs	3
7	Angola	2009	Fats, Animals, Raw	1
7	Angola	2009	Freshwater Fish	2
7	Angola	2009	Fruits, Other	8
7	Angola	2009	Groundnut Oil	2
7	Angola	2009	Groundnuts (Shelled Eq)	6
7	Angola	2009	Honey	4
7	Angola	2009	Maize	106
7	Angola	2009	Marine Fish, Other	3
7	Angola	2009	Meat, Other	1
7	Angola	2009	Milk, Whole	22
7	Angola	2009	Millet	5
7	Angola	2009	Mutton & Goat Meat	3
7	Angola	2009	Offals, Edible	3
7	Angola	2009	Oilcrops Oil, Other	3
7	Angola	2009	Palm Oil	9
7	Angola	2009	Palmkernel Oil	1
7	Angola	2009	Pelagic Fish	20
7	Angola	2009	Pigmeat	19
7	Angola	2009	Pineapples	38
7	Angola	2009	Potatoes	79
7	Angola	2009	Poultry Meat	26
7	Angola	2009	Rice (Milled Equivalent)	20
7	Angola	2009	Soyabean Oil	9
7	Angola	2009	Sugar, Refined Equivalent	34
7	Angola	2009	Sunflowerseed Oil	1
7	Angola	2009	Sweet Potatoes	127
7	Angola	2009	Tomatoes	11
7	Angola	2009	Vegetables, Other	171
7	Angola	2009	Wheat	95
7	Angola	2009	Wine	12
7	Angola	2010	Apples	1
7	Angola	2010	Bananas	146
7	Angola	2010	Beans	24
7	Angola	2010	Beer	126
7	Angola	2010	Beverages, Alcoholic	7
7	Angola	2010	Beverages, Fermented	43
7	Angola	2010	Bovine Meat	18
7	Angola	2010	Cassava	530
7	Angola	2010	Cheese	1
7	Angola	2010	Citrus, Other	31
7	Angola	2010	Coffee	7
7	Angola	2010	Demersal Fish	18
7	Angola	2010	Eggs	5
7	Angola	2010	Fats, Animals, Raw	1
7	Angola	2010	Freshwater Fish	3
7	Angola	2010	Fruits, Other	5
7	Angola	2010	Groundnut Oil	2
7	Angola	2010	Groundnuts (Shelled Eq)	6
7	Angola	2010	Honey	3
7	Angola	2010	Maize	110
7	Angola	2010	Marine Fish, Other	3
7	Angola	2010	Meat, Other	1
7	Angola	2010	Milk, Whole	24
7	Angola	2010	Millet	6
7	Angola	2010	Mutton & Goat Meat	3
7	Angola	2010	Offals, Edible	3
7	Angola	2010	Oilcrops Oil, Other	3
7	Angola	2010	Palm Oil	8
7	Angola	2010	Palmkernel Oil	1
7	Angola	2010	Pelagic Fish	17
7	Angola	2010	Pigmeat	18
7	Angola	2010	Pineapples	40
7	Angola	2010	Potatoes	78
7	Angola	2010	Poultry Meat	36

FAO country code	Country	Reference year	Food item	Gross food supply (g $capita^{-1}$ d^{-1})
7	Angola	2010	Rice (Milled Equivalent)	19
7	Angola	2010	Soyabean Oil	9
7	Angola	2010	Sugar, Refined Equivalent	33
7	Angola	2010	Sunflowerseed Oil	1
7	Angola	2010	Sweet Potatoes	123
7	Angola	2010	Tomatoes	10
7	Angola	2010	Vegetables, Other	174
7	Angola	2010	Wheat	97
7	Angola	2010	Wine	13
7	Angola	2011	Apples	2
7	Angola	2011	Bananas	134
7	Angola	2011	Beans	25
7	Angola	2011	Beer	137
7	Angola	2011	Beverages, Alcoholic	6
7	Angola	2011	Beverages, Fermented	46
7	Angola	2011	Bovine Meat	19
7	Angola	2011	Cassava	530
7	Angola	2011	Cheese	1
7	Angola	2011	Citrus, Other	33
7	Angola	2011	Coffee	7
7	Angola	2011	Demersal Fish	17
7	Angola	2011	Eggs	5
7	Angola	2011	Fats, Animals, Raw	2
7	Angola	2011	Freshwater Fish	3
7	Angola	2011	Fruits, Other	7
7	Angola	2011	Groundnut Oil	2
7	Angola	2011	Groundnuts (Shelled Eq)	6
7	Angola	2011	Honey	3
7	Angola	2011	Maize	108
7	Angola	2011	Marine Fish, Other	3
7	Angola	2011	Meat, Other	1
7	Angola	2011	Milk, Whole	25
7	Angola	2011	Millet	6
7	Angola	2011	Mutton & Goat Meat	3
7	Angola	2011	Offals, Edible	3
7	Angola	2011	Oilcrops Oil, Other	2
7	Angola	2011	Onions	1
7	Angola	2011	Palm Oil	8
7	Angola	2011	Palmkernel Oil	1
7	Angola	2011	Pelagic Fish	16
7	Angola	2011	Pigmeat	22
7	Angola	2011	Pineapples	40
7	Angola	2011	Potatoes	76
7	Angola	2011	Poultry Meat	42
7	Angola	2011	Rice (Milled Equivalent)	20
7	Angola	2011	Soyabean Oil	10
7	Angola	2011	Sugar, Refined Equivalent	34
7	Angola	2011	Sunflowerseed Oil	1
7	Angola	2011	Sweet Potatoes	126
7	Angola	2011	Tomatoes	12
7	Angola	2011	Vegetables, Other	184
7	Angola	2011	Wheat	105
7	Angola	2011	Wine	13
9	Argentina	1992	Apples	40
9	Argentina	1992	Bananas	25
9	Argentina	1992	Beer	83
9	Argentina	1992	Beverages, Alcoholic	6
9	Argentina	1992	Bovine Meat	178
9	Argentina	1992	Butter, Ghee	4
9	Argentina	1992	Cassava	6
9	Argentina	1992	Cephalopods	2
9	Argentina	1992	Cereals, Other	2
9	Argentina	1992	Cheese	28
9	Argentina	1992	Cocoa Beans	3
9	Argentina	1992	Coconuts - Incl Copra	1
9	Argentina	1992	Coffee	3
9	Argentina	1992	Cottonseed Oil	1
9	Argentina	1992	Cream	1
9	Argentina	1992	Demersal Fish	18
9	Argentina	1992	Eggs	19
9	Argentina	1992	Fats, Animals, Raw	7
9	Argentina	1992	Freshwater Fish	1
9	Argentina	1992	Fruits, Other	34
9	Argentina	1992	Grapefruit	7
9	Argentina	1992	Grapes	3
9	Argentina	1992	Lemons, Limes	41
9	Argentina	1992	Maize	14
9	Argentina	1992	Maize Germ Oil	2
9	Argentina	1992	Meat, Other	4
9	Argentina	1992	Milk, Whole	241
9	Argentina	1992	Mutton & Goat Meat	6
9	Argentina	1992	Nuts	1
9	Argentina	1992	Offals, Edible	17
9	Argentina	1992	Onions	37
9	Argentina	1992	Oranges, Mandarines	90
9	Argentina	1992	Peas	1
9	Argentina	1992	Pelagic Fish	3
9	Argentina	1992	Pigmeat	15
9	Argentina	1992	Pineapples	2
9	Argentina	1992	Potatoes	112
9	Argentina	1992	Poultry Meat	44
9	Argentina	1992	Pulses, Other	2
9	Argentina	1992	Rice (Milled Equivalent)	19
9	Argentina	1992	Soyabean Oil	6
9	Argentina	1992	Sugar, Refined Equivalent	88
9	Argentina	1992	Sunflowerseed Oil	29
9	Argentina	1992	Sweet Potatoes	20

FAO country code	Country	Reference year	Food item	Gross food supply (g $capita^{-1}$ d^{-1})
9	Argentina	1992	Sweeteners, Other	19
9	Argentina	1992	Tea	14
9	Argentina	1992	Tomatoes	59
9	Argentina	1992	Vegetables, Other	121
9	Argentina	1992	Wheat	297
9	Argentina	1992	Whey	1
9	Argentina	1992	Wine	137
9	Argentina	1993	Apples	37
9	Argentina	1993	Bananas	30
9	Argentina	1993	Beer	87
9	Argentina	1993	Beverages, Alcoholic	7
9	Argentina	1993	Bovine Meat	177
9	Argentina	1993	Butter, Ghee	3
9	Argentina	1993	Cassava	6
9	Argentina	1993	Cephalopods	1
9	Argentina	1993	Cereals, Other	1
9	Argentina	1993	Cheese	28
9	Argentina	1993	Cocoa Beans	3
9	Argentina	1993	Coconuts - Incl Copra	1
9	Argentina	1993	Coffee	3
9	Argentina	1993	Cream	1
9	Argentina	1993	Demersal Fish	21
9	Argentina	1993	Eggs	19
9	Argentina	1993	Fats, Animals, Raw	7
9	Argentina	1993	Freshwater Fish	1
9	Argentina	1993	Fruits, Other	44
9	Argentina	1993	Grapefruit	9
9	Argentina	1993	Grapes	4
9	Argentina	1993	Lemons, Limes	41
9	Argentina	1993	Maize	21
9	Argentina	1993	Maize Germ Oil	2
9	Argentina	1993	Meat, Other	4
9	Argentina	1993	Milk, Whole	251
9	Argentina	1993	Mutton & Goat Meat	6
9	Argentina	1993	Nuts	1
9	Argentina	1993	Oats	2
9	Argentina	1993	Offals, Edible	18
9	Argentina	1993	Olives	1
9	Argentina	1993	Onions	27
9	Argentina	1993	Oranges, Mandarines	81
9	Argentina	1993	Peas	1
9	Argentina	1993	Pelagic Fish	2
9	Argentina	1993	Pigmeat	21
9	Argentina	1993	Pineapples	3
9	Argentina	1993	Potatoes	145
9	Argentina	1993	Poultry Meat	62
9	Argentina	1993	Pulses, Other	2
9	Argentina	1993	Rice (Milled Equivalent)	8
9	Argentina	1993	Soyabean Oil	6
9	Argentina	1993	Sugar, Refined Equivalent	89
9	Argentina	1993	Sunflowerseed Oil	30
9	Argentina	1993	Sweet Potatoes	20
9	Argentina	1993	Sweeteners, Other	20
9	Argentina	1993	Tea	17
9	Argentina	1993	Tomatoes	65
9	Argentina	1993	Vegetables, Other	114
9	Argentina	1993	Wheat	289
9	Argentina	1993	Whey	1
9	Argentina	1993	Wine	127
9	Argentina	1994	Apples	40
9	Argentina	1994	Bananas	29
9	Argentina	1994	Beer	94
9	Argentina	1994	Beverages, Alcoholic	7
9	Argentina	1994	Bovine Meat	169
9	Argentina	1994	Butter, Ghee	4
9	Argentina	1994	Cassava	6
9	Argentina	1994	Cephalopods	1
9	Argentina	1994	Cereals, Other	1
9	Argentina	1994	Cheese	30
9	Argentina	1994	Cocoa Beans	3
9	Argentina	1994	Coconuts - Incl Copra	1
9	Argentina	1994	Coffee	3
9	Argentina	1994	Crustaceans	1
9	Argentina	1994	Demersal Fish	16
9	Argentina	1994	Eggs	19
9	Argentina	1994	Fats, Animals, Raw	8
9	Argentina	1994	Freshwater Fish	1
9	Argentina	1994	Fruits, Other	45
9	Argentina	1994	Grapefruit	15
9	Argentina	1994	Grapes	3
9	Argentina	1994	Lemons, Limes	41
9	Argentina	1994	Maize	10
9	Argentina	1994	Maize Germ Oil	2
9	Argentina	1994	Meat, Other	4
9	Argentina	1994	Milk, Whole	262
9	Argentina	1994	Mutton & Goat Meat	7
9	Argentina	1994	Nuts	1
9	Argentina	1994	Oats	1
9	Argentina	1994	Offals, Edible	18
9	Argentina	1994	Onions	24
9	Argentina	1994	Oranges, Mandarines	99
9	Argentina	1994	Peas	1
9	Argentina	1994	Pelagic Fish	3
9	Argentina	1994	Pigmeat	22
9	Argentina	1994	Pineapples	2
9	Argentina	1994	Potatoes	154
9	Argentina	1994	Poultry Meat	64

FAO country code	Country	Reference year	Food item	Gross food supply (g *capita*$^{-1}$ d^{-1})
9	Argentina	1994	Pulses, Other	3
9	Argentina	1994	Rape and Mustard Oil	1
9	Argentina	1994	Rice (Milled Equivalent)	12
9	Argentina	1994	Soyabean Oil	5
9	Argentina	1994	Sugar, Refined Equivalent	95
9	Argentina	1994	Sunflowerseed Oil	31
9	Argentina	1994	Sweet Potatoes	27
9	Argentina	1994	Sweeteners, Other	22
9	Argentina	1994	Tea	18
9	Argentina	1994	Tomatoes	62
9	Argentina	1994	Vegetables, Other	121
9	Argentina	1994	Wheat	291
9	Argentina	1994	Whey	1
9	Argentina	1994	Wine	115
9	Argentina	1995	Apples	40
9	Argentina	1995	Bananas	27
9	Argentina	1995	Beans	1
9	Argentina	1995	Beer	89
9	Argentina	1995	Beverages, Alcoholic	5
9	Argentina	1995	Bovine Meat	153
9	Argentina	1995	Butter, Ghee	4
9	Argentina	1995	Cassava	6
9	Argentina	1995	Cephalopods	1
9	Argentina	1995	Cereals, Other	1
9	Argentina	1995	Cheese	28
9	Argentina	1995	Cocoa Beans	2
9	Argentina	1995	Coconuts - Incl Copra	1
9	Argentina	1995	Coffee	3
9	Argentina	1995	Cream	1
9	Argentina	1995	Demersal Fish	19
9	Argentina	1995	Eggs	19
9	Argentina	1995	Fats, Animals, Raw	8
9	Argentina	1995	Freshwater Fish	1
9	Argentina	1995	Fruits, Other	41
9	Argentina	1995	Grapefruit	12
9	Argentina	1995	Grapes	2
9	Argentina	1995	Honey	1
9	Argentina	1995	Lemons, Limes	43
9	Argentina	1995	Maize	17
9	Argentina	1995	Maize Germ Oil	2
9	Argentina	1995	Marine Fish, Other	1
9	Argentina	1995	Meat, Other	4
9	Argentina	1995	Milk, Whole	290
9	Argentina	1995	Molluscs, Other	1
9	Argentina	1995	Mutton & Goat Meat	7
9	Argentina	1995	Nuts	1
9	Argentina	1995	Oats	1
9	Argentina	1995	Offals, Edible	18
9	Argentina	1995	Olives	2
9	Argentina	1995	Onions	22
9	Argentina	1995	Oranges, Mandarines	92
9	Argentina	1995	Peas	1
9	Argentina	1995	Pelagic Fish	4
9	Argentina	1995	Pigmeat	19
9	Argentina	1995	Pineapples	2
9	Argentina	1995	Potatoes	162
9	Argentina	1995	Poultry Meat	65
9	Argentina	1995	Pulses, Other	2
9	Argentina	1995	Rice (Milled Equivalent)	13
9	Argentina	1995	Soyabean Oil	5
9	Argentina	1995	Sugar, Refined Equivalent	98
9	Argentina	1995	Sunflowerseed Oil	31
9	Argentina	1995	Sweet Potatoes	24
9	Argentina	1995	Sweeteners, Other	22
9	Argentina	1995	Tea	18
9	Argentina	1995	Tomatoes	71
9	Argentina	1995	Vegetables, Other	121
9	Argentina	1995	Wheat	292
9	Argentina	1995	Whey	1
9	Argentina	1995	Wine	109
9	Argentina	1996	Apples	52
9	Argentina	1996	Bananas	26
9	Argentina	1996	Beer	93
9	Argentina	1996	Beverages, Alcoholic	6
9	Argentina	1996	Bovine Meat	149
9	Argentina	1996	Butter, Ghee	4
9	Argentina	1996	Cassava	5
9	Argentina	1996	Cephalopods	4
9	Argentina	1996	Cheese	29
9	Argentina	1996	Cocoa Beans	2
9	Argentina	1996	Coconut Oil	1
9	Argentina	1996	Coconuts - Incl Copra	1
9	Argentina	1996	Coffee	3
9	Argentina	1996	Cottonseed Oil	1
9	Argentina	1996	Demersal Fish	22
9	Argentina	1996	Eggs	19
9	Argentina	1996	Fats, Animals, Raw	8
9	Argentina	1996	Freshwater Fish	1
9	Argentina	1996	Fruits, Other	47
9	Argentina	1996	Grapefruit	11
9	Argentina	1996	Grapes	2
9	Argentina	1996	Lemons, Limes	43
9	Argentina	1996	Maize	17
9	Argentina	1996	Maize Germ Oil	2
9	Argentina	1996	Meat, Other	5
9	Argentina	1996	Milk, Whole	317
9	Argentina	1996	Molluscs, Other	1

FAO country code	Country	Reference year	Food item	Gross food supply (g $capita^{-1}$ d^{-1})
9	Argentina	1996	Mutton & Goat Meat	6
9	Argentina	1996	Nuts	1
9	Argentina	1996	Oats	1
9	Argentina	1996	Offals, Edible	17
9	Argentina	1996	Olives	1
9	Argentina	1996	Onions	26
9	Argentina	1996	Oranges, Mandarines	64
9	Argentina	1996	Peas	1
9	Argentina	1996	Pelagic Fish	3
9	Argentina	1996	Pigmeat	18
9	Argentina	1996	Pineapples	3
9	Argentina	1996	Potatoes	147
9	Argentina	1996	Poultry Meat	63
9	Argentina	1996	Pulses, Other	2
9	Argentina	1996	Rice (Milled Equivalent)	17
9	Argentina	1996	Soyabean Oil	6
9	Argentina	1996	Sugar, Refined Equivalent	96
9	Argentina	1996	Sunflowerseed Oil	29
9	Argentina	1996	Sweet Potatoes	24
9	Argentina	1996	Sweeteners, Other	19
9	Argentina	1996	Tea	19
9	Argentina	1996	Tomatoes	53
9	Argentina	1996	Vegetables, Other	121
9	Argentina	1996	Wheat	298
9	Argentina	1996	Whey	1
9	Argentina	1996	Wine	104
9	Argentina	1997	Apples	36
9	Argentina	1997	Bananas	29
9	Argentina	1997	Beans	1
9	Argentina	1997	Beer	101
9	Argentina	1997	Beverages, Alcoholic	6
9	Argentina	1997	Bovine Meat	154
9	Argentina	1997	Butter, Ghee	4
9	Argentina	1997	Cassava	5
9	Argentina	1997	Cephalopods	8
9	Argentina	1997	Cheese	33
9	Argentina	1997	Cocoa Beans	2
9	Argentina	1997	Coconuts - Incl Copra	1
9	Argentina	1997	Coffee	3
9	Argentina	1997	Demersal Fish	16
9	Argentina	1997	Eggs	19
9	Argentina	1997	Fats, Animals, Raw	8
9	Argentina	1997	Freshwater Fish	2
9	Argentina	1997	Fruits, Other	43
9	Argentina	1997	Grapefruit	16
9	Argentina	1997	Grapes	3
9	Argentina	1997	Lemons, Limes	52
9	Argentina	1997	Maize	17
9	Argentina	1997	Maize Germ Oil	2
9	Argentina	1997	Meat, Other	4
9	Argentina	1997	Milk, Whole	271
9	Argentina	1997	Molluscs, Other	2
9	Argentina	1997	Mutton & Goat Meat	5
9	Argentina	1997	Nuts	1
9	Argentina	1997	Oats	2
9	Argentina	1997	Offals, Edible	17
9	Argentina	1997	Olives	1
9	Argentina	1997	Onions	15
9	Argentina	1997	Oranges, Mandarines	108
9	Argentina	1997	Peas	1
9	Argentina	1997	Pelagic Fish	4
9	Argentina	1997	Pigmeat	17
9	Argentina	1997	Pineapples	3
9	Argentina	1997	Potatoes	194
9	Argentina	1997	Poultry Meat	65
9	Argentina	1997	Pulses, Other	2
9	Argentina	1997	Rice (Milled Equivalent)	16
9	Argentina	1997	Soyabean Oil	5
9	Argentina	1997	Sugar, Refined Equivalent	95
9	Argentina	1997	Sunflowerseed Oil	29
9	Argentina	1997	Sweet Potatoes	17
9	Argentina	1997	Sweeteners, Other	18
9	Argentina	1997	Tea	19
9	Argentina	1997	Tomatoes	56
9	Argentina	1997	Vegetables, Other	111
9	Argentina	1997	Wheat	288
9	Argentina	1997	Wine	104
9	Argentina	1998	Apples	37
9	Argentina	1998	Bananas	29
9	Argentina	1998	Beans	1
9	Argentina	1998	Beer	96
9	Argentina	1998	Beverages, Alcoholic	4
9	Argentina	1998	Bovine Meat	154
9	Argentina	1998	Butter, Ghee	4
9	Argentina	1998	Cassava	5
9	Argentina	1998	Cephalopods	4
9	Argentina	1998	Cheese	31
9	Argentina	1998	Cocoa Beans	2
9	Argentina	1998	Coconuts - Incl Copra	1
9	Argentina	1998	Coffee	3
9	Argentina	1998	Cottonseed Oil	1
9	Argentina	1998	Demersal Fish	20
9	Argentina	1998	Eggs	20
9	Argentina	1998	Fats, Animals, Raw	9
9	Argentina	1998	Freshwater Fish	2
9	Argentina	1998	Fruits, Other	43
9	Argentina	1998	Grapefruit	15

FAO country code	Country	Reference year	Food item	Gross food supply (g $capita^{-1}$ d^{-1})
9	Argentina	1998	Grapes	3
9	Argentina	1998	Honey	1
9	Argentina	1998	Lemons, Limes	56
9	Argentina	1998	Maize	19
9	Argentina	1998	Maize Germ Oil	2
9	Argentina	1998	Meat, Other	4
9	Argentina	1998	Milk, Whole	295
9	Argentina	1998	Molluscs, Other	3
9	Argentina	1998	Mutton & Goat Meat	4
9	Argentina	1998	Nuts	1
9	Argentina	1998	Oats	3
9	Argentina	1998	Offals, Edible	17
9	Argentina	1998	Olive Oil	1
9	Argentina	1998	Olives	2
9	Argentina	1998	Onions	28
9	Argentina	1998	Oranges, Mandarines	89
9	Argentina	1998	Peas	1
9	Argentina	1998	Pelagic Fish	2
9	Argentina	1998	Pigmeat	20
9	Argentina	1998	Pineapples	2
9	Argentina	1998	Potatoes	209
9	Argentina	1998	Poultry Meat	74
9	Argentina	1998	Pulses, Other	2
9	Argentina	1998	Rice (Milled Equivalent)	12
9	Argentina	1998	Soyabean Oil	6
9	Argentina	1998	Spices, Other	1
9	Argentina	1998	Sugar, Refined Equivalent	94
9	Argentina	1998	Sunflowerseed Oil	30
9	Argentina	1998	Sweet Potatoes	22
9	Argentina	1998	Sweeteners, Other	17
9	Argentina	1998	Tea	19
9	Argentina	1998	Tomatoes	51
9	Argentina	1998	Vegetables, Other	120
9	Argentina	1998	Wheat	288
9	Argentina	1998	Whey	1
9	Argentina	1998	Wine	96
9	Argentina	1999	Apples	36
9	Argentina	1999	Bananas	33
9	Argentina	1999	Beer	91
9	Argentina	1999	Beverages, Alcoholic	6
9	Argentina	1999	Bovine Meat	165
9	Argentina	1999	Butter, Ghee	3
9	Argentina	1999	Cassava	5
9	Argentina	1999	Cephalopods	5
9	Argentina	1999	Cereals, Other	1
9	Argentina	1999	Cheese	32
9	Argentina	1999	Cocoa Beans	2
9	Argentina	1999	Coconuts - Incl Copra	1
9	Argentina	1999	Coffee	3
9	Argentina	1999	Cottonseed Oil	2
9	Argentina	1999	Crustaceans	1
9	Argentina	1999	Demersal Fish	12
9	Argentina	1999	Eggs	21
9	Argentina	1999	Fats, Animals, Raw	8
9	Argentina	1999	Freshwater Fish	2
9	Argentina	1999	Fruits, Other	41
9	Argentina	1999	Grapefruit	13
9	Argentina	1999	Grapes	3
9	Argentina	1999	Honey	1
9	Argentina	1999	Lemons, Limes	54
9	Argentina	1999	Maize	24
9	Argentina	1999	Maize Germ Oil	2
9	Argentina	1999	Meat, Other	5
9	Argentina	1999	Milk, Whole	296
9	Argentina	1999	Molluscs, Other	3
9	Argentina	1999	Mutton & Goat Meat	4
9	Argentina	1999	Nuts	1
9	Argentina	1999	Oats	4
9	Argentina	1999	Offals, Edible	17
9	Argentina	1999	Olive Oil	1
9	Argentina	1999	Olives	2
9	Argentina	1999	Onions	42
9	Argentina	1999	Oranges, Mandarines	85
9	Argentina	1999	Peas	1
9	Argentina	1999	Pelagic Fish	4
9	Argentina	1999	Pigmeat	22
9	Argentina	1999	Pineapples	3
9	Argentina	1999	Potatoes	167
9	Argentina	1999	Poultry Meat	77
9	Argentina	1999	Pulses, Other	2
9	Argentina	1999	Rice (Milled Equivalent)	16
9	Argentina	1999	Soyabean Oil	6
9	Argentina	1999	Sugar, Refined Equivalent	100
9	Argentina	1999	Sunflowerseed Oil	30
9	Argentina	1999	Sweet Potatoes	17
9	Argentina	1999	Sweeteners, Other	21
9	Argentina	1999	Tea	21
9	Argentina	1999	Tomatoes	59
9	Argentina	1999	Vegetables, Other	115
9	Argentina	1999	Wheat	286
9	Argentina	1999	Wine	95
9	Argentina	2000	Apples	39
9	Argentina	2000	Bananas	36
9	Argentina	2000	Beer	91
9	Argentina	2000	Beverages, Alcoholic	5
9	Argentina	2000	Bovine Meat	162
9	Argentina	2000	Butter, Ghee	3

FAO country code	Country	Reference year	Food item	Gross food supply (g *capita*$^{-1}$ d^{-1})
9	Argentina	2000	Cassava	5
9	Argentina	2000	Cephalopods	1
9	Argentina	2000	Cereals, Other	1
9	Argentina	2000	Cheese	33
9	Argentina	2000	Cocoa Beans	2
9	Argentina	2000	Coconuts - Incl Copra	1
9	Argentina	2000	Coffee	3
9	Argentina	2000	Cottonseed Oil	1
9	Argentina	2000	Crustaceans	1
9	Argentina	2000	Demersal Fish	11
9	Argentina	2000	Eggs	21
9	Argentina	2000	Fats, Animals, Raw	7
9	Argentina	2000	Freshwater Fish	3
9	Argentina	2000	Fruits, Other	38
9	Argentina	2000	Grapefruit	15
9	Argentina	2000	Grapes	3
9	Argentina	2000	Lemons, Limes	61
9	Argentina	2000	Maize	30
9	Argentina	2000	Maize Germ Oil	1
9	Argentina	2000	Marine Fish, Other	1
9	Argentina	2000	Meat, Other	5
9	Argentina	2000	Milk, Whole	295
9	Argentina	2000	Molluscs, Other	3
9	Argentina	2000	Mutton & Goat Meat	4
9	Argentina	2000	Nuts	1
9	Argentina	2000	Oats	4
9	Argentina	2000	Offals, Edible	17
9	Argentina	2000	Olive Oil	1
9	Argentina	2000	Olives	3
9	Argentina	2000	Onions	33
9	Argentina	2000	Oranges, Mandarines	107
9	Argentina	2000	Peas	1
9	Argentina	2000	Pelagic Fish	4
9	Argentina	2000	Pigmeat	22
9	Argentina	2000	Pineapples	3
9	Argentina	2000	Potatoes	132
9	Argentina	2000	Poultry Meat	76
9	Argentina	2000	Pulses, Other	2
9	Argentina	2000	Rice (Milled Equivalent)	14
9	Argentina	2000	Soyabean Oil	6
9	Argentina	2000	Sugar, Refined Equivalent	101
9	Argentina	2000	Sunflowerseed Oil	29
9	Argentina	2000	Sweet Potatoes	18
9	Argentina	2000	Sweeteners, Other	19
9	Argentina	2000	Tea	20
9	Argentina	2000	Tomatoes	55
9	Argentina	2000	Vegetables, Other	109
9	Argentina	2000	Wheat	304
9	Argentina	2000	Whey	1
9	Argentina	2000	Wine	93
9	Argentina	2001	Apples	57
9	Argentina	2001	Bananas	36
9	Argentina	2001	Beer	91
9	Argentina	2001	Beverages, Alcoholic	4
9	Argentina	2001	Bovine Meat	156
9	Argentina	2001	Butter, Ghee	4
9	Argentina	2001	Cassava	5
9	Argentina	2001	Cephalopods	6
9	Argentina	2001	Cereals, Other	1
9	Argentina	2001	Cheese	30
9	Argentina	2001	Cocoa Beans	2
9	Argentina	2001	Coconuts - Incl Copra	1
9	Argentina	2001	Coffee	3
9	Argentina	2001	Cottonseed Oil	1
9	Argentina	2001	Crustaceans	1
9	Argentina	2001	Demersal Fish	12
9	Argentina	2001	Eggs	19
9	Argentina	2001	Fats, Animals, Raw	7
9	Argentina	2001	Freshwater Fish	1
9	Argentina	2001	Fruits, Other	42
9	Argentina	2001	Grapefruit	11
9	Argentina	2001	Grapes	3
9	Argentina	2001	Honey	1
9	Argentina	2001	Lemons, Limes	60
9	Argentina	2001	Maize	29
9	Argentina	2001	Maize Germ Oil	2
9	Argentina	2001	Marine Fish, Other	1
9	Argentina	2001	Meat, Other	4
9	Argentina	2001	Milk, Whole	267
9	Argentina	2001	Molluscs, Other	4
9	Argentina	2001	Mutton & Goat Meat	4
9	Argentina	2001	Nuts	1
9	Argentina	2001	Oats	5
9	Argentina	2001	Offals, Edible	17
9	Argentina	2001	Olive Oil	1
9	Argentina	2001	Olives	3
9	Argentina	2001	Onions	32
9	Argentina	2001	Oranges, Mandarines	109
9	Argentina	2001	Peas	1
9	Argentina	2001	Pelagic Fish	4
9	Argentina	2001	Pigmeat	20
9	Argentina	2001	Pineapples	3
9	Argentina	2001	Potatoes	147
9	Argentina	2001	Poultry Meat	73
9	Argentina	2001	Pulses, Other	2
9	Argentina	2001	Rice (Milled Equivalent)	17
9	Argentina	2001	Soyabean Oil	7

FAO country code	Country	Reference year	Food item	Gross food supply (g *capita*$^{-1}$ d^{-1})
9	Argentina	2001	Sugar, Refined Equivalent	103
9	Argentina	2001	Sunflowerseed Oil	30
9	Argentina	2001	Sweet Potatoes	18
9	Argentina	2001	Sweeteners, Other	19
9	Argentina	2001	Tea	21
9	Argentina	2001	Tomatoes	56
9	Argentina	2001	Vegetables, Other	109
9	Argentina	2001	Wheat	295
9	Argentina	2001	Wine	89
9	Argentina	2002	Apples	51
9	Argentina	2002	Bananas	28
9	Argentina	2002	Beer	87
9	Argentina	2002	Beverages, Alcoholic	3
9	Argentina	2002	Bovine Meat	145
9	Argentina	2002	Butter, Ghee	4
9	Argentina	2002	Cassava	5
9	Argentina	2002	Cephalopods	5
9	Argentina	2002	Cereals, Other	1
9	Argentina	2002	Cheese	25
9	Argentina	2002	Cocoa Beans	1
9	Argentina	2002	Coffee	2
9	Argentina	2002	Cottonseed Oil	1
9	Argentina	2002	Demersal Fish	8
9	Argentina	2002	Eggs	16
9	Argentina	2002	Fats, Animals, Raw	6
9	Argentina	2002	Freshwater Fish	1
9	Argentina	2002	Fruits, Other	28
9	Argentina	2002	Grapefruit	10
9	Argentina	2002	Grapes	3
9	Argentina	2002	Lemons, Limes	31
9	Argentina	2002	Maize	29
9	Argentina	2002	Maize Germ Oil	2
9	Argentina	2002	Meat, Other	4
9	Argentina	2002	Milk, Whole	211
9	Argentina	2002	Molluscs, Other	2
9	Argentina	2002	Mutton & Goat Meat	4
9	Argentina	2002	Nuts	1
9	Argentina	2002	Oats	3
9	Argentina	2002	Offals, Edible	16
9	Argentina	2002	Olives	1
9	Argentina	2002	Onions	35
9	Argentina	2002	Oranges, Mandarines	82
9	Argentina	2002	Peas	1
9	Argentina	2002	Pelagic Fish	2
9	Argentina	2002	Pigmeat	13
9	Argentina	2002	Pineapples	1
9	Argentina	2002	Potatoes	131
9	Argentina	2002	Poultry Meat	52
9	Argentina	2002	Pulses, Other	1
9	Argentina	2002	Rice (Milled Equivalent)	15
9	Argentina	2002	Soyabean Oil	5
9	Argentina	2002	Sugar, Refined Equivalent	101
9	Argentina	2002	Sunflowerseed Oil	28
9	Argentina	2002	Sweet Potatoes	19
9	Argentina	2002	Sweeteners, Other	19
9	Argentina	2002	Tea	19
9	Argentina	2002	Tomatoes	50
9	Argentina	2002	Vegetables, Other	111
9	Argentina	2002	Wheat	302
9	Argentina	2002	Whey	1
9	Argentina	2002	Wine	87
9	Argentina	2003	Apples	51
9	Argentina	2003	Bananas	32
9	Argentina	2003	Beer	93
9	Argentina	2003	Beverages, Alcoholic	3
9	Argentina	2003	Bovine Meat	152
9	Argentina	2003	Butter, Ghee	4
9	Argentina	2003	Cassava	5
9	Argentina	2003	Cephalopods	4
9	Argentina	2003	Cereals, Other	1
9	Argentina	2003	Cheese	24
9	Argentina	2003	Cocoa Beans	1
9	Argentina	2003	Coconuts - Incl Copra	1
9	Argentina	2003	Coffee	3
9	Argentina	2003	Crustaceans	1
9	Argentina	2003	Demersal Fish	9
9	Argentina	2003	Eggs	19
9	Argentina	2003	Fats, Animals, Raw	6
9	Argentina	2003	Freshwater Fish	2
9	Argentina	2003	Fruits, Other	35
9	Argentina	2003	Grapefruit	6
9	Argentina	2003	Grapes	3
9	Argentina	2003	Lemons, Limes	27
9	Argentina	2003	Maize	29
9	Argentina	2003	Maize Germ Oil	1
9	Argentina	2003	Meat, Other	5
9	Argentina	2003	Milk, Whole	207
9	Argentina	2003	Molluscs, Other	2
9	Argentina	2003	Mutton & Goat Meat	4
9	Argentina	2003	Nuts	1
9	Argentina	2003	Oats	3
9	Argentina	2003	Offals, Edible	17
9	Argentina	2003	Olives	1
9	Argentina	2003	Onions	32
9	Argentina	2003	Oranges, Mandarines	75
9	Argentina	2003	Peas	1
9	Argentina	2003	Pelagic Fish	2

FAO country code	Country	Reference year	Food item	Gross food supply (g *capita*$^{-1}$ d^{-1})
9	Argentina	2003	Pigmeat	14
9	Argentina	2003	Pineapples	2
9	Argentina	2003	Potatoes	120
9	Argentina	2003	Poultry Meat	54
9	Argentina	2003	Pulses, Other	2
9	Argentina	2003	Rice (Milled Equivalent)	20
9	Argentina	2003	Soyabean Oil	5
9	Argentina	2003	Sugar, Refined Equivalent	101
9	Argentina	2003	Sunflowerseed Oil	28
9	Argentina	2003	Sweet Potatoes	19
9	Argentina	2003	Sweeteners, Other	19
9	Argentina	2003	Tea	18
9	Argentina	2003	Tomatoes	46
9	Argentina	2003	Vegetables, Other	110
9	Argentina	2003	Wheat	310
9	Argentina	2003	Whey	1
9	Argentina	2003	Wine	89
9	Argentina	2004	Apples	54
9	Argentina	2004	Bananas	33
9	Argentina	2004	Beer	91
9	Argentina	2004	Beverages, Alcoholic	4
9	Argentina	2004	Bovine Meat	155
9	Argentina	2004	Butter, Ghee	4
9	Argentina	2004	Cassava	5
9	Argentina	2004	Cephalopods	2
9	Argentina	2004	Cereals, Other	1
9	Argentina	2004	Cheese	23
9	Argentina	2004	Cocoa Beans	1
9	Argentina	2004	Coconuts - Incl Copra	1
9	Argentina	2004	Coffee	3
9	Argentina	2004	Cottonseed Oil	1
9	Argentina	2004	Demersal Fish	12
9	Argentina	2004	Eggs	22
9	Argentina	2004	Fats, Animals, Raw	6
9	Argentina	2004	Fruits, Other	37
9	Argentina	2004	Grapefruit	7
9	Argentina	2004	Grapes	3
9	Argentina	2004	Honey	1
9	Argentina	2004	Lemons, Limes	33
9	Argentina	2004	Maize	28
9	Argentina	2004	Maize Germ Oil	1
9	Argentina	2004	Meat, Other	4
9	Argentina	2004	Milk, Whole	141
9	Argentina	2004	Molluscs, Other	2
9	Argentina	2004	Mutton & Goat Meat	3
9	Argentina	2004	Nuts	1
9	Argentina	2004	Oats	3
9	Argentina	2004	Offals, Edible	16
9	Argentina	2004	Olive Oil	1
9	Argentina	2004	Olives	1
9	Argentina	2004	Onions	33
9	Argentina	2004	Oranges, Mandarines	64
9	Argentina	2004	Pelagic Fish	3
9	Argentina	2004	Pigmeat	14
9	Argentina	2004	Pineapples	2
9	Argentina	2004	Potatoes	110
9	Argentina	2004	Poultry Meat	60
9	Argentina	2004	Pulses, Other	2
9	Argentina	2004	Rice (Milled Equivalent)	22
9	Argentina	2004	Soyabean Oil	5
9	Argentina	2004	Sugar, Refined Equivalent	104
9	Argentina	2004	Sunflowerseed Oil	29
9	Argentina	2004	Sweet Potatoes	23
9	Argentina	2004	Sweeteners, Other	18
9	Argentina	2004	Tea	16
9	Argentina	2004	Tomatoes	43
9	Argentina	2004	Vegetables, Other	107
9	Argentina	2004	Wheat	299
9	Argentina	2004	Whey	2
9	Argentina	2004	Wine	80
9	Argentina	2005	Apples	38
9	Argentina	2005	Bananas	33
9	Argentina	2005	Beer	95
9	Argentina	2005	Beverages, Alcoholic	4
9	Argentina	2005	Bovine Meat	154
9	Argentina	2005	Butter, Ghee	4
9	Argentina	2005	Cassava	6
9	Argentina	2005	Cephalopods	4
9	Argentina	2005	Cereals, Other	1
9	Argentina	2005	Cheese	22
9	Argentina	2005	Cocoa Beans	2
9	Argentina	2005	Coconuts - Incl Copra	1
9	Argentina	2005	Coffee	3
9	Argentina	2005	Cottonseed Oil	1
9	Argentina	2005	Demersal Fish	9
9	Argentina	2005	Eggs	23
9	Argentina	2005	Fats, Animals, Raw	7
9	Argentina	2005	Fruits, Other	36
9	Argentina	2005	Grapefruit	10
9	Argentina	2005	Grapes	4
9	Argentina	2005	Lemons, Limes	28
9	Argentina	2005	Maize	28
9	Argentina	2005	Maize Germ Oil	1
9	Argentina	2005	Meat, Other	4
9	Argentina	2005	Milk, Whole	281
9	Argentina	2005	Molluscs, Other	2
9	Argentina	2005	Mutton & Goat Meat	4

FAO country code	Country	Reference year	Food item	Gross food supply (g *capita*$^{-1}$ d^{-1})
9	Argentina	2005	Nuts	1
9	Argentina	2005	Oats	3
9	Argentina	2005	Offals, Edible	17
9	Argentina	2005	Olive Oil	1
9	Argentina	2005	Olives	1
9	Argentina	2005	Onions	38
9	Argentina	2005	Oranges, Mandarines	61
9	Argentina	2005	Pelagic Fish	3
9	Argentina	2005	Pigmeat	15
9	Argentina	2005	Pineapples	2
9	Argentina	2005	Potatoes	93
9	Argentina	2005	Poultry Meat	67
9	Argentina	2005	Pulses, Other	2
9	Argentina	2005	Rice (Milled Equivalent)	17
9	Argentina	2005	Soyabean Oil	7
9	Argentina	2005	Sugar, Refined Equivalent	108
9	Argentina	2005	Sunflowerseed Oil	29
9	Argentina	2005	Sweet Potatoes	25
9	Argentina	2005	Sweeteners, Other	18
9	Argentina	2005	Tea	17
9	Argentina	2005	Tomatoes	45
9	Argentina	2005	Vegetables, Other	109
9	Argentina	2005	Wheat	295
9	Argentina	2005	Whey	4
9	Argentina	2005	Wine	78
9	Argentina	2006	Apples	34
9	Argentina	2006	Bananas	31
9	Argentina	2006	Beans	5
9	Argentina	2006	Beer	97
9	Argentina	2006	Beverages, Alcoholic	4
9	Argentina	2006	Bovine Meat	152
9	Argentina	2006	Butter, Ghee	3
9	Argentina	2006	Cassava	5
9	Argentina	2006	Cephalopods	8
9	Argentina	2006	Cereals, Other	1
9	Argentina	2006	Cheese	21
9	Argentina	2006	Cocoa Beans	2
9	Argentina	2006	Coconuts - Incl Copra	1
9	Argentina	2006	Coffee	3
9	Argentina	2006	Demersal Fish	8
9	Argentina	2006	Eggs	25
9	Argentina	2006	Fats, Animals, Raw	6
9	Argentina	2006	Fruits, Other	32
9	Argentina	2006	Grapefruit	11
9	Argentina	2006	Grapes	3
9	Argentina	2006	Lemons, Limes	33
9	Argentina	2006	Maize	28
9	Argentina	2006	Maize Germ Oil	1
9	Argentina	2006	Meat, Other	4
9	Argentina	2006	Milk, Whole	283
9	Argentina	2006	Molluscs, Other	3
9	Argentina	2006	Mutton & Goat Meat	4
9	Argentina	2006	Nuts	1
9	Argentina	2006	Oats	3
9	Argentina	2006	Offals, Edible	15
9	Argentina	2006	Onions	30
9	Argentina	2006	Oranges, Mandarines	67
9	Argentina	2006	Pelagic Fish	4
9	Argentina	2006	Pigmeat	18
9	Argentina	2006	Pineapples	2
9	Argentina	2006	Potatoes	97
9	Argentina	2006	Poultry Meat	76
9	Argentina	2006	Pulses, Other	1
9	Argentina	2006	Rice (Milled Equivalent)	18
9	Argentina	2006	Soyabean Oil	7
9	Argentina	2006	Sugar, Refined Equivalent	105
9	Argentina	2006	Sunflowerseed Oil	29
9	Argentina	2006	Sweet Potatoes	21
9	Argentina	2006	Sweeteners, Other	18
9	Argentina	2006	Tea	18
9	Argentina	2006	Tomatoes	44
9	Argentina	2006	Vegetables, Other	103
9	Argentina	2006	Wheat	289
9	Argentina	2006	Wine	78
9	Argentina	2007	Apples	25
9	Argentina	2007	Bananas	32
9	Argentina	2007	Beans	2
9	Argentina	2007	Beer	99
9	Argentina	2007	Beverages, Alcoholic	5
9	Argentina	2007	Bovine Meat	151
9	Argentina	2007	Butter, Ghee	3
9	Argentina	2007	Cassava	5
9	Argentina	2007	Cephalopods	6
9	Argentina	2007	Cereals, Other	1
9	Argentina	2007	Cheese	22
9	Argentina	2007	Cocoa Beans	2
9	Argentina	2007	Coconuts - Incl Copra	1
9	Argentina	2007	Coffee	3
9	Argentina	2007	Cottonseed Oil	1
9	Argentina	2007	Demersal Fish	6
9	Argentina	2007	Eggs	26
9	Argentina	2007	Fats, Animals, Raw	6
9	Argentina	2007	Fruits, Other	27
9	Argentina	2007	Grapefruit	10
9	Argentina	2007	Grapes	4
9	Argentina	2007	Lemons, Limes	28
9	Argentina	2007	Maize	27

FAO country code	Country	Reference year	Food item	Gross food supply (g $capita^{-1}$ d^{-1})
9	Argentina	2007	Maize Germ Oil	1
9	Argentina	2007	Meat, Other	4
9	Argentina	2007	Milk, Whole	306
9	Argentina	2007	Molluscs, Other	2
9	Argentina	2007	Mutton & Goat Meat	4
9	Argentina	2007	Nuts	1
9	Argentina	2007	Oats	3
9	Argentina	2007	Offals, Edible	14
9	Argentina	2007	Onions	34
9	Argentina	2007	Oranges, Mandarines	52
9	Argentina	2007	Pelagic Fish	5
9	Argentina	2007	Pigmeat	19
9	Argentina	2007	Pineapples	2
9	Argentina	2007	Potatoes	98
9	Argentina	2007	Poultry Meat	79
9	Argentina	2007	Pulses, Other	1
9	Argentina	2007	Rape and Mustard Oil	1
9	Argentina	2007	Rice (Milled Equivalent)	19
9	Argentina	2007	Soyabean Oil	7
9	Argentina	2007	Sugar, Refined Equivalent	105
9	Argentina	2007	Sunflowerseed Oil	29
9	Argentina	2007	Sweet Potatoes	22
9	Argentina	2007	Sweeteners, Other	18
9	Argentina	2007	Tea	18
9	Argentina	2007	Tomatoes	47
9	Argentina	2007	Vegetables, Other	104
9	Argentina	2007	Wheat	291
9	Argentina	2007	Whey	4
9	Argentina	2007	Wine	78
9	Argentina	2008	Apples	30
9	Argentina	2008	Bananas	34
9	Argentina	2008	Beans	2
9	Argentina	2008	Beer	117
9	Argentina	2008	Beverages, Alcoholic	5
9	Argentina	2008	Bovine Meat	150
9	Argentina	2008	Butter, Ghee	3
9	Argentina	2008	Cassava	5
9	Argentina	2008	Cephalopods	6
9	Argentina	2008	Cereals, Other	1
9	Argentina	2008	Cheese	32
9	Argentina	2008	Cocoa Beans	2
9	Argentina	2008	Coconuts - Incl Copra	1
9	Argentina	2008	Coffee	3
9	Argentina	2008	Demersal Fish	9
9	Argentina	2008	Eggs	27
9	Argentina	2008	Fats, Animals, Raw	5
9	Argentina	2008	Fruits, Other	34
9	Argentina	2008	Grapefruit	11
9	Argentina	2008	Grapes	3
9	Argentina	2008	Lemons, Limes	26
9	Argentina	2008	Maize	27
9	Argentina	2008	Maize Germ Oil	1
9	Argentina	2008	Meat, Other	3
9	Argentina	2008	Milk, Whole	246
9	Argentina	2008	Molluscs, Other	3
9	Argentina	2008	Mutton & Goat Meat	3
9	Argentina	2008	Nuts	1
9	Argentina	2008	Oats	2
9	Argentina	2008	Offals, Edible	14
9	Argentina	2008	Onions	32
9	Argentina	2008	Oranges, Mandarines	66
9	Argentina	2008	Pelagic Fish	3
9	Argentina	2008	Pigmeat	21
9	Argentina	2008	Pineapples	2
9	Argentina	2008	Potatoes	93
9	Argentina	2008	Poultry Meat	86
9	Argentina	2008	Pulses, Other	1
9	Argentina	2008	Rice (Milled Equivalent)	22
9	Argentina	2008	Soyabean Oil	7
9	Argentina	2008	Sugar, Refined Equivalent	104
9	Argentina	2008	Sunflowerseed Oil	29
9	Argentina	2008	Sweet Potatoes	22
9	Argentina	2008	Sweeteners, Other	18
9	Argentina	2008	Tea	14
9	Argentina	2008	Tomatoes	47
9	Argentina	2008	Vegetables, Other	104
9	Argentina	2008	Wheat	288
9	Argentina	2008	Whey	4
9	Argentina	2008	Wine	74
9	Argentina	2009	Apples	32
9	Argentina	2009	Bananas	33
9	Argentina	2009	Beans	1
9	Argentina	2009	Beer	117
9	Argentina	2009	Beverages, Alcoholic	5
9	Argentina	2009	Bovine Meat	149
9	Argentina	2009	Butter, Ghee	3
9	Argentina	2009	Cassava	5
9	Argentina	2009	Cephalopods	1
9	Argentina	2009	Cereals, Other	1
9	Argentina	2009	Cheese	32
9	Argentina	2009	Cocoa Beans	2
9	Argentina	2009	Coconuts - Incl Copra	1
9	Argentina	2009	Coffee	3
9	Argentina	2009	Demersal Fish	6
9	Argentina	2009	Eggs	30
9	Argentina	2009	Fats, Animals, Raw	6
9	Argentina	2009	Fruits, Other	31

FAO country code	Country	Reference year	Food item	Gross food supply (g *capita*$^{-1}$ d^{-1})
9	Argentina	2009	Grapefruit	12
9	Argentina	2009	Grapes	2
9	Argentina	2009	Lemons, Limes	33
9	Argentina	2009	Maize	28
9	Argentina	2009	Maize Germ Oil	1
9	Argentina	2009	Meat, Other	4
9	Argentina	2009	Milk, Whole	209
9	Argentina	2009	Molluscs, Other	4
9	Argentina	2009	Mutton & Goat Meat	3
9	Argentina	2009	Nuts	1
9	Argentina	2009	Oats	2
9	Argentina	2009	Offals, Edible	15
9	Argentina	2009	Onions	30
9	Argentina	2009	Oranges, Mandarines	61
9	Argentina	2009	Pelagic Fish	4
9	Argentina	2009	Pigmeat	22
9	Argentina	2009	Pineapples	2
9	Argentina	2009	Potatoes	96
9	Argentina	2009	Poultry Meat	92
9	Argentina	2009	Pulses, Other	1
9	Argentina	2009	Rice (Milled Equivalent)	21
9	Argentina	2009	Soyabean Oil	7
9	Argentina	2009	Sugar, Refined Equivalent	103
9	Argentina	2009	Sunflowerseed Oil	30
9	Argentina	2009	Sweet Potatoes	22
9	Argentina	2009	Sweeteners, Other	17
9	Argentina	2009	Tea	13
9	Argentina	2009	Tomatoes	43
9	Argentina	2009	Vegetables, Other	107
9	Argentina	2009	Wheat	283
9	Argentina	2009	Whey	2
9	Argentina	2009	Wine	71
9	Argentina	2010	Apples	39
9	Argentina	2010	Bananas	33
9	Argentina	2010	Beer	116
9	Argentina	2010	Beverages, Alcoholic	5
9	Argentina	2010	Bovine Meat	150
9	Argentina	2010	Butter, Ghee	3
9	Argentina	2010	Cassava	5
9	Argentina	2010	Cephalopods	2
9	Argentina	2010	Cereals, Other	1
9	Argentina	2010	Cheese	32
9	Argentina	2010	Cocoa Beans	2
9	Argentina	2010	Coconuts - Incl Copra	1
9	Argentina	2010	Coffee	3
9	Argentina	2010	Crustaceans	1
9	Argentina	2010	Demersal Fish	6
9	Argentina	2010	Eggs	32
9	Argentina	2010	Fats, Animals, Raw	5
9	Argentina	2010	Fruits, Other	40
9	Argentina	2010	Grapefruit	9
9	Argentina	2010	Grapes	2
9	Argentina	2010	Lemons, Limes	23
9	Argentina	2010	Maize	27
9	Argentina	2010	Maize Germ Oil	1
9	Argentina	2010	Meat, Other	3
9	Argentina	2010	Milk, Whole	229
9	Argentina	2010	Molluscs, Other	2
9	Argentina	2010	Mutton & Goat Meat	3
9	Argentina	2010	Nuts	1
9	Argentina	2010	Oats	3
9	Argentina	2010	Offals, Edible	15
9	Argentina	2010	Onions	31
9	Argentina	2010	Oranges, Mandarines	58
9	Argentina	2010	Pelagic Fish	4
9	Argentina	2010	Pigmeat	23
9	Argentina	2010	Pineapples	2
9	Argentina	2010	Potatoes	93
9	Argentina	2010	Poultry Meat	95
9	Argentina	2010	Pulses, Other	1
9	Argentina	2010	Rice (Milled Equivalent)	23
9	Argentina	2010	Soyabean Oil	7
9	Argentina	2010	Sugar, Refined Equivalent	103
9	Argentina	2010	Sunflowerseed Oil	30
9	Argentina	2010	Sweet Potatoes	22
9	Argentina	2010	Sweeteners, Other	17
9	Argentina	2010	Tea	15
9	Argentina	2010	Tomatoes	45
9	Argentina	2010	Vegetables, Other	106
9	Argentina	2010	Wheat	284
9	Argentina	2010	Whey	1
9	Argentina	2010	Wine	66
9	Argentina	2011	Apples	39
9	Argentina	2011	Bananas	35
9	Argentina	2011	Beer	112
9	Argentina	2011	Beverages, Alcoholic	5
9	Argentina	2011	Bovine Meat	151
9	Argentina	2011	Butter, Ghee	2
9	Argentina	2011	Cassava	5
9	Argentina	2011	Cephalopods	2
9	Argentina	2011	Cereals, Other	1
9	Argentina	2011	Cheese	32
9	Argentina	2011	Cocoa Beans	2
9	Argentina	2011	Coconuts - Incl Copra	1
9	Argentina	2011	Coffee	3
9	Argentina	2011	Crustaceans	1
9	Argentina	2011	Demersal Fish	6

FAO country code	Country	Reference year	Food item	Gross food supply (g *capita*$^{-1}$ d^{-1})
9	Argentina	2011	Eggs	34
9	Argentina	2011	Fats, Animals, Raw	5
9	Argentina	2011	Fruits, Other	28
9	Argentina	2011	Grapefruit	10
9	Argentina	2011	Grapes	2
9	Argentina	2011	Lemons, Limes	14
9	Argentina	2011	Maize	27
9	Argentina	2011	Maize Germ Oil	1
9	Argentina	2011	Meat, Other	3
9	Argentina	2011	Milk, Whole	214
9	Argentina	2011	Molluscs, Other	2
9	Argentina	2011	Mutton & Goat Meat	3
9	Argentina	2011	Nuts	1
9	Argentina	2011	Oats	3
9	Argentina	2011	Offals, Edible	15
9	Argentina	2011	Onions	34
9	Argentina	2011	Oranges, Mandarines	59
9	Argentina	2011	Pelagic Fish	4
9	Argentina	2011	Pigmeat	25
9	Argentina	2011	Pineapples	3
9	Argentina	2011	Potatoes	99
9	Argentina	2011	Poultry Meat	97
9	Argentina	2011	Pulses, Other	1
9	Argentina	2011	Rice (Milled Equivalent)	21
9	Argentina	2011	Soyabean Oil	7
9	Argentina	2011	Sugar, Refined Equivalent	104
9	Argentina	2011	Sunflowerseed Oil	31
9	Argentina	2011	Sweet Potatoes	24
9	Argentina	2011	Sweeteners, Other	16
9	Argentina	2011	Tea	17
9	Argentina	2011	Tomatoes	47
9	Argentina	2011	Vegetables, Other	112
9	Argentina	2011	Wheat	283
9	Argentina	2011	Whey	2
9	Argentina	2011	Wine	66
1	Armenia	1992	Apples	48
1	Armenia	1992	Beer	1
1	Armenia	1992	Beverages, Alcoholic	6
1	Armenia	1992	Bovine Meat	22
1	Armenia	1992	Butter, Ghee	2
1	Armenia	1992	Cereals, Other	2
1	Armenia	1992	Cheese	1
1	Armenia	1992	Eggs	10
1	Armenia	1992	Fats, Animals, Raw	2
1	Armenia	1992	Freshwater Fish	4
1	Armenia	1992	Fruits, Other	47
1	Armenia	1992	Grapes	73
1	Armenia	1992	Honey	1
1	Armenia	1992	Milk, Whole	292
1	Armenia	1992	Mutton & Goat Meat	7
1	Armenia	1992	Offals, Edible	4
1	Armenia	1992	Onions	38
1	Armenia	1992	Pigmeat	13
1	Armenia	1992	Potatoes	175
1	Armenia	1992	Poultry Meat	9
1	Armenia	1992	Rice (Milled Equivalent)	20
1	Armenia	1992	Soyabean Oil	1
1	Armenia	1992	Sugar, Refined Equivalent	56
1	Armenia	1992	Tomatoes	147
1	Armenia	1992	Vegetables, Other	178
1	Armenia	1992	Wheat	428
1	Armenia	1992	Wine	20
1	Armenia	1993	Apples	16
1	Armenia	1993	Beverages, Alcoholic	6
1	Armenia	1993	Bovine Meat	34
1	Armenia	1993	Butter, Ghee	6
1	Armenia	1993	Cereals, Other	3
1	Armenia	1993	Cheese	1
1	Armenia	1993	Cottonseed Oil	1
1	Armenia	1993	Eggs	7
1	Armenia	1993	Fats, Animals, Raw	1
1	Armenia	1993	Freshwater Fish	3
1	Armenia	1993	Fruits, Other	17
1	Armenia	1993	Grapes	68
1	Armenia	1993	Honey	1
1	Armenia	1993	Milk, Whole	121
1	Armenia	1993	Mutton & Goat Meat	7
1	Armenia	1993	Offals, Edible	5
1	Armenia	1993	Onions	30
1	Armenia	1993	Pigmeat	4
1	Armenia	1993	Potatoes	200
1	Armenia	1993	Poultry Meat	5
1	Armenia	1993	Rice (Milled Equivalent)	15
1	Armenia	1993	Sugar, Refined Equivalent	57
1	Armenia	1993	Tomatoes	116
1	Armenia	1993	Vegetables, Other	147
1	Armenia	1993	Wheat	442
1	Armenia	1993	Wine	23
1	Armenia	1994	Apples	26
1	Armenia	1994	Beer	2
1	Armenia	1994	Beverages, Alcoholic	15
1	Armenia	1994	Bovine Meat	39
1	Armenia	1994	Butter, Ghee	6
1	Armenia	1994	Cereals, Other	1
1	Armenia	1994	Cheese	1
1	Armenia	1994	Cottonseed Oil	2
1	Armenia	1994	Eggs	9

FAO country code	Country	Reference year	Food item	Gross food supply (g *capita*$^{-1}$ d^{-1})
1	Armenia	1994	Fats, Animals, Raw	1
1	Armenia	1994	Freshwater Fish	2
1	Armenia	1994	Fruits, Other	47
1	Armenia	1994	Grapes	76
1	Armenia	1994	Honey	1
1	Armenia	1994	Milk, Whole	265
1	Armenia	1994	Mutton & Goat Meat	6
1	Armenia	1994	Offals, Edible	6
1	Armenia	1994	Onions	30
1	Armenia	1994	Pigmeat	5
1	Armenia	1994	Potatoes	219
1	Armenia	1994	Poultry Meat	4
1	Armenia	1994	Rice (Milled Equivalent)	14
1	Armenia	1994	Soyabean Oil	3
1	Armenia	1994	Sugar, Refined Equivalent	54
1	Armenia	1994	Sunflowerseed Oil	2
1	Armenia	1994	Tomatoes	168
1	Armenia	1994	Vegetables, Other	162
1	Armenia	1994	Wheat	421
1	Armenia	1994	Wine	20
1	Armenia	1995	Apples	46
1	Armenia	1995	Barley	1
1	Armenia	1995	Beverages, Alcoholic	12
1	Armenia	1995	Bovine Meat	45
1	Armenia	1995	Butter, Ghee	8
1	Armenia	1995	Cereals, Other	1
1	Armenia	1995	Cheese	1
1	Armenia	1995	Coffee	2
1	Armenia	1995	Cottonseed Oil	5
1	Armenia	1995	Cream	1
1	Armenia	1995	Dates	1
1	Armenia	1995	Eggs	10
1	Armenia	1995	Fats, Animals, Raw	2
1	Armenia	1995	Freshwater Fish	2
1	Armenia	1995	Fruits, Other	54
1	Armenia	1995	Grapes	86
1	Armenia	1995	Honey	1
1	Armenia	1995	Marine Fish, Other	1
1	Armenia	1995	Milk, Whole	133
1	Armenia	1995	Mutton & Goat Meat	6
1	Armenia	1995	Offals, Edible	7
1	Armenia	1995	Oilcrops Oil, Other	3
1	Armenia	1995	Onions	37
1	Armenia	1995	Oranges, Mandarines	4
1	Armenia	1995	Pelagic Fish	1
1	Armenia	1995	Pigmeat	4
1	Armenia	1995	Potatoes	250
1	Armenia	1995	Poultry Meat	7
1	Armenia	1995	Rice (Milled Equivalent)	8
1	Armenia	1995	Soyabean Oil	1
1	Armenia	1995	Sugar, Refined Equivalent	51
1	Armenia	1995	Sunflowerseed Oil	3
1	Armenia	1995	Sweeteners, Other	5
1	Armenia	1995	Tomatoes	143
1	Armenia	1995	Vegetables, Other	181
1	Armenia	1995	Wheat	423
1	Armenia	1995	Wine	9
1	Armenia	1996	Apples	33
1	Armenia	1996	Bananas	1
1	Armenia	1996	Barley	1
1	Armenia	1996	Beer	3
1	Armenia	1996	Beverages, Alcoholic	12
1	Armenia	1996	Bovine Meat	28
1	Armenia	1996	Butter, Ghee	5
1	Armenia	1996	Cereals, Other	49
1	Armenia	1996	Cheese	2
1	Armenia	1996	Cocoa Beans	1
1	Armenia	1996	Coffee	3
1	Armenia	1996	Dates	1
1	Armenia	1996	Eggs	11
1	Armenia	1996	Fats, Animals, Raw	3
1	Armenia	1996	Freshwater Fish	1
1	Armenia	1996	Fruits, Other	65
1	Armenia	1996	Grapefruit	1
1	Armenia	1996	Grapes	73
1	Armenia	1996	Honey	1
1	Armenia	1996	Milk, Whole	205
1	Armenia	1996	Mutton & Goat Meat	5
1	Armenia	1996	Offals, Edible	6
1	Armenia	1996	Oilcrops Oil, Other	5
1	Armenia	1996	Olive Oil	3
1	Armenia	1996	Onions	33
1	Armenia	1996	Oranges, Mandarines	11
1	Armenia	1996	Pigmeat	2
1	Armenia	1996	Potatoes	223
1	Armenia	1996	Poultry Meat	16
1	Armenia	1996	Rice (Milled Equivalent)	8
1	Armenia	1996	Rye	6
1	Armenia	1996	Sesameseed Oil	1
1	Armenia	1996	Sugar, Refined Equivalent	55
1	Armenia	1996	Sunflowerseed Oil	7
1	Armenia	1996	Sweeteners, Other	1
1	Armenia	1996	Tomatoes	127
1	Armenia	1996	Vegetables, Other	207
1	Armenia	1996	Wheat	428
1	Armenia	1996	Wine	7
1	Armenia	1997	Apples	27

FAO country code	Country	Reference year	Food item	Gross food supply (g *capita*$^{-1}$ d^{-1})
1	Armenia	1997	Bananas	1
1	Armenia	1997	Barley	3
1	Armenia	1997	Beer	6
1	Armenia	1997	Beverages, Alcoholic	12
1	Armenia	1997	Bovine Meat	33
1	Armenia	1997	Butter, Ghee	5
1	Armenia	1997	Cereals, Other	23
1	Armenia	1997	Cheese	4
1	Armenia	1997	Coffee	4
1	Armenia	1997	Cream	1
1	Armenia	1997	Eggs	11
1	Armenia	1997	Fats, Animals, Raw	3
1	Armenia	1997	Freshwater Fish	1
1	Armenia	1997	Fruits, Other	47
1	Armenia	1997	Grapefruit	1
1	Armenia	1997	Grapes	57
1	Armenia	1997	Honey	1
1	Armenia	1997	Milk, Whole	183
1	Armenia	1997	Mutton & Goat Meat	4
1	Armenia	1997	Offals, Edible	7
1	Armenia	1997	Oilcrops Oil, Other	6
1	Armenia	1997	Onions	28
1	Armenia	1997	Oranges, Mandarines	8
1	Armenia	1997	Pigmeat	2
1	Armenia	1997	Potatoes	207
1	Armenia	1997	Poultry Meat	17
1	Armenia	1997	Rice (Milled Equivalent)	6
1	Armenia	1997	Sesameseed Oil	1
1	Armenia	1997	Soyabean Oil	1
1	Armenia	1997	Sugar, Refined Equivalent	54
1	Armenia	1997	Sunflowerseed Oil	8
1	Armenia	1997	Sweeteners, Other	1
1	Armenia	1997	Tomatoes	106
1	Armenia	1997	Vegetables, Other	185
1	Armenia	1997	Wheat	439
1	Armenia	1997	Wine	4
1	Armenia	1998	Apples	14
1	Armenia	1998	Bananas	1
1	Armenia	1998	Barley	3
1	Armenia	1998	Beer	12
1	Armenia	1998	Beverages, Alcoholic	7
1	Armenia	1998	Bovine Meat	34
1	Armenia	1998	Butter, Ghee	4
1	Armenia	1998	Cereals, Other	22
1	Armenia	1998	Cheese	4
1	Armenia	1998	Citrus, Other	1
1	Armenia	1998	Cocoa Beans	1
1	Armenia	1998	Coffee	4
1	Armenia	1998	Eggs	9
1	Armenia	1998	Fats, Animals, Raw	2
1	Armenia	1998	Freshwater Fish	1
1	Armenia	1998	Fruits, Other	66
1	Armenia	1998	Grapefruit	1
1	Armenia	1998	Grapes	53
1	Armenia	1998	Honey	1
1	Armenia	1998	Marine Fish, Other	1
1	Armenia	1998	Milk, Whole	197
1	Armenia	1998	Mutton & Goat Meat	4
1	Armenia	1998	Offals, Edible	7
1	Armenia	1998	Oilcrops Oil, Other	3
1	Armenia	1998	Onions	31
1	Armenia	1998	Oranges, Mandarines	7
1	Armenia	1998	Pigmeat	3
1	Armenia	1998	Pineapples	1
1	Armenia	1998	Potatoes	229
1	Armenia	1998	Poultry Meat	18
1	Armenia	1998	Rice (Milled Equivalent)	10
1	Armenia	1998	Sesameseed Oil	2
1	Armenia	1998	Soyabean Oil	3
1	Armenia	1998	Sugar, Refined Equivalent	63
1	Armenia	1998	Sunflowerseed Oil	2
1	Armenia	1998	Tomatoes	100
1	Armenia	1998	Vegetables, Other	204
1	Armenia	1998	Wheat	435
1	Armenia	1998	Wine	2
1	Armenia	1999	Apples	8
1	Armenia	1999	Bananas	1
1	Armenia	1999	Barley	3
1	Armenia	1999	Beer	7
1	Armenia	1999	Beverages, Alcoholic	8
1	Armenia	1999	Bovine Meat	29
1	Armenia	1999	Butter, Ghee	4
1	Armenia	1999	Cereals, Other	16
1	Armenia	1999	Cheese	4
1	Armenia	1999	Citrus, Other	2
1	Armenia	1999	Cocoa Beans	1
1	Armenia	1999	Coffee	6
1	Armenia	1999	Eggs	13
1	Armenia	1999	Fats, Animals, Raw	3
1	Armenia	1999	Freshwater Fish	2
1	Armenia	1999	Fruits, Other	51
1	Armenia	1999	Grapes	40
1	Armenia	1999	Honey	1
1	Armenia	1999	Milk, Whole	221
1	Armenia	1999	Mutton & Goat Meat	4
1	Armenia	1999	Offals, Edible	7
1	Armenia	1999	Oilcrops Oil, Other	3

FAO country code	Country	Reference year	Food item	Gross food supply (g *capita*$^{-1}$ d^{-1})
1	Armenia	1999	Olives	1
1	Armenia	1999	Onions	35
1	Armenia	1999	Oranges, Mandarines	9
1	Armenia	1999	Pelagic Fish	1
1	Armenia	1999	Pigmeat	3
1	Armenia	1999	Potatoes	231
1	Armenia	1999	Poultry Meat	19
1	Armenia	1999	Rice (Milled Equivalent)	9
1	Armenia	1999	Soyabean Oil	4
1	Armenia	1999	Sugar, Refined Equivalent	62
1	Armenia	1999	Sunflowerseed Oil	1
1	Armenia	1999	Tomatoes	90
1	Armenia	1999	Vegetables, Other	253
1	Armenia	1999	Wheat	433
1	Armenia	1999	Wine	5
1	Armenia	2000	Apples	11
1	Armenia	2000	Bananas	1
1	Armenia	2000	Barley	4
1	Armenia	2000	Beer	6
1	Armenia	2000	Beverages, Alcoholic	7
1	Armenia	2000	Bovine Meat	29
1	Armenia	2000	Butter, Ghee	3
1	Armenia	2000	Cereals, Other	2
1	Armenia	2000	Cheese	4
1	Armenia	2000	Cocoa Beans	1
1	Armenia	2000	Coffee	7
1	Armenia	2000	Eggs	14
1	Armenia	2000	Fats, Animals, Raw	3
1	Armenia	2000	Freshwater Fish	2
1	Armenia	2000	Fruits, Other	85
1	Armenia	2000	Grapes	34
1	Armenia	2000	Honey	1
1	Armenia	2000	Milk, Whole	222
1	Armenia	2000	Mutton & Goat Meat	7
1	Armenia	2000	Nuts	1
1	Armenia	2000	Offals, Edible	8
1	Armenia	2000	Oilcrops Oil, Other	5
1	Armenia	2000	Olives	1
1	Armenia	2000	Onions	27
1	Armenia	2000	Oranges, Mandarines	1
1	Armenia	2000	Pelagic Fish	1
1	Armenia	2000	Pigmeat	7
1	Armenia	2000	Potatoes	175
1	Armenia	2000	Poultry Meat	15
1	Armenia	2000	Rice (Milled Equivalent)	8
1	Armenia	2000	Soyabean Oil	2
1	Armenia	2000	Sugar, Refined Equivalent	61
1	Armenia	2000	Sunflowerseed Oil	3
1	Armenia	2000	Tomatoes	107
1	Armenia	2000	Vegetables, Other	196
1	Armenia	2000	Wheat	429
1	Armenia	2000	Wine	4
1	Armenia	2001	Apples	19
1	Armenia	2001	Bananas	1
1	Armenia	2001	Barley	5
1	Armenia	2001	Beer	5
1	Armenia	2001	Beverages, Alcoholic	6
1	Armenia	2001	Bovine Meat	29
1	Armenia	2001	Butter, Ghee	3
1	Armenia	2001	Cereals, Other	6
1	Armenia	2001	Cheese	4
1	Armenia	2001	Citrus, Other	1
1	Armenia	2001	Cocoa Beans	2
1	Armenia	2001	Coffee	10
1	Armenia	2001	Eggs	15
1	Armenia	2001	Fats, Animals, Raw	3
1	Armenia	2001	Freshwater Fish	2
1	Armenia	2001	Fruits, Other	56
1	Armenia	2001	Grapes	54
1	Armenia	2001	Honey	1
1	Armenia	2001	Milk, Whole	228
1	Armenia	2001	Mutton & Goat Meat	6
1	Armenia	2001	Nuts	1
1	Armenia	2001	Offals, Edible	7
1	Armenia	2001	Oilcrops Oil, Other	2
1	Armenia	2001	Olives	1
1	Armenia	2001	Onions	29
1	Armenia	2001	Oranges, Mandarines	2
1	Armenia	2001	Pelagic Fish	1
1	Armenia	2001	Pigmeat	8
1	Armenia	2001	Potatoes	178
1	Armenia	2001	Poultry Meat	17
1	Armenia	2001	Rice (Milled Equivalent)	8
1	Armenia	2001	Soyabean Oil	2
1	Armenia	2001	Sugar, Refined Equivalent	65
1	Armenia	2001	Sunflowerseed Oil	7
1	Armenia	2001	Tomatoes	81
1	Armenia	2001	Vegetables, Other	251
1	Armenia	2001	Wheat	413
1	Armenia	2001	Wine	6
1	Armenia	2002	Apples	22
1	Armenia	2002	Bananas	2
1	Armenia	2002	Barley	6
1	Armenia	2002	Beer	5
1	Armenia	2002	Beverages, Alcoholic	5
1	Armenia	2002	Bovine Meat	32
1	Armenia	2002	Butter, Ghee	4

FAO countrycode	Country	Reference year	Food item	Gross food supply (g *capita*$^{-1}$ d^{-1})
1	Armenia	2002	Cereals, Other	4
1	Armenia	2002	Cheese	4
1	Armenia	2002	Cocoa Beans	2
1	Armenia	2002	Coffee	9
1	Armenia	2002	Eggs	16
1	Armenia	2002	Fats, Animals, Raw	4
1	Armenia	2002	Freshwater Fish	1
1	Armenia	2002	Fruits, Other	40
1	Armenia	2002	Grapes	51
1	Armenia	2002	Honey	1
1	Armenia	2002	Milk, Whole	302
1	Armenia	2002	Mutton & Goat Meat	5
1	Armenia	2002	Nuts	1
1	Armenia	2002	Offals, Edible	8
1	Armenia	2002	Oilcrops Oil, Other	2
1	Armenia	2002	Olives	1
1	Armenia	2002	Onions	33
1	Armenia	2002	Oranges, Mandarines	2
1	Armenia	2002	Pelagic Fish	2
1	Armenia	2002	Pigmeat	9
1	Armenia	2002	Pineapples	1
1	Armenia	2002	Potatoes	137
1	Armenia	2002	Poultry Meat	17
1	Armenia	2002	Rice (Milled Equivalent)	12
1	Armenia	2002	Soyabean Oil	1
1	Armenia	2002	Sugar, Refined Equivalent	70
1	Armenia	2002	Sunflowerseed Oil	9
1	Armenia	2002	Tomatoes	106
1	Armenia	2002	Vegetables, Other	284
1	Armenia	2002	Wheat	398
1	Armenia	2002	Wine	4
1	Armenia	2003	Apples	24
1	Armenia	2003	Bananas	2
1	Armenia	2003	Barley	7
1	Armenia	2003	Beer	5
1	Armenia	2003	Beverages, Alcoholic	5
1	Armenia	2003	Bovine Meat	38
1	Armenia	2003	Butter, Ghee	3
1	Armenia	2003	Cereals, Other	6
1	Armenia	2003	Cheese	4
1	Armenia	2003	Cocoa Beans	3
1	Armenia	2003	Coffee	8
1	Armenia	2003	Eggs	23
1	Armenia	2003	Fats, Animals, Raw	6
1	Armenia	2003	Freshwater Fish	1
1	Armenia	2003	Fruits, Other	43
1	Armenia	2003	Grapes	37
1	Armenia	2003	Honey	1
1	Armenia	2003	Milk, Whole	318
1	Armenia	2003	Mutton & Goat Meat	6
1	Armenia	2003	Nuts	1
1	Armenia	2003	Offals, Edible	8
1	Armenia	2003	Oilcrops Oil, Other	2
1	Armenia	2003	Olives	2
1	Armenia	2003	Onions	30
1	Armenia	2003	Oranges, Mandarines	2
1	Armenia	2003	Palmkernel Oil	1
1	Armenia	2003	Pelagic Fish	1
1	Armenia	2003	Pigmeat	9
1	Armenia	2003	Potatoes	132
1	Armenia	2003	Poultry Meat	16
1	Armenia	2003	Rice (Milled Equivalent)	11
1	Armenia	2003	Soyabean Oil	2
1	Armenia	2003	Sugar, Refined Equivalent	79
1	Armenia	2003	Sunflowerseed Oil	12
1	Armenia	2003	Tomatoes	144
1	Armenia	2003	Vegetables, Other	342
1	Armenia	2003	Wheat	409
1	Armenia	2003	Wine	2
1	Armenia	2004	Apples	29
1	Armenia	2004	Bananas	4
1	Armenia	2004	Barley	8
1	Armenia	2004	Beer	6
1	Armenia	2004	Beverages, Alcoholic	9
1	Armenia	2004	Bovine Meat	41
1	Armenia	2004	Butter, Ghee	5
1	Armenia	2004	Cereals, Other	4
1	Armenia	2004	Cheese	13
1	Armenia	2004	Cocoa Beans	3
1	Armenia	2004	Coffee	6
1	Armenia	2004	Eggs	24
1	Armenia	2004	Fats, Animals, Raw	6
1	Armenia	2004	Fruits, Other	57
1	Armenia	2004	Grapefruit	1
1	Armenia	2004	Grapes	31
1	Armenia	2004	Honey	1
1	Armenia	2004	Milk, Whole	247
1	Armenia	2004	Mutton & Goat Meat	7
1	Armenia	2004	Nuts	1
1	Armenia	2004	Offals, Edible	8
1	Armenia	2004	Oilcrops Oil, Other	2
1	Armenia	2004	Olives	2
1	Armenia	2004	Onions	39
1	Armenia	2004	Oranges, Mandarines	6
1	Armenia	2004	Palmkernel Oil	1
1	Armenia	2004	Pelagic Fish	1
1	Armenia	2004	Pigmeat	9

FAO country code	Country	Reference year	Food item	Gross food supply (g *capita*$^{-1}$ d^{-1})
1	Armenia	2004	Potatoes	165
1	Armenia	2004	Poultry Meat	16
1	Armenia	2004	Rice (Milled Equivalent)	10
1	Armenia	2004	Soyabean Oil	1
1	Armenia	2004	Sugar, Refined Equivalent	77
1	Armenia	2004	Sunflowerseed Oil	7
1	Armenia	2004	Tomatoes	185
1	Armenia	2004	Vegetables, Other	373
1	Armenia	2004	Wheat	436
1	Armenia	2004	Wine	6
1	Armenia	2005	Apples	68
1	Armenia	2005	Bananas	7
1	Armenia	2005	Barley	7
1	Armenia	2005	Beer	8
1	Armenia	2005	Beverages, Alcoholic	8
1	Armenia	2005	Bovine Meat	40
1	Armenia	2005	Butter, Ghee	4
1	Armenia	2005	Cereals, Other	5
1	Armenia	2005	Cheese	11
1	Armenia	2005	Cocoa Beans	3
1	Armenia	2005	Coconut Oil	1
1	Armenia	2005	Coffee	6
1	Armenia	2005	Dates	1
1	Armenia	2005	Eggs	24
1	Armenia	2005	Fats, Animals, Raw	5
1	Armenia	2005	Fruits, Other	93
1	Armenia	2005	Grapefruit	1
1	Armenia	2005	Grapes	77
1	Armenia	2005	Honey	1
1	Armenia	2005	Marine Fish, Other	5
1	Armenia	2005	Milk, Whole	316
1	Armenia	2005	Mutton & Goat Meat	7
1	Armenia	2005	Nuts	1
1	Armenia	2005	Offals, Edible	10
1	Armenia	2005	Oilcrops Oil, Other	3
1	Armenia	2005	Olives	2
1	Armenia	2005	Onions	45
1	Armenia	2005	Oranges, Mandarines	9
1	Armenia	2005	Palmkernel Oil	1
1	Armenia	2005	Pelagic Fish	2
1	Armenia	2005	Pigmeat	13
1	Armenia	2005	Pineapples	1
1	Armenia	2005	Potatoes	195
1	Armenia	2005	Poultry Meat	20
1	Armenia	2005	Rice (Milled Equivalent)	13
1	Armenia	2005	Soyabean Oil	1
1	Armenia	2005	Sugar, Refined Equivalent	85
1	Armenia	2005	Sunflowerseed Oil	11
1	Armenia	2005	Tomatoes	167
1	Armenia	2005	Vegetables, Other	428
1	Armenia	2005	Wheat	394
1	Armenia	2005	Wine	6
1	Armenia	2006	Apples	64
1	Armenia	2006	Bananas	5
1	Armenia	2006	Barley	7
1	Armenia	2006	Beer	13
1	Armenia	2006	Beverages, Alcoholic	8
1	Armenia	2006	Bovine Meat	45
1	Armenia	2006	Butter, Ghee	3
1	Armenia	2006	Cereals, Other	5
1	Armenia	2006	Cheese	13
1	Armenia	2006	Cocoa Beans	4
1	Armenia	2006	Coconut Oil	1
1	Armenia	2006	Coffee	4
1	Armenia	2006	Eggs	22
1	Armenia	2006	Fats, Animals, Raw	6
1	Armenia	2006	Fruits, Other	111
1	Armenia	2006	Grapefruit	1
1	Armenia	2006	Grapes	67
1	Armenia	2006	Honey	1
1	Armenia	2006	Lemons, Limes	1
1	Armenia	2006	Marine Fish, Other	2
1	Armenia	2006	Milk, Whole	349
1	Armenia	2006	Mutton & Goat Meat	7
1	Armenia	2006	Nuts	1
1	Armenia	2006	Offals, Edible	10
1	Armenia	2006	Oilcrops Oil, Other	4
1	Armenia	2006	Olives	3
1	Armenia	2006	Onions	61
1	Armenia	2006	Oranges, Mandarines	15
1	Armenia	2006	Palmkernel Oil	1
1	Armenia	2006	Pelagic Fish	2
1	Armenia	2006	Pigmeat	18
1	Armenia	2006	Pineapples	1
1	Armenia	2006	Potatoes	178
1	Armenia	2006	Poultry Meat	14
1	Armenia	2006	Rice (Milled Equivalent)	15
1	Armenia	2006	Soyabean Oil	1
1	Armenia	2006	Sugar, Refined Equivalent	84
1	Armenia	2006	Sunflowerseed Oil	10
1	Armenia	2006	Tomatoes	268
1	Armenia	2006	Vegetables, Other	465
1	Armenia	2006	Wheat	406
1	Armenia	2006	Wine	4
1	Armenia	2007	Apples	64
1	Armenia	2007	Bananas	14
1	Armenia	2007	Beer	16

FAO country code	Country	Reference year	Food item	Gross food supply (g *capita*$^{-1}$ d^{-1})
1	Armenia	2007	Beverages, Alcoholic	5
1	Armenia	2007	Bovine Meat	46
1	Armenia	2007	Butter, Ghee	2
1	Armenia	2007	Cereals, Other	6
1	Armenia	2007	Cheese	16
1	Armenia	2007	Cocoa Beans	5
1	Armenia	2007	Coconut Oil	1
1	Armenia	2007	Coffee	5
1	Armenia	2007	Dates	1
1	Armenia	2007	Eggs	25
1	Armenia	2007	Fats, Animals, Raw	8
1	Armenia	2007	Freshwater Fish	4
1	Armenia	2007	Fruits, Other	126
1	Armenia	2007	Grapefruit	1
1	Armenia	2007	Grapes	33
1	Armenia	2007	Honey	2
1	Armenia	2007	Lemons, Limes	1
1	Armenia	2007	Marine Fish, Other	1
1	Armenia	2007	Milk, Whole	347
1	Armenia	2007	Mutton & Goat Meat	7
1	Armenia	2007	Nuts	5
1	Armenia	2007	Offals, Edible	11
1	Armenia	2007	Oilcrops Oil, Other	4
1	Armenia	2007	Olives	3
1	Armenia	2007	Onions	58
1	Armenia	2007	Oranges, Mandarines	10
1	Armenia	2007	Palmkernel Oil	1
1	Armenia	2007	Pelagic Fish	2
1	Armenia	2007	Pigmeat	19
1	Armenia	2007	Pineapples	1
1	Armenia	2007	Potatoes	140
1	Armenia	2007	Poultry Meat	30
1	Armenia	2007	Rice (Milled Equivalent)	14
1	Armenia	2007	Soyabean Oil	2
1	Armenia	2007	Sugar, Refined Equivalent	78
1	Armenia	2007	Sunflowerseed Oil	12
1	Armenia	2007	Tomatoes	267
1	Armenia	2007	Vegetables, Other	577
1	Armenia	2007	Wheat	405
1	Armenia	2007	Wine	3
1	Armenia	2008	Apples	94
1	Armenia	2008	Bananas	8
1	Armenia	2008	Barley	8
1	Armenia	2008	Beer	16
1	Armenia	2008	Beverages, Alcoholic	5
1	Armenia	2008	Bovine Meat	63
1	Armenia	2008	Butter, Ghee	5
1	Armenia	2008	Cereals, Other	5
1	Armenia	2008	Cheese	16
1	Armenia	2008	Cocoa Beans	7
1	Armenia	2008	Coconut Oil	1
1	Armenia	2008	Coffee	7
1	Armenia	2008	Eggs	27
1	Armenia	2008	Fats, Animals, Raw	7
1	Armenia	2008	Freshwater Fish	4
1	Armenia	2008	Fruits, Other	116
1	Armenia	2008	Grapefruit	2
1	Armenia	2008	Grapes	72
1	Armenia	2008	Groundnuts (Shelled Eq)	1
1	Armenia	2008	Honey	1
1	Armenia	2008	Lemons, Limes	1
1	Armenia	2008	Marine Fish, Other	2
1	Armenia	2008	Milk, Whole	312
1	Armenia	2008	Mutton & Goat Meat	7
1	Armenia	2008	Nuts	4
1	Armenia	2008	Offals, Edible	12
1	Armenia	2008	Oilcrops Oil, Other	5
1	Armenia	2008	Olives	3
1	Armenia	2008	Onions	58
1	Armenia	2008	Oranges, Mandarines	13
1	Armenia	2008	Pelagic Fish	3
1	Armenia	2008	Pigmeat	23
1	Armenia	2008	Pineapples	1
1	Armenia	2008	Potatoes	138
1	Armenia	2008	Poultry Meat	40
1	Armenia	2008	Rice (Milled Equivalent)	12
1	Armenia	2008	Soyabean Oil	2
1	Armenia	2008	Sugar, Refined Equivalent	83
1	Armenia	2008	Sunflowerseed Oil	11
1	Armenia	2008	Tomatoes	261
1	Armenia	2008	Vegetables, Other	572
1	Armenia	2008	Wheat	386
1	Armenia	2008	Wine	3
1	Armenia	2009	Apples	91
1	Armenia	2009	Bananas	8
1	Armenia	2009	Barley	5
1	Armenia	2009	Beer	15
1	Armenia	2009	Beverages, Alcoholic	10
1	Armenia	2009	Bovine Meat	58
1	Armenia	2009	Butter, Ghee	3
1	Armenia	2009	Cereals, Other	4
1	Armenia	2009	Cheese	16
1	Armenia	2009	Citrus, Other	2
1	Armenia	2009	Cocoa Beans	5
1	Armenia	2009	Coconut Oil	1
1	Armenia	2009	Coffee	8
1	Armenia	2009	Eggs	30

FAO countrycode	Country	Reference year	Food item	Gross food supply (g *capita*$^{-1}$ d^{-1})
1	Armenia	2009	Fats, Animals, Raw	8
1	Armenia	2009	Freshwater Fish	5
1	Armenia	2009	Fruits, Other	119
1	Armenia	2009	Grapefruit	1
1	Armenia	2009	Grapes	98
1	Armenia	2009	Honey	2
1	Armenia	2009	Lemons, Limes	1
1	Armenia	2009	Marine Fish, Other	1
1	Armenia	2009	Milk, Whole	357
1	Armenia	2009	Mutton & Goat Meat	8
1	Armenia	2009	Nuts	4
1	Armenia	2009	Oats	1
1	Armenia	2009	Offals, Edible	12
1	Armenia	2009	Oilcrops Oil, Other	7
1	Armenia	2009	Olives	2
1	Armenia	2009	Onions	53
1	Armenia	2009	Oranges, Mandarines	10
1	Armenia	2009	Pelagic Fish	3
1	Armenia	2009	Pigmeat	14
1	Armenia	2009	Pineapples	1
1	Armenia	2009	Potatoes	157
1	Armenia	2009	Poultry Meat	31
1	Armenia	2009	Rice (Milled Equivalent)	12
1	Armenia	2009	Sugar, Refined Equivalent	83
1	Armenia	2009	Sunflowerseed Oil	11
1	Armenia	2009	Tomatoes	244
1	Armenia	2009	Vegetables, Other	621
1	Armenia	2009	Wheat	363
1	Armenia	2009	Wine	4
1	Armenia	2010	Apples	39
1	Armenia	2010	Bananas	8
1	Armenia	2010	Beer	17
1	Armenia	2010	Beverages, Alcoholic	9
1	Armenia	2010	Bovine Meat	49
1	Armenia	2010	Butter, Ghee	4
1	Armenia	2010	Cereals, Other	6
1	Armenia	2010	Cheese	16
1	Armenia	2010	Citrus, Other	3
1	Armenia	2010	Cocoa Beans	5
1	Armenia	2010	Coconut Oil	2
1	Armenia	2010	Coffee	11
1	Armenia	2010	Dates	1
1	Armenia	2010	Eggs	34
1	Armenia	2010	Fats, Animals, Raw	9
1	Armenia	2010	Freshwater Fish	4
1	Armenia	2010	Fruits, Other	62
1	Armenia	2010	Grapefruit	2
1	Armenia	2010	Grapes	49
1	Armenia	2010	Groundnuts (Shelled Eq)	1
1	Armenia	2010	Honey	2
1	Armenia	2010	Lemons, Limes	1
1	Armenia	2010	Milk, Whole	390
1	Armenia	2010	Mutton & Goat Meat	7
1	Armenia	2010	Nuts	4
1	Armenia	2010	Oats	1
1	Armenia	2010	Offals, Edible	12
1	Armenia	2010	Oilcrops Oil, Other	7
1	Armenia	2010	Olives	2
1	Armenia	2010	Onions	43
1	Armenia	2010	Oranges, Mandarines	15
1	Armenia	2010	Pelagic Fish	2
1	Armenia	2010	Pigmeat	17
1	Armenia	2010	Pineapples	1
1	Armenia	2010	Potatoes	133
1	Armenia	2010	Poultry Meat	38
1	Armenia	2010	Rice (Milled Equivalent)	10
1	Armenia	2010	Sesameseed	1
1	Armenia	2010	Soyabean Oil	1
1	Armenia	2010	Sugar, Refined Equivalent	101
1	Armenia	2010	Sunflowerseed Oil	10
1	Armenia	2010	Sweeteners, Other	1
1	Armenia	2010	Tomatoes	217
1	Armenia	2010	Vegetables, Other	483
1	Armenia	2010	Wheat	366
1	Armenia	2010	Wine	4
1	Armenia	2011	Apples	55
1	Armenia	2011	Bananas	10
1	Armenia	2011	Beer	15
1	Armenia	2011	Beverages, Alcoholic	4
1	Armenia	2011	Bovine Meat	51
1	Armenia	2011	Butter, Ghee	5
1	Armenia	2011	Cereals, Other	23
1	Armenia	2011	Cheese	17
1	Armenia	2011	Citrus, Other	2
1	Armenia	2011	Cocoa Beans	6
1	Armenia	2011	Coconut Oil	1
1	Armenia	2011	Coconuts - Incl Copra	1
1	Armenia	2011	Coffee	11
1	Armenia	2011	Dates	1
1	Armenia	2011	Eggs	31
1	Armenia	2011	Fats, Animals, Raw	8
1	Armenia	2011	Freshwater Fish	5
1	Armenia	2011	Fruits, Other	96
1	Armenia	2011	Grapefruit	2
1	Armenia	2011	Grapes	37
1	Armenia	2011	Groundnuts (Shelled Eq)	1
1	Armenia	2011	Honey	2

FAO country code	Country	Reference year	Food item	Gross food supply (g *capita*$^{-1}$ d^{-1})
1	Armenia	2011	Lemons, Limes	1
1	Armenia	2011	Milk, Whole	386
1	Armenia	2011	Mutton & Goat Meat	8
1	Armenia	2011	Nuts	4
1	Armenia	2011	Oats	1
1	Armenia	2011	Offals, Edible	12
1	Armenia	2011	Oilcrops Oil, Other	5
1	Armenia	2011	Olives	3
1	Armenia	2011	Onions	47
1	Armenia	2011	Oranges, Mandarines	14
1	Armenia	2011	Pelagic Fish	3
1	Armenia	2011	Pigmeat	18
1	Armenia	2011	Pineapples	1
1	Armenia	2011	Potatoes	121
1	Armenia	2011	Poultry Meat	40
1	Armenia	2011	Rice (Milled Equivalent)	8
1	Armenia	2011	Soyabean Oil	1
1	Armenia	2011	Sugar, Refined Equivalent	91
1	Armenia	2011	Sunflowerseed Oil	14
1	Armenia	2011	Sweeteners, Other	3
1	Armenia	2011	Tomatoes	234
1	Armenia	2011	Vegetables, Other	573
1	Armenia	2011	Wheat	364
1	Armenia	2011	Wine	5
10	Australia	1992	Apples	43
10	Australia	1992	Bananas	26
10	Australia	1992	Beer	282
10	Australia	1992	Beverages, Alcoholic	4
10	Australia	1992	Beverages, Fermented	1
10	Australia	1992	Bovine Meat	119
10	Australia	1992	Butter, Ghee	7
10	Australia	1992	Cephalopods	1
10	Australia	1992	Cheese	24
10	Australia	1992	Cocoa Beans	5
10	Australia	1992	Coconuts - Incl Copra	8
10	Australia	1992	Coffee	8
10	Australia	1992	Cottonseed Oil	8
10	Australia	1992	Crustaceans	7
10	Australia	1992	Dates	1
10	Australia	1992	Demersal Fish	20
10	Australia	1992	Eggs	18
10	Australia	1992	Fats, Animals, Raw	12
10	Australia	1992	Freshwater Fish	4
10	Australia	1992	Fruits, Other	46
10	Australia	1992	Grapefruit	4
10	Australia	1992	Grapes	21
10	Australia	1992	Groundnuts (Shelled Eq)	6
10	Australia	1992	Honey	2
10	Australia	1992	Lemons, Limes	5
10	Australia	1992	Maize	13
10	Australia	1992	Marine Fish, Other	9
10	Australia	1992	Meat, Other	2
10	Australia	1992	Milk, Whole	359
10	Australia	1992	Molluscs, Other	6
10	Australia	1992	Mutton & Goat Meat	57
10	Australia	1992	Nuts	9
10	Australia	1992	Oats	7
10	Australia	1992	Offals, Edible	29
10	Australia	1992	Olive Oil	2
10	Australia	1992	Olives	1
10	Australia	1992	Onions	21
10	Australia	1992	Oranges, Mandarines	72
10	Australia	1992	Palm Oil	13
10	Australia	1992	Peas	4
10	Australia	1992	Pelagic Fish	9
10	Australia	1992	Pigmeat	52
10	Australia	1992	Pineapples	24
10	Australia	1992	Potatoes	155
10	Australia	1992	Poultry Meat	69
10	Australia	1992	Pulses, Other	2
10	Australia	1992	Rape and Mustard Oil	10
10	Australia	1992	Rice (Milled Equivalent)	15
10	Australia	1992	Rye	4
10	Australia	1992	Soyabean Oil	6
10	Australia	1992	Sugar, Refined Equivalent	118
10	Australia	1992	Sunflowerseed Oil	8
10	Australia	1992	Sweet Potatoes	1
10	Australia	1992	Sweeteners, Other	9
10	Australia	1992	Tea	3
10	Australia	1992	Tomatoes	56
10	Australia	1992	Vegetables, Other	150
10	Australia	1992	Wheat	186
10	Australia	1992	Wine	51
10	Australia	1993	Apples	46
10	Australia	1993	Bananas	31
10	Australia	1993	Beer	272
10	Australia	1993	Beverages, Alcoholic	4
10	Australia	1993	Beverages, Fermented	1
10	Australia	1993	Bovine Meat	115
10	Australia	1993	Butter, Ghee	7
10	Australia	1993	Cephalopods	1
10	Australia	1993	Cheese	23
10	Australia	1993	Cocoa Beans	5
10	Australia	1993	Coconut Oil	2
10	Australia	1993	Coconuts - Incl Copra	8
10	Australia	1993	Coffee	8
10	Australia	1993	Cottonseed Oil	6

FAO country code	Country	Reference year	Food item	Gross food supply (g *capita*$^{-1}$ d^{-1})
10	Australia	1993	Crustaceans	8
10	Australia	1993	Dates	1
10	Australia	1993	Demersal Fish	20
10	Australia	1993	Eggs	18
10	Australia	1993	Fats, Animals, Raw	11
10	Australia	1993	Freshwater Fish	3
10	Australia	1993	Fruits, Other	45
10	Australia	1993	Grapefruit	3
10	Australia	1993	Grapes	15
10	Australia	1993	Groundnuts (Shelled Eq)	5
10	Australia	1993	Honey	2
10	Australia	1993	Lemons, Limes	5
10	Australia	1993	Maize	12
10	Australia	1993	Marine Fish, Other	8
10	Australia	1993	Meat, Other	2
10	Australia	1993	Milk, Whole	357
10	Australia	1993	Molluscs, Other	4
10	Australia	1993	Mutton & Goat Meat	57
10	Australia	1993	Nuts	9
10	Australia	1993	Oats	5
10	Australia	1993	Offals, Edible	28
10	Australia	1993	Olive Oil	3
10	Australia	1993	Olives	1
10	Australia	1993	Onions	19
10	Australia	1993	Oranges, Mandarines	90
10	Australia	1993	Palm Oil	12
10	Australia	1993	Peas	1
10	Australia	1993	Pelagic Fish	10
10	Australia	1993	Pigmeat	50
10	Australia	1993	Pineapples	24
10	Australia	1993	Potatoes	150
10	Australia	1993	Poultry Meat	71
10	Australia	1993	Pulses, Other	2
10	Australia	1993	Rape and Mustard Oil	13
10	Australia	1993	Rice (Milled Equivalent)	17
10	Australia	1993	Rye	3
10	Australia	1993	Soyabean Oil	6
10	Australia	1993	Sugar, Refined Equivalent	103
10	Australia	1993	Sunflowerseed Oil	7
10	Australia	1993	Sweet Potatoes	1
10	Australia	1993	Sweeteners, Other	12
10	Australia	1993	Tea	3
10	Australia	1993	Tomatoes	49
10	Australia	1993	Vegetables, Other	149
10	Australia	1993	Wheat	191
10	Australia	1993	Wine	50
10	Australia	1994	Apples	40
10	Australia	1994	Bananas	30
10	Australia	1994	Beer	264
10	Australia	1994	Beverages, Alcoholic	4
10	Australia	1994	Beverages, Fermented	1
10	Australia	1994	Bovine Meat	115
10	Australia	1994	Butter, Ghee	8
10	Australia	1994	Cephalopods	2
10	Australia	1994	Cheese	24
10	Australia	1994	Cocoa Beans	5
10	Australia	1994	Coconut Oil	1
10	Australia	1994	Coconuts - Incl Copra	8
10	Australia	1994	Coffee	9
10	Australia	1994	Cottonseed Oil	5
10	Australia	1994	Crustaceans	6
10	Australia	1994	Dates	1
10	Australia	1994	Demersal Fish	23
10	Australia	1994	Eggs	18
10	Australia	1994	Fats, Animals, Raw	10
10	Australia	1994	Freshwater Fish	2
10	Australia	1994	Fruits, Other	43
10	Australia	1994	Grapefruit	3
10	Australia	1994	Grapes	17
10	Australia	1994	Groundnuts (Shelled Eq)	3
10	Australia	1994	Honey	2
10	Australia	1994	Lemons, Limes	5
10	Australia	1994	Maize	12
10	Australia	1994	Marine Fish, Other	5
10	Australia	1994	Meat, Other	2
10	Australia	1994	Milk, Whole	363
10	Australia	1994	Molluscs, Other	6
10	Australia	1994	Mutton & Goat Meat	54
10	Australia	1994	Nuts	9
10	Australia	1994	Oats	8
10	Australia	1994	Offals, Edible	28
10	Australia	1994	Olive Oil	2
10	Australia	1994	Olives	1
10	Australia	1994	Onions	21
10	Australia	1994	Oranges, Mandarines	83
10	Australia	1994	Palm Oil	13
10	Australia	1994	Peas	2
10	Australia	1994	Pelagic Fish	10
10	Australia	1994	Pigmeat	52
10	Australia	1994	Pineapples	25
10	Australia	1994	Potatoes	155
10	Australia	1994	Poultry Meat	73
10	Australia	1994	Pulses, Other	2
10	Australia	1994	Rape and Mustard Oil	10
10	Australia	1994	Rice (Milled Equivalent)	18
10	Australia	1994	Rye	3
10	Australia	1994	Soyabean Oil	6

FAO countrycode	Country	Reference year	Food item	Gross food supply (g *capita*$^{-1}$ d^{-1})
10	Australia	1994	Sugar, Refined Equivalent	100
10	Australia	1994	Sunflowerseed Oil	6
10	Australia	1994	Sweet Potatoes	1
10	Australia	1994	Sweeteners, Other	12
10	Australia	1994	Tea	3
10	Australia	1994	Tomatoes	65
10	Australia	1994	Vegetables, Other	155
10	Australia	1994	Wheat	202
10	Australia	1994	Wine	50
10	Australia	1995	Apples	42
10	Australia	1995	Bananas	30
10	Australia	1995	Beans	1
10	Australia	1995	Beer	258
10	Australia	1995	Beverages, Alcoholic	4
10	Australia	1995	Beverages, Fermented	1
10	Australia	1995	Bovine Meat	112
10	Australia	1995	Butter, Ghee	9
10	Australia	1995	Cephalopods	2
10	Australia	1995	Cheese	22
10	Australia	1995	Cocoa Beans	6
10	Australia	1995	Coconut Oil	2
10	Australia	1995	Coconuts - Incl Copra	6
10	Australia	1995	Coffee	13
10	Australia	1995	Cottonseed Oil	7
10	Australia	1995	Crustaceans	5
10	Australia	1995	Demersal Fish	23
10	Australia	1995	Eggs	17
10	Australia	1995	Fats, Animals, Raw	9
10	Australia	1995	Freshwater Fish	3
10	Australia	1995	Fruits, Other	41
10	Australia	1995	Grapefruit	2
10	Australia	1995	Grapes	19
10	Australia	1995	Groundnuts (Shelled Eq)	4
10	Australia	1995	Honey	1
10	Australia	1995	Lemons, Limes	5
10	Australia	1995	Maize	12
10	Australia	1995	Marine Fish, Other	5
10	Australia	1995	Meat, Other	2
10	Australia	1995	Milk, Whole	377
10	Australia	1995	Molluscs, Other	4
10	Australia	1995	Mutton & Goat Meat	50
10	Australia	1995	Nuts	10
10	Australia	1995	Oats	6
10	Australia	1995	Offals, Edible	28
10	Australia	1995	Olive Oil	3
10	Australia	1995	Olives	1
10	Australia	1995	Onions	18
10	Australia	1995	Oranges, Mandarines	71
10	Australia	1995	Palm Oil	14
10	Australia	1995	Peas	2
10	Australia	1995	Pelagic Fish	10
10	Australia	1995	Pigmeat	53
10	Australia	1995	Pineapples	22
10	Australia	1995	Potatoes	145
10	Australia	1995	Poultry Meat	72
10	Australia	1995	Pulses, Other	3
10	Australia	1995	Rape and Mustard Oil	11
10	Australia	1995	Rice (Milled Equivalent)	19
10	Australia	1995	Rye	3
10	Australia	1995	Soyabean Oil	5
10	Australia	1995	Sugar, Refined Equivalent	110
10	Australia	1995	Sunflowerseed Oil	4
10	Australia	1995	Sweet Potatoes	1
10	Australia	1995	Sweeteners, Other	11
10	Australia	1995	Tea	2
10	Australia	1995	Tomatoes	60
10	Australia	1995	Vegetables, Other	158
10	Australia	1995	Wheat	214
10	Australia	1995	Wine	50
10	Australia	1996	Apples	38
10	Australia	1996	Bananas	30
10	Australia	1996	Beans	5
10	Australia	1996	Beer	253
10	Australia	1996	Beverages, Alcoholic	5
10	Australia	1996	Bovine Meat	115
10	Australia	1996	Butter, Ghee	8
10	Australia	1996	Cephalopods	2
10	Australia	1996	Cheese	24
10	Australia	1996	Cocoa Beans	6
10	Australia	1996	Coconut Oil	2
10	Australia	1996	Coconuts - Incl Copra	7
10	Australia	1996	Coffee	12
10	Australia	1996	Cottonseed Oil	7
10	Australia	1996	Crustaceans	7
10	Australia	1996	Dates	1
10	Australia	1996	Demersal Fish	23
10	Australia	1996	Eggs	16
10	Australia	1996	Fats, Animals, Raw	9
10	Australia	1996	Freshwater Fish	3
10	Australia	1996	Fruits, Other	44
10	Australia	1996	Grapefruit	3
10	Australia	1996	Grapes	25
10	Australia	1996	Groundnuts (Shelled Eq)	4
10	Australia	1996	Honey	2
10	Australia	1996	Lemons, Limes	4
10	Australia	1996	Maize	12
10	Australia	1996	Marine Fish, Other	7

FAO country code	Country	Reference year	Food item	Gross food supply (g *capita*$^{-1}$ d^{-1})
10	Australia	1996	Meat, Other	2
10	Australia	1996	Milk, Whole	371
10	Australia	1996	Molluscs, Other	6
10	Australia	1996	Mutton & Goat Meat	45
10	Australia	1996	Nuts	10
10	Australia	1996	Oats	4
10	Australia	1996	Offals, Edible	26
10	Australia	1996	Olive Oil	2
10	Australia	1996	Olives	1
10	Australia	1996	Onions	25
10	Australia	1996	Oranges, Mandarines	59
10	Australia	1996	Palm Oil	14
10	Australia	1996	Peas	2
10	Australia	1996	Pelagic Fish	8
10	Australia	1996	Pigmeat	50
10	Australia	1996	Pineapples	20
10	Australia	1996	Potatoes	166
10	Australia	1996	Poultry Meat	73
10	Australia	1996	Pulses, Other	2
10	Australia	1996	Rape and Mustard Oil	9
10	Australia	1996	Rice (Milled Equivalent)	22
10	Australia	1996	Rye	3
10	Australia	1996	Soyabean Oil	5
10	Australia	1996	Sugar, Refined Equivalent	106
10	Australia	1996	Sunflowerseed Oil	5
10	Australia	1996	Sweet Potatoes	1
10	Australia	1996	Sweeteners, Other	7
10	Australia	1996	Tea	2
10	Australia	1996	Tomatoes	60
10	Australia	1996	Vegetables, Other	166
10	Australia	1996	Wheat	207
10	Australia	1996	Wine	49
10	Australia	1997	Apples	46
10	Australia	1997	Bananas	28
10	Australia	1997	Beans	2
10	Australia	1997	Beer	254
10	Australia	1997	Beverages, Alcoholic	3
10	Australia	1997	Beverages, Fermented	1
10	Australia	1997	Bovine Meat	101
10	Australia	1997	Butter, Ghee	8
10	Australia	1997	Cephalopods	2
10	Australia	1997	Cheese	24
10	Australia	1997	Cocoa Beans	6
10	Australia	1997	Coconut Oil	1
10	Australia	1997	Coconuts - Incl Copra	7
10	Australia	1997	Coffee	9
10	Australia	1997	Cottonseed Oil	7
10	Australia	1997	Crustaceans	9
10	Australia	1997	Dates	1
10	Australia	1997	Demersal Fish	22
10	Australia	1997	Eggs	17
10	Australia	1997	Fats, Animals, Raw	9
10	Australia	1997	Freshwater Fish	3
10	Australia	1997	Fruits, Other	49
10	Australia	1997	Grapefruit	2
10	Australia	1997	Grapes	27
10	Australia	1997	Groundnuts (Shelled Eq)	4
10	Australia	1997	Honey	2
10	Australia	1997	Lemons, Limes	5
10	Australia	1997	Maize	13
10	Australia	1997	Marine Fish, Other	7
10	Australia	1997	Meat, Other	2
10	Australia	1997	Milk, Whole	343
10	Australia	1997	Molluscs, Other	6
10	Australia	1997	Mutton & Goat Meat	46
10	Australia	1997	Nuts	11
10	Australia	1997	Oats	3
10	Australia	1997	Offals, Edible	26
10	Australia	1997	Olive Oil	3
10	Australia	1997	Olives	1
10	Australia	1997	Onions	23
10	Australia	1997	Oranges, Mandarines	70
10	Australia	1997	Palm Oil	12
10	Australia	1997	Peas	1
10	Australia	1997	Pelagic Fish	7
10	Australia	1997	Pigmeat	52
10	Australia	1997	Pineapples	20
10	Australia	1997	Potatoes	160
10	Australia	1997	Poultry Meat	85
10	Australia	1997	Pulses, Other	2
10	Australia	1997	Rape and Mustard Oil	16
10	Australia	1997	Rice (Milled Equivalent)	22
10	Australia	1997	Rye	3
10	Australia	1997	Soyabean Oil	4
10	Australia	1997	Sugar, Refined Equivalent	112
10	Australia	1997	Sunflowerseed Oil	6
10	Australia	1997	Sweet Potatoes	1
10	Australia	1997	Sweeteners, Other	11
10	Australia	1997	Tea	2
10	Australia	1997	Tomatoes	63
10	Australia	1997	Vegetables, Other	160
10	Australia	1997	Wheat	204
10	Australia	1997	Wine	51
10	Australia	1998	Apples	40
10	Australia	1998	Bananas	31
10	Australia	1998	Beans	2
10	Australia	1998	Beer	249

FAO country code	Country	Reference year	Food item	Gross food supply (g *capita*$^{-1}$ d^{-1})
10	Australia	1998	Beverages, Alcoholic	4
10	Australia	1998	Beverages, Fermented	1
10	Australia	1998	Bovine Meat	107
10	Australia	1998	Butter, Ghee	8
10	Australia	1998	Cephalopods	2
10	Australia	1998	Cheese	22
10	Australia	1998	Cocoa Beans	6
10	Australia	1998	Coconut Oil	1
10	Australia	1998	Coconuts - Incl Copra	6
10	Australia	1998	Coffee	9
10	Australia	1998	Cottonseed Oil	6
10	Australia	1998	Crustaceans	9
10	Australia	1998	Dates	1
10	Australia	1998	Demersal Fish	23
10	Australia	1998	Eggs	17
10	Australia	1998	Fats, Animals, Raw	9
10	Australia	1998	Freshwater Fish	3
10	Australia	1998	Fruits, Other	45
10	Australia	1998	Grapefruit	3
10	Australia	1998	Grapes	28
10	Australia	1998	Groundnuts (Shelled Eq)	3
10	Australia	1998	Honey	2
10	Australia	1998	Lemons, Limes	4
10	Australia	1998	Maize	13
10	Australia	1998	Marine Fish, Other	7
10	Australia	1998	Meat, Other	2
10	Australia	1998	Milk, Whole	298
10	Australia	1998	Molluscs, Other	6
10	Australia	1998	Mutton & Goat Meat	46
10	Australia	1998	Nuts	11
10	Australia	1998	Oats	4
10	Australia	1998	Offals, Edible	28
10	Australia	1998	Olive Oil	2
10	Australia	1998	Olives	1
10	Australia	1998	Onions	23
10	Australia	1998	Oranges, Mandarines	66
10	Australia	1998	Palm Oil	14
10	Australia	1998	Peas	1
10	Australia	1998	Pelagic Fish	5
10	Australia	1998	Pigmeat	53
10	Australia	1998	Pineapples	19
10	Australia	1998	Potatoes	169
10	Australia	1998	Poultry Meat	89
10	Australia	1998	Pulses, Other	2
10	Australia	1998	Rape and Mustard Oil	15
10	Australia	1998	Rice (Milled Equivalent)	21
10	Australia	1998	Rye	3
10	Australia	1998	Soyabean Oil	2
10	Australia	1998	Sugar, Refined Equivalent	109
10	Australia	1998	Sunflowerseed Oil	6
10	Australia	1998	Sweet Potatoes	2
10	Australia	1998	Sweeteners, Other	11
10	Australia	1998	Tea	2
10	Australia	1998	Tomatoes	61
10	Australia	1998	Vegetables, Other	164
10	Australia	1998	Wheat	188
10	Australia	1998	Wine	53
10	Australia	1999	Apples	45
10	Australia	1999	Bananas	29
10	Australia	1999	Beans	1
10	Australia	1999	Beer	250
10	Australia	1999	Beverages, Alcoholic	3
10	Australia	1999	Beverages, Fermented	1
10	Australia	1999	Bovine Meat	116
10	Australia	1999	Butter, Ghee	8
10	Australia	1999	Cephalopods	2
10	Australia	1999	Cheese	24
10	Australia	1999	Cocoa Beans	4
10	Australia	1999	Coconut Oil	1
10	Australia	1999	Coconuts - Incl Copra	7
10	Australia	1999	Coffee	11
10	Australia	1999	Cottonseed Oil	5
10	Australia	1999	Crustaceans	9
10	Australia	1999	Dates	1
10	Australia	1999	Demersal Fish	23
10	Australia	1999	Eggs	18
10	Australia	1999	Fats, Animals, Raw	9
10	Australia	1999	Freshwater Fish	3
10	Australia	1999	Fruits, Other	49
10	Australia	1999	Grapefruit	2
10	Australia	1999	Grapes	28
10	Australia	1999	Groundnuts (Shelled Eq)	4
10	Australia	1999	Honey	1
10	Australia	1999	Lemons, Limes	4
10	Australia	1999	Maize	12
10	Australia	1999	Marine Fish, Other	7
10	Australia	1999	Meat, Other	2
10	Australia	1999	Milk, Whole	300
10	Australia	1999	Molluscs, Other	7
10	Australia	1999	Mutton & Goat Meat	43
10	Australia	1999	Nuts	12
10	Australia	1999	Oats	4
10	Australia	1999	Offals, Edible	28
10	Australia	1999	Olive Oil	3
10	Australia	1999	Olives	2
10	Australia	1999	Onions	22
10	Australia	1999	Oranges, Mandarines	61

FAO country code	Country	Reference year	Food item	Gross food supply (g $capita^{-1}$ d^{-1})
10	Australia	1999	Palm Oil	15
10	Australia	1999	Peas	1
10	Australia	1999	Pelagic Fish	6
10	Australia	1999	Pigmeat	52
10	Australia	1999	Pineapples	21
10	Australia	1999	Potatoes	161
10	Australia	1999	Poultry Meat	84
10	Australia	1999	Pulses, Other	2
10	Australia	1999	Rape and Mustard Oil	17
10	Australia	1999	Rice (Milled Equivalent)	22
10	Australia	1999	Rye	3
10	Australia	1999	Sesameseed Oil	1
10	Australia	1999	Soyabean Oil	3
10	Australia	1999	Sugar, Refined Equivalent	98
10	Australia	1999	Sunflowerseed Oil	7
10	Australia	1999	Sweet Potatoes	3
10	Australia	1999	Sweeteners, Other	13
10	Australia	1999	Tea	2
10	Australia	1999	Tomatoes	61
10	Australia	1999	Vegetables, Other	161
10	Australia	1999	Wheat	188
10	Australia	1999	Wine	54
10	Australia	2000	Apples	40
10	Australia	2000	Bananas	33
10	Australia	2000	Beer	246
10	Australia	2000	Beverages, Alcoholic	6
10	Australia	2000	Beverages, Fermented	1
10	Australia	2000	Bovine Meat	107
10	Australia	2000	Butter, Ghee	8
10	Australia	2000	Cephalopods	2
10	Australia	2000	Cheese	25
10	Australia	2000	Cocoa Beans	4
10	Australia	2000	Coconut Oil	1
10	Australia	2000	Coconuts - Incl Copra	6
10	Australia	2000	Coffee	8
10	Australia	2000	Cottonseed Oil	4
10	Australia	2000	Crustaceans	8
10	Australia	2000	Dates	1
10	Australia	2000	Demersal Fish	25
10	Australia	2000	Eggs	16
10	Australia	2000	Fats, Animals, Raw	10
10	Australia	2000	Freshwater Fish	4
10	Australia	2000	Fruits, Other	51
10	Australia	2000	Grapefruit	2
10	Australia	2000	Grapes	30
10	Australia	2000	Groundnuts (Shelled Eq)	4
10	Australia	2000	Honey	2
10	Australia	2000	Lemons, Limes	5
10	Australia	2000	Maize	12
10	Australia	2000	Marine Fish, Other	5
10	Australia	2000	Meat, Other	3
10	Australia	2000	Milk, Whole	289
10	Australia	2000	Molluscs, Other	7
10	Australia	2000	Mutton & Goat Meat	48
10	Australia	2000	Nuts	12
10	Australia	2000	Oats	4
10	Australia	2000	Offals, Edible	28
10	Australia	2000	Olive Oil	4
10	Australia	2000	Olives	2
10	Australia	2000	Onions	28
10	Australia	2000	Oranges, Mandarines	65
10	Australia	2000	Palm Oil	16
10	Australia	2000	Peas	1
10	Australia	2000	Pelagic Fish	7
10	Australia	2000	Pigmeat	52
10	Australia	2000	Pineapples	22
10	Australia	2000	Potatoes	144
10	Australia	2000	Poultry Meat	89
10	Australia	2000	Pulses, Other	2
10	Australia	2000	Rape and Mustard Oil	16
10	Australia	2000	Rice (Milled Equivalent)	24
10	Australia	2000	Rye	1
10	Australia	2000	Sesameseed Oil	1
10	Australia	2000	Soyabean Oil	2
10	Australia	2000	Sugar, Refined Equivalent	97
10	Australia	2000	Sunflowerseed Oil	8
10	Australia	2000	Sweet Potatoes	3
10	Australia	2000	Sweeteners, Other	16
10	Australia	2000	Tea	2
10	Australia	2000	Tomatoes	65
10	Australia	2000	Vegetables, Other	175
10	Australia	2000	Wheat	182
10	Australia	2000	Wine	56
10	Australia	2001	Apples	42
10	Australia	2001	Bananas	46
10	Australia	2001	Beans	1
10	Australia	2001	Beer	247
10	Australia	2001	Beverages, Alcoholic	5
10	Australia	2001	Beverages, Fermented	3
10	Australia	2001	Bovine Meat	116
10	Australia	2001	Butter, Ghee	9
10	Australia	2001	Cephalopods	2
10	Australia	2001	Cheese	30
10	Australia	2001	Cocoa Beans	4
10	Australia	2001	Coconut Oil	2
10	Australia	2001	Coconuts - Incl Copra	7
10	Australia	2001	Coffee	6

FAO country code	Country	Reference year	Food item	Gross food supply (g $capita^{-1}$ d^{-1})
10	Australia	2001	Cottonseed Oil	8
10	Australia	2001	Crustaceans	9
10	Australia	2001	Dates	1
10	Australia	2001	Demersal Fish	24
10	Australia	2001	Eggs	16
10	Australia	2001	Fats, Animals, Raw	9
10	Australia	2001	Freshwater Fish	4
10	Australia	2001	Fruits, Other	56
10	Australia	2001	Grapefruit	2
10	Australia	2001	Grapes	26
10	Australia	2001	Groundnuts (Shelled Eq)	3
10	Australia	2001	Honey	2
10	Australia	2001	Lemons, Limes	5
10	Australia	2001	Maize	11
10	Australia	2001	Marine Fish, Other	5
10	Australia	2001	Meat, Other	2
10	Australia	2001	Milk, Whole	250
10	Australia	2001	Molluscs, Other	7
10	Australia	2001	Mutton & Goat Meat	41
10	Australia	2001	Nuts	14
10	Australia	2001	Oats	4
10	Australia	2001	Offals, Edible	29
10	Australia	2001	Olive Oil	4
10	Australia	2001	Olives	2
10	Australia	2001	Onions	24
10	Australia	2001	Oranges, Mandarines	69
10	Australia	2001	Palm Oil	19
10	Australia	2001	Peas	1
10	Australia	2001	Pelagic Fish	7
10	Australia	2001	Pigmeat	48
10	Australia	2001	Pineapples	19
10	Australia	2001	Potatoes	155
10	Australia	2001	Poultry Meat	87
10	Australia	2001	Pulses, Other	2
10	Australia	2001	Rape and Mustard Oil	18
10	Australia	2001	Rice (Milled Equivalent)	26
10	Australia	2001	Rye	3
10	Australia	2001	Sesameseed Oil	1
10	Australia	2001	Soyabean Oil	1
10	Australia	2001	Sugar, Refined Equivalent	97
10	Australia	2001	Sunflowerseed Oil	7
10	Australia	2001	Sweet Potatoes	4
10	Australia	2001	Sweeteners, Other	18
10	Australia	2001	Tea	2
10	Australia	2001	Tomatoes	85
10	Australia	2001	Vegetables, Other	178
10	Australia	2001	Wheat	185
10	Australia	2001	Wine	56
10	Australia	2002	Apples	57
10	Australia	2002	Bananas	40
10	Australia	2002	Beer	239
10	Australia	2002	Beverages, Alcoholic	5
10	Australia	2002	Beverages, Fermented	1
10	Australia	2002	Bovine Meat	106
10	Australia	2002	Butter, Ghee	9
10	Australia	2002	Cephalopods	2
10	Australia	2002	Cheese	35
10	Australia	2002	Cocoa Beans	5
10	Australia	2002	Coconut Oil	1
10	Australia	2002	Coconuts - Incl Copra	6
10	Australia	2002	Coffee	9
10	Australia	2002	Cottonseed Oil	9
10	Australia	2002	Crustaceans	9
10	Australia	2002	Dates	1
10	Australia	2002	Demersal Fish	25
10	Australia	2002	Eggs	15
10	Australia	2002	Fats, Animals, Raw	9
10	Australia	2002	Freshwater Fish	4
10	Australia	2002	Fruits, Other	51
10	Australia	2002	Grapefruit	2
10	Australia	2002	Grapes	32
10	Australia	2002	Groundnuts (Shelled Eq)	5
10	Australia	2002	Honey	2
10	Australia	2002	Lemons, Limes	5
10	Australia	2002	Maize	14
10	Australia	2002	Marine Fish, Other	5
10	Australia	2002	Meat, Other	2
10	Australia	2002	Milk, Whole	243
10	Australia	2002	Molluscs, Other	8
10	Australia	2002	Mutton & Goat Meat	40
10	Australia	2002	Nuts	16
10	Australia	2002	Oats	4
10	Australia	2002	Offals, Edible	28
10	Australia	2002	Olive Oil	4
10	Australia	2002	Olives	2
10	Australia	2002	Onions	30
10	Australia	2002	Oranges, Mandarines	55
10	Australia	2002	Palm Oil	13
10	Australia	2002	Peas	1
10	Australia	2002	Pelagic Fish	8
10	Australia	2002	Pigmeat	54
10	Australia	2002	Pineapples	19
10	Australia	2002	Potatoes	158
10	Australia	2002	Poultry Meat	92
10	Australia	2002	Pulses, Other	2
10	Australia	2002	Rape and Mustard Oil	15
10	Australia	2002	Rice (Milled Equivalent)	27

FAO country code	Country	Reference year	Food item	Gross food supply (g *capita*$^{-1}$ d^{-1})
10	Australia	2002	Rye	4
10	Australia	2002	Soyabean Oil	2
10	Australia	2002	Sugar, Refined Equivalent	97
10	Australia	2002	Sunflowerseed Oil	5
10	Australia	2002	Sweet Potatoes	4
10	Australia	2002	Sweeteners, Other	18
10	Australia	2002	Tea	2
10	Australia	2002	Tomatoes	67
10	Australia	2002	Vegetables, Other	162
10	Australia	2002	Wheat	198
10	Australia	2002	Wine	56
10	Australia	2003	Apples	59
10	Australia	2003	Bananas	35
10	Australia	2003	Beer	236
10	Australia	2003	Beverages, Alcoholic	5
10	Australia	2003	Beverages, Fermented	1
10	Australia	2003	Bovine Meat	125
10	Australia	2003	Butter, Ghee	9
10	Australia	2003	Cephalopods	2
10	Australia	2003	Cheese	30
10	Australia	2003	Cocoa Beans	4
10	Australia	2003	Coconut Oil	1
10	Australia	2003	Coconuts - Incl Copra	6
10	Australia	2003	Coffee	9
10	Australia	2003	Cottonseed Oil	8
10	Australia	2003	Crustaceans	9
10	Australia	2003	Dates	1
10	Australia	2003	Demersal Fish	26
10	Australia	2003	Eggs	14
10	Australia	2003	Fats, Animals, Raw	9
10	Australia	2003	Freshwater Fish	5
10	Australia	2003	Fruits, Other	55
10	Australia	2003	Grapefruit	2
10	Australia	2003	Grapes	23
10	Australia	2003	Groundnuts (Shelled Eq)	6
10	Australia	2003	Honey	3
10	Australia	2003	Lemons, Limes	5
10	Australia	2003	Maize	14
10	Australia	2003	Marine Fish, Other	6
10	Australia	2003	Meat, Other	3
10	Australia	2003	Milk, Whole	241
10	Australia	2003	Molluscs, Other	9
10	Australia	2003	Mutton & Goat Meat	37
10	Australia	2003	Nuts	18
10	Australia	2003	Oats	12
10	Australia	2003	Offals, Edible	28
10	Australia	2003	Oilcrops Oil, Other	1
10	Australia	2003	Olive Oil	4
10	Australia	2003	Olives	2
10	Australia	2003	Onions	23
10	Australia	2003	Oranges, Mandarines	83
10	Australia	2003	Palm Oil	12
10	Australia	2003	Peas	1
10	Australia	2003	Pelagic Fish	10
10	Australia	2003	Pigmeat	57
10	Australia	2003	Pineapples	19
10	Australia	2003	Potatoes	144
10	Australia	2003	Poultry Meat	95
10	Australia	2003	Pulses, Other	2
10	Australia	2003	Rape and Mustard Oil	16
10	Australia	2003	Rice (Milled Equivalent)	27
10	Australia	2003	Rye	3
10	Australia	2003	Soyabean Oil	2
10	Australia	2003	Sugar, Refined Equivalent	100
10	Australia	2003	Sunflowerseed Oil	6
10	Australia	2003	Sweet Potatoes	5
10	Australia	2003	Sweeteners, Other	17
10	Australia	2003	Tea	2
10	Australia	2003	Tomatoes	60
10	Australia	2003	Vegetables, Other	166
10	Australia	2003	Wheat	196
10	Australia	2003	Wine	58
10	Australia	2004	Apples	71
10	Australia	2004	Bananas	35
10	Australia	2004	Beer	237
10	Australia	2004	Beverages, Alcoholic	6
10	Australia	2004	Beverages, Fermented	1
10	Australia	2004	Bovine Meat	101
10	Australia	2004	Butter, Ghee	10
10	Australia	2004	Cephalopods	2
10	Australia	2004	Cheese	27
10	Australia	2004	Cocoa Beans	5
10	Australia	2004	Coconut Oil	1
10	Australia	2004	Coconuts - Incl Copra	6
10	Australia	2004	Coffee	10
10	Australia	2004	Cottonseed Oil	12
10	Australia	2004	Crustaceans	11
10	Australia	2004	Dates	1
10	Australia	2004	Demersal Fish	25
10	Australia	2004	Eggs	14
10	Australia	2004	Fats, Animals, Raw	11
10	Australia	2004	Freshwater Fish	5
10	Australia	2004	Fruits, Other	54
10	Australia	2004	Grapefruit	2
10	Australia	2004	Grapes	29
10	Australia	2004	Groundnuts (Shelled Eq)	2
10	Australia	2004	Honey	1

FAO country code	Country	Reference year	Food item	Gross food supply (g *capita*$^{-1}$ d^{-1})
10	Australia	2004	Lemons, Limes	4
10	Australia	2004	Maize	14
10	Australia	2004	Maize Germ Oil	1
10	Australia	2004	Marine Fish, Other	6
10	Australia	2004	Meat, Other	3
10	Australia	2004	Milk, Whole	245
10	Australia	2004	Molluscs, Other	10
10	Australia	2004	Mutton & Goat Meat	35
10	Australia	2004	Nuts	17
10	Australia	2004	Oats	9
10	Australia	2004	Offals, Edible	26
10	Australia	2004	Olive Oil	4
10	Australia	2004	Olives	2
10	Australia	2004	Onions	21
10	Australia	2004	Oranges, Mandarines	62
10	Australia	2004	Palm Oil	15
10	Australia	2004	Peas	1
10	Australia	2004	Pelagic Fish	15
10	Australia	2004	Pigmeat	56
10	Australia	2004	Pineapples	19
10	Australia	2004	Potatoes	152
10	Australia	2004	Poultry Meat	94
10	Australia	2004	Pulses, Other	2
10	Australia	2004	Rape and Mustard Oil	21
10	Australia	2004	Rice (Milled Equivalent)	27
10	Australia	2004	Rye	2
10	Australia	2004	Soyabean Oil	2
10	Australia	2004	Sugar, Refined Equivalent	100
10	Australia	2004	Sunflowerseed Oil	1
10	Australia	2004	Sweet Potatoes	5
10	Australia	2004	Sweeteners, Other	18
10	Australia	2004	Tea	2
10	Australia	2004	Tomatoes	75
10	Australia	2004	Vegetables, Other	179
10	Australia	2004	Wheat	179
10	Australia	2004	Wine	59
10	Australia	2005	Apples	73
10	Australia	2005	Aquatic Animals, Others	1
10	Australia	2005	Bananas	32
10	Australia	2005	Beer	242
10	Australia	2005	Beverages, Alcoholic	3
10	Australia	2005	Beverages, Fermented	2
10	Australia	2005	Bovine Meat	116
10	Australia	2005	Butter, Ghee	11
10	Australia	2005	Cephalopods	2
10	Australia	2005	Cheese	31
10	Australia	2005	Cocoa Beans	5
10	Australia	2005	Coconut Oil	1
10	Australia	2005	Coconuts - Incl Copra	6
10	Australia	2005	Coffee	12
10	Australia	2005	Cottonseed Oil	6
10	Australia	2005	Crustaceans	10
10	Australia	2005	Dates	1
10	Australia	2005	Demersal Fish	25
10	Australia	2005	Eggs	14
10	Australia	2005	Fats, Animals, Raw	10
10	Australia	2005	Freshwater Fish	5
10	Australia	2005	Fruits, Other	66
10	Australia	2005	Grapefruit	2
10	Australia	2005	Grapes	30
10	Australia	2005	Groundnuts (Shelled Eq)	3
10	Australia	2005	Honey	1
10	Australia	2005	Lemons, Limes	4
10	Australia	2005	Maize	14
10	Australia	2005	Maize Germ Oil	1
10	Australia	2005	Marine Fish, Other	6
10	Australia	2005	Meat, Other	3
10	Australia	2005	Milk, Whole	256
10	Australia	2005	Molluscs, Other	9
10	Australia	2005	Mutton & Goat Meat	35
10	Australia	2005	Nuts	20
10	Australia	2005	Oats	8
10	Australia	2005	Offals, Edible	27
10	Australia	2005	Olive Oil	4
10	Australia	2005	Olives	3
10	Australia	2005	Onions	26
10	Australia	2005	Oranges, Mandarines	68
10	Australia	2005	Palm Oil	11
10	Australia	2005	Peas	1
10	Australia	2005	Pelagic Fish	10
10	Australia	2005	Pigmeat	60
10	Australia	2005	Pineapples	19
10	Australia	2005	Potatoes	149
10	Australia	2005	Poultry Meat	101
10	Australia	2005	Pulses, Other	2
10	Australia	2005	Rape and Mustard Oil	27
10	Australia	2005	Rice (Milled Equivalent)	27
10	Australia	2005	Rye	2
10	Australia	2005	Soyabean Oil	2
10	Australia	2005	Sugar, Refined Equivalent	99
10	Australia	2005	Sunflowerseed Oil	5
10	Australia	2005	Sweet Potatoes	5
10	Australia	2005	Sweeteners, Other	19
10	Australia	2005	Tea	2
10	Australia	2005	Tomatoes	68
10	Australia	2005	Vegetables, Other	174
10	Australia	2005	Wheat	175

FAO country code	Country	Reference year	Food item	Gross food supply (g $capita^{-1}$ d^{-1})
10	Australia	2005	Wine	61
10	Australia	2006	Apples	66
10	Australia	2006	Aquatic Animals, Others	1
10	Australia	2006	Bananas	24
10	Australia	2006	Beer	235
10	Australia	2006	Beverages, Alcoholic	4
10	Australia	2006	Beverages, Fermented	2
10	Australia	2006	Bovine Meat	97
10	Australia	2006	Butter, Ghee	11
10	Australia	2006	Cassava	1
10	Australia	2006	Cephalopods	2
10	Australia	2006	Cheese	30
10	Australia	2006	Cocoa Beans	6
10	Australia	2006	Coconuts - Incl Copra	6
10	Australia	2006	Coffee	12
10	Australia	2006	Cottonseed Oil	9
10	Australia	2006	Crustaceans	11
10	Australia	2006	Dates	1
10	Australia	2006	Demersal Fish	25
10	Australia	2006	Eggs	18
10	Australia	2006	Fats, Animals, Raw	9
10	Australia	2006	Freshwater Fish	6
10	Australia	2006	Fruits, Other	62
10	Australia	2006	Grapefruit	2
10	Australia	2006	Grapes	24
10	Australia	2006	Groundnuts (Shelled Eq)	3
10	Australia	2006	Honey	1
10	Australia	2006	Lemons, Limes	5
10	Australia	2006	Maize	14
10	Australia	2006	Maize Germ Oil	1
10	Australia	2006	Marine Fish, Other	5
10	Australia	2006	Meat, Other	3
10	Australia	2006	Milk, Whole	339
10	Australia	2006	Molluscs, Other	9
10	Australia	2006	Mutton & Goat Meat	37
10	Australia	2006	Nuts	21
10	Australia	2006	Oats	7
10	Australia	2006	Offals, Edible	26
10	Australia	2006	Olive Oil	4
10	Australia	2006	Olives	6
10	Australia	2006	Onions	23
10	Australia	2006	Oranges, Mandarines	68
10	Australia	2006	Palm Oil	12
10	Australia	2006	Peas	2
10	Australia	2006	Pelagic Fish	10
10	Australia	2006	Pigmeat	59
10	Australia	2006	Pineapples	25
10	Australia	2006	Potatoes	148
10	Australia	2006	Poultry Meat	103
10	Australia	2006	Pulses, Other	2
10	Australia	2006	Rape and Mustard Oil	25
10	Australia	2006	Rice (Milled Equivalent)	27
10	Australia	2006	Rye	1
10	Australia	2006	Soyabean Oil	4
10	Australia	2006	Sugar, Refined Equivalent	99
10	Australia	2006	Sunflowerseed Oil	6
10	Australia	2006	Sweet Potatoes	5
10	Australia	2006	Sweeteners, Other	19
10	Australia	2006	Tea	2
10	Australia	2006	Tomatoes	71
10	Australia	2006	Vegetables, Other	173
10	Australia	2006	Wheat	180
10	Australia	2006	Wine	61
10	Australia	2007	Apples	71
10	Australia	2007	Aquatic Animals, Others	1
10	Australia	2007	Bananas	25
10	Australia	2007	Beer	231
10	Australia	2007	Beverages, Alcoholic	4
10	Australia	2007	Beverages, Fermented	2
10	Australia	2007	Bovine Meat	118
10	Australia	2007	Butter, Ghee	11
10	Australia	2007	Cephalopods	2
10	Australia	2007	Cheese	27
10	Australia	2007	Cocoa Beans	8
10	Australia	2007	Coconut Oil	2
10	Australia	2007	Coconuts - Incl Copra	6
10	Australia	2007	Coffee	12
10	Australia	2007	Cottonseed Oil	7
10	Australia	2007	Crustaceans	10
10	Australia	2007	Dates	1
10	Australia	2007	Demersal Fish	25
10	Australia	2007	Eggs	17
10	Australia	2007	Fats, Animals, Raw	10
10	Australia	2007	Freshwater Fish	6
10	Australia	2007	Fruits, Other	67
10	Australia	2007	Grapefruit	1
10	Australia	2007	Grapes	29
10	Australia	2007	Groundnuts (Shelled Eq)	4
10	Australia	2007	Honey	2
10	Australia	2007	Lemons, Limes	5
10	Australia	2007	Maize	14
10	Australia	2007	Marine Fish, Other	7
10	Australia	2007	Meat, Other	3
10	Australia	2007	Milk, Whole	321
10	Australia	2007	Molluscs, Other	9
10	Australia	2007	Mutton & Goat Meat	39
10	Australia	2007	Nuts	24

FAO country code	Country	Reference year	Food item	Gross food supply (g $capita^{-1}$ d^{-1})
10	Australia	2007	Oats	7
10	Australia	2007	Offals, Edible	28
10	Australia	2007	Oilcrops Oil, Other	1
10	Australia	2007	Olive Oil	6
10	Australia	2007	Olives	7
10	Australia	2007	Onions	25
10	Australia	2007	Oranges, Mandarines	63
10	Australia	2007	Palm Oil	10
10	Australia	2007	Peas	1
10	Australia	2007	Pelagic Fish	10
10	Australia	2007	Pigmeat	62
10	Australia	2007	Pineapples	27
10	Australia	2007	Potatoes	148
10	Australia	2007	Poultry Meat	107
10	Australia	2007	Pulses, Other	2
10	Australia	2007	Rape and Mustard Oil	25
10	Australia	2007	Rice (Milled Equivalent)	27
10	Australia	2007	Roots, Other	1
10	Australia	2007	Rye	1
10	Australia	2007	Soyabean Oil	3
10	Australia	2007	Spices, Other	1
10	Australia	2007	Sugar, Refined Equivalent	98
10	Australia	2007	Sunflowerseed Oil	6
10	Australia	2007	Sweet Potatoes	6
10	Australia	2007	Sweeteners, Other	21
10	Australia	2007	Tea	2
10	Australia	2007	Tomatoes	57
10	Australia	2007	Vegetables, Other	191
10	Australia	2007	Wheat	186
10	Australia	2007	Wine	63
10	Australia	2008	Apples	71
10	Australia	2008	Aquatic Animals, Others	1
10	Australia	2008	Bananas	24
10	Australia	2008	Beer	218
10	Australia	2008	Beverages, Alcoholic	3
10	Australia	2008	Beverages, Fermented	2
10	Australia	2008	Bovine Meat	104
10	Australia	2008	Butter, Ghee	11
10	Australia	2008	Cephalopods	2
10	Australia	2008	Cheese	34
10	Australia	2008	Cocoa Beans	8
10	Australia	2008	Coconut Oil	4
10	Australia	2008	Coconuts - Incl Copra	6
10	Australia	2008	Coffee	13
10	Australia	2008	Cottonseed Oil	5
10	Australia	2008	Crustaceans	12
10	Australia	2008	Dates	1
10	Australia	2008	Demersal Fish	24
10	Australia	2008	Eggs	16
10	Australia	2008	Fats, Animals, Raw	10
10	Australia	2008	Freshwater Fish	6
10	Australia	2008	Fruits, Other	61
10	Australia	2008	Grapefruit	1
10	Australia	2008	Grapes	33
10	Australia	2008	Groundnuts (Shelled Eq)	3
10	Australia	2008	Honey	2
10	Australia	2008	Lemons, Limes	5
10	Australia	2008	Maize	14
10	Australia	2008	Marine Fish, Other	7
10	Australia	2008	Meat, Other	3
10	Australia	2008	Milk, Whole	280
10	Australia	2008	Molluscs, Other	8
10	Australia	2008	Mutton & Goat Meat	34
10	Australia	2008	Nuts	23
10	Australia	2008	Oats	4
10	Australia	2008	Offals, Edible	26
10	Australia	2008	Oilcrops Oil, Other	1
10	Australia	2008	Olive Oil	3
10	Australia	2008	Olives	5
10	Australia	2008	Onions	26
10	Australia	2008	Oranges, Mandarines	56
10	Australia	2008	Palm Oil	16
10	Australia	2008	Peas	1
10	Australia	2008	Pelagic Fish	12
10	Australia	2008	Pigmeat	61
10	Australia	2008	Pineapples	26
10	Australia	2008	Potatoes	176
10	Australia	2008	Poultry Meat	102
10	Australia	2008	Pulses, Other	3
10	Australia	2008	Rape and Mustard Oil	21
10	Australia	2008	Rice (Milled Equivalent)	31
10	Australia	2008	Rye	2
10	Australia	2008	Soyabean Oil	3
10	Australia	2008	Spices, Other	1
10	Australia	2008	Sugar, Refined Equivalent	97
10	Australia	2008	Sunflowerseed Oil	4
10	Australia	2008	Sweet Potatoes	4
10	Australia	2008	Sweeteners, Other	21
10	Australia	2008	Tea	2
10	Australia	2008	Tomatoes	76
10	Australia	2008	Vegetables, Other	178
10	Australia	2008	Wheat	198
10	Australia	2008	Wine	61
10	Australia	2009	Apples	76
10	Australia	2009	Aquatic Animals, Others	1
10	Australia	2009	Bananas	31
10	Australia	2009	Beer	232

FAO country code	Country	Reference year	Food item	Gross food supply (g $capita^{-1}$ d^{-1})
10	Australia	2009	Beverages, Alcoholic	3
10	Australia	2009	Beverages, Fermented	2
10	Australia	2009	Bovine Meat	106
10	Australia	2009	Butter, Ghee	11
10	Australia	2009	Cassava	1
10	Australia	2009	Cephalopods	2
10	Australia	2009	Cheese	30
10	Australia	2009	Cocoa Beans	8
10	Australia	2009	Coconuts - Incl Copra	6
10	Australia	2009	Coffee	13
10	Australia	2009	Cottonseed Oil	6
10	Australia	2009	Crustaceans	12
10	Australia	2009	Dates	1
10	Australia	2009	Demersal Fish	21
10	Australia	2009	Eggs	16
10	Australia	2009	Fats, Animals, Raw	11
10	Australia	2009	Freshwater Fish	6
10	Australia	2009	Fruits, Other	58
10	Australia	2009	Grapefruit	1
10	Australia	2009	Grapes	21
10	Australia	2009	Groundnuts (Shelled Eq)	2
10	Australia	2009	Honey	2
10	Australia	2009	Lemons, Limes	4
10	Australia	2009	Maize	14
10	Australia	2009	Marine Fish, Other	7
10	Australia	2009	Meat, Other	3
10	Australia	2009	Milk, Whole	261
10	Australia	2009	Molluscs, Other	9
10	Australia	2009	Mutton & Goat Meat	31
10	Australia	2009	Nuts	24
10	Australia	2009	Oats	2
10	Australia	2009	Offals, Edible	25
10	Australia	2009	Oilcrops Oil, Other	2
10	Australia	2009	Olive Oil	4
10	Australia	2009	Olives	5
10	Australia	2009	Onions	28
10	Australia	2009	Oranges, Mandarines	47
10	Australia	2009	Palm Oil	19
10	Australia	2009	Peas	2
10	Australia	2009	Pelagic Fish	10
10	Australia	2009	Pigmeat	59
10	Australia	2009	Pineapples	25
10	Australia	2009	Potatoes	145
10	Australia	2009	Poultry Meat	105
10	Australia	2009	Pulses, Other	4
10	Australia	2009	Rape and Mustard Oil	22
10	Australia	2009	Rice (Milled Equivalent)	31
10	Australia	2009	Rye	1
10	Australia	2009	Soyabean Oil	3
10	Australia	2009	Spices, Other	1
10	Australia	2009	Sugar, Refined Equivalent	96
10	Australia	2009	Sunflowerseed Oil	6
10	Australia	2009	Sweet Potatoes	5
10	Australia	2009	Sweeteners, Other	20
10	Australia	2009	Tea	2
10	Australia	2009	Tomatoes	72
10	Australia	2009	Vegetables, Other	166
10	Australia	2009	Wheat	209
10	Australia	2009	Wine	64
10	Australia	2010	Apples	73
10	Australia	2010	Aquatic Animals, Others	1
10	Australia	2010	Bananas	34
10	Australia	2010	Beer	210
10	Australia	2010	Beverages, Alcoholic	3
10	Australia	2010	Beverages, Fermented	3
10	Australia	2010	Bovine Meat	102
10	Australia	2010	Butter, Ghee	11
10	Australia	2010	Cassava	1
10	Australia	2010	Cephalopods	2
10	Australia	2010	Cheese	32
10	Australia	2010	Cocoa Beans	8
10	Australia	2010	Coconut Oil	1
10	Australia	2010	Coconuts - Incl Copra	6
10	Australia	2010	Coffee	13
10	Australia	2010	Cottonseed Oil	6
10	Australia	2010	Crustaceans	13
10	Australia	2010	Dates	1
10	Australia	2010	Demersal Fish	23
10	Australia	2010	Eggs	17
10	Australia	2010	Fats, Animals, Raw	10
10	Australia	2010	Freshwater Fish	6
10	Australia	2010	Fruits, Other	57
10	Australia	2010	Grapefruit	1
10	Australia	2010	Grapes	26
10	Australia	2010	Groundnuts (Shelled Eq)	4
10	Australia	2010	Honey	2
10	Australia	2010	Lemons, Limes	4
10	Australia	2010	Maize	14
10	Australia	2010	Maize Germ Oil	1
10	Australia	2010	Marine Fish, Other	7
10	Australia	2010	Meat, Other	3
10	Australia	2010	Milk, Whole	277
10	Australia	2010	Molluscs, Other	9
10	Australia	2010	Mutton & Goat Meat	31
10	Australia	2010	Nuts	25
10	Australia	2010	Offals, Edible	23
10	Australia	2010	Oilcrops Oil, Other	1

FAO country code	Country	Reference year	Food item	Gross food supply (g *capita*$^{-1}$ d^{-1})
10	Australia	2010	Olive Oil	5
10	Australia	2010	Olives	6
10	Australia	2010	Onions	26
10	Australia	2010	Oranges, Mandarines	57
10	Australia	2010	Palm Oil	15
10	Australia	2010	Peas	2
10	Australia	2010	Pelagic Fish	11
10	Australia	2010	Pigmeat	61
10	Australia	2010	Pineapples	20
10	Australia	2010	Potatoes	149
10	Australia	2010	Poultry Meat	110
10	Australia	2010	Pulses, Other	5
10	Australia	2010	Rape and Mustard Oil	27
10	Australia	2010	Rice (Milled Equivalent)	31
10	Australia	2010	Rye	2
10	Australia	2010	Soyabean Oil	3
10	Australia	2010	Spices, Other	1
10	Australia	2010	Sugar, Refined Equivalent	94
10	Australia	2010	Sunflowerseed Oil	3
10	Australia	2010	Sweet Potatoes	5
10	Australia	2010	Sweeteners, Other	22
10	Australia	2010	Tea	2
10	Australia	2010	Tomatoes	78
10	Australia	2010	Vegetables, Other	165
10	Australia	2010	Wheat	202
10	Australia	2010	Wine	66
10	Australia	2011	Apples	77
10	Australia	2011	Aquatic Animals, Others	1
10	Australia	2011	Bananas	22
10	Australia	2011	Beer	230
10	Australia	2011	Beverages, Fermented	4
10	Australia	2011	Bovine Meat	111
10	Australia	2011	Butter, Ghee	10
10	Australia	2011	Cassava	1
10	Australia	2011	Cephalopods	2
10	Australia	2011	Cheese	32
10	Australia	2011	Cocoa Beans	9
10	Australia	2011	Coconut Oil	2
10	Australia	2011	Coconuts - Incl Copra	6
10	Australia	2011	Coffee	13
10	Australia	2011	Cottonseed Oil	8
10	Australia	2011	Crustaceans	13
10	Australia	2011	Dates	1
10	Australia	2011	Demersal Fish	23
10	Australia	2011	Eggs	20
10	Australia	2011	Fats, Animals, Raw	9
10	Australia	2011	Freshwater Fish	6
10	Australia	2011	Fruits, Other	63
10	Australia	2011	Grapefruit	1
10	Australia	2011	Grapes	27
10	Australia	2011	Groundnuts (Shelled Eq)	3
10	Australia	2011	Honey	1
10	Australia	2011	Lemons, Limes	4
10	Australia	2011	Maize	14
10	Australia	2011	Maize Germ Oil	1
10	Australia	2011	Marine Fish, Other	7
10	Australia	2011	Meat, Other	3
10	Australia	2011	Milk, Whole	309
10	Australia	2011	Molluscs, Other	9
10	Australia	2011	Mutton & Goat Meat	31
10	Australia	2011	Nuts	24
10	Australia	2011	Oats	2
10	Australia	2011	Offals, Edible	22
10	Australia	2011	Oilcrops Oil, Other	2
10	Australia	2011	Olive Oil	4
10	Australia	2011	Olives	6
10	Australia	2011	Onions	32
10	Australia	2011	Oranges, Mandarines	45
10	Australia	2011	Palm Oil	13
10	Australia	2011	Peas	7
10	Australia	2011	Pelagic Fish	11
10	Australia	2011	Pigmeat	63
10	Australia	2011	Pineapples	17
10	Australia	2011	Potatoes	136
10	Australia	2011	Poultry Meat	124
10	Australia	2011	Pulses, Other	4
10	Australia	2011	Rape and Mustard Oil	26
10	Australia	2011	Rice (Milled Equivalent)	30
10	Australia	2011	Rye	3
10	Australia	2011	Soyabean Oil	5
10	Australia	2011	Spices, Other	1
10	Australia	2011	Sugar, Refined Equivalent	93
10	Australia	2011	Sunflowerseed Oil	4
10	Australia	2011	Sweet Potatoes	4
10	Australia	2011	Sweeteners, Other	25
10	Australia	2011	Tea	1
10	Australia	2011	Tomatoes	66
10	Australia	2011	Vegetables, Other	164
10	Australia	2011	Wheat	191
10	Australia	2011	Wine	55
11	Austria	1992	Apples	126
11	Austria	1992	Bananas	40
11	Austria	1992	Barley	1
11	Austria	1992	Beans	1
11	Austria	1992	Beer	335
11	Austria	1992	Beverages, Alcoholic	9
11	Austria	1992	Beverages, Fermented	5

FAO country code	Country	Reference year	Food item	Gross food supply (g $capita^{-1}$ d^{-1})
11	Austria	1992	Bovine Meat	68
11	Austria	1992	Butter, Ghee	15
11	Austria	1992	Cheese	35
11	Austria	1992	Cocoa Beans	8
11	Austria	1992	Coconut Oil	3
11	Austria	1992	Coconuts - Incl Copra	3
11	Austria	1992	Coffee	26
11	Austria	1992	Cream	16
11	Austria	1992	Crustaceans	1
11	Austria	1992	Demersal Fish	4
11	Austria	1992	Eggs	36
11	Austria	1992	Fats, Animals, Raw	27
11	Austria	1992	Freshwater Fish	4
11	Austria	1992	Fruits, Other	110
11	Austria	1992	Grapefruit	3
11	Austria	1992	Grapes	27
11	Austria	1992	Groundnut Oil	1
11	Austria	1992	Groundnuts (Shelled Eq)	2
11	Austria	1992	Honey	4
11	Austria	1992	Lemons, Limes	10
11	Austria	1992	Maize	12
11	Austria	1992	Maize Germ Oil	2
11	Austria	1992	Marine Fish, Other	7
11	Austria	1992	Meat, Other	4
11	Austria	1992	Milk, Whole	312
11	Austria	1992	Mutton & Goat Meat	3
11	Austria	1992	Nuts	20
11	Austria	1992	Oats	3
11	Austria	1992	Offals, Edible	11
11	Austria	1992	Oilcrops Oil, Other	8
11	Austria	1992	Oilcrops, Other	1
11	Austria	1992	Olive Oil	1
11	Austria	1992	Olives	1
11	Austria	1992	Onions	16
11	Austria	1992	Oranges, Mandarines	81
11	Austria	1992	Palm Oil	4
11	Austria	1992	Peas	1
11	Austria	1992	Pelagic Fish	12
11	Austria	1992	Pigmeat	185
11	Austria	1992	Pimento	1
11	Austria	1992	Pineapples	4
11	Austria	1992	Potatoes	169
11	Austria	1992	Poultry Meat	40
11	Austria	1992	Pulses, Other	1
11	Austria	1992	Rape and Mustard Oil	21
11	Austria	1992	Rice (Milled Equivalent)	16
11	Austria	1992	Rye	45
11	Austria	1992	Soyabean Oil	1
11	Austria	1992	Soyabeans	1
11	Austria	1992	Spices, Other	1
11	Austria	1992	Sugar, Refined Equivalent	100
11	Austria	1992	Sunflowerseed Oil	11
11	Austria	1992	Sweeteners, Other	7
11	Austria	1992	Tea	1
11	Austria	1992	Tomatoes	35
11	Austria	1992	Vegetables, Other	131
11	Austria	1992	Wheat	173
11	Austria	1992	Wine	93
11	Austria	1993	Apples	162
11	Austria	1993	Bananas	38
11	Austria	1993	Barley	1
11	Austria	1993	Beans	1
11	Austria	1993	Beer	318
11	Austria	1993	Beverages, Alcoholic	11
11	Austria	1993	Beverages, Fermented	5
11	Austria	1993	Bovine Meat	63
11	Austria	1993	Butter, Ghee	14
11	Austria	1993	Cheese	34
11	Austria	1993	Cocoa Beans	8
11	Austria	1993	Coconut Oil	2
11	Austria	1993	Coconuts - Incl Copra	3
11	Austria	1993	Coffee	27
11	Austria	1993	Cream	16
11	Austria	1993	Crustaceans	1
11	Austria	1993	Demersal Fish	5
11	Austria	1993	Eggs	37
11	Austria	1993	Fats, Animals, Raw	27
11	Austria	1993	Freshwater Fish	3
11	Austria	1993	Fruits, Other	109
11	Austria	1993	Grapefruit	2
11	Austria	1993	Grapes	25
11	Austria	1993	Groundnut Oil	1
11	Austria	1993	Groundnuts (Shelled Eq)	2
11	Austria	1993	Honey	5
11	Austria	1993	Lemons, Limes	10
11	Austria	1993	Maize	12
11	Austria	1993	Maize Germ Oil	2
11	Austria	1993	Marine Fish, Other	6
11	Austria	1993	Meat, Other	3
11	Austria	1993	Milk, Whole	312
11	Austria	1993	Mutton & Goat Meat	3
11	Austria	1993	Nuts	23
11	Austria	1993	Oats	3
11	Austria	1993	Offals, Edible	10
11	Austria	1993	Oilcrops Oil, Other	9
11	Austria	1993	Oilcrops, Other	1
11	Austria	1993	Olive Oil	1

FAO country code	Country	Reference year	Food item	Gross food supply (g *capita*$^{-1}$ d^{-1})
11	Austria	1993	Olives	1
11	Austria	1993	Onions	17
11	Austria	1993	Oranges, Mandarines	102
11	Austria	1993	Palm Oil	2
11	Austria	1993	Peas	1
11	Austria	1993	Pelagic Fish	11
11	Austria	1993	Pigmeat	180
11	Austria	1993	Pimento	1
11	Austria	1993	Pineapples	4
11	Austria	1993	Potatoes	170
11	Austria	1993	Poultry Meat	41
11	Austria	1993	Rape and Mustard Oil	19
11	Austria	1993	Rice (Milled Equivalent)	19
11	Austria	1993	Rye	43
11	Austria	1993	Soyabean Oil	4
11	Austria	1993	Soyabeans	2
11	Austria	1993	Spices, Other	1
11	Austria	1993	Sugar, Refined Equivalent	95
11	Austria	1993	Sunflowerseed Oil	11
11	Austria	1993	Sweeteners, Other	8
11	Austria	1993	Tomatoes	35
11	Austria	1993	Vegetables, Other	130
11	Austria	1993	Wheat	168
11	Austria	1993	Wine	87
11	Austria	1994	Apples	110
11	Austria	1994	Aquatic Animals, Others	1
11	Austria	1994	Bananas	37
11	Austria	1994	Barley	1
11	Austria	1994	Beans	2
11	Austria	1994	Beer	317
11	Austria	1994	Beverages, Alcoholic	11
11	Austria	1994	Beverages, Fermented	5
11	Austria	1994	Bovine Meat	60
11	Austria	1994	Butter, Ghee	13
11	Austria	1994	Cheese	40
11	Austria	1994	Cocoa Beans	7
11	Austria	1994	Coconut Oil	3
11	Austria	1994	Coconuts - Incl Copra	3
11	Austria	1994	Coffee	22
11	Austria	1994	Cream	16
11	Austria	1994	Crustaceans	1
11	Austria	1994	Demersal Fish	5
11	Austria	1994	Eggs	39
11	Austria	1994	Fats, Animals, Raw	26
11	Austria	1994	Freshwater Fish	4
11	Austria	1994	Fruits, Other	103
11	Austria	1994	Grapefruit	2
11	Austria	1994	Grapes	26
11	Austria	1994	Groundnut Oil	1
11	Austria	1994	Groundnuts (Shelled Eq)	2
11	Austria	1994	Honey	5
11	Austria	1994	Lemons, Limes	11
11	Austria	1994	Maize	23
11	Austria	1994	Maize Germ Oil	2
11	Austria	1994	Marine Fish, Other	7
11	Austria	1994	Meat, Other	3
11	Austria	1994	Milk, Whole	304
11	Austria	1994	Mutton & Goat Meat	3
11	Austria	1994	Nuts	18
11	Austria	1994	Oats	3
11	Austria	1994	Offals, Edible	10
11	Austria	1994	Oilcrops Oil, Other	9
11	Austria	1994	Oilcrops, Other	1
11	Austria	1994	Olive Oil	1
11	Austria	1994	Olives	1
11	Austria	1994	Onions	18
11	Austria	1994	Oranges, Mandarines	124
11	Austria	1994	Palm Oil	1
11	Austria	1994	Peas	1
11	Austria	1994	Pelagic Fish	15
11	Austria	1994	Pigmeat	196
11	Austria	1994	Pimento	1
11	Austria	1994	Pineapples	6
11	Austria	1994	Potatoes	148
11	Austria	1994	Poultry Meat	40
11	Austria	1994	Rape and Mustard Oil	17
11	Austria	1994	Rice (Milled Equivalent)	14
11	Austria	1994	Rye	39
11	Austria	1994	Soyabean Oil	4
11	Austria	1994	Soyabeans	1
11	Austria	1994	Spices, Other	1
11	Austria	1994	Sugar, Refined Equivalent	113
11	Austria	1994	Sunflowerseed Oil	9
11	Austria	1994	Sweeteners, Other	7
11	Austria	1994	Tea	1
11	Austria	1994	Tomatoes	39
11	Austria	1994	Vegetables, Other	135
11	Austria	1994	Wheat	170
11	Austria	1994	Wine	89
11	Austria	1995	Apples	108
11	Austria	1995	Bananas	28
11	Austria	1995	Barley	1
11	Austria	1995	Beans	1
11	Austria	1995	Beer	311
11	Austria	1995	Beverages, Alcoholic	13
11	Austria	1995	Beverages, Fermented	5
11	Austria	1995	Bovine Meat	55

FAO country code	Country	Reference year	Food item	Gross food supply (g *capita*$^{-1}$ d^{-1})
11	Austria	1995	Butter, Ghee	14
11	Austria	1995	Cheese	43
11	Austria	1995	Cocoa Beans	8
11	Austria	1995	Coconut Oil	2
11	Austria	1995	Coconuts - Incl Copra	2
11	Austria	1995	Coffee	19
11	Austria	1995	Cream	13
11	Austria	1995	Crustaceans	1
11	Austria	1995	Demersal Fish	9
11	Austria	1995	Eggs	37
11	Austria	1995	Fats, Animals, Raw	26
11	Austria	1995	Freshwater Fish	4
11	Austria	1995	Fruits, Other	85
11	Austria	1995	Grapefruit	3
11	Austria	1995	Grapes	19
11	Austria	1995	Groundnut Oil	1
11	Austria	1995	Groundnuts (Shelled Eq)	1
11	Austria	1995	Honey	4
11	Austria	1995	Lemons, Limes	11
11	Austria	1995	Maize	29
11	Austria	1995	Maize Germ Oil	2
11	Austria	1995	Marine Fish, Other	1
11	Austria	1995	Meat, Other	3
11	Austria	1995	Milk, Whole	301
11	Austria	1995	Molluscs, Other	1
11	Austria	1995	Mutton & Goat Meat	3
11	Austria	1995	Nuts	17
11	Austria	1995	Oats	3
11	Austria	1995	Offals, Edible	10
11	Austria	1995	Oilcrops Oil, Other	8
11	Austria	1995	Oilcrops, Other	1
11	Austria	1995	Onions	15
11	Austria	1995	Oranges, Mandarines	73
11	Austria	1995	Palm Oil	1
11	Austria	1995	Peas	1
11	Austria	1995	Pelagic Fish	7
11	Austria	1995	Pepper	1
11	Austria	1995	Pigmeat	191
11	Austria	1995	Pimento	1
11	Austria	1995	Pineapples	2
11	Austria	1995	Potatoes	184
11	Austria	1995	Poultry Meat	42
11	Austria	1995	Rape and Mustard Oil	18
11	Austria	1995	Rice (Milled Equivalent)	13
11	Austria	1995	Rye	33
11	Austria	1995	Soyabean Oil	6
11	Austria	1995	Soyabeans	2
11	Austria	1995	Spices, Other	1
11	Austria	1995	Sugar, Refined Equivalent	110
11	Austria	1995	Sunflowerseed Oil	9
11	Austria	1995	Sweeteners, Other	8
11	Austria	1995	Tomatoes	33
11	Austria	1995	Vegetables, Other	171
11	Austria	1995	Wheat	188
11	Austria	1995	Wine	88
11	Austria	1996	Apples	98
11	Austria	1996	Bananas	22
11	Austria	1996	Barley	1
11	Austria	1996	Beans	1
11	Austria	1996	Beer	308
11	Austria	1996	Beverages, Alcoholic	12
11	Austria	1996	Beverages, Fermented	6
11	Austria	1996	Bovine Meat	60
11	Austria	1996	Butter, Ghee	14
11	Austria	1996	Cheese	48
11	Austria	1996	Cocoa Beans	7
11	Austria	1996	Coconut Oil	3
11	Austria	1996	Coconuts - Incl Copra	2
11	Austria	1996	Coffee	20
11	Austria	1996	Cream	16
11	Austria	1996	Crustaceans	1
11	Austria	1996	Demersal Fish	11
11	Austria	1996	Eggs	38
11	Austria	1996	Fats, Animals, Raw	27
11	Austria	1996	Freshwater Fish	4
11	Austria	1996	Fruits, Other	91
11	Austria	1996	Grapefruit	2
11	Austria	1996	Grapes	18
11	Austria	1996	Groundnuts (Shelled Eq)	1
11	Austria	1996	Honey	4
11	Austria	1996	Lemons, Limes	6
11	Austria	1996	Maize	31
11	Austria	1996	Maize Germ Oil	2
11	Austria	1996	Marine Fish, Other	1
11	Austria	1996	Meat, Other	3
11	Austria	1996	Milk, Whole	244
11	Austria	1996	Molluscs, Other	1
11	Austria	1996	Mutton & Goat Meat	3
11	Austria	1996	Nuts	15
11	Austria	1996	Oats	3
11	Austria	1996	Offals, Edible	9
11	Austria	1996	Oilcrops Oil, Other	8
11	Austria	1996	Oilcrops, Other	1
11	Austria	1996	Onions	19
11	Austria	1996	Oranges, Mandarines	32
11	Austria	1996	Palm Oil	2
11	Austria	1996	Pelagic Fish	10

FAO country code	Country	Reference year	Food item	Gross food supply (g $capita^{-1}$ d^{-1})
11	Austria	1996	Pepper	1
11	Austria	1996	Pigmeat	201
11	Austria	1996	Pimento	1
11	Austria	1996	Pineapples	3
11	Austria	1996	Potatoes	171
11	Austria	1996	Poultry Meat	42
11	Austria	1996	Rape and Mustard Oil	20
11	Austria	1996	Rice (Milled Equivalent)	12
11	Austria	1996	Rye	34
11	Austria	1996	Soyabean Oil	3
11	Austria	1996	Soyabeans	2
11	Austria	1996	Spices, Other	1
11	Austria	1996	Sugar, Refined Equivalent	111
11	Austria	1996	Sunflowerseed Oil	10
11	Austria	1996	Sweeteners, Other	6
11	Austria	1996	Tea	1
11	Austria	1996	Tomatoes	34
11	Austria	1996	Vegetables, Other	172
11	Austria	1996	Wheat	200
11	Austria	1996	Wine	84
11	Austria	1997	Apples	127
11	Austria	1997	Bananas	27
11	Austria	1997	Barley	1
11	Austria	1997	Beans	1
11	Austria	1997	Beer	313
11	Austria	1997	Beverages, Alcoholic	14
11	Austria	1997	Beverages, Fermented	6
11	Austria	1997	Bovine Meat	55
11	Austria	1997	Butter, Ghee	14
11	Austria	1997	Cheese	49
11	Austria	1997	Citrus, Other	1
11	Austria	1997	Cocoa Beans	5
11	Austria	1997	Coconut Oil	2
11	Austria	1997	Coconuts - Incl Copra	2
11	Austria	1997	Coffee	22
11	Austria	1997	Cream	13
11	Austria	1997	Crustaceans	1
11	Austria	1997	Demersal Fish	10
11	Austria	1997	Eggs	39
11	Austria	1997	Fats, Animals, Raw	28
11	Austria	1997	Freshwater Fish	5
11	Austria	1997	Fruits, Other	91
11	Austria	1997	Grapefruit	1
11	Austria	1997	Grapes	19
11	Austria	1997	Groundnuts (Shelled Eq)	1
11	Austria	1997	Honey	3
11	Austria	1997	Lemons, Limes	6
11	Austria	1997	Maize	33
11	Austria	1997	Maize Germ Oil	2
11	Austria	1997	Marine Fish, Other	1
11	Austria	1997	Meat, Other	3
11	Austria	1997	Milk, Whole	285
11	Austria	1997	Molluscs, Other	1
11	Austria	1997	Mutton & Goat Meat	3
11	Austria	1997	Nuts	15
11	Austria	1997	Oats	4
11	Austria	1997	Offals, Edible	4
11	Austria	1997	Oilcrops Oil, Other	9
11	Austria	1997	Oilcrops, Other	1
11	Austria	1997	Olive Oil	1
11	Austria	1997	Olives	1
11	Austria	1997	Onions	16
11	Austria	1997	Oranges, Mandarines	52
11	Austria	1997	Palm Oil	1
11	Austria	1997	Peas	1
11	Austria	1997	Pelagic Fish	11
11	Austria	1997	Pepper	1
11	Austria	1997	Pigmeat	200
11	Austria	1997	Pimento	1
11	Austria	1997	Pineapples	2
11	Austria	1997	Potatoes	165
11	Austria	1997	Poultry Meat	44
11	Austria	1997	Rape and Mustard Oil	20
11	Austria	1997	Rice (Milled Equivalent)	12
11	Austria	1997	Rye	39
11	Austria	1997	Soyabean Oil	2
11	Austria	1997	Soyabeans	2
11	Austria	1997	Spices, Other	1
11	Austria	1997	Sugar, Refined Equivalent	111
11	Austria	1997	Sunflowerseed Oil	10
11	Austria	1997	Sweeteners, Other	4
11	Austria	1997	Tomatoes	33
11	Austria	1997	Vegetables, Other	174
11	Austria	1997	Wheat	181
11	Austria	1997	Wine	86
11	Austria	1998	Apples	90
11	Austria	1998	Bananas	26
11	Austria	1998	Barley	1
11	Austria	1998	Beans	1
11	Austria	1998	Beer	313
11	Austria	1998	Beverages, Alcoholic	14
11	Austria	1998	Beverages, Fermented	2
11	Austria	1998	Bovine Meat	55
11	Austria	1998	Butter, Ghee	14
11	Austria	1998	Cheese	53
11	Austria	1998	Citrus, Other	2
11	Austria	1998	Cocoa Beans	5

FAO countrycode	Country	Reference year	Food item	Gross food supply (g $capita^{-1}$ d^{-1})
11	Austria	1998	Coconut Oil	1
11	Austria	1998	Coconuts - Incl Copra	2
11	Austria	1998	Coffee	22
11	Austria	1998	Cream	11
11	Austria	1998	Crustaceans	1
11	Austria	1998	Demersal Fish	10
11	Austria	1998	Eggs	38
11	Austria	1998	Fats, Animals, Raw	27
11	Austria	1998	Freshwater Fish	5
11	Austria	1998	Fruits, Other	81
11	Austria	1998	Grapes	12
11	Austria	1998	Groundnuts (Shelled Eq)	2
11	Austria	1998	Honey	4
11	Austria	1998	Lemons, Limes	6
11	Austria	1998	Maize	39
11	Austria	1998	Maize Germ Oil	2
11	Austria	1998	Marine Fish, Other	1
11	Austria	1998	Meat, Other	3
11	Austria	1998	Milk, Whole	226
11	Austria	1998	Molluscs, Other	1
11	Austria	1998	Mutton & Goat Meat	3
11	Austria	1998	Nuts	16
11	Austria	1998	Oats	3
11	Austria	1998	Offals, Edible	3
11	Austria	1998	Oilcrops Oil, Other	8
11	Austria	1998	Oilcrops, Other	1
11	Austria	1998	Olive Oil	1
11	Austria	1998	Olives	1
11	Austria	1998	Onions	20
11	Austria	1998	Oranges, Mandarines	31
11	Austria	1998	Palm Oil	2
11	Austria	1998	Pelagic Fish	11
11	Austria	1998	Pepper	1
11	Austria	1998	Pigmeat	210
11	Austria	1998	Pimento	1
11	Austria	1998	Pineapples	2
11	Austria	1998	Potatoes	152
11	Austria	1998	Poultry Meat	45
11	Austria	1998	Rape and Mustard Oil	17
11	Austria	1998	Rice (Milled Equivalent)	11
11	Austria	1998	Rye	39
11	Austria	1998	Soyabean Oil	4
11	Austria	1998	Soyabeans	2
11	Austria	1998	Spices, Other	1
11	Austria	1998	Sugar, Refined Equivalent	107
11	Austria	1998	Sunflowerseed Oil	13
11	Austria	1998	Sweeteners, Other	7
11	Austria	1998	Tea	1
11	Austria	1998	Tomatoes	19
11	Austria	1998	Vegetables, Other	163
11	Austria	1998	Wheat	219
11	Austria	1998	Wine	86
11	Austria	1999	Apples	111
11	Austria	1999	Bananas	30
11	Austria	1999	Barley	1
11	Austria	1999	Beans	1
11	Austria	1999	Beer	316
11	Austria	1999	Beverages, Alcoholic	18
11	Austria	1999	Beverages, Fermented	1
11	Austria	1999	Bovine Meat	50
11	Austria	1999	Butter, Ghee	13
11	Austria	1999	Cheese	54
11	Austria	1999	Citrus, Other	1
11	Austria	1999	Cocoa Beans	8
11	Austria	1999	Coconut Oil	1
11	Austria	1999	Coconuts - Incl Copra	2
11	Austria	1999	Coffee	22
11	Austria	1999	Cream	11
11	Austria	1999	Crustaceans	1
11	Austria	1999	Demersal Fish	8
11	Austria	1999	Eggs	36
11	Austria	1999	Fats, Animals, Raw	27
11	Austria	1999	Freshwater Fish	6
11	Austria	1999	Fruits, Other	106
11	Austria	1999	Grapes	18
11	Austria	1999	Groundnuts (Shelled Eq)	2
11	Austria	1999	Honey	5
11	Austria	1999	Lemons, Limes	8
11	Austria	1999	Maize	41
11	Austria	1999	Maize Germ Oil	2
11	Austria	1999	Marine Fish, Other	2
11	Austria	1999	Meat, Other	3
11	Austria	1999	Milk, Whole	229
11	Austria	1999	Molluscs, Other	1
11	Austria	1999	Mutton & Goat Meat	3
11	Austria	1999	Nuts	17
11	Austria	1999	Oats	3
11	Austria	1999	Offals, Edible	4
11	Austria	1999	Oilcrops Oil, Other	9
11	Austria	1999	Oilcrops, Other	1
11	Austria	1999	Olive Oil	1
11	Austria	1999	Olives	1
11	Austria	1999	Onions	32
11	Austria	1999	Oranges, Mandarines	32
11	Austria	1999	Palm Oil	1
11	Austria	1999	Peas	1
11	Austria	1999	Pelagic Fish	11

FAO country code	Country	Reference year	Food item	Gross food supply (g $capita^{-1}$ d^{-1})
11	Austria	1999	Pigmeat	214
11	Austria	1999	Pimento	1
11	Austria	1999	Pineapples	2
11	Austria	1999	Potatoes	168
11	Austria	1999	Poultry Meat	46
11	Austria	1999	Rape and Mustard Oil	17
11	Austria	1999	Rice (Milled Equivalent)	11
11	Austria	1999	Rye	38
11	Austria	1999	Sesameseed	1
11	Austria	1999	Soyabean Oil	5
11	Austria	1999	Soyabeans	2
11	Austria	1999	Spices, Other	1
11	Austria	1999	Sugar, Refined Equivalent	107
11	Austria	1999	Sunflowerseed	1
11	Austria	1999	Sunflowerseed Oil	14
11	Austria	1999	Sweeteners, Other	5
11	Austria	1999	Tea	1
11	Austria	1999	Tomatoes	36
11	Austria	1999	Vegetables, Other	186
11	Austria	1999	Wheat	215
11	Austria	1999	Wine	87
11	Austria	2000	Apples	156
11	Austria	2000	Bananas	25
11	Austria	2000	Barley	1
11	Austria	2000	Beans	1
11	Austria	2000	Beer	301
11	Austria	2000	Beverages, Alcoholic	20
11	Austria	2000	Beverages, Fermented	1
11	Austria	2000	Bovine Meat	53
11	Austria	2000	Butter, Ghee	14
11	Austria	2000	Cheese	54
11	Austria	2000	Citrus, Other	11
11	Austria	2000	Cocoa Beans	7
11	Austria	2000	Coconut Oil	1
11	Austria	2000	Coconuts - Incl Copra	2
11	Austria	2000	Coffee	15
11	Austria	2000	Cream	10
11	Austria	2000	Crustaceans	1
11	Austria	2000	Demersal Fish	6
11	Austria	2000	Eggs	34
11	Austria	2000	Fats, Animals, Raw	26
11	Austria	2000	Freshwater Fish	5
11	Austria	2000	Fruits, Other	105
11	Austria	2000	Grapes	18
11	Austria	2000	Groundnuts (Shelled Eq)	2
11	Austria	2000	Honey	4
11	Austria	2000	Lemons, Limes	7
11	Austria	2000	Maize	48
11	Austria	2000	Maize Germ Oil	2
11	Austria	2000	Marine Fish, Other	2
11	Austria	2000	Meat, Other	3
11	Austria	2000	Milk, Whole	226
11	Austria	2000	Molluscs, Other	1
11	Austria	2000	Mutton & Goat Meat	3
11	Austria	2000	Nuts	17
11	Austria	2000	Oats	4
11	Austria	2000	Offals, Edible	1
11	Austria	2000	Oilcrops Oil, Other	11
11	Austria	2000	Oilcrops, Other	1
11	Austria	2000	Olive Oil	1
11	Austria	2000	Olives	1
11	Austria	2000	Onions	17
11	Austria	2000	Oranges, Mandarines	25
11	Austria	2000	Palm Oil	1
11	Austria	2000	Peas	1
11	Austria	2000	Pelagic Fish	10
11	Austria	2000	Pepper	1
11	Austria	2000	Pigmeat	206
11	Austria	2000	Pimento	1
11	Austria	2000	Pineapples	2
11	Austria	2000	Potatoes	170
11	Austria	2000	Poultry Meat	47
11	Austria	2000	Rape and Mustard Oil	18
11	Austria	2000	Rice (Milled Equivalent)	10
11	Austria	2000	Rye	39
11	Austria	2000	Sesameseed	1
11	Austria	2000	Soyabean Oil	3
11	Austria	2000	Soyabeans	3
11	Austria	2000	Spices, Other	1
11	Austria	2000	Sugar, Refined Equivalent	108
11	Austria	2000	Sunflowerseed	1
11	Austria	2000	Sunflowerseed Oil	13
11	Austria	2000	Sweeteners, Other	5
11	Austria	2000	Tea	1
11	Austria	2000	Tomatoes	33
11	Austria	2000	Vegetables, Other	175
11	Austria	2000	Wheat	220
11	Austria	2000	Wine	85
11	Austria	2001	Apples	143
11	Austria	2001	Bananas	25
11	Austria	2001	Barley	1
11	Austria	2001	Beans	1
11	Austria	2001	Beer	304
11	Austria	2001	Beverages, Alcoholic	19
11	Austria	2001	Beverages, Fermented	1
11	Austria	2001	Bovine Meat	51
11	Austria	2001	Butter, Ghee	15

FAO country code	Country	Reference year	Food item	Gross food supply (g *capita*$^{-1}$ d^{-1})
11	Austria	2001	Cheese	57
11	Austria	2001	Citrus, Other	7
11	Austria	2001	Cocoa Beans	7
11	Austria	2001	Coconut Oil	1
11	Austria	2001	Coconuts - Incl Copra	2
11	Austria	2001	Coffee	20
11	Austria	2001	Cream	8
11	Austria	2001	Crustaceans	2
11	Austria	2001	Demersal Fish	7
11	Austria	2001	Eggs	35
11	Austria	2001	Fats, Animals, Raw	27
11	Austria	2001	Freshwater Fish	6
11	Austria	2001	Fruits, Other	117
11	Austria	2001	Grapes	19
11	Austria	2001	Groundnut Oil	1
11	Austria	2001	Groundnuts (Shelled Eq)	2
11	Austria	2001	Honey	4
11	Austria	2001	Lemons, Limes	7
11	Austria	2001	Maize	49
11	Austria	2001	Maize Germ Oil	2
11	Austria	2001	Marine Fish, Other	2
11	Austria	2001	Meat, Other	3
11	Austria	2001	Milk, Whole	223
11	Austria	2001	Molluscs, Other	1
11	Austria	2001	Mutton & Goat Meat	3
11	Austria	2001	Nuts	17
11	Austria	2001	Oats	5
11	Austria	2001	Offals, Edible	2
11	Austria	2001	Oilcrops Oil, Other	8
11	Austria	2001	Oilcrops, Other	1
11	Austria	2001	Olive Oil	2
11	Austria	2001	Olives	1
11	Austria	2001	Onions	30
11	Austria	2001	Oranges, Mandarines	37
11	Austria	2001	Palm Oil	1
11	Austria	2001	Peas	1
11	Austria	2001	Pelagic Fish	11
11	Austria	2001	Pigmeat	197
11	Austria	2001	Pimento	1
11	Austria	2001	Pineapples	1
11	Austria	2001	Potatoes	163
11	Austria	2001	Poultry Meat	49
11	Austria	2001	Pulses, Other	1
11	Austria	2001	Rape and Mustard Oil	20
11	Austria	2001	Rice (Milled Equivalent)	10
11	Austria	2001	Rye	37
11	Austria	2001	Sesameseed	1
11	Austria	2001	Soyabean Oil	10
11	Austria	2001	Soyabeans	3
11	Austria	2001	Spices, Other	1
11	Austria	2001	Sugar, Refined Equivalent	105
11	Austria	2001	Sunflowerseed	1
11	Austria	2001	Sunflowerseed Oil	9
11	Austria	2001	Sweeteners, Other	6
11	Austria	2001	Tea	1
11	Austria	2001	Tomatoes	36
11	Austria	2001	Vegetables, Other	193
11	Austria	2001	Wheat	219
11	Austria	2001	Wine	79
11	Austria	2002	Apples	138
11	Austria	2002	Bananas	25
11	Austria	2002	Barley	1
11	Austria	2002	Beans	1
11	Austria	2002	Beer	306
11	Austria	2002	Beverages, Alcoholic	18
11	Austria	2002	Beverages, Fermented	1
11	Austria	2002	Bovine Meat	52
11	Austria	2002	Butter, Ghee	13
11	Austria	2002	Cheese	57
11	Austria	2002	Citrus, Other	4
11	Austria	2002	Cocoa Beans	6
11	Austria	2002	Coconut Oil	1
11	Austria	2002	Coconuts - Incl Copra	2
11	Austria	2002	Coffee	18
11	Austria	2002	Cream	7
11	Austria	2002	Crustaceans	1
11	Austria	2002	Demersal Fish	7
11	Austria	2002	Eggs	36
11	Austria	2002	Fats, Animals, Raw	27
11	Austria	2002	Freshwater Fish	5
11	Austria	2002	Fruits, Other	97
11	Austria	2002	Grapefruit	2
11	Austria	2002	Grapes	16
11	Austria	2002	Groundnut Oil	1
11	Austria	2002	Groundnuts (Shelled Eq)	2
11	Austria	2002	Honey	4
11	Austria	2002	Lemons, Limes	7
11	Austria	2002	Maize	44
11	Austria	2002	Maize Germ Oil	1
11	Austria	2002	Marine Fish, Other	2
11	Austria	2002	Meat, Other	2
11	Austria	2002	Milk, Whole	227
11	Austria	2002	Molluscs, Other	1
11	Austria	2002	Mutton & Goat Meat	3
11	Austria	2002	Nuts	16
11	Austria	2002	Oats	4
11	Austria	2002	Offals, Edible	3

FAO countrycode	Country	Reference year	Food item	Gross food supply (g *capita*$^{-1}$ d^{-1})
11	Austria	2002	Oilcrops Oil, Other	7
11	Austria	2002	Oilcrops, Other	1
11	Austria	2002	Olive Oil	1
11	Austria	2002	Olives	1
11	Austria	2002	Onions	22
11	Austria	2002	Oranges, Mandarines	34
11	Austria	2002	Palm Oil	1
11	Austria	2002	Peas	1
11	Austria	2002	Pelagic Fish	11
11	Austria	2002	Pigmeat	202
11	Austria	2002	Pimento	1
11	Austria	2002	Pineapples	5
11	Austria	2002	Potatoes	174
11	Austria	2002	Poultry Meat	47
11	Austria	2002	Rape and Mustard Oil	16
11	Austria	2002	Rice (Milled Equivalent)	11
11	Austria	2002	Rye	37
11	Austria	2002	Sesameseed	1
11	Austria	2002	Soyabean Oil	10
11	Austria	2002	Soyabeans	3
11	Austria	2002	Spices, Other	1
11	Austria	2002	Sugar, Refined Equivalent	104
11	Austria	2002	Sunflowerseed	1
11	Austria	2002	Sunflowerseed Oil	11
11	Austria	2002	Sweeteners, Other	6
11	Austria	2002	Tea	1
11	Austria	2002	Tomatoes	35
11	Austria	2002	Vegetables, Other	194
11	Austria	2002	Wheat	219
11	Austria	2002	Wine	82
11	Austria	2003	Apples	159
11	Austria	2003	Bananas	25
11	Austria	2003	Barley	1
11	Austria	2003	Beans	1
11	Austria	2003	Beer	308
11	Austria	2003	Beverages, Alcoholic	19
11	Austria	2003	Bovine Meat	51
11	Austria	2003	Butter, Ghee	14
11	Austria	2003	Cheese	63
11	Austria	2003	Citrus, Other	4
11	Austria	2003	Cocoa Beans	7
11	Austria	2003	Coconut Oil	1
11	Austria	2003	Coconuts - Incl Copra	2
11	Austria	2003	Coffee	17
11	Austria	2003	Cream	9
11	Austria	2003	Crustaceans	2
11	Austria	2003	Demersal Fish	7
11	Austria	2003	Eggs	35
11	Austria	2003	Fats, Animals, Raw	28
11	Austria	2003	Freshwater Fish	6
11	Austria	2003	Fruits, Other	129
11	Austria	2003	Grapefruit	2
11	Austria	2003	Grapes	15
11	Austria	2003	Groundnuts (Shelled Eq)	2
11	Austria	2003	Honey	4
11	Austria	2003	Lemons, Limes	8
11	Austria	2003	Maize	43
11	Austria	2003	Maize Germ Oil	1
11	Austria	2003	Marine Fish, Other	2
11	Austria	2003	Meat, Other	3
11	Austria	2003	Milk, Whole	58
11	Austria	2003	Molluscs, Other	1
11	Austria	2003	Mutton & Goat Meat	3
11	Austria	2003	Nuts	18
11	Austria	2003	Oats	4
11	Austria	2003	Offals, Edible	2
11	Austria	2003	Oilcrops Oil, Other	9
11	Austria	2003	Oilcrops, Other	1
11	Austria	2003	Olive Oil	2
11	Austria	2003	Olives	1
11	Austria	2003	Onions	20
11	Austria	2003	Oranges, Mandarines	35
11	Austria	2003	Palm Oil	2
11	Austria	2003	Peas	1
11	Austria	2003	Pelagic Fish	11
11	Austria	2003	Pigmeat	203
11	Austria	2003	Pimento	1
11	Austria	2003	Pineapples	8
11	Austria	2003	Potatoes	154
11	Austria	2003	Poultry Meat	46
11	Austria	2003	Rape and Mustard Oil	15
11	Austria	2003	Rice (Milled Equivalent)	9
11	Austria	2003	Rye	36
11	Austria	2003	Sesameseed	1
11	Austria	2003	Soyabean Oil	11
11	Austria	2003	Soyabeans	3
11	Austria	2003	Spices, Other	1
11	Austria	2003	Sugar, Refined Equivalent	106
11	Austria	2003	Sunflowerseed	1
11	Austria	2003	Sunflowerseed Oil	10
11	Austria	2003	Sweeteners, Other	8
11	Austria	2003	Tomatoes	39
11	Austria	2003	Vegetables, Other	191
11	Austria	2003	Wheat	226
11	Austria	2003	Wine	77
11	Austria	2004	Apples	133
11	Austria	2004	Bananas	26

FAO country code	Country	Reference year	Food item	Gross food supply (g $capita^{-1}$ d^{-1})
11	Austria	2004	Barley	1
11	Austria	2004	Beans	1
11	Austria	2004	Beer	297
11	Austria	2004	Beverages, Alcoholic	15
11	Austria	2004	Bovine Meat	50
11	Austria	2004	Butter, Ghee	14
11	Austria	2004	Cheese	56
11	Austria	2004	Citrus, Other	6
11	Austria	2004	Cocoa Beans	8
11	Austria	2004	Coconut Oil	2
11	Austria	2004	Coconuts - Incl Copra	2
11	Austria	2004	Coffee	21
11	Austria	2004	Cream	7
11	Austria	2004	Crustaceans	3
11	Austria	2004	Demersal Fish	7
11	Austria	2004	Eggs	35
11	Austria	2004	Fats, Animals, Raw	21
11	Austria	2004	Freshwater Fish	8
11	Austria	2004	Fruits, Other	128
11	Austria	2004	Grapefruit	4
11	Austria	2004	Grapes	33
11	Austria	2004	Groundnuts (Shelled Eq)	2
11	Austria	2004	Honey	3
11	Austria	2004	Lemons, Limes	7
11	Austria	2004	Maize	34
11	Austria	2004	Maize Germ Oil	2
11	Austria	2004	Marine Fish, Other	2
11	Austria	2004	Meat, Other	3
11	Austria	2004	Milk, Whole	45
11	Austria	2004	Molluscs, Other	2
11	Austria	2004	Mutton & Goat Meat	3
11	Austria	2004	Nuts	19
11	Austria	2004	Oats	4
11	Austria	2004	Offals, Edible	2
11	Austria	2004	Oilcrops Oil, Other	10
11	Austria	2004	Oilcrops, Other	1
11	Austria	2004	Olive Oil	2
11	Austria	2004	Olives	1
11	Austria	2004	Onions	27
11	Austria	2004	Oranges, Mandarines	44
11	Austria	2004	Palm Oil	2
11	Austria	2004	Peas	1
11	Austria	2004	Pelagic Fish	11
11	Austria	2004	Pepper	1
11	Austria	2004	Pigmeat	203
11	Austria	2004	Pimento	1
11	Austria	2004	Pineapples	6
11	Austria	2004	Potatoes	164
11	Austria	2004	Poultry Meat	49
11	Austria	2004	Rape and Mustard Oil	27
11	Austria	2004	Rice (Milled Equivalent)	8
11	Austria	2004	Rye	37
11	Austria	2004	Sesameseed	1
11	Austria	2004	Soyabean Oil	11
11	Austria	2004	Soyabeans	2
11	Austria	2004	Spices, Other	1
11	Austria	2004	Sugar, Refined Equivalent	107
11	Austria	2004	Sunflowerseed	1
11	Austria	2004	Sunflowerseed Oil	5
11	Austria	2004	Sweeteners, Other	11
11	Austria	2004	Tomatoes	40
11	Austria	2004	Vegetables, Other	213
11	Austria	2004	Wheat	222
11	Austria	2004	Wine	82
11	Austria	2005	Apples	146
11	Austria	2005	Bananas	27
11	Austria	2005	Barley	1
11	Austria	2005	Beans	1
11	Austria	2005	Beer	299
11	Austria	2005	Beverages, Alcoholic	13
11	Austria	2005	Beverages, Fermented	1
11	Austria	2005	Bovine Meat	49
11	Austria	2005	Butter, Ghee	14
11	Austria	2005	Cheese	57
11	Austria	2005	Citrus, Other	7
11	Austria	2005	Cocoa Beans	4
11	Austria	2005	Coconut Oil	1
11	Austria	2005	Coconuts - Incl Copra	2
11	Austria	2005	Coffee	14
11	Austria	2005	Cream	7
11	Austria	2005	Crustaceans	4
11	Austria	2005	Demersal Fish	6
11	Austria	2005	Eggs	36
11	Austria	2005	Fats, Animals, Raw	28
11	Austria	2005	Freshwater Fish	10
11	Austria	2005	Fruits, Other	118
11	Austria	2005	Grapefruit	4
11	Austria	2005	Grapes	21
11	Austria	2005	Groundnuts (Shelled Eq)	3
11	Austria	2005	Honey	3
11	Austria	2005	Lemons, Limes	8
11	Austria	2005	Maize	32
11	Austria	2005	Maize Germ Oil	1
11	Austria	2005	Marine Fish, Other	2
11	Austria	2005	Meat, Other	3
11	Austria	2005	Milk, Whole	53
11	Austria	2005	Molluscs, Other	2

FAO country code	Country	Reference year	Food item	Gross food supply (g *capita*$^{-1}$ d^{-1})
11	Austria	2005	Mutton & Goat Meat	3
11	Austria	2005	Nuts	17
11	Austria	2005	Oats	4
11	Austria	2005	Offals, Edible	4
11	Austria	2005	Oilcrops Oil, Other	6
11	Austria	2005	Oilcrops, Other	1
11	Austria	2005	Olive Oil	2
11	Austria	2005	Olives	1
11	Austria	2005	Onions	20
11	Austria	2005	Oranges, Mandarines	54
11	Austria	2005	Palm Oil	2
11	Austria	2005	Peas	1
11	Austria	2005	Pelagic Fish	13
11	Austria	2005	Pepper	1
11	Austria	2005	Pigmeat	197
11	Austria	2005	Pimento	1
11	Austria	2005	Pineapples	8
11	Austria	2005	Potatoes	163
11	Austria	2005	Poultry Meat	51
11	Austria	2005	Rape and Mustard Oil	17
11	Austria	2005	Rice (Milled Equivalent)	9
11	Austria	2005	Rye	37
11	Austria	2005	Sesameseed	1
11	Austria	2005	Soyabean Oil	10
11	Austria	2005	Soyabeans	3
11	Austria	2005	Spices, Other	1
11	Austria	2005	Sugar, Refined Equivalent	110
11	Austria	2005	Sunflowerseed	1
11	Austria	2005	Sunflowerseed Oil	9
11	Austria	2005	Sweeteners, Other	13
11	Austria	2005	Tea	5
11	Austria	2005	Tomatoes	41
11	Austria	2005	Vegetables, Other	195
11	Austria	2005	Wheat	244
11	Austria	2005	Wine	88
11	Austria	2006	Apples	136
11	Austria	2006	Bananas	36
11	Austria	2006	Barley	1
11	Austria	2006	Beer	297
11	Austria	2006	Beverages, Alcoholic	13
11	Austria	2006	Beverages, Fermented	1
11	Austria	2006	Bovine Meat	50
11	Austria	2006	Butter, Ghee	15
11	Austria	2006	Cheese	55
11	Austria	2006	Citrus, Other	5
11	Austria	2006	Cocoa Beans	5
11	Austria	2006	Coconut Oil	1
11	Austria	2006	Coconuts - Incl Copra	2
11	Austria	2006	Coffee	14
11	Austria	2006	Cream	11
11	Austria	2006	Crustaceans	3
11	Austria	2006	Demersal Fish	8
11	Austria	2006	Eggs	37
11	Austria	2006	Fats, Animals, Raw	25
11	Austria	2006	Freshwater Fish	8
11	Austria	2006	Fruits, Other	122
11	Austria	2006	Grapefruit	2
11	Austria	2006	Grapes	22
11	Austria	2006	Groundnuts (Shelled Eq)	2
11	Austria	2006	Honey	3
11	Austria	2006	Lemons, Limes	8
11	Austria	2006	Maize	35
11	Austria	2006	Maize Germ Oil	2
11	Austria	2006	Marine Fish, Other	3
11	Austria	2006	Meat, Other	3
11	Austria	2006	Milk, Whole	49
11	Austria	2006	Molluscs, Other	2
11	Austria	2006	Mutton & Goat Meat	3
11	Austria	2006	Nuts	17
11	Austria	2006	Oats	4
11	Austria	2006	Offals, Edible	3
11	Austria	2006	Oilcrops Oil, Other	5
11	Austria	2006	Oilcrops, Other	2
11	Austria	2006	Olive Oil	2
11	Austria	2006	Olives	1
11	Austria	2006	Onions	21
11	Austria	2006	Oranges, Mandarines	56
11	Austria	2006	Palm Oil	2
11	Austria	2006	Peas	1
11	Austria	2006	Pelagic Fish	12
11	Austria	2006	Pepper	1
11	Austria	2006	Pigmeat	192
11	Austria	2006	Pimento	1
11	Austria	2006	Pineapples	8
11	Austria	2006	Potatoes	145
11	Austria	2006	Poultry Meat	47
11	Austria	2006	Rape and Mustard Oil	31
11	Austria	2006	Rice (Milled Equivalent)	8
11	Austria	2006	Rye	35
11	Austria	2006	Sesameseed	1
11	Austria	2006	Soyabean Oil	10
11	Austria	2006	Soyabeans	3
11	Austria	2006	Spices, Other	1
11	Austria	2006	Sugar, Refined Equivalent	106
11	Austria	2006	Sunflowerseed	1
11	Austria	2006	Sunflowerseed Oil	11
11	Austria	2006	Sweeteners, Other	15

FAO country code	Country	Reference year	Food item	Gross food supply (g $capita^{-1}$ d^{-1})
11	Austria	2006	Tea	4
11	Austria	2006	Tomatoes	44
11	Austria	2006	Vegetables, Other	195
11	Austria	2006	Wheat	237
11	Austria	2006	Wine	89
11	Austria	2007	Apples	172
11	Austria	2007	Bananas	27
11	Austria	2007	Barley	1
11	Austria	2007	Beer	296
11	Austria	2007	Beverages, Alcoholic	16
11	Austria	2007	Bovine Meat	49
11	Austria	2007	Butter, Ghee	15
11	Austria	2007	Cheese	59
11	Austria	2007	Citrus, Other	3
11	Austria	2007	Cocoa Beans	3
11	Austria	2007	Coconut Oil	2
11	Austria	2007	Coconuts - Incl Copra	2
11	Austria	2007	Coffee	24
11	Austria	2007	Cottonseed Oil	1
11	Austria	2007	Cream	17
11	Austria	2007	Crustaceans	3
11	Austria	2007	Dates	1
11	Austria	2007	Demersal Fish	10
11	Austria	2007	Eggs	38
11	Austria	2007	Fats, Animals, Raw	26
11	Austria	2007	Freshwater Fish	10
11	Austria	2007	Fruits, Other	127
11	Austria	2007	Grapefruit	3
11	Austria	2007	Grapes	22
11	Austria	2007	Groundnuts (Shelled Eq)	2
11	Austria	2007	Honey	3
11	Austria	2007	Lemons, Limes	8
11	Austria	2007	Maize	32
11	Austria	2007	Maize Germ Oil	4
11	Austria	2007	Marine Fish, Other	3
11	Austria	2007	Meat, Other	2
11	Austria	2007	Milk, Whole	45
11	Austria	2007	Molluscs, Other	2
11	Austria	2007	Mutton & Goat Meat	3
11	Austria	2007	Nuts	18
11	Austria	2007	Oats	4
11	Austria	2007	Offals, Edible	3
11	Austria	2007	Oilcrops Oil, Other	3
11	Austria	2007	Oilcrops, Other	2
11	Austria	2007	Olive Oil	3
11	Austria	2007	Olives	2
11	Austria	2007	Onions	22
11	Austria	2007	Oranges, Mandarines	38
11	Austria	2007	Palm Oil	3
11	Austria	2007	Peas	1
11	Austria	2007	Pelagic Fish	13
11	Austria	2007	Pepper	1
11	Austria	2007	Pigmeat	198
11	Austria	2007	Pimento	1
11	Austria	2007	Pineapples	9
11	Austria	2007	Potatoes	153
11	Austria	2007	Poultry Meat	48
11	Austria	2007	Rape and Mustard Oil	20
11	Austria	2007	Rice (Milled Equivalent)	10
11	Austria	2007	Rye	37
11	Austria	2007	Sesameseed	1
11	Austria	2007	Soyabean Oil	10
11	Austria	2007	Soyabeans	4
11	Austria	2007	Spices, Other	1
11	Austria	2007	Sugar, Refined Equivalent	106
11	Austria	2007	Sunflowerseed	1
11	Austria	2007	Sunflowerseed Oil	15
11	Austria	2007	Sweeteners, Other	10
11	Austria	2007	Tea	3
11	Austria	2007	Tomatoes	49
11	Austria	2007	Vegetables, Other	191
11	Austria	2007	Wheat	242
11	Austria	2007	Wine	80
11	Austria	2008	Apples	184
11	Austria	2008	Bananas	31
11	Austria	2008	Barley	1
11	Austria	2008	Beer	285
11	Austria	2008	Beverages, Alcoholic	11
11	Austria	2008	Beverages, Fermented	1
11	Austria	2008	Bovine Meat	49
11	Austria	2008	Butter, Ghee	14
11	Austria	2008	Cheese	61
11	Austria	2008	Citrus, Other	4
11	Austria	2008	Cocoa Beans	2
11	Austria	2008	Coconut Oil	2
11	Austria	2008	Coconuts - Incl Copra	2
11	Austria	2008	Coffee	22
11	Austria	2008	Cream	16
11	Austria	2008	Crustaceans	3
11	Austria	2008	Demersal Fish	8
11	Austria	2008	Eggs	38
11	Austria	2008	Fats, Animals, Raw	24
11	Austria	2008	Freshwater Fish	10
11	Austria	2008	Fruits, Other	127
11	Austria	2008	Grapefruit	2
11	Austria	2008	Grapes	22
11	Austria	2008	Groundnuts (Shelled Eq)	2

FAO country code	Country	Reference year	Food item	Gross food supply (g $capita^{-1}$ d^{-1})
11	Austria	2008	Honey	3
11	Austria	2008	Lemons, Limes	8
11	Austria	2008	Maize	31
11	Austria	2008	Maize Germ Oil	2
11	Austria	2008	Marine Fish, Other	3
11	Austria	2008	Meat, Other	1
11	Austria	2008	Milk, Whole	46
11	Austria	2008	Molluscs, Other	2
11	Austria	2008	Mutton & Goat Meat	3
11	Austria	2008	Nuts	19
11	Austria	2008	Oats	4
11	Austria	2008	Offals, Edible	2
11	Austria	2008	Oilcrops Oil, Other	4
11	Austria	2008	Oilcrops, Other	1
11	Austria	2008	Olive Oil	3
11	Austria	2008	Olives	2
11	Austria	2008	Onions	32
11	Austria	2008	Oranges, Mandarines	42
11	Austria	2008	Palm Oil	3
11	Austria	2008	Peas	1
11	Austria	2008	Pelagic Fish	13
11	Austria	2008	Pigmeat	189
11	Austria	2008	Pimento	1
11	Austria	2008	Pineapples	10
11	Austria	2008	Potatoes	157
11	Austria	2008	Poultry Meat	47
11	Austria	2008	Pulses, Other	1
11	Austria	2008	Rape and Mustard Oil	24
11	Austria	2008	Rice (Milled Equivalent)	11
11	Austria	2008	Rye	34
11	Austria	2008	Sesameseed	1
11	Austria	2008	Soyabean Oil	9
11	Austria	2008	Soyabeans	4
11	Austria	2008	Spices, Other	1
11	Austria	2008	Sugar, Refined Equivalent	106
11	Austria	2008	Sunflowerseed	1
11	Austria	2008	Sunflowerseed Oil	16
11	Austria	2008	Sweeteners, Other	15
11	Austria	2008	Tea	1
11	Austria	2008	Tomatoes	49
11	Austria	2008	Vegetables, Other	197
11	Austria	2008	Wheat	234
11	Austria	2008	Wine	81
11	Austria	2009	Apples	122
11	Austria	2009	Bananas	31
11	Austria	2009	Barley	1
11	Austria	2009	Beans	1
11	Austria	2009	Beer	287
11	Austria	2009	Beverages, Alcoholic	14
11	Austria	2009	Beverages, Fermented	1
11	Austria	2009	Bovine Meat	48
11	Austria	2009	Butter, Ghee	15
11	Austria	2009	Cheese	59
11	Austria	2009	Citrus, Other	3
11	Austria	2009	Cocoa Beans	4
11	Austria	2009	Coconut Oil	2
11	Austria	2009	Coconuts - Incl Copra	2
11	Austria	2009	Coffee	18
11	Austria	2009	Cream	17
11	Austria	2009	Crustaceans	4
11	Austria	2009	Demersal Fish	7
11	Austria	2009	Eggs	37
11	Austria	2009	Fats, Animals, Raw	28
11	Austria	2009	Freshwater Fish	11
11	Austria	2009	Fruits, Other	133
11	Austria	2009	Grapefruit	2
11	Austria	2009	Grapes	22
11	Austria	2009	Groundnuts (Shelled Eq)	2
11	Austria	2009	Honey	3
11	Austria	2009	Lemons, Limes	9
11	Austria	2009	Maize	37
11	Austria	2009	Maize Germ Oil	2
11	Austria	2009	Marine Fish, Other	2
11	Austria	2009	Meat, Other	2
11	Austria	2009	Milk, Whole	47
11	Austria	2009	Molluscs, Other	2
11	Austria	2009	Mutton & Goat Meat	3
11	Austria	2009	Nuts	19
11	Austria	2009	Oats	5
11	Austria	2009	Offals, Edible	3
11	Austria	2009	Oilcrops Oil, Other	9
11	Austria	2009	Oilcrops, Other	2
11	Austria	2009	Olive Oil	3
11	Austria	2009	Olives	2
11	Austria	2009	Onions	32
11	Austria	2009	Oranges, Mandarines	28
11	Austria	2009	Palm Oil	2
11	Austria	2009	Peas	1
11	Austria	2009	Pelagic Fish	12
11	Austria	2009	Pigmeat	194
11	Austria	2009	Pimento	1
11	Austria	2009	Pineapples	7
11	Austria	2009	Potatoes	157
11	Austria	2009	Poultry Meat	48
11	Austria	2009	Pulses, Other	1
11	Austria	2009	Rape and Mustard Oil	29
11	Austria	2009	Rice (Milled Equivalent)	10

FAO country code	Country	Reference year	Food item	Gross food supply (g *capita*$^{-1}$ d^{-1})
11	Austria	2009	Rye	35
11	Austria	2009	Sesameseed	1
11	Austria	2009	Soyabean Oil	4
11	Austria	2009	Soyabeans	5
11	Austria	2009	Spices, Other	1
11	Austria	2009	Sugar, Refined Equivalent	103
11	Austria	2009	Sunflowerseed	1
11	Austria	2009	Sunflowerseed Oil	13
11	Austria	2009	Sweeteners, Other	13
11	Austria	2009	Tea	1
11	Austria	2009	Tomatoes	44
11	Austria	2009	Vegetables, Other	201
11	Austria	2009	Wheat	220
11	Austria	2009	Wine	88
11	Austria	2010	Apples	131
11	Austria	2010	Bananas	34
11	Austria	2010	Barley	1
11	Austria	2010	Beer	286
11	Austria	2010	Beverages, Alcoholic	12
11	Austria	2010	Beverages, Fermented	1
11	Austria	2010	Bovine Meat	48
11	Austria	2010	Butter, Ghee	15
11	Austria	2010	Cheese	59
11	Austria	2010	Citrus, Other	3
11	Austria	2010	Cocoa Beans	2
11	Austria	2010	Coconut Oil	3
11	Austria	2010	Coconuts - Incl Copra	2
11	Austria	2010	Coffee	17
11	Austria	2010	Cream	18
11	Austria	2010	Crustaceans	3
11	Austria	2010	Demersal Fish	7
11	Austria	2010	Eggs	37
11	Austria	2010	Fats, Animals, Raw	31
11	Austria	2010	Freshwater Fish	11
11	Austria	2010	Fruits, Other	106
11	Austria	2010	Grapefruit	2
11	Austria	2010	Grapes	21
11	Austria	2010	Groundnuts (Shelled Eq)	2
11	Austria	2010	Honey	3
11	Austria	2010	Lemons, Limes	8
11	Austria	2010	Maize	39
11	Austria	2010	Maize Germ Oil	2
11	Austria	2010	Marine Fish, Other	2
11	Austria	2010	Meat, Other	3
11	Austria	2010	Milk, Whole	43
11	Austria	2010	Molluscs, Other	2
11	Austria	2010	Mutton & Goat Meat	3
11	Austria	2010	Nuts	16
11	Austria	2010	Oats	5
11	Austria	2010	Offals, Edible	2
11	Austria	2010	Oilcrops Oil, Other	6
11	Austria	2010	Oilcrops, Other	2
11	Austria	2010	Olive Oil	3
11	Austria	2010	Olives	2
11	Austria	2010	Onions	40
11	Austria	2010	Oranges, Mandarines	27
11	Austria	2010	Palm Oil	2
11	Austria	2010	Peas	1
11	Austria	2010	Pelagic Fish	11
11	Austria	2010	Pigmeat	190
11	Austria	2010	Pimento	1
11	Austria	2010	Pineapples	7
11	Austria	2010	Potatoes	156
11	Austria	2010	Poultry Meat	52
11	Austria	2010	Rape and Mustard Oil	33
11	Austria	2010	Rice (Milled Equivalent)	9
11	Austria	2010	Rye	34
11	Austria	2010	Sesameseed	1
11	Austria	2010	Soyabean Oil	4
11	Austria	2010	Soyabeans	5
11	Austria	2010	Spices, Other	1
11	Austria	2010	Sugar, Refined Equivalent	103
11	Austria	2010	Sunflowerseed	2
11	Austria	2010	Sunflowerseed Oil	12
11	Austria	2010	Sweeteners, Other	17
11	Austria	2010	Tomatoes	50
11	Austria	2010	Vegetables, Other	200
11	Austria	2010	Wheat	212
11	Austria	2010	Wine	84
11	Austria	2011	Apples	145
11	Austria	2011	Bananas	32
11	Austria	2011	Barley	1
11	Austria	2011	Beans	1
11	Austria	2011	Beer	293
11	Austria	2011	Beverages, Alcoholic	12
11	Austria	2011	Beverages, Fermented	1
11	Austria	2011	Bovine Meat	48
11	Austria	2011	Butter, Ghee	15
11	Austria	2011	Cheese	60
11	Austria	2011	Citrus, Other	2
11	Austria	2011	Cocoa Beans	2
11	Austria	2011	Coconut Oil	3
11	Austria	2011	Coconuts - Incl Copra	2
11	Austria	2011	Coffee	22
11	Austria	2011	Cream	21
11	Austria	2011	Crustaceans	3
11	Austria	2011	Demersal Fish	7

FAO countrycode	Country	Reference year	Food item	Gross food supply (g *capita*$^{-1}$ d^{-1})
11	Austria	2011	Eggs	38
11	Austria	2011	Fats, Animals, Raw	31
11	Austria	2011	Freshwater Fish	11
11	Austria	2011	Fruits, Other	123
11	Austria	2011	Grapefruit	2
11	Austria	2011	Grapes	22
11	Austria	2011	Groundnuts (Shelled Eq)	2
11	Austria	2011	Honey	3
11	Austria	2011	Lemons, Limes	9
11	Austria	2011	Maize	34
11	Austria	2011	Maize Germ Oil	4
11	Austria	2011	Marine Fish, Other	2
11	Austria	2011	Meat, Other	3
11	Austria	2011	Milk, Whole	42
11	Austria	2011	Molluscs, Other	2
11	Austria	2011	Mutton & Goat Meat	3
11	Austria	2011	Nuts	16
11	Austria	2011	Oats	5
11	Austria	2011	Offals, Edible	2
11	Austria	2011	Oilcrops Oil, Other	6
11	Austria	2011	Oilcrops, Other	2
11	Austria	2011	Olive Oil	3
11	Austria	2011	Olives	2
11	Austria	2011	Onions	46
11	Austria	2011	Oranges, Mandarines	59
11	Austria	2011	Palm Oil	2
11	Austria	2011	Peas	1
11	Austria	2011	Pelagic Fish	11
11	Austria	2011	Pigmeat	185
11	Austria	2011	Pimento	1
11	Austria	2011	Pineapples	6
11	Austria	2011	Potatoes	160
11	Austria	2011	Poultry Meat	53
11	Austria	2011	Rape and Mustard Oil	25
11	Austria	2011	Rice (Milled Equivalent)	10
11	Austria	2011	Rye	35
11	Austria	2011	Sesameseed	1
11	Austria	2011	Soyabean Oil	4
11	Austria	2011	Soyabeans	5
11	Austria	2011	Spices, Other	1
11	Austria	2011	Sugar, Refined Equivalent	104
11	Austria	2011	Sunflowerseed	2
11	Austria	2011	Sunflowerseed Oil	13
11	Austria	2011	Sweeteners, Other	15
11	Austria	2011	Tea	1
11	Austria	2011	Tomatoes	53
11	Austria	2011	Vegetables, Other	200
11	Austria	2011	Wheat	222
11	Austria	2011	Wine	84
52	Azerbaijan	1992	Apples	38
52	Azerbaijan	1992	Barley	50
52	Azerbaijan	1992	Beer	8
52	Azerbaijan	1992	Beverages, Alcoholic	20
52	Azerbaijan	1992	Bovine Meat	25
52	Azerbaijan	1992	Butter, Ghee	7
52	Azerbaijan	1992	Cereals, Other	1
52	Azerbaijan	1992	Cheese	15
52	Azerbaijan	1992	Cottonseed Oil	4
52	Azerbaijan	1992	Eggs	16
52	Azerbaijan	1992	Fats, Animals, Raw	1
52	Azerbaijan	1992	Freshwater Fish	12
52	Azerbaijan	1992	Fruits, Other	77
52	Azerbaijan	1992	Grapes	116
52	Azerbaijan	1992	Milk, Whole	151
52	Azerbaijan	1992	Mutton & Goat Meat	10
52	Azerbaijan	1992	Nuts	8
52	Azerbaijan	1992	Offals, Edible	5
52	Azerbaijan	1992	Onions	19
52	Azerbaijan	1992	Oranges, Mandarines	11
52	Azerbaijan	1992	Pigmeat	2
52	Azerbaijan	1992	Potatoes	71
52	Azerbaijan	1992	Poultry Meat	13
52	Azerbaijan	1992	Rice (Milled Equivalent)	6
52	Azerbaijan	1992	Sugar, Refined Equivalent	34
52	Azerbaijan	1992	Tea	2
52	Azerbaijan	1992	Tomatoes	72
52	Azerbaijan	1992	Vegetables, Other	60
52	Azerbaijan	1992	Wheat	503
52	Azerbaijan	1992	Wine	1
52	Azerbaijan	1993	Apples	33
52	Azerbaijan	1993	Barley	34
52	Azerbaijan	1993	Beer	6
52	Azerbaijan	1993	Beverages, Alcoholic	12
52	Azerbaijan	1993	Bovine Meat	23
52	Azerbaijan	1993	Butter, Ghee	7
52	Azerbaijan	1993	Cereals, Other	1
52	Azerbaijan	1993	Cheese	13
52	Azerbaijan	1993	Cottonseed Oil	2
52	Azerbaijan	1993	Eggs	11
52	Azerbaijan	1993	Fats, Animals, Raw	1
52	Azerbaijan	1993	Freshwater Fish	8
52	Azerbaijan	1993	Fruits, Other	65
52	Azerbaijan	1993	Grapes	94
52	Azerbaijan	1993	Milk, Whole	152
52	Azerbaijan	1993	Mutton & Goat Meat	9
52	Azerbaijan	1993	Nuts	9
52	Azerbaijan	1993	Offals, Edible	4

FAO countrycode	Country	Reference year	Food item	Gross food supply (g *capita*$^{-1}$ d^{-1})
52	Azerbaijan	1993	Onions	21
52	Azerbaijan	1993	Oranges, Mandarines	12
52	Azerbaijan	1993	Pigmeat	1
52	Azerbaijan	1993	Potatoes	69
52	Azerbaijan	1993	Poultry Meat	9
52	Azerbaijan	1993	Rice (Milled Equivalent)	7
52	Azerbaijan	1993	Sugar, Refined Equivalent	28
52	Azerbaijan	1993	Tea	3
52	Azerbaijan	1993	Tomatoes	76
52	Azerbaijan	1993	Vegetables, Other	67
52	Azerbaijan	1993	Wheat	523
52	Azerbaijan	1993	Wine	2
52	Azerbaijan	1994	Apples	46
52	Azerbaijan	1994	Barley	27
52	Azerbaijan	1994	Beer	8
52	Azerbaijan	1994	Beverages, Alcoholic	5
52	Azerbaijan	1994	Bovine Meat	15
52	Azerbaijan	1994	Butter, Ghee	7
52	Azerbaijan	1994	Cheese	12
52	Azerbaijan	1994	Cottonseed Oil	2
52	Azerbaijan	1994	Dates	1
52	Azerbaijan	1994	Eggs	12
52	Azerbaijan	1994	Fats, Animals, Raw	4
52	Azerbaijan	1994	Freshwater Fish	6
52	Azerbaijan	1994	Fruits, Other	59
52	Azerbaijan	1994	Grapes	82
52	Azerbaijan	1994	Lemons, Limes	14
52	Azerbaijan	1994	Milk, Whole	170
52	Azerbaijan	1994	Mutton & Goat Meat	8
52	Azerbaijan	1994	Nuts	9
52	Azerbaijan	1994	Offals, Edible	1
52	Azerbaijan	1994	Onions	20
52	Azerbaijan	1994	Oranges, Mandarines	15
52	Azerbaijan	1994	Pigmeat	1
52	Azerbaijan	1994	Potatoes	63
52	Azerbaijan	1994	Poultry Meat	8
52	Azerbaijan	1994	Rice (Milled Equivalent)	3
52	Azerbaijan	1994	Soyabean Oil	1
52	Azerbaijan	1994	Sugar, Refined Equivalent	29
52	Azerbaijan	1994	Sunflowerseed Oil	2
52	Azerbaijan	1994	Sweeteners, Other	1
52	Azerbaijan	1994	Tea	2
52	Azerbaijan	1994	Tomatoes	63
52	Azerbaijan	1994	Vegetables, Other	73
52	Azerbaijan	1994	Wheat	488
52	Azerbaijan	1994	Wine	2
52	Azerbaijan	1995	Apples	47
52	Azerbaijan	1995	Barley	55
52	Azerbaijan	1995	Beer	9
52	Azerbaijan	1995	Beverages, Alcoholic	4
52	Azerbaijan	1995	Bovine Meat	18
52	Azerbaijan	1995	Butter, Ghee	5
52	Azerbaijan	1995	Cereals, Other	1
52	Azerbaijan	1995	Cheese	11
52	Azerbaijan	1995	Cocoa Beans	1
52	Azerbaijan	1995	Cottonseed Oil	2
52	Azerbaijan	1995	Eggs	12
52	Azerbaijan	1995	Fats, Animals, Raw	1
52	Azerbaijan	1995	Freshwater Fish	4
52	Azerbaijan	1995	Fruits, Other	87
52	Azerbaijan	1995	Grapes	78
52	Azerbaijan	1995	Milk, Whole	167
52	Azerbaijan	1995	Mutton & Goat Meat	8
52	Azerbaijan	1995	Nuts	7
52	Azerbaijan	1995	Offals, Edible	4
52	Azerbaijan	1995	Onions	15
52	Azerbaijan	1995	Oranges, Mandarines	12
52	Azerbaijan	1995	Pigmeat	1
52	Azerbaijan	1995	Potatoes	63
52	Azerbaijan	1995	Poultry Meat	10
52	Azerbaijan	1995	Rice (Milled Equivalent)	8
52	Azerbaijan	1995	Soyabean Oil	3
52	Azerbaijan	1995	Sugar, Refined Equivalent	32
52	Azerbaijan	1995	Sunflowerseed Oil	1
52	Azerbaijan	1995	Tea	2
52	Azerbaijan	1995	Tomatoes	61
52	Azerbaijan	1995	Vegetables, Other	82
52	Azerbaijan	1995	Wheat	456
52	Azerbaijan	1995	Wine	2
52	Azerbaijan	1996	Apples	36
52	Azerbaijan	1996	Barley	75
52	Azerbaijan	1996	Beer	7
52	Azerbaijan	1996	Beverages, Alcoholic	11
52	Azerbaijan	1996	Bovine Meat	17
52	Azerbaijan	1996	Butter, Ghee	11
52	Azerbaijan	1996	Cereals, Other	1
52	Azerbaijan	1996	Cheese	11
52	Azerbaijan	1996	Citrus, Other	3
52	Azerbaijan	1996	Cocoa Beans	1
52	Azerbaijan	1996	Cottonseed Oil	4
52	Azerbaijan	1996	Eggs	12
52	Azerbaijan	1996	Fats, Animals, Raw	1
52	Azerbaijan	1996	Freshwater Fish	2
52	Azerbaijan	1996	Fruits, Other	64
52	Azerbaijan	1996	Grapes	80
52	Azerbaijan	1996	Milk, Whole	110
52	Azerbaijan	1996	Mutton & Goat Meat	11

FAO countrycode	Country	Reference year	Food item	Gross food supply (g *capita*$^{-1}$ d^{-1})
52	Azerbaijan	1996	Nuts	6
52	Azerbaijan	1996	Offals, Edible	1
52	Azerbaijan	1996	Onions	25
52	Azerbaijan	1996	Oranges, Mandarines	14
52	Azerbaijan	1996	Pigmeat	2
52	Azerbaijan	1996	Potatoes	69
52	Azerbaijan	1996	Poultry Meat	14
52	Azerbaijan	1996	Rice (Milled Equivalent)	17
52	Azerbaijan	1996	Soyabean Oil	2
52	Azerbaijan	1996	Sugar, Refined Equivalent	35
52	Azerbaijan	1996	Sunflowerseed Oil	1
52	Azerbaijan	1996	Tea	4
52	Azerbaijan	1996	Tomatoes	83
52	Azerbaijan	1996	Vegetables, Other	103
52	Azerbaijan	1996	Wheat	460
52	Azerbaijan	1996	Wine	2
52	Azerbaijan	1997	Apples	31
52	Azerbaijan	1997	Bananas	1
52	Azerbaijan	1997	Barley	23
52	Azerbaijan	1997	Beer	6
52	Azerbaijan	1997	Beverages, Alcoholic	1
52	Azerbaijan	1997	Bovine Meat	17
52	Azerbaijan	1997	Butter, Ghee	6
52	Azerbaijan	1997	Cereals, Other	1
52	Azerbaijan	1997	Cheese	9
52	Azerbaijan	1997	Cottonseed Oil	2
52	Azerbaijan	1997	Dates	1
52	Azerbaijan	1997	Eggs	12
52	Azerbaijan	1997	Fats, Animals, Raw	1
52	Azerbaijan	1997	Freshwater Fish	1
52	Azerbaijan	1997	Fruits, Other	58
52	Azerbaijan	1997	Grapes	23
52	Azerbaijan	1997	Milk, Whole	157
52	Azerbaijan	1997	Mutton & Goat Meat	9
52	Azerbaijan	1997	Nuts	5
52	Azerbaijan	1997	Offals, Edible	1
52	Azerbaijan	1997	Onions	20
52	Azerbaijan	1997	Oranges, Mandarines	9
52	Azerbaijan	1997	Pigmeat	2
52	Azerbaijan	1997	Potatoes	75
52	Azerbaijan	1997	Poultry Meat	14
52	Azerbaijan	1997	Rice (Milled Equivalent)	15
52	Azerbaijan	1997	Soyabean Oil	1
52	Azerbaijan	1997	Sugar, Refined Equivalent	38
52	Azerbaijan	1997	Sunflowerseed Oil	1
52	Azerbaijan	1997	Tea	2
52	Azerbaijan	1997	Tomatoes	68
52	Azerbaijan	1997	Vegetables, Other	108
52	Azerbaijan	1997	Wheat	482
52	Azerbaijan	1997	Wine	2
52	Azerbaijan	1998	Apples	35
52	Azerbaijan	1998	Bananas	2
52	Azerbaijan	1998	Barley	51
52	Azerbaijan	1998	Beer	4
52	Azerbaijan	1998	Beverages, Alcoholic	3
52	Azerbaijan	1998	Bovine Meat	16
52	Azerbaijan	1998	Butter, Ghee	7
52	Azerbaijan	1998	Cereals, Other	1
52	Azerbaijan	1998	Cheese	13
52	Azerbaijan	1998	Cottonseed Oil	1
52	Azerbaijan	1998	Dates	1
52	Azerbaijan	1998	Demersal Fish	1
52	Azerbaijan	1998	Eggs	12
52	Azerbaijan	1998	Fats, Animals, Raw	2
52	Azerbaijan	1998	Freshwater Fish	2
52	Azerbaijan	1998	Fruits, Other	77
52	Azerbaijan	1998	Grapes	41
52	Azerbaijan	1998	Lemons, Limes	2
52	Azerbaijan	1998	Maize	1
52	Azerbaijan	1998	Milk, Whole	123
52	Azerbaijan	1998	Mutton & Goat Meat	11
52	Azerbaijan	1998	Nuts	4
52	Azerbaijan	1998	Offals, Edible	4
52	Azerbaijan	1998	Oilcrops Oil, Other	1
52	Azerbaijan	1998	Onions	22
52	Azerbaijan	1998	Oranges, Mandarines	5
52	Azerbaijan	1998	Pigmeat	3
52	Azerbaijan	1998	Potatoes	86
52	Azerbaijan	1998	Poultry Meat	21
52	Azerbaijan	1998	Rice (Milled Equivalent)	18
52	Azerbaijan	1998	Soyabean Oil	1
52	Azerbaijan	1998	Sugar, Refined Equivalent	37
52	Azerbaijan	1998	Sunflowerseed Oil	1
52	Azerbaijan	1998	Tea	2
52	Azerbaijan	1998	Tomatoes	67
52	Azerbaijan	1998	Vegetables, Other	98
52	Azerbaijan	1998	Wheat	452
52	Azerbaijan	1998	Wine	1
52	Azerbaijan	1999	Apples	39
52	Azerbaijan	1999	Bananas	1
52	Azerbaijan	1999	Barley	30
52	Azerbaijan	1999	Beer	24
52	Azerbaijan	1999	Beverages, Alcoholic	16
52	Azerbaijan	1999	Bovine Meat	18
52	Azerbaijan	1999	Butter, Ghee	7
52	Azerbaijan	1999	Cereals, Other	2
52	Azerbaijan	1999	Cheese	13

FAO country code	Country	Reference year	Food item	Gross food supply (g *capita*$^{-1}$ d^{-1})
52	Azerbaijan	1999	Cottonseed Oil	2
52	Azerbaijan	1999	Eggs	12
52	Azerbaijan	1999	Fats, Animals, Raw	2
52	Azerbaijan	1999	Freshwater Fish	6
52	Azerbaijan	1999	Fruits, Other	84
52	Azerbaijan	1999	Grapes	35
52	Azerbaijan	1999	Lemons, Limes	2
52	Azerbaijan	1999	Maize	4
52	Azerbaijan	1999	Maize Germ Oil	1
52	Azerbaijan	1999	Milk, Whole	124
52	Azerbaijan	1999	Mutton & Goat Meat	12
52	Azerbaijan	1999	Nuts	5
52	Azerbaijan	1999	Offals, Edible	5
52	Azerbaijan	1999	Onions	27
52	Azerbaijan	1999	Oranges, Mandarines	8
52	Azerbaijan	1999	Pigmeat	2
52	Azerbaijan	1999	Potatoes	104
52	Azerbaijan	1999	Poultry Meat	15
52	Azerbaijan	1999	Rice (Milled Equivalent)	9
52	Azerbaijan	1999	Soyabean Oil	2
52	Azerbaijan	1999	Sugar, Refined Equivalent	29
52	Azerbaijan	1999	Sunflowerseed Oil	2
52	Azerbaijan	1999	Sweeteners, Other	2
52	Azerbaijan	1999	Tea	1
52	Azerbaijan	1999	Tomatoes	112
52	Azerbaijan	1999	Vegetables, Other	113
52	Azerbaijan	1999	Wheat	503
52	Azerbaijan	1999	Wine	2
52	Azerbaijan	2000	Apples	42
52	Azerbaijan	2000	Bananas	1
52	Azerbaijan	2000	Barley	3
52	Azerbaijan	2000	Beer	24
52	Azerbaijan	2000	Beverages, Alcoholic	28
52	Azerbaijan	2000	Bovine Meat	19
52	Azerbaijan	2000	Butter, Ghee	6
52	Azerbaijan	2000	Cereals, Other	1
52	Azerbaijan	2000	Cheese	14
52	Azerbaijan	2000	Cottonseed Oil	2
52	Azerbaijan	2000	Eggs	11
52	Azerbaijan	2000	Fats, Animals, Raw	2
52	Azerbaijan	2000	Freshwater Fish	6
52	Azerbaijan	2000	Fruits, Other	87
52	Azerbaijan	2000	Grapes	22
52	Azerbaijan	2000	Lemons, Limes	2
52	Azerbaijan	2000	Maize	17
52	Azerbaijan	2000	Milk, Whole	129
52	Azerbaijan	2000	Mutton & Goat Meat	12
52	Azerbaijan	2000	Nuts	5
52	Azerbaijan	2000	Offals, Edible	5
52	Azerbaijan	2000	Onions	31
52	Azerbaijan	2000	Oranges, Mandarines	5
52	Azerbaijan	2000	Pelagic Fish	1
52	Azerbaijan	2000	Pigmeat	2
52	Azerbaijan	2000	Potatoes	129
52	Azerbaijan	2000	Poultry Meat	12
52	Azerbaijan	2000	Rice (Milled Equivalent)	9
52	Azerbaijan	2000	Soyabean Oil	1
52	Azerbaijan	2000	Sugar, Refined Equivalent	27
52	Azerbaijan	2000	Sunflowerseed Oil	2
52	Azerbaijan	2000	Tea	1
52	Azerbaijan	2000	Tomatoes	112
52	Azerbaijan	2000	Vegetables, Other	129
52	Azerbaijan	2000	Wheat	523
52	Azerbaijan	2000	Wine	2
52	Azerbaijan	2001	Apples	44
52	Azerbaijan	2001	Barley	4
52	Azerbaijan	2001	Beer	39
52	Azerbaijan	2001	Beverages, Alcoholic	22
52	Azerbaijan	2001	Bovine Meat	19
52	Azerbaijan	2001	Butter, Ghee	7
52	Azerbaijan	2001	Cereals, Other	2
52	Azerbaijan	2001	Cheese	14
52	Azerbaijan	2001	Cocoa Beans	1
52	Azerbaijan	2001	Cottonseed Oil	1
52	Azerbaijan	2001	Eggs	11
52	Azerbaijan	2001	Fats, Animals, Raw	2
52	Azerbaijan	2001	Freshwater Fish	4
52	Azerbaijan	2001	Fruits, Other	92
52	Azerbaijan	2001	Grapes	19
52	Azerbaijan	2001	Lemons, Limes	2
52	Azerbaijan	2001	Maize	19
52	Azerbaijan	2001	Maize Germ Oil	1
52	Azerbaijan	2001	Milk, Whole	127
52	Azerbaijan	2001	Mutton & Goat Meat	12
52	Azerbaijan	2001	Nuts	6
52	Azerbaijan	2001	Offals, Edible	5
52	Azerbaijan	2001	Onions	39
52	Azerbaijan	2001	Oranges, Mandarines	6
52	Azerbaijan	2001	Pigmeat	2
52	Azerbaijan	2001	Potatoes	156
52	Azerbaijan	2001	Poultry Meat	10
52	Azerbaijan	2001	Rice (Milled Equivalent)	8
52	Azerbaijan	2001	Soyabean Oil	3
52	Azerbaijan	2001	Sugar, Refined Equivalent	32
52	Azerbaijan	2001	Sunflowerseed Oil	2
52	Azerbaijan	2001	Tea	2
52	Azerbaijan	2001	Tomatoes	133

FAO countrycode	Country	Reference year	Food item	Gross food supply (g *capita*$^{-1}$ d^{-1})
52	Azerbaijan	2001	Vegetables, Other	155
52	Azerbaijan	2001	Wheat	549
52	Azerbaijan	2001	Wine	2
52	Azerbaijan	2002	Apples	39
52	Azerbaijan	2002	Barley	3
52	Azerbaijan	2002	Beer	42
52	Azerbaijan	2002	Beverages, Alcoholic	17
52	Azerbaijan	2002	Bovine Meat	21
52	Azerbaijan	2002	Butter, Ghee	9
52	Azerbaijan	2002	Cereals, Other	2
52	Azerbaijan	2002	Cheese	14
52	Azerbaijan	2002	Cocoa Beans	1
52	Azerbaijan	2002	Cottonseed Oil	1
52	Azerbaijan	2002	Eggs	11
52	Azerbaijan	2002	Fats, Animals, Raw	1
52	Azerbaijan	2002	Freshwater Fish	4
52	Azerbaijan	2002	Fruits, Other	87
52	Azerbaijan	2002	Grapes	18
52	Azerbaijan	2002	Lemons, Limes	1
52	Azerbaijan	2002	Maize	21
52	Azerbaijan	2002	Milk, Whole	127
52	Azerbaijan	2002	Mutton & Goat Meat	12
52	Azerbaijan	2002	Nuts	5
52	Azerbaijan	2002	Offals, Edible	7
52	Azerbaijan	2002	Onions	43
52	Azerbaijan	2002	Oranges, Mandarines	6
52	Azerbaijan	2002	Pelagic Fish	1
52	Azerbaijan	2002	Pigmeat	2
52	Azerbaijan	2002	Potatoes	178
52	Azerbaijan	2002	Poultry Meat	9
52	Azerbaijan	2002	Rice (Milled Equivalent)	9
52	Azerbaijan	2002	Soyabean Oil	4
52	Azerbaijan	2002	Sugar, Refined Equivalent	39
52	Azerbaijan	2002	Sunflowerseed Oil	2
52	Azerbaijan	2002	Tea	1
52	Azerbaijan	2002	Tomatoes	135
52	Azerbaijan	2002	Vegetables, Other	198
52	Azerbaijan	2002	Wheat	567
52	Azerbaijan	2002	Wine	1
52	Azerbaijan	2003	Apples	34
52	Azerbaijan	2003	Bananas	1
52	Azerbaijan	2003	Barley	1
52	Azerbaijan	2003	Beer	44
52	Azerbaijan	2003	Beverages, Alcoholic	15
52	Azerbaijan	2003	Bovine Meat	23
52	Azerbaijan	2003	Butter, Ghee	8
52	Azerbaijan	2003	Cereals, Other	1
52	Azerbaijan	2003	Cheese	14
52	Azerbaijan	2003	Cocoa Beans	2
52	Azerbaijan	2003	Eggs	12
52	Azerbaijan	2003	Fats, Animals, Raw	1
52	Azerbaijan	2003	Freshwater Fish	3
52	Azerbaijan	2003	Fruits, Other	81
52	Azerbaijan	2003	Grapes	19
52	Azerbaijan	2003	Lemons, Limes	2
52	Azerbaijan	2003	Maize	25
52	Azerbaijan	2003	Maize Germ Oil	2
52	Azerbaijan	2003	Milk, Whole	142
52	Azerbaijan	2003	Mutton & Goat Meat	13
52	Azerbaijan	2003	Nuts	5
52	Azerbaijan	2003	Offals, Edible	8
52	Azerbaijan	2003	Onions	52
52	Azerbaijan	2003	Oranges, Mandarines	7
52	Azerbaijan	2003	Pelagic Fish	1
52	Azerbaijan	2003	Pigmeat	2
52	Azerbaijan	2003	Potatoes	200
52	Azerbaijan	2003	Poultry Meat	10
52	Azerbaijan	2003	Rice (Milled Equivalent)	9
52	Azerbaijan	2003	Soyabean Oil	7
52	Azerbaijan	2003	Sugar, Refined Equivalent	41
52	Azerbaijan	2003	Sunflowerseed Oil	2
52	Azerbaijan	2003	Tea	1
52	Azerbaijan	2003	Tomatoes	135
52	Azerbaijan	2003	Vegetables, Other	236
52	Azerbaijan	2003	Wheat	585
52	Azerbaijan	2003	Wine	1
52	Azerbaijan	2004	Apples	32
52	Azerbaijan	2004	Bananas	2
52	Azerbaijan	2004	Barley	1
52	Azerbaijan	2004	Beer	58
52	Azerbaijan	2004	Beverages, Alcoholic	14
52	Azerbaijan	2004	Bovine Meat	23
52	Azerbaijan	2004	Butter, Ghee	9
52	Azerbaijan	2004	Cereals, Other	3
52	Azerbaijan	2004	Cheese	14
52	Azerbaijan	2004	Cocoa Beans	2
52	Azerbaijan	2004	Eggs	14
52	Azerbaijan	2004	Fats, Animals, Raw	1
52	Azerbaijan	2004	Freshwater Fish	4
52	Azerbaijan	2004	Fruits, Other	54
52	Azerbaijan	2004	Grapes	16
52	Azerbaijan	2004	Lemons, Limes	2
52	Azerbaijan	2004	Maize	28
52	Azerbaijan	2004	Maize Germ Oil	3
52	Azerbaijan	2004	Milk, Whole	117
52	Azerbaijan	2004	Mutton & Goat Meat	13
52	Azerbaijan	2004	Nuts	3

FAO country code	Country	Reference year	Food item	Gross food supply (g $capita^{-1}$ d^{-1})
52	Azerbaijan	2004	Offals, Edible	9
52	Azerbaijan	2004	Onions	54
52	Azerbaijan	2004	Oranges, Mandarines	7
52	Azerbaijan	2004	Pigmeat	2
52	Azerbaijan	2004	Potatoes	222
52	Azerbaijan	2004	Poultry Meat	12
52	Azerbaijan	2004	Rice (Milled Equivalent)	7
52	Azerbaijan	2004	Soyabean Oil	7
52	Azerbaijan	2004	Sugar, Refined Equivalent	39
52	Azerbaijan	2004	Sunflowerseed Oil	5
52	Azerbaijan	2004	Tea	1
52	Azerbaijan	2004	Tomatoes	128
52	Azerbaijan	2004	Vegetables, Other	244
52	Azerbaijan	2004	Wheat	623
52	Azerbaijan	2004	Wine	1
52	Azerbaijan	2005	Apples	32
52	Azerbaijan	2005	Bananas	2
52	Azerbaijan	2005	Beer	80
52	Azerbaijan	2005	Beverages, Alcoholic	14
52	Azerbaijan	2005	Bovine Meat	24
52	Azerbaijan	2005	Butter, Ghee	7
52	Azerbaijan	2005	Cereals, Other	2
52	Azerbaijan	2005	Cheese	14
52	Azerbaijan	2005	Cocoa Beans	3
52	Azerbaijan	2005	Eggs	15
52	Azerbaijan	2005	Freshwater Fish	3
52	Azerbaijan	2005	Fruits, Other	90
52	Azerbaijan	2005	Grapes	24
52	Azerbaijan	2005	Lemons, Limes	2
52	Azerbaijan	2005	Maize	30
52	Azerbaijan	2005	Maize Germ Oil	2
52	Azerbaijan	2005	Milk, Whole	166
52	Azerbaijan	2005	Mutton & Goat Meat	13
52	Azerbaijan	2005	Nuts	7
52	Azerbaijan	2005	Offals, Edible	9
52	Azerbaijan	2005	Onions	56
52	Azerbaijan	2005	Oranges, Mandarines	6
52	Azerbaijan	2005	Palm Oil	2
52	Azerbaijan	2005	Pelagic Fish	2
52	Azerbaijan	2005	Pigmeat	2
52	Azerbaijan	2005	Potatoes	249
52	Azerbaijan	2005	Poultry Meat	13
52	Azerbaijan	2005	Rice (Milled Equivalent)	6
52	Azerbaijan	2005	Soyabean Oil	4
52	Azerbaijan	2005	Sugar, Refined Equivalent	44
52	Azerbaijan	2005	Sunflowerseed Oil	5
52	Azerbaijan	2005	Tea	1
52	Azerbaijan	2005	Tomatoes	128
52	Azerbaijan	2005	Vegetables, Other	277
52	Azerbaijan	2005	Wheat	614
52	Azerbaijan	2005	Wine	1
52	Azerbaijan	2006	Apples	36
52	Azerbaijan	2006	Bananas	3
52	Azerbaijan	2006	Beer	101
52	Azerbaijan	2006	Beverages, Alcoholic	16
52	Azerbaijan	2006	Bovine Meat	25
52	Azerbaijan	2006	Butter, Ghee	7
52	Azerbaijan	2006	Cereals, Other	2
52	Azerbaijan	2006	Cheese	15
52	Azerbaijan	2006	Cocoa Beans	3
52	Azerbaijan	2006	Eggs	13
52	Azerbaijan	2006	Freshwater Fish	2
52	Azerbaijan	2006	Fruits, Other	93
52	Azerbaijan	2006	Grapes	28
52	Azerbaijan	2006	Lemons, Limes	2
52	Azerbaijan	2006	Maize	29
52	Azerbaijan	2006	Maize Germ Oil	1
52	Azerbaijan	2006	Milk, Whole	175
52	Azerbaijan	2006	Mutton & Goat Meat	14
52	Azerbaijan	2006	Nuts	9
52	Azerbaijan	2006	Offals, Edible	7
52	Azerbaijan	2006	Onions	59
52	Azerbaijan	2006	Oranges, Mandarines	7
52	Azerbaijan	2006	Pelagic Fish	2
52	Azerbaijan	2006	Pigmeat	2
52	Azerbaijan	2006	Potatoes	245
52	Azerbaijan	2006	Poultry Meat	13
52	Azerbaijan	2006	Rice (Milled Equivalent)	6
52	Azerbaijan	2006	Soyabean Oil	8
52	Azerbaijan	2006	Sugar, Refined Equivalent	46
52	Azerbaijan	2006	Sunflowerseed Oil	2
52	Azerbaijan	2006	Sweeteners, Other	1
52	Azerbaijan	2006	Tomatoes	128
52	Azerbaijan	2006	Vegetables, Other	281
52	Azerbaijan	2006	Wheat	607
52	Azerbaijan	2006	Wine	1
52	Azerbaijan	2007	Apples	19
52	Azerbaijan	2007	Bananas	4
52	Azerbaijan	2007	Beer	102
52	Azerbaijan	2007	Beverages, Alcoholic	41
52	Azerbaijan	2007	Bovine Meat	23
52	Azerbaijan	2007	Butter, Ghee	7
52	Azerbaijan	2007	Cereals, Other	1
52	Azerbaijan	2007	Cheese	15
52	Azerbaijan	2007	Cocoa Beans	3
52	Azerbaijan	2007	Eggs	15
52	Azerbaijan	2007	Freshwater Fish	1

FAO country code	Country	Reference year	Food item	Gross food supply (g $capita^{-1}$ d^{-1})
52	Azerbaijan	2007	Fruits, Other	94
52	Azerbaijan	2007	Grapes	27
52	Azerbaijan	2007	Lemons, Limes	2
52	Azerbaijan	2007	Maize	26
52	Azerbaijan	2007	Milk, Whole	178
52	Azerbaijan	2007	Mutton & Goat Meat	14
52	Azerbaijan	2007	Nuts	8
52	Azerbaijan	2007	Offals, Edible	6
52	Azerbaijan	2007	Onions	59
52	Azerbaijan	2007	Oranges, Mandarines	2
52	Azerbaijan	2007	Pelagic Fish	3
52	Azerbaijan	2007	Pigmeat	2
52	Azerbaijan	2007	Potatoes	241
52	Azerbaijan	2007	Poultry Meat	19
52	Azerbaijan	2007	Rice (Milled Equivalent)	5
52	Azerbaijan	2007	Soyabean Oil	5
52	Azerbaijan	2007	Sugar, Refined Equivalent	33
52	Azerbaijan	2007	Sunflowerseed Oil	2
52	Azerbaijan	2007	Sweeteners, Other	1
52	Azerbaijan	2007	Tea	3
52	Azerbaijan	2007	Tomatoes	125
52	Azerbaijan	2007	Vegetables, Other	294
52	Azerbaijan	2007	Wheat	599
52	Azerbaijan	2007	Wine	1
52	Azerbaijan	2008	Apples	25
52	Azerbaijan	2008	Bananas	5
52	Azerbaijan	2008	Beer	101
52	Azerbaijan	2008	Beverages, Alcoholic	23
52	Azerbaijan	2008	Bovine Meat	24
52	Azerbaijan	2008	Butter, Ghee	7
52	Azerbaijan	2008	Cereals, Other	3
52	Azerbaijan	2008	Cheese	17
52	Azerbaijan	2008	Citrus, Other	1
52	Azerbaijan	2008	Cocoa Beans	3
52	Azerbaijan	2008	Coffee	1
52	Azerbaijan	2008	Demersal Fish	1
52	Azerbaijan	2008	Eggs	20
52	Azerbaijan	2008	Freshwater Fish	1
52	Azerbaijan	2008	Fruits, Other	89
52	Azerbaijan	2008	Grapes	30
52	Azerbaijan	2008	Lemons, Limes	1
52	Azerbaijan	2008	Maize	31
52	Azerbaijan	2008	Maize Germ Oil	4
52	Azerbaijan	2008	Milk, Whole	171
52	Azerbaijan	2008	Mutton & Goat Meat	14
52	Azerbaijan	2008	Nuts	10
52	Azerbaijan	2008	Offals, Edible	4
52	Azerbaijan	2008	Onions	49
52	Azerbaijan	2008	Oranges, Mandarines	2
52	Azerbaijan	2008	Pelagic Fish	4
52	Azerbaijan	2008	Pigmeat	2
52	Azerbaijan	2008	Potatoes	223
52	Azerbaijan	2008	Poultry Meat	20
52	Azerbaijan	2008	Rice (Milled Equivalent)	8
52	Azerbaijan	2008	Soyabean Oil	2
52	Azerbaijan	2008	Sugar, Refined Equivalent	35
52	Azerbaijan	2008	Sweeteners, Other	1
52	Azerbaijan	2008	Tea	3
52	Azerbaijan	2008	Tomatoes	129
52	Azerbaijan	2008	Vegetables, Other	268
52	Azerbaijan	2008	Wheat	598
52	Azerbaijan	2008	Wine	1
52	Azerbaijan	2009	Apples	32
52	Azerbaijan	2009	Bananas	8
52	Azerbaijan	2009	Beer	110
52	Azerbaijan	2009	Beverages, Alcoholic	26
52	Azerbaijan	2009	Bovine Meat	28
52	Azerbaijan	2009	Butter, Ghee	10
52	Azerbaijan	2009	Cereals, Other	2
52	Azerbaijan	2009	Cheese	18
52	Azerbaijan	2009	Citrus, Other	1
52	Azerbaijan	2009	Cocoa Beans	3
52	Azerbaijan	2009	Coffee	1
52	Azerbaijan	2009	Eggs	21
52	Azerbaijan	2009	Freshwater Fish	2
52	Azerbaijan	2009	Fruits, Other	93
52	Azerbaijan	2009	Grapes	32
52	Azerbaijan	2009	Lemons, Limes	2
52	Azerbaijan	2009	Maize	31
52	Azerbaijan	2009	Milk, Whole	190
52	Azerbaijan	2009	Mutton & Goat Meat	21
52	Azerbaijan	2009	Nuts	8
52	Azerbaijan	2009	Offals, Edible	5
52	Azerbaijan	2009	Onions	49
52	Azerbaijan	2009	Oranges, Mandarines	6
52	Azerbaijan	2009	Palm Oil	1
52	Azerbaijan	2009	Pelagic Fish	3
52	Azerbaijan	2009	Pigmeat	2
52	Azerbaijan	2009	Potatoes	222
52	Azerbaijan	2009	Poultry Meat	24
52	Azerbaijan	2009	Rice (Milled Equivalent)	7
52	Azerbaijan	2009	Soyabean Oil	1
52	Azerbaijan	2009	Sugar, Refined Equivalent	34
52	Azerbaijan	2009	Sunflowerseed Oil	3
52	Azerbaijan	2009	Sweeteners, Other	2
52	Azerbaijan	2009	Tea	4
52	Azerbaijan	2009	Tomatoes	115

FAO country code	Country	Reference year	Food item	Gross food supply (g *capita*$^{-1}$ d^{-1})
52	Azerbaijan	2009	Vegetables, Other	273
52	Azerbaijan	2009	Wheat	592
52	Azerbaijan	2009	Wine	1
52	Azerbaijan	2010	Apples	32
52	Azerbaijan	2010	Bananas	6
52	Azerbaijan	2010	Beer	114
52	Azerbaijan	2010	Beverages, Alcoholic	28
52	Azerbaijan	2010	Bovine Meat	34
52	Azerbaijan	2010	Butter, Ghee	10
52	Azerbaijan	2010	Cereals, Other	5
52	Azerbaijan	2010	Cheese	18
52	Azerbaijan	2010	Citrus, Other	1
52	Azerbaijan	2010	Cocoa Beans	3
52	Azerbaijan	2010	Coffee	1
52	Azerbaijan	2010	Eggs	19
52	Azerbaijan	2010	Freshwater Fish	2
52	Azerbaijan	2010	Fruits, Other	94
52	Azerbaijan	2010	Grapes	32
52	Azerbaijan	2010	Honey	1
52	Azerbaijan	2010	Lemons, Limes	4
52	Azerbaijan	2010	Maize	32
52	Azerbaijan	2010	Maize Germ Oil	5
52	Azerbaijan	2010	Milk, Whole	224
52	Azerbaijan	2010	Mutton & Goat Meat	22
52	Azerbaijan	2010	Nuts	9
52	Azerbaijan	2010	Offals, Edible	7
52	Azerbaijan	2010	Onions	52
52	Azerbaijan	2010	Oranges, Mandarines	9
52	Azerbaijan	2010	Pelagic Fish	3
52	Azerbaijan	2010	Pigmeat	1
52	Azerbaijan	2010	Pineapples	1
52	Azerbaijan	2010	Potatoes	200
52	Azerbaijan	2010	Poultry Meat	24
52	Azerbaijan	2010	Rice (Milled Equivalent)	6
52	Azerbaijan	2010	Soyabean Oil	1
52	Azerbaijan	2010	Sugar, Refined Equivalent	37
52	Azerbaijan	2010	Sunflowerseed Oil	1
52	Azerbaijan	2010	Sweeteners, Other	2
52	Azerbaijan	2010	Tea	3
52	Azerbaijan	2010	Tomatoes	122
52	Azerbaijan	2010	Vegetables, Other	279
52	Azerbaijan	2010	Wheat	583
52	Azerbaijan	2010	Wine	1
52	Azerbaijan	2011	Apples	32
52	Azerbaijan	2011	Bananas	7
52	Azerbaijan	2011	Beer	117
52	Azerbaijan	2011	Beverages, Alcoholic	28
52	Azerbaijan	2011	Bovine Meat	36
52	Azerbaijan	2011	Butter, Ghee	9
52	Azerbaijan	2011	Cereals, Other	8
52	Azerbaijan	2011	Cheese	18
52	Azerbaijan	2011	Citrus, Other	1
52	Azerbaijan	2011	Cocoa Beans	3
52	Azerbaijan	2011	Coffee	1
52	Azerbaijan	2011	Eggs	20
52	Azerbaijan	2011	Fruits, Other	102
52	Azerbaijan	2011	Grapes	37
52	Azerbaijan	2011	Groundnuts (Shelled Eq)	1
52	Azerbaijan	2011	Honey	1
52	Azerbaijan	2011	Lemons, Limes	3
52	Azerbaijan	2011	Maize	29
52	Azerbaijan	2011	Maize Germ Oil	4
52	Azerbaijan	2011	Milk, Whole	244
52	Azerbaijan	2011	Mutton & Goat Meat	22
52	Azerbaijan	2011	Nuts	8
52	Azerbaijan	2011	Offals, Edible	6
52	Azerbaijan	2011	Onions	51
52	Azerbaijan	2011	Oranges, Mandarines	9
52	Azerbaijan	2011	Pelagic Fish	5
52	Azerbaijan	2011	Pigmeat	1
52	Azerbaijan	2011	Pineapples	1
52	Azerbaijan	2011	Potatoes	197
52	Azerbaijan	2011	Poultry Meat	27
52	Azerbaijan	2011	Rice (Milled Equivalent)	6
52	Azerbaijan	2011	Soyabean Oil	1
52	Azerbaijan	2011	Sugar, Refined Equivalent	38
52	Azerbaijan	2011	Sunflowerseed Oil	1
52	Azerbaijan	2011	Sweeteners, Other	2
52	Azerbaijan	2011	Tea	4
52	Azerbaijan	2011	Tomatoes	128
52	Azerbaijan	2011	Vegetables, Other	292
52	Azerbaijan	2011	Wheat	576
52	Azerbaijan	2011	Wine	1
16	Bangladesh	1992	Bananas	14
16	Bangladesh	1992	Beans	2
16	Bangladesh	1992	Bovine Meat	4
16	Bangladesh	1992	Coconut Oil	1
16	Bangladesh	1992	Demersal Fish	1
16	Bangladesh	1992	Eggs	2
16	Bangladesh	1992	Freshwater Fish	18
16	Bangladesh	1992	Fruits, Other	12
16	Bangladesh	1992	Marine Fish, Other	1
16	Bangladesh	1992	Milk, Whole	36
16	Bangladesh	1992	Millet	2
16	Bangladesh	1992	Mutton & Goat Meat	2
16	Bangladesh	1992	Nuts	1
16	Bangladesh	1992	Offals, Edible	1

FAO countrycode	Country	Reference year	Food item	Gross food supply (g *capita*$^{-1}$ d^{-1})
16	Bangladesh	1992	Onions	6
16	Bangladesh	1992	Palm Oil	2
16	Bangladesh	1992	Peas	1
16	Bangladesh	1992	Pelagic Fish	1
16	Bangladesh	1992	Pimento	2
16	Bangladesh	1992	Pineapples	3
16	Bangladesh	1992	Potatoes	27
16	Bangladesh	1992	Poultry Meat	2
16	Bangladesh	1992	Pulses, Other	10
16	Bangladesh	1992	Rape and Mustard Oil	2
16	Bangladesh	1992	Rice (Milled Equivalent)	428
16	Bangladesh	1992	Soyabean Oil	7
16	Bangladesh	1992	Spices, Other	2
16	Bangladesh	1992	Sugar Cane	10
16	Bangladesh	1992	Sugar, Non-Centrifugal	12
16	Bangladesh	1992	Sugar, Refined Equivalent	5
16	Bangladesh	1992	Sweet Potatoes	10
16	Bangladesh	1992	Sweeteners, Other	3
16	Bangladesh	1992	Tomatoes	2
16	Bangladesh	1992	Vegetables, Other	26
16	Bangladesh	1992	Wheat	61
16	Bangladesh	1993	Bananas	14
16	Bangladesh	1993	Beans	2
16	Bangladesh	1993	Bovine Meat	4
16	Bangladesh	1993	Coconut Oil	1
16	Bangladesh	1993	Crustaceans	1
16	Bangladesh	1993	Demersal Fish	1
16	Bangladesh	1993	Eggs	2
16	Bangladesh	1993	Freshwater Fish	18
16	Bangladesh	1993	Fruits, Other	12
16	Bangladesh	1993	Marine Fish, Other	2
16	Bangladesh	1993	Milk, Whole	37
16	Bangladesh	1993	Millet	1
16	Bangladesh	1993	Mutton & Goat Meat	2
16	Bangladesh	1993	Nuts	1
16	Bangladesh	1993	Offals, Edible	1
16	Bangladesh	1993	Onions	5
16	Bangladesh	1993	Palm Oil	1
16	Bangladesh	1993	Peas	1
16	Bangladesh	1993	Pelagic Fish	1
16	Bangladesh	1993	Pimento	1
16	Bangladesh	1993	Pineapples	3
16	Bangladesh	1993	Potatoes	27
16	Bangladesh	1993	Poultry Meat	3
16	Bangladesh	1993	Pulses, Other	10
16	Bangladesh	1993	Rape and Mustard Oil	2
16	Bangladesh	1993	Rice (Milled Equivalent)	415
16	Bangladesh	1993	Sesameseed Oil	1
16	Bangladesh	1993	Soyabean Oil	5
16	Bangladesh	1993	Spices, Other	2
16	Bangladesh	1993	Sugar Cane	10
16	Bangladesh	1993	Sugar, Non-Centrifugal	11
16	Bangladesh	1993	Sugar, Refined Equivalent	6
16	Bangladesh	1993	Sweet Potatoes	9
16	Bangladesh	1993	Sweeteners, Other	3
16	Bangladesh	1993	Tomatoes	2
16	Bangladesh	1993	Vegetables, Other	26
16	Bangladesh	1993	Wheat	48
16	Bangladesh	1994	Bananas	13
16	Bangladesh	1994	Beans	2
16	Bangladesh	1994	Bovine Meat	4
16	Bangladesh	1994	Coconut Oil	1
16	Bangladesh	1994	Demersal Fish	1
16	Bangladesh	1994	Eggs	2
16	Bangladesh	1994	Freshwater Fish	20
16	Bangladesh	1994	Fruits, Other	12
16	Bangladesh	1994	Marine Fish, Other	1
16	Bangladesh	1994	Milk, Whole	36
16	Bangladesh	1994	Millet	1
16	Bangladesh	1994	Mutton & Goat Meat	2
16	Bangladesh	1994	Nuts	1
16	Bangladesh	1994	Offals, Edible	1
16	Bangladesh	1994	Onions	4
16	Bangladesh	1994	Palm Oil	3
16	Bangladesh	1994	Peas	1
16	Bangladesh	1994	Pelagic Fish	1
16	Bangladesh	1994	Pimento	1
16	Bangladesh	1994	Pineapples	3
16	Bangladesh	1994	Potatoes	27
16	Bangladesh	1994	Poultry Meat	3
16	Bangladesh	1994	Pulses, Other	11
16	Bangladesh	1994	Rape and Mustard Oil	2
16	Bangladesh	1994	Rice (Milled Equivalent)	414
16	Bangladesh	1994	Soyabean Oil	3
16	Bangladesh	1994	Spices, Other	2
16	Bangladesh	1994	Sugar Cane	10
16	Bangladesh	1994	Sugar, Non-Centrifugal	10
16	Bangladesh	1994	Sugar, Refined Equivalent	7
16	Bangladesh	1994	Sweet Potatoes	9
16	Bangladesh	1994	Sweeteners, Other	3
16	Bangladesh	1994	Tomatoes	2
16	Bangladesh	1994	Vegetables, Other	26
16	Bangladesh	1994	Wheat	48
16	Bangladesh	1995	Bananas	13
16	Bangladesh	1995	Beans	2
16	Bangladesh	1995	Bovine Meat	3
16	Bangladesh	1995	Coconut Oil	1

FAO country code	Country	Reference year	Food item	Gross food supply (g $capita^{-1}$ d^{-1})
16	Bangladesh	1995	Crustaceans	1
16	Bangladesh	1995	Demersal Fish	1
16	Bangladesh	1995	Eggs	2
16	Bangladesh	1995	Freshwater Fish	21
16	Bangladesh	1995	Fruits, Other	12
16	Bangladesh	1995	Marine Fish, Other	1
16	Bangladesh	1995	Milk, Whole	35
16	Bangladesh	1995	Millet	1
16	Bangladesh	1995	Mutton & Goat Meat	2
16	Bangladesh	1995	Nuts	1
16	Bangladesh	1995	Offals, Edible	1
16	Bangladesh	1995	Onions	4
16	Bangladesh	1995	Oranges, Mandarines	1
16	Bangladesh	1995	Palm Oil	2
16	Bangladesh	1995	Peas	1
16	Bangladesh	1995	Pelagic Fish	1
16	Bangladesh	1995	Pimento	1
16	Bangladesh	1995	Pineapples	3
16	Bangladesh	1995	Potatoes	27
16	Bangladesh	1995	Poultry Meat	3
16	Bangladesh	1995	Pulses, Other	9
16	Bangladesh	1995	Rape and Mustard Oil	2
16	Bangladesh	1995	Rice (Milled Equivalent)	420
16	Bangladesh	1995	Soyabean Oil	5
16	Bangladesh	1995	Spices, Other	2
16	Bangladesh	1995	Sugar Cane	10
16	Bangladesh	1995	Sugar, Non-Centrifugal	8
16	Bangladesh	1995	Sugar, Refined Equivalent	8
16	Bangladesh	1995	Sweet Potatoes	9
16	Bangladesh	1995	Sweeteners, Other	3
16	Bangladesh	1995	Tea	1
16	Bangladesh	1995	Tomatoes	2
16	Bangladesh	1995	Vegetables, Other	26
16	Bangladesh	1995	Wheat	52
16	Bangladesh	1996	Bananas	13
16	Bangladesh	1996	Beans	2
16	Bangladesh	1996	Bovine Meat	3
16	Bangladesh	1996	Coconut Oil	1
16	Bangladesh	1996	Crustaceans	1
16	Bangladesh	1996	Demersal Fish	1
16	Bangladesh	1996	Eggs	3
16	Bangladesh	1996	Freshwater Fish	22
16	Bangladesh	1996	Fruits, Other	12
16	Bangladesh	1996	Marine Fish, Other	1
16	Bangladesh	1996	Milk, Whole	37
16	Bangladesh	1996	Millet	1
16	Bangladesh	1996	Mutton & Goat Meat	3
16	Bangladesh	1996	Nuts	1
16	Bangladesh	1996	Offals, Edible	1
16	Bangladesh	1996	Onions	4
16	Bangladesh	1996	Oranges, Mandarines	1
16	Bangladesh	1996	Palm Oil	3
16	Bangladesh	1996	Peas	1
16	Bangladesh	1996	Pelagic Fish	1
16	Bangladesh	1996	Pimento	1
16	Bangladesh	1996	Pineapples	3
16	Bangladesh	1996	Potatoes	27
16	Bangladesh	1996	Poultry Meat	2
16	Bangladesh	1996	Pulses, Other	9
16	Bangladesh	1996	Rape and Mustard Oil	2
16	Bangladesh	1996	Rice (Milled Equivalent)	419
16	Bangladesh	1996	Sorghum	1
16	Bangladesh	1996	Soyabean Oil	5
16	Bangladesh	1996	Spices, Other	2
16	Bangladesh	1996	Sugar Cane	10
16	Bangladesh	1996	Sugar, Non-Centrifugal	10
16	Bangladesh	1996	Sugar, Refined Equivalent	6
16	Bangladesh	1996	Sweet Potatoes	9
16	Bangladesh	1996	Sweeteners, Other	3
16	Bangladesh	1996	Tea	1
16	Bangladesh	1996	Tomatoes	2
16	Bangladesh	1996	Vegetables, Other	26
16	Bangladesh	1996	Wheat	54
16	Bangladesh	1997	Bananas	12
16	Bangladesh	1997	Beans	2
16	Bangladesh	1997	Bovine Meat	4
16	Bangladesh	1997	Cassava	1
16	Bangladesh	1997	Coconut Oil	1
16	Bangladesh	1997	Crustaceans	1
16	Bangladesh	1997	Demersal Fish	1
16	Bangladesh	1997	Eggs	3
16	Bangladesh	1997	Freshwater Fish	24
16	Bangladesh	1997	Fruits, Other	12
16	Bangladesh	1997	Marine Fish, Other	1
16	Bangladesh	1997	Milk, Whole	38
16	Bangladesh	1997	Millet	1
16	Bangladesh	1997	Mutton & Goat Meat	3
16	Bangladesh	1997	Nuts	1
16	Bangladesh	1997	Offals, Edible	1
16	Bangladesh	1997	Onions	4
16	Bangladesh	1997	Oranges, Mandarines	1
16	Bangladesh	1997	Palm Oil	3
16	Bangladesh	1997	Peas	2
16	Bangladesh	1997	Pelagic Fish	1
16	Bangladesh	1997	Pimento	1
16	Bangladesh	1997	Pineapples	3
16	Bangladesh	1997	Potatoes	27

FAO country code	Country	Reference year	Food item	Gross food supply (g $capita^{-1}$ d^{-1})
16	Bangladesh	1997	Poultry Meat	2
16	Bangladesh	1997	Pulses, Other	10
16	Bangladesh	1997	Rape and Mustard Oil	2
16	Bangladesh	1997	Rice (Milled Equivalent)	407
16	Bangladesh	1997	Soyabean Oil	9
16	Bangladesh	1997	Spices, Other	2
16	Bangladesh	1997	Sugar Cane	10
16	Bangladesh	1997	Sugar, Non-Centrifugal	12
16	Bangladesh	1997	Sugar, Refined Equivalent	8
16	Bangladesh	1997	Sweet Potatoes	8
16	Bangladesh	1997	Sweeteners, Other	3
16	Bangladesh	1997	Tea	1
16	Bangladesh	1997	Tomatoes	2
16	Bangladesh	1997	Vegetables, Other	26
16	Bangladesh	1997	Wheat	66
16	Bangladesh	1998	Bananas	12
16	Bangladesh	1998	Beans	1
16	Bangladesh	1998	Bovine Meat	4
16	Bangladesh	1998	Cassava	1
16	Bangladesh	1998	Coconut Oil	1
16	Bangladesh	1998	Crustaceans	2
16	Bangladesh	1998	Demersal Fish	1
16	Bangladesh	1998	Eggs	3
16	Bangladesh	1998	Freshwater Fish	25
16	Bangladesh	1998	Fruits, Other	12
16	Bangladesh	1998	Marine Fish, Other	1
16	Bangladesh	1998	Milk, Whole	35
16	Bangladesh	1998	Millet	1
16	Bangladesh	1998	Mutton & Goat Meat	3
16	Bangladesh	1998	Nuts	1
16	Bangladesh	1998	Offals, Edible	1
16	Bangladesh	1998	Onions	4
16	Bangladesh	1998	Palm Oil	5
16	Bangladesh	1998	Peas	2
16	Bangladesh	1998	Pelagic Fish	1
16	Bangladesh	1998	Pimento	1
16	Bangladesh	1998	Pineapples	3
16	Bangladesh	1998	Potatoes	25
16	Bangladesh	1998	Poultry Meat	2
16	Bangladesh	1998	Pulses, Other	9
16	Bangladesh	1998	Rape and Mustard Oil	3
16	Bangladesh	1998	Rice (Milled Equivalent)	419
16	Bangladesh	1998	Soyabean Oil	11
16	Bangladesh	1998	Spices, Other	2
16	Bangladesh	1998	Sugar Cane	9
16	Bangladesh	1998	Sugar, Non-Centrifugal	10
16	Bangladesh	1998	Sugar, Refined Equivalent	7
16	Bangladesh	1998	Sweet Potatoes	8
16	Bangladesh	1998	Sweeteners, Other	3
16	Bangladesh	1998	Tea	1
16	Bangladesh	1998	Tomatoes	2
16	Bangladesh	1998	Vegetables, Other	26
16	Bangladesh	1998	Wheat	61
16	Bangladesh	1999	Bananas	11
16	Bangladesh	1999	Beans	1
16	Bangladesh	1999	Bovine Meat	4
16	Bangladesh	1999	Coconut Oil	1
16	Bangladesh	1999	Crustaceans	2
16	Bangladesh	1999	Demersal Fish	1
16	Bangladesh	1999	Eggs	3
16	Bangladesh	1999	Freshwater Fish	27
16	Bangladesh	1999	Fruits, Other	12
16	Bangladesh	1999	Marine Fish, Other	1
16	Bangladesh	1999	Milk, Whole	36
16	Bangladesh	1999	Millet	1
16	Bangladesh	1999	Mutton & Goat Meat	3
16	Bangladesh	1999	Nuts	1
16	Bangladesh	1999	Offals, Edible	1
16	Bangladesh	1999	Onions	3
16	Bangladesh	1999	Oranges, Mandarines	1
16	Bangladesh	1999	Palm Oil	4
16	Bangladesh	1999	Peas	3
16	Bangladesh	1999	Pelagic Fish	1
16	Bangladesh	1999	Pimento	3
16	Bangladesh	1999	Pineapples	3
16	Bangladesh	1999	Potatoes	47
16	Bangladesh	1999	Poultry Meat	3
16	Bangladesh	1999	Pulses, Other	8
16	Bangladesh	1999	Rape and Mustard Oil	4
16	Bangladesh	1999	Rice (Milled Equivalent)	446
16	Bangladesh	1999	Soyabean Oil	5
16	Bangladesh	1999	Spices, Other	2
16	Bangladesh	1999	Sugar Cane	9
16	Bangladesh	1999	Sugar, Non-Centrifugal	9
16	Bangladesh	1999	Sugar, Refined Equivalent	6
16	Bangladesh	1999	Sweet Potatoes	7
16	Bangladesh	1999	Sweeteners, Other	3
16	Bangladesh	1999	Tea	1
16	Bangladesh	1999	Tomatoes	2
16	Bangladesh	1999	Vegetables, Other	30
16	Bangladesh	1999	Wheat	81
16	Bangladesh	2000	Bananas	11
16	Bangladesh	2000	Beans	1
16	Bangladesh	2000	Bovine Meat	4
16	Bangladesh	2000	Cassava	1
16	Bangladesh	2000	Coconut Oil	1
16	Bangladesh	2000	Crustaceans	1

FAO country code	Country	Reference year	Food item	Gross food supply (g $capita^{-1}$ d^{-1})
16	Bangladesh	2000	Demersal Fish	1
16	Bangladesh	2000	Eggs	3
16	Bangladesh	2000	Freshwater Fish	28
16	Bangladesh	2000	Fruits, Other	12
16	Bangladesh	2000	Marine Fish, Other	2
16	Bangladesh	2000	Milk, Whole	37
16	Bangladesh	2000	Mutton & Goat Meat	3
16	Bangladesh	2000	Nuts	1
16	Bangladesh	2000	Offals, Edible	1
16	Bangladesh	2000	Onions	3
16	Bangladesh	2000	Oranges, Mandarines	1
16	Bangladesh	2000	Palm Oil	3
16	Bangladesh	2000	Peas	3
16	Bangladesh	2000	Pelagic Fish	1
16	Bangladesh	2000	Pimento	3
16	Bangladesh	2000	Pineapples	3
16	Bangladesh	2000	Potatoes	50
16	Bangladesh	2000	Poultry Meat	3
16	Bangladesh	2000	Pulses, Other	7
16	Bangladesh	2000	Rape and Mustard Oil	3
16	Bangladesh	2000	Rice (Milled Equivalent)	449
16	Bangladesh	2000	Soyabean Oil	9
16	Bangladesh	2000	Spices, Other	2
16	Bangladesh	2000	Sugar Cane	8
16	Bangladesh	2000	Sugar, Non-Centrifugal	10
16	Bangladesh	2000	Sugar, Refined Equivalent	5
16	Bangladesh	2000	Sweet Potatoes	7
16	Bangladesh	2000	Sweeteners, Other	3
16	Bangladesh	2000	Tea	1
16	Bangladesh	2000	Tomatoes	2
16	Bangladesh	2000	Vegetables, Other	29
16	Bangladesh	2000	Wheat	66
16	Bangladesh	2001	Apples	1
16	Bangladesh	2001	Bananas	11
16	Bangladesh	2001	Beans	1
16	Bangladesh	2001	Bovine Meat	4
16	Bangladesh	2001	Cassava	1
16	Bangladesh	2001	Coconut Oil	1
16	Bangladesh	2001	Crustaceans	1
16	Bangladesh	2001	Demersal Fish	1
16	Bangladesh	2001	Eggs	3
16	Bangladesh	2001	Freshwater Fish	30
16	Bangladesh	2001	Fruits, Other	12
16	Bangladesh	2001	Marine Fish, Other	2
16	Bangladesh	2001	Milk, Whole	36
16	Bangladesh	2001	Mutton & Goat Meat	3
16	Bangladesh	2001	Nuts	1
16	Bangladesh	2001	Offals, Edible	1
16	Bangladesh	2001	Onions	3
16	Bangladesh	2001	Oranges, Mandarines	1
16	Bangladesh	2001	Palm Oil	4
16	Bangladesh	2001	Peas	6
16	Bangladesh	2001	Pelagic Fish	1
16	Bangladesh	2001	Pimento	3
16	Bangladesh	2001	Pineapples	3
16	Bangladesh	2001	Potatoes	54
16	Bangladesh	2001	Poultry Meat	3
16	Bangladesh	2001	Pulses, Other	7
16	Bangladesh	2001	Rape and Mustard Oil	3
16	Bangladesh	2001	Rice (Milled Equivalent)	474
16	Bangladesh	2001	Soyabean Oil	10
16	Bangladesh	2001	Spices, Other	2
16	Bangladesh	2001	Sugar Cane	8
16	Bangladesh	2001	Sugar, Non-Centrifugal	10
16	Bangladesh	2001	Sugar, Refined Equivalent	9
16	Bangladesh	2001	Sweet Potatoes	7
16	Bangladesh	2001	Sweeteners, Other	3
16	Bangladesh	2001	Tea	1
16	Bangladesh	2001	Tomatoes	2
16	Bangladesh	2001	Vegetables, Other	29
16	Bangladesh	2001	Wheat	52
16	Bangladesh	2002	Bananas	12
16	Bangladesh	2002	Beans	1
16	Bangladesh	2002	Bovine Meat	4
16	Bangladesh	2002	Cassava	1
16	Bangladesh	2002	Coconut Oil	1
16	Bangladesh	2002	Crustaceans	1
16	Bangladesh	2002	Demersal Fish	1
16	Bangladesh	2002	Eggs	3
16	Bangladesh	2002	Freshwater Fish	31
16	Bangladesh	2002	Fruits, Other	12
16	Bangladesh	2002	Marine Fish, Other	3
16	Bangladesh	2002	Milk, Whole	38
16	Bangladesh	2002	Mutton & Goat Meat	3
16	Bangladesh	2002	Nuts	1
16	Bangladesh	2002	Offals, Edible	1
16	Bangladesh	2002	Onions	4
16	Bangladesh	2002	Oranges, Mandarines	1
16	Bangladesh	2002	Palm Oil	4
16	Bangladesh	2002	Peas	6
16	Bangladesh	2002	Pelagic Fish	1
16	Bangladesh	2002	Pimento	3
16	Bangladesh	2002	Pineapples	3
16	Bangladesh	2002	Potatoes	49
16	Bangladesh	2002	Poultry Meat	3
16	Bangladesh	2002	Pulses, Other	7
16	Bangladesh	2002	Rape and Mustard Oil	2

FAO country code	Country	Reference year	Food item	Gross food supply (g $capita^{-1}$ d^{-1})
16	Bangladesh	2002	Rice (Milled Equivalent)	474
16	Bangladesh	2002	Soyabean Oil	5
16	Bangladesh	2002	Spices, Other	2
16	Bangladesh	2002	Sugar Cane	8
16	Bangladesh	2002	Sugar, Non-Centrifugal	8
16	Bangladesh	2002	Sugar, Refined Equivalent	8
16	Bangladesh	2002	Sweet Potatoes	6
16	Bangladesh	2002	Sweeteners, Other	2
16	Bangladesh	2002	Tea	1
16	Bangladesh	2002	Tomatoes	2
16	Bangladesh	2002	Vegetables, Other	29
16	Bangladesh	2002	Wheat	61
16	Bangladesh	2003	Bananas	12
16	Bangladesh	2003	Beans	1
16	Bangladesh	2003	Bovine Meat	4
16	Bangladesh	2003	Cassava	1
16	Bangladesh	2003	Coconut Oil	1
16	Bangladesh	2003	Crustaceans	1
16	Bangladesh	2003	Dates	1
16	Bangladesh	2003	Demersal Fish	2
16	Bangladesh	2003	Eggs	4
16	Bangladesh	2003	Freshwater Fish	32
16	Bangladesh	2003	Fruits, Other	14
16	Bangladesh	2003	Marine Fish, Other	3
16	Bangladesh	2003	Milk, Whole	38
16	Bangladesh	2003	Millet	1
16	Bangladesh	2003	Mutton & Goat Meat	3
16	Bangladesh	2003	Nuts	1
16	Bangladesh	2003	Offals, Edible	1
16	Bangladesh	2003	Onions	9
16	Bangladesh	2003	Oranges, Mandarines	1
16	Bangladesh	2003	Palm Oil	6
16	Bangladesh	2003	Peas	3
16	Bangladesh	2003	Pelagic Fish	1
16	Bangladesh	2003	Pimento	3
16	Bangladesh	2003	Pineapples	3
16	Bangladesh	2003	Potatoes	55
16	Bangladesh	2003	Poultry Meat	3
16	Bangladesh	2003	Pulses, Other	9
16	Bangladesh	2003	Rape and Mustard Oil	2
16	Bangladesh	2003	Rice (Milled Equivalent)	482
16	Bangladesh	2003	Soyabean Oil	6
16	Bangladesh	2003	Spices, Other	2
16	Bangladesh	2003	Sugar Cane	8
16	Bangladesh	2003	Sugar, Non-Centrifugal	7
16	Bangladesh	2003	Sugar, Refined Equivalent	6
16	Bangladesh	2003	Sweet Potatoes	6
16	Bangladesh	2003	Sweeteners, Other	3
16	Bangladesh	2003	Tea	1
16	Bangladesh	2003	Tomatoes	2
16	Bangladesh	2003	Vegetables, Other	29
16	Bangladesh	2003	Wheat	58
16	Bangladesh	2004	Apples	1
16	Bangladesh	2004	Bananas	12
16	Bangladesh	2004	Beans	1
16	Bangladesh	2004	Bovine Meat	4
16	Bangladesh	2004	Cassava	1
16	Bangladesh	2004	Crustaceans	1
16	Bangladesh	2004	Demersal Fish	1
16	Bangladesh	2004	Eggs	4
16	Bangladesh	2004	Freshwater Fish	33
16	Bangladesh	2004	Fruits, Other	17
16	Bangladesh	2004	Maize	1
16	Bangladesh	2004	Marine Fish, Other	3
16	Bangladesh	2004	Milk, Whole	39
16	Bangladesh	2004	Millet	1
16	Bangladesh	2004	Mutton & Goat Meat	3
16	Bangladesh	2004	Nuts	1
16	Bangladesh	2004	Offals, Edible	1
16	Bangladesh	2004	Onions	12
16	Bangladesh	2004	Oranges, Mandarines	1
16	Bangladesh	2004	Palm Oil	7
16	Bangladesh	2004	Peas	4
16	Bangladesh	2004	Pelagic Fish	1
16	Bangladesh	2004	Pimento	3
16	Bangladesh	2004	Pineapples	4
16	Bangladesh	2004	Potatoes	62
16	Bangladesh	2004	Poultry Meat	3
16	Bangladesh	2004	Pulses, Other	9
16	Bangladesh	2004	Rape and Mustard Oil	2
16	Bangladesh	2004	Rice (Milled Equivalent)	477
16	Bangladesh	2004	Soyabean Oil	5
16	Bangladesh	2004	Soyabeans	2
16	Bangladesh	2004	Spices, Other	3
16	Bangladesh	2004	Sugar Cane	7
16	Bangladesh	2004	Sugar, Non-Centrifugal	7
16	Bangladesh	2004	Sugar, Refined Equivalent	11
16	Bangladesh	2004	Sweet Potatoes	6
16	Bangladesh	2004	Sweeteners, Other	3
16	Bangladesh	2004	Tea	1
16	Bangladesh	2004	Tomatoes	2
16	Bangladesh	2004	Vegetables, Other	30
16	Bangladesh	2004	Wheat	60
16	Bangladesh	2005	Apples	1
16	Bangladesh	2005	Bananas	15
16	Bangladesh	2005	Beans	1
16	Bangladesh	2005	Bovine Meat	4

FAO country code	Country	Reference year	Food item	Gross food supply (g *capita*$^{-1}$ d^{-1})
16	Bangladesh	2005	Cassava	1
16	Bangladesh	2005	Crustaceans	2
16	Bangladesh	2005	Demersal Fish	1
16	Bangladesh	2005	Eggs	4
16	Bangladesh	2005	Freshwater Fish	35
16	Bangladesh	2005	Fruits, Other	30
16	Bangladesh	2005	Grapefruit	1
16	Bangladesh	2005	Lemons, Limes	1
16	Bangladesh	2005	Marine Fish, Other	3
16	Bangladesh	2005	Milk, Whole	41
16	Bangladesh	2005	Mutton & Goat Meat	3
16	Bangladesh	2005	Nuts	2
16	Bangladesh	2005	Offals, Edible	1
16	Bangladesh	2005	Onions	12
16	Bangladesh	2005	Palm Oil	8
16	Bangladesh	2005	Peas	3
16	Bangladesh	2005	Pelagic Fish	1
16	Bangladesh	2005	Pimento	4
16	Bangladesh	2005	Pineapples	4
16	Bangladesh	2005	Potatoes	76
16	Bangladesh	2005	Poultry Meat	3
16	Bangladesh	2005	Pulses, Other	8
16	Bangladesh	2005	Rape and Mustard Oil	2
16	Bangladesh	2005	Rice (Milled Equivalent)	464
16	Bangladesh	2005	Soyabean Oil	5
16	Bangladesh	2005	Soyabeans	2
16	Bangladesh	2005	Spices, Other	3
16	Bangladesh	2005	Sugar Cane	7
16	Bangladesh	2005	Sugar, Non-Centrifugal	9
16	Bangladesh	2005	Sugar, Refined Equivalent	15
16	Bangladesh	2005	Sweet Potatoes	5
16	Bangladesh	2005	Sweeteners, Other	3
16	Bangladesh	2005	Tea	1
16	Bangladesh	2005	Tomatoes	2
16	Bangladesh	2005	Vegetables, Other	32
16	Bangladesh	2005	Wheat	56
16	Bangladesh	2006	Bananas	15
16	Bangladesh	2006	Beans	1
16	Bangladesh	2006	Bovine Meat	4
16	Bangladesh	2006	Cassava	1
16	Bangladesh	2006	Coconut Oil	1
16	Bangladesh	2006	Crustaceans	2
16	Bangladesh	2006	Demersal Fish	2
16	Bangladesh	2006	Eggs	4
16	Bangladesh	2006	Freshwater Fish	36
16	Bangladesh	2006	Fruits, Other	33
16	Bangladesh	2006	Grapefruit	1
16	Bangladesh	2006	Lemons, Limes	1
16	Bangladesh	2006	Maize	1
16	Bangladesh	2006	Marine Fish, Other	2
16	Bangladesh	2006	Milk, Whole	43
16	Bangladesh	2006	Mutton & Goat Meat	3
16	Bangladesh	2006	Nuts	2
16	Bangladesh	2006	Offals, Edible	2
16	Bangladesh	2006	Onions	16
16	Bangladesh	2006	Palm Oil	8
16	Bangladesh	2006	Peas	2
16	Bangladesh	2006	Pelagic Fish	1
16	Bangladesh	2006	Pimento	3
16	Bangladesh	2006	Pineapples	4
16	Bangladesh	2006	Potatoes	85
16	Bangladesh	2006	Poultry Meat	3
16	Bangladesh	2006	Pulses, Other	7
16	Bangladesh	2006	Rape and Mustard Oil	2
16	Bangladesh	2006	Rice (Milled Equivalent)	469
16	Bangladesh	2006	Soyabean Oil	7
16	Bangladesh	2006	Soyabeans	3
16	Bangladesh	2006	Spices, Other	3
16	Bangladesh	2006	Sugar Cane	6
16	Bangladesh	2006	Sugar, Non-Centrifugal	6
16	Bangladesh	2006	Sugar, Refined Equivalent	14
16	Bangladesh	2006	Sweet Potatoes	5
16	Bangladesh	2006	Sweeteners, Other	3
16	Bangladesh	2006	Tea	1
16	Bangladesh	2006	Tomatoes	2
16	Bangladesh	2006	Vegetables, Other	34
16	Bangladesh	2006	Wheat	50
16	Bangladesh	2007	Apples	1
16	Bangladesh	2007	Bananas	17
16	Bangladesh	2007	Beans	1
16	Bangladesh	2007	Bovine Meat	4
16	Bangladesh	2007	Cassava	1
16	Bangladesh	2007	Crustaceans	1
16	Bangladesh	2007	Demersal Fish	2
16	Bangladesh	2007	Eggs	4
16	Bangladesh	2007	Freshwater Fish	38
16	Bangladesh	2007	Fruits, Other	38
16	Bangladesh	2007	Grapefruit	1
16	Bangladesh	2007	Lemons, Limes	1
16	Bangladesh	2007	Maize	1
16	Bangladesh	2007	Marine Fish, Other	2
16	Bangladesh	2007	Milk, Whole	45
16	Bangladesh	2007	Mutton & Goat Meat	3
16	Bangladesh	2007	Nuts	2
16	Bangladesh	2007	Offals, Edible	2
16	Bangladesh	2007	Onions	18
16	Bangladesh	2007	Palm Oil	8

FAO country code	Country	Reference year	Food item	Gross food supply (g *capita*$^{-1}$ d^{-1})
16	Bangladesh	2007	Peas	6
16	Bangladesh	2007	Pelagic Fish	1
16	Bangladesh	2007	Pimento	3
16	Bangladesh	2007	Pineapples	4
16	Bangladesh	2007	Potatoes	79
16	Bangladesh	2007	Poultry Meat	4
16	Bangladesh	2007	Pulses, Other	7
16	Bangladesh	2007	Rape and Mustard Oil	2
16	Bangladesh	2007	Rice (Milled Equivalent)	482
16	Bangladesh	2007	Soyabean Oil	6
16	Bangladesh	2007	Soyabeans	3
16	Bangladesh	2007	Spices, Other	4
16	Bangladesh	2007	Sugar Cane	6
16	Bangladesh	2007	Sugar, Non-Centrifugal	6
16	Bangladesh	2007	Sugar, Refined Equivalent	14
16	Bangladesh	2007	Sweet Potatoes	5
16	Bangladesh	2007	Sweeteners, Other	2
16	Bangladesh	2007	Tea	1
16	Bangladesh	2007	Tomatoes	2
16	Bangladesh	2007	Vegetables, Other	38
16	Bangladesh	2007	Wheat	43
16	Bangladesh	2008	Apples	2
16	Bangladesh	2008	Bananas	15
16	Bangladesh	2008	Beans	1
16	Bangladesh	2008	Bovine Meat	4
16	Bangladesh	2008	Crustaceans	3
16	Bangladesh	2008	Demersal Fish	1
16	Bangladesh	2008	Eggs	4
16	Bangladesh	2008	Freshwater Fish	38
16	Bangladesh	2008	Fruits, Other	40
16	Bangladesh	2008	Grapefruit	1
16	Bangladesh	2008	Grapes	1
16	Bangladesh	2008	Lemons, Limes	1
16	Bangladesh	2008	Maize	1
16	Bangladesh	2008	Marine Fish, Other	2
16	Bangladesh	2008	Milk, Whole	46
16	Bangladesh	2008	Mutton & Goat Meat	3
16	Bangladesh	2008	Nuts	2
16	Bangladesh	2008	Offals, Edible	2
16	Bangladesh	2008	Onions	28
16	Bangladesh	2008	Oranges, Mandarines	1
16	Bangladesh	2008	Palm Oil	8
16	Bangladesh	2008	Peas	2
16	Bangladesh	2008	Pelagic Fish	1
16	Bangladesh	2008	Pimento	2
16	Bangladesh	2008	Pineapples	4
16	Bangladesh	2008	Potatoes	104
16	Bangladesh	2008	Poultry Meat	4
16	Bangladesh	2008	Pulses, Other	5
16	Bangladesh	2008	Rape and Mustard Oil	2
16	Bangladesh	2008	Rice (Milled Equivalent)	477
16	Bangladesh	2008	Soyabean Oil	5
16	Bangladesh	2008	Soyabeans	3
16	Bangladesh	2008	Spices, Other	5
16	Bangladesh	2008	Sugar Cane	5
16	Bangladesh	2008	Sugar, Non-Centrifugal	6
16	Bangladesh	2008	Sugar, Refined Equivalent	14
16	Bangladesh	2008	Sweet Potatoes	5
16	Bangladesh	2008	Sweeteners, Other	2
16	Bangladesh	2008	Tea	1
16	Bangladesh	2008	Tomatoes	3
16	Bangladesh	2008	Vegetables, Other	42
16	Bangladesh	2008	Wheat	44
16	Bangladesh	2009	Apples	3
16	Bangladesh	2009	Bananas	14
16	Bangladesh	2009	Beans	1
16	Bangladesh	2009	Bovine Meat	4
16	Bangladesh	2009	Butter, Ghee	1
16	Bangladesh	2009	Crustaceans	3
16	Bangladesh	2009	Demersal Fish	2
16	Bangladesh	2009	Eggs	3
16	Bangladesh	2009	Freshwater Fish	41
16	Bangladesh	2009	Fruits, Other	41
16	Bangladesh	2009	Grapefruit	1
16	Bangladesh	2009	Grapes	1
16	Bangladesh	2009	Lemons, Limes	1
16	Bangladesh	2009	Maize	3
16	Bangladesh	2009	Marine Fish, Other	4
16	Bangladesh	2009	Milk, Whole	49
16	Bangladesh	2009	Mutton & Goat Meat	3
16	Bangladesh	2009	Nuts	3
16	Bangladesh	2009	Offals, Edible	2
16	Bangladesh	2009	Onions	26
16	Bangladesh	2009	Oranges, Mandarines	1
16	Bangladesh	2009	Palm Oil	8
16	Bangladesh	2009	Peas	9
16	Bangladesh	2009	Pelagic Fish	1
16	Bangladesh	2009	Pimento	2
16	Bangladesh	2009	Pineapples	4
16	Bangladesh	2009	Potatoes	79
16	Bangladesh	2009	Poultry Meat	4
16	Bangladesh	2009	Pulses, Other	8
16	Bangladesh	2009	Rape and Mustard Oil	2
16	Bangladesh	2009	Rice (Milled Equivalent)	474
16	Bangladesh	2009	Soyabean Oil	6
16	Bangladesh	2009	Soyabeans	4
16	Bangladesh	2009	Spices, Other	5

FAO country code	Country	Reference year	Food item	Gross food supply (g *capita*$^{-1}$ d^{-1})
16	Bangladesh	2009	Sugar Cane	6
16	Bangladesh	2009	Sugar, Non-Centrifugal	6
16	Bangladesh	2009	Sugar, Refined Equivalent	14
16	Bangladesh	2009	Sweet Potatoes	5
16	Bangladesh	2009	Sweeteners, Other	2
16	Bangladesh	2009	Tea	1
16	Bangladesh	2009	Tomatoes	3
16	Bangladesh	2009	Vegetables, Other	44
16	Bangladesh	2009	Wheat	45
16	Bangladesh	2010	Apples	3
16	Bangladesh	2010	Bananas	13
16	Bangladesh	2010	Beans	1
16	Bangladesh	2010	Bovine Meat	4
16	Bangladesh	2010	Butter, Ghee	1
16	Bangladesh	2010	Crustaceans	4
16	Bangladesh	2010	Demersal Fish	2
16	Bangladesh	2010	Eggs	4
16	Bangladesh	2010	Freshwater Fish	42
16	Bangladesh	2010	Fruits, Other	45
16	Bangladesh	2010	Grapefruit	1
16	Bangladesh	2010	Grapes	1
16	Bangladesh	2010	Lemons, Limes	1
16	Bangladesh	2010	Maize	2
16	Bangladesh	2010	Marine Fish, Other	4
16	Bangladesh	2010	Milk, Whole	52
16	Bangladesh	2010	Mutton & Goat Meat	4
16	Bangladesh	2010	Nuts	2
16	Bangladesh	2010	Offals, Edible	2
16	Bangladesh	2010	Onions	24
16	Bangladesh	2010	Oranges, Mandarines	2
16	Bangladesh	2010	Palm Oil	8
16	Bangladesh	2010	Peas	8
16	Bangladesh	2010	Pelagic Fish	1
16	Bangladesh	2010	Pimento	2
16	Bangladesh	2010	Pineapples	4
16	Bangladesh	2010	Potatoes	121
16	Bangladesh	2010	Poultry Meat	4
16	Bangladesh	2010	Pulses, Other	8
16	Bangladesh	2010	Rape and Mustard Oil	2
16	Bangladesh	2010	Rice (Milled Equivalent)	473
16	Bangladesh	2010	Soyabean Oil	5
16	Bangladesh	2010	Soyabeans	2
16	Bangladesh	2010	Spices, Other	5
16	Bangladesh	2010	Sugar Cane	5
16	Bangladesh	2010	Sugar, Non-Centrifugal	5
16	Bangladesh	2010	Sugar, Refined Equivalent	14
16	Bangladesh	2010	Sweet Potatoes	5
16	Bangladesh	2010	Sweeteners, Other	2
16	Bangladesh	2010	Tea	1
16	Bangladesh	2010	Tomatoes	3
16	Bangladesh	2010	Vegetables, Other	44
16	Bangladesh	2010	Wheat	46
16	Bangladesh	2011	Apples	3
16	Bangladesh	2011	Bananas	13
16	Bangladesh	2011	Beans	1
16	Bangladesh	2011	Bovine Meat	4
16	Bangladesh	2011	Butter, Ghee	1
16	Bangladesh	2011	Crustaceans	3
16	Bangladesh	2011	Demersal Fish	2
16	Bangladesh	2011	Eggs	4
16	Bangladesh	2011	Freshwater Fish	46
16	Bangladesh	2011	Fruits, Other	41
16	Bangladesh	2011	Grapefruit	1
16	Bangladesh	2011	Grapes	1
16	Bangladesh	2011	Lemons, Limes	1
16	Bangladesh	2011	Maize	1
16	Bangladesh	2011	Marine Fish, Other	2
16	Bangladesh	2011	Milk, Whole	52
16	Bangladesh	2011	Mutton & Goat Meat	4
16	Bangladesh	2011	Nuts	2
16	Bangladesh	2011	Offals, Edible	2
16	Bangladesh	2011	Onions	22
16	Bangladesh	2011	Oranges, Mandarines	2
16	Bangladesh	2011	Palm Oil	8
16	Bangladesh	2011	Peas	4
16	Bangladesh	2011	Pelagic Fish	1
16	Bangladesh	2011	Pimento	3
16	Bangladesh	2011	Pineapples	4
16	Bangladesh	2011	Potatoes	124
16	Bangladesh	2011	Poultry Meat	4
16	Bangladesh	2011	Pulses, Other	6
16	Bangladesh	2011	Rape and Mustard Oil	2
16	Bangladesh	2011	Rice (Milled Equivalent)	473
16	Bangladesh	2011	Soyabean Oil	6
16	Bangladesh	2011	Soyabeans	3
16	Bangladesh	2011	Spices, Other	5
16	Bangladesh	2011	Sugar Cane	5
16	Bangladesh	2011	Sugar, Non-Centrifugal	5
16	Bangladesh	2011	Sugar, Refined Equivalent	14
16	Bangladesh	2011	Sweet Potatoes	5
16	Bangladesh	2011	Sweeteners, Other	3
16	Bangladesh	2011	Tea	1
16	Bangladesh	2011	Tomatoes	4
16	Bangladesh	2011	Vegetables, Other	45
16	Bangladesh	2011	Wheat	47
57	Belarus	1992	Apples	55
57	Belarus	1992	Barley	35

FAO country code	Country	Reference year	Food item	Gross food supply (g *capita*$^{-1}$ d^{-1})
57	Belarus	1992	Beer	74
57	Belarus	1992	Beverages, Alcoholic	33
57	Belarus	1992	Beverages, Fermented	7
57	Belarus	1992	Bovine Meat	99
57	Belarus	1992	Butter, Ghee	21
57	Belarus	1992	Cereals, Other	1
57	Belarus	1992	Cheese	15
57	Belarus	1992	Cocoa Beans	1
57	Belarus	1992	Cream	7
57	Belarus	1992	Eggs	47
57	Belarus	1992	Fats, Animals, Raw	2
57	Belarus	1992	Freshwater Fish	3
57	Belarus	1992	Fruits, Other	48
57	Belarus	1992	Honey	1
57	Belarus	1992	Meat, Other	2
57	Belarus	1992	Milk, Whole	248
57	Belarus	1992	Mutton & Goat Meat	1
57	Belarus	1992	Nuts	2
57	Belarus	1992	Oats	36
57	Belarus	1992	Offals, Edible	4
57	Belarus	1992	Onions	14
57	Belarus	1992	Oranges, Mandarines	1
57	Belarus	1992	Pigmeat	82
57	Belarus	1992	Potatoes	468
57	Belarus	1992	Poultry Meat	32
57	Belarus	1992	Rape and Mustard Oil	3
57	Belarus	1992	Rice (Milled Equivalent)	7
57	Belarus	1992	Rye	183
57	Belarus	1992	Sugar, Refined Equivalent	92
57	Belarus	1992	Sunflowerseed Oil	9
57	Belarus	1992	Tomatoes	18
57	Belarus	1992	Vegetables, Other	179
57	Belarus	1992	Wheat	176
57	Belarus	1992	Wine	3
57	Belarus	1993	Apples	61
57	Belarus	1993	Barley	15
57	Belarus	1993	Beer	59
57	Belarus	1993	Beverages, Alcoholic	41
57	Belarus	1993	Beverages, Fermented	12
57	Belarus	1993	Bovine Meat	92
57	Belarus	1993	Butter, Ghee	17
57	Belarus	1993	Cereals, Other	1
57	Belarus	1993	Cheese	14
57	Belarus	1993	Cocoa Beans	1
57	Belarus	1993	Cream	5
57	Belarus	1993	Eggs	48
57	Belarus	1993	Fats, Animals, Raw	5
57	Belarus	1993	Freshwater Fish	3
57	Belarus	1993	Fruits, Other	60
57	Belarus	1993	Honey	1
57	Belarus	1993	Meat, Other	2
57	Belarus	1993	Milk, Whole	368
57	Belarus	1993	Mutton & Goat Meat	1
57	Belarus	1993	Nuts	3
57	Belarus	1993	Oats	36
57	Belarus	1993	Offals, Edible	9
57	Belarus	1993	Onions	14
57	Belarus	1993	Oranges, Mandarines	1
57	Belarus	1993	Pigmeat	72
57	Belarus	1993	Potatoes	493
57	Belarus	1993	Poultry Meat	35
57	Belarus	1993	Rape and Mustard Oil	3
57	Belarus	1993	Rice (Milled Equivalent)	7
57	Belarus	1993	Rye	203
57	Belarus	1993	Sugar, Refined Equivalent	88
57	Belarus	1993	Sunflowerseed Oil	10
57	Belarus	1993	Tomatoes	22
57	Belarus	1993	Vegetables, Other	182
57	Belarus	1993	Wheat	168
57	Belarus	1993	Wine	7
57	Belarus	1994	Apples	60
57	Belarus	1994	Bananas	1
57	Belarus	1994	Barley	18
57	Belarus	1994	Beer	42
57	Belarus	1994	Beverages, Alcoholic	39
57	Belarus	1994	Beverages, Fermented	15
57	Belarus	1994	Bovine Meat	82
57	Belarus	1994	Butter, Ghee	16
57	Belarus	1994	Cereals, Other	1
57	Belarus	1994	Cheese	13
57	Belarus	1994	Cocoa Beans	2
57	Belarus	1994	Cream	3
57	Belarus	1994	Crustaceans	1
57	Belarus	1994	Eggs	47
57	Belarus	1994	Fats, Animals, Raw	10
57	Belarus	1994	Freshwater Fish	1
57	Belarus	1994	Fruits, Other	45
57	Belarus	1994	Honey	1
57	Belarus	1994	Marine Fish, Other	1
57	Belarus	1994	Meat, Other	1
57	Belarus	1994	Milk, Whole	496
57	Belarus	1994	Mutton & Goat Meat	1
57	Belarus	1994	Nuts	2
57	Belarus	1994	Oats	29
57	Belarus	1994	Offals, Edible	9
57	Belarus	1994	Onions	16
57	Belarus	1994	Oranges, Mandarines	2

FAO country code	Country	Reference year	Food item	Gross food supply (g $capita^{-1}$ d^{-1})
57	Belarus	1994	Pelagic Fish	1
57	Belarus	1994	Pigmeat	68
57	Belarus	1994	Potatoes	488
57	Belarus	1994	Poultry Meat	25
57	Belarus	1994	Rape and Mustard Oil	2
57	Belarus	1994	Rice (Milled Equivalent)	4
57	Belarus	1994	Rye	210
57	Belarus	1994	Sugar, Refined Equivalent	73
57	Belarus	1994	Sunflowerseed Oil	11
57	Belarus	1994	Tomatoes	23
57	Belarus	1994	Vegetables, Other	182
57	Belarus	1994	Wheat	166
57	Belarus	1994	Wine	10
57	Belarus	1995	Apples	38
57	Belarus	1995	Bananas	10
57	Belarus	1995	Barley	11
57	Belarus	1995	Beer	38
57	Belarus	1995	Beverages, Alcoholic	38
57	Belarus	1995	Beverages, Fermented	14
57	Belarus	1995	Bovine Meat	73
57	Belarus	1995	Butter, Ghee	15
57	Belarus	1995	Cereals, Other	1
57	Belarus	1995	Cheese	8
57	Belarus	1995	Cocoa Beans	1
57	Belarus	1995	Cream	1
57	Belarus	1995	Eggs	46
57	Belarus	1995	Fats, Animals, Raw	11
57	Belarus	1995	Freshwater Fish	2
57	Belarus	1995	Fruits, Other	53
57	Belarus	1995	Honey	1
57	Belarus	1995	Lemons, Limes	3
57	Belarus	1995	Marine Fish, Other	14
57	Belarus	1995	Meat, Other	1
57	Belarus	1995	Milk, Whole	458
57	Belarus	1995	Mutton & Goat Meat	1
57	Belarus	1995	Nuts	2
57	Belarus	1995	Oats	29
57	Belarus	1995	Offals, Edible	10
57	Belarus	1995	Onions	14
57	Belarus	1995	Oranges, Mandarines	9
57	Belarus	1995	Pigmeat	70
57	Belarus	1995	Potatoes	499
57	Belarus	1995	Poultry Meat	19
57	Belarus	1995	Rape and Mustard Oil	3
57	Belarus	1995	Rice (Milled Equivalent)	1
57	Belarus	1995	Rye	214
57	Belarus	1995	Sugar, Refined Equivalent	96
57	Belarus	1995	Sunflowerseed Oil	14
57	Belarus	1995	Tomatoes	27
57	Belarus	1995	Vegetables, Other	189
57	Belarus	1995	Wheat	168
57	Belarus	1995	Wine	7
57	Belarus	1996	Apples	44
57	Belarus	1996	Bananas	12
57	Belarus	1996	Barley	13
57	Belarus	1996	Beer	47
57	Belarus	1996	Beverages, Alcoholic	45
57	Belarus	1996	Beverages, Fermented	21
57	Belarus	1996	Bovine Meat	71
57	Belarus	1996	Butter, Ghee	15
57	Belarus	1996	Cereals, Other	4
57	Belarus	1996	Cheese	9
57	Belarus	1996	Cocoa Beans	1
57	Belarus	1996	Cream	1
57	Belarus	1996	Eggs	46
57	Belarus	1996	Fats, Animals, Raw	12
57	Belarus	1996	Freshwater Fish	2
57	Belarus	1996	Fruits, Other	39
57	Belarus	1996	Honey	1
57	Belarus	1996	Lemons, Limes	1
57	Belarus	1996	Marine Fish, Other	16
57	Belarus	1996	Meat, Other	2
57	Belarus	1996	Milk, Whole	455
57	Belarus	1996	Mutton & Goat Meat	1
57	Belarus	1996	Nuts	3
57	Belarus	1996	Oats	30
57	Belarus	1996	Offals, Edible	15
57	Belarus	1996	Onions	16
57	Belarus	1996	Oranges, Mandarines	12
57	Belarus	1996	Pigmeat	72
57	Belarus	1996	Potatoes	515
57	Belarus	1996	Poultry Meat	17
57	Belarus	1996	Rape and Mustard Oil	1
57	Belarus	1996	Rice (Milled Equivalent)	4
57	Belarus	1996	Rye	202
57	Belarus	1996	Sugar, Refined Equivalent	97
57	Belarus	1996	Sunflowerseed Oil	16
57	Belarus	1996	Tomatoes	32
57	Belarus	1996	Vegetables, Other	195
57	Belarus	1996	Wheat	175
57	Belarus	1996	Wine	8
57	Belarus	1997	Apples	49
57	Belarus	1997	Bananas	10
57	Belarus	1997	Barley	8
57	Belarus	1997	Beer	56
57	Belarus	1997	Beverages, Alcoholic	43
57	Belarus	1997	Beverages, Fermented	31

FAO country code	Country	Reference year	Food item	Gross food supply (g *capita*$^{-1}$ d^{-1})
57	Belarus	1997	Bovine Meat	68
57	Belarus	1997	Butter, Ghee	15
57	Belarus	1997	Cereals, Other	10
57	Belarus	1997	Cheese	11
57	Belarus	1997	Cocoa Beans	2
57	Belarus	1997	Cream	1
57	Belarus	1997	Eggs	46
57	Belarus	1997	Fats, Animals, Raw	11
57	Belarus	1997	Freshwater Fish	1
57	Belarus	1997	Fruits, Other	37
57	Belarus	1997	Honey	1
57	Belarus	1997	Lemons, Limes	1
57	Belarus	1997	Marine Fish, Other	25
57	Belarus	1997	Meat, Other	2
57	Belarus	1997	Milk, Whole	353
57	Belarus	1997	Mutton & Goat Meat	1
57	Belarus	1997	Nuts	3
57	Belarus	1997	Oats	32
57	Belarus	1997	Offals, Edible	11
57	Belarus	1997	Onions	18
57	Belarus	1997	Oranges, Mandarines	9
57	Belarus	1997	Pelagic Fish	4
57	Belarus	1997	Pigmeat	77
57	Belarus	1997	Potatoes	499
57	Belarus	1997	Poultry Meat	20
57	Belarus	1997	Rape and Mustard Oil	3
57	Belarus	1997	Rice (Milled Equivalent)	3
57	Belarus	1997	Rye	194
57	Belarus	1997	Sugar, Refined Equivalent	105
57	Belarus	1997	Sunflowerseed Oil	15
57	Belarus	1997	Tomatoes	26
57	Belarus	1997	Vegetables, Other	200
57	Belarus	1997	Wheat	182
57	Belarus	1997	Wine	8
57	Belarus	1998	Apples	17
57	Belarus	1998	Bananas	4
57	Belarus	1998	Barley	9
57	Belarus	1998	Beer	59
57	Belarus	1998	Beverages, Alcoholic	32
57	Belarus	1998	Beverages, Fermented	18
57	Belarus	1998	Bovine Meat	71
57	Belarus	1998	Butter, Ghee	15
57	Belarus	1998	Cereals, Other	5
57	Belarus	1998	Cheese	12
57	Belarus	1998	Cocoa Beans	1
57	Belarus	1998	Cream	1
57	Belarus	1998	Demersal Fish	6
57	Belarus	1998	Eggs	42
57	Belarus	1998	Fats, Animals, Raw	11
57	Belarus	1998	Freshwater Fish	2
57	Belarus	1998	Fruits, Other	22
57	Belarus	1998	Grapefruit	1
57	Belarus	1998	Grapes	2
57	Belarus	1998	Honey	1
57	Belarus	1998	Lemons, Limes	1
57	Belarus	1998	Marine Fish, Other	1
57	Belarus	1998	Meat, Other	4
57	Belarus	1998	Milk, Whole	422
57	Belarus	1998	Millet	1
57	Belarus	1998	Mutton & Goat Meat	1
57	Belarus	1998	Nuts	4
57	Belarus	1998	Oats	21
57	Belarus	1998	Offals, Edible	12
57	Belarus	1998	Onions	19
57	Belarus	1998	Oranges, Mandarines	8
57	Belarus	1998	Pelagic Fish	11
57	Belarus	1998	Pigmeat	83
57	Belarus	1998	Potatoes	475
57	Belarus	1998	Poultry Meat	16
57	Belarus	1998	Rape and Mustard Oil	6
57	Belarus	1998	Rice (Milled Equivalent)	5
57	Belarus	1998	Rye	170
57	Belarus	1998	Sugar, Refined Equivalent	112
57	Belarus	1998	Sunflowerseed Oil	11
57	Belarus	1998	Sweeteners, Other	1
57	Belarus	1998	Tomatoes	28
57	Belarus	1998	Vegetables, Other	195
57	Belarus	1998	Wheat	201
57	Belarus	1998	Wine	8
57	Belarus	1999	Apples	39
57	Belarus	1999	Bananas	5
57	Belarus	1999	Barley	10
57	Belarus	1999	Beer	61
57	Belarus	1999	Beverages, Alcoholic	36
57	Belarus	1999	Beverages, Fermented	3
57	Belarus	1999	Bovine Meat	69
57	Belarus	1999	Butter, Ghee	13
57	Belarus	1999	Cereals, Other	5
57	Belarus	1999	Cheese	12
57	Belarus	1999	Cocoa Beans	2
57	Belarus	1999	Coffee	1
57	Belarus	1999	Cream	1
57	Belarus	1999	Demersal Fish	4
57	Belarus	1999	Eggs	36
57	Belarus	1999	Fats, Animals, Raw	12
57	Belarus	1999	Freshwater Fish	3
57	Belarus	1999	Fruits, Other	18

FAO countrycode	Country	Reference year	Food item	Gross food supply (g $capita^{-1}$ d^{-1})
57	Belarus	1999	Grapes	1
57	Belarus	1999	Honey	1
57	Belarus	1999	Lemons, Limes	1
57	Belarus	1999	Maize	1
57	Belarus	1999	Marine Fish, Other	1
57	Belarus	1999	Meat, Other	4
57	Belarus	1999	Milk, Whole	365
57	Belarus	1999	Mutton & Goat Meat	1
57	Belarus	1999	Nuts	4
57	Belarus	1999	Oats	24
57	Belarus	1999	Offals, Edible	11
57	Belarus	1999	Onions	20
57	Belarus	1999	Oranges, Mandarines	5
57	Belarus	1999	Pelagic Fish	10
57	Belarus	1999	Pigmeat	82
57	Belarus	1999	Potatoes	467
57	Belarus	1999	Poultry Meat	21
57	Belarus	1999	Rape and Mustard Oil	10
57	Belarus	1999	Rice (Milled Equivalent)	5
57	Belarus	1999	Rye	139
57	Belarus	1999	Soyabean Oil	1
57	Belarus	1999	Sugar, Refined Equivalent	92
57	Belarus	1999	Sunflowerseed Oil	11
57	Belarus	1999	Sweeteners, Other	1
57	Belarus	1999	Tomatoes	24
57	Belarus	1999	Vegetables, Other	205
57	Belarus	1999	Wheat	219
57	Belarus	1999	Wine	5
57	Belarus	2000	Apples	30
57	Belarus	2000	Bananas	8
57	Belarus	2000	Barley	12
57	Belarus	2000	Beer	53
57	Belarus	2000	Beverages, Alcoholic	29
57	Belarus	2000	Beverages, Fermented	7
57	Belarus	2000	Bovine Meat	56
57	Belarus	2000	Butter, Ghee	13
57	Belarus	2000	Cereals, Other	6
57	Belarus	2000	Cheese	10
57	Belarus	2000	Cocoa Beans	2
57	Belarus	2000	Coconut Oil	1
57	Belarus	2000	Cream	2
57	Belarus	2000	Demersal Fish	2
57	Belarus	2000	Eggs	34
57	Belarus	2000	Fats, Animals, Raw	10
57	Belarus	2000	Freshwater Fish	3
57	Belarus	2000	Fruits, Other	24
57	Belarus	2000	Grapes	1
57	Belarus	2000	Groundnuts (Shelled Eq)	1
57	Belarus	2000	Honey	1
57	Belarus	2000	Lemons, Limes	2
57	Belarus	2000	Maize	1
57	Belarus	2000	Marine Fish, Other	9
57	Belarus	2000	Meat, Other	3
57	Belarus	2000	Milk, Whole	245
57	Belarus	2000	Millet	1
57	Belarus	2000	Mutton & Goat Meat	1
57	Belarus	2000	Nuts	5
57	Belarus	2000	Oats	25
57	Belarus	2000	Offals, Edible	13
57	Belarus	2000	Onions	19
57	Belarus	2000	Oranges, Mandarines	7
57	Belarus	2000	Pelagic Fish	13
57	Belarus	2000	Pigmeat	78
57	Belarus	2000	Potatoes	478
57	Belarus	2000	Poultry Meat	25
57	Belarus	2000	Rape and Mustard Oil	7
57	Belarus	2000	Rice (Milled Equivalent)	5
57	Belarus	2000	Rye	109
57	Belarus	2000	Soyabean Oil	1
57	Belarus	2000	Soyabeans	1
57	Belarus	2000	Sugar, Refined Equivalent	94
57	Belarus	2000	Sunflowerseed Oil	15
57	Belarus	2000	Sweeteners, Other	4
57	Belarus	2000	Tea	1
57	Belarus	2000	Tomatoes	36
57	Belarus	2000	Vegetables, Other	205
57	Belarus	2000	Wheat	230
57	Belarus	2000	Wine	7
57	Belarus	2001	Apples	47
57	Belarus	2001	Bananas	5
57	Belarus	2001	Barley	13
57	Belarus	2001	Beer	52
57	Belarus	2001	Beverages, Alcoholic	27
57	Belarus	2001	Beverages, Fermented	7
57	Belarus	2001	Bovine Meat	57
57	Belarus	2001	Butter, Ghee	11
57	Belarus	2001	Cereals, Other	9
57	Belarus	2001	Cheese	13
57	Belarus	2001	Cocoa Beans	2
57	Belarus	2001	Coconut Oil	1
57	Belarus	2001	Cream	2
57	Belarus	2001	Demersal Fish	8
57	Belarus	2001	Eggs	35
57	Belarus	2001	Fats, Animals, Raw	9
57	Belarus	2001	Freshwater Fish	2
57	Belarus	2001	Fruits, Other	30
57	Belarus	2001	Grapefruit	1

FAO countrycode	Country	Reference year	Food item	Gross food supply (g *capita*$^{-1}$ d^{-1})
57	Belarus	2001	Grapes	2
57	Belarus	2001	Honey	1
57	Belarus	2001	Lemons, Limes	1
57	Belarus	2001	Marine Fish, Other	4
57	Belarus	2001	Meat, Other	3
57	Belarus	2001	Milk, Whole	286
57	Belarus	2001	Mutton & Goat Meat	1
57	Belarus	2001	Nuts	5
57	Belarus	2001	Oats	24
57	Belarus	2001	Offals, Edible	14
57	Belarus	2001	Onions	21
57	Belarus	2001	Oranges, Mandarines	9
57	Belarus	2001	Pelagic Fish	22
57	Belarus	2001	Pigmeat	76
57	Belarus	2001	Potatoes	473
57	Belarus	2001	Poultry Meat	26
57	Belarus	2001	Rape and Mustard Oil	4
57	Belarus	2001	Rice (Milled Equivalent)	6
57	Belarus	2001	Rye	106
57	Belarus	2001	Soyabean Oil	4
57	Belarus	2001	Sugar, Refined Equivalent	97
57	Belarus	2001	Sunflowerseed Oil	23
57	Belarus	2001	Sweeteners, Other	4
57	Belarus	2001	Tomatoes	37
57	Belarus	2001	Vegetables, Other	216
57	Belarus	2001	Wheat	212
57	Belarus	2001	Wine	7
57	Belarus	2002	Apples	73
57	Belarus	2002	Bananas	4
57	Belarus	2002	Barley	12
57	Belarus	2002	Beer	50
57	Belarus	2002	Beverages, Alcoholic	26
57	Belarus	2002	Beverages, Fermented	10
57	Belarus	2002	Bovine Meat	54
57	Belarus	2002	Butter, Ghee	10
57	Belarus	2002	Cereals, Other	11
57	Belarus	2002	Cheese	9
57	Belarus	2002	Citrus, Other	1
57	Belarus	2002	Cocoa Beans	2
57	Belarus	2002	Coconut Oil	1
57	Belarus	2002	Cream	2
57	Belarus	2002	Demersal Fish	8
57	Belarus	2002	Eggs	35
57	Belarus	2002	Fats, Animals, Raw	9
57	Belarus	2002	Freshwater Fish	4
57	Belarus	2002	Fruits, Other	44
57	Belarus	2002	Grapefruit	3
57	Belarus	2002	Grapes	1
57	Belarus	2002	Groundnuts (Shelled Eq)	1
57	Belarus	2002	Honey	1
57	Belarus	2002	Lemons, Limes	1
57	Belarus	2002	Maize	1
57	Belarus	2002	Marine Fish, Other	7
57	Belarus	2002	Meat, Other	1
57	Belarus	2002	Milk, Whole	290
57	Belarus	2002	Nuts	5
57	Belarus	2002	Oats	25
57	Belarus	2002	Offals, Edible	13
57	Belarus	2002	Onions	23
57	Belarus	2002	Oranges, Mandarines	19
57	Belarus	2002	Pelagic Fish	22
57	Belarus	2002	Pigmeat	78
57	Belarus	2002	Pineapples	2
57	Belarus	2002	Potatoes	469
57	Belarus	2002	Poultry Meat	26
57	Belarus	2002	Rape and Mustard Oil	4
57	Belarus	2002	Rice (Milled Equivalent)	7
57	Belarus	2002	Rye	90
57	Belarus	2002	Soyabean Oil	7
57	Belarus	2002	Sugar, Refined Equivalent	96
57	Belarus	2002	Sunflowerseed Oil	20
57	Belarus	2002	Sweeteners, Other	5
57	Belarus	2002	Tomatoes	37
57	Belarus	2002	Vegetables, Other	228
57	Belarus	2002	Wheat	201
57	Belarus	2002	Wine	10
57	Belarus	2003	Apples	77
57	Belarus	2003	Bananas	6
57	Belarus	2003	Barley	14
57	Belarus	2003	Beer	52
57	Belarus	2003	Beverages, Alcoholic	22
57	Belarus	2003	Beverages, Fermented	8
57	Belarus	2003	Bovine Meat	47
57	Belarus	2003	Butter, Ghee	10
57	Belarus	2003	Cereals, Other	6
57	Belarus	2003	Cheese	8
57	Belarus	2003	Cocoa Beans	2
57	Belarus	2003	Coconut Oil	1
57	Belarus	2003	Coffee	1
57	Belarus	2003	Cream	2
57	Belarus	2003	Demersal Fish	9
57	Belarus	2003	Eggs	35
57	Belarus	2003	Fats, Animals, Raw	10
57	Belarus	2003	Freshwater Fish	3
57	Belarus	2003	Fruits, Other	32
57	Belarus	2003	Grapefruit	3
57	Belarus	2003	Grapes	1

FAO country code	Country	Reference year	Food item	Gross food supply (g *capita*$^{-1}$ d^{-1})
57	Belarus	2003	Honey	1
57	Belarus	2003	Lemons, Limes	1
57	Belarus	2003	Maize	1
57	Belarus	2003	Marine Fish, Other	6
57	Belarus	2003	Meat, Other	1
57	Belarus	2003	Milk, Whole	225
57	Belarus	2003	Mutton & Goat Meat	1
57	Belarus	2003	Nuts	7
57	Belarus	2003	Oats	25
57	Belarus	2003	Offals, Edible	12
57	Belarus	2003	Onions	37
57	Belarus	2003	Oranges, Mandarines	16
57	Belarus	2003	Pelagic Fish	21
57	Belarus	2003	Pigmeat	89
57	Belarus	2003	Pineapples	4
57	Belarus	2003	Potatoes	474
57	Belarus	2003	Poultry Meat	25
57	Belarus	2003	Rape and Mustard Oil	2
57	Belarus	2003	Rice (Milled Equivalent)	15
57	Belarus	2003	Rye	87
57	Belarus	2003	Soyabean Oil	2
57	Belarus	2003	Sugar, Refined Equivalent	82
57	Belarus	2003	Sunflowerseed Oil	19
57	Belarus	2003	Sweeteners, Other	1
57	Belarus	2003	Tea	1
57	Belarus	2003	Tomatoes	61
57	Belarus	2003	Vegetables, Other	199
57	Belarus	2003	Wheat	193
57	Belarus	2003	Wine	11
57	Belarus	2004	Apples	73
57	Belarus	2004	Bananas	3
57	Belarus	2004	Barley	13
57	Belarus	2004	Beer	62
57	Belarus	2004	Beverages, Alcoholic	20
57	Belarus	2004	Beverages, Fermented	8
57	Belarus	2004	Bovine Meat	49
57	Belarus	2004	Butter, Ghee	10
57	Belarus	2004	Cereals, Other	12
57	Belarus	2004	Cheese	8
57	Belarus	2004	Cocoa Beans	2
57	Belarus	2004	Coconut Oil	1
57	Belarus	2004	Coffee	2
57	Belarus	2004	Cream	1
57	Belarus	2004	Demersal Fish	12
57	Belarus	2004	Eggs	37
57	Belarus	2004	Fats, Animals, Raw	11
57	Belarus	2004	Freshwater Fish	4
57	Belarus	2004	Fruits, Other	45
57	Belarus	2004	Grapefruit	2
57	Belarus	2004	Grapes	1
57	Belarus	2004	Groundnuts (Shelled Eq)	1
57	Belarus	2004	Honey	1
57	Belarus	2004	Lemons, Limes	1
57	Belarus	2004	Maize	1
57	Belarus	2004	Marine Fish, Other	5
57	Belarus	2004	Meat, Other	3
57	Belarus	2004	Milk, Whole	192
57	Belarus	2004	Nuts	6
57	Belarus	2004	Oats	32
57	Belarus	2004	Offals, Edible	15
57	Belarus	2004	Onions	45
57	Belarus	2004	Oranges, Mandarines	16
57	Belarus	2004	Pelagic Fish	21
57	Belarus	2004	Pigmeat	79
57	Belarus	2004	Pineapples	3
57	Belarus	2004	Potatoes	509
57	Belarus	2004	Poultry Meat	31
57	Belarus	2004	Rape and Mustard Oil	2
57	Belarus	2004	Rice (Milled Equivalent)	8
57	Belarus	2004	Rye	91
57	Belarus	2004	Soyabean Oil	2
57	Belarus	2004	Sugar, Refined Equivalent	83
57	Belarus	2004	Sunflowerseed Oil	20
57	Belarus	2004	Sweeteners, Other	5
57	Belarus	2004	Tea	1
57	Belarus	2004	Tomatoes	70
57	Belarus	2004	Vegetables, Other	213
57	Belarus	2004	Wheat	198
57	Belarus	2004	Wine	16
57	Belarus	2005	Apples	63
57	Belarus	2005	Bananas	5
57	Belarus	2005	Barley	13
57	Belarus	2005	Beer	89
57	Belarus	2005	Beverages, Alcoholic	24
57	Belarus	2005	Beverages, Fermented	13
57	Belarus	2005	Bovine Meat	59
57	Belarus	2005	Butter, Ghee	10
57	Belarus	2005	Cereals, Other	12
57	Belarus	2005	Cheese	9
57	Belarus	2005	Cocoa Beans	2
57	Belarus	2005	Coconut Oil	1
57	Belarus	2005	Coffee	3
57	Belarus	2005	Cream	2
57	Belarus	2005	Demersal Fish	13
57	Belarus	2005	Eggs	40
57	Belarus	2005	Fats, Animals, Raw	10
57	Belarus	2005	Freshwater Fish	4

FAO country code	Country	Reference year	Food item	Gross food supply (g $capita^{-1}$ d^{-1})
57	Belarus	2005	Fruits, Other	53
57	Belarus	2005	Grapefruit	2
57	Belarus	2005	Grapes	2
57	Belarus	2005	Groundnuts (Shelled Eq)	1
57	Belarus	2005	Honey	1
57	Belarus	2005	Lemons, Limes	1
57	Belarus	2005	Maize	1
57	Belarus	2005	Marine Fish, Other	5
57	Belarus	2005	Meat, Other	3
57	Belarus	2005	Milk, Whole	243
57	Belarus	2005	Nuts	7
57	Belarus	2005	Oats	27
57	Belarus	2005	Offals, Edible	17
57	Belarus	2005	Olives	1
57	Belarus	2005	Onions	44
57	Belarus	2005	Oranges, Mandarines	17
57	Belarus	2005	Pelagic Fish	21
57	Belarus	2005	Pigmeat	71
57	Belarus	2005	Pineapples	2
57	Belarus	2005	Potatoes	503
57	Belarus	2005	Poultry Meat	36
57	Belarus	2005	Rape and Mustard Oil	6
57	Belarus	2005	Rice (Milled Equivalent)	7
57	Belarus	2005	Rye	92
57	Belarus	2005	Soyabean Oil	3
57	Belarus	2005	Sugar, Refined Equivalent	91
57	Belarus	2005	Sunflowerseed Oil	22
57	Belarus	2005	Sweeteners, Other	5
57	Belarus	2005	Tea	1
57	Belarus	2005	Tomatoes	79
57	Belarus	2005	Vegetables, Other	234
57	Belarus	2005	Wheat	186
57	Belarus	2005	Wine	18
57	Belarus	2006	Apples	69
57	Belarus	2006	Bananas	7
57	Belarus	2006	Barley	12
57	Belarus	2006	Beer	115
57	Belarus	2006	Beverages, Alcoholic	31
57	Belarus	2006	Beverages, Fermented	6
57	Belarus	2006	Bovine Meat	62
57	Belarus	2006	Butter, Ghee	10
57	Belarus	2006	Cereals, Other	11
57	Belarus	2006	Cheese	10
57	Belarus	2006	Cocoa Beans	4
57	Belarus	2006	Coconut Oil	1
57	Belarus	2006	Coffee	4
57	Belarus	2006	Cream	2
57	Belarus	2006	Demersal Fish	12
57	Belarus	2006	Eggs	43
57	Belarus	2006	Fats, Animals, Raw	10
57	Belarus	2006	Freshwater Fish	5
57	Belarus	2006	Fruits, Other	77
57	Belarus	2006	Grapefruit	2
57	Belarus	2006	Grapes	3
57	Belarus	2006	Groundnuts (Shelled Eq)	1
57	Belarus	2006	Honey	1
57	Belarus	2006	Lemons, Limes	2
57	Belarus	2006	Maize	1
57	Belarus	2006	Marine Fish, Other	3
57	Belarus	2006	Meat, Other	1
57	Belarus	2006	Milk, Whole	183
57	Belarus	2006	Nuts	5
57	Belarus	2006	Oats	25
57	Belarus	2006	Offals, Edible	15
57	Belarus	2006	Olives	1
57	Belarus	2006	Onions	48
57	Belarus	2006	Oranges, Mandarines	21
57	Belarus	2006	Pelagic Fish	23
57	Belarus	2006	Pigmeat	80
57	Belarus	2006	Pineapples	2
57	Belarus	2006	Potatoes	525
57	Belarus	2006	Poultry Meat	45
57	Belarus	2006	Rape and Mustard Oil	4
57	Belarus	2006	Rice (Milled Equivalent)	9
57	Belarus	2006	Rye	82
57	Belarus	2006	Soyabean Oil	2
57	Belarus	2006	Sugar, Refined Equivalent	83
57	Belarus	2006	Sunflowerseed Oil	31
57	Belarus	2006	Sweeteners, Other	5
57	Belarus	2006	Tea	1
57	Belarus	2006	Tomatoes	82
57	Belarus	2006	Vegetables, Other	249
57	Belarus	2006	Wheat	191
57	Belarus	2006	Wine	18
57	Belarus	2007	Apples	81
57	Belarus	2007	Bananas	8
57	Belarus	2007	Barley	12
57	Belarus	2007	Beer	137
57	Belarus	2007	Beverages, Alcoholic	36
57	Belarus	2007	Beverages, Fermented	6
57	Belarus	2007	Bovine Meat	61
57	Belarus	2007	Butter, Ghee	9
57	Belarus	2007	Cephalopods	1
57	Belarus	2007	Cereals, Other	10
57	Belarus	2007	Cheese	9
57	Belarus	2007	Cocoa Beans	4
57	Belarus	2007	Coconut Oil	1

FAO country code	Country	Reference year	Food item	Gross food supply (g *capita*$^{-1}$ d^{-1})
57	Belarus	2007	Coffee	5
57	Belarus	2007	Cream	2
57	Belarus	2007	Demersal Fish	9
57	Belarus	2007	Eggs	42
57	Belarus	2007	Fats, Animals, Raw	11
57	Belarus	2007	Freshwater Fish	7
57	Belarus	2007	Fruits, Other	60
57	Belarus	2007	Grapefruit	4
57	Belarus	2007	Grapes	3
57	Belarus	2007	Groundnuts (Shelled Eq)	1
57	Belarus	2007	Honey	1
57	Belarus	2007	Lemons, Limes	2
57	Belarus	2007	Maize	1
57	Belarus	2007	Marine Fish, Other	3
57	Belarus	2007	Meat, Other	1
57	Belarus	2007	Milk, Whole	208
57	Belarus	2007	Nuts	5
57	Belarus	2007	Oats	26
57	Belarus	2007	Offals, Edible	14
57	Belarus	2007	Olives	1
57	Belarus	2007	Onions	50
57	Belarus	2007	Oranges, Mandarines	22
57	Belarus	2007	Pelagic Fish	26
57	Belarus	2007	Pigmeat	90
57	Belarus	2007	Pineapples	3
57	Belarus	2007	Potatoes	526
57	Belarus	2007	Poultry Meat	49
57	Belarus	2007	Rape and Mustard Oil	6
57	Belarus	2007	Rice (Milled Equivalent)	9
57	Belarus	2007	Rye	86
57	Belarus	2007	Soyabean Oil	3
57	Belarus	2007	Sugar, Refined Equivalent	91
57	Belarus	2007	Sunflowerseed Oil	32
57	Belarus	2007	Sweeteners, Other	4
57	Belarus	2007	Tea	2
57	Belarus	2007	Tomatoes	90
57	Belarus	2007	Vegetables, Other	249
57	Belarus	2007	Wheat	182
57	Belarus	2007	Wine	19
57	Belarus	2008	Apples	67
57	Belarus	2008	Bananas	12
57	Belarus	2008	Barley	12
57	Belarus	2008	Beer	142
57	Belarus	2008	Beverages, Alcoholic	41
57	Belarus	2008	Beverages, Fermented	6
57	Belarus	2008	Bovine Meat	58
57	Belarus	2008	Butter, Ghee	11
57	Belarus	2008	Cephalopods	1
57	Belarus	2008	Cereals, Other	11
57	Belarus	2008	Cheese	11
57	Belarus	2008	Cocoa Beans	5
57	Belarus	2008	Coconut Oil	1
57	Belarus	2008	Coffee	6
57	Belarus	2008	Cream	3
57	Belarus	2008	Crustaceans	1
57	Belarus	2008	Demersal Fish	9
57	Belarus	2008	Eggs	43
57	Belarus	2008	Fats, Animals, Raw	11
57	Belarus	2008	Freshwater Fish	10
57	Belarus	2008	Fruits, Other	72
57	Belarus	2008	Grapefruit	4
57	Belarus	2008	Grapes	3
57	Belarus	2008	Groundnuts (Shelled Eq)	1
57	Belarus	2008	Honey	1
57	Belarus	2008	Lemons, Limes	2
57	Belarus	2008	Maize	1
57	Belarus	2008	Marine Fish, Other	2
57	Belarus	2008	Meat, Other	1
57	Belarus	2008	Milk, Whole	105
57	Belarus	2008	Nuts	6
57	Belarus	2008	Oats	26
57	Belarus	2008	Offals, Edible	17
57	Belarus	2008	Olives	1
57	Belarus	2008	Onions	57
57	Belarus	2008	Oranges, Mandarines	21
57	Belarus	2008	Pelagic Fish	27
57	Belarus	2008	Pigmeat	95
57	Belarus	2008	Pineapples	3
57	Belarus	2008	Potatoes	529
57	Belarus	2008	Poultry Meat	57
57	Belarus	2008	Rape and Mustard Oil	12
57	Belarus	2008	Rice (Milled Equivalent)	9
57	Belarus	2008	Rye	87
57	Belarus	2008	Soyabean Oil	7
57	Belarus	2008	Sugar, Refined Equivalent	99
57	Belarus	2008	Sunflowerseed Oil	22
57	Belarus	2008	Sweeteners, Other	4
57	Belarus	2008	Tea	2
57	Belarus	2008	Tomatoes	91
57	Belarus	2008	Vegetables, Other	256
57	Belarus	2008	Wheat	170
57	Belarus	2008	Wine	21
57	Belarus	2009	Apples	64
57	Belarus	2009	Bananas	10
57	Belarus	2009	Barley	13
57	Belarus	2009	Beer	130
57	Belarus	2009	Beverages, Alcoholic	42

FAO country code	Country	Reference year	Food item	Gross food supply (g $capita^{-1}$ d^{-1})
57	Belarus	2009	Beverages, Fermented	6
57	Belarus	2009	Bovine Meat	56
57	Belarus	2009	Butter, Ghee	9
57	Belarus	2009	Cereals, Other	14
57	Belarus	2009	Cheese	8
57	Belarus	2009	Cocoa Beans	4
57	Belarus	2009	Coconut Oil	1
57	Belarus	2009	Coffee	4
57	Belarus	2009	Cream	2
57	Belarus	2009	Demersal Fish	7
57	Belarus	2009	Eggs	43
57	Belarus	2009	Fats, Animals, Raw	10
57	Belarus	2009	Freshwater Fish	12
57	Belarus	2009	Fruits, Other	79
57	Belarus	2009	Grapefruit	3
57	Belarus	2009	Grapes	3
57	Belarus	2009	Groundnuts (Shelled Eq)	1
57	Belarus	2009	Honey	1
57	Belarus	2009	Lemons, Limes	2
57	Belarus	2009	Maize	1
57	Belarus	2009	Marine Fish, Other	5
57	Belarus	2009	Meat, Other	1
57	Belarus	2009	Milk, Whole	174
57	Belarus	2009	Nuts	6
57	Belarus	2009	Oats	25
57	Belarus	2009	Offals, Edible	19
57	Belarus	2009	Olives	1
57	Belarus	2009	Onions	52
57	Belarus	2009	Oranges, Mandarines	19
57	Belarus	2009	Pelagic Fish	25
57	Belarus	2009	Pigmeat	100
57	Belarus	2009	Pineapples	2
57	Belarus	2009	Potatoes	509
57	Belarus	2009	Poultry Meat	61
57	Belarus	2009	Rape and Mustard Oil	14
57	Belarus	2009	Rice (Milled Equivalent)	8
57	Belarus	2009	Rye	88
57	Belarus	2009	Soyabean Oil	4
57	Belarus	2009	Sugar, Refined Equivalent	105
57	Belarus	2009	Sunflowerseed Oil	17
57	Belarus	2009	Sweeteners, Other	3
57	Belarus	2009	Tea	1
57	Belarus	2009	Tomatoes	92
57	Belarus	2009	Vegetables, Other	269
57	Belarus	2009	Wheat	156
57	Belarus	2009	Wine	18
57	Belarus	2010	Apples	62
57	Belarus	2010	Bananas	12
57	Belarus	2010	Barley	12
57	Belarus	2010	Beer	143
57	Belarus	2010	Beverages, Alcoholic	43
57	Belarus	2010	Beverages, Fermented	6
57	Belarus	2010	Bovine Meat	51
57	Belarus	2010	Butter, Ghee	11
57	Belarus	2010	Cephalopods	1
57	Belarus	2010	Cereals, Other	4
57	Belarus	2010	Cheese	12
57	Belarus	2010	Cocoa Beans	5
57	Belarus	2010	Coconut Oil	1
57	Belarus	2010	Coffee	4
57	Belarus	2010	Cream	1
57	Belarus	2010	Crustaceans	1
57	Belarus	2010	Demersal Fish	8
57	Belarus	2010	Eggs	45
57	Belarus	2010	Fats, Animals, Raw	10
57	Belarus	2010	Freshwater Fish	13
57	Belarus	2010	Fruits, Other	88
57	Belarus	2010	Grapefruit	4
57	Belarus	2010	Grapes	3
57	Belarus	2010	Groundnuts (Shelled Eq)	2
57	Belarus	2010	Honey	1
57	Belarus	2010	Lemons, Limes	2
57	Belarus	2010	Maize	1
57	Belarus	2010	Marine Fish, Other	5
57	Belarus	2010	Meat, Other	1
57	Belarus	2010	Milk, Whole	133
57	Belarus	2010	Nuts	6
57	Belarus	2010	Oats	25
57	Belarus	2010	Offals, Edible	19
57	Belarus	2010	Olives	1
57	Belarus	2010	Onions	52
57	Belarus	2010	Oranges, Mandarines	20
57	Belarus	2010	Pelagic Fish	21
57	Belarus	2010	Pigmeat	110
57	Belarus	2010	Pineapples	3
57	Belarus	2010	Potatoes	507
57	Belarus	2010	Poultry Meat	67
57	Belarus	2010	Rape and Mustard Oil	15
57	Belarus	2010	Rice (Milled Equivalent)	9
57	Belarus	2010	Rye	73
57	Belarus	2010	Soyabean Oil	5
57	Belarus	2010	Soyabeans	2
57	Belarus	2010	Sugar, Refined Equivalent	90
57	Belarus	2010	Sunflowerseed Oil	21
57	Belarus	2010	Sweeteners, Other	4
57	Belarus	2010	Tea	1
57	Belarus	2010	Tomatoes	95

FAO country code	Country	Reference year	Food item	Gross food supply (g $capita^{-1}$ d^{-1})
57	Belarus	2010	Vegetables, Other	269
57	Belarus	2010	Wheat	181
57	Belarus	2010	Wine	21
57	Belarus	2011	Apples	58
57	Belarus	2011	Bananas	11
57	Belarus	2011	Barley	12
57	Belarus	2011	Beer	143
57	Belarus	2011	Beverages, Alcoholic	47
57	Belarus	2011	Beverages, Fermented	5
57	Belarus	2011	Bovine Meat	50
57	Belarus	2011	Butter, Ghee	12
57	Belarus	2011	Cephalopods	1
57	Belarus	2011	Cereals, Other	16
57	Belarus	2011	Cheese	12
57	Belarus	2011	Cocoa Beans	4
57	Belarus	2011	Coffee	3
57	Belarus	2011	Cream	2
57	Belarus	2011	Crustaceans	1
57	Belarus	2011	Demersal Fish	8
57	Belarus	2011	Eggs	45
57	Belarus	2011	Fats, Animals, Raw	10
57	Belarus	2011	Freshwater Fish	13
57	Belarus	2011	Fruits, Other	72
57	Belarus	2011	Grapefruit	3
57	Belarus	2011	Grapes	3
57	Belarus	2011	Groundnuts (Shelled Eq)	1
57	Belarus	2011	Honey	1
57	Belarus	2011	Lemons, Limes	2
57	Belarus	2011	Maize	1
57	Belarus	2011	Marine Fish, Other	5
57	Belarus	2011	Meat, Other	1
57	Belarus	2011	Milk, Whole	126
57	Belarus	2011	Nuts	7
57	Belarus	2011	Oats	25
57	Belarus	2011	Offals, Edible	19
57	Belarus	2011	Olives	1
57	Belarus	2011	Onions	48
57	Belarus	2011	Oranges, Mandarines	20
57	Belarus	2011	Pelagic Fish	21
57	Belarus	2011	Pigmeat	95
57	Belarus	2011	Pineapples	3
57	Belarus	2011	Potatoes	507
57	Belarus	2011	Poultry Meat	69
57	Belarus	2011	Rape and Mustard Oil	18
57	Belarus	2011	Rice (Milled Equivalent)	8
57	Belarus	2011	Rye	88
57	Belarus	2011	Soyabean Oil	5
57	Belarus	2011	Soyabeans	3
57	Belarus	2011	Sugar, Refined Equivalent	87
57	Belarus	2011	Sunflowerseed Oil	24
57	Belarus	2011	Sweeteners, Other	4
57	Belarus	2011	Tea	1
57	Belarus	2011	Tomatoes	40
57	Belarus	2011	Vegetables, Other	313
57	Belarus	2011	Wheat	181
57	Belarus	2011	Wine	21
255	Belgium	1992	Apples	141
255	Belgium	1992	Bananas	42
255	Belgium	1992	Barley	2
255	Belgium	1992	Beans	2
255	Belgium	1992	Beer	315
255	Belgium	1992	Beverages, Alcoholic	8
255	Belgium	1992	Beverages, Fermented	2
255	Belgium	1992	Bovine Meat	54
255	Belgium	1992	Butter, Ghee	20
255	Belgium	1992	Cephalopods	1
255	Belgium	1992	Cereals, Other	4
255	Belgium	1992	Cheese	37
255	Belgium	1992	Cocoa Beans	8
255	Belgium	1992	Coconut Oil	1
255	Belgium	1992	Coconuts - Incl Copra	3
255	Belgium	1992	Coffee	21
255	Belgium	1992	Cream	11
255	Belgium	1992	Crustaceans	8
255	Belgium	1992	Dates	1
255	Belgium	1992	Demersal Fish	20
255	Belgium	1992	Eggs	35
255	Belgium	1992	Fats, Animals, Raw	40
255	Belgium	1992	Freshwater Fish	6
255	Belgium	1992	Fruits, Other	74
255	Belgium	1992	Grapefruit	17
255	Belgium	1992	Grapes	19
255	Belgium	1992	Groundnut Oil	3
255	Belgium	1992	Groundnuts (Shelled Eq)	1
255	Belgium	1992	Honey	2
255	Belgium	1992	Lemons, Limes	8
255	Belgium	1992	Maize	6
255	Belgium	1992	Maize Germ Oil	5
255	Belgium	1992	Marine Fish, Other	6
255	Belgium	1992	Meat, Other	14
255	Belgium	1992	Milk, Whole	187
255	Belgium	1992	Molluscs, Other	12
255	Belgium	1992	Mutton & Goat Meat	6
255	Belgium	1992	Nuts	11
255	Belgium	1992	Oats	1
255	Belgium	1992	Offals, Edible	18
255	Belgium	1992	Oilcrops Oil, Other	8

FAO country code	Country	Reference year	Food item	Gross food supply (g $capita^{-1}$ d^{-1})
255	Belgium	1992	Olive Oil	1
255	Belgium	1992	Olives	1
255	Belgium	1992	Onions	20
255	Belgium	1992	Oranges, Mandarines	141
255	Belgium	1992	Palm Oil	10
255	Belgium	1992	Palmkernel Oil	1
255	Belgium	1992	Peas	3
255	Belgium	1992	Pelagic Fish	13
255	Belgium	1992	Pigmeat	142
255	Belgium	1992	Pineapples	8
255	Belgium	1992	Potatoes	305
255	Belgium	1992	Poultry Meat	48
255	Belgium	1992	Pulses, Other	2
255	Belgium	1992	Rape and Mustard Oil	4
255	Belgium	1992	Rice (Milled Equivalent)	11
255	Belgium	1992	Rye	4
255	Belgium	1992	Soyabean Oil	11
255	Belgium	1992	Spices, Other	1
255	Belgium	1992	Sugar, Refined Equivalent	102
255	Belgium	1992	Sunflowerseed Oil	12
255	Belgium	1992	Sweeteners, Other	12
255	Belgium	1992	Tea	1
255	Belgium	1992	Tomatoes	67
255	Belgium	1992	Vegetables, Other	223
255	Belgium	1992	Wheat	258
255	Belgium	1992	Wine	69
255	Belgium	1993	Apples	97
255	Belgium	1993	Bananas	39
255	Belgium	1993	Barley	2
255	Belgium	1993	Beans	2
255	Belgium	1993	Beer	313
255	Belgium	1993	Beverages, Alcoholic	8
255	Belgium	1993	Bovine Meat	58
255	Belgium	1993	Butter, Ghee	19
255	Belgium	1993	Cereals, Other	4
255	Belgium	1993	Cheese	40
255	Belgium	1993	Cocoa Beans	9
255	Belgium	1993	Coconut Oil	7
255	Belgium	1993	Coconuts - Incl Copra	4
255	Belgium	1993	Coffee	10
255	Belgium	1993	Cream	11
255	Belgium	1993	Crustaceans	6
255	Belgium	1993	Dates	1
255	Belgium	1993	Demersal Fish	22
255	Belgium	1993	Eggs	36
255	Belgium	1993	Fats, Animals, Raw	37
255	Belgium	1993	Freshwater Fish	5
255	Belgium	1993	Fruits, Other	51
255	Belgium	1993	Grapefruit	5
255	Belgium	1993	Grapes	17
255	Belgium	1993	Groundnut Oil	2
255	Belgium	1993	Groundnuts (Shelled Eq)	1
255	Belgium	1993	Honey	1
255	Belgium	1993	Lemons, Limes	5
255	Belgium	1993	Maize	6
255	Belgium	1993	Maize Germ Oil	3
255	Belgium	1993	Marine Fish, Other	4
255	Belgium	1993	Meat, Other	13
255	Belgium	1993	Milk, Whole	186
255	Belgium	1993	Molluscs, Other	10
255	Belgium	1993	Mutton & Goat Meat	5
255	Belgium	1993	Nuts	11
255	Belgium	1993	Oats	2
255	Belgium	1993	Offals, Edible	16
255	Belgium	1993	Oilcrops Oil, Other	6
255	Belgium	1993	Olive Oil	1
255	Belgium	1993	Olives	1
255	Belgium	1993	Onions	21
255	Belgium	1993	Oranges, Mandarines	71
255	Belgium	1993	Palm Oil	7
255	Belgium	1993	Palmkernel Oil	2
255	Belgium	1993	Peas	2
255	Belgium	1993	Pelagic Fish	10
255	Belgium	1993	Pepper	1
255	Belgium	1993	Pigmeat	146
255	Belgium	1993	Pineapples	6
255	Belgium	1993	Potatoes	305
255	Belgium	1993	Poultry Meat	48
255	Belgium	1993	Pulses, Other	3
255	Belgium	1993	Rape and Mustard Oil	8
255	Belgium	1993	Rice (Milled Equivalent)	11
255	Belgium	1993	Rye	3
255	Belgium	1993	Soyabean Oil	12
255	Belgium	1993	Spices, Other	1
255	Belgium	1993	Sugar, Refined Equivalent	102
255	Belgium	1993	Sunflowerseed Oil	6
255	Belgium	1993	Sweeteners, Other	11
255	Belgium	1993	Tomatoes	71
255	Belgium	1993	Vegetables, Other	234
255	Belgium	1993	Wheat	257
255	Belgium	1993	Wine	66
255	Belgium	1994	Apples	105
255	Belgium	1994	Bananas	31
255	Belgium	1994	Barley	1
255	Belgium	1994	Beans	2
255	Belgium	1994	Beer	299
255	Belgium	1994	Beverages, Alcoholic	7

FAO country code	Country	Reference year	Food item	Gross food supply (g $capita^{-1}$ d^{-1})
255	Belgium	1994	Beverages, Fermented	1
255	Belgium	1994	Bovine Meat	59
255	Belgium	1994	Butter, Ghee	18
255	Belgium	1994	Cephalopods	1
255	Belgium	1994	Cereals, Other	1
255	Belgium	1994	Cheese	37
255	Belgium	1994	Cocoa Beans	11
255	Belgium	1994	Coconut Oil	9
255	Belgium	1994	Coconuts - Incl Copra	4
255	Belgium	1994	Coffee	8
255	Belgium	1994	Cream	10
255	Belgium	1994	Crustaceans	7
255	Belgium	1994	Dates	1
255	Belgium	1994	Demersal Fish	21
255	Belgium	1994	Eggs	40
255	Belgium	1994	Fats, Animals, Raw	37
255	Belgium	1994	Freshwater Fish	7
255	Belgium	1994	Fruits, Other	60
255	Belgium	1994	Grapefruit	11
255	Belgium	1994	Grapes	17
255	Belgium	1994	Groundnut Oil	3
255	Belgium	1994	Groundnuts (Shelled Eq)	1
255	Belgium	1994	Honey	1
255	Belgium	1994	Lemons, Limes	6
255	Belgium	1994	Maize	5
255	Belgium	1994	Maize Germ Oil	5
255	Belgium	1994	Marine Fish, Other	4
255	Belgium	1994	Meat, Other	12
255	Belgium	1994	Milk, Whole	186
255	Belgium	1994	Molluscs, Other	12
255	Belgium	1994	Mutton & Goat Meat	6
255	Belgium	1994	Nuts	14
255	Belgium	1994	Oats	1
255	Belgium	1994	Offals, Edible	16
255	Belgium	1994	Oilcrops Oil, Other	11
255	Belgium	1994	Olive Oil	1
255	Belgium	1994	Olives	1
255	Belgium	1994	Onions	26
255	Belgium	1994	Oranges, Mandarines	130
255	Belgium	1994	Palm Oil	2
255	Belgium	1994	Palmkernel Oil	5
255	Belgium	1994	Peas	3
255	Belgium	1994	Pelagic Fish	11
255	Belgium	1994	Pigmeat	114
255	Belgium	1994	Pineapples	3
255	Belgium	1994	Potatoes	296
255	Belgium	1994	Poultry Meat	54
255	Belgium	1994	Pulses, Other	2
255	Belgium	1994	Rape and Mustard Oil	7
255	Belgium	1994	Rice (Milled Equivalent)	10
255	Belgium	1994	Rye	4
255	Belgium	1994	Soyabean Oil	11
255	Belgium	1994	Spices, Other	1
255	Belgium	1994	Sugar, Refined Equivalent	107
255	Belgium	1994	Sunflowerseed Oil	6
255	Belgium	1994	Sweeteners, Other	9
255	Belgium	1994	Tomatoes	42
255	Belgium	1994	Vegetables, Other	235
255	Belgium	1994	Wheat	255
255	Belgium	1994	Wine	63
255	Belgium	1995	Apples	99
255	Belgium	1995	Bananas	20
255	Belgium	1995	Barley	2
255	Belgium	1995	Beans	2
255	Belgium	1995	Beer	299
255	Belgium	1995	Beverages, Alcoholic	8
255	Belgium	1995	Bovine Meat	59
255	Belgium	1995	Butter, Ghee	17
255	Belgium	1995	Cephalopods	1
255	Belgium	1995	Cereals, Other	1
255	Belgium	1995	Cheese	36
255	Belgium	1995	Citrus, Other	1
255	Belgium	1995	Cocoa Beans	4
255	Belgium	1995	Coconut Oil	13
255	Belgium	1995	Coconuts - Incl Copra	3
255	Belgium	1995	Coffee	6
255	Belgium	1995	Cream	18
255	Belgium	1995	Crustaceans	9
255	Belgium	1995	Demersal Fish	21
255	Belgium	1995	Eggs	38
255	Belgium	1995	Fats, Animals, Raw	36
255	Belgium	1995	Freshwater Fish	8
255	Belgium	1995	Fruits, Other	46
255	Belgium	1995	Grapefruit	3
255	Belgium	1995	Grapes	15
255	Belgium	1995	Groundnut Oil	2
255	Belgium	1995	Groundnuts (Shelled Eq)	1
255	Belgium	1995	Honey	1
255	Belgium	1995	Lemons, Limes	5
255	Belgium	1995	Maize	6
255	Belgium	1995	Maize Germ Oil	3
255	Belgium	1995	Marine Fish, Other	4
255	Belgium	1995	Meat, Other	12
255	Belgium	1995	Milk, Whole	175
255	Belgium	1995	Molluscs, Other	12
255	Belgium	1995	Mutton & Goat Meat	5
255	Belgium	1995	Nuts	12

FAO country code	Country	Reference year	Food item	Gross food supply (g *capita*$^{-1}$ d^{-1})
255	Belgium	1995	Offals, Edible	15
255	Belgium	1995	Oilcrops Oil, Other	6
255	Belgium	1995	Olive Oil	1
255	Belgium	1995	Olives	1
255	Belgium	1995	Onions	18
255	Belgium	1995	Oranges, Mandarines	122
255	Belgium	1995	Palm Oil	6
255	Belgium	1995	Palmkernel Oil	2
255	Belgium	1995	Peas	4
255	Belgium	1995	Pelagic Fish	12
255	Belgium	1995	Pigmeat	108
255	Belgium	1995	Pineapples	3
255	Belgium	1995	Potatoes	293
255	Belgium	1995	Poultry Meat	60
255	Belgium	1995	Pulses, Other	2
255	Belgium	1995	Rape and Mustard Oil	8
255	Belgium	1995	Rice (Milled Equivalent)	10
255	Belgium	1995	Rye	4
255	Belgium	1995	Soyabean Oil	13
255	Belgium	1995	Spices, Other	1
255	Belgium	1995	Sugar, Refined Equivalent	107
255	Belgium	1995	Sunflowerseed Oil	7
255	Belgium	1995	Sweeteners, Other	9
255	Belgium	1995	Tomatoes	76
255	Belgium	1995	Vegetables, Other	248
255	Belgium	1995	Wheat	255
255	Belgium	1995	Wine	60
255	Belgium	1996	Apples	76
255	Belgium	1996	Bananas	55
255	Belgium	1996	Barley	1
255	Belgium	1996	Beans	2
255	Belgium	1996	Beer	294
255	Belgium	1996	Beverages, Alcoholic	7
255	Belgium	1996	Bovine Meat	61
255	Belgium	1996	Butter, Ghee	17
255	Belgium	1996	Cereals, Other	1
255	Belgium	1996	Cheese	38
255	Belgium	1996	Citrus, Other	1
255	Belgium	1996	Cocoa Beans	2
255	Belgium	1996	Coconut Oil	7
255	Belgium	1996	Coconuts - Incl Copra	5
255	Belgium	1996	Coffee	15
255	Belgium	1996	Cottonseed Oil	1
255	Belgium	1996	Cream	19
255	Belgium	1996	Crustaceans	7
255	Belgium	1996	Dates	1
255	Belgium	1996	Demersal Fish	19
255	Belgium	1996	Eggs	36
255	Belgium	1996	Fats, Animals, Raw	36
255	Belgium	1996	Freshwater Fish	7
255	Belgium	1996	Fruits, Other	49
255	Belgium	1996	Grapefruit	15
255	Belgium	1996	Grapes	15
255	Belgium	1996	Groundnut Oil	1
255	Belgium	1996	Honey	2
255	Belgium	1996	Lemons, Limes	6
255	Belgium	1996	Maize	8
255	Belgium	1996	Maize Germ Oil	2
255	Belgium	1996	Marine Fish, Other	4
255	Belgium	1996	Meat, Other	11
255	Belgium	1996	Milk, Whole	171
255	Belgium	1996	Molluscs, Other	9
255	Belgium	1996	Mutton & Goat Meat	5
255	Belgium	1996	Nuts	12
255	Belgium	1996	Offals, Edible	13
255	Belgium	1996	Oilcrops Oil, Other	8
255	Belgium	1996	Olive Oil	2
255	Belgium	1996	Olives	1
255	Belgium	1996	Onions	22
255	Belgium	1996	Oranges, Mandarines	162
255	Belgium	1996	Palm Oil	3
255	Belgium	1996	Palmkernel Oil	3
255	Belgium	1996	Peas	4
255	Belgium	1996	Pelagic Fish	11
255	Belgium	1996	Pepper	1
255	Belgium	1996	Pigmeat	105
255	Belgium	1996	Pineapples	7
255	Belgium	1996	Plantains	1
255	Belgium	1996	Potatoes	290
255	Belgium	1996	Poultry Meat	58
255	Belgium	1996	Pulses, Other	1
255	Belgium	1996	Rape and Mustard Oil	12
255	Belgium	1996	Rice (Milled Equivalent)	10
255	Belgium	1996	Rye	3
255	Belgium	1996	Soyabean Oil	14
255	Belgium	1996	Spices, Other	1
255	Belgium	1996	Sugar, Refined Equivalent	114
255	Belgium	1996	Sunflowerseed Oil	12
255	Belgium	1996	Sweeteners, Other	8
255	Belgium	1996	Tomatoes	67
255	Belgium	1996	Vegetables, Other	259
255	Belgium	1996	Wheat	262
255	Belgium	1996	Wine	61
255	Belgium	1997	Apples	82
255	Belgium	1997	Bananas	55
255	Belgium	1997	Barley	2
255	Belgium	1997	Beans	2

FAO country code	Country	Reference year	Food item	Gross food supply (g *capita*$^{-1}$ d^{-1})
255	Belgium	1997	Beer	332
255	Belgium	1997	Beverages, Alcoholic	7
255	Belgium	1997	Bovine Meat	58
255	Belgium	1997	Butter, Ghee	18
255	Belgium	1997	Cereals, Other	2
255	Belgium	1997	Cheese	40
255	Belgium	1997	Citrus, Other	1
255	Belgium	1997	Coconut Oil	4
255	Belgium	1997	Coconuts - Incl Copra	11
255	Belgium	1997	Coffee	12
255	Belgium	1997	Cottonseed Oil	4
255	Belgium	1997	Cream	19
255	Belgium	1997	Crustaceans	6
255	Belgium	1997	Dates	1
255	Belgium	1997	Demersal Fish	20
255	Belgium	1997	Eggs	39
255	Belgium	1997	Fats, Animals, Raw	38
255	Belgium	1997	Freshwater Fish	8
255	Belgium	1997	Fruits, Other	52
255	Belgium	1997	Grapefruit	12
255	Belgium	1997	Grapes	13
255	Belgium	1997	Groundnut Oil	2
255	Belgium	1997	Groundnuts (Shelled Eq)	1
255	Belgium	1997	Honey	2
255	Belgium	1997	Lemons, Limes	6
255	Belgium	1997	Maize	6
255	Belgium	1997	Maize Germ Oil	3
255	Belgium	1997	Marine Fish, Other	5
255	Belgium	1997	Meat, Other	11
255	Belgium	1997	Milk, Whole	160
255	Belgium	1997	Molluscs, Other	10
255	Belgium	1997	Mutton & Goat Meat	5
255	Belgium	1997	Nuts	13
255	Belgium	1997	Offals, Edible	13
255	Belgium	1997	Oilcrops Oil, Other	6
255	Belgium	1997	Olive Oil	2
255	Belgium	1997	Olives	1
255	Belgium	1997	Onions	20
255	Belgium	1997	Oranges, Mandarines	78
255	Belgium	1997	Palm Oil	9
255	Belgium	1997	Palmkernel Oil	1
255	Belgium	1997	Peas	4
255	Belgium	1997	Pelagic Fish	12
255	Belgium	1997	Pepper	1
255	Belgium	1997	Pigmeat	94
255	Belgium	1997	Pineapples	10
255	Belgium	1997	Plantains	1
255	Belgium	1997	Potatoes	288
255	Belgium	1997	Poultry Meat	51
255	Belgium	1997	Pulses, Other	1
255	Belgium	1997	Rape and Mustard Oil	12
255	Belgium	1997	Rice (Milled Equivalent)	18
255	Belgium	1997	Rye	5
255	Belgium	1997	Soyabean Oil	12
255	Belgium	1997	Spices, Other	1
255	Belgium	1997	Sugar, Refined Equivalent	115
255	Belgium	1997	Sunflowerseed Oil	6
255	Belgium	1997	Sweeteners, Other	7
255	Belgium	1997	Tomatoes	77
255	Belgium	1997	Vegetables, Other	273
255	Belgium	1997	Wheat	262
255	Belgium	1997	Wine	60
255	Belgium	1998	Apples	93
255	Belgium	1998	Bananas	16
255	Belgium	1998	Barley	2
255	Belgium	1998	Beans	2
255	Belgium	1998	Beer	287
255	Belgium	1998	Beverages, Alcoholic	9
255	Belgium	1998	Bovine Meat	54
255	Belgium	1998	Butter, Ghee	16
255	Belgium	1998	Cereals, Other	1
255	Belgium	1998	Cheese	38
255	Belgium	1998	Citrus, Other	1
255	Belgium	1998	Cocoa Beans	1
255	Belgium	1998	Coconut Oil	3
255	Belgium	1998	Coconuts - Incl Copra	5
255	Belgium	1998	Coffee	19
255	Belgium	1998	Cottonseed Oil	4
255	Belgium	1998	Cream	21
255	Belgium	1998	Crustaceans	7
255	Belgium	1998	Dates	1
255	Belgium	1998	Demersal Fish	20
255	Belgium	1998	Eggs	41
255	Belgium	1998	Fats, Animals, Raw	37
255	Belgium	1998	Freshwater Fish	8
255	Belgium	1998	Fruits, Other	52
255	Belgium	1998	Grapefruit	7
255	Belgium	1998	Grapes	16
255	Belgium	1998	Groundnut Oil	2
255	Belgium	1998	Groundnuts (Shelled Eq)	1
255	Belgium	1998	Honey	2
255	Belgium	1998	Lemons, Limes	6
255	Belgium	1998	Maize	6
255	Belgium	1998	Maize Germ Oil	2
255	Belgium	1998	Marine Fish, Other	4
255	Belgium	1998	Meat, Other	12
255	Belgium	1998	Milk, Whole	153

FAO country code	Country	Reference year	Food item	Gross food supply (g *capita*$^{-1}$ d^{-1})
255	Belgium	1998	Molluscs, Other	10
255	Belgium	1998	Mutton & Goat Meat	5
255	Belgium	1998	Nuts	13
255	Belgium	1998	Offals, Edible	20
255	Belgium	1998	Oilcrops Oil, Other	6
255	Belgium	1998	Olive Oil	2
255	Belgium	1998	Olives	1
255	Belgium	1998	Onions	19
255	Belgium	1998	Oranges, Mandarines	115
255	Belgium	1998	Palm Oil	8
255	Belgium	1998	Palmkernel Oil	1
255	Belgium	1998	Peas	4
255	Belgium	1998	Pelagic Fish	13
255	Belgium	1998	Pigmeat	91
255	Belgium	1998	Pineapples	9
255	Belgium	1998	Plantains	1
255	Belgium	1998	Potatoes	280
255	Belgium	1998	Poultry Meat	51
255	Belgium	1998	Pulses, Other	1
255	Belgium	1998	Rape and Mustard Oil	12
255	Belgium	1998	Rice (Milled Equivalent)	10
255	Belgium	1998	Rye	5
255	Belgium	1998	Soyabean Oil	16
255	Belgium	1998	Spices, Other	1
255	Belgium	1998	Sugar, Refined Equivalent	122
255	Belgium	1998	Sunflowerseed Oil	8
255	Belgium	1998	Sweeteners, Other	9
255	Belgium	1998	Tomatoes	85
255	Belgium	1998	Vegetables, Other	265
255	Belgium	1998	Wheat	271
255	Belgium	1998	Wine	69
255	Belgium	1999	Apples	105
255	Belgium	1999	Bananas	11
255	Belgium	1999	Barley	1
255	Belgium	1999	Beans	3
255	Belgium	1999	Beer	273
255	Belgium	1999	Beverages, Alcoholic	6
255	Belgium	1999	Bovine Meat	56
255	Belgium	1999	Butter, Ghee	14
255	Belgium	1999	Cephalopods	1
255	Belgium	1999	Cheese	41
255	Belgium	1999	Citrus, Other	1
255	Belgium	1999	Coconut Oil	13
255	Belgium	1999	Coconuts - Incl Copra	4
255	Belgium	1999	Coffee	15
255	Belgium	1999	Cottonseed Oil	2
255	Belgium	1999	Cream	18
255	Belgium	1999	Crustaceans	6
255	Belgium	1999	Demersal Fish	18
255	Belgium	1999	Eggs	40
255	Belgium	1999	Fats, Animals, Raw	37
255	Belgium	1999	Freshwater Fish	9
255	Belgium	1999	Fruits, Other	80
255	Belgium	1999	Grapefruit	11
255	Belgium	1999	Grapes	16
255	Belgium	1999	Honey	2
255	Belgium	1999	Lemons, Limes	5
255	Belgium	1999	Maize	6
255	Belgium	1999	Maize Germ Oil	3
255	Belgium	1999	Marine Fish, Other	5
255	Belgium	1999	Meat, Other	12
255	Belgium	1999	Milk, Whole	152
255	Belgium	1999	Molluscs, Other	12
255	Belgium	1999	Mutton & Goat Meat	5
255	Belgium	1999	Nuts	12
255	Belgium	1999	Offals, Edible	22
255	Belgium	1999	Oilcrops Oil, Other	5
255	Belgium	1999	Olive Oil	3
255	Belgium	1999	Olives	2
255	Belgium	1999	Onions	30
255	Belgium	1999	Oranges, Mandarines	75
255	Belgium	1999	Palm Oil	7
255	Belgium	1999	Palmkernel Oil	1
255	Belgium	1999	Peas	3
255	Belgium	1999	Pelagic Fish	13
255	Belgium	1999	Pigmeat	106
255	Belgium	1999	Pineapples	7
255	Belgium	1999	Plantains	4
255	Belgium	1999	Potatoes	307
255	Belgium	1999	Poultry Meat	53
255	Belgium	1999	Pulses, Other	1
255	Belgium	1999	Rape and Mustard Oil	7
255	Belgium	1999	Rice (Milled Equivalent)	11
255	Belgium	1999	Rye	2
255	Belgium	1999	Soyabean Oil	15
255	Belgium	1999	Spices, Other	1
255	Belgium	1999	Sugar, Refined Equivalent	122
255	Belgium	1999	Sunflowerseed Oil	7
255	Belgium	1999	Sweeteners, Other	8
255	Belgium	1999	Tomatoes	77
255	Belgium	1999	Vegetables, Other	298
255	Belgium	1999	Wheat	271
255	Belgium	1999	Wine	66
255	Belgium	2000	Apples	86
255	Belgium	2000	Bananas	3
255	Belgium	2000	Barley	1
255	Belgium	2000	Beans	2

FAO country code	Country	Reference year	Food item	Gross food supply (g $capita^{-1}$ d^{-1})
255	Belgium	2000	Beer	278
255	Belgium	2000	Beverages, Alcoholic	4
255	Belgium	2000	Bovine Meat	53
255	Belgium	2000	Butter, Ghee	18
255	Belgium	2000	Cheese	48
255	Belgium	2000	Citrus, Other	1
255	Belgium	2000	Coconuts - Incl Copra	5
255	Belgium	2000	Coffee	10
255	Belgium	2000	Cottonseed Oil	1
255	Belgium	2000	Cream	19
255	Belgium	2000	Crustaceans	7
255	Belgium	2000	Dates	1
255	Belgium	2000	Demersal Fish	20
255	Belgium	2000	Eggs	30
255	Belgium	2000	Fats, Animals, Raw	34
255	Belgium	2000	Freshwater Fish	12
255	Belgium	2000	Fruits, Other	67
255	Belgium	2000	Grapefruit	11
255	Belgium	2000	Grapes	9
255	Belgium	2000	Groundnuts (Shelled Eq)	1
255	Belgium	2000	Honey	2
255	Belgium	2000	Lemons, Limes	5
255	Belgium	2000	Maize	6
255	Belgium	2000	Maize Germ Oil	3
255	Belgium	2000	Marine Fish, Other	4
255	Belgium	2000	Meat, Other	4
255	Belgium	2000	Milk, Whole	182
255	Belgium	2000	Molluscs, Other	11
255	Belgium	2000	Mutton & Goat Meat	6
255	Belgium	2000	Nuts	15
255	Belgium	2000	Offals, Edible	12
255	Belgium	2000	Oilcrops Oil, Other	2
255	Belgium	2000	Olive Oil	3
255	Belgium	2000	Olives	2
255	Belgium	2000	Onions	1
255	Belgium	2000	Oranges, Mandarines	12
255	Belgium	2000	Palm Oil	20
255	Belgium	2000	Palmkernel Oil	3
255	Belgium	2000	Peas	4
255	Belgium	2000	Pelagic Fish	11
255	Belgium	2000	Pigmeat	106
255	Belgium	2000	Pineapples	5
255	Belgium	2000	Potatoes	346
255	Belgium	2000	Poultry Meat	54
255	Belgium	2000	Pulses, Other	1
255	Belgium	2000	Rape and Mustard Oil	11
255	Belgium	2000	Rice (Milled Equivalent)	11
255	Belgium	2000	Rye	1
255	Belgium	2000	Soyabean Oil	13
255	Belgium	2000	Spices, Other	1
255	Belgium	2000	Sugar, Refined Equivalent	139
255	Belgium	2000	Sunflowerseed Oil	6
255	Belgium	2000	Sweeteners, Other	10
255	Belgium	2000	Tomatoes	63
255	Belgium	2000	Vegetables, Other	263
255	Belgium	2000	Wheat	274
255	Belgium	2000	Wine	71
255	Belgium	2001	Apples	78
255	Belgium	2001	Bananas	1
255	Belgium	2001	Barley	1
255	Belgium	2001	Beans	2
255	Belgium	2001	Beer	275
255	Belgium	2001	Beverages, Alcoholic	4
255	Belgium	2001	Bovine Meat	57
255	Belgium	2001	Butter, Ghee	17
255	Belgium	2001	Cheese	49
255	Belgium	2001	Coconut Oil	2
255	Belgium	2001	Coconuts - Incl Copra	4
255	Belgium	2001	Coffee	8
255	Belgium	2001	Cream	19
255	Belgium	2001	Crustaceans	6
255	Belgium	2001	Dates	1
255	Belgium	2001	Demersal Fish	20
255	Belgium	2001	Eggs	36
255	Belgium	2001	Fats, Animals, Raw	32
255	Belgium	2001	Freshwater Fish	11
255	Belgium	2001	Fruits, Other	72
255	Belgium	2001	Grapefruit	9
255	Belgium	2001	Grapes	10
255	Belgium	2001	Groundnuts (Shelled Eq)	1
255	Belgium	2001	Honey	2
255	Belgium	2001	Lemons, Limes	5
255	Belgium	2001	Maize	6
255	Belgium	2001	Maize Germ Oil	3
255	Belgium	2001	Marine Fish, Other	3
255	Belgium	2001	Meat, Other	7
255	Belgium	2001	Milk, Whole	185
255	Belgium	2001	Molluscs, Other	10
255	Belgium	2001	Mutton & Goat Meat	5
255	Belgium	2001	Nuts	14
255	Belgium	2001	Offals, Edible	9
255	Belgium	2001	Oilcrops Oil, Other	3
255	Belgium	2001	Olive Oil	3
255	Belgium	2001	Olives	2
255	Belgium	2001	Onions	4
255	Belgium	2001	Oranges, Mandarines	11
255	Belgium	2001	Palm Oil	21

FAO country code	Country	Reference year	Food item	Gross food supply (g *capita*$^{-1}$ d^{-1})
255	Belgium	2001	Peas	3
255	Belgium	2001	Pelagic Fish	11
255	Belgium	2001	Pigmeat	107
255	Belgium	2001	Pineapples	4
255	Belgium	2001	Potatoes	309
255	Belgium	2001	Poultry Meat	61
255	Belgium	2001	Pulses, Other	1
255	Belgium	2001	Rape and Mustard Oil	14
255	Belgium	2001	Rice (Milled Equivalent)	10
255	Belgium	2001	Rye	1
255	Belgium	2001	Soyabean Oil	13
255	Belgium	2001	Spices, Other	1
255	Belgium	2001	Sugar, Refined Equivalent	138
255	Belgium	2001	Sunflowerseed Oil	6
255	Belgium	2001	Sweeteners, Other	5
255	Belgium	2001	Tomatoes	68
255	Belgium	2001	Vegetables, Other	245
255	Belgium	2001	Wheat	286
255	Belgium	2001	Wine	69
255	Belgium	2002	Apples	63
255	Belgium	2002	Bananas	2
255	Belgium	2002	Beans	3
255	Belgium	2002	Beer	276
255	Belgium	2002	Beverages, Alcoholic	4
255	Belgium	2002	Bovine Meat	54
255	Belgium	2002	Butter, Ghee	17
255	Belgium	2002	Cheese	51
255	Belgium	2002	Coconut Oil	6
255	Belgium	2002	Coconuts - Incl Copra	5
255	Belgium	2002	Coffee	21
255	Belgium	2002	Cream	20
255	Belgium	2002	Crustaceans	7
255	Belgium	2002	Demersal Fish	19
255	Belgium	2002	Eggs	33
255	Belgium	2002	Fats, Animals, Raw	35
255	Belgium	2002	Freshwater Fish	9
255	Belgium	2002	Fruits, Other	85
255	Belgium	2002	Grapefruit	8
255	Belgium	2002	Grapes	10
255	Belgium	2002	Groundnuts (Shelled Eq)	1
255	Belgium	2002	Honey	2
255	Belgium	2002	Lemons, Limes	7
255	Belgium	2002	Maize	6
255	Belgium	2002	Maize Germ Oil	3
255	Belgium	2002	Marine Fish, Other	4
255	Belgium	2002	Meat, Other	5
255	Belgium	2002	Milk, Whole	198
255	Belgium	2002	Molluscs, Other	9
255	Belgium	2002	Mutton & Goat Meat	4
255	Belgium	2002	Nuts	16
255	Belgium	2002	Offals, Edible	7
255	Belgium	2002	Oilcrops Oil, Other	7
255	Belgium	2002	Olive Oil	3
255	Belgium	2002	Olives	2
255	Belgium	2002	Onions	1
255	Belgium	2002	Oranges, Mandarines	13
255	Belgium	2002	Palm Oil	8
255	Belgium	2002	Palmkernel Oil	1
255	Belgium	2002	Peas	3
255	Belgium	2002	Pelagic Fish	12
255	Belgium	2002	Pepper	1
255	Belgium	2002	Pigmeat	98
255	Belgium	2002	Pineapples	4
255	Belgium	2002	Potatoes	295
255	Belgium	2002	Poultry Meat	69
255	Belgium	2002	Pulses, Other	1
255	Belgium	2002	Rape and Mustard Oil	16
255	Belgium	2002	Rice (Milled Equivalent)	13
255	Belgium	2002	Rye	1
255	Belgium	2002	Soyabean Oil	13
255	Belgium	2002	Spices, Other	2
255	Belgium	2002	Sugar, Refined Equivalent	126
255	Belgium	2002	Sunflowerseed Oil	5
255	Belgium	2002	Sweeteners, Other	10
255	Belgium	2002	Tomatoes	71
255	Belgium	2002	Vegetables, Other	271
255	Belgium	2002	Wheat	286
255	Belgium	2002	Wine	73
255	Belgium	2003	Apples	77
255	Belgium	2003	Bananas	2
255	Belgium	2003	Beans	2
255	Belgium	2003	Beer	279
255	Belgium	2003	Beverages, Alcoholic	4
255	Belgium	2003	Bovine Meat	51
255	Belgium	2003	Butter, Ghee	14
255	Belgium	2003	Cheese	52
255	Belgium	2003	Coconut Oil	5
255	Belgium	2003	Coconuts - Incl Copra	6
255	Belgium	2003	Coffee	25
255	Belgium	2003	Cream	21
255	Belgium	2003	Crustaceans	9
255	Belgium	2003	Demersal Fish	16
255	Belgium	2003	Eggs	32
255	Belgium	2003	Fats, Animals, Raw	33
255	Belgium	2003	Freshwater Fish	11
255	Belgium	2003	Fruits, Other	81
255	Belgium	2003	Grapefruit	8

FAO country code	Country	Reference year	Food item	Gross food supply (g *capita*$^{-1}$ d^{-1})
255	Belgium	2003	Grapes	10
255	Belgium	2003	Honey	2
255	Belgium	2003	Lemons, Limes	6
255	Belgium	2003	Maize	6
255	Belgium	2003	Maize Germ Oil	3
255	Belgium	2003	Marine Fish, Other	5
255	Belgium	2003	Meat, Other	5
255	Belgium	2003	Milk, Whole	153
255	Belgium	2003	Molluscs, Other	13
255	Belgium	2003	Mutton & Goat Meat	5
255	Belgium	2003	Nuts	17
255	Belgium	2003	Offals, Edible	6
255	Belgium	2003	Oilcrops Oil, Other	6
255	Belgium	2003	Olive Oil	3
255	Belgium	2003	Olives	2
255	Belgium	2003	Onions	1
255	Belgium	2003	Oranges, Mandarines	14
255	Belgium	2003	Palm Oil	6
255	Belgium	2003	Palmkernel Oil	5
255	Belgium	2003	Peas	3
255	Belgium	2003	Pelagic Fish	13
255	Belgium	2003	Pepper	1
255	Belgium	2003	Pigmeat	97
255	Belgium	2003	Pineapples	4
255	Belgium	2003	Potatoes	290
255	Belgium	2003	Poultry Meat	60
255	Belgium	2003	Pulses, Other	1
255	Belgium	2003	Rape and Mustard Oil	10
255	Belgium	2003	Rice (Milled Equivalent)	11
255	Belgium	2003	Rye	2
255	Belgium	2003	Soyabean Oil	13
255	Belgium	2003	Spices, Other	2
255	Belgium	2003	Sugar, Refined Equivalent	125
255	Belgium	2003	Sunflowerseed Oil	12
255	Belgium	2003	Sweeteners, Other	13
255	Belgium	2003	Tea	1
255	Belgium	2003	Tomatoes	72
255	Belgium	2003	Vegetables, Other	280
255	Belgium	2003	Wheat	286
255	Belgium	2003	Wine	70
255	Belgium	2004	Apples	84
255	Belgium	2004	Bananas	33
255	Belgium	2004	Barley	1
255	Belgium	2004	Beans	2
255	Belgium	2004	Beer	270
255	Belgium	2004	Beverages, Alcoholic	4
255	Belgium	2004	Bovine Meat	54
255	Belgium	2004	Butter, Ghee	14
255	Belgium	2004	Cheese	53
255	Belgium	2004	Coconuts - Incl Copra	6
255	Belgium	2004	Coffee	23
255	Belgium	2004	Cream	22
255	Belgium	2004	Crustaceans	8
255	Belgium	2004	Demersal Fish	19
255	Belgium	2004	Eggs	39
255	Belgium	2004	Fats, Animals, Raw	34
255	Belgium	2004	Freshwater Fish	12
255	Belgium	2004	Fruits, Other	78
255	Belgium	2004	Grapefruit	6
255	Belgium	2004	Grapes	8
255	Belgium	2004	Groundnuts (Shelled Eq)	1
255	Belgium	2004	Honey	1
255	Belgium	2004	Lemons, Limes	5
255	Belgium	2004	Maize	6
255	Belgium	2004	Maize Germ Oil	3
255	Belgium	2004	Marine Fish, Other	5
255	Belgium	2004	Meat, Other	4
255	Belgium	2004	Milk, Whole	154
255	Belgium	2004	Molluscs, Other	12
255	Belgium	2004	Mutton & Goat Meat	5
255	Belgium	2004	Nuts	16
255	Belgium	2004	Offals, Edible	6
255	Belgium	2004	Oilcrops Oil, Other	4
255	Belgium	2004	Olive Oil	3
255	Belgium	2004	Olives	3
255	Belgium	2004	Onions	1
255	Belgium	2004	Oranges, Mandarines	18
255	Belgium	2004	Palm Oil	12
255	Belgium	2004	Palmkernel Oil	5
255	Belgium	2004	Peas	3
255	Belgium	2004	Pelagic Fish	11
255	Belgium	2004	Pepper	1
255	Belgium	2004	Pigmeat	93
255	Belgium	2004	Pineapples	4
255	Belgium	2004	Potatoes	255
255	Belgium	2004	Poultry Meat	60
255	Belgium	2004	Pulses, Other	1
255	Belgium	2004	Rape and Mustard Oil	14
255	Belgium	2004	Rice (Milled Equivalent)	23
255	Belgium	2004	Rye	1
255	Belgium	2004	Soyabean Oil	13
255	Belgium	2004	Spices, Other	2
255	Belgium	2004	Sugar, Refined Equivalent	125
255	Belgium	2004	Sunflowerseed Oil	8
255	Belgium	2004	Sweeteners, Other	11
255	Belgium	2004	Tea	1
255	Belgium	2004	Tomatoes	68

FAO country code	Country	Reference year	Food item	Gross food supply (g $capita^{-1}$ d^{-1})
255	Belgium	2004	Vegetables, Other	272
255	Belgium	2004	Wheat	286
255	Belgium	2004	Wine	74
255	Belgium	2005	Apples	83
255	Belgium	2005	Bananas	7
255	Belgium	2005	Beans	2
255	Belgium	2005	Beer	276
255	Belgium	2005	Beverages, Alcoholic	4
255	Belgium	2005	Bovine Meat	51
255	Belgium	2005	Butter, Ghee	15
255	Belgium	2005	Cheese	53
255	Belgium	2005	Coconut Oil	8
255	Belgium	2005	Coconuts - Incl Copra	7
255	Belgium	2005	Coffee	19
255	Belgium	2005	Cream	26
255	Belgium	2005	Crustaceans	8
255	Belgium	2005	Dates	1
255	Belgium	2005	Demersal Fish	17
255	Belgium	2005	Eggs	31
255	Belgium	2005	Fats, Animals, Raw	34
255	Belgium	2005	Freshwater Fish	12
255	Belgium	2005	Fruits, Other	57
255	Belgium	2005	Grapefruit	9
255	Belgium	2005	Grapes	10
255	Belgium	2005	Honey	1
255	Belgium	2005	Lemons, Limes	5
255	Belgium	2005	Maize	6
255	Belgium	2005	Maize Germ Oil	3
255	Belgium	2005	Marine Fish, Other	4
255	Belgium	2005	Meat, Other	5
255	Belgium	2005	Milk, Whole	158
255	Belgium	2005	Molluscs, Other	12
255	Belgium	2005	Mutton & Goat Meat	5
255	Belgium	2005	Nuts	19
255	Belgium	2005	Offals, Edible	5
255	Belgium	2005	Oilcrops Oil, Other	4
255	Belgium	2005	Olive Oil	3
255	Belgium	2005	Olives	3
255	Belgium	2005	Onions	1
255	Belgium	2005	Oranges, Mandarines	16
255	Belgium	2005	Palm Oil	11
255	Belgium	2005	Palmkernel Oil	4
255	Belgium	2005	Peas	3
255	Belgium	2005	Pelagic Fish	13
255	Belgium	2005	Pepper	1
255	Belgium	2005	Pigmeat	95
255	Belgium	2005	Pineapples	3
255	Belgium	2005	Potatoes	249
255	Belgium	2005	Poultry Meat	69
255	Belgium	2005	Pulses, Other	1
255	Belgium	2005	Rape and Mustard Oil	8
255	Belgium	2005	Rice (Milled Equivalent)	22
255	Belgium	2005	Rye	1
255	Belgium	2005	Soyabean Oil	13
255	Belgium	2005	Spices, Other	2
255	Belgium	2005	Sugar, Refined Equivalent	124
255	Belgium	2005	Sunflowerseed Oil	8
255	Belgium	2005	Sweeteners, Other	11
255	Belgium	2005	Tea	1
255	Belgium	2005	Tomatoes	70
255	Belgium	2005	Vegetables, Other	270
255	Belgium	2005	Wheat	284
255	Belgium	2005	Wine	73
255	Belgium	2006	Apples	100
255	Belgium	2006	Bananas	29
255	Belgium	2006	Barley	1
255	Belgium	2006	Beans	2
255	Belgium	2006	Beer	255
255	Belgium	2006	Beverages, Alcoholic	4
255	Belgium	2006	Bovine Meat	57
255	Belgium	2006	Butter, Ghee	17
255	Belgium	2006	Cheese	57
255	Belgium	2006	Cocoa Beans	1
255	Belgium	2006	Coconut Oil	6
255	Belgium	2006	Coconuts - Incl Copra	8
255	Belgium	2006	Coffee	25
255	Belgium	2006	Cream	30
255	Belgium	2006	Crustaceans	8
255	Belgium	2006	Demersal Fish	15
255	Belgium	2006	Eggs	37
255	Belgium	2006	Fats, Animals, Raw	32
255	Belgium	2006	Freshwater Fish	13
255	Belgium	2006	Fruits, Other	51
255	Belgium	2006	Grapefruit	7
255	Belgium	2006	Grapes	11
255	Belgium	2006	Honey	2
255	Belgium	2006	Lemons, Limes	4
255	Belgium	2006	Maize	6
255	Belgium	2006	Maize Germ Oil	6
255	Belgium	2006	Marine Fish, Other	5
255	Belgium	2006	Meat, Other	4
255	Belgium	2006	Milk, Whole	175
255	Belgium	2006	Molluscs, Other	11
255	Belgium	2006	Mutton & Goat Meat	4
255	Belgium	2006	Nuts	21
255	Belgium	2006	Offals, Edible	5
255	Belgium	2006	Oilcrops Oil, Other	3

FAO country code	Country	Reference year	Food item	Gross food supply (g $capita^{-1}$ d^{-1})
255	Belgium	2006	Olive Oil	3
255	Belgium	2006	Olives	2
255	Belgium	2006	Onions	3
255	Belgium	2006	Oranges, Mandarines	31
255	Belgium	2006	Palm Oil	20
255	Belgium	2006	Palmkernel Oil	2
255	Belgium	2006	Peas	3
255	Belgium	2006	Pelagic Fish	13
255	Belgium	2006	Pepper	1
255	Belgium	2006	Pigmeat	88
255	Belgium	2006	Pineapples	5
255	Belgium	2006	Potatoes	261
255	Belgium	2006	Poultry Meat	62
255	Belgium	2006	Pulses, Other	1
255	Belgium	2006	Rape and Mustard Oil	4
255	Belgium	2006	Rice (Milled Equivalent)	22
255	Belgium	2006	Rye	1
255	Belgium	2006	Soyabean Oil	12
255	Belgium	2006	Spices, Other	2
255	Belgium	2006	Sugar, Refined Equivalent	117
255	Belgium	2006	Sunflowerseed Oil	7
255	Belgium	2006	Sweeteners, Other	11
255	Belgium	2006	Tea	1
255	Belgium	2006	Tomatoes	70
255	Belgium	2006	Vegetables, Other	263
255	Belgium	2006	Wheat	284
255	Belgium	2006	Wine	77
255	Belgium	2007	Apples	89
255	Belgium	2007	Bananas	18
255	Belgium	2007	Beans	2
255	Belgium	2007	Beer	237
255	Belgium	2007	Beverages, Alcoholic	4
255	Belgium	2007	Bovine Meat	56
255	Belgium	2007	Butter, Ghee	19
255	Belgium	2007	Cheese	55
255	Belgium	2007	Coconut Oil	7
255	Belgium	2007	Coconuts - Incl Copra	9
255	Belgium	2007	Coffee	19
255	Belgium	2007	Cream	28
255	Belgium	2007	Crustaceans	9
255	Belgium	2007	Dates	1
255	Belgium	2007	Demersal Fish	16
255	Belgium	2007	Eggs	35
255	Belgium	2007	Fats, Animals, Raw	32
255	Belgium	2007	Freshwater Fish	14
255	Belgium	2007	Fruits, Other	55
255	Belgium	2007	Grapefruit	10
255	Belgium	2007	Grapes	14
255	Belgium	2007	Honey	2
255	Belgium	2007	Lemons, Limes	4
255	Belgium	2007	Maize	6
255	Belgium	2007	Maize Germ Oil	6
255	Belgium	2007	Marine Fish, Other	5
255	Belgium	2007	Meat, Other	6
255	Belgium	2007	Milk, Whole	165
255	Belgium	2007	Molluscs, Other	12
255	Belgium	2007	Mutton & Goat Meat	4
255	Belgium	2007	Nuts	21
255	Belgium	2007	Offals, Edible	5
255	Belgium	2007	Oilcrops Oil, Other	3
255	Belgium	2007	Olive Oil	3
255	Belgium	2007	Olives	2
255	Belgium	2007	Onions	6
255	Belgium	2007	Oranges, Mandarines	30
255	Belgium	2007	Palm Oil	5
255	Belgium	2007	Palmkernel Oil	1
255	Belgium	2007	Peas	3
255	Belgium	2007	Pelagic Fish	13
255	Belgium	2007	Pepper	1
255	Belgium	2007	Pigmeat	93
255	Belgium	2007	Pineapples	8
255	Belgium	2007	Potatoes	260
255	Belgium	2007	Poultry Meat	68
255	Belgium	2007	Pulses, Other	1
255	Belgium	2007	Rape and Mustard Oil	15
255	Belgium	2007	Rice (Milled Equivalent)	22
255	Belgium	2007	Rye	1
255	Belgium	2007	Soyabean Oil	12
255	Belgium	2007	Spices, Other	2
255	Belgium	2007	Sugar, Refined Equivalent	128
255	Belgium	2007	Sunflowerseed Oil	8
255	Belgium	2007	Sweeteners, Other	11
255	Belgium	2007	Tea	1
255	Belgium	2007	Tomatoes	67
255	Belgium	2007	Vegetables, Other	281
255	Belgium	2007	Wheat	284
255	Belgium	2007	Wine	75
255	Belgium	2008	Apples	85
255	Belgium	2008	Bananas	41
255	Belgium	2008	Barley	1
255	Belgium	2008	Beans	2
255	Belgium	2008	Beer	213
255	Belgium	2008	Beverages, Alcoholic	4
255	Belgium	2008	Bovine Meat	52
255	Belgium	2008	Butter, Ghee	15
255	Belgium	2008	Cheese	71
255	Belgium	2008	Coconut Oil	7

FAO country code	Country	Reference year	Food item	Gross food supply (g *capita*$^{-1}$ d^{-1})
255	Belgium	2008	Coconuts - Incl Copra	8
255	Belgium	2008	Coffee	14
255	Belgium	2008	Cream	26
255	Belgium	2008	Crustaceans	9
255	Belgium	2008	Dates	1
255	Belgium	2008	Demersal Fish	16
255	Belgium	2008	Eggs	33
255	Belgium	2008	Fats, Animals, Raw	32
255	Belgium	2008	Freshwater Fish	16
255	Belgium	2008	Fruits, Other	57
255	Belgium	2008	Grapefruit	13
255	Belgium	2008	Grapes	12
255	Belgium	2008	Honey	2
255	Belgium	2008	Lemons, Limes	3
255	Belgium	2008	Maize	6
255	Belgium	2008	Maize Germ Oil	8
255	Belgium	2008	Marine Fish, Other	5
255	Belgium	2008	Meat, Other	5
255	Belgium	2008	Milk, Whole	168
255	Belgium	2008	Molluscs, Other	13
255	Belgium	2008	Mutton & Goat Meat	4
255	Belgium	2008	Nuts	19
255	Belgium	2008	Offals, Edible	5
255	Belgium	2008	Oilcrops Oil, Other	4
255	Belgium	2008	Olive Oil	3
255	Belgium	2008	Olives	2
255	Belgium	2008	Onions	7
255	Belgium	2008	Oranges, Mandarines	18
255	Belgium	2008	Palm Oil	10
255	Belgium	2008	Palmkernel Oil	4
255	Belgium	2008	Peas	3
255	Belgium	2008	Pelagic Fish	13
255	Belgium	2008	Pigmeat	90
255	Belgium	2008	Pineapples	17
255	Belgium	2008	Plantains	2
255	Belgium	2008	Potatoes	233
255	Belgium	2008	Poultry Meat	66
255	Belgium	2008	Pulses, Other	1
255	Belgium	2008	Rape and Mustard Oil	10
255	Belgium	2008	Rice (Milled Equivalent)	22
255	Belgium	2008	Rye	1
255	Belgium	2008	Soyabean Oil	12
255	Belgium	2008	Spices, Other	2
255	Belgium	2008	Sugar, Refined Equivalent	127
255	Belgium	2008	Sunflowerseed Oil	6
255	Belgium	2008	Sweeteners, Other	11
255	Belgium	2008	Tea	1
255	Belgium	2008	Tomatoes	77
255	Belgium	2008	Vegetables, Other	270
255	Belgium	2008	Wheat	277
255	Belgium	2008	Wine	74
255	Belgium	2009	Apples	79
255	Belgium	2009	Bananas	18
255	Belgium	2009	Barley	1
255	Belgium	2009	Beans	2
255	Belgium	2009	Beer	206
255	Belgium	2009	Beverages, Alcoholic	4
255	Belgium	2009	Beverages, Fermented	1
255	Belgium	2009	Bovine Meat	50
255	Belgium	2009	Butter, Ghee	15
255	Belgium	2009	Cheese	54
255	Belgium	2009	Coconut Oil	1
255	Belgium	2009	Coconuts - Incl Copra	7
255	Belgium	2009	Coffee	16
255	Belgium	2009	Cream	27
255	Belgium	2009	Crustaceans	8
255	Belgium	2009	Demersal Fish	15
255	Belgium	2009	Eggs	27
255	Belgium	2009	Fats, Animals, Raw	32
255	Belgium	2009	Freshwater Fish	15
255	Belgium	2009	Fruits, Other	75
255	Belgium	2009	Grapefruit	9
255	Belgium	2009	Grapes	13
255	Belgium	2009	Groundnuts (Shelled Eq)	1
255	Belgium	2009	Honey	2
255	Belgium	2009	Lemons, Limes	3
255	Belgium	2009	Maize	6
255	Belgium	2009	Maize Germ Oil	7
255	Belgium	2009	Marine Fish, Other	4
255	Belgium	2009	Meat, Other	6
255	Belgium	2009	Milk, Whole	181
255	Belgium	2009	Molluscs, Other	12
255	Belgium	2009	Mutton & Goat Meat	4
255	Belgium	2009	Nuts	16
255	Belgium	2009	Offals, Edible	8
255	Belgium	2009	Oilcrops Oil, Other	5
255	Belgium	2009	Olive Oil	3
255	Belgium	2009	Olives	2
255	Belgium	2009	Onions	5
255	Belgium	2009	Oranges, Mandarines	18
255	Belgium	2009	Palm Oil	23
255	Belgium	2009	Palmkernel Oil	3
255	Belgium	2009	Peas	3
255	Belgium	2009	Pelagic Fish	13
255	Belgium	2009	Pigmeat	92
255	Belgium	2009	Pineapples	12
255	Belgium	2009	Plantains	1

FAO country code	Country	Reference year	Food item	Gross food supply (g *capita*$^{-1}$ d^{-1})
255	Belgium	2009	Potatoes	226
255	Belgium	2009	Poultry Meat	59
255	Belgium	2009	Pulses, Other	1
255	Belgium	2009	Rape and Mustard Oil	6
255	Belgium	2009	Rice (Milled Equivalent)	26
255	Belgium	2009	Rye	1
255	Belgium	2009	Soyabean Oil	12
255	Belgium	2009	Spices, Other	1
255	Belgium	2009	Sugar, Refined Equivalent	127
255	Belgium	2009	Sunflowerseed Oil	6
255	Belgium	2009	Sweeteners, Other	15
255	Belgium	2009	Tea	1
255	Belgium	2009	Tomatoes	74
255	Belgium	2009	Vegetables, Other	274
255	Belgium	2009	Wheat	305
255	Belgium	2009	Wine	76
255	Belgium	2010	Apples	62
255	Belgium	2010	Bananas	33
255	Belgium	2010	Beans	2
255	Belgium	2010	Beer	230
255	Belgium	2010	Beverages, Alcoholic	5
255	Belgium	2010	Beverages, Fermented	1
255	Belgium	2010	Bovine Meat	50
255	Belgium	2010	Butter, Ghee	15
255	Belgium	2010	Cheese	56
255	Belgium	2010	Coconut Oil	3
255	Belgium	2010	Coconuts - Incl Copra	8
255	Belgium	2010	Coffee	16
255	Belgium	2010	Cream	29
255	Belgium	2010	Crustaceans	9
255	Belgium	2010	Demersal Fish	17
255	Belgium	2010	Eggs	31
255	Belgium	2010	Fats, Animals, Raw	27
255	Belgium	2010	Fish, Liver Oil	1
255	Belgium	2010	Freshwater Fish	17
255	Belgium	2010	Fruits, Other	48
255	Belgium	2010	Grapefruit	5
255	Belgium	2010	Grapes	16
255	Belgium	2010	Groundnut Oil	1
255	Belgium	2010	Honey	2
255	Belgium	2010	Lemons, Limes	4
255	Belgium	2010	Maize	6
255	Belgium	2010	Maize Germ Oil	1
255	Belgium	2010	Marine Fish, Other	4
255	Belgium	2010	Meat, Other	3
255	Belgium	2010	Milk, Whole	162
255	Belgium	2010	Molluscs, Other	12
255	Belgium	2010	Mutton & Goat Meat	4
255	Belgium	2010	Nuts	16
255	Belgium	2010	Offals, Edible	9
255	Belgium	2010	Oilcrops Oil, Other	5
255	Belgium	2010	Olive Oil	3
255	Belgium	2010	Olives	3
255	Belgium	2010	Onions	1
255	Belgium	2010	Oranges, Mandarines	21
255	Belgium	2010	Palm Oil	18
255	Belgium	2010	Palmkernel Oil	1
255	Belgium	2010	Peas	3
255	Belgium	2010	Pelagic Fish	11
255	Belgium	2010	Pigmeat	99
255	Belgium	2010	Pineapples	8
255	Belgium	2010	Plantains	1
255	Belgium	2010	Potatoes	231
255	Belgium	2010	Poultry Meat	59
255	Belgium	2010	Pulses, Other	1
255	Belgium	2010	Rape and Mustard Oil	4
255	Belgium	2010	Rice (Milled Equivalent)	25
255	Belgium	2010	Rye	2
255	Belgium	2010	Soyabean Oil	9
255	Belgium	2010	Spices, Other	1
255	Belgium	2010	Sugar, Refined Equivalent	126
255	Belgium	2010	Sunflowerseed Oil	7
255	Belgium	2010	Sweeteners, Other	24
255	Belgium	2010	Tea	1
255	Belgium	2010	Tomatoes	74
255	Belgium	2010	Vegetables, Other	249
255	Belgium	2010	Wheat	360
255	Belgium	2010	Wine	71
255	Belgium	2011	Apples	57
255	Belgium	2011	Bananas	17
255	Belgium	2011	Beans	2
255	Belgium	2011	Beer	243
255	Belgium	2011	Beverages, Alcoholic	4
255	Belgium	2011	Beverages, Fermented	1
255	Belgium	2011	Bovine Meat	44
255	Belgium	2011	Butter, Ghee	20
255	Belgium	2011	Cheese	53
255	Belgium	2011	Cocoa Beans	1
255	Belgium	2011	Coconut Oil	2
255	Belgium	2011	Coconuts - Incl Copra	7
255	Belgium	2011	Coffee	14
255	Belgium	2011	Cream	25
255	Belgium	2011	Crustaceans	9
255	Belgium	2011	Demersal Fish	17
255	Belgium	2011	Eggs	38
255	Belgium	2011	Fats, Animals, Raw	32
255	Belgium	2011	Fish, Liver Oil	1

FAO country code	Country	Reference year	Food item	Gross food supply (g $capita^{-1}$ d^{-1})
255	Belgium	2011	Freshwater Fish	16
255	Belgium	2011	Fruits, Other	32
255	Belgium	2011	Grapefruit	6
255	Belgium	2011	Grapes	11
255	Belgium	2011	Groundnuts (Shelled Eq)	1
255	Belgium	2011	Honey	2
255	Belgium	2011	Lemons, Limes	6
255	Belgium	2011	Maize	6
255	Belgium	2011	Maize Germ Oil	5
255	Belgium	2011	Marine Fish, Other	4
255	Belgium	2011	Meat, Other	3
255	Belgium	2011	Milk, Whole	145
255	Belgium	2011	Molluscs, Other	12
255	Belgium	2011	Mutton & Goat Meat	4
255	Belgium	2011	Nuts	16
255	Belgium	2011	Offals, Edible	7
255	Belgium	2011	Oilcrops Oil, Other	8
255	Belgium	2011	Olive Oil	4
255	Belgium	2011	Olives	2
255	Belgium	2011	Onions	1
255	Belgium	2011	Oranges, Mandarines	21
255	Belgium	2011	Palm Oil	16
255	Belgium	2011	Peas	3
255	Belgium	2011	Pelagic Fish	11
255	Belgium	2011	Pigmeat	96
255	Belgium	2011	Pineapples	6
255	Belgium	2011	Plantains	7
255	Belgium	2011	Potatoes	290
255	Belgium	2011	Poultry Meat	63
255	Belgium	2011	Pulses, Other	1
255	Belgium	2011	Rape and Mustard Oil	8
255	Belgium	2011	Rice (Milled Equivalent)	23
255	Belgium	2011	Rye	2
255	Belgium	2011	Soyabean Oil	14
255	Belgium	2011	Spices, Other	1
255	Belgium	2011	Sugar, Refined Equivalent	113
255	Belgium	2011	Sunflowerseed Oil	5
255	Belgium	2011	Sweeteners, Other	16
255	Belgium	2011	Tea	1
255	Belgium	2011	Tomatoes	75
255	Belgium	2011	Vegetables, Other	274
255	Belgium	2011	Wheat	328
255	Belgium	2011	Wine	78
53	Benin	1992	Bananas	7
53	Benin	1992	Beans	21
53	Benin	1992	Beer	14
53	Benin	1992	Beverages, Alcoholic	1
53	Benin	1992	Beverages, Fermented	12
53	Benin	1992	Bovine Meat	11
53	Benin	1992	Cassava	381
53	Benin	1992	Cereals, Other	5
53	Benin	1992	Coconut Oil	1
53	Benin	1992	Coconuts - Incl Copra	1
53	Benin	1992	Crustaceans	3
53	Benin	1992	Dates	1
53	Benin	1992	Demersal Fish	2
53	Benin	1992	Eggs	2
53	Benin	1992	Fats, Animals, Raw	1
53	Benin	1992	Freshwater Fish	9
53	Benin	1992	Fruits, Other	59
53	Benin	1992	Groundnut Oil	3
53	Benin	1992	Groundnuts (Shelled Eq)	17
53	Benin	1992	Maize	150
53	Benin	1992	Marine Fish, Other	3
53	Benin	1992	Meat, Other	3
53	Benin	1992	Milk, Whole	14
53	Benin	1992	Millet	9
53	Benin	1992	Mutton & Goat Meat	3
53	Benin	1992	Nuts	2
53	Benin	1992	Offals, Edible	3
53	Benin	1992	Oilcrops Oil, Other	1
53	Benin	1992	Onions	4
53	Benin	1992	Oranges, Mandarines	6
53	Benin	1992	Palm Oil	7
53	Benin	1992	Palmkernel Oil	2
53	Benin	1992	Pelagic Fish	6
53	Benin	1992	Pigmeat	3
53	Benin	1992	Pimento	9
53	Benin	1992	Pineapples	13
53	Benin	1992	Poultry Meat	10
53	Benin	1992	Pulses, Other	4
53	Benin	1992	Rice (Milled Equivalent)	38
53	Benin	1992	Roots, Other	1
53	Benin	1992	Sesameseed	3
53	Benin	1992	Sorghum	45
53	Benin	1992	Sugar, Refined Equivalent	11
53	Benin	1992	Sweet Potatoes	16
53	Benin	1992	Tomatoes	42
53	Benin	1992	Vegetables, Other	74
53	Benin	1992	Wheat	38
53	Benin	1992	Wine	1
53	Benin	1992	Yams	290
53	Benin	1993	Bananas	9
53	Benin	1993	Beans	19
53	Benin	1993	Beer	16
53	Benin	1993	Beverages, Alcoholic	1
53	Benin	1993	Beverages, Fermented	11

FAO country code	Country	Reference year	Food item	Gross food supply (g *capita*$^{-1}$ d^{-1})
53	Benin	1993	Bovine Meat	12
53	Benin	1993	Cassava	380
53	Benin	1993	Cereals, Other	2
53	Benin	1993	Coconut Oil	1
53	Benin	1993	Coconuts - Incl Copra	1
53	Benin	1993	Cottonseed Oil	1
53	Benin	1993	Crustaceans	4
53	Benin	1993	Dates	1
53	Benin	1993	Demersal Fish	2
53	Benin	1993	Eggs	2
53	Benin	1993	Fats, Animals, Raw	1
53	Benin	1993	Freshwater Fish	11
53	Benin	1993	Fruits, Other	55
53	Benin	1993	Groundnut Oil	3
53	Benin	1993	Groundnuts (Shelled Eq)	17
53	Benin	1993	Maize	151
53	Benin	1993	Marine Fish, Other	5
53	Benin	1993	Meat, Other	3
53	Benin	1993	Milk, Whole	15
53	Benin	1993	Millet	7
53	Benin	1993	Mutton & Goat Meat	3
53	Benin	1993	Offals, Edible	2
53	Benin	1993	Oilcrops Oil, Other	3
53	Benin	1993	Onions	4
53	Benin	1993	Oranges, Mandarines	6
53	Benin	1993	Palm Oil	6
53	Benin	1993	Palmkernel Oil	2
53	Benin	1993	Pelagic Fish	7
53	Benin	1993	Pigmeat	3
53	Benin	1993	Pimento	7
53	Benin	1993	Pineapples	13
53	Benin	1993	Poultry Meat	14
53	Benin	1993	Pulses, Other	3
53	Benin	1993	Rice (Milled Equivalent)	50
53	Benin	1993	Roots, Other	1
53	Benin	1993	Sesameseed	3
53	Benin	1993	Sorghum	42
53	Benin	1993	Sugar, Refined Equivalent	14
53	Benin	1993	Sweet Potatoes	18
53	Benin	1993	Tomatoes	39
53	Benin	1993	Vegetables, Other	73
53	Benin	1993	Wheat	22
53	Benin	1993	Wine	1
53	Benin	1993	Yams	295
53	Benin	1994	Bananas	9
53	Benin	1994	Beans	21
53	Benin	1994	Beer	14
53	Benin	1994	Beverages, Alcoholic	1
53	Benin	1994	Beverages, Fermented	11
53	Benin	1994	Bovine Meat	10
53	Benin	1994	Cassava	379
53	Benin	1994	Cereals, Other	3
53	Benin	1994	Coconut Oil	1
53	Benin	1994	Coconuts - Incl Copra	1
53	Benin	1994	Cottonseed Oil	1
53	Benin	1994	Crustaceans	3
53	Benin	1994	Dates	1
53	Benin	1994	Demersal Fish	2
53	Benin	1994	Eggs	2
53	Benin	1994	Fats, Animals, Raw	1
53	Benin	1994	Freshwater Fish	11
53	Benin	1994	Fruits, Other	54
53	Benin	1994	Groundnut Oil	3
53	Benin	1994	Groundnuts (Shelled Eq)	16
53	Benin	1994	Maize	155
53	Benin	1994	Marine Fish, Other	4
53	Benin	1994	Meat, Other	4
53	Benin	1994	Milk, Whole	14
53	Benin	1994	Millet	7
53	Benin	1994	Mutton & Goat Meat	3
53	Benin	1994	Nuts	1
53	Benin	1994	Offals, Edible	3
53	Benin	1994	Oilcrops Oil, Other	1
53	Benin	1994	Onions	4
53	Benin	1994	Oranges, Mandarines	6
53	Benin	1994	Palm Oil	6
53	Benin	1994	Palmkernel Oil	1
53	Benin	1994	Pelagic Fish	5
53	Benin	1994	Pigmeat	3
53	Benin	1994	Pimento	7
53	Benin	1994	Pineapples	13
53	Benin	1994	Poultry Meat	12
53	Benin	1994	Pulses, Other	3
53	Benin	1994	Rice (Milled Equivalent)	48
53	Benin	1994	Roots, Other	2
53	Benin	1994	Sesameseed	3
53	Benin	1994	Sorghum	41
53	Benin	1994	Sugar, Refined Equivalent	21
53	Benin	1994	Sweet Potatoes	20
53	Benin	1994	Tomatoes	48
53	Benin	1994	Vegetables, Other	78
53	Benin	1994	Wheat	30
53	Benin	1994	Wine	1
53	Benin	1994	Yams	307
53	Benin	1995	Bananas	8
53	Benin	1995	Beans	18
53	Benin	1995	Beer	14

FAO country code	Country	Reference year	Food item	Gross food supply (g $capita^{-1}$ d^{-1})
53	Benin	1995	Beverages, Alcoholic	1
53	Benin	1995	Beverages, Fermented	12
53	Benin	1995	Bovine Meat	8
53	Benin	1995	Cassava	394
53	Benin	1995	Cereals, Other	2
53	Benin	1995	Coconut Oil	1
53	Benin	1995	Coconuts - Incl Copra	1
53	Benin	1995	Cottonseed Oil	1
53	Benin	1995	Crustaceans	3
53	Benin	1995	Dates	1
53	Benin	1995	Demersal Fish	1
53	Benin	1995	Eggs	2
53	Benin	1995	Fats, Animals, Raw	1
53	Benin	1995	Freshwater Fish	13
53	Benin	1995	Fruits, Other	51
53	Benin	1995	Groundnut Oil	3
53	Benin	1995	Groundnuts (Shelled Eq)	20
53	Benin	1995	Maize	140
53	Benin	1995	Marine Fish, Other	7
53	Benin	1995	Meat, Other	3
53	Benin	1995	Milk, Whole	15
53	Benin	1995	Millet	6
53	Benin	1995	Mutton & Goat Meat	3
53	Benin	1995	Nuts	3
53	Benin	1995	Offals, Edible	2
53	Benin	1995	Oilcrops Oil, Other	1
53	Benin	1995	Onions	1
53	Benin	1995	Oranges, Mandarines	5
53	Benin	1995	Palm Oil	6
53	Benin	1995	Palmkernel Oil	1
53	Benin	1995	Pelagic Fish	3
53	Benin	1995	Pigmeat	3
53	Benin	1995	Pimento	10
53	Benin	1995	Pineapples	15
53	Benin	1995	Poultry Meat	13
53	Benin	1995	Pulses, Other	3
53	Benin	1995	Rice (Milled Equivalent)	57
53	Benin	1995	Roots, Other	1
53	Benin	1995	Sesameseed	4
53	Benin	1995	Sorghum	43
53	Benin	1995	Sugar, Refined Equivalent	20
53	Benin	1995	Sweet Potatoes	20
53	Benin	1995	Tomatoes	53
53	Benin	1995	Vegetables, Other	65
53	Benin	1995	Wheat	32
53	Benin	1995	Wine	1
53	Benin	1995	Yams	291
53	Benin	1996	Bananas	5
53	Benin	1996	Beans	18
53	Benin	1996	Beer	20
53	Benin	1996	Beverages, Alcoholic	2
53	Benin	1996	Beverages, Fermented	10
53	Benin	1996	Bovine Meat	7
53	Benin	1996	Cassava	421
53	Benin	1996	Cereals, Other	2
53	Benin	1996	Coconut Oil	1
53	Benin	1996	Coconuts - Incl Copra	1
53	Benin	1996	Cottonseed Oil	1
53	Benin	1996	Crustaceans	3
53	Benin	1996	Dates	1
53	Benin	1996	Demersal Fish	2
53	Benin	1996	Eggs	2
53	Benin	1996	Fats, Animals, Raw	1
53	Benin	1996	Freshwater Fish	11
53	Benin	1996	Fruits, Other	49
53	Benin	1996	Grapes	1
53	Benin	1996	Groundnut Oil	4
53	Benin	1996	Groundnuts (Shelled Eq)	18
53	Benin	1996	Maize	157
53	Benin	1996	Marine Fish, Other	6
53	Benin	1996	Meat, Other	3
53	Benin	1996	Milk, Whole	17
53	Benin	1996	Millet	7
53	Benin	1996	Mutton & Goat Meat	3
53	Benin	1996	Nuts	4
53	Benin	1996	Offals, Edible	2
53	Benin	1996	Oilcrops Oil, Other	1
53	Benin	1996	Onions	3
53	Benin	1996	Oranges, Mandarines	6
53	Benin	1996	Palm Oil	5
53	Benin	1996	Palmkernel Oil	1
53	Benin	1996	Pelagic Fish	2
53	Benin	1996	Pigmeat	3
53	Benin	1996	Pimento	8
53	Benin	1996	Pineapples	35
53	Benin	1996	Poultry Meat	12
53	Benin	1996	Pulses, Other	4
53	Benin	1996	Rice (Milled Equivalent)	57
53	Benin	1996	Roots, Other	1
53	Benin	1996	Sesameseed	3
53	Benin	1996	Sorghum	38
53	Benin	1996	Sugar, Refined Equivalent	14
53	Benin	1996	Sweet Potatoes	25
53	Benin	1996	Tomatoes	52
53	Benin	1996	Vegetables, Other	78
53	Benin	1996	Wheat	22
53	Benin	1996	Wine	1

FAO countrycode	Country	Reference year	Food item	Gross food supply (g *capita*$^{-1}$ d^{-1})
53	Benin	1996	Yams	304
53	Benin	1997	Apples	1
53	Benin	1997	Bananas	3
53	Benin	1997	Beans	21
53	Benin	1997	Beer	18
53	Benin	1997	Beverages, Alcoholic	2
53	Benin	1997	Beverages, Fermented	11
53	Benin	1997	Bovine Meat	7
53	Benin	1997	Cassava	444
53	Benin	1997	Cereals, Other	3
53	Benin	1997	Coconut Oil	1
53	Benin	1997	Coconuts - Incl Copra	1
53	Benin	1997	Cottonseed Oil	1
53	Benin	1997	Crustaceans	3
53	Benin	1997	Dates	1
53	Benin	1997	Demersal Fish	2
53	Benin	1997	Eggs	2
53	Benin	1997	Fats, Animals, Raw	1
53	Benin	1997	Freshwater Fish	10
53	Benin	1997	Fruits, Other	48
53	Benin	1997	Grapes	1
53	Benin	1997	Groundnut Oil	4
53	Benin	1997	Groundnuts (Shelled Eq)	18
53	Benin	1997	Maize	176
53	Benin	1997	Marine Fish, Other	7
53	Benin	1997	Meat, Other	4
53	Benin	1997	Milk, Whole	19
53	Benin	1997	Millet	7
53	Benin	1997	Mutton & Goat Meat	3
53	Benin	1997	Nuts	3
53	Benin	1997	Offals, Edible	2
53	Benin	1997	Oilcrops Oil, Other	1
53	Benin	1997	Onions	4
53	Benin	1997	Oranges, Mandarines	6
53	Benin	1997	Palm Oil	3
53	Benin	1997	Palmkernel Oil	2
53	Benin	1997	Pelagic Fish	2
53	Benin	1997	Pigmeat	3
53	Benin	1997	Pimento	7
53	Benin	1997	Pineapples	23
53	Benin	1997	Poultry Meat	14
53	Benin	1997	Pulses, Other	5
53	Benin	1997	Rice (Milled Equivalent)	40
53	Benin	1997	Roots, Other	1
53	Benin	1997	Sesameseed	4
53	Benin	1997	Sorghum	40
53	Benin	1997	Sugar, Refined Equivalent	13
53	Benin	1997	Sweet Potatoes	22
53	Benin	1997	Tomatoes	63
53	Benin	1997	Vegetables, Other	68
53	Benin	1997	Wheat	19
53	Benin	1997	Wine	1
53	Benin	1997	Yams	298
53	Benin	1998	Apples	1
53	Benin	1998	Bananas	3
53	Benin	1998	Beans	21
53	Benin	1998	Beer	17
53	Benin	1998	Beverages, Alcoholic	2
53	Benin	1998	Beverages, Fermented	12
53	Benin	1998	Bovine Meat	8
53	Benin	1998	Cassava	369
53	Benin	1998	Cereals, Other	2
53	Benin	1998	Coconut Oil	1
53	Benin	1998	Coconuts - Incl Copra	1
53	Benin	1998	Cottonseed Oil	2
53	Benin	1998	Crustaceans	3
53	Benin	1998	Dates	1
53	Benin	1998	Demersal Fish	2
53	Benin	1998	Eggs	2
53	Benin	1998	Freshwater Fish	9
53	Benin	1998	Fruits, Other	37
53	Benin	1998	Groundnut Oil	4
53	Benin	1998	Groundnuts (Shelled Eq)	17
53	Benin	1998	Maize	175
53	Benin	1998	Marine Fish, Other	3
53	Benin	1998	Meat, Other	4
53	Benin	1998	Milk, Whole	33
53	Benin	1998	Millet	8
53	Benin	1998	Mutton & Goat Meat	3
53	Benin	1998	Nuts	5
53	Benin	1998	Offals, Edible	2
53	Benin	1998	Oilcrops Oil, Other	1
53	Benin	1998	Onions	4
53	Benin	1998	Oranges, Mandarines	6
53	Benin	1998	Palm Oil	3
53	Benin	1998	Palmkernel Oil	2
53	Benin	1998	Pelagic Fish	5
53	Benin	1998	Pigmeat	1
53	Benin	1998	Pimento	6
53	Benin	1998	Pineapples	17
53	Benin	1998	Poultry Meat	13
53	Benin	1998	Pulses, Other	5
53	Benin	1998	Rape and Mustard Oil	1
53	Benin	1998	Rice (Milled Equivalent)	37
53	Benin	1998	Roots, Other	1
53	Benin	1998	Sesameseed	4
53	Benin	1998	Sorghum	45

FAO country code	Country	Reference year	Food item	Gross food supply (g *capita*$^{-1}$ d^{-1})
53	Benin	1998	Soyabean Oil	1
53	Benin	1998	Sugar, Refined Equivalent	12
53	Benin	1998	Sweet Potatoes	15
53	Benin	1998	Tomatoes	66
53	Benin	1998	Vegetables, Other	63
53	Benin	1998	Wheat	25
53	Benin	1998	Wine	2
53	Benin	1998	Yams	348
53	Benin	1999	Apples	1
53	Benin	1999	Bananas	3
53	Benin	1999	Beans	20
53	Benin	1999	Beer	11
53	Benin	1999	Beverages, Alcoholic	3
53	Benin	1999	Beverages, Fermented	11
53	Benin	1999	Bovine Meat	8
53	Benin	1999	Cassava	374
53	Benin	1999	Cereals, Other	1
53	Benin	1999	Coconut Oil	1
53	Benin	1999	Coconuts - Incl Copra	1
53	Benin	1999	Cottonseed Oil	1
53	Benin	1999	Crustaceans	3
53	Benin	1999	Dates	1
53	Benin	1999	Demersal Fish	2
53	Benin	1999	Eggs	2
53	Benin	1999	Freshwater Fish	9
53	Benin	1999	Fruits, Other	44
53	Benin	1999	Groundnut Oil	4
53	Benin	1999	Groundnuts (Shelled Eq)	17
53	Benin	1999	Maize	183
53	Benin	1999	Marine Fish, Other	3
53	Benin	1999	Meat, Other	3
53	Benin	1999	Milk, Whole	23
53	Benin	1999	Millet	7
53	Benin	1999	Mutton & Goat Meat	3
53	Benin	1999	Nuts	2
53	Benin	1999	Offals, Edible	2
53	Benin	1999	Oilcrops Oil, Other	1
53	Benin	1999	Onions	5
53	Benin	1999	Oranges, Mandarines	6
53	Benin	1999	Palm Oil	3
53	Benin	1999	Palmkernel Oil	2
53	Benin	1999	Pelagic Fish	7
53	Benin	1999	Pigmeat	1
53	Benin	1999	Pimento	6
53	Benin	1999	Pineapples	29
53	Benin	1999	Poultry Meat	22
53	Benin	1999	Pulses, Other	6
53	Benin	1999	Rape and Mustard Oil	3
53	Benin	1999	Rice (Milled Equivalent)	28
53	Benin	1999	Roots, Other	2
53	Benin	1999	Sesameseed	3
53	Benin	1999	Sorghum	40
53	Benin	1999	Soyabean Oil	2
53	Benin	1999	Sugar, Refined Equivalent	15
53	Benin	1999	Sweet Potatoes	25
53	Benin	1999	Tomatoes	70
53	Benin	1999	Vegetables, Other	83
53	Benin	1999	Wheat	24
53	Benin	1999	Wine	2
53	Benin	1999	Yams	346
53	Benin	2000	Apples	2
53	Benin	2000	Bananas	3
53	Benin	2000	Beans	23
53	Benin	2000	Beer	14
53	Benin	2000	Beverages, Alcoholic	1
53	Benin	2000	Beverages, Fermented	13
53	Benin	2000	Bovine Meat	7
53	Benin	2000	Cassava	391
53	Benin	2000	Cereals, Other	1
53	Benin	2000	Coconut Oil	1
53	Benin	2000	Coconuts - Incl Copra	1
53	Benin	2000	Cottonseed Oil	1
53	Benin	2000	Crustaceans	2
53	Benin	2000	Dates	1
53	Benin	2000	Demersal Fish	1
53	Benin	2000	Eggs	2
53	Benin	2000	Freshwater Fish	7
53	Benin	2000	Fruits, Other	31
53	Benin	2000	Grapes	1
53	Benin	2000	Groundnut Oil	4
53	Benin	2000	Groundnuts (Shelled Eq)	21
53	Benin	2000	Maize	169
53	Benin	2000	Marine Fish, Other	2
53	Benin	2000	Meat, Other	3
53	Benin	2000	Milk, Whole	22
53	Benin	2000	Millet	9
53	Benin	2000	Mutton & Goat Meat	3
53	Benin	2000	Nuts	1
53	Benin	2000	Offals, Edible	2
53	Benin	2000	Oilcrops Oil, Other	1
53	Benin	2000	Onions	5
53	Benin	2000	Oranges, Mandarines	5
53	Benin	2000	Palm Oil	4
53	Benin	2000	Palmkernel Oil	2
53	Benin	2000	Pelagic Fish	9
53	Benin	2000	Pigmeat	2
53	Benin	2000	Pimento	6

FAO country code	Country	Reference year	Food item	Gross food supply (g *capita*$^{-1}$ d^{-1})
53	Benin	2000	Pineapples	19
53	Benin	2000	Poultry Meat	27
53	Benin	2000	Pulses, Other	6
53	Benin	2000	Rape and Mustard Oil	1
53	Benin	2000	Rice (Milled Equivalent)	26
53	Benin	2000	Roots, Other	1
53	Benin	2000	Sesameseed	4
53	Benin	2000	Sorghum	48
53	Benin	2000	Soyabean Oil	2
53	Benin	2000	Sugar, Refined Equivalent	12
53	Benin	2000	Sweet Potatoes	23
53	Benin	2000	Tomatoes	65
53	Benin	2000	Vegetables, Other	74
53	Benin	2000	Wheat	19
53	Benin	2000	Wine	2
53	Benin	2000	Yams	365
53	Benin	2001	Apples	2
53	Benin	2001	Bananas	3
53	Benin	2001	Beans	20
53	Benin	2001	Beer	15
53	Benin	2001	Beverages, Fermented	13
53	Benin	2001	Bovine Meat	7
53	Benin	2001	Cassava	438
53	Benin	2001	Cereals, Other	1
53	Benin	2001	Coconut Oil	1
53	Benin	2001	Coconuts - Incl Copra	1
53	Benin	2001	Cottonseed Oil	8
53	Benin	2001	Crustaceans	2
53	Benin	2001	Dates	1
53	Benin	2001	Demersal Fish	2
53	Benin	2001	Eggs	2
53	Benin	2001	Freshwater Fish	8
53	Benin	2001	Fruits, Other	33
53	Benin	2001	Grapes	1
53	Benin	2001	Groundnut Oil	4
53	Benin	2001	Groundnuts (Shelled Eq)	21
53	Benin	2001	Maize	165
53	Benin	2001	Marine Fish, Other	2
53	Benin	2001	Meat, Other	4
53	Benin	2001	Milk, Whole	24
53	Benin	2001	Millet	8
53	Benin	2001	Mutton & Goat Meat	3
53	Benin	2001	Nuts	2
53	Benin	2001	Offals, Edible	2
53	Benin	2001	Oilcrops Oil, Other	1
53	Benin	2001	Onions	6
53	Benin	2001	Oranges, Mandarines	6
53	Benin	2001	Palm Oil	5
53	Benin	2001	Palmkernel Oil	2
53	Benin	2001	Pelagic Fish	6
53	Benin	2001	Pigmeat	1
53	Benin	2001	Pimento	6
53	Benin	2001	Pineapples	19
53	Benin	2001	Poultry Meat	22
53	Benin	2001	Pulses, Other	5
53	Benin	2001	Rice (Milled Equivalent)	39
53	Benin	2001	Roots, Other	1
53	Benin	2001	Sesameseed	4
53	Benin	2001	Sorghum	49
53	Benin	2001	Soyabean Oil	1
53	Benin	2001	Sugar, Refined Equivalent	11
53	Benin	2001	Sweet Potatoes	20
53	Benin	2001	Tomatoes	55
53	Benin	2001	Vegetables, Other	66
53	Benin	2001	Wheat	19
53	Benin	2001	Wine	1
53	Benin	2001	Yams	319
53	Benin	2002	Apples	4
53	Benin	2002	Bananas	3
53	Benin	2002	Beans	25
53	Benin	2002	Beer	17
53	Benin	2002	Beverages, Fermented	13
53	Benin	2002	Bovine Meat	7
53	Benin	2002	Cassava	357
53	Benin	2002	Cereals, Other	1
53	Benin	2002	Coconut Oil	1
53	Benin	2002	Coconuts - Incl Copra	1
53	Benin	2002	Cottonseed Oil	8
53	Benin	2002	Crustaceans	2
53	Benin	2002	Dates	1
53	Benin	2002	Demersal Fish	2
53	Benin	2002	Eggs	2
53	Benin	2002	Freshwater Fish	7
53	Benin	2002	Fruits, Other	32
53	Benin	2002	Grapes	1
53	Benin	2002	Groundnut Oil	4
53	Benin	2002	Groundnuts (Shelled Eq)	26
53	Benin	2002	Maize	160
53	Benin	2002	Marine Fish, Other	3
53	Benin	2002	Meat, Other	7
53	Benin	2002	Milk, Whole	27
53	Benin	2002	Millet	10
53	Benin	2002	Mutton & Goat Meat	2
53	Benin	2002	Nuts	3
53	Benin	2002	Offals, Edible	2
53	Benin	2002	Oilcrops Oil, Other	1
53	Benin	2002	Onions	5

FAO country code	Country	Reference year	Food item	Gross food supply (g $capita^{-1}$ d^{-1})
53	Benin	2002	Oranges, Mandarines	6
53	Benin	2002	Palm Oil	6
53	Benin	2002	Palmkernel Oil	2
53	Benin	2002	Pelagic Fish	9
53	Benin	2002	Pigmeat	1
53	Benin	2002	Pimento	6
53	Benin	2002	Pineapples	28
53	Benin	2002	Potatoes	1
53	Benin	2002	Poultry Meat	30
53	Benin	2002	Pulses, Other	6
53	Benin	2002	Rice (Milled Equivalent)	48
53	Benin	2002	Roots, Other	1
53	Benin	2002	Sesameseed	4
53	Benin	2002	Sorghum	48
53	Benin	2002	Soyabean Oil	1
53	Benin	2002	Sugar, Refined Equivalent	13
53	Benin	2002	Sweet Potatoes	25
53	Benin	2002	Tomatoes	57
53	Benin	2002	Vegetables, Other	74
53	Benin	2002	Wheat	23
53	Benin	2002	Wine	1
53	Benin	2002	Yams	369
53	Benin	2003	Apples	3
53	Benin	2003	Bananas	4
53	Benin	2003	Beans	20
53	Benin	2003	Beer	17
53	Benin	2003	Beverages, Alcoholic	1
53	Benin	2003	Beverages, Fermented	12
53	Benin	2003	Bovine Meat	7
53	Benin	2003	Cassava	344
53	Benin	2003	Cereals, Other	1
53	Benin	2003	Coconut Oil	1
53	Benin	2003	Coconuts - Incl Copra	1
53	Benin	2003	Cottonseed Oil	9
53	Benin	2003	Crustaceans	2
53	Benin	2003	Demersal Fish	2
53	Benin	2003	Eggs	2
53	Benin	2003	Freshwater Fish	8
53	Benin	2003	Fruits, Other	33
53	Benin	2003	Groundnut Oil	4
53	Benin	2003	Groundnuts (Shelled Eq)	20
53	Benin	2003	Maize	158
53	Benin	2003	Marine Fish, Other	6
53	Benin	2003	Meat, Other	9
53	Benin	2003	Milk, Whole	22
53	Benin	2003	Millet	8
53	Benin	2003	Mutton & Goat Meat	2
53	Benin	2003	Nuts	3
53	Benin	2003	Offals, Edible	2
53	Benin	2003	Oilcrops Oil, Other	1
53	Benin	2003	Onions	6
53	Benin	2003	Oranges, Mandarines	6
53	Benin	2003	Palm Oil	5
53	Benin	2003	Palmkernel Oil	2
53	Benin	2003	Pelagic Fish	7
53	Benin	2003	Pigmeat	2
53	Benin	2003	Pimento	6
53	Benin	2003	Pineapples	34
53	Benin	2003	Potatoes	1
53	Benin	2003	Poultry Meat	30
53	Benin	2003	Pulses, Other	6
53	Benin	2003	Rice (Milled Equivalent)	58
53	Benin	2003	Roots, Other	1
53	Benin	2003	Sesameseed	4
53	Benin	2003	Sorghum	45
53	Benin	2003	Soyabean Oil	1
53	Benin	2003	Sugar, Refined Equivalent	14
53	Benin	2003	Sweet Potatoes	16
53	Benin	2003	Tomatoes	52
53	Benin	2003	Vegetables, Other	77
53	Benin	2003	Wheat	19
53	Benin	2003	Wine	2
53	Benin	2003	Yams	379
53	Benin	2004	Apples	2
53	Benin	2004	Bananas	4
53	Benin	2004	Beans	27
53	Benin	2004	Beer	21
53	Benin	2004	Beverages, Alcoholic	1
53	Benin	2004	Beverages, Fermented	12
53	Benin	2004	Bovine Meat	7
53	Benin	2004	Cassava	354
53	Benin	2004	Coconut Oil	1
53	Benin	2004	Coconuts - Incl Copra	1
53	Benin	2004	Cottonseed Oil	8
53	Benin	2004	Crustaceans	2
53	Benin	2004	Demersal Fish	2
53	Benin	2004	Eggs	2
53	Benin	2004	Fats, Animals, Raw	2
53	Benin	2004	Freshwater Fish	7
53	Benin	2004	Fruits, Other	32
53	Benin	2004	Groundnut Oil	4
53	Benin	2004	Groundnuts (Shelled Eq)	21
53	Benin	2004	Maize	158
53	Benin	2004	Marine Fish, Other	6
53	Benin	2004	Meat, Other	3
53	Benin	2004	Milk, Whole	24
53	Benin	2004	Millet	8

FAO country code	Country	Reference year	Food item	Gross food supply (g *capita*$^{-1}$ d^{-1})
53	Benin	2004	Mutton & Goat Meat	2
53	Benin	2004	Nuts	1
53	Benin	2004	Offals, Edible	2
53	Benin	2004	Oilcrops Oil, Other	1
53	Benin	2004	Onions	3
53	Benin	2004	Oranges, Mandarines	6
53	Benin	2004	Palm Oil	6
53	Benin	2004	Palmkernel Oil	2
53	Benin	2004	Pelagic Fish	6
53	Benin	2004	Pigmeat	2
53	Benin	2004	Pimento	5
53	Benin	2004	Pineapples	34
53	Benin	2004	Potatoes	1
53	Benin	2004	Poultry Meat	25
53	Benin	2004	Pulses, Other	8
53	Benin	2004	Rice (Milled Equivalent)	97
53	Benin	2004	Roots, Other	1
53	Benin	2004	Sesameseed	3
53	Benin	2004	Sorghum	44
53	Benin	2004	Sugar, Refined Equivalent	9
53	Benin	2004	Sweet Potatoes	16
53	Benin	2004	Tomatoes	53
53	Benin	2004	Vegetables, Other	70
53	Benin	2004	Wheat	13
53	Benin	2004	Wine	2
53	Benin	2004	Yams	381
53	Benin	2005	Apples	3
53	Benin	2005	Bananas	5
53	Benin	2005	Beans	22
53	Benin	2005	Beer	20
53	Benin	2005	Beverages, Alcoholic	1
53	Benin	2005	Beverages, Fermented	11
53	Benin	2005	Bovine Meat	7
53	Benin	2005	Cassava	350
53	Benin	2005	Cereals, Other	1
53	Benin	2005	Coconut Oil	1
53	Benin	2005	Coconuts - Incl Copra	1
53	Benin	2005	Cottonseed Oil	6
53	Benin	2005	Crustaceans	2
53	Benin	2005	Demersal Fish	2
53	Benin	2005	Eggs	2
53	Benin	2005	Fats, Animals, Raw	4
53	Benin	2005	Freshwater Fish	8
53	Benin	2005	Fruits, Other	32
53	Benin	2005	Groundnut Oil	3
53	Benin	2005	Groundnuts (Shelled Eq)	23
53	Benin	2005	Maize	156
53	Benin	2005	Marine Fish, Other	12
53	Benin	2005	Meat, Other	3
53	Benin	2005	Milk, Whole	23
53	Benin	2005	Millet	8
53	Benin	2005	Mutton & Goat Meat	2
53	Benin	2005	Nuts	1
53	Benin	2005	Offals, Edible	2
53	Benin	2005	Oilcrops Oil, Other	1
53	Benin	2005	Onions	4
53	Benin	2005	Oranges, Mandarines	6
53	Benin	2005	Palm Oil	7
53	Benin	2005	Palmkernel Oil	2
53	Benin	2005	Pelagic Fish	3
53	Benin	2005	Pigmeat	2
53	Benin	2005	Pimento	5
53	Benin	2005	Pineapples	36
53	Benin	2005	Potatoes	1
53	Benin	2005	Poultry Meat	22
53	Benin	2005	Pulses, Other	5
53	Benin	2005	Rice (Milled Equivalent)	116
53	Benin	2005	Roots, Other	1
53	Benin	2005	Sesameseed	4
53	Benin	2005	Sorghum	43
53	Benin	2005	Soyabean Oil	1
53	Benin	2005	Sugar, Refined Equivalent	8
53	Benin	2005	Sweet Potatoes	19
53	Benin	2005	Tomatoes	53
53	Benin	2005	Vegetables, Other	79
53	Benin	2005	Wheat	9
53	Benin	2005	Wine	2
53	Benin	2005	Yams	308
53	Benin	2006	Apples	3
53	Benin	2006	Bananas	3
53	Benin	2006	Beans	27
53	Benin	2006	Beer	22
53	Benin	2006	Beverages, Alcoholic	1
53	Benin	2006	Beverages, Fermented	11
53	Benin	2006	Bovine Meat	7
53	Benin	2006	Cassava	354
53	Benin	2006	Coconut Oil	1
53	Benin	2006	Coconuts - Incl Copra	1
53	Benin	2006	Cottonseed Oil	6
53	Benin	2006	Crustaceans	2
53	Benin	2006	Demersal Fish	2
53	Benin	2006	Eggs	2
53	Benin	2006	Fats, Animals, Raw	3
53	Benin	2006	Freshwater Fish	9
53	Benin	2006	Fruits, Other	32
53	Benin	2006	Grapes	1
53	Benin	2006	Groundnut Oil	3

FAO country code	Country	Reference year	Food item	Gross food supply (g $capita^{-1}$ d^{-1})
53	Benin	2006	Groundnuts (Shelled Eq)	18
53	Benin	2006	Maize	134
53	Benin	2006	Marine Fish, Other	18
53	Benin	2006	Meat, Other	3
53	Benin	2006	Milk, Whole	23
53	Benin	2006	Millet	8
53	Benin	2006	Mutton & Goat Meat	2
53	Benin	2006	Nuts	1
53	Benin	2006	Offals, Edible	2
53	Benin	2006	Oilcrops Oil, Other	1
53	Benin	2006	Onions	9
53	Benin	2006	Oranges, Mandarines	6
53	Benin	2006	Palm Oil	4
53	Benin	2006	Palmkernel Oil	2
53	Benin	2006	Pelagic Fish	3
53	Benin	2006	Pigmeat	1
53	Benin	2006	Pimento	6
53	Benin	2006	Pineapples	23
53	Benin	2006	Poultry Meat	21
53	Benin	2006	Pulses, Other	2
53	Benin	2006	Rice (Milled Equivalent)	134
53	Benin	2006	Roots, Other	1
53	Benin	2006	Sesameseed	2
53	Benin	2006	Sorghum	40
53	Benin	2006	Sugar, Refined Equivalent	8
53	Benin	2006	Sweet Potatoes	16
53	Benin	2006	Tomatoes	51
53	Benin	2006	Vegetables, Other	72
53	Benin	2006	Wheat	15
53	Benin	2006	Wine	2
53	Benin	2006	Yams	371
53	Benin	2007	Apples	4
53	Benin	2007	Bananas	4
53	Benin	2007	Barley	1
53	Benin	2007	Beans	28
53	Benin	2007	Beer	22
53	Benin	2007	Beverages, Fermented	10
53	Benin	2007	Bovine Meat	7
53	Benin	2007	Cassava	411
53	Benin	2007	Coconut Oil	1
53	Benin	2007	Coconuts - Incl Copra	1
53	Benin	2007	Cottonseed Oil	6
53	Benin	2007	Crustaceans	1
53	Benin	2007	Demersal Fish	2
53	Benin	2007	Eggs	3
53	Benin	2007	Fats, Animals, Raw	3
53	Benin	2007	Freshwater Fish	12
53	Benin	2007	Fruits, Other	34
53	Benin	2007	Grapes	1
53	Benin	2007	Groundnut Oil	3
53	Benin	2007	Groundnuts (Shelled Eq)	16
53	Benin	2007	Maize	128
53	Benin	2007	Marine Fish, Other	18
53	Benin	2007	Meat, Other	2
53	Benin	2007	Milk, Whole	22
53	Benin	2007	Millet	8
53	Benin	2007	Mutton & Goat Meat	2
53	Benin	2007	Nuts	6
53	Benin	2007	Offals, Edible	2
53	Benin	2007	Onions	6
53	Benin	2007	Oranges, Mandarines	5
53	Benin	2007	Palm Oil	6
53	Benin	2007	Palmkernel Oil	2
53	Benin	2007	Pelagic Fish	3
53	Benin	2007	Pigmeat	2
53	Benin	2007	Pimento	9
53	Benin	2007	Pineapples	37
53	Benin	2007	Poultry Meat	22
53	Benin	2007	Pulses, Other	13
53	Benin	2007	Rice (Milled Equivalent)	134
53	Benin	2007	Roots, Other	1
53	Benin	2007	Sesameseed	3
53	Benin	2007	Sorghum	39
53	Benin	2007	Soyabean Oil	1
53	Benin	2007	Sugar, Refined Equivalent	7
53	Benin	2007	Sweet Potatoes	19
53	Benin	2007	Tomatoes	57
53	Benin	2007	Vegetables, Other	57
53	Benin	2007	Wheat	13
53	Benin	2007	Wine	1
53	Benin	2007	Yams	372
53	Benin	2008	Apples	6
53	Benin	2008	Bananas	4
53	Benin	2008	Beans	29
53	Benin	2008	Beer	23
53	Benin	2008	Beverages, Alcoholic	1
53	Benin	2008	Beverages, Fermented	9
53	Benin	2008	Bovine Meat	7
53	Benin	2008	Cassava	258
53	Benin	2008	Cereals, Other	1
53	Benin	2008	Coconut Oil	1
53	Benin	2008	Coconuts - Incl Copra	1
53	Benin	2008	Cottonseed Oil	6
53	Benin	2008	Crustaceans	1
53	Benin	2008	Demersal Fish	2
53	Benin	2008	Eggs	3
53	Benin	2008	Fats, Animals, Raw	1

FAO country code	Country	Reference year	Food item	Gross food supply (g *capita*$^{-1}$ d^{-1})
53	Benin	2008	Freshwater Fish	13
53	Benin	2008	Fruits, Other	35
53	Benin	2008	Groundnut Oil	5
53	Benin	2008	Groundnuts (Shelled Eq)	16
53	Benin	2008	Maize	126
53	Benin	2008	Marine Fish, Other	21
53	Benin	2008	Meat, Other	2
53	Benin	2008	Milk, Whole	24
53	Benin	2008	Millet	7
53	Benin	2008	Mutton & Goat Meat	2
53	Benin	2008	Nuts	9
53	Benin	2008	Offals, Edible	2
53	Benin	2008	Onions	3
53	Benin	2008	Oranges, Mandarines	4
53	Benin	2008	Palm Oil	6
53	Benin	2008	Palmkernel Oil	3
53	Benin	2008	Pelagic Fish	3
53	Benin	2008	Pigmeat	2
53	Benin	2008	Pimento	14
53	Benin	2008	Pineapples	37
53	Benin	2008	Poultry Meat	35
53	Benin	2008	Pulses, Other	13
53	Benin	2008	Rice (Milled Equivalent)	146
53	Benin	2008	Roots, Other	1
53	Benin	2008	Sesameseed	3
53	Benin	2008	Sorghum	35
53	Benin	2008	Sugar, Refined Equivalent	13
53	Benin	2008	Sweet Potatoes	20
53	Benin	2008	Sweeteners, Other	1
53	Benin	2008	Tomatoes	77
53	Benin	2008	Vegetables, Other	56
53	Benin	2008	Wheat	20
53	Benin	2008	Wine	1
53	Benin	2008	Yams	387
53	Benin	2009	Apples	5
53	Benin	2009	Bananas	4
53	Benin	2009	Beans	31
53	Benin	2009	Beer	24
53	Benin	2009	Beverages, Alcoholic	1
53	Benin	2009	Beverages, Fermented	9
53	Benin	2009	Bovine Meat	9
53	Benin	2009	Cassava	327
53	Benin	2009	Coconuts - Incl Copra	1
53	Benin	2009	Cottonseed Oil	5
53	Benin	2009	Crustaceans	1
53	Benin	2009	Demersal Fish	2
53	Benin	2009	Eggs	3
53	Benin	2009	Fats, Animals, Raw	1
53	Benin	2009	Freshwater Fish	14
53	Benin	2009	Fruits, Other	36
53	Benin	2009	Grapes	1
53	Benin	2009	Groundnut Oil	5
53	Benin	2009	Groundnuts (Shelled Eq)	18
53	Benin	2009	Maize	127
53	Benin	2009	Marine Fish, Other	17
53	Benin	2009	Meat, Other	2
53	Benin	2009	Milk, Whole	28
53	Benin	2009	Millet	5
53	Benin	2009	Mutton & Goat Meat	2
53	Benin	2009	Nuts	18
53	Benin	2009	Offals, Edible	3
53	Benin	2009	Oilcrops Oil, Other	1
53	Benin	2009	Onions	11
53	Benin	2009	Oranges, Mandarines	4
53	Benin	2009	Palm Oil	5
53	Benin	2009	Palmkernel Oil	2
53	Benin	2009	Pelagic Fish	3
53	Benin	2009	Pigmeat	2
53	Benin	2009	Pimento	8
53	Benin	2009	Pineapples	59
53	Benin	2009	Poultry Meat	29
53	Benin	2009	Pulses, Other	10
53	Benin	2009	Rice (Milled Equivalent)	152
53	Benin	2009	Roots, Other	1
53	Benin	2009	Sesameseed	3
53	Benin	2009	Sorghum	33
53	Benin	2009	Soyabean Oil	1
53	Benin	2009	Sugar, Refined Equivalent	12
53	Benin	2009	Sweet Potatoes	17
53	Benin	2009	Sweeteners, Other	1
53	Benin	2009	Tomatoes	62
53	Benin	2009	Vegetables, Other	48
53	Benin	2009	Wheat	19
53	Benin	2009	Wine	1
53	Benin	2009	Yams	360
53	Benin	2010	Apples	5
53	Benin	2010	Bananas	5
53	Benin	2010	Beans	22
53	Benin	2010	Beer	26
53	Benin	2010	Beverages, Alcoholic	1
53	Benin	2010	Beverages, Fermented	9
53	Benin	2010	Bovine Meat	8
53	Benin	2010	Cassava	319
53	Benin	2010	Coconuts - Incl Copra	1
53	Benin	2010	Cottonseed Oil	2
53	Benin	2010	Crustaceans	1
53	Benin	2010	Demersal Fish	2

FAO country code	Country	Reference year	Food item	Gross food supply (g *capita*$^{-1}$ d^{-1})
53	Benin	2010	Eggs	2
53	Benin	2010	Freshwater Fish	15
53	Benin	2010	Fruits, Other	39
53	Benin	2010	Groundnut Oil	9
53	Benin	2010	Groundnuts (Shelled Eq)	23
53	Benin	2010	Maize	118
53	Benin	2010	Marine Fish, Other	17
53	Benin	2010	Meat, Other	2
53	Benin	2010	Milk, Whole	20
53	Benin	2010	Millet	5
53	Benin	2010	Mutton & Goat Meat	2
53	Benin	2010	Nuts	13
53	Benin	2010	Offals, Edible	2
53	Benin	2010	Onions	2
53	Benin	2010	Oranges, Mandarines	4
53	Benin	2010	Palm Oil	5
53	Benin	2010	Palmkernel Oil	2
53	Benin	2010	Pelagic Fish	3
53	Benin	2010	Pigmeat	2
53	Benin	2010	Pimento	8
53	Benin	2010	Pineapples	69
53	Benin	2010	Potatoes	1
53	Benin	2010	Poultry Meat	24
53	Benin	2010	Pulses, Other	11
53	Benin	2010	Rice (Milled Equivalent)	138
53	Benin	2010	Sesameseed	3
53	Benin	2010	Sorghum	33
53	Benin	2010	Soyabean Oil	1
53	Benin	2010	Sugar, Refined Equivalent	12
53	Benin	2010	Sweet Potatoes	20
53	Benin	2010	Sweeteners, Other	1
53	Benin	2010	Tomatoes	69
53	Benin	2010	Vegetables, Other	30
53	Benin	2010	Wheat	20
53	Benin	2010	Wine	3
53	Benin	2010	Yams	418
53	Benin	2011	Apples	7
53	Benin	2011	Bananas	5
53	Benin	2011	Beans	19
53	Benin	2011	Beer	27
53	Benin	2011	Beverages, Alcoholic	1
53	Benin	2011	Beverages, Fermented	9
53	Benin	2011	Bovine Meat	9
53	Benin	2011	Cassava	387
53	Benin	2011	Coconut Oil	1
53	Benin	2011	Coconuts - Incl Copra	1
53	Benin	2011	Cottonseed Oil	3
53	Benin	2011	Crustaceans	1
53	Benin	2011	Demersal Fish	2
53	Benin	2011	Eggs	2
53	Benin	2011	Fats, Animals, Raw	1
53	Benin	2011	Freshwater Fish	14
53	Benin	2011	Fruits, Other	38
53	Benin	2011	Grapes	1
53	Benin	2011	Groundnut Oil	7
53	Benin	2011	Groundnuts (Shelled Eq)	18
53	Benin	2011	Maize	110
53	Benin	2011	Marine Fish, Other	17
53	Benin	2011	Meat, Other	2
53	Benin	2011	Milk, Whole	21
53	Benin	2011	Millet	4
53	Benin	2011	Mutton & Goat Meat	2
53	Benin	2011	Nuts	12
53	Benin	2011	Offals, Edible	2
53	Benin	2011	Oilcrops Oil, Other	1
53	Benin	2011	Onions	4
53	Benin	2011	Oranges, Mandarines	4
53	Benin	2011	Palm Oil	6
53	Benin	2011	Palmkernel Oil	2
53	Benin	2011	Pelagic Fish	4
53	Benin	2011	Pigmeat	2
53	Benin	2011	Pimento	8
53	Benin	2011	Pineapples	62
53	Benin	2011	Potatoes	1
53	Benin	2011	Poultry Meat	34
53	Benin	2011	Pulses, Other	10
53	Benin	2011	Rice (Milled Equivalent)	146
53	Benin	2011	Sesameseed	3
53	Benin	2011	Sorghum	32
53	Benin	2011	Soyabean Oil	1
53	Benin	2011	Sugar, Refined Equivalent	13
53	Benin	2011	Sweet Potatoes	12
53	Benin	2011	Sweeteners, Other	1
53	Benin	2011	Tomatoes	56
53	Benin	2011	Vegetables, Other	29
53	Benin	2011	Wheat	24
53	Benin	2011	Wine	3
53	Benin	2011	Yams	422
19	Bolivia	1992	Apples	4
19	Bolivia	1992	Bananas	43
19	Bolivia	1992	Barley	1
19	Bolivia	1992	Beans	1
19	Bolivia	1992	Beer	43
19	Bolivia	1992	Beverages, Alcoholic	6
19	Bolivia	1992	Bovine Meat	49
19	Bolivia	1992	Cassava	48
19	Bolivia	1992	Cereals, Other	8

FAO country code	Country	Reference year	Food item	Gross food supply (g $capita^{-1}$ d^{-1})
19	Bolivia	1992	Cheese	3
19	Bolivia	1992	Cocoa Beans	2
19	Bolivia	1992	Coffee	4
19	Bolivia	1992	Cottonseed Oil	1
19	Bolivia	1992	Eggs	17
19	Bolivia	1992	Fats, Animals, Raw	6
19	Bolivia	1992	Freshwater Fish	1
19	Bolivia	1992	Fruits, Other	28
19	Bolivia	1992	Grapefruit	10
19	Bolivia	1992	Grapes	5
19	Bolivia	1992	Groundnuts (Shelled Eq)	2
19	Bolivia	1992	Lemons, Limes	19
19	Bolivia	1992	Maize	73
19	Bolivia	1992	Meat, Other	3
19	Bolivia	1992	Milk, Whole	52
19	Bolivia	1992	Mutton & Goat Meat	7
19	Bolivia	1992	Nuts	14
19	Bolivia	1992	Oats	1
19	Bolivia	1992	Offals, Edible	11
19	Bolivia	1992	Onions	14
19	Bolivia	1992	Oranges, Mandarines	42
19	Bolivia	1992	Peas	2
19	Bolivia	1992	Pelagic Fish	1
19	Bolivia	1992	Pigmeat	26
19	Bolivia	1992	Pineapples	5
19	Bolivia	1992	Plantains	91
19	Bolivia	1992	Potatoes	152
19	Bolivia	1992	Poultry Meat	21
19	Bolivia	1992	Pulses, Other	5
19	Bolivia	1992	Rice (Milled Equivalent)	54
19	Bolivia	1992	Roots, Other	22
19	Bolivia	1992	Soyabean Oil	6
19	Bolivia	1992	Sugar, Refined Equivalent	70
19	Bolivia	1992	Sunflowerseed Oil	5
19	Bolivia	1992	Sweet Potatoes	3
19	Bolivia	1992	Tomatoes	15
19	Bolivia	1992	Vegetables, Other	103
19	Bolivia	1992	Wheat	176
19	Bolivia	1992	Wine	1
19	Bolivia	1993	Apples	5
19	Bolivia	1993	Bananas	42
19	Bolivia	1993	Beer	52
19	Bolivia	1993	Beverages, Alcoholic	5
19	Bolivia	1993	Bovine Meat	49
19	Bolivia	1993	Cassava	43
19	Bolivia	1993	Cereals, Other	7
19	Bolivia	1993	Cheese	3
19	Bolivia	1993	Cocoa Beans	2
19	Bolivia	1993	Coffee	4
19	Bolivia	1993	Cottonseed Oil	1
19	Bolivia	1993	Eggs	17
19	Bolivia	1993	Fats, Animals, Raw	5
19	Bolivia	1993	Freshwater Fish	2
19	Bolivia	1993	Fruits, Other	30
19	Bolivia	1993	Grapefruit	9
19	Bolivia	1993	Grapes	6
19	Bolivia	1993	Groundnuts (Shelled Eq)	2
19	Bolivia	1993	Lemons, Limes	20
19	Bolivia	1993	Maize	79
19	Bolivia	1993	Marine Fish, Other	1
19	Bolivia	1993	Meat, Other	3
19	Bolivia	1993	Milk, Whole	53
19	Bolivia	1993	Mutton & Goat Meat	7
19	Bolivia	1993	Nuts	11
19	Bolivia	1993	Oats	2
19	Bolivia	1993	Offals, Edible	11
19	Bolivia	1993	Onions	15
19	Bolivia	1993	Oranges, Mandarines	44
19	Bolivia	1993	Peas	2
19	Bolivia	1993	Pigmeat	22
19	Bolivia	1993	Pineapples	5
19	Bolivia	1993	Plantains	84
19	Bolivia	1993	Potatoes	121
19	Bolivia	1993	Poultry Meat	27
19	Bolivia	1993	Pulses, Other	5
19	Bolivia	1993	Rice (Milled Equivalent)	51
19	Bolivia	1993	Roots, Other	23
19	Bolivia	1993	Soyabean Oil	6
19	Bolivia	1993	Sugar, Refined Equivalent	71
19	Bolivia	1993	Sunflowerseed Oil	4
19	Bolivia	1993	Sweet Potatoes	3
19	Bolivia	1993	Tomatoes	13
19	Bolivia	1993	Vegetables, Other	108
19	Bolivia	1993	Wheat	184
19	Bolivia	1993	Wine	1
19	Bolivia	1994	Apples	5
19	Bolivia	1994	Bananas	29
19	Bolivia	1994	Beans	1
19	Bolivia	1994	Beer	51
19	Bolivia	1994	Beverages, Alcoholic	6
19	Bolivia	1994	Bovine Meat	50
19	Bolivia	1994	Cassava	44
19	Bolivia	1994	Cereals, Other	7
19	Bolivia	1994	Cheese	3
19	Bolivia	1994	Cocoa Beans	2
19	Bolivia	1994	Coffee	6
19	Bolivia	1994	Cottonseed Oil	1

FAO countrycode	Country	Reference year	Food item	Gross food supply (g *capita*$^{-1}$ d^{-1})
19	Bolivia	1994	Eggs	19
19	Bolivia	1994	Fats, Animals, Raw	5
19	Bolivia	1994	Freshwater Fish	2
19	Bolivia	1994	Fruits, Other	30
19	Bolivia	1994	Grapefruit	9
19	Bolivia	1994	Grapes	7
19	Bolivia	1994	Groundnuts (Shelled Eq)	2
19	Bolivia	1994	Lemons, Limes	20
19	Bolivia	1994	Maize	79
19	Bolivia	1994	Marine Fish, Other	1
19	Bolivia	1994	Meat, Other	3
19	Bolivia	1994	Milk, Whole	58
19	Bolivia	1994	Mutton & Goat Meat	7
19	Bolivia	1994	Nuts	10
19	Bolivia	1994	Oats	2
19	Bolivia	1994	Offals, Edible	11
19	Bolivia	1994	Onions	17
19	Bolivia	1994	Oranges, Mandarines	45
19	Bolivia	1994	Peas	2
19	Bolivia	1994	Pelagic Fish	1
19	Bolivia	1994	Pigmeat	22
19	Bolivia	1994	Pineapples	5
19	Bolivia	1994	Plantains	88
19	Bolivia	1994	Potatoes	143
19	Bolivia	1994	Poultry Meat	32
19	Bolivia	1994	Pulses, Other	4
19	Bolivia	1994	Rice (Milled Equivalent)	56
19	Bolivia	1994	Roots, Other	22
19	Bolivia	1994	Soyabean Oil	7
19	Bolivia	1994	Sugar, Refined Equivalent	70
19	Bolivia	1994	Sunflowerseed Oil	3
19	Bolivia	1994	Sweet Potatoes	3
19	Bolivia	1994	Tomatoes	15
19	Bolivia	1994	Vegetables, Other	106
19	Bolivia	1994	Wheat	148
19	Bolivia	1994	Wine	1
19	Bolivia	1995	Apples	5
19	Bolivia	1995	Bananas	42
19	Bolivia	1995	Beans	1
19	Bolivia	1995	Beer	55
19	Bolivia	1995	Beverages, Alcoholic	7
19	Bolivia	1995	Bovine Meat	50
19	Bolivia	1995	Cassava	48
19	Bolivia	1995	Cereals, Other	7
19	Bolivia	1995	Cheese	4
19	Bolivia	1995	Cocoa Beans	2
19	Bolivia	1995	Coffee	6
19	Bolivia	1995	Cottonseed Oil	2
19	Bolivia	1995	Demersal Fish	1
19	Bolivia	1995	Eggs	20
19	Bolivia	1995	Fats, Animals, Raw	6
19	Bolivia	1995	Freshwater Fish	2
19	Bolivia	1995	Fruits, Other	30
19	Bolivia	1995	Grapefruit	9
19	Bolivia	1995	Grapes	6
19	Bolivia	1995	Groundnuts (Shelled Eq)	2
19	Bolivia	1995	Lemons, Limes	19
19	Bolivia	1995	Maize	110
19	Bolivia	1995	Meat, Other	3
19	Bolivia	1995	Milk, Whole	78
19	Bolivia	1995	Mutton & Goat Meat	7
19	Bolivia	1995	Nuts	13
19	Bolivia	1995	Oats	2
19	Bolivia	1995	Offals, Edible	11
19	Bolivia	1995	Onions	15
19	Bolivia	1995	Oranges, Mandarines	45
19	Bolivia	1995	Peas	1
19	Bolivia	1995	Pelagic Fish	1
19	Bolivia	1995	Pigmeat	22
19	Bolivia	1995	Pineapples	6
19	Bolivia	1995	Plantains	89
19	Bolivia	1995	Potatoes	118
19	Bolivia	1995	Poultry Meat	34
19	Bolivia	1995	Pulses, Other	3
19	Bolivia	1995	Rice (Milled Equivalent)	58
19	Bolivia	1995	Roots, Other	20
19	Bolivia	1995	Soyabean Oil	7
19	Bolivia	1995	Sugar, Refined Equivalent	70
19	Bolivia	1995	Sunflowerseed Oil	5
19	Bolivia	1995	Sweet Potatoes	3
19	Bolivia	1995	Sweeteners, Other	1
19	Bolivia	1995	Tomatoes	19
19	Bolivia	1995	Vegetables, Other	112
19	Bolivia	1995	Wheat	158
19	Bolivia	1995	Wine	1
19	Bolivia	1996	Apples	5
19	Bolivia	1996	Bananas	40
19	Bolivia	1996	Beans	1
19	Bolivia	1996	Beer	53
19	Bolivia	1996	Beverages, Alcoholic	7
19	Bolivia	1996	Bovine Meat	50
19	Bolivia	1996	Cassava	48
19	Bolivia	1996	Cereals, Other	8
19	Bolivia	1996	Cheese	4
19	Bolivia	1996	Cocoa Beans	2
19	Bolivia	1996	Coffee	6
19	Bolivia	1996	Cottonseed Oil	2

FAO country code	Country	Reference year	Food item	Gross food supply (g *capita*$^{-1}$ d^{-1})
19	Bolivia	1996	Demersal Fish	1
19	Bolivia	1996	Eggs	19
19	Bolivia	1996	Fats, Animals, Raw	6
19	Bolivia	1996	Freshwater Fish	2
19	Bolivia	1996	Fruits, Other	30
19	Bolivia	1996	Grapefruit	9
19	Bolivia	1996	Grapes	7
19	Bolivia	1996	Groundnuts (Shelled Eq)	3
19	Bolivia	1996	Lemons, Limes	19
19	Bolivia	1996	Maize	116
19	Bolivia	1996	Meat, Other	3
19	Bolivia	1996	Milk, Whole	87
19	Bolivia	1996	Mutton & Goat Meat	7
19	Bolivia	1996	Nuts	18
19	Bolivia	1996	Oats	2
19	Bolivia	1996	Offals, Edible	11
19	Bolivia	1996	Onions	15
19	Bolivia	1996	Oranges, Mandarines	45
19	Bolivia	1996	Peas	1
19	Bolivia	1996	Pelagic Fish	1
19	Bolivia	1996	Pigmeat	23
19	Bolivia	1996	Pineapples	7
19	Bolivia	1996	Plantains	89
19	Bolivia	1996	Potatoes	124
19	Bolivia	1996	Poultry Meat	36
19	Bolivia	1996	Pulses, Other	3
19	Bolivia	1996	Rice (Milled Equivalent)	64
19	Bolivia	1996	Roots, Other	19
19	Bolivia	1996	Soyabean Oil	8
19	Bolivia	1996	Sugar, Refined Equivalent	69
19	Bolivia	1996	Sunflowerseed Oil	5
19	Bolivia	1996	Sweet Potatoes	3
19	Bolivia	1996	Sweeteners, Other	1
19	Bolivia	1996	Tomatoes	22
19	Bolivia	1996	Vegetables, Other	114
19	Bolivia	1996	Wheat	103
19	Bolivia	1996	Wine	1
19	Bolivia	1997	Apples	6
19	Bolivia	1997	Bananas	39
19	Bolivia	1997	Beans	1
19	Bolivia	1997	Beer	56
19	Bolivia	1997	Beverages, Alcoholic	6
19	Bolivia	1997	Bovine Meat	51
19	Bolivia	1997	Cassava	44
19	Bolivia	1997	Cereals, Other	9
19	Bolivia	1997	Cheese	3
19	Bolivia	1997	Cocoa Beans	2
19	Bolivia	1997	Coffee	6
19	Bolivia	1997	Cottonseed Oil	2
19	Bolivia	1997	Demersal Fish	1
19	Bolivia	1997	Eggs	20
19	Bolivia	1997	Fats, Animals, Raw	5
19	Bolivia	1997	Freshwater Fish	2
19	Bolivia	1997	Fruits, Other	29
19	Bolivia	1997	Grapefruit	9
19	Bolivia	1997	Grapes	7
19	Bolivia	1997	Groundnuts (Shelled Eq)	3
19	Bolivia	1997	Lemons, Limes	19
19	Bolivia	1997	Maize	118
19	Bolivia	1997	Meat, Other	3
19	Bolivia	1997	Milk, Whole	85
19	Bolivia	1997	Mutton & Goat Meat	7
19	Bolivia	1997	Nuts	17
19	Bolivia	1997	Oats	2
19	Bolivia	1997	Offals, Edible	11
19	Bolivia	1997	Onions	15
19	Bolivia	1997	Oranges, Mandarines	46
19	Bolivia	1997	Peas	1
19	Bolivia	1997	Pelagic Fish	1
19	Bolivia	1997	Pigmeat	24
19	Bolivia	1997	Pineapples	14
19	Bolivia	1997	Plantains	92
19	Bolivia	1997	Potatoes	122
19	Bolivia	1997	Poultry Meat	39
19	Bolivia	1997	Pulses, Other	4
19	Bolivia	1997	Rice (Milled Equivalent)	64
19	Bolivia	1997	Roots, Other	17
19	Bolivia	1997	Soyabean Oil	8
19	Bolivia	1997	Sugar, Refined Equivalent	68
19	Bolivia	1997	Sunflowerseed Oil	5
19	Bolivia	1997	Sweet Potatoes	3
19	Bolivia	1997	Tomatoes	26
19	Bolivia	1997	Vegetables, Other	115
19	Bolivia	1997	Wheat	105
19	Bolivia	1997	Wine	1
19	Bolivia	1998	Apples	7
19	Bolivia	1998	Bananas	38
19	Bolivia	1998	Beans	1
19	Bolivia	1998	Beer	55
19	Bolivia	1998	Beverages, Alcoholic	6
19	Bolivia	1998	Bovine Meat	52
19	Bolivia	1998	Cassava	48
19	Bolivia	1998	Cereals, Other	8
19	Bolivia	1998	Cheese	2
19	Bolivia	1998	Cocoa Beans	2
19	Bolivia	1998	Coffee	7
19	Bolivia	1998	Cottonseed Oil	2

FAO country code	Country	Reference year	Food item	Gross food supply (g *capita*$^{-1}$ d^{-1})
19	Bolivia	1998	Eggs	10
19	Bolivia	1998	Fats, Animals, Raw	5
19	Bolivia	1998	Freshwater Fish	2
19	Bolivia	1998	Fruits, Other	29
19	Bolivia	1998	Grapefruit	9
19	Bolivia	1998	Grapes	6
19	Bolivia	1998	Groundnuts (Shelled Eq)	2
19	Bolivia	1998	Lemons, Limes	19
19	Bolivia	1998	Maize	112
19	Bolivia	1998	Marine Fish, Other	1
19	Bolivia	1998	Meat, Other	3
19	Bolivia	1998	Milk, Whole	86
19	Bolivia	1998	Mutton & Goat Meat	7
19	Bolivia	1998	Nuts	13
19	Bolivia	1998	Oats	2
19	Bolivia	1998	Offals, Edible	11
19	Bolivia	1998	Onions	15
19	Bolivia	1998	Oranges, Mandarines	46
19	Bolivia	1998	Peas	1
19	Bolivia	1998	Pelagic Fish	1
19	Bolivia	1998	Pigmeat	24
19	Bolivia	1998	Pineapples	14
19	Bolivia	1998	Plantains	88
19	Bolivia	1998	Potatoes	108
19	Bolivia	1998	Poultry Meat	45
19	Bolivia	1998	Pulses, Other	3
19	Bolivia	1998	Rice (Milled Equivalent)	59
19	Bolivia	1998	Roots, Other	15
19	Bolivia	1998	Soyabean Oil	5
19	Bolivia	1998	Sugar, Refined Equivalent	67
19	Bolivia	1998	Sunflowerseed Oil	4
19	Bolivia	1998	Sweet Potatoes	3
19	Bolivia	1998	Tomatoes	22
19	Bolivia	1998	Vegetables, Other	108
19	Bolivia	1998	Wheat	106
19	Bolivia	1998	Wine	1
19	Bolivia	1999	Apples	7
19	Bolivia	1999	Bananas	37
19	Bolivia	1999	Beer	71
19	Bolivia	1999	Beverages, Alcoholic	5
19	Bolivia	1999	Bovine Meat	51
19	Bolivia	1999	Cassava	51
19	Bolivia	1999	Cereals, Other	8
19	Bolivia	1999	Cheese	3
19	Bolivia	1999	Cocoa Beans	2
19	Bolivia	1999	Coffee	7
19	Bolivia	1999	Cottonseed Oil	1
19	Bolivia	1999	Eggs	10
19	Bolivia	1999	Fats, Animals, Raw	5
19	Bolivia	1999	Freshwater Fish	2
19	Bolivia	1999	Fruits, Other	29
19	Bolivia	1999	Grapefruit	9
19	Bolivia	1999	Grapes	7
19	Bolivia	1999	Groundnuts (Shelled Eq)	2
19	Bolivia	1999	Lemons, Limes	19
19	Bolivia	1999	Maize	100
19	Bolivia	1999	Marine Fish, Other	1
19	Bolivia	1999	Meat, Other	3
19	Bolivia	1999	Milk, Whole	87
19	Bolivia	1999	Mutton & Goat Meat	7
19	Bolivia	1999	Nuts	15
19	Bolivia	1999	Oats	2
19	Bolivia	1999	Offals, Edible	11
19	Bolivia	1999	Onions	15
19	Bolivia	1999	Oranges, Mandarines	48
19	Bolivia	1999	Peas	1
19	Bolivia	1999	Pelagic Fish	1
19	Bolivia	1999	Pigmeat	24
19	Bolivia	1999	Pineapples	16
19	Bolivia	1999	Plantains	88
19	Bolivia	1999	Potatoes	141
19	Bolivia	1999	Poultry Meat	46
19	Bolivia	1999	Pulses, Other	5
19	Bolivia	1999	Rice (Milled Equivalent)	56
19	Bolivia	1999	Roots, Other	16
19	Bolivia	1999	Soyabean Oil	5
19	Bolivia	1999	Sugar, Refined Equivalent	67
19	Bolivia	1999	Sunflowerseed Oil	5
19	Bolivia	1999	Sweet Potatoes	3
19	Bolivia	1999	Tomatoes	25
19	Bolivia	1999	Vegetables, Other	110
19	Bolivia	1999	Wheat	144
19	Bolivia	1999	Wine	1
19	Bolivia	2000	Apples	6
19	Bolivia	2000	Bananas	38
19	Bolivia	2000	Beans	1
19	Bolivia	2000	Beer	70
19	Bolivia	2000	Beverages, Alcoholic	6
19	Bolivia	2000	Bovine Meat	52
19	Bolivia	2000	Cassava	52
19	Bolivia	2000	Cereals, Other	8
19	Bolivia	2000	Cheese	2
19	Bolivia	2000	Cocoa Beans	2
19	Bolivia	2000	Coffee	8
19	Bolivia	2000	Cottonseed Oil	1
19	Bolivia	2000	Eggs	9
19	Bolivia	2000	Fats, Animals, Raw	5

FAO country code	Country	Reference year	Food item	Gross food supply (g *capita*$^{-1}$ d^{-1})
19	Bolivia	2000	Freshwater Fish	2
19	Bolivia	2000	Fruits, Other	32
19	Bolivia	2000	Grapefruit	9
19	Bolivia	2000	Grapes	9
19	Bolivia	2000	Groundnuts (Shelled Eq)	3
19	Bolivia	2000	Lemons, Limes	19
19	Bolivia	2000	Maize	102
19	Bolivia	2000	Marine Fish, Other	2
19	Bolivia	2000	Meat, Other	3
19	Bolivia	2000	Milk, Whole	82
19	Bolivia	2000	Mutton & Goat Meat	7
19	Bolivia	2000	Nuts	15
19	Bolivia	2000	Oats	2
19	Bolivia	2000	Offals, Edible	11
19	Bolivia	2000	Oilcrops Oil, Other	1
19	Bolivia	2000	Onions	14
19	Bolivia	2000	Oranges, Mandarines	49
19	Bolivia	2000	Peas	2
19	Bolivia	2000	Pelagic Fish	3
19	Bolivia	2000	Pigmeat	25
19	Bolivia	2000	Pineapples	17
19	Bolivia	2000	Plantains	89
19	Bolivia	2000	Potatoes	134
19	Bolivia	2000	Poultry Meat	44
19	Bolivia	2000	Pulses, Other	4
19	Bolivia	2000	Rice (Milled Equivalent)	59
19	Bolivia	2000	Roots, Other	16
19	Bolivia	2000	Soyabean Oil	3
19	Bolivia	2000	Sugar, Refined Equivalent	69
19	Bolivia	2000	Sunflowerseed Oil	4
19	Bolivia	2000	Sweet Potatoes	3
19	Bolivia	2000	Tea	1
19	Bolivia	2000	Tomatoes	26
19	Bolivia	2000	Vegetables, Other	110
19	Bolivia	2000	Wheat	149
19	Bolivia	2000	Wine	1
19	Bolivia	2001	Apples	9
19	Bolivia	2001	Bananas	36
19	Bolivia	2001	Beer	66
19	Bolivia	2001	Beverages, Alcoholic	6
19	Bolivia	2001	Beverages, Fermented	1
19	Bolivia	2001	Bovine Meat	51
19	Bolivia	2001	Cassava	47
19	Bolivia	2001	Cereals, Other	7
19	Bolivia	2001	Cheese	3
19	Bolivia	2001	Cocoa Beans	2
19	Bolivia	2001	Coffee	7
19	Bolivia	2001	Cottonseed Oil	2
19	Bolivia	2001	Eggs	9
19	Bolivia	2001	Fats, Animals, Raw	6
19	Bolivia	2001	Freshwater Fish	2
19	Bolivia	2001	Fruits, Other	32
19	Bolivia	2001	Grapefruit	9
19	Bolivia	2001	Grapes	8
19	Bolivia	2001	Groundnuts (Shelled Eq)	2
19	Bolivia	2001	Lemons, Limes	19
19	Bolivia	2001	Maize	100
19	Bolivia	2001	Marine Fish, Other	1
19	Bolivia	2001	Meat, Other	3
19	Bolivia	2001	Milk, Whole	58
19	Bolivia	2001	Mutton & Goat Meat	7
19	Bolivia	2001	Nuts	16
19	Bolivia	2001	Oats	2
19	Bolivia	2001	Offals, Edible	12
19	Bolivia	2001	Oilcrops Oil, Other	1
19	Bolivia	2001	Onions	14
19	Bolivia	2001	Oranges, Mandarines	48
19	Bolivia	2001	Peas	1
19	Bolivia	2001	Pelagic Fish	2
19	Bolivia	2001	Pigmeat	31
19	Bolivia	2001	Pineapples	17
19	Bolivia	2001	Plantains	92
19	Bolivia	2001	Potatoes	130
19	Bolivia	2001	Poultry Meat	41
19	Bolivia	2001	Pulses, Other	4
19	Bolivia	2001	Rice (Milled Equivalent)	62
19	Bolivia	2001	Roots, Other	14
19	Bolivia	2001	Soyabean Oil	4
19	Bolivia	2001	Sugar, Refined Equivalent	70
19	Bolivia	2001	Sunflowerseed Oil	4
19	Bolivia	2001	Sweet Potatoes	3
19	Bolivia	2001	Tea	1
19	Bolivia	2001	Tomatoes	27
19	Bolivia	2001	Vegetables, Other	106
19	Bolivia	2001	Wheat	154
19	Bolivia	2001	Wine	1
19	Bolivia	2002	Apples	7
19	Bolivia	2002	Bananas	36
19	Bolivia	2002	Beer	65
19	Bolivia	2002	Beverages, Alcoholic	6
19	Bolivia	2002	Bovine Meat	51
19	Bolivia	2002	Cassava	48
19	Bolivia	2002	Cereals, Other	6
19	Bolivia	2002	Cheese	2
19	Bolivia	2002	Cocoa Beans	2
19	Bolivia	2002	Coffee	7
19	Bolivia	2002	Cottonseed Oil	3

FAO country code	Country	Reference year	Food item	Gross food supply (g $capita^{-1}$ d^{-1})
19	Bolivia	2002	Eggs	9
19	Bolivia	2002	Fats, Animals, Raw	6
19	Bolivia	2002	Freshwater Fish	2
19	Bolivia	2002	Fruits, Other	29
19	Bolivia	2002	Grapefruit	9
19	Bolivia	2002	Grapes	8
19	Bolivia	2002	Groundnuts (Shelled Eq)	2
19	Bolivia	2002	Lemons, Limes	18
19	Bolivia	2002	Maize	100
19	Bolivia	2002	Marine Fish, Other	1
19	Bolivia	2002	Meat, Other	3
19	Bolivia	2002	Milk, Whole	88
19	Bolivia	2002	Mutton & Goat Meat	7
19	Bolivia	2002	Nuts	19
19	Bolivia	2002	Oats	2
19	Bolivia	2002	Offals, Edible	12
19	Bolivia	2002	Oilcrops Oil, Other	1
19	Bolivia	2002	Onions	14
19	Bolivia	2002	Oranges, Mandarines	45
19	Bolivia	2002	Peas	2
19	Bolivia	2002	Pelagic Fish	2
19	Bolivia	2002	Pigmeat	31
19	Bolivia	2002	Pineapples	16
19	Bolivia	2002	Plantains	93
19	Bolivia	2002	Potatoes	136
19	Bolivia	2002	Poultry Meat	42
19	Bolivia	2002	Pulses, Other	4
19	Bolivia	2002	Rice (Milled Equivalent)	60
19	Bolivia	2002	Roots, Other	14
19	Bolivia	2002	Soyabean Oil	4
19	Bolivia	2002	Sugar, Refined Equivalent	69
19	Bolivia	2002	Sunflowerseed Oil	3
19	Bolivia	2002	Sweet Potatoes	4
19	Bolivia	2002	Tomatoes	29
19	Bolivia	2002	Vegetables, Other	104
19	Bolivia	2002	Wheat	154
19	Bolivia	2002	Wine	1
19	Bolivia	2003	Apples	6
19	Bolivia	2003	Bananas	32
19	Bolivia	2003	Beer	49
19	Bolivia	2003	Beverages, Alcoholic	5
19	Bolivia	2003	Bovine Meat	51
19	Bolivia	2003	Cassava	45
19	Bolivia	2003	Cereals, Other	6
19	Bolivia	2003	Cheese	2
19	Bolivia	2003	Cocoa Beans	2
19	Bolivia	2003	Coffee	7
19	Bolivia	2003	Cottonseed Oil	3
19	Bolivia	2003	Eggs	11
19	Bolivia	2003	Fats, Animals, Raw	7
19	Bolivia	2003	Freshwater Fish	2
19	Bolivia	2003	Fruits, Other	21
19	Bolivia	2003	Grapefruit	8
19	Bolivia	2003	Grapes	7
19	Bolivia	2003	Groundnuts (Shelled Eq)	2
19	Bolivia	2003	Lemons, Limes	18
19	Bolivia	2003	Maize	101
19	Bolivia	2003	Marine Fish, Other	1
19	Bolivia	2003	Meat, Other	3
19	Bolivia	2003	Milk, Whole	82
19	Bolivia	2003	Mutton & Goat Meat	7
19	Bolivia	2003	Nuts	19
19	Bolivia	2003	Oats	2
19	Bolivia	2003	Offals, Edible	12
19	Bolivia	2003	Onions	9
19	Bolivia	2003	Oranges, Mandarines	32
19	Bolivia	2003	Peas	2
19	Bolivia	2003	Pelagic Fish	1
19	Bolivia	2003	Pigmeat	32
19	Bolivia	2003	Pineapples	16
19	Bolivia	2003	Plantains	94
19	Bolivia	2003	Potatoes	138
19	Bolivia	2003	Poultry Meat	41
19	Bolivia	2003	Pulses, Other	4
19	Bolivia	2003	Rice (Milled Equivalent)	65
19	Bolivia	2003	Roots, Other	12
19	Bolivia	2003	Soyabean Oil	3
19	Bolivia	2003	Sugar, Refined Equivalent	68
19	Bolivia	2003	Sunflowerseed Oil	3
19	Bolivia	2003	Sweet Potatoes	1
19	Bolivia	2003	Tomatoes	29
19	Bolivia	2003	Vegetables, Other	92
19	Bolivia	2003	Wheat	155
19	Bolivia	2003	Wine	2
19	Bolivia	2004	Apples	6
19	Bolivia	2004	Bananas	28
19	Bolivia	2004	Beer	60
19	Bolivia	2004	Beverages, Alcoholic	3
19	Bolivia	2004	Bovine Meat	51
19	Bolivia	2004	Cassava	47
19	Bolivia	2004	Cereals, Other	6
19	Bolivia	2004	Cheese	2
19	Bolivia	2004	Cocoa Beans	2
19	Bolivia	2004	Coffee	7
19	Bolivia	2004	Cottonseed Oil	3
19	Bolivia	2004	Eggs	12
19	Bolivia	2004	Fats, Animals, Raw	7

FAO countrycode	Country	Reference year	Food item	Gross food supply (g $capita^{-1}$ d^{-1})
19	Bolivia	2004	Freshwater Fish	2
19	Bolivia	2004	Fruits, Other	17
19	Bolivia	2004	Grapefruit	2
19	Bolivia	2004	Grapes	6
19	Bolivia	2004	Groundnuts (Shelled Eq)	2
19	Bolivia	2004	Lemons, Limes	7
19	Bolivia	2004	Maize	94
19	Bolivia	2004	Marine Fish, Other	2
19	Bolivia	2004	Meat, Other	3
19	Bolivia	2004	Milk, Whole	83
19	Bolivia	2004	Mutton & Goat Meat	7
19	Bolivia	2004	Nuts	22
19	Bolivia	2004	Oats	2
19	Bolivia	2004	Offals, Edible	12
19	Bolivia	2004	Onions	8
19	Bolivia	2004	Oranges, Mandarines	32
19	Bolivia	2004	Peas	2
19	Bolivia	2004	Pelagic Fish	1
19	Bolivia	2004	Pigmeat	32
19	Bolivia	2004	Pineapples	3
19	Bolivia	2004	Plantains	94
19	Bolivia	2004	Potatoes	137
19	Bolivia	2004	Poultry Meat	44
19	Bolivia	2004	Pulses, Other	4
19	Bolivia	2004	Rice (Milled Equivalent)	71
19	Bolivia	2004	Roots, Other	10
19	Bolivia	2004	Soyabean Oil	4
19	Bolivia	2004	Sugar, Refined Equivalent	68
19	Bolivia	2004	Sunflowerseed Oil	2
19	Bolivia	2004	Sweet Potatoes	1
19	Bolivia	2004	Tomatoes	30
19	Bolivia	2004	Vegetables, Other	90
19	Bolivia	2004	Wheat	135
19	Bolivia	2004	Wine	2
19	Bolivia	2005	Apples	5
19	Bolivia	2005	Bananas	26
19	Bolivia	2005	Beer	67
19	Bolivia	2005	Beverages, Alcoholic	3
19	Bolivia	2005	Bovine Meat	51
19	Bolivia	2005	Cassava	48
19	Bolivia	2005	Cereals, Other	5
19	Bolivia	2005	Cheese	2
19	Bolivia	2005	Cocoa Beans	3
19	Bolivia	2005	Coffee	7
19	Bolivia	2005	Cottonseed Oil	3
19	Bolivia	2005	Eggs	12
19	Bolivia	2005	Fats, Animals, Raw	8
19	Bolivia	2005	Freshwater Fish	2
19	Bolivia	2005	Fruits, Other	15
19	Bolivia	2005	Grapefruit	2
19	Bolivia	2005	Grapes	6
19	Bolivia	2005	Groundnuts (Shelled Eq)	2
19	Bolivia	2005	Lemons, Limes	7
19	Bolivia	2005	Maize	107
19	Bolivia	2005	Marine Fish, Other	2
19	Bolivia	2005	Meat, Other	2
19	Bolivia	2005	Milk, Whole	87
19	Bolivia	2005	Mutton & Goat Meat	7
19	Bolivia	2005	Nuts	22
19	Bolivia	2005	Oats	3
19	Bolivia	2005	Offals, Edible	12
19	Bolivia	2005	Onions	9
19	Bolivia	2005	Oranges, Mandarines	32
19	Bolivia	2005	Peas	2
19	Bolivia	2005	Pelagic Fish	1
19	Bolivia	2005	Pigmeat	32
19	Bolivia	2005	Pineapples	3
19	Bolivia	2005	Plantains	95
19	Bolivia	2005	Potatoes	138
19	Bolivia	2005	Poultry Meat	54
19	Bolivia	2005	Pulses, Other	4
19	Bolivia	2005	Rice (Milled Equivalent)	75
19	Bolivia	2005	Roots, Other	10
19	Bolivia	2005	Soyabean Oil	4
19	Bolivia	2005	Sugar, Refined Equivalent	68
19	Bolivia	2005	Sunflowerseed Oil	1
19	Bolivia	2005	Sweet Potatoes	1
19	Bolivia	2005	Tomatoes	31
19	Bolivia	2005	Vegetables, Other	91
19	Bolivia	2005	Wheat	133
19	Bolivia	2005	Wine	2
19	Bolivia	2006	Apples	5
19	Bolivia	2006	Bananas	17
19	Bolivia	2006	Beer	72
19	Bolivia	2006	Beverages, Alcoholic	3
19	Bolivia	2006	Bovine Meat	57
19	Bolivia	2006	Cassava	48
19	Bolivia	2006	Cereals, Other	5
19	Bolivia	2006	Cheese	2
19	Bolivia	2006	Cocoa Beans	3
19	Bolivia	2006	Coconuts - Incl Copra	1
19	Bolivia	2006	Coffee	7
19	Bolivia	2006	Cottonseed Oil	3
19	Bolivia	2006	Eggs	12
19	Bolivia	2006	Fats, Animals, Raw	9
19	Bolivia	2006	Freshwater Fish	2
19	Bolivia	2006	Fruits, Other	14

FAO country code	Country	Reference year	Food item	Gross food supply (g *capita*$^{-1}$ d^{-1})
19	Bolivia	2006	Grapefruit	2
19	Bolivia	2006	Grapes	6
19	Bolivia	2006	Groundnuts (Shelled Eq)	2
19	Bolivia	2006	Lemons, Limes	7
19	Bolivia	2006	Maize	113
19	Bolivia	2006	Marine Fish, Other	2
19	Bolivia	2006	Meat, Other	2
19	Bolivia	2006	Milk, Whole	64
19	Bolivia	2006	Mutton & Goat Meat	7
19	Bolivia	2006	Nuts	18
19	Bolivia	2006	Oats	3
19	Bolivia	2006	Offals, Edible	13
19	Bolivia	2006	Onions	9
19	Bolivia	2006	Oranges, Mandarines	32
19	Bolivia	2006	Peas	1
19	Bolivia	2006	Pelagic Fish	1
19	Bolivia	2006	Pigmeat	32
19	Bolivia	2006	Pineapples	2
19	Bolivia	2006	Plantains	93
19	Bolivia	2006	Potatoes	133
19	Bolivia	2006	Poultry Meat	72
19	Bolivia	2006	Pulses, Other	4
19	Bolivia	2006	Rice (Milled Equivalent)	80
19	Bolivia	2006	Roots, Other	8
19	Bolivia	2006	Soyabean Oil	4
19	Bolivia	2006	Sugar, Refined Equivalent	65
19	Bolivia	2006	Sunflowerseed Oil	1
19	Bolivia	2006	Sweet Potatoes	1
19	Bolivia	2006	Sweeteners, Other	1
19	Bolivia	2006	Tea	1
19	Bolivia	2006	Tomatoes	30
19	Bolivia	2006	Vegetables, Other	59
19	Bolivia	2006	Wheat	132
19	Bolivia	2006	Wine	2
19	Bolivia	2007	Apples	4
19	Bolivia	2007	Bananas	18
19	Bolivia	2007	Beer	77
19	Bolivia	2007	Beverages, Alcoholic	3
19	Bolivia	2007	Bovine Meat	69
19	Bolivia	2007	Cassava	44
19	Bolivia	2007	Cereals, Other	4
19	Bolivia	2007	Cheese	2
19	Bolivia	2007	Cocoa Beans	3
19	Bolivia	2007	Coffee	7
19	Bolivia	2007	Cottonseed Oil	3
19	Bolivia	2007	Eggs	12
19	Bolivia	2007	Fats, Animals, Raw	8
19	Bolivia	2007	Freshwater Fish	2
19	Bolivia	2007	Fruits, Other	13
19	Bolivia	2007	Grapefruit	2
19	Bolivia	2007	Grapes	6
19	Bolivia	2007	Groundnuts (Shelled Eq)	2
19	Bolivia	2007	Lemons, Limes	7
19	Bolivia	2007	Maize	106
19	Bolivia	2007	Marine Fish, Other	2
19	Bolivia	2007	Meat, Other	2
19	Bolivia	2007	Milk, Whole	63
19	Bolivia	2007	Mutton & Goat Meat	7
19	Bolivia	2007	Nuts	15
19	Bolivia	2007	Oats	3
19	Bolivia	2007	Offals, Edible	14
19	Bolivia	2007	Onions	8
19	Bolivia	2007	Oranges, Mandarines	33
19	Bolivia	2007	Peas	2
19	Bolivia	2007	Pelagic Fish	1
19	Bolivia	2007	Pigmeat	32
19	Bolivia	2007	Pineapples	3
19	Bolivia	2007	Plantains	92
19	Bolivia	2007	Potatoes	130
19	Bolivia	2007	Poultry Meat	81
19	Bolivia	2007	Pulses, Other	4
19	Bolivia	2007	Rice (Milled Equivalent)	67
19	Bolivia	2007	Roots, Other	10
19	Bolivia	2007	Soyabean Oil	4
19	Bolivia	2007	Sugar, Refined Equivalent	64
19	Bolivia	2007	Sunflowerseed Oil	1
19	Bolivia	2007	Sweet Potatoes	1
19	Bolivia	2007	Sweeteners, Other	1
19	Bolivia	2007	Tomatoes	29
19	Bolivia	2007	Vegetables, Other	61
19	Bolivia	2007	Wheat	143
19	Bolivia	2007	Wine	2
19	Bolivia	2008	Apples	4
19	Bolivia	2008	Bananas	16
19	Bolivia	2008	Beer	84
19	Bolivia	2008	Beverages, Alcoholic	3
19	Bolivia	2008	Bovine Meat	69
19	Bolivia	2008	Cassava	44
19	Bolivia	2008	Cereals, Other	4
19	Bolivia	2008	Cheese	2
19	Bolivia	2008	Cocoa Beans	3
19	Bolivia	2008	Coffee	7
19	Bolivia	2008	Cottonseed Oil	3
19	Bolivia	2008	Eggs	12
19	Bolivia	2008	Fats, Animals, Raw	8
19	Bolivia	2008	Freshwater Fish	2
19	Bolivia	2008	Fruits, Other	14

FAO country code	Country	Reference year	Food item	Gross food supply (g *capita*$^{-1}$ d^{-1})
19	Bolivia	2008	Grapefruit	2
19	Bolivia	2008	Grapes	5
19	Bolivia	2008	Groundnuts (Shelled Eq)	2
19	Bolivia	2008	Lemons, Limes	7
19	Bolivia	2008	Maize	99
19	Bolivia	2008	Marine Fish, Other	3
19	Bolivia	2008	Meat, Other	2
19	Bolivia	2008	Milk, Whole	66
19	Bolivia	2008	Mutton & Goat Meat	7
19	Bolivia	2008	Nuts	19
19	Bolivia	2008	Oats	3
19	Bolivia	2008	Offals, Edible	15
19	Bolivia	2008	Oilcrops Oil, Other	1
19	Bolivia	2008	Onions	8
19	Bolivia	2008	Oranges, Mandarines	33
19	Bolivia	2008	Peas	1
19	Bolivia	2008	Pelagic Fish	2
19	Bolivia	2008	Pigmeat	32
19	Bolivia	2008	Pineapples	3
19	Bolivia	2008	Plantains	92
19	Bolivia	2008	Potatoes	131
19	Bolivia	2008	Poultry Meat	85
19	Bolivia	2008	Pulses, Other	3
19	Bolivia	2008	Rice (Milled Equivalent)	67
19	Bolivia	2008	Roots, Other	10
19	Bolivia	2008	Soyabean Oil	4
19	Bolivia	2008	Sugar, Refined Equivalent	64
19	Bolivia	2008	Sunflowerseed Oil	2
19	Bolivia	2008	Sweet Potatoes	1
19	Bolivia	2008	Sweeteners, Other	1
19	Bolivia	2008	Tea	1
19	Bolivia	2008	Tomatoes	29
19	Bolivia	2008	Vegetables, Other	59
19	Bolivia	2008	Wheat	144
19	Bolivia	2008	Wine	2
19	Bolivia	2009	Apples	6
19	Bolivia	2009	Bananas	8
19	Bolivia	2009	Beer	83
19	Bolivia	2009	Beverages, Alcoholic	1
19	Bolivia	2009	Bovine Meat	56
19	Bolivia	2009	Cassava	42
19	Bolivia	2009	Cereals, Other	6
19	Bolivia	2009	Cheese	2
19	Bolivia	2009	Cocoa Beans	3
19	Bolivia	2009	Coconuts - Incl Copra	1
19	Bolivia	2009	Coffee	7
19	Bolivia	2009	Cottonseed Oil	3
19	Bolivia	2009	Eggs	13
19	Bolivia	2009	Fats, Animals, Raw	7
19	Bolivia	2009	Freshwater Fish	2
19	Bolivia	2009	Fruits, Other	19
19	Bolivia	2009	Grapefruit	2
19	Bolivia	2009	Grapes	4
19	Bolivia	2009	Groundnuts (Shelled Eq)	1
19	Bolivia	2009	Lemons, Limes	5
19	Bolivia	2009	Maize	108
19	Bolivia	2009	Marine Fish, Other	2
19	Bolivia	2009	Meat, Other	2
19	Bolivia	2009	Milk, Whole	86
19	Bolivia	2009	Mutton & Goat Meat	5
19	Bolivia	2009	Nuts	18
19	Bolivia	2009	Oats	2
19	Bolivia	2009	Offals, Edible	13
19	Bolivia	2009	Oilcrops Oil, Other	1
19	Bolivia	2009	Onions	20
19	Bolivia	2009	Oranges, Mandarines	71
19	Bolivia	2009	Peas	2
19	Bolivia	2009	Pelagic Fish	1
19	Bolivia	2009	Pigmeat	22
19	Bolivia	2009	Pineapples	11
19	Bolivia	2009	Plantains	66
19	Bolivia	2009	Potatoes	164
19	Bolivia	2009	Poultry Meat	89
19	Bolivia	2009	Pulses, Other	4
19	Bolivia	2009	Rice (Milled Equivalent)	69
19	Bolivia	2009	Roots, Other	10
19	Bolivia	2009	Soyabean Oil	3
19	Bolivia	2009	Sugar, Refined Equivalent	63
19	Bolivia	2009	Sunflowerseed Oil	2
19	Bolivia	2009	Sweet Potatoes	2
19	Bolivia	2009	Sweeteners, Other	1
19	Bolivia	2009	Tea	1
19	Bolivia	2009	Tomatoes	13
19	Bolivia	2009	Vegetables, Other	58
19	Bolivia	2009	Wheat	152
19	Bolivia	2009	Wine	2
19	Bolivia	2010	Apples	6
19	Bolivia	2010	Bananas	12
19	Bolivia	2010	Beans	5
19	Bolivia	2010	Beer	83
19	Bolivia	2010	Beverages, Alcoholic	1
19	Bolivia	2010	Bovine Meat	55
19	Bolivia	2010	Cassava	45
19	Bolivia	2010	Cereals, Other	5
19	Bolivia	2010	Cheese	3
19	Bolivia	2010	Cocoa Beans	3
19	Bolivia	2010	Coffee	7

FAO country code	Country	Reference year	Food item	Gross food supply (g *capita*$^{-1}$ d^{-1})
19	Bolivia	2010	Cottonseed Oil	3
19	Bolivia	2010	Eggs	14
19	Bolivia	2010	Fats, Animals, Raw	6
19	Bolivia	2010	Freshwater Fish	2
19	Bolivia	2010	Fruits, Other	19
19	Bolivia	2010	Grapefruit	2
19	Bolivia	2010	Grapes	4
19	Bolivia	2010	Groundnuts (Shelled Eq)	1
19	Bolivia	2010	Lemons, Limes	5
19	Bolivia	2010	Maize	104
19	Bolivia	2010	Marine Fish, Other	2
19	Bolivia	2010	Meat, Other	2
19	Bolivia	2010	Milk, Whole	91
19	Bolivia	2010	Mutton & Goat Meat	5
19	Bolivia	2010	Nuts	18
19	Bolivia	2010	Oats	2
19	Bolivia	2010	Offals, Edible	10
19	Bolivia	2010	Onions	18
19	Bolivia	2010	Oranges, Mandarines	69
19	Bolivia	2010	Peas	2
19	Bolivia	2010	Pelagic Fish	1
19	Bolivia	2010	Pigmeat	23
19	Bolivia	2010	Pineapples	11
19	Bolivia	2010	Plantains	68
19	Bolivia	2010	Potatoes	173
19	Bolivia	2010	Poultry Meat	103
19	Bolivia	2010	Pulses, Other	3
19	Bolivia	2010	Rice (Milled Equivalent)	70
19	Bolivia	2010	Roots, Other	10
19	Bolivia	2010	Soyabean Oil	4
19	Bolivia	2010	Sugar, Refined Equivalent	63
19	Bolivia	2010	Sunflowerseed Oil	1
19	Bolivia	2010	Sweet Potatoes	1
19	Bolivia	2010	Sweeteners, Other	1
19	Bolivia	2010	Tea	1
19	Bolivia	2010	Tomatoes	15
19	Bolivia	2010	Vegetables, Other	55
19	Bolivia	2010	Wheat	148
19	Bolivia	2010	Wine	2
19	Bolivia	2011	Apples	6
19	Bolivia	2011	Bananas	5
19	Bolivia	2011	Beans	9
19	Bolivia	2011	Beer	74
19	Bolivia	2011	Beverages, Alcoholic	2
19	Bolivia	2011	Bovine Meat	54
19	Bolivia	2011	Cassava	47
19	Bolivia	2011	Cereals, Other	4
19	Bolivia	2011	Cheese	3
19	Bolivia	2011	Cocoa Beans	3
19	Bolivia	2011	Coffee	6
19	Bolivia	2011	Cottonseed Oil	3
19	Bolivia	2011	Eggs	15
19	Bolivia	2011	Fats, Animals, Raw	6
19	Bolivia	2011	Freshwater Fish	2
19	Bolivia	2011	Fruits, Other	19
19	Bolivia	2011	Grapefruit	2
19	Bolivia	2011	Grapes	4
19	Bolivia	2011	Groundnuts (Shelled Eq)	1
19	Bolivia	2011	Lemons, Limes	5
19	Bolivia	2011	Maize	105
19	Bolivia	2011	Marine Fish, Other	2
19	Bolivia	2011	Meat, Other	2
19	Bolivia	2011	Milk, Whole	107
19	Bolivia	2011	Mutton & Goat Meat	5
19	Bolivia	2011	Nuts	18
19	Bolivia	2011	Oats	3
19	Bolivia	2011	Offals, Edible	10
19	Bolivia	2011	Oilcrops Oil, Other	1
19	Bolivia	2011	Onions	18
19	Bolivia	2011	Oranges, Mandarines	68
19	Bolivia	2011	Peas	1
19	Bolivia	2011	Pelagic Fish	1
19	Bolivia	2011	Pigmeat	23
19	Bolivia	2011	Pineapples	11
19	Bolivia	2011	Plantains	69
19	Bolivia	2011	Potatoes	181
19	Bolivia	2011	Poultry Meat	100
19	Bolivia	2011	Pulses, Other	3
19	Bolivia	2011	Rice (Milled Equivalent)	72
19	Bolivia	2011	Roots, Other	9
19	Bolivia	2011	Soyabean Oil	8
19	Bolivia	2011	Sugar, Refined Equivalent	65
19	Bolivia	2011	Sunflowerseed Oil	3
19	Bolivia	2011	Sweet Potatoes	1
19	Bolivia	2011	Sweeteners, Other	2
19	Bolivia	2011	Tea	1
19	Bolivia	2011	Tomatoes	15
19	Bolivia	2011	Vegetables, Other	55
19	Bolivia	2011	Wheat	148
19	Bolivia	2011	Wine	2
80	Bosnia and Herzegovina	1992	Apples	4
80	Bosnia and Herzegovina	1992	Beans	10
80	Bosnia and Herzegovina	1992	Beer	35
80	Bosnia and Herzegovina	1992	Beverages, Alcoholic	43
80	Bosnia and Herzegovina	1992	Bovine Meat	19
80	Bosnia and Herzegovina	1992	Cheese	13
80	Bosnia and Herzegovina	1992	Coffee	2

FAO country code	Country	Reference year	Food item	Gross food supply (g *capita*$^{-1}$ d^{-1})
80	Bosnia and Herzegovina	1992	Eggs	11
80	Bosnia and Herzegovina	1992	Fats, Animals, Raw	1
80	Bosnia and Herzegovina	1992	Freshwater Fish	1
80	Bosnia and Herzegovina	1992	Fruits, Other	50
80	Bosnia and Herzegovina	1992	Grapes	5
80	Bosnia and Herzegovina	1992	Lemons, Limes	1
80	Bosnia and Herzegovina	1992	Maize	263
80	Bosnia and Herzegovina	1992	Milk, Whole	174
80	Bosnia and Herzegovina	1992	Mutton & Goat Meat	1
80	Bosnia and Herzegovina	1992	Nuts	1
80	Bosnia and Herzegovina	1992	Offals, Edible	3
80	Bosnia and Herzegovina	1992	Onions	13
80	Bosnia and Herzegovina	1992	Oranges, Mandarines	2
80	Bosnia and Herzegovina	1992	Peas	4
80	Bosnia and Herzegovina	1992	Pigmeat	11
80	Bosnia and Herzegovina	1992	Pimento	26
80	Bosnia and Herzegovina	1992	Potatoes	152
80	Bosnia and Herzegovina	1992	Poultry Meat	10
80	Bosnia and Herzegovina	1992	Pulses, Other	1
80	Bosnia and Herzegovina	1992	Rice (Milled Equivalent)	1
80	Bosnia and Herzegovina	1992	Rye	4
80	Bosnia and Herzegovina	1992	Soyabean Oil	1
80	Bosnia and Herzegovina	1992	Sugar, Refined Equivalent	25
80	Bosnia and Herzegovina	1992	Sunflowerseed Oil	2
80	Bosnia and Herzegovina	1992	Sweeteners, Other	1
80	Bosnia and Herzegovina	1992	Tomatoes	11
80	Bosnia and Herzegovina	1992	Vegetables, Other	325
80	Bosnia and Herzegovina	1992	Wheat	224
80	Bosnia and Herzegovina	1992	Wine	14
80	Bosnia and Herzegovina	1993	Apples	13
80	Bosnia and Herzegovina	1993	Beans	13
80	Bosnia and Herzegovina	1993	Beer	37
80	Bosnia and Herzegovina	1993	Beverages, Alcoholic	37
80	Bosnia and Herzegovina	1993	Bovine Meat	21
80	Bosnia and Herzegovina	1993	Cheese	13
80	Bosnia and Herzegovina	1993	Coffee	2
80	Bosnia and Herzegovina	1993	Eggs	10
80	Bosnia and Herzegovina	1993	Fats, Animals, Raw	2
80	Bosnia and Herzegovina	1993	Freshwater Fish	1
80	Bosnia and Herzegovina	1993	Fruits, Other	40
80	Bosnia and Herzegovina	1993	Grapes	4
80	Bosnia and Herzegovina	1993	Lemons, Limes	2
80	Bosnia and Herzegovina	1993	Maize	255
80	Bosnia and Herzegovina	1993	Marine Fish, Other	2
80	Bosnia and Herzegovina	1993	Milk, Whole	190
80	Bosnia and Herzegovina	1993	Mutton & Goat Meat	1
80	Bosnia and Herzegovina	1993	Nuts	2
80	Bosnia and Herzegovina	1993	Offals, Edible	3
80	Bosnia and Herzegovina	1993	Onions	16
80	Bosnia and Herzegovina	1993	Oranges, Mandarines	10
80	Bosnia and Herzegovina	1993	Peas	7
80	Bosnia and Herzegovina	1993	Pigmeat	9
80	Bosnia and Herzegovina	1993	Pimento	27
80	Bosnia and Herzegovina	1993	Potatoes	167
80	Bosnia and Herzegovina	1993	Poultry Meat	10
80	Bosnia and Herzegovina	1993	Pulses, Other	2
80	Bosnia and Herzegovina	1993	Rice (Milled Equivalent)	1
80	Bosnia and Herzegovina	1993	Rye	4
80	Bosnia and Herzegovina	1993	Soyabean Oil	5
80	Bosnia and Herzegovina	1993	Sugar, Refined Equivalent	22
80	Bosnia and Herzegovina	1993	Sunflowerseed Oil	1
80	Bosnia and Herzegovina	1993	Sweeteners, Other	2
80	Bosnia and Herzegovina	1993	Tomatoes	14
80	Bosnia and Herzegovina	1993	Vegetables, Other	339
80	Bosnia and Herzegovina	1993	Wheat	252
80	Bosnia and Herzegovina	1993	Wine	8
80	Bosnia and Herzegovina	1994	Apples	16
80	Bosnia and Herzegovina	1994	Bananas	1
80	Bosnia and Herzegovina	1994	Beans	16
80	Bosnia and Herzegovina	1994	Beer	45
80	Bosnia and Herzegovina	1994	Beverages, Alcoholic	34
80	Bosnia and Herzegovina	1994	Bovine Meat	22
80	Bosnia and Herzegovina	1994	Cheese	12
80	Bosnia and Herzegovina	1994	Citrus, Other	1
80	Bosnia and Herzegovina	1994	Cocoa Beans	1
80	Bosnia and Herzegovina	1994	Coffee	1
80	Bosnia and Herzegovina	1994	Eggs	12
80	Bosnia and Herzegovina	1994	Fats, Animals, Raw	2
80	Bosnia and Herzegovina	1994	Freshwater Fish	1
80	Bosnia and Herzegovina	1994	Fruits, Other	37
80	Bosnia and Herzegovina	1994	Grapes	5
80	Bosnia and Herzegovina	1994	Honey	1
80	Bosnia and Herzegovina	1994	Maize	234
80	Bosnia and Herzegovina	1994	Milk, Whole	181
80	Bosnia and Herzegovina	1994	Mutton & Goat Meat	1
80	Bosnia and Herzegovina	1994	Nuts	3
80	Bosnia and Herzegovina	1994	Offals, Edible	4
80	Bosnia and Herzegovina	1994	Oilcrops Oil, Other	1
80	Bosnia and Herzegovina	1994	Onions	17
80	Bosnia and Herzegovina	1994	Oranges, Mandarines	6
80	Bosnia and Herzegovina	1994	Peas	5
80	Bosnia and Herzegovina	1994	Pigmeat	12
80	Bosnia and Herzegovina	1994	Pimento	25
80	Bosnia and Herzegovina	1994	Potatoes	143
80	Bosnia and Herzegovina	1994	Poultry Meat	14
80	Bosnia and Herzegovina	1994	Pulses, Other	2
80	Bosnia and Herzegovina	1994	Rice (Milled Equivalent)	4

FAO country code	Country	Reference year	Food item	Gross food supply (g $capita^{-1}$ d^{-1})
80	Bosnia and Herzegovina	1994	Rye	4
80	Bosnia and Herzegovina	1994	Soyabean Oil	3
80	Bosnia and Herzegovina	1994	Sugar, Refined Equivalent	23
80	Bosnia and Herzegovina	1994	Sunflowerseed Oil	2
80	Bosnia and Herzegovina	1994	Sweeteners, Other	4
80	Bosnia and Herzegovina	1994	Tomatoes	15
80	Bosnia and Herzegovina	1994	Vegetables, Other	357
80	Bosnia and Herzegovina	1994	Wheat	339
80	Bosnia and Herzegovina	1994	Wine	7
80	Bosnia and Herzegovina	1995	Apples	19
80	Bosnia and Herzegovina	1995	Bananas	1
80	Bosnia and Herzegovina	1995	Beans	11
80	Bosnia and Herzegovina	1995	Beer	46
80	Bosnia and Herzegovina	1995	Beverages, Alcoholic	34
80	Bosnia and Herzegovina	1995	Bovine Meat	20
80	Bosnia and Herzegovina	1995	Cheese	13
80	Bosnia and Herzegovina	1995	Citrus, Other	1
80	Bosnia and Herzegovina	1995	Cocoa Beans	2
80	Bosnia and Herzegovina	1995	Coffee	1
80	Bosnia and Herzegovina	1995	Cream	1
80	Bosnia and Herzegovina	1995	Eggs	14
80	Bosnia and Herzegovina	1995	Fats, Animals, Raw	2
80	Bosnia and Herzegovina	1995	Freshwater Fish	1
80	Bosnia and Herzegovina	1995	Fruits, Other	42
80	Bosnia and Herzegovina	1995	Grapes	3
80	Bosnia and Herzegovina	1995	Lemons, Limes	1
80	Bosnia and Herzegovina	1995	Maize	210
80	Bosnia and Herzegovina	1995	Milk, Whole	177
80	Bosnia and Herzegovina	1995	Mutton & Goat Meat	1
80	Bosnia and Herzegovina	1995	Nuts	2
80	Bosnia and Herzegovina	1995	Offals, Edible	3
80	Bosnia and Herzegovina	1995	Oilcrops Oil, Other	1
80	Bosnia and Herzegovina	1995	Onions	16
80	Bosnia and Herzegovina	1995	Oranges, Mandarines	11
80	Bosnia and Herzegovina	1995	Peas	3
80	Bosnia and Herzegovina	1995	Pelagic Fish	2
80	Bosnia and Herzegovina	1995	Pigmeat	15
80	Bosnia and Herzegovina	1995	Pimento	30
80	Bosnia and Herzegovina	1995	Potatoes	216
80	Bosnia and Herzegovina	1995	Poultry Meat	19
80	Bosnia and Herzegovina	1995	Pulses, Other	2
80	Bosnia and Herzegovina	1995	Rice (Milled Equivalent)	1
80	Bosnia and Herzegovina	1995	Rye	4
80	Bosnia and Herzegovina	1995	Soyabean Oil	1
80	Bosnia and Herzegovina	1995	Sugar, Refined Equivalent	21
80	Bosnia and Herzegovina	1995	Sunflowerseed Oil	3
80	Bosnia and Herzegovina	1995	Sweeteners, Other	6
80	Bosnia and Herzegovina	1995	Tomatoes	16
80	Bosnia and Herzegovina	1995	Vegetables, Other	400
80	Bosnia and Herzegovina	1995	Wheat	340
80	Bosnia and Herzegovina	1995	Wine	14
80	Bosnia and Herzegovina	1996	Apples	21
80	Bosnia and Herzegovina	1996	Beans	14
80	Bosnia and Herzegovina	1996	Beer	62
80	Bosnia and Herzegovina	1996	Beverages, Alcoholic	43
80	Bosnia and Herzegovina	1996	Bovine Meat	23
80	Bosnia and Herzegovina	1996	Butter, Ghee	1
80	Bosnia and Herzegovina	1996	Cheese	15
80	Bosnia and Herzegovina	1996	Citrus, Other	3
80	Bosnia and Herzegovina	1996	Cocoa Beans	3
80	Bosnia and Herzegovina	1996	Coffee	1
80	Bosnia and Herzegovina	1996	Cream	1
80	Bosnia and Herzegovina	1996	Eggs	21
80	Bosnia and Herzegovina	1996	Fats, Animals, Raw	1
80	Bosnia and Herzegovina	1996	Freshwater Fish	2
80	Bosnia and Herzegovina	1996	Fruits, Other	47
80	Bosnia and Herzegovina	1996	Grapes	2
80	Bosnia and Herzegovina	1996	Honey	1
80	Bosnia and Herzegovina	1996	Lemons, Limes	1
80	Bosnia and Herzegovina	1996	Maize	237
80	Bosnia and Herzegovina	1996	Milk, Whole	137
80	Bosnia and Herzegovina	1996	Mutton & Goat Meat	1
80	Bosnia and Herzegovina	1996	Nuts	3
80	Bosnia and Herzegovina	1996	Offals, Edible	2
80	Bosnia and Herzegovina	1996	Onions	27
80	Bosnia and Herzegovina	1996	Oranges, Mandarines	13
80	Bosnia and Herzegovina	1996	Peas	3
80	Bosnia and Herzegovina	1996	Pigmeat	23
80	Bosnia and Herzegovina	1996	Pimento	29
80	Bosnia and Herzegovina	1996	Potatoes	202
80	Bosnia and Herzegovina	1996	Poultry Meat	30
80	Bosnia and Herzegovina	1996	Pulses, Other	1
80	Bosnia and Herzegovina	1996	Rice (Milled Equivalent)	1
80	Bosnia and Herzegovina	1996	Rye	2
80	Bosnia and Herzegovina	1996	Soyabean Oil	5
80	Bosnia and Herzegovina	1996	Sugar, Refined Equivalent	22
80	Bosnia and Herzegovina	1996	Sunflowerseed Oil	11
80	Bosnia and Herzegovina	1996	Sweeteners, Other	8
80	Bosnia and Herzegovina	1996	Tomatoes	26
80	Bosnia and Herzegovina	1996	Vegetables, Other	375
80	Bosnia and Herzegovina	1996	Wheat	334
80	Bosnia and Herzegovina	1996	Wine	3
80	Bosnia and Herzegovina	1997	Apples	25
80	Bosnia and Herzegovina	1997	Bananas	1
80	Bosnia and Herzegovina	1997	Barley	8
80	Bosnia and Herzegovina	1997	Beans	18
80	Bosnia and Herzegovina	1997	Beer	75

FAO country code	Country	Reference year	Food item	Gross food supply (g *capita*$^{-1}$ d^{-1})
80	Bosnia and Herzegovina	1997	Beverages, Alcoholic	43
80	Bosnia and Herzegovina	1997	Bovine Meat	22
80	Bosnia and Herzegovina	1997	Butter, Ghee	1
80	Bosnia and Herzegovina	1997	Cheese	15
80	Bosnia and Herzegovina	1997	Citrus, Other	1
80	Bosnia and Herzegovina	1997	Cocoa Beans	4
80	Bosnia and Herzegovina	1997	Coffee	1
80	Bosnia and Herzegovina	1997	Eggs	14
80	Bosnia and Herzegovina	1997	Fats, Animals, Raw	2
80	Bosnia and Herzegovina	1997	Freshwater Fish	2
80	Bosnia and Herzegovina	1997	Fruits, Other	60
80	Bosnia and Herzegovina	1997	Grapes	4
80	Bosnia and Herzegovina	1997	Honey	1
80	Bosnia and Herzegovina	1997	Lemons, Limes	2
80	Bosnia and Herzegovina	1997	Maize	266
80	Bosnia and Herzegovina	1997	Milk, Whole	229
80	Bosnia and Herzegovina	1997	Mutton & Goat Meat	1
80	Bosnia and Herzegovina	1997	Nuts	3
80	Bosnia and Herzegovina	1997	Offals, Edible	2
80	Bosnia and Herzegovina	1997	Oilcrops Oil, Other	1
80	Bosnia and Herzegovina	1997	Onions	32
80	Bosnia and Herzegovina	1997	Oranges, Mandarines	14
80	Bosnia and Herzegovina	1997	Peas	2
80	Bosnia and Herzegovina	1997	Pelagic Fish	1
80	Bosnia and Herzegovina	1997	Pigmeat	20
80	Bosnia and Herzegovina	1997	Pimento	36
80	Bosnia and Herzegovina	1997	Potatoes	195
80	Bosnia and Herzegovina	1997	Poultry Meat	26
80	Bosnia and Herzegovina	1997	Pulses, Other	1
80	Bosnia and Herzegovina	1997	Rice (Milled Equivalent)	2
80	Bosnia and Herzegovina	1997	Rye	4
80	Bosnia and Herzegovina	1997	Soyabean Oil	4
80	Bosnia and Herzegovina	1997	Sugar, Refined Equivalent	24
80	Bosnia and Herzegovina	1997	Sunflowerseed Oil	9
80	Bosnia and Herzegovina	1997	Sweeteners, Other	16
80	Bosnia and Herzegovina	1997	Tomatoes	35
80	Bosnia and Herzegovina	1997	Vegetables, Other	421
80	Bosnia and Herzegovina	1997	Wheat	337
80	Bosnia and Herzegovina	1997	Wine	4
80	Bosnia and Herzegovina	1998	Apples	17
80	Bosnia and Herzegovina	1998	Bananas	1
80	Bosnia and Herzegovina	1998	Barley	9
80	Bosnia and Herzegovina	1998	Beans	11
80	Bosnia and Herzegovina	1998	Beer	75
80	Bosnia and Herzegovina	1998	Beverages, Alcoholic	35
80	Bosnia and Herzegovina	1998	Bovine Meat	20
80	Bosnia and Herzegovina	1998	Butter, Ghee	1
80	Bosnia and Herzegovina	1998	Cheese	15
80	Bosnia and Herzegovina	1998	Cocoa Beans	2
80	Bosnia and Herzegovina	1998	Eggs	15
80	Bosnia and Herzegovina	1998	Fats, Animals, Raw	1
80	Bosnia and Herzegovina	1998	Freshwater Fish	2
80	Bosnia and Herzegovina	1998	Fruits, Other	53
80	Bosnia and Herzegovina	1998	Grapes	3
80	Bosnia and Herzegovina	1998	Honey	1
80	Bosnia and Herzegovina	1998	Lemons, Limes	3
80	Bosnia and Herzegovina	1998	Maize	259
80	Bosnia and Herzegovina	1998	Milk, Whole	285
80	Bosnia and Herzegovina	1998	Mutton & Goat Meat	1
80	Bosnia and Herzegovina	1998	Nuts	3
80	Bosnia and Herzegovina	1998	Offals, Edible	3
80	Bosnia and Herzegovina	1998	Onions	32
80	Bosnia and Herzegovina	1998	Oranges, Mandarines	13
80	Bosnia and Herzegovina	1998	Peas	4
80	Bosnia and Herzegovina	1998	Pelagic Fish	1
80	Bosnia and Herzegovina	1998	Pigmeat	21
80	Bosnia and Herzegovina	1998	Pimento	27
80	Bosnia and Herzegovina	1998	Potatoes	236
80	Bosnia and Herzegovina	1998	Poultry Meat	20
80	Bosnia and Herzegovina	1998	Pulses, Other	1
80	Bosnia and Herzegovina	1998	Rice (Milled Equivalent)	3
80	Bosnia and Herzegovina	1998	Rye	5
80	Bosnia and Herzegovina	1998	Soyabean Oil	3
80	Bosnia and Herzegovina	1998	Sugar, Refined Equivalent	27
80	Bosnia and Herzegovina	1998	Sunflowerseed Oil	10
80	Bosnia and Herzegovina	1998	Sweeteners, Other	9
80	Bosnia and Herzegovina	1998	Tomatoes	37
80	Bosnia and Herzegovina	1998	Vegetables, Other	445
80	Bosnia and Herzegovina	1998	Wheat	329
80	Bosnia and Herzegovina	1998	Wine	5
80	Bosnia and Herzegovina	1999	Apples	15
80	Bosnia and Herzegovina	1999	Barley	9
80	Bosnia and Herzegovina	1999	Beans	10
80	Bosnia and Herzegovina	1999	Beer	70
80	Bosnia and Herzegovina	1999	Beverages, Alcoholic	37
80	Bosnia and Herzegovina	1999	Bovine Meat	17
80	Bosnia and Herzegovina	1999	Butter, Ghee	2
80	Bosnia and Herzegovina	1999	Cheese	13
80	Bosnia and Herzegovina	1999	Cocoa Beans	2
80	Bosnia and Herzegovina	1999	Coffee	1
80	Bosnia and Herzegovina	1999	Cream	1
80	Bosnia and Herzegovina	1999	Eggs	14
80	Bosnia and Herzegovina	1999	Fats, Animals, Raw	3
80	Bosnia and Herzegovina	1999	Freshwater Fish	2
80	Bosnia and Herzegovina	1999	Fruits, Other	36
80	Bosnia and Herzegovina	1999	Grapes	5
80	Bosnia and Herzegovina	1999	Honey	1

FAO country code	Country	Reference year	Food item	Gross food supply (g $capita^{-1}$ d^{-1})
80	Bosnia and Herzegovina	1999	Lemons, Limes	3
80	Bosnia and Herzegovina	1999	Maize	249
80	Bosnia and Herzegovina	1999	Milk, Whole	302
80	Bosnia and Herzegovina	1999	Mutton & Goat Meat	1
80	Bosnia and Herzegovina	1999	Nuts	2
80	Bosnia and Herzegovina	1999	Offals, Edible	3
80	Bosnia and Herzegovina	1999	Onions	28
80	Bosnia and Herzegovina	1999	Oranges, Mandarines	8
80	Bosnia and Herzegovina	1999	Peas	3
80	Bosnia and Herzegovina	1999	Pelagic Fish	3
80	Bosnia and Herzegovina	1999	Pigmeat	18
80	Bosnia and Herzegovina	1999	Pimento	31
80	Bosnia and Herzegovina	1999	Potatoes	205
80	Bosnia and Herzegovina	1999	Poultry Meat	15
80	Bosnia and Herzegovina	1999	Pulses, Other	1
80	Bosnia and Herzegovina	1999	Rice (Milled Equivalent)	2
80	Bosnia and Herzegovina	1999	Rye	4
80	Bosnia and Herzegovina	1999	Soyabean Oil	1
80	Bosnia and Herzegovina	1999	Sugar, Refined Equivalent	29
80	Bosnia and Herzegovina	1999	Sunflowerseed Oil	16
80	Bosnia and Herzegovina	1999	Sweeteners, Other	6
80	Bosnia and Herzegovina	1999	Tomatoes	31
80	Bosnia and Herzegovina	1999	Vegetables, Other	427
80	Bosnia and Herzegovina	1999	Wheat	306
80	Bosnia and Herzegovina	1999	Wine	4
80	Bosnia and Herzegovina	2000	Apples	20
80	Bosnia and Herzegovina	2000	Barley	14
80	Bosnia and Herzegovina	2000	Beans	5
80	Bosnia and Herzegovina	2000	Beer	71
80	Bosnia and Herzegovina	2000	Beverages, Alcoholic	40
80	Bosnia and Herzegovina	2000	Bovine Meat	19
80	Bosnia and Herzegovina	2000	Butter, Ghee	2
80	Bosnia and Herzegovina	2000	Cheese	11
80	Bosnia and Herzegovina	2000	Citrus, Other	1
80	Bosnia and Herzegovina	2000	Cocoa Beans	3
80	Bosnia and Herzegovina	2000	Coffee	1
80	Bosnia and Herzegovina	2000	Demersal Fish	1
80	Bosnia and Herzegovina	2000	Eggs	14
80	Bosnia and Herzegovina	2000	Fats, Animals, Raw	2
80	Bosnia and Herzegovina	2000	Freshwater Fish	2
80	Bosnia and Herzegovina	2000	Fruits, Other	35
80	Bosnia and Herzegovina	2000	Grapes	6
80	Bosnia and Herzegovina	2000	Honey	1
80	Bosnia and Herzegovina	2000	Lemons, Limes	4
80	Bosnia and Herzegovina	2000	Maize	252
80	Bosnia and Herzegovina	2000	Milk, Whole	322
80	Bosnia and Herzegovina	2000	Mutton & Goat Meat	1
80	Bosnia and Herzegovina	2000	Nuts	4
80	Bosnia and Herzegovina	2000	Offals, Edible	3
80	Bosnia and Herzegovina	2000	Onions	20
80	Bosnia and Herzegovina	2000	Oranges, Mandarines	15
80	Bosnia and Herzegovina	2000	Peas	1
80	Bosnia and Herzegovina	2000	Pelagic Fish	4
80	Bosnia and Herzegovina	2000	Pigmeat	16
80	Bosnia and Herzegovina	2000	Pimento	21
80	Bosnia and Herzegovina	2000	Potatoes	191
80	Bosnia and Herzegovina	2000	Poultry Meat	11
80	Bosnia and Herzegovina	2000	Pulses, Other	1
80	Bosnia and Herzegovina	2000	Rice (Milled Equivalent)	2
80	Bosnia and Herzegovina	2000	Rye	4
80	Bosnia and Herzegovina	2000	Soyabean Oil	3
80	Bosnia and Herzegovina	2000	Spices, Other	1
80	Bosnia and Herzegovina	2000	Sugar, Refined Equivalent	29
80	Bosnia and Herzegovina	2000	Sunflowerseed Oil	16
80	Bosnia and Herzegovina	2000	Sweeteners, Other	5
80	Bosnia and Herzegovina	2000	Tomatoes	24
80	Bosnia and Herzegovina	2000	Vegetables, Other	301
80	Bosnia and Herzegovina	2000	Wheat	275
80	Bosnia and Herzegovina	2000	Wine	4
80	Bosnia and Herzegovina	2001	Apples	20
80	Bosnia and Herzegovina	2001	Barley	13
80	Bosnia and Herzegovina	2001	Beans	11
80	Bosnia and Herzegovina	2001	Beer	85
80	Bosnia and Herzegovina	2001	Beverages, Alcoholic	35
80	Bosnia and Herzegovina	2001	Bovine Meat	22
80	Bosnia and Herzegovina	2001	Butter, Ghee	2
80	Bosnia and Herzegovina	2001	Cheese	11
80	Bosnia and Herzegovina	2001	Cocoa Beans	4
80	Bosnia and Herzegovina	2001	Coffee	1
80	Bosnia and Herzegovina	2001	Cream	3
80	Bosnia and Herzegovina	2001	Demersal Fish	1
80	Bosnia and Herzegovina	2001	Eggs	12
80	Bosnia and Herzegovina	2001	Fats, Animals, Raw	3
80	Bosnia and Herzegovina	2001	Freshwater Fish	2
80	Bosnia and Herzegovina	2001	Fruits, Other	38
80	Bosnia and Herzegovina	2001	Grapes	7
80	Bosnia and Herzegovina	2001	Honey	1
80	Bosnia and Herzegovina	2001	Lemons, Limes	4
80	Bosnia and Herzegovina	2001	Maize	265
80	Bosnia and Herzegovina	2001	Milk, Whole	326
80	Bosnia and Herzegovina	2001	Mutton & Goat Meat	1
80	Bosnia and Herzegovina	2001	Nuts	2
80	Bosnia and Herzegovina	2001	Offals, Edible	2
80	Bosnia and Herzegovina	2001	Onions	24
80	Bosnia and Herzegovina	2001	Oranges, Mandarines	15
80	Bosnia and Herzegovina	2001	Peas	2
80	Bosnia and Herzegovina	2001	Pelagic Fish	6

FAO country code	Country	Reference year	Food item	Gross food supply (g *capita*$^{-1}$ d^{-1})
80	Bosnia and Herzegovina	2001	Pigmeat	19
80	Bosnia and Herzegovina	2001	Pimento	22
80	Bosnia and Herzegovina	2001	Potatoes	252
80	Bosnia and Herzegovina	2001	Poultry Meat	11
80	Bosnia and Herzegovina	2001	Pulses, Other	2
80	Bosnia and Herzegovina	2001	Rice (Milled Equivalent)	2
80	Bosnia and Herzegovina	2001	Rye	4
80	Bosnia and Herzegovina	2001	Soyabean Oil	1
80	Bosnia and Herzegovina	2001	Spices, Other	1
80	Bosnia and Herzegovina	2001	Sugar, Refined Equivalent	29
80	Bosnia and Herzegovina	2001	Sunflowerseed Oil	19
80	Bosnia and Herzegovina	2001	Sweeteners, Other	13
80	Bosnia and Herzegovina	2001	Tomatoes	24
80	Bosnia and Herzegovina	2001	Vegetables, Other	326
80	Bosnia and Herzegovina	2001	Wheat	248
80	Bosnia and Herzegovina	2001	Wine	7
80	Bosnia and Herzegovina	2002	Apples	19
80	Bosnia and Herzegovina	2002	Barley	11
80	Bosnia and Herzegovina	2002	Beans	10
80	Bosnia and Herzegovina	2002	Beer	112
80	Bosnia and Herzegovina	2002	Beverages, Alcoholic	34
80	Bosnia and Herzegovina	2002	Bovine Meat	25
80	Bosnia and Herzegovina	2002	Butter, Ghee	2
80	Bosnia and Herzegovina	2002	Cheese	9
80	Bosnia and Herzegovina	2002	Cocoa Beans	4
80	Bosnia and Herzegovina	2002	Coffee	1
80	Bosnia and Herzegovina	2002	Cream	2
80	Bosnia and Herzegovina	2002	Demersal Fish	1
80	Bosnia and Herzegovina	2002	Eggs	13
80	Bosnia and Herzegovina	2002	Fats, Animals, Raw	3
80	Bosnia and Herzegovina	2002	Freshwater Fish	5
80	Bosnia and Herzegovina	2002	Fruits, Other	33
80	Bosnia and Herzegovina	2002	Grapes	9
80	Bosnia and Herzegovina	2002	Honey	1
80	Bosnia and Herzegovina	2002	Lemons, Limes	4
80	Bosnia and Herzegovina	2002	Maize	271
80	Bosnia and Herzegovina	2002	Marine Fish, Other	1
80	Bosnia and Herzegovina	2002	Milk, Whole	334
80	Bosnia and Herzegovina	2002	Mutton & Goat Meat	1
80	Bosnia and Herzegovina	2002	Nuts	2
80	Bosnia and Herzegovina	2002	Offals, Edible	3
80	Bosnia and Herzegovina	2002	Onions	24
80	Bosnia and Herzegovina	2002	Oranges, Mandarines	17
80	Bosnia and Herzegovina	2002	Peas	3
80	Bosnia and Herzegovina	2002	Pelagic Fish	6
80	Bosnia and Herzegovina	2002	Pigmeat	17
80	Bosnia and Herzegovina	2002	Pimento	16
80	Bosnia and Herzegovina	2002	Potatoes	210
80	Bosnia and Herzegovina	2002	Poultry Meat	18
80	Bosnia and Herzegovina	2002	Pulses, Other	3
80	Bosnia and Herzegovina	2002	Rice (Milled Equivalent)	3
80	Bosnia and Herzegovina	2002	Rye	6
80	Bosnia and Herzegovina	2002	Soyabean Oil	1
80	Bosnia and Herzegovina	2002	Soyabeans	3
80	Bosnia and Herzegovina	2002	Sugar, Refined Equivalent	30
80	Bosnia and Herzegovina	2002	Sunflowerseed Oil	18
80	Bosnia and Herzegovina	2002	Sweeteners, Other	13
80	Bosnia and Herzegovina	2002	Tomatoes	31
80	Bosnia and Herzegovina	2002	Vegetables, Other	372
80	Bosnia and Herzegovina	2002	Wheat	249
80	Bosnia and Herzegovina	2002	Wine	4
80	Bosnia and Herzegovina	2003	Apples	36
80	Bosnia and Herzegovina	2003	Bananas	26
80	Bosnia and Herzegovina	2003	Barley	12
80	Bosnia and Herzegovina	2003	Beans	10
80	Bosnia and Herzegovina	2003	Beer	106
80	Bosnia and Herzegovina	2003	Beverages, Alcoholic	42
80	Bosnia and Herzegovina	2003	Bovine Meat	23
80	Bosnia and Herzegovina	2003	Butter, Ghee	2
80	Bosnia and Herzegovina	2003	Cheese	6
80	Bosnia and Herzegovina	2003	Cocoa Beans	3
80	Bosnia and Herzegovina	2003	Coffee	8
80	Bosnia and Herzegovina	2003	Cream	1
80	Bosnia and Herzegovina	2003	Demersal Fish	2
80	Bosnia and Herzegovina	2003	Eggs	12
80	Bosnia and Herzegovina	2003	Fats, Animals, Raw	2
80	Bosnia and Herzegovina	2003	Freshwater Fish	6
80	Bosnia and Herzegovina	2003	Fruits, Other	65
80	Bosnia and Herzegovina	2003	Grapefruit	1
80	Bosnia and Herzegovina	2003	Grapes	8
80	Bosnia and Herzegovina	2003	Groundnuts (Shelled Eq)	1
80	Bosnia and Herzegovina	2003	Honey	1
80	Bosnia and Herzegovina	2003	Lemons, Limes	5
80	Bosnia and Herzegovina	2003	Maize	275
80	Bosnia and Herzegovina	2003	Marine Fish, Other	1
80	Bosnia and Herzegovina	2003	Milk, Whole	364
80	Bosnia and Herzegovina	2003	Mutton & Goat Meat	1
80	Bosnia and Herzegovina	2003	Nuts	5
80	Bosnia and Herzegovina	2003	Offals, Edible	6
80	Bosnia and Herzegovina	2003	Onions	19
80	Bosnia and Herzegovina	2003	Oranges, Mandarines	14
80	Bosnia and Herzegovina	2003	Peas	2
80	Bosnia and Herzegovina	2003	Pelagic Fish	6
80	Bosnia and Herzegovina	2003	Pigmeat	11
80	Bosnia and Herzegovina	2003	Pimento	19
80	Bosnia and Herzegovina	2003	Pineapples	1
80	Bosnia and Herzegovina	2003	Potatoes	219

FAO country code	Country	Reference year	Food item	Gross food supply (g $capita^{-1}$ d^{-1})
80	Bosnia and Herzegovina	2003	Poultry Meat	16
80	Bosnia and Herzegovina	2003	Pulses, Other	2
80	Bosnia and Herzegovina	2003	Rice (Milled Equivalent)	4
80	Bosnia and Herzegovina	2003	Rye	4
80	Bosnia and Herzegovina	2003	Soyabean Oil	1
80	Bosnia and Herzegovina	2003	Sugar, Refined Equivalent	30
80	Bosnia and Herzegovina	2003	Sunflowerseed Oil	15
80	Bosnia and Herzegovina	2003	Sweeteners, Other	8
80	Bosnia and Herzegovina	2003	Tomatoes	28
80	Bosnia and Herzegovina	2003	Vegetables, Other	429
80	Bosnia and Herzegovina	2003	Wheat	207
80	Bosnia and Herzegovina	2003	Wine	6
80	Bosnia and Herzegovina	2004	Apples	69
80	Bosnia and Herzegovina	2004	Bananas	29
80	Bosnia and Herzegovina	2004	Barley	3
80	Bosnia and Herzegovina	2004	Beans	14
80	Bosnia and Herzegovina	2004	Beer	124
80	Bosnia and Herzegovina	2004	Beverages, Alcoholic	41
80	Bosnia and Herzegovina	2004	Bovine Meat	17
80	Bosnia and Herzegovina	2004	Butter, Ghee	2
80	Bosnia and Herzegovina	2004	Cheese	7
80	Bosnia and Herzegovina	2004	Cocoa Beans	6
80	Bosnia and Herzegovina	2004	Coffee	17
80	Bosnia and Herzegovina	2004	Cream	1
80	Bosnia and Herzegovina	2004	Demersal Fish	3
80	Bosnia and Herzegovina	2004	Eggs	11
80	Bosnia and Herzegovina	2004	Fats, Animals, Raw	2
80	Bosnia and Herzegovina	2004	Freshwater Fish	5
80	Bosnia and Herzegovina	2004	Fruits, Other	131
80	Bosnia and Herzegovina	2004	Grapefruit	3
80	Bosnia and Herzegovina	2004	Grapes	14
80	Bosnia and Herzegovina	2004	Groundnuts (Shelled Eq)	1
80	Bosnia and Herzegovina	2004	Honey	2
80	Bosnia and Herzegovina	2004	Lemons, Limes	6
80	Bosnia and Herzegovina	2004	Maize	270
80	Bosnia and Herzegovina	2004	Marine Fish, Other	2
80	Bosnia and Herzegovina	2004	Milk, Whole	389
80	Bosnia and Herzegovina	2004	Mutton & Goat Meat	1
80	Bosnia and Herzegovina	2004	Nuts	5
80	Bosnia and Herzegovina	2004	Offals, Edible	8
80	Bosnia and Herzegovina	2004	Onions	27
80	Bosnia and Herzegovina	2004	Oranges, Mandarines	34
80	Bosnia and Herzegovina	2004	Palm Oil	1
80	Bosnia and Herzegovina	2004	Peas	3
80	Bosnia and Herzegovina	2004	Pelagic Fish	7
80	Bosnia and Herzegovina	2004	Pigmeat	14
80	Bosnia and Herzegovina	2004	Pimento	21
80	Bosnia and Herzegovina	2004	Pineapples	1
80	Bosnia and Herzegovina	2004	Potatoes	249
80	Bosnia and Herzegovina	2004	Poultry Meat	19
80	Bosnia and Herzegovina	2004	Pulses, Other	3
80	Bosnia and Herzegovina	2004	Rice (Milled Equivalent)	5
80	Bosnia and Herzegovina	2004	Rye	4
80	Bosnia and Herzegovina	2004	Soyabean Oil	1
80	Bosnia and Herzegovina	2004	Soyabeans	3
80	Bosnia and Herzegovina	2004	Sugar, Refined Equivalent	31
80	Bosnia and Herzegovina	2004	Sunflowerseed Oil	14
80	Bosnia and Herzegovina	2004	Sweeteners, Other	9
80	Bosnia and Herzegovina	2004	Tomatoes	39
80	Bosnia and Herzegovina	2004	Vegetables, Other	467
80	Bosnia and Herzegovina	2004	Wheat	239
80	Bosnia and Herzegovina	2004	Wine	6
80	Bosnia and Herzegovina	2005	Apples	87
80	Bosnia and Herzegovina	2005	Bananas	29
80	Bosnia and Herzegovina	2005	Barley	2
80	Bosnia and Herzegovina	2005	Beans	13
80	Bosnia and Herzegovina	2005	Beer	130
80	Bosnia and Herzegovina	2005	Beverages, Alcoholic	43
80	Bosnia and Herzegovina	2005	Bovine Meat	22
80	Bosnia and Herzegovina	2005	Butter, Ghee	2
80	Bosnia and Herzegovina	2005	Cheese	8
80	Bosnia and Herzegovina	2005	Cocoa Beans	6
80	Bosnia and Herzegovina	2005	Coconuts - Incl Copra	1
80	Bosnia and Herzegovina	2005	Coffee	16
80	Bosnia and Herzegovina	2005	Cream	1
80	Bosnia and Herzegovina	2005	Demersal Fish	3
80	Bosnia and Herzegovina	2005	Eggs	14
80	Bosnia and Herzegovina	2005	Fats, Animals, Raw	2
80	Bosnia and Herzegovina	2005	Freshwater Fish	5
80	Bosnia and Herzegovina	2005	Fruits, Other	99
80	Bosnia and Herzegovina	2005	Grapefruit	1
80	Bosnia and Herzegovina	2005	Grapes	18
80	Bosnia and Herzegovina	2005	Groundnuts (Shelled Eq)	1
80	Bosnia and Herzegovina	2005	Honey	2
80	Bosnia and Herzegovina	2005	Lemons, Limes	7
80	Bosnia and Herzegovina	2005	Maize	251
80	Bosnia and Herzegovina	2005	Marine Fish, Other	3
80	Bosnia and Herzegovina	2005	Milk, Whole	410
80	Bosnia and Herzegovina	2005	Molluscs, Other	1
80	Bosnia and Herzegovina	2005	Mutton & Goat Meat	1
80	Bosnia and Herzegovina	2005	Nuts	5
80	Bosnia and Herzegovina	2005	Offals, Edible	10
80	Bosnia and Herzegovina	2005	Onions	26
80	Bosnia and Herzegovina	2005	Oranges, Mandarines	34
80	Bosnia and Herzegovina	2005	Palm Oil	1
80	Bosnia and Herzegovina	2005	Peas	3
80	Bosnia and Herzegovina	2005	Pelagic Fish	8

FAO country code	Country	Reference year	Food item	Gross food supply (g *capita*$^{-1}$ d^{-1})
80	Bosnia and Herzegovina	2005	Pigmeat	16
80	Bosnia and Herzegovina	2005	Pimento	19
80	Bosnia and Herzegovina	2005	Pineapples	1
80	Bosnia and Herzegovina	2005	Potatoes	243
80	Bosnia and Herzegovina	2005	Poultry Meat	17
80	Bosnia and Herzegovina	2005	Pulses, Other	3
80	Bosnia and Herzegovina	2005	Rice (Milled Equivalent)	5
80	Bosnia and Herzegovina	2005	Rye	4
80	Bosnia and Herzegovina	2005	Soyabean Oil	1
80	Bosnia and Herzegovina	2005	Soyabeans	1
80	Bosnia and Herzegovina	2005	Sugar, Refined Equivalent	30
80	Bosnia and Herzegovina	2005	Sunflowerseed Oil	16
80	Bosnia and Herzegovina	2005	Sweeteners, Other	12
80	Bosnia and Herzegovina	2005	Tomatoes	36
80	Bosnia and Herzegovina	2005	Vegetables, Other	478
80	Bosnia and Herzegovina	2005	Wheat	226
80	Bosnia and Herzegovina	2005	Wine	7
80	Bosnia and Herzegovina	2006	Apples	52
80	Bosnia and Herzegovina	2006	Bananas	24
80	Bosnia and Herzegovina	2006	Barley	8
80	Bosnia and Herzegovina	2006	Beans	14
80	Bosnia and Herzegovina	2006	Beer	159
80	Bosnia and Herzegovina	2006	Beverages, Alcoholic	44
80	Bosnia and Herzegovina	2006	Bovine Meat	21
80	Bosnia and Herzegovina	2006	Butter, Ghee	2
80	Bosnia and Herzegovina	2006	Cheese	7
80	Bosnia and Herzegovina	2006	Cocoa Beans	6
80	Bosnia and Herzegovina	2006	Coffee	18
80	Bosnia and Herzegovina	2006	Cream	1
80	Bosnia and Herzegovina	2006	Demersal Fish	3
80	Bosnia and Herzegovina	2006	Eggs	12
80	Bosnia and Herzegovina	2006	Fats, Animals, Raw	2
80	Bosnia and Herzegovina	2006	Freshwater Fish	5
80	Bosnia and Herzegovina	2006	Fruits, Other	107
80	Bosnia and Herzegovina	2006	Grapefruit	1
80	Bosnia and Herzegovina	2006	Grapes	19
80	Bosnia and Herzegovina	2006	Groundnuts (Shelled Eq)	1
80	Bosnia and Herzegovina	2006	Honey	2
80	Bosnia and Herzegovina	2006	Lemons, Limes	6
80	Bosnia and Herzegovina	2006	Maize	224
80	Bosnia and Herzegovina	2006	Marine Fish, Other	2
80	Bosnia and Herzegovina	2006	Milk, Whole	422
80	Bosnia and Herzegovina	2006	Molluscs, Other	1
80	Bosnia and Herzegovina	2006	Mutton & Goat Meat	2
80	Bosnia and Herzegovina	2006	Nuts	5
80	Bosnia and Herzegovina	2006	Offals, Edible	10
80	Bosnia and Herzegovina	2006	Onions	29
80	Bosnia and Herzegovina	2006	Oranges, Mandarines	28
80	Bosnia and Herzegovina	2006	Palm Oil	1
80	Bosnia and Herzegovina	2006	Peas	4
80	Bosnia and Herzegovina	2006	Pelagic Fish	6
80	Bosnia and Herzegovina	2006	Pigmeat	14
80	Bosnia and Herzegovina	2006	Pimento	16
80	Bosnia and Herzegovina	2006	Pineapples	1
80	Bosnia and Herzegovina	2006	Potatoes	215
80	Bosnia and Herzegovina	2006	Poultry Meat	15
80	Bosnia and Herzegovina	2006	Pulses, Other	3
80	Bosnia and Herzegovina	2006	Rice (Milled Equivalent)	5
80	Bosnia and Herzegovina	2006	Rye	4
80	Bosnia and Herzegovina	2006	Soyabean Oil	2
80	Bosnia and Herzegovina	2006	Soyabeans	2
80	Bosnia and Herzegovina	2006	Sugar, Refined Equivalent	32
80	Bosnia and Herzegovina	2006	Sunflowerseed Oil	16
80	Bosnia and Herzegovina	2006	Sweeteners, Other	15
80	Bosnia and Herzegovina	2006	Tomatoes	43
80	Bosnia and Herzegovina	2006	Vegetables, Other	468
80	Bosnia and Herzegovina	2006	Wheat	238
80	Bosnia and Herzegovina	2006	Wine	7
80	Bosnia and Herzegovina	2007	Apples	49
80	Bosnia and Herzegovina	2007	Bananas	24
80	Bosnia and Herzegovina	2007	Barley	4
80	Bosnia and Herzegovina	2007	Beans	13
80	Bosnia and Herzegovina	2007	Beer	166
80	Bosnia and Herzegovina	2007	Beverages, Alcoholic	43
80	Bosnia and Herzegovina	2007	Bovine Meat	21
80	Bosnia and Herzegovina	2007	Butter, Ghee	2
80	Bosnia and Herzegovina	2007	Cereals, Other	4
80	Bosnia and Herzegovina	2007	Cheese	10
80	Bosnia and Herzegovina	2007	Cocoa Beans	7
80	Bosnia and Herzegovina	2007	Coffee	18
80	Bosnia and Herzegovina	2007	Cream	1
80	Bosnia and Herzegovina	2007	Demersal Fish	1
80	Bosnia and Herzegovina	2007	Eggs	14
80	Bosnia and Herzegovina	2007	Fats, Animals, Raw	1
80	Bosnia and Herzegovina	2007	Freshwater Fish	5
80	Bosnia and Herzegovina	2007	Fruits, Other	114
80	Bosnia and Herzegovina	2007	Grapefruit	1
80	Bosnia and Herzegovina	2007	Grapes	15
80	Bosnia and Herzegovina	2007	Groundnuts (Shelled Eq)	1
80	Bosnia and Herzegovina	2007	Honey	2
80	Bosnia and Herzegovina	2007	Lemons, Limes	6
80	Bosnia and Herzegovina	2007	Maize	222
80	Bosnia and Herzegovina	2007	Marine Fish, Other	3
80	Bosnia and Herzegovina	2007	Milk, Whole	428
80	Bosnia and Herzegovina	2007	Molluscs, Other	1
80	Bosnia and Herzegovina	2007	Mutton & Goat Meat	1
80	Bosnia and Herzegovina	2007	Nuts	5

FAO country code	Country	Reference year	Food item	Gross food supply (g *capita*$^{-1}$ d^{-1})
80	Bosnia and Herzegovina	2007	Offals, Edible	10
80	Bosnia and Herzegovina	2007	Olive Oil	1
80	Bosnia and Herzegovina	2007	Onions	26
80	Bosnia and Herzegovina	2007	Oranges, Mandarines	26
80	Bosnia and Herzegovina	2007	Palm Oil	1
80	Bosnia and Herzegovina	2007	Peas	3
80	Bosnia and Herzegovina	2007	Pelagic Fish	7
80	Bosnia and Herzegovina	2007	Pigmeat	14
80	Bosnia and Herzegovina	2007	Pimento	14
80	Bosnia and Herzegovina	2007	Pineapples	1
80	Bosnia and Herzegovina	2007	Potatoes	207
80	Bosnia and Herzegovina	2007	Poultry Meat	20
80	Bosnia and Herzegovina	2007	Pulses, Other	3
80	Bosnia and Herzegovina	2007	Rice (Milled Equivalent)	5
80	Bosnia and Herzegovina	2007	Rye	4
80	Bosnia and Herzegovina	2007	Soyabean Oil	3
80	Bosnia and Herzegovina	2007	Soyabeans	2
80	Bosnia and Herzegovina	2007	Sugar, Refined Equivalent	34
80	Bosnia and Herzegovina	2007	Sunflowerseed Oil	15
80	Bosnia and Herzegovina	2007	Sweeteners, Other	18
80	Bosnia and Herzegovina	2007	Tomatoes	36
80	Bosnia and Herzegovina	2007	Vegetables, Other	460
80	Bosnia and Herzegovina	2007	Wheat	251
80	Bosnia and Herzegovina	2007	Wine	9
80	Bosnia and Herzegovina	2008	Apples	48
80	Bosnia and Herzegovina	2008	Bananas	26
80	Bosnia and Herzegovina	2008	Barley	2
80	Bosnia and Herzegovina	2008	Beans	14
80	Bosnia and Herzegovina	2008	Beer	173
80	Bosnia and Herzegovina	2008	Beverages, Alcoholic	40
80	Bosnia and Herzegovina	2008	Bovine Meat	24
80	Bosnia and Herzegovina	2008	Butter, Ghee	2
80	Bosnia and Herzegovina	2008	Cereals, Other	3
80	Bosnia and Herzegovina	2008	Cheese	8
80	Bosnia and Herzegovina	2008	Cocoa Beans	7
80	Bosnia and Herzegovina	2008	Coffee	18
80	Bosnia and Herzegovina	2008	Cream	2
80	Bosnia and Herzegovina	2008	Demersal Fish	2
80	Bosnia and Herzegovina	2008	Eggs	17
80	Bosnia and Herzegovina	2008	Fats, Animals, Raw	2
80	Bosnia and Herzegovina	2008	Freshwater Fish	5
80	Bosnia and Herzegovina	2008	Fruits, Other	107
80	Bosnia and Herzegovina	2008	Grapefruit	2
80	Bosnia and Herzegovina	2008	Grapes	19
80	Bosnia and Herzegovina	2008	Groundnuts (Shelled Eq)	1
80	Bosnia and Herzegovina	2008	Honey	2
80	Bosnia and Herzegovina	2008	Lemons, Limes	6
80	Bosnia and Herzegovina	2008	Maize	222
80	Bosnia and Herzegovina	2008	Marine Fish, Other	3
80	Bosnia and Herzegovina	2008	Milk, Whole	425
80	Bosnia and Herzegovina	2008	Molluscs, Other	1
80	Bosnia and Herzegovina	2008	Mutton & Goat Meat	1
80	Bosnia and Herzegovina	2008	Nuts	5
80	Bosnia and Herzegovina	2008	Offals, Edible	9
80	Bosnia and Herzegovina	2008	Onions	30
80	Bosnia and Herzegovina	2008	Oranges, Mandarines	30
80	Bosnia and Herzegovina	2008	Palm Oil	2
80	Bosnia and Herzegovina	2008	Peas	3
80	Bosnia and Herzegovina	2008	Pelagic Fish	4
80	Bosnia and Herzegovina	2008	Pigmeat	14
80	Bosnia and Herzegovina	2008	Pimento	13
80	Bosnia and Herzegovina	2008	Pineapples	1
80	Bosnia and Herzegovina	2008	Potatoes	209
80	Bosnia and Herzegovina	2008	Poultry Meat	29
80	Bosnia and Herzegovina	2008	Pulses, Other	3
80	Bosnia and Herzegovina	2008	Rice (Milled Equivalent)	5
80	Bosnia and Herzegovina	2008	Rye	4
80	Bosnia and Herzegovina	2008	Soyabean Oil	4
80	Bosnia and Herzegovina	2008	Soyabeans	4
80	Bosnia and Herzegovina	2008	Sugar, Refined Equivalent	35
80	Bosnia and Herzegovina	2008	Sunflowerseed Oil	14
80	Bosnia and Herzegovina	2008	Sweeteners, Other	20
80	Bosnia and Herzegovina	2008	Tomatoes	43
80	Bosnia and Herzegovina	2008	Vegetables, Other	477
80	Bosnia and Herzegovina	2008	Wheat	251
80	Bosnia and Herzegovina	2008	Wine	8
80	Bosnia and Herzegovina	2009	Apples	62
80	Bosnia and Herzegovina	2009	Bananas	24
80	Bosnia and Herzegovina	2009	Barley	3
80	Bosnia and Herzegovina	2009	Beans	14
80	Bosnia and Herzegovina	2009	Beer	160
80	Bosnia and Herzegovina	2009	Beverages, Alcoholic	47
80	Bosnia and Herzegovina	2009	Bovine Meat	24
80	Bosnia and Herzegovina	2009	Butter, Ghee	2
80	Bosnia and Herzegovina	2009	Cereals, Other	2
80	Bosnia and Herzegovina	2009	Cheese	8
80	Bosnia and Herzegovina	2009	Cocoa Beans	6
80	Bosnia and Herzegovina	2009	Coffee	16
80	Bosnia and Herzegovina	2009	Cream	1
80	Bosnia and Herzegovina	2009	Demersal Fish	2
80	Bosnia and Herzegovina	2009	Eggs	15
80	Bosnia and Herzegovina	2009	Fats, Animals, Raw	3
80	Bosnia and Herzegovina	2009	Freshwater Fish	5
80	Bosnia and Herzegovina	2009	Fruits, Other	123
80	Bosnia and Herzegovina	2009	Grapefruit	1
80	Bosnia and Herzegovina	2009	Grapes	19
80	Bosnia and Herzegovina	2009	Groundnuts (Shelled Eq)	1

FAO countrycode	Country	Reference year	Food item	Gross food supply (g *capita*$^{-1}$ d^{-1})
80	Bosnia and Herzegovina	2009	Honey	3
80	Bosnia and Herzegovina	2009	Lemons, Limes	6
80	Bosnia and Herzegovina	2009	Maize	224
80	Bosnia and Herzegovina	2009	Marine Fish, Other	3
80	Bosnia and Herzegovina	2009	Milk, Whole	416
80	Bosnia and Herzegovina	2009	Molluscs, Other	1
80	Bosnia and Herzegovina	2009	Mutton & Goat Meat	1
80	Bosnia and Herzegovina	2009	Nuts	6
80	Bosnia and Herzegovina	2009	Offals, Edible	10
80	Bosnia and Herzegovina	2009	Onions	29
80	Bosnia and Herzegovina	2009	Oranges, Mandarines	28
80	Bosnia and Herzegovina	2009	Palm Oil	3
80	Bosnia and Herzegovina	2009	Peas	2
80	Bosnia and Herzegovina	2009	Pelagic Fish	6
80	Bosnia and Herzegovina	2009	Pigmeat	18
80	Bosnia and Herzegovina	2009	Pimento	17
80	Bosnia and Herzegovina	2009	Pineapples	1
80	Bosnia and Herzegovina	2009	Potatoes	208
80	Bosnia and Herzegovina	2009	Poultry Meat	31
80	Bosnia and Herzegovina	2009	Pulses, Other	3
80	Bosnia and Herzegovina	2009	Rice (Milled Equivalent)	5
80	Bosnia and Herzegovina	2009	Rye	4
80	Bosnia and Herzegovina	2009	Soyabean Oil	3
80	Bosnia and Herzegovina	2009	Soyabeans	4
80	Bosnia and Herzegovina	2009	Sugar, Refined Equivalent	31
80	Bosnia and Herzegovina	2009	Sunflowerseed Oil	11
80	Bosnia and Herzegovina	2009	Sweeteners, Other	17
80	Bosnia and Herzegovina	2009	Tomatoes	46
80	Bosnia and Herzegovina	2009	Vegetables, Other	461
80	Bosnia and Herzegovina	2009	Wheat	248
80	Bosnia and Herzegovina	2009	Wine	7
80	Bosnia and Herzegovina	2010	Apples	51
80	Bosnia and Herzegovina	2010	Bananas	24
80	Bosnia and Herzegovina	2010	Barley	7
80	Bosnia and Herzegovina	2010	Beans	12
80	Bosnia and Herzegovina	2010	Beer	153
80	Bosnia and Herzegovina	2010	Beverages, Alcoholic	46
80	Bosnia and Herzegovina	2010	Bovine Meat	25
80	Bosnia and Herzegovina	2010	Butter, Ghee	2
80	Bosnia and Herzegovina	2010	Cereals, Other	2
80	Bosnia and Herzegovina	2010	Cheese	8
80	Bosnia and Herzegovina	2010	Cocoa Beans	7
80	Bosnia and Herzegovina	2010	Coffee	15
80	Bosnia and Herzegovina	2010	Cream	1
80	Bosnia and Herzegovina	2010	Demersal Fish	2
80	Bosnia and Herzegovina	2010	Eggs	12
80	Bosnia and Herzegovina	2010	Fats, Animals, Raw	3
80	Bosnia and Herzegovina	2010	Freshwater Fish	5
80	Bosnia and Herzegovina	2010	Fruits, Other	115
80	Bosnia and Herzegovina	2010	Grapefruit	2
80	Bosnia and Herzegovina	2010	Grapes	16
80	Bosnia and Herzegovina	2010	Groundnuts (Shelled Eq)	1
80	Bosnia and Herzegovina	2010	Honey	3
80	Bosnia and Herzegovina	2010	Lemons, Limes	5
80	Bosnia and Herzegovina	2010	Maize	220
80	Bosnia and Herzegovina	2010	Marine Fish, Other	2
80	Bosnia and Herzegovina	2010	Milk, Whole	408
80	Bosnia and Herzegovina	2010	Molluscs, Other	1
80	Bosnia and Herzegovina	2010	Mutton & Goat Meat	2
80	Bosnia and Herzegovina	2010	Nuts	6
80	Bosnia and Herzegovina	2010	Offals, Edible	7
80	Bosnia and Herzegovina	2010	Olive Oil	1
80	Bosnia and Herzegovina	2010	Onions	28
80	Bosnia and Herzegovina	2010	Oranges, Mandarines	31
80	Bosnia and Herzegovina	2010	Palm Oil	2
80	Bosnia and Herzegovina	2010	Peas	2
80	Bosnia and Herzegovina	2010	Pelagic Fish	5
80	Bosnia and Herzegovina	2010	Pigmeat	22
80	Bosnia and Herzegovina	2010	Pimento	19
80	Bosnia and Herzegovina	2010	Pineapples	2
80	Bosnia and Herzegovina	2010	Potatoes	209
80	Bosnia and Herzegovina	2010	Poultry Meat	33
80	Bosnia and Herzegovina	2010	Pulses, Other	3
80	Bosnia and Herzegovina	2010	Rice (Milled Equivalent)	5
80	Bosnia and Herzegovina	2010	Rye	4
80	Bosnia and Herzegovina	2010	Soyabean Oil	3
80	Bosnia and Herzegovina	2010	Soyabeans	2
80	Bosnia and Herzegovina	2010	Sugar, Refined Equivalent	34
80	Bosnia and Herzegovina	2010	Sunflowerseed Oil	14
80	Bosnia and Herzegovina	2010	Sweeteners, Other	14
80	Bosnia and Herzegovina	2010	Tomatoes	41
80	Bosnia and Herzegovina	2010	Vegetables, Other	453
80	Bosnia and Herzegovina	2010	Wheat	250
80	Bosnia and Herzegovina	2010	Wine	7
80	Bosnia and Herzegovina	2011	Apples	48
80	Bosnia and Herzegovina	2011	Bananas	24
80	Bosnia and Herzegovina	2011	Barley	7
80	Bosnia and Herzegovina	2011	Beans	11
80	Bosnia and Herzegovina	2011	Beer	155
80	Bosnia and Herzegovina	2011	Beverages, Alcoholic	47
80	Bosnia and Herzegovina	2011	Beverages, Fermented	3
80	Bosnia and Herzegovina	2011	Bovine Meat	26
80	Bosnia and Herzegovina	2011	Butter, Ghee	2
80	Bosnia and Herzegovina	2011	Cereals, Other	2
80	Bosnia and Herzegovina	2011	Cheese	8
80	Bosnia and Herzegovina	2011	Cocoa Beans	7
80	Bosnia and Herzegovina	2011	Coffee	17

FAO country code	Country	Reference year	Food item	Gross food supply (g *capita*$^{-1}$ d^{-1})
80	Bosnia and Herzegovina	2011	Cream	2
80	Bosnia and Herzegovina	2011	Demersal Fish	2
80	Bosnia and Herzegovina	2011	Eggs	10
80	Bosnia and Herzegovina	2011	Fats, Animals, Raw	3
80	Bosnia and Herzegovina	2011	Freshwater Fish	6
80	Bosnia and Herzegovina	2011	Fruits, Other	121
80	Bosnia and Herzegovina	2011	Grapefruit	1
80	Bosnia and Herzegovina	2011	Grapes	17
80	Bosnia and Herzegovina	2011	Groundnuts (Shelled Eq)	1
80	Bosnia and Herzegovina	2011	Honey	2
80	Bosnia and Herzegovina	2011	Lemons, Limes	6
80	Bosnia and Herzegovina	2011	Maize	227
80	Bosnia and Herzegovina	2011	Marine Fish, Other	3
80	Bosnia and Herzegovina	2011	Milk, Whole	404
80	Bosnia and Herzegovina	2011	Molluscs, Other	1
80	Bosnia and Herzegovina	2011	Mutton & Goat Meat	2
80	Bosnia and Herzegovina	2011	Nuts	5
80	Bosnia and Herzegovina	2011	Offals, Edible	7
80	Bosnia and Herzegovina	2011	Olive Oil	1
80	Bosnia and Herzegovina	2011	Onions	29
80	Bosnia and Herzegovina	2011	Oranges, Mandarines	30
80	Bosnia and Herzegovina	2011	Palm Oil	2
80	Bosnia and Herzegovina	2011	Peas	2
80	Bosnia and Herzegovina	2011	Pelagic Fish	4
80	Bosnia and Herzegovina	2011	Pigmeat	25
80	Bosnia and Herzegovina	2011	Pimento	20
80	Bosnia and Herzegovina	2011	Pineapples	1
80	Bosnia and Herzegovina	2011	Potatoes	206
80	Bosnia and Herzegovina	2011	Poultry Meat	39
80	Bosnia and Herzegovina	2011	Pulses, Other	3
80	Bosnia and Herzegovina	2011	Rice (Milled Equivalent)	5
80	Bosnia and Herzegovina	2011	Rye	4
80	Bosnia and Herzegovina	2011	Soyabean Oil	4
80	Bosnia and Herzegovina	2011	Soyabeans	3
80	Bosnia and Herzegovina	2011	Sugar, Refined Equivalent	37
80	Bosnia and Herzegovina	2011	Sunflowerseed Oil	14
80	Bosnia and Herzegovina	2011	Sweeteners, Other	13
80	Bosnia and Herzegovina	2011	Tomatoes	45
80	Bosnia and Herzegovina	2011	Vegetables, Other	442
80	Bosnia and Herzegovina	2011	Wheat	245
80	Bosnia and Herzegovina	2011	Wine	5
20	Botswana	1992	Apples	8
20	Botswana	1992	Bananas	5
20	Botswana	1992	Beans	1
20	Botswana	1992	Beer	91
20	Botswana	1992	Beverages, Alcoholic	4
20	Botswana	1992	Beverages, Fermented	54
20	Botswana	1992	Bovine Meat	47
20	Botswana	1992	Butter, Ghee	3
20	Botswana	1992	Cereals, Other	5
20	Botswana	1992	Cheese	7
20	Botswana	1992	Citrus, Other	2
20	Botswana	1992	Cocoa Beans	1
20	Botswana	1992	Coffee	1
20	Botswana	1992	Cottonseed Oil	1
20	Botswana	1992	Cream	1
20	Botswana	1992	Crustaceans	1
20	Botswana	1992	Eggs	3
20	Botswana	1992	Fats, Animals, Raw	17
20	Botswana	1992	Freshwater Fish	2
20	Botswana	1992	Fruits, Other	64
20	Botswana	1992	Grapefruit	2
20	Botswana	1992	Grapes	5
20	Botswana	1992	Maize	98
20	Botswana	1992	Marine Fish, Other	9
20	Botswana	1992	Meat, Other	14
20	Botswana	1992	Milk, Whole	233
20	Botswana	1992	Mutton & Goat Meat	13
20	Botswana	1992	Nuts	1
20	Botswana	1992	Offals, Edible	12
20	Botswana	1992	Oilcrops Oil, Other	5
20	Botswana	1992	Onions	5
20	Botswana	1992	Oranges, Mandarines	27
20	Botswana	1992	Peas	5
20	Botswana	1992	Pelagic Fish	1
20	Botswana	1992	Pepper	1
20	Botswana	1992	Pigmeat	2
20	Botswana	1992	Pineapples	1
20	Botswana	1992	Potatoes	44
20	Botswana	1992	Poultry Meat	14
20	Botswana	1992	Pulses, Other	26
20	Botswana	1992	Rice (Milled Equivalent)	18
20	Botswana	1992	Roots, Other	119
20	Botswana	1992	Sorghum	64
20	Botswana	1992	Soyabean Oil	2
20	Botswana	1992	Soyabeans	1
20	Botswana	1992	Spices, Other	2
20	Botswana	1992	Sugar, Refined Equivalent	49
20	Botswana	1992	Sunflowerseed Oil	4
20	Botswana	1992	Sweeteners, Other	27
20	Botswana	1992	Tea	4
20	Botswana	1992	Tomatoes	7
20	Botswana	1992	Vegetables, Other	64
20	Botswana	1992	Wheat	97
20	Botswana	1992	Wine	12
20	Botswana	1993	Apples	7
20	Botswana	1993	Bananas	5

FAO country code	Country	Reference year	Food item	Gross food supply (g $capita^{-1}$ d^{-1})
20	Botswana	1993	Beans	1
20	Botswana	1993	Beer	96
20	Botswana	1993	Beverages, Alcoholic	2
20	Botswana	1993	Beverages, Fermented	55
20	Botswana	1993	Bovine Meat	40
20	Botswana	1993	Butter, Ghee	2
20	Botswana	1993	Cereals, Other	3
20	Botswana	1993	Cheese	5
20	Botswana	1993	Citrus, Other	1
20	Botswana	1993	Cocoa Beans	1
20	Botswana	1993	Coffee	3
20	Botswana	1993	Cottonseed Oil	1
20	Botswana	1993	Cream	1
20	Botswana	1993	Crustaceans	1
20	Botswana	1993	Eggs	3
20	Botswana	1993	Fats, Animals, Raw	14
20	Botswana	1993	Freshwater Fish	2
20	Botswana	1993	Fruits, Other	88
20	Botswana	1993	Grapefruit	2
20	Botswana	1993	Grapes	6
20	Botswana	1993	Groundnuts (Shelled Eq)	1
20	Botswana	1993	Maize	74
20	Botswana	1993	Marine Fish, Other	7
20	Botswana	1993	Meat, Other	13
20	Botswana	1993	Milk, Whole	199
20	Botswana	1993	Millet	2
20	Botswana	1993	Mutton & Goat Meat	13
20	Botswana	1993	Nuts	1
20	Botswana	1993	Offals, Edible	13
20	Botswana	1993	Oilcrops Oil, Other	4
20	Botswana	1993	Onions	6
20	Botswana	1993	Oranges, Mandarines	23
20	Botswana	1993	Peas	1
20	Botswana	1993	Pelagic Fish	1
20	Botswana	1993	Pigmeat	2
20	Botswana	1993	Potatoes	28
20	Botswana	1993	Poultry Meat	14
20	Botswana	1993	Pulses, Other	29
20	Botswana	1993	Rice (Milled Equivalent)	20
20	Botswana	1993	Roots, Other	116
20	Botswana	1993	Sorghum	63
20	Botswana	1993	Soyabean Oil	2
20	Botswana	1993	Soyabeans	1
20	Botswana	1993	Spices, Other	1
20	Botswana	1993	Sugar, Refined Equivalent	51
20	Botswana	1993	Sunflowerseed Oil	3
20	Botswana	1993	Sweet Potatoes	1
20	Botswana	1993	Sweeteners, Other	13
20	Botswana	1993	Tea	4
20	Botswana	1993	Tomatoes	8
20	Botswana	1993	Vegetables, Other	74
20	Botswana	1993	Wheat	132
20	Botswana	1993	Wine	3
20	Botswana	1994	Apples	7
20	Botswana	1994	Bananas	5
20	Botswana	1994	Beans	1
20	Botswana	1994	Beer	68
20	Botswana	1994	Beverages, Alcoholic	2
20	Botswana	1994	Beverages, Fermented	68
20	Botswana	1994	Bovine Meat	25
20	Botswana	1994	Butter, Ghee	3
20	Botswana	1994	Cassava	1
20	Botswana	1994	Cereals, Other	12
20	Botswana	1994	Cheese	5
20	Botswana	1994	Citrus, Other	1
20	Botswana	1994	Cocoa Beans	2
20	Botswana	1994	Coffee	3
20	Botswana	1994	Cottonseed Oil	1
20	Botswana	1994	Crustaceans	1
20	Botswana	1994	Eggs	3
20	Botswana	1994	Fats, Animals, Raw	9
20	Botswana	1994	Freshwater Fish	1
20	Botswana	1994	Fruits, Other	62
20	Botswana	1994	Grapefruit	1
20	Botswana	1994	Grapes	2
20	Botswana	1994	Groundnuts (Shelled Eq)	1
20	Botswana	1994	Maize	107
20	Botswana	1994	Marine Fish, Other	6
20	Botswana	1994	Meat, Other	14
20	Botswana	1994	Milk, Whole	231
20	Botswana	1994	Millet	3
20	Botswana	1994	Mutton & Goat Meat	12
20	Botswana	1994	Nuts	1
20	Botswana	1994	Offals, Edible	10
20	Botswana	1994	Oilcrops Oil, Other	2
20	Botswana	1994	Onions	7
20	Botswana	1994	Oranges, Mandarines	17
20	Botswana	1994	Peas	5
20	Botswana	1994	Pelagic Fish	1
20	Botswana	1994	Pigmeat	3
20	Botswana	1994	Potatoes	32
20	Botswana	1994	Poultry Meat	12
20	Botswana	1994	Pulses, Other	29
20	Botswana	1994	Rape and Mustard Oil	2
20	Botswana	1994	Rice (Milled Equivalent)	17
20	Botswana	1994	Roots, Other	117
20	Botswana	1994	Sorghum	77

FAO country code	Country	Reference year	Food item	Gross food supply (g *capita*$^{-1}$ d^{-1})
20	Botswana	1994	Soyabean Oil	2
20	Botswana	1994	Soyabeans	2
20	Botswana	1994	Spices, Other	2
20	Botswana	1994	Sugar, Refined Equivalent	51
20	Botswana	1994	Sunflowerseed Oil	3
20	Botswana	1994	Sweeteners, Other	10
20	Botswana	1994	Tea	2
20	Botswana	1994	Tomatoes	8
20	Botswana	1994	Vegetables, Other	65
20	Botswana	1994	Wheat	132
20	Botswana	1994	Wine	5
20	Botswana	1995	Apples	7
20	Botswana	1995	Bananas	4
20	Botswana	1995	Beans	5
20	Botswana	1995	Beer	66
20	Botswana	1995	Beverages, Alcoholic	2
20	Botswana	1995	Beverages, Fermented	79
20	Botswana	1995	Bovine Meat	41
20	Botswana	1995	Butter, Ghee	2
20	Botswana	1995	Cereals, Other	11
20	Botswana	1995	Cheese	5
20	Botswana	1995	Citrus, Other	1
20	Botswana	1995	Cocoa Beans	2
20	Botswana	1995	Coffee	2
20	Botswana	1995	Cottonseed Oil	1
20	Botswana	1995	Crustaceans	1
20	Botswana	1995	Eggs	5
20	Botswana	1995	Fats, Animals, Raw	4
20	Botswana	1995	Freshwater Fish	1
20	Botswana	1995	Fruits, Other	59
20	Botswana	1995	Grapes	2
20	Botswana	1995	Groundnuts (Shelled Eq)	1
20	Botswana	1995	Maize	133
20	Botswana	1995	Marine Fish, Other	5
20	Botswana	1995	Meat, Other	11
20	Botswana	1995	Milk, Whole	245
20	Botswana	1995	Millet	2
20	Botswana	1995	Mutton & Goat Meat	18
20	Botswana	1995	Nuts	1
20	Botswana	1995	Offals, Edible	12
20	Botswana	1995	Oilcrops Oil, Other	2
20	Botswana	1995	Onions	6
20	Botswana	1995	Oranges, Mandarines	17
20	Botswana	1995	Peas	1
20	Botswana	1995	Pelagic Fish	1
20	Botswana	1995	Pigmeat	2
20	Botswana	1995	Potatoes	25
20	Botswana	1995	Poultry Meat	15
20	Botswana	1995	Pulses, Other	23
20	Botswana	1995	Rape and Mustard Oil	1
20	Botswana	1995	Rice (Milled Equivalent)	13
20	Botswana	1995	Roots, Other	117
20	Botswana	1995	Rye	1
20	Botswana	1995	Sorghum	80
20	Botswana	1995	Soyabean Oil	3
20	Botswana	1995	Soyabeans	2
20	Botswana	1995	Spices, Other	2
20	Botswana	1995	Sugar Cane	1
20	Botswana	1995	Sugar, Refined Equivalent	50
20	Botswana	1995	Sunflowerseed Oil	10
20	Botswana	1995	Sweeteners, Other	8
20	Botswana	1995	Tea	2
20	Botswana	1995	Tomatoes	8
20	Botswana	1995	Vegetables, Other	51
20	Botswana	1995	Wheat	96
20	Botswana	1995	Wine	3
20	Botswana	1996	Apples	8
20	Botswana	1996	Bananas	5
20	Botswana	1996	Beans	6
20	Botswana	1996	Beer	75
20	Botswana	1996	Beverages, Alcoholic	2
20	Botswana	1996	Beverages, Fermented	84
20	Botswana	1996	Bovine Meat	43
20	Botswana	1996	Butter, Ghee	2
20	Botswana	1996	Cereals, Other	33
20	Botswana	1996	Cheese	7
20	Botswana	1996	Citrus, Other	1
20	Botswana	1996	Cocoa Beans	1
20	Botswana	1996	Coffee	2
20	Botswana	1996	Cottonseed Oil	1
20	Botswana	1996	Demersal Fish	1
20	Botswana	1996	Eggs	2
20	Botswana	1996	Fats, Animals, Raw	4
20	Botswana	1996	Freshwater Fish	1
20	Botswana	1996	Fruits, Other	54
20	Botswana	1996	Grapes	3
20	Botswana	1996	Groundnuts (Shelled Eq)	1
20	Botswana	1996	Maize	105
20	Botswana	1996	Marine Fish, Other	7
20	Botswana	1996	Meat, Other	12
20	Botswana	1996	Milk, Whole	225
20	Botswana	1996	Millet	3
20	Botswana	1996	Mutton & Goat Meat	14
20	Botswana	1996	Nuts	1
20	Botswana	1996	Offals, Edible	12
20	Botswana	1996	Oilcrops Oil, Other	6
20	Botswana	1996	Onions	8

FAO country code	Country	Reference year	Food item	Gross food supply (g $capita^{-1}$ d^{-1})
20	Botswana	1996	Oranges, Mandarines	19
20	Botswana	1996	Peas	4
20	Botswana	1996	Pigmeat	3
20	Botswana	1996	Potatoes	28
20	Botswana	1996	Poultry Meat	13
20	Botswana	1996	Pulses, Other	25
20	Botswana	1996	Rice (Milled Equivalent)	19
20	Botswana	1996	Roots, Other	116
20	Botswana	1996	Sorghum	96
20	Botswana	1996	Soyabean Oil	3
20	Botswana	1996	Soyabeans	2
20	Botswana	1996	Spices, Other	2
20	Botswana	1996	Sugar, Refined Equivalent	52
20	Botswana	1996	Sunflowerseed Oil	10
20	Botswana	1996	Sweeteners, Other	4
20	Botswana	1996	Tea	3
20	Botswana	1996	Tomatoes	8
20	Botswana	1996	Vegetables, Other	54
20	Botswana	1996	Wheat	84
20	Botswana	1996	Wine	3
20	Botswana	1997	Apples	8
20	Botswana	1997	Bananas	7
20	Botswana	1997	Beans	5
20	Botswana	1997	Beer	66
20	Botswana	1997	Beverages, Alcoholic	2
20	Botswana	1997	Beverages, Fermented	74
20	Botswana	1997	Bovine Meat	29
20	Botswana	1997	Butter, Ghee	2
20	Botswana	1997	Cereals, Other	23
20	Botswana	1997	Cheese	9
20	Botswana	1997	Citrus, Other	1
20	Botswana	1997	Cocoa Beans	1
20	Botswana	1997	Coffee	5
20	Botswana	1997	Cream	1
20	Botswana	1997	Crustaceans	1
20	Botswana	1997	Demersal Fish	1
20	Botswana	1997	Eggs	3
20	Botswana	1997	Fats, Animals, Raw	3
20	Botswana	1997	Freshwater Fish	2
20	Botswana	1997	Fruits, Other	60
20	Botswana	1997	Grapes	1
20	Botswana	1997	Groundnuts (Shelled Eq)	1
20	Botswana	1997	Maize	108
20	Botswana	1997	Marine Fish, Other	6
20	Botswana	1997	Meat, Other	10
20	Botswana	1997	Milk, Whole	221
20	Botswana	1997	Millet	3
20	Botswana	1997	Mutton & Goat Meat	16
20	Botswana	1997	Nuts	1
20	Botswana	1997	Offals, Edible	10
20	Botswana	1997	Oilcrops Oil, Other	7
20	Botswana	1997	Onions	7
20	Botswana	1997	Oranges, Mandarines	29
20	Botswana	1997	Pelagic Fish	1
20	Botswana	1997	Pepper	1
20	Botswana	1997	Pigmeat	4
20	Botswana	1997	Potatoes	36
20	Botswana	1997	Poultry Meat	15
20	Botswana	1997	Pulses, Other	23
20	Botswana	1997	Rice (Milled Equivalent)	19
20	Botswana	1997	Roots, Other	116
20	Botswana	1997	Rye	1
20	Botswana	1997	Sorghum	82
20	Botswana	1997	Soyabean Oil	3
20	Botswana	1997	Soyabeans	2
20	Botswana	1997	Spices, Other	2
20	Botswana	1997	Sugar, Refined Equivalent	53
20	Botswana	1997	Sunflowerseed Oil	10
20	Botswana	1997	Sweeteners, Other	8
20	Botswana	1997	Tea	4
20	Botswana	1997	Tomatoes	10
20	Botswana	1997	Vegetables, Other	56
20	Botswana	1997	Wheat	94
20	Botswana	1997	Wine	4
20	Botswana	1998	Apples	8
20	Botswana	1998	Bananas	8
20	Botswana	1998	Beans	6
20	Botswana	1998	Beer	69
20	Botswana	1998	Beverages, Alcoholic	3
20	Botswana	1998	Beverages, Fermented	74
20	Botswana	1998	Bovine Meat	21
20	Botswana	1998	Butter, Ghee	2
20	Botswana	1998	Cereals, Other	11
20	Botswana	1998	Cheese	7
20	Botswana	1998	Citrus, Other	1
20	Botswana	1998	Cocoa Beans	1
20	Botswana	1998	Coffee	2
20	Botswana	1998	Cottonseed Oil	1
20	Botswana	1998	Cream	1
20	Botswana	1998	Demersal Fish	1
20	Botswana	1998	Eggs	4
20	Botswana	1998	Fats, Animals, Raw	3
20	Botswana	1998	Freshwater Fish	1
20	Botswana	1998	Fruits, Other	61
20	Botswana	1998	Grapes	1
20	Botswana	1998	Maize	114
20	Botswana	1998	Marine Fish, Other	4

FAO country code	Country	Reference year	Food item	Gross food supply (g *capita*$^{-1}$ d^{-1})
20	Botswana	1998	Meat, Other	11
20	Botswana	1998	Milk, Whole	231
20	Botswana	1998	Millet	3
20	Botswana	1998	Mutton & Goat Meat	14
20	Botswana	1998	Nuts	1
20	Botswana	1998	Offals, Edible	10
20	Botswana	1998	Oilcrops Oil, Other	6
20	Botswana	1998	Onions	7
20	Botswana	1998	Oranges, Mandarines	35
20	Botswana	1998	Peas	1
20	Botswana	1998	Pelagic Fish	1
20	Botswana	1998	Pepper	1
20	Botswana	1998	Pigmeat	4
20	Botswana	1998	Potatoes	32
20	Botswana	1998	Poultry Meat	12
20	Botswana	1998	Pulses, Other	17
20	Botswana	1998	Rice (Milled Equivalent)	18
20	Botswana	1998	Roots, Other	118
20	Botswana	1998	Rye	2
20	Botswana	1998	Sorghum	81
20	Botswana	1998	Soyabean Oil	3
20	Botswana	1998	Soyabeans	1
20	Botswana	1998	Spices, Other	2
20	Botswana	1998	Sugar, Refined Equivalent	53
20	Botswana	1998	Sunflowerseed Oil	9
20	Botswana	1998	Sweeteners, Other	8
20	Botswana	1998	Tea	3
20	Botswana	1998	Tomatoes	10
20	Botswana	1998	Vegetables, Other	54
20	Botswana	1998	Wheat	85
20	Botswana	1998	Wine	3
20	Botswana	1999	Apples	9
20	Botswana	1999	Bananas	13
20	Botswana	1999	Beans	7
20	Botswana	1999	Beer	68
20	Botswana	1999	Beverages, Alcoholic	2
20	Botswana	1999	Beverages, Fermented	63
20	Botswana	1999	Bovine Meat	12
20	Botswana	1999	Butter, Ghee	2
20	Botswana	1999	Cereals, Other	4
20	Botswana	1999	Cheese	14
20	Botswana	1999	Citrus, Other	1
20	Botswana	1999	Cocoa Beans	1
20	Botswana	1999	Coconuts - Incl Copra	1
20	Botswana	1999	Coffee	2
20	Botswana	1999	Cream	4
20	Botswana	1999	Crustaceans	1
20	Botswana	1999	Demersal Fish	1
20	Botswana	1999	Eggs	5
20	Botswana	1999	Fats, Animals, Raw	2
20	Botswana	1999	Fruits, Other	112
20	Botswana	1999	Grapes	1
20	Botswana	1999	Maize	114
20	Botswana	1999	Marine Fish, Other	2
20	Botswana	1999	Meat, Other	15
20	Botswana	1999	Milk, Whole	210
20	Botswana	1999	Millet	1
20	Botswana	1999	Mutton & Goat Meat	13
20	Botswana	1999	Offals, Edible	8
20	Botswana	1999	Oilcrops Oil, Other	5
20	Botswana	1999	Onions	8
20	Botswana	1999	Oranges, Mandarines	29
20	Botswana	1999	Peas	2
20	Botswana	1999	Pelagic Fish	6
20	Botswana	1999	Pepper	1
20	Botswana	1999	Pigmeat	4
20	Botswana	1999	Potatoes	37
20	Botswana	1999	Poultry Meat	13
20	Botswana	1999	Pulses, Other	24
20	Botswana	1999	Rice (Milled Equivalent)	20
20	Botswana	1999	Roots, Other	118
20	Botswana	1999	Rye	1
20	Botswana	1999	Sorghum	65
20	Botswana	1999	Soyabean Oil	4
20	Botswana	1999	Soyabeans	2
20	Botswana	1999	Spices, Other	2
20	Botswana	1999	Sugar, Refined Equivalent	54
20	Botswana	1999	Sunflowerseed Oil	12
20	Botswana	1999	Sweeteners, Other	8
20	Botswana	1999	Tea	2
20	Botswana	1999	Tomatoes	10
20	Botswana	1999	Vegetables, Other	55
20	Botswana	1999	Wheat	95
20	Botswana	1999	Wine	4
20	Botswana	2000	Apples	27
20	Botswana	2000	Bananas	18
20	Botswana	2000	Beans	7
20	Botswana	2000	Beer	67
20	Botswana	2000	Beverages, Alcoholic	2
20	Botswana	2000	Beverages, Fermented	70
20	Botswana	2000	Bovine Meat	13
20	Botswana	2000	Butter, Ghee	2
20	Botswana	2000	Cereals, Other	3
20	Botswana	2000	Cheese	10
20	Botswana	2000	Citrus, Other	1
20	Botswana	2000	Cocoa Beans	1
20	Botswana	2000	Coconuts - Incl Copra	1

FAO country code	Country	Reference year	Food item	Gross food supply (g *capita*$^{-1}$ d^{-1})
20	Botswana	2000	Coffee	2
20	Botswana	2000	Cottonseed Oil	2
20	Botswana	2000	Cream	5
20	Botswana	2000	Crustaceans	1
20	Botswana	2000	Eggs	5
20	Botswana	2000	Fats, Animals, Raw	2
20	Botswana	2000	Freshwater Fish	1
20	Botswana	2000	Fruits, Other	72
20	Botswana	2000	Grapes	2
20	Botswana	2000	Groundnuts (Shelled Eq)	1
20	Botswana	2000	Maize	110
20	Botswana	2000	Marine Fish, Other	1
20	Botswana	2000	Meat, Other	18
20	Botswana	2000	Milk, Whole	268
20	Botswana	2000	Millet	1
20	Botswana	2000	Molluscs, Other	1
20	Botswana	2000	Mutton & Goat Meat	12
20	Botswana	2000	Nuts	1
20	Botswana	2000	Offals, Edible	8
20	Botswana	2000	Oilcrops Oil, Other	4
20	Botswana	2000	Onions	23
20	Botswana	2000	Oranges, Mandarines	57
20	Botswana	2000	Peas	1
20	Botswana	2000	Pelagic Fish	9
20	Botswana	2000	Pepper	1
20	Botswana	2000	Pigmeat	6
20	Botswana	2000	Pineapples	1
20	Botswana	2000	Potatoes	35
20	Botswana	2000	Poultry Meat	15
20	Botswana	2000	Pulses, Other	26
20	Botswana	2000	Rice (Milled Equivalent)	16
20	Botswana	2000	Roots, Other	117
20	Botswana	2000	Rye	1
20	Botswana	2000	Sorghum	65
20	Botswana	2000	Soyabean Oil	2
20	Botswana	2000	Soyabeans	1
20	Botswana	2000	Spices, Other	2
20	Botswana	2000	Sugar, Refined Equivalent	50
20	Botswana	2000	Sunflowerseed Oil	9
20	Botswana	2000	Sweet Potatoes	1
20	Botswana	2000	Sweeteners, Other	11
20	Botswana	2000	Tea	3
20	Botswana	2000	Tomatoes	37
20	Botswana	2000	Vegetables, Other	65
20	Botswana	2000	Wheat	88
20	Botswana	2000	Wine	5
20	Botswana	2001	Apples	14
20	Botswana	2001	Bananas	15
20	Botswana	2001	Beans	7
20	Botswana	2001	Beer	79
20	Botswana	2001	Beverages, Alcoholic	2
20	Botswana	2001	Beverages, Fermented	73
20	Botswana	2001	Bovine Meat	10
20	Botswana	2001	Butter, Ghee	2
20	Botswana	2001	Cereals, Other	2
20	Botswana	2001	Cheese	10
20	Botswana	2001	Citrus, Other	1
20	Botswana	2001	Cocoa Beans	1
20	Botswana	2001	Coconuts - Incl Copra	1
20	Botswana	2001	Coffee	2
20	Botswana	2001	Cottonseed Oil	4
20	Botswana	2001	Cream	1
20	Botswana	2001	Crustaceans	2
20	Botswana	2001	Eggs	7
20	Botswana	2001	Fats, Animals, Raw	2
20	Botswana	2001	Freshwater Fish	1
20	Botswana	2001	Fruits, Other	65
20	Botswana	2001	Grapes	1
20	Botswana	2001	Groundnuts (Shelled Eq)	1
20	Botswana	2001	Maize	118
20	Botswana	2001	Marine Fish, Other	1
20	Botswana	2001	Meat, Other	18
20	Botswana	2001	Milk, Whole	289
20	Botswana	2001	Mutton & Goat Meat	12
20	Botswana	2001	Nuts	2
20	Botswana	2001	Offals, Edible	8
20	Botswana	2001	Oilcrops Oil, Other	2
20	Botswana	2001	Onions	9
20	Botswana	2001	Oranges, Mandarines	30
20	Botswana	2001	Peas	3
20	Botswana	2001	Pelagic Fish	7
20	Botswana	2001	Pepper	1
20	Botswana	2001	Pigmeat	2
20	Botswana	2001	Potatoes	35
20	Botswana	2001	Poultry Meat	15
20	Botswana	2001	Pulses, Other	28
20	Botswana	2001	Rice (Milled Equivalent)	29
20	Botswana	2001	Roots, Other	124
20	Botswana	2001	Sorghum	70
20	Botswana	2001	Soyabean Oil	1
20	Botswana	2001	Soyabeans	3
20	Botswana	2001	Spices, Other	2
20	Botswana	2001	Sugar, Refined Equivalent	54
20	Botswana	2001	Sunflowerseed Oil	11
20	Botswana	2001	Sweet Potatoes	1
20	Botswana	2001	Sweeteners, Other	9
20	Botswana	2001	Tea	2

FAO country code	Country	Reference year	Food item	Gross food supply (g *capita*$^{-1}$ d^{-1})
20	Botswana	2001	Tomatoes	12
20	Botswana	2001	Vegetables, Other	73
20	Botswana	2001	Wheat	85
20	Botswana	2001	Wine	3
20	Botswana	2002	Apples	16
20	Botswana	2002	Bananas	12
20	Botswana	2002	Beans	6
20	Botswana	2002	Beer	88
20	Botswana	2002	Beverages, Alcoholic	5
20	Botswana	2002	Beverages, Fermented	68
20	Botswana	2002	Bovine Meat	18
20	Botswana	2002	Butter, Ghee	2
20	Botswana	2002	Cereals, Other	5
20	Botswana	2002	Cheese	12
20	Botswana	2002	Citrus, Other	3
20	Botswana	2002	Cocoa Beans	1
20	Botswana	2002	Coconuts - Incl Copra	2
20	Botswana	2002	Coffee	6
20	Botswana	2002	Cottonseed Oil	3
20	Botswana	2002	Cream	3
20	Botswana	2002	Crustaceans	1
20	Botswana	2002	Dates	1
20	Botswana	2002	Eggs	7
20	Botswana	2002	Fats, Animals, Raw	2
20	Botswana	2002	Freshwater Fish	1
20	Botswana	2002	Fruits, Other	113
20	Botswana	2002	Grapes	3
20	Botswana	2002	Groundnuts (Shelled Eq)	1
20	Botswana	2002	Maize	94
20	Botswana	2002	Meat, Other	18
20	Botswana	2002	Milk, Whole	226
20	Botswana	2002	Molluscs, Other	1
20	Botswana	2002	Mutton & Goat Meat	11
20	Botswana	2002	Nuts	3
20	Botswana	2002	Offals, Edible	8
20	Botswana	2002	Oilcrops Oil, Other	2
20	Botswana	2002	Oilcrops, Other	1
20	Botswana	2002	Onions	9
20	Botswana	2002	Oranges, Mandarines	27
20	Botswana	2002	Peas	1
20	Botswana	2002	Pelagic Fish	5
20	Botswana	2002	Pepper	1
20	Botswana	2002	Pigmeat	2
20	Botswana	2002	Pineapples	2
20	Botswana	2002	Potatoes	43
20	Botswana	2002	Poultry Meat	14
20	Botswana	2002	Pulses, Other	28
20	Botswana	2002	Rice (Milled Equivalent)	21
20	Botswana	2002	Roots, Other	124
20	Botswana	2002	Rye	4
20	Botswana	2002	Sorghum	70
20	Botswana	2002	Soyabean Oil	1
20	Botswana	2002	Soyabeans	3
20	Botswana	2002	Spices, Other	4
20	Botswana	2002	Sugar, Refined Equivalent	48
20	Botswana	2002	Sunflowerseed Oil	9
20	Botswana	2002	Sweet Potatoes	2
20	Botswana	2002	Sweeteners, Other	15
20	Botswana	2002	Tea	4
20	Botswana	2002	Tomatoes	13
20	Botswana	2002	Vegetables, Other	94
20	Botswana	2002	Wheat	90
20	Botswana	2002	Wine	7
20	Botswana	2003	Apples	10
20	Botswana	2003	Bananas	11
20	Botswana	2003	Beans	6
20	Botswana	2003	Beer	87
20	Botswana	2003	Beverages, Alcoholic	3
20	Botswana	2003	Beverages, Fermented	66
20	Botswana	2003	Bovine Meat	15
20	Botswana	2003	Butter, Ghee	2
20	Botswana	2003	Cereals, Other	6
20	Botswana	2003	Cheese	6
20	Botswana	2003	Citrus, Other	1
20	Botswana	2003	Cocoa Beans	1
20	Botswana	2003	Coconuts - Incl Copra	1
20	Botswana	2003	Coffee	3
20	Botswana	2003	Cottonseed Oil	2
20	Botswana	2003	Cream	2
20	Botswana	2003	Eggs	8
20	Botswana	2003	Fats, Animals, Raw	2
20	Botswana	2003	Fruits, Other	71
20	Botswana	2003	Grapefruit	1
20	Botswana	2003	Grapes	2
20	Botswana	2003	Groundnuts (Shelled Eq)	1
20	Botswana	2003	Maize	98
20	Botswana	2003	Meat, Other	20
20	Botswana	2003	Milk, Whole	175
20	Botswana	2003	Mutton & Goat Meat	8
20	Botswana	2003	Nuts	3
20	Botswana	2003	Offals, Edible	7
20	Botswana	2003	Oilcrops Oil, Other	1
20	Botswana	2003	Onions	7
20	Botswana	2003	Oranges, Mandarines	26
20	Botswana	2003	Peas	1
20	Botswana	2003	Pelagic Fish	6
20	Botswana	2003	Pepper	1

FAO country code	Country	Reference year	Food item	Gross food supply (g *capita*$^{-1}$ d^{-1})
20	Botswana	2003	Pigmeat	1
20	Botswana	2003	Potatoes	37
20	Botswana	2003	Poultry Meat	9
20	Botswana	2003	Pulses, Other	31
20	Botswana	2003	Rice (Milled Equivalent)	28
20	Botswana	2003	Roots, Other	111
20	Botswana	2003	Rye	1
20	Botswana	2003	Sorghum	69
20	Botswana	2003	Soyabean Oil	2
20	Botswana	2003	Soyabeans	3
20	Botswana	2003	Spices, Other	2
20	Botswana	2003	Sugar, Refined Equivalent	66
20	Botswana	2003	Sunflowerseed Oil	15
20	Botswana	2003	Sweet Potatoes	1
20	Botswana	2003	Sweeteners, Other	9
20	Botswana	2003	Tea	1
20	Botswana	2003	Tomatoes	10
20	Botswana	2003	Vegetables, Other	75
20	Botswana	2003	Wheat	85
20	Botswana	2003	Wine	6
20	Botswana	2004	Apples	11
20	Botswana	2004	Bananas	10
20	Botswana	2004	Beans	5
20	Botswana	2004	Beer	84
20	Botswana	2004	Beverages, Alcoholic	4
20	Botswana	2004	Beverages, Fermented	57
20	Botswana	2004	Bovine Meat	30
20	Botswana	2004	Butter, Ghee	2
20	Botswana	2004	Cereals, Other	5
20	Botswana	2004	Cheese	6
20	Botswana	2004	Citrus, Other	1
20	Botswana	2004	Cocoa Beans	1
20	Botswana	2004	Coconuts - Incl Copra	1
20	Botswana	2004	Coffee	2
20	Botswana	2004	Cottonseed Oil	2
20	Botswana	2004	Cream	3
20	Botswana	2004	Eggs	6
20	Botswana	2004	Fats, Animals, Raw	2
20	Botswana	2004	Fruits, Other	76
20	Botswana	2004	Grapefruit	1
20	Botswana	2004	Grapes	2
20	Botswana	2004	Maize	114
20	Botswana	2004	Meat, Other	21
20	Botswana	2004	Milk, Whole	194
20	Botswana	2004	Mutton & Goat Meat	10
20	Botswana	2004	Nuts	3
20	Botswana	2004	Offals, Edible	8
20	Botswana	2004	Oilcrops Oil, Other	5
20	Botswana	2004	Onions	7
20	Botswana	2004	Oranges, Mandarines	4
20	Botswana	2004	Peas	1
20	Botswana	2004	Pelagic Fish	6
20	Botswana	2004	Pigmeat	1
20	Botswana	2004	Potatoes	39
20	Botswana	2004	Poultry Meat	10
20	Botswana	2004	Pulses, Other	34
20	Botswana	2004	Rice (Milled Equivalent)	30
20	Botswana	2004	Roots, Other	132
20	Botswana	2004	Sorghum	57
20	Botswana	2004	Soyabean Oil	1
20	Botswana	2004	Spices, Other	2
20	Botswana	2004	Sugar, Refined Equivalent	65
20	Botswana	2004	Sunflowerseed Oil	18
20	Botswana	2004	Sweet Potatoes	1
20	Botswana	2004	Sweeteners, Other	7
20	Botswana	2004	Tomatoes	11
20	Botswana	2004	Vegetables, Other	54
20	Botswana	2004	Wheat	83
20	Botswana	2004	Wine	9
20	Botswana	2005	Apples	12
20	Botswana	2005	Bananas	9
20	Botswana	2005	Beans	4
20	Botswana	2005	Beer	78
20	Botswana	2005	Beverages, Alcoholic	4
20	Botswana	2005	Beverages, Fermented	64
20	Botswana	2005	Bovine Meat	35
20	Botswana	2005	Butter, Ghee	2
20	Botswana	2005	Cereals, Other	6
20	Botswana	2005	Cheese	5
20	Botswana	2005	Cocoa Beans	1
20	Botswana	2005	Coconuts - Incl Copra	1
20	Botswana	2005	Coffee	2
20	Botswana	2005	Cottonseed Oil	2
20	Botswana	2005	Cream	5
20	Botswana	2005	Demersal Fish	2
20	Botswana	2005	Eggs	6
20	Botswana	2005	Fats, Animals, Raw	2
20	Botswana	2005	Fruits, Other	81
20	Botswana	2005	Grapefruit	1
20	Botswana	2005	Grapes	2
20	Botswana	2005	Maize	104
20	Botswana	2005	Meat, Other	23
20	Botswana	2005	Milk, Whole	197
20	Botswana	2005	Mutton & Goat Meat	11
20	Botswana	2005	Offals, Edible	9
20	Botswana	2005	Oilcrops Oil, Other	8
20	Botswana	2005	Onions	7

FAO country code	Country	Reference year	Food item	Gross food supply (g *capita*$^{-1}$ d^{-1})
20	Botswana	2005	Oranges, Mandarines	3
20	Botswana	2005	Peas	1
20	Botswana	2005	Pelagic Fish	6
20	Botswana	2005	Pigmeat	1
20	Botswana	2005	Potatoes	38
20	Botswana	2005	Poultry Meat	8
20	Botswana	2005	Pulses, Other	20
20	Botswana	2005	Rice (Milled Equivalent)	32
20	Botswana	2005	Roots, Other	128
20	Botswana	2005	Sorghum	63
20	Botswana	2005	Soyabean Oil	2
20	Botswana	2005	Spices, Other	2
20	Botswana	2005	Sugar, Refined Equivalent	67
20	Botswana	2005	Sunflowerseed Oil	19
20	Botswana	2005	Sweeteners, Other	6
20	Botswana	2005	Tomatoes	12
20	Botswana	2005	Vegetables, Other	47
20	Botswana	2005	Wheat	86
20	Botswana	2005	Wine	14
20	Botswana	2006	Apples	12
20	Botswana	2006	Bananas	9
20	Botswana	2006	Beans	4
20	Botswana	2006	Beer	80
20	Botswana	2006	Beverages, Alcoholic	5
20	Botswana	2006	Beverages, Fermented	71
20	Botswana	2006	Bovine Meat	30
20	Botswana	2006	Butter, Ghee	2
20	Botswana	2006	Cereals, Other	5
20	Botswana	2006	Cheese	4
20	Botswana	2006	Cocoa Beans	1
20	Botswana	2006	Coconuts - Incl Copra	1
20	Botswana	2006	Coffee	2
20	Botswana	2006	Cottonseed Oil	3
20	Botswana	2006	Cream	9
20	Botswana	2006	Demersal Fish	1
20	Botswana	2006	Eggs	6
20	Botswana	2006	Fats, Animals, Raw	2
20	Botswana	2006	Fruits, Other	86
20	Botswana	2006	Grapes	2
20	Botswana	2006	Maize	103
20	Botswana	2006	Meat, Other	19
20	Botswana	2006	Milk, Whole	193
20	Botswana	2006	Millet	1
20	Botswana	2006	Mutton & Goat Meat	11
20	Botswana	2006	Offals, Edible	9
20	Botswana	2006	Oilcrops Oil, Other	2
20	Botswana	2006	Onions	7
20	Botswana	2006	Oranges, Mandarines	8
20	Botswana	2006	Palm Oil	1
20	Botswana	2006	Peas	1
20	Botswana	2006	Pelagic Fish	5
20	Botswana	2006	Pigmeat	1
20	Botswana	2006	Potatoes	39
20	Botswana	2006	Poultry Meat	9
20	Botswana	2006	Pulses, Other	10
20	Botswana	2006	Rice (Milled Equivalent)	35
20	Botswana	2006	Roots, Other	119
20	Botswana	2006	Sorghum	69
20	Botswana	2006	Spices, Other	2
20	Botswana	2006	Sugar, Refined Equivalent	68
20	Botswana	2006	Sunflowerseed Oil	20
20	Botswana	2006	Sweeteners, Other	8
20	Botswana	2006	Tomatoes	15
20	Botswana	2006	Vegetables, Other	50
20	Botswana	2006	Wheat	95
20	Botswana	2006	Wine	21
20	Botswana	2007	Apples	13
20	Botswana	2007	Bananas	8
20	Botswana	2007	Beans	4
20	Botswana	2007	Beer	82
20	Botswana	2007	Beverages, Alcoholic	2
20	Botswana	2007	Beverages, Fermented	68
20	Botswana	2007	Bovine Meat	20
20	Botswana	2007	Butter, Ghee	2
20	Botswana	2007	Cereals, Other	8
20	Botswana	2007	Cheese	5
20	Botswana	2007	Citrus, Other	1
20	Botswana	2007	Cocoa Beans	1
20	Botswana	2007	Coffee	1
20	Botswana	2007	Cottonseed Oil	3
20	Botswana	2007	Cream	12
20	Botswana	2007	Demersal Fish	1
20	Botswana	2007	Eggs	7
20	Botswana	2007	Fats, Animals, Raw	2
20	Botswana	2007	Fruits, Other	87
20	Botswana	2007	Grapes	3
20	Botswana	2007	Groundnuts (Shelled Eq)	1
20	Botswana	2007	Maize	104
20	Botswana	2007	Marine Fish, Other	1
20	Botswana	2007	Meat, Other	26
20	Botswana	2007	Milk, Whole	182
20	Botswana	2007	Millet	1
20	Botswana	2007	Mutton & Goat Meat	11
20	Botswana	2007	Nuts	2
20	Botswana	2007	Offals, Edible	9
20	Botswana	2007	Oilcrops Oil, Other	2
20	Botswana	2007	Onions	7

FAO country code	Country	Reference year	Food item	Gross food supply (g $capita^{-1}$ d^{-1})
20	Botswana	2007	Oranges, Mandarines	24
20	Botswana	2007	Palm Oil	1
20	Botswana	2007	Peas	1
20	Botswana	2007	Pelagic Fish	6
20	Botswana	2007	Pepper	2
20	Botswana	2007	Pigmeat	1
20	Botswana	2007	Potatoes	37
20	Botswana	2007	Poultry Meat	10
20	Botswana	2007	Pulses, Other	1
20	Botswana	2007	Rice (Milled Equivalent)	39
20	Botswana	2007	Roots, Other	118
20	Botswana	2007	Sorghum	65
20	Botswana	2007	Soyabean Oil	1
20	Botswana	2007	Soyabeans	2
20	Botswana	2007	Spices, Other	2
20	Botswana	2007	Sugar, Refined Equivalent	67
20	Botswana	2007	Sunflowerseed Oil	20
20	Botswana	2007	Sweeteners, Other	12
20	Botswana	2007	Tea	4
20	Botswana	2007	Tomatoes	17
20	Botswana	2007	Vegetables, Other	78
20	Botswana	2007	Wheat	85
20	Botswana	2007	Wine	27
20	Botswana	2008	Apples	13
20	Botswana	2008	Bananas	10
20	Botswana	2008	Beans	7
20	Botswana	2008	Beer	93
20	Botswana	2008	Beverages, Alcoholic	10
20	Botswana	2008	Beverages, Fermented	78
20	Botswana	2008	Bovine Meat	19
20	Botswana	2008	Butter, Ghee	2
20	Botswana	2008	Cereals, Other	10
20	Botswana	2008	Cheese	5
20	Botswana	2008	Citrus, Other	2
20	Botswana	2008	Cocoa Beans	1
20	Botswana	2008	Coffee	3
20	Botswana	2008	Cottonseed Oil	4
20	Botswana	2008	Cream	3
20	Botswana	2008	Demersal Fish	1
20	Botswana	2008	Eggs	6
20	Botswana	2008	Fats, Animals, Raw	2
20	Botswana	2008	Fruits, Other	94
20	Botswana	2008	Grapes	2
20	Botswana	2008	Groundnuts (Shelled Eq)	1
20	Botswana	2008	Maize	97
20	Botswana	2008	Marine Fish, Other	1
20	Botswana	2008	Meat, Other	30
20	Botswana	2008	Milk, Whole	174
20	Botswana	2008	Millet	2
20	Botswana	2008	Mutton & Goat Meat	11
20	Botswana	2008	Nuts	3
20	Botswana	2008	Offals, Edible	9
20	Botswana	2008	Oilcrops Oil, Other	2
20	Botswana	2008	Onions	6
20	Botswana	2008	Oranges, Mandarines	35
20	Botswana	2008	Palm Oil	1
20	Botswana	2008	Peas	1
20	Botswana	2008	Pelagic Fish	6
20	Botswana	2008	Pepper	3
20	Botswana	2008	Pigmeat	2
20	Botswana	2008	Potatoes	38
20	Botswana	2008	Poultry Meat	13
20	Botswana	2008	Pulses, Other	3
20	Botswana	2008	Rice (Milled Equivalent)	36
20	Botswana	2008	Roots, Other	112
20	Botswana	2008	Sorghum	58
20	Botswana	2008	Soyabeans	3
20	Botswana	2008	Spices, Other	3
20	Botswana	2008	Sugar, Refined Equivalent	65
20	Botswana	2008	Sunflowerseed Oil	17
20	Botswana	2008	Sweeteners, Other	8
20	Botswana	2008	Tea	3
20	Botswana	2008	Tomatoes	9
20	Botswana	2008	Vegetables, Other	77
20	Botswana	2008	Wheat	95
20	Botswana	2008	Wine	16
20	Botswana	2009	Apples	14
20	Botswana	2009	Bananas	11
20	Botswana	2009	Beans	8
20	Botswana	2009	Beer	102
20	Botswana	2009	Beverages, Alcoholic	3
20	Botswana	2009	Beverages, Fermented	63
20	Botswana	2009	Bovine Meat	23
20	Botswana	2009	Butter, Ghee	3
20	Botswana	2009	Cereals, Other	5
20	Botswana	2009	Cheese	5
20	Botswana	2009	Citrus, Other	3
20	Botswana	2009	Cocoa Beans	1
20	Botswana	2009	Coffee	2
20	Botswana	2009	Cottonseed Oil	2
20	Botswana	2009	Cream	2
20	Botswana	2009	Demersal Fish	3
20	Botswana	2009	Eggs	5
20	Botswana	2009	Fats, Animals, Raw	2
20	Botswana	2009	Fruits, Other	76
20	Botswana	2009	Grapes	2
20	Botswana	2009	Groundnut Oil	1

FAO country code	Country	Reference year	Food item	Gross food supply (g $capita^{-1}$ d^{-1})
20	Botswana	2009	Groundnuts (Shelled Eq)	1
20	Botswana	2009	Maize	104
20	Botswana	2009	Meat, Other	31
20	Botswana	2009	Milk, Whole	187
20	Botswana	2009	Millet	3
20	Botswana	2009	Mutton & Goat Meat	11
20	Botswana	2009	Nuts	2
20	Botswana	2009	Offals, Edible	10
20	Botswana	2009	Oilcrops Oil, Other	2
20	Botswana	2009	Onions	7
20	Botswana	2009	Oranges, Mandarines	24
20	Botswana	2009	Palm Oil	1
20	Botswana	2009	Peas	1
20	Botswana	2009	Pelagic Fish	6
20	Botswana	2009	Pepper	3
20	Botswana	2009	Pigmeat	2
20	Botswana	2009	Pineapples	1
20	Botswana	2009	Potatoes	15
20	Botswana	2009	Poultry Meat	13
20	Botswana	2009	Pulses, Other	5
20	Botswana	2009	Rice (Milled Equivalent)	27
20	Botswana	2009	Roots, Other	103
20	Botswana	2009	Sorghum	60
20	Botswana	2009	Soyabeans	10
20	Botswana	2009	Spices, Other	3
20	Botswana	2009	Sugar, Refined Equivalent	66
20	Botswana	2009	Sunflowerseed Oil	20
20	Botswana	2009	Sweeteners, Other	11
20	Botswana	2009	Tea	3
20	Botswana	2009	Tomatoes	8
20	Botswana	2009	Vegetables, Other	69
20	Botswana	2009	Wheat	96
20	Botswana	2009	Wine	10
20	Botswana	2010	Apples	13
20	Botswana	2010	Bananas	13
20	Botswana	2010	Beans	9
20	Botswana	2010	Beer	80
20	Botswana	2010	Beverages, Alcoholic	3
20	Botswana	2010	Beverages, Fermented	73
20	Botswana	2010	Bovine Meat	20
20	Botswana	2010	Butter, Ghee	2
20	Botswana	2010	Cereals, Other	16
20	Botswana	2010	Cheese	6
20	Botswana	2010	Citrus, Other	2
20	Botswana	2010	Cocoa Beans	1
20	Botswana	2010	Coffee	2
20	Botswana	2010	Cottonseed Oil	3
20	Botswana	2010	Cream	1
20	Botswana	2010	Demersal Fish	1
20	Botswana	2010	Eggs	6
20	Botswana	2010	Fats, Animals, Raw	3
20	Botswana	2010	Fruits, Other	81
20	Botswana	2010	Grapefruit	1
20	Botswana	2010	Grapes	2
20	Botswana	2010	Groundnut Oil	1
20	Botswana	2010	Groundnuts (Shelled Eq)	1
20	Botswana	2010	Maize	95
20	Botswana	2010	Marine Fish, Other	1
20	Botswana	2010	Meat, Other	35
20	Botswana	2010	Milk, Whole	184
20	Botswana	2010	Millet	4
20	Botswana	2010	Mutton & Goat Meat	11
20	Botswana	2010	Nuts	2
20	Botswana	2010	Offals, Edible	11
20	Botswana	2010	Oilcrops Oil, Other	1
20	Botswana	2010	Onions	7
20	Botswana	2010	Oranges, Mandarines	27
20	Botswana	2010	Palm Oil	1
20	Botswana	2010	Pelagic Fish	8
20	Botswana	2010	Pepper	3
20	Botswana	2010	Pigmeat	2
20	Botswana	2010	Pineapples	1
20	Botswana	2010	Potatoes	25
20	Botswana	2010	Poultry Meat	15
20	Botswana	2010	Pulses, Other	4
20	Botswana	2010	Rice (Milled Equivalent)	30
20	Botswana	2010	Roots, Other	114
20	Botswana	2010	Sorghum	67
20	Botswana	2010	Soyabeans	6
20	Botswana	2010	Spices, Other	4
20	Botswana	2010	Sugar, Refined Equivalent	65
20	Botswana	2010	Sunflowerseed Oil	18
20	Botswana	2010	Sweet Potatoes	1
20	Botswana	2010	Sweeteners, Other	22
20	Botswana	2010	Tea	3
20	Botswana	2010	Tomatoes	7
20	Botswana	2010	Vegetables, Other	90
20	Botswana	2010	Wheat	98
20	Botswana	2010	Wine	11
20	Botswana	2011	Apples	10
20	Botswana	2011	Bananas	9
20	Botswana	2011	Beans	9
20	Botswana	2011	Beer	86
20	Botswana	2011	Beverages, Alcoholic	7
20	Botswana	2011	Beverages, Fermented	79
20	Botswana	2011	Bovine Meat	22
20	Botswana	2011	Butter, Ghee	2

FAO country code	Country	Reference year	Food item	Gross food supply (g *capita*$^{-1}$ d^{-1})
20	Botswana	2011	Cereals, Other	17
20	Botswana	2011	Cheese	5
20	Botswana	2011	Citrus, Other	2
20	Botswana	2011	Cocoa Beans	2
20	Botswana	2011	Coffee	3
20	Botswana	2011	Cottonseed Oil	2
20	Botswana	2011	Demersal Fish	1
20	Botswana	2011	Eggs	5
20	Botswana	2011	Fats, Animals, Raw	3
20	Botswana	2011	Freshwater Fish	1
20	Botswana	2011	Fruits, Other	80
20	Botswana	2011	Grapes	2
20	Botswana	2011	Groundnut Oil	2
20	Botswana	2011	Groundnuts (Shelled Eq)	2
20	Botswana	2011	Maize	98
20	Botswana	2011	Marine Fish, Other	1
20	Botswana	2011	Meat, Other	33
20	Botswana	2011	Milk, Whole	222
20	Botswana	2011	Millet	2
20	Botswana	2011	Mutton & Goat Meat	11
20	Botswana	2011	Nuts	1
20	Botswana	2011	Oats	2
20	Botswana	2011	Offals, Edible	11
20	Botswana	2011	Oilcrops Oil, Other	2
20	Botswana	2011	Olive Oil	1
20	Botswana	2011	Onions	6
20	Botswana	2011	Oranges, Mandarines	26
20	Botswana	2011	Palm Oil	1
20	Botswana	2011	Pelagic Fish	6
20	Botswana	2011	Pepper	2
20	Botswana	2011	Pigmeat	2
20	Botswana	2011	Pineapples	1
20	Botswana	2011	Potatoes	26
20	Botswana	2011	Poultry Meat	14
20	Botswana	2011	Pulses, Other	4
20	Botswana	2011	Rice (Milled Equivalent)	30
20	Botswana	2011	Roots, Other	118
20	Botswana	2011	Rye	2
20	Botswana	2011	Sesameseed Oil	1
20	Botswana	2011	Sorghum	66
20	Botswana	2011	Soyabeans	4
20	Botswana	2011	Spices, Other	4
20	Botswana	2011	Sugar, Refined Equivalent	65
20	Botswana	2011	Sunflowerseed Oil	20
20	Botswana	2011	Sweet Potatoes	1
20	Botswana	2011	Sweeteners, Other	11
20	Botswana	2011	Tea	3
20	Botswana	2011	Tomatoes	5
20	Botswana	2011	Vegetables, Other	90
20	Botswana	2011	Wheat	98
20	Botswana	2011	Wine	9
21	Brazil	1992	Apples	10
21	Brazil	1992	Bananas	86
21	Brazil	1992	Beans	43
21	Brazil	1992	Beer	76
21	Brazil	1992	Beverages, Alcoholic	10
21	Brazil	1992	Bovine Meat	78
21	Brazil	1992	Butter, Ghee	1
21	Brazil	1992	Cassava	128
21	Brazil	1992	Cereals, Other	1
21	Brazil	1992	Cheese	1
21	Brazil	1992	Cocoa Beans	1
21	Brazil	1992	Coconuts - Incl Copra	9
21	Brazil	1992	Coffee	3
21	Brazil	1992	Cottonseed Oil	2
21	Brazil	1992	Crustaceans	1
21	Brazil	1992	Demersal Fish	4
21	Brazil	1992	Eggs	20
21	Brazil	1992	Fats, Animals, Raw	5
21	Brazil	1992	Freshwater Fish	3
21	Brazil	1992	Fruits, Other	50
21	Brazil	1992	Grapes	4
21	Brazil	1992	Groundnuts (Shelled Eq)	1
21	Brazil	1992	Lemons, Limes	7
21	Brazil	1992	Maize	60
21	Brazil	1992	Maize Germ Oil	1
21	Brazil	1992	Marine Fish, Other	2
21	Brazil	1992	Milk, Whole	239
21	Brazil	1992	Mutton & Goat Meat	2
21	Brazil	1992	Nuts	1
21	Brazil	1992	Oats	4
21	Brazil	1992	Offals, Edible	5
21	Brazil	1992	Olives	1
21	Brazil	1992	Onions	14
21	Brazil	1992	Oranges, Mandarines	106
21	Brazil	1992	Palm Oil	1
21	Brazil	1992	Pelagic Fish	2
21	Brazil	1992	Pigmeat	22
21	Brazil	1992	Pineapples	17
21	Brazil	1992	Potatoes	35
21	Brazil	1992	Poultry Meat	45
21	Brazil	1992	Rice (Milled Equivalent)	106
21	Brazil	1992	Soyabean Oil	36
21	Brazil	1992	Soyabeans	5
21	Brazil	1992	Sugar Cane	15
21	Brazil	1992	Sugar, Non-Centrifugal	4
21	Brazil	1992	Sugar, Refined Equivalent	122

FAO country code	Country	Reference year	Food item	Gross food supply (g *capita*$^{-1}$ d^{-1})
21	Brazil	1992	Sweet Potatoes	4
21	Brazil	1992	Tea	2
21	Brazil	1992	Tomatoes	35
21	Brazil	1992	Vegetables, Other	40
21	Brazil	1992	Wheat	123
21	Brazil	1992	Wine	6
21	Brazil	1992	Yams	2
21	Brazil	1993	Apples	11
21	Brazil	1993	Bananas	85
21	Brazil	1993	Beans	46
21	Brazil	1993	Beer	78
21	Brazil	1993	Beverages, Alcoholic	10
21	Brazil	1993	Bovine Meat	77
21	Brazil	1993	Butter, Ghee	1
21	Brazil	1993	Cassava	127
21	Brazil	1993	Cereals, Other	1
21	Brazil	1993	Cheese	1
21	Brazil	1993	Cocoa Beans	1
21	Brazil	1993	Coconuts - Incl Copra	9
21	Brazil	1993	Coffee	3
21	Brazil	1993	Cottonseed Oil	2
21	Brazil	1993	Crustaceans	1
21	Brazil	1993	Demersal Fish	5
21	Brazil	1993	Eggs	20
21	Brazil	1993	Fats, Animals, Raw	5
21	Brazil	1993	Freshwater Fish	3
21	Brazil	1993	Fruits, Other	52
21	Brazil	1993	Grapes	6
21	Brazil	1993	Groundnuts (Shelled Eq)	1
21	Brazil	1993	Lemons, Limes	6
21	Brazil	1993	Maize	60
21	Brazil	1993	Maize Germ Oil	1
21	Brazil	1993	Marine Fish, Other	3
21	Brazil	1993	Milk, Whole	232
21	Brazil	1993	Mutton & Goat Meat	2
21	Brazil	1993	Nuts	1
21	Brazil	1993	Oats	4
21	Brazil	1993	Offals, Edible	5
21	Brazil	1993	Olives	1
21	Brazil	1993	Onions	15
21	Brazil	1993	Oranges, Mandarines	49
21	Brazil	1993	Palm Oil	2
21	Brazil	1993	Pelagic Fish	2
21	Brazil	1993	Pigmeat	25
21	Brazil	1993	Pineapples	17
21	Brazil	1993	Potatoes	33
21	Brazil	1993	Poultry Meat	47
21	Brazil	1993	Rice (Milled Equivalent)	114
21	Brazil	1993	Soyabean Oil	35
21	Brazil	1993	Soyabeans	6
21	Brazil	1993	Sugar Cane	14
21	Brazil	1993	Sugar, Non-Centrifugal	4
21	Brazil	1993	Sugar, Refined Equivalent	123
21	Brazil	1993	Sweet Potatoes	4
21	Brazil	1993	Tea	3
21	Brazil	1993	Tomatoes	37
21	Brazil	1993	Vegetables, Other	42
21	Brazil	1993	Wheat	119
21	Brazil	1993	Wine	4
21	Brazil	1993	Yams	2
21	Brazil	1994	Apples	12
21	Brazil	1994	Bananas	86
21	Brazil	1994	Beans	48
21	Brazil	1994	Beer	89
21	Brazil	1994	Beverages, Alcoholic	10
21	Brazil	1994	Bovine Meat	83
21	Brazil	1994	Butter, Ghee	1
21	Brazil	1994	Cassava	138
21	Brazil	1994	Cereals, Other	1
21	Brazil	1994	Cheese	1
21	Brazil	1994	Cocoa Beans	2
21	Brazil	1994	Coconuts - Incl Copra	10
21	Brazil	1994	Coffee	3
21	Brazil	1994	Cottonseed Oil	2
21	Brazil	1994	Crustaceans	1
21	Brazil	1994	Demersal Fish	5
21	Brazil	1994	Eggs	20
21	Brazil	1994	Fats, Animals, Raw	5
21	Brazil	1994	Freshwater Fish	3
21	Brazil	1994	Fruits, Other	52
21	Brazil	1994	Grapefruit	1
21	Brazil	1994	Grapes	6
21	Brazil	1994	Groundnuts (Shelled Eq)	1
21	Brazil	1994	Lemons, Limes	6
21	Brazil	1994	Maize	56
21	Brazil	1994	Maize Germ Oil	1
21	Brazil	1994	Marine Fish, Other	3
21	Brazil	1994	Milk, Whole	240
21	Brazil	1994	Mutton & Goat Meat	2
21	Brazil	1994	Nuts	1
21	Brazil	1994	Oats	4
21	Brazil	1994	Offals, Edible	6
21	Brazil	1994	Olives	1
21	Brazil	1994	Onions	16
21	Brazil	1994	Oranges, Mandarines	31
21	Brazil	1994	Palm Oil	2
21	Brazil	1994	Pelagic Fish	2

FAO country code	Country	Reference year	Food item	Gross food supply (g $capita^{-1}$ d^{-1})
21	Brazil	1994	Pigmeat	29
21	Brazil	1994	Pineapples	20
21	Brazil	1994	Potatoes	37
21	Brazil	1994	Poultry Meat	52
21	Brazil	1994	Rice (Milled Equivalent)	108
21	Brazil	1994	Soyabean Oil	33
21	Brazil	1994	Soyabeans	6
21	Brazil	1994	Sugar Cane	15
21	Brazil	1994	Sugar, Non-Centrifugal	4
21	Brazil	1994	Sugar, Refined Equivalent	126
21	Brazil	1994	Sunflowerseed Oil	1
21	Brazil	1994	Sweet Potatoes	5
21	Brazil	1994	Tea	3
21	Brazil	1994	Tomatoes	42
21	Brazil	1994	Vegetables, Other	43
21	Brazil	1994	Wheat	126
21	Brazil	1994	Wine	5
21	Brazil	1994	Yams	2
21	Brazil	1995	Apples	14
21	Brazil	1995	Bananas	83
21	Brazil	1995	Beans	36
21	Brazil	1995	Beer	116
21	Brazil	1995	Beverages, Alcoholic	11
21	Brazil	1995	Bovine Meat	94
21	Brazil	1995	Butter, Ghee	1
21	Brazil	1995	Cassava	142
21	Brazil	1995	Cereals, Other	1
21	Brazil	1995	Cheese	2
21	Brazil	1995	Cocoa Beans	2
21	Brazil	1995	Coconuts - Incl Copra	11
21	Brazil	1995	Coffee	3
21	Brazil	1995	Cottonseed Oil	1
21	Brazil	1995	Crustaceans	1
21	Brazil	1995	Demersal Fish	7
21	Brazil	1995	Eggs	20
21	Brazil	1995	Fats, Animals, Raw	5
21	Brazil	1995	Freshwater Fish	4
21	Brazil	1995	Fruits, Other	57
21	Brazil	1995	Grapefruit	1
21	Brazil	1995	Grapes	8
21	Brazil	1995	Groundnuts (Shelled Eq)	1
21	Brazil	1995	Lemons, Limes	6
21	Brazil	1995	Maize	52
21	Brazil	1995	Maize Germ Oil	1
21	Brazil	1995	Marine Fish, Other	2
21	Brazil	1995	Milk, Whole	278
21	Brazil	1995	Mutton & Goat Meat	2
21	Brazil	1995	Nuts	1
21	Brazil	1995	Oats	3
21	Brazil	1995	Offals, Edible	6
21	Brazil	1995	Olives	1
21	Brazil	1995	Onions	14
21	Brazil	1995	Oranges, Mandarines	107
21	Brazil	1995	Palm Oil	1
21	Brazil	1995	Pelagic Fish	3
21	Brazil	1995	Pigmeat	30
21	Brazil	1995	Pineapples	19
21	Brazil	1995	Potatoes	39
21	Brazil	1995	Poultry Meat	63
21	Brazil	1995	Rice (Milled Equivalent)	89
21	Brazil	1995	Soyabean Oil	30
21	Brazil	1995	Soyabeans	7
21	Brazil	1995	Sugar Cane	14
21	Brazil	1995	Sugar, Non-Centrifugal	4
21	Brazil	1995	Sugar, Refined Equivalent	129
21	Brazil	1995	Sunflowerseed Oil	1
21	Brazil	1995	Sweet Potatoes	4
21	Brazil	1995	Tea	4
21	Brazil	1995	Tomatoes	43
21	Brazil	1995	Vegetables, Other	49
21	Brazil	1995	Wheat	139
21	Brazil	1995	Wine	5
21	Brazil	1995	Yams	2
21	Brazil	1996	Apples	14
21	Brazil	1996	Bananas	73
21	Brazil	1996	Beans	52
21	Brazil	1996	Beer	108
21	Brazil	1996	Beverages, Alcoholic	11
21	Brazil	1996	Bovine Meat	101
21	Brazil	1996	Butter, Ghee	1
21	Brazil	1996	Cassava	103
21	Brazil	1996	Cereals, Other	1
21	Brazil	1996	Cheese	1
21	Brazil	1996	Cocoa Beans	2
21	Brazil	1996	Coconuts - Incl Copra	10
21	Brazil	1996	Coffee	6
21	Brazil	1996	Cottonseed Oil	1
21	Brazil	1996	Crustaceans	1
21	Brazil	1996	Demersal Fish	8
21	Brazil	1996	Eggs	19
21	Brazil	1996	Fats, Animals, Raw	5
21	Brazil	1996	Freshwater Fish	5
21	Brazil	1996	Fruits, Other	52
21	Brazil	1996	Grapefruit	1
21	Brazil	1996	Grapes	6
21	Brazil	1996	Groundnuts (Shelled Eq)	1
21	Brazil	1996	Lemons, Limes	6

FAO country code	Country	Reference year	Food item	Gross food supply (g *capita*$^{-1}$ d^{-1})
21	Brazil	1996	Maize	51
21	Brazil	1996	Maize Germ Oil	1
21	Brazil	1996	Marine Fish, Other	2
21	Brazil	1996	Milk, Whole	288
21	Brazil	1996	Mutton & Goat Meat	2
21	Brazil	1996	Nuts	1
21	Brazil	1996	Oats	3
21	Brazil	1996	Offals, Edible	6
21	Brazil	1996	Olives	1
21	Brazil	1996	Onions	13
21	Brazil	1996	Oranges, Mandarines	127
21	Brazil	1996	Palm Oil	1
21	Brazil	1996	Peas	1
21	Brazil	1996	Pelagic Fish	3
21	Brazil	1996	Pigmeat	30
21	Brazil	1996	Pineapples	15
21	Brazil	1996	Potatoes	33
21	Brazil	1996	Poultry Meat	59
21	Brazil	1996	Rice (Milled Equivalent)	103
21	Brazil	1996	Soyabean Oil	31
21	Brazil	1996	Soyabeans	7
21	Brazil	1996	Sugar Cane	15
21	Brazil	1996	Sugar, Non-Centrifugal	4
21	Brazil	1996	Sugar, Refined Equivalent	125
21	Brazil	1996	Sunflowerseed Oil	1
21	Brazil	1996	Sweet Potatoes	3
21	Brazil	1996	Tea	3
21	Brazil	1996	Tomatoes	41
21	Brazil	1996	Vegetables, Other	47
21	Brazil	1996	Wheat	126
21	Brazil	1996	Wine	4
21	Brazil	1996	Yams	2
21	Brazil	1997	Apples	13
21	Brazil	1997	Bananas	75
21	Brazil	1997	Beans	41
21	Brazil	1997	Beer	110
21	Brazil	1997	Beverages, Alcoholic	12
21	Brazil	1997	Bovine Meat	95
21	Brazil	1997	Butter, Ghee	1
21	Brazil	1997	Cassava	112
21	Brazil	1997	Cereals, Other	1
21	Brazil	1997	Cheese	1
21	Brazil	1997	Cocoa Beans	2
21	Brazil	1997	Coconuts - Incl Copra	23
21	Brazil	1997	Coffee	7
21	Brazil	1997	Cottonseed Oil	2
21	Brazil	1997	Crustaceans	1
21	Brazil	1997	Demersal Fish	8
21	Brazil	1997	Eggs	21
21	Brazil	1997	Fats, Animals, Raw	5
21	Brazil	1997	Freshwater Fish	4
21	Brazil	1997	Fruits, Other	57
21	Brazil	1997	Grapefruit	1
21	Brazil	1997	Grapes	8
21	Brazil	1997	Groundnuts (Shelled Eq)	1
21	Brazil	1997	Lemons, Limes	6
21	Brazil	1997	Maize	53
21	Brazil	1997	Maize Germ Oil	1
21	Brazil	1997	Marine Fish, Other	2
21	Brazil	1997	Milk, Whole	285
21	Brazil	1997	Mutton & Goat Meat	2
21	Brazil	1997	Nuts	1
21	Brazil	1997	Oats	3
21	Brazil	1997	Offals, Edible	6
21	Brazil	1997	Olives	1
21	Brazil	1997	Onions	13
21	Brazil	1997	Oranges, Mandarines	156
21	Brazil	1997	Palm Oil	1
21	Brazil	1997	Peas	1
21	Brazil	1997	Pelagic Fish	2
21	Brazil	1997	Pigmeat	32
21	Brazil	1997	Pineapples	21
21	Brazil	1997	Potatoes	37
21	Brazil	1997	Poultry Meat	68
21	Brazil	1997	Rice (Milled Equivalent)	102
21	Brazil	1997	Soyabean Oil	30
21	Brazil	1997	Soyabeans	6
21	Brazil	1997	Sugar Cane	15
21	Brazil	1997	Sugar, Non-Centrifugal	4
21	Brazil	1997	Sugar, Refined Equivalent	123
21	Brazil	1997	Sunflowerseed Oil	1
21	Brazil	1997	Sweet Potatoes	3
21	Brazil	1997	Tea	5
21	Brazil	1997	Tomatoes	42
21	Brazil	1997	Vegetables, Other	49
21	Brazil	1997	Wheat	125
21	Brazil	1997	Wine	4
21	Brazil	1997	Yams	2
21	Brazil	1998	Apples	13
21	Brazil	1998	Bananas	72
21	Brazil	1998	Beans	42
21	Brazil	1998	Beer	107
21	Brazil	1998	Beverages, Alcoholic	11
21	Brazil	1998	Bovine Meat	89
21	Brazil	1998	Butter, Ghee	1
21	Brazil	1998	Cassava	108
21	Brazil	1998	Cereals, Other	1

FAO country code	Country	Reference year	Food item	Gross food supply (g $capita^{-1}$ d^{-1})
21	Brazil	1998	Cheese	1
21	Brazil	1998	Cocoa Beans	2
21	Brazil	1998	Coconuts - Incl Copra	22
21	Brazil	1998	Coffee	7
21	Brazil	1998	Cottonseed Oil	2
21	Brazil	1998	Crustaceans	1
21	Brazil	1998	Demersal Fish	7
21	Brazil	1998	Eggs	19
21	Brazil	1998	Fats, Animals, Raw	5
21	Brazil	1998	Freshwater Fish	4
21	Brazil	1998	Fruits, Other	52
21	Brazil	1998	Grapefruit	1
21	Brazil	1998	Grapes	7
21	Brazil	1998	Groundnuts (Shelled Eq)	1
21	Brazil	1998	Lemons, Limes	6
21	Brazil	1998	Maize	46
21	Brazil	1998	Maize Germ Oil	1
21	Brazil	1998	Marine Fish, Other	2
21	Brazil	1998	Milk, Whole	285
21	Brazil	1998	Mutton & Goat Meat	2
21	Brazil	1998	Nuts	1
21	Brazil	1998	Oats	3
21	Brazil	1998	Offals, Edible	6
21	Brazil	1998	Olives	1
21	Brazil	1998	Onions	12
21	Brazil	1998	Oranges, Mandarines	114
21	Brazil	1998	Palm Oil	2
21	Brazil	1998	Pelagic Fish	2
21	Brazil	1998	Pigmeat	37
21	Brazil	1998	Pineapples	22
21	Brazil	1998	Potatoes	40
21	Brazil	1998	Poultry Meat	70
21	Brazil	1998	Pulses, Other	1
21	Brazil	1998	Rice (Milled Equivalent)	109
21	Brazil	1998	Soyabean Oil	30
21	Brazil	1998	Soyabeans	7
21	Brazil	1998	Sugar Cane	15
21	Brazil	1998	Sugar, Non-Centrifugal	4
21	Brazil	1998	Sugar, Refined Equivalent	121
21	Brazil	1998	Sunflowerseed Oil	1
21	Brazil	1998	Sweet Potatoes	3
21	Brazil	1998	Tea	6
21	Brazil	1998	Tomatoes	41
21	Brazil	1998	Vegetables, Other	49
21	Brazil	1998	Wheat	127
21	Brazil	1998	Wine	4
21	Brazil	1998	Yams	2
21	Brazil	1999	Apples	13
21	Brazil	1999	Bananas	73
21	Brazil	1999	Beans	42
21	Brazil	1999	Beer	99
21	Brazil	1999	Beverages, Alcoholic	11
21	Brazil	1999	Bovine Meat	94
21	Brazil	1999	Butter, Ghee	1
21	Brazil	1999	Cassava	113
21	Brazil	1999	Cereals, Other	1
21	Brazil	1999	Cheese	1
21	Brazil	1999	Cocoa Beans	2
21	Brazil	1999	Coconuts - Incl Copra	26
21	Brazil	1999	Coffee	8
21	Brazil	1999	Cottonseed Oil	2
21	Brazil	1999	Crustaceans	1
21	Brazil	1999	Demersal Fish	6
21	Brazil	1999	Eggs	20
21	Brazil	1999	Fats, Animals, Raw	6
21	Brazil	1999	Freshwater Fish	5
21	Brazil	1999	Fruits, Other	51
21	Brazil	1999	Grapefruit	1
21	Brazil	1999	Grapes	7
21	Brazil	1999	Groundnuts (Shelled Eq)	1
21	Brazil	1999	Lemons, Limes	6
21	Brazil	1999	Maize	45
21	Brazil	1999	Maize Germ Oil	1
21	Brazil	1999	Marine Fish, Other	2
21	Brazil	1999	Milk, Whole	287
21	Brazil	1999	Mutton & Goat Meat	2
21	Brazil	1999	Nuts	1
21	Brazil	1999	Oats	4
21	Brazil	1999	Offals, Edible	7
21	Brazil	1999	Olives	1
21	Brazil	1999	Onions	14
21	Brazil	1999	Oranges, Mandarines	151
21	Brazil	1999	Palm Oil	1
21	Brazil	1999	Peas	1
21	Brazil	1999	Pelagic Fish	2
21	Brazil	1999	Pigmeat	36
21	Brazil	1999	Pineapples	23
21	Brazil	1999	Potatoes	41
21	Brazil	1999	Poultry Meat	77
21	Brazil	1999	Rice (Milled Equivalent)	109
21	Brazil	1999	Soyabean Oil	37
21	Brazil	1999	Soyabeans	7
21	Brazil	1999	Sugar Cane	14
21	Brazil	1999	Sugar, Non-Centrifugal	3
21	Brazil	1999	Sugar, Refined Equivalent	96
21	Brazil	1999	Sunflowerseed Oil	2
21	Brazil	1999	Sweet Potatoes	3

FAO countrycode	Country	Reference year	Food item	Gross food supply(g $capita^{-1}$ d^{-1})
21	Brazil	1999	Tea	7
21	Brazil	1999	Tomatoes	47
21	Brazil	1999	Vegetables, Other	47
21	Brazil	1999	Wheat	130
21	Brazil	1999	Wine	5
21	Brazil	1999	Yams	2
21	Brazil	2000	Apples	15
21	Brazil	2000	Bananas	74
21	Brazil	2000	Beans	45
21	Brazil	2000	Beer	137
21	Brazil	2000	Beverages, Alcoholic	11
21	Brazil	2000	Bovine Meat	96
21	Brazil	2000	Butter, Ghee	1
21	Brazil	2000	Cassava	124
21	Brazil	2000	Cereals, Other	1
21	Brazil	2000	Cheese	1
21	Brazil	2000	Cocoa Beans	2
21	Brazil	2000	Coconuts - Incl Copra	28
21	Brazil	2000	Coffee	8
21	Brazil	2000	Cottonseed Oil	2
21	Brazil	2000	Crustaceans	1
21	Brazil	2000	Demersal Fish	6
21	Brazil	2000	Eggs	20
21	Brazil	2000	Fats, Animals, Raw	6
21	Brazil	2000	Freshwater Fish	5
21	Brazil	2000	Fruits, Other	52
21	Brazil	2000	Grapefruit	1
21	Brazil	2000	Grapes	8
21	Brazil	2000	Groundnuts (Shelled Eq)	1
21	Brazil	2000	Lemons, Limes	6
21	Brazil	2000	Maize	43
21	Brazil	2000	Maize Germ Oil	1
21	Brazil	2000	Marine Fish, Other	2
21	Brazil	2000	Milk, Whole	288
21	Brazil	2000	Mutton & Goat Meat	2
21	Brazil	2000	Nuts	1
21	Brazil	2000	Oats	2
21	Brazil	2000	Offals, Edible	7
21	Brazil	2000	Olives	1
21	Brazil	2000	Onions	16
21	Brazil	2000	Oranges, Mandarines	118
21	Brazil	2000	Palm Oil	2
21	Brazil	2000	Peas	1
21	Brazil	2000	Pelagic Fish	2
21	Brazil	2000	Pigmeat	38
21	Brazil	2000	Pineapples	25
21	Brazil	2000	Potatoes	35
21	Brazil	2000	Poultry Meat	81
21	Brazil	2000	Rice (Milled Equivalent)	105
21	Brazil	2000	Soyabean Oil	38
21	Brazil	2000	Soyabeans	7
21	Brazil	2000	Sugar Cane	31
21	Brazil	2000	Sugar, Non-Centrifugal	5
21	Brazil	2000	Sugar, Refined Equivalent	95
21	Brazil	2000	Sunflowerseed Oil	2
21	Brazil	2000	Sweet Potatoes	3
21	Brazil	2000	Tea	8
21	Brazil	2000	Tomatoes	42
21	Brazil	2000	Vegetables, Other	45
21	Brazil	2000	Wheat	120
21	Brazil	2000	Wine	5
21	Brazil	2000	Yams	2
21	Brazil	2001	Apples	10
21	Brazil	2001	Bananas	80
21	Brazil	2001	Beans	45
21	Brazil	2001	Beer	141
21	Brazil	2001	Beverages, Alcoholic	11
21	Brazil	2001	Bovine Meat	94
21	Brazil	2001	Butter, Ghee	1
21	Brazil	2001	Cassava	119
21	Brazil	2001	Cereals, Other	1
21	Brazil	2001	Cheese	1
21	Brazil	2001	Cocoa Beans	2
21	Brazil	2001	Coconuts - Incl Copra	30
21	Brazil	2001	Coffee	9
21	Brazil	2001	Cottonseed Oil	3
21	Brazil	2001	Crustaceans	1
21	Brazil	2001	Demersal Fish	5
21	Brazil	2001	Eggs	20
21	Brazil	2001	Fats, Animals, Raw	6
21	Brazil	2001	Freshwater Fish	6
21	Brazil	2001	Fruits, Other	58
21	Brazil	2001	Grapefruit	1
21	Brazil	2001	Grapes	9
21	Brazil	2001	Groundnuts (Shelled Eq)	1
21	Brazil	2001	Lemons, Limes	12
21	Brazil	2001	Maize	52
21	Brazil	2001	Maize Germ Oil	1
21	Brazil	2001	Marine Fish, Other	2
21	Brazil	2001	Milk, Whole	282
21	Brazil	2001	Mutton & Goat Meat	2
21	Brazil	2001	Nuts	1
21	Brazil	2001	Oats	5
21	Brazil	2001	Offals, Edible	7
21	Brazil	2001	Olives	1
21	Brazil	2001	Onions	15
21	Brazil	2001	Oranges, Mandarines	37

FAO country code	Country	Reference year	Food item	Gross food supply (g *capita*$^{-1}$ d^{-1})
21	Brazil	2001	Palm Oil	2
21	Brazil	2001	Pelagic Fish	2
21	Brazil	2001	Pigmeat	36
21	Brazil	2001	Pineapples	26
21	Brazil	2001	Potatoes	39
21	Brazil	2001	Poultry Meat	78
21	Brazil	2001	Rice (Milled Equivalent)	97
21	Brazil	2001	Soyabean Oil	39
21	Brazil	2001	Soyabeans	7
21	Brazil	2001	Sugar Cane	15
21	Brazil	2001	Sugar, Non-Centrifugal	5
21	Brazil	2001	Sugar, Refined Equivalent	94
21	Brazil	2001	Sunflowerseed Oil	1
21	Brazil	2001	Sweet Potatoes	3
21	Brazil	2001	Tea	9
21	Brazil	2001	Tomatoes	43
21	Brazil	2001	Vegetables, Other	47
21	Brazil	2001	Wheat	135
21	Brazil	2001	Wine	5
21	Brazil	2001	Yams	2
21	Brazil	2002	Apples	10
21	Brazil	2002	Bananas	80
21	Brazil	2002	Beans	44
21	Brazil	2002	Beer	122
21	Brazil	2002	Beverages, Alcoholic	11
21	Brazil	2002	Bovine Meat	96
21	Brazil	2002	Butter, Ghee	1
21	Brazil	2002	Cassava	119
21	Brazil	2002	Cereals, Other	1
21	Brazil	2002	Cheese	1
21	Brazil	2002	Cocoa Beans	2
21	Brazil	2002	Coconuts - Incl Copra	40
21	Brazil	2002	Coffee	8
21	Brazil	2002	Cottonseed Oil	2
21	Brazil	2002	Crustaceans	1
21	Brazil	2002	Demersal Fish	6
21	Brazil	2002	Eggs	20
21	Brazil	2002	Fats, Animals, Raw	6
21	Brazil	2002	Freshwater Fish	6
21	Brazil	2002	Fruits, Other	60
21	Brazil	2002	Grapefruit	1
21	Brazil	2002	Grapes	9
21	Brazil	2002	Groundnuts (Shelled Eq)	1
21	Brazil	2002	Lemons, Limes	11
21	Brazil	2002	Maize	57
21	Brazil	2002	Maize Germ Oil	1
21	Brazil	2002	Marine Fish, Other	2
21	Brazil	2002	Milk, Whole	300
21	Brazil	2002	Mutton & Goat Meat	2
21	Brazil	2002	Nuts	1
21	Brazil	2002	Oats	4
21	Brazil	2002	Offals, Edible	7
21	Brazil	2002	Olives	1
21	Brazil	2002	Onions	17
21	Brazil	2002	Oranges, Mandarines	108
21	Brazil	2002	Palm Oil	2
21	Brazil	2002	Pelagic Fish	2
21	Brazil	2002	Pigmeat	34
21	Brazil	2002	Pineapples	26
21	Brazil	2002	Potatoes	42
21	Brazil	2002	Poultry Meat	84
21	Brazil	2002	Rice (Milled Equivalent)	94
21	Brazil	2002	Soyabean Oil	37
21	Brazil	2002	Soyabeans	8
21	Brazil	2002	Sugar Cane	15
21	Brazil	2002	Sugar, Non-Centrifugal	3
21	Brazil	2002	Sugar, Refined Equivalent	96
21	Brazil	2002	Sweet Potatoes	3
21	Brazil	2002	Tea	7
21	Brazil	2002	Tomatoes	50
21	Brazil	2002	Vegetables, Other	55
21	Brazil	2002	Wheat	133
21	Brazil	2002	Wine	5
21	Brazil	2002	Yams	2
21	Brazil	2003	Apples	9
21	Brazil	2003	Bananas	84
21	Brazil	2003	Beans	47
21	Brazil	2003	Beer	116
21	Brazil	2003	Beverages, Alcoholic	11
21	Brazil	2003	Bovine Meat	91
21	Brazil	2003	Butter, Ghee	1
21	Brazil	2003	Cassava	111
21	Brazil	2003	Cereals, Other	1
21	Brazil	2003	Cheese	1
21	Brazil	2003	Cocoa Beans	1
21	Brazil	2003	Coconuts - Incl Copra	40
21	Brazil	2003	Coffee	9
21	Brazil	2003	Cottonseed Oil	3
21	Brazil	2003	Crustaceans	1
21	Brazil	2003	Demersal Fish	5
21	Brazil	2003	Eggs	20
21	Brazil	2003	Fats, Animals, Raw	6
21	Brazil	2003	Freshwater Fish	6
21	Brazil	2003	Fruits, Other	60
21	Brazil	2003	Grapefruit	1
21	Brazil	2003	Grapes	9
21	Brazil	2003	Groundnuts (Shelled Eq)	1

FAO countrycode	Country	Reference year	Food item	Gross food supply (g *capita*$^{-1}$ d^{-1})
21	Brazil	2003	Lemons, Limes	11
21	Brazil	2003	Maize	70
21	Brazil	2003	Maize Germ Oil	1
21	Brazil	2003	Marine Fish, Other	1
21	Brazil	2003	Milk, Whole	295
21	Brazil	2003	Mutton & Goat Meat	2
21	Brazil	2003	Nuts	1
21	Brazil	2003	Oats	6
21	Brazil	2003	Offals, Edible	7
21	Brazil	2003	Olives	1
21	Brazil	2003	Onions	17
21	Brazil	2003	Oranges, Mandarines	71
21	Brazil	2003	Palm Oil	2
21	Brazil	2003	Pelagic Fish	2
21	Brazil	2003	Pigmeat	37
21	Brazil	2003	Pineapples	26
21	Brazil	2003	Potatoes	41
21	Brazil	2003	Poultry Meat	89
21	Brazil	2003	Rice (Milled Equivalent)	98
21	Brazil	2003	Soyabean Oil	38
21	Brazil	2003	Soyabeans	8
21	Brazil	2003	Sugar Cane	15
21	Brazil	2003	Sugar, Non-Centrifugal	5
21	Brazil	2003	Sugar, Refined Equivalent	99
21	Brazil	2003	Sunflowerseed Oil	1
21	Brazil	2003	Sweet Potatoes	3
21	Brazil	2003	Tea	7
21	Brazil	2003	Tomatoes	50
21	Brazil	2003	Vegetables, Other	62
21	Brazil	2003	Wheat	146
21	Brazil	2003	Wine	4
21	Brazil	2003	Yams	1
21	Brazil	2004	Apples	9
21	Brazil	2004	Bananas	81
21	Brazil	2004	Beans	42
21	Brazil	2004	Beer	129
21	Brazil	2004	Beverages, Alcoholic	10
21	Brazil	2004	Bovine Meat	92
21	Brazil	2004	Butter, Ghee	1
21	Brazil	2004	Cassava	120
21	Brazil	2004	Cereals, Other	1
21	Brazil	2004	Cheese	1
21	Brazil	2004	Cocoa Beans	1
21	Brazil	2004	Coconuts - Incl Copra	42
21	Brazil	2004	Coffee	10
21	Brazil	2004	Cottonseed Oil	3
21	Brazil	2004	Crustaceans	1
21	Brazil	2004	Demersal Fish	5
21	Brazil	2004	Eggs	20
21	Brazil	2004	Fats, Animals, Raw	8
21	Brazil	2004	Freshwater Fish	7
21	Brazil	2004	Fruits, Other	59
21	Brazil	2004	Grapefruit	1
21	Brazil	2004	Grapes	9
21	Brazil	2004	Groundnuts (Shelled Eq)	1
21	Brazil	2004	Lemons, Limes	10
21	Brazil	2004	Maize	66
21	Brazil	2004	Maize Germ Oil	1
21	Brazil	2004	Marine Fish, Other	1
21	Brazil	2004	Milk, Whole	304
21	Brazil	2004	Mutton & Goat Meat	2
21	Brazil	2004	Nuts	1
21	Brazil	2004	Oats	6
21	Brazil	2004	Offals, Edible	7
21	Brazil	2004	Olives	1
21	Brazil	2004	Onions	16
21	Brazil	2004	Oranges, Mandarines	93
21	Brazil	2004	Palm Oil	2
21	Brazil	2004	Pelagic Fish	2
21	Brazil	2004	Pigmeat	36
21	Brazil	2004	Pineapples	25
21	Brazil	2004	Potatoes	41
21	Brazil	2004	Poultry Meat	94
21	Brazil	2004	Rice (Milled Equivalent)	105
21	Brazil	2004	Soyabean Oil	38
21	Brazil	2004	Soyabeans	9
21	Brazil	2004	Sugar Cane	30
21	Brazil	2004	Sugar, Non-Centrifugal	4
21	Brazil	2004	Sugar, Refined Equivalent	101
21	Brazil	2004	Sweet Potatoes	3
21	Brazil	2004	Sweeteners, Other	1
21	Brazil	2004	Tea	5
21	Brazil	2004	Tomatoes	47
21	Brazil	2004	Vegetables, Other	60
21	Brazil	2004	Wheat	139
21	Brazil	2004	Wine	6
21	Brazil	2004	Yams	2
21	Brazil	2005	Apples	8
21	Brazil	2005	Bananas	81
21	Brazil	2005	Beans	42
21	Brazil	2005	Beer	132
21	Brazil	2005	Beverages, Alcoholic	10
21	Brazil	2005	Bovine Meat	99
21	Brazil	2005	Butter, Ghee	1
21	Brazil	2005	Cassava	127
21	Brazil	2005	Cereals, Other	5
21	Brazil	2005	Cheese	1

FAO country code	Country	Reference year	Food item	Gross food supply (g *capita*$^{-1}$ d^{-1})
21	Brazil	2005	Cocoa Beans	2
21	Brazil	2005	Coconuts - Incl Copra	42
21	Brazil	2005	Coffee	10
21	Brazil	2005	Cottonseed Oil	3
21	Brazil	2005	Crustaceans	1
21	Brazil	2005	Demersal Fish	5
21	Brazil	2005	Eggs	20
21	Brazil	2005	Fats, Animals, Raw	7
21	Brazil	2005	Freshwater Fish	6
21	Brazil	2005	Fruits, Other	59
21	Brazil	2005	Grapefruit	1
21	Brazil	2005	Grapes	10
21	Brazil	2005	Groundnuts (Shelled Eq)	1
21	Brazil	2005	Lemons, Limes	11
21	Brazil	2005	Maize	62
21	Brazil	2005	Maize Germ Oil	1
21	Brazil	2005	Marine Fish, Other	1
21	Brazil	2005	Milk, Whole	317
21	Brazil	2005	Mutton & Goat Meat	2
21	Brazil	2005	Nuts	1
21	Brazil	2005	Oats	7
21	Brazil	2005	Offals, Edible	7
21	Brazil	2005	Olives	1
21	Brazil	2005	Onions	15
21	Brazil	2005	Oranges, Mandarines	76
21	Brazil	2005	Palm Oil	2
21	Brazil	2005	Pelagic Fish	2
21	Brazil	2005	Pepper	1
21	Brazil	2005	Pigmeat	28
21	Brazil	2005	Pineapples	26
21	Brazil	2005	Potatoes	42
21	Brazil	2005	Poultry Meat	75
21	Brazil	2005	Rice (Milled Equivalent)	108
21	Brazil	2005	Soyabean Oil	38
21	Brazil	2005	Soyabeans	9
21	Brazil	2005	Sugar Cane	29
21	Brazil	2005	Sugar, Non-Centrifugal	4
21	Brazil	2005	Sugar, Refined Equivalent	102
21	Brazil	2005	Sweet Potatoes	3
21	Brazil	2005	Tea	6
21	Brazil	2005	Tomatoes	46
21	Brazil	2005	Vegetables, Other	59
21	Brazil	2005	Wheat	142
21	Brazil	2005	Wine	5
21	Brazil	2005	Yams	2
21	Brazil	2006	Apples	10
21	Brazil	2006	Bananas	83
21	Brazil	2006	Beans	44
21	Brazil	2006	Beer	135
21	Brazil	2006	Beverages, Alcoholic	10
21	Brazil	2006	Bovine Meat	100
21	Brazil	2006	Butter, Ghee	1
21	Brazil	2006	Cassava	129
21	Brazil	2006	Cereals, Other	4
21	Brazil	2006	Cheese	1
21	Brazil	2006	Cocoa Beans	2
21	Brazil	2006	Coconuts - Incl Copra	39
21	Brazil	2006	Coffee	9
21	Brazil	2006	Cottonseed Oil	3
21	Brazil	2006	Crustaceans	1
21	Brazil	2006	Demersal Fish	6
21	Brazil	2006	Eggs	21
21	Brazil	2006	Fats, Animals, Raw	7
21	Brazil	2006	Freshwater Fish	7
21	Brazil	2006	Fruits, Other	67
21	Brazil	2006	Grapefruit	1
21	Brazil	2006	Grapes	12
21	Brazil	2006	Groundnuts (Shelled Eq)	1
21	Brazil	2006	Lemons, Limes	10
21	Brazil	2006	Maize	67
21	Brazil	2006	Maize Germ Oil	1
21	Brazil	2006	Marine Fish, Other	2
21	Brazil	2006	Milk, Whole	323
21	Brazil	2006	Mutton & Goat Meat	2
21	Brazil	2006	Nuts	1
21	Brazil	2006	Oats	6
21	Brazil	2006	Offals, Edible	7
21	Brazil	2006	Olives	1
21	Brazil	2006	Onions	18
21	Brazil	2006	Oranges, Mandarines	88
21	Brazil	2006	Palm Oil	3
21	Brazil	2006	Pelagic Fish	2
21	Brazil	2006	Pepper	1
21	Brazil	2006	Pigmeat	30
21	Brazil	2006	Pineapples	29
21	Brazil	2006	Potatoes	42
21	Brazil	2006	Poultry Meat	81
21	Brazil	2006	Rice (Milled Equivalent)	95
21	Brazil	2006	Soyabean Oil	38
21	Brazil	2006	Soyabeans	8
21	Brazil	2006	Sugar Cane	36
21	Brazil	2006	Sugar, Non-Centrifugal	5
21	Brazil	2006	Sugar, Refined Equivalent	100
21	Brazil	2006	Sunflowerseed Oil	1
21	Brazil	2006	Sweet Potatoes	3
21	Brazil	2006	Tea	6
21	Brazil	2006	Tomatoes	44

FAO countrycode	Country	Reference year	Food item	Gross food supply (g $capita^{-1}$ d^{-1})
21	Brazil	2006	Vegetables, Other	65
21	Brazil	2006	Wheat	143
21	Brazil	2006	Wine	4
21	Brazil	2006	Yams	2
21	Brazil	2007	Apples	12
21	Brazil	2007	Bananas	84
21	Brazil	2007	Beans	44
21	Brazil	2007	Beer	138
21	Brazil	2007	Beverages, Alcoholic	10
21	Brazil	2007	Bovine Meat	102
21	Brazil	2007	Butter, Ghee	1
21	Brazil	2007	Cassava	127
21	Brazil	2007	Cereals, Other	3
21	Brazil	2007	Cheese	1
21	Brazil	2007	Cocoa Beans	2
21	Brazil	2007	Coconuts - Incl Copra	37
21	Brazil	2007	Coffee	9
21	Brazil	2007	Cottonseed Oil	4
21	Brazil	2007	Crustaceans	1
21	Brazil	2007	Demersal Fish	6
21	Brazil	2007	Eggs	20
21	Brazil	2007	Fats, Animals, Raw	8
21	Brazil	2007	Freshwater Fish	7
21	Brazil	2007	Fruits, Other	66
21	Brazil	2007	Grapefruit	1
21	Brazil	2007	Grapes	10
21	Brazil	2007	Groundnuts (Shelled Eq)	1
21	Brazil	2007	Lemons, Limes	9
21	Brazil	2007	Maize	69
21	Brazil	2007	Maize Germ Oil	1
21	Brazil	2007	Marine Fish, Other	2
21	Brazil	2007	Milk, Whole	316
21	Brazil	2007	Mutton & Goat Meat	2
21	Brazil	2007	Nuts	1
21	Brazil	2007	Oats	3
21	Brazil	2007	Offals, Edible	7
21	Brazil	2007	Olive Oil	1
21	Brazil	2007	Olives	1
21	Brazil	2007	Onions	18
21	Brazil	2007	Oranges, Mandarines	87
21	Brazil	2007	Palm Oil	4
21	Brazil	2007	Pelagic Fish	2
21	Brazil	2007	Pepper	1
21	Brazil	2007	Pigmeat	30
21	Brazil	2007	Pineapples	30
21	Brazil	2007	Potatoes	46
21	Brazil	2007	Poultry Meat	87
21	Brazil	2007	Rice (Milled Equivalent)	91
21	Brazil	2007	Soyabean Oil	37
21	Brazil	2007	Soyabeans	9
21	Brazil	2007	Sugar Cane	39
21	Brazil	2007	Sugar, Non-Centrifugal	5
21	Brazil	2007	Sugar, Refined Equivalent	100
21	Brazil	2007	Sunflowerseed Oil	1
21	Brazil	2007	Sweet Potatoes	3
21	Brazil	2007	Sweeteners, Other	1
21	Brazil	2007	Tea	6
21	Brazil	2007	Tomatoes	44
21	Brazil	2007	Vegetables, Other	65
21	Brazil	2007	Wheat	147
21	Brazil	2007	Wine	6
21	Brazil	2007	Yams	2
21	Brazil	2008	Apples	12
21	Brazil	2008	Bananas	83
21	Brazil	2008	Beans	45
21	Brazil	2008	Beer	151
21	Brazil	2008	Beverages, Alcoholic	10
21	Brazil	2008	Bovine Meat	103
21	Brazil	2008	Butter, Ghee	1
21	Brazil	2008	Cassava	127
21	Brazil	2008	Cereals, Other	3
21	Brazil	2008	Cheese	1
21	Brazil	2008	Cocoa Beans	2
21	Brazil	2008	Coconuts - Incl Copra	41
21	Brazil	2008	Coffee	11
21	Brazil	2008	Cottonseed Oil	4
21	Brazil	2008	Crustaceans	2
21	Brazil	2008	Demersal Fish	6
21	Brazil	2008	Eggs	21
21	Brazil	2008	Fats, Animals, Raw	8
21	Brazil	2008	Freshwater Fish	8
21	Brazil	2008	Fruits, Other	65
21	Brazil	2008	Grapefruit	1
21	Brazil	2008	Grapes	11
21	Brazil	2008	Groundnuts (Shelled Eq)	1
21	Brazil	2008	Lemons, Limes	7
21	Brazil	2008	Maize	69
21	Brazil	2008	Maize Germ Oil	1
21	Brazil	2008	Marine Fish, Other	2
21	Brazil	2008	Milk, Whole	338
21	Brazil	2008	Mutton & Goat Meat	2
21	Brazil	2008	Nuts	1
21	Brazil	2008	Oats	3
21	Brazil	2008	Offals, Edible	7
21	Brazil	2008	Olive Oil	1
21	Brazil	2008	Olives	1
21	Brazil	2008	Onions	18

FAO country code	Country	Reference year	Food item	Gross food supply (g *capita*$^{-1}$ d^{-1})
21	Brazil	2008	Oranges, Mandarines	106
21	Brazil	2008	Palm Oil	5
21	Brazil	2008	Peas	1
21	Brazil	2008	Pelagic Fish	2
21	Brazil	2008	Pigmeat	32
21	Brazil	2008	Pineapples	28
21	Brazil	2008	Potatoes	49
21	Brazil	2008	Poultry Meat	100
21	Brazil	2008	Rice (Milled Equivalent)	91
21	Brazil	2008	Soyabean Oil	37
21	Brazil	2008	Soyabeans	9
21	Brazil	2008	Sugar Cane	41
21	Brazil	2008	Sugar, Non-Centrifugal	6
21	Brazil	2008	Sugar, Refined Equivalent	100
21	Brazil	2008	Sunflowerseed Oil	1
21	Brazil	2008	Sweet Potatoes	3
21	Brazil	2008	Sweeteners, Other	1
21	Brazil	2008	Tea	6
21	Brazil	2008	Tomatoes	50
21	Brazil	2008	Vegetables, Other	62
21	Brazil	2008	Wheat	147
21	Brazil	2008	Wine	6
21	Brazil	2008	Yams	2
21	Brazil	2009	Apples	14
21	Brazil	2009	Bananas	80
21	Brazil	2009	Beans	45
21	Brazil	2009	Beer	151
21	Brazil	2009	Beverages, Alcoholic	10
21	Brazil	2009	Bovine Meat	110
21	Brazil	2009	Butter, Ghee	1
21	Brazil	2009	Cassava	116
21	Brazil	2009	Cereals, Other	2
21	Brazil	2009	Cheese	1
21	Brazil	2009	Cocoa Beans	3
21	Brazil	2009	Coconuts - Incl Copra	38
21	Brazil	2009	Coffee	8
21	Brazil	2009	Cottonseed Oil	3
21	Brazil	2009	Crustaceans	2
21	Brazil	2009	Demersal Fish	7
21	Brazil	2009	Eggs	22
21	Brazil	2009	Fats, Animals, Raw	8
21	Brazil	2009	Freshwater Fish	9
21	Brazil	2009	Fruits, Other	65
21	Brazil	2009	Grapefruit	1
21	Brazil	2009	Grapes	11
21	Brazil	2009	Lemons, Limes	8
21	Brazil	2009	Maize	67
21	Brazil	2009	Maize Germ Oil	1
21	Brazil	2009	Marine Fish, Other	2
21	Brazil	2009	Milk, Whole	357
21	Brazil	2009	Mutton & Goat Meat	2
21	Brazil	2009	Nuts	1
21	Brazil	2009	Oats	3
21	Brazil	2009	Offals, Edible	6
21	Brazil	2009	Olive Oil	1
21	Brazil	2009	Olives	1
21	Brazil	2009	Onions	19
21	Brazil	2009	Oranges, Mandarines	117
21	Brazil	2009	Palm Oil	5
21	Brazil	2009	Peas	1
21	Brazil	2009	Pelagic Fish	3
21	Brazil	2009	Pigmeat	32
21	Brazil	2009	Pineapples	24
21	Brazil	2009	Potatoes	45
21	Brazil	2009	Poultry Meat	96
21	Brazil	2009	Rice (Milled Equivalent)	95
21	Brazil	2009	Soyabean Oil	37
21	Brazil	2009	Soyabeans	9
21	Brazil	2009	Sugar Cane	44
21	Brazil	2009	Sugar, Non-Centrifugal	6
21	Brazil	2009	Sugar, Refined Equivalent	99
21	Brazil	2009	Sunflowerseed Oil	1
21	Brazil	2009	Sweet Potatoes	3
21	Brazil	2009	Sweeteners, Other	1
21	Brazil	2009	Tea	6
21	Brazil	2009	Tomatoes	55
21	Brazil	2009	Vegetables, Other	67
21	Brazil	2009	Wheat	146
21	Brazil	2009	Wine	5
21	Brazil	2009	Yams	1
21	Brazil	2010	Apples	13
21	Brazil	2010	Bananas	81
21	Brazil	2010	Beans	45
21	Brazil	2010	Beer	180
21	Brazil	2010	Beverages, Alcoholic	10
21	Brazil	2010	Bovine Meat	106
21	Brazil	2010	Butter, Ghee	1
21	Brazil	2010	Cassava	117
21	Brazil	2010	Cereals, Other	2
21	Brazil	2010	Cheese	1
21	Brazil	2010	Cocoa Beans	3
21	Brazil	2010	Coconuts - Incl Copra	36
21	Brazil	2010	Coffee	9
21	Brazil	2010	Cottonseed Oil	3
21	Brazil	2010	Crustaceans	2
21	Brazil	2010	Demersal Fish	9
21	Brazil	2010	Eggs	23

FAO countrycode	Country	Reference year	Food item	Gross food supply (g $capita^{-1}$ d^{-1})
21	Brazil	2010	Fats, Animals, Raw	8
21	Brazil	2010	Freshwater Fish	10
21	Brazil	2010	Fruits, Other	70
21	Brazil	2010	Grapefruit	1
21	Brazil	2010	Grapes	11
21	Brazil	2010	Groundnuts (Shelled Eq)	1
21	Brazil	2010	Lemons, Limes	10
21	Brazil	2010	Maize	67
21	Brazil	2010	Maize Germ Oil	1
21	Brazil	2010	Marine Fish, Other	2
21	Brazil	2010	Milk, Whole	372
21	Brazil	2010	Mutton & Goat Meat	2
21	Brazil	2010	Nuts	1
21	Brazil	2010	Oats	5
21	Brazil	2010	Offals, Edible	6
21	Brazil	2010	Olive Oil	1
21	Brazil	2010	Olives	1
21	Brazil	2010	Onions	22
21	Brazil	2010	Oranges, Mandarines	143
21	Brazil	2010	Palm Oil	5
21	Brazil	2010	Peas	1
21	Brazil	2010	Pelagic Fish	2
21	Brazil	2010	Pigmeat	34
21	Brazil	2010	Pineapples	24
21	Brazil	2010	Potatoes	48
21	Brazil	2010	Poultry Meat	103
21	Brazil	2010	Rice (Milled Equivalent)	92
21	Brazil	2010	Soyabean Oil	37
21	Brazil	2010	Soyabeans	10
21	Brazil	2010	Sugar Cane	45
21	Brazil	2010	Sugar, Non-Centrifugal	6
21	Brazil	2010	Sugar, Refined Equivalent	100
21	Brazil	2010	Sunflowerseed Oil	1
21	Brazil	2010	Sweet Potatoes	3
21	Brazil	2010	Sweeteners, Other	1
21	Brazil	2010	Tea	5
21	Brazil	2010	Tomatoes	53
21	Brazil	2010	Vegetables, Other	70
21	Brazil	2010	Wheat	148
21	Brazil	2010	Wine	6
21	Brazil	2010	Yams	2
21	Brazil	2011	Apples	15
21	Brazil	2011	Bananas	85
21	Brazil	2011	Beans	45
21	Brazil	2011	Beer	185
21	Brazil	2011	Beverages, Alcoholic	10
21	Brazil	2011	Bovine Meat	107
21	Brazil	2011	Butter, Ghee	1
21	Brazil	2011	Cassava	118
21	Brazil	2011	Cereals, Other	2
21	Brazil	2011	Cheese	1
21	Brazil	2011	Cocoa Beans	3
21	Brazil	2011	Coconuts - Incl Copra	37
21	Brazil	2011	Coffee	8
21	Brazil	2011	Cottonseed Oil	3
21	Brazil	2011	Crustaceans	2
21	Brazil	2011	Demersal Fish	11
21	Brazil	2011	Eggs	24
21	Brazil	2011	Fats, Animals, Raw	8
21	Brazil	2011	Freshwater Fish	12
21	Brazil	2011	Fruits, Other	71
21	Brazil	2011	Grapefruit	1
21	Brazil	2011	Grapes	13
21	Brazil	2011	Groundnuts (Shelled Eq)	2
21	Brazil	2011	Lemons, Limes	11
21	Brazil	2011	Maize	67
21	Brazil	2011	Maize Germ Oil	1
21	Brazil	2011	Marine Fish, Other	2
21	Brazil	2011	Milk, Whole	390
21	Brazil	2011	Molluscs, Other	1
21	Brazil	2011	Mutton & Goat Meat	2
21	Brazil	2011	Nuts	3
21	Brazil	2011	Oats	5
21	Brazil	2011	Offals, Edible	6
21	Brazil	2011	Olive Oil	1
21	Brazil	2011	Olives	1
21	Brazil	2011	Onions	19
21	Brazil	2011	Oranges, Mandarines	159
21	Brazil	2011	Palm Oil	5
21	Brazil	2011	Peas	1
21	Brazil	2011	Pelagic Fish	3
21	Brazil	2011	Pigmeat	34
21	Brazil	2011	Pineapples	26
21	Brazil	2011	Potatoes	53
21	Brazil	2011	Poultry Meat	111
21	Brazil	2011	Pulses, Other	1
21	Brazil	2011	Rice (Milled Equivalent)	94
21	Brazil	2011	Soyabean Oil	37
21	Brazil	2011	Soyabeans	10
21	Brazil	2011	Sugar Cane	46
21	Brazil	2011	Sugar, Non-Centrifugal	7
21	Brazil	2011	Sugar, Refined Equivalent	100
21	Brazil	2011	Sunflowerseed Oil	1
21	Brazil	2011	Sweet Potatoes	3
21	Brazil	2011	Sweeteners, Other	1
21	Brazil	2011	Tea	5
21	Brazil	2011	Tomatoes	56

FAO country code	Country	Reference year	Food item	Gross food supply (g $capita^{-1}$ d^{-1})
21	Brazil	2011	Vegetables, Other	73
21	Brazil	2011	Wheat	146
21	Brazil	2011	Wine	6
21	Brazil	2011	Yams	2
27	Bulgaria	1992	Apples	50
27	Bulgaria	1992	Bananas	6
27	Bulgaria	1992	Barley	4
27	Bulgaria	1992	Beans	8
27	Bulgaria	1992	Beer	119
27	Bulgaria	1992	Beverages, Alcoholic	29
27	Bulgaria	1992	Bovine Meat	41
27	Bulgaria	1992	Butter, Ghee	3
27	Bulgaria	1992	Cephalopods	2
27	Bulgaria	1992	Cheese	28
27	Bulgaria	1992	Citrus, Other	4
27	Bulgaria	1992	Cocoa Beans	3
27	Bulgaria	1992	Coffee	4
27	Bulgaria	1992	Eggs	26
27	Bulgaria	1992	Fats, Animals, Raw	21
27	Bulgaria	1992	Freshwater Fish	3
27	Bulgaria	1992	Fruits, Other	100
27	Bulgaria	1992	Grapes	27
27	Bulgaria	1992	Groundnut Oil	1
27	Bulgaria	1992	Groundnuts (Shelled Eq)	1
27	Bulgaria	1992	Honey	1
27	Bulgaria	1992	Lemons, Limes	2
27	Bulgaria	1992	Maize	4
27	Bulgaria	1992	Meat, Other	1
27	Bulgaria	1992	Milk, Whole	218
27	Bulgaria	1992	Mutton & Goat Meat	17
27	Bulgaria	1992	Nuts	6
27	Bulgaria	1992	Offals, Edible	21
27	Bulgaria	1992	Olives	2
27	Bulgaria	1992	Onions	29
27	Bulgaria	1992	Oranges, Mandarines	8
27	Bulgaria	1992	Peas	3
27	Bulgaria	1992	Pelagic Fish	2
27	Bulgaria	1992	Pigmeat	64
27	Bulgaria	1992	Potatoes	86
27	Bulgaria	1992	Poultry Meat	23
27	Bulgaria	1992	Rice (Milled Equivalent)	13
27	Bulgaria	1992	Rye	1
27	Bulgaria	1992	Sesameseed	2
27	Bulgaria	1992	Soyabean Oil	1
27	Bulgaria	1992	Sugar, Refined Equivalent	80
27	Bulgaria	1992	Sunflowerseed	5
27	Bulgaria	1992	Sunflowerseed Oil	35
27	Bulgaria	1992	Sweeteners, Other	2
27	Bulgaria	1992	Tomatoes	109
27	Bulgaria	1992	Vegetables, Other	184
27	Bulgaria	1992	Wheat	428
27	Bulgaria	1992	Wine	31
27	Bulgaria	1993	Apples	36
27	Bulgaria	1993	Bananas	12
27	Bulgaria	1993	Barley	4
27	Bulgaria	1993	Beans	8
27	Bulgaria	1993	Beer	137
27	Bulgaria	1993	Beverages, Alcoholic	32
27	Bulgaria	1993	Bovine Meat	44
27	Bulgaria	1993	Butter, Ghee	3
27	Bulgaria	1993	Cheese	31
27	Bulgaria	1993	Citrus, Other	10
27	Bulgaria	1993	Cocoa Beans	3
27	Bulgaria	1993	Coffee	5
27	Bulgaria	1993	Eggs	27
27	Bulgaria	1993	Fats, Animals, Raw	14
27	Bulgaria	1993	Freshwater Fish	3
27	Bulgaria	1993	Fruits, Other	58
27	Bulgaria	1993	Grapefruit	1
27	Bulgaria	1993	Grapes	15
27	Bulgaria	1993	Groundnut Oil	1
27	Bulgaria	1993	Groundnuts (Shelled Eq)	1
27	Bulgaria	1993	Honey	1
27	Bulgaria	1993	Lemons, Limes	3
27	Bulgaria	1993	Maize	4
27	Bulgaria	1993	Meat, Other	2
27	Bulgaria	1993	Milk, Whole	229
27	Bulgaria	1993	Mutton & Goat Meat	17
27	Bulgaria	1993	Nuts	5
27	Bulgaria	1993	Offals, Edible	19
27	Bulgaria	1993	Oilcrops Oil, Other	1
27	Bulgaria	1993	Olives	2
27	Bulgaria	1993	Onions	15
27	Bulgaria	1993	Oranges, Mandarines	14
27	Bulgaria	1993	Peas	3
27	Bulgaria	1993	Pelagic Fish	1
27	Bulgaria	1993	Pigmeat	81
27	Bulgaria	1993	Potatoes	80
27	Bulgaria	1993	Poultry Meat	28
27	Bulgaria	1993	Pulses, Other	1
27	Bulgaria	1993	Rice (Milled Equivalent)	10
27	Bulgaria	1993	Rye	1
27	Bulgaria	1993	Soyabean Oil	1
27	Bulgaria	1993	Spices, Other	1
27	Bulgaria	1993	Sugar, Refined Equivalent	79
27	Bulgaria	1993	Sunflowerseed	4
27	Bulgaria	1993	Sunflowerseed Oil	33

FAO countrycode	Country	Reference year	Food item	Gross food supply (g *capita*$^{-1}$ d^{-1})
27	Bulgaria	1993	Sweeteners, Other	1
27	Bulgaria	1993	Tomatoes	121
27	Bulgaria	1993	Vegetables, Other	170
27	Bulgaria	1993	Wheat	423
27	Bulgaria	1993	Wine	32
27	Bulgaria	1994	Apples	27
27	Bulgaria	1994	Bananas	15
27	Bulgaria	1994	Barley	5
27	Bulgaria	1994	Beans	7
27	Bulgaria	1994	Beer	154
27	Bulgaria	1994	Beverages, Alcoholic	32
27	Bulgaria	1994	Bovine Meat	35
27	Bulgaria	1994	Butter, Ghee	2
27	Bulgaria	1994	Cheese	24
27	Bulgaria	1994	Citrus, Other	2
27	Bulgaria	1994	Cocoa Beans	3
27	Bulgaria	1994	Coffee	7
27	Bulgaria	1994	Eggs	29
27	Bulgaria	1994	Fats, Animals, Raw	12
27	Bulgaria	1994	Freshwater Fish	2
27	Bulgaria	1994	Fruits, Other	75
27	Bulgaria	1994	Grapefruit	1
27	Bulgaria	1994	Grapes	14
27	Bulgaria	1994	Groundnut Oil	1
27	Bulgaria	1994	Groundnuts (Shelled Eq)	1
27	Bulgaria	1994	Lemons, Limes	2
27	Bulgaria	1994	Maize	8
27	Bulgaria	1994	Meat, Other	2
27	Bulgaria	1994	Milk, Whole	250
27	Bulgaria	1994	Mutton & Goat Meat	14
27	Bulgaria	1994	Nuts	4
27	Bulgaria	1994	Offals, Edible	15
27	Bulgaria	1994	Oilcrops Oil, Other	1
27	Bulgaria	1994	Olives	2
27	Bulgaria	1994	Onions	23
27	Bulgaria	1994	Oranges, Mandarines	12
27	Bulgaria	1994	Peas	3
27	Bulgaria	1994	Pelagic Fish	1
27	Bulgaria	1994	Pigmeat	78
27	Bulgaria	1994	Potatoes	80
27	Bulgaria	1994	Poultry Meat	28
27	Bulgaria	1994	Pulses, Other	1
27	Bulgaria	1994	Rice (Milled Equivalent)	13
27	Bulgaria	1994	Rye	1
27	Bulgaria	1994	Soyabean Oil	1
27	Bulgaria	1994	Sugar, Refined Equivalent	74
27	Bulgaria	1994	Sunflowerseed	5
27	Bulgaria	1994	Sunflowerseed Oil	29
27	Bulgaria	1994	Sweeteners, Other	2
27	Bulgaria	1994	Tomatoes	114
27	Bulgaria	1994	Vegetables, Other	183
27	Bulgaria	1994	Wheat	419
27	Bulgaria	1994	Wine	28
27	Bulgaria	1995	Apples	43
27	Bulgaria	1995	Bananas	10
27	Bulgaria	1995	Barley	5
27	Bulgaria	1995	Beans	8
27	Bulgaria	1995	Beer	138
27	Bulgaria	1995	Beverages, Alcoholic	26
27	Bulgaria	1995	Bovine Meat	29
27	Bulgaria	1995	Butter, Ghee	1
27	Bulgaria	1995	Cheese	24
27	Bulgaria	1995	Citrus, Other	1
27	Bulgaria	1995	Cocoa Beans	4
27	Bulgaria	1995	Coffee	8
27	Bulgaria	1995	Eggs	31
27	Bulgaria	1995	Fats, Animals, Raw	11
27	Bulgaria	1995	Freshwater Fish	1
27	Bulgaria	1995	Fruits, Other	82
27	Bulgaria	1995	Grapefruit	1
27	Bulgaria	1995	Grapes	30
27	Bulgaria	1995	Groundnut Oil	1
27	Bulgaria	1995	Groundnuts (Shelled Eq)	1
27	Bulgaria	1995	Lemons, Limes	3
27	Bulgaria	1995	Maize	9
27	Bulgaria	1995	Meat, Other	2
27	Bulgaria	1995	Milk, Whole	259
27	Bulgaria	1995	Mutton & Goat Meat	14
27	Bulgaria	1995	Nuts	1
27	Bulgaria	1995	Offals, Edible	16
27	Bulgaria	1995	Oilcrops Oil, Other	1
27	Bulgaria	1995	Olives	2
27	Bulgaria	1995	Onions	33
27	Bulgaria	1995	Oranges, Mandarines	14
27	Bulgaria	1995	Peas	3
27	Bulgaria	1995	Pigmeat	85
27	Bulgaria	1995	Potatoes	82
27	Bulgaria	1995	Poultry Meat	30
27	Bulgaria	1995	Pulses, Other	1
27	Bulgaria	1995	Rice (Milled Equivalent)	11
27	Bulgaria	1995	Rye	1
27	Bulgaria	1995	Soyabean Oil	1
27	Bulgaria	1995	Sugar, Refined Equivalent	74
27	Bulgaria	1995	Sunflowerseed	5
27	Bulgaria	1995	Sunflowerseed Oil	30
27	Bulgaria	1995	Sweeteners, Other	2
27	Bulgaria	1995	Tomatoes	100

FAO countrycode	Country	Reference year	Food item	Gross food supply (g *capita*$^{-1}$ d^{-1})
27	Bulgaria	1995	Vegetables, Other	222
27	Bulgaria	1995	Wheat	418
27	Bulgaria	1995	Wine	28
27	Bulgaria	1996	Apples	48
27	Bulgaria	1996	Bananas	6
27	Bulgaria	1996	Barley	6
27	Bulgaria	1996	Beans	6
27	Bulgaria	1996	Beer	136
27	Bulgaria	1996	Beverages, Alcoholic	20
27	Bulgaria	1996	Bovine Meat	26
27	Bulgaria	1996	Butter, Ghee	1
27	Bulgaria	1996	Cheese	21
27	Bulgaria	1996	Cocoa Beans	3
27	Bulgaria	1996	Coffee	5
27	Bulgaria	1996	Eggs	31
27	Bulgaria	1996	Fats, Animals, Raw	9
27	Bulgaria	1996	Freshwater Fish	1
27	Bulgaria	1996	Fruits, Other	79
27	Bulgaria	1996	Grapefruit	1
27	Bulgaria	1996	Grapes	24
27	Bulgaria	1996	Groundnut Oil	1
27	Bulgaria	1996	Groundnuts (Shelled Eq)	1
27	Bulgaria	1996	Lemons, Limes	2
27	Bulgaria	1996	Maize	11
27	Bulgaria	1996	Meat, Other	2
27	Bulgaria	1996	Milk, Whole	265
27	Bulgaria	1996	Molluscs, Other	1
27	Bulgaria	1996	Mutton & Goat Meat	17
27	Bulgaria	1996	Nuts	1
27	Bulgaria	1996	Offals, Edible	16
27	Bulgaria	1996	Oilcrops Oil, Other	1
27	Bulgaria	1996	Olives	1
27	Bulgaria	1996	Onions	28
27	Bulgaria	1996	Oranges, Mandarines	7
27	Bulgaria	1996	Peas	3
27	Bulgaria	1996	Pelagic Fish	1
27	Bulgaria	1996	Pigmeat	79
27	Bulgaria	1996	Potatoes	84
27	Bulgaria	1996	Poultry Meat	34
27	Bulgaria	1996	Pulses, Other	1
27	Bulgaria	1996	Rice (Milled Equivalent)	12
27	Bulgaria	1996	Rye	1
27	Bulgaria	1996	Spices, Other	1
27	Bulgaria	1996	Sugar, Refined Equivalent	69
27	Bulgaria	1996	Sunflowerseed	5
27	Bulgaria	1996	Sunflowerseed Oil	31
27	Bulgaria	1996	Sweeteners, Other	2
27	Bulgaria	1996	Tomatoes	96
27	Bulgaria	1996	Vegetables, Other	206
27	Bulgaria	1996	Wheat	390
27	Bulgaria	1996	Wine	29
27	Bulgaria	1997	Apples	45
27	Bulgaria	1997	Bananas	5
27	Bulgaria	1997	Barley	6
27	Bulgaria	1997	Beans	6
27	Bulgaria	1997	Beer	99
27	Bulgaria	1997	Beverages, Alcoholic	23
27	Bulgaria	1997	Bovine Meat	26
27	Bulgaria	1997	Butter, Ghee	1
27	Bulgaria	1997	Cheese	18
27	Bulgaria	1997	Cocoa Beans	3
27	Bulgaria	1997	Coffee	6
27	Bulgaria	1997	Crustaceans	1
27	Bulgaria	1997	Eggs	27
27	Bulgaria	1997	Fats, Animals, Raw	8
27	Bulgaria	1997	Freshwater Fish	2
27	Bulgaria	1997	Fruits, Other	66
27	Bulgaria	1997	Grapes	26
27	Bulgaria	1997	Groundnut Oil	1
27	Bulgaria	1997	Groundnuts (Shelled Eq)	1
27	Bulgaria	1997	Lemons, Limes	1
27	Bulgaria	1997	Maize	12
27	Bulgaria	1997	Marine Fish, Other	1
27	Bulgaria	1997	Meat, Other	4
27	Bulgaria	1997	Milk, Whole	300
27	Bulgaria	1997	Molluscs, Other	1
27	Bulgaria	1997	Mutton & Goat Meat	16
27	Bulgaria	1997	Nuts	1
27	Bulgaria	1997	Offals, Edible	15
27	Bulgaria	1997	Oilcrops Oil, Other	1
27	Bulgaria	1997	Olives	1
27	Bulgaria	1997	Onions	22
27	Bulgaria	1997	Oranges, Mandarines	7
27	Bulgaria	1997	Peas	3
27	Bulgaria	1997	Pelagic Fish	3
27	Bulgaria	1997	Pigmeat	75
27	Bulgaria	1997	Potatoes	77
27	Bulgaria	1997	Poultry Meat	37
27	Bulgaria	1997	Pulses, Other	2
27	Bulgaria	1997	Rice (Milled Equivalent)	10
27	Bulgaria	1997	Rye	2
27	Bulgaria	1997	Soyabean Oil	1
27	Bulgaria	1997	Spices, Other	1
27	Bulgaria	1997	Sugar, Refined Equivalent	71
27	Bulgaria	1997	Sunflowerseed	4
27	Bulgaria	1997	Sunflowerseed Oil	31
27	Bulgaria	1997	Sweeteners, Other	2

FAO country code	Country	Reference year	Food item	Gross food supply (g *capita*$^{-1}$ d^{-1})
27	Bulgaria	1997	Tomatoes	84
27	Bulgaria	1997	Vegetables, Other	237
27	Bulgaria	1997	Wheat	378
27	Bulgaria	1997	Wine	20
27	Bulgaria	1998	Apples	32
27	Bulgaria	1998	Bananas	9
27	Bulgaria	1998	Barley	5
27	Bulgaria	1998	Beans	7
27	Bulgaria	1998	Beer	126
27	Bulgaria	1998	Beverages, Alcoholic	22
27	Bulgaria	1998	Bovine Meat	28
27	Bulgaria	1998	Butter, Ghee	1
27	Bulgaria	1998	Cheese	21
27	Bulgaria	1998	Cocoa Beans	3
27	Bulgaria	1998	Coffee	6
27	Bulgaria	1998	Crustaceans	1
27	Bulgaria	1998	Demersal Fish	1
27	Bulgaria	1998	Eggs	31
27	Bulgaria	1998	Fats, Animals, Raw	8
27	Bulgaria	1998	Freshwater Fish	2
27	Bulgaria	1998	Fruits, Other	58
27	Bulgaria	1998	Grapefruit	1
27	Bulgaria	1998	Grapes	15
27	Bulgaria	1998	Groundnut Oil	1
27	Bulgaria	1998	Groundnuts (Shelled Eq)	1
27	Bulgaria	1998	Honey	1
27	Bulgaria	1998	Lemons, Limes	2
27	Bulgaria	1998	Maize	19
27	Bulgaria	1998	Marine Fish, Other	1
27	Bulgaria	1998	Meat, Other	2
27	Bulgaria	1998	Milk, Whole	318
27	Bulgaria	1998	Molluscs, Other	1
27	Bulgaria	1998	Mutton & Goat Meat	16
27	Bulgaria	1998	Nuts	1
27	Bulgaria	1998	Offals, Edible	18
27	Bulgaria	1998	Olives	2
27	Bulgaria	1998	Onions	29
27	Bulgaria	1998	Oranges, Mandarines	9
27	Bulgaria	1998	Peas	2
27	Bulgaria	1998	Pelagic Fish	9
27	Bulgaria	1998	Pigmeat	90
27	Bulgaria	1998	Potatoes	88
27	Bulgaria	1998	Poultry Meat	43
27	Bulgaria	1998	Pulses, Other	1
27	Bulgaria	1998	Rice (Milled Equivalent)	7
27	Bulgaria	1998	Rye	2
27	Bulgaria	1998	Soyabean Oil	2
27	Bulgaria	1998	Sugar, Refined Equivalent	69
27	Bulgaria	1998	Sunflowerseed	4
27	Bulgaria	1998	Sunflowerseed Oil	35
27	Bulgaria	1998	Sweeteners, Other	2
27	Bulgaria	1998	Tomatoes	132
27	Bulgaria	1998	Vegetables, Other	289
27	Bulgaria	1998	Wheat	384
27	Bulgaria	1998	Wine	9
27	Bulgaria	1999	Apples	30
27	Bulgaria	1999	Bananas	12
27	Bulgaria	1999	Barley	5
27	Bulgaria	1999	Beans	7
27	Bulgaria	1999	Beer	138
27	Bulgaria	1999	Beverages, Alcoholic	22
27	Bulgaria	1999	Bovine Meat	29
27	Bulgaria	1999	Butter, Ghee	1
27	Bulgaria	1999	Cheese	21
27	Bulgaria	1999	Cocoa Beans	4
27	Bulgaria	1999	Coffee	7
27	Bulgaria	1999	Eggs	29
27	Bulgaria	1999	Fats, Animals, Raw	8
27	Bulgaria	1999	Freshwater Fish	3
27	Bulgaria	1999	Fruits, Other	61
27	Bulgaria	1999	Grapefruit	2
27	Bulgaria	1999	Grapes	14
27	Bulgaria	1999	Groundnut Oil	1
27	Bulgaria	1999	Groundnuts (Shelled Eq)	1
27	Bulgaria	1999	Lemons, Limes	2
27	Bulgaria	1999	Maize	32
27	Bulgaria	1999	Maize Germ Oil	1
27	Bulgaria	1999	Marine Fish, Other	1
27	Bulgaria	1999	Meat, Other	2
27	Bulgaria	1999	Milk, Whole	308
27	Bulgaria	1999	Molluscs, Other	1
27	Bulgaria	1999	Mutton & Goat Meat	18
27	Bulgaria	1999	Nuts	1
27	Bulgaria	1999	Offals, Edible	18
27	Bulgaria	1999	Olives	3
27	Bulgaria	1999	Onions	28
27	Bulgaria	1999	Oranges, Mandarines	15
27	Bulgaria	1999	Peas	3
27	Bulgaria	1999	Pelagic Fish	6
27	Bulgaria	1999	Pigmeat	67
27	Bulgaria	1999	Potatoes	90
27	Bulgaria	1999	Poultry Meat	41
27	Bulgaria	1999	Pulses, Other	1
27	Bulgaria	1999	Rice (Milled Equivalent)	6
27	Bulgaria	1999	Rye	2
27	Bulgaria	1999	Soyabean Oil	1
27	Bulgaria	1999	Sugar, Refined Equivalent	73

FAO country code	Country	Reference year	Food item	Gross food supply (g *capita*$^{-1}$ d^{-1})
27	Bulgaria	1999	Sunflowerseed	4
27	Bulgaria	1999	Sunflowerseed Oil	34
27	Bulgaria	1999	Sweeteners, Other	2
27	Bulgaria	1999	Tomatoes	117
27	Bulgaria	1999	Vegetables, Other	269
27	Bulgaria	1999	Wheat	399
27	Bulgaria	1999	Wine	29
27	Bulgaria	2000	Apples	32
27	Bulgaria	2000	Bananas	10
27	Bulgaria	2000	Barley	6
27	Bulgaria	2000	Beans	5
27	Bulgaria	2000	Beer	141
27	Bulgaria	2000	Beverages, Alcoholic	19
27	Bulgaria	2000	Bovine Meat	24
27	Bulgaria	2000	Butter, Ghee	1
27	Bulgaria	2000	Cheese	24
27	Bulgaria	2000	Cocoa Beans	2
27	Bulgaria	2000	Coffee	6
27	Bulgaria	2000	Eggs	27
27	Bulgaria	2000	Fats, Animals, Raw	9
27	Bulgaria	2000	Freshwater Fish	2
27	Bulgaria	2000	Fruits, Other	61
27	Bulgaria	2000	Grapefruit	1
27	Bulgaria	2000	Grapes	22
27	Bulgaria	2000	Groundnut Oil	1
27	Bulgaria	2000	Groundnuts (Shelled Eq)	1
27	Bulgaria	2000	Honey	1
27	Bulgaria	2000	Lemons, Limes	3
27	Bulgaria	2000	Maize	37
27	Bulgaria	2000	Maize Germ Oil	1
27	Bulgaria	2000	Marine Fish, Other	1
27	Bulgaria	2000	Meat, Other	2
27	Bulgaria	2000	Milk, Whole	291
27	Bulgaria	2000	Mutton & Goat Meat	18
27	Bulgaria	2000	Nuts	1
27	Bulgaria	2000	Offals, Edible	17
27	Bulgaria	2000	Oilcrops Oil, Other	1
27	Bulgaria	2000	Olives	2
27	Bulgaria	2000	Onions	21
27	Bulgaria	2000	Oranges, Mandarines	12
27	Bulgaria	2000	Peas	2
27	Bulgaria	2000	Pelagic Fish	6
27	Bulgaria	2000	Pigmeat	67
27	Bulgaria	2000	Potatoes	88
27	Bulgaria	2000	Poultry Meat	45
27	Bulgaria	2000	Pulses, Other	1
27	Bulgaria	2000	Rice (Milled Equivalent)	9
27	Bulgaria	2000	Rye	2
27	Bulgaria	2000	Soyabean Oil	2
27	Bulgaria	2000	Spices, Other	1
27	Bulgaria	2000	Sugar, Refined Equivalent	71
27	Bulgaria	2000	Sunflowerseed	4
27	Bulgaria	2000	Sunflowerseed Oil	33
27	Bulgaria	2000	Sweeteners, Other	2
27	Bulgaria	2000	Tomatoes	114
27	Bulgaria	2000	Vegetables, Other	254
27	Bulgaria	2000	Wheat	375
27	Bulgaria	2000	Wine	30
27	Bulgaria	2001	Apples	20
27	Bulgaria	2001	Bananas	10
27	Bulgaria	2001	Barley	5
27	Bulgaria	2001	Beans	6
27	Bulgaria	2001	Beer	142
27	Bulgaria	2001	Beverages, Alcoholic	20
27	Bulgaria	2001	Bovine Meat	30
27	Bulgaria	2001	Butter, Ghee	1
27	Bulgaria	2001	Cheese	20
27	Bulgaria	2001	Cocoa Beans	4
27	Bulgaria	2001	Coffee	7
27	Bulgaria	2001	Demersal Fish	1
27	Bulgaria	2001	Eggs	27
27	Bulgaria	2001	Fats, Animals, Raw	9
27	Bulgaria	2001	Freshwater Fish	2
27	Bulgaria	2001	Fruits, Other	47
27	Bulgaria	2001	Grapefruit	1
27	Bulgaria	2001	Grapes	46
27	Bulgaria	2001	Groundnut Oil	1
27	Bulgaria	2001	Groundnuts (Shelled Eq)	1
27	Bulgaria	2001	Honey	1
27	Bulgaria	2001	Lemons, Limes	3
27	Bulgaria	2001	Maize	34
27	Bulgaria	2001	Marine Fish, Other	1
27	Bulgaria	2001	Meat, Other	2
27	Bulgaria	2001	Milk, Whole	247
27	Bulgaria	2001	Mutton & Goat Meat	16
27	Bulgaria	2001	Nuts	2
27	Bulgaria	2001	Offals, Edible	19
27	Bulgaria	2001	Oilcrops Oil, Other	1
27	Bulgaria	2001	Olives	2
27	Bulgaria	2001	Onions	16
27	Bulgaria	2001	Oranges, Mandarines	12
27	Bulgaria	2001	Peas	3
27	Bulgaria	2001	Pelagic Fish	6
27	Bulgaria	2001	Pigmeat	56
27	Bulgaria	2001	Potatoes	95
27	Bulgaria	2001	Poultry Meat	49
27	Bulgaria	2001	Pulses, Other	2

FAO countrycode	Country	Reference year	Food item	Gross food supply (g $capita^{-1}$ d^{-1})
27	Bulgaria	2001	Rice (Milled Equivalent)	10
27	Bulgaria	2001	Rye	2
27	Bulgaria	2001	Soyabean Oil	3
27	Bulgaria	2001	Sugar, Refined Equivalent	77
27	Bulgaria	2001	Sunflowerseed	4
27	Bulgaria	2001	Sunflowerseed Oil	35
27	Bulgaria	2001	Sweeteners, Other	2
27	Bulgaria	2001	Tomatoes	111
27	Bulgaria	2001	Vegetables, Other	188
27	Bulgaria	2001	Wheat	373
27	Bulgaria	2001	Wine	22
27	Bulgaria	2002	Apples	14
27	Bulgaria	2002	Bananas	11
27	Bulgaria	2002	Barley	5
27	Bulgaria	2002	Beans	7
27	Bulgaria	2002	Beer	138
27	Bulgaria	2002	Beverages, Alcoholic	20
27	Bulgaria	2002	Bovine Meat	18
27	Bulgaria	2002	Butter, Ghee	1
27	Bulgaria	2002	Cheese	23
27	Bulgaria	2002	Citrus, Other	2
27	Bulgaria	2002	Cocoa Beans	4
27	Bulgaria	2002	Coffee	7
27	Bulgaria	2002	Demersal Fish	1
27	Bulgaria	2002	Eggs	30
27	Bulgaria	2002	Fats, Animals, Raw	7
27	Bulgaria	2002	Freshwater Fish	1
27	Bulgaria	2002	Fruits, Other	34
27	Bulgaria	2002	Grapefruit	2
27	Bulgaria	2002	Grapes	43
27	Bulgaria	2002	Groundnut Oil	1
27	Bulgaria	2002	Groundnuts (Shelled Eq)	1
27	Bulgaria	2002	Lemons, Limes	3
27	Bulgaria	2002	Maize	35
27	Bulgaria	2002	Maize Germ Oil	1
27	Bulgaria	2002	Meat, Other	2
27	Bulgaria	2002	Milk, Whole	259
27	Bulgaria	2002	Mutton & Goat Meat	16
27	Bulgaria	2002	Nuts	2
27	Bulgaria	2002	Offals, Edible	8
27	Bulgaria	2002	Olives	3
27	Bulgaria	2002	Onions	13
27	Bulgaria	2002	Oranges, Mandarines	12
27	Bulgaria	2002	Peas	3
27	Bulgaria	2002	Pelagic Fish	9
27	Bulgaria	2002	Pigmeat	55
27	Bulgaria	2002	Potatoes	96
27	Bulgaria	2002	Poultry Meat	57
27	Bulgaria	2002	Pulses, Other	1
27	Bulgaria	2002	Rice (Milled Equivalent)	9
27	Bulgaria	2002	Rye	2
27	Bulgaria	2002	Soyabean Oil	5
27	Bulgaria	2002	Sugar, Refined Equivalent	76
27	Bulgaria	2002	Sunflowerseed	4
27	Bulgaria	2002	Sunflowerseed Oil	36
27	Bulgaria	2002	Sweeteners, Other	2
27	Bulgaria	2002	Tomatoes	79
27	Bulgaria	2002	Vegetables, Other	193
27	Bulgaria	2002	Wheat	366
27	Bulgaria	2002	Wine	24
27	Bulgaria	2003	Apples	11
27	Bulgaria	2003	Bananas	16
27	Bulgaria	2003	Barley	4
27	Bulgaria	2003	Beans	8
27	Bulgaria	2003	Beer	155
27	Bulgaria	2003	Beverages, Alcoholic	22
27	Bulgaria	2003	Bovine Meat	20
27	Bulgaria	2003	Butter, Ghee	1
27	Bulgaria	2003	Cheese	24
27	Bulgaria	2003	Citrus, Other	2
27	Bulgaria	2003	Cocoa Beans	5
27	Bulgaria	2003	Coffee	8
27	Bulgaria	2003	Demersal Fish	1
27	Bulgaria	2003	Eggs	31
27	Bulgaria	2003	Fats, Animals, Raw	8
27	Bulgaria	2003	Freshwater Fish	1
27	Bulgaria	2003	Fruits, Other	33
27	Bulgaria	2003	Grapefruit	2
27	Bulgaria	2003	Grapes	22
27	Bulgaria	2003	Groundnuts (Shelled Eq)	1
27	Bulgaria	2003	Honey	1
27	Bulgaria	2003	Lemons, Limes	3
27	Bulgaria	2003	Maize	38
27	Bulgaria	2003	Marine Fish, Other	1
27	Bulgaria	2003	Meat, Other	2
27	Bulgaria	2003	Milk, Whole	256
27	Bulgaria	2003	Mutton & Goat Meat	4
27	Bulgaria	2003	Nuts	2
27	Bulgaria	2003	Offals, Edible	8
27	Bulgaria	2003	Olives	3
27	Bulgaria	2003	Onions	12
27	Bulgaria	2003	Oranges, Mandarines	13
27	Bulgaria	2003	Peas	1
27	Bulgaria	2003	Pelagic Fish	10
27	Bulgaria	2003	Pigmeat	58
27	Bulgaria	2003	Potatoes	98
27	Bulgaria	2003	Poultry Meat	39

FAO country code	Country	Reference year	Food item	Gross food supply (g *capita*$^{-1}$ d^{-1})
27	Bulgaria	2003	Pulses, Other	1
27	Bulgaria	2003	Rice (Milled Equivalent)	10
27	Bulgaria	2003	Rye	2
27	Bulgaria	2003	Soyabean Oil	5
27	Bulgaria	2003	Sugar, Refined Equivalent	74
27	Bulgaria	2003	Sunflowerseed	5
27	Bulgaria	2003	Sunflowerseed Oil	40
27	Bulgaria	2003	Sweeteners, Other	2
27	Bulgaria	2003	Tomatoes	121
27	Bulgaria	2003	Vegetables, Other	233
27	Bulgaria	2003	Wheat	346
27	Bulgaria	2003	Wine	21
27	Bulgaria	2004	Apples	15
27	Bulgaria	2004	Bananas	17
27	Bulgaria	2004	Barley	4
27	Bulgaria	2004	Beans	6
27	Bulgaria	2004	Beer	140
27	Bulgaria	2004	Beverages, Alcoholic	21
27	Bulgaria	2004	Bovine Meat	26
27	Bulgaria	2004	Butter, Ghee	1
27	Bulgaria	2004	Cheese	24
27	Bulgaria	2004	Citrus, Other	3
27	Bulgaria	2004	Cocoa Beans	4
27	Bulgaria	2004	Coffee	9
27	Bulgaria	2004	Demersal Fish	1
27	Bulgaria	2004	Eggs	33
27	Bulgaria	2004	Fats, Animals, Raw	9
27	Bulgaria	2004	Freshwater Fish	1
27	Bulgaria	2004	Fruits, Other	41
27	Bulgaria	2004	Grapefruit	2
27	Bulgaria	2004	Grapes	19
27	Bulgaria	2004	Groundnut Oil	2
27	Bulgaria	2004	Groundnuts (Shelled Eq)	1
27	Bulgaria	2004	Honey	1
27	Bulgaria	2004	Lemons, Limes	3
27	Bulgaria	2004	Maize	50
27	Bulgaria	2004	Marine Fish, Other	1
27	Bulgaria	2004	Meat, Other	2
27	Bulgaria	2004	Milk, Whole	269
27	Bulgaria	2004	Mutton & Goat Meat	5
27	Bulgaria	2004	Nuts	3
27	Bulgaria	2004	Offals, Edible	10
27	Bulgaria	2004	Oilcrops Oil, Other	1
27	Bulgaria	2004	Olives	3
27	Bulgaria	2004	Onions	12
27	Bulgaria	2004	Oranges, Mandarines	12
27	Bulgaria	2004	Peas	1
27	Bulgaria	2004	Pelagic Fish	7
27	Bulgaria	2004	Pigmeat	61
27	Bulgaria	2004	Pineapples	1
27	Bulgaria	2004	Potatoes	101
27	Bulgaria	2004	Poultry Meat	44
27	Bulgaria	2004	Pulses, Other	1
27	Bulgaria	2004	Rice (Milled Equivalent)	11
27	Bulgaria	2004	Rye	2
27	Bulgaria	2004	Soyabean Oil	4
27	Bulgaria	2004	Sugar, Refined Equivalent	76
27	Bulgaria	2004	Sunflowerseed	5
27	Bulgaria	2004	Sunflowerseed Oil	36
27	Bulgaria	2004	Sweeteners, Other	3
27	Bulgaria	2004	Tomatoes	79
27	Bulgaria	2004	Vegetables, Other	204
27	Bulgaria	2004	Wheat	331
27	Bulgaria	2004	Wine	25
27	Bulgaria	2005	Apples	13
27	Bulgaria	2005	Bananas	19
27	Bulgaria	2005	Barley	4
27	Bulgaria	2005	Beans	7
27	Bulgaria	2005	Beer	148
27	Bulgaria	2005	Beverages, Alcoholic	18
27	Bulgaria	2005	Bovine Meat	34
27	Bulgaria	2005	Cheese	23
27	Bulgaria	2005	Citrus, Other	1
27	Bulgaria	2005	Cocoa Beans	5
27	Bulgaria	2005	Coffee	11
27	Bulgaria	2005	Demersal Fish	1
27	Bulgaria	2005	Eggs	34
27	Bulgaria	2005	Fats, Animals, Raw	11
27	Bulgaria	2005	Freshwater Fish	1
27	Bulgaria	2005	Fruits, Other	26
27	Bulgaria	2005	Grapefruit	2
27	Bulgaria	2005	Grapes	9
27	Bulgaria	2005	Groundnut Oil	1
27	Bulgaria	2005	Groundnuts (Shelled Eq)	1
27	Bulgaria	2005	Honey	3
27	Bulgaria	2005	Lemons, Limes	4
27	Bulgaria	2005	Maize	46
27	Bulgaria	2005	Marine Fish, Other	2
27	Bulgaria	2005	Meat, Other	2
27	Bulgaria	2005	Milk, Whole	257
27	Bulgaria	2005	Mutton & Goat Meat	6
27	Bulgaria	2005	Nuts	3
27	Bulgaria	2005	Offals, Edible	11
27	Bulgaria	2005	Oilcrops Oil, Other	1
27	Bulgaria	2005	Olives	4
27	Bulgaria	2005	Onions	7
27	Bulgaria	2005	Oranges, Mandarines	14

FAO country code	Country	Reference year	Food item	Gross food supply (g *capita*$^{-1}$ d^{-1})
27	Bulgaria	2005	Peas	1
27	Bulgaria	2005	Pelagic Fish	7
27	Bulgaria	2005	Pigmeat	49
27	Bulgaria	2005	Pineapples	1
27	Bulgaria	2005	Potatoes	104
27	Bulgaria	2005	Poultry Meat	47
27	Bulgaria	2005	Pulses, Other	1
27	Bulgaria	2005	Rice (Milled Equivalent)	11
27	Bulgaria	2005	Rye	2
27	Bulgaria	2005	Soyabean Oil	4
27	Bulgaria	2005	Sugar, Refined Equivalent	82
27	Bulgaria	2005	Sunflowerseed	5
27	Bulgaria	2005	Sunflowerseed Oil	38
27	Bulgaria	2005	Sweeteners, Other	3
27	Bulgaria	2005	Tomatoes	50
27	Bulgaria	2005	Vegetables, Other	117
27	Bulgaria	2005	Wheat	320
27	Bulgaria	2005	Wine	31
27	Bulgaria	2006	Apples	10
27	Bulgaria	2006	Bananas	17
27	Bulgaria	2006	Barley	5
27	Bulgaria	2006	Beans	5
27	Bulgaria	2006	Beer	179
27	Bulgaria	2006	Beverages, Alcoholic	18
27	Bulgaria	2006	Bovine Meat	38
27	Bulgaria	2006	Butter, Ghee	1
27	Bulgaria	2006	Cheese	27
27	Bulgaria	2006	Cocoa Beans	7
27	Bulgaria	2006	Coconuts - Incl Copra	1
27	Bulgaria	2006	Coffee	12
27	Bulgaria	2006	Demersal Fish	3
27	Bulgaria	2006	Eggs	34
27	Bulgaria	2006	Fats, Animals, Raw	12
27	Bulgaria	2006	Freshwater Fish	2
27	Bulgaria	2006	Fruits, Other	35
27	Bulgaria	2006	Grapefruit	3
27	Bulgaria	2006	Grapes	7
27	Bulgaria	2006	Groundnut Oil	2
27	Bulgaria	2006	Groundnuts (Shelled Eq)	1
27	Bulgaria	2006	Honey	2
27	Bulgaria	2006	Lemons, Limes	4
27	Bulgaria	2006	Maize	48
27	Bulgaria	2006	Marine Fish, Other	1
27	Bulgaria	2006	Meat, Other	3
27	Bulgaria	2006	Milk, Whole	259
27	Bulgaria	2006	Mutton & Goat Meat	6
27	Bulgaria	2006	Nuts	3
27	Bulgaria	2006	Offals, Edible	12
27	Bulgaria	2006	Oilcrops Oil, Other	1
27	Bulgaria	2006	Olives	5
27	Bulgaria	2006	Onions	14
27	Bulgaria	2006	Oranges, Mandarines	16
27	Bulgaria	2006	Pelagic Fish	9
27	Bulgaria	2006	Pigmeat	36
27	Bulgaria	2006	Pineapples	1
27	Bulgaria	2006	Potatoes	97
27	Bulgaria	2006	Poultry Meat	49
27	Bulgaria	2006	Pulses, Other	1
27	Bulgaria	2006	Rice (Milled Equivalent)	11
27	Bulgaria	2006	Rye	2
27	Bulgaria	2006	Soyabean Oil	4
27	Bulgaria	2006	Sugar, Refined Equivalent	84
27	Bulgaria	2006	Sunflowerseed	5
27	Bulgaria	2006	Sunflowerseed Oil	32
27	Bulgaria	2006	Sweeteners, Other	4
27	Bulgaria	2006	Tomatoes	75
27	Bulgaria	2006	Vegetables, Other	164
27	Bulgaria	2006	Wheat	328
27	Bulgaria	2006	Wine	36
27	Bulgaria	2007	Apples	19
27	Bulgaria	2007	Bananas	13
27	Bulgaria	2007	Barley	5
27	Bulgaria	2007	Beans	5
27	Bulgaria	2007	Beer	201
27	Bulgaria	2007	Beverages, Alcoholic	17
27	Bulgaria	2007	Bovine Meat	14
27	Bulgaria	2007	Butter, Ghee	1
27	Bulgaria	2007	Cheese	27
27	Bulgaria	2007	Cocoa Beans	4
27	Bulgaria	2007	Coconuts - Incl Copra	1
27	Bulgaria	2007	Coffee	12
27	Bulgaria	2007	Demersal Fish	2
27	Bulgaria	2007	Eggs	31
27	Bulgaria	2007	Fats, Animals, Raw	12
27	Bulgaria	2007	Freshwater Fish	2
27	Bulgaria	2007	Fruits, Other	46
27	Bulgaria	2007	Grapefruit	3
27	Bulgaria	2007	Grapes	33
27	Bulgaria	2007	Groundnut Oil	2
27	Bulgaria	2007	Groundnuts (Shelled Eq)	1
27	Bulgaria	2007	Honey	1
27	Bulgaria	2007	Lemons, Limes	4
27	Bulgaria	2007	Maize	45
27	Bulgaria	2007	Marine Fish, Other	1
27	Bulgaria	2007	Meat, Other	3
27	Bulgaria	2007	Milk, Whole	217
27	Bulgaria	2007	Mutton & Goat Meat	5

FAO country code	Country	Reference year	Food item	Gross food supply (g *capita*$^{-1}$ d^{-1})
27	Bulgaria	2007	Nuts	2
27	Bulgaria	2007	Offals, Edible	13
27	Bulgaria	2007	Oilcrops Oil, Other	2
27	Bulgaria	2007	Olives	4
27	Bulgaria	2007	Onions	14
27	Bulgaria	2007	Oranges, Mandarines	16
27	Bulgaria	2007	Peas	1
27	Bulgaria	2007	Pelagic Fish	7
27	Bulgaria	2007	Pigmeat	54
27	Bulgaria	2007	Pineapples	2
27	Bulgaria	2007	Plantains	1
27	Bulgaria	2007	Potatoes	99
27	Bulgaria	2007	Poultry Meat	53
27	Bulgaria	2007	Pulses, Other	1
27	Bulgaria	2007	Rice (Milled Equivalent)	8
27	Bulgaria	2007	Rye	2
27	Bulgaria	2007	Soyabean Oil	6
27	Bulgaria	2007	Spices, Other	1
27	Bulgaria	2007	Sugar, Refined Equivalent	80
27	Bulgaria	2007	Sunflowerseed	5
27	Bulgaria	2007	Sunflowerseed Oil	31
27	Bulgaria	2007	Sweeteners, Other	5
27	Bulgaria	2007	Tomatoes	64
27	Bulgaria	2007	Vegetables, Other	128
27	Bulgaria	2007	Wheat	323
27	Bulgaria	2007	Wine	37
27	Bulgaria	2008	Apples	16
27	Bulgaria	2008	Bananas	12
27	Bulgaria	2008	Barley	7
27	Bulgaria	2008	Beans	5
27	Bulgaria	2008	Beer	203
27	Bulgaria	2008	Beverages, Alcoholic	18
27	Bulgaria	2008	Bovine Meat	14
27	Bulgaria	2008	Butter, Ghee	1
27	Bulgaria	2008	Cheese	24
27	Bulgaria	2008	Citrus, Other	3
27	Bulgaria	2008	Cocoa Beans	5
27	Bulgaria	2008	Coconuts - Incl Copra	2
27	Bulgaria	2008	Coffee	14
27	Bulgaria	2008	Demersal Fish	3
27	Bulgaria	2008	Eggs	29
27	Bulgaria	2008	Fats, Animals, Raw	14
27	Bulgaria	2008	Freshwater Fish	4
27	Bulgaria	2008	Fruits, Other	35
27	Bulgaria	2008	Grapefruit	3
27	Bulgaria	2008	Grapes	5
27	Bulgaria	2008	Groundnut Oil	2
27	Bulgaria	2008	Groundnuts (Shelled Eq)	1
27	Bulgaria	2008	Honey	3
27	Bulgaria	2008	Lemons, Limes	3
27	Bulgaria	2008	Maize	68
27	Bulgaria	2008	Maize Germ Oil	1
27	Bulgaria	2008	Marine Fish, Other	1
27	Bulgaria	2008	Meat, Other	6
27	Bulgaria	2008	Milk, Whole	223
27	Bulgaria	2008	Mutton & Goat Meat	6
27	Bulgaria	2008	Nuts	4
27	Bulgaria	2008	Oats	1
27	Bulgaria	2008	Offals, Edible	13
27	Bulgaria	2008	Oilcrops Oil, Other	3
27	Bulgaria	2008	Olives	7
27	Bulgaria	2008	Onions	14
27	Bulgaria	2008	Oranges, Mandarines	12
27	Bulgaria	2008	Peas	1
27	Bulgaria	2008	Pelagic Fish	9
27	Bulgaria	2008	Pigmeat	61
27	Bulgaria	2008	Pimento	1
27	Bulgaria	2008	Pineapples	2
27	Bulgaria	2008	Plantains	2
27	Bulgaria	2008	Potatoes	110
27	Bulgaria	2008	Poultry Meat	55
27	Bulgaria	2008	Pulses, Other	1
27	Bulgaria	2008	Rice (Milled Equivalent)	6
27	Bulgaria	2008	Rye	2
27	Bulgaria	2008	Soyabean Oil	6
27	Bulgaria	2008	Spices, Other	1
27	Bulgaria	2008	Sugar, Refined Equivalent	77
27	Bulgaria	2008	Sunflowerseed	5
27	Bulgaria	2008	Sunflowerseed Oil	16
27	Bulgaria	2008	Sweeteners, Other	5
27	Bulgaria	2008	Tomatoes	65
27	Bulgaria	2008	Vegetables, Other	133
27	Bulgaria	2008	Wheat	320
27	Bulgaria	2008	Wine	41
27	Bulgaria	2009	Apples	19
27	Bulgaria	2009	Bananas	7
27	Bulgaria	2009	Barley	2
27	Bulgaria	2009	Beans	6
27	Bulgaria	2009	Beer	189
27	Bulgaria	2009	Beverages, Alcoholic	18
27	Bulgaria	2009	Bovine Meat	14
27	Bulgaria	2009	Cheese	24
27	Bulgaria	2009	Citrus, Other	4
27	Bulgaria	2009	Cocoa Beans	8
27	Bulgaria	2009	Coconuts - Incl Copra	2
27	Bulgaria	2009	Coffee	14
27	Bulgaria	2009	Cream	1

FAO country code	Country	Reference year	Food item	Gross food supply (g $capita^{-1}$ d^{-1})
27	Bulgaria	2009	Demersal Fish	2
27	Bulgaria	2009	Eggs	27
27	Bulgaria	2009	Fats, Animals, Raw	15
27	Bulgaria	2009	Freshwater Fish	6
27	Bulgaria	2009	Fruits, Other	34
27	Bulgaria	2009	Grapefruit	4
27	Bulgaria	2009	Grapes	2
27	Bulgaria	2009	Groundnut Oil	1
27	Bulgaria	2009	Groundnuts (Shelled Eq)	1
27	Bulgaria	2009	Honey	1
27	Bulgaria	2009	Lemons, Limes	6
27	Bulgaria	2009	Maize	69
27	Bulgaria	2009	Maize Germ Oil	2
27	Bulgaria	2009	Marine Fish, Other	1
27	Bulgaria	2009	Meat, Other	5
27	Bulgaria	2009	Milk, Whole	180
27	Bulgaria	2009	Mutton & Goat Meat	5
27	Bulgaria	2009	Nuts	3
27	Bulgaria	2009	Oats	1
27	Bulgaria	2009	Offals, Edible	12
27	Bulgaria	2009	Oilcrops Oil, Other	2
27	Bulgaria	2009	Olives	9
27	Bulgaria	2009	Onions	8
27	Bulgaria	2009	Oranges, Mandarines	15
27	Bulgaria	2009	Pelagic Fish	8
27	Bulgaria	2009	Pigmeat	69
27	Bulgaria	2009	Pimento	1
27	Bulgaria	2009	Pineapples	1
27	Bulgaria	2009	Plantains	6
27	Bulgaria	2009	Potatoes	91
27	Bulgaria	2009	Poultry Meat	60
27	Bulgaria	2009	Pulses, Other	1
27	Bulgaria	2009	Rice (Milled Equivalent)	8
27	Bulgaria	2009	Rye	2
27	Bulgaria	2009	Soyabean Oil	4
27	Bulgaria	2009	Spices, Other	1
27	Bulgaria	2009	Sugar, Refined Equivalent	74
27	Bulgaria	2009	Sunflowerseed	6
27	Bulgaria	2009	Sunflowerseed Oil	34
27	Bulgaria	2009	Sweeteners, Other	5
27	Bulgaria	2009	Tea	1
27	Bulgaria	2009	Tomatoes	64
27	Bulgaria	2009	Vegetables, Other	133
27	Bulgaria	2009	Wheat	328
27	Bulgaria	2009	Wine	34
27	Bulgaria	2010	Apples	10
27	Bulgaria	2010	Bananas	8
27	Bulgaria	2010	Barley	34
27	Bulgaria	2010	Beans	6
27	Bulgaria	2010	Beer	187
27	Bulgaria	2010	Beverages, Alcoholic	20
27	Bulgaria	2010	Bovine Meat	14
27	Bulgaria	2010	Butter, Ghee	1
27	Bulgaria	2010	Cheese	23
27	Bulgaria	2010	Cocoa Beans	5
27	Bulgaria	2010	Coconuts - Incl Copra	2
27	Bulgaria	2010	Coffee	14
27	Bulgaria	2010	Demersal Fish	2
27	Bulgaria	2010	Eggs	28
27	Bulgaria	2010	Fats, Animals, Raw	15
27	Bulgaria	2010	Freshwater Fish	7
27	Bulgaria	2010	Fruits, Other	45
27	Bulgaria	2010	Grapefruit	4
27	Bulgaria	2010	Grapes	3
27	Bulgaria	2010	Groundnut Oil	1
27	Bulgaria	2010	Groundnuts (Shelled Eq)	1
27	Bulgaria	2010	Honey	1
27	Bulgaria	2010	Lemons, Limes	4
27	Bulgaria	2010	Maize	87
27	Bulgaria	2010	Maize Germ Oil	2
27	Bulgaria	2010	Meat, Other	3
27	Bulgaria	2010	Milk, Whole	197
27	Bulgaria	2010	Molluscs, Other	1
27	Bulgaria	2010	Mutton & Goat Meat	5
27	Bulgaria	2010	Nuts	2
27	Bulgaria	2010	Oats	1
27	Bulgaria	2010	Offals, Edible	11
27	Bulgaria	2010	Oilcrops Oil, Other	2
27	Bulgaria	2010	Olive Oil	1
27	Bulgaria	2010	Olives	5
27	Bulgaria	2010	Onions	13
27	Bulgaria	2010	Oranges, Mandarines	17
27	Bulgaria	2010	Pelagic Fish	8
27	Bulgaria	2010	Pigmeat	73
27	Bulgaria	2010	Pimento	1
27	Bulgaria	2010	Pineapples	1
27	Bulgaria	2010	Plantains	7
27	Bulgaria	2010	Potatoes	86
27	Bulgaria	2010	Poultry Meat	51
27	Bulgaria	2010	Pulses, Other	1
27	Bulgaria	2010	Rice (Milled Equivalent)	5
27	Bulgaria	2010	Rye	2
27	Bulgaria	2010	Soyabean Oil	5
27	Bulgaria	2010	Spices, Other	4
27	Bulgaria	2010	Sugar, Refined Equivalent	61
27	Bulgaria	2010	Sunflowerseed	6
27	Bulgaria	2010	Sunflowerseed Oil	24

FAO countrycode	Country	Reference year	Food item	Gross food supply (g *capita*$^{-1}$ d^{-1})
27	Bulgaria	2010	Sweeteners, Other	5
27	Bulgaria	2010	Tomatoes	60
27	Bulgaria	2010	Vegetables, Other	141
27	Bulgaria	2010	Wheat	318
27	Bulgaria	2010	Wine	39
27	Bulgaria	2011	Apples	17
27	Bulgaria	2011	Bananas	10
27	Bulgaria	2011	Barley	2
27	Bulgaria	2011	Beans	4
27	Bulgaria	2011	Beer	192
27	Bulgaria	2011	Beverages, Alcoholic	22
27	Bulgaria	2011	Bovine Meat	12
27	Bulgaria	2011	Butter, Ghee	1
27	Bulgaria	2011	Cheese	24
27	Bulgaria	2011	Cocoa Beans	5
27	Bulgaria	2011	Coconuts - Incl Copra	1
27	Bulgaria	2011	Coffee	12
27	Bulgaria	2011	Cream	1
27	Bulgaria	2011	Demersal Fish	2
27	Bulgaria	2011	Eggs	22
27	Bulgaria	2011	Fats, Animals, Raw	14
27	Bulgaria	2011	Freshwater Fish	7
27	Bulgaria	2011	Fruits, Other	53
27	Bulgaria	2011	Grapefruit	5
27	Bulgaria	2011	Grapes	14
27	Bulgaria	2011	Groundnut Oil	1
27	Bulgaria	2011	Groundnuts (Shelled Eq)	1
27	Bulgaria	2011	Honey	1
27	Bulgaria	2011	Lemons, Limes	4
27	Bulgaria	2011	Maize	113
27	Bulgaria	2011	Maize Germ Oil	2
27	Bulgaria	2011	Meat, Other	3
27	Bulgaria	2011	Milk, Whole	213
27	Bulgaria	2011	Molluscs, Other	1
27	Bulgaria	2011	Mutton & Goat Meat	5
27	Bulgaria	2011	Nuts	3
27	Bulgaria	2011	Oats	1
27	Bulgaria	2011	Offals, Edible	11
27	Bulgaria	2011	Oilcrops Oil, Other	2
27	Bulgaria	2011	Olive Oil	1
27	Bulgaria	2011	Olives	5
27	Bulgaria	2011	Onions	12
27	Bulgaria	2011	Oranges, Mandarines	17
27	Bulgaria	2011	Pelagic Fish	8
27	Bulgaria	2011	Pigmeat	78
27	Bulgaria	2011	Pimento	1
27	Bulgaria	2011	Pineapples	1
27	Bulgaria	2011	Plantains	6
27	Bulgaria	2011	Potatoes	85
27	Bulgaria	2011	Poultry Meat	58
27	Bulgaria	2011	Pulses, Other	1
27	Bulgaria	2011	Rice (Milled Equivalent)	9
27	Bulgaria	2011	Rye	2
27	Bulgaria	2011	Soyabean Oil	4
27	Bulgaria	2011	Spices, Other	3
27	Bulgaria	2011	Sugar, Refined Equivalent	66
27	Bulgaria	2011	Sunflowerseed	6
27	Bulgaria	2011	Sunflowerseed Oil	24
27	Bulgaria	2011	Sweet Potatoes	2
27	Bulgaria	2011	Sweeteners, Other	5
27	Bulgaria	2011	Tomatoes	50
27	Bulgaria	2011	Vegetables, Other	138
27	Bulgaria	2011	Wheat	328
27	Bulgaria	2011	Wine	26
233	Burkina Faso	1992	Beer	11
233	Burkina Faso	1992	Beverages, Fermented	647
233	Burkina Faso	1992	Bovine Meat	17
233	Burkina Faso	1992	Cereals, Other	3
233	Burkina Faso	1992	Cottonseed Oil	5
233	Burkina Faso	1992	Eggs	7
233	Burkina Faso	1992	Fats, Animals, Raw	1
233	Burkina Faso	1992	Freshwater Fish	2
233	Burkina Faso	1992	Fruits, Other	18
233	Burkina Faso	1992	Groundnut Oil	1
233	Burkina Faso	1992	Groundnuts (Shelled Eq)	23
233	Burkina Faso	1992	Maize	77
233	Burkina Faso	1992	Marine Fish, Other	2
233	Burkina Faso	1992	Meat, Other	2
233	Burkina Faso	1992	Milk, Whole	49
233	Burkina Faso	1992	Millet	219
233	Burkina Faso	1992	Mutton & Goat Meat	9
233	Burkina Faso	1992	Nuts	5
233	Burkina Faso	1992	Offals, Edible	6
233	Burkina Faso	1992	Oilcrops Oil, Other	2
233	Burkina Faso	1992	Palm Oil	1
233	Burkina Faso	1992	Pigmeat	3
233	Burkina Faso	1992	Potatoes	2
233	Burkina Faso	1992	Poultry Meat	6
233	Burkina Faso	1992	Pulses, Other	35
233	Burkina Faso	1992	Rice (Milled Equivalent)	34
233	Burkina Faso	1992	Sesameseed	1
233	Burkina Faso	1992	Sorghum	190
233	Burkina Faso	1992	Soyabean Oil	1
233	Burkina Faso	1992	Spices, Other	2
233	Burkina Faso	1992	Sugar, Refined Equivalent	10
233	Burkina Faso	1992	Sweet Potatoes	4
233	Burkina Faso	1992	Tomatoes	6

FAO country code	Country	Reference year	Food item	Gross food supply (g *capita*$^{-1}$ d^{-1})
233	Burkina Faso	1992	Vegetables, Other	60
233	Burkina Faso	1992	Wheat	12
233	Burkina Faso	1992	Yams	19
233	Burkina Faso	1993	Beer	11
233	Burkina Faso	1993	Beverages, Fermented	666
233	Burkina Faso	1993	Bovine Meat	17
233	Burkina Faso	1993	Cereals, Other	6
233	Burkina Faso	1993	Cottonseed Oil	2
233	Burkina Faso	1993	Eggs	7
233	Burkina Faso	1993	Fats, Animals, Raw	1
233	Burkina Faso	1993	Freshwater Fish	2
233	Burkina Faso	1993	Fruits, Other	18
233	Burkina Faso	1993	Groundnut Oil	2
233	Burkina Faso	1993	Groundnuts (Shelled Eq)	33
233	Burkina Faso	1993	Maize	89
233	Burkina Faso	1993	Marine Fish, Other	2
233	Burkina Faso	1993	Meat, Other	2
233	Burkina Faso	1993	Milk, Whole	50
233	Burkina Faso	1993	Millet	202
233	Burkina Faso	1993	Mutton & Goat Meat	9
233	Burkina Faso	1993	Nuts	5
233	Burkina Faso	1993	Offals, Edible	6
233	Burkina Faso	1993	Oilcrops Oil, Other	2
233	Burkina Faso	1993	Palm Oil	1
233	Burkina Faso	1993	Pigmeat	3
233	Burkina Faso	1993	Potatoes	2
233	Burkina Faso	1993	Poultry Meat	6
233	Burkina Faso	1993	Pulses, Other	32
233	Burkina Faso	1993	Rice (Milled Equivalent)	33
233	Burkina Faso	1993	Sesameseed	1
233	Burkina Faso	1993	Sorghum	196
233	Burkina Faso	1993	Soyabean Oil	1
233	Burkina Faso	1993	Soyabeans	1
233	Burkina Faso	1993	Spices, Other	3
233	Burkina Faso	1993	Sugar, Refined Equivalent	10
233	Burkina Faso	1993	Sweet Potatoes	4
233	Burkina Faso	1993	Tomatoes	6
233	Burkina Faso	1993	Vegetables, Other	61
233	Burkina Faso	1993	Wheat	10
233	Burkina Faso	1993	Yams	11
233	Burkina Faso	1994	Beans	1
233	Burkina Faso	1994	Beer	9
233	Burkina Faso	1994	Beverages, Fermented	625
233	Burkina Faso	1994	Bovine Meat	17
233	Burkina Faso	1994	Cereals, Other	4
233	Burkina Faso	1994	Coffee	2
233	Burkina Faso	1994	Cottonseed Oil	5
233	Burkina Faso	1994	Eggs	7
233	Burkina Faso	1994	Fats, Animals, Raw	1
233	Burkina Faso	1994	Freshwater Fish	2
233	Burkina Faso	1994	Fruits, Other	18
233	Burkina Faso	1994	Groundnut Oil	2
233	Burkina Faso	1994	Groundnuts (Shelled Eq)	30
233	Burkina Faso	1994	Maize	64
233	Burkina Faso	1994	Marine Fish, Other	4
233	Burkina Faso	1994	Meat, Other	2
233	Burkina Faso	1994	Milk, Whole	49
233	Burkina Faso	1994	Millet	207
233	Burkina Faso	1994	Mutton & Goat Meat	9
233	Burkina Faso	1994	Nuts	6
233	Burkina Faso	1994	Offals, Edible	6
233	Burkina Faso	1994	Oilcrops Oil, Other	3
233	Burkina Faso	1994	Palm Oil	4
233	Burkina Faso	1994	Pigmeat	3
233	Burkina Faso	1994	Potatoes	2
233	Burkina Faso	1994	Poultry Meat	6
233	Burkina Faso	1994	Pulses, Other	36
233	Burkina Faso	1994	Rice (Milled Equivalent)	55
233	Burkina Faso	1994	Roots, Other	1
233	Burkina Faso	1994	Sorghum	184
233	Burkina Faso	1994	Soyabean Oil	1
233	Burkina Faso	1994	Spices, Other	3
233	Burkina Faso	1994	Sugar, Refined Equivalent	9
233	Burkina Faso	1994	Sweet Potatoes	3
233	Burkina Faso	1994	Tomatoes	5
233	Burkina Faso	1994	Vegetables, Other	67
233	Burkina Faso	1994	Wheat	17
233	Burkina Faso	1994	Yams	9
233	Burkina Faso	1995	Beer	10
233	Burkina Faso	1995	Beverages, Alcoholic	1
233	Burkina Faso	1995	Beverages, Fermented	610
233	Burkina Faso	1995	Bovine Meat	17
233	Burkina Faso	1995	Cassava	1
233	Burkina Faso	1995	Cereals, Other	3
233	Burkina Faso	1995	Cottonseed Oil	3
233	Burkina Faso	1995	Eggs	7
233	Burkina Faso	1995	Fats, Animals, Raw	1
233	Burkina Faso	1995	Freshwater Fish	2
233	Burkina Faso	1995	Fruits, Other	19
233	Burkina Faso	1995	Groundnut Oil	2
233	Burkina Faso	1995	Groundnuts (Shelled Eq)	27
233	Burkina Faso	1995	Maize	95
233	Burkina Faso	1995	Marine Fish, Other	4
233	Burkina Faso	1995	Meat, Other	2
233	Burkina Faso	1995	Milk, Whole	55
233	Burkina Faso	1995	Millet	197
233	Burkina Faso	1995	Mutton & Goat Meat	9

FAO country code	Country	Reference year	Food item	Gross food supply (g *capita*$^{-1}$ d^{-1})
233	Burkina Faso	1995	Nuts	5
233	Burkina Faso	1995	Offals, Edible	6
233	Burkina Faso	1995	Oilcrops Oil, Other	2
233	Burkina Faso	1995	Palm Oil	4
233	Burkina Faso	1995	Pigmeat	3
233	Burkina Faso	1995	Potatoes	1
233	Burkina Faso	1995	Poultry Meat	6
233	Burkina Faso	1995	Pulses, Other	31
233	Burkina Faso	1995	Rice (Milled Equivalent)	43
233	Burkina Faso	1995	Roots, Other	1
233	Burkina Faso	1995	Sesameseed	2
233	Burkina Faso	1995	Sorghum	179
233	Burkina Faso	1995	Soyabean Oil	1
233	Burkina Faso	1995	Soyabeans	1
233	Burkina Faso	1995	Spices, Other	3
233	Burkina Faso	1995	Sugar, Refined Equivalent	8
233	Burkina Faso	1995	Sweet Potatoes	3
233	Burkina Faso	1995	Tomatoes	3
233	Burkina Faso	1995	Vegetables, Other	62
233	Burkina Faso	1995	Wheat	18
233	Burkina Faso	1995	Yams	16
233	Burkina Faso	1996	Beer	15
233	Burkina Faso	1996	Beverages, Fermented	641
233	Burkina Faso	1996	Bovine Meat	17
233	Burkina Faso	1996	Cereals, Other	2
233	Burkina Faso	1996	Cottonseed Oil	5
233	Burkina Faso	1996	Eggs	7
233	Burkina Faso	1996	Fats, Animals, Raw	1
233	Burkina Faso	1996	Freshwater Fish	2
233	Burkina Faso	1996	Fruits, Other	19
233	Burkina Faso	1996	Groundnut Oil	2
233	Burkina Faso	1996	Groundnuts (Shelled Eq)	32
233	Burkina Faso	1996	Maize	64
233	Burkina Faso	1996	Marine Fish, Other	4
233	Burkina Faso	1996	Meat, Other	2
233	Burkina Faso	1996	Milk, Whole	50
233	Burkina Faso	1996	Millet	175
233	Burkina Faso	1996	Mutton & Goat Meat	9
233	Burkina Faso	1996	Nuts	6
233	Burkina Faso	1996	Offals, Edible	6
233	Burkina Faso	1996	Oilcrops Oil, Other	2
233	Burkina Faso	1996	Palm Oil	2
233	Burkina Faso	1996	Pigmeat	4
233	Burkina Faso	1996	Poultry Meat	6
233	Burkina Faso	1996	Pulses, Other	31
233	Burkina Faso	1996	Rice (Milled Equivalent)	66
233	Burkina Faso	1996	Sesameseed	1
233	Burkina Faso	1996	Sorghum	188
233	Burkina Faso	1996	Soyabean Oil	1
233	Burkina Faso	1996	Spices, Other	3
233	Burkina Faso	1996	Sugar, Refined Equivalent	13
233	Burkina Faso	1996	Sweet Potatoes	1
233	Burkina Faso	1996	Tomatoes	4
233	Burkina Faso	1996	Vegetables, Other	78
233	Burkina Faso	1996	Wheat	22
233	Burkina Faso	1996	Yams	12
233	Burkina Faso	1997	Beans	1
233	Burkina Faso	1997	Beer	14
233	Burkina Faso	1997	Beverages, Alcoholic	1
233	Burkina Faso	1997	Beverages, Fermented	593
233	Burkina Faso	1997	Bovine Meat	17
233	Burkina Faso	1997	Cereals, Other	11
233	Burkina Faso	1997	Cottonseed Oil	9
233	Burkina Faso	1997	Eggs	7
233	Burkina Faso	1997	Fats, Animals, Raw	1
233	Burkina Faso	1997	Freshwater Fish	2
233	Burkina Faso	1997	Fruits, Other	20
233	Burkina Faso	1997	Groundnut Oil	1
233	Burkina Faso	1997	Groundnuts (Shelled Eq)	20
233	Burkina Faso	1997	Maize	92
233	Burkina Faso	1997	Marine Fish, Other	5
233	Burkina Faso	1997	Meat, Other	2
233	Burkina Faso	1997	Milk, Whole	51
233	Burkina Faso	1997	Millet	165
233	Burkina Faso	1997	Mutton & Goat Meat	9
233	Burkina Faso	1997	Nuts	6
233	Burkina Faso	1997	Offals, Edible	6
233	Burkina Faso	1997	Oilcrops Oil, Other	3
233	Burkina Faso	1997	Palm Oil	2
233	Burkina Faso	1997	Palmkernel Oil	1
233	Burkina Faso	1997	Pigmeat	4
233	Burkina Faso	1997	Poultry Meat	6
233	Burkina Faso	1997	Pulses, Other	30
233	Burkina Faso	1997	Rice (Milled Equivalent)	62
233	Burkina Faso	1997	Sorghum	174
233	Burkina Faso	1997	Soyabeans	1
233	Burkina Faso	1997	Spices, Other	3
233	Burkina Faso	1997	Sugar, Refined Equivalent	14
233	Burkina Faso	1997	Sweet Potatoes	4
233	Burkina Faso	1997	Tomatoes	3
233	Burkina Faso	1997	Vegetables, Other	78
233	Burkina Faso	1997	Wheat	17
233	Burkina Faso	1997	Yams	8
233	Burkina Faso	1998	Bananas	1
233	Burkina Faso	1998	Beer	14
233	Burkina Faso	1998	Beverages, Fermented	540
233	Burkina Faso	1998	Bovine Meat	17

FAO country code	Country	Reference year	Food item	Gross food supply (g *capita*$^{-1}$ d^{-1})
233	Burkina Faso	1998	Cereals, Other	6
233	Burkina Faso	1998	Cottonseed Oil	7
233	Burkina Faso	1998	Eggs	7
233	Burkina Faso	1998	Fats, Animals, Raw	1
233	Burkina Faso	1998	Freshwater Fish	2
233	Burkina Faso	1998	Fruits, Other	20
233	Burkina Faso	1998	Groundnut Oil	1
233	Burkina Faso	1998	Groundnuts (Shelled Eq)	29
233	Burkina Faso	1998	Maize	97
233	Burkina Faso	1998	Marine Fish, Other	3
233	Burkina Faso	1998	Meat, Other	2
233	Burkina Faso	1998	Milk, Whole	51
233	Burkina Faso	1998	Millet	184
233	Burkina Faso	1998	Mutton & Goat Meat	9
233	Burkina Faso	1998	Nuts	4
233	Burkina Faso	1998	Offals, Edible	6
233	Burkina Faso	1998	Oilcrops Oil, Other	2
233	Burkina Faso	1998	Palm Oil	1
233	Burkina Faso	1998	Pigmeat	4
233	Burkina Faso	1998	Poultry Meat	6
233	Burkina Faso	1998	Pulses, Other	31
233	Burkina Faso	1998	Rice (Milled Equivalent)	52
233	Burkina Faso	1998	Sorghum	159
233	Burkina Faso	1998	Soyabeans	1
233	Burkina Faso	1998	Spices, Other	3
233	Burkina Faso	1998	Sugar, Refined Equivalent	12
233	Burkina Faso	1998	Sweet Potatoes	3
233	Burkina Faso	1998	Tomatoes	2
233	Burkina Faso	1998	Vegetables, Other	77
233	Burkina Faso	1998	Wheat	17
233	Burkina Faso	1998	Yams	10
233	Burkina Faso	1999	Bananas	1
233	Burkina Faso	1999	Beans	1
233	Burkina Faso	1999	Beer	12
233	Burkina Faso	1999	Beverages, Alcoholic	1
233	Burkina Faso	1999	Beverages, Fermented	559
233	Burkina Faso	1999	Bovine Meat	16
233	Burkina Faso	1999	Cereals, Other	8
233	Burkina Faso	1999	Cottonseed Oil	5
233	Burkina Faso	1999	Eggs	7
233	Burkina Faso	1999	Fats, Animals, Raw	1
233	Burkina Faso	1999	Freshwater Fish	2
233	Burkina Faso	1999	Fruits, Other	20
233	Burkina Faso	1999	Groundnut Oil	1
233	Burkina Faso	1999	Groundnuts (Shelled Eq)	28
233	Burkina Faso	1999	Maize	63
233	Burkina Faso	1999	Marine Fish, Other	6
233	Burkina Faso	1999	Meat, Other	2
233	Burkina Faso	1999	Milk, Whole	55
233	Burkina Faso	1999	Millet	205
233	Burkina Faso	1999	Mutton & Goat Meat	9
233	Burkina Faso	1999	Nuts	7
233	Burkina Faso	1999	Offals, Edible	6
233	Burkina Faso	1999	Oilcrops Oil, Other	3
233	Burkina Faso	1999	Palm Oil	3
233	Burkina Faso	1999	Pigmeat	4
233	Burkina Faso	1999	Potatoes	1
233	Burkina Faso	1999	Poultry Meat	6
233	Burkina Faso	1999	Pulses, Other	34
233	Burkina Faso	1999	Rice (Milled Equivalent)	66
233	Burkina Faso	1999	Sesameseed	1
233	Burkina Faso	1999	Sorghum	164
233	Burkina Faso	1999	Soyabeans	1
233	Burkina Faso	1999	Spices, Other	3
233	Burkina Faso	1999	Sugar, Refined Equivalent	13
233	Burkina Faso	1999	Sweet Potatoes	4
233	Burkina Faso	1999	Tomatoes	3
233	Burkina Faso	1999	Vegetables, Other	78
233	Burkina Faso	1999	Wheat	20
233	Burkina Faso	1999	Yams	9
233	Burkina Faso	2000	Bananas	2
233	Burkina Faso	2000	Beans	1
233	Burkina Faso	2000	Beer	13
233	Burkina Faso	2000	Beverages, Alcoholic	1
233	Burkina Faso	2000	Beverages, Fermented	433
233	Burkina Faso	2000	Bovine Meat	16
233	Burkina Faso	2000	Cereals, Other	12
233	Burkina Faso	2000	Cottonseed Oil	3
233	Burkina Faso	2000	Eggs	7
233	Burkina Faso	2000	Fats, Animals, Raw	1
233	Burkina Faso	2000	Freshwater Fish	2
233	Burkina Faso	2000	Fruits, Other	19
233	Burkina Faso	2000	Groundnut Oil	1
233	Burkina Faso	2000	Groundnuts (Shelled Eq)	30
233	Burkina Faso	2000	Maize	127
233	Burkina Faso	2000	Marine Fish, Other	4
233	Burkina Faso	2000	Meat, Other	2
233	Burkina Faso	2000	Milk, Whole	52
233	Burkina Faso	2000	Millet	192
233	Burkina Faso	2000	Mutton & Goat Meat	9
233	Burkina Faso	2000	Nuts	4
233	Burkina Faso	2000	Offals, Edible	6
233	Burkina Faso	2000	Oilcrops Oil, Other	2
233	Burkina Faso	2000	Palm Oil	6
233	Burkina Faso	2000	Pigmeat	5
233	Burkina Faso	2000	Potatoes	1
233	Burkina Faso	2000	Poultry Meat	6

FAO country code	Country	Reference year	Food item	Gross food supply (g $capita^{-1}$ d^{-1})
233	Burkina Faso	2000	Pulses, Other	29
233	Burkina Faso	2000	Rice (Milled Equivalent)	56
233	Burkina Faso	2000	Sorghum	127
233	Burkina Faso	2000	Soyabeans	1
233	Burkina Faso	2000	Spices, Other	3
233	Burkina Faso	2000	Sugar, Refined Equivalent	8
233	Burkina Faso	2000	Sweet Potatoes	6
233	Burkina Faso	2000	Tomatoes	4
233	Burkina Faso	2000	Vegetables, Other	77
233	Burkina Faso	2000	Wheat	22
233	Burkina Faso	2000	Wine	1
233	Burkina Faso	2000	Yams	12
233	Burkina Faso	2001	Bananas	1
233	Burkina Faso	2001	Beer	11
233	Burkina Faso	2001	Beverages, Alcoholic	1
233	Burkina Faso	2001	Beverages, Fermented	562
233	Burkina Faso	2001	Bovine Meat	16
233	Burkina Faso	2001	Cassava	1
233	Burkina Faso	2001	Cereals, Other	5
233	Burkina Faso	2001	Cottonseed Oil	7
233	Burkina Faso	2001	Eggs	7
233	Burkina Faso	2001	Fats, Animals, Raw	1
233	Burkina Faso	2001	Freshwater Fish	2
233	Burkina Faso	2001	Fruits, Other	18
233	Burkina Faso	2001	Groundnut Oil	1
233	Burkina Faso	2001	Groundnuts (Shelled Eq)	30
233	Burkina Faso	2001	Maize	91
233	Burkina Faso	2001	Marine Fish, Other	1
233	Burkina Faso	2001	Meat, Other	2
233	Burkina Faso	2001	Milk, Whole	46
233	Burkina Faso	2001	Millet	194
233	Burkina Faso	2001	Mutton & Goat Meat	9
233	Burkina Faso	2001	Nuts	3
233	Burkina Faso	2001	Offals, Edible	6
233	Burkina Faso	2001	Oilcrops Oil, Other	1
233	Burkina Faso	2001	Palm Oil	2
233	Burkina Faso	2001	Pelagic Fish	1
233	Burkina Faso	2001	Pigmeat	5
233	Burkina Faso	2001	Poultry Meat	6
233	Burkina Faso	2001	Pulses, Other	32
233	Burkina Faso	2001	Rice (Milled Equivalent)	60
233	Burkina Faso	2001	Sesameseed	1
233	Burkina Faso	2001	Sorghum	165
233	Burkina Faso	2001	Soyabeans	1
233	Burkina Faso	2001	Spices, Other	3
233	Burkina Faso	2001	Sugar, Refined Equivalent	11
233	Burkina Faso	2001	Sweet Potatoes	9
233	Burkina Faso	2001	Tomatoes	2
233	Burkina Faso	2001	Vegetables, Other	75
233	Burkina Faso	2001	Wheat	11
233	Burkina Faso	2001	Yams	15
233	Burkina Faso	2002	Bananas	1
233	Burkina Faso	2002	Beer	11
233	Burkina Faso	2002	Beverages, Alcoholic	1
233	Burkina Faso	2002	Beverages, Fermented	549
233	Burkina Faso	2002	Bovine Meat	16
233	Burkina Faso	2002	Cassava	1
233	Burkina Faso	2002	Cereals, Other	3
233	Burkina Faso	2002	Cottonseed Oil	8
233	Burkina Faso	2002	Eggs	7
233	Burkina Faso	2002	Fats, Animals, Raw	1
233	Burkina Faso	2002	Freshwater Fish	2
233	Burkina Faso	2002	Fruits, Other	18
233	Burkina Faso	2002	Groundnut Oil	2
233	Burkina Faso	2002	Groundnuts (Shelled Eq)	39
233	Burkina Faso	2002	Maize	102
233	Burkina Faso	2002	Marine Fish, Other	1
233	Burkina Faso	2002	Meat, Other	2
233	Burkina Faso	2002	Milk, Whole	45
233	Burkina Faso	2002	Millet	189
233	Burkina Faso	2002	Mutton & Goat Meat	9
233	Burkina Faso	2002	Nuts	3
233	Burkina Faso	2002	Offals, Edible	6
233	Burkina Faso	2002	Oilcrops Oil, Other	1
233	Burkina Faso	2002	Palm Oil	3
233	Burkina Faso	2002	Pelagic Fish	2
233	Burkina Faso	2002	Pigmeat	6
233	Burkina Faso	2002	Poultry Meat	6
233	Burkina Faso	2002	Pulses, Other	32
233	Burkina Faso	2002	Rice (Milled Equivalent)	63
233	Burkina Faso	2002	Sesameseed	1
233	Burkina Faso	2002	Sorghum	161
233	Burkina Faso	2002	Soyabeans	1
233	Burkina Faso	2002	Spices, Other	3
233	Burkina Faso	2002	Sugar, Refined Equivalent	15
233	Burkina Faso	2002	Sweet Potatoes	7
233	Burkina Faso	2002	Tomatoes	2
233	Burkina Faso	2002	Vegetables, Other	78
233	Burkina Faso	2002	Wheat	11
233	Burkina Faso	2002	Yams	5
233	Burkina Faso	2003	Beer	10
233	Burkina Faso	2003	Beverages, Alcoholic	1
233	Burkina Faso	2003	Beverages, Fermented	565
233	Burkina Faso	2003	Bovine Meat	23
233	Burkina Faso	2003	Cassava	1
233	Burkina Faso	2003	Cereals, Other	2
233	Burkina Faso	2003	Cottonseed Oil	6

FAO country code	Country	Reference year	Food item	Gross food supply (g $capita^{-1}$ d^{-1})
233	Burkina Faso	2003	Eggs	7
233	Burkina Faso	2003	Fats, Animals, Raw	1
233	Burkina Faso	2003	Freshwater Fish	2
233	Burkina Faso	2003	Fruits, Other	15
233	Burkina Faso	2003	Groundnut Oil	2
233	Burkina Faso	2003	Groundnuts (Shelled Eq)	43
233	Burkina Faso	2003	Maize	134
233	Burkina Faso	2003	Marine Fish, Other	1
233	Burkina Faso	2003	Meat, Other	2
233	Burkina Faso	2003	Milk, Whole	52
233	Burkina Faso	2003	Millet	202
233	Burkina Faso	2003	Mutton & Goat Meat	9
233	Burkina Faso	2003	Nuts	2
233	Burkina Faso	2003	Offals, Edible	7
233	Burkina Faso	2003	Oilcrops Oil, Other	1
233	Burkina Faso	2003	Palm Oil	1
233	Burkina Faso	2003	Peas	1
233	Burkina Faso	2003	Pelagic Fish	2
233	Burkina Faso	2003	Pigmeat	6
233	Burkina Faso	2003	Poultry Meat	6
233	Burkina Faso	2003	Pulses, Other	30
233	Burkina Faso	2003	Rice (Milled Equivalent)	46
233	Burkina Faso	2003	Sesameseed	1
233	Burkina Faso	2003	Sorghum	166
233	Burkina Faso	2003	Soyabeans	1
233	Burkina Faso	2003	Spices, Other	3
233	Burkina Faso	2003	Sugar, Refined Equivalent	11
233	Burkina Faso	2003	Sweet Potatoes	6
233	Burkina Faso	2003	Tomatoes	4
233	Burkina Faso	2003	Vegetables, Other	79
233	Burkina Faso	2003	Wheat	6
233	Burkina Faso	2003	Wine	1
233	Burkina Faso	2003	Yams	7
233	Burkina Faso	2004	Beer	9
233	Burkina Faso	2004	Beverages, Alcoholic	1
233	Burkina Faso	2004	Beverages, Fermented	566
233	Burkina Faso	2004	Bovine Meat	22
233	Burkina Faso	2004	Cassava	1
233	Burkina Faso	2004	Cereals, Other	2
233	Burkina Faso	2004	Cottonseed Oil	10
233	Burkina Faso	2004	Eggs	7
233	Burkina Faso	2004	Fats, Animals, Raw	2
233	Burkina Faso	2004	Freshwater Fish	2
233	Burkina Faso	2004	Fruits, Other	16
233	Burkina Faso	2004	Groundnut Oil	2
233	Burkina Faso	2004	Groundnuts (Shelled Eq)	36
233	Burkina Faso	2004	Maize	130
233	Burkina Faso	2004	Marine Fish, Other	1
233	Burkina Faso	2004	Meat, Other	2
233	Burkina Faso	2004	Milk, Whole	52
233	Burkina Faso	2004	Millet	197
233	Burkina Faso	2004	Mutton & Goat Meat	9
233	Burkina Faso	2004	Nuts	2
233	Burkina Faso	2004	Offals, Edible	7
233	Burkina Faso	2004	Oilcrops Oil, Other	1
233	Burkina Faso	2004	Palm Oil	1
233	Burkina Faso	2004	Pelagic Fish	2
233	Burkina Faso	2004	Pigmeat	6
233	Burkina Faso	2004	Poultry Meat	6
233	Burkina Faso	2004	Pulses, Other	33
233	Burkina Faso	2004	Rice (Milled Equivalent)	50
233	Burkina Faso	2004	Sorghum	166
233	Burkina Faso	2004	Spices, Other	3
233	Burkina Faso	2004	Sugar, Refined Equivalent	9
233	Burkina Faso	2004	Sweet Potatoes	8
233	Burkina Faso	2004	Tomatoes	4
233	Burkina Faso	2004	Vegetables, Other	82
233	Burkina Faso	2004	Wheat	15
233	Burkina Faso	2004	Wine	1
233	Burkina Faso	2004	Yams	17
233	Burkina Faso	2005	Beer	13
233	Burkina Faso	2005	Beverages, Alcoholic	2
233	Burkina Faso	2005	Beverages, Fermented	578
233	Burkina Faso	2005	Bovine Meat	23
233	Burkina Faso	2005	Cassava	1
233	Burkina Faso	2005	Cereals, Other	2
233	Burkina Faso	2005	Cottonseed Oil	9
233	Burkina Faso	2005	Eggs	7
233	Burkina Faso	2005	Fats, Animals, Raw	2
233	Burkina Faso	2005	Freshwater Fish	2
233	Burkina Faso	2005	Fruits, Other	17
233	Burkina Faso	2005	Groundnut Oil	1
233	Burkina Faso	2005	Groundnuts (Shelled Eq)	24
233	Burkina Faso	2005	Maize	130
233	Burkina Faso	2005	Meat, Other	2
233	Burkina Faso	2005	Milk, Whole	53
233	Burkina Faso	2005	Millet	193
233	Burkina Faso	2005	Mutton & Goat Meat	9
233	Burkina Faso	2005	Nuts	2
233	Burkina Faso	2005	Offals, Edible	7
233	Burkina Faso	2005	Oilcrops Oil, Other	1
233	Burkina Faso	2005	Palm Oil	4
233	Burkina Faso	2005	Pelagic Fish	5
233	Burkina Faso	2005	Pigmeat	7
233	Burkina Faso	2005	Poultry Meat	6
233	Burkina Faso	2005	Pulses, Other	35
233	Burkina Faso	2005	Rice (Milled Equivalent)	38

FAO country code	Country	Reference year	Food item	Gross food supply (g $capita^{-1}$ d^{-1})
233	Burkina Faso	2005	Sesameseed	1
233	Burkina Faso	2005	Sorghum	170
233	Burkina Faso	2005	Soyabeans	1
233	Burkina Faso	2005	Spices, Other	1
233	Burkina Faso	2005	Sugar, Refined Equivalent	15
233	Burkina Faso	2005	Sweet Potatoes	13
233	Burkina Faso	2005	Tomatoes	7
233	Burkina Faso	2005	Vegetables, Other	74
233	Burkina Faso	2005	Wheat	23
233	Burkina Faso	2005	Wine	1
233	Burkina Faso	2005	Yams	3
233	Burkina Faso	2006	Beer	13
233	Burkina Faso	2006	Beverages, Alcoholic	2
233	Burkina Faso	2006	Beverages, Fermented	543
233	Burkina Faso	2006	Bovine Meat	23
233	Burkina Faso	2006	Cassava	1
233	Burkina Faso	2006	Cereals, Other	2
233	Burkina Faso	2006	Cottonseed Oil	11
233	Burkina Faso	2006	Eggs	7
233	Burkina Faso	2006	Fats, Animals, Raw	2
233	Burkina Faso	2006	Freshwater Fish	2
233	Burkina Faso	2006	Fruits, Other	13
233	Burkina Faso	2006	Groundnut Oil	1
233	Burkina Faso	2006	Groundnuts (Shelled Eq)	22
233	Burkina Faso	2006	Maize	137
233	Burkina Faso	2006	Meat, Other	2
233	Burkina Faso	2006	Milk, Whole	58
233	Burkina Faso	2006	Millet	201
233	Burkina Faso	2006	Mutton & Goat Meat	9
233	Burkina Faso	2006	Nuts	1
233	Burkina Faso	2006	Offals, Edible	7
233	Burkina Faso	2006	Oilcrops Oil, Other	1
233	Burkina Faso	2006	Palm Oil	4
233	Burkina Faso	2006	Pelagic Fish	6
233	Burkina Faso	2006	Pigmeat	7
233	Burkina Faso	2006	Poultry Meat	6
233	Burkina Faso	2006	Pulses, Other	40
233	Burkina Faso	2006	Rice (Milled Equivalent)	57
233	Burkina Faso	2006	Sesameseed	1
233	Burkina Faso	2006	Sorghum	160
233	Burkina Faso	2006	Soyabeans	1
233	Burkina Faso	2006	Spices, Other	1
233	Burkina Faso	2006	Sugar, Refined Equivalent	15
233	Burkina Faso	2006	Sweet Potatoes	15
233	Burkina Faso	2006	Tomatoes	4
233	Burkina Faso	2006	Vegetables, Other	78
233	Burkina Faso	2006	Wheat	23
233	Burkina Faso	2006	Wine	1
233	Burkina Faso	2006	Yams	4
233	Burkina Faso	2007	Beer	14
233	Burkina Faso	2007	Beverages, Alcoholic	1
233	Burkina Faso	2007	Beverages, Fermented	566
233	Burkina Faso	2007	Bovine Meat	22
233	Burkina Faso	2007	Cassava	1
233	Burkina Faso	2007	Cereals, Other	2
233	Burkina Faso	2007	Cottonseed Oil	9
233	Burkina Faso	2007	Eggs	7
233	Burkina Faso	2007	Fats, Animals, Raw	2
233	Burkina Faso	2007	Freshwater Fish	1
233	Burkina Faso	2007	Fruits, Other	14
233	Burkina Faso	2007	Groundnut Oil	1
233	Burkina Faso	2007	Groundnuts (Shelled Eq)	25
233	Burkina Faso	2007	Maize	157
233	Burkina Faso	2007	Meat, Other	2
233	Burkina Faso	2007	Milk, Whole	52
233	Burkina Faso	2007	Millet	190
233	Burkina Faso	2007	Mutton & Goat Meat	9
233	Burkina Faso	2007	Nuts	1
233	Burkina Faso	2007	Offals, Edible	7
233	Burkina Faso	2007	Oilcrops Oil, Other	1
233	Burkina Faso	2007	Palm Oil	3
233	Burkina Faso	2007	Pelagic Fish	6
233	Burkina Faso	2007	Pigmeat	7
233	Burkina Faso	2007	Poultry Meat	6
233	Burkina Faso	2007	Pulses, Other	43
233	Burkina Faso	2007	Rice (Milled Equivalent)	44
233	Burkina Faso	2007	Sesameseed	1
233	Burkina Faso	2007	Sorghum	166
233	Burkina Faso	2007	Soyabeans	1
233	Burkina Faso	2007	Spices, Other	1
233	Burkina Faso	2007	Sugar, Refined Equivalent	17
233	Burkina Faso	2007	Sweet Potatoes	11
233	Burkina Faso	2007	Tomatoes	3
233	Burkina Faso	2007	Vegetables, Other	41
233	Burkina Faso	2007	Wheat	22
233	Burkina Faso	2007	Wine	1
233	Burkina Faso	2007	Yams	3
233	Burkina Faso	2008	Beer	13
233	Burkina Faso	2008	Beverages, Alcoholic	1
233	Burkina Faso	2008	Beverages, Fermented	558
233	Burkina Faso	2008	Bovine Meat	22
233	Burkina Faso	2008	Cassava	1
233	Burkina Faso	2008	Cereals, Other	3
233	Burkina Faso	2008	Cottonseed Oil	10
233	Burkina Faso	2008	Eggs	7
233	Burkina Faso	2008	Fats, Animals, Raw	1
233	Burkina Faso	2008	Freshwater Fish	1

FAO countrycode	Country	Reference year	Food item	Gross food supply (g *capita*$^{-1}$ d^{-1})
233	Burkina Faso	2008	Fruits, Other	15
233	Burkina Faso	2008	Groundnut Oil	1
233	Burkina Faso	2008	Groundnuts (Shelled Eq)	29
233	Burkina Faso	2008	Maize	160
233	Burkina Faso	2008	Meat, Other	2
233	Burkina Faso	2008	Milk, Whole	54
233	Burkina Faso	2008	Millet	183
233	Burkina Faso	2008	Mutton & Goat Meat	9
233	Burkina Faso	2008	Nuts	2
233	Burkina Faso	2008	Offals, Edible	7
233	Burkina Faso	2008	Oilcrops Oil, Other	1
233	Burkina Faso	2008	Palm Oil	4
233	Burkina Faso	2008	Pelagic Fish	6
233	Burkina Faso	2008	Pigmeat	6
233	Burkina Faso	2008	Poultry Meat	6
233	Burkina Faso	2008	Pulses, Other	42
233	Burkina Faso	2008	Rice (Milled Equivalent)	49
233	Burkina Faso	2008	Sesameseed	2
233	Burkina Faso	2008	Sorghum	164
233	Burkina Faso	2008	Soyabeans	5
233	Burkina Faso	2008	Spices, Other	1
233	Burkina Faso	2008	Sugar, Refined Equivalent	12
233	Burkina Faso	2008	Sweet Potatoes	12
233	Burkina Faso	2008	Tomatoes	2
233	Burkina Faso	2008	Vegetables, Other	45
233	Burkina Faso	2008	Wheat	16
233	Burkina Faso	2008	Wine	1
233	Burkina Faso	2008	Yams	7
233	Burkina Faso	2009	Beer	14
233	Burkina Faso	2009	Beverages, Alcoholic	1
233	Burkina Faso	2009	Beverages, Fermented	560
233	Burkina Faso	2009	Bovine Meat	22
233	Burkina Faso	2009	Cassava	2
233	Burkina Faso	2009	Cereals, Other	6
233	Burkina Faso	2009	Coconut Oil	1
233	Burkina Faso	2009	Coffee	1
233	Burkina Faso	2009	Cottonseed Oil	8
233	Burkina Faso	2009	Eggs	7
233	Burkina Faso	2009	Fats, Animals, Raw	1
233	Burkina Faso	2009	Freshwater Fish	1
233	Burkina Faso	2009	Fruits, Other	14
233	Burkina Faso	2009	Groundnut Oil	2
233	Burkina Faso	2009	Groundnuts (Shelled Eq)	33
233	Burkina Faso	2009	Maize	161
233	Burkina Faso	2009	Marine Fish, Other	3
233	Burkina Faso	2009	Meat, Other	2
233	Burkina Faso	2009	Milk, Whole	55
233	Burkina Faso	2009	Millet	170
233	Burkina Faso	2009	Mutton & Goat Meat	9
233	Burkina Faso	2009	Nuts	2
233	Burkina Faso	2009	Offals, Edible	7
233	Burkina Faso	2009	Oilcrops Oil, Other	1
233	Burkina Faso	2009	Palm Oil	3
233	Burkina Faso	2009	Pelagic Fish	7
233	Burkina Faso	2009	Pigmeat	6
233	Burkina Faso	2009	Potatoes	1
233	Burkina Faso	2009	Poultry Meat	6
233	Burkina Faso	2009	Pulses, Other	42
233	Burkina Faso	2009	Rice (Milled Equivalent)	61
233	Burkina Faso	2009	Sesameseed	1
233	Burkina Faso	2009	Sorghum	165
233	Burkina Faso	2009	Soyabeans	3
233	Burkina Faso	2009	Spices, Other	1
233	Burkina Faso	2009	Sugar, Refined Equivalent	12
233	Burkina Faso	2009	Sweet Potatoes	13
233	Burkina Faso	2009	Tomatoes	5
233	Burkina Faso	2009	Vegetables, Other	39
233	Burkina Faso	2009	Wheat	23
233	Burkina Faso	2009	Wine	1
233	Burkina Faso	2009	Yams	13
233	Burkina Faso	2010	Beer	13
233	Burkina Faso	2010	Beverages, Fermented	565
233	Burkina Faso	2010	Bovine Meat	22
233	Burkina Faso	2010	Cassava	2
233	Burkina Faso	2010	Cereals, Other	3
233	Burkina Faso	2010	Coconut Oil	1
233	Burkina Faso	2010	Coffee	1
233	Burkina Faso	2010	Cottonseed Oil	10
233	Burkina Faso	2010	Eggs	7
233	Burkina Faso	2010	Fats, Animals, Raw	1
233	Burkina Faso	2010	Freshwater Fish	2
233	Burkina Faso	2010	Fruits, Other	14
233	Burkina Faso	2010	Groundnut Oil	2
233	Burkina Faso	2010	Groundnuts (Shelled Eq)	33
233	Burkina Faso	2010	Maize	163
233	Burkina Faso	2010	Marine Fish, Other	4
233	Burkina Faso	2010	Meat, Other	2
233	Burkina Faso	2010	Milk, Whole	53
233	Burkina Faso	2010	Millet	169
233	Burkina Faso	2010	Mutton & Goat Meat	9
233	Burkina Faso	2010	Nuts	2
233	Burkina Faso	2010	Offals, Edible	7
233	Burkina Faso	2010	Oranges, Mandarines	2
233	Burkina Faso	2010	Palm Oil	5
233	Burkina Faso	2010	Pelagic Fish	8
233	Burkina Faso	2010	Pigmeat	6
233	Burkina Faso	2010	Potatoes	1

FAO country code	Country	Reference year	Food item	Gross food supply (g $capita^{-1}$ d^{-1})
233	Burkina Faso	2010	Poultry Meat	7
233	Burkina Faso	2010	Pulses, Other	40
233	Burkina Faso	2010	Rice (Milled Equivalent)	58
233	Burkina Faso	2010	Sesameseed	4
233	Burkina Faso	2010	Sorghum	166
233	Burkina Faso	2010	Soyabeans	4
233	Burkina Faso	2010	Spices, Other	1
233	Burkina Faso	2010	Sugar, Refined Equivalent	12
233	Burkina Faso	2010	Sweet Potatoes	15
233	Burkina Faso	2010	Sweeteners, Other	1
233	Burkina Faso	2010	Tomatoes	4
233	Burkina Faso	2010	Vegetables, Other	46
233	Burkina Faso	2010	Wheat	24
233	Burkina Faso	2010	Wine	1
233	Burkina Faso	2010	Yams	15
233	Burkina Faso	2011	Beer	15
233	Burkina Faso	2011	Beverages, Alcoholic	1
233	Burkina Faso	2011	Beverages, Fermented	547
233	Burkina Faso	2011	Bovine Meat	22
233	Burkina Faso	2011	Cassava	1
233	Burkina Faso	2011	Cereals, Other	2
233	Burkina Faso	2011	Coconut Oil	1
233	Burkina Faso	2011	Coffee	1
233	Burkina Faso	2011	Cottonseed Oil	8
233	Burkina Faso	2011	Eggs	7
233	Burkina Faso	2011	Fats, Animals, Raw	1
233	Burkina Faso	2011	Freshwater Fish	2
233	Burkina Faso	2011	Fruits, Other	14
233	Burkina Faso	2011	Groundnut Oil	2
233	Burkina Faso	2011	Groundnuts (Shelled Eq)	30
233	Burkina Faso	2011	Maize	167
233	Burkina Faso	2011	Marine Fish, Other	8
233	Burkina Faso	2011	Meat, Other	2
233	Burkina Faso	2011	Milk, Whole	55
233	Burkina Faso	2011	Millet	167
233	Burkina Faso	2011	Mutton & Goat Meat	9
233	Burkina Faso	2011	Nuts	2
233	Burkina Faso	2011	Offals, Edible	7
233	Burkina Faso	2011	Oranges, Mandarines	1
233	Burkina Faso	2011	Palm Oil	7
233	Burkina Faso	2011	Pelagic Fish	8
233	Burkina Faso	2011	Pigmeat	5
233	Burkina Faso	2011	Pineapples	1
233	Burkina Faso	2011	Potatoes	1
233	Burkina Faso	2011	Poultry Meat	7
233	Burkina Faso	2011	Pulses, Other	39
233	Burkina Faso	2011	Rice (Milled Equivalent)	56
233	Burkina Faso	2011	Sesameseed	4
233	Burkina Faso	2011	Sorghum	161
233	Burkina Faso	2011	Soyabeans	4
233	Burkina Faso	2011	Spices, Other	1
233	Burkina Faso	2011	Sugar, Refined Equivalent	17
233	Burkina Faso	2011	Sweet Potatoes	22
233	Burkina Faso	2011	Sweeteners, Other	1
233	Burkina Faso	2011	Tomatoes	3
233	Burkina Faso	2011	Vegetables, Other	42
233	Burkina Faso	2011	Wheat	24
233	Burkina Faso	2011	Wine	1
233	Burkina Faso	2011	Yams	15
115	Cambodia	1992	Bananas	33
115	Cambodia	1992	Beans	3
115	Cambodia	1992	Beer	2
115	Cambodia	1992	Beverages, Alcoholic	1
115	Cambodia	1992	Bovine Meat	13
115	Cambodia	1992	Cassava	40
115	Cambodia	1992	Coconut Oil	2
115	Cambodia	1992	Coconuts - Incl Copra	2
115	Cambodia	1992	Crustaceans	2
115	Cambodia	1992	Eggs	3
115	Cambodia	1992	Fats, Animals, Raw	2
115	Cambodia	1992	Freshwater Fish	12
115	Cambodia	1992	Fruits, Other	17
115	Cambodia	1992	Grapefruit	1
115	Cambodia	1992	Groundnuts (Shelled Eq)	1
115	Cambodia	1992	Maize	13
115	Cambodia	1992	Marine Fish, Other	6
115	Cambodia	1992	Milk, Whole	6
115	Cambodia	1992	Molluscs, Other	1
115	Cambodia	1992	Offals, Edible	4
115	Cambodia	1992	Oranges, Mandarines	12
115	Cambodia	1992	Palm Oil	2
115	Cambodia	1992	Pepper	1
115	Cambodia	1992	Pigmeat	23
115	Cambodia	1992	Pimento	2
115	Cambodia	1992	Pineapples	3
115	Cambodia	1992	Poultry Meat	5
115	Cambodia	1992	Rice (Milled Equivalent)	474
115	Cambodia	1992	Roots, Other	4
115	Cambodia	1992	Sesameseed	2
115	Cambodia	1992	Soyabean Oil	1
115	Cambodia	1992	Soyabeans	1
115	Cambodia	1992	Sugar Cane	8
115	Cambodia	1992	Sugar, Refined Equivalent	10
115	Cambodia	1992	Sweet Potatoes	16
115	Cambodia	1992	Vegetables, Other	121
115	Cambodia	1993	Bananas	32
115	Cambodia	1993	Beans	3

FAO country code	Country	Reference year	Food item	Gross food supply (g *capita*$^{-1}$ d^{-1})
115	Cambodia	1993	Beer	2
115	Cambodia	1993	Beverages, Alcoholic	1
115	Cambodia	1993	Bovine Meat	13
115	Cambodia	1993	Cassava	13
115	Cambodia	1993	Coconut Oil	2
115	Cambodia	1993	Coconuts - Incl Copra	2
115	Cambodia	1993	Crustaceans	2
115	Cambodia	1993	Eggs	3
115	Cambodia	1993	Fats, Animals, Raw	2
115	Cambodia	1993	Freshwater Fish	12
115	Cambodia	1993	Fruits, Other	18
115	Cambodia	1993	Grapefruit	1
115	Cambodia	1993	Groundnuts (Shelled Eq)	1
115	Cambodia	1993	Maize	9
115	Cambodia	1993	Marine Fish, Other	6
115	Cambodia	1993	Milk, Whole	8
115	Cambodia	1993	Offals, Edible	4
115	Cambodia	1993	Oranges, Mandarines	12
115	Cambodia	1993	Palm Oil	4
115	Cambodia	1993	Pepper	1
115	Cambodia	1993	Pigmeat	23
115	Cambodia	1993	Pimento	2
115	Cambodia	1993	Pineapples	3
115	Cambodia	1993	Poultry Meat	6
115	Cambodia	1993	Rice (Milled Equivalent)	477
115	Cambodia	1993	Roots, Other	4
115	Cambodia	1993	Sesameseed	1
115	Cambodia	1993	Soyabean Oil	1
115	Cambodia	1993	Soyabeans	1
115	Cambodia	1993	Sugar Cane	8
115	Cambodia	1993	Sugar, Refined Equivalent	4
115	Cambodia	1993	Sweet Potatoes	12
115	Cambodia	1993	Vegetables, Other	118
115	Cambodia	1993	Wheat	6
115	Cambodia	1994	Bananas	32
115	Cambodia	1994	Beans	3
115	Cambodia	1994	Beer	7
115	Cambodia	1994	Beverages, Alcoholic	4
115	Cambodia	1994	Bovine Meat	13
115	Cambodia	1994	Cassava	16
115	Cambodia	1994	Coconut Oil	2
115	Cambodia	1994	Coconuts - Incl Copra	2
115	Cambodia	1994	Crustaceans	2
115	Cambodia	1994	Eggs	3
115	Cambodia	1994	Fats, Animals, Raw	2
115	Cambodia	1994	Freshwater Fish	12
115	Cambodia	1994	Fruits, Other	18
115	Cambodia	1994	Grapefruit	1
115	Cambodia	1994	Groundnuts (Shelled Eq)	1
115	Cambodia	1994	Maize	10
115	Cambodia	1994	Marine Fish, Other	5
115	Cambodia	1994	Milk, Whole	11
115	Cambodia	1994	Offals, Edible	4
115	Cambodia	1994	Oranges, Mandarines	11
115	Cambodia	1994	Palm Oil	5
115	Cambodia	1994	Pelagic Fish	1
115	Cambodia	1994	Pepper	1
115	Cambodia	1994	Pigmeat	21
115	Cambodia	1994	Pimento	2
115	Cambodia	1994	Pineapples	3
115	Cambodia	1994	Poultry Meat	5
115	Cambodia	1994	Rice (Milled Equivalent)	480
115	Cambodia	1994	Roots, Other	4
115	Cambodia	1994	Sesameseed	1
115	Cambodia	1994	Soyabean Oil	1
115	Cambodia	1994	Sugar Cane	12
115	Cambodia	1994	Sugar, Refined Equivalent	5
115	Cambodia	1994	Sweet Potatoes	9
115	Cambodia	1994	Vegetables, Other	115
115	Cambodia	1994	Wheat	2
115	Cambodia	1995	Bananas	32
115	Cambodia	1995	Beans	3
115	Cambodia	1995	Beer	6
115	Cambodia	1995	Beverages, Alcoholic	3
115	Cambodia	1995	Bovine Meat	13
115	Cambodia	1995	Cassava	20
115	Cambodia	1995	Coconut Oil	2
115	Cambodia	1995	Coconuts - Incl Copra	1
115	Cambodia	1995	Crustaceans	2
115	Cambodia	1995	Eggs	3
115	Cambodia	1995	Fats, Animals, Raw	2
115	Cambodia	1995	Freshwater Fish	15
115	Cambodia	1995	Fruits, Other	18
115	Cambodia	1995	Grapefruit	1
115	Cambodia	1995	Groundnuts (Shelled Eq)	1
115	Cambodia	1995	Maize	11
115	Cambodia	1995	Marine Fish, Other	5
115	Cambodia	1995	Milk, Whole	12
115	Cambodia	1995	Offals, Edible	4
115	Cambodia	1995	Oranges, Mandarines	11
115	Cambodia	1995	Palm Oil	5
115	Cambodia	1995	Pelagic Fish	3
115	Cambodia	1995	Pepper	1
115	Cambodia	1995	Pigmeat	21
115	Cambodia	1995	Pimento	2
115	Cambodia	1995	Pineapples	3
115	Cambodia	1995	Poultry Meat	5

FAO country code	Country	Reference year	Food item	Gross food supply (g *capita*$^{-1}$ d^{-1})
115	Cambodia	1995	Rice (Milled Equivalent)	478
115	Cambodia	1995	Roots, Other	5
115	Cambodia	1995	Sesameseed	1
115	Cambodia	1995	Soyabean Oil	1
115	Cambodia	1995	Sugar Cane	10
115	Cambodia	1995	Sugar, Refined Equivalent	5
115	Cambodia	1995	Sweet Potatoes	9
115	Cambodia	1995	Vegetables, Other	103
115	Cambodia	1995	Wheat	4
115	Cambodia	1996	Apples	1
115	Cambodia	1996	Bananas	33
115	Cambodia	1996	Beans	3
115	Cambodia	1996	Beer	3
115	Cambodia	1996	Beverages, Alcoholic	6
115	Cambodia	1996	Bovine Meat	13
115	Cambodia	1996	Cassava	16
115	Cambodia	1996	Coconut Oil	2
115	Cambodia	1996	Coconuts - Incl Copra	5
115	Cambodia	1996	Crustaceans	2
115	Cambodia	1996	Eggs	3
115	Cambodia	1996	Fats, Animals, Raw	2
115	Cambodia	1996	Freshwater Fish	12
115	Cambodia	1996	Fruits, Other	19
115	Cambodia	1996	Grapefruit	1
115	Cambodia	1996	Groundnuts (Shelled Eq)	1
115	Cambodia	1996	Maize	11
115	Cambodia	1996	Marine Fish, Other	7
115	Cambodia	1996	Milk, Whole	10
115	Cambodia	1996	Offals, Edible	4
115	Cambodia	1996	Oranges, Mandarines	13
115	Cambodia	1996	Palm Oil	4
115	Cambodia	1996	Pigmeat	21
115	Cambodia	1996	Pimento	2
115	Cambodia	1996	Pineapples	3
115	Cambodia	1996	Poultry Meat	5
115	Cambodia	1996	Rice (Milled Equivalent)	430
115	Cambodia	1996	Roots, Other	5
115	Cambodia	1996	Sesameseed	1
115	Cambodia	1996	Soyabean Oil	1
115	Cambodia	1996	Sugar Cane	8
115	Cambodia	1996	Sugar, Refined Equivalent	9
115	Cambodia	1996	Sweet Potatoes	9
115	Cambodia	1996	Vegetables, Other	102
115	Cambodia	1996	Wheat	6
115	Cambodia	1997	Apples	1
115	Cambodia	1997	Bananas	33
115	Cambodia	1997	Beans	4
115	Cambodia	1997	Beer	6
115	Cambodia	1997	Beverages, Alcoholic	6
115	Cambodia	1997	Bovine Meat	13
115	Cambodia	1997	Cassava	18
115	Cambodia	1997	Coconut Oil	2
115	Cambodia	1997	Coconuts - Incl Copra	1
115	Cambodia	1997	Crustaceans	2
115	Cambodia	1997	Eggs	3
115	Cambodia	1997	Fats, Animals, Raw	2
115	Cambodia	1997	Freshwater Fish	11
115	Cambodia	1997	Fruits, Other	20
115	Cambodia	1997	Grapefruit	1
115	Cambodia	1997	Groundnuts (Shelled Eq)	1
115	Cambodia	1997	Maize	9
115	Cambodia	1997	Marine Fish, Other	4
115	Cambodia	1997	Milk, Whole	7
115	Cambodia	1997	Offals, Edible	4
115	Cambodia	1997	Oranges, Mandarines	13
115	Cambodia	1997	Palm Oil	4
115	Cambodia	1997	Pelagic Fish	1
115	Cambodia	1997	Pigmeat	23
115	Cambodia	1997	Pimento	2
115	Cambodia	1997	Pineapples	3
115	Cambodia	1997	Poultry Meat	6
115	Cambodia	1997	Rice (Milled Equivalent)	428
115	Cambodia	1997	Roots, Other	5
115	Cambodia	1997	Sesameseed	2
115	Cambodia	1997	Soyabean Oil	1
115	Cambodia	1997	Sugar Cane	9
115	Cambodia	1997	Sugar, Refined Equivalent	13
115	Cambodia	1997	Sweet Potatoes	7
115	Cambodia	1997	Sweeteners, Other	1
115	Cambodia	1997	Vegetables, Other	100
115	Cambodia	1997	Wheat	4
115	Cambodia	1998	Bananas	33
115	Cambodia	1998	Beans	3
115	Cambodia	1998	Beer	6
115	Cambodia	1998	Beverages, Alcoholic	7
115	Cambodia	1998	Bovine Meat	12
115	Cambodia	1998	Cassava	15
115	Cambodia	1998	Coconut Oil	2
115	Cambodia	1998	Coconuts - Incl Copra	1
115	Cambodia	1998	Crustaceans	1
115	Cambodia	1998	Eggs	3
115	Cambodia	1998	Fats, Animals, Raw	2
115	Cambodia	1998	Freshwater Fish	11
115	Cambodia	1998	Fruits, Other	20
115	Cambodia	1998	Grapefruit	1
115	Cambodia	1998	Groundnuts (Shelled Eq)	1
115	Cambodia	1998	Maize	11

FAO country code	Country	Reference year	Food item	Gross food supply (g *capita*$^{-1}$ d^{-1})
115	Cambodia	1998	Marine Fish, Other	5
115	Cambodia	1998	Milk, Whole	8
115	Cambodia	1998	Molluscs, Other	1
115	Cambodia	1998	Nuts	1
115	Cambodia	1998	Offals, Edible	4
115	Cambodia	1998	Oranges, Mandarines	13
115	Cambodia	1998	Palm Oil	3
115	Cambodia	1998	Pelagic Fish	1
115	Cambodia	1998	Pigmeat	23
115	Cambodia	1998	Pimento	2
115	Cambodia	1998	Pineapples	3
115	Cambodia	1998	Poultry Meat	6
115	Cambodia	1998	Rice (Milled Equivalent)	448
115	Cambodia	1998	Roots, Other	5
115	Cambodia	1998	Sesameseed	1
115	Cambodia	1998	Soyabean Oil	1
115	Cambodia	1998	Sugar Cane	6
115	Cambodia	1998	Sugar, Refined Equivalent	14
115	Cambodia	1998	Sweet Potatoes	7
115	Cambodia	1998	Sweeteners, Other	1
115	Cambodia	1998	Vegetables, Other	98
115	Cambodia	1998	Wheat	6
115	Cambodia	1999	Bananas	32
115	Cambodia	1999	Beans	3
115	Cambodia	1999	Beer	7
115	Cambodia	1999	Beverages, Alcoholic	7
115	Cambodia	1999	Bovine Meat	12
115	Cambodia	1999	Cassava	27
115	Cambodia	1999	Coconut Oil	2
115	Cambodia	1999	Coconuts - Incl Copra	2
115	Cambodia	1999	Crustaceans	2
115	Cambodia	1999	Eggs	3
115	Cambodia	1999	Fats, Animals, Raw	2
115	Cambodia	1999	Freshwater Fish	46
115	Cambodia	1999	Fruits, Other	20
115	Cambodia	1999	Grapefruit	1
115	Cambodia	1999	Groundnuts (Shelled Eq)	1
115	Cambodia	1999	Maize	18
115	Cambodia	1999	Marine Fish, Other	6
115	Cambodia	1999	Milk, Whole	8
115	Cambodia	1999	Nuts	1
115	Cambodia	1999	Offals, Edible	4
115	Cambodia	1999	Oranges, Mandarines	13
115	Cambodia	1999	Palm Oil	3
115	Cambodia	1999	Pelagic Fish	1
115	Cambodia	1999	Pepper	1
115	Cambodia	1999	Pigmeat	23
115	Cambodia	1999	Pimento	2
115	Cambodia	1999	Pineapples	3
115	Cambodia	1999	Poultry Meat	6
115	Cambodia	1999	Rice (Milled Equivalent)	448
115	Cambodia	1999	Roots, Other	5
115	Cambodia	1999	Sesameseed	2
115	Cambodia	1999	Soyabean Oil	1
115	Cambodia	1999	Sugar Cane	7
115	Cambodia	1999	Sugar, Refined Equivalent	16
115	Cambodia	1999	Sweet Potatoes	7
115	Cambodia	1999	Sweeteners, Other	1
115	Cambodia	1999	Vegetables, Other	97
115	Cambodia	1999	Wheat	7
115	Cambodia	2000	Apples	1
115	Cambodia	2000	Bananas	31
115	Cambodia	2000	Beans	3
115	Cambodia	2000	Beer	10
115	Cambodia	2000	Beverages, Alcoholic	5
115	Cambodia	2000	Bovine Meat	15
115	Cambodia	2000	Cassava	29
115	Cambodia	2000	Cephalopods	1
115	Cambodia	2000	Cereals, Other	1
115	Cambodia	2000	Coconut Oil	2
115	Cambodia	2000	Coconuts - Incl Copra	3
115	Cambodia	2000	Crustaceans	2
115	Cambodia	2000	Eggs	3
115	Cambodia	2000	Fats, Animals, Raw	2
115	Cambodia	2000	Freshwater Fish	48
115	Cambodia	2000	Fruits, Other	19
115	Cambodia	2000	Grapefruit	1
115	Cambodia	2000	Groundnuts (Shelled Eq)	1
115	Cambodia	2000	Maize	19
115	Cambodia	2000	Marine Fish, Other	5
115	Cambodia	2000	Milk, Whole	6
115	Cambodia	2000	Nuts	1
115	Cambodia	2000	Offals, Edible	5
115	Cambodia	2000	Oranges, Mandarines	13
115	Cambodia	2000	Palm Oil	2
115	Cambodia	2000	Pelagic Fish	1
115	Cambodia	2000	Pigmeat	24
115	Cambodia	2000	Pimento	2
115	Cambodia	2000	Pineapples	3
115	Cambodia	2000	Poultry Meat	6
115	Cambodia	2000	Rice (Milled Equivalent)	459
115	Cambodia	2000	Roots, Other	6
115	Cambodia	2000	Sesameseed	2
115	Cambodia	2000	Soyabean Oil	1
115	Cambodia	2000	Soyabeans	1
115	Cambodia	2000	Sugar Cane	7
115	Cambodia	2000	Sugar, Refined Equivalent	21

FAO countrycode	Country	Reference year	Food item	Gross food supply (g *capita*$^{-1}$ d^{-1})
115	Cambodia	2000	Sweet Potatoes	6
115	Cambodia	2000	Vegetables, Other	103
115	Cambodia	2000	Wheat	6
115	Cambodia	2000	Whey	1
115	Cambodia	2001	Apples	1
115	Cambodia	2001	Bananas	31
115	Cambodia	2001	Beans	3
115	Cambodia	2001	Beer	9
115	Cambodia	2001	Beverages, Alcoholic	6
115	Cambodia	2001	Bovine Meat	15
115	Cambodia	2001	Cassava	29
115	Cambodia	2001	Cephalopods	1
115	Cambodia	2001	Cereals, Other	2
115	Cambodia	2001	Coconut Oil	2
115	Cambodia	2001	Coconuts - Incl Copra	2
115	Cambodia	2001	Crustaceans	3
115	Cambodia	2001	Eggs	3
115	Cambodia	2001	Fats, Animals, Raw	2
115	Cambodia	2001	Freshwater Fish	79
115	Cambodia	2001	Fruits, Other	19
115	Cambodia	2001	Grapefruit	1
115	Cambodia	2001	Groundnuts (Shelled Eq)	1
115	Cambodia	2001	Maize	23
115	Cambodia	2001	Marine Fish, Other	5
115	Cambodia	2001	Milk, Whole	7
115	Cambodia	2001	Nuts	1
115	Cambodia	2001	Offals, Edible	4
115	Cambodia	2001	Oilcrops Oil, Other	1
115	Cambodia	2001	Oranges, Mandarines	13
115	Cambodia	2001	Palm Oil	3
115	Cambodia	2001	Pepper	1
115	Cambodia	2001	Pigmeat	23
115	Cambodia	2001	Pimento	2
115	Cambodia	2001	Pineapples	3
115	Cambodia	2001	Poultry Meat	6
115	Cambodia	2001	Rice (Milled Equivalent)	439
115	Cambodia	2001	Roots, Other	5
115	Cambodia	2001	Sesameseed	2
115	Cambodia	2001	Soyabean Oil	1
115	Cambodia	2001	Sugar Cane	7
115	Cambodia	2001	Sugar, Refined Equivalent	29
115	Cambodia	2001	Sweet Potatoes	5
115	Cambodia	2001	Vegetables, Other	94
115	Cambodia	2001	Wheat	5
115	Cambodia	2001	Whey	1
115	Cambodia	2002	Apples	1
115	Cambodia	2002	Bananas	30
115	Cambodia	2002	Beans	4
115	Cambodia	2002	Beer	9
115	Cambodia	2002	Beverages, Alcoholic	5
115	Cambodia	2002	Bovine Meat	13
115	Cambodia	2002	Cassava	24
115	Cambodia	2002	Cephalopods	1
115	Cambodia	2002	Coconut Oil	2
115	Cambodia	2002	Coconuts - Incl Copra	5
115	Cambodia	2002	Crustaceans	3
115	Cambodia	2002	Eggs	3
115	Cambodia	2002	Fats, Animals, Raw	2
115	Cambodia	2002	Freshwater Fish	70
115	Cambodia	2002	Fruits, Other	22
115	Cambodia	2002	Grapefruit	1
115	Cambodia	2002	Groundnuts (Shelled Eq)	1
115	Cambodia	2002	Maize	26
115	Cambodia	2002	Marine Fish, Other	5
115	Cambodia	2002	Milk, Whole	6
115	Cambodia	2002	Nuts	1
115	Cambodia	2002	Offals, Edible	4
115	Cambodia	2002	Oilcrops Oil, Other	1
115	Cambodia	2002	Oranges, Mandarines	13
115	Cambodia	2002	Palm Oil	3
115	Cambodia	2002	Pelagic Fish	1
115	Cambodia	2002	Pepper	1
115	Cambodia	2002	Pigmeat	23
115	Cambodia	2002	Pimento	2
115	Cambodia	2002	Pineapples	3
115	Cambodia	2002	Poultry Meat	6
115	Cambodia	2002	Rice (Milled Equivalent)	443
115	Cambodia	2002	Roots, Other	6
115	Cambodia	2002	Sesameseed	2
115	Cambodia	2002	Soyabean Oil	1
115	Cambodia	2002	Soyabeans	2
115	Cambodia	2002	Sugar Cane	9
115	Cambodia	2002	Sugar, Refined Equivalent	47
115	Cambodia	2002	Sweet Potatoes	6
115	Cambodia	2002	Vegetables, Other	92
115	Cambodia	2002	Wheat	5
115	Cambodia	2003	Apples	1
115	Cambodia	2003	Bananas	29
115	Cambodia	2003	Beans	6
115	Cambodia	2003	Beer	12
115	Cambodia	2003	Beverages, Alcoholic	5
115	Cambodia	2003	Bovine Meat	13
115	Cambodia	2003	Cassava	59
115	Cambodia	2003	Cephalopods	1
115	Cambodia	2003	Coconut Oil	1
115	Cambodia	2003	Coconuts - Incl Copra	6
115	Cambodia	2003	Crustaceans	4

FAO country code	Country	Reference year	Food item	Gross food supply (g *capita*$^{-1}$ d^{-1})
115	Cambodia	2003	Eggs	3
115	Cambodia	2003	Fats, Animals, Raw	2
115	Cambodia	2003	Freshwater Fish	58
115	Cambodia	2003	Fruits, Other	20
115	Cambodia	2003	Grapefruit	1
115	Cambodia	2003	Groundnuts (Shelled Eq)	2
115	Cambodia	2003	Maize	28
115	Cambodia	2003	Marine Fish, Other	6
115	Cambodia	2003	Milk, Whole	7
115	Cambodia	2003	Nuts	1
115	Cambodia	2003	Offals, Edible	4
115	Cambodia	2003	Oilcrops Oil, Other	1
115	Cambodia	2003	Oranges, Mandarines	13
115	Cambodia	2003	Palm Oil	2
115	Cambodia	2003	Pelagic Fish	1
115	Cambodia	2003	Pepper	1
115	Cambodia	2003	Pigmeat	25
115	Cambodia	2003	Pimento	2
115	Cambodia	2003	Pineapples	3
115	Cambodia	2003	Poultry Meat	6
115	Cambodia	2003	Rice (Milled Equivalent)	428
115	Cambodia	2003	Roots, Other	5
115	Cambodia	2003	Sesameseed	4
115	Cambodia	2003	Soyabean Oil	2
115	Cambodia	2003	Soyabeans	4
115	Cambodia	2003	Sugar Cane	7
115	Cambodia	2003	Sugar, Refined Equivalent	44
115	Cambodia	2003	Sweet Potatoes	7
115	Cambodia	2003	Vegetables, Other	90
115	Cambodia	2003	Wheat	7
115	Cambodia	2004	Apples	1
115	Cambodia	2004	Bananas	27
115	Cambodia	2004	Beans	6
115	Cambodia	2004	Beer	15
115	Cambodia	2004	Beverages, Alcoholic	5
115	Cambodia	2004	Bovine Meat	13
115	Cambodia	2004	Cassava	63
115	Cambodia	2004	Cephalopods	1
115	Cambodia	2004	Coconut Oil	1
115	Cambodia	2004	Coconuts - Incl Copra	5
115	Cambodia	2004	Crustaceans	2
115	Cambodia	2004	Eggs	3
115	Cambodia	2004	Fats, Animals, Raw	3
115	Cambodia	2004	Freshwater Fish	48
115	Cambodia	2004	Fruits, Other	21
115	Cambodia	2004	Grapefruit	1
115	Cambodia	2004	Groundnuts (Shelled Eq)	3
115	Cambodia	2004	Maize	29
115	Cambodia	2004	Marine Fish, Other	7
115	Cambodia	2004	Milk, Whole	6
115	Cambodia	2004	Nuts	1
115	Cambodia	2004	Offals, Edible	4
115	Cambodia	2004	Oranges, Mandarines	12
115	Cambodia	2004	Palm Oil	2
115	Cambodia	2004	Pelagic Fish	1
115	Cambodia	2004	Pepper	1
115	Cambodia	2004	Pigmeat	25
115	Cambodia	2004	Pimento	2
115	Cambodia	2004	Pineapples	3
115	Cambodia	2004	Poultry Meat	6
115	Cambodia	2004	Rice (Milled Equivalent)	414
115	Cambodia	2004	Roots, Other	5
115	Cambodia	2004	Sesameseed	6
115	Cambodia	2004	Soyabean Oil	2
115	Cambodia	2004	Soyabeans	4
115	Cambodia	2004	Sugar Cane	5
115	Cambodia	2004	Sugar, Refined Equivalent	58
115	Cambodia	2004	Sweet Potatoes	7
115	Cambodia	2004	Vegetables, Other	95
115	Cambodia	2004	Wheat	8
115	Cambodia	2005	Apples	1
115	Cambodia	2005	Bananas	25
115	Cambodia	2005	Beans	7
115	Cambodia	2005	Beer	27
115	Cambodia	2005	Beverages, Alcoholic	5
115	Cambodia	2005	Bovine Meat	14
115	Cambodia	2005	Cassava	63
115	Cambodia	2005	Cephalopods	1
115	Cambodia	2005	Coconut Oil	1
115	Cambodia	2005	Coconuts - Incl Copra	5
115	Cambodia	2005	Crustaceans	4
115	Cambodia	2005	Eggs	3
115	Cambodia	2005	Fats, Animals, Raw	3
115	Cambodia	2005	Freshwater Fish	62
115	Cambodia	2005	Fruits, Other	27
115	Cambodia	2005	Grapefruit	1
115	Cambodia	2005	Groundnuts (Shelled Eq)	3
115	Cambodia	2005	Maize	31
115	Cambodia	2005	Marine Fish, Other	7
115	Cambodia	2005	Milk, Whole	11
115	Cambodia	2005	Nuts	1
115	Cambodia	2005	Offals, Edible	4
115	Cambodia	2005	Oilcrops Oil, Other	1
115	Cambodia	2005	Oranges, Mandarines	12
115	Cambodia	2005	Palm Oil	2
115	Cambodia	2005	Pepper	1
115	Cambodia	2005	Pigmeat	25

FAO country code	Country	Reference year	Food item	Gross food supply (g *capita*$^{-1}$ d^{-1})
115	Cambodia	2005	Pimento	2
115	Cambodia	2005	Pineapples	4
115	Cambodia	2005	Poultry Meat	5
115	Cambodia	2005	Rice (Milled Equivalent)	431
115	Cambodia	2005	Roots, Other	5
115	Cambodia	2005	Sesameseed	5
115	Cambodia	2005	Soyabean Oil	2
115	Cambodia	2005	Soyabeans	6
115	Cambodia	2005	Sugar Cane	5
115	Cambodia	2005	Sugar, Refined Equivalent	43
115	Cambodia	2005	Sweet Potatoes	8
115	Cambodia	2005	Sweeteners, Other	3
115	Cambodia	2005	Vegetables, Other	89
115	Cambodia	2005	Wheat	8
115	Cambodia	2006	Bananas	25
115	Cambodia	2006	Beans	7
115	Cambodia	2006	Beer	29
115	Cambodia	2006	Beverages, Alcoholic	5
115	Cambodia	2006	Bovine Meat	14
115	Cambodia	2006	Cassava	66
115	Cambodia	2006	Cephalopods	1
115	Cambodia	2006	Coconut Oil	1
115	Cambodia	2006	Coconuts - Incl Copra	5
115	Cambodia	2006	Crustaceans	4
115	Cambodia	2006	Eggs	3
115	Cambodia	2006	Fats, Animals, Raw	3
115	Cambodia	2006	Freshwater Fish	87
115	Cambodia	2006	Fruits, Other	31
115	Cambodia	2006	Grapefruit	1
115	Cambodia	2006	Groundnuts (Shelled Eq)	3
115	Cambodia	2006	Maize	31
115	Cambodia	2006	Marine Fish, Other	7
115	Cambodia	2006	Milk, Whole	18
115	Cambodia	2006	Molluscs, Other	1
115	Cambodia	2006	Nuts	1
115	Cambodia	2006	Offals, Edible	5
115	Cambodia	2006	Oilcrops Oil, Other	1
115	Cambodia	2006	Oranges, Mandarines	13
115	Cambodia	2006	Palm Oil	2
115	Cambodia	2006	Pelagic Fish	1
115	Cambodia	2006	Pigmeat	25
115	Cambodia	2006	Pimento	2
115	Cambodia	2006	Pineapples	4
115	Cambodia	2006	Poultry Meat	5
115	Cambodia	2006	Rice (Milled Equivalent)	433
115	Cambodia	2006	Roots, Other	6
115	Cambodia	2006	Sesameseed	7
115	Cambodia	2006	Soyabean Oil	2
115	Cambodia	2006	Soyabeans	7
115	Cambodia	2006	Sugar Cane	6
115	Cambodia	2006	Sugar, Refined Equivalent	42
115	Cambodia	2006	Sweet Potatoes	9
115	Cambodia	2006	Sweeteners, Other	2
115	Cambodia	2006	Tea	2
115	Cambodia	2006	Vegetables, Other	88
115	Cambodia	2006	Wheat	9
115	Cambodia	2007	Apples	1
115	Cambodia	2007	Bananas	27
115	Cambodia	2007	Beans	7
115	Cambodia	2007	Beer	32
115	Cambodia	2007	Beverages, Alcoholic	5
115	Cambodia	2007	Bovine Meat	14
115	Cambodia	2007	Cassava	67
115	Cambodia	2007	Cephalopods	1
115	Cambodia	2007	Citrus, Other	1
115	Cambodia	2007	Coconut Oil	1
115	Cambodia	2007	Coconuts - Incl Copra	5
115	Cambodia	2007	Crustaceans	3
115	Cambodia	2007	Eggs	4
115	Cambodia	2007	Fats, Animals, Raw	2
115	Cambodia	2007	Freshwater Fish	81
115	Cambodia	2007	Fruits, Other	26
115	Cambodia	2007	Grapefruit	1
115	Cambodia	2007	Groundnuts (Shelled Eq)	4
115	Cambodia	2007	Maize	32
115	Cambodia	2007	Marine Fish, Other	8
115	Cambodia	2007	Milk, Whole	14
115	Cambodia	2007	Molluscs, Other	1
115	Cambodia	2007	Nuts	1
115	Cambodia	2007	Offals, Edible	4
115	Cambodia	2007	Oilcrops Oil, Other	1
115	Cambodia	2007	Oranges, Mandarines	13
115	Cambodia	2007	Palm Oil	1
115	Cambodia	2007	Pelagic Fish	1
115	Cambodia	2007	Pigmeat	25
115	Cambodia	2007	Pimento	2
115	Cambodia	2007	Pineapples	4
115	Cambodia	2007	Poultry Meat	5
115	Cambodia	2007	Rice (Milled Equivalent)	434
115	Cambodia	2007	Roots, Other	7
115	Cambodia	2007	Sesameseed	6
115	Cambodia	2007	Soyabean Oil	2
115	Cambodia	2007	Soyabeans	7
115	Cambodia	2007	Sugar Cane	11
115	Cambodia	2007	Sugar, Refined Equivalent	43
115	Cambodia	2007	Sweet Potatoes	7
115	Cambodia	2007	Sweeteners, Other	2

FAO country code	Country	Reference year	Food item	Gross food supply (g *capita*$^{-1}$ d^{-1})
115	Cambodia	2007	Tea	1
115	Cambodia	2007	Vegetables, Other	88
115	Cambodia	2007	Wheat	9
115	Cambodia	2007	Whey	2
115	Cambodia	2008	Apples	1
115	Cambodia	2008	Bananas	32
115	Cambodia	2008	Beans	9
115	Cambodia	2008	Beer	35
115	Cambodia	2008	Beverages, Alcoholic	6
115	Cambodia	2008	Bovine Meat	14
115	Cambodia	2008	Cassava	69
115	Cambodia	2008	Cephalopods	1
115	Cambodia	2008	Citrus, Other	1
115	Cambodia	2008	Coconut Oil	1
115	Cambodia	2008	Coconuts - Incl Copra	5
115	Cambodia	2008	Crustaceans	3
115	Cambodia	2008	Eggs	4
115	Cambodia	2008	Fats, Animals, Raw	2
115	Cambodia	2008	Freshwater Fish	75
115	Cambodia	2008	Fruits, Other	27
115	Cambodia	2008	Grapefruit	1
115	Cambodia	2008	Groundnuts (Shelled Eq)	3
115	Cambodia	2008	Maize	30
115	Cambodia	2008	Marine Fish, Other	8
115	Cambodia	2008	Milk, Whole	11
115	Cambodia	2008	Molluscs, Other	1
115	Cambodia	2008	Nuts	1
115	Cambodia	2008	Offals, Edible	4
115	Cambodia	2008	Oilcrops Oil, Other	1
115	Cambodia	2008	Oranges, Mandarines	13
115	Cambodia	2008	Palm Oil	1
115	Cambodia	2008	Pigmeat	24
115	Cambodia	2008	Pimento	2
115	Cambodia	2008	Pineapples	4
115	Cambodia	2008	Poultry Meat	5
115	Cambodia	2008	Rice (Milled Equivalent)	435
115	Cambodia	2008	Roots, Other	7
115	Cambodia	2008	Sesameseed	6
115	Cambodia	2008	Soyabean Oil	2
115	Cambodia	2008	Soyabeans	8
115	Cambodia	2008	Sugar Cane	15
115	Cambodia	2008	Sugar, Refined Equivalent	46
115	Cambodia	2008	Sweet Potatoes	7
115	Cambodia	2008	Sweeteners, Other	7
115	Cambodia	2008	Vegetables, Other	82
115	Cambodia	2008	Wheat	6
115	Cambodia	2008	Whey	13
115	Cambodia	2009	Bananas	31
115	Cambodia	2009	Beans	11
115	Cambodia	2009	Beer	35
115	Cambodia	2009	Beverages, Alcoholic	5
115	Cambodia	2009	Bovine Meat	15
115	Cambodia	2009	Cassava	68
115	Cambodia	2009	Cephalopods	1
115	Cambodia	2009	Citrus, Other	1
115	Cambodia	2009	Coconut Oil	1
115	Cambodia	2009	Coconuts - Incl Copra	3
115	Cambodia	2009	Coffee	2
115	Cambodia	2009	Crustaceans	2
115	Cambodia	2009	Eggs	3
115	Cambodia	2009	Fats, Animals, Raw	2
115	Cambodia	2009	Freshwater Fish	80
115	Cambodia	2009	Fruits, Other	27
115	Cambodia	2009	Grapefruit	1
115	Cambodia	2009	Groundnuts (Shelled Eq)	2
115	Cambodia	2009	Maize	31
115	Cambodia	2009	Marine Fish, Other	10
115	Cambodia	2009	Milk, Whole	12
115	Cambodia	2009	Molluscs, Other	1
115	Cambodia	2009	Nuts	1
115	Cambodia	2009	Offals, Edible	4
115	Cambodia	2009	Oilcrops Oil, Other	1
115	Cambodia	2009	Oranges, Mandarines	12
115	Cambodia	2009	Palm Oil	1
115	Cambodia	2009	Pigmeat	24
115	Cambodia	2009	Pimento	2
115	Cambodia	2009	Pineapples	4
115	Cambodia	2009	Poultry Meat	6
115	Cambodia	2009	Rice (Milled Equivalent)	434
115	Cambodia	2009	Roots, Other	6
115	Cambodia	2009	Sesameseed	6
115	Cambodia	2009	Soyabean Oil	2
115	Cambodia	2009	Soyabeans	9
115	Cambodia	2009	Sugar Cane	14
115	Cambodia	2009	Sugar, Refined Equivalent	46
115	Cambodia	2009	Sweet Potatoes	15
115	Cambodia	2009	Sweeteners, Other	4
115	Cambodia	2009	Vegetables, Other	94
115	Cambodia	2009	Wheat	7
115	Cambodia	2009	Whey	11
115	Cambodia	2010	Bananas	31
115	Cambodia	2010	Beans	13
115	Cambodia	2010	Beer	34
115	Cambodia	2010	Beverages, Alcoholic	6
115	Cambodia	2010	Bovine Meat	14
115	Cambodia	2010	Cassava	71
115	Cambodia	2010	Cephalopods	1

FAO country code	Country	Reference year	Food item	Gross food supply (g $capita^{-1}$ d^{-1})
115	Cambodia	2010	Cereals, Other	1
115	Cambodia	2010	Coconut Oil	2
115	Cambodia	2010	Crustaceans	3
115	Cambodia	2010	Eggs	4
115	Cambodia	2010	Fats, Animals, Raw	2
115	Cambodia	2010	Freshwater Fish	82
115	Cambodia	2010	Fruits, Other	27
115	Cambodia	2010	Grapefruit	1
115	Cambodia	2010	Groundnuts (Shelled Eq)	2
115	Cambodia	2010	Maize	32
115	Cambodia	2010	Marine Fish, Other	11
115	Cambodia	2010	Milk, Whole	5
115	Cambodia	2010	Molluscs, Other	1
115	Cambodia	2010	Nuts	1
115	Cambodia	2010	Offals, Edible	4
115	Cambodia	2010	Oilcrops Oil, Other	1
115	Cambodia	2010	Oranges, Mandarines	11
115	Cambodia	2010	Pigmeat	21
115	Cambodia	2010	Pimento	2
115	Cambodia	2010	Pineapples	4
115	Cambodia	2010	Poultry Meat	5
115	Cambodia	2010	Rice (Milled Equivalent)	438
115	Cambodia	2010	Roots, Other	6
115	Cambodia	2010	Sesameseed	6
115	Cambodia	2010	Soyabean Oil	2
115	Cambodia	2010	Soyabeans	9
115	Cambodia	2010	Sugar Cane	14
115	Cambodia	2010	Sugar, Refined Equivalent	47
115	Cambodia	2010	Sweet Potatoes	14
115	Cambodia	2010	Vegetables, Other	92
115	Cambodia	2010	Wheat	6
115	Cambodia	2011	Bananas	29
115	Cambodia	2011	Beans	13
115	Cambodia	2011	Beer	51
115	Cambodia	2011	Beverages, Alcoholic	4
115	Cambodia	2011	Bovine Meat	14
115	Cambodia	2011	Cassava	74
115	Cambodia	2011	Cephalopods	1
115	Cambodia	2011	Cereals, Other	1
115	Cambodia	2011	Coconut Oil	2
115	Cambodia	2011	Crustaceans	3
115	Cambodia	2011	Eggs	4
115	Cambodia	2011	Fats, Animals, Raw	2
115	Cambodia	2011	Freshwater Fish	81
115	Cambodia	2011	Fruits, Other	24
115	Cambodia	2011	Grapefruit	2
115	Cambodia	2011	Groundnuts (Shelled Eq)	2
115	Cambodia	2011	Maize	31
115	Cambodia	2011	Marine Fish, Other	11
115	Cambodia	2011	Milk, Whole	5
115	Cambodia	2011	Molluscs, Other	1
115	Cambodia	2011	Nuts	1
115	Cambodia	2011	Offals, Edible	4
115	Cambodia	2011	Oilcrops Oil, Other	1
115	Cambodia	2011	Oranges, Mandarines	10
115	Cambodia	2011	Pepper	1
115	Cambodia	2011	Pigmeat	22
115	Cambodia	2011	Pimento	2
115	Cambodia	2011	Pineapples	4
115	Cambodia	2011	Poultry Meat	5
115	Cambodia	2011	Rice (Milled Equivalent)	436
115	Cambodia	2011	Roots, Other	6
115	Cambodia	2011	Sesameseed	7
115	Cambodia	2011	Soyabean Oil	2
115	Cambodia	2011	Soyabeans	10
115	Cambodia	2011	Sugar Cane	18
115	Cambodia	2011	Sugar, Refined Equivalent	45
115	Cambodia	2011	Sweet Potatoes	8
115	Cambodia	2011	Sweeteners, Other	1
115	Cambodia	2011	Vegetables, Other	106
115	Cambodia	2011	Wheat	7
32	Cameroon	1992	Bananas	145
32	Cameroon	1992	Barley	3
32	Cameroon	1992	Beans	15
32	Cameroon	1992	Beer	54
32	Cameroon	1992	Beverages, Fermented	78
32	Cameroon	1992	Bovine Meat	17
32	Cameroon	1992	Cassava	263
32	Cameroon	1992	Cocoa Beans	1
32	Cameroon	1992	Coffee	1
32	Cameroon	1992	Cottonseed Oil	2
32	Cameroon	1992	Demersal Fish	3
32	Cameroon	1992	Eggs	2
32	Cameroon	1992	Fats, Animals, Raw	1
32	Cameroon	1992	Freshwater Fish	5
32	Cameroon	1992	Fruits, Other	26
32	Cameroon	1992	Groundnut Oil	2
32	Cameroon	1992	Groundnuts (Shelled Eq)	2
32	Cameroon	1992	Honey	1
32	Cameroon	1992	Maize	112
32	Cameroon	1992	Marine Fish, Other	11
32	Cameroon	1992	Meat, Other	10
32	Cameroon	1992	Milk, Whole	39
32	Cameroon	1992	Millet	11
32	Cameroon	1992	Mutton & Goat Meat	6
32	Cameroon	1992	Nuts	6
32	Cameroon	1992	Offals, Edible	4

FAO country code	Country	Reference year	Food item	Gross food supply (g $capita^{-1}$ d^{-1})
32	Cameroon	1992	Oilcrops Oil, Other	1
32	Cameroon	1992	Oilcrops, Other	8
32	Cameroon	1992	Onions	4
32	Cameroon	1992	Palm Oil	13
32	Cameroon	1992	Palmkernel Oil	1
32	Cameroon	1992	Pelagic Fish	11
32	Cameroon	1992	Pigmeat	3
32	Cameroon	1992	Pimento	2
32	Cameroon	1992	Pineapples	7
32	Cameroon	1992	Plantains	133
32	Cameroon	1992	Potatoes	5
32	Cameroon	1992	Poultry Meat	4
32	Cameroon	1992	Pulses, Other	1
32	Cameroon	1992	Rice (Milled Equivalent)	24
32	Cameroon	1992	Roots, Other	74
32	Cameroon	1992	Sesameseed	1
32	Cameroon	1992	Sorghum	63
32	Cameroon	1992	Soyabean Oil	1
32	Cameroon	1992	Soyabeans	1
32	Cameroon	1992	Sugar Cane	122
32	Cameroon	1992	Sugar, Refined Equivalent	16
32	Cameroon	1992	Sweet Potatoes	35
32	Cameroon	1992	Tea	1
32	Cameroon	1992	Tomatoes	14
32	Cameroon	1992	Vegetables, Other	111
32	Cameroon	1992	Wheat	67
32	Cameroon	1992	Wine	1
32	Cameroon	1992	Yams	15
32	Cameroon	1993	Bananas	147
32	Cameroon	1993	Barley	3
32	Cameroon	1993	Beans	17
32	Cameroon	1993	Beer	49
32	Cameroon	1993	Beverages, Fermented	81
32	Cameroon	1993	Bovine Meat	16
32	Cameroon	1993	Cassava	257
32	Cameroon	1993	Cocoa Beans	1
32	Cameroon	1993	Coffee	1
32	Cameroon	1993	Cottonseed Oil	2
32	Cameroon	1993	Demersal Fish	3
32	Cameroon	1993	Eggs	2
32	Cameroon	1993	Fats, Animals, Raw	1
32	Cameroon	1993	Freshwater Fish	5
32	Cameroon	1993	Fruits, Other	26
32	Cameroon	1993	Groundnut Oil	2
32	Cameroon	1993	Groundnuts (Shelled Eq)	3
32	Cameroon	1993	Honey	1
32	Cameroon	1993	Maize	96
32	Cameroon	1993	Marine Fish, Other	8
32	Cameroon	1993	Meat, Other	10
32	Cameroon	1993	Milk, Whole	40
32	Cameroon	1993	Millet	11
32	Cameroon	1993	Mutton & Goat Meat	6
32	Cameroon	1993	Nuts	7
32	Cameroon	1993	Offals, Edible	4
32	Cameroon	1993	Oilcrops Oil, Other	1
32	Cameroon	1993	Oilcrops, Other	8
32	Cameroon	1993	Onions	4
32	Cameroon	1993	Palm Oil	13
32	Cameroon	1993	Palmkernel Oil	1
32	Cameroon	1993	Pelagic Fish	9
32	Cameroon	1993	Pigmeat	3
32	Cameroon	1993	Pimento	2
32	Cameroon	1993	Pineapples	7
32	Cameroon	1993	Plantains	140
32	Cameroon	1993	Potatoes	4
32	Cameroon	1993	Poultry Meat	4
32	Cameroon	1993	Pulses, Other	1
32	Cameroon	1993	Rice (Milled Equivalent)	24
32	Cameroon	1993	Roots, Other	72
32	Cameroon	1993	Sesameseed	1
32	Cameroon	1993	Sorghum	76
32	Cameroon	1993	Soyabeans	1
32	Cameroon	1993	Sugar Cane	137
32	Cameroon	1993	Sugar, Refined Equivalent	17
32	Cameroon	1993	Sweet Potatoes	37
32	Cameroon	1993	Tea	1
32	Cameroon	1993	Tomatoes	13
32	Cameroon	1993	Vegetables, Other	115
32	Cameroon	1993	Wheat	36
32	Cameroon	1993	Wine	1
32	Cameroon	1993	Yams	13
32	Cameroon	1994	Bananas	145
32	Cameroon	1994	Barley	6
32	Cameroon	1994	Beans	18
32	Cameroon	1994	Beer	38
32	Cameroon	1994	Beverages, Fermented	73
32	Cameroon	1994	Bovine Meat	15
32	Cameroon	1994	Cassava	260
32	Cameroon	1994	Cocoa Beans	1
32	Cameroon	1994	Coffee	1
32	Cameroon	1994	Cottonseed Oil	2
32	Cameroon	1994	Demersal Fish	2
32	Cameroon	1994	Eggs	2
32	Cameroon	1994	Fats, Animals, Raw	1
32	Cameroon	1994	Freshwater Fish	5
32	Cameroon	1994	Fruits, Other	26
32	Cameroon	1994	Groundnut Oil	2

FAO country code	Country	Reference year	Food item	Gross food supply (g $capita^{-1}$ d^{-1})
32	Cameroon	1994	Groundnuts (Shelled Eq)	6
32	Cameroon	1994	Honey	1
32	Cameroon	1994	Maize	102
32	Cameroon	1994	Marine Fish, Other	7
32	Cameroon	1994	Meat, Other	10
32	Cameroon	1994	Milk, Whole	39
32	Cameroon	1994	Millet	11
32	Cameroon	1994	Mutton & Goat Meat	6
32	Cameroon	1994	Nuts	8
32	Cameroon	1994	Offals, Edible	4
32	Cameroon	1994	Oilcrops, Other	10
32	Cameroon	1994	Onions	4
32	Cameroon	1994	Palm Oil	13
32	Cameroon	1994	Palmkernel Oil	1
32	Cameroon	1994	Pelagic Fish	10
32	Cameroon	1994	Pigmeat	3
32	Cameroon	1994	Pimento	2
32	Cameroon	1994	Pineapples	8
32	Cameroon	1994	Plantains	147
32	Cameroon	1994	Potatoes	5
32	Cameroon	1994	Poultry Meat	4
32	Cameroon	1994	Pulses, Other	3
32	Cameroon	1994	Rice (Milled Equivalent)	24
32	Cameroon	1994	Roots, Other	70
32	Cameroon	1994	Sesameseed	1
32	Cameroon	1994	Sorghum	65
32	Cameroon	1994	Soyabeans	1
32	Cameroon	1994	Spices, Other	1
32	Cameroon	1994	Sugar Cane	129
32	Cameroon	1994	Sugar, Refined Equivalent	15
32	Cameroon	1994	Sweet Potatoes	38
32	Cameroon	1994	Tea	1
32	Cameroon	1994	Tomatoes	14
32	Cameroon	1994	Vegetables, Other	118
32	Cameroon	1994	Wheat	32
32	Cameroon	1994	Wine	1
32	Cameroon	1994	Yams	15
32	Cameroon	1995	Bananas	142
32	Cameroon	1995	Barley	14
32	Cameroon	1995	Beans	19
32	Cameroon	1995	Beer	34
32	Cameroon	1995	Beverages, Fermented	88
32	Cameroon	1995	Bovine Meat	14
32	Cameroon	1995	Cassava	263
32	Cameroon	1995	Cocoa Beans	1
32	Cameroon	1995	Coffee	1
32	Cameroon	1995	Cottonseed Oil	3
32	Cameroon	1995	Demersal Fish	3
32	Cameroon	1995	Eggs	2
32	Cameroon	1995	Fats, Animals, Raw	1
32	Cameroon	1995	Freshwater Fish	6
32	Cameroon	1995	Fruits, Other	26
32	Cameroon	1995	Groundnut Oil	2
32	Cameroon	1995	Groundnuts (Shelled Eq)	3
32	Cameroon	1995	Honey	1
32	Cameroon	1995	Maize	105
32	Cameroon	1995	Marine Fish, Other	8
32	Cameroon	1995	Meat, Other	10
32	Cameroon	1995	Milk, Whole	39
32	Cameroon	1995	Millet	12
32	Cameroon	1995	Mutton & Goat Meat	5
32	Cameroon	1995	Nuts	7
32	Cameroon	1995	Offals, Edible	4
32	Cameroon	1995	Oilcrops, Other	8
32	Cameroon	1995	Onions	4
32	Cameroon	1995	Palm Oil	13
32	Cameroon	1995	Palmkernel Oil	1
32	Cameroon	1995	Pelagic Fish	11
32	Cameroon	1995	Pigmeat	3
32	Cameroon	1995	Pimento	2
32	Cameroon	1995	Pineapples	8
32	Cameroon	1995	Plantains	148
32	Cameroon	1995	Potatoes	4
32	Cameroon	1995	Poultry Meat	4
32	Cameroon	1995	Pulses, Other	5
32	Cameroon	1995	Rice (Milled Equivalent)	23
32	Cameroon	1995	Roots, Other	72
32	Cameroon	1995	Sesameseed	1
32	Cameroon	1995	Sorghum	71
32	Cameroon	1995	Soyabeans	1
32	Cameroon	1995	Spices, Other	1
32	Cameroon	1995	Sugar Cane	130
32	Cameroon	1995	Sugar, Refined Equivalent	16
32	Cameroon	1995	Sweet Potatoes	38
32	Cameroon	1995	Tea	1
32	Cameroon	1995	Tomatoes	12
32	Cameroon	1995	Vegetables, Other	122
32	Cameroon	1995	Wheat	21
32	Cameroon	1995	Wine	1
32	Cameroon	1995	Yams	14
32	Cameroon	1996	Bananas	145
32	Cameroon	1996	Barley	6
32	Cameroon	1996	Beans	20
32	Cameroon	1996	Beer	29
32	Cameroon	1996	Beverages, Fermented	84
32	Cameroon	1996	Bovine Meat	17
32	Cameroon	1996	Cassava	265

FAO country code	Country	Reference year	Food item	Gross food supply (g $capita^{-1}$ d^{-1})
32	Cameroon	1996	Cocoa Beans	1
32	Cameroon	1996	Coffee	1
32	Cameroon	1996	Cottonseed Oil	3
32	Cameroon	1996	Demersal Fish	3
32	Cameroon	1996	Eggs	2
32	Cameroon	1996	Fats, Animals, Raw	1
32	Cameroon	1996	Freshwater Fish	6
32	Cameroon	1996	Fruits, Other	25
32	Cameroon	1996	Groundnut Oil	3
32	Cameroon	1996	Groundnuts (Shelled Eq)	5
32	Cameroon	1996	Honey	1
32	Cameroon	1996	Maize	110
32	Cameroon	1996	Marine Fish, Other	5
32	Cameroon	1996	Meat, Other	9
32	Cameroon	1996	Milk, Whole	38
32	Cameroon	1996	Millet	12
32	Cameroon	1996	Mutton & Goat Meat	5
32	Cameroon	1996	Nuts	7
32	Cameroon	1996	Offals, Edible	3
32	Cameroon	1996	Oilcrops, Other	7
32	Cameroon	1996	Onions	3
32	Cameroon	1996	Palm Oil	11
32	Cameroon	1996	Palmkernel Oil	1
32	Cameroon	1996	Pelagic Fish	13
32	Cameroon	1996	Pigmeat	2
32	Cameroon	1996	Pimento	2
32	Cameroon	1996	Pineapples	8
32	Cameroon	1996	Plantains	148
32	Cameroon	1996	Potatoes	4
32	Cameroon	1996	Poultry Meat	5
32	Cameroon	1996	Pulses, Other	7
32	Cameroon	1996	Rice (Milled Equivalent)	28
32	Cameroon	1996	Roots, Other	50
32	Cameroon	1996	Sesameseed	1
32	Cameroon	1996	Sorghum	66
32	Cameroon	1996	Soyabeans	1
32	Cameroon	1996	Spices, Other	1
32	Cameroon	1996	Sugar Cane	103
32	Cameroon	1996	Sugar, Refined Equivalent	17
32	Cameroon	1996	Sweet Potatoes	38
32	Cameroon	1996	Tea	1
32	Cameroon	1996	Tomatoes	12
32	Cameroon	1996	Vegetables, Other	126
32	Cameroon	1996	Wheat	22
32	Cameroon	1996	Wine	1
32	Cameroon	1996	Yams	14
32	Cameroon	1997	Bananas	105
32	Cameroon	1997	Barley	6
32	Cameroon	1997	Beans	21
32	Cameroon	1997	Beer	28
32	Cameroon	1997	Beverages, Fermented	77
32	Cameroon	1997	Bovine Meat	14
32	Cameroon	1997	Cassava	268
32	Cameroon	1997	Cocoa Beans	1
32	Cameroon	1997	Coffee	1
32	Cameroon	1997	Cottonseed Oil	4
32	Cameroon	1997	Demersal Fish	3
32	Cameroon	1997	Eggs	2
32	Cameroon	1997	Fats, Animals, Raw	1
32	Cameroon	1997	Freshwater Fish	7
32	Cameroon	1997	Fruits, Other	25
32	Cameroon	1997	Groundnut Oil	2
32	Cameroon	1997	Groundnuts (Shelled Eq)	4
32	Cameroon	1997	Honey	1
32	Cameroon	1997	Maize	106
32	Cameroon	1997	Marine Fish, Other	9
32	Cameroon	1997	Meat, Other	9
32	Cameroon	1997	Milk, Whole	39
32	Cameroon	1997	Millet	12
32	Cameroon	1997	Mutton & Goat Meat	5
32	Cameroon	1997	Nuts	7
32	Cameroon	1997	Offals, Edible	3
32	Cameroon	1997	Oilcrops, Other	7
32	Cameroon	1997	Onions	7
32	Cameroon	1997	Palm Oil	12
32	Cameroon	1997	Palmkernel Oil	1
32	Cameroon	1997	Pelagic Fish	13
32	Cameroon	1997	Pigmeat	2
32	Cameroon	1997	Pimento	1
32	Cameroon	1997	Pineapples	7
32	Cameroon	1997	Plantains	148
32	Cameroon	1997	Potatoes	4
32	Cameroon	1997	Poultry Meat	6
32	Cameroon	1997	Pulses, Other	8
32	Cameroon	1997	Rice (Milled Equivalent)	31
32	Cameroon	1997	Roots, Other	93
32	Cameroon	1997	Sesameseed	1
32	Cameroon	1997	Sorghum	58
32	Cameroon	1997	Soyabeans	1
32	Cameroon	1997	Spices, Other	1
32	Cameroon	1997	Sugar Cane	100
32	Cameroon	1997	Sugar, Refined Equivalent	22
32	Cameroon	1997	Sweet Potatoes	38
32	Cameroon	1997	Tea	1
32	Cameroon	1997	Tomatoes	12
32	Cameroon	1997	Vegetables, Other	130
32	Cameroon	1997	Wheat	38

FAO country code	Country	Reference year	Food item	Gross food supply (g *capita*$^{-1}$ d^{-1})
32	Cameroon	1997	Wine	1
32	Cameroon	1997	Yams	17
32	Cameroon	1998	Bananas	99
32	Cameroon	1998	Barley	6
32	Cameroon	1998	Beans	21
32	Cameroon	1998	Beer	43
32	Cameroon	1998	Beverages, Fermented	90
32	Cameroon	1998	Bovine Meat	14
32	Cameroon	1998	Cassava	267
32	Cameroon	1998	Cocoa Beans	1
32	Cameroon	1998	Coffee	1
32	Cameroon	1998	Cottonseed Oil	3
32	Cameroon	1998	Demersal Fish	3
32	Cameroon	1998	Eggs	2
32	Cameroon	1998	Fats, Animals, Raw	1
32	Cameroon	1998	Freshwater Fish	8
32	Cameroon	1998	Fruits, Other	25
32	Cameroon	1998	Groundnut Oil	1
32	Cameroon	1998	Groundnuts (Shelled Eq)	5
32	Cameroon	1998	Honey	1
32	Cameroon	1998	Maize	107
32	Cameroon	1998	Marine Fish, Other	12
32	Cameroon	1998	Meat, Other	9
32	Cameroon	1998	Milk, Whole	37
32	Cameroon	1998	Millet	11
32	Cameroon	1998	Mutton & Goat Meat	5
32	Cameroon	1998	Nuts	7
32	Cameroon	1998	Offals, Edible	3
32	Cameroon	1998	Oilcrops, Other	7
32	Cameroon	1998	Onions	7
32	Cameroon	1998	Palm Oil	14
32	Cameroon	1998	Palmkernel Oil	1
32	Cameroon	1998	Pelagic Fish	12
32	Cameroon	1998	Pigmeat	3
32	Cameroon	1998	Pimento	1
32	Cameroon	1998	Pineapples	8
32	Cameroon	1998	Plantains	148
32	Cameroon	1998	Potatoes	4
32	Cameroon	1998	Poultry Meat	6
32	Cameroon	1998	Pulses, Other	10
32	Cameroon	1998	Rice (Milled Equivalent)	26
32	Cameroon	1998	Roots, Other	78
32	Cameroon	1998	Sesameseed	1
32	Cameroon	1998	Sorghum	73
32	Cameroon	1998	Soyabeans	1
32	Cameroon	1998	Spices, Other	1
32	Cameroon	1998	Sugar Cane	98
32	Cameroon	1998	Sugar, Refined Equivalent	18
32	Cameroon	1998	Sweet Potatoes	23
32	Cameroon	1998	Sweeteners, Other	1
32	Cameroon	1998	Tea	1
32	Cameroon	1998	Tomatoes	13
32	Cameroon	1998	Vegetables, Other	136
32	Cameroon	1998	Wheat	29
32	Cameroon	1998	Wine	1
32	Cameroon	1998	Yams	27
32	Cameroon	1999	Bananas	73
32	Cameroon	1999	Beans	24
32	Cameroon	1999	Beer	64
32	Cameroon	1999	Beverages, Fermented	54
32	Cameroon	1999	Bovine Meat	16
32	Cameroon	1999	Cassava	250
32	Cameroon	1999	Cocoa Beans	1
32	Cameroon	1999	Coffee	1
32	Cameroon	1999	Cottonseed Oil	3
32	Cameroon	1999	Demersal Fish	2
32	Cameroon	1999	Eggs	2
32	Cameroon	1999	Fats, Animals, Raw	1
32	Cameroon	1999	Freshwater Fish	9
32	Cameroon	1999	Fruits, Other	24
32	Cameroon	1999	Groundnut Oil	2
32	Cameroon	1999	Groundnuts (Shelled Eq)	7
32	Cameroon	1999	Honey	1
32	Cameroon	1999	Maize	135
32	Cameroon	1999	Marine Fish, Other	11
32	Cameroon	1999	Meat, Other	9
32	Cameroon	1999	Milk, Whole	39
32	Cameroon	1999	Millet	10
32	Cameroon	1999	Mutton & Goat Meat	5
32	Cameroon	1999	Nuts	6
32	Cameroon	1999	Offals, Edible	4
32	Cameroon	1999	Oilcrops, Other	7
32	Cameroon	1999	Onions	9
32	Cameroon	1999	Palm Oil	14
32	Cameroon	1999	Palmkernel Oil	1
32	Cameroon	1999	Pelagic Fish	12
32	Cameroon	1999	Pigmeat	2
32	Cameroon	1999	Pimento	1
32	Cameroon	1999	Pineapples	6
32	Cameroon	1999	Plantains	123
32	Cameroon	1999	Potatoes	17
32	Cameroon	1999	Poultry Meat	7
32	Cameroon	1999	Pulses, Other	12
32	Cameroon	1999	Rice (Milled Equivalent)	34
32	Cameroon	1999	Roots, Other	76
32	Cameroon	1999	Sorghum	37
32	Cameroon	1999	Soyabean Oil	1

FAO country code	Country	Reference year	Food item	Gross food supply (g $capita^{-1}$ d^{-1})
32	Cameroon	1999	Soyabeans	1
32	Cameroon	1999	Spices, Other	1
32	Cameroon	1999	Sugar Cane	95
32	Cameroon	1999	Sugar, Refined Equivalent	17
32	Cameroon	1999	Sweet Potatoes	28
32	Cameroon	1999	Tea	1
32	Cameroon	1999	Tomatoes	56
32	Cameroon	1999	Vegetables, Other	144
32	Cameroon	1999	Wheat	42
32	Cameroon	1999	Wine	1
32	Cameroon	1999	Yams	28
32	Cameroon	2000	Bananas	59
32	Cameroon	2000	Beans	24
32	Cameroon	2000	Beer	63
32	Cameroon	2000	Beverages, Fermented	71
32	Cameroon	2000	Bovine Meat	16
32	Cameroon	2000	Cassava	247
32	Cameroon	2000	Cocoa Beans	1
32	Cameroon	2000	Coffee	1
32	Cameroon	2000	Cottonseed Oil	3
32	Cameroon	2000	Demersal Fish	2
32	Cameroon	2000	Eggs	2
32	Cameroon	2000	Fats, Animals, Raw	1
32	Cameroon	2000	Freshwater Fish	9
32	Cameroon	2000	Fruits, Other	24
32	Cameroon	2000	Groundnut Oil	1
32	Cameroon	2000	Groundnuts (Shelled Eq)	11
32	Cameroon	2000	Honey	1
32	Cameroon	2000	Maize	118
32	Cameroon	2000	Marine Fish, Other	10
32	Cameroon	2000	Meat, Other	9
32	Cameroon	2000	Milk, Whole	38
32	Cameroon	2000	Millet	8
32	Cameroon	2000	Mutton & Goat Meat	5
32	Cameroon	2000	Nuts	6
32	Cameroon	2000	Offals, Edible	4
32	Cameroon	2000	Oilcrops Oil, Other	1
32	Cameroon	2000	Oilcrops, Other	7
32	Cameroon	2000	Onions	12
32	Cameroon	2000	Palm Oil	14
32	Cameroon	2000	Palmkernel Oil	1
32	Cameroon	2000	Pelagic Fish	13
32	Cameroon	2000	Pigmeat	3
32	Cameroon	2000	Pimento	1
32	Cameroon	2000	Pineapples	6
32	Cameroon	2000	Plantains	120
32	Cameroon	2000	Potatoes	17
32	Cameroon	2000	Poultry Meat	6
32	Cameroon	2000	Pulses, Other	13
32	Cameroon	2000	Rice (Milled Equivalent)	27
32	Cameroon	2000	Roots, Other	74
32	Cameroon	2000	Sesameseed	1
32	Cameroon	2000	Sorghum	56
32	Cameroon	2000	Soyabean Oil	1
32	Cameroon	2000	Soyabeans	1
32	Cameroon	2000	Spices, Other	1
32	Cameroon	2000	Sugar Cane	93
32	Cameroon	2000	Sugar, Refined Equivalent	21
32	Cameroon	2000	Sweet Potatoes	26
32	Cameroon	2000	Tea	1
32	Cameroon	2000	Tomatoes	59
32	Cameroon	2000	Vegetables, Other	143
32	Cameroon	2000	Wheat	37
32	Cameroon	2000	Wine	1
32	Cameroon	2000	Yams	27
32	Cameroon	2001	Bananas	56
32	Cameroon	2001	Beans	23
32	Cameroon	2001	Beer	68
32	Cameroon	2001	Beverages, Fermented	80
32	Cameroon	2001	Bovine Meat	16
32	Cameroon	2001	Cassava	245
32	Cameroon	2001	Cocoa Beans	1
32	Cameroon	2001	Coffee	1
32	Cameroon	2001	Cottonseed Oil	3
32	Cameroon	2001	Crustaceans	2
32	Cameroon	2001	Demersal Fish	3
32	Cameroon	2001	Eggs	2
32	Cameroon	2001	Fats, Animals, Raw	1
32	Cameroon	2001	Freshwater Fish	9
32	Cameroon	2001	Fruits, Other	24
32	Cameroon	2001	Groundnut Oil	1
32	Cameroon	2001	Groundnuts (Shelled Eq)	12
32	Cameroon	2001	Honey	1
32	Cameroon	2001	Maize	97
32	Cameroon	2001	Marine Fish, Other	11
32	Cameroon	2001	Meat, Other	9
32	Cameroon	2001	Milk, Whole	38
32	Cameroon	2001	Millet	8
32	Cameroon	2001	Mutton & Goat Meat	5
32	Cameroon	2001	Nuts	6
32	Cameroon	2001	Offals, Edible	4
32	Cameroon	2001	Oilcrops Oil, Other	1
32	Cameroon	2001	Oilcrops, Other	7
32	Cameroon	2001	Onions	10
32	Cameroon	2001	Palm Oil	10
32	Cameroon	2001	Palmkernel Oil	1
32	Cameroon	2001	Pelagic Fish	15

FAO country code	Country	Reference year	Food item	Gross food supply (g $capita^{-1}$ d^{-1})
32	Cameroon	2001	Pigmeat	3
32	Cameroon	2001	Pimento	1
32	Cameroon	2001	Pineapples	6
32	Cameroon	2001	Plantains	119
32	Cameroon	2001	Potatoes	16
32	Cameroon	2001	Poultry Meat	6
32	Cameroon	2001	Pulses, Other	15
32	Cameroon	2001	Rice (Milled Equivalent)	47
32	Cameroon	2001	Roots, Other	72
32	Cameroon	2001	Sesameseed	1
32	Cameroon	2001	Sorghum	67
32	Cameroon	2001	Soyabean Oil	1
32	Cameroon	2001	Soyabeans	1
32	Cameroon	2001	Spices, Other	1
32	Cameroon	2001	Sugar Cane	90
32	Cameroon	2001	Sugar, Refined Equivalent	22
32	Cameroon	2001	Sweet Potatoes	26
32	Cameroon	2001	Tea	1
32	Cameroon	2001	Tomatoes	57
32	Cameroon	2001	Vegetables, Other	140
32	Cameroon	2001	Wheat	49
32	Cameroon	2001	Wine	1
32	Cameroon	2001	Yams	27
32	Cameroon	2002	Bananas	66
32	Cameroon	2002	Barley	1
32	Cameroon	2002	Beans	24
32	Cameroon	2002	Beer	71
32	Cameroon	2002	Beverages, Fermented	83
32	Cameroon	2002	Bovine Meat	15
32	Cameroon	2002	Cassava	245
32	Cameroon	2002	Coffee	1
32	Cameroon	2002	Cottonseed Oil	3
32	Cameroon	2002	Crustaceans	2
32	Cameroon	2002	Demersal Fish	2
32	Cameroon	2002	Eggs	2
32	Cameroon	2002	Fats, Animals, Raw	1
32	Cameroon	2002	Freshwater Fish	11
32	Cameroon	2002	Fruits, Other	24
32	Cameroon	2002	Groundnut Oil	3
32	Cameroon	2002	Groundnuts (Shelled Eq)	12
32	Cameroon	2002	Maize	86
32	Cameroon	2002	Marine Fish, Other	9
32	Cameroon	2002	Meat, Other	9
32	Cameroon	2002	Milk, Whole	37
32	Cameroon	2002	Millet	7
32	Cameroon	2002	Mutton & Goat Meat	5
32	Cameroon	2002	Nuts	6
32	Cameroon	2002	Offals, Edible	4
32	Cameroon	2002	Oilcrops Oil, Other	1
32	Cameroon	2002	Oilcrops, Other	6
32	Cameroon	2002	Onions	10
32	Cameroon	2002	Palm Oil	15
32	Cameroon	2002	Palmkernel Oil	1
32	Cameroon	2002	Pelagic Fish	12
32	Cameroon	2002	Pigmeat	3
32	Cameroon	2002	Pimento	1
32	Cameroon	2002	Pineapples	7
32	Cameroon	2002	Plantains	121
32	Cameroon	2002	Potatoes	15
32	Cameroon	2002	Poultry Meat	8
32	Cameroon	2002	Pulses, Other	15
32	Cameroon	2002	Rice (Milled Equivalent)	47
32	Cameroon	2002	Roots, Other	70
32	Cameroon	2002	Sesameseed	1
32	Cameroon	2002	Sorghum	70
32	Cameroon	2002	Soyabean Oil	2
32	Cameroon	2002	Soyabeans	1
32	Cameroon	2002	Spices, Other	1
32	Cameroon	2002	Sugar Cane	66
32	Cameroon	2002	Sugar, Refined Equivalent	26
32	Cameroon	2002	Sweet Potatoes	26
32	Cameroon	2002	Sweeteners, Other	1
32	Cameroon	2002	Tea	1
32	Cameroon	2002	Tomatoes	59
32	Cameroon	2002	Vegetables, Other	142
32	Cameroon	2002	Wheat	49
32	Cameroon	2002	Wine	1
32	Cameroon	2002	Yams	27
32	Cameroon	2003	Bananas	60
32	Cameroon	2003	Barley	1
32	Cameroon	2003	Beans	24
32	Cameroon	2003	Beer	72
32	Cameroon	2003	Beverages, Fermented	84
32	Cameroon	2003	Bovine Meat	14
32	Cameroon	2003	Cassava	244
32	Cameroon	2003	Coffee	1
32	Cameroon	2003	Cottonseed Oil	3
32	Cameroon	2003	Crustaceans	2
32	Cameroon	2003	Demersal Fish	2
32	Cameroon	2003	Eggs	2
32	Cameroon	2003	Fats, Animals, Raw	1
32	Cameroon	2003	Freshwater Fish	9
32	Cameroon	2003	Fruits, Other	24
32	Cameroon	2003	Groundnut Oil	3
32	Cameroon	2003	Groundnuts (Shelled Eq)	12
32	Cameroon	2003	Honey	1
32	Cameroon	2003	Maize	96

FAO country code	Country	Reference year	Food item	Gross food supply (g $capita^{-1}$ d^{-1})
32	Cameroon	2003	Marine Fish, Other	15
32	Cameroon	2003	Meat, Other	9
32	Cameroon	2003	Milk, Whole	40
32	Cameroon	2003	Millet	8
32	Cameroon	2003	Mutton & Goat Meat	5
32	Cameroon	2003	Nuts	6
32	Cameroon	2003	Offals, Edible	4
32	Cameroon	2003	Oilcrops, Other	7
32	Cameroon	2003	Onions	10
32	Cameroon	2003	Palm Oil	15
32	Cameroon	2003	Palmkernel Oil	1
32	Cameroon	2003	Pelagic Fish	14
32	Cameroon	2003	Pigmeat	3
32	Cameroon	2003	Pimento	2
32	Cameroon	2003	Pineapples	7
32	Cameroon	2003	Plantains	122
32	Cameroon	2003	Potatoes	14
32	Cameroon	2003	Poultry Meat	8
32	Cameroon	2003	Pulses, Other	15
32	Cameroon	2003	Rice (Milled Equivalent)	51
32	Cameroon	2003	Roots, Other	69
32	Cameroon	2003	Sesameseed	1
32	Cameroon	2003	Sorghum	72
32	Cameroon	2003	Soyabean Oil	3
32	Cameroon	2003	Soyabeans	1
32	Cameroon	2003	Spices, Other	1
32	Cameroon	2003	Sugar Cane	61
32	Cameroon	2003	Sugar, Refined Equivalent	24
32	Cameroon	2003	Sweet Potatoes	26
32	Cameroon	2003	Sweeteners, Other	1
32	Cameroon	2003	Tea	1
32	Cameroon	2003	Tomatoes	59
32	Cameroon	2003	Vegetables, Other	137
32	Cameroon	2003	Wheat	44
32	Cameroon	2003	Wine	1
32	Cameroon	2003	Yams	27
32	Cameroon	2004	Bananas	69
32	Cameroon	2004	Barley	1
32	Cameroon	2004	Beans	25
32	Cameroon	2004	Beer	73
32	Cameroon	2004	Beverages, Fermented	84
32	Cameroon	2004	Bovine Meat	14
32	Cameroon	2004	Cassava	243
32	Cameroon	2004	Coffee	1
32	Cameroon	2004	Cottonseed Oil	3
32	Cameroon	2004	Crustaceans	2
32	Cameroon	2004	Demersal Fish	1
32	Cameroon	2004	Eggs	1
32	Cameroon	2004	Fats, Animals, Raw	1
32	Cameroon	2004	Freshwater Fish	10
32	Cameroon	2004	Fruits, Other	23
32	Cameroon	2004	Groundnut Oil	3
32	Cameroon	2004	Groundnuts (Shelled Eq)	12
32	Cameroon	2004	Honey	1
32	Cameroon	2004	Maize	97
32	Cameroon	2004	Marine Fish, Other	7
32	Cameroon	2004	Meat, Other	9
32	Cameroon	2004	Milk, Whole	40
32	Cameroon	2004	Millet	9
32	Cameroon	2004	Mutton & Goat Meat	5
32	Cameroon	2004	Nuts	6
32	Cameroon	2004	Offals, Edible	4
32	Cameroon	2004	Oilcrops Oil, Other	1
32	Cameroon	2004	Oilcrops, Other	7
32	Cameroon	2004	Onions	10
32	Cameroon	2004	Palm Oil	14
32	Cameroon	2004	Palmkernel Oil	1
32	Cameroon	2004	Pelagic Fish	21
32	Cameroon	2004	Pigmeat	5
32	Cameroon	2004	Pimento	1
32	Cameroon	2004	Pineapples	7
32	Cameroon	2004	Plantains	122
32	Cameroon	2004	Potatoes	13
32	Cameroon	2004	Poultry Meat	13
32	Cameroon	2004	Pulses, Other	16
32	Cameroon	2004	Rice (Milled Equivalent)	52
32	Cameroon	2004	Roots, Other	67
32	Cameroon	2004	Sesameseed	1
32	Cameroon	2004	Sorghum	71
32	Cameroon	2004	Soyabean Oil	2
32	Cameroon	2004	Soyabeans	1
32	Cameroon	2004	Spices, Other	1
32	Cameroon	2004	Sugar Cane	67
32	Cameroon	2004	Sugar, Refined Equivalent	24
32	Cameroon	2004	Sweet Potatoes	26
32	Cameroon	2004	Tea	1
32	Cameroon	2004	Tomatoes	58
32	Cameroon	2004	Vegetables, Other	137
32	Cameroon	2004	Wheat	43
32	Cameroon	2004	Yams	27
32	Cameroon	2005	Bananas	91
32	Cameroon	2005	Barley	1
32	Cameroon	2005	Beans	27
32	Cameroon	2005	Beer	64
32	Cameroon	2005	Beverages, Fermented	92
32	Cameroon	2005	Bovine Meat	14
32	Cameroon	2005	Cassava	248

FAO country code	Country	Reference year	Food item	Gross food supply (g $capita^{-1}$ d^{-1})
32	Cameroon	2005	Coffee	2
32	Cameroon	2005	Cottonseed Oil	2
32	Cameroon	2005	Crustaceans	2
32	Cameroon	2005	Demersal Fish	1
32	Cameroon	2005	Eggs	1
32	Cameroon	2005	Fats, Animals, Raw	1
32	Cameroon	2005	Freshwater Fish	11
32	Cameroon	2005	Fruits, Other	23
32	Cameroon	2005	Grapes	1
32	Cameroon	2005	Groundnut Oil	3
32	Cameroon	2005	Groundnuts (Shelled Eq)	18
32	Cameroon	2005	Maize	97
32	Cameroon	2005	Marine Fish, Other	5
32	Cameroon	2005	Meat, Other	9
32	Cameroon	2005	Milk, Whole	39
32	Cameroon	2005	Millet	9
32	Cameroon	2005	Mutton & Goat Meat	5
32	Cameroon	2005	Nuts	6
32	Cameroon	2005	Offals, Edible	4
32	Cameroon	2005	Oilcrops, Other	9
32	Cameroon	2005	Onions	11
32	Cameroon	2005	Palm Oil	14
32	Cameroon	2005	Palmkernel Oil	1
32	Cameroon	2005	Pelagic Fish	20
32	Cameroon	2005	Pigmeat	5
32	Cameroon	2005	Pimento	2
32	Cameroon	2005	Pineapples	13
32	Cameroon	2005	Plantains	122
32	Cameroon	2005	Potatoes	12
32	Cameroon	2005	Poultry Meat	9
32	Cameroon	2005	Pulses, Other	16
32	Cameroon	2005	Rice (Milled Equivalent)	55
32	Cameroon	2005	Roots, Other	67
32	Cameroon	2005	Sesameseed	1
32	Cameroon	2005	Sorghum	72
32	Cameroon	2005	Soyabean Oil	1
32	Cameroon	2005	Soyabeans	1
32	Cameroon	2005	Spices, Other	1
32	Cameroon	2005	Sugar Cane	67
32	Cameroon	2005	Sugar, Refined Equivalent	23
32	Cameroon	2005	Sweet Potatoes	22
32	Cameroon	2005	Tea	1
32	Cameroon	2005	Tomatoes	68
32	Cameroon	2005	Vegetables, Other	138
32	Cameroon	2005	Wheat	45
32	Cameroon	2005	Wine	1
32	Cameroon	2005	Yams	28
32	Cameroon	2006	Bananas	95
32	Cameroon	2006	Beans	30
32	Cameroon	2006	Beer	64
32	Cameroon	2006	Beverages, Fermented	95
32	Cameroon	2006	Bovine Meat	14
32	Cameroon	2006	Cassava	253
32	Cameroon	2006	Coffee	2
32	Cameroon	2006	Cottonseed Oil	3
32	Cameroon	2006	Crustaceans	2
32	Cameroon	2006	Demersal Fish	1
32	Cameroon	2006	Eggs	1
32	Cameroon	2006	Fats, Animals, Raw	1
32	Cameroon	2006	Freshwater Fish	11
32	Cameroon	2006	Fruits, Other	25
32	Cameroon	2006	Groundnut Oil	3
32	Cameroon	2006	Groundnuts (Shelled Eq)	18
32	Cameroon	2006	Maize	105
32	Cameroon	2006	Marine Fish, Other	3
32	Cameroon	2006	Meat, Other	9
32	Cameroon	2006	Milk, Whole	42
32	Cameroon	2006	Millet	9
32	Cameroon	2006	Mutton & Goat Meat	5
32	Cameroon	2006	Nuts	5
32	Cameroon	2006	Offals, Edible	4
32	Cameroon	2006	Oilcrops, Other	7
32	Cameroon	2006	Onions	13
32	Cameroon	2006	Palm Oil	14
32	Cameroon	2006	Palmkernel Oil	1
32	Cameroon	2006	Pelagic Fish	20
32	Cameroon	2006	Pigmeat	5
32	Cameroon	2006	Pimento	3
32	Cameroon	2006	Pineapples	13
32	Cameroon	2006	Plantains	119
32	Cameroon	2006	Potatoes	12
32	Cameroon	2006	Poultry Meat	9
32	Cameroon	2006	Pulses, Other	18
32	Cameroon	2006	Rice (Milled Equivalent)	54
32	Cameroon	2006	Roots, Other	68
32	Cameroon	2006	Sesameseed	1
32	Cameroon	2006	Sorghum	74
32	Cameroon	2006	Soyabean Oil	1
32	Cameroon	2006	Soyabeans	1
32	Cameroon	2006	Spices, Other	2
32	Cameroon	2006	Sugar Cane	48
32	Cameroon	2006	Sugar, Refined Equivalent	21
32	Cameroon	2006	Sweet Potatoes	28
32	Cameroon	2006	Tea	1
32	Cameroon	2006	Tomatoes	71
32	Cameroon	2006	Vegetables, Other	158
32	Cameroon	2006	Wheat	46

FAO country code	Country	Reference year	Food item	Gross food supply (g $capita^{-1}$ d^{-1})
32	Cameroon	2006	Wine	1
32	Cameroon	2006	Yams	32
32	Cameroon	2007	Bananas	96
32	Cameroon	2007	Barley	1
32	Cameroon	2007	Beans	31
32	Cameroon	2007	Beer	64
32	Cameroon	2007	Beverages, Fermented	97
32	Cameroon	2007	Bovine Meat	14
32	Cameroon	2007	Cassava	259
32	Cameroon	2007	Cocoa Beans	1
32	Cameroon	2007	Cottonseed Oil	2
32	Cameroon	2007	Crustaceans	2
32	Cameroon	2007	Demersal Fish	1
32	Cameroon	2007	Eggs	1
32	Cameroon	2007	Fats, Animals, Raw	1
32	Cameroon	2007	Freshwater Fish	11
32	Cameroon	2007	Fruits, Other	22
32	Cameroon	2007	Grapes	1
32	Cameroon	2007	Groundnut Oil	4
32	Cameroon	2007	Groundnuts (Shelled Eq)	18
32	Cameroon	2007	Maize	105
32	Cameroon	2007	Marine Fish, Other	6
32	Cameroon	2007	Meat, Other	9
32	Cameroon	2007	Milk, Whole	39
32	Cameroon	2007	Millet	9
32	Cameroon	2007	Mutton & Goat Meat	5
32	Cameroon	2007	Nuts	5
32	Cameroon	2007	Offals, Edible	4
32	Cameroon	2007	Oilcrops, Other	6
32	Cameroon	2007	Onions	14
32	Cameroon	2007	Palm Oil	14
32	Cameroon	2007	Palmkernel Oil	1
32	Cameroon	2007	Pelagic Fish	22
32	Cameroon	2007	Pigmeat	5
32	Cameroon	2007	Pimento	3
32	Cameroon	2007	Pineapples	14
32	Cameroon	2007	Plantains	121
32	Cameroon	2007	Potatoes	12
32	Cameroon	2007	Poultry Meat	10
32	Cameroon	2007	Pulses, Other	19
32	Cameroon	2007	Rice (Milled Equivalent)	53
32	Cameroon	2007	Roots, Other	68
32	Cameroon	2007	Sesameseed	1
32	Cameroon	2007	Sorghum	74
32	Cameroon	2007	Soyabean Oil	1
32	Cameroon	2007	Soyabeans	1
32	Cameroon	2007	Spices, Other	2
32	Cameroon	2007	Sugar Cane	33
32	Cameroon	2007	Sugar, Refined Equivalent	25
32	Cameroon	2007	Sweet Potatoes	26
32	Cameroon	2007	Tea	1
32	Cameroon	2007	Tomatoes	73
32	Cameroon	2007	Vegetables, Other	178
32	Cameroon	2007	Wheat	45
32	Cameroon	2007	Wine	1
32	Cameroon	2007	Yams	33
32	Cameroon	2008	Apples	1
32	Cameroon	2008	Bananas	94
32	Cameroon	2008	Beans	32
32	Cameroon	2008	Beer	70
32	Cameroon	2008	Beverages, Fermented	99
32	Cameroon	2008	Bovine Meat	15
32	Cameroon	2008	Cassava	263
32	Cameroon	2008	Cocoa Beans	1
32	Cameroon	2008	Coffee	2
32	Cameroon	2008	Cottonseed Oil	2
32	Cameroon	2008	Crustaceans	2
32	Cameroon	2008	Demersal Fish	1
32	Cameroon	2008	Eggs	1
32	Cameroon	2008	Fats, Animals, Raw	1
32	Cameroon	2008	Freshwater Fish	11
32	Cameroon	2008	Fruits, Other	22
32	Cameroon	2008	Groundnut Oil	5
32	Cameroon	2008	Groundnuts (Shelled Eq)	19
32	Cameroon	2008	Honey	1
32	Cameroon	2008	Maize	113
32	Cameroon	2008	Marine Fish, Other	6
32	Cameroon	2008	Meat, Other	9
32	Cameroon	2008	Milk, Whole	38
32	Cameroon	2008	Millet	10
32	Cameroon	2008	Mutton & Goat Meat	4
32	Cameroon	2008	Nuts	6
32	Cameroon	2008	Offals, Edible	4
32	Cameroon	2008	Oilcrops Oil, Other	1
32	Cameroon	2008	Oilcrops, Other	6
32	Cameroon	2008	Onions	14
32	Cameroon	2008	Palm Oil	14
32	Cameroon	2008	Palmkernel Oil	1
32	Cameroon	2008	Pelagic Fish	24
32	Cameroon	2008	Pigmeat	5
32	Cameroon	2008	Pimento	3
32	Cameroon	2008	Pineapples	15
32	Cameroon	2008	Plantains	122
32	Cameroon	2008	Potatoes	12
32	Cameroon	2008	Poultry Meat	10
32	Cameroon	2008	Pulses, Other	20
32	Cameroon	2008	Rice (Milled Equivalent)	55

FAO country code	Country	Reference year	Food item	Gross food supply (g *capita*$^{-1}$ d^{-1})
32	Cameroon	2008	Roots, Other	68
32	Cameroon	2008	Sesameseed	1
32	Cameroon	2008	Sorghum	75
32	Cameroon	2008	Soyabean Oil	1
32	Cameroon	2008	Soyabeans	1
32	Cameroon	2008	Spices, Other	2
32	Cameroon	2008	Sugar Cane	35
32	Cameroon	2008	Sugar, Refined Equivalent	23
32	Cameroon	2008	Sweet Potatoes	28
32	Cameroon	2008	Tea	1
32	Cameroon	2008	Tomatoes	74
32	Cameroon	2008	Vegetables, Other	176
32	Cameroon	2008	Wheat	47
32	Cameroon	2008	Wine	1
32	Cameroon	2008	Yams	34
32	Cameroon	2009	Apples	1
32	Cameroon	2009	Bananas	104
32	Cameroon	2009	Barley	1
32	Cameroon	2009	Beans	32
32	Cameroon	2009	Beer	83
32	Cameroon	2009	Beverages, Fermented	102
32	Cameroon	2009	Bovine Meat	15
32	Cameroon	2009	Cassava	268
32	Cameroon	2009	Cocoa Beans	1
32	Cameroon	2009	Coffee	1
32	Cameroon	2009	Cottonseed Oil	2
32	Cameroon	2009	Crustaceans	2
32	Cameroon	2009	Demersal Fish	2
32	Cameroon	2009	Eggs	1
32	Cameroon	2009	Fats, Animals, Raw	1
32	Cameroon	2009	Freshwater Fish	10
32	Cameroon	2009	Fruits, Other	22
32	Cameroon	2009	Grapes	1
32	Cameroon	2009	Groundnut Oil	6
32	Cameroon	2009	Groundnuts (Shelled Eq)	19
32	Cameroon	2009	Honey	1
32	Cameroon	2009	Maize	112
32	Cameroon	2009	Marine Fish, Other	8
32	Cameroon	2009	Meat, Other	9
32	Cameroon	2009	Milk, Whole	39
32	Cameroon	2009	Millet	11
32	Cameroon	2009	Mutton & Goat Meat	4
32	Cameroon	2009	Nuts	5
32	Cameroon	2009	Offals, Edible	4
32	Cameroon	2009	Oilcrops Oil, Other	1
32	Cameroon	2009	Oilcrops, Other	6
32	Cameroon	2009	Onions	7
32	Cameroon	2009	Palm Oil	14
32	Cameroon	2009	Palmkernel Oil	1
32	Cameroon	2009	Pelagic Fish	28
32	Cameroon	2009	Pigmeat	5
32	Cameroon	2009	Pimento	3
32	Cameroon	2009	Pineapples	17
32	Cameroon	2009	Plantains	125
32	Cameroon	2009	Potatoes	13
32	Cameroon	2009	Poultry Meat	9
32	Cameroon	2009	Pulses, Other	21
32	Cameroon	2009	Rice (Milled Equivalent)	58
32	Cameroon	2009	Roots, Other	68
32	Cameroon	2009	Sesameseed	1
32	Cameroon	2009	Sorghum	76
32	Cameroon	2009	Soyabeans	2
32	Cameroon	2009	Spices, Other	2
32	Cameroon	2009	Sugar Cane	59
32	Cameroon	2009	Sugar, Refined Equivalent	19
32	Cameroon	2009	Sweet Potatoes	31
32	Cameroon	2009	Tea	1
32	Cameroon	2009	Tomatoes	83
32	Cameroon	2009	Vegetables, Other	184
32	Cameroon	2009	Wheat	49
32	Cameroon	2009	Wine	1
32	Cameroon	2009	Yams	38
32	Cameroon	2010	Apples	1
32	Cameroon	2010	Bananas	117
32	Cameroon	2010	Barley	1
32	Cameroon	2010	Beans	32
32	Cameroon	2010	Beer	78
32	Cameroon	2010	Beverages, Fermented	110
32	Cameroon	2010	Bovine Meat	14
32	Cameroon	2010	Cassava	258
32	Cameroon	2010	Cocoa Beans	2
32	Cameroon	2010	Coffee	2
32	Cameroon	2010	Cottonseed Oil	2
32	Cameroon	2010	Crustaceans	2
32	Cameroon	2010	Demersal Fish	1
32	Cameroon	2010	Eggs	1
32	Cameroon	2010	Fats, Animals, Raw	1
32	Cameroon	2010	Freshwater Fish	10
32	Cameroon	2010	Fruits, Other	22
32	Cameroon	2010	Groundnut Oil	6
32	Cameroon	2010	Groundnuts (Shelled Eq)	19
32	Cameroon	2010	Honey	1
32	Cameroon	2010	Maize	114
32	Cameroon	2010	Marine Fish, Other	7
32	Cameroon	2010	Meat, Other	9
32	Cameroon	2010	Milk, Whole	39
32	Cameroon	2010	Millet	11

FAO country code	Country	Reference year	Food item	Gross food supply (g $capita^{-1}$ d^{-1})
32	Cameroon	2010	Mutton & Goat Meat	5
32	Cameroon	2010	Nuts	5
32	Cameroon	2010	Offals, Edible	4
32	Cameroon	2010	Oilcrops Oil, Other	1
32	Cameroon	2010	Oilcrops, Other	6
32	Cameroon	2010	Onions	8
32	Cameroon	2010	Palm Oil	14
32	Cameroon	2010	Palmkernel Oil	1
32	Cameroon	2010	Pelagic Fish	22
32	Cameroon	2010	Pigmeat	5
32	Cameroon	2010	Pimento	3
32	Cameroon	2010	Pineapples	18
32	Cameroon	2010	Plantains	122
32	Cameroon	2010	Potatoes	15
32	Cameroon	2010	Poultry Meat	9
32	Cameroon	2010	Pulses, Other	22
32	Cameroon	2010	Rice (Milled Equivalent)	57
32	Cameroon	2010	Roots, Other	68
32	Cameroon	2010	Sesameseed	1
32	Cameroon	2010	Sorghum	75
32	Cameroon	2010	Soyabean Oil	1
32	Cameroon	2010	Soyabeans	2
32	Cameroon	2010	Spices, Other	2
32	Cameroon	2010	Sugar Cane	58
32	Cameroon	2010	Sugar, Refined Equivalent	24
32	Cameroon	2010	Sweet Potatoes	32
32	Cameroon	2010	Tea	1
32	Cameroon	2010	Tomatoes	97
32	Cameroon	2010	Vegetables, Other	165
32	Cameroon	2010	Wheat	49
32	Cameroon	2010	Wine	1
32	Cameroon	2010	Yams	40
32	Cameroon	2011	Apples	1
32	Cameroon	2011	Bananas	121
32	Cameroon	2011	Barley	1
32	Cameroon	2011	Beans	33
32	Cameroon	2011	Beer	78
32	Cameroon	2011	Beverages, Fermented	113
32	Cameroon	2011	Bovine Meat	15
32	Cameroon	2011	Cassava	258
32	Cameroon	2011	Cocoa Beans	2
32	Cameroon	2011	Coffee	4
32	Cameroon	2011	Cottonseed Oil	2
32	Cameroon	2011	Crustaceans	2
32	Cameroon	2011	Demersal Fish	2
32	Cameroon	2011	Eggs	1
32	Cameroon	2011	Fats, Animals, Raw	1
32	Cameroon	2011	Freshwater Fish	10
32	Cameroon	2011	Fruits, Other	26
32	Cameroon	2011	Grapes	1
32	Cameroon	2011	Groundnut Oil	6
32	Cameroon	2011	Groundnuts (Shelled Eq)	19
32	Cameroon	2011	Honey	1
32	Cameroon	2011	Maize	115
32	Cameroon	2011	Marine Fish, Other	11
32	Cameroon	2011	Meat, Other	9
32	Cameroon	2011	Milk, Whole	39
32	Cameroon	2011	Millet	11
32	Cameroon	2011	Mutton & Goat Meat	5
32	Cameroon	2011	Nuts	6
32	Cameroon	2011	Offals, Edible	4
32	Cameroon	2011	Oilcrops Oil, Other	1
32	Cameroon	2011	Oilcrops, Other	4
32	Cameroon	2011	Onions	22
32	Cameroon	2011	Palm Oil	15
32	Cameroon	2011	Palmkernel Oil	1
32	Cameroon	2011	Pelagic Fish	26
32	Cameroon	2011	Pigmeat	5
32	Cameroon	2011	Pimento	3
32	Cameroon	2011	Pineapples	18
32	Cameroon	2011	Plantains	126
32	Cameroon	2011	Potatoes	16
32	Cameroon	2011	Poultry Meat	9
32	Cameroon	2011	Pulses, Other	22
32	Cameroon	2011	Rice (Milled Equivalent)	59
32	Cameroon	2011	Roots, Other	67
32	Cameroon	2011	Sesameseed	1
32	Cameroon	2011	Sorghum	76
32	Cameroon	2011	Soyabeans	2
32	Cameroon	2011	Spices, Other	2
32	Cameroon	2011	Sugar Cane	57
32	Cameroon	2011	Sugar, Refined Equivalent	26
32	Cameroon	2011	Sweet Potatoes	33
32	Cameroon	2011	Tea	1
32	Cameroon	2011	Tomatoes	101
32	Cameroon	2011	Vegetables, Other	167
32	Cameroon	2011	Wheat	51
32	Cameroon	2011	Wine	2
32	Cameroon	2011	Yams	40
33	Canada	1992	Apples	64
33	Canada	1992	Bananas	36
33	Canada	1992	Barley	1
33	Canada	1992	Beans	7
33	Canada	1992	Beer	196
33	Canada	1992	Beverages, Alcoholic	3
33	Canada	1992	Beverages, Fermented	54
33	Canada	1992	Bovine Meat	93

FAO country code	Country	Reference year	Food item	Gross food supply (g *capita*$^{-1}$ d^{-1})
33	Canada	1992	Butter, Ghee	8
33	Canada	1992	Cheese	29
33	Canada	1992	Cocoa Beans	7
33	Canada	1992	Coconut Oil	1
33	Canada	1992	Coconuts - Incl Copra	4
33	Canada	1992	Coffee	11
33	Canada	1992	Cream	14
33	Canada	1992	Crustaceans	9
33	Canada	1992	Demersal Fish	18
33	Canada	1992	Eggs	29
33	Canada	1992	Fats, Animals, Raw	18
33	Canada	1992	Freshwater Fish	9
33	Canada	1992	Fruits, Other	54
33	Canada	1992	Grapefruit	11
33	Canada	1992	Grapes	27
33	Canada	1992	Groundnuts (Shelled Eq)	7
33	Canada	1992	Honey	2
33	Canada	1992	Lemons, Limes	6
33	Canada	1992	Maize	9
33	Canada	1992	Maize Germ Oil	2
33	Canada	1992	Marine Fish, Other	2
33	Canada	1992	Milk, Whole	207
33	Canada	1992	Molluscs, Other	13
33	Canada	1992	Mutton & Goat Meat	2
33	Canada	1992	Nuts	7
33	Canada	1992	Oats	9
33	Canada	1992	Offals, Edible	4
33	Canada	1992	Olive Oil	1
33	Canada	1992	Olives	1
33	Canada	1992	Onions	19
33	Canada	1992	Oranges, Mandarines	102
33	Canada	1992	Palm Oil	1
33	Canada	1992	Peas	3
33	Canada	1992	Pelagic Fish	14
33	Canada	1992	Pigmeat	82
33	Canada	1992	Pineapples	7
33	Canada	1992	Potatoes	226
33	Canada	1992	Poultry Meat	76
33	Canada	1992	Pulses, Other	9
33	Canada	1992	Rape and Mustard Oil	36
33	Canada	1992	Rice (Milled Equivalent)	15
33	Canada	1992	Roots, Other	1
33	Canada	1992	Rye	1
33	Canada	1992	Soyabean Oil	13
33	Canada	1992	Soyabeans	1
33	Canada	1992	Spices, Other	1
33	Canada	1992	Sugar, Refined Equivalent	100
33	Canada	1992	Sunflowerseed Oil	2
33	Canada	1992	Sweet Potatoes	1
33	Canada	1992	Sweeteners, Other	9
33	Canada	1992	Tea	1
33	Canada	1992	Tomatoes	75
33	Canada	1992	Vegetables, Other	218
33	Canada	1992	Wheat	208
33	Canada	1992	Whey	29
33	Canada	1992	Wine	17
33	Canada	1993	Apples	55
33	Canada	1993	Bananas	36
33	Canada	1993	Barley	1
33	Canada	1993	Beans	4
33	Canada	1993	Beer	201
33	Canada	1993	Beverages, Alcoholic	4
33	Canada	1993	Beverages, Fermented	42
33	Canada	1993	Bovine Meat	89
33	Canada	1993	Butter, Ghee	8
33	Canada	1993	Cheese	29
33	Canada	1993	Cocoa Beans	6
33	Canada	1993	Coconut Oil	1
33	Canada	1993	Coconuts - Incl Copra	4
33	Canada	1993	Coffee	12
33	Canada	1993	Cream	14
33	Canada	1993	Crustaceans	10
33	Canada	1993	Dates	1
33	Canada	1993	Demersal Fish	17
33	Canada	1993	Eggs	28
33	Canada	1993	Fats, Animals, Raw	16
33	Canada	1993	Freshwater Fish	7
33	Canada	1993	Fruits, Other	52
33	Canada	1993	Grapefruit	11
33	Canada	1993	Grapes	29
33	Canada	1993	Groundnuts (Shelled Eq)	7
33	Canada	1993	Honey	2
33	Canada	1993	Lemons, Limes	6
33	Canada	1993	Maize	9
33	Canada	1993	Maize Germ Oil	3
33	Canada	1993	Marine Fish, Other	1
33	Canada	1993	Milk, Whole	192
33	Canada	1993	Molluscs, Other	12
33	Canada	1993	Mutton & Goat Meat	2
33	Canada	1993	Nuts	8
33	Canada	1993	Oats	9
33	Canada	1993	Offals, Edible	3
33	Canada	1993	Olive Oil	1
33	Canada	1993	Olives	1
33	Canada	1993	Onions	25
33	Canada	1993	Oranges, Mandarines	112
33	Canada	1993	Palm Oil	1

FAO country code	Country	Reference year	Food item	Gross food supply (g *capita*$^{-1}$ d^{-1})
33	Canada	1993	Peas	3
33	Canada	1993	Pelagic Fish	17
33	Canada	1993	Pigmeat	80
33	Canada	1993	Pineapples	7
33	Canada	1993	Potatoes	222
33	Canada	1993	Poultry Meat	79
33	Canada	1993	Pulses, Other	9
33	Canada	1993	Rape and Mustard Oil	39
33	Canada	1993	Rice (Milled Equivalent)	16
33	Canada	1993	Roots, Other	1
33	Canada	1993	Rye	1
33	Canada	1993	Soyabean Oil	11
33	Canada	1993	Soyabeans	1
33	Canada	1993	Spices, Other	1
33	Canada	1993	Sugar, Refined Equivalent	105
33	Canada	1993	Sunflowerseed Oil	2
33	Canada	1993	Sweet Potatoes	1
33	Canada	1993	Sweeteners, Other	8
33	Canada	1993	Tea	1
33	Canada	1993	Tomatoes	88
33	Canada	1993	Vegetables, Other	215
33	Canada	1993	Wheat	207
33	Canada	1993	Whey	37
33	Canada	1993	Wine	17
33	Canada	1994	Apples	62
33	Canada	1994	Bananas	36
33	Canada	1994	Barley	1
33	Canada	1994	Beans	7
33	Canada	1994	Beer	202
33	Canada	1994	Beverages, Alcoholic	4
33	Canada	1994	Beverages, Fermented	48
33	Canada	1994	Bovine Meat	91
33	Canada	1994	Butter, Ghee	8
33	Canada	1994	Cheese	30
33	Canada	1994	Cocoa Beans	5
33	Canada	1994	Coconuts - Incl Copra	3
33	Canada	1994	Coffee	14
33	Canada	1994	Cream	15
33	Canada	1994	Crustaceans	8
33	Canada	1994	Dates	1
33	Canada	1994	Demersal Fish	20
33	Canada	1994	Eggs	28
33	Canada	1994	Fats, Animals, Raw	16
33	Canada	1994	Freshwater Fish	7
33	Canada	1994	Fruits, Other	53
33	Canada	1994	Grapefruit	12
33	Canada	1994	Grapes	27
33	Canada	1994	Groundnuts (Shelled Eq)	7
33	Canada	1994	Honey	3
33	Canada	1994	Lemons, Limes	6
33	Canada	1994	Maize	11
33	Canada	1994	Maize Germ Oil	2
33	Canada	1994	Marine Fish, Other	2
33	Canada	1994	Milk, Whole	185
33	Canada	1994	Molluscs, Other	12
33	Canada	1994	Mutton & Goat Meat	2
33	Canada	1994	Nuts	7
33	Canada	1994	Oats	8
33	Canada	1994	Offals, Edible	5
33	Canada	1994	Olive Oil	1
33	Canada	1994	Olives	1
33	Canada	1994	Onions	20
33	Canada	1994	Oranges, Mandarines	125
33	Canada	1994	Peas	4
33	Canada	1994	Pelagic Fish	15
33	Canada	1994	Pigmeat	82
33	Canada	1994	Pineapples	6
33	Canada	1994	Potatoes	226
33	Canada	1994	Poultry Meat	85
33	Canada	1994	Pulses, Other	10
33	Canada	1994	Rape and Mustard Oil	43
33	Canada	1994	Rice (Milled Equivalent)	15
33	Canada	1994	Roots, Other	1
33	Canada	1994	Rye	1
33	Canada	1994	Soyabean Oil	8
33	Canada	1994	Soyabeans	1
33	Canada	1994	Spices, Other	1
33	Canada	1994	Sugar, Refined Equivalent	104
33	Canada	1994	Sunflowerseed Oil	2
33	Canada	1994	Sweet Potatoes	1
33	Canada	1994	Sweeteners, Other	12
33	Canada	1994	Tea	1
33	Canada	1994	Tomatoes	89
33	Canada	1994	Vegetables, Other	217
33	Canada	1994	Wheat	237
33	Canada	1994	Whey	36
33	Canada	1994	Wine	18
33	Canada	1995	Apples	58
33	Canada	1995	Bananas	37
33	Canada	1995	Barley	1
33	Canada	1995	Beans	3
33	Canada	1995	Beer	205
33	Canada	1995	Beverages, Alcoholic	5
33	Canada	1995	Beverages, Fermented	45
33	Canada	1995	Bovine Meat	91
33	Canada	1995	Butter, Ghee	8
33	Canada	1995	Cheese	30

FAO country code	Country	Reference year	Food item	Gross food supply (g *capita*$^{-1}$ d^{-1})
33	Canada	1995	Cocoa Beans	6
33	Canada	1995	Coconut Oil	1
33	Canada	1995	Coconuts - Incl Copra	3
33	Canada	1995	Coffee	12
33	Canada	1995	Cream	15
33	Canada	1995	Crustaceans	11
33	Canada	1995	Demersal Fish	12
33	Canada	1995	Eggs	28
33	Canada	1995	Fats, Animals, Raw	16
33	Canada	1995	Freshwater Fish	10
33	Canada	1995	Fruits, Other	55
33	Canada	1995	Grapefruit	12
33	Canada	1995	Grapes	28
33	Canada	1995	Groundnuts (Shelled Eq)	8
33	Canada	1995	Honey	2
33	Canada	1995	Lemons, Limes	6
33	Canada	1995	Maize	11
33	Canada	1995	Maize Germ Oil	3
33	Canada	1995	Marine Fish, Other	1
33	Canada	1995	Milk, Whole	182
33	Canada	1995	Molluscs, Other	12
33	Canada	1995	Mutton & Goat Meat	2
33	Canada	1995	Nuts	6
33	Canada	1995	Oats	5
33	Canada	1995	Offals, Edible	4
33	Canada	1995	Olive Oil	1
33	Canada	1995	Olives	1
33	Canada	1995	Onions	23
33	Canada	1995	Oranges, Mandarines	127
33	Canada	1995	Palm Oil	1
33	Canada	1995	Peas	3
33	Canada	1995	Pelagic Fish	16
33	Canada	1995	Pigmeat	80
33	Canada	1995	Pineapples	6
33	Canada	1995	Potatoes	224
33	Canada	1995	Poultry Meat	83
33	Canada	1995	Pulses, Other	11
33	Canada	1995	Rape and Mustard Oil	46
33	Canada	1995	Rice (Milled Equivalent)	17
33	Canada	1995	Roots, Other	1
33	Canada	1995	Rye	1
33	Canada	1995	Soyabean Oil	9
33	Canada	1995	Soyabeans	1
33	Canada	1995	Spices, Other	1
33	Canada	1995	Sugar, Refined Equivalent	101
33	Canada	1995	Sunflowerseed Oil	1
33	Canada	1995	Sweet Potatoes	1
33	Canada	1995	Sweeteners, Other	18
33	Canada	1995	Tea	1
33	Canada	1995	Tomatoes	86
33	Canada	1995	Vegetables, Other	217
33	Canada	1995	Wheat	230
33	Canada	1995	Whey	35
33	Canada	1995	Wine	17
33	Canada	1996	Apples	47
33	Canada	1996	Bananas	37
33	Canada	1996	Barley	1
33	Canada	1996	Beans	4
33	Canada	1996	Beer	198
33	Canada	1996	Beverages, Alcoholic	4
33	Canada	1996	Beverages, Fermented	44
33	Canada	1996	Bovine Meat	91
33	Canada	1996	Butter, Ghee	8
33	Canada	1996	Cheese	30
33	Canada	1996	Cocoa Beans	5
33	Canada	1996	Coconut Oil	1
33	Canada	1996	Coconuts - Incl Copra	2
33	Canada	1996	Coffee	13
33	Canada	1996	Cottonseed Oil	1
33	Canada	1996	Cream	15
33	Canada	1996	Crustaceans	13
33	Canada	1996	Demersal Fish	17
33	Canada	1996	Eggs	29
33	Canada	1996	Fats, Animals, Raw	13
33	Canada	1996	Freshwater Fish	6
33	Canada	1996	Fruits, Other	53
33	Canada	1996	Grapefruit	12
33	Canada	1996	Grapes	27
33	Canada	1996	Groundnuts (Shelled Eq)	7
33	Canada	1996	Honey	3
33	Canada	1996	Lemons, Limes	6
33	Canada	1996	Maize	19
33	Canada	1996	Maize Germ Oil	3
33	Canada	1996	Marine Fish, Other	1
33	Canada	1996	Milk, Whole	171
33	Canada	1996	Molluscs, Other	12
33	Canada	1996	Mutton & Goat Meat	2
33	Canada	1996	Nuts	6
33	Canada	1996	Oats	8
33	Canada	1996	Offals, Edible	4
33	Canada	1996	Olive Oil	1
33	Canada	1996	Olives	1
33	Canada	1996	Onions	23
33	Canada	1996	Oranges, Mandarines	137
33	Canada	1996	Palm Oil	1
33	Canada	1996	Peas	3
33	Canada	1996	Pelagic Fish	15

FAO country code	Country	Reference year	Food item	Gross food supply (g *capita*$^{-1}$ d^{-1})
33	Canada	1996	Pigmeat	74
33	Canada	1996	Pineapples	6
33	Canada	1996	Potatoes	235
33	Canada	1996	Poultry Meat	84
33	Canada	1996	Pulses, Other	9
33	Canada	1996	Rape and Mustard Oil	43
33	Canada	1996	Rice (Milled Equivalent)	18
33	Canada	1996	Roots, Other	1
33	Canada	1996	Rye	1
33	Canada	1996	Soyabean Oil	15
33	Canada	1996	Soyabeans	1
33	Canada	1996	Spices, Other	1
33	Canada	1996	Sugar, Refined Equivalent	97
33	Canada	1996	Sunflowerseed Oil	1
33	Canada	1996	Sweet Potatoes	1
33	Canada	1996	Sweeteners, Other	28
33	Canada	1996	Tea	1
33	Canada	1996	Tomatoes	88
33	Canada	1996	Vegetables, Other	225
33	Canada	1996	Wheat	244
33	Canada	1996	Whey	41
33	Canada	1996	Wine	19
33	Canada	1997	Apples	46
33	Canada	1997	Bananas	38
33	Canada	1997	Barley	1
33	Canada	1997	Beans	5
33	Canada	1997	Beer	194
33	Canada	1997	Beverages, Alcoholic	4
33	Canada	1997	Beverages, Fermented	45
33	Canada	1997	Bovine Meat	91
33	Canada	1997	Butter, Ghee	8
33	Canada	1997	Cassava	1
33	Canada	1997	Cephalopods	1
33	Canada	1997	Cheese	32
33	Canada	1997	Cocoa Beans	5
33	Canada	1997	Coconut Oil	1
33	Canada	1997	Coconuts - Incl Copra	3
33	Canada	1997	Coffee	12
33	Canada	1997	Cottonseed Oil	1
33	Canada	1997	Cream	15
33	Canada	1997	Crustaceans	12
33	Canada	1997	Demersal Fish	17
33	Canada	1997	Eggs	30
33	Canada	1997	Fats, Animals, Raw	14
33	Canada	1997	Freshwater Fish	7
33	Canada	1997	Fruits, Other	54
33	Canada	1997	Grapefruit	12
33	Canada	1997	Grapes	30
33	Canada	1997	Groundnuts (Shelled Eq)	7
33	Canada	1997	Honey	2
33	Canada	1997	Lemons, Limes	6
33	Canada	1997	Maize	31
33	Canada	1997	Maize Germ Oil	3
33	Canada	1997	Marine Fish, Other	1
33	Canada	1997	Milk, Whole	143
33	Canada	1997	Molluscs, Other	12
33	Canada	1997	Mutton & Goat Meat	2
33	Canada	1997	Nuts	6
33	Canada	1997	Oats	7
33	Canada	1997	Offals, Edible	4
33	Canada	1997	Olive Oil	2
33	Canada	1997	Olives	1
33	Canada	1997	Onions	23
33	Canada	1997	Oranges, Mandarines	146
33	Canada	1997	Peas	5
33	Canada	1997	Pelagic Fish	15
33	Canada	1997	Pigmeat	74
33	Canada	1997	Pineapples	7
33	Canada	1997	Potatoes	238
33	Canada	1997	Poultry Meat	87
33	Canada	1997	Pulses, Other	8
33	Canada	1997	Rape and Mustard Oil	48
33	Canada	1997	Rice (Milled Equivalent)	21
33	Canada	1997	Roots, Other	1
33	Canada	1997	Rye	1
33	Canada	1997	Soyabean Oil	15
33	Canada	1997	Soyabeans	2
33	Canada	1997	Spices, Other	1
33	Canada	1997	Sugar, Refined Equivalent	98
33	Canada	1997	Sunflowerseed Oil	1
33	Canada	1997	Sweet Potatoes	1
33	Canada	1997	Sweeteners, Other	32
33	Canada	1997	Tea	2
33	Canada	1997	Tomatoes	80
33	Canada	1997	Vegetables, Other	230
33	Canada	1997	Wheat	244
33	Canada	1997	Whey	30
33	Canada	1997	Wine	20
33	Canada	1998	Apples	51
33	Canada	1998	Bananas	37
33	Canada	1998	Barley	1
33	Canada	1998	Beans	3
33	Canada	1998	Beer	200
33	Canada	1998	Beverages, Alcoholic	5
33	Canada	1998	Beverages, Fermented	40
33	Canada	1998	Bovine Meat	93
33	Canada	1998	Butter, Ghee	8

FAO country code	Country	Reference year	Food item	Gross food supply (g *capita*$^{-1}$ d^{-1})
33	Canada	1998	Cereals, Other	1
33	Canada	1998	Cheese	32
33	Canada	1998	Cocoa Beans	6
33	Canada	1998	Coconut Oil	1
33	Canada	1998	Coconuts - Incl Copra	3
33	Canada	1998	Coffee	13
33	Canada	1998	Cottonseed Oil	1
33	Canada	1998	Cream	17
33	Canada	1998	Crustaceans	14
33	Canada	1998	Dates	1
33	Canada	1998	Demersal Fish	18
33	Canada	1998	Eggs	31
33	Canada	1998	Fats, Animals, Raw	17
33	Canada	1998	Freshwater Fish	7
33	Canada	1998	Fruits, Other	64
33	Canada	1998	Grapefruit	9
33	Canada	1998	Grapes	26
33	Canada	1998	Groundnuts (Shelled Eq)	7
33	Canada	1998	Honey	3
33	Canada	1998	Lemons, Limes	7
33	Canada	1998	Maize	30
33	Canada	1998	Maize Germ Oil	3
33	Canada	1998	Marine Fish, Other	1
33	Canada	1998	Milk, Whole	157
33	Canada	1998	Molluscs, Other	12
33	Canada	1998	Mutton & Goat Meat	2
33	Canada	1998	Nuts	7
33	Canada	1998	Oats	7
33	Canada	1998	Offals, Edible	4
33	Canada	1998	Olive Oil	2
33	Canada	1998	Olives	2
33	Canada	1998	Onions	20
33	Canada	1998	Oranges, Mandarines	141
33	Canada	1998	Peas	3
33	Canada	1998	Pelagic Fish	14
33	Canada	1998	Pigmeat	82
33	Canada	1998	Pineapples	6
33	Canada	1998	Potatoes	235
33	Canada	1998	Poultry Meat	91
33	Canada	1998	Pulses, Other	9
33	Canada	1998	Rape and Mustard Oil	50
33	Canada	1998	Rice (Milled Equivalent)	20
33	Canada	1998	Roots, Other	1
33	Canada	1998	Rye	1
33	Canada	1998	Soyabean Oil	14
33	Canada	1998	Soyabeans	2
33	Canada	1998	Spices, Other	1
33	Canada	1998	Sugar, Refined Equivalent	90
33	Canada	1998	Sunflowerseed Oil	1
33	Canada	1998	Sweet Potatoes	2
33	Canada	1998	Sweeteners, Other	42
33	Canada	1998	Tea	2
33	Canada	1998	Tomatoes	83
33	Canada	1998	Vegetables, Other	232
33	Canada	1998	Wheat	254
33	Canada	1998	Whey	24
33	Canada	1998	Wine	22
33	Canada	1999	Apples	57
33	Canada	1999	Bananas	37
33	Canada	1999	Barley	1
33	Canada	1999	Beans	4
33	Canada	1999	Beer	203
33	Canada	1999	Beverages, Alcoholic	5
33	Canada	1999	Beverages, Fermented	47
33	Canada	1999	Bovine Meat	95
33	Canada	1999	Butter, Ghee	7
33	Canada	1999	Cereals, Other	3
33	Canada	1999	Cheese	32
33	Canada	1999	Cocoa Beans	5
33	Canada	1999	Coconut Oil	1
33	Canada	1999	Coconuts - Incl Copra	3
33	Canada	1999	Coffee	13
33	Canada	1999	Cottonseed Oil	2
33	Canada	1999	Cream	17
33	Canada	1999	Crustaceans	15
33	Canada	1999	Demersal Fish	19
33	Canada	1999	Eggs	30
33	Canada	1999	Fats, Animals, Raw	19
33	Canada	1999	Freshwater Fish	8
33	Canada	1999	Fruits, Other	69
33	Canada	1999	Grapefruit	11
33	Canada	1999	Grapes	26
33	Canada	1999	Groundnuts (Shelled Eq)	7
33	Canada	1999	Honey	2
33	Canada	1999	Lemons, Limes	7
33	Canada	1999	Maize	48
33	Canada	1999	Maize Germ Oil	3
33	Canada	1999	Marine Fish, Other	1
33	Canada	1999	Milk, Whole	152
33	Canada	1999	Molluscs, Other	12
33	Canada	1999	Mutton & Goat Meat	2
33	Canada	1999	Nuts	7
33	Canada	1999	Oats	7
33	Canada	1999	Offals, Edible	5
33	Canada	1999	Olive Oil	2
33	Canada	1999	Olives	2
33	Canada	1999	Onions	24

FAO country code	Country	Reference year	Food item	Gross food supply (g $capita^{-1}$ d^{-1})
33	Canada	1999	Oranges, Mandarines	138
33	Canada	1999	Peas	3
33	Canada	1999	Pelagic Fish	14
33	Canada	1999	Pigmeat	87
33	Canada	1999	Pineapples	7
33	Canada	1999	Potatoes	224
33	Canada	1999	Poultry Meat	95
33	Canada	1999	Pulses, Other	14
33	Canada	1999	Rape and Mustard Oil	43
33	Canada	1999	Rice (Milled Equivalent)	21
33	Canada	1999	Roots, Other	1
33	Canada	1999	Rye	1
33	Canada	1999	Soyabean Oil	16
33	Canada	1999	Soyabeans	2
33	Canada	1999	Spices, Other	1
33	Canada	1999	Sugar, Refined Equivalent	90
33	Canada	1999	Sunflowerseed Oil	2
33	Canada	1999	Sweet Potatoes	2
33	Canada	1999	Sweeteners, Other	34
33	Canada	1999	Tea	2
33	Canada	1999	Tomatoes	84
33	Canada	1999	Vegetables, Other	232
33	Canada	1999	Wheat	247
33	Canada	1999	Whey	16
33	Canada	1999	Wine	25
33	Canada	2000	Apples	46
33	Canada	2000	Bananas	35
33	Canada	2000	Barley	1
33	Canada	2000	Beans	3
33	Canada	2000	Beer	199
33	Canada	2000	Beverages, Alcoholic	5
33	Canada	2000	Beverages, Fermented	57
33	Canada	2000	Bovine Meat	93
33	Canada	2000	Butter, Ghee	8
33	Canada	2000	Cereals, Other	3
33	Canada	2000	Cheese	33
33	Canada	2000	Cocoa Beans	5
33	Canada	2000	Coconut Oil	1
33	Canada	2000	Coconuts - Incl Copra	3
33	Canada	2000	Coffee	13
33	Canada	2000	Cottonseed Oil	2
33	Canada	2000	Cream	19
33	Canada	2000	Crustaceans	14
33	Canada	2000	Demersal Fish	14
33	Canada	2000	Eggs	30
33	Canada	2000	Fats, Animals, Raw	19
33	Canada	2000	Freshwater Fish	10
33	Canada	2000	Fruits, Other	67
33	Canada	2000	Grapefruit	11
33	Canada	2000	Grapes	28
33	Canada	2000	Groundnuts (Shelled Eq)	7
33	Canada	2000	Honey	2
33	Canada	2000	Lemons, Limes	6
33	Canada	2000	Maize	52
33	Canada	2000	Maize Germ Oil	4
33	Canada	2000	Marine Fish, Other	1
33	Canada	2000	Milk, Whole	162
33	Canada	2000	Molluscs, Other	11
33	Canada	2000	Mutton & Goat Meat	2
33	Canada	2000	Nuts	8
33	Canada	2000	Oats	2
33	Canada	2000	Offals, Edible	3
33	Canada	2000	Olive Oil	2
33	Canada	2000	Olives	2
33	Canada	2000	Onions	24
33	Canada	2000	Oranges, Mandarines	144
33	Canada	2000	Peas	3
33	Canada	2000	Pelagic Fish	15
33	Canada	2000	Pigmeat	83
33	Canada	2000	Pineapples	8
33	Canada	2000	Potatoes	219
33	Canada	2000	Poultry Meat	98
33	Canada	2000	Pulses, Other	15
33	Canada	2000	Rape and Mustard Oil	43
33	Canada	2000	Rice (Milled Equivalent)	20
33	Canada	2000	Roots, Other	1
33	Canada	2000	Rye	1
33	Canada	2000	Soyabean Oil	15
33	Canada	2000	Soyabeans	2
33	Canada	2000	Spices, Other	1
33	Canada	2000	Sugar, Refined Equivalent	93
33	Canada	2000	Sunflowerseed Oil	2
33	Canada	2000	Sweet Potatoes	2
33	Canada	2000	Sweeteners, Other	36
33	Canada	2000	Tea	2
33	Canada	2000	Tomatoes	84
33	Canada	2000	Vegetables, Other	222
33	Canada	2000	Wheat	245
33	Canada	2000	Whey	34
33	Canada	2000	Wine	25
33	Canada	2001	Apples	40
33	Canada	2001	Bananas	35
33	Canada	2001	Barley	2
33	Canada	2001	Beans	3
33	Canada	2001	Beer	205
33	Canada	2001	Beverages, Alcoholic	6
33	Canada	2001	Beverages, Fermented	49

FAO country code	Country	Reference year	Food item	Gross food supply (g $capita^{-1}$ d^{-1})
33	Canada	2001	Bovine Meat	90
33	Canada	2001	Butter, Ghee	8
33	Canada	2001	Cassava	1
33	Canada	2001	Cereals, Other	4
33	Canada	2001	Cheese	32
33	Canada	2001	Cocoa Beans	4
33	Canada	2001	Coconut Oil	1
33	Canada	2001	Coconuts - Incl Copra	3
33	Canada	2001	Coffee	14
33	Canada	2001	Cottonseed Oil	1
33	Canada	2001	Cream	19
33	Canada	2001	Crustaceans	14
33	Canada	2001	Demersal Fish	20
33	Canada	2001	Eggs	32
33	Canada	2001	Fats, Animals, Raw	19
33	Canada	2001	Freshwater Fish	8
33	Canada	2001	Fruits, Other	71
33	Canada	2001	Grapefruit	10
33	Canada	2001	Grapes	27
33	Canada	2001	Groundnuts (Shelled Eq)	6
33	Canada	2001	Honey	2
33	Canada	2001	Lemons, Limes	7
33	Canada	2001	Maize	50
33	Canada	2001	Maize Germ Oil	4
33	Canada	2001	Marine Fish, Other	1
33	Canada	2001	Milk, Whole	145
33	Canada	2001	Molluscs, Other	12
33	Canada	2001	Mutton & Goat Meat	3
33	Canada	2001	Nuts	9
33	Canada	2001	Oats	2
33	Canada	2001	Offals, Edible	3
33	Canada	2001	Olive Oil	2
33	Canada	2001	Olives	2
33	Canada	2001	Onions	24
33	Canada	2001	Oranges, Mandarines	141
33	Canada	2001	Peas	4
33	Canada	2001	Pelagic Fish	12
33	Canada	2001	Pigmeat	84
33	Canada	2001	Pineapples	9
33	Canada	2001	Potatoes	218
33	Canada	2001	Poultry Meat	101
33	Canada	2001	Pulses, Other	14
33	Canada	2001	Rape and Mustard Oil	41
33	Canada	2001	Rice (Milled Equivalent)	19
33	Canada	2001	Roots, Other	1
33	Canada	2001	Rye	1
33	Canada	2001	Sesameseed	1
33	Canada	2001	Soyabean Oil	16
33	Canada	2001	Soyabeans	2
33	Canada	2001	Spices, Other	1
33	Canada	2001	Sugar, Refined Equivalent	99
33	Canada	2001	Sunflowerseed Oil	2
33	Canada	2001	Sweet Potatoes	2
33	Canada	2001	Sweeteners, Other	35
33	Canada	2001	Tea	2
33	Canada	2001	Tomatoes	81
33	Canada	2001	Vegetables, Other	226
33	Canada	2001	Wheat	242
33	Canada	2001	Whey	34
33	Canada	2001	Wine	25
33	Canada	2002	Apples	39
33	Canada	2002	Bananas	36
33	Canada	2002	Barley	1
33	Canada	2002	Beans	4
33	Canada	2002	Beer	207
33	Canada	2002	Beverages, Alcoholic	6
33	Canada	2002	Beverages, Fermented	51
33	Canada	2002	Bovine Meat	90
33	Canada	2002	Butter, Ghee	8
33	Canada	2002	Cereals, Other	5
33	Canada	2002	Cheese	32
33	Canada	2002	Citrus, Other	1
33	Canada	2002	Cocoa Beans	3
33	Canada	2002	Coconut Oil	1
33	Canada	2002	Coconuts - Incl Copra	3
33	Canada	2002	Coffee	12
33	Canada	2002	Cottonseed Oil	1
33	Canada	2002	Cream	20
33	Canada	2002	Crustaceans	12
33	Canada	2002	Demersal Fish	18
33	Canada	2002	Eggs	31
33	Canada	2002	Fats, Animals, Raw	20
33	Canada	2002	Freshwater Fish	8
33	Canada	2002	Fruits, Other	75
33	Canada	2002	Grapefruit	11
33	Canada	2002	Grapes	28
33	Canada	2002	Groundnuts (Shelled Eq)	7
33	Canada	2002	Honey	2
33	Canada	2002	Lemons, Limes	6
33	Canada	2002	Maize	55
33	Canada	2002	Maize Germ Oil	3
33	Canada	2002	Marine Fish, Other	1
33	Canada	2002	Milk, Whole	142
33	Canada	2002	Molluscs, Other	12
33	Canada	2002	Mutton & Goat Meat	3
33	Canada	2002	Nuts	9
33	Canada	2002	Oats	2

FAO countrycode	Country	Reference year	Food item	Gross food supply (g $capita^{-1}$ d^{-1})
33	Canada	2002	Offals, Edible	4
33	Canada	2002	Olive Oil	2
33	Canada	2002	Olives	2
33	Canada	2002	Onions	22
33	Canada	2002	Oranges, Mandarines	135
33	Canada	2002	Peas	3
33	Canada	2002	Pelagic Fish	14
33	Canada	2002	Pigmeat	83
33	Canada	2002	Pineapples	9
33	Canada	2002	Potatoes	220
33	Canada	2002	Poultry Meat	101
33	Canada	2002	Pulses, Other	14
33	Canada	2002	Rape and Mustard Oil	36
33	Canada	2002	Rice (Milled Equivalent)	18
33	Canada	2002	Roots, Other	1
33	Canada	2002	Rye	1
33	Canada	2002	Sesameseed	1
33	Canada	2002	Soyabean Oil	17
33	Canada	2002	Soyabeans	2
33	Canada	2002	Spices, Other	1
33	Canada	2002	Sugar, Refined Equivalent	107
33	Canada	2002	Sunflowerseed Oil	2
33	Canada	2002	Sweet Potatoes	2
33	Canada	2002	Sweeteners, Other	40
33	Canada	2002	Tea	2
33	Canada	2002	Tomatoes	89
33	Canada	2002	Vegetables, Other	233
33	Canada	2002	Wheat	243
33	Canada	2002	Whey	34
33	Canada	2002	Wine	26
33	Canada	2003	Apples	30
33	Canada	2003	Bananas	36
33	Canada	2003	Barley	1
33	Canada	2003	Beans	3
33	Canada	2003	Beer	174
33	Canada	2003	Beverages, Alcoholic	5
33	Canada	2003	Beverages, Fermented	47
33	Canada	2003	Bovine Meat	95
33	Canada	2003	Butter, Ghee	8
33	Canada	2003	Cereals, Other	4
33	Canada	2003	Cheese	32
33	Canada	2003	Citrus, Other	1
33	Canada	2003	Cocoa Beans	3
33	Canada	2003	Coconut Oil	1
33	Canada	2003	Coconuts - Incl Copra	3
33	Canada	2003	Coffee	11
33	Canada	2003	Cottonseed Oil	1
33	Canada	2003	Cream	22
33	Canada	2003	Crustaceans	13
33	Canada	2003	Demersal Fish	18
33	Canada	2003	Eggs	31
33	Canada	2003	Fats, Animals, Raw	21
33	Canada	2003	Freshwater Fish	11
33	Canada	2003	Fruits, Other	73
33	Canada	2003	Grapefruit	10
33	Canada	2003	Grapes	29
33	Canada	2003	Groundnuts (Shelled Eq)	6
33	Canada	2003	Honey	2
33	Canada	2003	Lemons, Limes	7
33	Canada	2003	Maize	57
33	Canada	2003	Maize Germ Oil	3
33	Canada	2003	Marine Fish, Other	1
33	Canada	2003	Milk, Whole	96
33	Canada	2003	Molluscs, Other	13
33	Canada	2003	Mutton & Goat Meat	3
33	Canada	2003	Nuts	10
33	Canada	2003	Oats	1
33	Canada	2003	Offals, Edible	5
33	Canada	2003	Olive Oil	2
33	Canada	2003	Olives	2
33	Canada	2003	Onions	23
33	Canada	2003	Oranges, Mandarines	132
33	Canada	2003	Peas	3
33	Canada	2003	Pelagic Fish	11
33	Canada	2003	Pigmeat	75
33	Canada	2003	Pineapples	9
33	Canada	2003	Potatoes	229
33	Canada	2003	Poultry Meat	99
33	Canada	2003	Pulses, Other	12
33	Canada	2003	Rape and Mustard Oil	43
33	Canada	2003	Rice (Milled Equivalent)	16
33	Canada	2003	Roots, Other	1
33	Canada	2003	Rye	2
33	Canada	2003	Sesameseed	1
33	Canada	2003	Soyabean Oil	18
33	Canada	2003	Soyabeans	2
33	Canada	2003	Spices, Other	1
33	Canada	2003	Sugar, Refined Equivalent	107
33	Canada	2003	Sunflowerseed Oil	2
33	Canada	2003	Sweet Potatoes	2
33	Canada	2003	Sweeteners, Other	40
33	Canada	2003	Tea	2
33	Canada	2003	Tomatoes	77
33	Canada	2003	Vegetables, Other	235
33	Canada	2003	Wheat	237
33	Canada	2003	Whey	29
33	Canada	2003	Wine	27

FAO country code	Country	Reference year	Food item	Gross food supply (g *capita*$^{-1}$ d^{-1})
33	Canada	2004	Apples	38
33	Canada	2004	Bananas	37
33	Canada	2004	Barley	1
33	Canada	2004	Beans	2
33	Canada	2004	Beer	199
33	Canada	2004	Beverages, Alcoholic	5
33	Canada	2004	Beverages, Fermented	48
33	Canada	2004	Bovine Meat	91
33	Canada	2004	Butter, Ghee	8
33	Canada	2004	Cassava	1
33	Canada	2004	Cereals, Other	2
33	Canada	2004	Cheese	33
33	Canada	2004	Citrus, Other	1
33	Canada	2004	Cocoa Beans	5
33	Canada	2004	Coconut Oil	1
33	Canada	2004	Coconuts - Incl Copra	3
33	Canada	2004	Coffee	15
33	Canada	2004	Cottonseed Oil	1
33	Canada	2004	Cream	23
33	Canada	2004	Crustaceans	12
33	Canada	2004	Demersal Fish	20
33	Canada	2004	Eggs	31
33	Canada	2004	Fats, Animals, Raw	22
33	Canada	2004	Freshwater Fish	8
33	Canada	2004	Fruits, Other	70
33	Canada	2004	Grapefruit	10
33	Canada	2004	Grapes	28
33	Canada	2004	Groundnuts (Shelled Eq)	7
33	Canada	2004	Honey	3
33	Canada	2004	Lemons, Limes	6
33	Canada	2004	Maize	54
33	Canada	2004	Maize Germ Oil	4
33	Canada	2004	Marine Fish, Other	1
33	Canada	2004	Milk, Whole	104
33	Canada	2004	Molluscs, Other	11
33	Canada	2004	Mutton & Goat Meat	3
33	Canada	2004	Nuts	11
33	Canada	2004	Oats	1
33	Canada	2004	Offals, Edible	5
33	Canada	2004	Olive Oil	2
33	Canada	2004	Olives	2
33	Canada	2004	Onions	23
33	Canada	2004	Oranges, Mandarines	130
33	Canada	2004	Peas	4
33	Canada	2004	Pelagic Fish	12
33	Canada	2004	Pigmeat	79
33	Canada	2004	Pineapples	10
33	Canada	2004	Potatoes	212
33	Canada	2004	Poultry Meat	100
33	Canada	2004	Pulses, Other	13
33	Canada	2004	Rape and Mustard Oil	39
33	Canada	2004	Rice (Milled Equivalent)	23
33	Canada	2004	Roots, Other	1
33	Canada	2004	Rye	2
33	Canada	2004	Soyabean Oil	17
33	Canada	2004	Soyabeans	2
33	Canada	2004	Spices, Other	2
33	Canada	2004	Sugar, Refined Equivalent	104
33	Canada	2004	Sunflowerseed Oil	2
33	Canada	2004	Sweet Potatoes	3
33	Canada	2004	Sweeteners, Other	38
33	Canada	2004	Tea	1
33	Canada	2004	Tomatoes	89
33	Canada	2004	Vegetables, Other	223
33	Canada	2004	Wheat	241
33	Canada	2004	Whey	23
33	Canada	2004	Wine	27
33	Canada	2005	Apples	50
33	Canada	2005	Bananas	37
33	Canada	2005	Barley	1
33	Canada	2005	Beans	3
33	Canada	2005	Beer	207
33	Canada	2005	Beverages, Alcoholic	3
33	Canada	2005	Beverages, Fermented	52
33	Canada	2005	Bovine Meat	89
33	Canada	2005	Butter, Ghee	8
33	Canada	2005	Cassava	1
33	Canada	2005	Cereals, Other	2
33	Canada	2005	Cheese	33
33	Canada	2005	Citrus, Other	1
33	Canada	2005	Cocoa Beans	2
33	Canada	2005	Coconut Oil	1
33	Canada	2005	Coconuts - Incl Copra	3
33	Canada	2005	Coffee	15
33	Canada	2005	Cottonseed Oil	1
33	Canada	2005	Cream	23
33	Canada	2005	Crustaceans	12
33	Canada	2005	Dates	1
33	Canada	2005	Demersal Fish	20
33	Canada	2005	Eggs	31
33	Canada	2005	Fats, Animals, Raw	21
33	Canada	2005	Freshwater Fish	8
33	Canada	2005	Fruits, Other	75
33	Canada	2005	Grapefruit	9
33	Canada	2005	Grapes	28
33	Canada	2005	Groundnuts (Shelled Eq)	7
33	Canada	2005	Honey	3

FAO country code	Country	Reference year	Food item	Gross food supply (g $capita^{-1}$ d^{-1})
33	Canada	2005	Lemons, Limes	7
33	Canada	2005	Maize	54
33	Canada	2005	Maize Germ Oil	4
33	Canada	2005	Marine Fish, Other	2
33	Canada	2005	Milk, Whole	95
33	Canada	2005	Molluscs, Other	10
33	Canada	2005	Mutton & Goat Meat	3
33	Canada	2005	Nuts	10
33	Canada	2005	Oats	1
33	Canada	2005	Offals, Edible	4
33	Canada	2005	Olive Oil	2
33	Canada	2005	Olives	2
33	Canada	2005	Onions	21
33	Canada	2005	Oranges, Mandarines	135
33	Canada	2005	Peas	4
33	Canada	2005	Pelagic Fish	12
33	Canada	2005	Pigmeat	69
33	Canada	2005	Pineapples	11
33	Canada	2005	Potatoes	215
33	Canada	2005	Poultry Meat	100
33	Canada	2005	Pulses, Other	13
33	Canada	2005	Rape and Mustard Oil	40
33	Canada	2005	Rice (Milled Equivalent)	23
33	Canada	2005	Roots, Other	1
33	Canada	2005	Rye	1
33	Canada	2005	Soyabean Oil	17
33	Canada	2005	Soyabeans	2
33	Canada	2005	Spices, Other	1
33	Canada	2005	Sugar, Refined Equivalent	104
33	Canada	2005	Sunflowerseed Oil	2
33	Canada	2005	Sweet Potatoes	2
33	Canada	2005	Sweeteners, Other	36
33	Canada	2005	Tea	1
33	Canada	2005	Tomatoes	89
33	Canada	2005	Vegetables, Other	216
33	Canada	2005	Wheat	245
33	Canada	2005	Whey	17
33	Canada	2005	Wine	26
33	Canada	2006	Apples	50
33	Canada	2006	Bananas	38
33	Canada	2006	Beans	3
33	Canada	2006	Beer	189
33	Canada	2006	Beverages, Alcoholic	4
33	Canada	2006	Beverages, Fermented	40
33	Canada	2006	Bovine Meat	88
33	Canada	2006	Butter, Ghee	6
33	Canada	2006	Cassava	1
33	Canada	2006	Cephalopods	1
33	Canada	2006	Cereals, Other	1
33	Canada	2006	Cheese	34
33	Canada	2006	Citrus, Other	1
33	Canada	2006	Cocoa Beans	4
33	Canada	2006	Coconut Oil	1
33	Canada	2006	Coconuts - Incl Copra	3
33	Canada	2006	Coffee	17
33	Canada	2006	Cottonseed Oil	1
33	Canada	2006	Cream	24
33	Canada	2006	Crustaceans	11
33	Canada	2006	Dates	1
33	Canada	2006	Demersal Fish	19
33	Canada	2006	Eggs	30
33	Canada	2006	Fats, Animals, Raw	19
33	Canada	2006	Freshwater Fish	9
33	Canada	2006	Fruits, Other	81
33	Canada	2006	Grapefruit	8
33	Canada	2006	Grapes	29
33	Canada	2006	Groundnuts (Shelled Eq)	7
33	Canada	2006	Honey	3
33	Canada	2006	Lemons, Limes	7
33	Canada	2006	Maize	53
33	Canada	2006	Maize Germ Oil	4
33	Canada	2006	Marine Fish, Other	1
33	Canada	2006	Milk, Whole	112
33	Canada	2006	Molluscs, Other	10
33	Canada	2006	Mutton & Goat Meat	3
33	Canada	2006	Nuts	10
33	Canada	2006	Oats	1
33	Canada	2006	Offals, Edible	4
33	Canada	2006	Olive Oil	3
33	Canada	2006	Olives	2
33	Canada	2006	Onions	23
33	Canada	2006	Oranges, Mandarines	133
33	Canada	2006	Peas	1
33	Canada	2006	Pelagic Fish	13
33	Canada	2006	Pigmeat	69
33	Canada	2006	Pimento	1
33	Canada	2006	Pineapples	13
33	Canada	2006	Potatoes	210
33	Canada	2006	Poultry Meat	101
33	Canada	2006	Pulses, Other	13
33	Canada	2006	Rape and Mustard Oil	41
33	Canada	2006	Rice (Milled Equivalent)	25
33	Canada	2006	Roots, Other	1
33	Canada	2006	Rye	1
33	Canada	2006	Sesameseed	1
33	Canada	2006	Soyabean Oil	15
33	Canada	2006	Soyabeans	2

FAO country code	Country	Reference year	Food item	Gross food supply (g *capita*$^{-1}$ d^{-1})
33	Canada	2006	Spices, Other	1
33	Canada	2006	Sugar, Refined Equivalent	98
33	Canada	2006	Sunflowerseed Oil	2
33	Canada	2006	Sweet Potatoes	2
33	Canada	2006	Sweeteners, Other	36
33	Canada	2006	Tea	1
33	Canada	2006	Tomatoes	86
33	Canada	2006	Vegetables, Other	222
33	Canada	2006	Wheat	244
33	Canada	2006	Whey	20
33	Canada	2006	Wine	30
33	Canada	2007	Apples	56
33	Canada	2007	Bananas	38
33	Canada	2007	Barley	1
33	Canada	2007	Beans	2
33	Canada	2007	Beer	190
33	Canada	2007	Beverages, Alcoholic	1
33	Canada	2007	Beverages, Fermented	43
33	Canada	2007	Bovine Meat	90
33	Canada	2007	Butter, Ghee	7
33	Canada	2007	Cassava	1
33	Canada	2007	Cereals, Other	1
33	Canada	2007	Cheese	35
33	Canada	2007	Citrus, Other	1
33	Canada	2007	Cocoa Beans	2
33	Canada	2007	Coconut Oil	1
33	Canada	2007	Coconuts - Incl Copra	3
33	Canada	2007	Coffee	20
33	Canada	2007	Cottonseed Oil	1
33	Canada	2007	Cream	24
33	Canada	2007	Crustaceans	12
33	Canada	2007	Dates	1
33	Canada	2007	Demersal Fish	17
33	Canada	2007	Eggs	30
33	Canada	2007	Fats, Animals, Raw	19
33	Canada	2007	Freshwater Fish	10
33	Canada	2007	Fruits, Other	86
33	Canada	2007	Grapefruit	8
33	Canada	2007	Grapes	30
33	Canada	2007	Groundnuts (Shelled Eq)	7
33	Canada	2007	Honey	2
33	Canada	2007	Lemons, Limes	8
33	Canada	2007	Maize	53
33	Canada	2007	Maize Germ Oil	4
33	Canada	2007	Marine Fish, Other	1
33	Canada	2007	Milk, Whole	97
33	Canada	2007	Molluscs, Other	11
33	Canada	2007	Mutton & Goat Meat	3
33	Canada	2007	Nuts	12
33	Canada	2007	Oats	2
33	Canada	2007	Offals, Edible	4
33	Canada	2007	Olive Oil	3
33	Canada	2007	Olives	2
33	Canada	2007	Onions	23
33	Canada	2007	Oranges, Mandarines	150
33	Canada	2007	Peas	4
33	Canada	2007	Pelagic Fish	12
33	Canada	2007	Pigmeat	75
33	Canada	2007	Pineapples	14
33	Canada	2007	Potatoes	190
33	Canada	2007	Poultry Meat	103
33	Canada	2007	Pulses, Other	14
33	Canada	2007	Rape and Mustard Oil	41
33	Canada	2007	Rice (Milled Equivalent)	25
33	Canada	2007	Roots, Other	1
33	Canada	2007	Rye	1
33	Canada	2007	Soyabean Oil	16
33	Canada	2007	Soyabeans	2
33	Canada	2007	Spices, Other	1
33	Canada	2007	Sugar, Refined Equivalent	97
33	Canada	2007	Sunflowerseed Oil	2
33	Canada	2007	Sweet Potatoes	2
33	Canada	2007	Sweeteners, Other	39
33	Canada	2007	Tea	1
33	Canada	2007	Tomatoes	87
33	Canada	2007	Vegetables, Other	226
33	Canada	2007	Wheat	244
33	Canada	2007	Whey	20
33	Canada	2007	Wine	30
33	Canada	2008	Apples	49
33	Canada	2008	Bananas	38
33	Canada	2008	Beans	3
33	Canada	2008	Beer	190
33	Canada	2008	Beverages, Alcoholic	2
33	Canada	2008	Beverages, Fermented	43
33	Canada	2008	Bovine Meat	86
33	Canada	2008	Butter, Ghee	8
33	Canada	2008	Cassava	1
33	Canada	2008	Cheese	34
33	Canada	2008	Citrus, Other	4
33	Canada	2008	Cocoa Beans	6
33	Canada	2008	Coconut Oil	1
33	Canada	2008	Coconuts - Incl Copra	3
33	Canada	2008	Coffee	18
33	Canada	2008	Cottonseed Oil	1
33	Canada	2008	Cream	23
33	Canada	2008	Crustaceans	15

FAO country code	Country	Reference year	Food item	Gross food supply (g *capita*$^{-1}$ d^{-1})
33	Canada	2008	Dates	1
33	Canada	2008	Demersal Fish	16
33	Canada	2008	Eggs	32
33	Canada	2008	Fats, Animals, Raw	18
33	Canada	2008	Freshwater Fish	9
33	Canada	2008	Fruits, Other	89
33	Canada	2008	Grapefruit	8
33	Canada	2008	Grapes	28
33	Canada	2008	Groundnuts (Shelled Eq)	7
33	Canada	2008	Honey	1
33	Canada	2008	Lemons, Limes	7
33	Canada	2008	Maize	53
33	Canada	2008	Maize Germ Oil	4
33	Canada	2008	Marine Fish, Other	1
33	Canada	2008	Milk, Whole	96
33	Canada	2008	Molluscs, Other	10
33	Canada	2008	Mutton & Goat Meat	3
33	Canada	2008	Nuts	12
33	Canada	2008	Oats	2
33	Canada	2008	Offals, Edible	3
33	Canada	2008	Oilcrops, Other	5
33	Canada	2008	Olive Oil	3
33	Canada	2008	Olives	2
33	Canada	2008	Onions	21
33	Canada	2008	Oranges, Mandarines	126
33	Canada	2008	Peas	6
33	Canada	2008	Pelagic Fish	10
33	Canada	2008	Pigmeat	72
33	Canada	2008	Pineapples	14
33	Canada	2008	Potatoes	173
33	Canada	2008	Poultry Meat	103
33	Canada	2008	Pulses, Other	15
33	Canada	2008	Rape and Mustard Oil	41
33	Canada	2008	Rice (Milled Equivalent)	27
33	Canada	2008	Roots, Other	1
33	Canada	2008	Rye	2
33	Canada	2008	Soyabean Oil	16
33	Canada	2008	Soyabeans	2
33	Canada	2008	Spices, Other	2
33	Canada	2008	Sugar, Refined Equivalent	96
33	Canada	2008	Sunflowerseed Oil	2
33	Canada	2008	Sweet Potatoes	2
33	Canada	2008	Sweeteners, Other	35
33	Canada	2008	Tea	1
33	Canada	2008	Tomatoes	80
33	Canada	2008	Vegetables, Other	212
33	Canada	2008	Wheat	232
33	Canada	2008	Whey	22
33	Canada	2008	Wine	30
33	Canada	2009	Apples	60
33	Canada	2009	Bananas	38
33	Canada	2009	Beans	2
33	Canada	2009	Beer	186
33	Canada	2009	Beverages, Alcoholic	1
33	Canada	2009	Beverages, Fermented	40
33	Canada	2009	Bovine Meat	84
33	Canada	2009	Butter, Ghee	8
33	Canada	2009	Cassava	1
33	Canada	2009	Cheese	34
33	Canada	2009	Citrus, Other	4
33	Canada	2009	Cocoa Beans	6
33	Canada	2009	Coconut Oil	1
33	Canada	2009	Coconuts - Incl Copra	3
33	Canada	2009	Coffee	18
33	Canada	2009	Cottonseed Oil	1
33	Canada	2009	Cream	23
33	Canada	2009	Crustaceans	14
33	Canada	2009	Dates	1
33	Canada	2009	Demersal Fish	15
33	Canada	2009	Eggs	33
33	Canada	2009	Fats, Animals, Raw	18
33	Canada	2009	Freshwater Fish	11
33	Canada	2009	Fruits, Other	85
33	Canada	2009	Grapefruit	7
33	Canada	2009	Grapes	26
33	Canada	2009	Groundnuts (Shelled Eq)	7
33	Canada	2009	Honey	2
33	Canada	2009	Lemons, Limes	7
33	Canada	2009	Maize	53
33	Canada	2009	Maize Germ Oil	4
33	Canada	2009	Marine Fish, Other	2
33	Canada	2009	Milk, Whole	99
33	Canada	2009	Molluscs, Other	9
33	Canada	2009	Mutton & Goat Meat	3
33	Canada	2009	Nuts	11
33	Canada	2009	Oats	2
33	Canada	2009	Offals, Edible	4
33	Canada	2009	Oilcrops, Other	1
33	Canada	2009	Olive Oil	3
33	Canada	2009	Olives	2
33	Canada	2009	Onions	28
33	Canada	2009	Oranges, Mandarines	128
33	Canada	2009	Palm Oil	2
33	Canada	2009	Peas	4
33	Canada	2009	Pelagic Fish	12
33	Canada	2009	Pigmeat	70
33	Canada	2009	Pimento	1

FAO country code	Country	Reference year	Food item	Gross food supply (g *capita*$^{-1}$ d^{-1})
33	Canada	2009	Pineapples	12
33	Canada	2009	Potatoes	199
33	Canada	2009	Poultry Meat	100
33	Canada	2009	Pulses, Other	17
33	Canada	2009	Rape and Mustard Oil	41
33	Canada	2009	Rice (Milled Equivalent)	25
33	Canada	2009	Roots, Other	1
33	Canada	2009	Rye	2
33	Canada	2009	Sesameseed	1
33	Canada	2009	Soyabean Oil	15
33	Canada	2009	Soyabeans	2
33	Canada	2009	Spices, Other	2
33	Canada	2009	Sugar, Refined Equivalent	78
33	Canada	2009	Sunflowerseed Oil	2
33	Canada	2009	Sweet Potatoes	3
33	Canada	2009	Sweeteners, Other	36
33	Canada	2009	Tea	1
33	Canada	2009	Tomatoes	57
33	Canada	2009	Vegetables, Other	228
33	Canada	2009	Wheat	232
33	Canada	2009	Whey	15
33	Canada	2009	Wine	30
33	Canada	2010	Apples	56
33	Canada	2010	Bananas	39
33	Canada	2010	Barley	1
33	Canada	2010	Beans	2
33	Canada	2010	Beer	158
33	Canada	2010	Beverages, Alcoholic	1
33	Canada	2010	Beverages, Fermented	39
33	Canada	2010	Bovine Meat	82
33	Canada	2010	Butter, Ghee	7
33	Canada	2010	Cassava	1
33	Canada	2010	Cereals, Other	1
33	Canada	2010	Cheese	35
33	Canada	2010	Citrus, Other	4
33	Canada	2010	Cocoa Beans	6
33	Canada	2010	Coconut Oil	1
33	Canada	2010	Coconuts - Incl Copra	3
33	Canada	2010	Coffee	21
33	Canada	2010	Cottonseed Oil	1
33	Canada	2010	Cream	23
33	Canada	2010	Crustaceans	15
33	Canada	2010	Dates	1
33	Canada	2010	Demersal Fish	14
33	Canada	2010	Eggs	33
33	Canada	2010	Fats, Animals, Raw	18
33	Canada	2010	Freshwater Fish	9
33	Canada	2010	Fruits, Other	83
33	Canada	2010	Grapefruit	6
33	Canada	2010	Grapes	27
33	Canada	2010	Groundnuts (Shelled Eq)	8
33	Canada	2010	Honey	2
33	Canada	2010	Lemons, Limes	7
33	Canada	2010	Maize	54
33	Canada	2010	Maize Germ Oil	5
33	Canada	2010	Marine Fish, Other	2
33	Canada	2010	Milk, Whole	103
33	Canada	2010	Molluscs, Other	9
33	Canada	2010	Mutton & Goat Meat	3
33	Canada	2010	Nuts	12
33	Canada	2010	Oats	1
33	Canada	2010	Offals, Edible	4
33	Canada	2010	Olive Oil	3
33	Canada	2010	Olives	2
33	Canada	2010	Onions	23
33	Canada	2010	Oranges, Mandarines	131
33	Canada	2010	Palm Oil	2
33	Canada	2010	Peas	3
33	Canada	2010	Pelagic Fish	13
33	Canada	2010	Pigmeat	66
33	Canada	2010	Pineapples	12
33	Canada	2010	Potatoes	223
33	Canada	2010	Poultry Meat	101
33	Canada	2010	Pulses, Other	22
33	Canada	2010	Rape and Mustard Oil	42
33	Canada	2010	Rice (Milled Equivalent)	24
33	Canada	2010	Roots, Other	1
33	Canada	2010	Rye	2
33	Canada	2010	Soyabean Oil	16
33	Canada	2010	Soyabeans	2
33	Canada	2010	Spices, Other	2
33	Canada	2010	Sugar, Refined Equivalent	70
33	Canada	2010	Sunflowerseed Oil	2
33	Canada	2010	Sweet Potatoes	4
33	Canada	2010	Sweeteners, Other	36
33	Canada	2010	Tea	1
33	Canada	2010	Tomatoes	57
33	Canada	2010	Vegetables, Other	225
33	Canada	2010	Wheat	229
33	Canada	2010	Whey	14
33	Canada	2010	Wine	32
33	Canada	2011	Apples	58
33	Canada	2011	Bananas	39
33	Canada	2011	Barley	1
33	Canada	2011	Beans	3
33	Canada	2011	Beer	158
33	Canada	2011	Beverages, Fermented	43

FAO country code	Country	Reference year	Food item	Gross food supply (g *capita*$^{-1}$ d^{-1})
33	Canada	2011	Bovine Meat	82
33	Canada	2011	Butter, Ghee	8
33	Canada	2011	Cassava	1
33	Canada	2011	Cereals, Other	2
33	Canada	2011	Cheese	36
33	Canada	2011	Citrus, Other	2
33	Canada	2011	Cocoa Beans	8
33	Canada	2011	Coconut Oil	1
33	Canada	2011	Coconuts - Incl Copra	3
33	Canada	2011	Coffee	21
33	Canada	2011	Cottonseed Oil	1
33	Canada	2011	Cream	24
33	Canada	2011	Crustaceans	15
33	Canada	2011	Dates	1
33	Canada	2011	Demersal Fish	14
33	Canada	2011	Eggs	33
33	Canada	2011	Fats, Animals, Raw	18
33	Canada	2011	Freshwater Fish	9
33	Canada	2011	Fruits, Other	87
33	Canada	2011	Grapefruit	6
33	Canada	2011	Grapes	26
33	Canada	2011	Groundnuts (Shelled Eq)	7
33	Canada	2011	Honey	2
33	Canada	2011	Lemons, Limes	9
33	Canada	2011	Maize	53
33	Canada	2011	Maize Germ Oil	4
33	Canada	2011	Marine Fish, Other	2
33	Canada	2011	Milk, Whole	112
33	Canada	2011	Molluscs, Other	9
33	Canada	2011	Mutton & Goat Meat	3
33	Canada	2011	Nuts	12
33	Canada	2011	Oats	2
33	Canada	2011	Offals, Edible	2
33	Canada	2011	Olive Oil	3
33	Canada	2011	Olives	2
33	Canada	2011	Onions	27
33	Canada	2011	Oranges, Mandarines	114
33	Canada	2011	Palm Oil	2
33	Canada	2011	Peas	5
33	Canada	2011	Pelagic Fish	12
33	Canada	2011	Pigmeat	67
33	Canada	2011	Pineapples	12
33	Canada	2011	Potatoes	215
33	Canada	2011	Poultry Meat	102
33	Canada	2011	Pulses, Other	24
33	Canada	2011	Rape and Mustard Oil	42
33	Canada	2011	Rice (Milled Equivalent)	27
33	Canada	2011	Roots, Other	1
33	Canada	2011	Rye	2
33	Canada	2011	Sesameseed	1
33	Canada	2011	Soyabean Oil	16
33	Canada	2011	Soyabeans	2
33	Canada	2011	Spices, Other	2
33	Canada	2011	Sugar, Refined Equivalent	86
33	Canada	2011	Sunflowerseed Oil	2
33	Canada	2011	Sweet Potatoes	4
33	Canada	2011	Sweeteners, Other	37
33	Canada	2011	Tea	1
33	Canada	2011	Tomatoes	58
33	Canada	2011	Vegetables, Other	228
33	Canada	2011	Wheat	191
33	Canada	2011	Whey	26
33	Canada	2011	Wine	31
37	Central African Republic	1992	Bananas	76
37	Central African Republic	1992	Beer	25
37	Central African Republic	1992	Beverages, Fermented	37
37	Central African Republic	1992	Bovine Meat	40
37	Central African Republic	1992	Cassava	505
37	Central African Republic	1992	Cereals, Other	3
37	Central African Republic	1992	Coffee	1
37	Central African Republic	1992	Cottonseed Oil	1
37	Central African Republic	1992	Eggs	1
37	Central African Republic	1992	Fats, Animals, Raw	2
37	Central African Republic	1992	Freshwater Fish	12
37	Central African Republic	1992	Fruits, Other	9
37	Central African Republic	1992	Grapefruit	3
37	Central African Republic	1992	Groundnut Oil	17
37	Central African Republic	1992	Honey	9
37	Central African Republic	1992	Lemons, Limes	1
37	Central African Republic	1992	Maize	58
37	Central African Republic	1992	Marine Fish, Other	1
37	Central African Republic	1992	Meat, Other	17
37	Central African Republic	1992	Milk, Whole	42
37	Central African Republic	1992	Millet	8
37	Central African Republic	1992	Mutton & Goat Meat	6
37	Central African Republic	1992	Offals, Edible	7
37	Central African Republic	1992	Oilcrops, Other	8
37	Central African Republic	1992	Oranges, Mandarines	13
37	Central African Republic	1992	Palm Oil	6
37	Central African Republic	1992	Pigmeat	7
37	Central African Republic	1992	Pineapples	9
37	Central African Republic	1992	Plantains	26
37	Central African Republic	1992	Potatoes	1
37	Central African Republic	1992	Poultry Meat	2
37	Central African Republic	1992	Pulses, Other	15
37	Central African Republic	1992	Rice (Milled Equivalent)	8
37	Central African Republic	1992	Roots, Other	51

FAO country code	Country	Reference year	Food item	Gross food supply (g $capita^{-1}$ d^{-1})
37	Central African Republic	1992	Sesameseed	12
37	Central African Republic	1992	Sesameseed Oil	4
37	Central African Republic	1992	Sorghum	24
37	Central African Republic	1992	Sugar, Refined Equivalent	8
37	Central African Republic	1992	Tomatoes	1
37	Central African Republic	1992	Vegetables, Other	57
37	Central African Republic	1992	Wheat	24
37	Central African Republic	1992	Wine	1
37	Central African Republic	1992	Yams	198
37	Central African Republic	1993	Bananas	76
37	Central African Republic	1993	Beer	34
37	Central African Republic	1993	Beverages, Fermented	38
37	Central African Republic	1993	Bovine Meat	40
37	Central African Republic	1993	Cassava	489
37	Central African Republic	1993	Coffee	5
37	Central African Republic	1993	Cottonseed Oil	1
37	Central African Republic	1993	Eggs	1
37	Central African Republic	1993	Fats, Animals, Raw	2
37	Central African Republic	1993	Freshwater Fish	12
37	Central African Republic	1993	Fruits, Other	10
37	Central African Republic	1993	Grapefruit	3
37	Central African Republic	1993	Groundnut Oil	17
37	Central African Republic	1993	Honey	9
37	Central African Republic	1993	Lemons, Limes	1
37	Central African Republic	1993	Maize	61
37	Central African Republic	1993	Meat, Other	17
37	Central African Republic	1993	Milk, Whole	46
37	Central African Republic	1993	Millet	8
37	Central African Republic	1993	Mutton & Goat Meat	6
37	Central African Republic	1993	Offals, Edible	7
37	Central African Republic	1993	Oilcrops, Other	8
37	Central African Republic	1993	Oranges, Mandarines	14
37	Central African Republic	1993	Palm Oil	6
37	Central African Republic	1993	Pigmeat	8
37	Central African Republic	1993	Pineapples	9
37	Central African Republic	1993	Plantains	26
37	Central African Republic	1993	Potatoes	1
37	Central African Republic	1993	Poultry Meat	2
37	Central African Republic	1993	Pulses, Other	17
37	Central African Republic	1993	Rice (Milled Equivalent)	8
37	Central African Republic	1993	Roots, Other	50
37	Central African Republic	1993	Sesameseed	11
37	Central African Republic	1993	Sesameseed Oil	4
37	Central African Republic	1993	Sorghum	23
37	Central African Republic	1993	Sugar, Refined Equivalent	8
37	Central African Republic	1993	Tomatoes	1
37	Central African Republic	1993	Vegetables, Other	58
37	Central African Republic	1993	Wheat	27
37	Central African Republic	1993	Wine	1
37	Central African Republic	1993	Yams	192
37	Central African Republic	1994	Bananas	75
37	Central African Republic	1994	Beer	23
37	Central African Republic	1994	Beverages, Fermented	41
37	Central African Republic	1994	Bovine Meat	39
37	Central African Republic	1994	Cassava	430
37	Central African Republic	1994	Coffee	6
37	Central African Republic	1994	Cottonseed Oil	2
37	Central African Republic	1994	Eggs	1
37	Central African Republic	1994	Fats, Animals, Raw	2
37	Central African Republic	1994	Freshwater Fish	12
37	Central African Republic	1994	Fruits, Other	10
37	Central African Republic	1994	Grapefruit	3
37	Central African Republic	1994	Groundnut Oil	19
37	Central African Republic	1994	Honey	9
37	Central African Republic	1994	Lemons, Limes	1
37	Central African Republic	1994	Maize	67
37	Central African Republic	1994	Meat, Other	17
37	Central African Republic	1994	Milk, Whole	43
37	Central African Republic	1994	Millet	7
37	Central African Republic	1994	Mutton & Goat Meat	6
37	Central African Republic	1994	Offals, Edible	7
37	Central African Republic	1994	Oilcrops, Other	9
37	Central African Republic	1994	Oranges, Mandarines	14
37	Central African Republic	1994	Palm Oil	6
37	Central African Republic	1994	Pigmeat	8
37	Central African Republic	1994	Pineapples	9
37	Central African Republic	1994	Plantains	26
37	Central African Republic	1994	Potatoes	1
37	Central African Republic	1994	Poultry Meat	2
37	Central African Republic	1994	Pulses, Other	15
37	Central African Republic	1994	Rice (Milled Equivalent)	8
37	Central African Republic	1994	Roots, Other	57
37	Central African Republic	1994	Sesameseed	13
37	Central African Republic	1994	Sesameseed Oil	4
37	Central African Republic	1994	Sorghum	25
37	Central African Republic	1994	Sugar, Refined Equivalent	11
37	Central African Republic	1994	Vegetables, Other	59
37	Central African Republic	1994	Wheat	18
37	Central African Republic	1994	Wine	1
37	Central African Republic	1994	Yams	204
37	Central African Republic	1995	Bananas	75
37	Central African Republic	1995	Beer	20
37	Central African Republic	1995	Beverages, Fermented	36
37	Central African Republic	1995	Bovine Meat	40
37	Central African Republic	1995	Cassava	399
37	Central African Republic	1995	Coffee	4
37	Central African Republic	1995	Cottonseed Oil	2

FAO country code	Country	Reference year	Food item	Gross food supply (g *capita*$^{-1}$ d^{-1})
37	Central African Republic	1995	Eggs	1
37	Central African Republic	1995	Fats, Animals, Raw	2
37	Central African Republic	1995	Freshwater Fish	12
37	Central African Republic	1995	Fruits, Other	8
37	Central African Republic	1995	Grapefruit	3
37	Central African Republic	1995	Groundnut Oil	17
37	Central African Republic	1995	Honey	9
37	Central African Republic	1995	Lemons, Limes	1
37	Central African Republic	1995	Maize	60
37	Central African Republic	1995	Meat, Other	17
37	Central African Republic	1995	Milk, Whole	44
37	Central African Republic	1995	Millet	7
37	Central African Republic	1995	Mutton & Goat Meat	6
37	Central African Republic	1995	Offals, Edible	7
37	Central African Republic	1995	Oilcrops, Other	9
37	Central African Republic	1995	Oranges, Mandarines	15
37	Central African Republic	1995	Palm Oil	6
37	Central African Republic	1995	Pigmeat	8
37	Central African Republic	1995	Pineapples	9
37	Central African Republic	1995	Plantains	26
37	Central African Republic	1995	Potatoes	1
37	Central African Republic	1995	Poultry Meat	2
37	Central African Republic	1995	Pulses, Other	16
37	Central African Republic	1995	Rice (Milled Equivalent)	8
37	Central African Republic	1995	Roots, Other	72
37	Central African Republic	1995	Sesameseed	14
37	Central African Republic	1995	Sesameseed Oil	4
37	Central African Republic	1995	Sorghum	22
37	Central African Republic	1995	Sugar, Refined Equivalent	12
37	Central African Republic	1995	Tomatoes	1
37	Central African Republic	1995	Vegetables, Other	63
37	Central African Republic	1995	Wheat	27
37	Central African Republic	1995	Wine	2
37	Central African Republic	1995	Yams	227
37	Central African Republic	1996	Bananas	77
37	Central African Republic	1996	Beer	9
37	Central African Republic	1996	Beverages, Fermented	45
37	Central African Republic	1996	Bovine Meat	50
37	Central African Republic	1996	Cassava	417
37	Central African Republic	1996	Coffee	4
37	Central African Republic	1996	Cottonseed Oil	1
37	Central African Republic	1996	Eggs	1
37	Central African Republic	1996	Fats, Animals, Raw	2
37	Central African Republic	1996	Freshwater Fish	12
37	Central African Republic	1996	Fruits, Other	10
37	Central African Republic	1996	Grapefruit	3
37	Central African Republic	1996	Groundnut Oil	19
37	Central African Republic	1996	Honey	9
37	Central African Republic	1996	Lemons, Limes	1
37	Central African Republic	1996	Maize	75
37	Central African Republic	1996	Meat, Other	17
37	Central African Republic	1996	Milk, Whole	45
37	Central African Republic	1996	Millet	7
37	Central African Republic	1996	Mutton & Goat Meat	7
37	Central African Republic	1996	Offals, Edible	7
37	Central African Republic	1996	Oilcrops, Other	10
37	Central African Republic	1996	Oranges, Mandarines	14
37	Central African Republic	1996	Palm Oil	2
37	Central African Republic	1996	Pigmeat	9
37	Central African Republic	1996	Pineapples	9
37	Central African Republic	1996	Plantains	26
37	Central African Republic	1996	Potatoes	1
37	Central African Republic	1996	Poultry Meat	3
37	Central African Republic	1996	Pulses, Other	21
37	Central African Republic	1996	Rice (Milled Equivalent)	4
37	Central African Republic	1996	Roots, Other	75
37	Central African Republic	1996	Sesameseed	14
37	Central African Republic	1996	Sesameseed Oil	4
37	Central African Republic	1996	Sorghum	25
37	Central African Republic	1996	Sugar, Refined Equivalent	7
37	Central African Republic	1996	Vegetables, Other	56
37	Central African Republic	1996	Wheat	7
37	Central African Republic	1996	Yams	243
37	Central African Republic	1997	Bananas	79
37	Central African Republic	1997	Beer	11
37	Central African Republic	1997	Beverages, Fermented	36
37	Central African Republic	1997	Bovine Meat	40
37	Central African Republic	1997	Cassava	449
37	Central African Republic	1997	Coffee	3
37	Central African Republic	1997	Cottonseed Oil	2
37	Central African Republic	1997	Eggs	1
37	Central African Republic	1997	Fats, Animals, Raw	2
37	Central African Republic	1997	Freshwater Fish	11
37	Central African Republic	1997	Fruits, Other	10
37	Central African Republic	1997	Grapefruit	3
37	Central African Republic	1997	Groundnut Oil	20
37	Central African Republic	1997	Honey	9
37	Central African Republic	1997	Lemons, Limes	1
37	Central African Republic	1997	Maize	55
37	Central African Republic	1997	Meat, Other	17
37	Central African Republic	1997	Milk, Whole	47
37	Central African Republic	1997	Millet	8
37	Central African Republic	1997	Mutton & Goat Meat	7
37	Central African Republic	1997	Offals, Edible	7
37	Central African Republic	1997	Oilcrops, Other	10
37	Central African Republic	1997	Oranges, Mandarines	16
37	Central African Republic	1997	Palm Oil	3

FAO countrycode	Country	Reference year	Food item	Gross food supply (g $capita^{-1}$ d^{-1})
37	Central African Republic	1997	Pelagic Fish	1
37	Central African Republic	1997	Pigmeat	9
37	Central African Republic	1997	Pineapples	10
37	Central African Republic	1997	Plantains	26
37	Central African Republic	1997	Potatoes	1
37	Central African Republic	1997	Poultry Meat	3
37	Central African Republic	1997	Pulses, Other	16
37	Central African Republic	1997	Rice (Milled Equivalent)	9
37	Central African Republic	1997	Roots, Other	76
37	Central African Republic	1997	Sesameseed	13
37	Central African Republic	1997	Sesameseed Oil	5
37	Central African Republic	1997	Sorghum	23
37	Central African Republic	1997	Sugar, Refined Equivalent	14
37	Central African Republic	1997	Vegetables, Other	60
37	Central African Republic	1997	Wheat	12
37	Central African Republic	1997	Yams	258
37	Central African Republic	1998	Bananas	81
37	Central African Republic	1998	Beer	15
37	Central African Republic	1998	Beverages, Fermented	37
37	Central African Republic	1998	Bovine Meat	40
37	Central African Republic	1998	Cassava	462
37	Central African Republic	1998	Cereals, Other	1
37	Central African Republic	1998	Coffee	6
37	Central African Republic	1998	Cottonseed Oil	2
37	Central African Republic	1998	Eggs	1
37	Central African Republic	1998	Fats, Animals, Raw	3
37	Central African Republic	1998	Freshwater Fish	11
37	Central African Republic	1998	Fruits, Other	10
37	Central African Republic	1998	Grapefruit	3
37	Central African Republic	1998	Groundnut Oil	19
37	Central African Republic	1998	Honey	9
37	Central African Republic	1998	Lemons, Limes	1
37	Central African Republic	1998	Maize	58
37	Central African Republic	1998	Meat, Other	17
37	Central African Republic	1998	Milk, Whole	46
37	Central African Republic	1998	Millet	10
37	Central African Republic	1998	Mutton & Goat Meat	7
37	Central African Republic	1998	Offals, Edible	7
37	Central African Republic	1998	Oilcrops, Other	11
37	Central African Republic	1998	Oranges, Mandarines	16
37	Central African Republic	1998	Palm Oil	2
37	Central African Republic	1998	Pigmeat	9
37	Central African Republic	1998	Pineapples	10
37	Central African Republic	1998	Plantains	26
37	Central African Republic	1998	Potatoes	1
37	Central African Republic	1998	Poultry Meat	2
37	Central African Republic	1998	Pulses, Other	21
37	Central African Republic	1998	Rice (Milled Equivalent)	11
37	Central African Republic	1998	Roots, Other	82
37	Central African Republic	1998	Sesameseed	12
37	Central African Republic	1998	Sesameseed Oil	5
37	Central African Republic	1998	Sorghum	23
37	Central African Republic	1998	Sugar, Refined Equivalent	14
37	Central African Republic	1998	Vegetables, Other	60
37	Central African Republic	1998	Wheat	22
37	Central African Republic	1998	Yams	260
37	Central African Republic	1999	Bananas	74
37	Central African Republic	1999	Beer	9
37	Central African Republic	1999	Beverages, Fermented	37
37	Central African Republic	1999	Bovine Meat	39
37	Central African Republic	1999	Cassava	416
37	Central African Republic	1999	Coffee	1
37	Central African Republic	1999	Cottonseed Oil	1
37	Central African Republic	1999	Eggs	1
37	Central African Republic	1999	Fats, Animals, Raw	3
37	Central African Republic	1999	Freshwater Fish	12
37	Central African Republic	1999	Fruits, Other	10
37	Central African Republic	1999	Grapefruit	3
37	Central African Republic	1999	Groundnut Oil	20
37	Central African Republic	1999	Honey	10
37	Central African Republic	1999	Lemons, Limes	1
37	Central African Republic	1999	Maize	62
37	Central African Republic	1999	Meat, Other	17
37	Central African Republic	1999	Milk, Whole	47
37	Central African Republic	1999	Millet	6
37	Central African Republic	1999	Mutton & Goat Meat	7
37	Central African Republic	1999	Offals, Edible	7
37	Central African Republic	1999	Oilcrops, Other	12
37	Central African Republic	1999	Oranges, Mandarines	16
37	Central African Republic	1999	Palm Oil	2
37	Central African Republic	1999	Pelagic Fish	1
37	Central African Republic	1999	Pigmeat	9
37	Central African Republic	1999	Pineapples	10
37	Central African Republic	1999	Plantains	23
37	Central African Republic	1999	Poultry Meat	2
37	Central African Republic	1999	Pulses, Other	16
37	Central African Republic	1999	Rice (Milled Equivalent)	11
37	Central African Republic	1999	Roots, Other	73
37	Central African Republic	1999	Sesameseed	12
37	Central African Republic	1999	Sesameseed Oil	6
37	Central African Republic	1999	Sorghum	28
37	Central African Republic	1999	Sugar, Refined Equivalent	10
37	Central African Republic	1999	Tomatoes	1
37	Central African Republic	1999	Vegetables, Other	61
37	Central African Republic	1999	Wheat	26
37	Central African Republic	1999	Yams	235
37	Central African Republic	2000	Bananas	80

FAO country code	Country	Reference year	Food item	Gross food supply (g *capita*$^{-1}$ d^{-1})
37	Central African Republic	2000	Beer	18
37	Central African Republic	2000	Beverages, Fermented	39
37	Central African Republic	2000	Bovine Meat	51
37	Central African Republic	2000	Cassava	409
37	Central African Republic	2000	Coffee	3
37	Central African Republic	2000	Cottonseed Oil	1
37	Central African Republic	2000	Eggs	1
37	Central African Republic	2000	Fats, Animals, Raw	3
37	Central African Republic	2000	Freshwater Fish	11
37	Central African Republic	2000	Fruits, Other	10
37	Central African Republic	2000	Grapefruit	3
37	Central African Republic	2000	Groundnut Oil	18
37	Central African Republic	2000	Honey	10
37	Central African Republic	2000	Lemons, Limes	1
37	Central African Republic	2000	Maize	63
37	Central African Republic	2000	Meat, Other	17
37	Central African Republic	2000	Milk, Whole	47
37	Central African Republic	2000	Millet	5
37	Central African Republic	2000	Mutton & Goat Meat	9
37	Central African Republic	2000	Offals, Edible	8
37	Central African Republic	2000	Oilcrops, Other	13
37	Central African Republic	2000	Oranges, Mandarines	16
37	Central African Republic	2000	Palm Oil	2
37	Central African Republic	2000	Pigmeat	9
37	Central African Republic	2000	Pineapples	9
37	Central African Republic	2000	Plantains	25
37	Central African Republic	2000	Potatoes	1
37	Central African Republic	2000	Poultry Meat	2
37	Central African Republic	2000	Pulses, Other	17
37	Central African Republic	2000	Rice (Milled Equivalent)	12
37	Central African Republic	2000	Roots, Other	75
37	Central African Republic	2000	Sesameseed	11
37	Central African Republic	2000	Sesameseed Oil	7
37	Central African Republic	2000	Sorghum	25
37	Central African Republic	2000	Sugar, Refined Equivalent	16
37	Central African Republic	2000	Tomatoes	1
37	Central African Republic	2000	Vegetables, Other	61
37	Central African Republic	2000	Wheat	29
37	Central African Republic	2000	Yams	243
37	Central African Republic	2001	Bananas	79
37	Central African Republic	2001	Beer	14
37	Central African Republic	2001	Beverages, Fermented	40
37	Central African Republic	2001	Bovine Meat	50
37	Central African Republic	2001	Cassava	403
37	Central African Republic	2001	Coffee	3
37	Central African Republic	2001	Cottonseed Oil	1
37	Central African Republic	2001	Eggs	1
37	Central African Republic	2001	Fats, Animals, Raw	3
37	Central African Republic	2001	Freshwater Fish	13
37	Central African Republic	2001	Fruits, Other	10
37	Central African Republic	2001	Grapefruit	3
37	Central African Republic	2001	Groundnut Oil	21
37	Central African Republic	2001	Honey	10
37	Central African Republic	2001	Lemons, Limes	1
37	Central African Republic	2001	Maize	66
37	Central African Republic	2001	Meat, Other	17
37	Central African Republic	2001	Milk, Whole	46
37	Central African Republic	2001	Millet	7
37	Central African Republic	2001	Mutton & Goat Meat	9
37	Central African Republic	2001	Offals, Edible	8
37	Central African Republic	2001	Oilcrops, Other	13
37	Central African Republic	2001	Oranges, Mandarines	15
37	Central African Republic	2001	Palm Oil	2
37	Central African Republic	2001	Pelagic Fish	1
37	Central African Republic	2001	Pigmeat	9
37	Central African Republic	2001	Pineapples	9
37	Central African Republic	2001	Plantains	25
37	Central African Republic	2001	Poultry Meat	2
37	Central African Republic	2001	Pulses, Other	18
37	Central African Republic	2001	Rice (Milled Equivalent)	14
37	Central African Republic	2001	Roots, Other	74
37	Central African Republic	2001	Rye	1
37	Central African Republic	2001	Sesameseed	14
37	Central African Republic	2001	Sesameseed Oil	6
37	Central African Republic	2001	Sorghum	26
37	Central African Republic	2001	Sugar, Refined Equivalent	10
37	Central African Republic	2001	Vegetables, Other	60
37	Central African Republic	2001	Wheat	21
37	Central African Republic	2001	Wine	1
37	Central African Republic	2001	Yams	239
37	Central African Republic	2002	Bananas	75
37	Central African Republic	2002	Beer	9
37	Central African Republic	2002	Beverages, Fermented	41
37	Central African Republic	2002	Bovine Meat	50
37	Central African Republic	2002	Cassava	397
37	Central African Republic	2002	Coffee	3
37	Central African Republic	2002	Cottonseed Oil	1
37	Central African Republic	2002	Eggs	1
37	Central African Republic	2002	Fats, Animals, Raw	3
37	Central African Republic	2002	Freshwater Fish	14
37	Central African Republic	2002	Fruits, Other	9
37	Central African Republic	2002	Grapefruit	3
37	Central African Republic	2002	Groundnut Oil	22
37	Central African Republic	2002	Honey	11
37	Central African Republic	2002	Lemons, Limes	1
37	Central African Republic	2002	Maize	69
37	Central African Republic	2002	Meat, Other	17

FAO country code	Country	Reference year	Food item	Gross food supply (g *capita*$^{-1}$ d^{-1})
37	Central African Republic	2002	Milk, Whole	47
37	Central African Republic	2002	Millet	7
37	Central African Republic	2002	Mutton & Goat Meat	9
37	Central African Republic	2002	Offals, Edible	8
37	Central African Republic	2002	Oilcrops Oil, Other	1
37	Central African Republic	2002	Oilcrops, Other	14
37	Central African Republic	2002	Oranges, Mandarines	14
37	Central African Republic	2002	Palm Oil	1
37	Central African Republic	2002	Pigmeat	9
37	Central African Republic	2002	Pineapples	9
37	Central African Republic	2002	Plantains	24
37	Central African Republic	2002	Poultry Meat	3
37	Central African Republic	2002	Pulses, Other	19
37	Central African Republic	2002	Rice (Milled Equivalent)	14
37	Central African Republic	2002	Roots, Other	69
37	Central African Republic	2002	Sesameseed	15
37	Central African Republic	2002	Sesameseed Oil	6
37	Central African Republic	2002	Sorghum	31
37	Central African Republic	2002	Sugar, Refined Equivalent	10
37	Central African Republic	2002	Vegetables, Other	62
37	Central African Republic	2002	Wheat	22
37	Central African Republic	2002	Yams	216
37	Central African Republic	2003	Bananas	71
37	Central African Republic	2003	Beer	7
37	Central African Republic	2003	Beverages, Fermented	41
37	Central African Republic	2003	Bovine Meat	47
37	Central African Republic	2003	Cassava	392
37	Central African Republic	2003	Coffee	3
37	Central African Republic	2003	Eggs	1
37	Central African Republic	2003	Fats, Animals, Raw	3
37	Central African Republic	2003	Freshwater Fish	15
37	Central African Republic	2003	Fruits, Other	9
37	Central African Republic	2003	Grapefruit	3
37	Central African Republic	2003	Groundnut Oil	23
37	Central African Republic	2003	Honey	11
37	Central African Republic	2003	Lemons, Limes	1
37	Central African Republic	2003	Maize	72
37	Central African Republic	2003	Meat, Other	17
37	Central African Republic	2003	Milk, Whole	47
37	Central African Republic	2003	Millet	7
37	Central African Republic	2003	Mutton & Goat Meat	9
37	Central African Republic	2003	Offals, Edible	8
37	Central African Republic	2003	Oilcrops, Other	14
37	Central African Republic	2003	Oranges, Mandarines	13
37	Central African Republic	2003	Palm Oil	1
37	Central African Republic	2003	Pelagic Fish	1
37	Central African Republic	2003	Pigmeat	9
37	Central African Republic	2003	Pineapples	9
37	Central African Republic	2003	Plantains	23
37	Central African Republic	2003	Poultry Meat	3
37	Central African Republic	2003	Pulses, Other	15
37	Central African Republic	2003	Rice (Milled Equivalent)	15
37	Central African Republic	2003	Roots, Other	54
37	Central African Republic	2003	Sesameseed	16
37	Central African Republic	2003	Sesameseed Oil	5
37	Central African Republic	2003	Sorghum	26
37	Central African Republic	2003	Soyabean Oil	1
37	Central African Republic	2003	Sugar, Refined Equivalent	14
37	Central African Republic	2003	Tomatoes	1
37	Central African Republic	2003	Vegetables, Other	62
37	Central African Republic	2003	Wheat	25
37	Central African Republic	2003	Yams	201
37	Central African Republic	2004	Bananas	73
37	Central African Republic	2004	Beer	10
37	Central African Republic	2004	Beverages, Fermented	42
37	Central African Republic	2004	Bovine Meat	52
37	Central African Republic	2004	Cassava	386
37	Central African Republic	2004	Coffee	3
37	Central African Republic	2004	Eggs	1
37	Central African Republic	2004	Fats, Animals, Raw	3
37	Central African Republic	2004	Freshwater Fish	16
37	Central African Republic	2004	Fruits, Other	9
37	Central African Republic	2004	Grapefruit	3
37	Central African Republic	2004	Groundnut Oil	23
37	Central African Republic	2004	Honey	11
37	Central African Republic	2004	Lemons, Limes	1
37	Central African Republic	2004	Maize	75
37	Central African Republic	2004	Meat, Other	17
37	Central African Republic	2004	Milk, Whole	48
37	Central African Republic	2004	Millet	6
37	Central African Republic	2004	Mutton & Goat Meat	9
37	Central African Republic	2004	Offals, Edible	9
37	Central African Republic	2004	Oilcrops Oil, Other	1
37	Central African Republic	2004	Oilcrops, Other	14
37	Central African Republic	2004	Oranges, Mandarines	13
37	Central African Republic	2004	Palm Oil	2
37	Central African Republic	2004	Peas	1
37	Central African Republic	2004	Pelagic Fish	1
37	Central African Republic	2004	Pigmeat	10
37	Central African Republic	2004	Pineapples	9
37	Central African Republic	2004	Plantains	23
37	Central African Republic	2004	Poultry Meat	3
37	Central African Republic	2004	Pulses, Other	15
37	Central African Republic	2004	Rice (Milled Equivalent)	10
37	Central African Republic	2004	Roots, Other	67
37	Central African Republic	2004	Sesameseed	17
37	Central African Republic	2004	Sesameseed Oil	5

FAO countrycode	Country	Reference year	Food item	Gross food supply (g *capita*$^{-1}$ d^{-1})
37	Central African Republic	2004	Sorghum	26
37	Central African Republic	2004	Sugar, Refined Equivalent	16
37	Central African Republic	2004	Tomatoes	1
37	Central African Republic	2004	Vegetables, Other	60
37	Central African Republic	2004	Wheat	19
37	Central African Republic	2004	Yams	209
37	Central African Republic	2005	Bananas	70
37	Central African Republic	2005	Beer	14
37	Central African Republic	2005	Beverages, Fermented	43
37	Central African Republic	2005	Bovine Meat	53
37	Central African Republic	2005	Cassava	388
37	Central African Republic	2005	Coffee	2
37	Central African Republic	2005	Eggs	1
37	Central African Republic	2005	Fats, Animals, Raw	3
37	Central African Republic	2005	Freshwater Fish	17
37	Central African Republic	2005	Fruits, Other	9
37	Central African Republic	2005	Grapefruit	3
37	Central African Republic	2005	Groundnut Oil	24
37	Central African Republic	2005	Honey	9
37	Central African Republic	2005	Lemons, Limes	1
37	Central African Republic	2005	Maize	79
37	Central African Republic	2005	Meat, Other	17
37	Central African Republic	2005	Milk, Whole	47
37	Central African Republic	2005	Millet	7
37	Central African Republic	2005	Mutton & Goat Meat	10
37	Central African Republic	2005	Offals, Edible	9
37	Central African Republic	2005	Oilcrops Oil, Other	1
37	Central African Republic	2005	Oilcrops, Other	13
37	Central African Republic	2005	Oranges, Mandarines	13
37	Central African Republic	2005	Palm Oil	3
37	Central African Republic	2005	Peas	1
37	Central African Republic	2005	Pelagic Fish	1
37	Central African Republic	2005	Pigmeat	9
37	Central African Republic	2005	Pineapples	9
37	Central African Republic	2005	Plantains	22
37	Central African Republic	2005	Poultry Meat	3
37	Central African Republic	2005	Pulses, Other	16
37	Central African Republic	2005	Rice (Milled Equivalent)	16
37	Central African Republic	2005	Roots, Other	67
37	Central African Republic	2005	Sesameseed	10
37	Central African Republic	2005	Sesameseed Oil	5
37	Central African Republic	2005	Sorghum	28
37	Central African Republic	2005	Sugar, Refined Equivalent	18
37	Central African Republic	2005	Tomatoes	1
37	Central African Republic	2005	Vegetables, Other	61
37	Central African Republic	2005	Wheat	24
37	Central African Republic	2005	Yams	210
37	Central African Republic	2006	Bananas	70
37	Central African Republic	2006	Beer	16
37	Central African Republic	2006	Beverages, Fermented	43
37	Central African Republic	2006	Bovine Meat	53
37	Central African Republic	2006	Cassava	377
37	Central African Republic	2006	Coffee	1
37	Central African Republic	2006	Eggs	1
37	Central African Republic	2006	Fats, Animals, Raw	3
37	Central African Republic	2006	Freshwater Fish	18
37	Central African Republic	2006	Fruits, Other	9
37	Central African Republic	2006	Grapefruit	3
37	Central African Republic	2006	Groundnut Oil	23
37	Central African Republic	2006	Honey	10
37	Central African Republic	2006	Lemons, Limes	1
37	Central African Republic	2006	Maize	79
37	Central African Republic	2006	Meat, Other	17
37	Central African Republic	2006	Milk, Whole	45
37	Central African Republic	2006	Millet	7
37	Central African Republic	2006	Mutton & Goat Meat	10
37	Central African Republic	2006	Offals, Edible	9
37	Central African Republic	2006	Oilcrops Oil, Other	1
37	Central African Republic	2006	Oilcrops, Other	14
37	Central African Republic	2006	Oranges, Mandarines	13
37	Central African Republic	2006	Palm Oil	1
37	Central African Republic	2006	Peas	1
37	Central African Republic	2006	Pelagic Fish	1
37	Central African Republic	2006	Pigmeat	9
37	Central African Republic	2006	Pineapples	9
37	Central African Republic	2006	Plantains	24
37	Central African Republic	2006	Poultry Meat	3
37	Central African Republic	2006	Pulses, Other	16
37	Central African Republic	2006	Rice (Milled Equivalent)	18
37	Central African Republic	2006	Roots, Other	65
37	Central African Republic	2006	Sesameseed	14
37	Central African Republic	2006	Sesameseed Oil	5
37	Central African Republic	2006	Sorghum	26
37	Central African Republic	2006	Sugar, Refined Equivalent	13
37	Central African Republic	2006	Vegetables, Other	61
37	Central African Republic	2006	Wheat	29
37	Central African Republic	2006	Yams	214
37	Central African Republic	2007	Bananas	72
37	Central African Republic	2007	Beer	11
37	Central African Republic	2007	Beverages, Fermented	44
37	Central African Republic	2007	Bovine Meat	56
37	Central African Republic	2007	Cassava	387
37	Central African Republic	2007	Cereals, Other	1
37	Central African Republic	2007	Coffee	1
37	Central African Republic	2007	Eggs	1
37	Central African Republic	2007	Fats, Animals, Raw	3
37	Central African Republic	2007	Freshwater Fish	19

FAO country code	Country	Reference year	Food item	Gross food supply (g *capita*$^{-1}$ d^{-1})
37	Central African Republic	2007	Fruits, Other	9
37	Central African Republic	2007	Grapefruit	3
37	Central African Republic	2007	Groundnut Oil	25
37	Central African Republic	2007	Honey	9
37	Central African Republic	2007	Lemons, Limes	1
37	Central African Republic	2007	Maize	83
37	Central African Republic	2007	Meat, Other	17
37	Central African Republic	2007	Milk, Whole	47
37	Central African Republic	2007	Millet	6
37	Central African Republic	2007	Mutton & Goat Meat	10
37	Central African Republic	2007	Offals, Edible	9
37	Central African Republic	2007	Oilcrops Oil, Other	1
37	Central African Republic	2007	Oilcrops, Other	15
37	Central African Republic	2007	Oranges, Mandarines	13
37	Central African Republic	2007	Palm Oil	3
37	Central African Republic	2007	Peas	1
37	Central African Republic	2007	Pelagic Fish	2
37	Central African Republic	2007	Pigmeat	9
37	Central African Republic	2007	Pineapples	9
37	Central African Republic	2007	Plantains	22
37	Central African Republic	2007	Poultry Meat	3
37	Central African Republic	2007	Pulses, Other	13
37	Central African Republic	2007	Rice (Milled Equivalent)	19
37	Central African Republic	2007	Roots, Other	67
37	Central African Republic	2007	Sesameseed	18
37	Central African Republic	2007	Sesameseed Oil	5
37	Central African Republic	2007	Sorghum	29
37	Central African Republic	2007	Sugar, Refined Equivalent	15
37	Central African Republic	2007	Tomatoes	1
37	Central African Republic	2007	Vegetables, Other	63
37	Central African Republic	2007	Wheat	17
37	Central African Republic	2007	Yams	219
37	Central African Republic	2008	Bananas	74
37	Central African Republic	2008	Beer	14
37	Central African Republic	2008	Beverages, Fermented	45
37	Central African Republic	2008	Bovine Meat	53
37	Central African Republic	2008	Cassava	395
37	Central African Republic	2008	Coffee	2
37	Central African Republic	2008	Eggs	1
37	Central African Republic	2008	Fats, Animals, Raw	3
37	Central African Republic	2008	Freshwater Fish	20
37	Central African Republic	2008	Fruits, Other	9
37	Central African Republic	2008	Grapefruit	3
37	Central African Republic	2008	Groundnut Oil	25
37	Central African Republic	2008	Honey	9
37	Central African Republic	2008	Lemons, Limes	1
37	Central African Republic	2008	Maize	86
37	Central African Republic	2008	Meat, Other	16
37	Central African Republic	2008	Milk, Whole	46
37	Central African Republic	2008	Millet	6
37	Central African Republic	2008	Mutton & Goat Meat	12
37	Central African Republic	2008	Offals, Edible	9
37	Central African Republic	2008	Oilcrops Oil, Other	1
37	Central African Republic	2008	Oilcrops, Other	15
37	Central African Republic	2008	Oranges, Mandarines	13
37	Central African Republic	2008	Palm Oil	5
37	Central African Republic	2008	Peas	1
37	Central African Republic	2008	Pelagic Fish	2
37	Central African Republic	2008	Pigmeat	11
37	Central African Republic	2008	Pineapples	8
37	Central African Republic	2008	Plantains	22
37	Central African Republic	2008	Poultry Meat	3
37	Central African Republic	2008	Pulses, Other	17
37	Central African Republic	2008	Rice (Milled Equivalent)	20
37	Central African Republic	2008	Roots, Other	68
37	Central African Republic	2008	Sesameseed	18
37	Central African Republic	2008	Sesameseed Oil	5
37	Central African Republic	2008	Sorghum	23
37	Central African Republic	2008	Sugar, Refined Equivalent	15
37	Central African Republic	2008	Vegetables, Other	62
37	Central African Republic	2008	Wheat	18
37	Central African Republic	2008	Yams	224
37	Central African Republic	2009	Bananas	72
37	Central African Republic	2009	Beer	11
37	Central African Republic	2009	Beverages, Fermented	42
37	Central African Republic	2009	Bovine Meat	53
37	Central African Republic	2009	Cassava	401
37	Central African Republic	2009	Cereals, Other	1
37	Central African Republic	2009	Coffee	1
37	Central African Republic	2009	Eggs	1
37	Central African Republic	2009	Fats, Animals, Raw	3
37	Central African Republic	2009	Freshwater Fish	21
37	Central African Republic	2009	Fruits, Other	8
37	Central African Republic	2009	Grapefruit	3
37	Central African Republic	2009	Groundnut Oil	23
37	Central African Republic	2009	Honey	9
37	Central African Republic	2009	Lemons, Limes	1
37	Central African Republic	2009	Maize	90
37	Central African Republic	2009	Marine Fish, Other	1
37	Central African Republic	2009	Meat, Other	16
37	Central African Republic	2009	Milk, Whole	46
37	Central African Republic	2009	Millet	6
37	Central African Republic	2009	Mutton & Goat Meat	12
37	Central African Republic	2009	Offals, Edible	9
37	Central African Republic	2009	Oilcrops Oil, Other	1
37	Central African Republic	2009	Oilcrops, Other	15
37	Central African Republic	2009	Oranges, Mandarines	13

FAO countrycode	Country	Reference year	Food item	Gross food supply (g *capita*$^{-1}$ d^{-1})
37	Central African Republic	2009	Palm Oil	4
37	Central African Republic	2009	Peas	1
37	Central African Republic	2009	Pelagic Fish	1
37	Central African Republic	2009	Pigmeat	11
37	Central African Republic	2009	Pineapples	8
37	Central African Republic	2009	Plantains	22
37	Central African Republic	2009	Poultry Meat	4
37	Central African Republic	2009	Pulses, Other	18
37	Central African Republic	2009	Rice (Milled Equivalent)	17
37	Central African Republic	2009	Roots, Other	69
37	Central African Republic	2009	Sesameseed	15
37	Central African Republic	2009	Sesameseed Oil	5
37	Central African Republic	2009	Sorghum	28
37	Central African Republic	2009	Soyabean Oil	1
37	Central African Republic	2009	Sugar, Refined Equivalent	14
37	Central African Republic	2009	Vegetables, Other	62
37	Central African Republic	2009	Wheat	20
37	Central African Republic	2009	Yams	227
37	Central African Republic	2010	Bananas	71
37	Central African Republic	2010	Beer	10
37	Central African Republic	2010	Beverages, Fermented	48
37	Central African Republic	2010	Bovine Meat	54
37	Central African Republic	2010	Cassava	415
37	Central African Republic	2010	Coffee	2
37	Central African Republic	2010	Eggs	2
37	Central African Republic	2010	Fats, Animals, Raw	3
37	Central African Republic	2010	Freshwater Fish	22
37	Central African Republic	2010	Fruits, Other	9
37	Central African Republic	2010	Grapefruit	3
37	Central African Republic	2010	Groundnut Oil	23
37	Central African Republic	2010	Honey	9
37	Central African Republic	2010	Lemons, Limes	1
37	Central African Republic	2010	Maize	96
37	Central African Republic	2010	Marine Fish, Other	2
37	Central African Republic	2010	Meat, Other	20
37	Central African Republic	2010	Milk, Whole	46
37	Central African Republic	2010	Millet	6
37	Central African Republic	2010	Mutton & Goat Meat	13
37	Central African Republic	2010	Offals, Edible	9
37	Central African Republic	2010	Oilcrops, Other	16
37	Central African Republic	2010	Oranges, Mandarines	14
37	Central African Republic	2010	Palm Oil	4
37	Central African Republic	2010	Peas	2
37	Central African Republic	2010	Pelagic Fish	2
37	Central African Republic	2010	Pigmeat	12
37	Central African Republic	2010	Pineapples	8
37	Central African Republic	2010	Plantains	22
37	Central African Republic	2010	Poultry Meat	4
37	Central African Republic	2010	Pulses, Other	21
37	Central African Republic	2010	Rice (Milled Equivalent)	19
37	Central African Republic	2010	Roots, Other	71
37	Central African Republic	2010	Sesameseed	8
37	Central African Republic	2010	Sesameseed Oil	5
37	Central African Republic	2010	Sorghum	28
37	Central African Republic	2010	Soyabean Oil	1
37	Central African Republic	2010	Sugar, Refined Equivalent	13
37	Central African Republic	2010	Tomatoes	1
37	Central African Republic	2010	Vegetables, Other	53
37	Central African Republic	2010	Wheat	23
37	Central African Republic	2010	Yams	233
37	Central African Republic	2011	Bananas	69
37	Central African Republic	2011	Beer	11
37	Central African Republic	2011	Beverages, Fermented	47
37	Central African Republic	2011	Bovine Meat	54
37	Central African Republic	2011	Cassava	423
37	Central African Republic	2011	Coffee	3
37	Central African Republic	2011	Eggs	2
37	Central African Republic	2011	Fats, Animals, Raw	3
37	Central African Republic	2011	Freshwater Fish	22
37	Central African Republic	2011	Fruits, Other	10
37	Central African Republic	2011	Grapefruit	3
37	Central African Republic	2011	Groundnut Oil	24
37	Central African Republic	2011	Honey	10
37	Central African Republic	2011	Lemons, Limes	1
37	Central African Republic	2011	Maize	94
37	Central African Republic	2011	Marine Fish, Other	2
37	Central African Republic	2011	Meat, Other	20
37	Central African Republic	2011	Milk, Whole	46
37	Central African Republic	2011	Millet	6
37	Central African Republic	2011	Mutton & Goat Meat	15
37	Central African Republic	2011	Offals, Edible	9
37	Central African Republic	2011	Oilcrops, Other	15
37	Central African Republic	2011	Oranges, Mandarines	17
37	Central African Republic	2011	Palm Oil	3
37	Central African Republic	2011	Peas	1
37	Central African Republic	2011	Pelagic Fish	2
37	Central African Republic	2011	Pigmeat	11
37	Central African Republic	2011	Pineapples	8
37	Central African Republic	2011	Plantains	21
37	Central African Republic	2011	Poultry Meat	4
37	Central African Republic	2011	Pulses, Other	22
37	Central African Republic	2011	Rice (Milled Equivalent)	18
37	Central African Republic	2011	Roots, Other	72
37	Central African Republic	2011	Sesameseed	6
37	Central African Republic	2011	Sesameseed Oil	5
37	Central African Republic	2011	Sorghum	26
37	Central African Republic	2011	Sugar, Refined Equivalent	13

FAO country code	Country	Reference year	Food item	Gross food supply (g *capita*$^{-1}$ d^{-1})
37	Central African Republic	2011	Tomatoes	1
37	Central African Republic	2011	Vegetables, Other	53
37	Central African Republic	2011	Wheat	26
37	Central African Republic	2011	Yams	237
39	Chad	1992	Beans	5
39	Chad	1992	Beer	6
39	Chad	1992	Beverages, Fermented	4
39	Chad	1992	Bovine Meat	34
39	Chad	1992	Cassava	76
39	Chad	1992	Cereals, Other	36
39	Chad	1992	Cottonseed Oil	4
39	Chad	1992	Dates	10
39	Chad	1992	Eggs	1
39	Chad	1992	Fats, Animals, Raw	1
39	Chad	1992	Freshwater Fish	15
39	Chad	1992	Fruits, Other	35
39	Chad	1992	Groundnut Oil	7
39	Chad	1992	Groundnuts (Shelled Eq)	35
39	Chad	1992	Maize	31
39	Chad	1992	Meat, Other	2
39	Chad	1992	Milk, Whole	65
39	Chad	1992	Millet	92
39	Chad	1992	Mutton & Goat Meat	9
39	Chad	1992	Offals, Edible	7
39	Chad	1992	Oilcrops, Other	7
39	Chad	1992	Onions	6
39	Chad	1992	Potatoes	7
39	Chad	1992	Poultry Meat	2
39	Chad	1992	Pulses, Other	8
39	Chad	1992	Rice (Milled Equivalent)	27
39	Chad	1992	Roots, Other	11
39	Chad	1992	Sesameseed	6
39	Chad	1992	Sorghum	125
39	Chad	1992	Sugar, Refined Equivalent	13
39	Chad	1992	Sweet Potatoes	17
39	Chad	1992	Vegetables, Other	24
39	Chad	1992	Wheat	18
39	Chad	1992	Yams	80
39	Chad	1993	Beans	3
39	Chad	1993	Beer	4
39	Chad	1993	Beverages, Fermented	3
39	Chad	1993	Bovine Meat	29
39	Chad	1993	Cassava	72
39	Chad	1993	Cereals, Other	36
39	Chad	1993	Cottonseed Oil	3
39	Chad	1993	Dates	7
39	Chad	1993	Eggs	1
39	Chad	1993	Fats, Animals, Raw	1
39	Chad	1993	Freshwater Fish	16
39	Chad	1993	Fruits, Other	35
39	Chad	1993	Groundnut Oil	7
39	Chad	1993	Groundnuts (Shelled Eq)	37
39	Chad	1993	Maize	20
39	Chad	1993	Meat, Other	2
39	Chad	1993	Milk, Whole	66
39	Chad	1993	Millet	83
39	Chad	1993	Mutton & Goat Meat	9
39	Chad	1993	Offals, Edible	6
39	Chad	1993	Oilcrops, Other	5
39	Chad	1993	Onions	5
39	Chad	1993	Potatoes	6
39	Chad	1993	Poultry Meat	2
39	Chad	1993	Pulses, Other	8
39	Chad	1993	Rice (Milled Equivalent)	27
39	Chad	1993	Roots, Other	11
39	Chad	1993	Sesameseed	4
39	Chad	1993	Sorghum	103
39	Chad	1993	Sugar, Refined Equivalent	15
39	Chad	1993	Sweet Potatoes	16
39	Chad	1993	Vegetables, Other	23
39	Chad	1993	Wheat	19
39	Chad	1993	Yams	70
39	Chad	1994	Beans	8
39	Chad	1994	Beer	4
39	Chad	1994	Beverages, Fermented	4
39	Chad	1994	Bovine Meat	23
39	Chad	1994	Cassava	67
39	Chad	1994	Cereals, Other	44
39	Chad	1994	Cottonseed Oil	4
39	Chad	1994	Dates	7
39	Chad	1994	Eggs	1
39	Chad	1994	Fats, Animals, Raw	1
39	Chad	1994	Freshwater Fish	17
39	Chad	1994	Fruits, Other	35
39	Chad	1994	Groundnut Oil	6
39	Chad	1994	Groundnuts (Shelled Eq)	31
39	Chad	1994	Maize	55
39	Chad	1994	Meat, Other	2
39	Chad	1994	Milk, Whole	63
39	Chad	1994	Millet	99
39	Chad	1994	Mutton & Goat Meat	9
39	Chad	1994	Offals, Edible	5
39	Chad	1994	Oilcrops, Other	7
39	Chad	1994	Onions	5
39	Chad	1994	Potatoes	6
39	Chad	1994	Poultry Meat	2
39	Chad	1994	Pulses, Other	7

FAO country code	Country	Reference year	Food item	Gross food supply (g $capita^{-1}$ d^{-1})
39	Chad	1994	Rice (Milled Equivalent)	27
39	Chad	1994	Roots, Other	12
39	Chad	1994	Sesameseed	5
39	Chad	1994	Sorghum	164
39	Chad	1994	Sugar, Refined Equivalent	14
39	Chad	1994	Sweet Potatoes	15
39	Chad	1994	Tea	1
39	Chad	1994	Vegetables, Other	22
39	Chad	1994	Wheat	17
39	Chad	1994	Yams	78
39	Chad	1995	Beans	7
39	Chad	1995	Beer	3
39	Chad	1995	Beverages, Fermented	3
39	Chad	1995	Bovine Meat	25
39	Chad	1995	Cassava	95
39	Chad	1995	Cereals, Other	33
39	Chad	1995	Cottonseed Oil	4
39	Chad	1995	Dates	5
39	Chad	1995	Eggs	1
39	Chad	1995	Fats, Animals, Raw	1
39	Chad	1995	Freshwater Fish	17
39	Chad	1995	Fruits, Other	26
39	Chad	1995	Groundnut Oil	9
39	Chad	1995	Groundnuts (Shelled Eq)	45
39	Chad	1995	Maize	21
39	Chad	1995	Meat, Other	2
39	Chad	1995	Milk, Whole	68
39	Chad	1995	Millet	78
39	Chad	1995	Mutton & Goat Meat	9
39	Chad	1995	Offals, Edible	6
39	Chad	1995	Oilcrops, Other	8
39	Chad	1995	Onions	5
39	Chad	1995	Potatoes	6
39	Chad	1995	Poultry Meat	2
39	Chad	1995	Pulses, Other	6
39	Chad	1995	Rice (Milled Equivalent)	25
39	Chad	1995	Roots, Other	12
39	Chad	1995	Sesameseed	4
39	Chad	1995	Sorghum	133
39	Chad	1995	Sugar, Refined Equivalent	17
39	Chad	1995	Sweet Potatoes	18
39	Chad	1995	Vegetables, Other	31
39	Chad	1995	Wheat	13
39	Chad	1995	Yams	68
39	Chad	1996	Beans	6
39	Chad	1996	Beer	7
39	Chad	1996	Beverages, Fermented	3
39	Chad	1996	Bovine Meat	25
39	Chad	1996	Cassava	94
39	Chad	1996	Cereals, Other	30
39	Chad	1996	Cottonseed Oil	4
39	Chad	1996	Dates	5
39	Chad	1996	Eggs	1
39	Chad	1996	Fats, Animals, Raw	1
39	Chad	1996	Freshwater Fish	18
39	Chad	1996	Fruits, Other	25
39	Chad	1996	Groundnut Oil	9
39	Chad	1996	Groundnuts (Shelled Eq)	44
39	Chad	1996	Maize	27
39	Chad	1996	Marine Fish, Other	1
39	Chad	1996	Meat, Other	2
39	Chad	1996	Milk, Whole	65
39	Chad	1996	Millet	82
39	Chad	1996	Mutton & Goat Meat	9
39	Chad	1996	Offals, Edible	6
39	Chad	1996	Oilcrops, Other	8
39	Chad	1996	Onions	5
39	Chad	1996	Potatoes	6
39	Chad	1996	Poultry Meat	2
39	Chad	1996	Pulses, Other	8
39	Chad	1996	Rice (Milled Equivalent)	23
39	Chad	1996	Roots, Other	12
39	Chad	1996	Sesameseed	4
39	Chad	1996	Sorghum	121
39	Chad	1996	Sugar, Refined Equivalent	22
39	Chad	1996	Sweet Potatoes	18
39	Chad	1996	Tea	1
39	Chad	1996	Vegetables, Other	30
39	Chad	1996	Wheat	16
39	Chad	1996	Yams	66
39	Chad	1997	Beans	6
39	Chad	1997	Beer	5
39	Chad	1997	Beverages, Fermented	3
39	Chad	1997	Bovine Meat	26
39	Chad	1997	Cassava	93
39	Chad	1997	Cereals, Other	29
39	Chad	1997	Cottonseed Oil	5
39	Chad	1997	Dates	4
39	Chad	1997	Eggs	1
39	Chad	1997	Fats, Animals, Raw	1
39	Chad	1997	Freshwater Fish	17
39	Chad	1997	Fruits, Other	30
39	Chad	1997	Groundnut Oil	10
39	Chad	1997	Groundnuts (Shelled Eq)	50
39	Chad	1997	Maize	31
39	Chad	1997	Meat, Other	2
39	Chad	1997	Milk, Whole	69

FAO countrycode	Country	Reference year	Food item	Gross food supply (g *capita*$^{-1}$ d^{-1})
39	Chad	1997	Millet	74
39	Chad	1997	Mutton & Goat Meat	11
39	Chad	1997	Offals, Edible	6
39	Chad	1997	Oilcrops, Other	10
39	Chad	1997	Onions	5
39	Chad	1997	Potatoes	6
39	Chad	1997	Poultry Meat	2
39	Chad	1997	Pulses, Other	7
39	Chad	1997	Rice (Milled Equivalent)	26
39	Chad	1997	Roots, Other	12
39	Chad	1997	Sesameseed	8
39	Chad	1997	Sorghum	131
39	Chad	1997	Sugar, Refined Equivalent	16
39	Chad	1997	Sweet Potatoes	18
39	Chad	1997	Vegetables, Other	29
39	Chad	1997	Wheat	19
39	Chad	1997	Yams	63
39	Chad	1998	Beans	11
39	Chad	1998	Beer	4
39	Chad	1998	Beverages, Fermented	4
39	Chad	1998	Bovine Meat	28
39	Chad	1998	Cassava	93
39	Chad	1998	Cereals, Other	41
39	Chad	1998	Cottonseed Oil	5
39	Chad	1998	Dates	4
39	Chad	1998	Eggs	1
39	Chad	1998	Fats, Animals, Raw	1
39	Chad	1998	Freshwater Fish	18
39	Chad	1998	Fruits, Other	29
39	Chad	1998	Groundnut Oil	10
39	Chad	1998	Groundnuts (Shelled Eq)	48
39	Chad	1998	Maize	48
39	Chad	1998	Meat, Other	2
39	Chad	1998	Milk, Whole	68
39	Chad	1998	Millet	87
39	Chad	1998	Mutton & Goat Meat	10
39	Chad	1998	Offals, Edible	7
39	Chad	1998	Oilcrops, Other	9
39	Chad	1998	Onions	4
39	Chad	1998	Potatoes	6
39	Chad	1998	Poultry Meat	2
39	Chad	1998	Pulses, Other	17
39	Chad	1998	Rice (Milled Equivalent)	26
39	Chad	1998	Roots, Other	11
39	Chad	1998	Sesameseed	9
39	Chad	1998	Sorghum	126
39	Chad	1998	Sugar, Refined Equivalent	20
39	Chad	1998	Sweet Potatoes	18
39	Chad	1998	Vegetables, Other	26
39	Chad	1998	Wheat	14
39	Chad	1998	Yams	65
39	Chad	1999	Beans	13
39	Chad	1999	Beer	3
39	Chad	1999	Beverages, Fermented	4
39	Chad	1999	Bovine Meat	27
39	Chad	1999	Cassava	100
39	Chad	1999	Cereals, Other	52
39	Chad	1999	Cottonseed Oil	4
39	Chad	1999	Dates	4
39	Chad	1999	Eggs	1
39	Chad	1999	Fats, Animals, Raw	1
39	Chad	1999	Freshwater Fish	19
39	Chad	1999	Fruits, Other	26
39	Chad	1999	Groundnut Oil	11
39	Chad	1999	Groundnuts (Shelled Eq)	54
39	Chad	1999	Maize	18
39	Chad	1999	Meat, Other	2
39	Chad	1999	Milk, Whole	69
39	Chad	1999	Millet	93
39	Chad	1999	Mutton & Goat Meat	10
39	Chad	1999	Offals, Edible	7
39	Chad	1999	Oilcrops, Other	8
39	Chad	1999	Onions	6
39	Chad	1999	Potatoes	7
39	Chad	1999	Poultry Meat	2
39	Chad	1999	Pulses, Other	11
39	Chad	1999	Rice (Milled Equivalent)	22
39	Chad	1999	Roots, Other	14
39	Chad	1999	Sesameseed	6
39	Chad	1999	Sorghum	127
39	Chad	1999	Sugar, Refined Equivalent	15
39	Chad	1999	Sweet Potatoes	17
39	Chad	1999	Vegetables, Other	25
39	Chad	1999	Wheat	17
39	Chad	1999	Yams	63
39	Chad	2000	Beans	18
39	Chad	2000	Beer	3
39	Chad	2000	Beverages, Fermented	3
39	Chad	2000	Bovine Meat	24
39	Chad	2000	Cassava	76
39	Chad	2000	Cereals, Other	34
39	Chad	2000	Cottonseed Oil	4
39	Chad	2000	Dates	4
39	Chad	2000	Eggs	1
39	Chad	2000	Fats, Animals, Raw	1
39	Chad	2000	Freshwater Fish	24
39	Chad	2000	Fruits, Other	25

FAO country code	Country	Reference year	Food item	Gross food supply (g $capita^{-1}$ d^{-1})
39	Chad	2000	Groundnut Oil	6
39	Chad	2000	Groundnuts (Shelled Eq)	56
39	Chad	2000	Maize	28
39	Chad	2000	Meat, Other	2
39	Chad	2000	Milk, Whole	68
39	Chad	2000	Millet	91
39	Chad	2000	Mutton & Goat Meat	10
39	Chad	2000	Offals, Edible	7
39	Chad	2000	Oilcrops, Other	7
39	Chad	2000	Onions	4
39	Chad	2000	Potatoes	5
39	Chad	2000	Poultry Meat	2
39	Chad	2000	Pulses, Other	6
39	Chad	2000	Rice (Milled Equivalent)	24
39	Chad	2000	Roots, Other	13
39	Chad	2000	Sesameseed	9
39	Chad	2000	Sorghum	135
39	Chad	2000	Sugar, Refined Equivalent	18
39	Chad	2000	Sweet Potatoes	13
39	Chad	2000	Vegetables, Other	25
39	Chad	2000	Wheat	17
39	Chad	2000	Yams	48
39	Chad	2001	Beans	21
39	Chad	2001	Beer	3
39	Chad	2001	Beverages, Fermented	4
39	Chad	2001	Bovine Meat	24
39	Chad	2001	Cassava	88
39	Chad	2001	Cereals, Other	58
39	Chad	2001	Cottonseed Oil	4
39	Chad	2001	Dates	4
39	Chad	2001	Eggs	1
39	Chad	2001	Fats, Animals, Raw	1
39	Chad	2001	Freshwater Fish	22
39	Chad	2001	Fruits, Other	24
39	Chad	2001	Groundnut Oil	9
39	Chad	2001	Groundnuts (Shelled Eq)	55
39	Chad	2001	Maize	22
39	Chad	2001	Meat, Other	2
39	Chad	2001	Milk, Whole	67
39	Chad	2001	Millet	90
39	Chad	2001	Mutton & Goat Meat	10
39	Chad	2001	Offals, Edible	7
39	Chad	2001	Oilcrops, Other	8
39	Chad	2001	Onions	6
39	Chad	2001	Potatoes	6
39	Chad	2001	Poultry Meat	1
39	Chad	2001	Pulses, Other	11
39	Chad	2001	Rice (Milled Equivalent)	22
39	Chad	2001	Roots, Other	13
39	Chad	2001	Sesameseed	9
39	Chad	2001	Sorghum	129
39	Chad	2001	Sugar, Refined Equivalent	18
39	Chad	2001	Sweet Potatoes	16
39	Chad	2001	Vegetables, Other	24
39	Chad	2001	Wheat	16
39	Chad	2001	Yams	55
39	Chad	2002	Beans	19
39	Chad	2002	Beer	2
39	Chad	2002	Beverages, Fermented	4
39	Chad	2002	Bovine Meat	23
39	Chad	2002	Cassava	89
39	Chad	2002	Cereals, Other	38
39	Chad	2002	Cottonseed Oil	4
39	Chad	2002	Dates	4
39	Chad	2002	Eggs	1
39	Chad	2002	Fats, Animals, Raw	1
39	Chad	2002	Freshwater Fish	20
39	Chad	2002	Fruits, Other	24
39	Chad	2002	Groundnut Oil	9
39	Chad	2002	Groundnuts (Shelled Eq)	54
39	Chad	2002	Maize	22
39	Chad	2002	Meat, Other	2
39	Chad	2002	Milk, Whole	65
39	Chad	2002	Millet	90
39	Chad	2002	Mutton & Goat Meat	10
39	Chad	2002	Offals, Edible	6
39	Chad	2002	Oilcrops, Other	8
39	Chad	2002	Onions	7
39	Chad	2002	Potatoes	6
39	Chad	2002	Poultry Meat	1
39	Chad	2002	Pulses, Other	11
39	Chad	2002	Rice (Milled Equivalent)	25
39	Chad	2002	Roots, Other	13
39	Chad	2002	Sesameseed	12
39	Chad	2002	Sorghum	123
39	Chad	2002	Sugar, Refined Equivalent	19
39	Chad	2002	Sweet Potatoes	16
39	Chad	2002	Vegetables, Other	24
39	Chad	2002	Wheat	18
39	Chad	2002	Yams	56
39	Chad	2003	Beans	19
39	Chad	2003	Beer	3
39	Chad	2003	Beverages, Fermented	5
39	Chad	2003	Bovine Meat	23
39	Chad	2003	Cassava	86
39	Chad	2003	Cereals, Other	73
39	Chad	2003	Cottonseed Oil	4

FAO countrycode	Country	Reference year	Food item	Gross food supply (g *capita*$^{-1}$ d^{-1})
39	Chad	2003	Dates	4
39	Chad	2003	Eggs	1
39	Chad	2003	Fats, Animals, Raw	1
39	Chad	2003	Freshwater Fish	18
39	Chad	2003	Fruits, Other	24
39	Chad	2003	Groundnut Oil	8
39	Chad	2003	Groundnuts (Shelled Eq)	52
39	Chad	2003	Maize	23
39	Chad	2003	Meat, Other	2
39	Chad	2003	Milk, Whole	67
39	Chad	2003	Millet	99
39	Chad	2003	Mutton & Goat Meat	10
39	Chad	2003	Offals, Edible	6
39	Chad	2003	Oilcrops, Other	7
39	Chad	2003	Onions	6
39	Chad	2003	Potatoes	6
39	Chad	2003	Poultry Meat	1
39	Chad	2003	Pulses, Other	11
39	Chad	2003	Rice (Milled Equivalent)	19
39	Chad	2003	Roots, Other	13
39	Chad	2003	Sesameseed	9
39	Chad	2003	Sorghum	123
39	Chad	2003	Sugar, Refined Equivalent	18
39	Chad	2003	Sweet Potatoes	18
39	Chad	2003	Vegetables, Other	24
39	Chad	2003	Wheat	19
39	Chad	2003	Yams	55
39	Chad	2004	Beans	18
39	Chad	2004	Beer	3
39	Chad	2004	Beverages, Fermented	3
39	Chad	2004	Bovine Meat	23
39	Chad	2004	Cassava	84
39	Chad	2004	Cereals, Other	65
39	Chad	2004	Cottonseed Oil	4
39	Chad	2004	Dates	3
39	Chad	2004	Eggs	1
39	Chad	2004	Fats, Animals, Raw	1
39	Chad	2004	Freshwater Fish	17
39	Chad	2004	Fruits, Other	23
39	Chad	2004	Groundnut Oil	8
39	Chad	2004	Groundnuts (Shelled Eq)	50
39	Chad	2004	Maize	32
39	Chad	2004	Meat, Other	2
39	Chad	2004	Milk, Whole	63
39	Chad	2004	Millet	99
39	Chad	2004	Mutton & Goat Meat	10
39	Chad	2004	Offals, Edible	6
39	Chad	2004	Oilcrops Oil, Other	1
39	Chad	2004	Oilcrops, Other	8
39	Chad	2004	Onions	6
39	Chad	2004	Potatoes	6
39	Chad	2004	Poultry Meat	1
39	Chad	2004	Pulses, Other	11
39	Chad	2004	Rice (Milled Equivalent)	19
39	Chad	2004	Roots, Other	13
39	Chad	2004	Sesameseed	9
39	Chad	2004	Sorghum	129
39	Chad	2004	Sugar, Refined Equivalent	21
39	Chad	2004	Sweet Potatoes	19
39	Chad	2004	Vegetables, Other	23
39	Chad	2004	Wheat	12
39	Chad	2004	Yams	54
39	Chad	2005	Beans	21
39	Chad	2005	Beer	3
39	Chad	2005	Beverages, Fermented	5
39	Chad	2005	Bovine Meat	22
39	Chad	2005	Cassava	74
39	Chad	2005	Cereals, Other	79
39	Chad	2005	Cottonseed Oil	3
39	Chad	2005	Dates	3
39	Chad	2005	Eggs	1
39	Chad	2005	Fats, Animals, Raw	1
39	Chad	2005	Freshwater Fish	15
39	Chad	2005	Fruits, Other	23
39	Chad	2005	Groundnut Oil	8
39	Chad	2005	Groundnuts (Shelled Eq)	48
39	Chad	2005	Maize	32
39	Chad	2005	Meat, Other	1
39	Chad	2005	Milk, Whole	62
39	Chad	2005	Millet	102
39	Chad	2005	Mutton & Goat Meat	10
39	Chad	2005	Offals, Edible	6
39	Chad	2005	Oilcrops Oil, Other	1
39	Chad	2005	Oilcrops, Other	8
39	Chad	2005	Onions	6
39	Chad	2005	Potatoes	7
39	Chad	2005	Poultry Meat	1
39	Chad	2005	Pulses, Other	12
39	Chad	2005	Rice (Milled Equivalent)	21
39	Chad	2005	Roots, Other	8
39	Chad	2005	Sesameseed	8
39	Chad	2005	Sorghum	113
39	Chad	2005	Sugar, Refined Equivalent	23
39	Chad	2005	Sweet Potatoes	20
39	Chad	2005	Vegetables, Other	23
39	Chad	2005	Wheat	17
39	Chad	2005	Yams	55

FAO country code	Country	Reference year	Food item	Gross food supply (g *capita*$^{-1}$ d^{-1})
39	Chad	2006	Beans	20
39	Chad	2006	Beer	8
39	Chad	2006	Beverages, Fermented	5
39	Chad	2006	Bovine Meat	22
39	Chad	2006	Cassava	67
39	Chad	2006	Cereals, Other	76
39	Chad	2006	Cottonseed Oil	2
39	Chad	2006	Dates	4
39	Chad	2006	Eggs	1
39	Chad	2006	Fats, Animals, Raw	1
39	Chad	2006	Freshwater Fish	13
39	Chad	2006	Fruits, Other	24
39	Chad	2006	Groundnut Oil	8
39	Chad	2006	Groundnuts (Shelled Eq)	47
39	Chad	2006	Maize	32
39	Chad	2006	Meat, Other	1
39	Chad	2006	Milk, Whole	63
39	Chad	2006	Millet	102
39	Chad	2006	Mutton & Goat Meat	10
39	Chad	2006	Offals, Edible	6
39	Chad	2006	Oilcrops, Other	6
39	Chad	2006	Onions	8
39	Chad	2006	Potatoes	9
39	Chad	2006	Poultry Meat	1
39	Chad	2006	Pulses, Other	11
39	Chad	2006	Rice (Milled Equivalent)	17
39	Chad	2006	Roots, Other	7
39	Chad	2006	Sesameseed	8
39	Chad	2006	Sorghum	123
39	Chad	2006	Sugar, Refined Equivalent	23
39	Chad	2006	Sweet Potatoes	20
39	Chad	2006	Vegetables, Other	27
39	Chad	2006	Wheat	21
39	Chad	2006	Yams	58
39	Chad	2007	Beans	14
39	Chad	2007	Beer	7
39	Chad	2007	Beverages, Fermented	4
39	Chad	2007	Bovine Meat	22
39	Chad	2007	Cassava	58
39	Chad	2007	Cereals, Other	96
39	Chad	2007	Cottonseed Oil	2
39	Chad	2007	Dates	4
39	Chad	2007	Eggs	1
39	Chad	2007	Fats, Animals, Raw	1
39	Chad	2007	Freshwater Fish	11
39	Chad	2007	Fruits, Other	24
39	Chad	2007	Groundnut Oil	7
39	Chad	2007	Groundnuts (Shelled Eq)	32
39	Chad	2007	Maize	32
39	Chad	2007	Meat, Other	1
39	Chad	2007	Milk, Whole	62
39	Chad	2007	Millet	105
39	Chad	2007	Mutton & Goat Meat	10
39	Chad	2007	Offals, Edible	6
39	Chad	2007	Oilcrops Oil, Other	1
39	Chad	2007	Oilcrops, Other	4
39	Chad	2007	Onions	4
39	Chad	2007	Potatoes	14
39	Chad	2007	Poultry Meat	1
39	Chad	2007	Pulses, Other	11
39	Chad	2007	Rice (Milled Equivalent)	19
39	Chad	2007	Roots, Other	6
39	Chad	2007	Sesameseed	9
39	Chad	2007	Sorghum	133
39	Chad	2007	Sugar, Refined Equivalent	23
39	Chad	2007	Sweet Potatoes	13
39	Chad	2007	Vegetables, Other	19
39	Chad	2007	Wheat	26
39	Chad	2007	Yams	66
39	Chad	2008	Beans	14
39	Chad	2008	Beer	8
39	Chad	2008	Beverages, Fermented	4
39	Chad	2008	Bovine Meat	22
39	Chad	2008	Cassava	36
39	Chad	2008	Cereals, Other	87
39	Chad	2008	Cottonseed Oil	2
39	Chad	2008	Dates	4
39	Chad	2008	Eggs	1
39	Chad	2008	Fats, Animals, Raw	1
39	Chad	2008	Freshwater Fish	10
39	Chad	2008	Fruits, Other	23
39	Chad	2008	Groundnut Oil	8
39	Chad	2008	Groundnuts (Shelled Eq)	39
39	Chad	2008	Maize	32
39	Chad	2008	Meat, Other	1
39	Chad	2008	Milk, Whole	61
39	Chad	2008	Millet	113
39	Chad	2008	Mutton & Goat Meat	10
39	Chad	2008	Offals, Edible	6
39	Chad	2008	Oilcrops Oil, Other	1
39	Chad	2008	Oilcrops, Other	4
39	Chad	2008	Onions	4
39	Chad	2008	Potatoes	8
39	Chad	2008	Poultry Meat	1
39	Chad	2008	Pulses, Other	10
39	Chad	2008	Rice (Milled Equivalent)	26
39	Chad	2008	Roots, Other	5

FAO country code	Country	Reference year	Food item	Gross food supply (g $capita^{-1}$ d^{-1})
39	Chad	2008	Sesameseed	8
39	Chad	2008	Sorghum	131
39	Chad	2008	Sugar, Refined Equivalent	20
39	Chad	2008	Sweet Potatoes	15
39	Chad	2008	Vegetables, Other	19
39	Chad	2008	Wheat	26
39	Chad	2008	Yams	65
39	Chad	2009	Beans	16
39	Chad	2009	Beer	8
39	Chad	2009	Beverages, Fermented	5
39	Chad	2009	Bovine Meat	22
39	Chad	2009	Cassava	50
39	Chad	2009	Cereals, Other	95
39	Chad	2009	Cottonseed Oil	1
39	Chad	2009	Dates	4
39	Chad	2009	Eggs	1
39	Chad	2009	Fats, Animals, Raw	1
39	Chad	2009	Freshwater Fish	10
39	Chad	2009	Fruits, Other	21
39	Chad	2009	Groundnut Oil	8
39	Chad	2009	Groundnuts (Shelled Eq)	34
39	Chad	2009	Maize	35
39	Chad	2009	Meat, Other	1
39	Chad	2009	Milk, Whole	61
39	Chad	2009	Millet	104
39	Chad	2009	Mutton & Goat Meat	10
39	Chad	2009	Offals, Edible	6
39	Chad	2009	Oilcrops Oil, Other	1
39	Chad	2009	Oilcrops, Other	4
39	Chad	2009	Onions	4
39	Chad	2009	Potatoes	10
39	Chad	2009	Poultry Meat	1
39	Chad	2009	Pulses, Other	10
39	Chad	2009	Rice (Milled Equivalent)	26
39	Chad	2009	Roots, Other	7
39	Chad	2009	Sesameseed	7
39	Chad	2009	Sorghum	128
39	Chad	2009	Sugar, Refined Equivalent	19
39	Chad	2009	Sweet Potatoes	17
39	Chad	2009	Vegetables, Other	19
39	Chad	2009	Wheat	29
39	Chad	2009	Yams	64
39	Chad	2010	Beans	14
39	Chad	2010	Beer	9
39	Chad	2010	Beverages, Fermented	5
39	Chad	2010	Bovine Meat	22
39	Chad	2010	Cassava	49
39	Chad	2010	Cereals, Other	94
39	Chad	2010	Cottonseed Oil	1
39	Chad	2010	Dates	4
39	Chad	2010	Eggs	1
39	Chad	2010	Fats, Animals, Raw	1
39	Chad	2010	Freshwater Fish	12
39	Chad	2010	Fruits, Other	20
39	Chad	2010	Groundnut Oil	8
39	Chad	2010	Groundnuts (Shelled Eq)	36
39	Chad	2010	Maize	40
39	Chad	2010	Meat, Other	1
39	Chad	2010	Milk, Whole	61
39	Chad	2010	Millet	98
39	Chad	2010	Mutton & Goat Meat	10
39	Chad	2010	Offals, Edible	6
39	Chad	2010	Oilcrops, Other	4
39	Chad	2010	Onions	4
39	Chad	2010	Potatoes	9
39	Chad	2010	Poultry Meat	1
39	Chad	2010	Pulses, Other	10
39	Chad	2010	Rice (Milled Equivalent)	25
39	Chad	2010	Roots, Other	5
39	Chad	2010	Sesameseed	8
39	Chad	2010	Sorghum	135
39	Chad	2010	Sugar, Refined Equivalent	23
39	Chad	2010	Sweet Potatoes	16
39	Chad	2010	Vegetables, Other	17
39	Chad	2010	Wheat	29
39	Chad	2010	Wine	1
39	Chad	2010	Yams	78
39	Chad	2011	Beans	13
39	Chad	2011	Beer	10
39	Chad	2011	Beverages, Fermented	2
39	Chad	2011	Bovine Meat	22
39	Chad	2011	Cassava	47
39	Chad	2011	Cereals, Other	91
39	Chad	2011	Cottonseed Oil	1
39	Chad	2011	Dates	4
39	Chad	2011	Eggs	1
39	Chad	2011	Fats, Animals, Raw	1
39	Chad	2011	Freshwater Fish	14
39	Chad	2011	Fruits, Other	20
39	Chad	2011	Groundnut Oil	8
39	Chad	2011	Groundnuts (Shelled Eq)	37
39	Chad	2011	Maize	42
39	Chad	2011	Meat, Other	1
39	Chad	2011	Milk, Whole	59
39	Chad	2011	Millet	101
39	Chad	2011	Mutton & Goat Meat	9
39	Chad	2011	Offals, Edible	6

FAO countrycode	Country	Reference year	Food item	Gross food supply (g $capita^{-1}$ d^{-1})
39	Chad	2011	Oilcrops, Other	3
39	Chad	2011	Onions	4
39	Chad	2011	Potatoes	9
39	Chad	2011	Poultry Meat	1
39	Chad	2011	Pulses, Other	10
39	Chad	2011	Rice (Milled Equivalent)	25
39	Chad	2011	Roots, Other	5
39	Chad	2011	Sesameseed	7
39	Chad	2011	Sorghum	141
39	Chad	2011	Sugar, Refined Equivalent	25
39	Chad	2011	Sweet Potatoes	15
39	Chad	2011	Vegetables, Other	17
39	Chad	2011	Wheat	26
39	Chad	2011	Yams	73
40	Chile	1992	Apples	25
40	Chile	1992	Bananas	16
40	Chile	1992	Barley	4
40	Chile	1992	Beans	4
40	Chile	1992	Beer	62
40	Chile	1992	Beverages, Alcoholic	4
40	Chile	1992	Bovine Meat	47
40	Chile	1992	Butter, Ghee	2
40	Chile	1992	Cheese	8
40	Chile	1992	Cocoa Beans	1
40	Chile	1992	Coconuts - Incl Copra	1
40	Chile	1992	Coffee	2
40	Chile	1992	Crustaceans	2
40	Chile	1992	Demersal Fish	7
40	Chile	1992	Eggs	17
40	Chile	1992	Fats, Animals, Raw	3
40	Chile	1992	Freshwater Fish	2
40	Chile	1992	Fruits, Other	39
40	Chile	1992	Grapes	15
40	Chile	1992	Groundnuts (Shelled Eq)	1
40	Chile	1992	Honey	1
40	Chile	1992	Lemons, Limes	16
40	Chile	1992	Maize	15
40	Chile	1992	Meat, Other	1
40	Chile	1992	Milk, Whole	187
40	Chile	1992	Molluscs, Other	5
40	Chile	1992	Mutton & Goat Meat	3
40	Chile	1992	Oats	2
40	Chile	1992	Offals, Edible	8
40	Chile	1992	Onions	37
40	Chile	1992	Oranges, Mandarines	19
40	Chile	1992	Peas	2
40	Chile	1992	Pelagic Fish	32
40	Chile	1992	Pigmeat	26
40	Chile	1992	Pineapples	1
40	Chile	1992	Potatoes	170
40	Chile	1992	Poultry Meat	40
40	Chile	1992	Pulses, Other	4
40	Chile	1992	Rape and Mustard Oil	5
40	Chile	1992	Rice (Milled Equivalent)	21
40	Chile	1992	Rye	1
40	Chile	1992	Soyabean Oil	15
40	Chile	1992	Sugar, Refined Equivalent	108
40	Chile	1992	Sunflowerseed Oil	7
40	Chile	1992	Sweet Potatoes	2
40	Chile	1992	Tea	4
40	Chile	1992	Tomatoes	28
40	Chile	1992	Vegetables, Other	183
40	Chile	1992	Wheat	341
40	Chile	1992	Wine	47
40	Chile	1993	Apples	34
40	Chile	1993	Aquatic Animals, Others	1
40	Chile	1993	Bananas	24
40	Chile	1993	Barley	3
40	Chile	1993	Beans	3
40	Chile	1993	Beer	66
40	Chile	1993	Beverages, Alcoholic	4
40	Chile	1993	Bovine Meat	53
40	Chile	1993	Butter, Ghee	2
40	Chile	1993	Cheese	9
40	Chile	1993	Cocoa Beans	2
40	Chile	1993	Coconuts - Incl Copra	1
40	Chile	1993	Coffee	2
40	Chile	1993	Crustaceans	1
40	Chile	1993	Demersal Fish	11
40	Chile	1993	Eggs	17
40	Chile	1993	Fats, Animals, Raw	3
40	Chile	1993	Freshwater Fish	2
40	Chile	1993	Fruits, Other	51
40	Chile	1993	Grapes	17
40	Chile	1993	Groundnuts (Shelled Eq)	1
40	Chile	1993	Honey	1
40	Chile	1993	Lemons, Limes	16
40	Chile	1993	Maize	19
40	Chile	1993	Meat, Other	1
40	Chile	1993	Milk, Whole	195
40	Chile	1993	Molluscs, Other	6
40	Chile	1993	Mutton & Goat Meat	3
40	Chile	1993	Nuts	1
40	Chile	1993	Oats	1
40	Chile	1993	Offals, Edible	9
40	Chile	1993	Onions	39
40	Chile	1993	Oranges, Mandarines	20

FAO country code	Country	Reference year	Food item	Gross food supply (g $capita^{-1}$ d^{-1})
40	Chile	1993	Peas	1
40	Chile	1993	Pelagic Fish	29
40	Chile	1993	Pigmeat	28
40	Chile	1993	Pineapples	2
40	Chile	1993	Potatoes	151
40	Chile	1993	Poultry Meat	47
40	Chile	1993	Pulses, Other	4
40	Chile	1993	Rape and Mustard Oil	2
40	Chile	1993	Rice (Milled Equivalent)	19
40	Chile	1993	Rye	1
40	Chile	1993	Soyabean Oil	16
40	Chile	1993	Sugar, Refined Equivalent	108
40	Chile	1993	Sunflowerseed Oil	8
40	Chile	1993	Sweet Potatoes	1
40	Chile	1993	Tea	4
40	Chile	1993	Tomatoes	68
40	Chile	1993	Vegetables, Other	191
40	Chile	1993	Wheat	329
40	Chile	1993	Wine	44
40	Chile	1994	Apples	32
40	Chile	1994	Aquatic Animals, Others	1
40	Chile	1994	Bananas	27
40	Chile	1994	Barley	2
40	Chile	1994	Beans	3
40	Chile	1994	Beer	59
40	Chile	1994	Beverages, Alcoholic	4
40	Chile	1994	Bovine Meat	56
40	Chile	1994	Butter, Ghee	2
40	Chile	1994	Cheese	10
40	Chile	1994	Cocoa Beans	2
40	Chile	1994	Coconuts - Incl Copra	1
40	Chile	1994	Coffee	1
40	Chile	1994	Crustaceans	1
40	Chile	1994	Demersal Fish	6
40	Chile	1994	Eggs	15
40	Chile	1994	Fats, Animals, Raw	3
40	Chile	1994	Freshwater Fish	2
40	Chile	1994	Fruits, Other	55
40	Chile	1994	Grapes	11
40	Chile	1994	Groundnuts (Shelled Eq)	1
40	Chile	1994	Lemons, Limes	16
40	Chile	1994	Maize	22
40	Chile	1994	Meat, Other	1
40	Chile	1994	Milk, Whole	191
40	Chile	1994	Molluscs, Other	3
40	Chile	1994	Mutton & Goat Meat	2
40	Chile	1994	Oats	1
40	Chile	1994	Offals, Edible	9
40	Chile	1994	Onions	43
40	Chile	1994	Oranges, Mandarines	20
40	Chile	1994	Peas	1
40	Chile	1994	Pelagic Fish	36
40	Chile	1994	Pigmeat	30
40	Chile	1994	Pineapples	2
40	Chile	1994	Potatoes	145
40	Chile	1994	Poultry Meat	56
40	Chile	1994	Pulses, Other	4
40	Chile	1994	Rape and Mustard Oil	2
40	Chile	1994	Rice (Milled Equivalent)	21
40	Chile	1994	Soyabean Oil	16
40	Chile	1994	Sugar, Refined Equivalent	109
40	Chile	1994	Sunflowerseed Oil	9
40	Chile	1994	Sweet Potatoes	1
40	Chile	1994	Tea	4
40	Chile	1994	Tomatoes	82
40	Chile	1994	Vegetables, Other	200
40	Chile	1994	Wheat	326
40	Chile	1994	Wine	49
40	Chile	1995	Apples	17
40	Chile	1995	Aquatic Animals, Others	2
40	Chile	1995	Bananas	26
40	Chile	1995	Barley	2
40	Chile	1995	Beans	3
40	Chile	1995	Beer	63
40	Chile	1995	Beverages, Alcoholic	4
40	Chile	1995	Bovine Meat	61
40	Chile	1995	Butter, Ghee	2
40	Chile	1995	Cheese	10
40	Chile	1995	Cocoa Beans	2
40	Chile	1995	Coconuts - Incl Copra	1
40	Chile	1995	Coffee	2
40	Chile	1995	Crustaceans	3
40	Chile	1995	Demersal Fish	6
40	Chile	1995	Eggs	14
40	Chile	1995	Fats, Animals, Raw	3
40	Chile	1995	Freshwater Fish	4
40	Chile	1995	Fruits, Other	59
40	Chile	1995	Grapes	24
40	Chile	1995	Groundnuts (Shelled Eq)	1
40	Chile	1995	Lemons, Limes	18
40	Chile	1995	Maize	23
40	Chile	1995	Meat, Other	1
40	Chile	1995	Milk, Whole	208
40	Chile	1995	Molluscs, Other	2
40	Chile	1995	Mutton & Goat Meat	2
40	Chile	1995	Nuts	1
40	Chile	1995	Oats	1

FAO country code	Country	Reference year	Food item	Gross food supply (g *capita*$^{-1}$ d^{-1})
40	Chile	1995	Offals, Edible	9
40	Chile	1995	Onions	45
40	Chile	1995	Oranges, Mandarines	19
40	Chile	1995	Peas	1
40	Chile	1995	Pelagic Fish	30
40	Chile	1995	Pigmeat	32
40	Chile	1995	Pineapples	2
40	Chile	1995	Potatoes	137
40	Chile	1995	Poultry Meat	58
40	Chile	1995	Pulses, Other	5
40	Chile	1995	Rape and Mustard Oil	2
40	Chile	1995	Rice (Milled Equivalent)	17
40	Chile	1995	Soyabean Oil	15
40	Chile	1995	Sugar, Refined Equivalent	111
40	Chile	1995	Sunflowerseed Oil	10
40	Chile	1995	Sweet Potatoes	1
40	Chile	1995	Sweeteners, Other	1
40	Chile	1995	Tea	4
40	Chile	1995	Tomatoes	72
40	Chile	1995	Vegetables, Other	200
40	Chile	1995	Wheat	320
40	Chile	1995	Wine	40
40	Chile	1996	Apples	24
40	Chile	1996	Aquatic Animals, Others	2
40	Chile	1996	Bananas	26
40	Chile	1996	Barley	3
40	Chile	1996	Beans	5
40	Chile	1996	Beer	62
40	Chile	1996	Beverages, Alcoholic	4
40	Chile	1996	Bovine Meat	63
40	Chile	1996	Butter, Ghee	2
40	Chile	1996	Cheese	10
40	Chile	1996	Cocoa Beans	2
40	Chile	1996	Coconuts - Incl Copra	1
40	Chile	1996	Coffee	2
40	Chile	1996	Crustaceans	3
40	Chile	1996	Demersal Fish	7
40	Chile	1996	Eggs	15
40	Chile	1996	Fats, Animals, Raw	3
40	Chile	1996	Freshwater Fish	4
40	Chile	1996	Fruits, Other	66
40	Chile	1996	Grapes	26
40	Chile	1996	Groundnuts (Shelled Eq)	1
40	Chile	1996	Lemons, Limes	17
40	Chile	1996	Maize	19
40	Chile	1996	Meat, Other	1
40	Chile	1996	Milk, Whole	212
40	Chile	1996	Molluscs, Other	3
40	Chile	1996	Mutton & Goat Meat	2
40	Chile	1996	Nuts	1
40	Chile	1996	Oats	1
40	Chile	1996	Offals, Edible	9
40	Chile	1996	Onions	57
40	Chile	1996	Oranges, Mandarines	22
40	Chile	1996	Peas	2
40	Chile	1996	Pelagic Fish	28
40	Chile	1996	Pigmeat	34
40	Chile	1996	Pineapples	2
40	Chile	1996	Potatoes	126
40	Chile	1996	Poultry Meat	61
40	Chile	1996	Pulses, Other	5
40	Chile	1996	Rape and Mustard Oil	2
40	Chile	1996	Rice (Milled Equivalent)	20
40	Chile	1996	Soyabean Oil	15
40	Chile	1996	Sugar, Refined Equivalent	113
40	Chile	1996	Sunflowerseed Oil	10
40	Chile	1996	Sweet Potatoes	1
40	Chile	1996	Sweeteners, Other	2
40	Chile	1996	Tea	4
40	Chile	1996	Tomatoes	92
40	Chile	1996	Vegetables, Other	179
40	Chile	1996	Wheat	310
40	Chile	1996	Wine	43
40	Chile	1997	Apples	9
40	Chile	1997	Aquatic Animals, Others	2
40	Chile	1997	Bananas	25
40	Chile	1997	Barley	3
40	Chile	1997	Beans	5
40	Chile	1997	Beer	64
40	Chile	1997	Beverages, Alcoholic	4
40	Chile	1997	Bovine Meat	67
40	Chile	1997	Butter, Ghee	2
40	Chile	1997	Cheese	11
40	Chile	1997	Cocoa Beans	2
40	Chile	1997	Coconuts - Incl Copra	1
40	Chile	1997	Coffee	2
40	Chile	1997	Crustaceans	3
40	Chile	1997	Demersal Fish	4
40	Chile	1997	Eggs	15
40	Chile	1997	Fats, Animals, Raw	4
40	Chile	1997	Freshwater Fish	2
40	Chile	1997	Fruits, Other	54
40	Chile	1997	Grapes	27
40	Chile	1997	Groundnuts (Shelled Eq)	1
40	Chile	1997	Lemons, Limes	19
40	Chile	1997	Maize	43
40	Chile	1997	Meat, Other	1

FAO country code	Country	Reference year	Food item	Gross food supply (g $capita^{-1}$ d^{-1})
40	Chile	1997	Milk, Whole	197
40	Chile	1997	Molluscs, Other	4
40	Chile	1997	Mutton & Goat Meat	2
40	Chile	1997	Nuts	1
40	Chile	1997	Oats	1
40	Chile	1997	Offals, Edible	9
40	Chile	1997	Onions	29
40	Chile	1997	Oranges, Mandarines	16
40	Chile	1997	Peas	1
40	Chile	1997	Pelagic Fish	27
40	Chile	1997	Pigmeat	36
40	Chile	1997	Pineapples	2
40	Chile	1997	Potatoes	159
40	Chile	1997	Poultry Meat	60
40	Chile	1997	Pulses, Other	4
40	Chile	1997	Rape and Mustard Oil	3
40	Chile	1997	Rice (Milled Equivalent)	20
40	Chile	1997	Rye	1
40	Chile	1997	Soyabean Oil	12
40	Chile	1997	Sugar, Refined Equivalent	114
40	Chile	1997	Sunflowerseed Oil	11
40	Chile	1997	Sweet Potatoes	2
40	Chile	1997	Sweeteners, Other	2
40	Chile	1997	Tea	3
40	Chile	1997	Tomatoes	64
40	Chile	1997	Vegetables, Other	159
40	Chile	1997	Wheat	296
40	Chile	1997	Wine	35
40	Chile	1998	Apples	8
40	Chile	1998	Aquatic Animals, Others	2
40	Chile	1998	Bananas	24
40	Chile	1998	Barley	3
40	Chile	1998	Beans	4
40	Chile	1998	Beer	63
40	Chile	1998	Beverages, Alcoholic	4
40	Chile	1998	Bovine Meat	63
40	Chile	1998	Butter, Ghee	2
40	Chile	1998	Cheese	11
40	Chile	1998	Cocoa Beans	3
40	Chile	1998	Coconuts - Incl Copra	1
40	Chile	1998	Coffee	2
40	Chile	1998	Crustaceans	3
40	Chile	1998	Demersal Fish	2
40	Chile	1998	Eggs	15
40	Chile	1998	Fats, Animals, Raw	5
40	Chile	1998	Freshwater Fish	2
40	Chile	1998	Fruits, Other	44
40	Chile	1998	Grapes	31
40	Chile	1998	Groundnuts (Shelled Eq)	1
40	Chile	1998	Lemons, Limes	17
40	Chile	1998	Maize	39
40	Chile	1998	Meat, Other	1
40	Chile	1998	Milk, Whole	192
40	Chile	1998	Molluscs, Other	4
40	Chile	1998	Mutton & Goat Meat	2
40	Chile	1998	Nuts	1
40	Chile	1998	Oats	1
40	Chile	1998	Offals, Edible	9
40	Chile	1998	Onions	32
40	Chile	1998	Oranges, Mandarines	18
40	Chile	1998	Peas	1
40	Chile	1998	Pelagic Fish	25
40	Chile	1998	Pigmeat	40
40	Chile	1998	Pineapples	2
40	Chile	1998	Potatoes	138
40	Chile	1998	Poultry Meat	65
40	Chile	1998	Pulses, Other	4
40	Chile	1998	Rape and Mustard Oil	3
40	Chile	1998	Rice (Milled Equivalent)	18
40	Chile	1998	Soyabean Oil	12
40	Chile	1998	Sugar, Refined Equivalent	116
40	Chile	1998	Sunflowerseed Oil	10
40	Chile	1998	Sweet Potatoes	1
40	Chile	1998	Sweeteners, Other	1
40	Chile	1998	Tea	4
40	Chile	1998	Tomatoes	62
40	Chile	1998	Vegetables, Other	163
40	Chile	1998	Wheat	303
40	Chile	1998	Wine	49
40	Chile	1999	Apples	12
40	Chile	1999	Aquatic Animals, Others	4
40	Chile	1999	Bananas	26
40	Chile	1999	Barley	3
40	Chile	1999	Beans	4
40	Chile	1999	Beer	61
40	Chile	1999	Beverages, Alcoholic	3
40	Chile	1999	Bovine Meat	59
40	Chile	1999	Butter, Ghee	2
40	Chile	1999	Cheese	10
40	Chile	1999	Cocoa Beans	2
40	Chile	1999	Coconuts - Incl Copra	1
40	Chile	1999	Coffee	1
40	Chile	1999	Crustaceans	2
40	Chile	1999	Demersal Fish	8
40	Chile	1999	Eggs	16
40	Chile	1999	Fats, Animals, Raw	4
40	Chile	1999	Freshwater Fish	2

FAO countrycode	Country	Reference year	Food item	Gross food supply (g $capita^{-1}$ d^{-1})
40	Chile	1999	Fruits, Other	45
40	Chile	1999	Grapes	30
40	Chile	1999	Groundnuts (Shelled Eq)	1
40	Chile	1999	Honey	1
40	Chile	1999	Lemons, Limes	15
40	Chile	1999	Maize	42
40	Chile	1999	Meat, Other	1
40	Chile	1999	Milk, Whole	177
40	Chile	1999	Molluscs, Other	6
40	Chile	1999	Mutton & Goat Meat	2
40	Chile	1999	Nuts	1
40	Chile	1999	Oats	1
40	Chile	1999	Offals, Edible	8
40	Chile	1999	Olives	1
40	Chile	1999	Onions	35
40	Chile	1999	Oranges, Mandarines	16
40	Chile	1999	Peas	1
40	Chile	1999	Pelagic Fish	16
40	Chile	1999	Pigmeat	43
40	Chile	1999	Pineapples	2
40	Chile	1999	Potatoes	152
40	Chile	1999	Poultry Meat	66
40	Chile	1999	Pulses, Other	4
40	Chile	1999	Rape and Mustard Oil	3
40	Chile	1999	Rice (Milled Equivalent)	18
40	Chile	1999	Rye	1
40	Chile	1999	Soyabean Oil	13
40	Chile	1999	Sugar, Refined Equivalent	117
40	Chile	1999	Sunflowerseed Oil	11
40	Chile	1999	Sweet Potatoes	1
40	Chile	1999	Sweeteners, Other	3
40	Chile	1999	Tea	3
40	Chile	1999	Tomatoes	58
40	Chile	1999	Vegetables, Other	163
40	Chile	1999	Wheat	304
40	Chile	1999	Wine	36
40	Chile	2000	Apples	3
40	Chile	2000	Aquatic Animals, Others	3
40	Chile	2000	Bananas	32
40	Chile	2000	Barley	3
40	Chile	2000	Beans	5
40	Chile	2000	Beer	53
40	Chile	2000	Beverages, Alcoholic	5
40	Chile	2000	Bovine Meat	59
40	Chile	2000	Butter, Ghee	2
40	Chile	2000	Cheese	10
40	Chile	2000	Cocoa Beans	2
40	Chile	2000	Coconuts - Incl Copra	1
40	Chile	2000	Coffee	2
40	Chile	2000	Crustaceans	1
40	Chile	2000	Demersal Fish	5
40	Chile	2000	Eggs	16
40	Chile	2000	Fats, Animals, Raw	5
40	Chile	2000	Freshwater Fish	5
40	Chile	2000	Fruits, Other	52
40	Chile	2000	Grapes	29
40	Chile	2000	Groundnuts (Shelled Eq)	1
40	Chile	2000	Honey	1
40	Chile	2000	Lemons, Limes	17
40	Chile	2000	Maize	43
40	Chile	2000	Meat, Other	1
40	Chile	2000	Milk, Whole	180
40	Chile	2000	Molluscs, Other	8
40	Chile	2000	Mutton & Goat Meat	2
40	Chile	2000	Nuts	1
40	Chile	2000	Oats	1
40	Chile	2000	Offals, Edible	8
40	Chile	2000	Olives	1
40	Chile	2000	Onions	40
40	Chile	2000	Oranges, Mandarines	17
40	Chile	2000	Peas	1
40	Chile	2000	Pelagic Fish	14
40	Chile	2000	Pigmeat	43
40	Chile	2000	Pineapples	2
40	Chile	2000	Potatoes	148
40	Chile	2000	Poultry Meat	72
40	Chile	2000	Pulses, Other	4
40	Chile	2000	Rape and Mustard Oil	3
40	Chile	2000	Rice (Milled Equivalent)	19
40	Chile	2000	Soyabean Oil	13
40	Chile	2000	Sugar, Refined Equivalent	111
40	Chile	2000	Sunflowerseed Oil	12
40	Chile	2000	Sweet Potatoes	1
40	Chile	2000	Sweeteners, Other	3
40	Chile	2000	Tea	3
40	Chile	2000	Tomatoes	74
40	Chile	2000	Vegetables, Other	158
40	Chile	2000	Wheat	304
40	Chile	2000	Wine	35
40	Chile	2001	Apples	2
40	Chile	2001	Aquatic Animals, Others	1
40	Chile	2001	Bananas	20
40	Chile	2001	Barley	3
40	Chile	2001	Beans	6
40	Chile	2001	Beer	61
40	Chile	2001	Beverages, Alcoholic	6
40	Chile	2001	Bovine Meat	54

FAO country code	Country	Reference year	Food item	Gross food supply (g *capita*$^{-1}$ d^{-1})
40	Chile	2001	Butter, Ghee	2
40	Chile	2001	Cheese	10
40	Chile	2001	Cocoa Beans	2
40	Chile	2001	Coconuts - Incl Copra	1
40	Chile	2001	Coffee	1
40	Chile	2001	Crustaceans	1
40	Chile	2001	Demersal Fish	9
40	Chile	2001	Eggs	17
40	Chile	2001	Fats, Animals, Raw	6
40	Chile	2001	Freshwater Fish	2
40	Chile	2001	Fruits, Other	44
40	Chile	2001	Grapes	26
40	Chile	2001	Groundnuts (Shelled Eq)	1
40	Chile	2001	Lemons, Limes	17
40	Chile	2001	Maize	43
40	Chile	2001	Meat, Other	1
40	Chile	2001	Milk, Whole	178
40	Chile	2001	Molluscs, Other	8
40	Chile	2001	Mutton & Goat Meat	2
40	Chile	2001	Nuts	1
40	Chile	2001	Oats	1
40	Chile	2001	Offals, Edible	7
40	Chile	2001	Olives	1
40	Chile	2001	Onions	39
40	Chile	2001	Oranges, Mandarines	17
40	Chile	2001	Peas	1
40	Chile	2001	Pelagic Fish	14
40	Chile	2001	Pigmeat	46
40	Chile	2001	Pineapples	1
40	Chile	2001	Potatoes	183
40	Chile	2001	Poultry Meat	77
40	Chile	2001	Pulses, Other	4
40	Chile	2001	Rape and Mustard Oil	3
40	Chile	2001	Rice (Milled Equivalent)	19
40	Chile	2001	Rye	1
40	Chile	2001	Soyabean Oil	15
40	Chile	2001	Sugar, Refined Equivalent	109
40	Chile	2001	Sunflowerseed Oil	10
40	Chile	2001	Sweet Potatoes	1
40	Chile	2001	Sweeteners, Other	3
40	Chile	2001	Tea	3
40	Chile	2001	Tomatoes	67
40	Chile	2001	Vegetables, Other	159
40	Chile	2001	Wheat	313
40	Chile	2001	Wine	39
40	Chile	2002	Apples	20
40	Chile	2002	Aquatic Animals, Others	4
40	Chile	2002	Bananas	24
40	Chile	2002	Barley	3
40	Chile	2002	Beans	6
40	Chile	2002	Beer	59
40	Chile	2002	Beverages, Alcoholic	5
40	Chile	2002	Bovine Meat	57
40	Chile	2002	Butter, Ghee	2
40	Chile	2002	Cephalopods	1
40	Chile	2002	Cheese	10
40	Chile	2002	Cocoa Beans	3
40	Chile	2002	Coconuts - Incl Copra	1
40	Chile	2002	Coffee	1
40	Chile	2002	Crustaceans	2
40	Chile	2002	Demersal Fish	6
40	Chile	2002	Eggs	17
40	Chile	2002	Fats, Animals, Raw	6
40	Chile	2002	Freshwater Fish	1
40	Chile	2002	Fruits, Other	55
40	Chile	2002	Grapes	20
40	Chile	2002	Groundnuts (Shelled Eq)	1
40	Chile	2002	Honey	1
40	Chile	2002	Lemons, Limes	19
40	Chile	2002	Maize	34
40	Chile	2002	Meat, Other	1
40	Chile	2002	Milk, Whole	169
40	Chile	2002	Molluscs, Other	4
40	Chile	2002	Mutton & Goat Meat	2
40	Chile	2002	Nuts	2
40	Chile	2002	Oats	1
40	Chile	2002	Offals, Edible	8
40	Chile	2002	Olives	1
40	Chile	2002	Onions	42
40	Chile	2002	Oranges, Mandarines	20
40	Chile	2002	Peas	1
40	Chile	2002	Pelagic Fish	19
40	Chile	2002	Pigmeat	45
40	Chile	2002	Pineapples	2
40	Chile	2002	Potatoes	200
40	Chile	2002	Poultry Meat	73
40	Chile	2002	Pulses, Other	4
40	Chile	2002	Rape and Mustard Oil	3
40	Chile	2002	Rice (Milled Equivalent)	19
40	Chile	2002	Rye	1
40	Chile	2002	Soyabean Oil	15
40	Chile	2002	Sugar, Refined Equivalent	111
40	Chile	2002	Sunflowerseed Oil	10
40	Chile	2002	Sweet Potatoes	1
40	Chile	2002	Sweeteners, Other	3
40	Chile	2002	Tea	4
40	Chile	2002	Tomatoes	75

FAO country code	Country	Reference year	Food item	Gross food supply (g $capita^{-1}$ d^{-1})
40	Chile	2002	Vegetables, Other	161
40	Chile	2002	Wheat	316
40	Chile	2002	Wine	38
40	Chile	2003	Apples	30
40	Chile	2003	Aquatic Animals, Others	3
40	Chile	2003	Bananas	25
40	Chile	2003	Barley	3
40	Chile	2003	Beans	6
40	Chile	2003	Beer	72
40	Chile	2003	Beverages, Alcoholic	4
40	Chile	2003	Bovine Meat	58
40	Chile	2003	Butter, Ghee	2
40	Chile	2003	Cheese	10
40	Chile	2003	Cocoa Beans	3
40	Chile	2003	Coconuts - Incl Copra	1
40	Chile	2003	Coffee	2
40	Chile	2003	Crustaceans	1
40	Chile	2003	Demersal Fish	11
40	Chile	2003	Eggs	17
40	Chile	2003	Fats, Animals, Raw	7
40	Chile	2003	Freshwater Fish	2
40	Chile	2003	Fruits, Other	45
40	Chile	2003	Grapes	8
40	Chile	2003	Groundnuts (Shelled Eq)	1
40	Chile	2003	Lemons, Limes	22
40	Chile	2003	Maize	46
40	Chile	2003	Meat, Other	1
40	Chile	2003	Milk, Whole	179
40	Chile	2003	Molluscs, Other	5
40	Chile	2003	Mutton & Goat Meat	2
40	Chile	2003	Nuts	2
40	Chile	2003	Oats	1
40	Chile	2003	Offals, Edible	7
40	Chile	2003	Olive Oil	1
40	Chile	2003	Olives	2
40	Chile	2003	Onions	43
40	Chile	2003	Oranges, Mandarines	20
40	Chile	2003	Peas	1
40	Chile	2003	Pelagic Fish	21
40	Chile	2003	Pigmeat	50
40	Chile	2003	Pineapples	2
40	Chile	2003	Potatoes	164
40	Chile	2003	Poultry Meat	73
40	Chile	2003	Pulses, Other	4
40	Chile	2003	Rape and Mustard Oil	3
40	Chile	2003	Rice (Milled Equivalent)	20
40	Chile	2003	Rye	2
40	Chile	2003	Soyabean Oil	14
40	Chile	2003	Sugar, Refined Equivalent	104
40	Chile	2003	Sunflowerseed Oil	10
40	Chile	2003	Sweet Potatoes	1
40	Chile	2003	Sweeteners, Other	3
40	Chile	2003	Tea	4
40	Chile	2003	Tomatoes	96
40	Chile	2003	Vegetables, Other	159
40	Chile	2003	Wheat	309
40	Chile	2003	Wine	40
40	Chile	2004	Apples	12
40	Chile	2004	Aquatic Animals, Others	4
40	Chile	2004	Bananas	25
40	Chile	2004	Barley	3
40	Chile	2004	Beans	7
40	Chile	2004	Beer	72
40	Chile	2004	Beverages, Alcoholic	5
40	Chile	2004	Bovine Meat	60
40	Chile	2004	Butter, Ghee	2
40	Chile	2004	Cephalopods	2
40	Chile	2004	Cheese	10
40	Chile	2004	Cocoa Beans	3
40	Chile	2004	Coconuts - Incl Copra	1
40	Chile	2004	Coffee	2
40	Chile	2004	Crustaceans	1
40	Chile	2004	Demersal Fish	15
40	Chile	2004	Eggs	17
40	Chile	2004	Fats, Animals, Raw	7
40	Chile	2004	Freshwater Fish	1
40	Chile	2004	Fruits, Other	52
40	Chile	2004	Grapes	24
40	Chile	2004	Groundnuts (Shelled Eq)	1
40	Chile	2004	Honey	1
40	Chile	2004	Lemons, Limes	19
40	Chile	2004	Maize	48
40	Chile	2004	Meat, Other	1
40	Chile	2004	Milk, Whole	156
40	Chile	2004	Molluscs, Other	4
40	Chile	2004	Mutton & Goat Meat	2
40	Chile	2004	Nuts	2
40	Chile	2004	Oats	1
40	Chile	2004	Offals, Edible	7
40	Chile	2004	Olive Oil	1
40	Chile	2004	Olives	2
40	Chile	2004	Onions	42
40	Chile	2004	Oranges, Mandarines	21
40	Chile	2004	Peas	1
40	Chile	2004	Pelagic Fish	20
40	Chile	2004	Pigmeat	46
40	Chile	2004	Pineapples	3

FAO country code	Country	Reference year	Food item	Gross food supply (g $capita^{-1}$ d^{-1})
40	Chile	2004	Potatoes	169
40	Chile	2004	Poultry Meat	80
40	Chile	2004	Pulses, Other	5
40	Chile	2004	Rape and Mustard Oil	3
40	Chile	2004	Rice (Milled Equivalent)	23
40	Chile	2004	Rye	2
40	Chile	2004	Soyabean Oil	14
40	Chile	2004	Sugar, Refined Equivalent	106
40	Chile	2004	Sunflowerseed Oil	10
40	Chile	2004	Sweet Potatoes	1
40	Chile	2004	Sweeteners, Other	3
40	Chile	2004	Tea	4
40	Chile	2004	Tomatoes	90
40	Chile	2004	Vegetables, Other	157
40	Chile	2004	Wheat	311
40	Chile	2004	Wine	39
40	Chile	2005	Apples	16
40	Chile	2005	Aquatic Animals, Others	2
40	Chile	2005	Bananas	26
40	Chile	2005	Barley	3
40	Chile	2005	Beans	6
40	Chile	2005	Beer	75
40	Chile	2005	Beverages, Alcoholic	5
40	Chile	2005	Bovine Meat	62
40	Chile	2005	Butter, Ghee	3
40	Chile	2005	Cephalopods	4
40	Chile	2005	Cheese	11
40	Chile	2005	Cocoa Beans	3
40	Chile	2005	Coconuts - Incl Copra	1
40	Chile	2005	Coffee	2
40	Chile	2005	Crustaceans	1
40	Chile	2005	Demersal Fish	11
40	Chile	2005	Eggs	18
40	Chile	2005	Fats, Animals, Raw	7
40	Chile	2005	Freshwater Fish	1
40	Chile	2005	Fruits, Other	50
40	Chile	2005	Grapes	22
40	Chile	2005	Groundnuts (Shelled Eq)	1
40	Chile	2005	Lemons, Limes	19
40	Chile	2005	Maize	56
40	Chile	2005	Meat, Other	1
40	Chile	2005	Milk, Whole	146
40	Chile	2005	Molluscs, Other	5
40	Chile	2005	Mutton & Goat Meat	2
40	Chile	2005	Nuts	1
40	Chile	2005	Oats	1
40	Chile	2005	Offals, Edible	7
40	Chile	2005	Olive Oil	1
40	Chile	2005	Olives	2
40	Chile	2005	Onions	49
40	Chile	2005	Oranges, Mandarines	20
40	Chile	2005	Peas	1
40	Chile	2005	Pelagic Fish	19
40	Chile	2005	Pigmeat	49
40	Chile	2005	Pineapples	3
40	Chile	2005	Potatoes	164
40	Chile	2005	Poultry Meat	80
40	Chile	2005	Pulses, Other	5
40	Chile	2005	Rape and Mustard Oil	2
40	Chile	2005	Rice (Milled Equivalent)	23
40	Chile	2005	Soyabean Oil	14
40	Chile	2005	Sugar, Refined Equivalent	106
40	Chile	2005	Sunflowerseed Oil	5
40	Chile	2005	Sweet Potatoes	1
40	Chile	2005	Sweeteners, Other	4
40	Chile	2005	Tea	4
40	Chile	2005	Tomatoes	81
40	Chile	2005	Vegetables, Other	148
40	Chile	2005	Wheat	311
40	Chile	2005	Wine	42
40	Chile	2006	Apples	7
40	Chile	2006	Aquatic Animals, Others	2
40	Chile	2006	Bananas	26
40	Chile	2006	Barley	2
40	Chile	2006	Beans	6
40	Chile	2006	Beer	93
40	Chile	2006	Beverages, Alcoholic	6
40	Chile	2006	Bovine Meat	56
40	Chile	2006	Butter, Ghee	3
40	Chile	2006	Cephalopods	1
40	Chile	2006	Cheese	11
40	Chile	2006	Cocoa Beans	3
40	Chile	2006	Coconuts - Incl Copra	1
40	Chile	2006	Coffee	2
40	Chile	2006	Crustaceans	2
40	Chile	2006	Demersal Fish	8
40	Chile	2006	Eggs	17
40	Chile	2006	Fats, Animals, Raw	8
40	Chile	2006	Freshwater Fish	1
40	Chile	2006	Fruits, Other	63
40	Chile	2006	Grapes	18
40	Chile	2006	Groundnuts (Shelled Eq)	1
40	Chile	2006	Lemons, Limes	16
40	Chile	2006	Maize	57
40	Chile	2006	Meat, Other	1
40	Chile	2006	Milk, Whole	152
40	Chile	2006	Molluscs, Other	4

FAO country code	Country	Reference year	Food item	Gross food supply (g $capita^{-1}$ d^{-1})
40	Chile	2006	Mutton & Goat Meat	2
40	Chile	2006	Nuts	4
40	Chile	2006	Oats	1
40	Chile	2006	Offals, Edible	7
40	Chile	2006	Olive Oil	1
40	Chile	2006	Olives	2
40	Chile	2006	Onions	53
40	Chile	2006	Oranges, Mandarines	20
40	Chile	2006	Peas	1
40	Chile	2006	Pelagic Fish	24
40	Chile	2006	Pigmeat	57
40	Chile	2006	Pineapples	4
40	Chile	2006	Potatoes	169
40	Chile	2006	Poultry Meat	91
40	Chile	2006	Pulses, Other	6
40	Chile	2006	Rape and Mustard Oil	4
40	Chile	2006	Rice (Milled Equivalent)	24
40	Chile	2006	Soyabean Oil	14
40	Chile	2006	Sugar, Refined Equivalent	105
40	Chile	2006	Sunflowerseed Oil	4
40	Chile	2006	Sweet Potatoes	1
40	Chile	2006	Sweeteners, Other	4
40	Chile	2006	Tea	4
40	Chile	2006	Tomatoes	79
40	Chile	2006	Vegetables, Other	142
40	Chile	2006	Wheat	304
40	Chile	2006	Wine	42
40	Chile	2007	Apples	19
40	Chile	2007	Aquatic Animals, Others	2
40	Chile	2007	Bananas	28
40	Chile	2007	Barley	2
40	Chile	2007	Beans	4
40	Chile	2007	Beer	96
40	Chile	2007	Beverages, Alcoholic	7
40	Chile	2007	Bovine Meat	60
40	Chile	2007	Butter, Ghee	3
40	Chile	2007	Cephalopods	1
40	Chile	2007	Cereals, Other	2
40	Chile	2007	Cheese	10
40	Chile	2007	Cocoa Beans	3
40	Chile	2007	Coconuts - Incl Copra	1
40	Chile	2007	Coffee	2
40	Chile	2007	Crustaceans	2
40	Chile	2007	Demersal Fish	6
40	Chile	2007	Eggs	19
40	Chile	2007	Fats, Animals, Raw	9
40	Chile	2007	Fruits, Other	57
40	Chile	2007	Grapes	15
40	Chile	2007	Groundnuts (Shelled Eq)	2
40	Chile	2007	Lemons, Limes	15
40	Chile	2007	Maize	58
40	Chile	2007	Meat, Other	1
40	Chile	2007	Milk, Whole	141
40	Chile	2007	Molluscs, Other	6
40	Chile	2007	Mutton & Goat Meat	2
40	Chile	2007	Nuts	3
40	Chile	2007	Oats	1
40	Chile	2007	Offals, Edible	7
40	Chile	2007	Olive Oil	1
40	Chile	2007	Olives	3
40	Chile	2007	Onions	34
40	Chile	2007	Oranges, Mandarines	21
40	Chile	2007	Peas	1
40	Chile	2007	Pelagic Fish	23
40	Chile	2007	Pigmeat	57
40	Chile	2007	Pineapples	4
40	Chile	2007	Potatoes	144
40	Chile	2007	Poultry Meat	94
40	Chile	2007	Pulses, Other	5
40	Chile	2007	Rape and Mustard Oil	4
40	Chile	2007	Rice (Milled Equivalent)	25
40	Chile	2007	Rye	1
40	Chile	2007	Soyabean Oil	16
40	Chile	2007	Sugar, Refined Equivalent	115
40	Chile	2007	Sunflowerseed Oil	3
40	Chile	2007	Sweet Potatoes	1
40	Chile	2007	Sweeteners, Other	4
40	Chile	2007	Tea	4
40	Chile	2007	Tomatoes	41
40	Chile	2007	Vegetables, Other	133
40	Chile	2007	Wheat	287
40	Chile	2007	Wine	41
40	Chile	2008	Apples	30
40	Chile	2008	Aquatic Animals, Others	2
40	Chile	2008	Bananas	27
40	Chile	2008	Barley	2
40	Chile	2008	Beans	4
40	Chile	2008	Beer	100
40	Chile	2008	Beverages, Alcoholic	7
40	Chile	2008	Bovine Meat	57
40	Chile	2008	Butter, Ghee	2
40	Chile	2008	Cephalopods	4
40	Chile	2008	Cereals, Other	2
40	Chile	2008	Cheese	10
40	Chile	2008	Citrus, Other	1
40	Chile	2008	Cocoa Beans	4
40	Chile	2008	Coconuts - Incl Copra	1

FAO countrycode	Country	Reference year	Food item	Gross food supply (g $capita^{-1}$ d^{-1})
40	Chile	2008	Coffee	1
40	Chile	2008	Crustaceans	3
40	Chile	2008	Demersal Fish	9
40	Chile	2008	Eggs	19
40	Chile	2008	Fats, Animals, Raw	9
40	Chile	2008	Freshwater Fish	1
40	Chile	2008	Fruits, Other	52
40	Chile	2008	Grapes	13
40	Chile	2008	Groundnuts (Shelled Eq)	2
40	Chile	2008	Lemons, Limes	18
40	Chile	2008	Maize	61
40	Chile	2008	Meat, Other	1
40	Chile	2008	Milk, Whole	171
40	Chile	2008	Molluscs, Other	5
40	Chile	2008	Mutton & Goat Meat	2
40	Chile	2008	Nuts	2
40	Chile	2008	Oats	4
40	Chile	2008	Offals, Edible	9
40	Chile	2008	Olives	3
40	Chile	2008	Onions	28
40	Chile	2008	Oranges, Mandarines	20
40	Chile	2008	Peas	1
40	Chile	2008	Pelagic Fish	21
40	Chile	2008	Pigmeat	62
40	Chile	2008	Pineapples	5
40	Chile	2008	Potatoes	144
40	Chile	2008	Poultry Meat	91
40	Chile	2008	Pulses, Other	5
40	Chile	2008	Rape and Mustard Oil	4
40	Chile	2008	Rice (Milled Equivalent)	26
40	Chile	2008	Rye	1
40	Chile	2008	Soyabean Oil	13
40	Chile	2008	Sugar, Refined Equivalent	117
40	Chile	2008	Sunflowerseed Oil	4
40	Chile	2008	Sweet Potatoes	2
40	Chile	2008	Sweeteners, Other	3
40	Chile	2008	Tea	4
40	Chile	2008	Tomatoes	52
40	Chile	2008	Vegetables, Other	139
40	Chile	2008	Wheat	283
40	Chile	2008	Wine	44
40	Chile	2009	Apples	26
40	Chile	2009	Aquatic Animals, Others	2
40	Chile	2009	Bananas	27
40	Chile	2009	Barley	2
40	Chile	2009	Beans	4
40	Chile	2009	Beer	99
40	Chile	2009	Beverages, Alcoholic	8
40	Chile	2009	Bovine Meat	57
40	Chile	2009	Butter, Ghee	3
40	Chile	2009	Cephalopods	1
40	Chile	2009	Cereals, Other	3
40	Chile	2009	Cheese	10
40	Chile	2009	Cocoa Beans	3
40	Chile	2009	Coconuts - Incl Copra	1
40	Chile	2009	Crustaceans	3
40	Chile	2009	Demersal Fish	10
40	Chile	2009	Eggs	26
40	Chile	2009	Fats, Animals, Raw	8
40	Chile	2009	Freshwater Fish	1
40	Chile	2009	Fruits, Other	70
40	Chile	2009	Grapes	11
40	Chile	2009	Groundnuts (Shelled Eq)	2
40	Chile	2009	Lemons, Limes	18
40	Chile	2009	Maize	62
40	Chile	2009	Meat, Other	1
40	Chile	2009	Milk, Whole	138
40	Chile	2009	Molluscs, Other	4
40	Chile	2009	Mutton & Goat Meat	2
40	Chile	2009	Nuts	2
40	Chile	2009	Oats	4
40	Chile	2009	Offals, Edible	7
40	Chile	2009	Olives	4
40	Chile	2009	Onions	38
40	Chile	2009	Oranges, Mandarines	16
40	Chile	2009	Peas	1
40	Chile	2009	Pelagic Fish	20
40	Chile	2009	Pigmeat	58
40	Chile	2009	Pineapples	4
40	Chile	2009	Potatoes	137
40	Chile	2009	Poultry Meat	85
40	Chile	2009	Pulses, Other	3
40	Chile	2009	Rape and Mustard Oil	4
40	Chile	2009	Rice (Milled Equivalent)	25
40	Chile	2009	Rye	1
40	Chile	2009	Soyabean Oil	13
40	Chile	2009	Sugar, Refined Equivalent	116
40	Chile	2009	Sunflowerseed Oil	4
40	Chile	2009	Sweet Potatoes	2
40	Chile	2009	Sweeteners, Other	4
40	Chile	2009	Tea	4
40	Chile	2009	Tomatoes	22
40	Chile	2009	Vegetables, Other	154
40	Chile	2009	Wheat	284
40	Chile	2009	Wine	44
40	Chile	2010	Apples	41
40	Chile	2010	Aquatic Animals, Others	2

FAO countrycode	Country	Reference year	Food item	Gross food supply (g *capita*$^{-1}$ d^{-1})
40	Chile	2010	Bananas	26
40	Chile	2010	Barley	2
40	Chile	2010	Beans	3
40	Chile	2010	Beer	101
40	Chile	2010	Beverages, Alcoholic	8
40	Chile	2010	Bovine Meat	59
40	Chile	2010	Butter, Ghee	3
40	Chile	2010	Cephalopods	4
40	Chile	2010	Cereals, Other	4
40	Chile	2010	Cheese	11
40	Chile	2010	Cocoa Beans	4
40	Chile	2010	Coconuts - Incl Copra	1
40	Chile	2010	Coffee	1
40	Chile	2010	Crustaceans	3
40	Chile	2010	Demersal Fish	12
40	Chile	2010	Eggs	26
40	Chile	2010	Fats, Animals, Raw	9
40	Chile	2010	Freshwater Fish	1
40	Chile	2010	Fruits, Other	58
40	Chile	2010	Grapes	18
40	Chile	2010	Groundnuts (Shelled Eq)	2
40	Chile	2010	Lemons, Limes	16
40	Chile	2010	Maize	58
40	Chile	2010	Meat, Other	1
40	Chile	2010	Milk, Whole	136
40	Chile	2010	Molluscs, Other	2
40	Chile	2010	Mutton & Goat Meat	2
40	Chile	2010	Nuts	3
40	Chile	2010	Oats	5
40	Chile	2010	Offals, Edible	6
40	Chile	2010	Olives	5
40	Chile	2010	Onions	30
40	Chile	2010	Oranges, Mandarines	13
40	Chile	2010	Peas	1
40	Chile	2010	Pelagic Fish	14
40	Chile	2010	Pigmeat	60
40	Chile	2010	Pineapples	5
40	Chile	2010	Potatoes	160
40	Chile	2010	Poultry Meat	90
40	Chile	2010	Pulses, Other	6
40	Chile	2010	Rape and Mustard Oil	4
40	Chile	2010	Rice (Milled Equivalent)	22
40	Chile	2010	Rye	1
40	Chile	2010	Soyabean Oil	13
40	Chile	2010	Sugar, Refined Equivalent	116
40	Chile	2010	Sunflowerseed Oil	4
40	Chile	2010	Sweet Potatoes	2
40	Chile	2010	Sweeteners, Other	7
40	Chile	2010	Tea	4
40	Chile	2010	Tomatoes	17
40	Chile	2010	Vegetables, Other	144
40	Chile	2010	Wheat	290
40	Chile	2010	Wine	44
40	Chile	2011	Apples	24
40	Chile	2011	Aquatic Animals, Others	2
40	Chile	2011	Bananas	27
40	Chile	2011	Barley	2
40	Chile	2011	Beans	4
40	Chile	2011	Beer	106
40	Chile	2011	Beverages, Alcoholic	7
40	Chile	2011	Bovine Meat	54
40	Chile	2011	Butter, Ghee	3
40	Chile	2011	Cephalopods	4
40	Chile	2011	Cereals, Other	5
40	Chile	2011	Cheese	12
40	Chile	2011	Cocoa Beans	3
40	Chile	2011	Coconuts - Incl Copra	1
40	Chile	2011	Coffee	1
40	Chile	2011	Crustaceans	3
40	Chile	2011	Demersal Fish	12
40	Chile	2011	Eggs	27
40	Chile	2011	Fats, Animals, Raw	10
40	Chile	2011	Freshwater Fish	1
40	Chile	2011	Fruits, Other	44
40	Chile	2011	Grapes	13
40	Chile	2011	Groundnuts (Shelled Eq)	2
40	Chile	2011	Lemons, Limes	16
40	Chile	2011	Maize	59
40	Chile	2011	Meat, Other	1
40	Chile	2011	Milk, Whole	140
40	Chile	2011	Molluscs, Other	2
40	Chile	2011	Mutton & Goat Meat	2
40	Chile	2011	Nuts	2
40	Chile	2011	Oats	5
40	Chile	2011	Offals, Edible	5
40	Chile	2011	Olives	4
40	Chile	2011	Onions	29
40	Chile	2011	Oranges, Mandarines	12
40	Chile	2011	Peas	1
40	Chile	2011	Pelagic Fish	13
40	Chile	2011	Pigmeat	64
40	Chile	2011	Pineapples	6
40	Chile	2011	Potatoes	185
40	Chile	2011	Poultry Meat	99
40	Chile	2011	Pulses, Other	5
40	Chile	2011	Rape and Mustard Oil	4
40	Chile	2011	Rice (Milled Equivalent)	22

FAO countrycode	Country	Reference year	Food item	Gross food supply (g *capita*$^{-1}$ d^{-1})
40	Chile	2011	Rye	1
40	Chile	2011	Soyabean Oil	13
40	Chile	2011	Sugar, Refined Equivalent	114
40	Chile	2011	Sunflowerseed Oil	4
40	Chile	2011	Sweet Potatoes	2
40	Chile	2011	Sweeteners, Other	9
40	Chile	2011	Tea	4
40	Chile	2011	Tomatoes	14
40	Chile	2011	Vegetables, Other	143
40	Chile	2011	Wheat	295
40	Chile	2011	Wine	47
351	China	1992	Apples	12
351	China	1992	Aquatic Plants	7
351	China	1992	Bananas	5
351	China	1992	Barley	3
351	China	1992	Beans	1
351	China	1992	Beer	24
351	China	1992	Beverages, Alcoholic	17
351	China	1992	Beverages, Fermented	1
351	China	1992	Bovine Meat	4
351	China	1992	Cassava	3
351	China	1992	Cephalopods	1
351	China	1992	Cereals, Other	2
351	China	1992	Citrus, Other	1
351	China	1992	Cottonseed Oil	2
351	China	1992	Crustaceans	3
351	China	1992	Demersal Fish	4
351	China	1992	Eggs	21
351	China	1992	Fats, Animals, Raw	4
351	China	1992	Freshwater Fish	14
351	China	1992	Fruits, Other	20
351	China	1992	Grapes	2
351	China	1992	Groundnut Oil	2
351	China	1992	Groundnuts (Shelled Eq)	3
351	China	1992	Maize	14
351	China	1992	Marine Fish, Other	6
351	China	1992	Meat, Other	1
351	China	1992	Milk, Whole	14
351	China	1992	Millet	5
351	China	1992	Molluscs, Other	7
351	China	1992	Mutton & Goat Meat	3
351	China	1992	Nuts	1
351	China	1992	Oats	1
351	China	1992	Offals, Edible	4
351	China	1992	Onions	11
351	China	1992	Oranges, Mandarines	11
351	China	1992	Palm Oil	2
351	China	1992	Peas	1
351	China	1992	Pelagic Fish	1
351	China	1992	Pigmeat	59
351	China	1992	Pineapples	1
351	China	1992	Potatoes	40
351	China	1992	Poultry Meat	12
351	China	1992	Pulses, Other	1
351	China	1992	Rape and Mustard Oil	5
351	China	1992	Rice (Milled Equivalent)	200
351	China	1992	Roots, Other	3
351	China	1992	Rye	2
351	China	1992	Sorghum	5
351	China	1992	Soyabean Oil	3
351	China	1992	Soyabeans	10
351	China	1992	Sugar, Non-Centrifugal	1
351	China	1992	Sugar, Refined Equivalent	17
351	China	1992	Sunflowerseed Oil	1
351	China	1992	Sweet Potatoes	128
351	China	1992	Tea	1
351	China	1992	Tomatoes	17
351	China	1992	Vegetables, Other	275
351	China	1992	Wheat	214
351	China	1992	Wine	1
351	China	1993	Apples	17
351	China	1993	Aquatic Plants	9
351	China	1993	Bananas	6
351	China	1993	Barley	2
351	China	1993	Beans	1
351	China	1993	Beer	28
351	China	1993	Beverages, Alcoholic	18
351	China	1993	Beverages, Fermented	1
351	China	1993	Bovine Meat	5
351	China	1993	Cassava	3
351	China	1993	Cephalopods	1
351	China	1993	Cereals, Other	2
351	China	1993	Citrus, Other	1
351	China	1993	Cottonseed Oil	2
351	China	1993	Crustaceans	3
351	China	1993	Demersal Fish	4
351	China	1993	Eggs	24
351	China	1993	Fats, Animals, Raw	4
351	China	1993	Freshwater Fish	16
351	China	1993	Fruits, Other	23
351	China	1993	Grapes	2
351	China	1993	Groundnut Oil	2
351	China	1993	Groundnuts (Shelled Eq)	5
351	China	1993	Maize	15
351	China	1993	Marine Fish, Other	6
351	China	1993	Meat, Other	1
351	China	1993	Milk, Whole	14

FAO country code	Country	Reference year	Food item	Gross food supply (g *capita*$^{-1}$ d^{-1})
351	China	1993	Millet	6
351	China	1993	Molluscs, Other	9
351	China	1993	Mutton & Goat Meat	3
351	China	1993	Nuts	2
351	China	1993	Oats	1
351	China	1993	Offals, Edible	4
351	China	1993	Onions	13
351	China	1993	Oranges, Mandarines	14
351	China	1993	Palm Oil	2
351	China	1993	Peas	1
351	China	1993	Pelagic Fish	2
351	China	1993	Pigmeat	61
351	China	1993	Pineapples	2
351	China	1993	Potatoes	39
351	China	1993	Poultry Meat	15
351	China	1993	Pulses, Other	2
351	China	1993	Rape and Mustard Oil	5
351	China	1993	Rice (Milled Equivalent)	204
351	China	1993	Roots, Other	2
351	China	1993	Rye	1
351	China	1993	Sorghum	6
351	China	1993	Soyabean Oil	3
351	China	1993	Soyabeans	13
351	China	1993	Sugar Cane	1
351	China	1993	Sugar, Non-Centrifugal	1
351	China	1993	Sugar, Refined Equivalent	13
351	China	1993	Sunflowerseed Oil	1
351	China	1993	Sweet Potatoes	138
351	China	1993	Tea	1
351	China	1993	Tomatoes	21
351	China	1993	Vegetables, Other	317
351	China	1993	Wheat	217
351	China	1993	Wine	1
351	China	1994	Apples	20
351	China	1994	Aquatic Plants	9
351	China	1994	Bananas	6
351	China	1994	Barley	2
351	China	1994	Beans	1
351	China	1994	Beer	32
351	China	1994	Beverages, Alcoholic	17
351	China	1994	Beverages, Fermented	1
351	China	1994	Bovine Meat	5
351	China	1994	Cassava	3
351	China	1994	Cephalopods	1
351	China	1994	Cereals, Other	2
351	China	1994	Citrus, Other	1
351	China	1994	Cottonseed Oil	2
351	China	1994	Crustaceans	4
351	China	1994	Demersal Fish	5
351	China	1994	Eggs	30
351	China	1994	Fats, Animals, Raw	4
351	China	1994	Freshwater Fish	19
351	China	1994	Fruits, Other	27
351	China	1994	Grapes	2
351	China	1994	Groundnut Oil	2
351	China	1994	Groundnuts (Shelled Eq)	5
351	China	1994	Maize	17
351	China	1994	Marine Fish, Other	7
351	China	1994	Meat, Other	1
351	China	1994	Milk, Whole	15
351	China	1994	Millet	6
351	China	1994	Molluscs, Other	12
351	China	1994	Mutton & Goat Meat	3
351	China	1994	Nuts	2
351	China	1994	Oats	1
351	China	1994	Offals, Edible	5
351	China	1994	Onions	15
351	China	1994	Oranges, Mandarines	14
351	China	1994	Palm Oil	3
351	China	1994	Peas	2
351	China	1994	Pelagic Fish	2
351	China	1994	Pigmeat	65
351	China	1994	Pineapples	2
351	China	1994	Potatoes	34
351	China	1994	Poultry Meat	16
351	China	1994	Pulses, Other	2
351	China	1994	Rape and Mustard Oil	5
351	China	1994	Rice (Milled Equivalent)	204
351	China	1994	Roots, Other	2
351	China	1994	Rye	1
351	China	1994	Sorghum	6
351	China	1994	Soyabean Oil	3
351	China	1994	Soyabeans	15
351	China	1994	Sugar Cane	1
351	China	1994	Sugar, Non-Centrifugal	1
351	China	1994	Sugar, Refined Equivalent	14
351	China	1994	Sunflowerseed Oil	1
351	China	1994	Sweet Potatoes	130
351	China	1994	Tea	1
351	China	1994	Tomatoes	24
351	China	1994	Vegetables, Other	342
351	China	1994	Wheat	214
351	China	1994	Wine	1
351	China	1995	Apples	25
351	China	1995	Aquatic Plants	9
351	China	1995	Bananas	7
351	China	1995	Barley	2

FAO country code	Country	Reference year	Food item	Gross food supply (g *capita*$^{-1}$ d^{-1})
351	China	1995	Beans	1
351	China	1995	Beer	35
351	China	1995	Beverages, Alcoholic	21
351	China	1995	Beverages, Fermented	2
351	China	1995	Bovine Meat	6
351	China	1995	Cassava	4
351	China	1995	Cephalopods	1
351	China	1995	Cereals, Other	2
351	China	1995	Citrus, Other	1
351	China	1995	Cottonseed Oil	2
351	China	1995	Crustaceans	5
351	China	1995	Demersal Fish	6
351	China	1995	Eggs	34
351	China	1995	Fats, Animals, Raw	4
351	China	1995	Freshwater Fish	21
351	China	1995	Fruits, Other	31
351	China	1995	Grapefruit	1
351	China	1995	Grapes	2
351	China	1995	Groundnut Oil	3
351	China	1995	Groundnuts (Shelled Eq)	5
351	China	1995	Maize	19
351	China	1995	Marine Fish, Other	7
351	China	1995	Meat, Other	2
351	China	1995	Milk, Whole	16
351	China	1995	Millet	3
351	China	1995	Molluscs, Other	15
351	China	1995	Mutton & Goat Meat	4
351	China	1995	Nuts	2
351	China	1995	Oats	1
351	China	1995	Offals, Edible	5
351	China	1995	Onions	16
351	China	1995	Oranges, Mandarines	17
351	China	1995	Palm Oil	3
351	China	1995	Peas	2
351	China	1995	Pelagic Fish	1
351	China	1995	Pigmeat	66
351	China	1995	Pineapples	2
351	China	1995	Potatoes	40
351	China	1995	Poultry Meat	19
351	China	1995	Pulses, Other	2
351	China	1995	Rape and Mustard Oil	5
351	China	1995	Rice (Milled Equivalent)	210
351	China	1995	Roots, Other	3
351	China	1995	Rye	1
351	China	1995	Sorghum	4
351	China	1995	Soyabean Oil	3
351	China	1995	Soyabeans	13
351	China	1995	Sugar, Non-Centrifugal	1
351	China	1995	Sugar, Refined Equivalent	17
351	China	1995	Sunflowerseed Oil	1
351	China	1995	Sweet Potatoes	137
351	China	1995	Tea	1
351	China	1995	Tomatoes	26
351	China	1995	Vegetables, Other	365
351	China	1995	Wheat	215
351	China	1995	Wine	2
351	China	1996	Apples	30
351	China	1996	Aquatic Animals, Others	1
351	China	1996	Aquatic Plants	10
351	China	1996	Bananas	6
351	China	1996	Barley	2
351	China	1996	Beans	1
351	China	1996	Beer	38
351	China	1996	Beverages, Alcoholic	16
351	China	1996	Beverages, Fermented	2
351	China	1996	Bovine Meat	7
351	China	1996	Cassava	3
351	China	1996	Cephalopods	1
351	China	1996	Cereals, Other	1
351	China	1996	Citrus, Other	1
351	China	1996	Cottonseed Oil	1
351	China	1996	Crustaceans	6
351	China	1996	Demersal Fish	6
351	China	1996	Eggs	39
351	China	1996	Fats, Animals, Raw	4
351	China	1996	Freshwater Fish	24
351	China	1996	Fruits, Other	34
351	China	1996	Grapefruit	1
351	China	1996	Grapes	2
351	China	1996	Groundnut Oil	3
351	China	1996	Groundnuts (Shelled Eq)	5
351	China	1996	Maize	21
351	China	1996	Marine Fish, Other	9
351	China	1996	Meat, Other	2
351	China	1996	Milk, Whole	17
351	China	1996	Millet	4
351	China	1996	Molluscs, Other	14
351	China	1996	Mutton & Goat Meat	4
351	China	1996	Nuts	2
351	China	1996	Oats	1
351	China	1996	Offals, Edible	5
351	China	1996	Onions	19
351	China	1996	Oranges, Mandarines	17
351	China	1996	Palm Oil	2
351	China	1996	Peas	2
351	China	1996	Pelagic Fish	1
351	China	1996	Pigmeat	66

FAO country code	Country	Reference year	Food item	Gross food supply (g *capita*$^{-1}$ d^{-1})
351	China	1996	Pineapples	2
351	China	1996	Potatoes	45
351	China	1996	Poultry Meat	19
351	China	1996	Pulses, Other	2
351	China	1996	Rape and Mustard Oil	5
351	China	1996	Rice (Milled Equivalent)	211
351	China	1996	Roots, Other	3
351	China	1996	Rye	1
351	China	1996	Sorghum	5
351	China	1996	Soyabean Oil	4
351	China	1996	Soyabeans	13
351	China	1996	Sugar, Non-Centrifugal	1
351	China	1996	Sugar, Refined Equivalent	19
351	China	1996	Sunflowerseed Oil	1
351	China	1996	Sweet Potatoes	119
351	China	1996	Tea	1
351	China	1996	Tomatoes	31
351	China	1996	Vegetables, Other	401
351	China	1996	Wheat	213
351	China	1996	Wine	2
351	China	1997	Apples	29
351	China	1997	Aquatic Animals, Others	1
351	China	1997	Aquatic Plants	10
351	China	1997	Bananas	7
351	China	1997	Barley	2
351	China	1997	Beans	1
351	China	1997	Beer	42
351	China	1997	Beverages, Alcoholic	16
351	China	1997	Beverages, Fermented	2
351	China	1997	Bovine Meat	9
351	China	1997	Cassava	3
351	China	1997	Cephalopods	1
351	China	1997	Cereals, Other	3
351	China	1997	Citrus, Other	1
351	China	1997	Coconuts - Incl Copra	1
351	China	1997	Cottonseed Oil	1
351	China	1997	Crustaceans	6
351	China	1997	Demersal Fish	6
351	China	1997	Eggs	38
351	China	1997	Fats, Animals, Raw	4
351	China	1997	Freshwater Fish	25
351	China	1997	Fruits, Other	39
351	China	1997	Grapefruit	1
351	China	1997	Grapes	2
351	China	1997	Groundnut Oil	3
351	China	1997	Groundnuts (Shelled Eq)	5
351	China	1997	Maize	17
351	China	1997	Marine Fish, Other	8
351	China	1997	Meat, Other	2
351	China	1997	Milk, Whole	17
351	China	1997	Millet	3
351	China	1997	Molluscs, Other	15
351	China	1997	Mutton & Goat Meat	5
351	China	1997	Nuts	2
351	China	1997	Oats	1
351	China	1997	Offals, Edible	6
351	China	1997	Onions	19
351	China	1997	Oranges, Mandarines	20
351	China	1997	Palm Oil	2
351	China	1997	Peas	1
351	China	1997	Pelagic Fish	1
351	China	1997	Pigmeat	68
351	China	1997	Pineapples	2
351	China	1997	Potatoes	66
351	China	1997	Poultry Meat	22
351	China	1997	Pulses, Other	1
351	China	1997	Rape and Mustard Oil	4
351	China	1997	Rice (Milled Equivalent)	212
351	China	1997	Roots, Other	3
351	China	1997	Rye	1
351	China	1997	Sorghum	3
351	China	1997	Soyabean Oil	4
351	China	1997	Soyabeans	16
351	China	1997	Sugar, Non-Centrifugal	1
351	China	1997	Sugar, Refined Equivalent	21
351	China	1997	Sunflowerseed Oil	1
351	China	1997	Sweet Potatoes	104
351	China	1997	Tea	1
351	China	1997	Tomatoes	32
351	China	1997	Vegetables, Other	428
351	China	1997	Wheat	211
351	China	1997	Wine	2
351	China	1998	Apples	32
351	China	1998	Aquatic Animals, Others	1
351	China	1998	Aquatic Plants	12
351	China	1998	Bananas	8
351	China	1998	Barley	2
351	China	1998	Beans	1
351	China	1998	Beer	44
351	China	1998	Beverages, Alcoholic	12
351	China	1998	Beverages, Fermented	4
351	China	1998	Bovine Meat	9
351	China	1998	Cassava	4
351	China	1998	Cephalopods	1
351	China	1998	Cereals, Other	2
351	China	1998	Citrus, Other	1
351	China	1998	Coconuts - Incl Copra	1

FAO country code	Country	Reference year	Food item	Gross food supply (g $capita^{-1}$ d^{-1})
351	China	1998	Cottonseed Oil	1
351	China	1998	Crustaceans	6
351	China	1998	Demersal Fish	6
351	China	1998	Eggs	40
351	China	1998	Fats, Animals, Raw	4
351	China	1998	Freshwater Fish	25
351	China	1998	Fruits, Other	40
351	China	1998	Grapefruit	1
351	China	1998	Grapes	2
351	China	1998	Groundnut Oil	3
351	China	1998	Groundnuts (Shelled Eq)	7
351	China	1998	Lemons, Limes	1
351	China	1998	Maize	18
351	China	1998	Marine Fish, Other	8
351	China	1998	Meat, Other	2
351	China	1998	Milk, Whole	18
351	China	1998	Millet	3
351	China	1998	Molluscs, Other	16
351	China	1998	Mutton & Goat Meat	5
351	China	1998	Nuts	2
351	China	1998	Oats	1
351	China	1998	Offals, Edible	6
351	China	1998	Onions	20
351	China	1998	Oranges, Mandarines	17
351	China	1998	Palm Oil	3
351	China	1998	Peas	1
351	China	1998	Pelagic Fish	1
351	China	1998	Pigmeat	72
351	China	1998	Pineapples	2
351	China	1998	Potatoes	81
351	China	1998	Poultry Meat	24
351	China	1998	Pulses, Other	2
351	China	1998	Rape and Mustard Oil	5
351	China	1998	Rice (Milled Equivalent)	213
351	China	1998	Roots, Other	3
351	China	1998	Rye	1
351	China	1998	Sorghum	3
351	China	1998	Soyabean Oil	4
351	China	1998	Soyabeans	13
351	China	1998	Sugar, Non-Centrifugal	1
351	China	1998	Sugar, Refined Equivalent	20
351	China	1998	Sunflowerseed Oil	1
351	China	1998	Sweet Potatoes	107
351	China	1998	Tea	1
351	China	1998	Tomatoes	33
351	China	1998	Vegetables, Other	440
351	China	1998	Wheat	210
351	China	1998	Wine	2
351	China	1999	Apples	32
351	China	1999	Aquatic Animals, Others	1
351	China	1999	Aquatic Plants	14
351	China	1999	Bananas	9
351	China	1999	Barley	1
351	China	1999	Beans	1
351	China	1999	Beer	46
351	China	1999	Beverages, Alcoholic	11
351	China	1999	Beverages, Fermented	4
351	China	1999	Bovine Meat	10
351	China	1999	Cassava	4
351	China	1999	Cephalopods	1
351	China	1999	Cereals, Other	2
351	China	1999	Citrus, Other	2
351	China	1999	Coconuts - Incl Copra	1
351	China	1999	Cottonseed Oil	2
351	China	1999	Crustaceans	6
351	China	1999	Demersal Fish	6
351	China	1999	Eggs	42
351	China	1999	Fats, Animals, Raw	5
351	China	1999	Freshwater Fish	26
351	China	1999	Fruits, Other	47
351	China	1999	Grapefruit	1
351	China	1999	Grapes	3
351	China	1999	Groundnut Oil	3
351	China	1999	Groundnuts (Shelled Eq)	7
351	China	1999	Lemons, Limes	1
351	China	1999	Maize	18
351	China	1999	Marine Fish, Other	7
351	China	1999	Meat, Other	2
351	China	1999	Milk, Whole	19
351	China	1999	Millet	2
351	China	1999	Molluscs, Other	17
351	China	1999	Mutton & Goat Meat	5
351	China	1999	Nuts	2
351	China	1999	Oats	1
351	China	1999	Offals, Edible	7
351	China	1999	Onions	22
351	China	1999	Oranges, Mandarines	20
351	China	1999	Palm Oil	3
351	China	1999	Peas	1
351	China	1999	Pelagic Fish	1
351	China	1999	Pigmeat	73
351	China	1999	Pineapples	2
351	China	1999	Potatoes	78
351	China	1999	Poultry Meat	26
351	China	1999	Pulses, Other	2
351	China	1999	Rape and Mustard Oil	5
351	China	1999	Rice (Milled Equivalent)	214

FAO country code	Country	Reference year	Food item	Gross food supply (g *capita*$^{-1}$ d^{-1})
351	China	1999	Roots, Other	3
351	China	1999	Rye	1
351	China	1999	Sorghum	3
351	China	1999	Soyabean Oil	5
351	China	1999	Soyabeans	10
351	China	1999	Sugar, Non-Centrifugal	1
351	China	1999	Sugar, Refined Equivalent	16
351	China	1999	Sunflowerseed Oil	1
351	China	1999	Sweet Potatoes	114
351	China	1999	Tea	1
351	China	1999	Tomatoes	36
351	China	1999	Vegetables, Other	488
351	China	1999	Wheat	206
351	China	1999	Wine	2
351	China	2000	Apples	31
351	China	2000	Aquatic Animals, Others	1
351	China	2000	Aquatic Plants	15
351	China	2000	Bananas	11
351	China	2000	Barley	1
351	China	2000	Beans	1
351	China	2000	Beer	48
351	China	2000	Beverages, Alcoholic	10
351	China	2000	Beverages, Fermented	5
351	China	2000	Bovine Meat	11
351	China	2000	Cassava	3
351	China	2000	Cephalopods	2
351	China	2000	Cereals, Other	2
351	China	2000	Citrus, Other	2
351	China	2000	Coconuts - Incl Copra	1
351	China	2000	Cottonseed Oil	2
351	China	2000	Crustaceans	7
351	China	2000	Demersal Fish	7
351	China	2000	Eggs	42
351	China	2000	Fats, Animals, Raw	5
351	China	2000	Freshwater Fish	26
351	China	2000	Fruits, Other	48
351	China	2000	Grapefruit	1
351	China	2000	Grapes	4
351	China	2000	Groundnut Oil	3
351	China	2000	Groundnuts (Shelled Eq)	7
351	China	2000	Lemons, Limes	1
351	China	2000	Maize	18
351	China	2000	Marine Fish, Other	6
351	China	2000	Meat, Other	2
351	China	2000	Milk, Whole	21
351	China	2000	Millet	2
351	China	2000	Molluscs, Other	17
351	China	2000	Mutton & Goat Meat	6
351	China	2000	Nuts	2
351	China	2000	Oats	1
351	China	2000	Offals, Edible	7
351	China	2000	Onions	26
351	China	2000	Oranges, Mandarines	17
351	China	2000	Palm Oil	3
351	China	2000	Peas	1
351	China	2000	Pelagic Fish	2
351	China	2000	Pigmeat	78
351	China	2000	Pineapples	2
351	China	2000	Potatoes	90
351	China	2000	Poultry Meat	28
351	China	2000	Pulses, Other	2
351	China	2000	Rape and Mustard Oil	4
351	China	2000	Rice (Milled Equivalent)	214
351	China	2000	Roots, Other	3
351	China	2000	Rye	1
351	China	2000	Sesameseed	1
351	China	2000	Sorghum	2
351	China	2000	Soyabean Oil	5
351	China	2000	Soyabeans	11
351	China	2000	Sugar, Non-Centrifugal	1
351	China	2000	Sugar, Refined Equivalent	14
351	China	2000	Sunflowerseed Oil	1
351	China	2000	Sweet Potatoes	111
351	China	2000	Tea	1
351	China	2000	Tomatoes	42
351	China	2000	Vegetables, Other	590
351	China	2000	Wheat	201
351	China	2000	Wine	2
351	China	2001	Apples	30
351	China	2001	Aquatic Animals, Others	1
351	China	2001	Aquatic Plants	15
351	China	2001	Bananas	11
351	China	2001	Barley	1
351	China	2001	Beans	1
351	China	2001	Beer	49
351	China	2001	Beverages, Alcoholic	15
351	China	2001	Beverages, Fermented	5
351	China	2001	Bovine Meat	11
351	China	2001	Cassava	3
351	China	2001	Cephalopods	1
351	China	2001	Cereals, Other	2
351	China	2001	Citrus, Other	2
351	China	2001	Coconuts - Incl Copra	1
351	China	2001	Cottonseed Oil	2
351	China	2001	Crustaceans	7
351	China	2001	Demersal Fish	7
351	China	2001	Eggs	43

FAO country code	Country	Reference year	Food item	Gross food supply (g *capita*$^{-1}$ d^{-1})
351	China	2001	Fats, Animals, Raw	5
351	China	2001	Freshwater Fish	27
351	China	2001	Fruits, Other	51
351	China	2001	Grapefruit	1
351	China	2001	Grapes	5
351	China	2001	Groundnut Oil	3
351	China	2001	Groundnuts (Shelled Eq)	7
351	China	2001	Lemons, Limes	1
351	China	2001	Maize	18
351	China	2001	Marine Fish, Other	4
351	China	2001	Meat, Other	2
351	China	2001	Milk, Whole	25
351	China	2001	Millet	2
351	China	2001	Molluscs, Other	17
351	China	2001	Mutton & Goat Meat	6
351	China	2001	Nuts	2
351	China	2001	Offals, Edible	7
351	China	2001	Onions	28
351	China	2001	Oranges, Mandarines	22
351	China	2001	Palm Oil	3
351	China	2001	Peas	1
351	China	2001	Pelagic Fish	1
351	China	2001	Pigmeat	77
351	China	2001	Pineapples	2
351	China	2001	Potatoes	87
351	China	2001	Poultry Meat	27
351	China	2001	Pulses, Other	2
351	China	2001	Rape and Mustard Oil	4
351	China	2001	Rice (Milled Equivalent)	212
351	China	2001	Roots, Other	3
351	China	2001	Sorghum	2
351	China	2001	Soyabean Oil	5
351	China	2001	Soyabeans	11
351	China	2001	Sugar, Non-Centrifugal	1
351	China	2001	Sugar, Refined Equivalent	15
351	China	2001	Sweet Potatoes	113
351	China	2001	Tea	1
351	China	2001	Tomatoes	44
351	China	2001	Vegetables, Other	616
351	China	2001	Wheat	197
351	China	2001	Wine	2
351	China	2002	Apples	27
351	China	2002	Aquatic Animals, Others	1
351	China	2002	Aquatic Plants	16
351	China	2002	Bananas	11
351	China	2002	Barley	1
351	China	2002	Beans	1
351	China	2002	Beer	51
351	China	2002	Beverages, Alcoholic	15
351	China	2002	Beverages, Fermented	5
351	China	2002	Bovine Meat	11
351	China	2002	Cassava	3
351	China	2002	Cephalopods	1
351	China	2002	Cereals, Other	2
351	China	2002	Cheese	1
351	China	2002	Citrus, Other	2
351	China	2002	Coconuts - Incl Copra	1
351	China	2002	Cottonseed Oil	2
351	China	2002	Crustaceans	7
351	China	2002	Demersal Fish	6
351	China	2002	Eggs	43
351	China	2002	Fats, Animals, Raw	5
351	China	2002	Freshwater Fish	28
351	China	2002	Fruits, Other	55
351	China	2002	Grapefruit	1
351	China	2002	Grapes	6
351	China	2002	Groundnut Oil	3
351	China	2002	Groundnuts (Shelled Eq)	7
351	China	2002	Lemons, Limes	1
351	China	2002	Maize	19
351	China	2002	Marine Fish, Other	4
351	China	2002	Meat, Other	2
351	China	2002	Milk, Whole	31
351	China	2002	Millet	2
351	China	2002	Molluscs, Other	18
351	China	2002	Mutton & Goat Meat	6
351	China	2002	Nuts	3
351	China	2002	Offals, Edible	7
351	China	2002	Onions	30
351	China	2002	Oranges, Mandarines	22
351	China	2002	Palm Oil	3
351	China	2002	Peas	1
351	China	2002	Pelagic Fish	2
351	China	2002	Pigmeat	79
351	China	2002	Pineapples	2
351	China	2002	Potatoes	96
351	China	2002	Poultry Meat	27
351	China	2002	Pulses, Other	2
351	China	2002	Rape and Mustard Oil	4
351	China	2002	Rice (Milled Equivalent)	211
351	China	2002	Roots, Other	3
351	China	2002	Sorghum	4
351	China	2002	Soyabean Oil	5
351	China	2002	Soyabeans	10
351	China	2002	Sugar, Non-Centrifugal	1
351	China	2002	Sugar, Refined Equivalent	15
351	China	2002	Sunflowerseed Oil	1

FAO country code	Country	Reference year	Food item	Gross food supply (g $capita^{-1}$ d^{-1})
351	China	2002	Sweet Potatoes	109
351	China	2002	Tea	1
351	China	2002	Tomatoes	49
351	China	2002	Vegetables, Other	657
351	China	2002	Wheat	192
351	China	2002	Wine	2
351	China	2003	Apples	29
351	China	2003	Aquatic Animals, Others	1
351	China	2003	Aquatic Plants	18
351	China	2003	Bananas	12
351	China	2003	Barley	1
351	China	2003	Beans	1
351	China	2003	Beer	53
351	China	2003	Beverages, Alcoholic	12
351	China	2003	Beverages, Fermented	5
351	China	2003	Bovine Meat	11
351	China	2003	Cassava	4
351	China	2003	Cephalopods	2
351	China	2003	Cereals, Other	1
351	China	2003	Cheese	1
351	China	2003	Citrus, Other	2
351	China	2003	Coconuts - Incl Copra	1
351	China	2003	Cottonseed Oil	2
351	China	2003	Crustaceans	7
351	China	2003	Demersal Fish	9
351	China	2003	Eggs	44
351	China	2003	Fats, Animals, Raw	5
351	China	2003	Freshwater Fish	29
351	China	2003	Fruits, Other	56
351	China	2003	Grapefruit	1
351	China	2003	Grapes	7
351	China	2003	Groundnut Oil	3
351	China	2003	Groundnuts (Shelled Eq)	6
351	China	2003	Lemons, Limes	1
351	China	2003	Maize	19
351	China	2003	Marine Fish, Other	2
351	China	2003	Meat, Other	2
351	China	2003	Milk, Whole	40
351	China	2003	Millet	2
351	China	2003	Molluscs, Other	17
351	China	2003	Mutton & Goat Meat	6
351	China	2003	Nuts	3
351	China	2003	Offals, Edible	7
351	China	2003	Onions	32
351	China	2003	Oranges, Mandarines	25
351	China	2003	Palm Oil	4
351	China	2003	Peas	1
351	China	2003	Pelagic Fish	3
351	China	2003	Pigmeat	82
351	China	2003	Pineapples	2
351	China	2003	Potatoes	96
351	China	2003	Poultry Meat	28
351	China	2003	Pulses, Other	2
351	China	2003	Rape and Mustard Oil	4
351	China	2003	Rice (Milled Equivalent)	208
351	China	2003	Roots, Other	3
351	China	2003	Sorghum	3
351	China	2003	Soyabean Oil	6
351	China	2003	Soyabeans	10
351	China	2003	Sugar, Non-Centrifugal	1
351	China	2003	Sugar, Refined Equivalent	16
351	China	2003	Sunflowerseed Oil	1
351	China	2003	Sweet Potatoes	107
351	China	2003	Tea	1
351	China	2003	Tomatoes	52
351	China	2003	Vegetables, Other	653
351	China	2003	Wheat	188
351	China	2003	Wine	3
351	China	2004	Apples	33
351	China	2004	Aquatic Animals, Others	1
351	China	2004	Aquatic Plants	20
351	China	2004	Bananas	12
351	China	2004	Barley	1
351	China	2004	Beans	1
351	China	2004	Beer	61
351	China	2004	Beverages, Alcoholic	13
351	China	2004	Beverages, Fermented	5
351	China	2004	Bovine Meat	12
351	China	2004	Cassava	4
351	China	2004	Cephalopods	2
351	China	2004	Cereals, Other	1
351	China	2004	Cheese	1
351	China	2004	Citrus, Other	2
351	China	2004	Coconuts - Incl Copra	1
351	China	2004	Cottonseed Oil	2
351	China	2004	Crustaceans	7
351	China	2004	Demersal Fish	9
351	China	2004	Eggs	45
351	China	2004	Fats, Animals, Raw	5
351	China	2004	Freshwater Fish	30
351	China	2004	Fruits, Other	63
351	China	2004	Grapefruit	1
351	China	2004	Grapes	8
351	China	2004	Groundnut Oil	2
351	China	2004	Groundnuts (Shelled Eq)	6
351	China	2004	Lemons, Limes	1
351	China	2004	Maize	19

FAO country code	Country	Reference year	Food item	Gross food supply (g $capita^{-1}$ d^{-1})
351	China	2004	Marine Fish, Other	2
351	China	2004	Meat, Other	2
351	China	2004	Milk, Whole	51
351	China	2004	Millet	2
351	China	2004	Molluscs, Other	18
351	China	2004	Mutton & Goat Meat	7
351	China	2004	Nuts	3
351	China	2004	Offals, Edible	8
351	China	2004	Onions	32
351	China	2004	Oranges, Mandarines	27
351	China	2004	Palm Oil	4
351	China	2004	Peas	1
351	China	2004	Pelagic Fish	3
351	China	2004	Pigmeat	82
351	China	2004	Pineapples	2
351	China	2004	Potatoes	108
351	China	2004	Poultry Meat	28
351	China	2004	Pulses, Other	2
351	China	2004	Rape and Mustard Oil	4
351	China	2004	Rice (Milled Equivalent)	207
351	China	2004	Roots, Other	3
351	China	2004	Sorghum	2
351	China	2004	Soyabean Oil	6
351	China	2004	Soyabeans	10
351	China	2004	Sugar, Non-Centrifugal	1
351	China	2004	Sugar, Refined Equivalent	15
351	China	2004	Sweet Potatoes	104
351	China	2004	Tea	1
351	China	2004	Tomatoes	54
351	China	2004	Vegetables, Other	659
351	China	2004	Wheat	187
351	China	2004	Wine	3
351	China	2005	Apples	32
351	China	2005	Aquatic Animals, Others	1
351	China	2005	Aquatic Plants	20
351	China	2005	Bananas	13
351	China	2005	Barley	1
351	China	2005	Beer	65
351	China	2005	Beverages, Alcoholic	13
351	China	2005	Beverages, Fermented	5
351	China	2005	Bovine Meat	12
351	China	2005	Cassava	4
351	China	2005	Cephalopods	2
351	China	2005	Cereals, Other	1
351	China	2005	Cheese	1
351	China	2005	Citrus, Other	2
351	China	2005	Coconuts - Incl Copra	1
351	China	2005	Cottonseed Oil	2
351	China	2005	Crustaceans	7
351	China	2005	Demersal Fish	9
351	China	2005	Eggs	46
351	China	2005	Fats, Animals, Raw	5
351	China	2005	Freshwater Fish	32
351	China	2005	Fruits, Other	67
351	China	2005	Grapefruit	1
351	China	2005	Grapes	8
351	China	2005	Groundnut Oil	2
351	China	2005	Groundnuts (Shelled Eq)	6
351	China	2005	Lemons, Limes	1
351	China	2005	Maize	19
351	China	2005	Marine Fish, Other	2
351	China	2005	Meat, Other	3
351	China	2005	Milk, Whole	58
351	China	2005	Millet	2
351	China	2005	Molluscs, Other	18
351	China	2005	Mutton & Goat Meat	7
351	China	2005	Nuts	4
351	China	2005	Offals, Edible	8
351	China	2005	Onions	34
351	China	2005	Oranges, Mandarines	28
351	China	2005	Palm Oil	4
351	China	2005	Peas	1
351	China	2005	Pelagic Fish	3
351	China	2005	Pigmeat	85
351	China	2005	Pineapples	2
351	China	2005	Potatoes	106
351	China	2005	Poultry Meat	29
351	China	2005	Pulses, Other	2
351	China	2005	Rape and Mustard Oil	4
351	China	2005	Rice (Milled Equivalent)	208
351	China	2005	Roots, Other	3
351	China	2005	Sorghum	2
351	China	2005	Soyabean Oil	6
351	China	2005	Soyabeans	11
351	China	2005	Sugar, Non-Centrifugal	1
351	China	2005	Sugar, Refined Equivalent	16
351	China	2005	Sweet Potatoes	103
351	China	2005	Tea	1
351	China	2005	Tomatoes	55
351	China	2005	Vegetables, Other	677
351	China	2005	Wheat	185
351	China	2005	Wine	3
351	China	2006	Apples	37
351	China	2006	Aquatic Animals, Others	1
351	China	2006	Aquatic Plants	20
351	China	2006	Bananas	13
351	China	2006	Barley	1

FAO country code	Country	Reference year	Food item	Gross food supply (g $capita^{-1}$ d^{-1})
351	China	2006	Beer	73
351	China	2006	Beverages, Alcoholic	13
351	China	2006	Beverages, Fermented	6
351	China	2006	Bovine Meat	12
351	China	2006	Cassava	5
351	China	2006	Cephalopods	2
351	China	2006	Cheese	1
351	China	2006	Citrus, Other	2
351	China	2006	Coconuts - Incl Copra	1
351	China	2006	Cottonseed Oil	2
351	China	2006	Crustaceans	7
351	China	2006	Demersal Fish	10
351	China	2006	Eggs	45
351	China	2006	Fats, Animals, Raw	5
351	China	2006	Freshwater Fish	34
351	China	2006	Fruits, Other	67
351	China	2006	Grapefruit	1
351	China	2006	Grapes	9
351	China	2006	Groundnut Oil	2
351	China	2006	Groundnuts (Shelled Eq)	4
351	China	2006	Honey	1
351	China	2006	Lemons, Limes	1
351	China	2006	Maize	18
351	China	2006	Marine Fish, Other	1
351	China	2006	Meat, Other	3
351	China	2006	Milk, Whole	66
351	China	2006	Millet	2
351	China	2006	Molluscs, Other	18
351	China	2006	Mutton & Goat Meat	7
351	China	2006	Nuts	4
351	China	2006	Offals, Edible	8
351	China	2006	Onions	35
351	China	2006	Oranges, Mandarines	32
351	China	2006	Palm Oil	5
351	China	2006	Peas	1
351	China	2006	Pelagic Fish	3
351	China	2006	Pigmeat	88
351	China	2006	Pineapples	2
351	China	2006	Potatoes	80
351	China	2006	Poultry Meat	30
351	China	2006	Pulses, Other	2
351	China	2006	Rape and Mustard Oil	5
351	China	2006	Rice (Milled Equivalent)	207
351	China	2006	Roots, Other	3
351	China	2006	Sesameseed	1
351	China	2006	Sorghum	2
351	China	2006	Soyabean Oil	7
351	China	2006	Soyabeans	11
351	China	2006	Sugar, Non-Centrifugal	1
351	China	2006	Sugar, Refined Equivalent	16
351	China	2006	Sunflowerseed Oil	1
351	China	2006	Sweet Potatoes	85
351	China	2006	Tea	2
351	China	2006	Tomatoes	56
351	China	2006	Vegetables, Other	700
351	China	2006	Wheat	184
351	China	2006	Wine	3
351	China	2007	Apples	37
351	China	2007	Aquatic Animals, Others	1
351	China	2007	Aquatic Plants	20
351	China	2007	Bananas	14
351	China	2007	Beans	1
351	China	2007	Beer	80
351	China	2007	Beverages, Alcoholic	15
351	China	2007	Beverages, Fermented	6
351	China	2007	Bovine Meat	13
351	China	2007	Cassava	5
351	China	2007	Cephalopods	3
351	China	2007	Cereals, Other	1
351	China	2007	Cheese	1
351	China	2007	Citrus, Other	7
351	China	2007	Coconuts - Incl Copra	1
351	China	2007	Cottonseed Oil	2
351	China	2007	Crustaceans	8
351	China	2007	Demersal Fish	10
351	China	2007	Eggs	47
351	China	2007	Fats, Animals, Raw	6
351	China	2007	Freshwater Fish	35
351	China	2007	Fruits, Other	70
351	China	2007	Grapefruit	4
351	China	2007	Grapes	9
351	China	2007	Groundnut Oil	2
351	China	2007	Groundnuts (Shelled Eq)	6
351	China	2007	Honey	1
351	China	2007	Lemons, Limes	2
351	China	2007	Maize	18
351	China	2007	Marine Fish, Other	1
351	China	2007	Meat, Other	3
351	China	2007	Milk, Whole	72
351	China	2007	Millet	1
351	China	2007	Molluscs, Other	18
351	China	2007	Mutton & Goat Meat	8
351	China	2007	Nuts	4
351	China	2007	Offals, Edible	8
351	China	2007	Onions	36
351	China	2007	Oranges, Mandarines	22
351	China	2007	Palm Oil	5

FAO country code	Country	Reference year	Food item	Gross food supply (g *capita*$^{-1}$ d^{-1})
351	China	2007	Peas	1
351	China	2007	Pelagic Fish	3
351	China	2007	Pigmeat	88
351	China	2007	Pineapples	2
351	China	2007	Potatoes	95
351	China	2007	Poultry Meat	32
351	China	2007	Pulses, Other	2
351	China	2007	Rape and Mustard Oil	5
351	China	2007	Rice (Milled Equivalent)	206
351	China	2007	Roots, Other	3
351	China	2007	Sesameseed	1
351	China	2007	Sorghum	1
351	China	2007	Soyabean Oil	7
351	China	2007	Soyabeans	11
351	China	2007	Sugar, Refined Equivalent	17
351	China	2007	Sweet Potatoes	72
351	China	2007	Tea	2
351	China	2007	Tomatoes	61
351	China	2007	Vegetables, Other	719
351	China	2007	Wheat	181
351	China	2007	Wine	3
351	China	2008	Apples	39
351	China	2008	Aquatic Animals, Others	2
351	China	2008	Aquatic Plants	21
351	China	2008	Bananas	14
351	China	2008	Barley	1
351	China	2008	Beer	84
351	China	2008	Beverages, Alcoholic	15
351	China	2008	Beverages, Fermented	6
351	China	2008	Bovine Meat	13
351	China	2008	Cassava	5
351	China	2008	Cephalopods	3
351	China	2008	Cereals, Other	1
351	China	2008	Cheese	1
351	China	2008	Citrus, Other	8
351	China	2008	Coconuts - Incl Copra	1
351	China	2008	Cottonseed Oil	2
351	China	2008	Crustaceans	8
351	China	2008	Demersal Fish	10
351	China	2008	Eggs	50
351	China	2008	Fats, Animals, Raw	5
351	China	2008	Freshwater Fish	37
351	China	2008	Fruits, Other	73
351	China	2008	Grapefruit	5
351	China	2008	Grapes	10
351	China	2008	Groundnut Oil	2
351	China	2008	Groundnuts (Shelled Eq)	7
351	China	2008	Honey	1
351	China	2008	Lemons, Limes	7
351	China	2008	Maize	19
351	China	2008	Marine Fish, Other	2
351	China	2008	Meat, Other	3
351	China	2008	Milk, Whole	72
351	China	2008	Millet	1
351	China	2008	Molluscs, Other	19
351	China	2008	Mutton & Goat Meat	8
351	China	2008	Nuts	5
351	China	2008	Offals, Edible	9
351	China	2008	Onions	36
351	China	2008	Oranges, Mandarines	25
351	China	2008	Palm Oil	5
351	China	2008	Peas	1
351	China	2008	Pelagic Fish	3
351	China	2008	Pigmeat	93
351	China	2008	Pineapples	2
351	China	2008	Potatoes	103
351	China	2008	Poultry Meat	34
351	China	2008	Pulses, Other	2
351	China	2008	Rape and Mustard Oil	5
351	China	2008	Rice (Milled Equivalent)	211
351	China	2008	Roots, Other	3
351	China	2008	Sesameseed	1
351	China	2008	Sorghum	2
351	China	2008	Soyabean Oil	6
351	China	2008	Soyabeans	11
351	China	2008	Sugar, Non-Centrifugal	1
351	China	2008	Sugar, Refined Equivalent	16
351	China	2008	Sweet Potatoes	76
351	China	2008	Tea	2
351	China	2008	Tomatoes	68
351	China	2008	Vegetables, Other	751
351	China	2008	Wheat	176
351	China	2008	Wine	3
351	China	2009	Apples	41
351	China	2009	Aquatic Animals, Others	2
351	China	2009	Aquatic Plants	21
351	China	2009	Bananas	15
351	China	2009	Barley	1
351	China	2009	Beer	84
351	China	2009	Beverages, Alcoholic	15
351	China	2009	Beverages, Fermented	6
351	China	2009	Bovine Meat	13
351	China	2009	Cassava	5
351	China	2009	Cephalopods	2
351	China	2009	Cereals, Other	1
351	China	2009	Cheese	1
351	China	2009	Citrus, Other	9

FAO country code	Country	Reference year	Food item	Gross food supply (g *capita*$^{-1}$ d^{-1})
351	China	2009	Coconuts - Incl Copra	1
351	China	2009	Cottonseed Oil	2
351	China	2009	Crustaceans	9
351	China	2009	Demersal Fish	9
351	China	2009	Eggs	50
351	China	2009	Fats, Animals, Raw	5
351	China	2009	Freshwater Fish	39
351	China	2009	Fruits, Other	78
351	China	2009	Grapefruit	5
351	China	2009	Grapes	11
351	China	2009	Groundnut Oil	2
351	China	2009	Groundnuts (Shelled Eq)	7
351	China	2009	Honey	1
351	China	2009	Lemons, Limes	7
351	China	2009	Maize	19
351	China	2009	Marine Fish, Other	4
351	China	2009	Meat, Other	3
351	China	2009	Milk, Whole	75
351	China	2009	Millet	1
351	China	2009	Molluscs, Other	21
351	China	2009	Mutton & Goat Meat	8
351	China	2009	Nuts	6
351	China	2009	Offals, Edible	9
351	China	2009	Onions	37
351	China	2009	Oranges, Mandarines	27
351	China	2009	Palm Oil	6
351	China	2009	Peas	1
351	China	2009	Pelagic Fish	2
351	China	2009	Pigmeat	96
351	China	2009	Pineapples	3
351	China	2009	Potatoes	103
351	China	2009	Poultry Meat	35
351	China	2009	Pulses, Other	2
351	China	2009	Rape and Mustard Oil	5
351	China	2009	Rice (Milled Equivalent)	212
351	China	2009	Roots, Other	3
351	China	2009	Sesameseed	1
351	China	2009	Sorghum	2
351	China	2009	Soyabean Oil	6
351	China	2009	Soyabeans	11
351	China	2009	Sugar, Non-Centrifugal	1
351	China	2009	Sugar, Refined Equivalent	16
351	China	2009	Sweet Potatoes	69
351	China	2009	Tea	2
351	China	2009	Tomatoes	77
351	China	2009	Vegetables, Other	754
351	China	2009	Wheat	170
351	China	2009	Wine	3
351	China	2010	Apples	44
351	China	2010	Aquatic Animals, Others	2
351	China	2010	Aquatic Plants	23
351	China	2010	Bananas	17
351	China	2010	Beer	90
351	China	2010	Beverages, Alcoholic	14
351	China	2010	Beverages, Fermented	6
351	China	2010	Bovine Meat	13
351	China	2010	Cassava	5
351	China	2010	Cephalopods	2
351	China	2010	Cereals, Other	1
351	China	2010	Cheese	1
351	China	2010	Citrus, Other	10
351	China	2010	Coconut Oil	1
351	China	2010	Coconuts - Incl Copra	1
351	China	2010	Cottonseed Oil	1
351	China	2010	Crustaceans	9
351	China	2010	Demersal Fish	9
351	China	2010	Eggs	50
351	China	2010	Fats, Animals, Raw	5
351	China	2010	Freshwater Fish	41
351	China	2010	Fruits, Other	80
351	China	2010	Grapefruit	5
351	China	2010	Grapes	12
351	China	2010	Groundnut Oil	2
351	China	2010	Groundnuts (Shelled Eq)	9
351	China	2010	Honey	1
351	China	2010	Lemons, Limes	4
351	China	2010	Maize	20
351	China	2010	Marine Fish, Other	4
351	China	2010	Meat, Other	3
351	China	2010	Milk, Whole	78
351	China	2010	Millet	1
351	China	2010	Molluscs, Other	22
351	China	2010	Mutton & Goat Meat	8
351	China	2010	Nuts	7
351	China	2010	Offals, Edible	9
351	China	2010	Onions	37
351	China	2010	Oranges, Mandarines	31
351	China	2010	Palm Oil	5
351	China	2010	Peas	1
351	China	2010	Pelagic Fish	2
351	China	2010	Pigmeat	98
351	China	2010	Pineapples	2
351	China	2010	Potatoes	113
351	China	2010	Poultry Meat	35
351	China	2010	Pulses, Other	2
351	China	2010	Rape and Mustard Oil	5
351	China	2010	Rice (Milled Equivalent)	212

FAO country code	Country	Reference year	Food item	Gross food supply (g $capita^{-1}$ d^{-1})
351	China	2010	Roots, Other	3
351	China	2010	Sesameseed	1
351	China	2010	Sorghum	2
351	China	2010	Soyabean Oil	6
351	China	2010	Soyabeans	11
351	China	2010	Sugar, Non-Centrifugal	1
351	China	2010	Sugar, Refined Equivalent	16
351	China	2010	Sweet Potatoes	65
351	China	2010	Tea	2
351	China	2010	Tomatoes	78
351	China	2010	Vegetables, Other	781
351	China	2010	Wheat	171
351	China	2010	Wine	4
351	China	2011	Apples	48
351	China	2011	Aquatic Animals, Others	2
351	China	2011	Aquatic Plants	23
351	China	2011	Bananas	18
351	China	2011	Beer	96
351	China	2011	Beverages, Alcoholic	14
351	China	2011	Beverages, Fermented	6
351	China	2011	Bovine Meat	13
351	China	2011	Cassava	5
351	China	2011	Cephalopods	2
351	China	2011	Cereals, Other	1
351	China	2011	Cheese	1
351	China	2011	Citrus, Other	11
351	China	2011	Coconuts - Incl Copra	1
351	China	2011	Cottonseed Oil	1
351	China	2011	Crustaceans	10
351	China	2011	Demersal Fish	9
351	China	2011	Eggs	51
351	China	2011	Fats, Animals, Raw	5
351	China	2011	Freshwater Fish	42
351	China	2011	Fruits, Other	85
351	China	2011	Grapefruit	6
351	China	2011	Grapes	13
351	China	2011	Groundnut Oil	2
351	China	2011	Groundnuts (Shelled Eq)	8
351	China	2011	Honey	1
351	China	2011	Lemons, Limes	4
351	China	2011	Maize	20
351	China	2011	Marine Fish, Other	4
351	China	2011	Meat, Other	3
351	China	2011	Milk, Whole	79
351	China	2011	Millet	1
351	China	2011	Molluscs, Other	22
351	China	2011	Mutton & Goat Meat	8
351	China	2011	Nuts	7
351	China	2011	Offals, Edible	10
351	China	2011	Onions	38
351	China	2011	Oranges, Mandarines	35
351	China	2011	Palm Oil	6
351	China	2011	Peas	1
351	China	2011	Pelagic Fish	1
351	China	2011	Pigmeat	98
351	China	2011	Pineapples	2
351	China	2011	Potatoes	113
351	China	2011	Poultry Meat	35
351	China	2011	Pulses, Other	2
351	China	2011	Rape and Mustard Oil	4
351	China	2011	Rice (Milled Equivalent)	217
351	China	2011	Roots, Other	3
351	China	2011	Sesameseed	1
351	China	2011	Sorghum	2
351	China	2011	Soyabean Oil	6
351	China	2011	Soyabeans	11
351	China	2011	Sugar, Non-Centrifugal	1
351	China	2011	Sugar, Refined Equivalent	17
351	China	2011	Sweet Potatoes	66
351	China	2011	Tea	3
351	China	2011	Tomatoes	80
351	China	2011	Vegetables, Other	793
351	China	2011	Wheat	173
351	China	2011	Wine	4
44	Colombia	1992	Apples	1
44	Colombia	1992	Bananas	24
44	Colombia	1992	Barley	2
44	Colombia	1992	Beans	9
44	Colombia	1992	Beer	48
44	Colombia	1992	Beverages, Alcoholic	10
44	Colombia	1992	Bovine Meat	46
44	Colombia	1992	Butter, Ghee	1
44	Colombia	1992	Cassava	103
44	Colombia	1992	Cheese	4
44	Colombia	1992	Cocoa Beans	4
44	Colombia	1992	Coconuts - Incl Copra	6
44	Colombia	1992	Coffee	7
44	Colombia	1992	Cottonseed Oil	2
44	Colombia	1992	Eggs	18
44	Colombia	1992	Fats, Animals, Raw	3
44	Colombia	1992	Freshwater Fish	4
44	Colombia	1992	Fruits, Other	52
44	Colombia	1992	Grapes	3
44	Colombia	1992	Maize	96
44	Colombia	1992	Maize Germ Oil	2
44	Colombia	1992	Marine Fish, Other	1
44	Colombia	1992	Meat, Other	1

FAO countrycode	Country	Reference year	Food item	Gross food supply (g $capita^{-1}$ d^{-1})
44	Colombia	1992	Milk, Whole	252
44	Colombia	1992	Mutton & Goat Meat	1
44	Colombia	1992	Oats	2
44	Colombia	1992	Offals, Edible	9
44	Colombia	1992	Oilcrops, Other	4
44	Colombia	1992	Onions	12
44	Colombia	1992	Oranges, Mandarines	18
44	Colombia	1992	Palm Oil	12
44	Colombia	1992	Palmkernel Oil	2
44	Colombia	1992	Peas	6
44	Colombia	1992	Pelagic Fish	3
44	Colombia	1992	Pigmeat	11
44	Colombia	1992	Pineapples	20
44	Colombia	1992	Plantains	151
44	Colombia	1992	Potatoes	122
44	Colombia	1992	Poultry Meat	33
44	Colombia	1992	Pulses, Other	5
44	Colombia	1992	Rice (Milled Equivalent)	83
44	Colombia	1992	Roots, Other	5
44	Colombia	1992	Soyabean Oil	7
44	Colombia	1992	Sugar, Non-Centrifugal	33
44	Colombia	1992	Sugar, Refined Equivalent	89
44	Colombia	1992	Sunflowerseed Oil	1
44	Colombia	1992	Tomatoes	23
44	Colombia	1992	Vegetables, Other	28
44	Colombia	1992	Wheat	72
44	Colombia	1992	Yams	5
44	Colombia	1993	Apples	2
44	Colombia	1993	Bananas	25
44	Colombia	1993	Barley	5
44	Colombia	1993	Beans	9
44	Colombia	1993	Beer	86
44	Colombia	1993	Beverages, Alcoholic	11
44	Colombia	1993	Bovine Meat	46
44	Colombia	1993	Butter, Ghee	1
44	Colombia	1993	Cassava	106
44	Colombia	1993	Cheese	4
44	Colombia	1993	Cocoa Beans	3
44	Colombia	1993	Coconuts - Incl Copra	5
44	Colombia	1993	Coffee	7
44	Colombia	1993	Cottonseed Oil	1
44	Colombia	1993	Eggs	19
44	Colombia	1993	Fats, Animals, Raw	3
44	Colombia	1993	Freshwater Fish	4
44	Colombia	1993	Fruits, Other	42
44	Colombia	1993	Grapes	2
44	Colombia	1993	Maize	102
44	Colombia	1993	Maize Germ Oil	2
44	Colombia	1993	Marine Fish, Other	1
44	Colombia	1993	Meat, Other	1
44	Colombia	1993	Milk, Whole	264
44	Colombia	1993	Mutton & Goat Meat	1
44	Colombia	1993	Oats	1
44	Colombia	1993	Offals, Edible	9
44	Colombia	1993	Oilcrops, Other	5
44	Colombia	1993	Onions	14
44	Colombia	1993	Oranges, Mandarines	23
44	Colombia	1993	Palm Oil	14
44	Colombia	1993	Palmkernel Oil	2
44	Colombia	1993	Peas	4
44	Colombia	1993	Pelagic Fish	5
44	Colombia	1993	Pigmeat	11
44	Colombia	1993	Pineapples	22
44	Colombia	1993	Plantains	145
44	Colombia	1993	Potatoes	155
44	Colombia	1993	Poultry Meat	35
44	Colombia	1993	Pulses, Other	5
44	Colombia	1993	Rice (Milled Equivalent)	75
44	Colombia	1993	Roots, Other	5
44	Colombia	1993	Soyabean Oil	9
44	Colombia	1993	Sugar, Non-Centrifugal	34
44	Colombia	1993	Sugar, Refined Equivalent	83
44	Colombia	1993	Sunflowerseed Oil	1
44	Colombia	1993	Tomatoes	20
44	Colombia	1993	Vegetables, Other	31
44	Colombia	1993	Wheat	73
44	Colombia	1993	Yams	5
44	Colombia	1994	Apples	4
44	Colombia	1994	Bananas	20
44	Colombia	1994	Barley	6
44	Colombia	1994	Beans	11
44	Colombia	1994	Beer	76
44	Colombia	1994	Beverages, Alcoholic	9
44	Colombia	1994	Bovine Meat	48
44	Colombia	1994	Butter, Ghee	1
44	Colombia	1994	Cassava	99
44	Colombia	1994	Cheese	4
44	Colombia	1994	Cocoa Beans	3
44	Colombia	1994	Coconuts - Incl Copra	6
44	Colombia	1994	Coffee	7
44	Colombia	1994	Cottonseed Oil	1
44	Colombia	1994	Eggs	19
44	Colombia	1994	Fats, Animals, Raw	4
44	Colombia	1994	Freshwater Fish	4
44	Colombia	1994	Fruits, Other	42
44	Colombia	1994	Grapes	3
44	Colombia	1994	Maize	100

FAO countrycode	Country	Reference year	Food item	Gross food supply (g $capita^{-1}$ d^{-1})
44	Colombia	1994	Maize Germ Oil	2
44	Colombia	1994	Meat, Other	1
44	Colombia	1994	Milk, Whole	270
44	Colombia	1994	Mutton & Goat Meat	1
44	Colombia	1994	Oats	1
44	Colombia	1994	Offals, Edible	9
44	Colombia	1994	Oilcrops, Other	8
44	Colombia	1994	Onions	15
44	Colombia	1994	Oranges, Mandarines	26
44	Colombia	1994	Palm Oil	14
44	Colombia	1994	Palmkernel Oil	2
44	Colombia	1994	Peas	3
44	Colombia	1994	Pelagic Fish	3
44	Colombia	1994	Pigmeat	10
44	Colombia	1994	Pineapples	22
44	Colombia	1994	Plantains	134
44	Colombia	1994	Potatoes	157
44	Colombia	1994	Poultry Meat	39
44	Colombia	1994	Pulses, Other	4
44	Colombia	1994	Rice (Milled Equivalent)	83
44	Colombia	1994	Roots, Other	6
44	Colombia	1994	Soyabean Oil	8
44	Colombia	1994	Sugar, Non-Centrifugal	33
44	Colombia	1994	Sugar, Refined Equivalent	83
44	Colombia	1994	Sunflowerseed Oil	1
44	Colombia	1994	Sweeteners, Other	1
44	Colombia	1994	Tomatoes	22
44	Colombia	1994	Vegetables, Other	32
44	Colombia	1994	Wheat	72
44	Colombia	1994	Yams	8
44	Colombia	1995	Apples	4
44	Colombia	1995	Bananas	22
44	Colombia	1995	Barley	5
44	Colombia	1995	Beans	12
44	Colombia	1995	Beer	80
44	Colombia	1995	Beverages, Alcoholic	10
44	Colombia	1995	Bovine Meat	52
44	Colombia	1995	Butter, Ghee	1
44	Colombia	1995	Cassava	98
44	Colombia	1995	Cheese	4
44	Colombia	1995	Cocoa Beans	4
44	Colombia	1995	Coconuts - Incl Copra	7
44	Colombia	1995	Coffee	6
44	Colombia	1995	Cottonseed Oil	1
44	Colombia	1995	Eggs	20
44	Colombia	1995	Fats, Animals, Raw	4
44	Colombia	1995	Freshwater Fish	4
44	Colombia	1995	Fruits, Other	47
44	Colombia	1995	Grapes	2
44	Colombia	1995	Maize	99
44	Colombia	1995	Maize Germ Oil	2
44	Colombia	1995	Marine Fish, Other	1
44	Colombia	1995	Meat, Other	1
44	Colombia	1995	Milk, Whole	288
44	Colombia	1995	Mutton & Goat Meat	1
44	Colombia	1995	Oats	1
44	Colombia	1995	Offals, Edible	10
44	Colombia	1995	Oilcrops, Other	8
44	Colombia	1995	Onions	31
44	Colombia	1995	Oranges, Mandarines	28
44	Colombia	1995	Palm Oil	14
44	Colombia	1995	Palmkernel Oil	2
44	Colombia	1995	Peas	3
44	Colombia	1995	Pelagic Fish	8
44	Colombia	1995	Pigmeat	11
44	Colombia	1995	Pineapples	22
44	Colombia	1995	Plantains	154
44	Colombia	1995	Potatoes	154
44	Colombia	1995	Poultry Meat	43
44	Colombia	1995	Pulses, Other	3
44	Colombia	1995	Rice (Milled Equivalent)	77
44	Colombia	1995	Roots, Other	5
44	Colombia	1995	Soyabean Oil	8
44	Colombia	1995	Sugar, Non-Centrifugal	33
44	Colombia	1995	Sugar, Refined Equivalent	84
44	Colombia	1995	Sunflowerseed Oil	1
44	Colombia	1995	Sweeteners, Other	1
44	Colombia	1995	Tomatoes	19
44	Colombia	1995	Vegetables, Other	32
44	Colombia	1995	Wheat	79
44	Colombia	1995	Yams	8
44	Colombia	1996	Apples	4
44	Colombia	1996	Bananas	2
44	Colombia	1996	Barley	5
44	Colombia	1996	Beans	11
44	Colombia	1996	Beer	75
44	Colombia	1996	Beverages, Alcoholic	9
44	Colombia	1996	Bovine Meat	53
44	Colombia	1996	Butter, Ghee	1
44	Colombia	1996	Cassava	101
44	Colombia	1996	Cheese	4
44	Colombia	1996	Cocoa Beans	3
44	Colombia	1996	Coconuts - Incl Copra	8
44	Colombia	1996	Coffee	3
44	Colombia	1996	Cottonseed Oil	2
44	Colombia	1996	Eggs	21
44	Colombia	1996	Fats, Animals, Raw	4

FAO country code	Country	Reference year	Food item	Gross food supply (g *capita*$^{-1}$ d^{-1})
44	Colombia	1996	Freshwater Fish	4
44	Colombia	1996	Fruits, Other	50
44	Colombia	1996	Grapes	2
44	Colombia	1996	Groundnuts (Shelled Eq)	1
44	Colombia	1996	Maize	105
44	Colombia	1996	Maize Germ Oil	2
44	Colombia	1996	Marine Fish, Other	1
44	Colombia	1996	Meat, Other	1
44	Colombia	1996	Milk, Whole	300
44	Colombia	1996	Mutton & Goat Meat	1
44	Colombia	1996	Oats	1
44	Colombia	1996	Offals, Edible	10
44	Colombia	1996	Oilcrops, Other	8
44	Colombia	1996	Onions	46
44	Colombia	1996	Oranges, Mandarines	27
44	Colombia	1996	Palm Oil	13
44	Colombia	1996	Palmkernel Oil	2
44	Colombia	1996	Peas	5
44	Colombia	1996	Pelagic Fish	8
44	Colombia	1996	Pigmeat	10
44	Colombia	1996	Pineapples	18
44	Colombia	1996	Plantains	135
44	Colombia	1996	Potatoes	147
44	Colombia	1996	Poultry Meat	36
44	Colombia	1996	Pulses, Other	3
44	Colombia	1996	Rice (Milled Equivalent)	80
44	Colombia	1996	Roots, Other	4
44	Colombia	1996	Soyabean Oil	10
44	Colombia	1996	Soyabeans	3
44	Colombia	1996	Sugar, Non-Centrifugal	32
44	Colombia	1996	Sugar, Refined Equivalent	78
44	Colombia	1996	Sunflowerseed Oil	1
44	Colombia	1996	Sweeteners, Other	1
44	Colombia	1996	Tomatoes	21
44	Colombia	1996	Vegetables, Other	31
44	Colombia	1996	Wheat	76
44	Colombia	1996	Yams	13
44	Colombia	1997	Apples	4
44	Colombia	1997	Bananas	3
44	Colombia	1997	Barley	5
44	Colombia	1997	Beans	10
44	Colombia	1997	Beer	75
44	Colombia	1997	Beverages, Alcoholic	9
44	Colombia	1997	Bovine Meat	54
44	Colombia	1997	Butter, Ghee	1
44	Colombia	1997	Cassava	101
44	Colombia	1997	Cheese	4
44	Colombia	1997	Cocoa Beans	3
44	Colombia	1997	Coconuts - Incl Copra	6
44	Colombia	1997	Coffee	3
44	Colombia	1997	Cottonseed Oil	1
44	Colombia	1997	Eggs	22
44	Colombia	1997	Fats, Animals, Raw	3
44	Colombia	1997	Freshwater Fish	4
44	Colombia	1997	Fruits, Other	54
44	Colombia	1997	Grapes	3
44	Colombia	1997	Maize	104
44	Colombia	1997	Maize Germ Oil	2
44	Colombia	1997	Marine Fish, Other	1
44	Colombia	1997	Meat, Other	1
44	Colombia	1997	Milk, Whole	306
44	Colombia	1997	Mutton & Goat Meat	1
44	Colombia	1997	Oats	2
44	Colombia	1997	Offals, Edible	9
44	Colombia	1997	Oilcrops, Other	8
44	Colombia	1997	Onions	56
44	Colombia	1997	Oranges, Mandarines	28
44	Colombia	1997	Palm Oil	14
44	Colombia	1997	Palmkernel Oil	2
44	Colombia	1997	Peas	5
44	Colombia	1997	Pelagic Fish	8
44	Colombia	1997	Pigmeat	8
44	Colombia	1997	Pineapples	17
44	Colombia	1997	Plantains	134
44	Colombia	1997	Potatoes	142
44	Colombia	1997	Poultry Meat	33
44	Colombia	1997	Pulses, Other	5
44	Colombia	1997	Rice (Milled Equivalent)	82
44	Colombia	1997	Roots, Other	6
44	Colombia	1997	Soyabean Oil	9
44	Colombia	1997	Soyabeans	2
44	Colombia	1997	Sugar Beet	1
44	Colombia	1997	Sugar, Non-Centrifugal	32
44	Colombia	1997	Sugar, Refined Equivalent	80
44	Colombia	1997	Sunflowerseed Oil	2
44	Colombia	1997	Sweeteners, Other	1
44	Colombia	1997	Tomatoes	22
44	Colombia	1997	Vegetables, Other	34
44	Colombia	1997	Wheat	77
44	Colombia	1997	Yams	8
44	Colombia	1998	Apples	4
44	Colombia	1998	Bananas	7
44	Colombia	1998	Barley	4
44	Colombia	1998	Beans	10
44	Colombia	1998	Beer	73
44	Colombia	1998	Beverages, Alcoholic	8
44	Colombia	1998	Bovine Meat	53

FAO countrycode	Country	Reference year	Food item	Gross food supply (g *capita*$^{-1}$ d^{-1})
44	Colombia	1998	Butter, Ghee	1
44	Colombia	1998	Cassava	99
44	Colombia	1998	Cheese	4
44	Colombia	1998	Cocoa Beans	4
44	Colombia	1998	Coconuts - Incl Copra	5
44	Colombia	1998	Coffee	3
44	Colombia	1998	Cottonseed Oil	1
44	Colombia	1998	Demersal Fish	1
44	Colombia	1998	Eggs	23
44	Colombia	1998	Fats, Animals, Raw	3
44	Colombia	1998	Freshwater Fish	5
44	Colombia	1998	Fruits, Other	47
44	Colombia	1998	Grapes	2
44	Colombia	1998	Maize	104
44	Colombia	1998	Maize Germ Oil	2
44	Colombia	1998	Marine Fish, Other	1
44	Colombia	1998	Meat, Other	1
44	Colombia	1998	Milk, Whole	314
44	Colombia	1998	Mutton & Goat Meat	1
44	Colombia	1998	Oats	1
44	Colombia	1998	Offals, Edible	9
44	Colombia	1998	Oilcrops, Other	7
44	Colombia	1998	Onions	47
44	Colombia	1998	Oranges, Mandarines	20
44	Colombia	1998	Palm Oil	15
44	Colombia	1998	Palmkernel Oil	2
44	Colombia	1998	Peas	5
44	Colombia	1998	Pelagic Fish	7
44	Colombia	1998	Pigmeat	6
44	Colombia	1998	Pineapples	6
44	Colombia	1998	Plantains	119
44	Colombia	1998	Potatoes	130
44	Colombia	1998	Poultry Meat	38
44	Colombia	1998	Pulses, Other	4
44	Colombia	1998	Rice (Milled Equivalent)	83
44	Colombia	1998	Roots, Other	5
44	Colombia	1998	Soyabean Oil	10
44	Colombia	1998	Soyabeans	2
44	Colombia	1998	Sugar Beet	1
44	Colombia	1998	Sugar, Non-Centrifugal	33
44	Colombia	1998	Sugar, Refined Equivalent	80
44	Colombia	1998	Sunflowerseed Oil	2
44	Colombia	1998	Sweeteners, Other	1
44	Colombia	1998	Tomatoes	20
44	Colombia	1998	Vegetables, Other	32
44	Colombia	1998	Wheat	78
44	Colombia	1998	Yams	10
44	Colombia	1999	Apples	4
44	Colombia	1999	Bananas	2
44	Colombia	1999	Barley	2
44	Colombia	1999	Beans	10
44	Colombia	1999	Beer	65
44	Colombia	1999	Beverages, Alcoholic	10
44	Colombia	1999	Bovine Meat	49
44	Colombia	1999	Butter, Ghee	1
44	Colombia	1999	Cassava	99
44	Colombia	1999	Cheese	4
44	Colombia	1999	Citrus, Other	46
44	Colombia	1999	Cocoa Beans	4
44	Colombia	1999	Coconuts - Incl Copra	7
44	Colombia	1999	Coffee	2
44	Colombia	1999	Cottonseed Oil	1
44	Colombia	1999	Eggs	23
44	Colombia	1999	Fats, Animals, Raw	3
44	Colombia	1999	Freshwater Fish	5
44	Colombia	1999	Fruits, Other	61
44	Colombia	1999	Grapes	2
44	Colombia	1999	Lemons, Limes	4
44	Colombia	1999	Maize	106
44	Colombia	1999	Maize Germ Oil	2
44	Colombia	1999	Meat, Other	1
44	Colombia	1999	Milk, Whole	299
44	Colombia	1999	Mutton & Goat Meat	1
44	Colombia	1999	Oats	2
44	Colombia	1999	Offals, Edible	9
44	Colombia	1999	Oilcrops, Other	4
44	Colombia	1999	Onions	33
44	Colombia	1999	Oranges, Mandarines	25
44	Colombia	1999	Palm Oil	16
44	Colombia	1999	Palmkernel Oil	2
44	Colombia	1999	Peas	5
44	Colombia	1999	Pelagic Fish	5
44	Colombia	1999	Pigmeat	8
44	Colombia	1999	Pineapples	20
44	Colombia	1999	Plantains	109
44	Colombia	1999	Potatoes	142
44	Colombia	1999	Poultry Meat	36
44	Colombia	1999	Pulses, Other	5
44	Colombia	1999	Rice (Milled Equivalent)	86
44	Colombia	1999	Roots, Other	3
44	Colombia	1999	Soyabean Oil	10
44	Colombia	1999	Soyabeans	2
44	Colombia	1999	Spices, Other	1
44	Colombia	1999	Sugar Beet	1
44	Colombia	1999	Sugar, Non-Centrifugal	32
44	Colombia	1999	Sugar, Refined Equivalent	87
44	Colombia	1999	Sunflowerseed Oil	1

FAO country code	Country	Reference year	Food item	Gross food supply (g *capita*$^{-1}$ d^{-1})
44	Colombia	1999	Tomatoes	25
44	Colombia	1999	Vegetables, Other	38
44	Colombia	1999	Wheat	78
44	Colombia	1999	Yams	13
44	Colombia	2000	Apples	4
44	Colombia	2000	Bananas	7
44	Colombia	2000	Barley	2
44	Colombia	2000	Beans	10
44	Colombia	2000	Beer	92
44	Colombia	2000	Beverages, Alcoholic	9
44	Colombia	2000	Bovine Meat	50
44	Colombia	2000	Butter, Ghee	1
44	Colombia	2000	Cassava	99
44	Colombia	2000	Cheese	3
44	Colombia	2000	Citrus, Other	46
44	Colombia	2000	Cocoa Beans	3
44	Colombia	2000	Coconuts - Incl Copra	6
44	Colombia	2000	Coffee	3
44	Colombia	2000	Cottonseed Oil	1
44	Colombia	2000	Eggs	23
44	Colombia	2000	Fats, Animals, Raw	4
44	Colombia	2000	Freshwater Fish	6
44	Colombia	2000	Fruits, Other	63
44	Colombia	2000	Grapes	2
44	Colombia	2000	Lemons, Limes	4
44	Colombia	2000	Maize	107
44	Colombia	2000	Maize Germ Oil	2
44	Colombia	2000	Marine Fish, Other	1
44	Colombia	2000	Meat, Other	1
44	Colombia	2000	Milk, Whole	320
44	Colombia	2000	Mutton & Goat Meat	1
44	Colombia	2000	Oats	2
44	Colombia	2000	Offals, Edible	9
44	Colombia	2000	Oilcrops, Other	4
44	Colombia	2000	Onions	42
44	Colombia	2000	Oranges, Mandarines	26
44	Colombia	2000	Palm Oil	16
44	Colombia	2000	Palmkernel Oil	2
44	Colombia	2000	Peas	6
44	Colombia	2000	Pelagic Fish	6
44	Colombia	2000	Pigmeat	8
44	Colombia	2000	Pineapples	17
44	Colombia	2000	Plantains	126
44	Colombia	2000	Potatoes	66
44	Colombia	2000	Poultry Meat	37
44	Colombia	2000	Pulses, Other	5
44	Colombia	2000	Rice (Milled Equivalent)	98
44	Colombia	2000	Roots, Other	3
44	Colombia	2000	Soyabean Oil	13
44	Colombia	2000	Soyabeans	3
44	Colombia	2000	Spices, Other	1
44	Colombia	2000	Sugar Beet	1
44	Colombia	2000	Sugar, Non-Centrifugal	32
44	Colombia	2000	Sugar, Refined Equivalent	82
44	Colombia	2000	Sunflowerseed Oil	2
44	Colombia	2000	Sweeteners, Other	1
44	Colombia	2000	Tomatoes	26
44	Colombia	2000	Vegetables, Other	40
44	Colombia	2000	Wheat	78
44	Colombia	2000	Yams	15
44	Colombia	2001	Apples	4
44	Colombia	2001	Bananas	7
44	Colombia	2001	Barley	4
44	Colombia	2001	Beans	10
44	Colombia	2001	Beer	94
44	Colombia	2001	Beverages, Alcoholic	10
44	Colombia	2001	Bovine Meat	46
44	Colombia	2001	Butter, Ghee	1
44	Colombia	2001	Cassava	101
44	Colombia	2001	Cheese	3
44	Colombia	2001	Citrus, Other	47
44	Colombia	2001	Cocoa Beans	2
44	Colombia	2001	Coconuts - Incl Copra	6
44	Colombia	2001	Coffee	3
44	Colombia	2001	Cottonseed Oil	1
44	Colombia	2001	Eggs	25
44	Colombia	2001	Fats, Animals, Raw	4
44	Colombia	2001	Freshwater Fish	5
44	Colombia	2001	Fruits, Other	62
44	Colombia	2001	Grapes	2
44	Colombia	2001	Lemons, Limes	6
44	Colombia	2001	Maize	106
44	Colombia	2001	Maize Germ Oil	2
44	Colombia	2001	Marine Fish, Other	1
44	Colombia	2001	Meat, Other	1
44	Colombia	2001	Milk, Whole	321
44	Colombia	2001	Mutton & Goat Meat	1
44	Colombia	2001	Oats	2
44	Colombia	2001	Offals, Edible	8
44	Colombia	2001	Oilcrops, Other	2
44	Colombia	2001	Onions	24
44	Colombia	2001	Oranges, Mandarines	18
44	Colombia	2001	Palm Oil	16
44	Colombia	2001	Palmkernel Oil	2
44	Colombia	2001	Peas	6
44	Colombia	2001	Pelagic Fish	8
44	Colombia	2001	Pigmeat	7

FAO country code	Country	Reference year	Food item	Gross food supply (g *capita*$^{-1}$ d^{-1})
44	Colombia	2001	Pineapples	16
44	Colombia	2001	Plantains	138
44	Colombia	2001	Potatoes	84
44	Colombia	2001	Poultry Meat	43
44	Colombia	2001	Pulses, Other	5
44	Colombia	2001	Rice (Milled Equivalent)	97
44	Colombia	2001	Roots, Other	2
44	Colombia	2001	Soyabean Oil	13
44	Colombia	2001	Soyabeans	4
44	Colombia	2001	Spices, Other	1
44	Colombia	2001	Sugar Beet	1
44	Colombia	2001	Sugar, Non-Centrifugal	35
44	Colombia	2001	Sugar, Refined Equivalent	78
44	Colombia	2001	Sunflowerseed Oil	2
44	Colombia	2001	Sweeteners, Other	1
44	Colombia	2001	Tomatoes	27
44	Colombia	2001	Vegetables, Other	43
44	Colombia	2001	Wheat	82
44	Colombia	2001	Yams	15
44	Colombia	2002	Apples	4
44	Colombia	2002	Bananas	5
44	Colombia	2002	Barley	4
44	Colombia	2002	Beans	10
44	Colombia	2002	Beer	81
44	Colombia	2002	Beverages, Alcoholic	10
44	Colombia	2002	Bovine Meat	44
44	Colombia	2002	Butter, Ghee	1
44	Colombia	2002	Cassava	85
44	Colombia	2002	Cheese	4
44	Colombia	2002	Citrus, Other	47
44	Colombia	2002	Cocoa Beans	3
44	Colombia	2002	Coconuts - Incl Copra	6
44	Colombia	2002	Coffee	4
44	Colombia	2002	Cottonseed Oil	1
44	Colombia	2002	Demersal Fish	1
44	Colombia	2002	Eggs	24
44	Colombia	2002	Fats, Animals, Raw	4
44	Colombia	2002	Freshwater Fish	5
44	Colombia	2002	Fruits, Other	62
44	Colombia	2002	Grapes	2
44	Colombia	2002	Lemons, Limes	5
44	Colombia	2002	Maize	107
44	Colombia	2002	Maize Germ Oil	2
44	Colombia	2002	Marine Fish, Other	1
44	Colombia	2002	Meat, Other	1
44	Colombia	2002	Milk, Whole	327
44	Colombia	2002	Mutton & Goat Meat	1
44	Colombia	2002	Oats	2
44	Colombia	2002	Offals, Edible	8
44	Colombia	2002	Oilcrops, Other	1
44	Colombia	2002	Onions	25
44	Colombia	2002	Oranges, Mandarines	21
44	Colombia	2002	Palm Oil	16
44	Colombia	2002	Palmkernel Oil	2
44	Colombia	2002	Peas	6
44	Colombia	2002	Pelagic Fish	7
44	Colombia	2002	Pigmeat	8
44	Colombia	2002	Pineapples	18
44	Colombia	2002	Plantains	137
44	Colombia	2002	Potatoes	81
44	Colombia	2002	Poultry Meat	45
44	Colombia	2002	Pulses, Other	5
44	Colombia	2002	Rice (Milled Equivalent)	91
44	Colombia	2002	Roots, Other	4
44	Colombia	2002	Soyabean Oil	13
44	Colombia	2002	Soyabeans	5
44	Colombia	2002	Spices, Other	1
44	Colombia	2002	Sugar Beet	1
44	Colombia	2002	Sugar, Non-Centrifugal	33
44	Colombia	2002	Sugar, Refined Equivalent	79
44	Colombia	2002	Sunflowerseed Oil	1
44	Colombia	2002	Tomatoes	28
44	Colombia	2002	Vegetables, Other	43
44	Colombia	2002	Wheat	81
44	Colombia	2002	Wine	1
44	Colombia	2002	Yams	14
44	Colombia	2003	Apples	3
44	Colombia	2003	Bananas	13
44	Colombia	2003	Barley	4
44	Colombia	2003	Beans	8
44	Colombia	2003	Beer	99
44	Colombia	2003	Beverages, Alcoholic	10
44	Colombia	2003	Bovine Meat	41
44	Colombia	2003	Butter, Ghee	1
44	Colombia	2003	Cassava	78
44	Colombia	2003	Cheese	3
44	Colombia	2003	Citrus, Other	46
44	Colombia	2003	Cocoa Beans	2
44	Colombia	2003	Coconuts - Incl Copra	7
44	Colombia	2003	Coffee	6
44	Colombia	2003	Demersal Fish	1
44	Colombia	2003	Eggs	26
44	Colombia	2003	Fats, Animals, Raw	4
44	Colombia	2003	Freshwater Fish	5
44	Colombia	2003	Fruits, Other	65
44	Colombia	2003	Grapes	3
44	Colombia	2003	Lemons, Limes	5

FAO countrycode	Country	Reference year	Food item	Gross food supply (g *capita*$^{-1}$ d^{-1})
44	Colombia	2003	Maize	107
44	Colombia	2003	Maize Germ Oil	2
44	Colombia	2003	Marine Fish, Other	1
44	Colombia	2003	Meat, Other	1
44	Colombia	2003	Milk, Whole	324
44	Colombia	2003	Mutton & Goat Meat	1
44	Colombia	2003	Oats	2
44	Colombia	2003	Offals, Edible	7
44	Colombia	2003	Onions	32
44	Colombia	2003	Oranges, Mandarines	8
44	Colombia	2003	Palm Oil	16
44	Colombia	2003	Palmkernel Oil	2
44	Colombia	2003	Peas	6
44	Colombia	2003	Pelagic Fish	8
44	Colombia	2003	Pigmeat	8
44	Colombia	2003	Pineapples	19
44	Colombia	2003	Plantains	129
44	Colombia	2003	Potatoes	100
44	Colombia	2003	Poultry Meat	46
44	Colombia	2003	Pulses, Other	5
44	Colombia	2003	Rice (Milled Equivalent)	92
44	Colombia	2003	Roots, Other	6
44	Colombia	2003	Soyabean Oil	13
44	Colombia	2003	Soyabeans	3
44	Colombia	2003	Spices, Other	1
44	Colombia	2003	Sugar Beet	1
44	Colombia	2003	Sugar, Non-Centrifugal	34
44	Colombia	2003	Sugar, Refined Equivalent	80
44	Colombia	2003	Sunflowerseed Oil	2
44	Colombia	2003	Tomatoes	26
44	Colombia	2003	Vegetables, Other	46
44	Colombia	2003	Wheat	82
44	Colombia	2003	Wine	1
44	Colombia	2003	Yams	16
44	Colombia	2004	Apples	4
44	Colombia	2004	Bananas	13
44	Colombia	2004	Barley	4
44	Colombia	2004	Beans	8
44	Colombia	2004	Beer	103
44	Colombia	2004	Beverages, Alcoholic	9
44	Colombia	2004	Bovine Meat	44
44	Colombia	2004	Butter, Ghee	1
44	Colombia	2004	Cassava	78
44	Colombia	2004	Cheese	3
44	Colombia	2004	Citrus, Other	46
44	Colombia	2004	Cocoa Beans	2
44	Colombia	2004	Coconuts - Incl Copra	6
44	Colombia	2004	Coffee	5
44	Colombia	2004	Cottonseed Oil	1
44	Colombia	2004	Demersal Fish	1
44	Colombia	2004	Eggs	25
44	Colombia	2004	Fats, Animals, Raw	4
44	Colombia	2004	Freshwater Fish	5
44	Colombia	2004	Fruits, Other	66
44	Colombia	2004	Grapes	3
44	Colombia	2004	Lemons, Limes	6
44	Colombia	2004	Maize	107
44	Colombia	2004	Maize Germ Oil	2
44	Colombia	2004	Marine Fish, Other	1
44	Colombia	2004	Meat, Other	2
44	Colombia	2004	Milk, Whole	306
44	Colombia	2004	Mutton & Goat Meat	1
44	Colombia	2004	Oats	2
44	Colombia	2004	Offals, Edible	8
44	Colombia	2004	Onions	27
44	Colombia	2004	Oranges, Mandarines	10
44	Colombia	2004	Palm Oil	15
44	Colombia	2004	Palmkernel Oil	3
44	Colombia	2004	Peas	5
44	Colombia	2004	Pelagic Fish	7
44	Colombia	2004	Pigmeat	9
44	Colombia	2004	Pineapples	19
44	Colombia	2004	Plantains	134
44	Colombia	2004	Potatoes	93
44	Colombia	2004	Poultry Meat	47
44	Colombia	2004	Pulses, Other	5
44	Colombia	2004	Rice (Milled Equivalent)	92
44	Colombia	2004	Roots, Other	6
44	Colombia	2004	Soyabean Oil	13
44	Colombia	2004	Soyabeans	1
44	Colombia	2004	Spices, Other	1
44	Colombia	2004	Sugar Beet	1
44	Colombia	2004	Sugar, Non-Centrifugal	33
44	Colombia	2004	Sugar, Refined Equivalent	81
44	Colombia	2004	Sunflowerseed Oil	2
44	Colombia	2004	Tomatoes	27
44	Colombia	2004	Vegetables, Other	47
44	Colombia	2004	Wheat	82
44	Colombia	2004	Wine	1
44	Colombia	2004	Yams	17
44	Colombia	2005	Apples	4
44	Colombia	2005	Bananas	9
44	Colombia	2005	Barley	4
44	Colombia	2005	Beans	10
44	Colombia	2005	Beer	105
44	Colombia	2005	Beverages, Alcoholic	10
44	Colombia	2005	Bovine Meat	49

FAO country code	Country	Reference year	Food item	Gross food supply (g $capita^{-1}$ d^{-1})
44	Colombia	2005	Butter, Ghee	1
44	Colombia	2005	Cassava	74
44	Colombia	2005	Cheese	3
44	Colombia	2005	Citrus, Other	47
44	Colombia	2005	Cocoa Beans	2
44	Colombia	2005	Coconuts - Incl Copra	7
44	Colombia	2005	Coffee	5
44	Colombia	2005	Cottonseed Oil	1
44	Colombia	2005	Demersal Fish	1
44	Colombia	2005	Eggs	27
44	Colombia	2005	Fats, Animals, Raw	4
44	Colombia	2005	Freshwater Fish	4
44	Colombia	2005	Fruits, Other	67
44	Colombia	2005	Grapes	3
44	Colombia	2005	Lemons, Limes	6
44	Colombia	2005	Maize	102
44	Colombia	2005	Maize Germ Oil	2
44	Colombia	2005	Marine Fish, Other	2
44	Colombia	2005	Meat, Other	2
44	Colombia	2005	Milk, Whole	293
44	Colombia	2005	Mutton & Goat Meat	1
44	Colombia	2005	Oats	2
44	Colombia	2005	Offals, Edible	9
44	Colombia	2005	Onions	27
44	Colombia	2005	Oranges, Mandarines	11
44	Colombia	2005	Palm Oil	16
44	Colombia	2005	Palmkernel Oil	3
44	Colombia	2005	Peas	5
44	Colombia	2005	Pelagic Fish	8
44	Colombia	2005	Pigmeat	8
44	Colombia	2005	Pineapples	18
44	Colombia	2005	Plantains	129
44	Colombia	2005	Potatoes	81
44	Colombia	2005	Poultry Meat	50
44	Colombia	2005	Pulses, Other	5
44	Colombia	2005	Rice (Milled Equivalent)	89
44	Colombia	2005	Roots, Other	6
44	Colombia	2005	Soyabean Oil	13
44	Colombia	2005	Soyabeans	1
44	Colombia	2005	Spices, Other	1
44	Colombia	2005	Sugar Beet	1
44	Colombia	2005	Sugar, Non-Centrifugal	31
44	Colombia	2005	Sugar, Refined Equivalent	81
44	Colombia	2005	Sunflowerseed Oil	1
44	Colombia	2005	Tomatoes	27
44	Colombia	2005	Vegetables, Other	47
44	Colombia	2005	Wheat	80
44	Colombia	2005	Wine	1
44	Colombia	2005	Yams	15
44	Colombia	2006	Apples	4
44	Colombia	2006	Bananas	16
44	Colombia	2006	Barley	4
44	Colombia	2006	Beans	9
44	Colombia	2006	Beer	112
44	Colombia	2006	Beverages, Alcoholic	7
44	Colombia	2006	Bovine Meat	49
44	Colombia	2006	Butter, Ghee	1
44	Colombia	2006	Cassava	75
44	Colombia	2006	Cheese	3
44	Colombia	2006	Citrus, Other	46
44	Colombia	2006	Cocoa Beans	3
44	Colombia	2006	Coconuts - Incl Copra	7
44	Colombia	2006	Coffee	5
44	Colombia	2006	Cottonseed Oil	1
44	Colombia	2006	Demersal Fish	1
44	Colombia	2006	Eggs	29
44	Colombia	2006	Fats, Animals, Raw	4
44	Colombia	2006	Freshwater Fish	5
44	Colombia	2006	Fruits, Other	74
44	Colombia	2006	Grapes	3
44	Colombia	2006	Lemons, Limes	6
44	Colombia	2006	Maize	99
44	Colombia	2006	Maize Germ Oil	2
44	Colombia	2006	Marine Fish, Other	1
44	Colombia	2006	Meat, Other	2
44	Colombia	2006	Milk, Whole	320
44	Colombia	2006	Mutton & Goat Meat	1
44	Colombia	2006	Oats	2
44	Colombia	2006	Offals, Edible	9
44	Colombia	2006	Onions	17
44	Colombia	2006	Oranges, Mandarines	29
44	Colombia	2006	Palm Oil	16
44	Colombia	2006	Palmkernel Oil	3
44	Colombia	2006	Peas	4
44	Colombia	2006	Pelagic Fish	9
44	Colombia	2006	Pigmeat	10
44	Colombia	2006	Pineapples	19
44	Colombia	2006	Plantains	131
44	Colombia	2006	Potatoes	96
44	Colombia	2006	Poultry Meat	55
44	Colombia	2006	Pulses, Other	6
44	Colombia	2006	Rice (Milled Equivalent)	86
44	Colombia	2006	Roots, Other	6
44	Colombia	2006	Soyabean Oil	13
44	Colombia	2006	Soyabeans	1
44	Colombia	2006	Spices, Other	1
44	Colombia	2006	Sugar Beet	1

FAO country code	Country	Reference year	Food item	Gross food supply (g *capita*$^{-1}$ d^{-1})
44	Colombia	2006	Sugar, Non-Centrifugal	31
44	Colombia	2006	Sugar, Refined Equivalent	71
44	Colombia	2006	Sunflowerseed Oil	1
44	Colombia	2006	Tomatoes	29
44	Colombia	2006	Vegetables, Other	45
44	Colombia	2006	Wheat	83
44	Colombia	2006	Wine	1
44	Colombia	2006	Yams	14
44	Colombia	2007	Apples	4
44	Colombia	2007	Bananas	9
44	Colombia	2007	Barley	4
44	Colombia	2007	Beans	7
44	Colombia	2007	Beer	112
44	Colombia	2007	Beverages, Alcoholic	7
44	Colombia	2007	Bovine Meat	47
44	Colombia	2007	Butter, Ghee	1
44	Colombia	2007	Cassava	93
44	Colombia	2007	Cheese	3
44	Colombia	2007	Citrus, Other	46
44	Colombia	2007	Cocoa Beans	3
44	Colombia	2007	Coconuts - Incl Copra	6
44	Colombia	2007	Coffee	3
44	Colombia	2007	Cottonseed Oil	1
44	Colombia	2007	Demersal Fish	1
44	Colombia	2007	Eggs	26
44	Colombia	2007	Fats, Animals, Raw	4
44	Colombia	2007	Freshwater Fish	5
44	Colombia	2007	Fruits, Other	78
44	Colombia	2007	Grapes	4
44	Colombia	2007	Lemons, Limes	6
44	Colombia	2007	Maize	98
44	Colombia	2007	Maize Germ Oil	2
44	Colombia	2007	Marine Fish, Other	2
44	Colombia	2007	Meat, Other	2
44	Colombia	2007	Milk, Whole	312
44	Colombia	2007	Mutton & Goat Meat	1
44	Colombia	2007	Oats	2
44	Colombia	2007	Offals, Edible	9
44	Colombia	2007	Onions	18
44	Colombia	2007	Oranges, Mandarines	24
44	Colombia	2007	Palm Oil	16
44	Colombia	2007	Palmkernel Oil	3
44	Colombia	2007	Peas	5
44	Colombia	2007	Pelagic Fish	9
44	Colombia	2007	Pigmeat	12
44	Colombia	2007	Pineapples	20
44	Colombia	2007	Plantains	136
44	Colombia	2007	Potatoes	130
44	Colombia	2007	Poultry Meat	59
44	Colombia	2007	Pulses, Other	6
44	Colombia	2007	Rice (Milled Equivalent)	83
44	Colombia	2007	Roots, Other	6
44	Colombia	2007	Soyabean Oil	13
44	Colombia	2007	Soyabeans	1
44	Colombia	2007	Spices, Other	1
44	Colombia	2007	Sugar Beet	1
44	Colombia	2007	Sugar, Non-Centrifugal	30
44	Colombia	2007	Sugar, Refined Equivalent	71
44	Colombia	2007	Sunflowerseed Oil	2
44	Colombia	2007	Sweeteners, Other	1
44	Colombia	2007	Tomatoes	30
44	Colombia	2007	Vegetables, Other	47
44	Colombia	2007	Wheat	79
44	Colombia	2007	Wine	1
44	Colombia	2007	Yams	14
44	Colombia	2008	Apples	4
44	Colombia	2008	Bananas	16
44	Colombia	2008	Barley	4
44	Colombia	2008	Beans	8
44	Colombia	2008	Beer	115
44	Colombia	2008	Beverages, Alcoholic	6
44	Colombia	2008	Bovine Meat	45
44	Colombia	2008	Butter, Ghee	1
44	Colombia	2008	Cassava	88
44	Colombia	2008	Cheese	3
44	Colombia	2008	Citrus, Other	47
44	Colombia	2008	Cocoa Beans	3
44	Colombia	2008	Coconuts - Incl Copra	6
44	Colombia	2008	Coffee	2
44	Colombia	2008	Cottonseed Oil	1
44	Colombia	2008	Demersal Fish	1
44	Colombia	2008	Eggs	28
44	Colombia	2008	Fats, Animals, Raw	4
44	Colombia	2008	Freshwater Fish	5
44	Colombia	2008	Fruits, Other	75
44	Colombia	2008	Grapes	4
44	Colombia	2008	Groundnuts (Shelled Eq)	1
44	Colombia	2008	Lemons, Limes	4
44	Colombia	2008	Maize	93
44	Colombia	2008	Maize Germ Oil	2
44	Colombia	2008	Marine Fish, Other	1
44	Colombia	2008	Meat, Other	2
44	Colombia	2008	Milk, Whole	344
44	Colombia	2008	Mutton & Goat Meat	1
44	Colombia	2008	Oats	1
44	Colombia	2008	Offals, Edible	9
44	Colombia	2008	Onions	19

FAO country code	Country	Reference year	Food item	Gross food supply (g $capita^{-1}$ d^{-1})
44	Colombia	2008	Oranges, Mandarines	18
44	Colombia	2008	Palm Oil	16
44	Colombia	2008	Palmkernel Oil	3
44	Colombia	2008	Peas	5
44	Colombia	2008	Pelagic Fish	9
44	Colombia	2008	Pigmeat	11
44	Colombia	2008	Pineapples	20
44	Colombia	2008	Plantains	140
44	Colombia	2008	Potatoes	108
44	Colombia	2008	Poultry Meat	64
44	Colombia	2008	Pulses, Other	5
44	Colombia	2008	Rice (Milled Equivalent)	84
44	Colombia	2008	Roots, Other	5
44	Colombia	2008	Soyabean Oil	13
44	Colombia	2008	Soyabeans	1
44	Colombia	2008	Spices, Other	1
44	Colombia	2008	Sugar Beet	2
44	Colombia	2008	Sugar, Non-Centrifugal	30
44	Colombia	2008	Sugar, Refined Equivalent	75
44	Colombia	2008	Sunflowerseed Oil	1
44	Colombia	2008	Sweeteners, Other	1
44	Colombia	2008	Tomatoes	31
44	Colombia	2008	Vegetables, Other	54
44	Colombia	2008	Wheat	84
44	Colombia	2008	Wine	1
44	Colombia	2008	Yams	14
44	Colombia	2009	Apples	5
44	Colombia	2009	Bananas	7
44	Colombia	2009	Barley	4
44	Colombia	2009	Beans	9
44	Colombia	2009	Beer	115
44	Colombia	2009	Beverages, Alcoholic	6
44	Colombia	2009	Bovine Meat	41
44	Colombia	2009	Butter, Ghee	1
44	Colombia	2009	Cassava	106
44	Colombia	2009	Cheese	3
44	Colombia	2009	Citrus, Other	44
44	Colombia	2009	Cocoa Beans	3
44	Colombia	2009	Coconuts - Incl Copra	5
44	Colombia	2009	Coffee	1
44	Colombia	2009	Demersal Fish	1
44	Colombia	2009	Eggs	30
44	Colombia	2009	Fats, Animals, Raw	3
44	Colombia	2009	Freshwater Fish	5
44	Colombia	2009	Fruits, Other	75
44	Colombia	2009	Grapes	4
44	Colombia	2009	Groundnuts (Shelled Eq)	1
44	Colombia	2009	Lemons, Limes	5
44	Colombia	2009	Maize	84
44	Colombia	2009	Maize Germ Oil	1
44	Colombia	2009	Marine Fish, Other	1
44	Colombia	2009	Meat, Other	3
44	Colombia	2009	Milk, Whole	281
44	Colombia	2009	Mutton & Goat Meat	1
44	Colombia	2009	Offals, Edible	8
44	Colombia	2009	Onions	24
44	Colombia	2009	Oranges, Mandarines	17
44	Colombia	2009	Palm Oil	16
44	Colombia	2009	Palmkernel Oil	3
44	Colombia	2009	Peas	5
44	Colombia	2009	Pelagic Fish	8
44	Colombia	2009	Pigmeat	11
44	Colombia	2009	Pineapples	15
44	Colombia	2009	Plantains	127
44	Colombia	2009	Potatoes	102
44	Colombia	2009	Poultry Meat	63
44	Colombia	2009	Pulses, Other	4
44	Colombia	2009	Rape and Mustard Oil	1
44	Colombia	2009	Rice (Milled Equivalent)	84
44	Colombia	2009	Roots, Other	3
44	Colombia	2009	Soyabean Oil	13
44	Colombia	2009	Soyabeans	2
44	Colombia	2009	Spices, Other	1
44	Colombia	2009	Sugar Beet	1
44	Colombia	2009	Sugar, Non-Centrifugal	27
44	Colombia	2009	Sugar, Refined Equivalent	75
44	Colombia	2009	Sunflowerseed Oil	1
44	Colombia	2009	Tomatoes	32
44	Colombia	2009	Vegetables, Other	51
44	Colombia	2009	Wheat	81
44	Colombia	2009	Wine	1
44	Colombia	2009	Yams	20
44	Colombia	2010	Apples	5
44	Colombia	2010	Bananas	17
44	Colombia	2010	Barley	3
44	Colombia	2010	Beans	9
44	Colombia	2010	Beer	109
44	Colombia	2010	Beverages, Alcoholic	6
44	Colombia	2010	Bovine Meat	44
44	Colombia	2010	Butter, Ghee	1
44	Colombia	2010	Cassava	105
44	Colombia	2010	Cheese	3
44	Colombia	2010	Citrus, Other	43
44	Colombia	2010	Cocoa Beans	3
44	Colombia	2010	Coconuts - Incl Copra	6
44	Colombia	2010	Coffee	4
44	Colombia	2010	Eggs	30

FAO country code	Country	Reference year	Food item	Gross food supply (g *capita*$^{-1}$ d^{-1})
44	Colombia	2010	Fats, Animals, Raw	3
44	Colombia	2010	Freshwater Fish	5
44	Colombia	2010	Fruits, Other	71
44	Colombia	2010	Grapes	4
44	Colombia	2010	Groundnuts (Shelled Eq)	1
44	Colombia	2010	Lemons, Limes	5
44	Colombia	2010	Maize	77
44	Colombia	2010	Maize Germ Oil	1
44	Colombia	2010	Marine Fish, Other	1
44	Colombia	2010	Meat, Other	3
44	Colombia	2010	Milk, Whole	276
44	Colombia	2010	Mutton & Goat Meat	1
44	Colombia	2010	Offals, Edible	8
44	Colombia	2010	Onions	27
44	Colombia	2010	Oranges, Mandarines	19
44	Colombia	2010	Palm Oil	26
44	Colombia	2010	Palmkernel Oil	3
44	Colombia	2010	Peas	5
44	Colombia	2010	Pelagic Fish	8
44	Colombia	2010	Pigmeat	12
44	Colombia	2010	Pineapples	20
44	Colombia	2010	Plantains	135
44	Colombia	2010	Potatoes	83
44	Colombia	2010	Poultry Meat	66
44	Colombia	2010	Pulses, Other	5
44	Colombia	2010	Rape and Mustard Oil	1
44	Colombia	2010	Rice (Milled Equivalent)	80
44	Colombia	2010	Roots, Other	3
44	Colombia	2010	Soyabean Oil	15
44	Colombia	2010	Soyabeans	2
44	Colombia	2010	Spices, Other	1
44	Colombia	2010	Sugar Beet	1
44	Colombia	2010	Sugar, Non-Centrifugal	26
44	Colombia	2010	Sugar, Refined Equivalent	58
44	Colombia	2010	Sunflowerseed Oil	2
44	Colombia	2010	Sweeteners, Other	1
44	Colombia	2010	Tomatoes	32
44	Colombia	2010	Vegetables, Other	47
44	Colombia	2010	Wheat	84
44	Colombia	2010	Wine	1
44	Colombia	2010	Yams	19
44	Colombia	2011	Apples	6
44	Colombia	2011	Bananas	10
44	Colombia	2011	Barley	3
44	Colombia	2011	Beans	9
44	Colombia	2011	Beer	111
44	Colombia	2011	Beverages, Alcoholic	6
44	Colombia	2011	Bovine Meat	47
44	Colombia	2011	Butter, Ghee	1
44	Colombia	2011	Cassava	100
44	Colombia	2011	Cheese	3
44	Colombia	2011	Citrus, Other	44
44	Colombia	2011	Cocoa Beans	3
44	Colombia	2011	Coconuts - Incl Copra	6
44	Colombia	2011	Coffee	2
44	Colombia	2011	Cottonseed Oil	1
44	Colombia	2011	Eggs	33
44	Colombia	2011	Fats, Animals, Raw	3
44	Colombia	2011	Freshwater Fish	5
44	Colombia	2011	Fruits, Other	74
44	Colombia	2011	Grapes	3
44	Colombia	2011	Groundnuts (Shelled Eq)	1
44	Colombia	2011	Lemons, Limes	5
44	Colombia	2011	Maize	79
44	Colombia	2011	Maize Germ Oil	1
44	Colombia	2011	Marine Fish, Other	1
44	Colombia	2011	Meat, Other	3
44	Colombia	2011	Milk, Whole	276
44	Colombia	2011	Mutton & Goat Meat	2
44	Colombia	2011	Offals, Edible	8
44	Colombia	2011	Onions	24
44	Colombia	2011	Oranges, Mandarines	22
44	Colombia	2011	Palm Oil	27
44	Colombia	2011	Palmkernel Oil	3
44	Colombia	2011	Peas	4
44	Colombia	2011	Pelagic Fish	8
44	Colombia	2011	Pigmeat	14
44	Colombia	2011	Pineapples	22
44	Colombia	2011	Plantains	134
44	Colombia	2011	Potatoes	75
44	Colombia	2011	Poultry Meat	66
44	Colombia	2011	Pulses, Other	5
44	Colombia	2011	Rape and Mustard Oil	1
44	Colombia	2011	Rice (Milled Equivalent)	77
44	Colombia	2011	Roots, Other	4
44	Colombia	2011	Soyabean Oil	15
44	Colombia	2011	Spices, Other	1
44	Colombia	2011	Sugar Beet	1
44	Colombia	2011	Sugar, Non-Centrifugal	26
44	Colombia	2011	Sugar, Refined Equivalent	64
44	Colombia	2011	Sunflowerseed Oil	1
44	Colombia	2011	Sweeteners, Other	1
44	Colombia	2011	Tomatoes	36
44	Colombia	2011	Vegetables, Other	46
44	Colombia	2011	Wheat	90
44	Colombia	2011	Wine	1
44	Colombia	2011	Yams	18

FAO country code	Country	Reference year	Food item	Gross food supply (g $capita^{-1}$ d^{-1})
46	Congo	1992	Bananas	37
46	Congo	1992	Beans	6
46	Congo	1992	Beer	77
46	Congo	1992	Bovine Meat	35
46	Congo	1992	Butter, Ghee	1
46	Congo	1992	Cassava	641
46	Congo	1992	Cereals, Other	4
46	Congo	1992	Demersal Fish	21
46	Congo	1992	Eggs	1
46	Congo	1992	Freshwater Fish	23
46	Congo	1992	Fruits, Other	27
46	Congo	1992	Groundnut Oil	2
46	Congo	1992	Groundnuts (Shelled Eq)	14
46	Congo	1992	Maize	7
46	Congo	1992	Marine Fish, Other	1
46	Congo	1992	Meat, Other	13
46	Congo	1992	Milk, Whole	21
46	Congo	1992	Mutton & Goat Meat	1
46	Congo	1992	Offals, Edible	1
46	Congo	1992	Oilcrops Oil, Other	9
46	Congo	1992	Oilcrops, Other	3
46	Congo	1992	Onions	5
46	Congo	1992	Oranges, Mandarines	3
46	Congo	1992	Palm Oil	16
46	Congo	1992	Peas	4
46	Congo	1992	Pelagic Fish	39
46	Congo	1992	Pigmeat	7
46	Congo	1992	Pineapples	5
46	Congo	1992	Plantains	72
46	Congo	1992	Potatoes	3
46	Congo	1992	Poultry Meat	20
46	Congo	1992	Rape and Mustard Oil	2
46	Congo	1992	Rice (Milled Equivalent)	33
46	Congo	1992	Roots, Other	34
46	Congo	1992	Soyabean Oil	2
46	Congo	1992	Sugar Cane	5
46	Congo	1992	Sugar, Refined Equivalent	17
46	Congo	1992	Sunflowerseed Oil	1
46	Congo	1992	Sweet Potatoes	10
46	Congo	1992	Tomatoes	12
46	Congo	1992	Vegetables, Other	79
46	Congo	1992	Wheat	98
46	Congo	1992	Wine	4
46	Congo	1992	Yams	11
46	Congo	1993	Apples	1
46	Congo	1993	Bananas	38
46	Congo	1993	Beans	2
46	Congo	1993	Beer	81
46	Congo	1993	Bovine Meat	25
46	Congo	1993	Cassava	708
46	Congo	1993	Cereals, Other	5
46	Congo	1993	Demersal Fish	22
46	Congo	1993	Eggs	1
46	Congo	1993	Freshwater Fish	29
46	Congo	1993	Fruits, Other	26
46	Congo	1993	Groundnut Oil	2
46	Congo	1993	Groundnuts (Shelled Eq)	14
46	Congo	1993	Maize	5
46	Congo	1993	Marine Fish, Other	1
46	Congo	1993	Meat, Other	13
46	Congo	1993	Milk, Whole	33
46	Congo	1993	Mutton & Goat Meat	1
46	Congo	1993	Offals, Edible	2
46	Congo	1993	Oilcrops Oil, Other	6
46	Congo	1993	Oilcrops, Other	3
46	Congo	1993	Onions	4
46	Congo	1993	Oranges, Mandarines	3
46	Congo	1993	Palm Oil	15
46	Congo	1993	Peas	4
46	Congo	1993	Pelagic Fish	36
46	Congo	1993	Pigmeat	7
46	Congo	1993	Pineapples	5
46	Congo	1993	Plantains	72
46	Congo	1993	Potatoes	3
46	Congo	1993	Poultry Meat	20
46	Congo	1993	Rape and Mustard Oil	1
46	Congo	1993	Rice (Milled Equivalent)	23
46	Congo	1993	Roots, Other	35
46	Congo	1993	Soyabean Oil	2
46	Congo	1993	Sugar Cane	5
46	Congo	1993	Sugar, Refined Equivalent	16
46	Congo	1993	Sweet Potatoes	11
46	Congo	1993	Tomatoes	10
46	Congo	1993	Vegetables, Other	79
46	Congo	1993	Wheat	101
46	Congo	1993	Wine	4
46	Congo	1993	Yams	11
46	Congo	1994	Bananas	39
46	Congo	1994	Beans	2
46	Congo	1994	Beer	56
46	Congo	1994	Bovine Meat	20
46	Congo	1994	Cassava	737
46	Congo	1994	Cereals, Other	4
46	Congo	1994	Demersal Fish	15
46	Congo	1994	Eggs	1
46	Congo	1994	Freshwater Fish	25
46	Congo	1994	Fruits, Other	26

FAO country code	Country	Reference year	Food item	Gross food supply (g *capita*$^{-1}$ d^{-1})
46	Congo	1994	Groundnut Oil	2
46	Congo	1994	Groundnuts (Shelled Eq)	15
46	Congo	1994	Maize	5
46	Congo	1994	Marine Fish, Other	1
46	Congo	1994	Meat, Other	13
46	Congo	1994	Milk, Whole	21
46	Congo	1994	Mutton & Goat Meat	1
46	Congo	1994	Offals, Edible	1
46	Congo	1994	Oilcrops Oil, Other	4
46	Congo	1994	Oilcrops, Other	3
46	Congo	1994	Onions	1
46	Congo	1994	Oranges, Mandarines	3
46	Congo	1994	Palm Oil	14
46	Congo	1994	Peas	4
46	Congo	1994	Pelagic Fish	32
46	Congo	1994	Pigmeat	6
46	Congo	1994	Pineapples	5
46	Congo	1994	Plantains	72
46	Congo	1994	Potatoes	2
46	Congo	1994	Poultry Meat	12
46	Congo	1994	Rape and Mustard Oil	1
46	Congo	1994	Rice (Milled Equivalent)	30
46	Congo	1994	Roots, Other	36
46	Congo	1994	Soyabean Oil	3
46	Congo	1994	Sugar Cane	5
46	Congo	1994	Sugar, Refined Equivalent	19
46	Congo	1994	Sunflowerseed Oil	1
46	Congo	1994	Sweet Potatoes	12
46	Congo	1994	Tomatoes	9
46	Congo	1994	Vegetables, Other	78
46	Congo	1994	Wheat	90
46	Congo	1994	Wine	3
46	Congo	1994	Yams	11
46	Congo	1995	Apples	1
46	Congo	1995	Bananas	40
46	Congo	1995	Beans	2
46	Congo	1995	Beer	51
46	Congo	1995	Bovine Meat	17
46	Congo	1995	Cassava	763
46	Congo	1995	Demersal Fish	7
46	Congo	1995	Eggs	1
46	Congo	1995	Freshwater Fish	26
46	Congo	1995	Fruits, Other	45
46	Congo	1995	Groundnut Oil	2
46	Congo	1995	Groundnuts (Shelled Eq)	13
46	Congo	1995	Maize	11
46	Congo	1995	Marine Fish, Other	1
46	Congo	1995	Meat, Other	13
46	Congo	1995	Milk, Whole	27
46	Congo	1995	Mutton & Goat Meat	1
46	Congo	1995	Offals, Edible	1
46	Congo	1995	Oilcrops Oil, Other	5
46	Congo	1995	Oilcrops, Other	3
46	Congo	1995	Onions	2
46	Congo	1995	Oranges, Mandarines	2
46	Congo	1995	Palm Oil	13
46	Congo	1995	Peas	4
46	Congo	1995	Pelagic Fish	27
46	Congo	1995	Pigmeat	7
46	Congo	1995	Pineapples	3
46	Congo	1995	Plantains	70
46	Congo	1995	Potatoes	2
46	Congo	1995	Poultry Meat	15
46	Congo	1995	Rice (Milled Equivalent)	35
46	Congo	1995	Roots, Other	37
46	Congo	1995	Soyabean Oil	5
46	Congo	1995	Sugar Cane	5
46	Congo	1995	Sugar, Refined Equivalent	20
46	Congo	1995	Sweet Potatoes	12
46	Congo	1995	Tomatoes	7
46	Congo	1995	Vegetables, Other	78
46	Congo	1995	Wheat	74
46	Congo	1995	Wine	4
46	Congo	1995	Yams	11
46	Congo	1996	Bananas	43
46	Congo	1996	Beans	3
46	Congo	1996	Beer	50
46	Congo	1996	Beverages, Alcoholic	1
46	Congo	1996	Bovine Meat	5
46	Congo	1996	Cassava	786
46	Congo	1996	Coconuts - Incl Copra	4
46	Congo	1996	Demersal Fish	6
46	Congo	1996	Eggs	1
46	Congo	1996	Freshwater Fish	25
46	Congo	1996	Fruits, Other	46
46	Congo	1996	Grapefruit	3
46	Congo	1996	Groundnut Oil	2
46	Congo	1996	Groundnuts (Shelled Eq)	9
46	Congo	1996	Lemons, Limes	3
46	Congo	1996	Maize	10
46	Congo	1996	Marine Fish, Other	2
46	Congo	1996	Meat, Other	12
46	Congo	1996	Milk, Whole	35
46	Congo	1996	Mutton & Goat Meat	1
46	Congo	1996	Offals, Edible	2
46	Congo	1996	Oilcrops Oil, Other	2
46	Congo	1996	Oilcrops, Other	3

FAO country code	Country	Reference year	Food item	Gross food supply (g $capita^{-1}$ d^{-1})
46	Congo	1996	Onions	2
46	Congo	1996	Oranges, Mandarines	5
46	Congo	1996	Palm Oil	14
46	Congo	1996	Peas	4
46	Congo	1996	Pelagic Fish	30
46	Congo	1996	Pigmeat	9
46	Congo	1996	Pineapples	2
46	Congo	1996	Plantains	67
46	Congo	1996	Potatoes	3
46	Congo	1996	Poultry Meat	19
46	Congo	1996	Rape and Mustard Oil	2
46	Congo	1996	Rice (Milled Equivalent)	18
46	Congo	1996	Roots, Other	37
46	Congo	1996	Soyabean Oil	7
46	Congo	1996	Sugar Cane	5
46	Congo	1996	Sugar, Refined Equivalent	20
46	Congo	1996	Sunflowerseed Oil	1
46	Congo	1996	Sweet Potatoes	9
46	Congo	1996	Tomatoes	8
46	Congo	1996	Vegetables, Other	83
46	Congo	1996	Wheat	77
46	Congo	1996	Wine	4
46	Congo	1996	Yams	7
46	Congo	1997	Bananas	43
46	Congo	1997	Beans	4
46	Congo	1997	Beer	33
46	Congo	1997	Beverages, Alcoholic	2
46	Congo	1997	Bovine Meat	7
46	Congo	1997	Cassava	774
46	Congo	1997	Coconuts - Incl Copra	4
46	Congo	1997	Cottonseed Oil	3
46	Congo	1997	Demersal Fish	10
46	Congo	1997	Eggs	1
46	Congo	1997	Freshwater Fish	18
46	Congo	1997	Fruits, Other	46
46	Congo	1997	Grapefruit	3
46	Congo	1997	Groundnut Oil	2
46	Congo	1997	Groundnuts (Shelled Eq)	9
46	Congo	1997	Lemons, Limes	3
46	Congo	1997	Maize	11
46	Congo	1997	Marine Fish, Other	2
46	Congo	1997	Meat, Other	12
46	Congo	1997	Milk, Whole	30
46	Congo	1997	Mutton & Goat Meat	1
46	Congo	1997	Offals, Edible	2
46	Congo	1997	Oilcrops Oil, Other	2
46	Congo	1997	Oilcrops, Other	3
46	Congo	1997	Onions	2
46	Congo	1997	Oranges, Mandarines	5
46	Congo	1997	Palm Oil	12
46	Congo	1997	Peas	4
46	Congo	1997	Pelagic Fish	27
46	Congo	1997	Pigmeat	7
46	Congo	1997	Pineapples	3
46	Congo	1997	Plantains	65
46	Congo	1997	Potatoes	3
46	Congo	1997	Poultry Meat	11
46	Congo	1997	Rape and Mustard Oil	1
46	Congo	1997	Rice (Milled Equivalent)	24
46	Congo	1997	Roots, Other	34
46	Congo	1997	Soyabean Oil	8
46	Congo	1997	Sugar Cane	5
46	Congo	1997	Sugar, Refined Equivalent	15
46	Congo	1997	Sunflowerseed Oil	1
46	Congo	1997	Sweet Potatoes	9
46	Congo	1997	Tomatoes	16
46	Congo	1997	Vegetables, Other	79
46	Congo	1997	Wheat	97
46	Congo	1997	Wine	4
46	Congo	1997	Yams	8
46	Congo	1998	Bananas	43
46	Congo	1998	Beans	6
46	Congo	1998	Beer	46
46	Congo	1998	Beverages, Alcoholic	1
46	Congo	1998	Bovine Meat	5
46	Congo	1998	Cassava	706
46	Congo	1998	Coconuts - Incl Copra	3
46	Congo	1998	Cottonseed Oil	1
46	Congo	1998	Demersal Fish	8
46	Congo	1998	Eggs	2
46	Congo	1998	Freshwater Fish	24
46	Congo	1998	Fruits, Other	47
46	Congo	1998	Grapefruit	3
46	Congo	1998	Groundnut Oil	1
46	Congo	1998	Groundnuts (Shelled Eq)	6
46	Congo	1998	Lemons, Limes	3
46	Congo	1998	Maize	15
46	Congo	1998	Marine Fish, Other	2
46	Congo	1998	Meat, Other	14
46	Congo	1998	Milk, Whole	38
46	Congo	1998	Mutton & Goat Meat	1
46	Congo	1998	Offals, Edible	2
46	Congo	1998	Oilcrops Oil, Other	2
46	Congo	1998	Oilcrops, Other	3
46	Congo	1998	Onions	2
46	Congo	1998	Oranges, Mandarines	66
46	Congo	1998	Palm Oil	13

FAO country code	Country	Reference year	Food item	Gross food supply (g *capita*$^{-1}$ d^{-1})
46	Congo	1998	Peas	4
46	Congo	1998	Pelagic Fish	21
46	Congo	1998	Pigmeat	10
46	Congo	1998	Pineapples	3
46	Congo	1998	Plantains	59
46	Congo	1998	Potatoes	3
46	Congo	1998	Poultry Meat	15
46	Congo	1998	Rape and Mustard Oil	1
46	Congo	1998	Rice (Milled Equivalent)	34
46	Congo	1998	Roots, Other	32
46	Congo	1998	Soyabean Oil	10
46	Congo	1998	Sugar Cane	5
46	Congo	1998	Sugar, Refined Equivalent	21
46	Congo	1998	Sweet Potatoes	9
46	Congo	1998	Tomatoes	16
46	Congo	1998	Vegetables, Other	75
46	Congo	1998	Wheat	114
46	Congo	1998	Wine	4
46	Congo	1998	Yams	8
46	Congo	1999	Bananas	64
46	Congo	1999	Beans	4
46	Congo	1999	Beer	45
46	Congo	1999	Beverages, Alcoholic	2
46	Congo	1999	Bovine Meat	3
46	Congo	1999	Cassava	711
46	Congo	1999	Coconuts - Incl Copra	3
46	Congo	1999	Cottonseed Oil	1
46	Congo	1999	Demersal Fish	6
46	Congo	1999	Eggs	1
46	Congo	1999	Freshwater Fish	23
46	Congo	1999	Fruits, Other	47
46	Congo	1999	Grapefruit	3
46	Congo	1999	Groundnut Oil	2
46	Congo	1999	Groundnuts (Shelled Eq)	8
46	Congo	1999	Lemons, Limes	4
46	Congo	1999	Maize	17
46	Congo	1999	Marine Fish, Other	3
46	Congo	1999	Meat, Other	14
46	Congo	1999	Milk, Whole	30
46	Congo	1999	Mutton & Goat Meat	1
46	Congo	1999	Offals, Edible	1
46	Congo	1999	Oilcrops Oil, Other	3
46	Congo	1999	Oilcrops, Other	3
46	Congo	1999	Onions	2
46	Congo	1999	Oranges, Mandarines	5
46	Congo	1999	Palm Oil	15
46	Congo	1999	Peas	4
46	Congo	1999	Pelagic Fish	21
46	Congo	1999	Pigmeat	6
46	Congo	1999	Pineapples	3
46	Congo	1999	Plantains	55
46	Congo	1999	Potatoes	3
46	Congo	1999	Poultry Meat	12
46	Congo	1999	Rape and Mustard Oil	5
46	Congo	1999	Rice (Milled Equivalent)	33
46	Congo	1999	Roots, Other	38
46	Congo	1999	Soyabean Oil	8
46	Congo	1999	Sugar Cane	5
46	Congo	1999	Sugar, Refined Equivalent	22
46	Congo	1999	Sweet Potatoes	7
46	Congo	1999	Tomatoes	17
46	Congo	1999	Vegetables, Other	78
46	Congo	1999	Wheat	121
46	Congo	1999	Wine	2
46	Congo	1999	Yams	7
46	Congo	2000	Bananas	64
46	Congo	2000	Beans	4
46	Congo	2000	Beer	51
46	Congo	2000	Beverages, Alcoholic	2
46	Congo	2000	Bovine Meat	4
46	Congo	2000	Cassava	726
46	Congo	2000	Coconuts - Incl Copra	3
46	Congo	2000	Cottonseed Oil	1
46	Congo	2000	Crustaceans	1
46	Congo	2000	Demersal Fish	7
46	Congo	2000	Eggs	1
46	Congo	2000	Freshwater Fish	22
46	Congo	2000	Fruits, Other	46
46	Congo	2000	Grapefruit	3
46	Congo	2000	Groundnut Oil	2
46	Congo	2000	Groundnuts (Shelled Eq)	7
46	Congo	2000	Lemons, Limes	3
46	Congo	2000	Maize	17
46	Congo	2000	Marine Fish, Other	4
46	Congo	2000	Meat, Other	14
46	Congo	2000	Milk, Whole	41
46	Congo	2000	Mutton & Goat Meat	1
46	Congo	2000	Offals, Edible	3
46	Congo	2000	Oilcrops Oil, Other	4
46	Congo	2000	Oilcrops, Other	3
46	Congo	2000	Onions	3
46	Congo	2000	Oranges, Mandarines	5
46	Congo	2000	Palm Oil	14
46	Congo	2000	Peas	4
46	Congo	2000	Pelagic Fish	21
46	Congo	2000	Pigmeat	7
46	Congo	2000	Pineapples	2

FAO country code	Country	Reference year	Food item	Gross food supply (g $capita^{-1}$ d^{-1})
46	Congo	2000	Plantains	55
46	Congo	2000	Potatoes	3
46	Congo	2000	Poultry Meat	14
46	Congo	2000	Rape and Mustard Oil	3
46	Congo	2000	Rice (Milled Equivalent)	33
46	Congo	2000	Roots, Other	31
46	Congo	2000	Soyabean Oil	11
46	Congo	2000	Sugar Cane	4
46	Congo	2000	Sugar, Refined Equivalent	25
46	Congo	2000	Sunflowerseed Oil	1
46	Congo	2000	Sweet Potatoes	6
46	Congo	2000	Tomatoes	34
46	Congo	2000	Vegetables, Other	78
46	Congo	2000	Wheat	123
46	Congo	2000	Wine	3
46	Congo	2000	Yams	8
46	Congo	2001	Bananas	64
46	Congo	2001	Beans	5
46	Congo	2001	Beer	49
46	Congo	2001	Beverages, Alcoholic	2
46	Congo	2001	Bovine Meat	2
46	Congo	2001	Cassava	716
46	Congo	2001	Coconuts - Incl Copra	3
46	Congo	2001	Demersal Fish	14
46	Congo	2001	Eggs	2
46	Congo	2001	Freshwater Fish	23
46	Congo	2001	Fruits, Other	46
46	Congo	2001	Grapefruit	3
46	Congo	2001	Groundnut Oil	2
46	Congo	2001	Groundnuts (Shelled Eq)	8
46	Congo	2001	Lemons, Limes	3
46	Congo	2001	Maize	17
46	Congo	2001	Marine Fish, Other	5
46	Congo	2001	Meat, Other	14
46	Congo	2001	Milk, Whole	34
46	Congo	2001	Mutton & Goat Meat	1
46	Congo	2001	Offals, Edible	4
46	Congo	2001	Oilcrops Oil, Other	5
46	Congo	2001	Oilcrops, Other	3
46	Congo	2001	Onions	3
46	Congo	2001	Oranges, Mandarines	5
46	Congo	2001	Palm Oil	17
46	Congo	2001	Peas	4
46	Congo	2001	Pelagic Fish	24
46	Congo	2001	Pigmeat	7
46	Congo	2001	Pineapples	2
46	Congo	2001	Plantains	55
46	Congo	2001	Potatoes	3
46	Congo	2001	Poultry Meat	12
46	Congo	2001	Pulses, Other	2
46	Congo	2001	Rice (Milled Equivalent)	35
46	Congo	2001	Roots, Other	29
46	Congo	2001	Soyabean Oil	13
46	Congo	2001	Sugar Cane	4
46	Congo	2001	Sugar, Refined Equivalent	29
46	Congo	2001	Sunflowerseed Oil	1
46	Congo	2001	Sweet Potatoes	5
46	Congo	2001	Tomatoes	24
46	Congo	2001	Vegetables, Other	77
46	Congo	2001	Wheat	129
46	Congo	2001	Wine	4
46	Congo	2001	Yams	6
46	Congo	2002	Bananas	63
46	Congo	2002	Beans	5
46	Congo	2002	Beer	56
46	Congo	2002	Beverages, Alcoholic	2
46	Congo	2002	Bovine Meat	4
46	Congo	2002	Cassava	712
46	Congo	2002	Coconuts - Incl Copra	3
46	Congo	2002	Demersal Fish	8
46	Congo	2002	Eggs	2
46	Congo	2002	Freshwater Fish	25
46	Congo	2002	Fruits, Other	47
46	Congo	2002	Grapefruit	3
46	Congo	2002	Groundnut Oil	2
46	Congo	2002	Groundnuts (Shelled Eq)	8
46	Congo	2002	Lemons, Limes	2
46	Congo	2002	Maize	17
46	Congo	2002	Marine Fish, Other	3
46	Congo	2002	Meat, Other	15
46	Congo	2002	Milk, Whole	40
46	Congo	2002	Mutton & Goat Meat	1
46	Congo	2002	Offals, Edible	6
46	Congo	2002	Oilcrops Oil, Other	6
46	Congo	2002	Oilcrops, Other	4
46	Congo	2002	Onions	3
46	Congo	2002	Oranges, Mandarines	4
46	Congo	2002	Palm Oil	18
46	Congo	2002	Peas	4
46	Congo	2002	Pelagic Fish	18
46	Congo	2002	Pigmeat	8
46	Congo	2002	Pineapples	2
46	Congo	2002	Plantains	45
46	Congo	2002	Potatoes	4
46	Congo	2002	Poultry Meat	13
46	Congo	2002	Pulses, Other	1
46	Congo	2002	Rice (Milled Equivalent)	38

FAO country code	Country	Reference year	Food item	Gross food supply (g $capita^{-1}$ d^{-1})
46	Congo	2002	Roots, Other	30
46	Congo	2002	Soyabean Oil	12
46	Congo	2002	Sugar Cane	4
46	Congo	2002	Sugar, Refined Equivalent	32
46	Congo	2002	Sweet Potatoes	4
46	Congo	2002	Tomatoes	20
46	Congo	2002	Vegetables, Other	80
46	Congo	2002	Wheat	127
46	Congo	2002	Wine	3
46	Congo	2002	Yams	8
46	Congo	2003	Bananas	59
46	Congo	2003	Beans	4
46	Congo	2003	Beer	55
46	Congo	2003	Beverages, Alcoholic	2
46	Congo	2003	Bovine Meat	4
46	Congo	2003	Cassava	709
46	Congo	2003	Coconuts - Incl Copra	3
46	Congo	2003	Coffee	1
46	Congo	2003	Demersal Fish	12
46	Congo	2003	Eggs	2
46	Congo	2003	Freshwater Fish	26
46	Congo	2003	Fruits, Other	47
46	Congo	2003	Grapefruit	3
46	Congo	2003	Groundnut Oil	2
46	Congo	2003	Groundnuts (Shelled Eq)	7
46	Congo	2003	Lemons, Limes	3
46	Congo	2003	Maize	17
46	Congo	2003	Marine Fish, Other	1
46	Congo	2003	Meat, Other	15
46	Congo	2003	Milk, Whole	43
46	Congo	2003	Mutton & Goat Meat	1
46	Congo	2003	Offals, Edible	9
46	Congo	2003	Oilcrops Oil, Other	4
46	Congo	2003	Oilcrops, Other	3
46	Congo	2003	Onions	3
46	Congo	2003	Oranges, Mandarines	4
46	Congo	2003	Palm Oil	19
46	Congo	2003	Peas	4
46	Congo	2003	Pelagic Fish	15
46	Congo	2003	Pigmeat	5
46	Congo	2003	Pineapples	2
46	Congo	2003	Plantains	43
46	Congo	2003	Potatoes	3
46	Congo	2003	Poultry Meat	34
46	Congo	2003	Pulses, Other	2
46	Congo	2003	Rape and Mustard Oil	1
46	Congo	2003	Rice (Milled Equivalent)	35
46	Congo	2003	Roots, Other	29
46	Congo	2003	Soyabean Oil	10
46	Congo	2003	Sugar Cane	4
46	Congo	2003	Sugar, Refined Equivalent	34
46	Congo	2003	Sweet Potatoes	4
46	Congo	2003	Tomatoes	27
46	Congo	2003	Vegetables, Other	79
46	Congo	2003	Wheat	125
46	Congo	2003	Wine	2
46	Congo	2003	Yams	8
46	Congo	2004	Bananas	56
46	Congo	2004	Beans	5
46	Congo	2004	Beer	54
46	Congo	2004	Beverages, Alcoholic	2
46	Congo	2004	Bovine Meat	7
46	Congo	2004	Cassava	735
46	Congo	2004	Coconuts - Incl Copra	2
46	Congo	2004	Coffee	1
46	Congo	2004	Demersal Fish	15
46	Congo	2004	Eggs	2
46	Congo	2004	Freshwater Fish	26
46	Congo	2004	Fruits, Other	48
46	Congo	2004	Grapefruit	3
46	Congo	2004	Groundnut Oil	2
46	Congo	2004	Groundnuts (Shelled Eq)	7
46	Congo	2004	Lemons, Limes	3
46	Congo	2004	Maize	16
46	Congo	2004	Marine Fish, Other	2
46	Congo	2004	Meat, Other	17
46	Congo	2004	Milk, Whole	63
46	Congo	2004	Mutton & Goat Meat	1
46	Congo	2004	Offals, Edible	11
46	Congo	2004	Oilcrops Oil, Other	5
46	Congo	2004	Oilcrops, Other	3
46	Congo	2004	Onions	3
46	Congo	2004	Oranges, Mandarines	4
46	Congo	2004	Palm Oil	20
46	Congo	2004	Peas	4
46	Congo	2004	Pelagic Fish	21
46	Congo	2004	Pigmeat	5
46	Congo	2004	Pineapples	2
46	Congo	2004	Plantains	44
46	Congo	2004	Potatoes	3
46	Congo	2004	Poultry Meat	26
46	Congo	2004	Rice (Milled Equivalent)	35
46	Congo	2004	Roots, Other	29
46	Congo	2004	Soyabean Oil	6
46	Congo	2004	Sugar Cane	4
46	Congo	2004	Sugar, Refined Equivalent	38
46	Congo	2004	Sweet Potatoes	5

FAO countrycode	Country	Reference year	Food item	Gross food supply (g *capita*$^{-1}$ d^{-1})
46	Congo	2004	Tomatoes	44
46	Congo	2004	Vegetables, Other	79
46	Congo	2004	Wheat	119
46	Congo	2004	Wine	4
46	Congo	2004	Yams	8
46	Congo	2005	Bananas	52
46	Congo	2005	Beans	5
46	Congo	2005	Beer	49
46	Congo	2005	Beverages, Alcoholic	1
46	Congo	2005	Bovine Meat	7
46	Congo	2005	Cassava	719
46	Congo	2005	Coconuts - Incl Copra	2
46	Congo	2005	Cottonseed Oil	1
46	Congo	2005	Crustaceans	1
46	Congo	2005	Demersal Fish	15
46	Congo	2005	Eggs	2
46	Congo	2005	Freshwater Fish	25
46	Congo	2005	Fruits, Other	50
46	Congo	2005	Grapefruit	3
46	Congo	2005	Groundnut Oil	2
46	Congo	2005	Groundnuts (Shelled Eq)	6
46	Congo	2005	Lemons, Limes	3
46	Congo	2005	Maize	13
46	Congo	2005	Marine Fish, Other	1
46	Congo	2005	Meat, Other	19
46	Congo	2005	Milk, Whole	25
46	Congo	2005	Mutton & Goat Meat	1
46	Congo	2005	Offals, Edible	3
46	Congo	2005	Oilcrops Oil, Other	5
46	Congo	2005	Oilcrops, Other	3
46	Congo	2005	Onions	4
46	Congo	2005	Oranges, Mandarines	4
46	Congo	2005	Palm Oil	19
46	Congo	2005	Peas	5
46	Congo	2005	Pelagic Fish	14
46	Congo	2005	Pigmeat	3
46	Congo	2005	Pineapples	2
46	Congo	2005	Plantains	45
46	Congo	2005	Potatoes	3
46	Congo	2005	Poultry Meat	14
46	Congo	2005	Rice (Milled Equivalent)	46
46	Congo	2005	Roots, Other	28
46	Congo	2005	Soyabean Oil	4
46	Congo	2005	Sugar Cane	4
46	Congo	2005	Sugar, Refined Equivalent	36
46	Congo	2005	Sweet Potatoes	5
46	Congo	2005	Tomatoes	4
46	Congo	2005	Vegetables, Other	79
46	Congo	2005	Wheat	76
46	Congo	2005	Wine	3
46	Congo	2005	Yams	8
46	Congo	2006	Bananas	51
46	Congo	2006	Beans	4
46	Congo	2006	Beer	56
46	Congo	2006	Beverages, Alcoholic	1
46	Congo	2006	Bovine Meat	5
46	Congo	2006	Cassava	721
46	Congo	2006	Coconuts - Incl Copra	3
46	Congo	2006	Cottonseed Oil	1
46	Congo	2006	Crustaceans	1
46	Congo	2006	Demersal Fish	15
46	Congo	2006	Eggs	1
46	Congo	2006	Freshwater Fish	23
46	Congo	2006	Fruits, Other	51
46	Congo	2006	Grapefruit	3
46	Congo	2006	Groundnut Oil	2
46	Congo	2006	Groundnuts (Shelled Eq)	6
46	Congo	2006	Lemons, Limes	3
46	Congo	2006	Maize	10
46	Congo	2006	Marine Fish, Other	3
46	Congo	2006	Meat, Other	20
46	Congo	2006	Milk, Whole	61
46	Congo	2006	Mutton & Goat Meat	1
46	Congo	2006	Offals, Edible	13
46	Congo	2006	Oilcrops Oil, Other	3
46	Congo	2006	Oilcrops, Other	2
46	Congo	2006	Onions	3
46	Congo	2006	Oranges, Mandarines	4
46	Congo	2006	Palm Oil	25
46	Congo	2006	Peas	5
46	Congo	2006	Pelagic Fish	13
46	Congo	2006	Pigmeat	5
46	Congo	2006	Pineapples	2
46	Congo	2006	Plantains	47
46	Congo	2006	Potatoes	3
46	Congo	2006	Poultry Meat	22
46	Congo	2006	Rice (Milled Equivalent)	52
46	Congo	2006	Roots, Other	28
46	Congo	2006	Soyabean Oil	3
46	Congo	2006	Sugar Cane	4
46	Congo	2006	Sugar, Refined Equivalent	38
46	Congo	2006	Sweet Potatoes	5
46	Congo	2006	Tomatoes	7
46	Congo	2006	Vegetables, Other	83
46	Congo	2006	Wheat	80
46	Congo	2006	Wine	3
46	Congo	2006	Yams	8

FAO countrycode	Country	Reference year	Food item	Gross food supply (g *capita*$^{-1}$ d^{-1})
46	Congo	2007	Bananas	50
46	Congo	2007	Beans	6
46	Congo	2007	Beer	68
46	Congo	2007	Beverages, Alcoholic	1
46	Congo	2007	Bovine Meat	5
46	Congo	2007	Cassava	692
46	Congo	2007	Coconuts - Incl Copra	2
46	Congo	2007	Coffee	1
46	Congo	2007	Crustaceans	1
46	Congo	2007	Demersal Fish	16
46	Congo	2007	Eggs	1
46	Congo	2007	Freshwater Fish	22
46	Congo	2007	Fruits, Other	51
46	Congo	2007	Grapefruit	3
46	Congo	2007	Groundnut Oil	2
46	Congo	2007	Groundnuts (Shelled Eq)	6
46	Congo	2007	Lemons, Limes	3
46	Congo	2007	Maize	10
46	Congo	2007	Marine Fish, Other	2
46	Congo	2007	Meat, Other	20
46	Congo	2007	Milk, Whole	26
46	Congo	2007	Mutton & Goat Meat	1
46	Congo	2007	Offals, Edible	14
46	Congo	2007	Oilcrops Oil, Other	6
46	Congo	2007	Oilcrops, Other	2
46	Congo	2007	Onions	3
46	Congo	2007	Oranges, Mandarines	4
46	Congo	2007	Palm Oil	21
46	Congo	2007	Peas	4
46	Congo	2007	Pelagic Fish	11
46	Congo	2007	Pigmeat	7
46	Congo	2007	Pineapples	3
46	Congo	2007	Plantains	48
46	Congo	2007	Potatoes	3
46	Congo	2007	Poultry Meat	26
46	Congo	2007	Rice (Milled Equivalent)	53
46	Congo	2007	Roots, Other	26
46	Congo	2007	Soyabean Oil	2
46	Congo	2007	Sugar Cane	4
46	Congo	2007	Sugar, Refined Equivalent	48
46	Congo	2007	Sweet Potatoes	4
46	Congo	2007	Tomatoes	5
46	Congo	2007	Vegetables, Other	79
46	Congo	2007	Wheat	82
46	Congo	2007	Wine	3
46	Congo	2007	Yams	8
46	Congo	2008	Bananas	51
46	Congo	2008	Beans	5
46	Congo	2008	Beer	62
46	Congo	2008	Beverages, Alcoholic	1
46	Congo	2008	Bovine Meat	10
46	Congo	2008	Cassava	710
46	Congo	2008	Coconuts - Incl Copra	2
46	Congo	2008	Coffee	1
46	Congo	2008	Cottonseed Oil	1
46	Congo	2008	Crustaceans	1
46	Congo	2008	Demersal Fish	11
46	Congo	2008	Eggs	1
46	Congo	2008	Freshwater Fish	21
46	Congo	2008	Fruits, Other	52
46	Congo	2008	Grapefruit	3
46	Congo	2008	Groundnut Oil	2
46	Congo	2008	Groundnuts (Shelled Eq)	8
46	Congo	2008	Lemons, Limes	3
46	Congo	2008	Maize	10
46	Congo	2008	Marine Fish, Other	3
46	Congo	2008	Meat, Other	21
46	Congo	2008	Milk, Whole	22
46	Congo	2008	Mutton & Goat Meat	1
46	Congo	2008	Offals, Edible	5
46	Congo	2008	Oilcrops Oil, Other	1
46	Congo	2008	Oilcrops, Other	2
46	Congo	2008	Onions	5
46	Congo	2008	Oranges, Mandarines	6
46	Congo	2008	Palm Oil	20
46	Congo	2008	Peas	3
46	Congo	2008	Pelagic Fish	24
46	Congo	2008	Pigmeat	3
46	Congo	2008	Pineapples	3
46	Congo	2008	Plantains	50
46	Congo	2008	Potatoes	3
46	Congo	2008	Poultry Meat	24
46	Congo	2008	Rice (Milled Equivalent)	51
46	Congo	2008	Roots, Other	25
46	Congo	2008	Soyabean Oil	2
46	Congo	2008	Sugar Cane	4
46	Congo	2008	Sugar, Refined Equivalent	48
46	Congo	2008	Sweet Potatoes	4
46	Congo	2008	Tomatoes	6
46	Congo	2008	Vegetables, Other	81
46	Congo	2008	Wheat	83
46	Congo	2008	Wine	4
46	Congo	2008	Yams	8
46	Congo	2009	Bananas	50
46	Congo	2009	Barley	5
46	Congo	2009	Beans	5
46	Congo	2009	Beer	91

FAO country code	Country	Reference year	Food item	Gross food supply (g *capita*$^{-1}$ d^{-1})
46	Congo	2009	Beverages, Alcoholic	1
46	Congo	2009	Bovine Meat	10
46	Congo	2009	Cassava	706
46	Congo	2009	Coconuts - Incl Copra	2
46	Congo	2009	Coffee	1
46	Congo	2009	Cottonseed Oil	1
46	Congo	2009	Crustaceans	1
46	Congo	2009	Demersal Fish	15
46	Congo	2009	Eggs	1
46	Congo	2009	Freshwater Fish	20
46	Congo	2009	Fruits, Other	51
46	Congo	2009	Grapefruit	3
46	Congo	2009	Groundnut Oil	2
46	Congo	2009	Groundnuts (Shelled Eq)	8
46	Congo	2009	Lemons, Limes	3
46	Congo	2009	Maize	10
46	Congo	2009	Marine Fish, Other	2
46	Congo	2009	Meat, Other	22
46	Congo	2009	Milk, Whole	30
46	Congo	2009	Mutton & Goat Meat	1
46	Congo	2009	Offals, Edible	4
46	Congo	2009	Oilcrops Oil, Other	1
46	Congo	2009	Oilcrops, Other	2
46	Congo	2009	Onions	6
46	Congo	2009	Oranges, Mandarines	5
46	Congo	2009	Palm Oil	19
46	Congo	2009	Peas	4
46	Congo	2009	Pelagic Fish	24
46	Congo	2009	Pigmeat	5
46	Congo	2009	Pineapples	3
46	Congo	2009	Plantains	50
46	Congo	2009	Potatoes	4
46	Congo	2009	Poultry Meat	27
46	Congo	2009	Rice (Milled Equivalent)	50
46	Congo	2009	Roots, Other	32
46	Congo	2009	Soyabean Oil	2
46	Congo	2009	Sugar Cane	3
46	Congo	2009	Sugar, Refined Equivalent	35
46	Congo	2009	Sweet Potatoes	4
46	Congo	2009	Tomatoes	8
46	Congo	2009	Vegetables, Other	84
46	Congo	2009	Wheat	104
46	Congo	2009	Wine	3
46	Congo	2009	Yams	8
46	Congo	2010	Bananas	51
46	Congo	2010	Barley	5
46	Congo	2010	Beans	5
46	Congo	2010	Beer	92
46	Congo	2010	Beverages, Alcoholic	1
46	Congo	2010	Bovine Meat	7
46	Congo	2010	Cassava	693
46	Congo	2010	Coconuts - Incl Copra	2
46	Congo	2010	Crustaceans	1
46	Congo	2010	Demersal Fish	19
46	Congo	2010	Eggs	1
46	Congo	2010	Freshwater Fish	21
46	Congo	2010	Fruits, Other	52
46	Congo	2010	Grapefruit	3
46	Congo	2010	Groundnut Oil	2
46	Congo	2010	Groundnuts (Shelled Eq)	7
46	Congo	2010	Lemons, Limes	3
46	Congo	2010	Maize	10
46	Congo	2010	Marine Fish, Other	1
46	Congo	2010	Meat, Other	26
46	Congo	2010	Milk, Whole	29
46	Congo	2010	Mutton & Goat Meat	1
46	Congo	2010	Offals, Edible	2
46	Congo	2010	Oilcrops Oil, Other	1
46	Congo	2010	Oilcrops, Other	2
46	Congo	2010	Onions	6
46	Congo	2010	Oranges, Mandarines	6
46	Congo	2010	Palm Oil	21
46	Congo	2010	Peas	4
46	Congo	2010	Pelagic Fish	13
46	Congo	2010	Pigmeat	3
46	Congo	2010	Pineapples	3
46	Congo	2010	Plantains	49
46	Congo	2010	Potatoes	4
46	Congo	2010	Poultry Meat	33
46	Congo	2010	Rice (Milled Equivalent)	54
46	Congo	2010	Roots, Other	33
46	Congo	2010	Sugar Cane	3
46	Congo	2010	Sugar, Refined Equivalent	34
46	Congo	2010	Sweet Potatoes	5
46	Congo	2010	Tomatoes	10
46	Congo	2010	Vegetables, Other	90
46	Congo	2010	Wheat	109
46	Congo	2010	Wine	3
46	Congo	2010	Yams	8
46	Congo	2011	Apples	1
46	Congo	2011	Bananas	54
46	Congo	2011	Barley	5
46	Congo	2011	Beans	6
46	Congo	2011	Beer	103
46	Congo	2011	Beverages, Alcoholic	1
46	Congo	2011	Bovine Meat	7
46	Congo	2011	Cassava	674

FAO country code	Country	Reference year	Food item	Gross food supply (g *capita*$^{-1}$ d^{-1})
46	Congo	2011	Cocoa Beans	1
46	Congo	2011	Coconuts - Incl Copra	2
46	Congo	2011	Demersal Fish	23
46	Congo	2011	Eggs	2
46	Congo	2011	Freshwater Fish	22
46	Congo	2011	Fruits, Other	50
46	Congo	2011	Grapefruit	3
46	Congo	2011	Groundnut Oil	2
46	Congo	2011	Groundnuts (Shelled Eq)	7
46	Congo	2011	Lemons, Limes	3
46	Congo	2011	Maize	10
46	Congo	2011	Marine Fish, Other	4
46	Congo	2011	Meat, Other	27
46	Congo	2011	Milk, Whole	32
46	Congo	2011	Molluscs, Other	1
46	Congo	2011	Mutton & Goat Meat	1
46	Congo	2011	Offals, Edible	8
46	Congo	2011	Oilcrops Oil, Other	2
46	Congo	2011	Oilcrops, Other	2
46	Congo	2011	Onions	6
46	Congo	2011	Oranges, Mandarines	6
46	Congo	2011	Palm Oil	20
46	Congo	2011	Peas	4
46	Congo	2011	Pelagic Fish	22
46	Congo	2011	Pigmeat	7
46	Congo	2011	Pineapples	3
46	Congo	2011	Plantains	48
46	Congo	2011	Potatoes	4
46	Congo	2011	Poultry Meat	61
46	Congo	2011	Rice (Milled Equivalent)	54
46	Congo	2011	Roots, Other	33
46	Congo	2011	Sugar Cane	3
46	Congo	2011	Sugar, Refined Equivalent	34
46	Congo	2011	Sweet Potatoes	5
46	Congo	2011	Sweeteners, Other	1
46	Congo	2011	Tomatoes	7
46	Congo	2011	Vegetables, Other	88
46	Congo	2011	Wheat	110
46	Congo	2011	Wine	2
46	Congo	2011	Yams	8
48	Costa Rica	1992	Apples	1
48	Costa Rica	1992	Bananas	76
48	Costa Rica	1992	Barley	4
48	Costa Rica	1992	Beans	25
48	Costa Rica	1992	Beer	28
48	Costa Rica	1992	Beverages, Alcoholic	21
48	Costa Rica	1992	Bovine Meat	59
48	Costa Rica	1992	Butter, Ghee	3
48	Costa Rica	1992	Cassava	2
48	Costa Rica	1992	Cereals, Other	2
48	Costa Rica	1992	Cheese	5
48	Costa Rica	1992	Cocoa Beans	2
48	Costa Rica	1992	Coconut Oil	1
48	Costa Rica	1992	Coconuts - Incl Copra	4
48	Costa Rica	1992	Coffee	22
48	Costa Rica	1992	Crustaceans	2
48	Costa Rica	1992	Demersal Fish	3
48	Costa Rica	1992	Eggs	30
48	Costa Rica	1992	Fats, Animals, Raw	3
48	Costa Rica	1992	Freshwater Fish	2
48	Costa Rica	1992	Fruits, Other	63
48	Costa Rica	1992	Grapefruit	12
48	Costa Rica	1992	Grapes	1
48	Costa Rica	1992	Groundnuts (Shelled Eq)	1
48	Costa Rica	1992	Honey	1
48	Costa Rica	1992	Maize	50
48	Costa Rica	1992	Marine Fish, Other	2
48	Costa Rica	1992	Milk, Whole	337
48	Costa Rica	1992	Nuts	1
48	Costa Rica	1992	Offals, Edible	8
48	Costa Rica	1992	Onions	16
48	Costa Rica	1992	Oranges, Mandarines	38
48	Costa Rica	1992	Palm Oil	26
48	Costa Rica	1992	Pelagic Fish	3
48	Costa Rica	1992	Pepper	1
48	Costa Rica	1992	Pigmeat	16
48	Costa Rica	1992	Pineapples	253
48	Costa Rica	1992	Plantains	27
48	Costa Rica	1992	Potatoes	45
48	Costa Rica	1992	Poultry Meat	42
48	Costa Rica	1992	Pulses, Other	1
48	Costa Rica	1992	Rice (Milled Equivalent)	130
48	Costa Rica	1992	Roots, Other	12
48	Costa Rica	1992	Soyabean Oil	12
48	Costa Rica	1992	Spices, Other	5
48	Costa Rica	1992	Sugar, Non-Centrifugal	10
48	Costa Rica	1992	Sugar, Refined Equivalent	140
48	Costa Rica	1992	Sweeteners, Other	2
48	Costa Rica	1992	Tomatoes	8
48	Costa Rica	1992	Vegetables, Other	78
48	Costa Rica	1992	Wheat	79
48	Costa Rica	1992	Wine	1
48	Costa Rica	1993	Apples	2
48	Costa Rica	1993	Barley	4
48	Costa Rica	1993	Beans	23
48	Costa Rica	1993	Beer	30
48	Costa Rica	1993	Beverages, Alcoholic	21

FAO country code	Country	Reference year	Food item	Gross food supply (g *capita*$^{-1}$ d^{-1})
48	Costa Rica	1993	Bovine Meat	57
48	Costa Rica	1993	Butter, Ghee	3
48	Costa Rica	1993	Cassava	3
48	Costa Rica	1993	Cereals, Other	3
48	Costa Rica	1993	Cheese	5
48	Costa Rica	1993	Cocoa Beans	2
48	Costa Rica	1993	Coconut Oil	1
48	Costa Rica	1993	Coconuts - Incl Copra	2
48	Costa Rica	1993	Coffee	22
48	Costa Rica	1993	Crustaceans	3
48	Costa Rica	1993	Demersal Fish	3
48	Costa Rica	1993	Eggs	32
48	Costa Rica	1993	Fats, Animals, Raw	3
48	Costa Rica	1993	Freshwater Fish	3
48	Costa Rica	1993	Fruits, Other	80
48	Costa Rica	1993	Grapefruit	15
48	Costa Rica	1993	Grapes	1
48	Costa Rica	1993	Groundnuts (Shelled Eq)	1
48	Costa Rica	1993	Honey	1
48	Costa Rica	1993	Maize	46
48	Costa Rica	1993	Marine Fish, Other	6
48	Costa Rica	1993	Milk, Whole	367
48	Costa Rica	1993	Nuts	2
48	Costa Rica	1993	Offals, Edible	8
48	Costa Rica	1993	Onions	19
48	Costa Rica	1993	Oranges, Mandarines	44
48	Costa Rica	1993	Palm Oil	25
48	Costa Rica	1993	Pelagic Fish	2
48	Costa Rica	1993	Pepper	1
48	Costa Rica	1993	Pigmeat	16
48	Costa Rica	1993	Pineapples	244
48	Costa Rica	1993	Plantains	28
48	Costa Rica	1993	Potatoes	36
48	Costa Rica	1993	Poultry Meat	43
48	Costa Rica	1993	Pulses, Other	1
48	Costa Rica	1993	Rice (Milled Equivalent)	172
48	Costa Rica	1993	Roots, Other	22
48	Costa Rica	1993	Soyabean Oil	11
48	Costa Rica	1993	Soyabeans	1
48	Costa Rica	1993	Spices, Other	2
48	Costa Rica	1993	Sugar, Non-Centrifugal	10
48	Costa Rica	1993	Sugar, Refined Equivalent	141
48	Costa Rica	1993	Sweeteners, Other	2
48	Costa Rica	1993	Tomatoes	8
48	Costa Rica	1993	Vegetables, Other	73
48	Costa Rica	1993	Wheat	65
48	Costa Rica	1993	Wine	1
48	Costa Rica	1994	Apples	7
48	Costa Rica	1994	Bananas	1
48	Costa Rica	1994	Barley	9
48	Costa Rica	1994	Beans	26
48	Costa Rica	1994	Beer	34
48	Costa Rica	1994	Beverages, Alcoholic	22
48	Costa Rica	1994	Bovine Meat	54
48	Costa Rica	1994	Butter, Ghee	3
48	Costa Rica	1994	Cassava	2
48	Costa Rica	1994	Cheese	5
48	Costa Rica	1994	Cocoa Beans	1
48	Costa Rica	1994	Coconut Oil	1
48	Costa Rica	1994	Coconuts - Incl Copra	2
48	Costa Rica	1994	Coffee	15
48	Costa Rica	1994	Crustaceans	2
48	Costa Rica	1994	Eggs	31
48	Costa Rica	1994	Fats, Animals, Raw	6
48	Costa Rica	1994	Freshwater Fish	3
48	Costa Rica	1994	Fruits, Other	93
48	Costa Rica	1994	Grapefruit	10
48	Costa Rica	1994	Grapes	4
48	Costa Rica	1994	Groundnuts (Shelled Eq)	1
48	Costa Rica	1994	Honey	1
48	Costa Rica	1994	Maize	24
48	Costa Rica	1994	Marine Fish, Other	5
48	Costa Rica	1994	Milk, Whole	364
48	Costa Rica	1994	Molluscs, Other	1
48	Costa Rica	1994	Nuts	1
48	Costa Rica	1994	Oats	3
48	Costa Rica	1994	Offals, Edible	8
48	Costa Rica	1994	Onions	18
48	Costa Rica	1994	Oranges, Mandarines	63
48	Costa Rica	1994	Palm Oil	20
48	Costa Rica	1994	Palmkernel Oil	1
48	Costa Rica	1994	Pelagic Fish	2
48	Costa Rica	1994	Pepper	1
48	Costa Rica	1994	Pigmeat	18
48	Costa Rica	1994	Pineapples	247
48	Costa Rica	1994	Plantains	19
48	Costa Rica	1994	Potatoes	28
48	Costa Rica	1994	Poultry Meat	43
48	Costa Rica	1994	Pulses, Other	1
48	Costa Rica	1994	Rice (Milled Equivalent)	130
48	Costa Rica	1994	Roots, Other	27
48	Costa Rica	1994	Soyabean Oil	12
48	Costa Rica	1994	Soyabeans	6
48	Costa Rica	1994	Spices, Other	7
48	Costa Rica	1994	Sugar, Non-Centrifugal	9
48	Costa Rica	1994	Sugar, Refined Equivalent	138
48	Costa Rica	1994	Sweeteners, Other	3

FAO country code	Country	Reference year	Food item	Gross food supply (g $capita^{-1}$ d^{-1})
48	Costa Rica	1994	Tomatoes	13
48	Costa Rica	1994	Vegetables, Other	70
48	Costa Rica	1994	Wheat	117
48	Costa Rica	1994	Wine	2
48	Costa Rica	1995	Apples	8
48	Costa Rica	1995	Barley	10
48	Costa Rica	1995	Beans	29
48	Costa Rica	1995	Beer	34
48	Costa Rica	1995	Beverages, Alcoholic	22
48	Costa Rica	1995	Bovine Meat	53
48	Costa Rica	1995	Butter, Ghee	3
48	Costa Rica	1995	Cassava	3
48	Costa Rica	1995	Cheese	5
48	Costa Rica	1995	Cocoa Beans	1
48	Costa Rica	1995	Coconut Oil	1
48	Costa Rica	1995	Coconuts - Incl Copra	3
48	Costa Rica	1995	Coffee	12
48	Costa Rica	1995	Crustaceans	3
48	Costa Rica	1995	Eggs	36
48	Costa Rica	1995	Fats, Animals, Raw	6
48	Costa Rica	1995	Freshwater Fish	4
48	Costa Rica	1995	Fruits, Other	91
48	Costa Rica	1995	Grapefruit	11
48	Costa Rica	1995	Grapes	4
48	Costa Rica	1995	Groundnuts (Shelled Eq)	1
48	Costa Rica	1995	Honey	1
48	Costa Rica	1995	Maize	25
48	Costa Rica	1995	Maize Germ Oil	1
48	Costa Rica	1995	Marine Fish, Other	5
48	Costa Rica	1995	Milk, Whole	377
48	Costa Rica	1995	Molluscs, Other	1
48	Costa Rica	1995	Nuts	1
48	Costa Rica	1995	Oats	2
48	Costa Rica	1995	Offals, Edible	8
48	Costa Rica	1995	Onions	11
48	Costa Rica	1995	Oranges, Mandarines	34
48	Costa Rica	1995	Palm Oil	22
48	Costa Rica	1995	Pelagic Fish	2
48	Costa Rica	1995	Pepper	2
48	Costa Rica	1995	Pigmeat	18
48	Costa Rica	1995	Pineapples	120
48	Costa Rica	1995	Plantains	15
48	Costa Rica	1995	Potatoes	30
48	Costa Rica	1995	Poultry Meat	46
48	Costa Rica	1995	Pulses, Other	1
48	Costa Rica	1995	Rice (Milled Equivalent)	133
48	Costa Rica	1995	Roots, Other	34
48	Costa Rica	1995	Soyabean Oil	13
48	Costa Rica	1995	Soyabeans	11
48	Costa Rica	1995	Spices, Other	8
48	Costa Rica	1995	Sugar, Non-Centrifugal	9
48	Costa Rica	1995	Sugar, Refined Equivalent	136
48	Costa Rica	1995	Sweeteners, Other	3
48	Costa Rica	1995	Tomatoes	14
48	Costa Rica	1995	Vegetables, Other	69
48	Costa Rica	1995	Wheat	118
48	Costa Rica	1995	Wine	2
48	Costa Rica	1996	Apples	7
48	Costa Rica	1996	Bananas	1
48	Costa Rica	1996	Barley	5
48	Costa Rica	1996	Beans	14
48	Costa Rica	1996	Beer	33
48	Costa Rica	1996	Beverages, Alcoholic	21
48	Costa Rica	1996	Bovine Meat	54
48	Costa Rica	1996	Butter, Ghee	3
48	Costa Rica	1996	Cassava	3
48	Costa Rica	1996	Cheese	4
48	Costa Rica	1996	Coconut Oil	1
48	Costa Rica	1996	Coconuts - Incl Copra	3
48	Costa Rica	1996	Coffee	13
48	Costa Rica	1996	Eggs	30
48	Costa Rica	1996	Fats, Animals, Raw	5
48	Costa Rica	1996	Freshwater Fish	4
48	Costa Rica	1996	Fruits, Other	91
48	Costa Rica	1996	Grapefruit	10
48	Costa Rica	1996	Grapes	4
48	Costa Rica	1996	Groundnuts (Shelled Eq)	1
48	Costa Rica	1996	Honey	1
48	Costa Rica	1996	Lemons, Limes	1
48	Costa Rica	1996	Maize	18
48	Costa Rica	1996	Maize Germ Oil	1
48	Costa Rica	1996	Marine Fish, Other	4
48	Costa Rica	1996	Milk, Whole	342
48	Costa Rica	1996	Molluscs, Other	1
48	Costa Rica	1996	Nuts	2
48	Costa Rica	1996	Oats	2
48	Costa Rica	1996	Offals, Edible	8
48	Costa Rica	1996	Onions	12
48	Costa Rica	1996	Oranges, Mandarines	40
48	Costa Rica	1996	Palm Oil	22
48	Costa Rica	1996	Palmkernel Oil	1
48	Costa Rica	1996	Pelagic Fish	2
48	Costa Rica	1996	Pepper	1
48	Costa Rica	1996	Pigmeat	15
48	Costa Rica	1996	Pineapples	207
48	Costa Rica	1996	Plantains	13
48	Costa Rica	1996	Potatoes	39

FAO country code	Country	Reference year	Food item	Gross food supply (g *capita*$^{-1}$ d^{-1})
48	Costa Rica	1996	Poultry Meat	50
48	Costa Rica	1996	Pulses, Other	1
48	Costa Rica	1996	Rice (Milled Equivalent)	116
48	Costa Rica	1996	Roots, Other	17
48	Costa Rica	1996	Soyabean Oil	16
48	Costa Rica	1996	Soyabeans	10
48	Costa Rica	1996	Spices, Other	5
48	Costa Rica	1996	Sugar, Non-Centrifugal	9
48	Costa Rica	1996	Sugar, Refined Equivalent	135
48	Costa Rica	1996	Sweeteners, Other	3
48	Costa Rica	1996	Tomatoes	12
48	Costa Rica	1996	Vegetables, Other	68
48	Costa Rica	1996	Wheat	159
48	Costa Rica	1996	Wine	2
48	Costa Rica	1997	Apples	9
48	Costa Rica	1997	Bananas	1
48	Costa Rica	1997	Barley	7
48	Costa Rica	1997	Beans	23
48	Costa Rica	1997	Beer	33
48	Costa Rica	1997	Beverages, Alcoholic	21
48	Costa Rica	1997	Bovine Meat	52
48	Costa Rica	1997	Butter, Ghee	3
48	Costa Rica	1997	Cassava	4
48	Costa Rica	1997	Cephalopods	1
48	Costa Rica	1997	Cheese	5
48	Costa Rica	1997	Citrus, Other	1
48	Costa Rica	1997	Coconuts - Incl Copra	3
48	Costa Rica	1997	Coffee	13
48	Costa Rica	1997	Eggs	31
48	Costa Rica	1997	Fats, Animals, Raw	5
48	Costa Rica	1997	Freshwater Fish	3
48	Costa Rica	1997	Fruits, Other	80
48	Costa Rica	1997	Grapefruit	13
48	Costa Rica	1997	Grapes	4
48	Costa Rica	1997	Groundnuts (Shelled Eq)	2
48	Costa Rica	1997	Honey	1
48	Costa Rica	1997	Lemons, Limes	1
48	Costa Rica	1997	Maize	17
48	Costa Rica	1997	Maize Germ Oil	1
48	Costa Rica	1997	Marine Fish, Other	6
48	Costa Rica	1997	Milk, Whole	360
48	Costa Rica	1997	Molluscs, Other	1
48	Costa Rica	1997	Nuts	1
48	Costa Rica	1997	Oats	3
48	Costa Rica	1997	Offals, Edible	7
48	Costa Rica	1997	Onions	10
48	Costa Rica	1997	Oranges, Mandarines	69
48	Costa Rica	1997	Palm Oil	15
48	Costa Rica	1997	Palmkernel Oil	1
48	Costa Rica	1997	Pelagic Fish	2
48	Costa Rica	1997	Pepper	2
48	Costa Rica	1997	Pigmeat	16
48	Costa Rica	1997	Pineapples	188
48	Costa Rica	1997	Plantains	13
48	Costa Rica	1997	Potatoes	37
48	Costa Rica	1997	Poultry Meat	46
48	Costa Rica	1997	Pulses, Other	1
48	Costa Rica	1997	Rice (Milled Equivalent)	145
48	Costa Rica	1997	Roots, Other	14
48	Costa Rica	1997	Soyabean Oil	17
48	Costa Rica	1997	Soyabeans	10
48	Costa Rica	1997	Spices, Other	5
48	Costa Rica	1997	Sugar, Non-Centrifugal	9
48	Costa Rica	1997	Sugar, Refined Equivalent	133
48	Costa Rica	1997	Sweeteners, Other	3
48	Costa Rica	1997	Tomatoes	16
48	Costa Rica	1997	Vegetables, Other	83
48	Costa Rica	1997	Wheat	128
48	Costa Rica	1997	Wine	2
48	Costa Rica	1998	Apples	10
48	Costa Rica	1998	Bananas	1
48	Costa Rica	1998	Barley	8
48	Costa Rica	1998	Beans	20
48	Costa Rica	1998	Beer	32
48	Costa Rica	1998	Beverages, Alcoholic	21
48	Costa Rica	1998	Bovine Meat	51
48	Costa Rica	1998	Butter, Ghee	3
48	Costa Rica	1998	Cassava	15
48	Costa Rica	1998	Cephalopods	2
48	Costa Rica	1998	Cheese	5
48	Costa Rica	1998	Coconuts - Incl Copra	4
48	Costa Rica	1998	Coffee	13
48	Costa Rica	1998	Demersal Fish	3
48	Costa Rica	1998	Eggs	26
48	Costa Rica	1998	Fats, Animals, Raw	5
48	Costa Rica	1998	Freshwater Fish	5
48	Costa Rica	1998	Fruits, Other	55
48	Costa Rica	1998	Grapefruit	10
48	Costa Rica	1998	Grapes	4
48	Costa Rica	1998	Groundnuts (Shelled Eq)	1
48	Costa Rica	1998	Honey	1
48	Costa Rica	1998	Lemons, Limes	1
48	Costa Rica	1998	Maize	12
48	Costa Rica	1998	Maize Germ Oil	1
48	Costa Rica	1998	Marine Fish, Other	2
48	Costa Rica	1998	Milk, Whole	393
48	Costa Rica	1998	Molluscs, Other	1

FAO country code	Country	Reference year	Food item	Gross food supply (g *capita*$^{-1}$ d^{-1})
48	Costa Rica	1998	Nuts	1
48	Costa Rica	1998	Oats	3
48	Costa Rica	1998	Offals, Edible	7
48	Costa Rica	1998	Onions	12
48	Costa Rica	1998	Oranges, Mandarines	76
48	Costa Rica	1998	Palm Oil	13
48	Costa Rica	1998	Pelagic Fish	2
48	Costa Rica	1998	Pigmeat	17
48	Costa Rica	1998	Pineapples	175
48	Costa Rica	1998	Plantains	17
48	Costa Rica	1998	Potatoes	57
48	Costa Rica	1998	Poultry Meat	47
48	Costa Rica	1998	Pulses, Other	1
48	Costa Rica	1998	Rice (Milled Equivalent)	154
48	Costa Rica	1998	Roots, Other	10
48	Costa Rica	1998	Soyabean Oil	17
48	Costa Rica	1998	Soyabeans	3
48	Costa Rica	1998	Spices, Other	5
48	Costa Rica	1998	Sugar, Non-Centrifugal	8
48	Costa Rica	1998	Sugar, Refined Equivalent	134
48	Costa Rica	1998	Sunflowerseed Oil	1
48	Costa Rica	1998	Sweeteners, Other	3
48	Costa Rica	1998	Tomatoes	31
48	Costa Rica	1998	Vegetables, Other	84
48	Costa Rica	1998	Wheat	135
48	Costa Rica	1998	Wine	2
48	Costa Rica	1999	Apples	10
48	Costa Rica	1999	Bananas	1
48	Costa Rica	1999	Barley	9
48	Costa Rica	1999	Beans	27
48	Costa Rica	1999	Beer	31
48	Costa Rica	1999	Beverages, Alcoholic	20
48	Costa Rica	1999	Bovine Meat	49
48	Costa Rica	1999	Butter, Ghee	3
48	Costa Rica	1999	Cassava	15
48	Costa Rica	1999	Cephalopods	2
48	Costa Rica	1999	Cheese	6
48	Costa Rica	1999	Coconuts - Incl Copra	4
48	Costa Rica	1999	Coffee	17
48	Costa Rica	1999	Crustaceans	1
48	Costa Rica	1999	Demersal Fish	5
48	Costa Rica	1999	Eggs	25
48	Costa Rica	1999	Fats, Animals, Raw	6
48	Costa Rica	1999	Freshwater Fish	6
48	Costa Rica	1999	Fruits, Other	67
48	Costa Rica	1999	Grapefruit	12
48	Costa Rica	1999	Grapes	5
48	Costa Rica	1999	Groundnuts (Shelled Eq)	1
48	Costa Rica	1999	Honey	1
48	Costa Rica	1999	Lemons, Limes	1
48	Costa Rica	1999	Maize	13
48	Costa Rica	1999	Maize Germ Oil	1
48	Costa Rica	1999	Marine Fish, Other	1
48	Costa Rica	1999	Milk, Whole	403
48	Costa Rica	1999	Molluscs, Other	1
48	Costa Rica	1999	Nuts	1
48	Costa Rica	1999	Oats	3
48	Costa Rica	1999	Offals, Edible	7
48	Costa Rica	1999	Onions	19
48	Costa Rica	1999	Oranges, Mandarines	78
48	Costa Rica	1999	Palm Oil	14
48	Costa Rica	1999	Palmkernel Oil	1
48	Costa Rica	1999	Pelagic Fish	1
48	Costa Rica	1999	Pigmeat	20
48	Costa Rica	1999	Pineapples	266
48	Costa Rica	1999	Plantains	19
48	Costa Rica	1999	Potatoes	50
48	Costa Rica	1999	Poultry Meat	52
48	Costa Rica	1999	Pulses, Other	1
48	Costa Rica	1999	Rice (Milled Equivalent)	141
48	Costa Rica	1999	Roots, Other	29
48	Costa Rica	1999	Soyabean Oil	17
48	Costa Rica	1999	Soyabeans	5
48	Costa Rica	1999	Spices, Other	3
48	Costa Rica	1999	Sugar, Non-Centrifugal	8
48	Costa Rica	1999	Sugar, Refined Equivalent	134
48	Costa Rica	1999	Sunflowerseed Oil	1
48	Costa Rica	1999	Sweeteners, Other	4
48	Costa Rica	1999	Tomatoes	24
48	Costa Rica	1999	Vegetables, Other	62
48	Costa Rica	1999	Wheat	132
48	Costa Rica	1999	Wine	3
48	Costa Rica	2000	Apples	12
48	Costa Rica	2000	Bananas	1
48	Costa Rica	2000	Barley	8
48	Costa Rica	2000	Beans	28
48	Costa Rica	2000	Beer	32
48	Costa Rica	2000	Beverages, Alcoholic	21
48	Costa Rica	2000	Bovine Meat	46
48	Costa Rica	2000	Butter, Ghee	3
48	Costa Rica	2000	Cassava	9
48	Costa Rica	2000	Cephalopods	1
48	Costa Rica	2000	Cheese	5
48	Costa Rica	2000	Cocoa Beans	1
48	Costa Rica	2000	Coconuts - Incl Copra	4
48	Costa Rica	2000	Coffee	15
48	Costa Rica	2000	Crustaceans	2

FAO country code	Country	Reference year	Food item	Gross food supply (g $capita^{-1}$ d^{-1})
48	Costa Rica	2000	Demersal Fish	1
48	Costa Rica	2000	Eggs	25
48	Costa Rica	2000	Fats, Animals, Raw	7
48	Costa Rica	2000	Freshwater Fish	3
48	Costa Rica	2000	Fruits, Other	81
48	Costa Rica	2000	Grapefruit	10
48	Costa Rica	2000	Grapes	5
48	Costa Rica	2000	Groundnuts (Shelled Eq)	1
48	Costa Rica	2000	Honey	1
48	Costa Rica	2000	Lemons, Limes	4
48	Costa Rica	2000	Maize	8
48	Costa Rica	2000	Marine Fish, Other	2
48	Costa Rica	2000	Milk, Whole	403
48	Costa Rica	2000	Molluscs, Other	2
48	Costa Rica	2000	Nuts	1
48	Costa Rica	2000	Oats	2
48	Costa Rica	2000	Offals, Edible	6
48	Costa Rica	2000	Onions	14
48	Costa Rica	2000	Oranges, Mandarines	29
48	Costa Rica	2000	Palm Oil	14
48	Costa Rica	2000	Palmkernel Oil	1
48	Costa Rica	2000	Pelagic Fish	1
48	Costa Rica	2000	Pigmeat	21
48	Costa Rica	2000	Pineapples	269
48	Costa Rica	2000	Plantains	20
48	Costa Rica	2000	Potatoes	54
48	Costa Rica	2000	Poultry Meat	50
48	Costa Rica	2000	Pulses, Other	1
48	Costa Rica	2000	Rice (Milled Equivalent)	145
48	Costa Rica	2000	Roots, Other	14
48	Costa Rica	2000	Soyabean Oil	17
48	Costa Rica	2000	Soyabeans	17
48	Costa Rica	2000	Spices, Other	2
48	Costa Rica	2000	Sugar, Non-Centrifugal	8
48	Costa Rica	2000	Sugar, Refined Equivalent	131
48	Costa Rica	2000	Sunflowerseed Oil	1
48	Costa Rica	2000	Sweeteners, Other	3
48	Costa Rica	2000	Tomatoes	29
48	Costa Rica	2000	Vegetables, Other	66
48	Costa Rica	2000	Wheat	128
48	Costa Rica	2000	Wine	3
48	Costa Rica	2000	Yams	1
48	Costa Rica	2001	Apples	11
48	Costa Rica	2001	Bananas	1
48	Costa Rica	2001	Barley	9
48	Costa Rica	2001	Beans	27
48	Costa Rica	2001	Beer	33
48	Costa Rica	2001	Beverages, Alcoholic	21
48	Costa Rica	2001	Bovine Meat	43
48	Costa Rica	2001	Butter, Ghee	3
48	Costa Rica	2001	Cassava	11
48	Costa Rica	2001	Cephalopods	1
48	Costa Rica	2001	Cheese	6
48	Costa Rica	2001	Cocoa Beans	1
48	Costa Rica	2001	Coconuts - Incl Copra	5
48	Costa Rica	2001	Coffee	12
48	Costa Rica	2001	Crustaceans	1
48	Costa Rica	2001	Demersal Fish	1
48	Costa Rica	2001	Eggs	28
48	Costa Rica	2001	Fats, Animals, Raw	8
48	Costa Rica	2001	Freshwater Fish	3
48	Costa Rica	2001	Fruits, Other	75
48	Costa Rica	2001	Grapefruit	11
48	Costa Rica	2001	Grapes	5
48	Costa Rica	2001	Groundnuts (Shelled Eq)	1
48	Costa Rica	2001	Honey	1
48	Costa Rica	2001	Lemons, Limes	8
48	Costa Rica	2001	Maize	9
48	Costa Rica	2001	Maize Germ Oil	1
48	Costa Rica	2001	Marine Fish, Other	1
48	Costa Rica	2001	Milk, Whole	407
48	Costa Rica	2001	Molluscs, Other	2
48	Costa Rica	2001	Nuts	1
48	Costa Rica	2001	Oats	3
48	Costa Rica	2001	Offals, Edible	6
48	Costa Rica	2001	Onions	19
48	Costa Rica	2001	Oranges, Mandarines	76
48	Costa Rica	2001	Palm Oil	14
48	Costa Rica	2001	Pelagic Fish	2
48	Costa Rica	2001	Pigmeat	23
48	Costa Rica	2001	Pineapples	244
48	Costa Rica	2001	Plantains	31
48	Costa Rica	2001	Potatoes	58
48	Costa Rica	2001	Poultry Meat	51
48	Costa Rica	2001	Pulses, Other	1
48	Costa Rica	2001	Rice (Milled Equivalent)	147
48	Costa Rica	2001	Roots, Other	21
48	Costa Rica	2001	Soyabean Oil	17
48	Costa Rica	2001	Soyabeans	4
48	Costa Rica	2001	Spices, Other	1
48	Costa Rica	2001	Sugar, Non-Centrifugal	8
48	Costa Rica	2001	Sugar, Refined Equivalent	128
48	Costa Rica	2001	Sunflowerseed Oil	1
48	Costa Rica	2001	Sweeteners, Other	3
48	Costa Rica	2001	Tomatoes	41
48	Costa Rica	2001	Vegetables, Other	64
48	Costa Rica	2001	Wheat	130

FAO country code	Country	Reference year	Food item	Gross food supply (g *capita*$^{-1}$ d^{-1})
48	Costa Rica	2001	Wine	3
48	Costa Rica	2001	Yams	1
48	Costa Rica	2002	Apples	14
48	Costa Rica	2002	Bananas	1
48	Costa Rica	2002	Barley	10
48	Costa Rica	2002	Beans	27
48	Costa Rica	2002	Beer	32
48	Costa Rica	2002	Beverages, Alcoholic	21
48	Costa Rica	2002	Bovine Meat	33
48	Costa Rica	2002	Butter, Ghee	3
48	Costa Rica	2002	Cassava	5
48	Costa Rica	2002	Cephalopods	1
48	Costa Rica	2002	Cheese	6
48	Costa Rica	2002	Cocoa Beans	1
48	Costa Rica	2002	Coconuts - Incl Copra	4
48	Costa Rica	2002	Coffee	11
48	Costa Rica	2002	Crustaceans	2
48	Costa Rica	2002	Demersal Fish	1
48	Costa Rica	2002	Eggs	28
48	Costa Rica	2002	Fats, Animals, Raw	8
48	Costa Rica	2002	Freshwater Fish	8
48	Costa Rica	2002	Fruits, Other	80
48	Costa Rica	2002	Grapefruit	11
48	Costa Rica	2002	Grapes	5
48	Costa Rica	2002	Groundnuts (Shelled Eq)	1
48	Costa Rica	2002	Honey	1
48	Costa Rica	2002	Lemons, Limes	7
48	Costa Rica	2002	Maize	10
48	Costa Rica	2002	Marine Fish, Other	1
48	Costa Rica	2002	Milk, Whole	416
48	Costa Rica	2002	Molluscs, Other	2
48	Costa Rica	2002	Nuts	2
48	Costa Rica	2002	Oats	3
48	Costa Rica	2002	Offals, Edible	5
48	Costa Rica	2002	Olive Oil	1
48	Costa Rica	2002	Onions	18
48	Costa Rica	2002	Oranges, Mandarines	106
48	Costa Rica	2002	Palm Oil	14
48	Costa Rica	2002	Pelagic Fish	2
48	Costa Rica	2002	Pigmeat	23
48	Costa Rica	2002	Pineapples	185
48	Costa Rica	2002	Plantains	20
48	Costa Rica	2002	Potatoes	56
48	Costa Rica	2002	Poultry Meat	52
48	Costa Rica	2002	Pulses, Other	1
48	Costa Rica	2002	Rice (Milled Equivalent)	143
48	Costa Rica	2002	Roots, Other	28
48	Costa Rica	2002	Soyabean Oil	17
48	Costa Rica	2002	Soyabeans	8
48	Costa Rica	2002	Sugar, Non-Centrifugal	8
48	Costa Rica	2002	Sugar, Refined Equivalent	128
48	Costa Rica	2002	Sunflowerseed	1
48	Costa Rica	2002	Sunflowerseed Oil	1
48	Costa Rica	2002	Sweeteners, Other	4
48	Costa Rica	2002	Tomatoes	47
48	Costa Rica	2002	Vegetables, Other	61
48	Costa Rica	2002	Wheat	113
48	Costa Rica	2002	Wine	3
48	Costa Rica	2003	Apples	15
48	Costa Rica	2003	Bananas	1
48	Costa Rica	2003	Barley	9
48	Costa Rica	2003	Beans	27
48	Costa Rica	2003	Beer	31
48	Costa Rica	2003	Beverages, Alcoholic	20
48	Costa Rica	2003	Bovine Meat	40
48	Costa Rica	2003	Butter, Ghee	3
48	Costa Rica	2003	Cassava	5
48	Costa Rica	2003	Cephalopods	1
48	Costa Rica	2003	Cheese	6
48	Costa Rica	2003	Citrus, Other	1
48	Costa Rica	2003	Cocoa Beans	2
48	Costa Rica	2003	Coconuts - Incl Copra	5
48	Costa Rica	2003	Coffee	6
48	Costa Rica	2003	Crustaceans	4
48	Costa Rica	2003	Demersal Fish	1
48	Costa Rica	2003	Eggs	26
48	Costa Rica	2003	Fats, Animals, Raw	7
48	Costa Rica	2003	Freshwater Fish	11
48	Costa Rica	2003	Fruits, Other	78
48	Costa Rica	2003	Grapefruit	11
48	Costa Rica	2003	Grapes	6
48	Costa Rica	2003	Groundnuts (Shelled Eq)	2
48	Costa Rica	2003	Honey	1
48	Costa Rica	2003	Lemons, Limes	7
48	Costa Rica	2003	Maize	18
48	Costa Rica	2003	Milk, Whole	409
48	Costa Rica	2003	Molluscs, Other	1
48	Costa Rica	2003	Nuts	3
48	Costa Rica	2003	Oats	3
48	Costa Rica	2003	Offals, Edible	5
48	Costa Rica	2003	Olive Oil	1
48	Costa Rica	2003	Onions	16
48	Costa Rica	2003	Oranges, Mandarines	57
48	Costa Rica	2003	Palm Oil	14
48	Costa Rica	2003	Pelagic Fish	2
48	Costa Rica	2003	Pepper	1
48	Costa Rica	2003	Pigmeat	24

FAO country code	Country	Reference year	Food item	Gross food supply (g $capita^{-1}$ d^{-1})
48	Costa Rica	2003	Pineapples	134
48	Costa Rica	2003	Plantains	20
48	Costa Rica	2003	Potatoes	52
48	Costa Rica	2003	Poultry Meat	48
48	Costa Rica	2003	Pulses, Other	1
48	Costa Rica	2003	Rice (Milled Equivalent)	142
48	Costa Rica	2003	Roots, Other	28
48	Costa Rica	2003	Soyabean Oil	17
48	Costa Rica	2003	Soyabeans	7
48	Costa Rica	2003	Spices, Other	1
48	Costa Rica	2003	Sugar, Non-Centrifugal	8
48	Costa Rica	2003	Sugar, Refined Equivalent	130
48	Costa Rica	2003	Sunflowerseed Oil	1
48	Costa Rica	2003	Sweeteners, Other	4
48	Costa Rica	2003	Tomatoes	40
48	Costa Rica	2003	Vegetables, Other	65
48	Costa Rica	2003	Wheat	114
48	Costa Rica	2003	Wine	3
48	Costa Rica	2004	Apples	15
48	Costa Rica	2004	Bananas	2
48	Costa Rica	2004	Barley	7
48	Costa Rica	2004	Beans	27
48	Costa Rica	2004	Beer	30
48	Costa Rica	2004	Beverages, Alcoholic	21
48	Costa Rica	2004	Bovine Meat	36
48	Costa Rica	2004	Butter, Ghee	3
48	Costa Rica	2004	Cassava	6
48	Costa Rica	2004	Cephalopods	1
48	Costa Rica	2004	Cheese	5
48	Costa Rica	2004	Cocoa Beans	3
48	Costa Rica	2004	Coconuts - Incl Copra	6
48	Costa Rica	2004	Coffee	11
48	Costa Rica	2004	Crustaceans	4
48	Costa Rica	2004	Demersal Fish	1
48	Costa Rica	2004	Eggs	28
48	Costa Rica	2004	Fats, Animals, Raw	8
48	Costa Rica	2004	Freshwater Fish	12
48	Costa Rica	2004	Fruits, Other	83
48	Costa Rica	2004	Grapefruit	13
48	Costa Rica	2004	Grapes	6
48	Costa Rica	2004	Groundnuts (Shelled Eq)	1
48	Costa Rica	2004	Honey	1
48	Costa Rica	2004	Lemons, Limes	6
48	Costa Rica	2004	Maize	32
48	Costa Rica	2004	Milk, Whole	382
48	Costa Rica	2004	Molluscs, Other	2
48	Costa Rica	2004	Nuts	2
48	Costa Rica	2004	Oats	3
48	Costa Rica	2004	Offals, Edible	5
48	Costa Rica	2004	Olive Oil	1
48	Costa Rica	2004	Onions	20
48	Costa Rica	2004	Oranges, Mandarines	5
48	Costa Rica	2004	Palm Oil	15
48	Costa Rica	2004	Pelagic Fish	2
48	Costa Rica	2004	Pepper	1
48	Costa Rica	2004	Pigmeat	23
48	Costa Rica	2004	Pineapples	81
48	Costa Rica	2004	Plantains	19
48	Costa Rica	2004	Potatoes	50
48	Costa Rica	2004	Poultry Meat	54
48	Costa Rica	2004	Pulses, Other	1
48	Costa Rica	2004	Rice (Milled Equivalent)	147
48	Costa Rica	2004	Roots, Other	24
48	Costa Rica	2004	Soyabean Oil	18
48	Costa Rica	2004	Soyabeans	7
48	Costa Rica	2004	Sugar, Non-Centrifugal	7
48	Costa Rica	2004	Sugar, Refined Equivalent	128
48	Costa Rica	2004	Sunflowerseed Oil	2
48	Costa Rica	2004	Sweeteners, Other	4
48	Costa Rica	2004	Tea	1
48	Costa Rica	2004	Tomatoes	60
48	Costa Rica	2004	Vegetables, Other	46
48	Costa Rica	2004	Wheat	97
48	Costa Rica	2004	Wine	3
48	Costa Rica	2005	Apples	12
48	Costa Rica	2005	Bananas	2
48	Costa Rica	2005	Barley	6
48	Costa Rica	2005	Beans	28
48	Costa Rica	2005	Beer	27
48	Costa Rica	2005	Beverages, Alcoholic	20
48	Costa Rica	2005	Bovine Meat	40
48	Costa Rica	2005	Butter, Ghee	3
48	Costa Rica	2005	Cassava	2
48	Costa Rica	2005	Cephalopods	1
48	Costa Rica	2005	Cheese	6
48	Costa Rica	2005	Citrus, Other	1
48	Costa Rica	2005	Cocoa Beans	2
48	Costa Rica	2005	Coconut Oil	1
48	Costa Rica	2005	Coconuts - Incl Copra	5
48	Costa Rica	2005	Coffee	8
48	Costa Rica	2005	Crustaceans	4
48	Costa Rica	2005	Demersal Fish	1
48	Costa Rica	2005	Eggs	25
48	Costa Rica	2005	Fats, Animals, Raw	7
48	Costa Rica	2005	Freshwater Fish	12
48	Costa Rica	2005	Fruits, Other	76
48	Costa Rica	2005	Grapefruit	11

FAO country code	Country	Reference year	Food item	Gross food supply (g *capita*$^{-1}$ d^{-1})
48	Costa Rica	2005	Grapes	3
48	Costa Rica	2005	Groundnuts (Shelled Eq)	1
48	Costa Rica	2005	Honey	1
48	Costa Rica	2005	Lemons, Limes	15
48	Costa Rica	2005	Maize	33
48	Costa Rica	2005	Milk, Whole	386
48	Costa Rica	2005	Molluscs, Other	2
48	Costa Rica	2005	Nuts	2
48	Costa Rica	2005	Oats	1
48	Costa Rica	2005	Offals, Edible	4
48	Costa Rica	2005	Olive Oil	1
48	Costa Rica	2005	Onions	18
48	Costa Rica	2005	Oranges, Mandarines	79
48	Costa Rica	2005	Palm Oil	15
48	Costa Rica	2005	Pelagic Fish	2
48	Costa Rica	2005	Pigmeat	22
48	Costa Rica	2005	Pineapples	200
48	Costa Rica	2005	Plantains	15
48	Costa Rica	2005	Potatoes	47
48	Costa Rica	2005	Poultry Meat	56
48	Costa Rica	2005	Pulses, Other	1
48	Costa Rica	2005	Rice (Milled Equivalent)	146
48	Costa Rica	2005	Roots, Other	22
48	Costa Rica	2005	Soyabean Oil	18
48	Costa Rica	2005	Soyabeans	8
48	Costa Rica	2005	Spices, Other	2
48	Costa Rica	2005	Sugar, Non-Centrifugal	7
48	Costa Rica	2005	Sugar, Refined Equivalent	133
48	Costa Rica	2005	Sunflowerseed Oil	4
48	Costa Rica	2005	Sweeteners, Other	4
48	Costa Rica	2005	Tomatoes	57
48	Costa Rica	2005	Vegetables, Other	32
48	Costa Rica	2005	Wheat	95
48	Costa Rica	2005	Wine	3
48	Costa Rica	2005	Yams	1
48	Costa Rica	2006	Apples	15
48	Costa Rica	2006	Bananas	1
48	Costa Rica	2006	Barley	11
48	Costa Rica	2006	Beans	30
48	Costa Rica	2006	Beer	44
48	Costa Rica	2006	Beverages, Alcoholic	20
48	Costa Rica	2006	Bovine Meat	39
48	Costa Rica	2006	Butter, Ghee	3
48	Costa Rica	2006	Cassava	1
48	Costa Rica	2006	Cephalopods	1
48	Costa Rica	2006	Cereals, Other	1
48	Costa Rica	2006	Cheese	6
48	Costa Rica	2006	Citrus, Other	1
48	Costa Rica	2006	Cocoa Beans	4
48	Costa Rica	2006	Coconuts - Incl Copra	5
48	Costa Rica	2006	Coffee	2
48	Costa Rica	2006	Crustaceans	5
48	Costa Rica	2006	Demersal Fish	1
48	Costa Rica	2006	Eggs	24
48	Costa Rica	2006	Fats, Animals, Raw	8
48	Costa Rica	2006	Freshwater Fish	10
48	Costa Rica	2006	Fruits, Other	83
48	Costa Rica	2006	Grapefruit	13
48	Costa Rica	2006	Grapes	7
48	Costa Rica	2006	Groundnuts (Shelled Eq)	2
48	Costa Rica	2006	Honey	1
48	Costa Rica	2006	Lemons, Limes	18
48	Costa Rica	2006	Maize	32
48	Costa Rica	2006	Marine Fish, Other	1
48	Costa Rica	2006	Milk, Whole	397
48	Costa Rica	2006	Molluscs, Other	3
48	Costa Rica	2006	Nuts	2
48	Costa Rica	2006	Oats	3
48	Costa Rica	2006	Offals, Edible	5
48	Costa Rica	2006	Olive Oil	1
48	Costa Rica	2006	Onions	27
48	Costa Rica	2006	Oranges, Mandarines	98
48	Costa Rica	2006	Palm Oil	16
48	Costa Rica	2006	Peas	1
48	Costa Rica	2006	Pelagic Fish	3
48	Costa Rica	2006	Pepper	1
48	Costa Rica	2006	Pigmeat	23
48	Costa Rica	2006	Pineapples	182
48	Costa Rica	2006	Plantains	17
48	Costa Rica	2006	Potatoes	43
48	Costa Rica	2006	Poultry Meat	62
48	Costa Rica	2006	Pulses, Other	1
48	Costa Rica	2006	Rice (Milled Equivalent)	133
48	Costa Rica	2006	Roots, Other	17
48	Costa Rica	2006	Soyabean Oil	18
48	Costa Rica	2006	Soyabeans	8
48	Costa Rica	2006	Spices, Other	1
48	Costa Rica	2006	Sugar, Non-Centrifugal	7
48	Costa Rica	2006	Sugar, Refined Equivalent	133
48	Costa Rica	2006	Sunflowerseed	1
48	Costa Rica	2006	Sunflowerseed Oil	4
48	Costa Rica	2006	Sweeteners, Other	4
48	Costa Rica	2006	Tomatoes	69
48	Costa Rica	2006	Vegetables, Other	61
48	Costa Rica	2006	Wheat	95
48	Costa Rica	2006	Wine	5
48	Costa Rica	2006	Yams	7

FAO country code	Country	Reference year	Food item	Gross food supply (g $capita^{-1}$ d^{-1})
48	Costa Rica	2007	Apples	13
48	Costa Rica	2007	Bananas	1
48	Costa Rica	2007	Barley	12
48	Costa Rica	2007	Beans	30
48	Costa Rica	2007	Beer	42
48	Costa Rica	2007	Beverages, Alcoholic	22
48	Costa Rica	2007	Bovine Meat	43
48	Costa Rica	2007	Butter, Ghee	4
48	Costa Rica	2007	Cassava	1
48	Costa Rica	2007	Cephalopods	1
48	Costa Rica	2007	Cereals, Other	1
48	Costa Rica	2007	Cheese	7
48	Costa Rica	2007	Cocoa Beans	3
48	Costa Rica	2007	Coconuts - Incl Copra	4
48	Costa Rica	2007	Coffee	11
48	Costa Rica	2007	Crustaceans	5
48	Costa Rica	2007	Demersal Fish	1
48	Costa Rica	2007	Eggs	21
48	Costa Rica	2007	Fats, Animals, Raw	9
48	Costa Rica	2007	Freshwater Fish	13
48	Costa Rica	2007	Fruits, Other	90
48	Costa Rica	2007	Grapefruit	11
48	Costa Rica	2007	Grapes	6
48	Costa Rica	2007	Groundnuts (Shelled Eq)	2
48	Costa Rica	2007	Honey	1
48	Costa Rica	2007	Lemons, Limes	16
48	Costa Rica	2007	Maize	29
48	Costa Rica	2007	Marine Fish, Other	2
48	Costa Rica	2007	Milk, Whole	400
48	Costa Rica	2007	Molluscs, Other	2
48	Costa Rica	2007	Nuts	2
48	Costa Rica	2007	Oats	4
48	Costa Rica	2007	Offals, Edible	5
48	Costa Rica	2007	Olive Oil	1
48	Costa Rica	2007	Onions	19
48	Costa Rica	2007	Oranges, Mandarines	76
48	Costa Rica	2007	Palm Oil	16
48	Costa Rica	2007	Pelagic Fish	4
48	Costa Rica	2007	Pepper	1
48	Costa Rica	2007	Pigmeat	26
48	Costa Rica	2007	Pineapples	38
48	Costa Rica	2007	Plantains	24
48	Costa Rica	2007	Potatoes	45
48	Costa Rica	2007	Poultry Meat	67
48	Costa Rica	2007	Pulses, Other	1
48	Costa Rica	2007	Rice (Milled Equivalent)	135
48	Costa Rica	2007	Roots, Other	6
48	Costa Rica	2007	Soyabean Oil	18
48	Costa Rica	2007	Soyabeans	8
48	Costa Rica	2007	Spices, Other	1
48	Costa Rica	2007	Sugar, Non-Centrifugal	7
48	Costa Rica	2007	Sugar, Refined Equivalent	135
48	Costa Rica	2007	Sunflowerseed Oil	4
48	Costa Rica	2007	Sweeteners, Other	6
48	Costa Rica	2007	Tomatoes	65
48	Costa Rica	2007	Vegetables, Other	61
48	Costa Rica	2007	Wheat	103
48	Costa Rica	2007	Wine	5
48	Costa Rica	2007	Yams	7
48	Costa Rica	2008	Apples	18
48	Costa Rica	2008	Bananas	1
48	Costa Rica	2008	Barley	10
48	Costa Rica	2008	Beans	29
48	Costa Rica	2008	Beer	38
48	Costa Rica	2008	Beverages, Alcoholic	21
48	Costa Rica	2008	Bovine Meat	43
48	Costa Rica	2008	Butter, Ghee	4
48	Costa Rica	2008	Cassava	2
48	Costa Rica	2008	Cephalopods	1
48	Costa Rica	2008	Cereals, Other	1
48	Costa Rica	2008	Cheese	7
48	Costa Rica	2008	Citrus, Other	1
48	Costa Rica	2008	Cocoa Beans	2
48	Costa Rica	2008	Coconuts - Incl Copra	5
48	Costa Rica	2008	Coffee	6
48	Costa Rica	2008	Crustaceans	5
48	Costa Rica	2008	Demersal Fish	1
48	Costa Rica	2008	Eggs	27
48	Costa Rica	2008	Fats, Animals, Raw	9
48	Costa Rica	2008	Freshwater Fish	15
48	Costa Rica	2008	Fruits, Other	94
48	Costa Rica	2008	Grapefruit	9
48	Costa Rica	2008	Grapes	6
48	Costa Rica	2008	Groundnuts (Shelled Eq)	2
48	Costa Rica	2008	Honey	1
48	Costa Rica	2008	Lemons, Limes	13
48	Costa Rica	2008	Maize	29
48	Costa Rica	2008	Marine Fish, Other	2
48	Costa Rica	2008	Milk, Whole	419
48	Costa Rica	2008	Molluscs, Other	2
48	Costa Rica	2008	Nuts	1
48	Costa Rica	2008	Oats	4
48	Costa Rica	2008	Offals, Edible	5
48	Costa Rica	2008	Olive Oil	1
48	Costa Rica	2008	Onions	22
48	Costa Rica	2008	Oranges, Mandarines	18
48	Costa Rica	2008	Palm Oil	17

FAO country code	Country	Reference year	Food item	Gross food supply (g $capita^{-1}$ d^{-1})
48	Costa Rica	2008	Pelagic Fish	4
48	Costa Rica	2008	Pepper	1
48	Costa Rica	2008	Pigmeat	27
48	Costa Rica	2008	Pineapples	43
48	Costa Rica	2008	Plantains	30
48	Costa Rica	2008	Potatoes	47
48	Costa Rica	2008	Poultry Meat	64
48	Costa Rica	2008	Pulses, Other	1
48	Costa Rica	2008	Rice (Milled Equivalent)	133
48	Costa Rica	2008	Roots, Other	16
48	Costa Rica	2008	Soyabean Oil	18
48	Costa Rica	2008	Soyabeans	7
48	Costa Rica	2008	Sugar, Non-Centrifugal	7
48	Costa Rica	2008	Sugar, Refined Equivalent	130
48	Costa Rica	2008	Sunflowerseed	1
48	Costa Rica	2008	Sunflowerseed Oil	2
48	Costa Rica	2008	Sweeteners, Other	4
48	Costa Rica	2008	Tomatoes	59
48	Costa Rica	2008	Vegetables, Other	74
48	Costa Rica	2008	Wheat	107
48	Costa Rica	2008	Wine	5
48	Costa Rica	2008	Yams	7
48	Costa Rica	2009	Apples	18
48	Costa Rica	2009	Bananas	1
48	Costa Rica	2009	Barley	8
48	Costa Rica	2009	Beans	29
48	Costa Rica	2009	Beer	39
48	Costa Rica	2009	Beverages, Alcoholic	21
48	Costa Rica	2009	Bovine Meat	45
48	Costa Rica	2009	Butter, Ghee	4
48	Costa Rica	2009	Cassava	6
48	Costa Rica	2009	Cephalopods	1
48	Costa Rica	2009	Cereals, Other	1
48	Costa Rica	2009	Cheese	7
48	Costa Rica	2009	Cocoa Beans	3
48	Costa Rica	2009	Coconuts - Incl Copra	6
48	Costa Rica	2009	Coffee	9
48	Costa Rica	2009	Crustaceans	4
48	Costa Rica	2009	Demersal Fish	2
48	Costa Rica	2009	Eggs	26
48	Costa Rica	2009	Fats, Animals, Raw	9
48	Costa Rica	2009	Freshwater Fish	14
48	Costa Rica	2009	Fruits, Other	98
48	Costa Rica	2009	Grapefruit	9
48	Costa Rica	2009	Grapes	6
48	Costa Rica	2009	Groundnuts (Shelled Eq)	2
48	Costa Rica	2009	Honey	1
48	Costa Rica	2009	Lemons, Limes	14
48	Costa Rica	2009	Maize	26
48	Costa Rica	2009	Marine Fish, Other	4
48	Costa Rica	2009	Milk, Whole	411
48	Costa Rica	2009	Molluscs, Other	2
48	Costa Rica	2009	Nuts	2
48	Costa Rica	2009	Oats	4
48	Costa Rica	2009	Offals, Edible	5
48	Costa Rica	2009	Olive Oil	1
48	Costa Rica	2009	Onions	21
48	Costa Rica	2009	Oranges, Mandarines	13
48	Costa Rica	2009	Palm Oil	17
48	Costa Rica	2009	Pelagic Fish	4
48	Costa Rica	2009	Pepper	1
48	Costa Rica	2009	Pigmeat	27
48	Costa Rica	2009	Pineapples	43
48	Costa Rica	2009	Plantains	30
48	Costa Rica	2009	Potatoes	51
48	Costa Rica	2009	Poultry Meat	66
48	Costa Rica	2009	Pulses, Other	1
48	Costa Rica	2009	Rice (Milled Equivalent)	135
48	Costa Rica	2009	Roots, Other	18
48	Costa Rica	2009	Soyabean Oil	18
48	Costa Rica	2009	Soyabeans	8
48	Costa Rica	2009	Spices, Other	1
48	Costa Rica	2009	Sugar, Non-Centrifugal	7
48	Costa Rica	2009	Sugar, Refined Equivalent	128
48	Costa Rica	2009	Sunflowerseed Oil	2
48	Costa Rica	2009	Sweeteners, Other	4
48	Costa Rica	2009	Tomatoes	55
48	Costa Rica	2009	Vegetables, Other	70
48	Costa Rica	2009	Wheat	109
48	Costa Rica	2009	Wine	4
48	Costa Rica	2009	Yams	7
48	Costa Rica	2010	Apples	28
48	Costa Rica	2010	Bananas	1
48	Costa Rica	2010	Barley	8
48	Costa Rica	2010	Beans	29
48	Costa Rica	2010	Beer	36
48	Costa Rica	2010	Beverages, Alcoholic	22
48	Costa Rica	2010	Bovine Meat	49
48	Costa Rica	2010	Butter, Ghee	3
48	Costa Rica	2010	Cassava	5
48	Costa Rica	2010	Cephalopods	1
48	Costa Rica	2010	Cereals, Other	1
48	Costa Rica	2010	Cheese	7
48	Costa Rica	2010	Cocoa Beans	2
48	Costa Rica	2010	Coconuts - Incl Copra	6
48	Costa Rica	2010	Coffee	18
48	Costa Rica	2010	Crustaceans	4

FAO country code	Country	Reference year	Food item	Gross food supply (g $capita^{-1}$ d^{-1})
48	Costa Rica	2010	Demersal Fish	1
48	Costa Rica	2010	Eggs	26
48	Costa Rica	2010	Fats, Animals, Raw	9
48	Costa Rica	2010	Freshwater Fish	14
48	Costa Rica	2010	Fruits, Other	98
48	Costa Rica	2010	Grapefruit	11
48	Costa Rica	2010	Grapes	6
48	Costa Rica	2010	Groundnuts (Shelled Eq)	2
48	Costa Rica	2010	Honey	1
48	Costa Rica	2010	Lemons, Limes	16
48	Costa Rica	2010	Maize	29
48	Costa Rica	2010	Marine Fish, Other	3
48	Costa Rica	2010	Milk, Whole	389
48	Costa Rica	2010	Molluscs, Other	2
48	Costa Rica	2010	Nuts	2
48	Costa Rica	2010	Oats	4
48	Costa Rica	2010	Offals, Edible	5
48	Costa Rica	2010	Olive Oil	1
48	Costa Rica	2010	Onions	21
48	Costa Rica	2010	Oranges, Mandarines	6
48	Costa Rica	2010	Palm Oil	17
48	Costa Rica	2010	Pelagic Fish	6
48	Costa Rica	2010	Pepper	1
48	Costa Rica	2010	Pigmeat	27
48	Costa Rica	2010	Pineapples	99
48	Costa Rica	2010	Plantains	39
48	Costa Rica	2010	Potatoes	42
48	Costa Rica	2010	Poultry Meat	61
48	Costa Rica	2010	Pulses, Other	1
48	Costa Rica	2010	Rice (Milled Equivalent)	136
48	Costa Rica	2010	Roots, Other	12
48	Costa Rica	2010	Soyabean Oil	18
48	Costa Rica	2010	Soyabeans	7
48	Costa Rica	2010	Spices, Other	1
48	Costa Rica	2010	Sugar, Non-Centrifugal	6
48	Costa Rica	2010	Sugar, Refined Equivalent	131
48	Costa Rica	2010	Sunflowerseed Oil	2
48	Costa Rica	2010	Sweeteners, Other	5
48	Costa Rica	2010	Tomatoes	51
48	Costa Rica	2010	Vegetables, Other	59
48	Costa Rica	2010	Wheat	108
48	Costa Rica	2010	Wine	5
48	Costa Rica	2010	Yams	7
48	Costa Rica	2011	Apples	29
48	Costa Rica	2011	Bananas	1
48	Costa Rica	2011	Barley	11
48	Costa Rica	2011	Beans	29
48	Costa Rica	2011	Beer	40
48	Costa Rica	2011	Beverages, Alcoholic	22
48	Costa Rica	2011	Bovine Meat	49
48	Costa Rica	2011	Butter, Ghee	4
48	Costa Rica	2011	Cassava	16
48	Costa Rica	2011	Cephalopods	1
48	Costa Rica	2011	Cereals, Other	1
48	Costa Rica	2011	Cheese	7
48	Costa Rica	2011	Citrus, Other	1
48	Costa Rica	2011	Cocoa Beans	3
48	Costa Rica	2011	Coconuts - Incl Copra	7
48	Costa Rica	2011	Coffee	20
48	Costa Rica	2011	Crustaceans	4
48	Costa Rica	2011	Demersal Fish	1
48	Costa Rica	2011	Eggs	26
48	Costa Rica	2011	Fats, Animals, Raw	10
48	Costa Rica	2011	Freshwater Fish	14
48	Costa Rica	2011	Fruits, Other	94
48	Costa Rica	2011	Grapefruit	11
48	Costa Rica	2011	Grapes	7
48	Costa Rica	2011	Groundnuts (Shelled Eq)	2
48	Costa Rica	2011	Honey	1
48	Costa Rica	2011	Lemons, Limes	17
48	Costa Rica	2011	Maize	29
48	Costa Rica	2011	Marine Fish, Other	3
48	Costa Rica	2011	Milk, Whole	369
48	Costa Rica	2011	Molluscs, Other	2
48	Costa Rica	2011	Nuts	2
48	Costa Rica	2011	Oats	4
48	Costa Rica	2011	Offals, Edible	4
48	Costa Rica	2011	Olive Oil	1
48	Costa Rica	2011	Onions	22
48	Costa Rica	2011	Oranges, Mandarines	6
48	Costa Rica	2011	Palm Oil	18
48	Costa Rica	2011	Pelagic Fish	6
48	Costa Rica	2011	Pepper	1
48	Costa Rica	2011	Pigmeat	28
48	Costa Rica	2011	Pineapples	132
48	Costa Rica	2011	Plantains	40
48	Costa Rica	2011	Potatoes	46
48	Costa Rica	2011	Poultry Meat	58
48	Costa Rica	2011	Pulses, Other	1
48	Costa Rica	2011	Rice (Milled Equivalent)	135
48	Costa Rica	2011	Roots, Other	18
48	Costa Rica	2011	Soyabean Oil	18
48	Costa Rica	2011	Soyabeans	8
48	Costa Rica	2011	Spices, Other	1
48	Costa Rica	2011	Sugar, Non-Centrifugal	6
48	Costa Rica	2011	Sugar, Refined Equivalent	129
48	Costa Rica	2011	Sunflowerseed Oil	2

FAO country code	Country	Reference year	Food item	Gross food supply (g $capita^{-1}$ d^{-1})
48	Costa Rica	2011	Sweeteners, Other	4
48	Costa Rica	2011	Tomatoes	60
48	Costa Rica	2011	Vegetables, Other	50
48	Costa Rica	2011	Wheat	110
48	Costa Rica	2011	Wine	5
48	Costa Rica	2011	Yams	6
107	Côte d'Ivoire	1992	Bananas	6
107	Côte d'Ivoire	1992	Beer	28
107	Côte d'Ivoire	1992	Beverages, Fermented	57
107	Côte d'Ivoire	1992	Bovine Meat	15
107	Côte d'Ivoire	1992	Cassava	286
107	Côte d'Ivoire	1992	Cereals, Other	2
107	Côte d'Ivoire	1992	Citrus, Other	2
107	Côte d'Ivoire	1992	Cocoa Beans	2
107	Côte d'Ivoire	1992	Coconut Oil	1
107	Côte d'Ivoire	1992	Coconuts - Incl Copra	3
107	Côte d'Ivoire	1992	Coffee	2
107	Côte d'Ivoire	1992	Cottonseed Oil	3
107	Côte d'Ivoire	1992	Demersal Fish	4
107	Côte d'Ivoire	1992	Eggs	3
107	Côte d'Ivoire	1992	Freshwater Fish	3
107	Côte d'Ivoire	1992	Fruits, Other	10
107	Côte d'Ivoire	1992	Grapefruit	3
107	Côte d'Ivoire	1992	Groundnuts (Shelled Eq)	8
107	Côte d'Ivoire	1992	Maize	75
107	Côte d'Ivoire	1992	Marine Fish, Other	6
107	Côte d'Ivoire	1992	Meat, Other	25
107	Côte d'Ivoire	1992	Milk, Whole	10
107	Côte d'Ivoire	1992	Millet	5
107	Côte d'Ivoire	1992	Mutton & Goat Meat	2
107	Côte d'Ivoire	1992	Nuts	6
107	Côte d'Ivoire	1992	Offals, Edible	3
107	Côte d'Ivoire	1992	Oranges, Mandarines	5
107	Côte d'Ivoire	1992	Palm Oil	23
107	Côte d'Ivoire	1992	Pelagic Fish	33
107	Côte d'Ivoire	1992	Pigmeat	2
107	Côte d'Ivoire	1992	Pimento	3
107	Côte d'Ivoire	1992	Pineapples	9
107	Côte d'Ivoire	1992	Plantains	207
107	Côte d'Ivoire	1992	Potatoes	2
107	Côte d'Ivoire	1992	Poultry Meat	5
107	Côte d'Ivoire	1992	Pulses, Other	2
107	Côte d'Ivoire	1992	Rape and Mustard Oil	1
107	Côte d'Ivoire	1992	Rice (Milled Equivalent)	150
107	Côte d'Ivoire	1992	Roots, Other	10
107	Côte d'Ivoire	1992	Sorghum	3
107	Côte d'Ivoire	1992	Soyabean Oil	1
107	Côte d'Ivoire	1992	Soyabeans	1
107	Côte d'Ivoire	1992	Sugar Cane	2
107	Côte d'Ivoire	1992	Sugar, Refined Equivalent	27
107	Côte d'Ivoire	1992	Sweet Potatoes	6
107	Côte d'Ivoire	1992	Tomatoes	11
107	Côte d'Ivoire	1992	Vegetables, Other	118
107	Côte d'Ivoire	1992	Wheat	46
107	Côte d'Ivoire	1992	Wine	4
107	Côte d'Ivoire	1992	Yams	492
107	Côte d'Ivoire	1993	Apples	1
107	Côte d'Ivoire	1993	Bananas	7
107	Côte d'Ivoire	1993	Beer	30
107	Côte d'Ivoire	1993	Beverages, Fermented	58
107	Côte d'Ivoire	1993	Bovine Meat	12
107	Côte d'Ivoire	1993	Cassava	278
107	Côte d'Ivoire	1993	Cereals, Other	3
107	Côte d'Ivoire	1993	Citrus, Other	2
107	Côte d'Ivoire	1993	Cocoa Beans	2
107	Côte d'Ivoire	1993	Coconut Oil	1
107	Côte d'Ivoire	1993	Coconuts - Incl Copra	2
107	Côte d'Ivoire	1993	Coffee	1
107	Côte d'Ivoire	1993	Cottonseed Oil	3
107	Côte d'Ivoire	1993	Demersal Fish	3
107	Côte d'Ivoire	1993	Eggs	3
107	Côte d'Ivoire	1993	Freshwater Fish	3
107	Côte d'Ivoire	1993	Fruits, Other	10
107	Côte d'Ivoire	1993	Grapefruit	3
107	Côte d'Ivoire	1993	Groundnuts (Shelled Eq)	8
107	Côte d'Ivoire	1993	Maize	73
107	Côte d'Ivoire	1993	Marine Fish, Other	7
107	Côte d'Ivoire	1993	Meat, Other	27
107	Côte d'Ivoire	1993	Milk, Whole	15
107	Côte d'Ivoire	1993	Millet	5
107	Côte d'Ivoire	1993	Mutton & Goat Meat	2
107	Côte d'Ivoire	1993	Nuts	6
107	Côte d'Ivoire	1993	Offals, Edible	3
107	Côte d'Ivoire	1993	Oranges, Mandarines	5
107	Côte d'Ivoire	1993	Palm Oil	20
107	Côte d'Ivoire	1993	Pelagic Fish	27
107	Côte d'Ivoire	1993	Pigmeat	2
107	Côte d'Ivoire	1993	Pimento	3
107	Côte d'Ivoire	1993	Pineapples	13
107	Côte d'Ivoire	1993	Plantains	202
107	Côte d'Ivoire	1993	Potatoes	2
107	Côte d'Ivoire	1993	Poultry Meat	5
107	Côte d'Ivoire	1993	Pulses, Other	1
107	Côte d'Ivoire	1993	Rape and Mustard Oil	1
107	Côte d'Ivoire	1993	Rice (Milled Equivalent)	155
107	Côte d'Ivoire	1993	Roots, Other	8
107	Côte d'Ivoire	1993	Sorghum	3

FAO country code	Country	Reference year	Food item	Gross food supply (g *capita*$^{-1}$ d^{-1})
107	Côte d'Ivoire	1993	Soyabeans	1
107	Côte d'Ivoire	1993	Sugar Cane	2
107	Côte d'Ivoire	1993	Sugar, Refined Equivalent	26
107	Côte d'Ivoire	1993	Sweet Potatoes	6
107	Côte d'Ivoire	1993	Tomatoes	9
107	Côte d'Ivoire	1993	Vegetables, Other	119
107	Côte d'Ivoire	1993	Wheat	44
107	Côte d'Ivoire	1993	Wine	3
107	Côte d'Ivoire	1993	Yams	465
107	Côte d'Ivoire	1994	Bananas	4
107	Côte d'Ivoire	1994	Beer	26
107	Côte d'Ivoire	1994	Beverages, Fermented	56
107	Côte d'Ivoire	1994	Bovine Meat	8
107	Côte d'Ivoire	1994	Cassava	279
107	Côte d'Ivoire	1994	Cereals, Other	4
107	Côte d'Ivoire	1994	Citrus, Other	2
107	Côte d'Ivoire	1994	Cocoa Beans	3
107	Côte d'Ivoire	1994	Coconut Oil	1
107	Côte d'Ivoire	1994	Coconuts - Incl Copra	1
107	Côte d'Ivoire	1994	Coffee	1
107	Côte d'Ivoire	1994	Cottonseed Oil	3
107	Côte d'Ivoire	1994	Demersal Fish	2
107	Côte d'Ivoire	1994	Eggs	3
107	Côte d'Ivoire	1994	Freshwater Fish	3
107	Côte d'Ivoire	1994	Fruits, Other	11
107	Côte d'Ivoire	1994	Grapefruit	3
107	Côte d'Ivoire	1994	Groundnuts (Shelled Eq)	7
107	Côte d'Ivoire	1994	Maize	72
107	Côte d'Ivoire	1994	Marine Fish, Other	9
107	Côte d'Ivoire	1994	Meat, Other	29
107	Côte d'Ivoire	1994	Milk, Whole	12
107	Côte d'Ivoire	1994	Millet	5
107	Côte d'Ivoire	1994	Mutton & Goat Meat	2
107	Côte d'Ivoire	1994	Nuts	6
107	Côte d'Ivoire	1994	Offals, Edible	3
107	Côte d'Ivoire	1994	Oranges, Mandarines	4
107	Côte d'Ivoire	1994	Palm Oil	25
107	Côte d'Ivoire	1994	Pelagic Fish	20
107	Côte d'Ivoire	1994	Pigmeat	2
107	Côte d'Ivoire	1994	Pimento	3
107	Côte d'Ivoire	1994	Pineapples	13
107	Côte d'Ivoire	1994	Plantains	233
107	Côte d'Ivoire	1994	Potatoes	1
107	Côte d'Ivoire	1994	Poultry Meat	5
107	Côte d'Ivoire	1994	Pulses, Other	1
107	Côte d'Ivoire	1994	Rice (Milled Equivalent)	151
107	Côte d'Ivoire	1994	Roots, Other	8
107	Côte d'Ivoire	1994	Sorghum	4
107	Côte d'Ivoire	1994	Soyabeans	1
107	Côte d'Ivoire	1994	Sugar Cane	2
107	Côte d'Ivoire	1994	Sugar, Refined Equivalent	26
107	Côte d'Ivoire	1994	Sweet Potatoes	6
107	Côte d'Ivoire	1994	Tomatoes	7
107	Côte d'Ivoire	1994	Vegetables, Other	126
107	Côte d'Ivoire	1994	Wheat	43
107	Côte d'Ivoire	1994	Wine	3
107	Côte d'Ivoire	1994	Yams	462
107	Côte d'Ivoire	1995	Bananas	3
107	Côte d'Ivoire	1995	Beer	40
107	Côte d'Ivoire	1995	Beverages, Fermented	58
107	Côte d'Ivoire	1995	Bovine Meat	8
107	Côte d'Ivoire	1995	Cassava	279
107	Côte d'Ivoire	1995	Cereals, Other	2
107	Côte d'Ivoire	1995	Citrus, Other	2
107	Côte d'Ivoire	1995	Cocoa Beans	4
107	Côte d'Ivoire	1995	Coconut Oil	1
107	Côte d'Ivoire	1995	Coconuts - Incl Copra	3
107	Côte d'Ivoire	1995	Coffee	1
107	Côte d'Ivoire	1995	Cottonseed Oil	2
107	Côte d'Ivoire	1995	Demersal Fish	1
107	Côte d'Ivoire	1995	Eggs	3
107	Côte d'Ivoire	1995	Freshwater Fish	2
107	Côte d'Ivoire	1995	Fruits, Other	10
107	Côte d'Ivoire	1995	Grapefruit	3
107	Côte d'Ivoire	1995	Groundnuts (Shelled Eq)	7
107	Côte d'Ivoire	1995	Maize	76
107	Côte d'Ivoire	1995	Marine Fish, Other	19
107	Côte d'Ivoire	1995	Meat, Other	28
107	Côte d'Ivoire	1995	Milk, Whole	24
107	Côte d'Ivoire	1995	Millet	4
107	Côte d'Ivoire	1995	Mutton & Goat Meat	2
107	Côte d'Ivoire	1995	Nuts	6
107	Côte d'Ivoire	1995	Offals, Edible	3
107	Côte d'Ivoire	1995	Oilcrops Oil, Other	1
107	Côte d'Ivoire	1995	Oranges, Mandarines	4
107	Côte d'Ivoire	1995	Palm Oil	25
107	Côte d'Ivoire	1995	Pelagic Fish	14
107	Côte d'Ivoire	1995	Pigmeat	3
107	Côte d'Ivoire	1995	Pimento	4
107	Côte d'Ivoire	1995	Pineapples	13
107	Côte d'Ivoire	1995	Plantains	224
107	Côte d'Ivoire	1995	Potatoes	1
107	Côte d'Ivoire	1995	Poultry Meat	5
107	Côte d'Ivoire	1995	Pulses, Other	1
107	Côte d'Ivoire	1995	Rape and Mustard Oil	1
107	Côte d'Ivoire	1995	Rice (Milled Equivalent)	155
107	Côte d'Ivoire	1995	Roots, Other	7

FAO country code	Country	Reference year	Food item	Gross food supply (g *capita*$^{-1}$ d^{-1})
107	Côte d'Ivoire	1995	Sorghum	4
107	Côte d'Ivoire	1995	Sugar Cane	2
107	Côte d'Ivoire	1995	Sugar, Refined Equivalent	25
107	Côte d'Ivoire	1995	Sweet Potatoes	6
107	Côte d'Ivoire	1995	Tomatoes	8
107	Côte d'Ivoire	1995	Vegetables, Other	118
107	Côte d'Ivoire	1995	Wheat	43
107	Côte d'Ivoire	1995	Wine	4
107	Côte d'Ivoire	1995	Yams	460
107	Côte d'Ivoire	1996	Bananas	3
107	Côte d'Ivoire	1996	Beer	42
107	Côte d'Ivoire	1996	Beverages, Fermented	56
107	Côte d'Ivoire	1996	Bovine Meat	7
107	Côte d'Ivoire	1996	Cassava	278
107	Côte d'Ivoire	1996	Cereals, Other	2
107	Côte d'Ivoire	1996	Citrus, Other	2
107	Côte d'Ivoire	1996	Cocoa Beans	5
107	Côte d'Ivoire	1996	Coconut Oil	1
107	Côte d'Ivoire	1996	Coconuts - Incl Copra	2
107	Côte d'Ivoire	1996	Coffee	1
107	Côte d'Ivoire	1996	Cottonseed Oil	2
107	Côte d'Ivoire	1996	Demersal Fish	1
107	Côte d'Ivoire	1996	Eggs	3
107	Côte d'Ivoire	1996	Freshwater Fish	2
107	Côte d'Ivoire	1996	Fruits, Other	10
107	Côte d'Ivoire	1996	Grapefruit	3
107	Côte d'Ivoire	1996	Groundnuts (Shelled Eq)	6
107	Côte d'Ivoire	1996	Maize	75
107	Côte d'Ivoire	1996	Marine Fish, Other	16
107	Côte d'Ivoire	1996	Meat, Other	28
107	Côte d'Ivoire	1996	Milk, Whole	23
107	Côte d'Ivoire	1996	Millet	4
107	Côte d'Ivoire	1996	Mutton & Goat Meat	2
107	Côte d'Ivoire	1996	Nuts	4
107	Côte d'Ivoire	1996	Offals, Edible	3
107	Côte d'Ivoire	1996	Oranges, Mandarines	4
107	Côte d'Ivoire	1996	Palm Oil	29
107	Côte d'Ivoire	1996	Pelagic Fish	18
107	Côte d'Ivoire	1996	Pigmeat	1
107	Côte d'Ivoire	1996	Pimento	4
107	Côte d'Ivoire	1996	Pineapples	12
107	Côte d'Ivoire	1996	Plantains	217
107	Côte d'Ivoire	1996	Potatoes	2
107	Côte d'Ivoire	1996	Poultry Meat	4
107	Côte d'Ivoire	1996	Pulses, Other	1
107	Côte d'Ivoire	1996	Rice (Milled Equivalent)	163
107	Côte d'Ivoire	1996	Roots, Other	7
107	Côte d'Ivoire	1996	Sorghum	4
107	Côte d'Ivoire	1996	Sugar Cane	2
107	Côte d'Ivoire	1996	Sugar, Refined Equivalent	22
107	Côte d'Ivoire	1996	Sweet Potatoes	6
107	Côte d'Ivoire	1996	Sweeteners, Other	1
107	Côte d'Ivoire	1996	Tomatoes	8
107	Côte d'Ivoire	1996	Vegetables, Other	141
107	Côte d'Ivoire	1996	Wheat	41
107	Côte d'Ivoire	1996	Wine	4
107	Côte d'Ivoire	1996	Yams	457
107	Côte d'Ivoire	1997	Apples	1
107	Côte d'Ivoire	1997	Bananas	3
107	Côte d'Ivoire	1997	Beans	3
107	Côte d'Ivoire	1997	Beer	34
107	Côte d'Ivoire	1997	Beverages, Fermented	61
107	Côte d'Ivoire	1997	Bovine Meat	8
107	Côte d'Ivoire	1997	Cassava	322
107	Côte d'Ivoire	1997	Cereals, Other	2
107	Côte d'Ivoire	1997	Citrus, Other	2
107	Côte d'Ivoire	1997	Cocoa Beans	5
107	Côte d'Ivoire	1997	Coconut Oil	1
107	Côte d'Ivoire	1997	Coconuts - Incl Copra	8
107	Côte d'Ivoire	1997	Coffee	2
107	Côte d'Ivoire	1997	Cottonseed Oil	3
107	Côte d'Ivoire	1997	Demersal Fish	2
107	Côte d'Ivoire	1997	Eggs	4
107	Côte d'Ivoire	1997	Freshwater Fish	2
107	Côte d'Ivoire	1997	Fruits, Other	11
107	Côte d'Ivoire	1997	Groundnuts (Shelled Eq)	7
107	Côte d'Ivoire	1997	Maize	72
107	Côte d'Ivoire	1997	Marine Fish, Other	19
107	Côte d'Ivoire	1997	Meat, Other	29
107	Côte d'Ivoire	1997	Milk, Whole	22
107	Côte d'Ivoire	1997	Millet	5
107	Côte d'Ivoire	1997	Mutton & Goat Meat	2
107	Côte d'Ivoire	1997	Nuts	10
107	Côte d'Ivoire	1997	Offals, Edible	3
107	Côte d'Ivoire	1997	Oilcrops Oil, Other	1
107	Côte d'Ivoire	1997	Oranges, Mandarines	4
107	Côte d'Ivoire	1997	Palm Oil	30
107	Côte d'Ivoire	1997	Pelagic Fish	13
107	Côte d'Ivoire	1997	Pigmeat	1
107	Côte d'Ivoire	1997	Pimento	4
107	Côte d'Ivoire	1997	Pineapples	12
107	Côte d'Ivoire	1997	Plantains	242
107	Côte d'Ivoire	1997	Potatoes	3
107	Côte d'Ivoire	1997	Poultry Meat	5
107	Côte d'Ivoire	1997	Pulses, Other	1
107	Côte d'Ivoire	1997	Rape and Mustard Oil	1
107	Côte d'Ivoire	1997	Rice (Milled Equivalent)	140

FAO country code	Country	Reference year	Food item	Gross food supply (g $capita^{-1}$ d^{-1})
107	Côte d'Ivoire	1997	Roots, Other	8
107	Côte d'Ivoire	1997	Sesameseed	1
107	Côte d'Ivoire	1997	Sorghum	4
107	Côte d'Ivoire	1997	Spices, Other	1
107	Côte d'Ivoire	1997	Sugar Cane	2
107	Côte d'Ivoire	1997	Sugar, Refined Equivalent	21
107	Côte d'Ivoire	1997	Sweet Potatoes	8
107	Côte d'Ivoire	1997	Sweeteners, Other	1
107	Côte d'Ivoire	1997	Tomatoes	8
107	Côte d'Ivoire	1997	Vegetables, Other	136
107	Côte d'Ivoire	1997	Wheat	38
107	Côte d'Ivoire	1997	Wine	4
107	Côte d'Ivoire	1997	Yams	493
107	Côte d'Ivoire	1998	Apples	1
107	Côte d'Ivoire	1998	Bananas	3
107	Côte d'Ivoire	1998	Beans	3
107	Côte d'Ivoire	1998	Beer	36
107	Côte d'Ivoire	1998	Beverages, Fermented	59
107	Côte d'Ivoire	1998	Bovine Meat	7
107	Côte d'Ivoire	1998	Cassava	311
107	Côte d'Ivoire	1998	Cereals, Other	2
107	Côte d'Ivoire	1998	Citrus, Other	3
107	Côte d'Ivoire	1998	Cocoa Beans	4
107	Côte d'Ivoire	1998	Coconut Oil	1
107	Côte d'Ivoire	1998	Coconuts - Incl Copra	8
107	Côte d'Ivoire	1998	Coffee	3
107	Côte d'Ivoire	1998	Cottonseed Oil	4
107	Côte d'Ivoire	1998	Demersal Fish	1
107	Côte d'Ivoire	1998	Eggs	6
107	Côte d'Ivoire	1998	Freshwater Fish	2
107	Côte d'Ivoire	1998	Fruits, Other	12
107	Côte d'Ivoire	1998	Grapefruit	3
107	Côte d'Ivoire	1998	Groundnuts (Shelled Eq)	6
107	Côte d'Ivoire	1998	Maize	72
107	Côte d'Ivoire	1998	Marine Fish, Other	21
107	Côte d'Ivoire	1998	Meat, Other	22
107	Côte d'Ivoire	1998	Milk, Whole	24
107	Côte d'Ivoire	1998	Millet	5
107	Côte d'Ivoire	1998	Mutton & Goat Meat	2
107	Côte d'Ivoire	1998	Nuts	9
107	Côte d'Ivoire	1998	Offals, Edible	3
107	Côte d'Ivoire	1998	Oilcrops Oil, Other	1
107	Côte d'Ivoire	1998	Oranges, Mandarines	4
107	Côte d'Ivoire	1998	Palm Oil	27
107	Côte d'Ivoire	1998	Pelagic Fish	16
107	Côte d'Ivoire	1998	Pigmeat	1
107	Côte d'Ivoire	1998	Pimento	3
107	Côte d'Ivoire	1998	Pineapples	10
107	Côte d'Ivoire	1998	Plantains	243
107	Côte d'Ivoire	1998	Potatoes	3
107	Côte d'Ivoire	1998	Poultry Meat	4
107	Côte d'Ivoire	1998	Pulses, Other	1
107	Côte d'Ivoire	1998	Rape and Mustard Oil	1
107	Côte d'Ivoire	1998	Rice (Milled Equivalent)	132
107	Côte d'Ivoire	1998	Roots, Other	9
107	Côte d'Ivoire	1998	Sesameseed	1
107	Côte d'Ivoire	1998	Sorghum	4
107	Côte d'Ivoire	1998	Spices, Other	1
107	Côte d'Ivoire	1998	Sugar Cane	2
107	Côte d'Ivoire	1998	Sugar, Refined Equivalent	22
107	Côte d'Ivoire	1998	Sweet Potatoes	8
107	Côte d'Ivoire	1998	Sweeteners, Other	1
107	Côte d'Ivoire	1998	Tomatoes	5
107	Côte d'Ivoire	1998	Vegetables, Other	133
107	Côte d'Ivoire	1998	Wheat	48
107	Côte d'Ivoire	1998	Wine	4
107	Côte d'Ivoire	1998	Yams	494
107	Côte d'Ivoire	1999	Apples	1
107	Côte d'Ivoire	1999	Bananas	2
107	Côte d'Ivoire	1999	Beans	3
107	Côte d'Ivoire	1999	Beer	35
107	Côte d'Ivoire	1999	Beverages, Fermented	63
107	Côte d'Ivoire	1999	Bovine Meat	7
107	Côte d'Ivoire	1999	Cassava	330
107	Côte d'Ivoire	1999	Cereals, Other	2
107	Côte d'Ivoire	1999	Citrus, Other	2
107	Côte d'Ivoire	1999	Cocoa Beans	4
107	Côte d'Ivoire	1999	Coconut Oil	2
107	Côte d'Ivoire	1999	Coconuts - Incl Copra	7
107	Côte d'Ivoire	1999	Coffee	4
107	Côte d'Ivoire	1999	Cottonseed Oil	4
107	Côte d'Ivoire	1999	Demersal Fish	1
107	Côte d'Ivoire	1999	Eggs	5
107	Côte d'Ivoire	1999	Freshwater Fish	2
107	Côte d'Ivoire	1999	Fruits, Other	10
107	Côte d'Ivoire	1999	Grapefruit	3
107	Côte d'Ivoire	1999	Groundnuts (Shelled Eq)	6
107	Côte d'Ivoire	1999	Maize	65
107	Côte d'Ivoire	1999	Marine Fish, Other	23
107	Côte d'Ivoire	1999	Meat, Other	22
107	Côte d'Ivoire	1999	Milk, Whole	19
107	Côte d'Ivoire	1999	Millet	5
107	Côte d'Ivoire	1999	Mutton & Goat Meat	2
107	Côte d'Ivoire	1999	Nuts	10
107	Côte d'Ivoire	1999	Offals, Edible	3
107	Côte d'Ivoire	1999	Oilcrops Oil, Other	1
107	Côte d'Ivoire	1999	Oranges, Mandarines	3

FAO country code	Country	Reference year	Food item	Gross food supply (g $capita^{-1}$ d^{-1})
107	Côte d'Ivoire	1999	Palm Oil	27
107	Côte d'Ivoire	1999	Pelagic Fish	23
107	Côte d'Ivoire	1999	Pigmeat	1
107	Côte d'Ivoire	1999	Pimento	3
107	Côte d'Ivoire	1999	Pineapples	8
107	Côte d'Ivoire	1999	Plantains	231
107	Côte d'Ivoire	1999	Potatoes	2
107	Côte d'Ivoire	1999	Poultry Meat	4
107	Côte d'Ivoire	1999	Pulses, Other	1
107	Côte d'Ivoire	1999	Rape and Mustard Oil	1
107	Côte d'Ivoire	1999	Rice (Milled Equivalent)	133
107	Côte d'Ivoire	1999	Roots, Other	8
107	Côte d'Ivoire	1999	Sesameseed	1
107	Côte d'Ivoire	1999	Sorghum	4
107	Côte d'Ivoire	1999	Spices, Other	1
107	Côte d'Ivoire	1999	Sugar Cane	2
107	Côte d'Ivoire	1999	Sugar, Refined Equivalent	22
107	Côte d'Ivoire	1999	Sweet Potatoes	7
107	Côte d'Ivoire	1999	Sweeteners, Other	1
107	Côte d'Ivoire	1999	Tomatoes	6
107	Côte d'Ivoire	1999	Vegetables, Other	130
107	Côte d'Ivoire	1999	Wheat	48
107	Côte d'Ivoire	1999	Wine	4
107	Côte d'Ivoire	1999	Yams	496
107	Côte d'Ivoire	2000	Apples	1
107	Côte d'Ivoire	2000	Bananas	2
107	Côte d'Ivoire	2000	Beans	3
107	Côte d'Ivoire	2000	Beer	32
107	Côte d'Ivoire	2000	Beverages, Fermented	62
107	Côte d'Ivoire	2000	Bovine Meat	7
107	Côte d'Ivoire	2000	Cassava	321
107	Côte d'Ivoire	2000	Cereals, Other	2
107	Côte d'Ivoire	2000	Citrus, Other	1
107	Côte d'Ivoire	2000	Cocoa Beans	3
107	Côte d'Ivoire	2000	Coconut Oil	2
107	Côte d'Ivoire	2000	Coconuts - Incl Copra	9
107	Côte d'Ivoire	2000	Coffee	4
107	Côte d'Ivoire	2000	Cottonseed Oil	4
107	Côte d'Ivoire	2000	Demersal Fish	2
107	Côte d'Ivoire	2000	Eggs	5
107	Côte d'Ivoire	2000	Freshwater Fish	2
107	Côte d'Ivoire	2000	Fruits, Other	10
107	Côte d'Ivoire	2000	Grapefruit	3
107	Côte d'Ivoire	2000	Groundnuts (Shelled Eq)	6
107	Côte d'Ivoire	2000	Maize	63
107	Côte d'Ivoire	2000	Marine Fish, Other	21
107	Côte d'Ivoire	2000	Meat, Other	22
107	Côte d'Ivoire	2000	Milk, Whole	15
107	Côte d'Ivoire	2000	Millet	5
107	Côte d'Ivoire	2000	Mutton & Goat Meat	2
107	Côte d'Ivoire	2000	Nuts	11
107	Côte d'Ivoire	2000	Offals, Edible	3
107	Côte d'Ivoire	2000	Oilcrops Oil, Other	2
107	Côte d'Ivoire	2000	Oranges, Mandarines	3
107	Côte d'Ivoire	2000	Palm Oil	27
107	Côte d'Ivoire	2000	Pelagic Fish	11
107	Côte d'Ivoire	2000	Pigmeat	1
107	Côte d'Ivoire	2000	Pimento	3
107	Côte d'Ivoire	2000	Pineapples	5
107	Côte d'Ivoire	2000	Plantains	220
107	Côte d'Ivoire	2000	Potatoes	3
107	Côte d'Ivoire	2000	Poultry Meat	4
107	Côte d'Ivoire	2000	Pulses, Other	1
107	Côte d'Ivoire	2000	Rice (Milled Equivalent)	139
107	Côte d'Ivoire	2000	Roots, Other	8
107	Côte d'Ivoire	2000	Sesameseed	1
107	Côte d'Ivoire	2000	Sorghum	4
107	Côte d'Ivoire	2000	Soyabeans	1
107	Côte d'Ivoire	2000	Spices, Other	1
107	Côte d'Ivoire	2000	Sugar Cane	2
107	Côte d'Ivoire	2000	Sugar, Refined Equivalent	19
107	Côte d'Ivoire	2000	Sweet Potatoes	7
107	Côte d'Ivoire	2000	Sweeteners, Other	1
107	Côte d'Ivoire	2000	Tomatoes	5
107	Côte d'Ivoire	2000	Vegetables, Other	132
107	Côte d'Ivoire	2000	Wheat	46
107	Côte d'Ivoire	2000	Whey	1
107	Côte d'Ivoire	2000	Wine	4
107	Côte d'Ivoire	2000	Yams	499
107	Côte d'Ivoire	2001	Apples	1
107	Côte d'Ivoire	2001	Bananas	3
107	Côte d'Ivoire	2001	Beans	3
107	Côte d'Ivoire	2001	Beer	42
107	Côte d'Ivoire	2001	Beverages, Fermented	69
107	Côte d'Ivoire	2001	Bovine Meat	6
107	Côte d'Ivoire	2001	Cassava	313
107	Côte d'Ivoire	2001	Cereals, Other	2
107	Côte d'Ivoire	2001	Citrus, Other	2
107	Côte d'Ivoire	2001	Cocoa Beans	4
107	Côte d'Ivoire	2001	Coconut Oil	3
107	Côte d'Ivoire	2001	Coconuts - Incl Copra	6
107	Côte d'Ivoire	2001	Coffee	5
107	Côte d'Ivoire	2001	Cottonseed Oil	3
107	Côte d'Ivoire	2001	Demersal Fish	1
107	Côte d'Ivoire	2001	Eggs	5
107	Côte d'Ivoire	2001	Freshwater Fish	2
107	Côte d'Ivoire	2001	Fruits, Other	11

FAO countrycode	Country	Reference year	Food item	Gross food supply (g *capita*$^{-1}$ d^{-1})
107	Côte d'Ivoire	2001	Grapefruit	3
107	Côte d'Ivoire	2001	Groundnuts (Shelled Eq)	6
107	Côte d'Ivoire	2001	Maize	64
107	Côte d'Ivoire	2001	Marine Fish, Other	20
107	Côte d'Ivoire	2001	Meat, Other	21
107	Côte d'Ivoire	2001	Milk, Whole	17
107	Côte d'Ivoire	2001	Millet	5
107	Côte d'Ivoire	2001	Mutton & Goat Meat	2
107	Côte d'Ivoire	2001	Nuts	14
107	Côte d'Ivoire	2001	Offals, Edible	5
107	Côte d'Ivoire	2001	Oilcrops Oil, Other	1
107	Côte d'Ivoire	2001	Onions	2
107	Côte d'Ivoire	2001	Oranges, Mandarines	3
107	Côte d'Ivoire	2001	Palm Oil	27
107	Côte d'Ivoire	2001	Palmkernel Oil	1
107	Côte d'Ivoire	2001	Pelagic Fish	17
107	Côte d'Ivoire	2001	Pigmeat	1
107	Côte d'Ivoire	2001	Pimento	3
107	Côte d'Ivoire	2001	Pineapples	6
107	Côte d'Ivoire	2001	Plantains	169
107	Côte d'Ivoire	2001	Potatoes	3
107	Côte d'Ivoire	2001	Poultry Meat	4
107	Côte d'Ivoire	2001	Pulses, Other	1
107	Côte d'Ivoire	2001	Rape and Mustard Oil	1
107	Côte d'Ivoire	2001	Rice (Milled Equivalent)	147
107	Côte d'Ivoire	2001	Roots, Other	8
107	Côte d'Ivoire	2001	Sesameseed	1
107	Côte d'Ivoire	2001	Sorghum	4
107	Côte d'Ivoire	2001	Spices, Other	1
107	Côte d'Ivoire	2001	Sugar Cane	2
107	Côte d'Ivoire	2001	Sugar, Refined Equivalent	20
107	Côte d'Ivoire	2001	Sweet Potatoes	8
107	Côte d'Ivoire	2001	Sweeteners, Other	1
107	Côte d'Ivoire	2001	Tomatoes	8
107	Côte d'Ivoire	2001	Vegetables, Other	109
107	Côte d'Ivoire	2001	Wheat	48
107	Côte d'Ivoire	2001	Whey	2
107	Côte d'Ivoire	2001	Wine	4
107	Côte d'Ivoire	2001	Yams	504
107	Côte d'Ivoire	2002	Apples	1
107	Côte d'Ivoire	2002	Bananas	2
107	Côte d'Ivoire	2002	Barley	1
107	Côte d'Ivoire	2002	Beans	3
107	Côte d'Ivoire	2002	Beer	49
107	Côte d'Ivoire	2002	Beverages, Fermented	70
107	Côte d'Ivoire	2002	Bovine Meat	5
107	Côte d'Ivoire	2002	Cassava	306
107	Côte d'Ivoire	2002	Cereals, Other	2
107	Côte d'Ivoire	2002	Citrus, Other	3
107	Côte d'Ivoire	2002	Cocoa Beans	5
107	Côte d'Ivoire	2002	Coconut Oil	3
107	Côte d'Ivoire	2002	Coconuts - Incl Copra	5
107	Côte d'Ivoire	2002	Coffee	6
107	Côte d'Ivoire	2002	Cottonseed Oil	3
107	Côte d'Ivoire	2002	Demersal Fish	1
107	Côte d'Ivoire	2002	Eggs	5
107	Côte d'Ivoire	2002	Freshwater Fish	4
107	Côte d'Ivoire	2002	Fruits, Other	12
107	Côte d'Ivoire	2002	Grapefruit	3
107	Côte d'Ivoire	2002	Groundnuts (Shelled Eq)	5
107	Côte d'Ivoire	2002	Maize	61
107	Côte d'Ivoire	2002	Marine Fish, Other	16
107	Côte d'Ivoire	2002	Meat, Other	22
107	Côte d'Ivoire	2002	Milk, Whole	16
107	Côte d'Ivoire	2002	Millet	5
107	Côte d'Ivoire	2002	Mutton & Goat Meat	2
107	Côte d'Ivoire	2002	Nuts	15
107	Côte d'Ivoire	2002	Offals, Edible	6
107	Côte d'Ivoire	2002	Oilcrops Oil, Other	1
107	Côte d'Ivoire	2002	Onions	3
107	Côte d'Ivoire	2002	Oranges, Mandarines	4
107	Côte d'Ivoire	2002	Palm Oil	27
107	Côte d'Ivoire	2002	Palmkernel Oil	1
107	Côte d'Ivoire	2002	Pelagic Fish	18
107	Côte d'Ivoire	2002	Pigmeat	1
107	Côte d'Ivoire	2002	Pimento	3
107	Côte d'Ivoire	2002	Pineapples	7
107	Côte d'Ivoire	2002	Plantains	202
107	Côte d'Ivoire	2002	Potatoes	3
107	Côte d'Ivoire	2002	Poultry Meat	5
107	Côte d'Ivoire	2002	Pulses, Other	1
107	Côte d'Ivoire	2002	Rape and Mustard Oil	1
107	Côte d'Ivoire	2002	Rice (Milled Equivalent)	143
107	Côte d'Ivoire	2002	Roots, Other	9
107	Côte d'Ivoire	2002	Sesameseed	1
107	Côte d'Ivoire	2002	Sorghum	4
107	Côte d'Ivoire	2002	Spices, Other	1
107	Côte d'Ivoire	2002	Sugar Cane	2
107	Côte d'Ivoire	2002	Sugar, Refined Equivalent	25
107	Côte d'Ivoire	2002	Sweet Potatoes	8
107	Côte d'Ivoire	2002	Sweeteners, Other	1
107	Côte d'Ivoire	2002	Tomatoes	8
107	Côte d'Ivoire	2002	Vegetables, Other	109
107	Côte d'Ivoire	2002	Wheat	48
107	Côte d'Ivoire	2002	Whey	2
107	Côte d'Ivoire	2002	Wine	5
107	Côte d'Ivoire	2002	Yams	510

FAO country code	Country	Reference year	Food item	Gross food supply (g $capita^{-1}$ d^{-1})
107	Côte d'Ivoire	2003	Apples	1
107	Côte d'Ivoire	2003	Bananas	2
107	Côte d'Ivoire	2003	Beans	4
107	Côte d'Ivoire	2003	Beer	45
107	Côte d'Ivoire	2003	Beverages, Fermented	70
107	Côte d'Ivoire	2003	Bovine Meat	6
107	Côte d'Ivoire	2003	Cassava	300
107	Côte d'Ivoire	2003	Cereals, Other	2
107	Côte d'Ivoire	2003	Citrus, Other	3
107	Côte d'Ivoire	2003	Cocoa Beans	5
107	Côte d'Ivoire	2003	Coconut Oil	3
107	Côte d'Ivoire	2003	Coconuts - Incl Copra	10
107	Côte d'Ivoire	2003	Coffee	6
107	Côte d'Ivoire	2003	Cottonseed Oil	3
107	Côte d'Ivoire	2003	Demersal Fish	1
107	Côte d'Ivoire	2003	Eggs	5
107	Côte d'Ivoire	2003	Freshwater Fish	4
107	Côte d'Ivoire	2003	Fruits, Other	12
107	Côte d'Ivoire	2003	Grapefruit	3
107	Côte d'Ivoire	2003	Groundnuts (Shelled Eq)	5
107	Côte d'Ivoire	2003	Maize	59
107	Côte d'Ivoire	2003	Marine Fish, Other	18
107	Côte d'Ivoire	2003	Meat, Other	22
107	Côte d'Ivoire	2003	Milk, Whole	20
107	Côte d'Ivoire	2003	Millet	4
107	Côte d'Ivoire	2003	Mutton & Goat Meat	2
107	Côte d'Ivoire	2003	Nuts	12
107	Côte d'Ivoire	2003	Offals, Edible	9
107	Côte d'Ivoire	2003	Oilcrops Oil, Other	1
107	Côte d'Ivoire	2003	Onions	3
107	Côte d'Ivoire	2003	Oranges, Mandarines	4
107	Côte d'Ivoire	2003	Palm Oil	28
107	Côte d'Ivoire	2003	Palmkernel Oil	2
107	Côte d'Ivoire	2003	Pelagic Fish	20
107	Côte d'Ivoire	2003	Pigmeat	2
107	Côte d'Ivoire	2003	Pimento	3
107	Côte d'Ivoire	2003	Pineapples	7
107	Côte d'Ivoire	2003	Plantains	194
107	Côte d'Ivoire	2003	Potatoes	3
107	Côte d'Ivoire	2003	Poultry Meat	6
107	Côte d'Ivoire	2003	Pulses, Other	2
107	Côte d'Ivoire	2003	Rice (Milled Equivalent)	153
107	Côte d'Ivoire	2003	Roots, Other	7
107	Côte d'Ivoire	2003	Sesameseed	1
107	Côte d'Ivoire	2003	Sorghum	4
107	Côte d'Ivoire	2003	Spices, Other	1
107	Côte d'Ivoire	2003	Sugar Cane	2
107	Côte d'Ivoire	2003	Sugar, Refined Equivalent	26
107	Côte d'Ivoire	2003	Sweet Potatoes	6
107	Côte d'Ivoire	2003	Sweeteners, Other	1
107	Côte d'Ivoire	2003	Tomatoes	8
107	Côte d'Ivoire	2003	Vegetables, Other	81
107	Côte d'Ivoire	2003	Wheat	49
107	Côte d'Ivoire	2003	Whey	4
107	Côte d'Ivoire	2003	Wine	5
107	Côte d'Ivoire	2003	Yams	517
107	Côte d'Ivoire	2004	Apples	1
107	Côte d'Ivoire	2004	Bananas	2
107	Côte d'Ivoire	2004	Barley	1
107	Côte d'Ivoire	2004	Beans	4
107	Côte d'Ivoire	2004	Beer	51
107	Côte d'Ivoire	2004	Beverages, Fermented	71
107	Côte d'Ivoire	2004	Bovine Meat	6
107	Côte d'Ivoire	2004	Cassava	298
107	Côte d'Ivoire	2004	Cereals, Other	2
107	Côte d'Ivoire	2004	Citrus, Other	3
107	Côte d'Ivoire	2004	Cocoa Beans	6
107	Côte d'Ivoire	2004	Coconut Oil	2
107	Côte d'Ivoire	2004	Coconuts - Incl Copra	9
107	Côte d'Ivoire	2004	Coffee	6
107	Côte d'Ivoire	2004	Cottonseed Oil	1
107	Côte d'Ivoire	2004	Demersal Fish	1
107	Côte d'Ivoire	2004	Eggs	5
107	Côte d'Ivoire	2004	Freshwater Fish	1
107	Côte d'Ivoire	2004	Fruits, Other	13
107	Côte d'Ivoire	2004	Grapefruit	3
107	Côte d'Ivoire	2004	Groundnuts (Shelled Eq)	5
107	Côte d'Ivoire	2004	Maize	59
107	Côte d'Ivoire	2004	Marine Fish, Other	19
107	Côte d'Ivoire	2004	Meat, Other	23
107	Côte d'Ivoire	2004	Milk, Whole	22
107	Côte d'Ivoire	2004	Millet	4
107	Côte d'Ivoire	2004	Mutton & Goat Meat	2
107	Côte d'Ivoire	2004	Nuts	13
107	Côte d'Ivoire	2004	Offals, Edible	8
107	Côte d'Ivoire	2004	Oilcrops Oil, Other	1
107	Côte d'Ivoire	2004	Onions	4
107	Côte d'Ivoire	2004	Oranges, Mandarines	4
107	Côte d'Ivoire	2004	Palm Oil	28
107	Côte d'Ivoire	2004	Palmkernel Oil	2
107	Côte d'Ivoire	2004	Pelagic Fish	16
107	Côte d'Ivoire	2004	Pigmeat	2
107	Côte d'Ivoire	2004	Pimento	3
107	Côte d'Ivoire	2004	Pineapples	7
107	Côte d'Ivoire	2004	Plantains	194
107	Côte d'Ivoire	2004	Potatoes	3
107	Côte d'Ivoire	2004	Poultry Meat	6

FAO country code	Country	Reference year	Food item	Gross food supply (g *capita*$^{-1}$ d^{-1})
107	Côte d'Ivoire	2004	Pulses, Other	1
107	Côte d'Ivoire	2004	Rape and Mustard Oil	1
107	Côte d'Ivoire	2004	Rice (Milled Equivalent)	173
107	Côte d'Ivoire	2004	Roots, Other	7
107	Côte d'Ivoire	2004	Sorghum	4
107	Côte d'Ivoire	2004	Spices, Other	1
107	Côte d'Ivoire	2004	Sugar Cane	2
107	Côte d'Ivoire	2004	Sugar, Refined Equivalent	24
107	Côte d'Ivoire	2004	Sweet Potatoes	6
107	Côte d'Ivoire	2004	Sweeteners, Other	1
107	Côte d'Ivoire	2004	Tomatoes	7
107	Côte d'Ivoire	2004	Vegetables, Other	88
107	Côte d'Ivoire	2004	Wheat	45
107	Côte d'Ivoire	2004	Whey	2
107	Côte d'Ivoire	2004	Wine	3
107	Côte d'Ivoire	2004	Yams	527
107	Côte d'Ivoire	2005	Apples	1
107	Côte d'Ivoire	2005	Bananas	2
107	Côte d'Ivoire	2005	Beans	4
107	Côte d'Ivoire	2005	Beer	49
107	Côte d'Ivoire	2005	Beverages, Fermented	73
107	Côte d'Ivoire	2005	Bovine Meat	6
107	Côte d'Ivoire	2005	Cassava	311
107	Côte d'Ivoire	2005	Cereals, Other	2
107	Côte d'Ivoire	2005	Citrus, Other	3
107	Côte d'Ivoire	2005	Cocoa Beans	6
107	Côte d'Ivoire	2005	Coconut Oil	2
107	Côte d'Ivoire	2005	Coconuts - Incl Copra	8
107	Côte d'Ivoire	2005	Coffee	5
107	Côte d'Ivoire	2005	Cottonseed Oil	3
107	Côte d'Ivoire	2005	Demersal Fish	1
107	Côte d'Ivoire	2005	Eggs	4
107	Côte d'Ivoire	2005	Freshwater Fish	2
107	Côte d'Ivoire	2005	Fruits, Other	13
107	Côte d'Ivoire	2005	Grapefruit	3
107	Côte d'Ivoire	2005	Groundnuts (Shelled Eq)	5
107	Côte d'Ivoire	2005	Maize	64
107	Côte d'Ivoire	2005	Marine Fish, Other	20
107	Côte d'Ivoire	2005	Meat, Other	23
107	Côte d'Ivoire	2005	Milk, Whole	23
107	Côte d'Ivoire	2005	Millet	4
107	Côte d'Ivoire	2005	Mutton & Goat Meat	2
107	Côte d'Ivoire	2005	Nuts	15
107	Côte d'Ivoire	2005	Offals, Edible	8
107	Côte d'Ivoire	2005	Oilcrops Oil, Other	1
107	Côte d'Ivoire	2005	Onions	3
107	Côte d'Ivoire	2005	Oranges, Mandarines	5
107	Côte d'Ivoire	2005	Palm Oil	28
107	Côte d'Ivoire	2005	Palmkernel Oil	1
107	Côte d'Ivoire	2005	Pelagic Fish	19
107	Côte d'Ivoire	2005	Pigmeat	2
107	Côte d'Ivoire	2005	Pimento	3
107	Côte d'Ivoire	2005	Pineapples	7
107	Côte d'Ivoire	2005	Plantains	198
107	Côte d'Ivoire	2005	Potatoes	3
107	Côte d'Ivoire	2005	Poultry Meat	5
107	Côte d'Ivoire	2005	Pulses, Other	2
107	Côte d'Ivoire	2005	Rape and Mustard Oil	1
107	Côte d'Ivoire	2005	Rice (Milled Equivalent)	166
107	Côte d'Ivoire	2005	Roots, Other	9
107	Côte d'Ivoire	2005	Sesameseed	1
107	Côte d'Ivoire	2005	Sorghum	4
107	Côte d'Ivoire	2005	Spices, Other	1
107	Côte d'Ivoire	2005	Sugar Cane	2
107	Côte d'Ivoire	2005	Sugar, Refined Equivalent	24
107	Côte d'Ivoire	2005	Sweet Potatoes	6
107	Côte d'Ivoire	2005	Sweeteners, Other	1
107	Côte d'Ivoire	2005	Tomatoes	11
107	Côte d'Ivoire	2005	Vegetables, Other	98
107	Côte d'Ivoire	2005	Wheat	46
107	Côte d'Ivoire	2005	Whey	2
107	Côte d'Ivoire	2005	Wine	2
107	Côte d'Ivoire	2005	Yams	536
107	Côte d'Ivoire	2006	Apples	1
107	Côte d'Ivoire	2006	Bananas	3
107	Côte d'Ivoire	2006	Beans	4
107	Côte d'Ivoire	2006	Beer	45
107	Côte d'Ivoire	2006	Beverages, Fermented	70
107	Côte d'Ivoire	2006	Bovine Meat	7
107	Côte d'Ivoire	2006	Cassava	316
107	Côte d'Ivoire	2006	Cereals, Other	2
107	Côte d'Ivoire	2006	Citrus, Other	3
107	Côte d'Ivoire	2006	Cocoa Beans	5
107	Côte d'Ivoire	2006	Coconut Oil	1
107	Côte d'Ivoire	2006	Coconuts - Incl Copra	3
107	Côte d'Ivoire	2006	Coffee	5
107	Côte d'Ivoire	2006	Cottonseed Oil	3
107	Côte d'Ivoire	2006	Demersal Fish	1
107	Côte d'Ivoire	2006	Eggs	4
107	Côte d'Ivoire	2006	Freshwater Fish	1
107	Côte d'Ivoire	2006	Fruits, Other	14
107	Côte d'Ivoire	2006	Grapefruit	3
107	Côte d'Ivoire	2006	Groundnuts (Shelled Eq)	6
107	Côte d'Ivoire	2006	Maize	61
107	Côte d'Ivoire	2006	Marine Fish, Other	20
107	Côte d'Ivoire	2006	Meat, Other	24
107	Côte d'Ivoire	2006	Milk, Whole	16

FAO country code	Country	Reference year	Food item	Gross food supply (g $capita^{-1}$ d^{-1})
107	Côte d'Ivoire	2006	Millet	4
107	Côte d'Ivoire	2006	Mutton & Goat Meat	2
107	Côte d'Ivoire	2006	Nuts	15
107	Côte d'Ivoire	2006	Offals, Edible	9
107	Côte d'Ivoire	2006	Oilcrops Oil, Other	1
107	Côte d'Ivoire	2006	Oranges, Mandarines	4
107	Côte d'Ivoire	2006	Palm Oil	28
107	Côte d'Ivoire	2006	Palmkernel Oil	2
107	Côte d'Ivoire	2006	Pelagic Fish	19
107	Côte d'Ivoire	2006	Pigmeat	2
107	Côte d'Ivoire	2006	Pimento	3
107	Côte d'Ivoire	2006	Pineapples	7
107	Côte d'Ivoire	2006	Plantains	191
107	Côte d'Ivoire	2006	Potatoes	2
107	Côte d'Ivoire	2006	Poultry Meat	4
107	Côte d'Ivoire	2006	Pulses, Other	2
107	Côte d'Ivoire	2006	Rice (Milled Equivalent)	170
107	Côte d'Ivoire	2006	Roots, Other	9
107	Côte d'Ivoire	2006	Sorghum	3
107	Côte d'Ivoire	2006	Spices, Other	1
107	Côte d'Ivoire	2006	Sugar Cane	2
107	Côte d'Ivoire	2006	Sugar, Refined Equivalent	23
107	Côte d'Ivoire	2006	Sweet Potatoes	6
107	Côte d'Ivoire	2006	Sweeteners, Other	1
107	Côte d'Ivoire	2006	Tomatoes	11
107	Côte d'Ivoire	2006	Vegetables, Other	97
107	Côte d'Ivoire	2006	Wheat	48
107	Côte d'Ivoire	2006	Whey	3
107	Côte d'Ivoire	2006	Wine	2
107	Côte d'Ivoire	2006	Yams	570
107	Côte d'Ivoire	2007	Apples	1
107	Côte d'Ivoire	2007	Bananas	3
107	Côte d'Ivoire	2007	Beans	4
107	Côte d'Ivoire	2007	Beer	42
107	Côte d'Ivoire	2007	Beverages, Fermented	69
107	Côte d'Ivoire	2007	Bovine Meat	6
107	Côte d'Ivoire	2007	Cassava	322
107	Côte d'Ivoire	2007	Cereals, Other	2
107	Côte d'Ivoire	2007	Citrus, Other	3
107	Côte d'Ivoire	2007	Cocoa Beans	9
107	Côte d'Ivoire	2007	Coconuts - Incl Copra	4
107	Côte d'Ivoire	2007	Coffee	5
107	Côte d'Ivoire	2007	Cottonseed Oil	5
107	Côte d'Ivoire	2007	Demersal Fish	1
107	Côte d'Ivoire	2007	Eggs	3
107	Côte d'Ivoire	2007	Freshwater Fish	1
107	Côte d'Ivoire	2007	Fruits, Other	15
107	Côte d'Ivoire	2007	Grapefruit	3
107	Côte d'Ivoire	2007	Groundnuts (Shelled Eq)	5
107	Côte d'Ivoire	2007	Maize	49
107	Côte d'Ivoire	2007	Marine Fish, Other	20
107	Côte d'Ivoire	2007	Meat, Other	24
107	Côte d'Ivoire	2007	Milk, Whole	14
107	Côte d'Ivoire	2007	Millet	3
107	Côte d'Ivoire	2007	Mutton & Goat Meat	2
107	Côte d'Ivoire	2007	Nuts	15
107	Côte d'Ivoire	2007	Offals, Edible	9
107	Côte d'Ivoire	2007	Oilcrops Oil, Other	1
107	Côte d'Ivoire	2007	Oranges, Mandarines	5
107	Côte d'Ivoire	2007	Palm Oil	28
107	Côte d'Ivoire	2007	Palmkernel Oil	2
107	Côte d'Ivoire	2007	Pelagic Fish	19
107	Côte d'Ivoire	2007	Pigmeat	2
107	Côte d'Ivoire	2007	Pimento	3
107	Côte d'Ivoire	2007	Pineapples	7
107	Côte d'Ivoire	2007	Plantains	184
107	Côte d'Ivoire	2007	Potatoes	2
107	Côte d'Ivoire	2007	Poultry Meat	4
107	Côte d'Ivoire	2007	Pulses, Other	2
107	Côte d'Ivoire	2007	Rice (Milled Equivalent)	187
107	Côte d'Ivoire	2007	Roots, Other	9
107	Côte d'Ivoire	2007	Sorghum	3
107	Côte d'Ivoire	2007	Spices, Other	1
107	Côte d'Ivoire	2007	Sugar Cane	2
107	Côte d'Ivoire	2007	Sugar, Refined Equivalent	25
107	Côte d'Ivoire	2007	Sweet Potatoes	6
107	Côte d'Ivoire	2007	Sweeteners, Other	1
107	Côte d'Ivoire	2007	Tomatoes	10
107	Côte d'Ivoire	2007	Vegetables, Other	96
107	Côte d'Ivoire	2007	Wheat	48
107	Côte d'Ivoire	2007	Whey	2
107	Côte d'Ivoire	2007	Wine	3
107	Côte d'Ivoire	2007	Yams	554
107	Côte d'Ivoire	2008	Apples	1
107	Côte d'Ivoire	2008	Bananas	1
107	Côte d'Ivoire	2008	Beans	5
107	Côte d'Ivoire	2008	Beer	47
107	Côte d'Ivoire	2008	Beverages, Fermented	69
107	Côte d'Ivoire	2008	Bovine Meat	6
107	Côte d'Ivoire	2008	Cassava	306
107	Côte d'Ivoire	2008	Cereals, Other	2
107	Côte d'Ivoire	2008	Citrus, Other	2
107	Côte d'Ivoire	2008	Cocoa Beans	9
107	Côte d'Ivoire	2008	Coconut Oil	1
107	Côte d'Ivoire	2008	Coconuts - Incl Copra	1
107	Côte d'Ivoire	2008	Coffee	5
107	Côte d'Ivoire	2008	Cottonseed Oil	1

FAO country code	Country	Reference year	Food item	Gross food supply (g *capita*$^{-1}$ d^{-1})
107	Côte d'Ivoire	2008	Demersal Fish	1
107	Côte d'Ivoire	2008	Eggs	4
107	Côte d'Ivoire	2008	Freshwater Fish	1
107	Côte d'Ivoire	2008	Fruits, Other	15
107	Côte d'Ivoire	2008	Grapefruit	3
107	Côte d'Ivoire	2008	Groundnuts (Shelled Eq)	6
107	Côte d'Ivoire	2008	Maize	62
107	Côte d'Ivoire	2008	Marine Fish, Other	23
107	Côte d'Ivoire	2008	Meat, Other	24
107	Côte d'Ivoire	2008	Milk, Whole	20
107	Côte d'Ivoire	2008	Millet	4
107	Côte d'Ivoire	2008	Mutton & Goat Meat	2
107	Côte d'Ivoire	2008	Nuts	14
107	Côte d'Ivoire	2008	Offals, Edible	8
107	Côte d'Ivoire	2008	Oilcrops Oil, Other	1
107	Côte d'Ivoire	2008	Oranges, Mandarines	5
107	Côte d'Ivoire	2008	Palm Oil	26
107	Côte d'Ivoire	2008	Palmkernel Oil	1
107	Côte d'Ivoire	2008	Pelagic Fish	23
107	Côte d'Ivoire	2008	Pigmeat	2
107	Côte d'Ivoire	2008	Pimento	4
107	Côte d'Ivoire	2008	Pineapples	2
107	Côte d'Ivoire	2008	Plantains	201
107	Côte d'Ivoire	2008	Potatoes	2
107	Côte d'Ivoire	2008	Poultry Meat	4
107	Côte d'Ivoire	2008	Pulses, Other	2
107	Côte d'Ivoire	2008	Rice (Milled Equivalent)	181
107	Côte d'Ivoire	2008	Roots, Other	10
107	Côte d'Ivoire	2008	Sorghum	4
107	Côte d'Ivoire	2008	Spices, Other	1
107	Côte d'Ivoire	2008	Sugar Cane	4
107	Côte d'Ivoire	2008	Sugar, Refined Equivalent	24
107	Côte d'Ivoire	2008	Sweet Potatoes	7
107	Côte d'Ivoire	2008	Sweeteners, Other	1
107	Côte d'Ivoire	2008	Tomatoes	15
107	Côte d'Ivoire	2008	Vegetables, Other	91
107	Côte d'Ivoire	2008	Wheat	40
107	Côte d'Ivoire	2008	Whey	2
107	Côte d'Ivoire	2008	Wine	4
107	Côte d'Ivoire	2008	Yams	544
107	Côte d'Ivoire	2009	Apples	1
107	Côte d'Ivoire	2009	Bananas	1
107	Côte d'Ivoire	2009	Beans	5
107	Côte d'Ivoire	2009	Beer	48
107	Côte d'Ivoire	2009	Beverages, Fermented	70
107	Côte d'Ivoire	2009	Bovine Meat	6
107	Côte d'Ivoire	2009	Cassava	301
107	Côte d'Ivoire	2009	Cereals, Other	2
107	Côte d'Ivoire	2009	Citrus, Other	3
107	Côte d'Ivoire	2009	Cocoa Beans	13
107	Côte d'Ivoire	2009	Coconut Oil	3
107	Côte d'Ivoire	2009	Coconuts - Incl Copra	3
107	Côte d'Ivoire	2009	Coffee	5
107	Côte d'Ivoire	2009	Cottonseed Oil	2
107	Côte d'Ivoire	2009	Demersal Fish	1
107	Côte d'Ivoire	2009	Eggs	4
107	Côte d'Ivoire	2009	Freshwater Fish	1
107	Côte d'Ivoire	2009	Fruits, Other	16
107	Côte d'Ivoire	2009	Grapefruit	3
107	Côte d'Ivoire	2009	Groundnuts (Shelled Eq)	7
107	Côte d'Ivoire	2009	Maize	60
107	Côte d'Ivoire	2009	Marine Fish, Other	25
107	Côte d'Ivoire	2009	Meat, Other	24
107	Côte d'Ivoire	2009	Milk, Whole	18
107	Côte d'Ivoire	2009	Millet	4
107	Côte d'Ivoire	2009	Mutton & Goat Meat	2
107	Côte d'Ivoire	2009	Nuts	12
107	Côte d'Ivoire	2009	Offals, Edible	9
107	Côte d'Ivoire	2009	Oilcrops Oil, Other	1
107	Côte d'Ivoire	2009	Oilcrops, Other	1
107	Côte d'Ivoire	2009	Oranges, Mandarines	4
107	Côte d'Ivoire	2009	Palm Oil	24
107	Côte d'Ivoire	2009	Palmkernel Oil	3
107	Côte d'Ivoire	2009	Peas	1
107	Côte d'Ivoire	2009	Pelagic Fish	26
107	Côte d'Ivoire	2009	Pigmeat	2
107	Côte d'Ivoire	2009	Pimento	3
107	Côte d'Ivoire	2009	Pineapples	2
107	Côte d'Ivoire	2009	Plantains	176
107	Côte d'Ivoire	2009	Potatoes	3
107	Côte d'Ivoire	2009	Poultry Meat	4
107	Côte d'Ivoire	2009	Pulses, Other	2
107	Côte d'Ivoire	2009	Rice (Milled Equivalent)	177
107	Côte d'Ivoire	2009	Roots, Other	8
107	Côte d'Ivoire	2009	Sorghum	4
107	Côte d'Ivoire	2009	Spices, Other	1
107	Côte d'Ivoire	2009	Sugar Cane	4
107	Côte d'Ivoire	2009	Sugar, Refined Equivalent	22
107	Côte d'Ivoire	2009	Sweet Potatoes	6
107	Côte d'Ivoire	2009	Sweeteners, Other	1
107	Côte d'Ivoire	2009	Tomatoes	11
107	Côte d'Ivoire	2009	Vegetables, Other	98
107	Côte d'Ivoire	2009	Wheat	50
107	Côte d'Ivoire	2009	Whey	2
107	Côte d'Ivoire	2009	Wine	4
107	Côte d'Ivoire	2009	Yams	526
107	Côte d'Ivoire	2010	Apples	1

FAO country code	Country	Reference year	Food item	Gross food supply (g *capita*$^{-1}$ d^{-1})
107	Côte d'Ivoire	2010	Bananas	3
107	Côte d'Ivoire	2010	Beans	5
107	Côte d'Ivoire	2010	Beer	51
107	Côte d'Ivoire	2010	Beverages, Fermented	69
107	Côte d'Ivoire	2010	Bovine Meat	6
107	Côte d'Ivoire	2010	Cassava	300
107	Côte d'Ivoire	2010	Cereals, Other	2
107	Côte d'Ivoire	2010	Citrus, Other	1
107	Côte d'Ivoire	2010	Cocoa Beans	13
107	Côte d'Ivoire	2010	Coconut Oil	3
107	Côte d'Ivoire	2010	Coconuts - Incl Copra	3
107	Côte d'Ivoire	2010	Coffee	6
107	Côte d'Ivoire	2010	Cottonseed Oil	1
107	Côte d'Ivoire	2010	Demersal Fish	1
107	Côte d'Ivoire	2010	Eggs	4
107	Côte d'Ivoire	2010	Freshwater Fish	1
107	Côte d'Ivoire	2010	Fruits, Other	18
107	Côte d'Ivoire	2010	Grapefruit	3
107	Côte d'Ivoire	2010	Groundnuts (Shelled Eq)	7
107	Côte d'Ivoire	2010	Maize	66
107	Côte d'Ivoire	2010	Marine Fish, Other	24
107	Côte d'Ivoire	2010	Meat, Other	24
107	Côte d'Ivoire	2010	Milk, Whole	23
107	Côte d'Ivoire	2010	Millet	4
107	Côte d'Ivoire	2010	Mutton & Goat Meat	2
107	Côte d'Ivoire	2010	Nuts	15
107	Côte d'Ivoire	2010	Offals, Edible	9
107	Côte d'Ivoire	2010	Oilcrops Oil, Other	1
107	Côte d'Ivoire	2010	Oranges, Mandarines	6
107	Côte d'Ivoire	2010	Palm Oil	25
107	Côte d'Ivoire	2010	Palmkernel Oil	2
107	Côte d'Ivoire	2010	Pelagic Fish	29
107	Côte d'Ivoire	2010	Pigmeat	3
107	Côte d'Ivoire	2010	Pimento	3
107	Côte d'Ivoire	2010	Pineapples	1
107	Côte d'Ivoire	2010	Plantains	178
107	Côte d'Ivoire	2010	Potatoes	2
107	Côte d'Ivoire	2010	Poultry Meat	5
107	Côte d'Ivoire	2010	Pulses, Other	2
107	Côte d'Ivoire	2010	Rice (Milled Equivalent)	167
107	Côte d'Ivoire	2010	Roots, Other	9
107	Côte d'Ivoire	2010	Sorghum	5
107	Côte d'Ivoire	2010	Spices, Other	1
107	Côte d'Ivoire	2010	Sugar Cane	4
107	Côte d'Ivoire	2010	Sugar, Refined Equivalent	21
107	Côte d'Ivoire	2010	Sweet Potatoes	6
107	Côte d'Ivoire	2010	Sweeteners, Other	1
107	Côte d'Ivoire	2010	Tomatoes	12
107	Côte d'Ivoire	2010	Vegetables, Other	92
107	Côte d'Ivoire	2010	Wheat	52
107	Côte d'Ivoire	2010	Whey	2
107	Côte d'Ivoire	2010	Wine	4
107	Côte d'Ivoire	2010	Yams	525
107	Côte d'Ivoire	2011	Apples	1
107	Côte d'Ivoire	2011	Bananas	3
107	Côte d'Ivoire	2011	Beans	5
107	Côte d'Ivoire	2011	Beer	59
107	Côte d'Ivoire	2011	Beverages, Fermented	66
107	Côte d'Ivoire	2011	Bovine Meat	5
107	Côte d'Ivoire	2011	Cassava	301
107	Côte d'Ivoire	2011	Cereals, Other	2
107	Côte d'Ivoire	2011	Cocoa Beans	17
107	Côte d'Ivoire	2011	Coconut Oil	4
107	Côte d'Ivoire	2011	Coconuts - Incl Copra	6
107	Côte d'Ivoire	2011	Coffee	6
107	Côte d'Ivoire	2011	Cottonseed Oil	3
107	Côte d'Ivoire	2011	Demersal Fish	1
107	Côte d'Ivoire	2011	Eggs	4
107	Côte d'Ivoire	2011	Freshwater Fish	1
107	Côte d'Ivoire	2011	Fruits, Other	18
107	Côte d'Ivoire	2011	Grapefruit	3
107	Côte d'Ivoire	2011	Groundnuts (Shelled Eq)	7
107	Côte d'Ivoire	2011	Maize	60
107	Côte d'Ivoire	2011	Marine Fish, Other	24
107	Côte d'Ivoire	2011	Meat, Other	24
107	Côte d'Ivoire	2011	Milk, Whole	18
107	Côte d'Ivoire	2011	Millet	4
107	Côte d'Ivoire	2011	Mutton & Goat Meat	2
107	Côte d'Ivoire	2011	Nuts	17
107	Côte d'Ivoire	2011	Offals, Edible	8
107	Côte d'Ivoire	2011	Oilcrops Oil, Other	1
107	Côte d'Ivoire	2011	Onions	10
107	Côte d'Ivoire	2011	Oranges, Mandarines	5
107	Côte d'Ivoire	2011	Palm Oil	24
107	Côte d'Ivoire	2011	Palmkernel Oil	3
107	Côte d'Ivoire	2011	Pelagic Fish	28
107	Côte d'Ivoire	2011	Pigmeat	3
107	Côte d'Ivoire	2011	Pimento	4
107	Côte d'Ivoire	2011	Pineapples	1
107	Côte d'Ivoire	2011	Plantains	176
107	Côte d'Ivoire	2011	Potatoes	3
107	Côte d'Ivoire	2011	Poultry Meat	5
107	Côte d'Ivoire	2011	Pulses, Other	2
107	Côte d'Ivoire	2011	Rice (Milled Equivalent)	167
107	Côte d'Ivoire	2011	Roots, Other	8
107	Côte d'Ivoire	2011	Sorghum	4
107	Côte d'Ivoire	2011	Spices, Other	1

FAO country code	Country	Reference year	Food item	Gross food supply (g *capita*$^{-1}$ d^{-1})
107	Côte d'Ivoire	2011	Sugar Cane	4
107	Côte d'Ivoire	2011	Sugar, Refined Equivalent	25
107	Côte d'Ivoire	2011	Sweet Potatoes	5
107	Côte d'Ivoire	2011	Sweeteners, Other	1
107	Côte d'Ivoire	2011	Tomatoes	11
107	Côte d'Ivoire	2011	Vegetables, Other	83
107	Côte d'Ivoire	2011	Wheat	55
107	Côte d'Ivoire	2011	Whey	1
107	Côte d'Ivoire	2011	Wine	4
107	Côte d'Ivoire	2011	Yams	523
98	Croatia	1992	Apples	35
98	Croatia	1992	Bananas	13
98	Croatia	1992	Beans	7
98	Croatia	1992	Beer	147
98	Croatia	1992	Beverages, Alcoholic	3
98	Croatia	1992	Bovine Meat	22
98	Croatia	1992	Butter, Ghee	1
98	Croatia	1992	Cephalopods	1
98	Croatia	1992	Cereals, Other	1
98	Croatia	1992	Cheese	10
98	Croatia	1992	Cocoa Beans	1
98	Croatia	1992	Coffee	4
98	Croatia	1992	Cream	1
98	Croatia	1992	Demersal Fish	3
98	Croatia	1992	Eggs	27
98	Croatia	1992	Fats, Animals, Raw	10
98	Croatia	1992	Freshwater Fish	2
98	Croatia	1992	Fruits, Other	45
98	Croatia	1992	Grapefruit	1
98	Croatia	1992	Grapes	45
98	Croatia	1992	Honey	1
98	Croatia	1992	Lemons, Limes	4
98	Croatia	1992	Maize	14
98	Croatia	1992	Meat, Other	2
98	Croatia	1992	Milk, Whole	330
98	Croatia	1992	Mutton & Goat Meat	2
98	Croatia	1992	Nuts	4
98	Croatia	1992	Offals, Edible	4
98	Croatia	1992	Oilcrops Oil, Other	1
98	Croatia	1992	Olive Oil	2
98	Croatia	1992	Olives	3
98	Croatia	1992	Onions	20
98	Croatia	1992	Oranges, Mandarines	17
98	Croatia	1992	Pelagic Fish	1
98	Croatia	1992	Pigmeat	49
98	Croatia	1992	Potatoes	192
98	Croatia	1992	Poultry Meat	23
98	Croatia	1992	Rice (Milled Equivalent)	2
98	Croatia	1992	Rye	1
98	Croatia	1992	Soyabean Oil	10
98	Croatia	1992	Sugar, Refined Equivalent	98
98	Croatia	1992	Sunflowerseed Oil	10
98	Croatia	1992	Sweeteners, Other	15
98	Croatia	1992	Tomatoes	27
98	Croatia	1992	Vegetables, Other	100
98	Croatia	1992	Wheat	252
98	Croatia	1992	Wine	111
98	Croatia	1993	Apples	39
98	Croatia	1993	Bananas	12
98	Croatia	1993	Beans	9
98	Croatia	1993	Beer	141
98	Croatia	1993	Beverages, Alcoholic	5
98	Croatia	1993	Bovine Meat	22
98	Croatia	1993	Butter, Ghee	1
98	Croatia	1993	Cephalopods	1
98	Croatia	1993	Cheese	11
98	Croatia	1993	Cocoa Beans	1
98	Croatia	1993	Coffee	4
98	Croatia	1993	Cream	1
98	Croatia	1993	Demersal Fish	1
98	Croatia	1993	Eggs	27
98	Croatia	1993	Fats, Animals, Raw	11
98	Croatia	1993	Freshwater Fish	2
98	Croatia	1993	Fruits, Other	51
98	Croatia	1993	Grapes	53
98	Croatia	1993	Honey	1
98	Croatia	1993	Lemons, Limes	3
98	Croatia	1993	Maize	17
98	Croatia	1993	Meat, Other	2
98	Croatia	1993	Milk, Whole	276
98	Croatia	1993	Mutton & Goat Meat	2
98	Croatia	1993	Nuts	4
98	Croatia	1993	Offals, Edible	4
98	Croatia	1993	Olive Oil	1
98	Croatia	1993	Olives	1
98	Croatia	1993	Onions	23
98	Croatia	1993	Oranges, Mandarines	28
98	Croatia	1993	Pelagic Fish	1
98	Croatia	1993	Pigmeat	40
98	Croatia	1993	Potatoes	222
98	Croatia	1993	Poultry Meat	18
98	Croatia	1993	Rice (Milled Equivalent)	2
98	Croatia	1993	Rye	1
98	Croatia	1993	Soyabean Oil	8
98	Croatia	1993	Sugar, Refined Equivalent	91
98	Croatia	1993	Sunflowerseed Oil	10
98	Croatia	1993	Sweeteners, Other	14

FAO country code	Country	Reference year	Food item	Gross food supply (g $capita^{-1}$ d^{-1})
98	Croatia	1993	Tomatoes	29
98	Croatia	1993	Vegetables, Other	125
98	Croatia	1993	Wheat	250
98	Croatia	1993	Wine	108
98	Croatia	1994	Apples	33
98	Croatia	1994	Bananas	24
98	Croatia	1994	Beans	10
98	Croatia	1994	Beer	179
98	Croatia	1994	Beverages, Alcoholic	5
98	Croatia	1994	Bovine Meat	21
98	Croatia	1994	Butter, Ghee	1
98	Croatia	1994	Cephalopods	2
98	Croatia	1994	Cereals, Other	1
98	Croatia	1994	Cheese	12
98	Croatia	1994	Citrus, Other	2
98	Croatia	1994	Cocoa Beans	1
98	Croatia	1994	Coffee	5
98	Croatia	1994	Cream	2
98	Croatia	1994	Demersal Fish	2
98	Croatia	1994	Eggs	26
98	Croatia	1994	Fats, Animals, Raw	10
98	Croatia	1994	Freshwater Fish	2
98	Croatia	1994	Fruits, Other	49
98	Croatia	1994	Grapefruit	1
98	Croatia	1994	Grapes	55
98	Croatia	1994	Groundnuts (Shelled Eq)	1
98	Croatia	1994	Lemons, Limes	5
98	Croatia	1994	Maize	20
98	Croatia	1994	Meat, Other	2
98	Croatia	1994	Milk, Whole	269
98	Croatia	1994	Mutton & Goat Meat	2
98	Croatia	1994	Nuts	5
98	Croatia	1994	Offals, Edible	3
98	Croatia	1994	Olive Oil	2
98	Croatia	1994	Olives	3
98	Croatia	1994	Onions	27
98	Croatia	1994	Oranges, Mandarines	43
98	Croatia	1994	Pelagic Fish	1
98	Croatia	1994	Pigmeat	45
98	Croatia	1994	Pineapples	1
98	Croatia	1994	Potatoes	239
98	Croatia	1994	Poultry Meat	20
98	Croatia	1994	Rice (Milled Equivalent)	4
98	Croatia	1994	Rye	1
98	Croatia	1994	Soyabean Oil	7
98	Croatia	1994	Sugar, Refined Equivalent	93
98	Croatia	1994	Sunflowerseed Oil	11
98	Croatia	1994	Sweeteners, Other	27
98	Croatia	1994	Tomatoes	34
98	Croatia	1994	Vegetables, Other	151
98	Croatia	1994	Wheat	245
98	Croatia	1994	Wine	97
98	Croatia	1995	Apples	36
98	Croatia	1995	Bananas	37
98	Croatia	1995	Beans	12
98	Croatia	1995	Beer	184
98	Croatia	1995	Beverages, Alcoholic	5
98	Croatia	1995	Bovine Meat	19
98	Croatia	1995	Butter, Ghee	1
98	Croatia	1995	Cephalopods	2
98	Croatia	1995	Cereals, Other	1
98	Croatia	1995	Cheese	13
98	Croatia	1995	Citrus, Other	2
98	Croatia	1995	Cocoa Beans	3
98	Croatia	1995	Coffee	9
98	Croatia	1995	Cream	3
98	Croatia	1995	Demersal Fish	2
98	Croatia	1995	Eggs	26
98	Croatia	1995	Fats, Animals, Raw	11
98	Croatia	1995	Freshwater Fish	2
98	Croatia	1995	Fruits, Other	55
98	Croatia	1995	Grapefruit	1
98	Croatia	1995	Grapes	48
98	Croatia	1995	Groundnuts (Shelled Eq)	1
98	Croatia	1995	Lemons, Limes	5
98	Croatia	1995	Maize	21
98	Croatia	1995	Maize Germ Oil	1
98	Croatia	1995	Meat, Other	2
98	Croatia	1995	Milk, Whole	299
98	Croatia	1995	Mutton & Goat Meat	4
98	Croatia	1995	Nuts	6
98	Croatia	1995	Offals, Edible	3
98	Croatia	1995	Oilcrops Oil, Other	1
98	Croatia	1995	Olive Oil	4
98	Croatia	1995	Olives	4
98	Croatia	1995	Onions	28
98	Croatia	1995	Oranges, Mandarines	66
98	Croatia	1995	Pigmeat	50
98	Croatia	1995	Pineapples	1
98	Croatia	1995	Potatoes	315
98	Croatia	1995	Poultry Meat	22
98	Croatia	1995	Rice (Milled Equivalent)	3
98	Croatia	1995	Rye	1
98	Croatia	1995	Soyabean Oil	7
98	Croatia	1995	Sugar, Refined Equivalent	93
98	Croatia	1995	Sunflowerseed Oil	12
98	Croatia	1995	Sweeteners, Other	29

FAO countrycode	Country	Reference year	Food item	Gross food supply (g $capita^{-1}$ d^{-1})
98	Croatia	1995	Tomatoes	42
98	Croatia	1995	Vegetables, Other	167
98	Croatia	1995	Wheat	248
98	Croatia	1995	Wine	92
98	Croatia	1996	Apples	47
98	Croatia	1996	Bananas	22
98	Croatia	1996	Beans	11
98	Croatia	1996	Beer	184
98	Croatia	1996	Beverages, Alcoholic	5
98	Croatia	1996	Bovine Meat	12
98	Croatia	1996	Butter, Ghee	2
98	Croatia	1996	Cephalopods	3
98	Croatia	1996	Cereals, Other	1
98	Croatia	1996	Cheese	13
98	Croatia	1996	Cocoa Beans	2
98	Croatia	1996	Coffee	9
98	Croatia	1996	Demersal Fish	4
98	Croatia	1996	Eggs	29
98	Croatia	1996	Fats, Animals, Raw	11
98	Croatia	1996	Freshwater Fish	1
98	Croatia	1996	Fruits, Other	76
98	Croatia	1996	Grapefruit	1
98	Croatia	1996	Grapes	58
98	Croatia	1996	Groundnuts (Shelled Eq)	1
98	Croatia	1996	Honey	1
98	Croatia	1996	Lemons, Limes	5
98	Croatia	1996	Maize	23
98	Croatia	1996	Maize Germ Oil	1
98	Croatia	1996	Marine Fish, Other	1
98	Croatia	1996	Meat, Other	2
98	Croatia	1996	Milk, Whole	298
98	Croatia	1996	Molluscs, Other	1
98	Croatia	1996	Mutton & Goat Meat	2
98	Croatia	1996	Nuts	7
98	Croatia	1996	Offals, Edible	3
98	Croatia	1996	Oilcrops Oil, Other	1
98	Croatia	1996	Olive Oil	1
98	Croatia	1996	Olives	3
98	Croatia	1996	Onions	28
98	Croatia	1996	Oranges, Mandarines	55
98	Croatia	1996	Palm Oil	1
98	Croatia	1996	Peas	1
98	Croatia	1996	Pelagic Fish	1
98	Croatia	1996	Pigmeat	47
98	Croatia	1996	Pineapples	1
98	Croatia	1996	Potatoes	300
98	Croatia	1996	Poultry Meat	18
98	Croatia	1996	Rice (Milled Equivalent)	4
98	Croatia	1996	Rye	1
98	Croatia	1996	Soyabean Oil	8
98	Croatia	1996	Sugar, Refined Equivalent	100
98	Croatia	1996	Sunflowerseed Oil	13
98	Croatia	1996	Sweeteners, Other	17
98	Croatia	1996	Tomatoes	42
98	Croatia	1996	Vegetables, Other	182
98	Croatia	1996	Wheat	256
98	Croatia	1996	Wine	101
98	Croatia	1997	Apples	35
98	Croatia	1997	Bananas	33
98	Croatia	1997	Beans	12
98	Croatia	1997	Beer	199
98	Croatia	1997	Beverages, Alcoholic	6
98	Croatia	1997	Bovine Meat	20
98	Croatia	1997	Butter, Ghee	1
98	Croatia	1997	Cephalopods	2
98	Croatia	1997	Cereals, Other	1
98	Croatia	1997	Cheese	12
98	Croatia	1997	Cocoa Beans	3
98	Croatia	1997	Coffee	12
98	Croatia	1997	Demersal Fish	3
98	Croatia	1997	Eggs	26
98	Croatia	1997	Fats, Animals, Raw	12
98	Croatia	1997	Freshwater Fish	2
98	Croatia	1997	Fruits, Other	59
98	Croatia	1997	Grapefruit	1
98	Croatia	1997	Grapes	53
98	Croatia	1997	Groundnuts (Shelled Eq)	1
98	Croatia	1997	Honey	1
98	Croatia	1997	Lemons, Limes	6
98	Croatia	1997	Maize	32
98	Croatia	1997	Meat, Other	2
98	Croatia	1997	Milk, Whole	237
98	Croatia	1997	Molluscs, Other	1
98	Croatia	1997	Mutton & Goat Meat	2
98	Croatia	1997	Nuts	6
98	Croatia	1997	Offals, Edible	3
98	Croatia	1997	Oilcrops Oil, Other	1
98	Croatia	1997	Olive Oil	1
98	Croatia	1997	Olives	2
98	Croatia	1997	Onions	30
98	Croatia	1997	Oranges, Mandarines	42
98	Croatia	1997	Peas	1
98	Croatia	1997	Pelagic Fish	1
98	Croatia	1997	Pigmeat	47
98	Croatia	1997	Pineapples	1
98	Croatia	1997	Potatoes	280
98	Croatia	1997	Poultry Meat	20

FAO countrycode	Country	Reference year	Food item	Gross food supply (g *capita*$^{-1}$ d^{-1})
98	Croatia	1997	Rice (Milled Equivalent)	5
98	Croatia	1997	Rye	1
98	Croatia	1997	Soyabean Oil	9
98	Croatia	1997	Sugar, Refined Equivalent	86
98	Croatia	1997	Sunflowerseed Oil	14
98	Croatia	1997	Sweeteners, Other	20
98	Croatia	1997	Tomatoes	41
98	Croatia	1997	Vegetables, Other	191
98	Croatia	1997	Wheat	256
98	Croatia	1997	Wine	128
98	Croatia	1998	Apples	41
98	Croatia	1998	Bananas	22
98	Croatia	1998	Beans	11
98	Croatia	1998	Beer	198
98	Croatia	1998	Beverages, Alcoholic	13
98	Croatia	1998	Bovine Meat	15
98	Croatia	1998	Butter, Ghee	1
98	Croatia	1998	Cephalopods	2
98	Croatia	1998	Cereals, Other	1
98	Croatia	1998	Cheese	11
98	Croatia	1998	Cocoa Beans	2
98	Croatia	1998	Coffee	9
98	Croatia	1998	Cream	1
98	Croatia	1998	Demersal Fish	4
98	Croatia	1998	Eggs	28
98	Croatia	1998	Fats, Animals, Raw	8
98	Croatia	1998	Freshwater Fish	2
98	Croatia	1998	Fruits, Other	66
98	Croatia	1998	Grapefruit	1
98	Croatia	1998	Grapes	59
98	Croatia	1998	Groundnuts (Shelled Eq)	1
98	Croatia	1998	Honey	1
98	Croatia	1998	Lemons, Limes	2
98	Croatia	1998	Maize	34
98	Croatia	1998	Meat, Other	2
98	Croatia	1998	Milk, Whole	276
98	Croatia	1998	Molluscs, Other	1
98	Croatia	1998	Mutton & Goat Meat	1
98	Croatia	1998	Nuts	6
98	Croatia	1998	Offals, Edible	3
98	Croatia	1998	Oilcrops Oil, Other	1
98	Croatia	1998	Olive Oil	3
98	Croatia	1998	Olives	3
98	Croatia	1998	Onions	33
98	Croatia	1998	Oranges, Mandarines	40
98	Croatia	1998	Palm Oil	1
98	Croatia	1998	Pelagic Fish	1
98	Croatia	1998	Pigmeat	43
98	Croatia	1998	Potatoes	306
98	Croatia	1998	Poultry Meat	17
98	Croatia	1998	Rice (Milled Equivalent)	4
98	Croatia	1998	Rye	1
98	Croatia	1998	Soyabean Oil	8
98	Croatia	1998	Sugar, Refined Equivalent	87
98	Croatia	1998	Sunflowerseed Oil	15
98	Croatia	1998	Sweeteners, Other	18
98	Croatia	1998	Tomatoes	47
98	Croatia	1998	Vegetables, Other	208
98	Croatia	1998	Wheat	246
98	Croatia	1998	Wine	128
98	Croatia	1999	Apples	43
98	Croatia	1999	Bananas	27
98	Croatia	1999	Beans	12
98	Croatia	1999	Beer	207
98	Croatia	1999	Beverages, Alcoholic	2
98	Croatia	1999	Bovine Meat	18
98	Croatia	1999	Butter, Ghee	1
98	Croatia	1999	Cephalopods	2
98	Croatia	1999	Cereals, Other	1
98	Croatia	1999	Cheese	13
98	Croatia	1999	Cocoa Beans	3
98	Croatia	1999	Coffee	12
98	Croatia	1999	Cream	1
98	Croatia	1999	Demersal Fish	4
98	Croatia	1999	Eggs	28
98	Croatia	1999	Fats, Animals, Raw	9
98	Croatia	1999	Freshwater Fish	2
98	Croatia	1999	Fruits, Other	55
98	Croatia	1999	Grapefruit	2
98	Croatia	1999	Grapes	59
98	Croatia	1999	Groundnuts (Shelled Eq)	1
98	Croatia	1999	Honey	1
98	Croatia	1999	Lemons, Limes	7
98	Croatia	1999	Maize	34
98	Croatia	1999	Meat, Other	2
98	Croatia	1999	Milk, Whole	295
98	Croatia	1999	Molluscs, Other	1
98	Croatia	1999	Mutton & Goat Meat	3
98	Croatia	1999	Nuts	8
98	Croatia	1999	Offals, Edible	5
98	Croatia	1999	Oilcrops Oil, Other	1
98	Croatia	1999	Olive Oil	4
98	Croatia	1999	Olives	5
98	Croatia	1999	Onions	39
98	Croatia	1999	Oranges, Mandarines	44
98	Croatia	1999	Palm Oil	1
98	Croatia	1999	Peas	1

FAO country code	Country	Reference year	Food item	Gross food supply (g $capita^{-1}$ d^{-1})
98	Croatia	1999	Pelagic Fish	2
98	Croatia	1999	Pigmeat	51
98	Croatia	1999	Pineapples	1
98	Croatia	1999	Potatoes	311
98	Croatia	1999	Poultry Meat	18
98	Croatia	1999	Rice (Milled Equivalent)	5
98	Croatia	1999	Rye	1
98	Croatia	1999	Soyabean Oil	8
98	Croatia	1999	Sugar, Refined Equivalent	70
98	Croatia	1999	Sunflowerseed Oil	17
98	Croatia	1999	Sweeteners, Other	27
98	Croatia	1999	Tomatoes	54
98	Croatia	1999	Vegetables, Other	228
98	Croatia	1999	Wheat	237
98	Croatia	1999	Wine	125
98	Croatia	2000	Apples	43
98	Croatia	2000	Bananas	24
98	Croatia	2000	Beans	6
98	Croatia	2000	Beer	231
98	Croatia	2000	Beverages, Alcoholic	3
98	Croatia	2000	Bovine Meat	19
98	Croatia	2000	Butter, Ghee	1
98	Croatia	2000	Cephalopods	3
98	Croatia	2000	Cereals, Other	1
98	Croatia	2000	Cheese	15
98	Croatia	2000	Cocoa Beans	3
98	Croatia	2000	Coffee	12
98	Croatia	2000	Cream	2
98	Croatia	2000	Demersal Fish	4
98	Croatia	2000	Eggs	27
98	Croatia	2000	Fats, Animals, Raw	10
98	Croatia	2000	Freshwater Fish	2
98	Croatia	2000	Fruits, Other	58
98	Croatia	2000	Grapefruit	2
98	Croatia	2000	Grapes	48
98	Croatia	2000	Groundnuts (Shelled Eq)	1
98	Croatia	2000	Honey	1
98	Croatia	2000	Lemons, Limes	7
98	Croatia	2000	Maize	36
98	Croatia	2000	Marine Fish, Other	1
98	Croatia	2000	Meat, Other	2
98	Croatia	2000	Milk, Whole	287
98	Croatia	2000	Molluscs, Other	2
98	Croatia	2000	Mutton & Goat Meat	2
98	Croatia	2000	Nuts	8
98	Croatia	2000	Offals, Edible	4
98	Croatia	2000	Oilcrops Oil, Other	1
98	Croatia	2000	Olive Oil	2
98	Croatia	2000	Olives	3
98	Croatia	2000	Onions	33
98	Croatia	2000	Oranges, Mandarines	44
98	Croatia	2000	Palm Oil	1
98	Croatia	2000	Pelagic Fish	9
98	Croatia	2000	Pigmeat	56
98	Croatia	2000	Pineapples	1
98	Croatia	2000	Potatoes	290
98	Croatia	2000	Poultry Meat	19
98	Croatia	2000	Rice (Milled Equivalent)	5
98	Croatia	2000	Rye	1
98	Croatia	2000	Soyabean Oil	13
98	Croatia	2000	Sugar, Refined Equivalent	67
98	Croatia	2000	Sunflowerseed Oil	15
98	Croatia	2000	Sweeteners, Other	51
98	Croatia	2000	Tomatoes	55
98	Croatia	2000	Vegetables, Other	183
98	Croatia	2000	Wheat	243
98	Croatia	2000	Wine	111
98	Croatia	2001	Apples	43
98	Croatia	2001	Bananas	25
98	Croatia	2001	Beans	11
98	Croatia	2001	Beer	236
98	Croatia	2001	Beverages, Alcoholic	4
98	Croatia	2001	Bovine Meat	15
98	Croatia	2001	Butter, Ghee	1
98	Croatia	2001	Cephalopods	3
98	Croatia	2001	Cereals, Other	1
98	Croatia	2001	Cheese	18
98	Croatia	2001	Citrus, Other	1
98	Croatia	2001	Cocoa Beans	4
98	Croatia	2001	Coffee	12
98	Croatia	2001	Cream	1
98	Croatia	2001	Crustaceans	1
98	Croatia	2001	Demersal Fish	4
98	Croatia	2001	Eggs	27
98	Croatia	2001	Fats, Animals, Raw	10
98	Croatia	2001	Freshwater Fish	2
98	Croatia	2001	Fruits, Other	58
98	Croatia	2001	Grapefruit	1
98	Croatia	2001	Grapes	51
98	Croatia	2001	Groundnuts (Shelled Eq)	1
98	Croatia	2001	Honey	1
98	Croatia	2001	Lemons, Limes	6
98	Croatia	2001	Maize	37
98	Croatia	2001	Marine Fish, Other	1
98	Croatia	2001	Meat, Other	2
98	Croatia	2001	Milk, Whole	296
98	Croatia	2001	Molluscs, Other	3

FAO country code	Country	Reference year	Food item	Gross food supply (g *capita*$^{-1}$ d^{-1})
98	Croatia	2001	Mutton & Goat Meat	2
98	Croatia	2001	Nuts	7
98	Croatia	2001	Offals, Edible	4
98	Croatia	2001	Oilcrops Oil, Other	1
98	Croatia	2001	Olive Oil	3
98	Croatia	2001	Olives	4
98	Croatia	2001	Onions	40
98	Croatia	2001	Oranges, Mandarines	44
98	Croatia	2001	Palm Oil	2
98	Croatia	2001	Peas	1
98	Croatia	2001	Pelagic Fish	14
98	Croatia	2001	Pigmeat	59
98	Croatia	2001	Pineapples	1
98	Croatia	2001	Potatoes	343
98	Croatia	2001	Poultry Meat	20
98	Croatia	2001	Rice (Milled Equivalent)	6
98	Croatia	2001	Rye	1
98	Croatia	2001	Soyabean Oil	11
98	Croatia	2001	Sugar, Refined Equivalent	73
98	Croatia	2001	Sunflowerseed Oil	15
98	Croatia	2001	Sweeteners, Other	77
98	Croatia	2001	Tomatoes	60
98	Croatia	2001	Vegetables, Other	206
98	Croatia	2001	Wheat	256
98	Croatia	2001	Wine	117
98	Croatia	2002	Apples	61
98	Croatia	2002	Bananas	26
98	Croatia	2002	Beans	6
98	Croatia	2002	Beer	228
98	Croatia	2002	Beverages, Alcoholic	5
98	Croatia	2002	Bovine Meat	16
98	Croatia	2002	Butter, Ghee	1
98	Croatia	2002	Cephalopods	3
98	Croatia	2002	Cereals, Other	2
98	Croatia	2002	Cheese	19
98	Croatia	2002	Citrus, Other	1
98	Croatia	2002	Cocoa Beans	5
98	Croatia	2002	Coffee	13
98	Croatia	2002	Cream	1
98	Croatia	2002	Demersal Fish	4
98	Croatia	2002	Eggs	27
98	Croatia	2002	Fats, Animals, Raw	8
98	Croatia	2002	Freshwater Fish	2
98	Croatia	2002	Fruits, Other	52
98	Croatia	2002	Grapefruit	2
98	Croatia	2002	Grapes	41
98	Croatia	2002	Groundnuts (Shelled Eq)	2
98	Croatia	2002	Honey	1
98	Croatia	2002	Lemons, Limes	7
98	Croatia	2002	Maize	35
98	Croatia	2002	Marine Fish, Other	2
98	Croatia	2002	Meat, Other	2
98	Croatia	2002	Milk, Whole	308
98	Croatia	2002	Molluscs, Other	3
98	Croatia	2002	Mutton & Goat Meat	2
98	Croatia	2002	Nuts	8
98	Croatia	2002	Offals, Edible	5
98	Croatia	2002	Oilcrops Oil, Other	1
98	Croatia	2002	Olive Oil	3
98	Croatia	2002	Olives	7
98	Croatia	2002	Onions	40
98	Croatia	2002	Oranges, Mandarines	34
98	Croatia	2002	Palm Oil	3
98	Croatia	2002	Peas	1
98	Croatia	2002	Pelagic Fish	24
98	Croatia	2002	Pigmeat	65
98	Croatia	2002	Pineapples	1
98	Croatia	2002	Potatoes	302
98	Croatia	2002	Poultry Meat	26
98	Croatia	2002	Rice (Milled Equivalent)	6
98	Croatia	2002	Rye	1
98	Croatia	2002	Soyabean Oil	14
98	Croatia	2002	Sugar, Refined Equivalent	70
98	Croatia	2002	Sunflowerseed Oil	14
98	Croatia	2002	Sweeteners, Other	93
98	Croatia	2002	Tomatoes	63
98	Croatia	2002	Vegetables, Other	228
98	Croatia	2002	Wheat	281
98	Croatia	2002	Wine	115
98	Croatia	2003	Apples	54
98	Croatia	2003	Bananas	30
98	Croatia	2003	Beans	6
98	Croatia	2003	Beer	234
98	Croatia	2003	Beverages, Alcoholic	7
98	Croatia	2003	Bovine Meat	17
98	Croatia	2003	Butter, Ghee	1
98	Croatia	2003	Cephalopods	3
98	Croatia	2003	Cereals, Other	3
98	Croatia	2003	Cheese	20
98	Croatia	2003	Cocoa Beans	5
98	Croatia	2003	Coffee	14
98	Croatia	2003	Cream	1
98	Croatia	2003	Crustaceans	1
98	Croatia	2003	Demersal Fish	3
98	Croatia	2003	Eggs	31
98	Croatia	2003	Fats, Animals, Raw	7
98	Croatia	2003	Freshwater Fish	2

FAO country code	Country	Reference year	Food item	Gross food supply (g *capita*$^{-1}$ d^{-1})
98	Croatia	2003	Fruits, Other	63
98	Croatia	2003	Grapefruit	2
98	Croatia	2003	Grapes	51
98	Croatia	2003	Groundnuts (Shelled Eq)	2
98	Croatia	2003	Lemons, Limes	7
98	Croatia	2003	Maize	22
98	Croatia	2003	Maize Germ Oil	4
98	Croatia	2003	Marine Fish, Other	1
98	Croatia	2003	Meat, Other	2
98	Croatia	2003	Milk, Whole	274
98	Croatia	2003	Molluscs, Other	5
98	Croatia	2003	Mutton & Goat Meat	3
98	Croatia	2003	Nuts	8
98	Croatia	2003	Offals, Edible	4
98	Croatia	2003	Oilcrops Oil, Other	1
98	Croatia	2003	Olive Oil	3
98	Croatia	2003	Olives	2
98	Croatia	2003	Onions	35
98	Croatia	2003	Oranges, Mandarines	41
98	Croatia	2003	Palm Oil	3
98	Croatia	2003	Peas	1
98	Croatia	2003	Pelagic Fish	26
98	Croatia	2003	Pigmeat	64
98	Croatia	2003	Pineapples	2
98	Croatia	2003	Potatoes	281
98	Croatia	2003	Poultry Meat	30
98	Croatia	2003	Rice (Milled Equivalent)	6
98	Croatia	2003	Rye	1
98	Croatia	2003	Soyabean Oil	13
98	Croatia	2003	Sugar, Refined Equivalent	83
98	Croatia	2003	Sunflowerseed Oil	13
98	Croatia	2003	Sweeteners, Other	96
98	Croatia	2003	Tomatoes	57
98	Croatia	2003	Vegetables, Other	188
98	Croatia	2003	Wheat	284
98	Croatia	2003	Wine	110
98	Croatia	2004	Apples	50
98	Croatia	2004	Bananas	30
98	Croatia	2004	Beans	3
98	Croatia	2004	Beer	225
98	Croatia	2004	Beverages, Alcoholic	8
98	Croatia	2004	Bovine Meat	21
98	Croatia	2004	Butter, Ghee	1
98	Croatia	2004	Cephalopods	4
98	Croatia	2004	Cereals, Other	5
98	Croatia	2004	Cheese	20
98	Croatia	2004	Citrus, Other	1
98	Croatia	2004	Cocoa Beans	3
98	Croatia	2004	Coffee	15
98	Croatia	2004	Crustaceans	1
98	Croatia	2004	Demersal Fish	6
98	Croatia	2004	Eggs	28
98	Croatia	2004	Fats, Animals, Raw	14
98	Croatia	2004	Freshwater Fish	2
98	Croatia	2004	Fruits, Other	77
98	Croatia	2004	Grapefruit	2
98	Croatia	2004	Grapes	26
98	Croatia	2004	Groundnuts (Shelled Eq)	2
98	Croatia	2004	Honey	1
98	Croatia	2004	Lemons, Limes	7
98	Croatia	2004	Maize	19
98	Croatia	2004	Maize Germ Oil	4
98	Croatia	2004	Marine Fish, Other	3
98	Croatia	2004	Meat, Other	2
98	Croatia	2004	Milk, Whole	321
98	Croatia	2004	Molluscs, Other	2
98	Croatia	2004	Mutton & Goat Meat	3
98	Croatia	2004	Nuts	9
98	Croatia	2004	Offals, Edible	4
98	Croatia	2004	Oilcrops Oil, Other	1
98	Croatia	2004	Olive Oil	4
98	Croatia	2004	Olives	11
98	Croatia	2004	Onions	14
98	Croatia	2004	Oranges, Mandarines	28
98	Croatia	2004	Palm Oil	4
98	Croatia	2004	Peas	1
98	Croatia	2004	Pelagic Fish	19
98	Croatia	2004	Pigmeat	77
98	Croatia	2004	Pineapples	2
98	Croatia	2004	Potatoes	183
98	Croatia	2004	Poultry Meat	31
98	Croatia	2004	Rice (Milled Equivalent)	6
98	Croatia	2004	Rye	1
98	Croatia	2004	Soyabean Oil	12
98	Croatia	2004	Sugar, Refined Equivalent	97
98	Croatia	2004	Sunflowerseed Oil	15
98	Croatia	2004	Sweeteners, Other	71
98	Croatia	2004	Tomatoes	38
98	Croatia	2004	Vegetables, Other	145
98	Croatia	2004	Wheat	289
98	Croatia	2004	Wine	116
98	Croatia	2005	Apples	30
98	Croatia	2005	Bananas	32
98	Croatia	2005	Beans	5
98	Croatia	2005	Beer	220
98	Croatia	2005	Beverages, Alcoholic	6
98	Croatia	2005	Bovine Meat	20

FAO country code	Country	Reference year	Food item	Gross food supply (g *capita*$^{-1}$ d^{-1})
98	Croatia	2005	Butter, Ghee	1
98	Croatia	2005	Cephalopods	4
98	Croatia	2005	Cereals, Other	9
98	Croatia	2005	Cheese	19
98	Croatia	2005	Citrus, Other	1
98	Croatia	2005	Cocoa Beans	4
98	Croatia	2005	Coffee	15
98	Croatia	2005	Crustaceans	1
98	Croatia	2005	Demersal Fish	6
98	Croatia	2005	Eggs	29
98	Croatia	2005	Fats, Animals, Raw	18
98	Croatia	2005	Freshwater Fish	3
98	Croatia	2005	Fruits, Other	48
98	Croatia	2005	Grapefruit	2
98	Croatia	2005	Grapes	55
98	Croatia	2005	Groundnuts (Shelled Eq)	2
98	Croatia	2005	Honey	2
98	Croatia	2005	Lemons, Limes	7
98	Croatia	2005	Maize	15
98	Croatia	2005	Maize Germ Oil	4
98	Croatia	2005	Marine Fish, Other	2
98	Croatia	2005	Meat, Other	2
98	Croatia	2005	Milk, Whole	361
98	Croatia	2005	Molluscs, Other	3
98	Croatia	2005	Mutton & Goat Meat	3
98	Croatia	2005	Nuts	11
98	Croatia	2005	Offals, Edible	6
98	Croatia	2005	Oilcrops Oil, Other	1
98	Croatia	2005	Olive Oil	4
98	Croatia	2005	Olives	8
98	Croatia	2005	Onions	15
98	Croatia	2005	Oranges, Mandarines	27
98	Croatia	2005	Palm Oil	8
98	Croatia	2005	Peas	1
98	Croatia	2005	Pelagic Fish	29
98	Croatia	2005	Pigmeat	87
98	Croatia	2005	Pineapples	2
98	Croatia	2005	Potatoes	178
98	Croatia	2005	Poultry Meat	24
98	Croatia	2005	Rice (Milled Equivalent)	7
98	Croatia	2005	Rye	1
98	Croatia	2005	Soyabean Oil	9
98	Croatia	2005	Sugar, Refined Equivalent	98
98	Croatia	2005	Sunflowerseed Oil	13
98	Croatia	2005	Sweeteners, Other	60
98	Croatia	2005	Tomatoes	38
98	Croatia	2005	Vegetables, Other	132
98	Croatia	2005	Wheat	291
98	Croatia	2005	Wine	80
98	Croatia	2006	Apples	55
98	Croatia	2006	Bananas	31
98	Croatia	2006	Beans	3
98	Croatia	2006	Beer	222
98	Croatia	2006	Beverages, Alcoholic	8
98	Croatia	2006	Bovine Meat	24
98	Croatia	2006	Butter, Ghee	1
98	Croatia	2006	Cephalopods	5
98	Croatia	2006	Cereals, Other	5
98	Croatia	2006	Cheese	20
98	Croatia	2006	Citrus, Other	1
98	Croatia	2006	Coffee	16
98	Croatia	2006	Crustaceans	1
98	Croatia	2006	Demersal Fish	6
98	Croatia	2006	Eggs	30
98	Croatia	2006	Fats, Animals, Raw	22
98	Croatia	2006	Freshwater Fish	3
98	Croatia	2006	Fruits, Other	65
98	Croatia	2006	Grapefruit	2
98	Croatia	2006	Grapes	59
98	Croatia	2006	Groundnuts (Shelled Eq)	2
98	Croatia	2006	Honey	1
98	Croatia	2006	Lemons, Limes	7
98	Croatia	2006	Maize	15
98	Croatia	2006	Maize Germ Oil	5
98	Croatia	2006	Marine Fish, Other	2
98	Croatia	2006	Meat, Other	2
98	Croatia	2006	Milk, Whole	401
98	Croatia	2006	Molluscs, Other	5
98	Croatia	2006	Mutton & Goat Meat	3
98	Croatia	2006	Nuts	11
98	Croatia	2006	Offals, Edible	5
98	Croatia	2006	Oilcrops Oil, Other	1
98	Croatia	2006	Olive Oil	4
98	Croatia	2006	Olives	4
98	Croatia	2006	Onions	15
98	Croatia	2006	Oranges, Mandarines	45
98	Croatia	2006	Palm Oil	7
98	Croatia	2006	Pelagic Fish	28
98	Croatia	2006	Pigmeat	104
98	Croatia	2006	Pineapples	3
98	Croatia	2006	Potatoes	181
98	Croatia	2006	Poultry Meat	25
98	Croatia	2006	Rice (Milled Equivalent)	7
98	Croatia	2006	Rye	2
98	Croatia	2006	Soyabean Oil	9
98	Croatia	2006	Sugar, Refined Equivalent	95
98	Croatia	2006	Sunflowerseed Oil	16

FAO countrycode	Country	Reference year	Food item	Gross food supply (g *capita*$^{-1}$ d^{-1})
98	Croatia	2006	Sweeteners, Other	66
98	Croatia	2006	Tomatoes	49
98	Croatia	2006	Vegetables, Other	157
98	Croatia	2006	Wheat	285
98	Croatia	2006	Wine	51
98	Croatia	2007	Apples	47
98	Croatia	2007	Bananas	33
98	Croatia	2007	Beans	3
98	Croatia	2007	Beer	237
98	Croatia	2007	Beverages, Alcoholic	10
98	Croatia	2007	Bovine Meat	24
98	Croatia	2007	Butter, Ghee	2
98	Croatia	2007	Cephalopods	6
98	Croatia	2007	Cereals, Other	6
98	Croatia	2007	Cheese	24
98	Croatia	2007	Citrus, Other	1
98	Croatia	2007	Cocoa Beans	3
98	Croatia	2007	Coffee	16
98	Croatia	2007	Crustaceans	1
98	Croatia	2007	Demersal Fish	7
98	Croatia	2007	Eggs	30
98	Croatia	2007	Fats, Animals, Raw	26
98	Croatia	2007	Freshwater Fish	3
98	Croatia	2007	Fruits, Other	76
98	Croatia	2007	Grapefruit	3
98	Croatia	2007	Grapes	74
98	Croatia	2007	Groundnuts (Shelled Eq)	2
98	Croatia	2007	Honey	1
98	Croatia	2007	Lemons, Limes	8
98	Croatia	2007	Maize	22
98	Croatia	2007	Maize Germ Oil	5
98	Croatia	2007	Marine Fish, Other	1
98	Croatia	2007	Meat, Other	2
98	Croatia	2007	Milk, Whole	350
98	Croatia	2007	Molluscs, Other	6
98	Croatia	2007	Mutton & Goat Meat	3
98	Croatia	2007	Nuts	12
98	Croatia	2007	Offals, Edible	6
98	Croatia	2007	Oilcrops Oil, Other	1
98	Croatia	2007	Olive Oil	4
98	Croatia	2007	Olives	9
98	Croatia	2007	Onions	22
98	Croatia	2007	Oranges, Mandarines	49
98	Croatia	2007	Palm Oil	6
98	Croatia	2007	Peas	1
98	Croatia	2007	Pelagic Fish	21
98	Croatia	2007	Pigmeat	114
98	Croatia	2007	Pineapples	3
98	Croatia	2007	Potatoes	191
98	Croatia	2007	Poultry Meat	29
98	Croatia	2007	Rice (Milled Equivalent)	7
98	Croatia	2007	Rye	2
98	Croatia	2007	Soyabean Oil	7
98	Croatia	2007	Sugar, Refined Equivalent	85
98	Croatia	2007	Sunflowerseed Oil	16
98	Croatia	2007	Sweeteners, Other	44
98	Croatia	2007	Tomatoes	58
98	Croatia	2007	Vegetables, Other	161
98	Croatia	2007	Wheat	267
98	Croatia	2007	Wine	49
98	Croatia	2008	Apples	56
98	Croatia	2008	Bananas	34
98	Croatia	2008	Barley	2
98	Croatia	2008	Beans	3
98	Croatia	2008	Beer	233
98	Croatia	2008	Beverages, Alcoholic	8
98	Croatia	2008	Bovine Meat	27
98	Croatia	2008	Butter, Ghee	3
98	Croatia	2008	Cephalopods	6
98	Croatia	2008	Cereals, Other	8
98	Croatia	2008	Cheese	26
98	Croatia	2008	Citrus, Other	1
98	Croatia	2008	Cocoa Beans	7
98	Croatia	2008	Coffee	16
98	Croatia	2008	Cream	1
98	Croatia	2008	Crustaceans	1
98	Croatia	2008	Demersal Fish	8
98	Croatia	2008	Eggs	29
98	Croatia	2008	Fats, Animals, Raw	22
98	Croatia	2008	Freshwater Fish	3
98	Croatia	2008	Fruits, Other	68
98	Croatia	2008	Grapefruit	2
98	Croatia	2008	Grapes	77
98	Croatia	2008	Groundnuts (Shelled Eq)	2
98	Croatia	2008	Honey	1
98	Croatia	2008	Lemons, Limes	7
98	Croatia	2008	Maize	22
98	Croatia	2008	Maize Germ Oil	5
98	Croatia	2008	Marine Fish, Other	6
98	Croatia	2008	Meat, Other	2
98	Croatia	2008	Milk, Whole	322
98	Croatia	2008	Molluscs, Other	5
98	Croatia	2008	Mutton & Goat Meat	3
98	Croatia	2008	Nuts	11
98	Croatia	2008	Offals, Edible	6
98	Croatia	2008	Oilcrops Oil, Other	1
98	Croatia	2008	Oilcrops, Other	1

FAO country code	Country	Reference year	Food item	Gross food supply (g $capita^{-1}$ d^{-1})
98	Croatia	2008	Olive Oil	4
98	Croatia	2008	Olives	8
98	Croatia	2008	Onions	27
98	Croatia	2008	Oranges, Mandarines	43
98	Croatia	2008	Palm Oil	8
98	Croatia	2008	Peas	1
98	Croatia	2008	Pelagic Fish	24
98	Croatia	2008	Pigmeat	111
98	Croatia	2008	Pineapples	4
98	Croatia	2008	Potatoes	168
98	Croatia	2008	Poultry Meat	29
98	Croatia	2008	Rice (Milled Equivalent)	7
98	Croatia	2008	Rye	2
98	Croatia	2008	Soyabean Oil	8
98	Croatia	2008	Sugar, Refined Equivalent	88
98	Croatia	2008	Sunflowerseed Oil	17
98	Croatia	2008	Sweeteners, Other	53
98	Croatia	2008	Tomatoes	49
98	Croatia	2008	Vegetables, Other	168
98	Croatia	2008	Wheat	272
98	Croatia	2008	Wine	39
98	Croatia	2009	Apples	64
98	Croatia	2009	Bananas	31
98	Croatia	2009	Barley	2
98	Croatia	2009	Beans	2
98	Croatia	2009	Beer	220
98	Croatia	2009	Beverages, Alcoholic	7
98	Croatia	2009	Bovine Meat	28
98	Croatia	2009	Butter, Ghee	3
98	Croatia	2009	Cephalopods	6
98	Croatia	2009	Cereals, Other	8
98	Croatia	2009	Cheese	24
98	Croatia	2009	Citrus, Other	1
98	Croatia	2009	Cocoa Beans	6
98	Croatia	2009	Coffee	15
98	Croatia	2009	Crustaceans	1
98	Croatia	2009	Demersal Fish	7
98	Croatia	2009	Eggs	30
98	Croatia	2009	Fats, Animals, Raw	21
98	Croatia	2009	Freshwater Fish	4
98	Croatia	2009	Fruits, Other	66
98	Croatia	2009	Grapefruit	2
98	Croatia	2009	Grapes	85
98	Croatia	2009	Groundnuts (Shelled Eq)	2
98	Croatia	2009	Honey	1
98	Croatia	2009	Lemons, Limes	8
98	Croatia	2009	Maize	24
98	Croatia	2009	Maize Germ Oil	5
98	Croatia	2009	Marine Fish, Other	1
98	Croatia	2009	Meat, Other	2
98	Croatia	2009	Milk, Whole	299
98	Croatia	2009	Molluscs, Other	5
98	Croatia	2009	Mutton & Goat Meat	3
98	Croatia	2009	Nuts	12
98	Croatia	2009	Offals, Edible	7
98	Croatia	2009	Oilcrops Oil, Other	1
98	Croatia	2009	Oilcrops, Other	1
98	Croatia	2009	Olive Oil	2
98	Croatia	2009	Olives	18
98	Croatia	2009	Onions	26
98	Croatia	2009	Oranges, Mandarines	41
98	Croatia	2009	Palm Oil	7
98	Croatia	2009	Peas	1
98	Croatia	2009	Pelagic Fish	29
98	Croatia	2009	Pigmeat	123
98	Croatia	2009	Pineapples	3
98	Croatia	2009	Potatoes	174
98	Croatia	2009	Poultry Meat	28
98	Croatia	2009	Rice (Milled Equivalent)	7
98	Croatia	2009	Rye	2
98	Croatia	2009	Soyabean Oil	9
98	Croatia	2009	Sugar, Refined Equivalent	88
98	Croatia	2009	Sunflowerseed Oil	13
98	Croatia	2009	Sweeteners, Other	51
98	Croatia	2009	Tomatoes	53
98	Croatia	2009	Vegetables, Other	187
98	Croatia	2009	Wheat	282
98	Croatia	2009	Wine	42
98	Croatia	2010	Apples	56
98	Croatia	2010	Bananas	30
98	Croatia	2010	Barley	2
98	Croatia	2010	Beans	1
98	Croatia	2010	Beer	210
98	Croatia	2010	Beverages, Alcoholic	8
98	Croatia	2010	Beverages, Fermented	1
98	Croatia	2010	Bovine Meat	25
98	Croatia	2010	Butter, Ghee	2
98	Croatia	2010	Cephalopods	5
98	Croatia	2010	Cereals, Other	1
98	Croatia	2010	Cheese	25
98	Croatia	2010	Cocoa Beans	7
98	Croatia	2010	Coffee	15
98	Croatia	2010	Cream	1
98	Croatia	2010	Crustaceans	1
98	Croatia	2010	Demersal Fish	10
98	Croatia	2010	Eggs	27
98	Croatia	2010	Fats, Animals, Raw	19

FAO country code	Country	Reference year	Food item	Gross food supply (g $capita^{-1}$ d^{-1})
98	Croatia	2010	Freshwater Fish	4
98	Croatia	2010	Fruits, Other	59
98	Croatia	2010	Grapefruit	2
98	Croatia	2010	Grapes	94
98	Croatia	2010	Groundnuts (Shelled Eq)	2
98	Croatia	2010	Honey	1
98	Croatia	2010	Lemons, Limes	7
98	Croatia	2010	Maize	20
98	Croatia	2010	Maize Germ Oil	5
98	Croatia	2010	Marine Fish, Other	1
98	Croatia	2010	Meat, Other	2
98	Croatia	2010	Milk, Whole	308
98	Croatia	2010	Molluscs, Other	5
98	Croatia	2010	Mutton & Goat Meat	2
98	Croatia	2010	Nuts	13
98	Croatia	2010	Offals, Edible	6
98	Croatia	2010	Oilcrops Oil, Other	2
98	Croatia	2010	Oilcrops, Other	1
98	Croatia	2010	Olive Oil	2
98	Croatia	2010	Olives	20
98	Croatia	2010	Onions	23
98	Croatia	2010	Oranges, Mandarines	39
98	Croatia	2010	Palm Oil	6
98	Croatia	2010	Pelagic Fish	24
98	Croatia	2010	Pigmeat	115
98	Croatia	2010	Pineapples	3
98	Croatia	2010	Potatoes	129
98	Croatia	2010	Poultry Meat	22
98	Croatia	2010	Rice (Milled Equivalent)	7
98	Croatia	2010	Rye	2
98	Croatia	2010	Soyabean Oil	8
98	Croatia	2010	Sugar, Refined Equivalent	96
98	Croatia	2010	Sunflowerseed Oil	8
98	Croatia	2010	Sweeteners, Other	60
98	Croatia	2010	Tomatoes	51
98	Croatia	2010	Vegetables, Other	148
98	Croatia	2010	Wheat	292
98	Croatia	2010	Wine	37
98	Croatia	2011	Apples	69
98	Croatia	2011	Bananas	29
98	Croatia	2011	Barley	2
98	Croatia	2011	Beans	2
98	Croatia	2011	Beer	230
98	Croatia	2011	Beverages, Alcoholic	9
98	Croatia	2011	Beverages, Fermented	5
98	Croatia	2011	Bovine Meat	25
98	Croatia	2011	Butter, Ghee	2
98	Croatia	2011	Cephalopods	7
98	Croatia	2011	Cereals, Other	2
98	Croatia	2011	Cheese	26
98	Croatia	2011	Cocoa Beans	7
98	Croatia	2011	Coffee	15
98	Croatia	2011	Cream	1
98	Croatia	2011	Crustaceans	1
98	Croatia	2011	Demersal Fish	10
98	Croatia	2011	Eggs	26
98	Croatia	2011	Fats, Animals, Raw	19
98	Croatia	2011	Freshwater Fish	4
98	Croatia	2011	Fruits, Other	62
98	Croatia	2011	Grapefruit	2
98	Croatia	2011	Grapes	93
98	Croatia	2011	Groundnuts (Shelled Eq)	2
98	Croatia	2011	Honey	2
98	Croatia	2011	Lemons, Limes	8
98	Croatia	2011	Maize	22
98	Croatia	2011	Maize Germ Oil	5
98	Croatia	2011	Marine Fish, Other	2
98	Croatia	2011	Meat, Other	2
98	Croatia	2011	Milk, Whole	322
98	Croatia	2011	Molluscs, Other	4
98	Croatia	2011	Mutton & Goat Meat	2
98	Croatia	2011	Nuts	10
98	Croatia	2011	Offals, Edible	5
98	Croatia	2011	Oilcrops Oil, Other	1
98	Croatia	2011	Oilcrops, Other	1
98	Croatia	2011	Olive Oil	2
98	Croatia	2011	Olives	19
98	Croatia	2011	Onions	24
98	Croatia	2011	Oranges, Mandarines	43
98	Croatia	2011	Palm Oil	6
98	Croatia	2011	Pelagic Fish	27
98	Croatia	2011	Pigmeat	117
98	Croatia	2011	Pineapples	2
98	Croatia	2011	Potatoes	116
98	Croatia	2011	Poultry Meat	24
98	Croatia	2011	Rice (Milled Equivalent)	7
98	Croatia	2011	Rye	2
98	Croatia	2011	Soyabean Oil	8
98	Croatia	2011	Sugar, Refined Equivalent	93
98	Croatia	2011	Sunflowerseed Oil	9
98	Croatia	2011	Sweeteners, Other	42
98	Croatia	2011	Tomatoes	50
98	Croatia	2011	Vegetables, Other	149
98	Croatia	2011	Wheat	291
98	Croatia	2011	Wine	38
49	Cuba	1992	Bananas	64
49	Cuba	1992	Beans	4

FAO countrycode	Country	Reference year	Food item	Gross food supply (g *capita*$^{-1}$ d^{-1})
49	Cuba	1992	Beer	49
49	Cuba	1992	Beverages, Alcoholic	14
49	Cuba	1992	Bovine Meat	20
49	Cuba	1992	Butter, Ghee	4
49	Cuba	1992	Cassava	46
49	Cuba	1992	Cheese	4
49	Cuba	1992	Citrus, Other	1
49	Cuba	1992	Cocoa Beans	1
49	Cuba	1992	Coconuts - Incl Copra	2
49	Cuba	1992	Coffee	2
49	Cuba	1992	Demersal Fish	19
49	Cuba	1992	Eggs	24
49	Cuba	1992	Fats, Animals, Raw	8
49	Cuba	1992	Freshwater Fish	6
49	Cuba	1992	Fruits, Other	37
49	Cuba	1992	Grapefruit	43
49	Cuba	1992	Groundnuts (Shelled Eq)	1
49	Cuba	1992	Honey	1
49	Cuba	1992	Lemons, Limes	4
49	Cuba	1992	Maize	8
49	Cuba	1992	Marine Fish, Other	6
49	Cuba	1992	Meat, Other	3
49	Cuba	1992	Milk, Whole	83
49	Cuba	1992	Molluscs, Other	1
49	Cuba	1992	Mutton & Goat Meat	2
49	Cuba	1992	Offals, Edible	7
49	Cuba	1992	Onions	4
49	Cuba	1992	Oranges, Mandarines	85
49	Cuba	1992	Palm Oil	4
49	Cuba	1992	Pelagic Fish	2
49	Cuba	1992	Pigmeat	21
49	Cuba	1992	Pineapples	5
49	Cuba	1992	Plantains	69
49	Cuba	1992	Potatoes	67
49	Cuba	1992	Poultry Meat	18
49	Cuba	1992	Pulses, Other	40
49	Cuba	1992	Rice (Milled Equivalent)	127
49	Cuba	1992	Roots, Other	4
49	Cuba	1992	Soyabean Oil	9
49	Cuba	1992	Sugar, Refined Equivalent	147
49	Cuba	1992	Sunflowerseed Oil	8
49	Cuba	1992	Sweet Potatoes	51
49	Cuba	1992	Tomatoes	60
49	Cuba	1992	Vegetables, Other	74
49	Cuba	1992	Wheat	129
49	Cuba	1992	Yams	2
49	Cuba	1993	Bananas	38
49	Cuba	1993	Beans	3
49	Cuba	1993	Beer	35
49	Cuba	1993	Beverages, Alcoholic	11
49	Cuba	1993	Bovine Meat	17
49	Cuba	1993	Butter, Ghee	4
49	Cuba	1993	Cassava	34
49	Cuba	1993	Cephalopods	1
49	Cuba	1993	Cheese	4
49	Cuba	1993	Citrus, Other	1
49	Cuba	1993	Coconuts - Incl Copra	3
49	Cuba	1993	Coffee	2
49	Cuba	1993	Demersal Fish	25
49	Cuba	1993	Eggs	16
49	Cuba	1993	Fats, Animals, Raw	6
49	Cuba	1993	Freshwater Fish	6
49	Cuba	1993	Fruits, Other	24
49	Cuba	1993	Grapefruit	47
49	Cuba	1993	Groundnuts (Shelled Eq)	1
49	Cuba	1993	Lemons, Limes	3
49	Cuba	1993	Maize	8
49	Cuba	1993	Marine Fish, Other	4
49	Cuba	1993	Meat, Other	3
49	Cuba	1993	Milk, Whole	75
49	Cuba	1993	Molluscs, Other	1
49	Cuba	1993	Mutton & Goat Meat	1
49	Cuba	1993	Offals, Edible	6
49	Cuba	1993	Onions	2
49	Cuba	1993	Oranges, Mandarines	79
49	Cuba	1993	Palm Oil	8
49	Cuba	1993	Pelagic Fish	1
49	Cuba	1993	Pigmeat	28
49	Cuba	1993	Pineapples	2
49	Cuba	1993	Plantains	54
49	Cuba	1993	Potatoes	65
49	Cuba	1993	Poultry Meat	17
49	Cuba	1993	Pulses, Other	29
49	Cuba	1993	Rice (Milled Equivalent)	122
49	Cuba	1993	Roots, Other	2
49	Cuba	1993	Soyabean Oil	10
49	Cuba	1993	Sugar, Refined Equivalent	147
49	Cuba	1993	Sunflowerseed Oil	5
49	Cuba	1993	Sweet Potatoes	33
49	Cuba	1993	Tomatoes	38
49	Cuba	1993	Vegetables, Other	64
49	Cuba	1993	Wheat	115
49	Cuba	1993	Yams	2
49	Cuba	1994	Bananas	37
49	Cuba	1994	Beans	4
49	Cuba	1994	Beer	32
49	Cuba	1994	Beverages, Alcoholic	16

FAO country code	Country	Reference year	Food item	Gross food supply (g *capita*$^{-1}$ d^{-1})
49	Cuba	1994	Bovine Meat	16
49	Cuba	1994	Butter, Ghee	4
49	Cuba	1994	Cassava	28
49	Cuba	1994	Cephalopods	1
49	Cuba	1994	Cheese	4
49	Cuba	1994	Citrus, Other	1
49	Cuba	1994	Coconuts - Incl Copra	2
49	Cuba	1994	Coffee	2
49	Cuba	1994	Demersal Fish	19
49	Cuba	1994	Eggs	16
49	Cuba	1994	Fats, Animals, Raw	6
49	Cuba	1994	Freshwater Fish	6
49	Cuba	1994	Fruits, Other	30
49	Cuba	1994	Grapefruit	45
49	Cuba	1994	Groundnuts (Shelled Eq)	1
49	Cuba	1994	Lemons, Limes	3
49	Cuba	1994	Maize	6
49	Cuba	1994	Marine Fish, Other	5
49	Cuba	1994	Meat, Other	3
49	Cuba	1994	Milk, Whole	86
49	Cuba	1994	Molluscs, Other	1
49	Cuba	1994	Mutton & Goat Meat	2
49	Cuba	1994	Offals, Edible	5
49	Cuba	1994	Onions	2
49	Cuba	1994	Oranges, Mandarines	61
49	Cuba	1994	Palm Oil	11
49	Cuba	1994	Pelagic Fish	1
49	Cuba	1994	Pigmeat	30
49	Cuba	1994	Pineapples	2
49	Cuba	1994	Plantains	53
49	Cuba	1994	Potatoes	51
49	Cuba	1994	Poultry Meat	17
49	Cuba	1994	Pulses, Other	42
49	Cuba	1994	Rice (Milled Equivalent)	134
49	Cuba	1994	Roots, Other	2
49	Cuba	1994	Soyabean Oil	11
49	Cuba	1994	Sugar, Refined Equivalent	138
49	Cuba	1994	Sunflowerseed Oil	1
49	Cuba	1994	Sweet Potatoes	36
49	Cuba	1994	Tomatoes	21
49	Cuba	1994	Vegetables, Other	63
49	Cuba	1994	Wheat	119
49	Cuba	1994	Yams	2
49	Cuba	1995	Bananas	41
49	Cuba	1995	Beans	5
49	Cuba	1995	Beer	35
49	Cuba	1995	Beverages, Alcoholic	15
49	Cuba	1995	Bovine Meat	17
49	Cuba	1995	Butter, Ghee	1
49	Cuba	1995	Cassava	31
49	Cuba	1995	Cheese	4
49	Cuba	1995	Citrus, Other	1
49	Cuba	1995	Cocoa Beans	1
49	Cuba	1995	Coconuts - Incl Copra	3
49	Cuba	1995	Coffee	2
49	Cuba	1995	Demersal Fish	23
49	Cuba	1995	Eggs	15
49	Cuba	1995	Fats, Animals, Raw	3
49	Cuba	1995	Freshwater Fish	7
49	Cuba	1995	Fruits, Other	45
49	Cuba	1995	Grapefruit	54
49	Cuba	1995	Groundnut Oil	1
49	Cuba	1995	Groundnuts (Shelled Eq)	2
49	Cuba	1995	Lemons, Limes	4
49	Cuba	1995	Maize	7
49	Cuba	1995	Marine Fish, Other	5
49	Cuba	1995	Meat, Other	4
49	Cuba	1995	Milk, Whole	125
49	Cuba	1995	Molluscs, Other	1
49	Cuba	1995	Mutton & Goat Meat	1
49	Cuba	1995	Offals, Edible	5
49	Cuba	1995	Onions	2
49	Cuba	1995	Oranges, Mandarines	65
49	Cuba	1995	Palm Oil	7
49	Cuba	1995	Pelagic Fish	1
49	Cuba	1995	Pigmeat	27
49	Cuba	1995	Pineapples	2
49	Cuba	1995	Plantains	57
49	Cuba	1995	Potatoes	69
49	Cuba	1995	Poultry Meat	18
49	Cuba	1995	Pulses, Other	32
49	Cuba	1995	Rice (Milled Equivalent)	135
49	Cuba	1995	Roots, Other	2
49	Cuba	1995	Soyabean Oil	8
49	Cuba	1995	Sugar, Refined Equivalent	147
49	Cuba	1995	Sunflowerseed Oil	2
49	Cuba	1995	Sweet Potatoes	39
49	Cuba	1995	Tomatoes	40
49	Cuba	1995	Vegetables, Other	68
49	Cuba	1995	Wheat	120
49	Cuba	1995	Yams	1
49	Cuba	1996	Bananas	44
49	Cuba	1996	Beans	6
49	Cuba	1996	Beer	40
49	Cuba	1996	Beverages, Alcoholic	13
49	Cuba	1996	Bovine Meat	18
49	Cuba	1996	Cassava	37

FAO country code	Country	Reference year	Food item	Gross food supply (g *capita*$^{-1}$ d^{-1})
49	Cuba	1996	Cheese	4
49	Cuba	1996	Citrus, Other	1
49	Cuba	1996	Cocoa Beans	1
49	Cuba	1996	Coconuts - Incl Copra	4
49	Cuba	1996	Coffee	2
49	Cuba	1996	Demersal Fish	19
49	Cuba	1996	Eggs	14
49	Cuba	1996	Fats, Animals, Raw	3
49	Cuba	1996	Freshwater Fish	11
49	Cuba	1996	Fruits, Other	42
49	Cuba	1996	Grapefruit	74
49	Cuba	1996	Groundnuts (Shelled Eq)	2
49	Cuba	1996	Lemons, Limes	4
49	Cuba	1996	Maize	7
49	Cuba	1996	Marine Fish, Other	4
49	Cuba	1996	Meat, Other	4
49	Cuba	1996	Milk, Whole	124
49	Cuba	1996	Molluscs, Other	1
49	Cuba	1996	Mutton & Goat Meat	1
49	Cuba	1996	Offals, Edible	5
49	Cuba	1996	Onions	3
49	Cuba	1996	Oranges, Mandarines	68
49	Cuba	1996	Palm Oil	6
49	Cuba	1996	Pelagic Fish	1
49	Cuba	1996	Pigmeat	28
49	Cuba	1996	Pineapples	2
49	Cuba	1996	Plantains	88
49	Cuba	1996	Potatoes	90
49	Cuba	1996	Poultry Meat	19
49	Cuba	1996	Pulses, Other	33
49	Cuba	1996	Rice (Milled Equivalent)	143
49	Cuba	1996	Roots, Other	3
49	Cuba	1996	Soyabean Oil	8
49	Cuba	1996	Sugar, Refined Equivalent	154
49	Cuba	1996	Sunflowerseed Oil	2
49	Cuba	1996	Sweet Potatoes	39
49	Cuba	1996	Tomatoes	46
49	Cuba	1996	Vegetables, Other	89
49	Cuba	1996	Wheat	127
49	Cuba	1996	Yams	1
49	Cuba	1997	Bananas	33
49	Cuba	1997	Beans	7
49	Cuba	1997	Beer	37
49	Cuba	1997	Beverages, Alcoholic	13
49	Cuba	1997	Bovine Meat	18
49	Cuba	1997	Cassava	37
49	Cuba	1997	Cephalopods	1
49	Cuba	1997	Cheese	5
49	Cuba	1997	Citrus, Other	1
49	Cuba	1997	Coconuts - Incl Copra	5
49	Cuba	1997	Coffee	4
49	Cuba	1997	Crustaceans	1
49	Cuba	1997	Demersal Fish	15
49	Cuba	1997	Eggs	16
49	Cuba	1997	Fats, Animals, Raw	3
49	Cuba	1997	Freshwater Fish	10
49	Cuba	1997	Fruits, Other	41
49	Cuba	1997	Grapefruit	60
49	Cuba	1997	Groundnut Oil	1
49	Cuba	1997	Groundnuts (Shelled Eq)	2
49	Cuba	1997	Lemons, Limes	4
49	Cuba	1997	Maize	9
49	Cuba	1997	Marine Fish, Other	4
49	Cuba	1997	Meat, Other	4
49	Cuba	1997	Milk, Whole	126
49	Cuba	1997	Molluscs, Other	1
49	Cuba	1997	Mutton & Goat Meat	1
49	Cuba	1997	Offals, Edible	4
49	Cuba	1997	Onions	3
49	Cuba	1997	Oranges, Mandarines	114
49	Cuba	1997	Palm Oil	6
49	Cuba	1997	Pelagic Fish	1
49	Cuba	1997	Pigmeat	21
49	Cuba	1997	Pineapples	1
49	Cuba	1997	Plantains	73
49	Cuba	1997	Potatoes	84
49	Cuba	1997	Poultry Meat	23
49	Cuba	1997	Pulses, Other	37
49	Cuba	1997	Rice (Milled Equivalent)	151
49	Cuba	1997	Roots, Other	5
49	Cuba	1997	Soyabean Oil	8
49	Cuba	1997	Sugar, Refined Equivalent	168
49	Cuba	1997	Sunflowerseed Oil	2
49	Cuba	1997	Sweet Potatoes	38
49	Cuba	1997	Tomatoes	41
49	Cuba	1997	Vegetables, Other	86
49	Cuba	1997	Wheat	137
49	Cuba	1998	Bananas	37
49	Cuba	1998	Beans	9
49	Cuba	1998	Beer	49
49	Cuba	1998	Beverages, Alcoholic	14
49	Cuba	1998	Bovine Meat	18
49	Cuba	1998	Butter, Ghee	1
49	Cuba	1998	Cassava	56
49	Cuba	1998	Cheese	4
49	Cuba	1998	Citrus, Other	1
49	Cuba	1998	Cocoa Beans	1

FAO country code	Country	Reference year	Food item	Gross food supply (g $capita^{-1}$ d^{-1})
49	Cuba	1998	Coconuts - Incl Copra	8
49	Cuba	1998	Coffee	2
49	Cuba	1998	Demersal Fish	14
49	Cuba	1998	Eggs	14
49	Cuba	1998	Fats, Animals, Raw	3
49	Cuba	1998	Freshwater Fish	10
49	Cuba	1998	Fruits, Other	57
49	Cuba	1998	Grapefruit	69
49	Cuba	1998	Groundnut Oil	1
49	Cuba	1998	Groundnuts (Shelled Eq)	2
49	Cuba	1998	Lemons, Limes	4
49	Cuba	1998	Maize	13
49	Cuba	1998	Marine Fish, Other	3
49	Cuba	1998	Meat, Other	4
49	Cuba	1998	Milk, Whole	127
49	Cuba	1998	Molluscs, Other	1
49	Cuba	1998	Mutton & Goat Meat	1
49	Cuba	1998	Offals, Edible	5
49	Cuba	1998	Oilcrops Oil, Other	1
49	Cuba	1998	Onions	5
49	Cuba	1998	Oranges, Mandarines	87
49	Cuba	1998	Palm Oil	7
49	Cuba	1998	Pelagic Fish	3
49	Cuba	1998	Pigmeat	22
49	Cuba	1998	Pineapples	2
49	Cuba	1998	Plantains	75
49	Cuba	1998	Potatoes	54
49	Cuba	1998	Poultry Meat	27
49	Cuba	1998	Pulses, Other	40
49	Cuba	1998	Rice (Milled Equivalent)	153
49	Cuba	1998	Roots, Other	9
49	Cuba	1998	Soyabean Oil	9
49	Cuba	1998	Soyabeans	1
49	Cuba	1998	Sugar, Refined Equivalent	162
49	Cuba	1998	Sunflowerseed Oil	2
49	Cuba	1998	Sweet Potatoes	45
49	Cuba	1998	Tomatoes	32
49	Cuba	1998	Vegetables, Other	125
49	Cuba	1998	Wheat	138
49	Cuba	1998	Yams	2
49	Cuba	1999	Bananas	34
49	Cuba	1999	Beans	34
49	Cuba	1999	Beer	51
49	Cuba	1999	Beverages, Alcoholic	15
49	Cuba	1999	Bovine Meat	20
49	Cuba	1999	Butter, Ghee	1
49	Cuba	1999	Cassava	68
49	Cuba	1999	Cheese	4
49	Cuba	1999	Citrus, Other	1
49	Cuba	1999	Cocoa Beans	1
49	Cuba	1999	Coconuts - Incl Copra	10
49	Cuba	1999	Coffee	5
49	Cuba	1999	Crustaceans	1
49	Cuba	1999	Demersal Fish	4
49	Cuba	1999	Eggs	17
49	Cuba	1999	Fats, Animals, Raw	3
49	Cuba	1999	Freshwater Fish	10
49	Cuba	1999	Fruits, Other	104
49	Cuba	1999	Grapefruit	25
49	Cuba	1999	Grapes	1
49	Cuba	1999	Groundnuts (Shelled Eq)	1
49	Cuba	1999	Lemons, Limes	5
49	Cuba	1999	Maize	19
49	Cuba	1999	Marine Fish, Other	9
49	Cuba	1999	Milk, Whole	165
49	Cuba	1999	Molluscs, Other	1
49	Cuba	1999	Mutton & Goat Meat	2
49	Cuba	1999	Offals, Edible	6
49	Cuba	1999	Oilcrops Oil, Other	1
49	Cuba	1999	Onions	10
49	Cuba	1999	Oranges, Mandarines	67
49	Cuba	1999	Pelagic Fish	3
49	Cuba	1999	Pigmeat	29
49	Cuba	1999	Pineapples	3
49	Cuba	1999	Plantains	84
49	Cuba	1999	Potatoes	85
49	Cuba	1999	Poultry Meat	30
49	Cuba	1999	Pulses, Other	32
49	Cuba	1999	Rice (Milled Equivalent)	164
49	Cuba	1999	Roots, Other	17
49	Cuba	1999	Soyabean Oil	7
49	Cuba	1999	Soyabeans	1
49	Cuba	1999	Sugar, Refined Equivalent	171
49	Cuba	1999	Sunflowerseed Oil	7
49	Cuba	1999	Sweet Potatoes	47
49	Cuba	1999	Sweeteners, Other	1
49	Cuba	1999	Tomatoes	90
49	Cuba	1999	Vegetables, Other	168
49	Cuba	1999	Wheat	144
49	Cuba	1999	Wine	4
49	Cuba	1999	Yams	2
49	Cuba	2000	Bananas	49
49	Cuba	2000	Beans	36
49	Cuba	2000	Beer	53
49	Cuba	2000	Beverages, Alcoholic	14
49	Cuba	2000	Bovine Meat	20
49	Cuba	2000	Butter, Ghee	1

FAO country code	Country	Reference year	Food item	Gross food supply (g $capita^{-1}$ d^{-1})
49	Cuba	2000	Cassava	69
49	Cuba	2000	Cheese	4
49	Cuba	2000	Cocoa Beans	1
49	Cuba	2000	Coconuts - Incl Copra	18
49	Cuba	2000	Coffee	3
49	Cuba	2000	Demersal Fish	3
49	Cuba	2000	Eggs	17
49	Cuba	2000	Fats, Animals, Raw	2
49	Cuba	2000	Freshwater Fish	8
49	Cuba	2000	Fruits, Other	123
49	Cuba	2000	Grapefruit	36
49	Cuba	2000	Grapes	1
49	Cuba	2000	Groundnuts (Shelled Eq)	1
49	Cuba	2000	Lemons, Limes	4
49	Cuba	2000	Maize	30
49	Cuba	2000	Marine Fish, Other	13
49	Cuba	2000	Milk, Whole	170
49	Cuba	2000	Molluscs, Other	2
49	Cuba	2000	Mutton & Goat Meat	2
49	Cuba	2000	Offals, Edible	5
49	Cuba	2000	Oilcrops Oil, Other	1
49	Cuba	2000	Onions	16
49	Cuba	2000	Oranges, Mandarines	64
49	Cuba	2000	Pelagic Fish	6
49	Cuba	2000	Pigmeat	29
49	Cuba	2000	Pineapples	5
49	Cuba	2000	Plantains	117
49	Cuba	2000	Potatoes	91
49	Cuba	2000	Poultry Meat	30
49	Cuba	2000	Pulses, Other	22
49	Cuba	2000	Rice (Milled Equivalent)	163
49	Cuba	2000	Roots, Other	14
49	Cuba	2000	Soyabean Oil	10
49	Cuba	2000	Soyabeans	1
49	Cuba	2000	Sugar, Refined Equivalent	164
49	Cuba	2000	Sunflowerseed Oil	8
49	Cuba	2000	Sweet Potatoes	54
49	Cuba	2000	Sweeteners, Other	1
49	Cuba	2000	Tomatoes	122
49	Cuba	2000	Vegetables, Other	210
49	Cuba	2000	Wheat	154
49	Cuba	2000	Whey	1
49	Cuba	2000	Wine	5
49	Cuba	2000	Yams	5
49	Cuba	2001	Apples	1
49	Cuba	2001	Bananas	58
49	Cuba	2001	Beans	48
49	Cuba	2001	Beer	55
49	Cuba	2001	Beverages, Alcoholic	14
49	Cuba	2001	Bovine Meat	19
49	Cuba	2001	Butter, Ghee	1
49	Cuba	2001	Cassava	68
49	Cuba	2001	Cheese	4
49	Cuba	2001	Cocoa Beans	1
49	Cuba	2001	Coconuts - Incl Copra	20
49	Cuba	2001	Coffee	3
49	Cuba	2001	Demersal Fish	2
49	Cuba	2001	Eggs	15
49	Cuba	2001	Fats, Animals, Raw	2
49	Cuba	2001	Freshwater Fish	6
49	Cuba	2001	Fruits, Other	130
49	Cuba	2001	Grapefruit	31
49	Cuba	2001	Grapes	1
49	Cuba	2001	Groundnuts (Shelled Eq)	1
49	Cuba	2001	Lemons, Limes	4
49	Cuba	2001	Maize	39
49	Cuba	2001	Marine Fish, Other	7
49	Cuba	2001	Milk, Whole	162
49	Cuba	2001	Molluscs, Other	3
49	Cuba	2001	Mutton & Goat Meat	2
49	Cuba	2001	Offals, Edible	6
49	Cuba	2001	Oilcrops Oil, Other	1
49	Cuba	2001	Onions	20
49	Cuba	2001	Oranges, Mandarines	94
49	Cuba	2001	Pelagic Fish	9
49	Cuba	2001	Pigmeat	26
49	Cuba	2001	Pineapples	7
49	Cuba	2001	Plantains	131
49	Cuba	2001	Potatoes	84
49	Cuba	2001	Poultry Meat	33
49	Cuba	2001	Pulses, Other	16
49	Cuba	2001	Rice (Milled Equivalent)	163
49	Cuba	2001	Roots, Other	15
49	Cuba	2001	Soyabean Oil	15
49	Cuba	2001	Soyabeans	1
49	Cuba	2001	Sugar, Refined Equivalent	156
49	Cuba	2001	Sunflowerseed Oil	2
49	Cuba	2001	Sweet Potatoes	64
49	Cuba	2001	Sweeteners, Other	1
49	Cuba	2001	Tomatoes	101
49	Cuba	2001	Vegetables, Other	265
49	Cuba	2001	Wheat	159
49	Cuba	2001	Wine	4
49	Cuba	2001	Yams	7
49	Cuba	2002	Apples	1
49	Cuba	2002	Bananas	38
49	Cuba	2002	Beans	28

FAO countrycode	Country	Reference year	Food item	Gross food supply (g $capita^{-1}$ d^{-1})
49	Cuba	2002	Beer	58
49	Cuba	2002	Beverages, Alcoholic	15
49	Cuba	2002	Bovine Meat	17
49	Cuba	2002	Butter, Ghee	1
49	Cuba	2002	Cassava	57
49	Cuba	2002	Cephalopods	2
49	Cuba	2002	Cheese	4
49	Cuba	2002	Coconuts - Incl Copra	24
49	Cuba	2002	Coffee	3
49	Cuba	2002	Demersal Fish	3
49	Cuba	2002	Eggs	18
49	Cuba	2002	Fats, Animals, Raw	2
49	Cuba	2002	Freshwater Fish	6
49	Cuba	2002	Fruits, Other	137
49	Cuba	2002	Grapefruit	17
49	Cuba	2002	Grapes	1
49	Cuba	2002	Groundnut Oil	1
49	Cuba	2002	Groundnuts (Shelled Eq)	2
49	Cuba	2002	Lemons, Limes	4
49	Cuba	2002	Maize	50
49	Cuba	2002	Marine Fish, Other	5
49	Cuba	2002	Milk, Whole	143
49	Cuba	2002	Molluscs, Other	1
49	Cuba	2002	Mutton & Goat Meat	2
49	Cuba	2002	Offals, Edible	5
49	Cuba	2002	Onions	21
49	Cuba	2002	Oranges, Mandarines	51
49	Cuba	2002	Pelagic Fish	1
49	Cuba	2002	Pigmeat	28
49	Cuba	2002	Pineapples	8
49	Cuba	2002	Plantains	105
49	Cuba	2002	Potatoes	81
49	Cuba	2002	Poultry Meat	38
49	Cuba	2002	Pulses, Other	14
49	Cuba	2002	Rice (Milled Equivalent)	210
49	Cuba	2002	Roots, Other	31
49	Cuba	2002	Soyabean Oil	18
49	Cuba	2002	Sugar, Refined Equivalent	143
49	Cuba	2002	Sweet Potatoes	72
49	Cuba	2002	Tomatoes	109
49	Cuba	2002	Vegetables, Other	262
49	Cuba	2002	Wheat	161
49	Cuba	2002	Wine	3
49	Cuba	2002	Yams	22
49	Cuba	2003	Apples	1
49	Cuba	2003	Bananas	57
49	Cuba	2003	Beans	62
49	Cuba	2003	Beer	57
49	Cuba	2003	Beverages, Alcoholic	14
49	Cuba	2003	Bovine Meat	15
49	Cuba	2003	Butter, Ghee	1
49	Cuba	2003	Cassava	117
49	Cuba	2003	Cephalopods	2
49	Cuba	2003	Cheese	4
49	Cuba	2003	Citrus, Other	1
49	Cuba	2003	Cocoa Beans	1
49	Cuba	2003	Coconuts - Incl Copra	24
49	Cuba	2003	Coffee	3
49	Cuba	2003	Demersal Fish	2
49	Cuba	2003	Eggs	18
49	Cuba	2003	Fats, Animals, Raw	2
49	Cuba	2003	Freshwater Fish	7
49	Cuba	2003	Fruits, Other	154
49	Cuba	2003	Grapefruit	32
49	Cuba	2003	Grapes	1
49	Cuba	2003	Groundnuts (Shelled Eq)	3
49	Cuba	2003	Lemons, Limes	6
49	Cuba	2003	Maize	66
49	Cuba	2003	Marine Fish, Other	9
49	Cuba	2003	Milk, Whole	145
49	Cuba	2003	Molluscs, Other	1
49	Cuba	2003	Mutton & Goat Meat	2
49	Cuba	2003	Offals, Edible	4
49	Cuba	2003	Onions	23
49	Cuba	2003	Oranges, Mandarines	84
49	Cuba	2003	Pelagic Fish	2
49	Cuba	2003	Pigmeat	28
49	Cuba	2003	Pineapples	9
49	Cuba	2003	Plantains	159
49	Cuba	2003	Potatoes	77
49	Cuba	2003	Poultry Meat	38
49	Cuba	2003	Pulses, Other	5
49	Cuba	2003	Rice (Milled Equivalent)	163
49	Cuba	2003	Roots, Other	29
49	Cuba	2003	Soyabean Oil	14
49	Cuba	2003	Sugar, Refined Equivalent	136
49	Cuba	2003	Sweet Potatoes	92
49	Cuba	2003	Sweeteners, Other	1
49	Cuba	2003	Tomatoes	142
49	Cuba	2003	Vegetables, Other	307
49	Cuba	2003	Wheat	170
49	Cuba	2003	Wine	4
49	Cuba	2003	Yams	16
49	Cuba	2004	Apples	1
49	Cuba	2004	Bananas	82
49	Cuba	2004	Beans	53
49	Cuba	2004	Beer	55

FAO country code	Country	Reference year	Food item	Gross food supply (g *capita*$^{-1}$ d^{-1})
49	Cuba	2004	Beverages, Alcoholic	13
49	Cuba	2004	Bovine Meat	16
49	Cuba	2004	Butter, Ghee	1
49	Cuba	2004	Cassava	77
49	Cuba	2004	Cheese	4
49	Cuba	2004	Citrus, Other	1
49	Cuba	2004	Cocoa Beans	1
49	Cuba	2004	Coconuts - Incl Copra	26
49	Cuba	2004	Coffee	4
49	Cuba	2004	Demersal Fish	2
49	Cuba	2004	Eggs	18
49	Cuba	2004	Fats, Animals, Raw	2
49	Cuba	2004	Freshwater Fish	7
49	Cuba	2004	Fruits, Other	157
49	Cuba	2004	Grapefruit	27
49	Cuba	2004	Grapes	1
49	Cuba	2004	Groundnuts (Shelled Eq)	3
49	Cuba	2004	Lemons, Limes	5
49	Cuba	2004	Maize	74
49	Cuba	2004	Marine Fish, Other	15
49	Cuba	2004	Milk, Whole	133
49	Cuba	2004	Molluscs, Other	1
49	Cuba	2004	Mutton & Goat Meat	2
49	Cuba	2004	Offals, Edible	4
49	Cuba	2004	Oilcrops Oil, Other	1
49	Cuba	2004	Onions	32
49	Cuba	2004	Oranges, Mandarines	69
49	Cuba	2004	Pelagic Fish	2
49	Cuba	2004	Pigmeat	31
49	Cuba	2004	Pineapples	10
49	Cuba	2004	Plantains	152
49	Cuba	2004	Potatoes	81
49	Cuba	2004	Poultry Meat	44
49	Cuba	2004	Pulses, Other	10
49	Cuba	2004	Rice (Milled Equivalent)	183
49	Cuba	2004	Roots, Other	19
49	Cuba	2004	Soyabean Oil	21
49	Cuba	2004	Sugar, Refined Equivalent	155
49	Cuba	2004	Sweet Potatoes	88
49	Cuba	2004	Sweeteners, Other	1
49	Cuba	2004	Tomatoes	173
49	Cuba	2004	Vegetables, Other	227
49	Cuba	2004	Wheat	140
49	Cuba	2004	Wine	3
49	Cuba	2004	Yams	13
49	Cuba	2005	Apples	1
49	Cuba	2005	Bananas	57
49	Cuba	2005	Beans	49
49	Cuba	2005	Beer	55
49	Cuba	2005	Beverages, Alcoholic	16
49	Cuba	2005	Bovine Meat	18
49	Cuba	2005	Butter, Ghee	1
49	Cuba	2005	Cassava	70
49	Cuba	2005	Cheese	4
49	Cuba	2005	Citrus, Other	1
49	Cuba	2005	Cocoa Beans	1
49	Cuba	2005	Coconuts - Incl Copra	25
49	Cuba	2005	Coffee	6
49	Cuba	2005	Demersal Fish	1
49	Cuba	2005	Eggs	21
49	Cuba	2005	Fats, Animals, Raw	1
49	Cuba	2005	Freshwater Fish	5
49	Cuba	2005	Fruits, Other	137
49	Cuba	2005	Grapefruit	20
49	Cuba	2005	Grapes	1
49	Cuba	2005	Groundnuts (Shelled Eq)	3
49	Cuba	2005	Lemons, Limes	2
49	Cuba	2005	Maize	84
49	Cuba	2005	Marine Fish, Other	13
49	Cuba	2005	Milk, Whole	149
49	Cuba	2005	Molluscs, Other	1
49	Cuba	2005	Mutton & Goat Meat	2
49	Cuba	2005	Offals, Edible	5
49	Cuba	2005	Oilcrops Oil, Other	1
49	Cuba	2005	Onions	29
49	Cuba	2005	Oranges, Mandarines	48
49	Cuba	2005	Pelagic Fish	3
49	Cuba	2005	Pigmeat	30
49	Cuba	2005	Pineapples	9
49	Cuba	2005	Plantains	107
49	Cuba	2005	Potatoes	78
49	Cuba	2005	Poultry Meat	32
49	Cuba	2005	Pulses, Other	17
49	Cuba	2005	Rice (Milled Equivalent)	180
49	Cuba	2005	Roots, Other	19
49	Cuba	2005	Soyabean Oil	23
49	Cuba	2005	Soyabeans	5
49	Cuba	2005	Sugar, Refined Equivalent	136
49	Cuba	2005	Sunflowerseed Oil	2
49	Cuba	2005	Sweet Potatoes	82
49	Cuba	2005	Tomatoes	177
49	Cuba	2005	Vegetables, Other	243
49	Cuba	2005	Wheat	136
49	Cuba	2005	Wine	3
49	Cuba	2005	Yams	12
49	Cuba	2006	Apples	1
49	Cuba	2006	Bananas	74

FAO country code	Country	Reference year	Food item	Gross food supply (g *capita*$^{-1}$ d^{-1})
49	Cuba	2006	Beans	49
49	Cuba	2006	Beer	56
49	Cuba	2006	Beverages, Alcoholic	16
49	Cuba	2006	Bovine Meat	19
49	Cuba	2006	Butter, Ghee	1
49	Cuba	2006	Cassava	73
49	Cuba	2006	Cereals, Other	1
49	Cuba	2006	Cheese	4
49	Cuba	2006	Citrus, Other	1
49	Cuba	2006	Coconuts - Incl Copra	23
49	Cuba	2006	Coffee	4
49	Cuba	2006	Demersal Fish	3
49	Cuba	2006	Eggs	23
49	Cuba	2006	Fats, Animals, Raw	1
49	Cuba	2006	Freshwater Fish	6
49	Cuba	2006	Fruits, Other	132
49	Cuba	2006	Grapefruit	31
49	Cuba	2006	Grapes	1
49	Cuba	2006	Groundnuts (Shelled Eq)	2
49	Cuba	2006	Honey	1
49	Cuba	2006	Lemons, Limes	1
49	Cuba	2006	Maize	73
49	Cuba	2006	Marine Fish, Other	7
49	Cuba	2006	Milk, Whole	149
49	Cuba	2006	Molluscs, Other	1
49	Cuba	2006	Mutton & Goat Meat	2
49	Cuba	2006	Offals, Edible	5
49	Cuba	2006	Onions	25
49	Cuba	2006	Oranges, Mandarines	37
49	Cuba	2006	Pelagic Fish	3
49	Cuba	2006	Pigmeat	33
49	Cuba	2006	Pineapples	10
49	Cuba	2006	Plantains	125
49	Cuba	2006	Potatoes	66
49	Cuba	2006	Poultry Meat	48
49	Cuba	2006	Pulses, Other	17
49	Cuba	2006	Rice (Milled Equivalent)	180
49	Cuba	2006	Roots, Other	31
49	Cuba	2006	Soyabean Oil	23
49	Cuba	2006	Soyabeans	7
49	Cuba	2006	Sugar, Refined Equivalent	128
49	Cuba	2006	Sunflowerseed Oil	1
49	Cuba	2006	Sweet Potatoes	66
49	Cuba	2006	Tomatoes	152
49	Cuba	2006	Vegetables, Other	257
49	Cuba	2006	Wheat	153
49	Cuba	2006	Whey	1
49	Cuba	2006	Wine	3
49	Cuba	2006	Yams	22
49	Cuba	2007	Apples	2
49	Cuba	2007	Bananas	84
49	Cuba	2007	Beans	54
49	Cuba	2007	Beer	60
49	Cuba	2007	Beverages, Alcoholic	18
49	Cuba	2007	Bovine Meat	16
49	Cuba	2007	Butter, Ghee	1
49	Cuba	2007	Cassava	54
49	Cuba	2007	Cephalopods	3
49	Cuba	2007	Cheese	5
49	Cuba	2007	Citrus, Other	1
49	Cuba	2007	Coconuts - Incl Copra	27
49	Cuba	2007	Coffee	7
49	Cuba	2007	Demersal Fish	2
49	Cuba	2007	Eggs	24
49	Cuba	2007	Fats, Animals, Raw	2
49	Cuba	2007	Freshwater Fish	9
49	Cuba	2007	Fruits, Other	123
49	Cuba	2007	Grapefruit	26
49	Cuba	2007	Grapes	1
49	Cuba	2007	Groundnuts (Shelled Eq)	1
49	Cuba	2007	Lemons, Limes	1
49	Cuba	2007	Maize	88
49	Cuba	2007	Marine Fish, Other	8
49	Cuba	2007	Milk, Whole	147
49	Cuba	2007	Molluscs, Other	1
49	Cuba	2007	Mutton & Goat Meat	3
49	Cuba	2007	Offals, Edible	5
49	Cuba	2007	Onions	23
49	Cuba	2007	Oranges, Mandarines	46
49	Cuba	2007	Pelagic Fish	2
49	Cuba	2007	Pigmeat	53
49	Cuba	2007	Pineapples	12
49	Cuba	2007	Plantains	142
49	Cuba	2007	Potatoes	33
49	Cuba	2007	Poultry Meat	41
49	Cuba	2007	Pulses, Other	17
49	Cuba	2007	Rice (Milled Equivalent)	174
49	Cuba	2007	Roots, Other	22
49	Cuba	2007	Soyabean Oil	17
49	Cuba	2007	Soyabeans	3
49	Cuba	2007	Sugar, Refined Equivalent	126
49	Cuba	2007	Sunflowerseed Oil	2
49	Cuba	2007	Sweet Potatoes	79
49	Cuba	2007	Tomatoes	150
49	Cuba	2007	Vegetables, Other	247
49	Cuba	2007	Wheat	138
49	Cuba	2007	Whey	1

FAO countrycode	Country	Reference year	Food item	Gross food supply (g $capita^{-1}$ d^{-1})
49	Cuba	2007	Wine	4
49	Cuba	2007	Yams	27
49	Cuba	2008	Apples	1
49	Cuba	2008	Bananas	55
49	Cuba	2008	Beans	34
49	Cuba	2008	Beer	62
49	Cuba	2008	Beverages, Alcoholic	22
49	Cuba	2008	Bovine Meat	19
49	Cuba	2008	Butter, Ghee	1
49	Cuba	2008	Cassava	57
49	Cuba	2008	Cephalopods	3
49	Cuba	2008	Cereals, Other	4
49	Cuba	2008	Cheese	4
49	Cuba	2008	Citrus, Other	1
49	Cuba	2008	Cocoa Beans	1
49	Cuba	2008	Coconuts - Incl Copra	23
49	Cuba	2008	Coffee	9
49	Cuba	2008	Demersal Fish	2
49	Cuba	2008	Eggs	24
49	Cuba	2008	Fats, Animals, Raw	2
49	Cuba	2008	Freshwater Fish	9
49	Cuba	2008	Fruits, Other	126
49	Cuba	2008	Grapefruit	28
49	Cuba	2008	Grapes	1
49	Cuba	2008	Groundnuts (Shelled Eq)	1
49	Cuba	2008	Lemons, Limes	1
49	Cuba	2008	Maize	51
49	Cuba	2008	Maize Germ Oil	1
49	Cuba	2008	Marine Fish, Other	7
49	Cuba	2008	Meat, Other	15
49	Cuba	2008	Milk, Whole	212
49	Cuba	2008	Molluscs, Other	1
49	Cuba	2008	Mutton & Goat Meat	3
49	Cuba	2008	Offals, Edible	5
49	Cuba	2008	Onions	28
49	Cuba	2008	Oranges, Mandarines	43
49	Cuba	2008	Pelagic Fish	4
49	Cuba	2008	Pigmeat	56
49	Cuba	2008	Pineapples	12
49	Cuba	2008	Plantains	115
49	Cuba	2008	Potatoes	46
49	Cuba	2008	Poultry Meat	43
49	Cuba	2008	Pulses, Other	36
49	Cuba	2008	Rice (Milled Equivalent)	167
49	Cuba	2008	Roots, Other	38
49	Cuba	2008	Soyabean Oil	8
49	Cuba	2008	Soyabeans	17
49	Cuba	2008	Sugar, Refined Equivalent	112
49	Cuba	2008	Sweet Potatoes	62
49	Cuba	2008	Sweeteners, Other	1
49	Cuba	2008	Tomatoes	128
49	Cuba	2008	Vegetables, Other	250
49	Cuba	2008	Wheat	154
49	Cuba	2008	Whey	1
49	Cuba	2008	Wine	4
49	Cuba	2008	Yams	40
49	Cuba	2009	Apples	1
49	Cuba	2009	Bananas	48
49	Cuba	2009	Beans	35
49	Cuba	2009	Beer	60
49	Cuba	2009	Beverages, Alcoholic	19
49	Cuba	2009	Bovine Meat	18
49	Cuba	2009	Butter, Ghee	1
49	Cuba	2009	Cassava	53
49	Cuba	2009	Cephalopods	2
49	Cuba	2009	Cheese	4
49	Cuba	2009	Citrus, Other	1
49	Cuba	2009	Cocoa Beans	1
49	Cuba	2009	Coconuts - Incl Copra	17
49	Cuba	2009	Coffee	6
49	Cuba	2009	Demersal Fish	2
49	Cuba	2009	Eggs	24
49	Cuba	2009	Fats, Animals, Raw	2
49	Cuba	2009	Freshwater Fish	8
49	Cuba	2009	Fruits, Other	130
49	Cuba	2009	Grapefruit	14
49	Cuba	2009	Grapes	1
49	Cuba	2009	Groundnuts (Shelled Eq)	1
49	Cuba	2009	Lemons, Limes	2
49	Cuba	2009	Maize	49
49	Cuba	2009	Marine Fish, Other	3
49	Cuba	2009	Meat, Other	18
49	Cuba	2009	Milk, Whole	212
49	Cuba	2009	Molluscs, Other	1
49	Cuba	2009	Mutton & Goat Meat	3
49	Cuba	2009	Offals, Edible	5
49	Cuba	2009	Onions	29
49	Cuba	2009	Oranges, Mandarines	60
49	Cuba	2009	Pelagic Fish	6
49	Cuba	2009	Pigmeat	54
49	Cuba	2009	Pineapples	16
49	Cuba	2009	Plantains	102
49	Cuba	2009	Potatoes	68
49	Cuba	2009	Poultry Meat	54
49	Cuba	2009	Pulses, Other	25
49	Cuba	2009	Rice (Milled Equivalent)	170
49	Cuba	2009	Roots, Other	31

FAO country code	Country	Reference year	Food item	Gross food supply (g *capita*$^{-1}$ d^{-1})
49	Cuba	2009	Soyabean Oil	15
49	Cuba	2009	Soyabeans	16
49	Cuba	2009	Sugar, Refined Equivalent	148
49	Cuba	2009	Sweet Potatoes	73
49	Cuba	2009	Tomatoes	164
49	Cuba	2009	Vegetables, Other	236
49	Cuba	2009	Wheat	135
49	Cuba	2009	Whey	2
49	Cuba	2009	Wine	4
49	Cuba	2009	Yams	56
49	Cuba	2010	Apples	1
49	Cuba	2010	Bananas	48
49	Cuba	2010	Beans	33
49	Cuba	2010	Beer	63
49	Cuba	2010	Beverages, Alcoholic	21
49	Cuba	2010	Bovine Meat	18
49	Cuba	2010	Butter, Ghee	1
49	Cuba	2010	Cassava	53
49	Cuba	2010	Cheese	4
49	Cuba	2010	Citrus, Other	1
49	Cuba	2010	Cocoa Beans	2
49	Cuba	2010	Coconuts - Incl Copra	16
49	Cuba	2010	Coffee	6
49	Cuba	2010	Crustaceans	1
49	Cuba	2010	Demersal Fish	2
49	Cuba	2010	Eggs	24
49	Cuba	2010	Fats, Animals, Raw	2
49	Cuba	2010	Freshwater Fish	7
49	Cuba	2010	Fruits, Other	152
49	Cuba	2010	Grapefruit	20
49	Cuba	2010	Grapes	1
49	Cuba	2010	Groundnuts (Shelled Eq)	1
49	Cuba	2010	Lemons, Limes	1
49	Cuba	2010	Maize	54
49	Cuba	2010	Marine Fish, Other	3
49	Cuba	2010	Meat, Other	18
49	Cuba	2010	Milk, Whole	197
49	Cuba	2010	Molluscs, Other	1
49	Cuba	2010	Mutton & Goat Meat	3
49	Cuba	2010	Offals, Edible	5
49	Cuba	2010	Onions	25
49	Cuba	2010	Oranges, Mandarines	39
49	Cuba	2010	Pelagic Fish	2
49	Cuba	2010	Pigmeat	55
49	Cuba	2010	Pineapples	14
49	Cuba	2010	Plantains	117
49	Cuba	2010	Potatoes	47
49	Cuba	2010	Poultry Meat	44
49	Cuba	2010	Pulses, Other	6
49	Cuba	2010	Rice (Milled Equivalent)	153
49	Cuba	2010	Roots, Other	25
49	Cuba	2010	Soyabean Oil	17
49	Cuba	2010	Soyabeans	14
49	Cuba	2010	Sugar, Refined Equivalent	151
49	Cuba	2010	Sweet Potatoes	73
49	Cuba	2010	Sweeteners, Other	1
49	Cuba	2010	Tomatoes	115
49	Cuba	2010	Vegetables, Other	204
49	Cuba	2010	Wheat	150
49	Cuba	2010	Wine	4
49	Cuba	2010	Yams	67
49	Cuba	2011	Apples	1
49	Cuba	2011	Bananas	49
49	Cuba	2011	Beans	43
49	Cuba	2011	Beer	63
49	Cuba	2011	Beverages, Alcoholic	21
49	Cuba	2011	Bovine Meat	17
49	Cuba	2011	Butter, Ghee	1
49	Cuba	2011	Cassava	64
49	Cuba	2011	Cheese	4
49	Cuba	2011	Citrus, Other	1
49	Cuba	2011	Cocoa Beans	1
49	Cuba	2011	Coconuts - Incl Copra	14
49	Cuba	2011	Coffee	4
49	Cuba	2011	Crustaceans	1
49	Cuba	2011	Demersal Fish	2
49	Cuba	2011	Eggs	26
49	Cuba	2011	Fats, Animals, Raw	2
49	Cuba	2011	Freshwater Fish	7
49	Cuba	2011	Fruits, Other	169
49	Cuba	2011	Grapefruit	16
49	Cuba	2011	Grapes	1
49	Cuba	2011	Lemons, Limes	2
49	Cuba	2011	Maize	65
49	Cuba	2011	Marine Fish, Other	3
49	Cuba	2011	Meat, Other	16
49	Cuba	2011	Milk, Whole	209
49	Cuba	2011	Molluscs, Other	1
49	Cuba	2011	Mutton & Goat Meat	3
49	Cuba	2011	Offals, Edible	5
49	Cuba	2011	Onions	31
49	Cuba	2011	Oranges, Mandarines	31
49	Cuba	2011	Pelagic Fish	2
49	Cuba	2011	Pigmeat	52
49	Cuba	2011	Pineapples	17
49	Cuba	2011	Plantains	141
49	Cuba	2011	Potatoes	41

FAO countrycode	Country	Reference year	Food item	Gross food supply (g *capita*$^{-1}$ d^{-1})
49	Cuba	2011	Poultry Meat	47
49	Cuba	2011	Pulses, Other	8
49	Cuba	2011	Rice (Milled Equivalent)	174
49	Cuba	2011	Roots, Other	24
49	Cuba	2011	Soyabean Oil	19
49	Cuba	2011	Soyabeans	15
49	Cuba	2011	Sugar, Refined Equivalent	141
49	Cuba	2011	Sweet Potatoes	60
49	Cuba	2011	Tomatoes	132
49	Cuba	2011	Vegetables, Other	192
49	Cuba	2011	Wheat	142
49	Cuba	2011	Wine	4
49	Cuba	2011	Yams	58
50	Cyprus	2004	Apples	35
50	Cyprus	2004	Bananas	22
50	Cyprus	2004	Beans	5
50	Cyprus	2004	Beer	97
50	Cyprus	2004	Beverages, Alcoholic	8
50	Cyprus	2004	Beverages, Fermented	1
50	Cyprus	2004	Bovine Meat	13
50	Cyprus	2004	Butter, Ghee	2
50	Cyprus	2004	Cephalopods	8
50	Cyprus	2004	Cheese	16
50	Cyprus	2004	Citrus, Other	1
50	Cyprus	2004	Cocoa Beans	6
50	Cyprus	2004	Coconuts - Incl Copra	2
50	Cyprus	2004	Coffee	11
50	Cyprus	2004	Cream	1
50	Cyprus	2004	Crustaceans	4
50	Cyprus	2004	Dates	1
50	Cyprus	2004	Demersal Fish	16
50	Cyprus	2004	Eggs	25
50	Cyprus	2004	Fats, Animals, Raw	3
50	Cyprus	2004	Freshwater Fish	3
50	Cyprus	2004	Fruits, Other	51
50	Cyprus	2004	Grapefruit	19
50	Cyprus	2004	Grapes	15
50	Cyprus	2004	Groundnuts (Shelled Eq)	4
50	Cyprus	2004	Honey	2
50	Cyprus	2004	Lemons, Limes	19
50	Cyprus	2004	Maize	3
50	Cyprus	2004	Maize Germ Oil	4
50	Cyprus	2004	Marine Fish, Other	3
50	Cyprus	2004	Meat, Other	6
50	Cyprus	2004	Milk, Whole	312
50	Cyprus	2004	Molluscs, Other	1
50	Cyprus	2004	Mutton & Goat Meat	21
50	Cyprus	2004	Nuts	11
50	Cyprus	2004	Oats	1
50	Cyprus	2004	Offals, Edible	10
50	Cyprus	2004	Oilcrops Oil, Other	2
50	Cyprus	2004	Olive Oil	6
50	Cyprus	2004	Olives	22
50	Cyprus	2004	Onions	17
50	Cyprus	2004	Oranges, Mandarines	97
50	Cyprus	2004	Pelagic Fish	14
50	Cyprus	2004	Pigmeat	104
50	Cyprus	2004	Pineapples	1
50	Cyprus	2004	Potatoes	101
50	Cyprus	2004	Poultry Meat	78
50	Cyprus	2004	Pulses, Other	5
50	Cyprus	2004	Rape and Mustard Oil	4
50	Cyprus	2004	Rice (Milled Equivalent)	10
50	Cyprus	2004	Roots, Other	4
50	Cyprus	2004	Sesameseed	4
50	Cyprus	2004	Soyabean Oil	9
50	Cyprus	2004	Spices, Other	1
50	Cyprus	2004	Sugar, Refined Equivalent	91
50	Cyprus	2004	Sunflowerseed Oil	6
50	Cyprus	2004	Sweeteners, Other	13
50	Cyprus	2004	Tea	1
50	Cyprus	2004	Tomatoes	95
50	Cyprus	2004	Vegetables, Other	228
50	Cyprus	2004	Wheat	184
50	Cyprus	2004	Wine	45
50	Cyprus	2005	Apples	43
50	Cyprus	2005	Bananas	22
50	Cyprus	2005	Beans	5
50	Cyprus	2005	Beer	96
50	Cyprus	2005	Beverages, Alcoholic	7
50	Cyprus	2005	Beverages, Fermented	1
50	Cyprus	2005	Bovine Meat	15
50	Cyprus	2005	Butter, Ghee	2
50	Cyprus	2005	Cephalopods	7
50	Cyprus	2005	Cheese	15
50	Cyprus	2005	Citrus, Other	1
50	Cyprus	2005	Cocoa Beans	6
50	Cyprus	2005	Coconuts - Incl Copra	2
50	Cyprus	2005	Coffee	12
50	Cyprus	2005	Cream	1
50	Cyprus	2005	Crustaceans	4
50	Cyprus	2005	Dates	1
50	Cyprus	2005	Demersal Fish	17
50	Cyprus	2005	Eggs	22
50	Cyprus	2005	Fats, Animals, Raw	2
50	Cyprus	2005	Freshwater Fish	4
50	Cyprus	2005	Fruits, Other	55

FAO country code	Country	Reference year	Food item	Gross food supply (g $capita^{-1}$ d^{-1})
50	Cyprus	2005	Grapefruit	20
50	Cyprus	2005	Grapes	16
50	Cyprus	2005	Groundnuts (Shelled Eq)	4
50	Cyprus	2005	Honey	2
50	Cyprus	2005	Lemons, Limes	24
50	Cyprus	2005	Maize	3
50	Cyprus	2005	Maize Germ Oil	4
50	Cyprus	2005	Marine Fish, Other	3
50	Cyprus	2005	Meat, Other	4
50	Cyprus	2005	Milk, Whole	269
50	Cyprus	2005	Molluscs, Other	2
50	Cyprus	2005	Mutton & Goat Meat	19
50	Cyprus	2005	Nuts	10
50	Cyprus	2005	Oats	1
50	Cyprus	2005	Offals, Edible	9
50	Cyprus	2005	Oilcrops Oil, Other	3
50	Cyprus	2005	Olive Oil	4
50	Cyprus	2005	Olives	21
50	Cyprus	2005	Onions	24
50	Cyprus	2005	Oranges, Mandarines	85
50	Cyprus	2005	Pelagic Fish	16
50	Cyprus	2005	Pigmeat	111
50	Cyprus	2005	Pineapples	1
50	Cyprus	2005	Potatoes	99
50	Cyprus	2005	Poultry Meat	81
50	Cyprus	2005	Pulses, Other	6
50	Cyprus	2005	Rape and Mustard Oil	3
50	Cyprus	2005	Rice (Milled Equivalent)	12
50	Cyprus	2005	Roots, Other	4
50	Cyprus	2005	Sesameseed	5
50	Cyprus	2005	Soyabean Oil	12
50	Cyprus	2005	Spices, Other	1
50	Cyprus	2005	Sugar, Refined Equivalent	77
50	Cyprus	2005	Sunflowerseed Oil	12
50	Cyprus	2005	Sweeteners, Other	19
50	Cyprus	2005	Tea	1
50	Cyprus	2005	Tomatoes	96
50	Cyprus	2005	Vegetables, Other	234
50	Cyprus	2005	Wheat	170
50	Cyprus	2005	Wine	42
50	Cyprus	2006	Apples	44
50	Cyprus	2006	Bananas	22
50	Cyprus	2006	Beans	4
50	Cyprus	2006	Beer	88
50	Cyprus	2006	Beverages, Alcoholic	7
50	Cyprus	2006	Beverages, Fermented	1
50	Cyprus	2006	Bovine Meat	14
50	Cyprus	2006	Butter, Ghee	2
50	Cyprus	2006	Cephalopods	9
50	Cyprus	2006	Cheese	13
50	Cyprus	2006	Citrus, Other	1
50	Cyprus	2006	Cocoa Beans	6
50	Cyprus	2006	Coconuts - Incl Copra	2
50	Cyprus	2006	Coffee	9
50	Cyprus	2006	Cream	1
50	Cyprus	2006	Crustaceans	6
50	Cyprus	2006	Dates	1
50	Cyprus	2006	Demersal Fish	16
50	Cyprus	2006	Eggs	22
50	Cyprus	2006	Fats, Animals, Raw	2
50	Cyprus	2006	Freshwater Fish	5
50	Cyprus	2006	Fruits, Other	60
50	Cyprus	2006	Grapefruit	14
50	Cyprus	2006	Grapes	46
50	Cyprus	2006	Groundnuts (Shelled Eq)	4
50	Cyprus	2006	Honey	2
50	Cyprus	2006	Lemons, Limes	31
50	Cyprus	2006	Maize	5
50	Cyprus	2006	Maize Germ Oil	4
50	Cyprus	2006	Marine Fish, Other	2
50	Cyprus	2006	Meat, Other	5
50	Cyprus	2006	Milk, Whole	261
50	Cyprus	2006	Molluscs, Other	2
50	Cyprus	2006	Mutton & Goat Meat	22
50	Cyprus	2006	Nuts	14
50	Cyprus	2006	Oats	1
50	Cyprus	2006	Offals, Edible	9
50	Cyprus	2006	Oilcrops Oil, Other	3
50	Cyprus	2006	Olive Oil	5
50	Cyprus	2006	Olives	36
50	Cyprus	2006	Onions	23
50	Cyprus	2006	Oranges, Mandarines	69
50	Cyprus	2006	Pelagic Fish	14
50	Cyprus	2006	Pigmeat	115
50	Cyprus	2006	Pineapples	2
50	Cyprus	2006	Plantains	1
50	Cyprus	2006	Potatoes	95
50	Cyprus	2006	Poultry Meat	66
50	Cyprus	2006	Pulses, Other	5
50	Cyprus	2006	Rape and Mustard Oil	4
50	Cyprus	2006	Rice (Milled Equivalent)	14
50	Cyprus	2006	Roots, Other	4
50	Cyprus	2006	Sesameseed	5
50	Cyprus	2006	Soyabean Oil	11
50	Cyprus	2006	Spices, Other	1
50	Cyprus	2006	Sugar, Refined Equivalent	67
50	Cyprus	2006	Sunflowerseed	1

FAO countrycode	Country	Reference year	Food item	Gross food supply (g *capita*$^{-1}$ d^{-1})
50	Cyprus	2006	Sunflowerseed Oil	15
50	Cyprus	2006	Sweeteners, Other	16
50	Cyprus	2006	Tea	1
50	Cyprus	2006	Tomatoes	85
50	Cyprus	2006	Vegetables, Other	234
50	Cyprus	2006	Wheat	163
50	Cyprus	2006	Wine	34
50	Cyprus	2007	Apples	41
50	Cyprus	2007	Bananas	24
50	Cyprus	2007	Beans	5
50	Cyprus	2007	Beer	88
50	Cyprus	2007	Beverages, Alcoholic	7
50	Cyprus	2007	Beverages, Fermented	2
50	Cyprus	2007	Bovine Meat	16
50	Cyprus	2007	Butter, Ghee	2
50	Cyprus	2007	Cephalopods	9
50	Cyprus	2007	Cereals, Other	1
50	Cyprus	2007	Cheese	13
50	Cyprus	2007	Citrus, Other	1
50	Cyprus	2007	Cocoa Beans	6
50	Cyprus	2007	Coconuts - Incl Copra	2
50	Cyprus	2007	Coffee	11
50	Cyprus	2007	Cream	4
50	Cyprus	2007	Crustaceans	6
50	Cyprus	2007	Dates	2
50	Cyprus	2007	Demersal Fish	15
50	Cyprus	2007	Eggs	23
50	Cyprus	2007	Fats, Animals, Raw	2
50	Cyprus	2007	Freshwater Fish	7
50	Cyprus	2007	Fruits, Other	62
50	Cyprus	2007	Grapefruit	21
50	Cyprus	2007	Grapes	54
50	Cyprus	2007	Groundnuts (Shelled Eq)	4
50	Cyprus	2007	Honey	3
50	Cyprus	2007	Lemons, Limes	25
50	Cyprus	2007	Maize	3
50	Cyprus	2007	Maize Germ Oil	4
50	Cyprus	2007	Marine Fish, Other	3
50	Cyprus	2007	Meat, Other	7
50	Cyprus	2007	Milk, Whole	254
50	Cyprus	2007	Molluscs, Other	2
50	Cyprus	2007	Mutton & Goat Meat	21
50	Cyprus	2007	Nuts	14
50	Cyprus	2007	Oats	1
50	Cyprus	2007	Offals, Edible	10
50	Cyprus	2007	Oilcrops Oil, Other	3
50	Cyprus	2007	Olive Oil	4
50	Cyprus	2007	Olives	18
50	Cyprus	2007	Onions	22
50	Cyprus	2007	Oranges, Mandarines	69
50	Cyprus	2007	Pelagic Fish	17
50	Cyprus	2007	Pigmeat	111
50	Cyprus	2007	Pineapples	2
50	Cyprus	2007	Plantains	1
50	Cyprus	2007	Potatoes	99
50	Cyprus	2007	Poultry Meat	71
50	Cyprus	2007	Pulses, Other	5
50	Cyprus	2007	Rape and Mustard Oil	2
50	Cyprus	2007	Rice (Milled Equivalent)	15
50	Cyprus	2007	Roots, Other	4
50	Cyprus	2007	Rye	1
50	Cyprus	2007	Sesameseed	4
50	Cyprus	2007	Soyabean Oil	9
50	Cyprus	2007	Spices, Other	1
50	Cyprus	2007	Sugar, Refined Equivalent	70
50	Cyprus	2007	Sunflowerseed Oil	16
50	Cyprus	2007	Sweeteners, Other	22
50	Cyprus	2007	Tea	1
50	Cyprus	2007	Tomatoes	84
50	Cyprus	2007	Vegetables, Other	224
50	Cyprus	2007	Wheat	192
50	Cyprus	2007	Wine	42
50	Cyprus	2008	Apples	38
50	Cyprus	2008	Bananas	21
50	Cyprus	2008	Barley	1
50	Cyprus	2008	Beans	6
50	Cyprus	2008	Beer	88
50	Cyprus	2008	Beverages, Alcoholic	7
50	Cyprus	2008	Beverages, Fermented	2
50	Cyprus	2008	Bovine Meat	16
50	Cyprus	2008	Butter, Ghee	2
50	Cyprus	2008	Cephalopods	10
50	Cyprus	2008	Cereals, Other	1
50	Cyprus	2008	Cheese	15
50	Cyprus	2008	Citrus, Other	1
50	Cyprus	2008	Cocoa Beans	7
50	Cyprus	2008	Coconuts - Incl Copra	2
50	Cyprus	2008	Coffee	13
50	Cyprus	2008	Crustaceans	8
50	Cyprus	2008	Dates	2
50	Cyprus	2008	Demersal Fish	16
50	Cyprus	2008	Eggs	25
50	Cyprus	2008	Fats, Animals, Raw	1
50	Cyprus	2008	Freshwater Fish	9
50	Cyprus	2008	Fruits, Other	63
50	Cyprus	2008	Grapefruit	26
50	Cyprus	2008	Grapes	23

FAO countrycode	Country	Reference year	Food item	Gross food supply (g $capita^{-1}$ d^{-1})
50	Cyprus	2008	Groundnuts (Shelled Eq)	3
50	Cyprus	2008	Honey	2
50	Cyprus	2008	Lemons, Limes	29
50	Cyprus	2008	Maize	4
50	Cyprus	2008	Maize Germ Oil	2
50	Cyprus	2008	Marine Fish, Other	2
50	Cyprus	2008	Meat, Other	7
50	Cyprus	2008	Milk, Whole	264
50	Cyprus	2008	Molluscs, Other	2
50	Cyprus	2008	Mutton & Goat Meat	20
50	Cyprus	2008	Nuts	15
50	Cyprus	2008	Oats	1
50	Cyprus	2008	Offals, Edible	9
50	Cyprus	2008	Oilcrops Oil, Other	3
50	Cyprus	2008	Olive Oil	6
50	Cyprus	2008	Olives	19
50	Cyprus	2008	Onions	26
50	Cyprus	2008	Oranges, Mandarines	47
50	Cyprus	2008	Peas	1
50	Cyprus	2008	Pelagic Fish	14
50	Cyprus	2008	Pigmeat	102
50	Cyprus	2008	Pineapples	2
50	Cyprus	2008	Potatoes	88
50	Cyprus	2008	Poultry Meat	75
50	Cyprus	2008	Pulses, Other	6
50	Cyprus	2008	Rape and Mustard Oil	2
50	Cyprus	2008	Rice (Milled Equivalent)	18
50	Cyprus	2008	Roots, Other	4
50	Cyprus	2008	Sesameseed	4
50	Cyprus	2008	Soyabean Oil	12
50	Cyprus	2008	Spices, Other	1
50	Cyprus	2008	Sugar, Refined Equivalent	67
50	Cyprus	2008	Sunflowerseed Oil	16
50	Cyprus	2008	Sweeteners, Other	21
50	Cyprus	2008	Tea	2
50	Cyprus	2008	Tomatoes	72
50	Cyprus	2008	Vegetables, Other	198
50	Cyprus	2008	Wheat	198
50	Cyprus	2008	Wine	51
50	Cyprus	2009	Apples	42
50	Cyprus	2009	Bananas	24
50	Cyprus	2009	Beans	4
50	Cyprus	2009	Beer	88
50	Cyprus	2009	Beverages, Alcoholic	7
50	Cyprus	2009	Beverages, Fermented	1
50	Cyprus	2009	Bovine Meat	17
50	Cyprus	2009	Butter, Ghee	3
50	Cyprus	2009	Cephalopods	9
50	Cyprus	2009	Cereals, Other	1
50	Cyprus	2009	Cheese	15
50	Cyprus	2009	Citrus, Other	1
50	Cyprus	2009	Cocoa Beans	7
50	Cyprus	2009	Coconuts - Incl Copra	2
50	Cyprus	2009	Coffee	17
50	Cyprus	2009	Crustaceans	8
50	Cyprus	2009	Dates	1
50	Cyprus	2009	Demersal Fish	13
50	Cyprus	2009	Eggs	23
50	Cyprus	2009	Fats, Animals, Raw	2
50	Cyprus	2009	Freshwater Fish	12
50	Cyprus	2009	Fruits, Other	64
50	Cyprus	2009	Grapefruit	23
50	Cyprus	2009	Grapes	22
50	Cyprus	2009	Groundnuts (Shelled Eq)	3
50	Cyprus	2009	Honey	2
50	Cyprus	2009	Lemons, Limes	28
50	Cyprus	2009	Maize	4
50	Cyprus	2009	Maize Germ Oil	3
50	Cyprus	2009	Marine Fish, Other	2
50	Cyprus	2009	Meat, Other	5
50	Cyprus	2009	Milk, Whole	237
50	Cyprus	2009	Molluscs, Other	2
50	Cyprus	2009	Mutton & Goat Meat	16
50	Cyprus	2009	Nuts	13
50	Cyprus	2009	Oats	1
50	Cyprus	2009	Offals, Edible	9
50	Cyprus	2009	Oilcrops Oil, Other	3
50	Cyprus	2009	Olive Oil	7
50	Cyprus	2009	Olives	12
50	Cyprus	2009	Onions	27
50	Cyprus	2009	Oranges, Mandarines	53
50	Cyprus	2009	Peas	1
50	Cyprus	2009	Pelagic Fish	14
50	Cyprus	2009	Pigmeat	105
50	Cyprus	2009	Pineapples	2
50	Cyprus	2009	Plantains	1
50	Cyprus	2009	Potatoes	83
50	Cyprus	2009	Poultry Meat	72
50	Cyprus	2009	Pulses, Other	5
50	Cyprus	2009	Rape and Mustard Oil	4
50	Cyprus	2009	Rice (Milled Equivalent)	12
50	Cyprus	2009	Roots, Other	3
50	Cyprus	2009	Sesameseed	5
50	Cyprus	2009	Soyabean Oil	12
50	Cyprus	2009	Spices, Other	1
50	Cyprus	2009	Sugar, Refined Equivalent	64
50	Cyprus	2009	Sunflowerseed Oil	17

FAO country code	Country	Reference year	Food item	Gross food supply (g $capita^{-1}$ d^{-1})
50	Cyprus	2009	Sweeteners, Other	19
50	Cyprus	2009	Tea	1
50	Cyprus	2009	Tomatoes	61
50	Cyprus	2009	Vegetables, Other	199
50	Cyprus	2009	Wheat	209
50	Cyprus	2009	Wine	41
50	Cyprus	2010	Apples	33
50	Cyprus	2010	Bananas	28
50	Cyprus	2010	Barley	1
50	Cyprus	2010	Beans	4
50	Cyprus	2010	Beer	88
50	Cyprus	2010	Beverages, Alcoholic	7
50	Cyprus	2010	Beverages, Fermented	2
50	Cyprus	2010	Bovine Meat	18
50	Cyprus	2010	Butter, Ghee	3
50	Cyprus	2010	Cephalopods	8
50	Cyprus	2010	Cheese	15
50	Cyprus	2010	Citrus, Other	1
50	Cyprus	2010	Cocoa Beans	8
50	Cyprus	2010	Coconuts - Incl Copra	1
50	Cyprus	2010	Coffee	15
50	Cyprus	2010	Crustaceans	8
50	Cyprus	2010	Dates	1
50	Cyprus	2010	Demersal Fish	13
50	Cyprus	2010	Eggs	21
50	Cyprus	2010	Fats, Animals, Raw	2
50	Cyprus	2010	Freshwater Fish	15
50	Cyprus	2010	Fruits, Other	55
50	Cyprus	2010	Grapefruit	13
50	Cyprus	2010	Grapes	25
50	Cyprus	2010	Groundnuts (Shelled Eq)	3
50	Cyprus	2010	Honey	2
50	Cyprus	2010	Lemons, Limes	26
50	Cyprus	2010	Maize	3
50	Cyprus	2010	Maize Germ Oil	2
50	Cyprus	2010	Marine Fish, Other	2
50	Cyprus	2010	Meat, Other	7
50	Cyprus	2010	Milk, Whole	247
50	Cyprus	2010	Molluscs, Other	2
50	Cyprus	2010	Mutton & Goat Meat	15
50	Cyprus	2010	Nuts	13
50	Cyprus	2010	Oats	1
50	Cyprus	2010	Offals, Edible	9
50	Cyprus	2010	Oilcrops Oil, Other	4
50	Cyprus	2010	Olive Oil	8
50	Cyprus	2010	Olives	11
50	Cyprus	2010	Onions	30
50	Cyprus	2010	Oranges, Mandarines	48
50	Cyprus	2010	Peas	1
50	Cyprus	2010	Pelagic Fish	12
50	Cyprus	2010	Pigmeat	103
50	Cyprus	2010	Pineapples	2
50	Cyprus	2010	Plantains	1
50	Cyprus	2010	Potatoes	84
50	Cyprus	2010	Poultry Meat	73
50	Cyprus	2010	Pulses, Other	6
50	Cyprus	2010	Rape and Mustard Oil	9
50	Cyprus	2010	Rice (Milled Equivalent)	16
50	Cyprus	2010	Roots, Other	4
50	Cyprus	2010	Sesameseed	4
50	Cyprus	2010	Soyabean Oil	9
50	Cyprus	2010	Spices, Other	1
50	Cyprus	2010	Sugar, Refined Equivalent	61
50	Cyprus	2010	Sunflowerseed	1
50	Cyprus	2010	Sunflowerseed Oil	15
50	Cyprus	2010	Sweeteners, Other	20
50	Cyprus	2010	Tea	1
50	Cyprus	2010	Tomatoes	61
50	Cyprus	2010	Vegetables, Other	204
50	Cyprus	2010	Wheat	211
50	Cyprus	2010	Wine	41
50	Cyprus	2011	Apples	37
50	Cyprus	2011	Aquatic Animals, Others	1
50	Cyprus	2011	Bananas	23
50	Cyprus	2011	Barley	1
50	Cyprus	2011	Beans	3
50	Cyprus	2011	Beer	88
50	Cyprus	2011	Beverages, Alcoholic	7
50	Cyprus	2011	Beverages, Fermented	3
50	Cyprus	2011	Bovine Meat	19
50	Cyprus	2011	Butter, Ghee	3
50	Cyprus	2011	Cephalopods	9
50	Cyprus	2011	Cereals, Other	2
50	Cyprus	2011	Cheese	10
50	Cyprus	2011	Citrus, Other	1
50	Cyprus	2011	Cocoa Beans	8
50	Cyprus	2011	Coconuts - Incl Copra	2
50	Cyprus	2011	Coffee	15
50	Cyprus	2011	Crustaceans	8
50	Cyprus	2011	Dates	1
50	Cyprus	2011	Demersal Fish	12
50	Cyprus	2011	Eggs	21
50	Cyprus	2011	Fats, Animals, Raw	2
50	Cyprus	2011	Freshwater Fish	16
50	Cyprus	2011	Fruits, Other	55
50	Cyprus	2011	Grapefruit	18
50	Cyprus	2011	Grapes	15

FAO country code	Country	Reference year	Food item	Gross food supply (g *capita*$^{-1}$ d^{-1})
50	Cyprus	2011	Groundnuts (Shelled Eq)	4
50	Cyprus	2011	Honey	2
50	Cyprus	2011	Lemons, Limes	28
50	Cyprus	2011	Maize	3
50	Cyprus	2011	Maize Germ Oil	2
50	Cyprus	2011	Marine Fish, Other	2
50	Cyprus	2011	Meat, Other	6
50	Cyprus	2011	Milk, Whole	258
50	Cyprus	2011	Molluscs, Other	2
50	Cyprus	2011	Mutton & Goat Meat	15
50	Cyprus	2011	Nuts	11
50	Cyprus	2011	Oats	1
50	Cyprus	2011	Offals, Edible	8
50	Cyprus	2011	Oilcrops Oil, Other	3
50	Cyprus	2011	Olive Oil	8
50	Cyprus	2011	Olives	11
50	Cyprus	2011	Onions	28
50	Cyprus	2011	Oranges, Mandarines	46
50	Cyprus	2011	Peas	1
50	Cyprus	2011	Pelagic Fish	12
50	Cyprus	2011	Pigmeat	105
50	Cyprus	2011	Pineapples	3
50	Cyprus	2011	Potatoes	65
50	Cyprus	2011	Poultry Meat	73
50	Cyprus	2011	Pulses, Other	5
50	Cyprus	2011	Rape and Mustard Oil	11
50	Cyprus	2011	Rice (Milled Equivalent)	14
50	Cyprus	2011	Roots, Other	5
50	Cyprus	2011	Sesameseed	3
50	Cyprus	2011	Soyabean Oil	9
50	Cyprus	2011	Spices, Other	1
50	Cyprus	2011	Sugar, Refined Equivalent	58
50	Cyprus	2011	Sunflowerseed Oil	17
50	Cyprus	2011	Sweeteners, Other	28
50	Cyprus	2011	Tea	1
50	Cyprus	2011	Tomatoes	51
50	Cyprus	2011	Vegetables, Other	203
50	Cyprus	2011	Wheat	212
50	Cyprus	2011	Wine	40
167	Czech Republic	1993	Apples	61
167	Czech Republic	1993	Bananas	24
167	Czech Republic	1993	Beer	408
167	Czech Republic	1993	Beverages, Alcoholic	15
167	Czech Republic	1993	Bovine Meat	56
167	Czech Republic	1993	Butter, Ghee	13
167	Czech Republic	1993	Cereals, Other	1
167	Czech Republic	1993	Cheese	28
167	Czech Republic	1993	Citrus, Other	2
167	Czech Republic	1993	Cocoa Beans	4
167	Czech Republic	1993	Coconuts - Incl Copra	1
167	Czech Republic	1993	Coffee	7
167	Czech Republic	1993	Cream	5
167	Czech Republic	1993	Eggs	35
167	Czech Republic	1993	Fats, Animals, Raw	12
167	Czech Republic	1993	Freshwater Fish	4
167	Czech Republic	1993	Fruits, Other	43
167	Czech Republic	1993	Grapefruit	2
167	Czech Republic	1993	Grapes	9
167	Czech Republic	1993	Groundnuts (Shelled Eq)	2
167	Czech Republic	1993	Honey	2
167	Czech Republic	1993	Lemons, Limes	5
167	Czech Republic	1993	Marine Fish, Other	7
167	Czech Republic	1993	Meat, Other	9
167	Czech Republic	1993	Milk, Whole	200
167	Czech Republic	1993	Mutton & Goat Meat	1
167	Czech Republic	1993	Nuts	3
167	Czech Republic	1993	Offals, Edible	14
167	Czech Republic	1993	Oilcrops Oil, Other	1
167	Czech Republic	1993	Onions	25
167	Czech Republic	1993	Oranges, Mandarines	34
167	Czech Republic	1993	Peas	2
167	Czech Republic	1993	Pelagic Fish	7
167	Czech Republic	1993	Pigmeat	160
167	Czech Republic	1993	Pimento	1
167	Czech Republic	1993	Pineapples	1
167	Czech Republic	1993	Potatoes	232
167	Czech Republic	1993	Poultry Meat	33
167	Czech Republic	1993	Pulses, Other	3
167	Czech Republic	1993	Rape and Mustard Oil	32
167	Czech Republic	1993	Rice (Milled Equivalent)	8
167	Czech Republic	1993	Rye	21
167	Czech Republic	1993	Soyabean Oil	3
167	Czech Republic	1993	Sugar, Refined Equivalent	95
167	Czech Republic	1993	Sunflowerseed Oil	5
167	Czech Republic	1993	Sweeteners, Other	7
167	Czech Republic	1993	Tea	1
167	Czech Republic	1993	Tomatoes	19
167	Czech Republic	1993	Vegetables, Other	150
167	Czech Republic	1993	Wheat	267
167	Czech Republic	1993	Wine	17
167	Czech Republic	1994	Apples	34
167	Czech Republic	1994	Bananas	29
167	Czech Republic	1994	Beer	437
167	Czech Republic	1994	Beverages, Alcoholic	16
167	Czech Republic	1994	Bovine Meat	46
167	Czech Republic	1994	Butter, Ghee	13
167	Czech Republic	1994	Cereals, Other	2

FAO country code	Country	Reference year	Food item	Gross food supply (g *capita*$^{-1}$ d^{-1})
167	Czech Republic	1994	Cheese	25
167	Czech Republic	1994	Citrus, Other	2
167	Czech Republic	1994	Cocoa Beans	5
167	Czech Republic	1994	Coconuts - Incl Copra	1
167	Czech Republic	1994	Coffee	8
167	Czech Republic	1994	Cream	5
167	Czech Republic	1994	Eggs	37
167	Czech Republic	1994	Fats, Animals, Raw	10
167	Czech Republic	1994	Freshwater Fish	3
167	Czech Republic	1994	Fruits, Other	49
167	Czech Republic	1994	Grapefruit	4
167	Czech Republic	1994	Grapes	12
167	Czech Republic	1994	Groundnuts (Shelled Eq)	1
167	Czech Republic	1994	Honey	1
167	Czech Republic	1994	Lemons, Limes	5
167	Czech Republic	1994	Marine Fish, Other	12
167	Czech Republic	1994	Meat, Other	9
167	Czech Republic	1994	Milk, Whole	169
167	Czech Republic	1994	Mutton & Goat Meat	1
167	Czech Republic	1994	Nuts	3
167	Czech Republic	1994	Oats	1
167	Czech Republic	1994	Offals, Edible	13
167	Czech Republic	1994	Oilcrops Oil, Other	1
167	Czech Republic	1994	Olives	1
167	Czech Republic	1994	Onions	26
167	Czech Republic	1994	Oranges, Mandarines	67
167	Czech Republic	1994	Peas	2
167	Czech Republic	1994	Pelagic Fish	7
167	Czech Republic	1994	Pigmeat	127
167	Czech Republic	1994	Pimento	1
167	Czech Republic	1994	Pineapples	2
167	Czech Republic	1994	Potatoes	217
167	Czech Republic	1994	Poultry Meat	32
167	Czech Republic	1994	Pulses, Other	2
167	Czech Republic	1994	Rape and Mustard Oil	23
167	Czech Republic	1994	Rice (Milled Equivalent)	9
167	Czech Republic	1994	Rye	45
167	Czech Republic	1994	Soyabean Oil	8
167	Czech Republic	1994	Sugar, Refined Equivalent	93
167	Czech Republic	1994	Sunflowerseed Oil	6
167	Czech Republic	1994	Sweeteners, Other	6
167	Czech Republic	1994	Tomatoes	19
167	Czech Republic	1994	Vegetables, Other	145
167	Czech Republic	1994	Wheat	278
167	Czech Republic	1994	Wine	17
167	Czech Republic	1995	Apples	40
167	Czech Republic	1995	Bananas	38
167	Czech Republic	1995	Beans	1
167	Czech Republic	1995	Beer	427
167	Czech Republic	1995	Beverages, Alcoholic	17
167	Czech Republic	1995	Bovine Meat	44
167	Czech Republic	1995	Butter, Ghee	12
167	Czech Republic	1995	Cereals, Other	2
167	Czech Republic	1995	Cheese	25
167	Czech Republic	1995	Cocoa Beans	4
167	Czech Republic	1995	Coconuts - Incl Copra	1
167	Czech Republic	1995	Coffee	8
167	Czech Republic	1995	Cream	6
167	Czech Republic	1995	Eggs	36
167	Czech Republic	1995	Fats, Animals, Raw	12
167	Czech Republic	1995	Freshwater Fish	4
167	Czech Republic	1995	Fruits, Other	47
167	Czech Republic	1995	Grapefruit	3
167	Czech Republic	1995	Grapes	8
167	Czech Republic	1995	Groundnut Oil	1
167	Czech Republic	1995	Honey	1
167	Czech Republic	1995	Lemons, Limes	3
167	Czech Republic	1995	Marine Fish, Other	12
167	Czech Republic	1995	Meat, Other	9
167	Czech Republic	1995	Milk, Whole	168
167	Czech Republic	1995	Mutton & Goat Meat	1
167	Czech Republic	1995	Nuts	3
167	Czech Republic	1995	Oats	1
167	Czech Republic	1995	Offals, Edible	14
167	Czech Republic	1995	Onions	30
167	Czech Republic	1995	Oranges, Mandarines	74
167	Czech Republic	1995	Peas	3
167	Czech Republic	1995	Pelagic Fish	6
167	Czech Republic	1995	Pigmeat	136
167	Czech Republic	1995	Pimento	1
167	Czech Republic	1995	Pineapples	2
167	Czech Republic	1995	Potatoes	219
167	Czech Republic	1995	Poultry Meat	41
167	Czech Republic	1995	Pulses, Other	2
167	Czech Republic	1995	Rape and Mustard Oil	33
167	Czech Republic	1995	Rice (Milled Equivalent)	12
167	Czech Republic	1995	Rye	73
167	Czech Republic	1995	Soyabean Oil	2
167	Czech Republic	1995	Soyabeans	1
167	Czech Republic	1995	Sugar, Refined Equivalent	107
167	Czech Republic	1995	Sunflowerseed Oil	4
167	Czech Republic	1995	Sweeteners, Other	10
167	Czech Republic	1995	Tea	1
167	Czech Republic	1995	Tomatoes	23
167	Czech Republic	1995	Vegetables, Other	149
167	Czech Republic	1995	Wheat	277
167	Czech Republic	1995	Wine	17

FAO country code	Country	Reference year	Food item	Gross food supply (g *capita*$^{-1}$ d^{-1})
167	Czech Republic	1996	Apples	49
167	Czech Republic	1996	Bananas	35
167	Czech Republic	1996	Beans	1
167	Czech Republic	1996	Beer	427
167	Czech Republic	1996	Beverages, Alcoholic	22
167	Czech Republic	1996	Bovine Meat	42
167	Czech Republic	1996	Butter, Ghee	11
167	Czech Republic	1996	Cereals, Other	2
167	Czech Republic	1996	Cheese	31
167	Czech Republic	1996	Cocoa Beans	5
167	Czech Republic	1996	Coconuts - Incl Copra	2
167	Czech Republic	1996	Coffee	9
167	Czech Republic	1996	Cream	6
167	Czech Republic	1996	Demersal Fish	9
167	Czech Republic	1996	Eggs	34
167	Czech Republic	1996	Fats, Animals, Raw	10
167	Czech Republic	1996	Freshwater Fish	4
167	Czech Republic	1996	Fruits, Other	56
167	Czech Republic	1996	Grapefruit	2
167	Czech Republic	1996	Grapes	16
167	Czech Republic	1996	Groundnut Oil	1
167	Czech Republic	1996	Groundnuts (Shelled Eq)	2
167	Czech Republic	1996	Honey	1
167	Czech Republic	1996	Lemons, Limes	4
167	Czech Republic	1996	Marine Fish, Other	5
167	Czech Republic	1996	Meat, Other	9
167	Czech Republic	1996	Milk, Whole	133
167	Czech Republic	1996	Mutton & Goat Meat	1
167	Czech Republic	1996	Nuts	4
167	Czech Republic	1996	Oats	1
167	Czech Republic	1996	Offals, Edible	15
167	Czech Republic	1996	Oilcrops Oil, Other	1
167	Czech Republic	1996	Onions	31
167	Czech Republic	1996	Oranges, Mandarines	65
167	Czech Republic	1996	Palm Oil	4
167	Czech Republic	1996	Peas	2
167	Czech Republic	1996	Pelagic Fish	9
167	Czech Republic	1996	Pigmeat	136
167	Czech Republic	1996	Pimento	2
167	Czech Republic	1996	Pineapples	2
167	Czech Republic	1996	Potatoes	221
167	Czech Republic	1996	Poultry Meat	41
167	Czech Republic	1996	Pulses, Other	2
167	Czech Republic	1996	Rape and Mustard Oil	30
167	Czech Republic	1996	Rice (Milled Equivalent)	13
167	Czech Republic	1996	Rye	64
167	Czech Republic	1996	Soyabean Oil	5
167	Czech Republic	1996	Sugar, Refined Equivalent	109
167	Czech Republic	1996	Sunflowerseed Oil	3
167	Czech Republic	1996	Sweeteners, Other	21
167	Czech Republic	1996	Tea	1
167	Czech Republic	1996	Tomatoes	19
167	Czech Republic	1996	Vegetables, Other	163
167	Czech Republic	1996	Wheat	287
167	Czech Republic	1996	Wine	18
167	Czech Republic	1997	Apples	43
167	Czech Republic	1997	Bananas	31
167	Czech Republic	1997	Beer	440
167	Czech Republic	1997	Beverages, Alcoholic	24
167	Czech Republic	1997	Bovine Meat	36
167	Czech Republic	1997	Butter, Ghee	10
167	Czech Republic	1997	Cereals, Other	2
167	Czech Republic	1997	Cheese	32
167	Czech Republic	1997	Cocoa Beans	6
167	Czech Republic	1997	Coconuts - Incl Copra	3
167	Czech Republic	1997	Coffee	11
167	Czech Republic	1997	Cream	5
167	Czech Republic	1997	Demersal Fish	10
167	Czech Republic	1997	Eggs	39
167	Czech Republic	1997	Fats, Animals, Raw	9
167	Czech Republic	1997	Freshwater Fish	4
167	Czech Republic	1997	Fruits, Other	48
167	Czech Republic	1997	Grapefruit	3
167	Czech Republic	1997	Grapes	14
167	Czech Republic	1997	Groundnut Oil	1
167	Czech Republic	1997	Groundnuts (Shelled Eq)	2
167	Czech Republic	1997	Honey	1
167	Czech Republic	1997	Lemons, Limes	6
167	Czech Republic	1997	Marine Fish, Other	3
167	Czech Republic	1997	Meat, Other	9
167	Czech Republic	1997	Milk, Whole	105
167	Czech Republic	1997	Mutton & Goat Meat	1
167	Czech Republic	1997	Nuts	4
167	Czech Republic	1997	Oats	1
167	Czech Republic	1997	Offals, Edible	14
167	Czech Republic	1997	Onions	27
167	Czech Republic	1997	Oranges, Mandarines	56
167	Czech Republic	1997	Peas	3
167	Czech Republic	1997	Pelagic Fish	8
167	Czech Republic	1997	Pigmeat	122
167	Czech Republic	1997	Pimento	2
167	Czech Republic	1997	Pineapples	2
167	Czech Republic	1997	Potatoes	220
167	Czech Republic	1997	Poultry Meat	51
167	Czech Republic	1997	Pulses, Other	2
167	Czech Republic	1997	Rape and Mustard Oil	35
167	Czech Republic	1997	Rice (Milled Equivalent)	11

FAO country code	Country	Reference year	Food item	Gross food supply (g $capita^{-1}$ d^{-1})
167	Czech Republic	1997	Rye	56
167	Czech Republic	1997	Soyabean Oil	4
167	Czech Republic	1997	Soyabeans	1
167	Czech Republic	1997	Sugar, Refined Equivalent	108
167	Czech Republic	1997	Sunflowerseed Oil	4
167	Czech Republic	1997	Sweeteners, Other	11
167	Czech Republic	1997	Tea	1
167	Czech Republic	1997	Tomatoes	20
167	Czech Republic	1997	Vegetables, Other	159
167	Czech Republic	1997	Wheat	302
167	Czech Republic	1997	Wine	18
167	Czech Republic	1998	Apples	57
167	Czech Republic	1998	Bananas	27
167	Czech Republic	1998	Beer	440
167	Czech Republic	1998	Beverages, Alcoholic	19
167	Czech Republic	1998	Bovine Meat	30
167	Czech Republic	1998	Butter, Ghee	11
167	Czech Republic	1998	Cereals, Other	2
167	Czech Republic	1998	Cheese	35
167	Czech Republic	1998	Cocoa Beans	5
167	Czech Republic	1998	Coconuts - Incl Copra	3
167	Czech Republic	1998	Coffee	8
167	Czech Republic	1998	Cream	4
167	Czech Republic	1998	Demersal Fish	9
167	Czech Republic	1998	Eggs	53
167	Czech Republic	1998	Fats, Animals, Raw	10
167	Czech Republic	1998	Freshwater Fish	4
167	Czech Republic	1998	Fruits, Other	44
167	Czech Republic	1998	Grapefruit	3
167	Czech Republic	1998	Grapes	15
167	Czech Republic	1998	Groundnut Oil	1
167	Czech Republic	1998	Groundnuts (Shelled Eq)	2
167	Czech Republic	1998	Honey	2
167	Czech Republic	1998	Lemons, Limes	5
167	Czech Republic	1998	Marine Fish, Other	3
167	Czech Republic	1998	Meat, Other	11
167	Czech Republic	1998	Milk, Whole	97
167	Czech Republic	1998	Mutton & Goat Meat	1
167	Czech Republic	1998	Nuts	4
167	Czech Republic	1998	Oats	1
167	Czech Republic	1998	Offals, Edible	14
167	Czech Republic	1998	Onions	31
167	Czech Republic	1998	Oranges, Mandarines	50
167	Czech Republic	1998	Peas	3
167	Czech Republic	1998	Pelagic Fish	8
167	Czech Republic	1998	Pigmeat	128
167	Czech Republic	1998	Pimento	1
167	Czech Republic	1998	Pineapples	2
167	Czech Republic	1998	Potatoes	220
167	Czech Republic	1998	Poultry Meat	56
167	Czech Republic	1998	Pulses, Other	2
167	Czech Republic	1998	Rape and Mustard Oil	37
167	Czech Republic	1998	Rice (Milled Equivalent)	12
167	Czech Republic	1998	Rye	48
167	Czech Republic	1998	Soyabean Oil	2
167	Czech Republic	1998	Soyabeans	1
167	Czech Republic	1998	Sugar, Refined Equivalent	103
167	Czech Republic	1998	Sunflowerseed Oil	5
167	Czech Republic	1998	Sweeteners, Other	14
167	Czech Republic	1998	Tea	1
167	Czech Republic	1998	Tomatoes	24
167	Czech Republic	1998	Vegetables, Other	159
167	Czech Republic	1998	Wheat	276
167	Czech Republic	1998	Wine	18
167	Czech Republic	1999	Apples	56
167	Czech Republic	1999	Bananas	31
167	Czech Republic	1999	Beer	443
167	Czech Republic	1999	Beverages, Alcoholic	20
167	Czech Republic	1999	Bovine Meat	27
167	Czech Republic	1999	Butter, Ghee	11
167	Czech Republic	1999	Cereals, Other	2
167	Czech Republic	1999	Cheese	35
167	Czech Republic	1999	Cocoa Beans	6
167	Czech Republic	1999	Coconuts - Incl Copra	3
167	Czech Republic	1999	Coffee	10
167	Czech Republic	1999	Cream	5
167	Czech Republic	1999	Demersal Fish	9
167	Czech Republic	1999	Eggs	48
167	Czech Republic	1999	Fats, Animals, Raw	10
167	Czech Republic	1999	Freshwater Fish	4
167	Czech Republic	1999	Fruits, Other	51
167	Czech Republic	1999	Grapefruit	3
167	Czech Republic	1999	Grapes	11
167	Czech Republic	1999	Groundnut Oil	1
167	Czech Republic	1999	Groundnuts (Shelled Eq)	2
167	Czech Republic	1999	Honey	2
167	Czech Republic	1999	Lemons, Limes	5
167	Czech Republic	1999	Marine Fish, Other	4
167	Czech Republic	1999	Meat, Other	11
167	Czech Republic	1999	Milk, Whole	96
167	Czech Republic	1999	Mutton & Goat Meat	1
167	Czech Republic	1999	Nuts	4
167	Czech Republic	1999	Oats	1
167	Czech Republic	1999	Offals, Edible	13
167	Czech Republic	1999	Onions	29
167	Czech Republic	1999	Oranges, Mandarines	43
167	Czech Republic	1999	Peas	3

FAO country code	Country	Reference year	Food item	Gross food supply (g *capita*$^{-1}$ d^{-1})
167	Czech Republic	1999	Pelagic Fish	8
167	Czech Republic	1999	Pigmeat	125
167	Czech Republic	1999	Pimento	1
167	Czech Republic	1999	Pineapples	2
167	Czech Republic	1999	Potatoes	217
167	Czech Republic	1999	Poultry Meat	58
167	Czech Republic	1999	Pulses, Other	2
167	Czech Republic	1999	Rape and Mustard Oil	36
167	Czech Republic	1999	Rice (Milled Equivalent)	12
167	Czech Republic	1999	Rye	38
167	Czech Republic	1999	Soyabean Oil	4
167	Czech Republic	1999	Sugar, Refined Equivalent	97
167	Czech Republic	1999	Sunflowerseed Oil	4
167	Czech Republic	1999	Sweeteners, Other	14
167	Czech Republic	1999	Tea	1
167	Czech Republic	1999	Tomatoes	28
167	Czech Republic	1999	Vegetables, Other	167
167	Czech Republic	1999	Wheat	247
167	Czech Republic	1999	Wine	18
167	Czech Republic	2000	Apples	52
167	Czech Republic	2000	Bananas	25
167	Czech Republic	2000	Barley	1
167	Czech Republic	2000	Beer	435
167	Czech Republic	2000	Beverages, Alcoholic	19
167	Czech Republic	2000	Bovine Meat	22
167	Czech Republic	2000	Butter, Ghee	11
167	Czech Republic	2000	Cereals, Other	2
167	Czech Republic	2000	Cheese	37
167	Czech Republic	2000	Cocoa Beans	6
167	Czech Republic	2000	Coconuts - Incl Copra	3
167	Czech Republic	2000	Coffee	10
167	Czech Republic	2000	Cream	3
167	Czech Republic	2000	Demersal Fish	10
167	Czech Republic	2000	Eggs	45
167	Czech Republic	2000	Fats, Animals, Raw	10
167	Czech Republic	2000	Freshwater Fish	5
167	Czech Republic	2000	Fruits, Other	53
167	Czech Republic	2000	Grapefruit	3
167	Czech Republic	2000	Grapes	13
167	Czech Republic	2000	Groundnuts (Shelled Eq)	2
167	Czech Republic	2000	Honey	2
167	Czech Republic	2000	Lemons, Limes	4
167	Czech Republic	2000	Maize	1
167	Czech Republic	2000	Marine Fish, Other	5
167	Czech Republic	2000	Meat, Other	11
167	Czech Republic	2000	Milk, Whole	129
167	Czech Republic	2000	Nuts	4
167	Czech Republic	2000	Oats	1
167	Czech Republic	2000	Offals, Edible	13
167	Czech Republic	2000	Onions	27
167	Czech Republic	2000	Oranges, Mandarines	43
167	Czech Republic	2000	Palm Oil	3
167	Czech Republic	2000	Peas	2
167	Czech Republic	2000	Pelagic Fish	8
167	Czech Republic	2000	Pigmeat	116
167	Czech Republic	2000	Pimento	1
167	Czech Republic	2000	Pineapples	2
167	Czech Republic	2000	Potatoes	219
167	Czech Republic	2000	Poultry Meat	61
167	Czech Republic	2000	Pulses, Other	2
167	Czech Republic	2000	Rape and Mustard Oil	36
167	Czech Republic	2000	Rice (Milled Equivalent)	13
167	Czech Republic	2000	Rye	20
167	Czech Republic	2000	Soyabean Oil	4
167	Czech Republic	2000	Soyabeans	1
167	Czech Republic	2000	Sugar, Refined Equivalent	99
167	Czech Republic	2000	Sunflowerseed Oil	1
167	Czech Republic	2000	Sweeteners, Other	12
167	Czech Republic	2000	Tea	1
167	Czech Republic	2000	Tomatoes	30
167	Czech Republic	2000	Vegetables, Other	152
167	Czech Republic	2000	Wheat	264
167	Czech Republic	2000	Wine	18
167	Czech Republic	2001	Apples	40
167	Czech Republic	2001	Bananas	24
167	Czech Republic	2001	Beans	1
167	Czech Republic	2001	Beer	428
167	Czech Republic	2001	Beverages, Alcoholic	18
167	Czech Republic	2001	Bovine Meat	18
167	Czech Republic	2001	Butter, Ghee	11
167	Czech Republic	2001	Cereals, Other	3
167	Czech Republic	2001	Cheese	37
167	Czech Republic	2001	Cocoa Beans	8
167	Czech Republic	2001	Coconuts - Incl Copra	3
167	Czech Republic	2001	Coffee	11
167	Czech Republic	2001	Cream	4
167	Czech Republic	2001	Demersal Fish	10
167	Czech Republic	2001	Eggs	46
167	Czech Republic	2001	Fats, Animals, Raw	10
167	Czech Republic	2001	Freshwater Fish	5
167	Czech Republic	2001	Fruits, Other	47
167	Czech Republic	2001	Grapefruit	3
167	Czech Republic	2001	Grapes	13
167	Czech Republic	2001	Groundnuts (Shelled Eq)	3
167	Czech Republic	2001	Honey	1
167	Czech Republic	2001	Lemons, Limes	5
167	Czech Republic	2001	Marine Fish, Other	5

FAO countrycode	Country	Reference year	Food item	Gross food supply (g $capita^{-1}$ d^{-1})
167	Czech Republic	2001	Meat, Other	11
167	Czech Republic	2001	Milk, Whole	92
167	Czech Republic	2001	Nuts	4
167	Czech Republic	2001	Oats	1
167	Czech Republic	2001	Offals, Edible	12
167	Czech Republic	2001	Olives	1
167	Czech Republic	2001	Onions	29
167	Czech Republic	2001	Oranges, Mandarines	39
167	Czech Republic	2001	Peas	2
167	Czech Republic	2001	Pelagic Fish	7
167	Czech Republic	2001	Pigmeat	114
167	Czech Republic	2001	Pimento	1
167	Czech Republic	2001	Pineapples	3
167	Czech Republic	2001	Potatoes	223
167	Czech Republic	2001	Poultry Meat	66
167	Czech Republic	2001	Pulses, Other	2
167	Czech Republic	2001	Rape and Mustard Oil	33
167	Czech Republic	2001	Rice (Milled Equivalent)	12
167	Czech Republic	2001	Rye	37
167	Czech Republic	2001	Soyabean Oil	7
167	Czech Republic	2001	Soyabeans	3
167	Czech Republic	2001	Sugar, Refined Equivalent	108
167	Czech Republic	2001	Sunflowerseed Oil	4
167	Czech Republic	2001	Sweeteners, Other	8
167	Czech Republic	2001	Tea	1
167	Czech Republic	2001	Tomatoes	31
167	Czech Republic	2001	Vegetables, Other	146
167	Czech Republic	2001	Wheat	260
167	Czech Republic	2001	Wine	24
167	Czech Republic	2002	Apples	25
167	Czech Republic	2002	Bananas	24
167	Czech Republic	2002	Beer	431
167	Czech Republic	2002	Beverages, Alcoholic	17
167	Czech Republic	2002	Bovine Meat	22
167	Czech Republic	2002	Butter, Ghee	13
167	Czech Republic	2002	Cereals, Other	4
167	Czech Republic	2002	Cheese	37
167	Czech Republic	2002	Cocoa Beans	9
167	Czech Republic	2002	Coconuts - Incl Copra	2
167	Czech Republic	2002	Coffee	11
167	Czech Republic	2002	Cream	4
167	Czech Republic	2002	Demersal Fish	9
167	Czech Republic	2002	Eggs	41
167	Czech Republic	2002	Fats, Animals, Raw	10
167	Czech Republic	2002	Freshwater Fish	5
167	Czech Republic	2002	Fruits, Other	44
167	Czech Republic	2002	Grapefruit	4
167	Czech Republic	2002	Grapes	5
167	Czech Republic	2002	Groundnuts (Shelled Eq)	3
167	Czech Republic	2002	Honey	1
167	Czech Republic	2002	Lemons, Limes	5
167	Czech Republic	2002	Marine Fish, Other	5
167	Czech Republic	2002	Meat, Other	11
167	Czech Republic	2002	Milk, Whole	79
167	Czech Republic	2002	Nuts	5
167	Czech Republic	2002	Oats	1
167	Czech Republic	2002	Offals, Edible	12
167	Czech Republic	2002	Olives	1
167	Czech Republic	2002	Onions	23
167	Czech Republic	2002	Oranges, Mandarines	48
167	Czech Republic	2002	Peas	1
167	Czech Republic	2002	Pelagic Fish	7
167	Czech Republic	2002	Pigmeat	116
167	Czech Republic	2002	Pimento	2
167	Czech Republic	2002	Pineapples	2
167	Czech Republic	2002	Potatoes	222
167	Czech Republic	2002	Poultry Meat	63
167	Czech Republic	2002	Pulses, Other	2
167	Czech Republic	2002	Rape and Mustard Oil	32
167	Czech Republic	2002	Rice (Milled Equivalent)	13
167	Czech Republic	2002	Rye	33
167	Czech Republic	2002	Soyabean Oil	6
167	Czech Republic	2002	Soyabeans	2
167	Czech Republic	2002	Sugar, Refined Equivalent	115
167	Czech Republic	2002	Sunflowerseed Oil	6
167	Czech Republic	2002	Sweeteners, Other	14
167	Czech Republic	2002	Tea	1
167	Czech Republic	2002	Tomatoes	33
167	Czech Republic	2002	Vegetables, Other	144
167	Czech Republic	2002	Wheat	277
167	Czech Republic	2002	Wine	30
167	Czech Republic	2003	Apples	31
167	Czech Republic	2003	Bananas	26
167	Czech Republic	2003	Beer	434
167	Czech Republic	2003	Beverages, Alcoholic	18
167	Czech Republic	2003	Bovine Meat	24
167	Czech Republic	2003	Butter, Ghee	11
167	Czech Republic	2003	Cereals, Other	7
167	Czech Republic	2003	Cheese	37
167	Czech Republic	2003	Cocoa Beans	8
167	Czech Republic	2003	Coconuts - Incl Copra	3
167	Czech Republic	2003	Coffee	9
167	Czech Republic	2003	Cream	4
167	Czech Republic	2003	Demersal Fish	9
167	Czech Republic	2003	Eggs	40
167	Czech Republic	2003	Fats, Animals, Raw	10
167	Czech Republic	2003	Freshwater Fish	5

FAO country code	Country	Reference year	Food item	Gross food supply (g $capita^{-1}$ d^{-1})
167	Czech Republic	2003	Fruits, Other	46
167	Czech Republic	2003	Grapefruit	4
167	Czech Republic	2003	Grapes	9
167	Czech Republic	2003	Groundnuts (Shelled Eq)	3
167	Czech Republic	2003	Honey	2
167	Czech Republic	2003	Lemons, Limes	5
167	Czech Republic	2003	Marine Fish, Other	5
167	Czech Republic	2003	Meat, Other	11
167	Czech Republic	2003	Milk, Whole	94
167	Czech Republic	2003	Nuts	5
167	Czech Republic	2003	Oats	1
167	Czech Republic	2003	Offals, Edible	12
167	Czech Republic	2003	Olives	1
167	Czech Republic	2003	Onions	22
167	Czech Republic	2003	Oranges, Mandarines	58
167	Czech Republic	2003	Peas	3
167	Czech Republic	2003	Pelagic Fish	7
167	Czech Republic	2003	Pigmeat	119
167	Czech Republic	2003	Pimento	2
167	Czech Republic	2003	Pineapples	3
167	Czech Republic	2003	Potatoes	216
167	Czech Republic	2003	Poultry Meat	65
167	Czech Republic	2003	Pulses, Other	2
167	Czech Republic	2003	Rape and Mustard Oil	29
167	Czech Republic	2003	Rice (Milled Equivalent)	13
167	Czech Republic	2003	Rye	39
167	Czech Republic	2003	Soyabean Oil	11
167	Czech Republic	2003	Soyabeans	1
167	Czech Republic	2003	Sugar, Refined Equivalent	121
167	Czech Republic	2003	Sunflowerseed Oil	8
167	Czech Republic	2003	Sweeteners, Other	17
167	Czech Republic	2003	Tea	1
167	Czech Republic	2003	Tomatoes	32
167	Czech Republic	2003	Vegetables, Other	140
167	Czech Republic	2003	Wheat	267
167	Czech Republic	2003	Wine	32
167	Czech Republic	2004	Apples	21
167	Czech Republic	2004	Bananas	25
167	Czech Republic	2004	Beans	1
167	Czech Republic	2004	Beer	438
167	Czech Republic	2004	Beverages, Alcoholic	21
167	Czech Republic	2004	Bovine Meat	23
167	Czech Republic	2004	Butter, Ghee	9
167	Czech Republic	2004	Cereals, Other	5
167	Czech Republic	2004	Cheese	39
167	Czech Republic	2004	Cocoa Beans	8
167	Czech Republic	2004	Coconuts - Incl Copra	3
167	Czech Republic	2004	Coffee	8
167	Czech Republic	2004	Cream	4
167	Czech Republic	2004	Demersal Fish	7
167	Czech Republic	2004	Eggs	39
167	Czech Republic	2004	Fats, Animals, Raw	11
167	Czech Republic	2004	Freshwater Fish	5
167	Czech Republic	2004	Fruits, Other	46
167	Czech Republic	2004	Grapefruit	5
167	Czech Republic	2004	Grapes	10
167	Czech Republic	2004	Groundnuts (Shelled Eq)	3
167	Czech Republic	2004	Honey	2
167	Czech Republic	2004	Lemons, Limes	6
167	Czech Republic	2004	Marine Fish, Other	5
167	Czech Republic	2004	Meat, Other	11
167	Czech Republic	2004	Milk, Whole	75
167	Czech Republic	2004	Nuts	5
167	Czech Republic	2004	Oats	1
167	Czech Republic	2004	Offals, Edible	14
167	Czech Republic	2004	Oilcrops Oil, Other	1
167	Czech Republic	2004	Olive Oil	1
167	Czech Republic	2004	Olives	6
167	Czech Republic	2004	Onions	28
167	Czech Republic	2004	Oranges, Mandarines	85
167	Czech Republic	2004	Peas	3
167	Czech Republic	2004	Pelagic Fish	9
167	Czech Republic	2004	Pigmeat	133
167	Czech Republic	2004	Pimento	2
167	Czech Republic	2004	Pineapples	4
167	Czech Republic	2004	Potatoes	199
167	Czech Republic	2004	Poultry Meat	70
167	Czech Republic	2004	Pulses, Other	2
167	Czech Republic	2004	Rape and Mustard Oil	26
167	Czech Republic	2004	Rice (Milled Equivalent)	13
167	Czech Republic	2004	Rye	46
167	Czech Republic	2004	Soyabean Oil	7
167	Czech Republic	2004	Soyabeans	2
167	Czech Republic	2004	Sugar, Refined Equivalent	118
167	Czech Republic	2004	Sunflowerseed Oil	7
167	Czech Republic	2004	Sweeteners, Other	21
167	Czech Republic	2004	Tea	1
167	Czech Republic	2004	Tomatoes	31
167	Czech Republic	2004	Vegetables, Other	133
167	Czech Republic	2004	Wheat	272
167	Czech Republic	2004	Wine	36
167	Czech Republic	2005	Apples	40
167	Czech Republic	2005	Bananas	20
167	Czech Republic	2005	Beans	1
167	Czech Republic	2005	Beer	444
167	Czech Republic	2005	Beverages, Alcoholic	14
167	Czech Republic	2005	Bovine Meat	22

FAO country code	Country	Reference year	Food item	Gross food supply (g *capita*$^{-1}$ d^{-1})
167	Czech Republic	2005	Butter, Ghee	12
167	Czech Republic	2005	Cereals, Other	4
167	Czech Republic	2005	Cheese	41
167	Czech Republic	2005	Cocoa Beans	8
167	Czech Republic	2005	Coconuts - Incl Copra	3
167	Czech Republic	2005	Coffee	8
167	Czech Republic	2005	Cream	11
167	Czech Republic	2005	Demersal Fish	8
167	Czech Republic	2005	Eggs	38
167	Czech Republic	2005	Fats, Animals, Raw	12
167	Czech Republic	2005	Freshwater Fish	6
167	Czech Republic	2005	Fruits, Other	48
167	Czech Republic	2005	Grapefruit	5
167	Czech Republic	2005	Grapes	9
167	Czech Republic	2005	Groundnuts (Shelled Eq)	2
167	Czech Republic	2005	Honey	2
167	Czech Republic	2005	Lemons, Limes	7
167	Czech Republic	2005	Marine Fish, Other	4
167	Czech Republic	2005	Meat, Other	11
167	Czech Republic	2005	Milk, Whole	130
167	Czech Republic	2005	Mutton & Goat Meat	1
167	Czech Republic	2005	Nuts	5
167	Czech Republic	2005	Oats	3
167	Czech Republic	2005	Offals, Edible	13
167	Czech Republic	2005	Olive Oil	1
167	Czech Republic	2005	Olives	1
167	Czech Republic	2005	Onions	23
167	Czech Republic	2005	Oranges, Mandarines	58
167	Czech Republic	2005	Palm Oil	7
167	Czech Republic	2005	Peas	5
167	Czech Republic	2005	Pelagic Fish	9
167	Czech Republic	2005	Pigmeat	134
167	Czech Republic	2005	Pimento	2
167	Czech Republic	2005	Pineapples	4
167	Czech Republic	2005	Potatoes	213
167	Czech Republic	2005	Poultry Meat	72
167	Czech Republic	2005	Pulses, Other	2
167	Czech Republic	2005	Rape and Mustard Oil	24
167	Czech Republic	2005	Rice (Milled Equivalent)	11
167	Czech Republic	2005	Rye	38
167	Czech Republic	2005	Soyabean Oil	6
167	Czech Republic	2005	Soyabeans	1
167	Czech Republic	2005	Sugar, Refined Equivalent	78
167	Czech Republic	2005	Sunflowerseed Oil	9
167	Czech Republic	2005	Sweeteners, Other	16
167	Czech Republic	2005	Tea	1
167	Czech Republic	2005	Tomatoes	35
167	Czech Republic	2005	Vegetables, Other	159
167	Czech Republic	2005	Wheat	301
167	Czech Republic	2005	Wine	33
167	Czech Republic	2006	Apples	36
167	Czech Republic	2006	Bananas	20
167	Czech Republic	2006	Beer	445
167	Czech Republic	2006	Beverages, Alcoholic	19
167	Czech Republic	2006	Bovine Meat	21
167	Czech Republic	2006	Butter, Ghee	11
167	Czech Republic	2006	Cereals, Other	4
167	Czech Republic	2006	Cheese	43
167	Czech Republic	2006	Citrus, Other	1
167	Czech Republic	2006	Cocoa Beans	9
167	Czech Republic	2006	Coconuts - Incl Copra	3
167	Czech Republic	2006	Coffee	8
167	Czech Republic	2006	Cream	12
167	Czech Republic	2006	Demersal Fish	6
167	Czech Republic	2006	Eggs	43
167	Czech Republic	2006	Fats, Animals, Raw	12
167	Czech Republic	2006	Freshwater Fish	6
167	Czech Republic	2006	Fruits, Other	48
167	Czech Republic	2006	Grapefruit	4
167	Czech Republic	2006	Grapes	8
167	Czech Republic	2006	Groundnuts (Shelled Eq)	2
167	Czech Republic	2006	Honey	2
167	Czech Republic	2006	Lemons, Limes	7
167	Czech Republic	2006	Marine Fish, Other	4
167	Czech Republic	2006	Meat, Other	11
167	Czech Republic	2006	Milk, Whole	96
167	Czech Republic	2006	Mutton & Goat Meat	1
167	Czech Republic	2006	Nuts	7
167	Czech Republic	2006	Oats	5
167	Czech Republic	2006	Offals, Edible	11
167	Czech Republic	2006	Oilcrops Oil, Other	1
167	Czech Republic	2006	Olive Oil	1
167	Czech Republic	2006	Olives	1
167	Czech Republic	2006	Onions	25
167	Czech Republic	2006	Oranges, Mandarines	58
167	Czech Republic	2006	Palm Oil	3
167	Czech Republic	2006	Peas	5
167	Czech Republic	2006	Pelagic Fish	9
167	Czech Republic	2006	Pigmeat	127
167	Czech Republic	2006	Pimento	1
167	Czech Republic	2006	Pineapples	6
167	Czech Republic	2006	Potatoes	174
167	Czech Republic	2006	Poultry Meat	72
167	Czech Republic	2006	Pulses, Other	2
167	Czech Republic	2006	Rape and Mustard Oil	31
167	Czech Republic	2006	Rice (Milled Equivalent)	14
167	Czech Republic	2006	Rye	10

FAO countrycode	Country	Reference year	Food item	Gross food supply (g *capita*$^{-1}$ d^{-1})
167	Czech Republic	2006	Soyabean Oil	11
167	Czech Republic	2006	Soyabeans	1
167	Czech Republic	2006	Sugar, Refined Equivalent	64
167	Czech Republic	2006	Sunflowerseed Oil	12
167	Czech Republic	2006	Sweeteners, Other	16
167	Czech Republic	2006	Tea	1
167	Czech Republic	2006	Tomatoes	37
167	Czech Republic	2006	Vegetables, Other	151
167	Czech Republic	2006	Wheat	305
167	Czech Republic	2006	Wine	34
167	Czech Republic	2007	Apples	34
167	Czech Republic	2007	Bananas	22
167	Czech Republic	2007	Beer	402
167	Czech Republic	2007	Beverages, Alcoholic	20
167	Czech Republic	2007	Beverages, Fermented	1
167	Czech Republic	2007	Bovine Meat	22
167	Czech Republic	2007	Butter, Ghee	12
167	Czech Republic	2007	Cereals, Other	3
167	Czech Republic	2007	Cheese	44
167	Czech Republic	2007	Citrus, Other	1
167	Czech Republic	2007	Cocoa Beans	8
167	Czech Republic	2007	Coconuts - Incl Copra	2
167	Czech Republic	2007	Coffee	10
167	Czech Republic	2007	Cream	11
167	Czech Republic	2007	Demersal Fish	6
167	Czech Republic	2007	Eggs	41
167	Czech Republic	2007	Fats, Animals, Raw	11
167	Czech Republic	2007	Freshwater Fish	7
167	Czech Republic	2007	Fruits, Other	48
167	Czech Republic	2007	Grapefruit	5
167	Czech Republic	2007	Grapes	19
167	Czech Republic	2007	Groundnuts (Shelled Eq)	2
167	Czech Republic	2007	Honey	2
167	Czech Republic	2007	Lemons, Limes	6
167	Czech Republic	2007	Marine Fish, Other	4
167	Czech Republic	2007	Meat, Other	15
167	Czech Republic	2007	Milk, Whole	123
167	Czech Republic	2007	Mutton & Goat Meat	1
167	Czech Republic	2007	Nuts	7
167	Czech Republic	2007	Oats	3
167	Czech Republic	2007	Offals, Edible	11
167	Czech Republic	2007	Olive Oil	1
167	Czech Republic	2007	Onions	25
167	Czech Republic	2007	Oranges, Mandarines	50
167	Czech Republic	2007	Peas	5
167	Czech Republic	2007	Pelagic Fish	9
167	Czech Republic	2007	Pigmeat	129
167	Czech Republic	2007	Pimento	1
167	Czech Republic	2007	Pineapples	5
167	Czech Republic	2007	Potatoes	186
167	Czech Republic	2007	Poultry Meat	67
167	Czech Republic	2007	Pulses, Other	2
167	Czech Republic	2007	Rape and Mustard Oil	41
167	Czech Republic	2007	Rice (Milled Equivalent)	13
167	Czech Republic	2007	Rye	26
167	Czech Republic	2007	Soyabean Oil	5
167	Czech Republic	2007	Sugar, Refined Equivalent	75
167	Czech Republic	2007	Sunflowerseed Oil	8
167	Czech Republic	2007	Sweeteners, Other	12
167	Czech Republic	2007	Tea	2
167	Czech Republic	2007	Tomatoes	37
167	Czech Republic	2007	Vegetables, Other	145
167	Czech Republic	2007	Wheat	283
167	Czech Republic	2007	Wine	33
167	Czech Republic	2008	Apples	43
167	Czech Republic	2008	Bananas	27
167	Czech Republic	2008	Barley	1
167	Czech Republic	2008	Beans	1
167	Czech Republic	2008	Beer	416
167	Czech Republic	2008	Beverages, Alcoholic	20
167	Czech Republic	2008	Bovine Meat	21
167	Czech Republic	2008	Butter, Ghee	12
167	Czech Republic	2008	Cereals, Other	2
167	Czech Republic	2008	Cheese	43
167	Czech Republic	2008	Citrus, Other	1
167	Czech Republic	2008	Cocoa Beans	9
167	Czech Republic	2008	Coconuts - Incl Copra	2
167	Czech Republic	2008	Coffee	7
167	Czech Republic	2008	Cream	11
167	Czech Republic	2008	Demersal Fish	6
167	Czech Republic	2008	Eggs	41
167	Czech Republic	2008	Fats, Animals, Raw	12
167	Czech Republic	2008	Freshwater Fish	10
167	Czech Republic	2008	Fruits, Other	51
167	Czech Republic	2008	Grapefruit	5
167	Czech Republic	2008	Grapes	25
167	Czech Republic	2008	Groundnuts (Shelled Eq)	2
167	Czech Republic	2008	Honey	1
167	Czech Republic	2008	Lemons, Limes	6
167	Czech Republic	2008	Marine Fish, Other	3
167	Czech Republic	2008	Meat, Other	15
167	Czech Republic	2008	Milk, Whole	109
167	Czech Republic	2008	Molluscs, Other	1
167	Czech Republic	2008	Mutton & Goat Meat	1
167	Czech Republic	2008	Nuts	7
167	Czech Republic	2008	Oats	4
167	Czech Republic	2008	Offals, Edible	11

FAO country code	Country	Reference year	Food item	Gross food supply (g *capita*$^{-1}$ d^{-1})
167	Czech Republic	2008	Olive Oil	1
167	Czech Republic	2008	Olives	1
167	Czech Republic	2008	Onions	23
167	Czech Republic	2008	Oranges, Mandarines	45
167	Czech Republic	2008	Palm Oil	4
167	Czech Republic	2008	Peas	5
167	Czech Republic	2008	Pelagic Fish	10
167	Czech Republic	2008	Pigmeat	126
167	Czech Republic	2008	Pimento	1
167	Czech Republic	2008	Pineapples	7
167	Czech Republic	2008	Potatoes	188
167	Czech Republic	2008	Poultry Meat	68
167	Czech Republic	2008	Pulses, Other	2
167	Czech Republic	2008	Rape and Mustard Oil	38
167	Czech Republic	2008	Rice (Milled Equivalent)	13
167	Czech Republic	2008	Rye	28
167	Czech Republic	2008	Soyabean Oil	10
167	Czech Republic	2008	Soyabeans	1
167	Czech Republic	2008	Sugar, Refined Equivalent	76
167	Czech Republic	2008	Sunflowerseed Oil	5
167	Czech Republic	2008	Sweeteners, Other	13
167	Czech Republic	2008	Tea	4
167	Czech Republic	2008	Tomatoes	40
167	Czech Republic	2008	Vegetables, Other	149
167	Czech Republic	2008	Wheat	269
167	Czech Republic	2008	Wine	33
167	Czech Republic	2009	Apples	42
167	Czech Republic	2009	Bananas	28
167	Czech Republic	2009	Barley	1
167	Czech Republic	2009	Beer	393
167	Czech Republic	2009	Beverages, Alcoholic	23
167	Czech Republic	2009	Beverages, Fermented	1
167	Czech Republic	2009	Bovine Meat	22
167	Czech Republic	2009	Butter, Ghee	13
167	Czech Republic	2009	Cereals, Other	2
167	Czech Republic	2009	Cheese	44
167	Czech Republic	2009	Citrus, Other	1
167	Czech Republic	2009	Cocoa Beans	9
167	Czech Republic	2009	Coconuts - Incl Copra	3
167	Czech Republic	2009	Coffee	8
167	Czech Republic	2009	Cream	14
167	Czech Republic	2009	Demersal Fish	5
167	Czech Republic	2009	Eggs	42
167	Czech Republic	2009	Fats, Animals, Raw	12
167	Czech Republic	2009	Freshwater Fish	11
167	Czech Republic	2009	Fruits, Other	49
167	Czech Republic	2009	Grapefruit	5
167	Czech Republic	2009	Grapes	16
167	Czech Republic	2009	Groundnuts (Shelled Eq)	2
167	Czech Republic	2009	Honey	2
167	Czech Republic	2009	Lemons, Limes	7
167	Czech Republic	2009	Marine Fish, Other	2
167	Czech Republic	2009	Meat, Other	15
167	Czech Republic	2009	Milk, Whole	72
167	Czech Republic	2009	Molluscs, Other	1
167	Czech Republic	2009	Mutton & Goat Meat	1
167	Czech Republic	2009	Nuts	7
167	Czech Republic	2009	Oats	6
167	Czech Republic	2009	Offals, Edible	11
167	Czech Republic	2009	Olive Oil	1
167	Czech Republic	2009	Olives	2
167	Czech Republic	2009	Onions	23
167	Czech Republic	2009	Oranges, Mandarines	56
167	Czech Republic	2009	Peas	5
167	Czech Republic	2009	Pelagic Fish	8
167	Czech Republic	2009	Pigmeat	124
167	Czech Republic	2009	Pimento	1
167	Czech Republic	2009	Pineapples	4
167	Czech Republic	2009	Potatoes	180
167	Czech Republic	2009	Poultry Meat	69
167	Czech Republic	2009	Pulses, Other	2
167	Czech Republic	2009	Rape and Mustard Oil	45
167	Czech Republic	2009	Rice (Milled Equivalent)	11
167	Czech Republic	2009	Rye	30
167	Czech Republic	2009	Soyabean Oil	6
167	Czech Republic	2009	Sugar, Refined Equivalent	86
167	Czech Republic	2009	Sunflowerseed Oil	5
167	Czech Republic	2009	Sweeteners, Other	14
167	Czech Republic	2009	Tea	1
167	Czech Republic	2009	Tomatoes	32
167	Czech Republic	2009	Vegetables, Other	142
167	Czech Republic	2009	Wheat	271
167	Czech Republic	2009	Wine	33
167	Czech Republic	2010	Apples	33
167	Czech Republic	2010	Bananas	28
167	Czech Republic	2010	Beer	374
167	Czech Republic	2010	Beverages, Alcoholic	13
167	Czech Republic	2010	Beverages, Fermented	1
167	Czech Republic	2010	Bovine Meat	21
167	Czech Republic	2010	Butter, Ghee	13
167	Czech Republic	2010	Cereals, Other	1
167	Czech Republic	2010	Cheese	46
167	Czech Republic	2010	Citrus, Other	2
167	Czech Republic	2010	Cocoa Beans	6
167	Czech Republic	2010	Coconuts - Incl Copra	2
167	Czech Republic	2010	Coffee	7
167	Czech Republic	2010	Cream	11

FAO country code	Country	Reference year	Food item	Gross food supply (g *capita*$^{-1}$ d^{-1})
167	Czech Republic	2010	Demersal Fish	6
167	Czech Republic	2010	Eggs	35
167	Czech Republic	2010	Fats, Animals, Raw	11
167	Czech Republic	2010	Freshwater Fish	10
167	Czech Republic	2010	Fruits, Other	47
167	Czech Republic	2010	Grapefruit	5
167	Czech Republic	2010	Grapes	9
167	Czech Republic	2010	Groundnuts (Shelled Eq)	2
167	Czech Republic	2010	Honey	2
167	Czech Republic	2010	Lemons, Limes	6
167	Czech Republic	2010	Marine Fish, Other	2
167	Czech Republic	2010	Meat, Other	14
167	Czech Republic	2010	Milk, Whole	67
167	Czech Republic	2010	Molluscs, Other	1
167	Czech Republic	2010	Mutton & Goat Meat	1
167	Czech Republic	2010	Nuts	6
167	Czech Republic	2010	Oats	5
167	Czech Republic	2010	Offals, Edible	11
167	Czech Republic	2010	Olive Oil	1
167	Czech Republic	2010	Olives	1
167	Czech Republic	2010	Onions	22
167	Czech Republic	2010	Oranges, Mandarines	61
167	Czech Republic	2010	Peas	5
167	Czech Republic	2010	Pelagic Fish	8
167	Czech Republic	2010	Pigmeat	126
167	Czech Republic	2010	Pimento	1
167	Czech Republic	2010	Pineapples	4
167	Czech Republic	2010	Potatoes	170
167	Czech Republic	2010	Poultry Meat	66
167	Czech Republic	2010	Pulses, Other	2
167	Czech Republic	2010	Rape and Mustard Oil	44
167	Czech Republic	2010	Rice (Milled Equivalent)	12
167	Czech Republic	2010	Rye	28
167	Czech Republic	2010	Soyabean Oil	10
167	Czech Republic	2010	Soyabeans	1
167	Czech Republic	2010	Sugar, Refined Equivalent	90
167	Czech Republic	2010	Sunflowerseed Oil	9
167	Czech Republic	2010	Sweeteners, Other	12
167	Czech Republic	2010	Tea	1
167	Czech Republic	2010	Tomatoes	34
167	Czech Republic	2010	Vegetables, Other	137
167	Czech Republic	2010	Wheat	273
167	Czech Republic	2010	Wine	33
167	Czech Republic	2011	Apples	32
167	Czech Republic	2011	Bananas	22
167	Czech Republic	2011	Beer	374
167	Czech Republic	2011	Beverages, Alcoholic	18
167	Czech Republic	2011	Beverages, Fermented	1
167	Czech Republic	2011	Bovine Meat	20
167	Czech Republic	2011	Butter, Ghee	14
167	Czech Republic	2011	Cereals, Other	1
167	Czech Republic	2011	Cheese	45
167	Czech Republic	2011	Citrus, Other	1
167	Czech Republic	2011	Cocoa Beans	7
167	Czech Republic	2011	Coconuts - Incl Copra	2
167	Czech Republic	2011	Coffee	10
167	Czech Republic	2011	Cream	9
167	Czech Republic	2011	Demersal Fish	6
167	Czech Republic	2011	Eggs	36
167	Czech Republic	2011	Fats, Animals, Raw	12
167	Czech Republic	2011	Freshwater Fish	10
167	Czech Republic	2011	Fruits, Other	50
167	Czech Republic	2011	Grapefruit	5
167	Czech Republic	2011	Grapes	21
167	Czech Republic	2011	Groundnuts (Shelled Eq)	2
167	Czech Republic	2011	Honey	3
167	Czech Republic	2011	Lemons, Limes	7
167	Czech Republic	2011	Marine Fish, Other	2
167	Czech Republic	2011	Meat, Other	13
167	Czech Republic	2011	Milk, Whole	62
167	Czech Republic	2011	Molluscs, Other	1
167	Czech Republic	2011	Mutton & Goat Meat	1
167	Czech Republic	2011	Nuts	5
167	Czech Republic	2011	Oats	5
167	Czech Republic	2011	Offals, Edible	10
167	Czech Republic	2011	Olive Oil	1
167	Czech Republic	2011	Olives	2
167	Czech Republic	2011	Onions	24
167	Czech Republic	2011	Oranges, Mandarines	44
167	Czech Republic	2011	Peas	5
167	Czech Republic	2011	Pelagic Fish	8
167	Czech Republic	2011	Pigmeat	124
167	Czech Republic	2011	Pimento	2
167	Czech Republic	2011	Pineapples	5
167	Czech Republic	2011	Potatoes	181
167	Czech Republic	2011	Poultry Meat	63
167	Czech Republic	2011	Pulses, Other	2
167	Czech Republic	2011	Rape and Mustard Oil	48
167	Czech Republic	2011	Rice (Milled Equivalent)	12
167	Czech Republic	2011	Rye	29
167	Czech Republic	2011	Soyabean Oil	5
167	Czech Republic	2011	Soyabeans	2
167	Czech Republic	2011	Sugar, Refined Equivalent	84
167	Czech Republic	2011	Sunflowerseed Oil	5
167	Czech Republic	2011	Sweeteners, Other	22
167	Czech Republic	2011	Tea	1
167	Czech Republic	2011	Tomatoes	36

FAO country code	Country	Reference year	Food item	Gross food supply (g $capita^{-1}$ d^{-1})
167	Czech Republic	2011	Vegetables, Other	143
167	Czech Republic	2011	Wheat	279
167	Czech Republic	2011	Wine	34
116	Democratic People's Republic of Korea	1992	Apples	77
116	Democratic People's Republic of Korea	1992	Barley	11
116	Democratic People's Republic of Korea	1992	Beans	36
116	Democratic People's Republic of Korea	1992	Beer	15
116	Democratic People's Republic of Korea	1992	Beverages, Alcoholic	16
116	Democratic People's Republic of Korea	1992	Bovine Meat	4
116	Democratic People's Republic of Korea	1992	Crustaceans	1
116	Democratic People's Republic of Korea	1992	Demersal Fish	1
116	Democratic People's Republic of Korea	1992	Eggs	14
116	Democratic People's Republic of Korea	1992	Fats, Animals, Raw	2
116	Democratic People's Republic of Korea	1992	Freshwater Fish	4
116	Democratic People's Republic of Korea	1992	Fruits, Other	82
116	Democratic People's Republic of Korea	1992	Maize	149
116	Democratic People's Republic of Korea	1992	Maize Germ Oil	3
116	Democratic People's Republic of Korea	1992	Marine Fish, Other	36
116	Democratic People's Republic of Korea	1992	Milk, Whole	12
116	Democratic People's Republic of Korea	1992	Millet	4
116	Democratic People's Republic of Korea	1992	Molluscs, Other	5
116	Democratic People's Republic of Korea	1992	Mutton & Goat Meat	1
116	Democratic People's Republic of Korea	1992	Nuts	1
116	Democratic People's Republic of Korea	1992	Oats	4
116	Democratic People's Republic of Korea	1992	Offals, Edible	3
116	Democratic People's Republic of Korea	1992	Onions	13
116	Democratic People's Republic of Korea	1992	Palm Oil	1
116	Democratic People's Republic of Korea	1992	Pigmeat	24
116	Democratic People's Republic of Korea	1992	Potatoes	78
116	Democratic People's Republic of Korea	1992	Poultry Meat	6
116	Democratic People's Republic of Korea	1992	Rice (Milled Equivalent)	192
116	Democratic People's Republic of Korea	1992	Rye	9
116	Democratic People's Republic of Korea	1992	Sorghum	1
116	Democratic People's Republic of Korea	1992	Soyabean Oil	7
116	Democratic People's Republic of Korea	1992	Soyabeans	26
116	Democratic People's Republic of Korea	1992	Sugar, Refined Equivalent	11
116	Democratic People's Republic of Korea	1992	Sweet Potatoes	30
116	Democratic People's Republic of Korea	1992	Tomatoes	8
116	Democratic People's Republic of Korea	1992	Vegetables, Other	454
116	Democratic People's Republic of Korea	1992	Wheat	52
116	Democratic People's Republic of Korea	1993	Apples	70
116	Democratic People's Republic of Korea	1993	Barley	9
116	Democratic People's Republic of Korea	1993	Beans	33
116	Democratic People's Republic of Korea	1993	Beer	12
116	Democratic People's Republic of Korea	1993	Beverages, Alcoholic	19
116	Democratic People's Republic of Korea	1993	Bovine Meat	4
116	Democratic People's Republic of Korea	1993	Crustaceans	3
116	Democratic People's Republic of Korea	1993	Eggs	12
116	Democratic People's Republic of Korea	1993	Fats, Animals, Raw	1
116	Democratic People's Republic of Korea	1993	Freshwater Fish	4
116	Democratic People's Republic of Korea	1993	Fruits, Other	77
116	Democratic People's Republic of Korea	1993	Maize	176
116	Democratic People's Republic of Korea	1993	Maize Germ Oil	3
116	Democratic People's Republic of Korea	1993	Marine Fish, Other	38
116	Democratic People's Republic of Korea	1993	Milk, Whole	10
116	Democratic People's Republic of Korea	1993	Millet	3
116	Democratic People's Republic of Korea	1993	Molasses	2
116	Democratic People's Republic of Korea	1993	Molluscs, Other	5
116	Democratic People's Republic of Korea	1993	Mutton & Goat Meat	1
116	Democratic People's Republic of Korea	1993	Nuts	1
116	Democratic People's Republic of Korea	1993	Oats	3
116	Democratic People's Republic of Korea	1993	Offals, Edible	2
116	Democratic People's Republic of Korea	1993	Onions	9
116	Democratic People's Republic of Korea	1993	Oranges, Mandarines	1
116	Democratic People's Republic of Korea	1993	Palm Oil	2
116	Democratic People's Republic of Korea	1993	Pigmeat	21
116	Democratic People's Republic of Korea	1993	Potatoes	32
116	Democratic People's Republic of Korea	1993	Poultry Meat	4
116	Democratic People's Republic of Korea	1993	Rice (Milled Equivalent)	195
116	Democratic People's Republic of Korea	1993	Rye	7
116	Democratic People's Republic of Korea	1993	Sorghum	2
116	Democratic People's Republic of Korea	1993	Soyabean Oil	7
116	Democratic People's Republic of Korea	1993	Soyabeans	22
116	Democratic People's Republic of Korea	1993	Sugar, Refined Equivalent	12
116	Democratic People's Republic of Korea	1993	Sweet Potatoes	14
116	Democratic People's Republic of Korea	1993	Tomatoes	7
116	Democratic People's Republic of Korea	1993	Vegetables, Other	402
116	Democratic People's Republic of Korea	1993	Wheat	73
116	Democratic People's Republic of Korea	1994	Apples	75
116	Democratic People's Republic of Korea	1994	Barley	13
116	Democratic People's Republic of Korea	1994	Beans	34
116	Democratic People's Republic of Korea	1994	Beer	18
116	Democratic People's Republic of Korea	1994	Beverages, Alcoholic	20
116	Democratic People's Republic of Korea	1994	Bovine Meat	4
116	Democratic People's Republic of Korea	1994	Crustaceans	3
116	Democratic People's Republic of Korea	1994	Demersal Fish	5
116	Democratic People's Republic of Korea	1994	Eggs	11
116	Democratic People's Republic of Korea	1994	Fats, Animals, Raw	1
116	Democratic People's Republic of Korea	1994	Freshwater Fish	3
116	Democratic People's Republic of Korea	1994	Fruits, Other	83
116	Democratic People's Republic of Korea	1994	Maize	168
116	Democratic People's Republic of Korea	1994	Maize Germ Oil	3
116	Democratic People's Republic of Korea	1994	Marine Fish, Other	27
116	Democratic People's Republic of Korea	1994	Milk, Whole	11
116	Democratic People's Republic of Korea	1994	Millet	3
116	Democratic People's Republic of Korea	1994	Molasses	1
116	Democratic People's Republic of Korea	1994	Molluscs, Other	8

FAO country code	Country	Reference year	Food item	Gross food supply (g *capita*$^{-1}$ d^{-1})
116	Democratic People's Republic of Korea	1994	Mutton & Goat Meat	1
116	Democratic People's Republic of Korea	1994	Nuts	1
116	Democratic People's Republic of Korea	1994	Oats	2
116	Democratic People's Republic of Korea	1994	Offals, Edible	2
116	Democratic People's Republic of Korea	1994	Onions	10
116	Democratic People's Republic of Korea	1994	Palm Oil	3
116	Democratic People's Republic of Korea	1994	Pelagic Fish	2
116	Democratic People's Republic of Korea	1994	Pigmeat	17
116	Democratic People's Republic of Korea	1994	Potatoes	48
116	Democratic People's Republic of Korea	1994	Poultry Meat	3
116	Democratic People's Republic of Korea	1994	Rice (Milled Equivalent)	207
116	Democratic People's Republic of Korea	1994	Rye	7
116	Democratic People's Republic of Korea	1994	Soyabean Oil	6
116	Democratic People's Republic of Korea	1994	Soyabeans	25
116	Democratic People's Republic of Korea	1994	Sugar, Refined Equivalent	9
116	Democratic People's Republic of Korea	1994	Sweet Potatoes	19
116	Democratic People's Republic of Korea	1994	Tomatoes	7
116	Democratic People's Republic of Korea	1994	Vegetables, Other	410
116	Democratic People's Republic of Korea	1994	Wheat	55
116	Democratic People's Republic of Korea	1995	Apples	71
116	Democratic People's Republic of Korea	1995	Barley	12
116	Democratic People's Republic of Korea	1995	Beans	32
116	Democratic People's Republic of Korea	1995	Beer	20
116	Democratic People's Republic of Korea	1995	Beverages, Alcoholic	18
116	Democratic People's Republic of Korea	1995	Bovine Meat	5
116	Democratic People's Republic of Korea	1995	Crustaceans	2
116	Democratic People's Republic of Korea	1995	Demersal Fish	11
116	Democratic People's Republic of Korea	1995	Eggs	9
116	Democratic People's Republic of Korea	1995	Fats, Animals, Raw	1
116	Democratic People's Republic of Korea	1995	Freshwater Fish	3
116	Democratic People's Republic of Korea	1995	Fruits, Other	75
116	Democratic People's Republic of Korea	1995	Maize	127
116	Democratic People's Republic of Korea	1995	Maize Germ Oil	3
116	Democratic People's Republic of Korea	1995	Marine Fish, Other	19
116	Democratic People's Republic of Korea	1995	Milk, Whole	10
116	Democratic People's Republic of Korea	1995	Millet	2
116	Democratic People's Republic of Korea	1995	Molasses	2
116	Democratic People's Republic of Korea	1995	Molluscs, Other	6
116	Democratic People's Republic of Korea	1995	Mutton & Goat Meat	1
116	Democratic People's Republic of Korea	1995	Nuts	1
116	Democratic People's Republic of Korea	1995	Offals, Edible	2
116	Democratic People's Republic of Korea	1995	Onions	9
116	Democratic People's Republic of Korea	1995	Palm Oil	4
116	Democratic People's Republic of Korea	1995	Pigmeat	15
116	Democratic People's Republic of Korea	1995	Potatoes	37
116	Democratic People's Republic of Korea	1995	Poultry Meat	3
116	Democratic People's Republic of Korea	1995	Rice (Milled Equivalent)	216
116	Democratic People's Republic of Korea	1995	Rye	6
116	Democratic People's Republic of Korea	1995	Sorghum	2
116	Democratic People's Republic of Korea	1995	Soyabean Oil	7
116	Democratic People's Republic of Korea	1995	Soyabeans	23
116	Democratic People's Republic of Korea	1995	Sugar, Refined Equivalent	6
116	Democratic People's Republic of Korea	1995	Sweet Potatoes	14
116	Democratic People's Republic of Korea	1995	Tomatoes	7
116	Democratic People's Republic of Korea	1995	Vegetables, Other	362
116	Democratic People's Republic of Korea	1995	Wheat	62
116	Democratic People's Republic of Korea	1996	Apples	84
116	Democratic People's Republic of Korea	1996	Barley	12
116	Democratic People's Republic of Korea	1996	Beans	30
116	Democratic People's Republic of Korea	1996	Beer	17
116	Democratic People's Republic of Korea	1996	Beverages, Alcoholic	18
116	Democratic People's Republic of Korea	1996	Bovine Meat	3
116	Democratic People's Republic of Korea	1996	Cephalopods	1
116	Democratic People's Republic of Korea	1996	Crustaceans	4
116	Democratic People's Republic of Korea	1996	Demersal Fish	2
116	Democratic People's Republic of Korea	1996	Eggs	11
116	Democratic People's Republic of Korea	1996	Fats, Animals, Raw	1
116	Democratic People's Republic of Korea	1996	Freshwater Fish	3
116	Democratic People's Republic of Korea	1996	Fruits, Other	80
116	Democratic People's Republic of Korea	1996	Maize	129
116	Democratic People's Republic of Korea	1996	Maize Germ Oil	3
116	Democratic People's Republic of Korea	1996	Marine Fish, Other	20
116	Democratic People's Republic of Korea	1996	Meat, Other	2
116	Democratic People's Republic of Korea	1996	Milk, Whole	9
116	Democratic People's Republic of Korea	1996	Millet	2
116	Democratic People's Republic of Korea	1996	Molasses	3
116	Democratic People's Republic of Korea	1996	Molluscs, Other	6
116	Democratic People's Republic of Korea	1996	Mutton & Goat Meat	1
116	Democratic People's Republic of Korea	1996	Nuts	1
116	Democratic People's Republic of Korea	1996	Offals, Edible	2
116	Democratic People's Republic of Korea	1996	Onions	7
116	Democratic People's Republic of Korea	1996	Palm Oil	2
116	Democratic People's Republic of Korea	1996	Pigmeat	13
116	Democratic People's Republic of Korea	1996	Potatoes	43
116	Democratic People's Republic of Korea	1996	Poultry Meat	3
116	Democratic People's Republic of Korea	1996	Rice (Milled Equivalent)	216
116	Democratic People's Republic of Korea	1996	Rye	5
116	Democratic People's Republic of Korea	1996	Sorghum	1
116	Democratic People's Republic of Korea	1996	Soyabean Oil	7
116	Democratic People's Republic of Korea	1996	Soyabeans	23
116	Democratic People's Republic of Korea	1996	Sugar, Refined Equivalent	5
116	Democratic People's Republic of Korea	1996	Sweet Potatoes	17
116	Democratic People's Republic of Korea	1996	Tomatoes	7
116	Democratic People's Republic of Korea	1996	Vegetables, Other	360
116	Democratic People's Republic of Korea	1996	Wheat	64
116	Democratic People's Republic of Korea	1997	Apples	84
116	Democratic People's Republic of Korea	1997	Barley	8

FAO country code	Country	Reference year	Food item	Gross food supply (g *capita*$^{-1}$ d^{-1})
116	Democratic People's Republic of Korea	1997	Beans	30
116	Democratic People's Republic of Korea	1997	Beer	13
116	Democratic People's Republic of Korea	1997	Beverages, Alcoholic	18
116	Democratic People's Republic of Korea	1997	Bovine Meat	2
116	Democratic People's Republic of Korea	1997	Cephalopods	2
116	Democratic People's Republic of Korea	1997	Crustaceans	2
116	Democratic People's Republic of Korea	1997	Demersal Fish	9
116	Democratic People's Republic of Korea	1997	Eggs	8
116	Democratic People's Republic of Korea	1997	Fats, Animals, Raw	1
116	Democratic People's Republic of Korea	1997	Freshwater Fish	3
116	Democratic People's Republic of Korea	1997	Fruits, Other	90
116	Democratic People's Republic of Korea	1997	Maize	132
116	Democratic People's Republic of Korea	1997	Maize Germ Oil	3
116	Democratic People's Republic of Korea	1997	Marine Fish, Other	13
116	Democratic People's Republic of Korea	1997	Meat, Other	2
116	Democratic People's Republic of Korea	1997	Milk, Whole	9
116	Democratic People's Republic of Korea	1997	Millet	2
116	Democratic People's Republic of Korea	1997	Molluscs, Other	4
116	Democratic People's Republic of Korea	1997	Mutton & Goat Meat	1
116	Democratic People's Republic of Korea	1997	Nuts	1
116	Democratic People's Republic of Korea	1997	Oats	1
116	Democratic People's Republic of Korea	1997	Offals, Edible	1
116	Democratic People's Republic of Korea	1997	Onions	8
116	Democratic People's Republic of Korea	1997	Palm Oil	3
116	Democratic People's Republic of Korea	1997	Pigmeat	11
116	Democratic People's Republic of Korea	1997	Potatoes	67
116	Democratic People's Republic of Korea	1997	Poultry Meat	3
116	Democratic People's Republic of Korea	1997	Rape and Mustard Oil	1
116	Democratic People's Republic of Korea	1997	Rice (Milled Equivalent)	217
116	Democratic People's Republic of Korea	1997	Rye	4
116	Democratic People's Republic of Korea	1997	Sorghum	5
116	Democratic People's Republic of Korea	1997	Soyabean Oil	7
116	Democratic People's Republic of Korea	1997	Soyabeans	25
116	Democratic People's Republic of Korea	1997	Sugar, Refined Equivalent	5
116	Democratic People's Republic of Korea	1997	Sweet Potatoes	29
116	Democratic People's Republic of Korea	1997	Tomatoes	7
116	Democratic People's Republic of Korea	1997	Vegetables, Other	345
116	Democratic People's Republic of Korea	1997	Wheat	67
116	Democratic People's Republic of Korea	1998	Apples	70
116	Democratic People's Republic of Korea	1998	Barley	7
116	Democratic People's Republic of Korea	1998	Beans	30
116	Democratic People's Republic of Korea	1998	Beer	10
116	Democratic People's Republic of Korea	1998	Beverages, Alcoholic	17
116	Democratic People's Republic of Korea	1998	Bovine Meat	3
116	Democratic People's Republic of Korea	1998	Cephalopods	1
116	Democratic People's Republic of Korea	1998	Crustaceans	2
116	Democratic People's Republic of Korea	1998	Demersal Fish	8
116	Democratic People's Republic of Korea	1998	Eggs	9
116	Democratic People's Republic of Korea	1998	Fats, Animals, Raw	1
116	Democratic People's Republic of Korea	1998	Freshwater Fish	2
116	Democratic People's Republic of Korea	1998	Fruits, Other	76
116	Democratic People's Republic of Korea	1998	Maize	129
116	Democratic People's Republic of Korea	1998	Maize Germ Oil	3
116	Democratic People's Republic of Korea	1998	Marine Fish, Other	13
116	Democratic People's Republic of Korea	1998	Meat, Other	2
116	Democratic People's Republic of Korea	1998	Milk, Whole	10
116	Democratic People's Republic of Korea	1998	Millet	2
116	Democratic People's Republic of Korea	1998	Molluscs, Other	3
116	Democratic People's Republic of Korea	1998	Mutton & Goat Meat	1
116	Democratic People's Republic of Korea	1998	Nuts	1
116	Democratic People's Republic of Korea	1998	Oats	1
116	Democratic People's Republic of Korea	1998	Offals, Edible	2
116	Democratic People's Republic of Korea	1998	Onions	9
116	Democratic People's Republic of Korea	1998	Palm Oil	1
116	Democratic People's Republic of Korea	1998	Pigmeat	14
116	Democratic People's Republic of Korea	1998	Potatoes	105
116	Democratic People's Republic of Korea	1998	Poultry Meat	3
116	Democratic People's Republic of Korea	1998	Rape and Mustard Oil	2
116	Democratic People's Republic of Korea	1998	Rice (Milled Equivalent)	221
116	Democratic People's Republic of Korea	1998	Rye	4
116	Democratic People's Republic of Korea	1998	Sorghum	1
116	Democratic People's Republic of Korea	1998	Soyabean Oil	7
116	Democratic People's Republic of Korea	1998	Soyabeans	18
116	Democratic People's Republic of Korea	1998	Sugar, Refined Equivalent	4
116	Democratic People's Republic of Korea	1998	Sweet Potatoes	40
116	Democratic People's Republic of Korea	1998	Tomatoes	7
116	Democratic People's Republic of Korea	1998	Vegetables, Other	373
116	Democratic People's Republic of Korea	1998	Wheat	64
116	Democratic People's Republic of Korea	1999	Apples	71
116	Democratic People's Republic of Korea	1999	Barley	5
116	Democratic People's Republic of Korea	1999	Beans	30
116	Democratic People's Republic of Korea	1999	Beer	7
116	Democratic People's Republic of Korea	1999	Beverages, Alcoholic	18
116	Democratic People's Republic of Korea	1999	Bovine Meat	2
116	Democratic People's Republic of Korea	1999	Cephalopods	1
116	Democratic People's Republic of Korea	1999	Crustaceans	1
116	Democratic People's Republic of Korea	1999	Demersal Fish	8
116	Democratic People's Republic of Korea	1999	Eggs	11
116	Democratic People's Republic of Korea	1999	Fats, Animals, Raw	1
116	Democratic People's Republic of Korea	1999	Freshwater Fish	2
116	Democratic People's Republic of Korea	1999	Fruits, Other	76
116	Democratic People's Republic of Korea	1999	Maize	125
116	Democratic People's Republic of Korea	1999	Maize Germ Oil	3
116	Democratic People's Republic of Korea	1999	Marine Fish, Other	13
116	Democratic People's Republic of Korea	1999	Meat, Other	3
116	Democratic People's Republic of Korea	1999	Milk, Whole	10
116	Democratic People's Republic of Korea	1999	Millet	3

FAO country code	Country	Reference year	Food item	Gross food supply (g $capita^{-1}$ d^{-1})
116	Democratic People's Republic of Korea	1999	Molasses	3
116	Democratic People's Republic of Korea	1999	Molluscs, Other	2
116	Democratic People's Republic of Korea	1999	Mutton & Goat Meat	1
116	Democratic People's Republic of Korea	1999	Nuts	1
116	Democratic People's Republic of Korea	1999	Oats	1
116	Democratic People's Republic of Korea	1999	Offals, Edible	2
116	Democratic People's Republic of Korea	1999	Onions	9
116	Democratic People's Republic of Korea	1999	Palm Oil	1
116	Democratic People's Republic of Korea	1999	Pigmeat	16
116	Democratic People's Republic of Korea	1999	Potatoes	121
116	Democratic People's Republic of Korea	1999	Poultry Meat	3
116	Democratic People's Republic of Korea	1999	Pulses, Other	2
116	Democratic People's Republic of Korea	1999	Rape and Mustard Oil	1
116	Democratic People's Republic of Korea	1999	Rice (Milled Equivalent)	214
116	Democratic People's Republic of Korea	1999	Rye	5
116	Democratic People's Republic of Korea	1999	Sorghum	1
116	Democratic People's Republic of Korea	1999	Soyabean Oil	6
116	Democratic People's Republic of Korea	1999	Soyabeans	16
116	Democratic People's Republic of Korea	1999	Sugar, Refined Equivalent	6
116	Democratic People's Republic of Korea	1999	Sweet Potatoes	46
116	Democratic People's Republic of Korea	1999	Tomatoes	7
116	Democratic People's Republic of Korea	1999	Vegetables, Other	379
116	Democratic People's Republic of Korea	1999	Wheat	65
116	Democratic People's Republic of Korea	2000	Apples	69
116	Democratic People's Republic of Korea	2000	Barley	5
116	Democratic People's Republic of Korea	2000	Beans	37
116	Democratic People's Republic of Korea	2000	Beer	9
116	Democratic People's Republic of Korea	2000	Beverages, Alcoholic	16
116	Democratic People's Republic of Korea	2000	Bovine Meat	2
116	Democratic People's Republic of Korea	2000	Cephalopods	1
116	Democratic People's Republic of Korea	2000	Crustaceans	1
116	Democratic People's Republic of Korea	2000	Demersal Fish	8
116	Democratic People's Republic of Korea	2000	Eggs	12
116	Democratic People's Republic of Korea	2000	Fats, Animals, Raw	1
116	Democratic People's Republic of Korea	2000	Freshwater Fish	1
116	Democratic People's Republic of Korea	2000	Fruits, Other	75
116	Democratic People's Republic of Korea	2000	Maize	117
116	Democratic People's Republic of Korea	2000	Maize Germ Oil	3
116	Democratic People's Republic of Korea	2000	Marine Fish, Other	13
116	Democratic People's Republic of Korea	2000	Meat, Other	6
116	Democratic People's Republic of Korea	2000	Milk, Whole	10
116	Democratic People's Republic of Korea	2000	Millet	3
116	Democratic People's Republic of Korea	2000	Molluscs, Other	2
116	Democratic People's Republic of Korea	2000	Mutton & Goat Meat	1
116	Democratic People's Republic of Korea	2000	Nuts	1
116	Democratic People's Republic of Korea	2000	Oats	1
116	Democratic People's Republic of Korea	2000	Offals, Edible	2
116	Democratic People's Republic of Korea	2000	Oilcrops Oil, Other	2
116	Democratic People's Republic of Korea	2000	Onions	9
116	Democratic People's Republic of Korea	2000	Pigmeat	17
116	Democratic People's Republic of Korea	2000	Potatoes	152
116	Democratic People's Republic of Korea	2000	Poultry Meat	4
116	Democratic People's Republic of Korea	2000	Pulses, Other	3
116	Democratic People's Republic of Korea	2000	Rape and Mustard Oil	1
116	Democratic People's Republic of Korea	2000	Rice (Milled Equivalent)	208
116	Democratic People's Republic of Korea	2000	Ricebran Oil	1
116	Democratic People's Republic of Korea	2000	Rye	5
116	Democratic People's Republic of Korea	2000	Sorghum	1
116	Democratic People's Republic of Korea	2000	Soyabean Oil	7
116	Democratic People's Republic of Korea	2000	Soyabeans	20
116	Democratic People's Republic of Korea	2000	Sugar, Refined Equivalent	7
116	Democratic People's Republic of Korea	2000	Sweet Potatoes	27
116	Democratic People's Republic of Korea	2000	Tomatoes	7
116	Democratic People's Republic of Korea	2000	Vegetables, Other	391
116	Democratic People's Republic of Korea	2000	Wheat	68
116	Democratic People's Republic of Korea	2001	Apples	71
116	Democratic People's Republic of Korea	2001	Barley	5
116	Democratic People's Republic of Korea	2001	Beans	34
116	Democratic People's Republic of Korea	2001	Beer	8
116	Democratic People's Republic of Korea	2001	Beverages, Alcoholic	12
116	Democratic People's Republic of Korea	2001	Bovine Meat	3
116	Democratic People's Republic of Korea	2001	Cephalopods	1
116	Democratic People's Republic of Korea	2001	Cottonseed Oil	1
116	Democratic People's Republic of Korea	2001	Crustaceans	1
116	Democratic People's Republic of Korea	2001	Demersal Fish	8
116	Democratic People's Republic of Korea	2001	Eggs	13
116	Democratic People's Republic of Korea	2001	Fats, Animals, Raw	1
116	Democratic People's Republic of Korea	2001	Freshwater Fish	1
116	Democratic People's Republic of Korea	2001	Fruits, Other	78
116	Democratic People's Republic of Korea	2001	Maize	93
116	Democratic People's Republic of Korea	2001	Maize Germ Oil	2
116	Democratic People's Republic of Korea	2001	Marine Fish, Other	14
116	Democratic People's Republic of Korea	2001	Meat, Other	11
116	Democratic People's Republic of Korea	2001	Milk, Whole	10
116	Democratic People's Republic of Korea	2001	Millet	4
116	Democratic People's Republic of Korea	2001	Molluscs, Other	1
116	Democratic People's Republic of Korea	2001	Mutton & Goat Meat	1
116	Democratic People's Republic of Korea	2001	Nuts	1
116	Democratic People's Republic of Korea	2001	Offals, Edible	2
116	Democratic People's Republic of Korea	2001	Oilcrops Oil, Other	2
116	Democratic People's Republic of Korea	2001	Onions	9
116	Democratic People's Republic of Korea	2001	Palm Oil	3
116	Democratic People's Republic of Korea	2001	Pigmeat	17
116	Democratic People's Republic of Korea	2001	Potatoes	184
116	Democratic People's Republic of Korea	2001	Poultry Meat	4
116	Democratic People's Republic of Korea	2001	Pulses, Other	2
116	Democratic People's Republic of Korea	2001	Rice (Milled Equivalent)	209

FAO country code	Country	Reference year	Food item	Gross food supply (g $capita^{-1}$ d^{-1})
116	Democratic People's Republic of Korea	2001	Ricebran Oil	1
116	Democratic People's Republic of Korea	2001	Rye	5
116	Democratic People's Republic of Korea	2001	Sorghum	1
116	Democratic People's Republic of Korea	2001	Soyabean Oil	6
116	Democratic People's Republic of Korea	2001	Soyabeans	14
116	Democratic People's Republic of Korea	2001	Sugar, Refined Equivalent	7
116	Democratic People's Republic of Korea	2001	Sweet Potatoes	30
116	Democratic People's Republic of Korea	2001	Tomatoes	7
116	Democratic People's Republic of Korea	2001	Vegetables, Other	391
116	Democratic People's Republic of Korea	2001	Wheat	71
116	Democratic People's Republic of Korea	2002	Apples	68
116	Democratic People's Republic of Korea	2002	Barley	5
116	Democratic People's Republic of Korea	2002	Beans	35
116	Democratic People's Republic of Korea	2002	Beer	8
116	Democratic People's Republic of Korea	2002	Beverages, Alcoholic	15
116	Democratic People's Republic of Korea	2002	Bovine Meat	3
116	Democratic People's Republic of Korea	2002	Cephalopods	1
116	Democratic People's Republic of Korea	2002	Coffee	1
116	Democratic People's Republic of Korea	2002	Crustaceans	1
116	Democratic People's Republic of Korea	2002	Demersal Fish	8
116	Democratic People's Republic of Korea	2002	Eggs	14
116	Democratic People's Republic of Korea	2002	Fats, Animals, Raw	1
116	Democratic People's Republic of Korea	2002	Freshwater Fish	1
116	Democratic People's Republic of Korea	2002	Fruits, Other	81
116	Democratic People's Republic of Korea	2002	Maize	110
116	Democratic People's Republic of Korea	2002	Maize Germ Oil	2
116	Democratic People's Republic of Korea	2002	Marine Fish, Other	10
116	Democratic People's Republic of Korea	2002	Meat, Other	11
116	Democratic People's Republic of Korea	2002	Milk, Whole	12
116	Democratic People's Republic of Korea	2002	Millet	5
116	Democratic People's Republic of Korea	2002	Molluscs, Other	1
116	Democratic People's Republic of Korea	2002	Mutton & Goat Meat	1
116	Democratic People's Republic of Korea	2002	Nuts	2
116	Democratic People's Republic of Korea	2002	Oats	1
116	Democratic People's Republic of Korea	2002	Offals, Edible	2
116	Democratic People's Republic of Korea	2002	Oilcrops Oil, Other	3
116	Democratic People's Republic of Korea	2002	Onions	9
116	Democratic People's Republic of Korea	2002	Palm Oil	1
116	Democratic People's Republic of Korea	2002	Pigmeat	24
116	Democratic People's Republic of Korea	2002	Potatoes	151
116	Democratic People's Republic of Korea	2002	Poultry Meat	5
116	Democratic People's Republic of Korea	2002	Pulses, Other	5
116	Democratic People's Republic of Korea	2002	Rice (Milled Equivalent)	211
116	Democratic People's Republic of Korea	2002	Ricebran Oil	1
116	Democratic People's Republic of Korea	2002	Rye	5
116	Democratic People's Republic of Korea	2002	Sorghum	2
116	Democratic People's Republic of Korea	2002	Soyabean Oil	6
116	Democratic People's Republic of Korea	2002	Soyabeans	16
116	Democratic People's Republic of Korea	2002	Sugar, Refined Equivalent	7
116	Democratic People's Republic of Korea	2002	Sweet Potatoes	31
116	Democratic People's Republic of Korea	2002	Tomatoes	8
116	Democratic People's Republic of Korea	2002	Vegetables, Other	393
116	Democratic People's Republic of Korea	2002	Wheat	74
116	Democratic People's Republic of Korea	2003	Apples	70
116	Democratic People's Republic of Korea	2003	Barley	5
116	Democratic People's Republic of Korea	2003	Beans	32
116	Democratic People's Republic of Korea	2003	Beer	9
116	Democratic People's Republic of Korea	2003	Beverages, Alcoholic	15
116	Democratic People's Republic of Korea	2003	Bovine Meat	3
116	Democratic People's Republic of Korea	2003	Cephalopods	1
116	Democratic People's Republic of Korea	2003	Demersal Fish	8
116	Democratic People's Republic of Korea	2003	Eggs	15
116	Democratic People's Republic of Korea	2003	Fats, Animals, Raw	1
116	Democratic People's Republic of Korea	2003	Freshwater Fish	1
116	Democratic People's Republic of Korea	2003	Fruits, Other	80
116	Democratic People's Republic of Korea	2003	Maize	111
116	Democratic People's Republic of Korea	2003	Maize Germ Oil	3
116	Democratic People's Republic of Korea	2003	Marine Fish, Other	8
116	Democratic People's Republic of Korea	2003	Meat, Other	11
116	Democratic People's Republic of Korea	2003	Milk, Whole	10
116	Democratic People's Republic of Korea	2003	Millet	7
116	Democratic People's Republic of Korea	2003	Molasses	3
116	Democratic People's Republic of Korea	2003	Mutton & Goat Meat	1
116	Democratic People's Republic of Korea	2003	Nuts	2
116	Democratic People's Republic of Korea	2003	Oats	1
116	Democratic People's Republic of Korea	2003	Offals, Edible	2
116	Democratic People's Republic of Korea	2003	Oilcrops Oil, Other	1
116	Democratic People's Republic of Korea	2003	Onions	10
116	Democratic People's Republic of Korea	2003	Palm Oil	1
116	Democratic People's Republic of Korea	2003	Pigmeat	19
116	Democratic People's Republic of Korea	2003	Potatoes	161
116	Democratic People's Republic of Korea	2003	Poultry Meat	5
116	Democratic People's Republic of Korea	2003	Pulses, Other	3
116	Democratic People's Republic of Korea	2003	Rice (Milled Equivalent)	214
116	Democratic People's Republic of Korea	2003	Ricebran Oil	1
116	Democratic People's Republic of Korea	2003	Rye	5
116	Democratic People's Republic of Korea	2003	Sorghum	3
116	Democratic People's Republic of Korea	2003	Soyabean Oil	8
116	Democratic People's Republic of Korea	2003	Soyabeans	12
116	Democratic People's Republic of Korea	2003	Sugar, Refined Equivalent	8
116	Democratic People's Republic of Korea	2003	Sweet Potatoes	32
116	Democratic People's Republic of Korea	2003	Tomatoes	7
116	Democratic People's Republic of Korea	2003	Vegetables, Other	395
116	Democratic People's Republic of Korea	2003	Wheat	77
116	Democratic People's Republic of Korea	2004	Apples	72
116	Democratic People's Republic of Korea	2004	Barley	5
116	Democratic People's Republic of Korea	2004	Beans	34

FAO country code	Country	Reference year	Food item	Gross food supply (g *capita*$^{-1}$ d^{-1})
116	Democratic People's Republic of Korea	2004	Beer	7
116	Democratic People's Republic of Korea	2004	Beverages, Alcoholic	15
116	Democratic People's Republic of Korea	2004	Bovine Meat	2
116	Democratic People's Republic of Korea	2004	Cephalopods	1
116	Democratic People's Republic of Korea	2004	Demersal Fish	8
116	Democratic People's Republic of Korea	2004	Eggs	15
116	Democratic People's Republic of Korea	2004	Fats, Animals, Raw	1
116	Democratic People's Republic of Korea	2004	Freshwater Fish	1
116	Democratic People's Republic of Korea	2004	Fruits, Other	90
116	Democratic People's Republic of Korea	2004	Maize	111
116	Democratic People's Republic of Korea	2004	Maize Germ Oil	3
116	Democratic People's Republic of Korea	2004	Marine Fish, Other	7
116	Democratic People's Republic of Korea	2004	Meat, Other	11
116	Democratic People's Republic of Korea	2004	Milk, Whole	12
116	Democratic People's Republic of Korea	2004	Millet	4
116	Democratic People's Republic of Korea	2004	Molasses	3
116	Democratic People's Republic of Korea	2004	Mutton & Goat Meat	1
116	Democratic People's Republic of Korea	2004	Nuts	2
116	Democratic People's Republic of Korea	2004	Oats	1
116	Democratic People's Republic of Korea	2004	Offals, Edible	2
116	Democratic People's Republic of Korea	2004	Oilcrops Oil, Other	2
116	Democratic People's Republic of Korea	2004	Onions	10
116	Democratic People's Republic of Korea	2004	Palm Oil	1
116	Democratic People's Republic of Korea	2004	Pigmeat	19
116	Democratic People's Republic of Korea	2004	Potatoes	162
116	Democratic People's Republic of Korea	2004	Poultry Meat	5
116	Democratic People's Republic of Korea	2004	Pulses, Other	1
116	Democratic People's Republic of Korea	2004	Rice (Milled Equivalent)	215
116	Democratic People's Republic of Korea	2004	Ricebran Oil	1
116	Democratic People's Republic of Korea	2004	Rye	5
116	Democratic People's Republic of Korea	2004	Sorghum	3
116	Democratic People's Republic of Korea	2004	Soyabean Oil	8
116	Democratic People's Republic of Korea	2004	Soyabeans	14
116	Democratic People's Republic of Korea	2004	Sugar, Refined Equivalent	8
116	Democratic People's Republic of Korea	2004	Sweet Potatoes	33
116	Democratic People's Republic of Korea	2004	Tomatoes	8
116	Democratic People's Republic of Korea	2004	Vegetables, Other	390
116	Democratic People's Republic of Korea	2004	Wheat	77
116	Democratic People's Republic of Korea	2005	Apples	71
116	Democratic People's Republic of Korea	2005	Barley	5
116	Democratic People's Republic of Korea	2005	Beans	33
116	Democratic People's Republic of Korea	2005	Beer	7
116	Democratic People's Republic of Korea	2005	Beverages, Alcoholic	16
116	Democratic People's Republic of Korea	2005	Bovine Meat	2
116	Democratic People's Republic of Korea	2005	Cephalopods	1
116	Democratic People's Republic of Korea	2005	Crustaceans	1
116	Democratic People's Republic of Korea	2005	Demersal Fish	8
116	Democratic People's Republic of Korea	2005	Eggs	14
116	Democratic People's Republic of Korea	2005	Fats, Animals, Raw	1
116	Democratic People's Republic of Korea	2005	Freshwater Fish	1
116	Democratic People's Republic of Korea	2005	Fruits, Other	78
116	Democratic People's Republic of Korea	2005	Maize	114
116	Democratic People's Republic of Korea	2005	Maize Germ Oil	3
116	Democratic People's Republic of Korea	2005	Marine Fish, Other	12
116	Democratic People's Republic of Korea	2005	Meat, Other	11
116	Democratic People's Republic of Korea	2005	Milk, Whole	14
116	Democratic People's Republic of Korea	2005	Millet	4
116	Democratic People's Republic of Korea	2005	Molasses	1
116	Democratic People's Republic of Korea	2005	Mutton & Goat Meat	1
116	Democratic People's Republic of Korea	2005	Nuts	2
116	Democratic People's Republic of Korea	2005	Oats	1
116	Democratic People's Republic of Korea	2005	Offals, Edible	2
116	Democratic People's Republic of Korea	2005	Onions	10
116	Democratic People's Republic of Korea	2005	Palm Oil	1
116	Democratic People's Republic of Korea	2005	Pigmeat	20
116	Democratic People's Republic of Korea	2005	Potatoes	162
116	Democratic People's Republic of Korea	2005	Poultry Meat	5
116	Democratic People's Republic of Korea	2005	Rice (Milled Equivalent)	215
116	Democratic People's Republic of Korea	2005	Ricebran Oil	2
116	Democratic People's Republic of Korea	2005	Rye	5
116	Democratic People's Republic of Korea	2005	Sorghum	3
116	Democratic People's Republic of Korea	2005	Soyabean Oil	9
116	Democratic People's Republic of Korea	2005	Soyabeans	11
116	Democratic People's Republic of Korea	2005	Sugar, Refined Equivalent	6
116	Democratic People's Republic of Korea	2005	Sweet Potatoes	31
116	Democratic People's Republic of Korea	2005	Tomatoes	8
116	Democratic People's Republic of Korea	2005	Vegetables, Other	392
116	Democratic People's Republic of Korea	2005	Wheat	77
116	Democratic People's Republic of Korea	2006	Apples	70
116	Democratic People's Republic of Korea	2006	Barley	5
116	Democratic People's Republic of Korea	2006	Beans	33
116	Democratic People's Republic of Korea	2006	Beer	8
116	Democratic People's Republic of Korea	2006	Beverages, Alcoholic	16
116	Democratic People's Republic of Korea	2006	Bovine Meat	2
116	Democratic People's Republic of Korea	2006	Cephalopods	1
116	Democratic People's Republic of Korea	2006	Coffee	1
116	Democratic People's Republic of Korea	2006	Crustaceans	2
116	Democratic People's Republic of Korea	2006	Demersal Fish	8
116	Democratic People's Republic of Korea	2006	Eggs	12
116	Democratic People's Republic of Korea	2006	Fats, Animals, Raw	1
116	Democratic People's Republic of Korea	2006	Freshwater Fish	1
116	Democratic People's Republic of Korea	2006	Fruits, Other	78
116	Democratic People's Republic of Korea	2006	Maize	116
116	Democratic People's Republic of Korea	2006	Maize Germ Oil	3
116	Democratic People's Republic of Korea	2006	Marine Fish, Other	12
116	Democratic People's Republic of Korea	2006	Meat, Other	12
116	Democratic People's Republic of Korea	2006	Milk, Whole	10

FAO country code	Country	Reference year	Food item	Gross food supply (g *capita*$^{-1}$ d^{-1})
116	Democratic People's Republic of Korea	2006	Millet	5
116	Democratic People's Republic of Korea	2006	Molluscs, Other	1
116	Democratic People's Republic of Korea	2006	Mutton & Goat Meat	2
116	Democratic People's Republic of Korea	2006	Nuts	1
116	Democratic People's Republic of Korea	2006	Oats	1
116	Democratic People's Republic of Korea	2006	Offals, Edible	2
116	Democratic People's Republic of Korea	2006	Onions	9
116	Democratic People's Republic of Korea	2006	Pigmeat	17
116	Democratic People's Republic of Korea	2006	Potatoes	155
116	Democratic People's Republic of Korea	2006	Poultry Meat	5
116	Democratic People's Republic of Korea	2006	Rice (Milled Equivalent)	213
116	Democratic People's Republic of Korea	2006	Ricebran Oil	1
116	Democratic People's Republic of Korea	2006	Rye	5
116	Democratic People's Republic of Korea	2006	Sorghum	3
116	Democratic People's Republic of Korea	2006	Soyabean Oil	9
116	Democratic People's Republic of Korea	2006	Soyabeans	9
116	Democratic People's Republic of Korea	2006	Sugar, Refined Equivalent	7
116	Democratic People's Republic of Korea	2006	Sweet Potatoes	32
116	Democratic People's Republic of Korea	2006	Tomatoes	7
116	Democratic People's Republic of Korea	2006	Vegetables, Other	378
116	Democratic People's Republic of Korea	2006	Wheat	73
116	Democratic People's Republic of Korea	2007	Apples	66
116	Democratic People's Republic of Korea	2007	Barley	5
116	Democratic People's Republic of Korea	2007	Beans	31
116	Democratic People's Republic of Korea	2007	Beer	8
116	Democratic People's Republic of Korea	2007	Beverages, Alcoholic	15
116	Democratic People's Republic of Korea	2007	Bovine Meat	2
116	Democratic People's Republic of Korea	2007	Cephalopods	1
116	Democratic People's Republic of Korea	2007	Coffee	1
116	Democratic People's Republic of Korea	2007	Cottonseed Oil	1
116	Democratic People's Republic of Korea	2007	Crustaceans	3
116	Democratic People's Republic of Korea	2007	Demersal Fish	8
116	Democratic People's Republic of Korea	2007	Eggs	11
116	Democratic People's Republic of Korea	2007	Fats, Animals, Raw	1
116	Democratic People's Republic of Korea	2007	Freshwater Fish	1
116	Democratic People's Republic of Korea	2007	Fruits, Other	77
116	Democratic People's Republic of Korea	2007	Maize	112
116	Democratic People's Republic of Korea	2007	Maize Germ Oil	3
116	Democratic People's Republic of Korea	2007	Marine Fish, Other	12
116	Democratic People's Republic of Korea	2007	Meat, Other	13
116	Democratic People's Republic of Korea	2007	Milk, Whole	10
116	Democratic People's Republic of Korea	2007	Millet	4
116	Democratic People's Republic of Korea	2007	Molluscs, Other	7
116	Democratic People's Republic of Korea	2007	Mutton & Goat Meat	2
116	Democratic People's Republic of Korea	2007	Nuts	2
116	Democratic People's Republic of Korea	2007	Oats	1
116	Democratic People's Republic of Korea	2007	Offals, Edible	2
116	Democratic People's Republic of Korea	2007	Onions	9
116	Democratic People's Republic of Korea	2007	Palm Oil	1
116	Democratic People's Republic of Korea	2007	Pigmeat	15
116	Democratic People's Republic of Korea	2007	Potatoes	146
116	Democratic People's Republic of Korea	2007	Poultry Meat	5
116	Democratic People's Republic of Korea	2007	Rice (Milled Equivalent)	209
116	Democratic People's Republic of Korea	2007	Ricebran Oil	1
116	Democratic People's Republic of Korea	2007	Rye	5
116	Democratic People's Republic of Korea	2007	Sorghum	3
116	Democratic People's Republic of Korea	2007	Soyabean Oil	9
116	Democratic People's Republic of Korea	2007	Soyabeans	11
116	Democratic People's Republic of Korea	2007	Sugar, Refined Equivalent	12
116	Democratic People's Republic of Korea	2007	Sweet Potatoes	33
116	Democratic People's Republic of Korea	2007	Tomatoes	7
116	Democratic People's Republic of Korea	2007	Vegetables, Other	365
116	Democratic People's Republic of Korea	2007	Wheat	71
116	Democratic People's Republic of Korea	2008	Apples	70
116	Democratic People's Republic of Korea	2008	Barley	4
116	Democratic People's Republic of Korea	2008	Beans	33
116	Democratic People's Republic of Korea	2008	Beer	6
116	Democratic People's Republic of Korea	2008	Beverages, Alcoholic	16
116	Democratic People's Republic of Korea	2008	Bovine Meat	2
116	Democratic People's Republic of Korea	2008	Cephalopods	1
116	Democratic People's Republic of Korea	2008	Coffee	1
116	Democratic People's Republic of Korea	2008	Crustaceans	3
116	Democratic People's Republic of Korea	2008	Demersal Fish	8
116	Democratic People's Republic of Korea	2008	Eggs	11
116	Democratic People's Republic of Korea	2008	Fats, Animals, Raw	1
116	Democratic People's Republic of Korea	2008	Freshwater Fish	1
116	Democratic People's Republic of Korea	2008	Fruits, Other	77
116	Democratic People's Republic of Korea	2008	Maize	114
116	Democratic People's Republic of Korea	2008	Maize Germ Oil	3
116	Democratic People's Republic of Korea	2008	Marine Fish, Other	12
116	Democratic People's Republic of Korea	2008	Meat, Other	14
116	Democratic People's Republic of Korea	2008	Milk, Whole	10
116	Democratic People's Republic of Korea	2008	Millet	4
116	Democratic People's Republic of Korea	2008	Molluscs, Other	6
116	Democratic People's Republic of Korea	2008	Mutton & Goat Meat	2
116	Democratic People's Republic of Korea	2008	Nuts	2
116	Democratic People's Republic of Korea	2008	Oats	1
116	Democratic People's Republic of Korea	2008	Offals, Edible	2
116	Democratic People's Republic of Korea	2008	Onions	8
116	Democratic People's Republic of Korea	2008	Palm Oil	1
116	Democratic People's Republic of Korea	2008	Pigmeat	13
116	Democratic People's Republic of Korea	2008	Potatoes	115
116	Democratic People's Republic of Korea	2008	Poultry Meat	5
116	Democratic People's Republic of Korea	2008	Rice (Milled Equivalent)	206
116	Democratic People's Republic of Korea	2008	Ricebran Oil	1
116	Democratic People's Republic of Korea	2008	Rye	6
116	Democratic People's Republic of Korea	2008	Sorghum	3

FAO country code	Country	Reference year	Food item	Gross food supply (g *capita*$^{-1}$ d^{-1})
116	Democratic People's Republic of Korea	2008	Soyabean Oil	10
116	Democratic People's Republic of Korea	2008	Soyabeans	11
116	Democratic People's Republic of Korea	2008	Sugar, Refined Equivalent	14
116	Democratic People's Republic of Korea	2008	Sweet Potatoes	34
116	Democratic People's Republic of Korea	2008	Tomatoes	6
116	Democratic People's Republic of Korea	2008	Vegetables, Other	384
116	Democratic People's Republic of Korea	2008	Wheat	68
116	Democratic People's Republic of Korea	2009	Apples	74
116	Democratic People's Republic of Korea	2009	Barley	4
116	Democratic People's Republic of Korea	2009	Beans	33
116	Democratic People's Republic of Korea	2009	Beer	6
116	Democratic People's Republic of Korea	2009	Beverages, Alcoholic	17
116	Democratic People's Republic of Korea	2009	Bovine Meat	2
116	Democratic People's Republic of Korea	2009	Cephalopods	1
116	Democratic People's Republic of Korea	2009	Coffee	1
116	Democratic People's Republic of Korea	2009	Crustaceans	3
116	Democratic People's Republic of Korea	2009	Demersal Fish	9
116	Democratic People's Republic of Korea	2009	Eggs	11
116	Democratic People's Republic of Korea	2009	Fats, Animals, Raw	1
116	Democratic People's Republic of Korea	2009	Freshwater Fish	1
116	Democratic People's Republic of Korea	2009	Fruits, Other	82
116	Democratic People's Republic of Korea	2009	Maize	122
116	Democratic People's Republic of Korea	2009	Maize Germ Oil	3
116	Democratic People's Republic of Korea	2009	Marine Fish, Other	12
116	Democratic People's Republic of Korea	2009	Meat, Other	15
116	Democratic People's Republic of Korea	2009	Milk, Whole	10
116	Democratic People's Republic of Korea	2009	Millet	4
116	Democratic People's Republic of Korea	2009	Molluscs, Other	3
116	Democratic People's Republic of Korea	2009	Mutton & Goat Meat	2
116	Democratic People's Republic of Korea	2009	Nuts	2
116	Democratic People's Republic of Korea	2009	Oats	1
116	Democratic People's Republic of Korea	2009	Offals, Edible	2
116	Democratic People's Republic of Korea	2009	Onions	9
116	Democratic People's Republic of Korea	2009	Palm Oil	1
116	Democratic People's Republic of Korea	2009	Pigmeat	12
116	Democratic People's Republic of Korea	2009	Potatoes	118
116	Democratic People's Republic of Korea	2009	Poultry Meat	5
116	Democratic People's Republic of Korea	2009	Rice (Milled Equivalent)	204
116	Democratic People's Republic of Korea	2009	Ricebran Oil	1
116	Democratic People's Republic of Korea	2009	Rye	5
116	Democratic People's Republic of Korea	2009	Sorghum	3
116	Democratic People's Republic of Korea	2009	Soyabean Oil	9
116	Democratic People's Republic of Korea	2009	Soyabeans	9
116	Democratic People's Republic of Korea	2009	Sugar, Refined Equivalent	12
116	Democratic People's Republic of Korea	2009	Sweet Potatoes	34
116	Democratic People's Republic of Korea	2009	Tomatoes	7
116	Democratic People's Republic of Korea	2009	Vegetables, Other	384
116	Democratic People's Republic of Korea	2009	Wheat	64
116	Democratic People's Republic of Korea	2010	Apples	76
116	Democratic People's Republic of Korea	2010	Barley	5
116	Democratic People's Republic of Korea	2010	Beans	30
116	Democratic People's Republic of Korea	2010	Beer	7
116	Democratic People's Republic of Korea	2010	Beverages, Alcoholic	16
116	Democratic People's Republic of Korea	2010	Bovine Meat	2
116	Democratic People's Republic of Korea	2010	Cephalopods	1
116	Democratic People's Republic of Korea	2010	Coffee	1
116	Democratic People's Republic of Korea	2010	Crustaceans	2
116	Democratic People's Republic of Korea	2010	Demersal Fish	9
116	Democratic People's Republic of Korea	2010	Eggs	12
116	Democratic People's Republic of Korea	2010	Fats, Animals, Raw	1
116	Democratic People's Republic of Korea	2010	Freshwater Fish	1
116	Democratic People's Republic of Korea	2010	Fruits, Other	81
116	Democratic People's Republic of Korea	2010	Maize	127
116	Democratic People's Republic of Korea	2010	Maize Germ Oil	3
116	Democratic People's Republic of Korea	2010	Marine Fish, Other	11
116	Democratic People's Republic of Korea	2010	Meat, Other	15
116	Democratic People's Republic of Korea	2010	Milk, Whole	10
116	Democratic People's Republic of Korea	2010	Millet	5
116	Democratic People's Republic of Korea	2010	Molluscs, Other	1
116	Democratic People's Republic of Korea	2010	Mutton & Goat Meat	2
116	Democratic People's Republic of Korea	2010	Nuts	2
116	Democratic People's Republic of Korea	2010	Oats	1
116	Democratic People's Republic of Korea	2010	Offals, Edible	2
116	Democratic People's Republic of Korea	2010	Onions	9
116	Democratic People's Republic of Korea	2010	Palm Oil	1
116	Democratic People's Republic of Korea	2010	Pigmeat	13
116	Democratic People's Republic of Korea	2010	Potatoes	129
116	Democratic People's Republic of Korea	2010	Poultry Meat	5
116	Democratic People's Republic of Korea	2010	Rice (Milled Equivalent)	203
116	Democratic People's Republic of Korea	2010	Ricebran Oil	1
116	Democratic People's Republic of Korea	2010	Rye	5
116	Democratic People's Republic of Korea	2010	Sorghum	3
116	Democratic People's Republic of Korea	2010	Soyabean Oil	9
116	Democratic People's Republic of Korea	2010	Soyabeans	9
116	Democratic People's Republic of Korea	2010	Sugar, Refined Equivalent	12
116	Democratic People's Republic of Korea	2010	Sweet Potatoes	37
116	Democratic People's Republic of Korea	2010	Tomatoes	7
116	Democratic People's Republic of Korea	2010	Vegetables, Other	366
116	Democratic People's Republic of Korea	2010	Wheat	62
116	Democratic People's Republic of Korea	2011	Apples	76
116	Democratic People's Republic of Korea	2011	Barley	4
116	Democratic People's Republic of Korea	2011	Beans	34
116	Democratic People's Republic of Korea	2011	Beer	7
116	Democratic People's Republic of Korea	2011	Beverages, Alcoholic	17
116	Democratic People's Republic of Korea	2011	Bovine Meat	2
116	Democratic People's Republic of Korea	2011	Cephalopods	1
116	Democratic People's Republic of Korea	2011	Crustaceans	2

FAO country code	Country	Reference year	Food item	Gross food supply (g $capita^{-1}$ d^{-1})
116	Democratic People's Republic of Korea	2011	Demersal Fish	10
116	Democratic People's Republic of Korea	2011	Eggs	12
116	Democratic People's Republic of Korea	2011	Fats, Animals, Raw	1
116	Democratic People's Republic of Korea	2011	Freshwater Fish	1
116	Democratic People's Republic of Korea	2011	Fruits, Other	81
116	Democratic People's Republic of Korea	2011	Maize	124
116	Democratic People's Republic of Korea	2011	Maize Germ Oil	3
116	Democratic People's Republic of Korea	2011	Marine Fish, Other	11
116	Democratic People's Republic of Korea	2011	Meat, Other	17
116	Democratic People's Republic of Korea	2011	Milk, Whole	10
116	Democratic People's Republic of Korea	2011	Millet	5
116	Democratic People's Republic of Korea	2011	Molluscs, Other	1
116	Democratic People's Republic of Korea	2011	Mutton & Goat Meat	2
116	Democratic People's Republic of Korea	2011	Nuts	2
116	Democratic People's Republic of Korea	2011	Oats	1
116	Democratic People's Republic of Korea	2011	Offals, Edible	2
116	Democratic People's Republic of Korea	2011	Onions	9
116	Democratic People's Republic of Korea	2011	Palm Oil	1
116	Democratic People's Republic of Korea	2011	Pigmeat	12
116	Democratic People's Republic of Korea	2011	Potatoes	132
116	Democratic People's Republic of Korea	2011	Poultry Meat	5
116	Democratic People's Republic of Korea	2011	Rice (Milled Equivalent)	205
116	Democratic People's Republic of Korea	2011	Ricebran Oil	1
116	Democratic People's Republic of Korea	2011	Rye	5
116	Democratic People's Republic of Korea	2011	Sorghum	3
116	Democratic People's Republic of Korea	2011	Soyabean Oil	9
116	Democratic People's Republic of Korea	2011	Soyabeans	9
116	Democratic People's Republic of Korea	2011	Sugar, Refined Equivalent	10
116	Democratic People's Republic of Korea	2011	Sweet Potatoes	38
116	Democratic People's Republic of Korea	2011	Tomatoes	6
116	Democratic People's Republic of Korea	2011	Vegetables, Other	319
116	Democratic People's Republic of Korea	2011	Wheat	64
54	Denmark	1992	Apples	68
54	Denmark	1992	Bananas	29
54	Denmark	1992	Beer	332
54	Denmark	1992	Beverages, Alcoholic	8
54	Denmark	1992	Bovine Meat	58
54	Denmark	1992	Butter, Ghee	8
54	Denmark	1992	Cheese	42
54	Denmark	1992	Cocoa Beans	9
54	Denmark	1992	Coffee	30
54	Denmark	1992	Cream	26
54	Denmark	1992	Crustaceans	9
54	Denmark	1992	Dates	1
54	Denmark	1992	Demersal Fish	18
54	Denmark	1992	Eggs	42
54	Denmark	1992	Fats, Animals, Raw	34
54	Denmark	1992	Fish, Body Oil	14
54	Denmark	1992	Freshwater Fish	6
54	Denmark	1992	Fruits, Other	49
54	Denmark	1992	Grapefruit	3
54	Denmark	1992	Grapes	20
54	Denmark	1992	Honey	1
54	Denmark	1992	Lemons, Limes	6
54	Denmark	1992	Maize	34
54	Denmark	1992	Maize Germ Oil	1
54	Denmark	1992	Meat, Other	2
54	Denmark	1992	Milk, Whole	98
54	Denmark	1992	Molluscs, Other	6
54	Denmark	1992	Mutton & Goat Meat	3
54	Denmark	1992	Nuts	10
54	Denmark	1992	Oats	7
54	Denmark	1992	Offals, Edible	32
54	Denmark	1992	Oilcrops Oil, Other	1
54	Denmark	1992	Olive Oil	1
54	Denmark	1992	Olives	1
54	Denmark	1992	Onions	22
54	Denmark	1992	Oranges, Mandarines	27
54	Denmark	1992	Palmkernel Oil	6
54	Denmark	1992	Peas	2
54	Denmark	1992	Pelagic Fish	27
54	Denmark	1992	Pigmeat	178
54	Denmark	1992	Pineapples	3
54	Denmark	1992	Potatoes	206
54	Denmark	1992	Poultry Meat	40
54	Denmark	1992	Rape and Mustard Oil	1
54	Denmark	1992	Rice (Milled Equivalent)	3
54	Denmark	1992	Rye	46
54	Denmark	1992	Soyabean Oil	14
54	Denmark	1992	Spices, Other	1
54	Denmark	1992	Sugar, Refined Equivalent	110
54	Denmark	1992	Sunflowerseed Oil	2
54	Denmark	1992	Sweet Potatoes	8
54	Denmark	1992	Sweeteners, Other	14
54	Denmark	1992	Tea	1
54	Denmark	1992	Tomatoes	36
54	Denmark	1992	Vegetables, Other	162
54	Denmark	1992	Wheat	177
54	Denmark	1992	Wine	68
54	Denmark	1993	Apples	63
54	Denmark	1993	Bananas	23
54	Denmark	1993	Beer	329
54	Denmark	1993	Beverages, Alcoholic	7
54	Denmark	1993	Bovine Meat	56
54	Denmark	1993	Butter, Ghee	7
54	Denmark	1993	Cheese	44
54	Denmark	1993	Cocoa Beans	10

FAO country code	Country	Reference year	Food item	Gross food supply (g *capita*$^{-1}$ d^{-1})
54	Denmark	1993	Coffee	28
54	Denmark	1993	Cream	20
54	Denmark	1993	Crustaceans	7
54	Denmark	1993	Demersal Fish	25
54	Denmark	1993	Eggs	42
54	Denmark	1993	Fats, Animals, Raw	34
54	Denmark	1993	Fish, Body Oil	14
54	Denmark	1993	Freshwater Fish	4
54	Denmark	1993	Fruits, Other	36
54	Denmark	1993	Grapefruit	3
54	Denmark	1993	Grapes	20
54	Denmark	1993	Honey	1
54	Denmark	1993	Lemons, Limes	4
54	Denmark	1993	Maize	36
54	Denmark	1993	Maize Germ Oil	1
54	Denmark	1993	Meat, Other	3
54	Denmark	1993	Milk, Whole	113
54	Denmark	1993	Molluscs, Other	5
54	Denmark	1993	Mutton & Goat Meat	3
54	Denmark	1993	Nuts	10
54	Denmark	1993	Oats	10
54	Denmark	1993	Offals, Edible	35
54	Denmark	1993	Oilcrops Oil, Other	1
54	Denmark	1993	Olive Oil	1
54	Denmark	1993	Onions	25
54	Denmark	1993	Oranges, Mandarines	30
54	Denmark	1993	Palmkernel Oil	2
54	Denmark	1993	Peas	2
54	Denmark	1993	Pelagic Fish	23
54	Denmark	1993	Pepper	1
54	Denmark	1993	Pigmeat	178
54	Denmark	1993	Pineapples	4
54	Denmark	1993	Potatoes	208
54	Denmark	1993	Poultry Meat	36
54	Denmark	1993	Rape and Mustard Oil	2
54	Denmark	1993	Rice (Milled Equivalent)	4
54	Denmark	1993	Rye	44
54	Denmark	1993	Soyabean Oil	15
54	Denmark	1993	Spices, Other	1
54	Denmark	1993	Sugar, Refined Equivalent	114
54	Denmark	1993	Sunflowerseed Oil	2
54	Denmark	1993	Sweet Potatoes	6
54	Denmark	1993	Sweeteners, Other	16
54	Denmark	1993	Tea	1
54	Denmark	1993	Tomatoes	33
54	Denmark	1993	Vegetables, Other	149
54	Denmark	1993	Wheat	186
54	Denmark	1993	Wine	62
54	Denmark	1994	Apples	40
54	Denmark	1994	Bananas	19
54	Denmark	1994	Beer	332
54	Denmark	1994	Beverages, Alcoholic	7
54	Denmark	1994	Bovine Meat	53
54	Denmark	1994	Butter, Ghee	6
54	Denmark	1994	Cheese	39
54	Denmark	1994	Cocoa Beans	10
54	Denmark	1994	Coffee	29
54	Denmark	1994	Cream	20
54	Denmark	1994	Crustaceans	17
54	Denmark	1994	Dates	1
54	Denmark	1994	Demersal Fish	13
54	Denmark	1994	Eggs	43
54	Denmark	1994	Fats, Animals, Raw	34
54	Denmark	1994	Fish, Body Oil	14
54	Denmark	1994	Freshwater Fish	9
54	Denmark	1994	Fruits, Other	40
54	Denmark	1994	Grapefruit	3
54	Denmark	1994	Grapes	21
54	Denmark	1994	Groundnut Oil	1
54	Denmark	1994	Honey	1
54	Denmark	1994	Lemons, Limes	4
54	Denmark	1994	Maize	33
54	Denmark	1994	Maize Germ Oil	1
54	Denmark	1994	Meat, Other	3
54	Denmark	1994	Milk, Whole	134
54	Denmark	1994	Molluscs, Other	6
54	Denmark	1994	Mutton & Goat Meat	3
54	Denmark	1994	Nuts	9
54	Denmark	1994	Oats	12
54	Denmark	1994	Offals, Edible	30
54	Denmark	1994	Oilcrops Oil, Other	1
54	Denmark	1994	Olive Oil	1
54	Denmark	1994	Onions	27
54	Denmark	1994	Oranges, Mandarines	39
54	Denmark	1994	Palmkernel Oil	3
54	Denmark	1994	Peas	2
54	Denmark	1994	Pelagic Fish	25
54	Denmark	1994	Pepper	1
54	Denmark	1994	Pigmeat	174
54	Denmark	1994	Pineapples	3
54	Denmark	1994	Potatoes	199
54	Denmark	1994	Poultry Meat	42
54	Denmark	1994	Rape and Mustard Oil	1
54	Denmark	1994	Rice (Milled Equivalent)	4
54	Denmark	1994	Rye	44
54	Denmark	1994	Soyabean Oil	11
54	Denmark	1994	Spices, Other	1

FAO country code	Country	Reference year	Food item	Gross food supply (g $capita^{-1}$ d^{-1})
54	Denmark	1994	Sugar, Refined Equivalent	115
54	Denmark	1994	Sunflowerseed Oil	2
54	Denmark	1994	Sweet Potatoes	3
54	Denmark	1994	Sweeteners, Other	16
54	Denmark	1994	Tea	1
54	Denmark	1994	Tomatoes	39
54	Denmark	1994	Vegetables, Other	145
54	Denmark	1994	Wheat	190
54	Denmark	1994	Whey	1
54	Denmark	1994	Wine	64
54	Denmark	1995	Apples	48
54	Denmark	1995	Bananas	20
54	Denmark	1995	Beer	329
54	Denmark	1995	Beverages, Alcoholic	7
54	Denmark	1995	Bovine Meat	50
54	Denmark	1995	Butter, Ghee	6
54	Denmark	1995	Cheese	47
54	Denmark	1995	Cocoa Beans	10
54	Denmark	1995	Coffee	24
54	Denmark	1995	Cream	21
54	Denmark	1995	Crustaceans	16
54	Denmark	1995	Dates	1
54	Denmark	1995	Demersal Fish	9
54	Denmark	1995	Eggs	44
54	Denmark	1995	Fats, Animals, Raw	34
54	Denmark	1995	Fish, Body Oil	13
54	Denmark	1995	Freshwater Fish	12
54	Denmark	1995	Fruits, Other	39
54	Denmark	1995	Grapefruit	2
54	Denmark	1995	Grapes	18
54	Denmark	1995	Honey	1
54	Denmark	1995	Lemons, Limes	4
54	Denmark	1995	Maize	38
54	Denmark	1995	Maize Germ Oil	1
54	Denmark	1995	Meat, Other	3
54	Denmark	1995	Milk, Whole	224
54	Denmark	1995	Molluscs, Other	6
54	Denmark	1995	Mutton & Goat Meat	3
54	Denmark	1995	Nuts	9
54	Denmark	1995	Oats	13
54	Denmark	1995	Offals, Edible	23
54	Denmark	1995	Oilcrops Oil, Other	1
54	Denmark	1995	Olives	1
54	Denmark	1995	Onions	25
54	Denmark	1995	Oranges, Mandarines	51
54	Denmark	1995	Palmkernel Oil	7
54	Denmark	1995	Peas	2
54	Denmark	1995	Pelagic Fish	26
54	Denmark	1995	Pepper	1
54	Denmark	1995	Pigmeat	177
54	Denmark	1995	Pineapples	4
54	Denmark	1995	Potatoes	203
54	Denmark	1995	Poultry Meat	46
54	Denmark	1995	Rape and Mustard Oil	1
54	Denmark	1995	Rice (Milled Equivalent)	3
54	Denmark	1995	Rye	42
54	Denmark	1995	Soyabean Oil	13
54	Denmark	1995	Spices, Other	1
54	Denmark	1995	Sugar, Refined Equivalent	111
54	Denmark	1995	Sunflowerseed Oil	1
54	Denmark	1995	Sweeteners, Other	16
54	Denmark	1995	Tea	1
54	Denmark	1995	Tomatoes	35
54	Denmark	1995	Vegetables, Other	136
54	Denmark	1995	Wheat	204
54	Denmark	1995	Wine	68
54	Denmark	1996	Apples	42
54	Denmark	1996	Bananas	27
54	Denmark	1996	Beer	323
54	Denmark	1996	Beverages, Alcoholic	8
54	Denmark	1996	Beverages, Fermented	1
54	Denmark	1996	Bovine Meat	53
54	Denmark	1996	Butter, Ghee	6
54	Denmark	1996	Cheese	47
54	Denmark	1996	Cocoa Beans	10
54	Denmark	1996	Coconuts - Incl Copra	1
54	Denmark	1996	Coffee	27
54	Denmark	1996	Cream	22
54	Denmark	1996	Crustaceans	16
54	Denmark	1996	Dates	1
54	Denmark	1996	Demersal Fish	9
54	Denmark	1996	Eggs	37
54	Denmark	1996	Fats, Animals, Raw	32
54	Denmark	1996	Fish, Body Oil	14
54	Denmark	1996	Freshwater Fish	12
54	Denmark	1996	Fruits, Other	42
54	Denmark	1996	Grapefruit	2
54	Denmark	1996	Grapes	20
54	Denmark	1996	Groundnut Oil	1
54	Denmark	1996	Honey	1
54	Denmark	1996	Lemons, Limes	3
54	Denmark	1996	Maize	27
54	Denmark	1996	Maize Germ Oil	1
54	Denmark	1996	Meat, Other	3
54	Denmark	1996	Milk, Whole	197
54	Denmark	1996	Molluscs, Other	11
54	Denmark	1996	Mutton & Goat Meat	2

FAO country code	Country	Reference year	Food item	Gross food supply (g *capita*$^{-1}$ d^{-1})
54	Denmark	1996	Nuts	10
54	Denmark	1996	Oats	12
54	Denmark	1996	Offals, Edible	22
54	Denmark	1996	Oilcrops Oil, Other	1
54	Denmark	1996	Olive Oil	1
54	Denmark	1996	Olives	1
54	Denmark	1996	Onions	26
54	Denmark	1996	Oranges, Mandarines	60
54	Denmark	1996	Peas	2
54	Denmark	1996	Pelagic Fish	16
54	Denmark	1996	Pepper	1
54	Denmark	1996	Pigmeat	179
54	Denmark	1996	Pineapples	4
54	Denmark	1996	Potatoes	210
54	Denmark	1996	Poultry Meat	44
54	Denmark	1996	Rape and Mustard Oil	4
54	Denmark	1996	Rice (Milled Equivalent)	3
54	Denmark	1996	Rye	44
54	Denmark	1996	Soyabean Oil	11
54	Denmark	1996	Spices, Other	1
54	Denmark	1996	Sugar, Refined Equivalent	106
54	Denmark	1996	Sweeteners, Other	16
54	Denmark	1996	Tea	1
54	Denmark	1996	Tomatoes	35
54	Denmark	1996	Vegetables, Other	148
54	Denmark	1996	Wheat	203
54	Denmark	1996	Whey	1
54	Denmark	1996	Wine	77
54	Denmark	1997	Apples	58
54	Denmark	1997	Bananas	26
54	Denmark	1997	Beer	312
54	Denmark	1997	Beverages, Alcoholic	8
54	Denmark	1997	Beverages, Fermented	1
54	Denmark	1997	Bovine Meat	54
54	Denmark	1997	Butter, Ghee	5
54	Denmark	1997	Cheese	38
54	Denmark	1997	Cocoa Beans	14
54	Denmark	1997	Coconuts - Incl Copra	2
54	Denmark	1997	Coffee	25
54	Denmark	1997	Cream	22
54	Denmark	1997	Crustaceans	12
54	Denmark	1997	Dates	1
54	Denmark	1997	Demersal Fish	14
54	Denmark	1997	Eggs	44
54	Denmark	1997	Fats, Animals, Raw	32
54	Denmark	1997	Fish, Body Oil	14
54	Denmark	1997	Freshwater Fish	4
54	Denmark	1997	Fruits, Other	49
54	Denmark	1997	Grapefruit	2
54	Denmark	1997	Grapes	23
54	Denmark	1997	Honey	1
54	Denmark	1997	Lemons, Limes	5
54	Denmark	1997	Maize	27
54	Denmark	1997	Maize Germ Oil	1
54	Denmark	1997	Meat, Other	3
54	Denmark	1997	Milk, Whole	160
54	Denmark	1997	Molluscs, Other	13
54	Denmark	1997	Mutton & Goat Meat	2
54	Denmark	1997	Nuts	11
54	Denmark	1997	Oats	11
54	Denmark	1997	Offals, Edible	12
54	Denmark	1997	Oilcrops Oil, Other	2
54	Denmark	1997	Olive Oil	1
54	Denmark	1997	Olives	1
54	Denmark	1997	Onions	27
54	Denmark	1997	Oranges, Mandarines	88
54	Denmark	1997	Palmkernel Oil	5
54	Denmark	1997	Peas	2
54	Denmark	1997	Pelagic Fish	21
54	Denmark	1997	Pepper	1
54	Denmark	1997	Pigmeat	159
54	Denmark	1997	Pineapples	4
54	Denmark	1997	Potatoes	217
54	Denmark	1997	Poultry Meat	50
54	Denmark	1997	Rice (Milled Equivalent)	3
54	Denmark	1997	Rye	45
54	Denmark	1997	Sesameseed Oil	1
54	Denmark	1997	Soyabean Oil	7
54	Denmark	1997	Spices, Other	1
54	Denmark	1997	Sugar, Refined Equivalent	115
54	Denmark	1997	Sunflowerseed Oil	2
54	Denmark	1997	Sweeteners, Other	20
54	Denmark	1997	Tea	1
54	Denmark	1997	Tomatoes	44
54	Denmark	1997	Vegetables, Other	164
54	Denmark	1997	Wheat	225
54	Denmark	1997	Whey	1
54	Denmark	1997	Wine	86
54	Denmark	1998	Apples	41
54	Denmark	1998	Bananas	21
54	Denmark	1998	Beer	288
54	Denmark	1998	Beverages, Alcoholic	8
54	Denmark	1998	Beverages, Fermented	2
54	Denmark	1998	Bovine Meat	59
54	Denmark	1998	Butter, Ghee	5
54	Denmark	1998	Cheese	39
54	Denmark	1998	Cocoa Beans	16

FAO countrycode	Country	Reference year	Food item	Gross food supply (g *capita*$^{-1}$ d^{-1})
54	Denmark	1998	Coconuts - Incl Copra	2
54	Denmark	1998	Coffee	26
54	Denmark	1998	Cream	24
54	Denmark	1998	Crustaceans	8
54	Denmark	1998	Dates	1
54	Denmark	1998	Demersal Fish	14
54	Denmark	1998	Eggs	42
54	Denmark	1998	Fats, Animals, Raw	32
54	Denmark	1998	Fish, Body Oil	14
54	Denmark	1998	Freshwater Fish	5
54	Denmark	1998	Fruits, Other	36
54	Denmark	1998	Grapefruit	2
54	Denmark	1998	Grapes	20
54	Denmark	1998	Honey	3
54	Denmark	1998	Lemons, Limes	3
54	Denmark	1998	Maize	23
54	Denmark	1998	Maize Germ Oil	1
54	Denmark	1998	Meat, Other	2
54	Denmark	1998	Milk, Whole	165
54	Denmark	1998	Molluscs, Other	19
54	Denmark	1998	Mutton & Goat Meat	3
54	Denmark	1998	Nuts	10
54	Denmark	1998	Oats	17
54	Denmark	1998	Offals, Edible	15
54	Denmark	1998	Oilcrops Oil, Other	2
54	Denmark	1998	Olive Oil	1
54	Denmark	1998	Olives	1
54	Denmark	1998	Onions	25
54	Denmark	1998	Oranges, Mandarines	82
54	Denmark	1998	Palmkernel Oil	3
54	Denmark	1998	Peas	2
54	Denmark	1998	Pelagic Fish	17
54	Denmark	1998	Pigmeat	188
54	Denmark	1998	Pineapples	4
54	Denmark	1998	Potatoes	222
54	Denmark	1998	Poultry Meat	45
54	Denmark	1998	Rape and Mustard Oil	2
54	Denmark	1998	Rice (Milled Equivalent)	5
54	Denmark	1998	Rye	42
54	Denmark	1998	Sesameseed Oil	1
54	Denmark	1998	Soyabean Oil	7
54	Denmark	1998	Spices, Other	1
54	Denmark	1998	Sugar, Refined Equivalent	101
54	Denmark	1998	Sunflowerseed Oil	3
54	Denmark	1998	Sweeteners, Other	26
54	Denmark	1998	Tea	1
54	Denmark	1998	Tomatoes	39
54	Denmark	1998	Vegetables, Other	157
54	Denmark	1998	Wheat	235
54	Denmark	1998	Wine	85
54	Denmark	1999	Apples	68
54	Denmark	1999	Bananas	31
54	Denmark	1999	Beer	279
54	Denmark	1999	Beverages, Alcoholic	8
54	Denmark	1999	Beverages, Fermented	2
54	Denmark	1999	Bovine Meat	69
54	Denmark	1999	Butter, Ghee	5
54	Denmark	1999	Cheese	40
54	Denmark	1999	Cocoa Beans	16
54	Denmark	1999	Coconuts - Incl Copra	2
54	Denmark	1999	Coffee	26
54	Denmark	1999	Cream	26
54	Denmark	1999	Crustaceans	14
54	Denmark	1999	Dates	1
54	Denmark	1999	Demersal Fish	14
54	Denmark	1999	Eggs	40
54	Denmark	1999	Fats, Animals, Raw	35
54	Denmark	1999	Fish, Body Oil	14
54	Denmark	1999	Freshwater Fish	5
54	Denmark	1999	Fruits, Other	53
54	Denmark	1999	Grapefruit	3
54	Denmark	1999	Grapes	20
54	Denmark	1999	Honey	1
54	Denmark	1999	Lemons, Limes	5
54	Denmark	1999	Maize	14
54	Denmark	1999	Maize Germ Oil	1
54	Denmark	1999	Meat, Other	2
54	Denmark	1999	Milk, Whole	181
54	Denmark	1999	Molluscs, Other	14
54	Denmark	1999	Mutton & Goat Meat	3
54	Denmark	1999	Nuts	10
54	Denmark	1999	Oats	14
54	Denmark	1999	Offals, Edible	42
54	Denmark	1999	Oilcrops Oil, Other	3
54	Denmark	1999	Olive Oil	1
54	Denmark	1999	Olives	1
54	Denmark	1999	Onions	28
54	Denmark	1999	Oranges, Mandarines	74
54	Denmark	1999	Palmkernel Oil	2
54	Denmark	1999	Peas	4
54	Denmark	1999	Pelagic Fish	16
54	Denmark	1999	Pepper	1
54	Denmark	1999	Pigmeat	65
54	Denmark	1999	Pineapples	4
54	Denmark	1999	Potatoes	215
54	Denmark	1999	Poultry Meat	48
54	Denmark	1999	Rape and Mustard Oil	3

FAO country code	Country	Reference year	Food item	Gross food supply (g *capita*$^{-1}$ d^{-1})
54	Denmark	1999	Rice (Milled Equivalent)	11
54	Denmark	1999	Rye	39
54	Denmark	1999	Sesameseed Oil	1
54	Denmark	1999	Soyabean Oil	12
54	Denmark	1999	Spices, Other	1
54	Denmark	1999	Sugar, Refined Equivalent	93
54	Denmark	1999	Sunflowerseed Oil	2
54	Denmark	1999	Sweeteners, Other	34
54	Denmark	1999	Tea	1
54	Denmark	1999	Tomatoes	47
54	Denmark	1999	Vegetables, Other	179
54	Denmark	1999	Wheat	237
54	Denmark	1999	Wine	83
54	Denmark	2000	Apples	80
54	Denmark	2000	Aquatic Animals, Others	1
54	Denmark	2000	Bananas	31
54	Denmark	2000	Beans	1
54	Denmark	2000	Beer	273
54	Denmark	2000	Beverages, Alcoholic	8
54	Denmark	2000	Beverages, Fermented	2
54	Denmark	2000	Bovine Meat	65
54	Denmark	2000	Butter, Ghee	4
54	Denmark	2000	Cheese	45
54	Denmark	2000	Cocoa Beans	16
54	Denmark	2000	Coconuts - Incl Copra	3
54	Denmark	2000	Coffee	23
54	Denmark	2000	Cream	25
54	Denmark	2000	Crustaceans	12
54	Denmark	2000	Dates	1
54	Denmark	2000	Demersal Fish	4
54	Denmark	2000	Eggs	38
54	Denmark	2000	Fats, Animals, Raw	30
54	Denmark	2000	Fish, Body Oil	8
54	Denmark	2000	Freshwater Fish	9
54	Denmark	2000	Fruits, Other	52
54	Denmark	2000	Grapefruit	3
54	Denmark	2000	Grapes	22
54	Denmark	2000	Honey	2
54	Denmark	2000	Lemons, Limes	5
54	Denmark	2000	Maize	8
54	Denmark	2000	Maize Germ Oil	1
54	Denmark	2000	Meat, Other	2
54	Denmark	2000	Milk, Whole	184
54	Denmark	2000	Molluscs, Other	19
54	Denmark	2000	Mutton & Goat Meat	3
54	Denmark	2000	Nuts	12
54	Denmark	2000	Oats	14
54	Denmark	2000	Offals, Edible	34
54	Denmark	2000	Oilcrops Oil, Other	2
54	Denmark	2000	Olive Oil	1
54	Denmark	2000	Olives	2
54	Denmark	2000	Onions	20
54	Denmark	2000	Oranges, Mandarines	83
54	Denmark	2000	Palm Oil	4
54	Denmark	2000	Palmkernel Oil	4
54	Denmark	2000	Peas	2
54	Denmark	2000	Pelagic Fish	16
54	Denmark	2000	Pepper	1
54	Denmark	2000	Pigmeat	74
54	Denmark	2000	Pineapples	5
54	Denmark	2000	Potatoes	218
54	Denmark	2000	Poultry Meat	48
54	Denmark	2000	Rape and Mustard Oil	5
54	Denmark	2000	Rice (Milled Equivalent)	9
54	Denmark	2000	Rye	36
54	Denmark	2000	Soyabean Oil	13
54	Denmark	2000	Spices, Other	1
54	Denmark	2000	Sugar, Refined Equivalent	101
54	Denmark	2000	Sunflowerseed Oil	4
54	Denmark	2000	Sweeteners, Other	35
54	Denmark	2000	Tea	1
54	Denmark	2000	Tomatoes	47
54	Denmark	2000	Vegetables, Other	178
54	Denmark	2000	Wheat	227
54	Denmark	2000	Wine	86
54	Denmark	2001	Apples	81
54	Denmark	2001	Bananas	30
54	Denmark	2001	Beer	264
54	Denmark	2001	Beverages, Alcoholic	8
54	Denmark	2001	Beverages, Fermented	2
54	Denmark	2001	Bovine Meat	67
54	Denmark	2001	Butter, Ghee	4
54	Denmark	2001	Cheese	59
54	Denmark	2001	Cocoa Beans	12
54	Denmark	2001	Coconuts - Incl Copra	3
54	Denmark	2001	Coffee	26
54	Denmark	2001	Cream	28
54	Denmark	2001	Crustaceans	6
54	Denmark	2001	Dates	1
54	Denmark	2001	Demersal Fish	6
54	Denmark	2001	Eggs	44
54	Denmark	2001	Fats, Animals, Raw	28
54	Denmark	2001	Fish, Body Oil	11
54	Denmark	2001	Freshwater Fish	6
54	Denmark	2001	Fruits, Other	48
54	Denmark	2001	Grapefruit	3
54	Denmark	2001	Grapes	21

FAO country code	Country	Reference year	Food item	Gross food supply (g $capita^{-1}$ d^{-1})
54	Denmark	2001	Honey	2
54	Denmark	2001	Lemons, Limes	5
54	Denmark	2001	Maize	8
54	Denmark	2001	Maize Germ Oil	1
54	Denmark	2001	Meat, Other	2
54	Denmark	2001	Milk, Whole	106
54	Denmark	2001	Molluscs, Other	23
54	Denmark	2001	Mutton & Goat Meat	3
54	Denmark	2001	Nuts	13
54	Denmark	2001	Oats	14
54	Denmark	2001	Offals, Edible	51
54	Denmark	2001	Oilcrops Oil, Other	2
54	Denmark	2001	Olive Oil	1
54	Denmark	2001	Olives	2
54	Denmark	2001	Onions	19
54	Denmark	2001	Oranges, Mandarines	63
54	Denmark	2001	Palm Oil	3
54	Denmark	2001	Peas	2
54	Denmark	2001	Pelagic Fish	18
54	Denmark	2001	Pigmeat	72
54	Denmark	2001	Pineapples	6
54	Denmark	2001	Potatoes	223
54	Denmark	2001	Poultry Meat	56
54	Denmark	2001	Rape and Mustard Oil	7
54	Denmark	2001	Rice (Milled Equivalent)	9
54	Denmark	2001	Rye	41
54	Denmark	2001	Soyabean Oil	13
54	Denmark	2001	Spices, Other	1
54	Denmark	2001	Sugar, Refined Equivalent	96
54	Denmark	2001	Sunflowerseed Oil	3
54	Denmark	2001	Sweeteners, Other	36
54	Denmark	2001	Tea	1
54	Denmark	2001	Tomatoes	46
54	Denmark	2001	Vegetables, Other	181
54	Denmark	2001	Wheat	270
54	Denmark	2001	Wine	93
54	Denmark	2002	Apples	132
54	Denmark	2002	Bananas	39
54	Denmark	2002	Beer	259
54	Denmark	2002	Beverages, Alcoholic	10
54	Denmark	2002	Beverages, Fermented	1
54	Denmark	2002	Bovine Meat	75
54	Denmark	2002	Butter, Ghee	5
54	Denmark	2002	Cheese	53
54	Denmark	2002	Cocoa Beans	9
54	Denmark	2002	Coconuts - Incl Copra	3
54	Denmark	2002	Coffee	24
54	Denmark	2002	Cream	30
54	Denmark	2002	Crustaceans	9
54	Denmark	2002	Dates	1
54	Denmark	2002	Demersal Fish	4
54	Denmark	2002	Eggs	45
54	Denmark	2002	Fats, Animals, Raw	23
54	Denmark	2002	Fish, Body Oil	11
54	Denmark	2002	Freshwater Fish	3
54	Denmark	2002	Fruits, Other	51
54	Denmark	2002	Grapefruit	2
54	Denmark	2002	Grapes	24
54	Denmark	2002	Honey	1
54	Denmark	2002	Lemons, Limes	5
54	Denmark	2002	Maize	8
54	Denmark	2002	Maize Germ Oil	1
54	Denmark	2002	Meat, Other	2
54	Denmark	2002	Milk, Whole	99
54	Denmark	2002	Molluscs, Other	18
54	Denmark	2002	Mutton & Goat Meat	3
54	Denmark	2002	Nuts	11
54	Denmark	2002	Oats	16
54	Denmark	2002	Offals, Edible	46
54	Denmark	2002	Oilcrops Oil, Other	2
54	Denmark	2002	Olive Oil	1
54	Denmark	2002	Olives	2
54	Denmark	2002	Onions	19
54	Denmark	2002	Oranges, Mandarines	183
54	Denmark	2002	Palmkernel Oil	2
54	Denmark	2002	Peas	2
54	Denmark	2002	Pelagic Fish	22
54	Denmark	2002	Pepper	1
54	Denmark	2002	Pigmeat	94
54	Denmark	2002	Pineapples	13
54	Denmark	2002	Potatoes	211
54	Denmark	2002	Poultry Meat	55
54	Denmark	2002	Rape and Mustard Oil	8
54	Denmark	2002	Rice (Milled Equivalent)	10
54	Denmark	2002	Rye	39
54	Denmark	2002	Soyabean Oil	9
54	Denmark	2002	Spices, Other	1
54	Denmark	2002	Sugar, Refined Equivalent	110
54	Denmark	2002	Sunflowerseed Oil	2
54	Denmark	2002	Sweeteners, Other	37
54	Denmark	2002	Tea	1
54	Denmark	2002	Tomatoes	51
54	Denmark	2002	Vegetables, Other	190
54	Denmark	2002	Wheat	297
54	Denmark	2002	Whey	13
54	Denmark	2002	Wine	89
54	Denmark	2003	Apples	144

FAO country code	Country	Reference year	Food item	Gross food supply (g *capita*$^{-1}$ d^{-1})
54	Denmark	2003	Bananas	39
54	Denmark	2003	Beer	258
54	Denmark	2003	Beverages, Alcoholic	13
54	Denmark	2003	Beverages, Fermented	1
54	Denmark	2003	Bovine Meat	78
54	Denmark	2003	Butter, Ghee	5
54	Denmark	2003	Cheese	55
54	Denmark	2003	Cocoa Beans	9
54	Denmark	2003	Coconuts - Incl Copra	3
54	Denmark	2003	Coffee	22
54	Denmark	2003	Cream	31
54	Denmark	2003	Crustaceans	10
54	Denmark	2003	Dates	1
54	Denmark	2003	Demersal Fish	9
54	Denmark	2003	Eggs	48
54	Denmark	2003	Fats, Animals, Raw	22
54	Denmark	2003	Fish, Body Oil	12
54	Denmark	2003	Freshwater Fish	4
54	Denmark	2003	Fruits, Other	54
54	Denmark	2003	Grapefruit	2
54	Denmark	2003	Grapes	24
54	Denmark	2003	Honey	1
54	Denmark	2003	Lemons, Limes	5
54	Denmark	2003	Maize	7
54	Denmark	2003	Maize Germ Oil	1
54	Denmark	2003	Meat, Other	2
54	Denmark	2003	Milk, Whole	40
54	Denmark	2003	Molluscs, Other	18
54	Denmark	2003	Mutton & Goat Meat	3
54	Denmark	2003	Nuts	12
54	Denmark	2003	Oats	16
54	Denmark	2003	Offals, Edible	41
54	Denmark	2003	Oilcrops Oil, Other	1
54	Denmark	2003	Olive Oil	2
54	Denmark	2003	Olives	2
54	Denmark	2003	Onions	22
54	Denmark	2003	Oranges, Mandarines	99
54	Denmark	2003	Palm Oil	2
54	Denmark	2003	Palmkernel Oil	3
54	Denmark	2003	Peas	2
54	Denmark	2003	Pelagic Fish	20
54	Denmark	2003	Pigmeat	79
54	Denmark	2003	Pineapples	8
54	Denmark	2003	Plantains	1
54	Denmark	2003	Potatoes	210
54	Denmark	2003	Poultry Meat	50
54	Denmark	2003	Rape and Mustard Oil	3
54	Denmark	2003	Rice (Milled Equivalent)	12
54	Denmark	2003	Rye	35
54	Denmark	2003	Soyabean Oil	13
54	Denmark	2003	Spices, Other	2
54	Denmark	2003	Sugar, Refined Equivalent	111
54	Denmark	2003	Sunflowerseed Oil	2
54	Denmark	2003	Sweeteners, Other	36
54	Denmark	2003	Tea	1
54	Denmark	2003	Tomatoes	50
54	Denmark	2003	Vegetables, Other	201
54	Denmark	2003	Wheat	298
54	Denmark	2003	Whey	12
54	Denmark	2003	Wine	89
54	Denmark	2004	Apples	122
54	Denmark	2004	Bananas	37
54	Denmark	2004	Beer	242
54	Denmark	2004	Beverages, Alcoholic	13
54	Denmark	2004	Bovine Meat	79
54	Denmark	2004	Butter, Ghee	5
54	Denmark	2004	Cheese	52
54	Denmark	2004	Cocoa Beans	12
54	Denmark	2004	Coconuts - Incl Copra	2
54	Denmark	2004	Coffee	25
54	Denmark	2004	Cream	32
54	Denmark	2004	Crustaceans	13
54	Denmark	2004	Dates	1
54	Denmark	2004	Demersal Fish	9
54	Denmark	2004	Eggs	55
54	Denmark	2004	Fats, Animals, Raw	23
54	Denmark	2004	Fish, Body Oil	12
54	Denmark	2004	Freshwater Fish	7
54	Denmark	2004	Fruits, Other	64
54	Denmark	2004	Grapefruit	3
54	Denmark	2004	Grapes	22
54	Denmark	2004	Honey	2
54	Denmark	2004	Lemons, Limes	5
54	Denmark	2004	Maize	7
54	Denmark	2004	Maize Germ Oil	1
54	Denmark	2004	Meat, Other	2
54	Denmark	2004	Milk, Whole	84
54	Denmark	2004	Molluscs, Other	10
54	Denmark	2004	Mutton & Goat Meat	3
54	Denmark	2004	Nuts	13
54	Denmark	2004	Oats	17
54	Denmark	2004	Offals, Edible	44
54	Denmark	2004	Oilcrops Oil, Other	3
54	Denmark	2004	Olive Oil	2
54	Denmark	2004	Olives	2
54	Denmark	2004	Onions	22
54	Denmark	2004	Oranges, Mandarines	103

FAO country code	Country	Reference year	Food item	Gross food supply (g *capita*$^{-1}$ d^{-1})
54	Denmark	2004	Palm Oil	12
54	Denmark	2004	Peas	2
54	Denmark	2004	Pelagic Fish	25
54	Denmark	2004	Pepper	1
54	Denmark	2004	Pigmeat	67
54	Denmark	2004	Pineapples	10
54	Denmark	2004	Plantains	3
54	Denmark	2004	Potatoes	207
54	Denmark	2004	Poultry Meat	46
54	Denmark	2004	Rape and Mustard Oil	5
54	Denmark	2004	Rice (Milled Equivalent)	12
54	Denmark	2004	Rye	37
54	Denmark	2004	Soyabean Oil	6
54	Denmark	2004	Spices, Other	2
54	Denmark	2004	Sugar, Refined Equivalent	112
54	Denmark	2004	Sunflowerseed Oil	2
54	Denmark	2004	Sweeteners, Other	35
54	Denmark	2004	Tea	1
54	Denmark	2004	Tomatoes	57
54	Denmark	2004	Vegetables, Other	202
54	Denmark	2004	Wheat	280
54	Denmark	2004	Whey	27
54	Denmark	2004	Wine	84
54	Denmark	2005	Apples	136
54	Denmark	2005	Bananas	33
54	Denmark	2005	Beer	240
54	Denmark	2005	Beverages, Alcoholic	14
54	Denmark	2005	Bovine Meat	74
54	Denmark	2005	Butter, Ghee	5
54	Denmark	2005	Cheese	72
54	Denmark	2005	Citrus, Other	1
54	Denmark	2005	Cocoa Beans	18
54	Denmark	2005	Coconuts - Incl Copra	3
54	Denmark	2005	Coffee	24
54	Denmark	2005	Cream	28
54	Denmark	2005	Crustaceans	17
54	Denmark	2005	Dates	1
54	Denmark	2005	Demersal Fish	8
54	Denmark	2005	Eggs	52
54	Denmark	2005	Fats, Animals, Raw	26
54	Denmark	2005	Fish, Body Oil	12
54	Denmark	2005	Freshwater Fish	12
54	Denmark	2005	Fruits, Other	60
54	Denmark	2005	Grapefruit	2
54	Denmark	2005	Grapes	23
54	Denmark	2005	Honey	2
54	Denmark	2005	Lemons, Limes	5
54	Denmark	2005	Maize	7
54	Denmark	2005	Maize Germ Oil	2
54	Denmark	2005	Meat, Other	2
54	Denmark	2005	Milk, Whole	89
54	Denmark	2005	Molluscs, Other	7
54	Denmark	2005	Mutton & Goat Meat	3
54	Denmark	2005	Nuts	11
54	Denmark	2005	Oats	19
54	Denmark	2005	Offals, Edible	33
54	Denmark	2005	Oilcrops Oil, Other	5
54	Denmark	2005	Olive Oil	1
54	Denmark	2005	Olives	2
54	Denmark	2005	Onions	27
54	Denmark	2005	Oranges, Mandarines	102
54	Denmark	2005	Palm Oil	3
54	Denmark	2005	Palmkernel Oil	2
54	Denmark	2005	Peas	2
54	Denmark	2005	Pelagic Fish	23
54	Denmark	2005	Pigmeat	121
54	Denmark	2005	Pineapples	10
54	Denmark	2005	Plantains	1
54	Denmark	2005	Potatoes	208
54	Denmark	2005	Poultry Meat	54
54	Denmark	2005	Rape and Mustard Oil	2
54	Denmark	2005	Rice (Milled Equivalent)	14
54	Denmark	2005	Rye	36
54	Denmark	2005	Soyabean Oil	6
54	Denmark	2005	Spices, Other	1
54	Denmark	2005	Sugar, Refined Equivalent	94
54	Denmark	2005	Sweeteners, Other	34
54	Denmark	2005	Tea	2
54	Denmark	2005	Tomatoes	48
54	Denmark	2005	Vegetables, Other	193
54	Denmark	2005	Wheat	291
54	Denmark	2005	Whey	31
54	Denmark	2005	Wine	77
54	Denmark	2006	Apples	96
54	Denmark	2006	Bananas	36
54	Denmark	2006	Beer	234
54	Denmark	2006	Beverages, Alcoholic	12
54	Denmark	2006	Beverages, Fermented	2
54	Denmark	2006	Bovine Meat	71
54	Denmark	2006	Butter, Ghee	5
54	Denmark	2006	Cheese	67
54	Denmark	2006	Citrus, Other	1
54	Denmark	2006	Cocoa Beans	11
54	Denmark	2006	Coconuts - Incl Copra	3
54	Denmark	2006	Coffee	23
54	Denmark	2006	Cream	20
54	Denmark	2006	Crustaceans	14

FAO country code	Country	Reference year	Food item	Gross food supply (g $capita^{-1}$ d^{-1})
54	Denmark	2006	Dates	1
54	Denmark	2006	Demersal Fish	12
54	Denmark	2006	Eggs	53
54	Denmark	2006	Fats, Animals, Raw	23
54	Denmark	2006	Fish, Body Oil	12
54	Denmark	2006	Freshwater Fish	9
54	Denmark	2006	Fruits, Other	64
54	Denmark	2006	Grapefruit	2
54	Denmark	2006	Grapes	23
54	Denmark	2006	Honey	2
54	Denmark	2006	Lemons, Limes	5
54	Denmark	2006	Maize	6
54	Denmark	2006	Maize Germ Oil	1
54	Denmark	2006	Meat, Other	3
54	Denmark	2006	Milk, Whole	98
54	Denmark	2006	Molluscs, Other	4
54	Denmark	2006	Mutton & Goat Meat	3
54	Denmark	2006	Nuts	12
54	Denmark	2006	Oats	22
54	Denmark	2006	Offals, Edible	35
54	Denmark	2006	Oilcrops Oil, Other	4
54	Denmark	2006	Olive Oil	1
54	Denmark	2006	Olives	2
54	Denmark	2006	Onions	27
54	Denmark	2006	Oranges, Mandarines	108
54	Denmark	2006	Palm Oil	4
54	Denmark	2006	Peas	2
54	Denmark	2006	Pelagic Fish	24
54	Denmark	2006	Pepper	1
54	Denmark	2006	Pigmeat	88
54	Denmark	2006	Pimento	1
54	Denmark	2006	Pineapples	12
54	Denmark	2006	Plantains	2
54	Denmark	2006	Potatoes	230
54	Denmark	2006	Poultry Meat	52
54	Denmark	2006	Rape and Mustard Oil	1
54	Denmark	2006	Rice (Milled Equivalent)	17
54	Denmark	2006	Rye	35
54	Denmark	2006	Soyabean Oil	10
54	Denmark	2006	Spices, Other	2
54	Denmark	2006	Sugar, Refined Equivalent	104
54	Denmark	2006	Sunflowerseed Oil	1
54	Denmark	2006	Sweeteners, Other	33
54	Denmark	2006	Tea	1
54	Denmark	2006	Tomatoes	49
54	Denmark	2006	Vegetables, Other	196
54	Denmark	2006	Wheat	288
54	Denmark	2006	Whey	26
54	Denmark	2006	Wine	78
54	Denmark	2007	Apples	57
54	Denmark	2007	Bananas	39
54	Denmark	2007	Beer	227
54	Denmark	2007	Beverages, Alcoholic	12
54	Denmark	2007	Beverages, Fermented	3
54	Denmark	2007	Bovine Meat	72
54	Denmark	2007	Butter, Ghee	5
54	Denmark	2007	Cheese	58
54	Denmark	2007	Citrus, Other	2
54	Denmark	2007	Cocoa Beans	12
54	Denmark	2007	Coconuts - Incl Copra	4
54	Denmark	2007	Coffee	21
54	Denmark	2007	Cream	30
54	Denmark	2007	Crustaceans	11
54	Denmark	2007	Dates	1
54	Denmark	2007	Demersal Fish	12
54	Denmark	2007	Eggs	52
54	Denmark	2007	Fats, Animals, Raw	19
54	Denmark	2007	Fish, Body Oil	12
54	Denmark	2007	Freshwater Fish	6
54	Denmark	2007	Fruits, Other	59
54	Denmark	2007	Grapefruit	2
54	Denmark	2007	Grapes	25
54	Denmark	2007	Honey	2
54	Denmark	2007	Lemons, Limes	4
54	Denmark	2007	Maize	17
54	Denmark	2007	Maize Germ Oil	1
54	Denmark	2007	Meat, Other	6
54	Denmark	2007	Milk, Whole	85
54	Denmark	2007	Molluscs, Other	5
54	Denmark	2007	Mutton & Goat Meat	3
54	Denmark	2007	Nuts	11
54	Denmark	2007	Oats	21
54	Denmark	2007	Offals, Edible	19
54	Denmark	2007	Oilcrops Oil, Other	3
54	Denmark	2007	Olive Oil	2
54	Denmark	2007	Olives	2
54	Denmark	2007	Onions	27
54	Denmark	2007	Oranges, Mandarines	105
54	Denmark	2007	Palm Oil	4
54	Denmark	2007	Peas	2
54	Denmark	2007	Pelagic Fish	26
54	Denmark	2007	Pepper	1
54	Denmark	2007	Pigmeat	110
54	Denmark	2007	Pineapples	11
54	Denmark	2007	Plantains	1
54	Denmark	2007	Potatoes	223
54	Denmark	2007	Poultry Meat	55

FAO country code	Country	Reference year	Food item	Gross food supply (g $capita^{-1}$ d^{-1})
54	Denmark	2007	Rape and Mustard Oil	2
54	Denmark	2007	Rice (Milled Equivalent)	14
54	Denmark	2007	Rye	46
54	Denmark	2007	Soyabean Oil	9
54	Denmark	2007	Spices, Other	1
54	Denmark	2007	Sugar, Refined Equivalent	124
54	Denmark	2007	Sunflowerseed Oil	1
54	Denmark	2007	Sweeteners, Other	36
54	Denmark	2007	Tea	1
54	Denmark	2007	Tomatoes	46
54	Denmark	2007	Vegetables, Other	193
54	Denmark	2007	Wheat	257
54	Denmark	2007	Whey	27
54	Denmark	2007	Wine	78
54	Denmark	2008	Apples	57
54	Denmark	2008	Bananas	43
54	Denmark	2008	Beer	215
54	Denmark	2008	Beverages, Alcoholic	12
54	Denmark	2008	Beverages, Fermented	9
54	Denmark	2008	Bovine Meat	75
54	Denmark	2008	Butter, Ghee	5
54	Denmark	2008	Cheese	64
54	Denmark	2008	Citrus, Other	7
54	Denmark	2008	Cocoa Beans	10
54	Denmark	2008	Coconuts - Incl Copra	4
54	Denmark	2008	Coffee	22
54	Denmark	2008	Cream	28
54	Denmark	2008	Crustaceans	21
54	Denmark	2008	Dates	1
54	Denmark	2008	Demersal Fish	7
54	Denmark	2008	Eggs	52
54	Denmark	2008	Fats, Animals, Raw	25
54	Denmark	2008	Fish, Body Oil	10
54	Denmark	2008	Freshwater Fish	11
54	Denmark	2008	Fruits, Other	51
54	Denmark	2008	Grapefruit	2
54	Denmark	2008	Grapes	27
54	Denmark	2008	Groundnut Oil	1
54	Denmark	2008	Groundnuts (Shelled Eq)	1
54	Denmark	2008	Honey	2
54	Denmark	2008	Lemons, Limes	5
54	Denmark	2008	Maize	7
54	Denmark	2008	Meat, Other	3
54	Denmark	2008	Milk, Whole	106
54	Denmark	2008	Molluscs, Other	4
54	Denmark	2008	Mutton & Goat Meat	3
54	Denmark	2008	Nuts	11
54	Denmark	2008	Oats	28
54	Denmark	2008	Offals, Edible	27
54	Denmark	2008	Oilcrops Oil, Other	2
54	Denmark	2008	Olive Oil	2
54	Denmark	2008	Olives	2
54	Denmark	2008	Onions	27
54	Denmark	2008	Oranges, Mandarines	100
54	Denmark	2008	Palm Oil	4
54	Denmark	2008	Peas	2
54	Denmark	2008	Pelagic Fish	19
54	Denmark	2008	Pigmeat	72
54	Denmark	2008	Pineapples	13
54	Denmark	2008	Potatoes	204
54	Denmark	2008	Poultry Meat	62
54	Denmark	2008	Rape and Mustard Oil	3
54	Denmark	2008	Rice (Milled Equivalent)	13
54	Denmark	2008	Rye	35
54	Denmark	2008	Soyabean Oil	5
54	Denmark	2008	Spices, Other	1
54	Denmark	2008	Sugar, Refined Equivalent	105
54	Denmark	2008	Sunflowerseed Oil	2
54	Denmark	2008	Sweeteners, Other	30
54	Denmark	2008	Tea	1
54	Denmark	2008	Tomatoes	49
54	Denmark	2008	Vegetables, Other	199
54	Denmark	2008	Wheat	287
54	Denmark	2008	Whey	28
54	Denmark	2008	Wine	74
54	Denmark	2009	Apples	48
54	Denmark	2009	Bananas	45
54	Denmark	2009	Beer	197
54	Denmark	2009	Beverages, Alcoholic	9
54	Denmark	2009	Beverages, Fermented	5
54	Denmark	2009	Bovine Meat	71
54	Denmark	2009	Butter, Ghee	5
54	Denmark	2009	Cheese	63
54	Denmark	2009	Citrus, Other	2
54	Denmark	2009	Cocoa Beans	9
54	Denmark	2009	Coconuts - Incl Copra	4
54	Denmark	2009	Coffee	22
54	Denmark	2009	Cream	30
54	Denmark	2009	Crustaceans	14
54	Denmark	2009	Dates	1
54	Denmark	2009	Demersal Fish	7
54	Denmark	2009	Eggs	46
54	Denmark	2009	Fats, Animals, Raw	26
54	Denmark	2009	Fish, Body Oil	10
54	Denmark	2009	Freshwater Fish	5
54	Denmark	2009	Fruits, Other	69
54	Denmark	2009	Grapefruit	2

FAO country code	Country	Reference year	Food item	Gross food supply (g *capita*$^{-1}$ d^{-1})
54	Denmark	2009	Grapes	30
54	Denmark	2009	Groundnuts (Shelled Eq)	1
54	Denmark	2009	Honey	2
54	Denmark	2009	Lemons, Limes	5
54	Denmark	2009	Maize	13
54	Denmark	2009	Meat, Other	3
54	Denmark	2009	Milk, Whole	98
54	Denmark	2009	Molluscs, Other	4
54	Denmark	2009	Mutton & Goat Meat	3
54	Denmark	2009	Nuts	12
54	Denmark	2009	Oats	27
54	Denmark	2009	Offals, Edible	8
54	Denmark	2009	Oilcrops Oil, Other	1
54	Denmark	2009	Olive Oil	2
54	Denmark	2009	Olives	2
54	Denmark	2009	Onions	25
54	Denmark	2009	Oranges, Mandarines	120
54	Denmark	2009	Palm Oil	8
54	Denmark	2009	Peas	2
54	Denmark	2009	Pelagic Fish	33
54	Denmark	2009	Pigmeat	53
54	Denmark	2009	Pimento	1
54	Denmark	2009	Pineapples	13
54	Denmark	2009	Plantains	1
54	Denmark	2009	Potatoes	208
54	Denmark	2009	Poultry Meat	52
54	Denmark	2009	Rape and Mustard Oil	2
54	Denmark	2009	Rice (Milled Equivalent)	14
54	Denmark	2009	Rye	47
54	Denmark	2009	Soyabean Oil	4
54	Denmark	2009	Spices, Other	2
54	Denmark	2009	Sugar, Refined Equivalent	105
54	Denmark	2009	Sunflowerseed Oil	3
54	Denmark	2009	Sweeteners, Other	28
54	Denmark	2009	Tea	1
54	Denmark	2009	Tomatoes	55
54	Denmark	2009	Vegetables, Other	241
54	Denmark	2009	Wheat	280
54	Denmark	2009	Whey	9
54	Denmark	2009	Wine	77
54	Denmark	2010	Apples	54
54	Denmark	2010	Bananas	37
54	Denmark	2010	Beans	1
54	Denmark	2010	Beer	185
54	Denmark	2010	Beverages, Alcoholic	8
54	Denmark	2010	Beverages, Fermented	5
54	Denmark	2010	Bovine Meat	80
54	Denmark	2010	Butter, Ghee	5
54	Denmark	2010	Cheese	52
54	Denmark	2010	Cocoa Beans	10
54	Denmark	2010	Coconuts - Incl Copra	4
54	Denmark	2010	Coffee	27
54	Denmark	2010	Cream	28
54	Denmark	2010	Crustaceans	11
54	Denmark	2010	Dates	1
54	Denmark	2010	Demersal Fish	7
54	Denmark	2010	Eggs	45
54	Denmark	2010	Fats, Animals, Raw	26
54	Denmark	2010	Fish, Body Oil	10
54	Denmark	2010	Freshwater Fish	6
54	Denmark	2010	Fruits, Other	74
54	Denmark	2010	Grapefruit	2
54	Denmark	2010	Grapes	30
54	Denmark	2010	Groundnuts (Shelled Eq)	1
54	Denmark	2010	Honey	2
54	Denmark	2010	Lemons, Limes	6
54	Denmark	2010	Maize	13
54	Denmark	2010	Maize Germ Oil	1
54	Denmark	2010	Meat, Other	3
54	Denmark	2010	Milk, Whole	117
54	Denmark	2010	Molluscs, Other	2
54	Denmark	2010	Mutton & Goat Meat	3
54	Denmark	2010	Nuts	12
54	Denmark	2010	Oats	27
54	Denmark	2010	Offals, Edible	23
54	Denmark	2010	Oilcrops Oil, Other	2
54	Denmark	2010	Olive Oil	2
54	Denmark	2010	Olives	2
54	Denmark	2010	Onions	24
54	Denmark	2010	Oranges, Mandarines	58
54	Denmark	2010	Palm Oil	9
54	Denmark	2010	Peas	2
54	Denmark	2010	Pelagic Fish	36
54	Denmark	2010	Pigmeat	62
54	Denmark	2010	Pineapples	12
54	Denmark	2010	Plantains	1
54	Denmark	2010	Potatoes	161
54	Denmark	2010	Poultry Meat	60
54	Denmark	2010	Pulses, Other	1
54	Denmark	2010	Rice (Milled Equivalent)	13
54	Denmark	2010	Rye	48
54	Denmark	2010	Soyabean Oil	3
54	Denmark	2010	Spices, Other	2
54	Denmark	2010	Sugar, Refined Equivalent	104
54	Denmark	2010	Sunflowerseed Oil	1
54	Denmark	2010	Sweeteners, Other	31
54	Denmark	2010	Tea	1

FAO country code	Country	Reference year	Food item	Gross food supply (g *capita*$^{-1}$ d^{-1})
54	Denmark	2010	Tomatoes	56
54	Denmark	2010	Vegetables, Other	250
54	Denmark	2010	Wheat	297
54	Denmark	2010	Whey	19
54	Denmark	2010	Wine	80
54	Denmark	2011	Apples	45
54	Denmark	2011	Bananas	35
54	Denmark	2011	Beer	183
54	Denmark	2011	Beverages, Alcoholic	9
54	Denmark	2011	Beverages, Fermented	1
54	Denmark	2011	Bovine Meat	81
54	Denmark	2011	Butter, Ghee	5
54	Denmark	2011	Cheese	45
54	Denmark	2011	Citrus, Other	1
54	Denmark	2011	Cocoa Beans	11
54	Denmark	2011	Coconuts - Incl Copra	4
54	Denmark	2011	Coffee	26
54	Denmark	2011	Cream	27
54	Denmark	2011	Crustaceans	11
54	Denmark	2011	Dates	1
54	Denmark	2011	Demersal Fish	7
54	Denmark	2011	Eggs	42
54	Denmark	2011	Fats, Animals, Raw	26
54	Denmark	2011	Fish, Body Oil	10
54	Denmark	2011	Freshwater Fish	6
54	Denmark	2011	Fruits, Other	78
54	Denmark	2011	Grapefruit	1
54	Denmark	2011	Grapes	26
54	Denmark	2011	Groundnuts (Shelled Eq)	1
54	Denmark	2011	Honey	2
54	Denmark	2011	Lemons, Limes	6
54	Denmark	2011	Maize	13
54	Denmark	2011	Maize Germ Oil	1
54	Denmark	2011	Meat, Other	2
54	Denmark	2011	Milk, Whole	154
54	Denmark	2011	Molluscs, Other	2
54	Denmark	2011	Mutton & Goat Meat	3
54	Denmark	2011	Nuts	14
54	Denmark	2011	Oats	28
54	Denmark	2011	Offals, Edible	14
54	Denmark	2011	Oilcrops Oil, Other	2
54	Denmark	2011	Olive Oil	3
54	Denmark	2011	Olives	2
54	Denmark	2011	Onions	28
54	Denmark	2011	Oranges, Mandarines	67
54	Denmark	2011	Palm Oil	9
54	Denmark	2011	Peas	2
54	Denmark	2011	Pelagic Fish	36
54	Denmark	2011	Pepper	1
54	Denmark	2011	Pigmeat	50
54	Denmark	2011	Pineapples	11
54	Denmark	2011	Plantains	1
54	Denmark	2011	Potatoes	164
54	Denmark	2011	Poultry Meat	70
54	Denmark	2011	Rice (Milled Equivalent)	15
54	Denmark	2011	Rye	45
54	Denmark	2011	Soyabean Oil	6
54	Denmark	2011	Spices, Other	2
54	Denmark	2011	Sugar, Refined Equivalent	104
54	Denmark	2011	Sunflowerseed Oil	2
54	Denmark	2011	Sweeteners, Other	31
54	Denmark	2011	Tea	1
54	Denmark	2011	Tomatoes	57
54	Denmark	2011	Vegetables, Other	249
54	Denmark	2011	Wheat	276
54	Denmark	2011	Whey	46
54	Denmark	2011	Wine	84
56	Dominican Republic	1992	Apples	1
56	Dominican Republic	1992	Bananas	117
56	Dominican Republic	1992	Beans	17
56	Dominican Republic	1992	Beer	75
56	Dominican Republic	1992	Beverages, Alcoholic	17
56	Dominican Republic	1992	Bovine Meat	27
56	Dominican Republic	1992	Butter, Ghee	1
56	Dominican Republic	1992	Cassava	46
56	Dominican Republic	1992	Cheese	1
56	Dominican Republic	1992	Cocoa Beans	3
56	Dominican Republic	1992	Coconut Oil	6
56	Dominican Republic	1992	Coconuts - Incl Copra	10
56	Dominican Republic	1992	Coffee	7
56	Dominican Republic	1992	Cottonseed Oil	1
56	Dominican Republic	1992	Demersal Fish	8
56	Dominican Republic	1992	Eggs	12
56	Dominican Republic	1992	Fats, Animals, Raw	3
56	Dominican Republic	1992	Freshwater Fish	1
56	Dominican Republic	1992	Fruits, Other	80
56	Dominican Republic	1992	Grapefruit	1
56	Dominican Republic	1992	Grapes	1
56	Dominican Republic	1992	Groundnut Oil	1
56	Dominican Republic	1992	Lemons, Limes	3
56	Dominican Republic	1992	Maize	9
56	Dominican Republic	1992	Milk, Whole	107
56	Dominican Republic	1992	Molluscs, Other	1
56	Dominican Republic	1992	Mutton & Goat Meat	1
56	Dominican Republic	1992	Oats	1
56	Dominican Republic	1992	Offals, Edible	5
56	Dominican Republic	1992	Onions	7

FAO country code	Country	Reference year	Food item	Gross food supply (g $capita^{-1}$ d^{-1})
56	Dominican Republic	1992	Oranges, Mandarines	17
56	Dominican Republic	1992	Palm Oil	6
56	Dominican Republic	1992	Pelagic Fish	6
56	Dominican Republic	1992	Pigmeat	16
56	Dominican Republic	1992	Pineapples	3
56	Dominican Republic	1992	Plantains	105
56	Dominican Republic	1992	Potatoes	11
56	Dominican Republic	1992	Poultry Meat	44
56	Dominican Republic	1992	Pulses, Other	10
56	Dominican Republic	1992	Rice (Milled Equivalent)	123
56	Dominican Republic	1992	Roots, Other	4
56	Dominican Republic	1992	Soyabean Oil	20
56	Dominican Republic	1992	Spices, Other	1
56	Dominican Republic	1992	Sugar, Refined Equivalent	93
56	Dominican Republic	1992	Sunflowerseed Oil	3
56	Dominican Republic	1992	Sweet Potatoes	14
56	Dominican Republic	1992	Tomatoes	33
56	Dominican Republic	1992	Vegetables, Other	31
56	Dominican Republic	1992	Wheat	71
56	Dominican Republic	1992	Wine	1
56	Dominican Republic	1992	Yams	2
56	Dominican Republic	1993	Apples	1
56	Dominican Republic	1993	Bananas	122
56	Dominican Republic	1993	Beans	17
56	Dominican Republic	1993	Beer	67
56	Dominican Republic	1993	Beverages, Alcoholic	16
56	Dominican Republic	1993	Bovine Meat	28
56	Dominican Republic	1993	Butter, Ghee	1
56	Dominican Republic	1993	Cassava	30
56	Dominican Republic	1993	Cheese	1
56	Dominican Republic	1993	Cocoa Beans	3
56	Dominican Republic	1993	Coconut Oil	2
56	Dominican Republic	1993	Coconuts - Incl Copra	22
56	Dominican Republic	1993	Coffee	6
56	Dominican Republic	1993	Crustaceans	1
56	Dominican Republic	1993	Demersal Fish	9
56	Dominican Republic	1993	Eggs	12
56	Dominican Republic	1993	Fats, Animals, Raw	3
56	Dominican Republic	1993	Freshwater Fish	1
56	Dominican Republic	1993	Fruits, Other	82
56	Dominican Republic	1993	Grapefruit	1
56	Dominican Republic	1993	Grapes	1
56	Dominican Republic	1993	Lemons, Limes	3
56	Dominican Republic	1993	Maize	27
56	Dominican Republic	1993	Maize Germ Oil	1
56	Dominican Republic	1993	Milk, Whole	111
56	Dominican Republic	1993	Molluscs, Other	1
56	Dominican Republic	1993	Mutton & Goat Meat	1
56	Dominican Republic	1993	Oats	2
56	Dominican Republic	1993	Offals, Edible	5
56	Dominican Republic	1993	Onions	8
56	Dominican Republic	1993	Oranges, Mandarines	20
56	Dominican Republic	1993	Palm Oil	7
56	Dominican Republic	1993	Pelagic Fish	8
56	Dominican Republic	1993	Pigmeat	19
56	Dominican Republic	1993	Pineapples	9
56	Dominican Republic	1993	Plantains	132
56	Dominican Republic	1993	Potatoes	6
56	Dominican Republic	1993	Poultry Meat	46
56	Dominican Republic	1993	Pulses, Other	10
56	Dominican Republic	1993	Rice (Milled Equivalent)	118
56	Dominican Republic	1993	Roots, Other	5
56	Dominican Republic	1993	Soyabean Oil	24
56	Dominican Republic	1993	Spices, Other	1
56	Dominican Republic	1993	Sugar, Refined Equivalent	100
56	Dominican Republic	1993	Sunflowerseed Oil	3
56	Dominican Republic	1993	Sweet Potatoes	7
56	Dominican Republic	1993	Tomatoes	28
56	Dominican Republic	1993	Vegetables, Other	29
56	Dominican Republic	1993	Wheat	64
56	Dominican Republic	1993	Wine	1
56	Dominican Republic	1993	Yams	2
56	Dominican Republic	1994	Apples	1
56	Dominican Republic	1994	Bananas	106
56	Dominican Republic	1994	Beans	15
56	Dominican Republic	1994	Beer	77
56	Dominican Republic	1994	Beverages, Alcoholic	16
56	Dominican Republic	1994	Bovine Meat	27
56	Dominican Republic	1994	Butter, Ghee	1
56	Dominican Republic	1994	Cassava	30
56	Dominican Republic	1994	Cheese	1
56	Dominican Republic	1994	Cocoa Beans	4
56	Dominican Republic	1994	Coconut Oil	2
56	Dominican Republic	1994	Coconuts - Incl Copra	23
56	Dominican Republic	1994	Coffee	6
56	Dominican Republic	1994	Crustaceans	1
56	Dominican Republic	1994	Demersal Fish	11
56	Dominican Republic	1994	Eggs	12
56	Dominican Republic	1994	Fats, Animals, Raw	3
56	Dominican Republic	1994	Freshwater Fish	2
56	Dominican Republic	1994	Fruits, Other	90
56	Dominican Republic	1994	Grapefruit	1
56	Dominican Republic	1994	Grapes	1
56	Dominican Republic	1994	Lemons, Limes	3
56	Dominican Republic	1994	Maize	30
56	Dominican Republic	1994	Maize Germ Oil	1
56	Dominican Republic	1994	Milk, Whole	105

FAO country code	Country	Reference year	Food item	Gross food supply (g $capita^{-1}$ d^{-1})
56	Dominican Republic	1994	Molluscs, Other	2
56	Dominican Republic	1994	Mutton & Goat Meat	1
56	Dominican Republic	1994	Oats	1
56	Dominican Republic	1994	Offals, Edible	5
56	Dominican Republic	1994	Onions	7
56	Dominican Republic	1994	Oranges, Mandarines	20
56	Dominican Republic	1994	Palm Oil	9
56	Dominican Republic	1994	Pelagic Fish	8
56	Dominican Republic	1994	Pigmeat	20
56	Dominican Republic	1994	Pineapples	37
56	Dominican Republic	1994	Plantains	89
56	Dominican Republic	1994	Potatoes	6
56	Dominican Republic	1994	Poultry Meat	46
56	Dominican Republic	1994	Pulses, Other	9
56	Dominican Republic	1994	Rice (Milled Equivalent)	117
56	Dominican Republic	1994	Roots, Other	6
56	Dominican Republic	1994	Soyabean Oil	25
56	Dominican Republic	1994	Spices, Other	1
56	Dominican Republic	1994	Sugar, Refined Equivalent	100
56	Dominican Republic	1994	Sunflowerseed Oil	4
56	Dominican Republic	1994	Sweet Potatoes	9
56	Dominican Republic	1994	Sweeteners, Other	1
56	Dominican Republic	1994	Tomatoes	25
56	Dominican Republic	1994	Vegetables, Other	33
56	Dominican Republic	1994	Wheat	77
56	Dominican Republic	1994	Wine	1
56	Dominican Republic	1994	Yams	2
56	Dominican Republic	1995	Apples	1
56	Dominican Republic	1995	Bananas	87
56	Dominican Republic	1995	Beans	15
56	Dominican Republic	1995	Beer	71
56	Dominican Republic	1995	Beverages, Alcoholic	15
56	Dominican Republic	1995	Bovine Meat	27
56	Dominican Republic	1995	Butter, Ghee	1
56	Dominican Republic	1995	Cassava	42
56	Dominican Republic	1995	Cheese	1
56	Dominican Republic	1995	Cocoa Beans	4
56	Dominican Republic	1995	Coconut Oil	2
56	Dominican Republic	1995	Coconuts - Incl Copra	22
56	Dominican Republic	1995	Coffee	6
56	Dominican Republic	1995	Demersal Fish	11
56	Dominican Republic	1995	Eggs	12
56	Dominican Republic	1995	Fats, Animals, Raw	3
56	Dominican Republic	1995	Freshwater Fish	1
56	Dominican Republic	1995	Fruits, Other	87
56	Dominican Republic	1995	Grapefruit	1
56	Dominican Republic	1995	Grapes	1
56	Dominican Republic	1995	Lemons, Limes	2
56	Dominican Republic	1995	Maize	19
56	Dominican Republic	1995	Maize Germ Oil	1
56	Dominican Republic	1995	Milk, Whole	105
56	Dominican Republic	1995	Molluscs, Other	1
56	Dominican Republic	1995	Mutton & Goat Meat	1
56	Dominican Republic	1995	Oats	1
56	Dominican Republic	1995	Offals, Edible	5
56	Dominican Republic	1995	Oilcrops Oil, Other	1
56	Dominican Republic	1995	Onions	9
56	Dominican Republic	1995	Oranges, Mandarines	20
56	Dominican Republic	1995	Palm Oil	8
56	Dominican Republic	1995	Pelagic Fish	8
56	Dominican Republic	1995	Pigmeat	21
56	Dominican Republic	1995	Pineapples	28
56	Dominican Republic	1995	Plantains	98
56	Dominican Republic	1995	Potatoes	9
56	Dominican Republic	1995	Poultry Meat	47
56	Dominican Republic	1995	Pulses, Other	10
56	Dominican Republic	1995	Rice (Milled Equivalent)	118
56	Dominican Republic	1995	Roots, Other	9
56	Dominican Republic	1995	Soyabean Oil	26
56	Dominican Republic	1995	Spices, Other	1
56	Dominican Republic	1995	Sugar, Refined Equivalent	99
56	Dominican Republic	1995	Sunflowerseed Oil	4
56	Dominican Republic	1995	Sweet Potatoes	10
56	Dominican Republic	1995	Sweeteners, Other	2
56	Dominican Republic	1995	Tomatoes	28
56	Dominican Republic	1995	Vegetables, Other	28
56	Dominican Republic	1995	Wheat	82
56	Dominican Republic	1995	Wine	1
56	Dominican Republic	1995	Yams	3
56	Dominican Republic	1996	Apples	2
56	Dominican Republic	1996	Bananas	92
56	Dominican Republic	1996	Beans	12
56	Dominican Republic	1996	Beer	69
56	Dominican Republic	1996	Beverages, Alcoholic	16
56	Dominican Republic	1996	Bovine Meat	27
56	Dominican Republic	1996	Butter, Ghee	1
56	Dominican Republic	1996	Cassava	39
56	Dominican Republic	1996	Cheese	1
56	Dominican Republic	1996	Cocoa Beans	4
56	Dominican Republic	1996	Coconut Oil	2
56	Dominican Republic	1996	Coconuts - Incl Copra	27
56	Dominican Republic	1996	Coffee	5
56	Dominican Republic	1996	Demersal Fish	9
56	Dominican Republic	1996	Eggs	12
56	Dominican Republic	1996	Fats, Animals, Raw	3
56	Dominican Republic	1996	Fruits, Other	78
56	Dominican Republic	1996	Grapefruit	1

FAO country code	Country	Reference year	Food item	Gross food supply (g $capita^{-1}$ d^{-1})
56	Dominican Republic	1996	Grapes	1
56	Dominican Republic	1996	Lemons, Limes	2
56	Dominican Republic	1996	Maize	22
56	Dominican Republic	1996	Maize Germ Oil	1
56	Dominican Republic	1996	Marine Fish, Other	1
56	Dominican Republic	1996	Milk, Whole	105
56	Dominican Republic	1996	Molluscs, Other	1
56	Dominican Republic	1996	Mutton & Goat Meat	1
56	Dominican Republic	1996	Oats	3
56	Dominican Republic	1996	Offals, Edible	5
56	Dominican Republic	1996	Onions	8
56	Dominican Republic	1996	Oranges, Mandarines	26
56	Dominican Republic	1996	Palm Oil	9
56	Dominican Republic	1996	Pelagic Fish	9
56	Dominican Republic	1996	Pigmeat	21
56	Dominican Republic	1996	Pineapples	8
56	Dominican Republic	1996	Plantains	94
56	Dominican Republic	1996	Potatoes	7
56	Dominican Republic	1996	Poultry Meat	50
56	Dominican Republic	1996	Pulses, Other	9
56	Dominican Republic	1996	Rice (Milled Equivalent)	114
56	Dominican Republic	1996	Roots, Other	7
56	Dominican Republic	1996	Soyabean Oil	24
56	Dominican Republic	1996	Spices, Other	1
56	Dominican Republic	1996	Sugar, Refined Equivalent	98
56	Dominican Republic	1996	Sunflowerseed Oil	3
56	Dominican Republic	1996	Sweet Potatoes	10
56	Dominican Republic	1996	Sweeteners, Other	1
56	Dominican Republic	1996	Tomatoes	60
56	Dominican Republic	1996	Vegetables, Other	30
56	Dominican Republic	1996	Wheat	64
56	Dominican Republic	1996	Wine	1
56	Dominican Republic	1996	Yams	3
56	Dominican Republic	1997	Apples	1
56	Dominican Republic	1997	Bananas	93
56	Dominican Republic	1997	Beans	10
56	Dominican Republic	1997	Beer	78
56	Dominican Republic	1997	Beverages, Alcoholic	16
56	Dominican Republic	1997	Bovine Meat	26
56	Dominican Republic	1997	Butter, Ghee	1
56	Dominican Republic	1997	Cassava	29
56	Dominican Republic	1997	Cheese	1
56	Dominican Republic	1997	Cocoa Beans	3
56	Dominican Republic	1997	Coconut Oil	2
56	Dominican Republic	1997	Coconuts - Incl Copra	18
56	Dominican Republic	1997	Coffee	8
56	Dominican Republic	1997	Demersal Fish	10
56	Dominican Republic	1997	Eggs	12
56	Dominican Republic	1997	Fats, Animals, Raw	3
56	Dominican Republic	1997	Freshwater Fish	1
56	Dominican Republic	1997	Fruits, Other	96
56	Dominican Republic	1997	Grapefruit	1
56	Dominican Republic	1997	Grapes	2
56	Dominican Republic	1997	Honey	1
56	Dominican Republic	1997	Lemons, Limes	2
56	Dominican Republic	1997	Maize	21
56	Dominican Republic	1997	Maize Germ Oil	1
56	Dominican Republic	1997	Marine Fish, Other	1
56	Dominican Republic	1997	Milk, Whole	102
56	Dominican Republic	1997	Molluscs, Other	1
56	Dominican Republic	1997	Oats	2
56	Dominican Republic	1997	Offals, Edible	6
56	Dominican Republic	1997	Oilcrops Oil, Other	1
56	Dominican Republic	1997	Onions	8
56	Dominican Republic	1997	Oranges, Mandarines	26
56	Dominican Republic	1997	Palm Oil	9
56	Dominican Republic	1997	Pelagic Fish	10
56	Dominican Republic	1997	Pigmeat	21
56	Dominican Republic	1997	Pineapples	25
56	Dominican Republic	1997	Plantains	84
56	Dominican Republic	1997	Potatoes	6
56	Dominican Republic	1997	Poultry Meat	54
56	Dominican Republic	1997	Pulses, Other	7
56	Dominican Republic	1997	Rice (Milled Equivalent)	116
56	Dominican Republic	1997	Roots, Other	8
56	Dominican Republic	1997	Soyabean Oil	26
56	Dominican Republic	1997	Spices, Other	1
56	Dominican Republic	1997	Sugar, Refined Equivalent	95
56	Dominican Republic	1997	Sunflowerseed Oil	3
56	Dominican Republic	1997	Sweet Potatoes	6
56	Dominican Republic	1997	Sweeteners, Other	1
56	Dominican Republic	1997	Tomatoes	76
56	Dominican Republic	1997	Vegetables, Other	29
56	Dominican Republic	1997	Wheat	56
56	Dominican Republic	1997	Wine	1
56	Dominican Republic	1997	Yams	3
56	Dominican Republic	1998	Apples	2
56	Dominican Republic	1998	Bananas	72
56	Dominican Republic	1998	Beans	12
56	Dominican Republic	1998	Beer	91
56	Dominican Republic	1998	Beverages, Alcoholic	15
56	Dominican Republic	1998	Bovine Meat	26
56	Dominican Republic	1998	Butter, Ghee	1
56	Dominican Republic	1998	Cassava	37
56	Dominican Republic	1998	Cheese	2
56	Dominican Republic	1998	Cocoa Beans	3
56	Dominican Republic	1998	Coconut Oil	2

FAO country code	Country	Reference year	Food item	Gross food supply (g $capita^{-1}$ d^{-1})
56	Dominican Republic	1998	Coconuts - Incl Copra	26
56	Dominican Republic	1998	Coffee	11
56	Dominican Republic	1998	Demersal Fish	9
56	Dominican Republic	1998	Eggs	13
56	Dominican Republic	1998	Fats, Animals, Raw	4
56	Dominican Republic	1998	Freshwater Fish	1
56	Dominican Republic	1998	Fruits, Other	94
56	Dominican Republic	1998	Grapefruit	2
56	Dominican Republic	1998	Grapes	2
56	Dominican Republic	1998	Groundnut Oil	1
56	Dominican Republic	1998	Lemons, Limes	2
56	Dominican Republic	1998	Maize	20
56	Dominican Republic	1998	Maize Germ Oil	1
56	Dominican Republic	1998	Milk, Whole	103
56	Dominican Republic	1998	Molluscs, Other	1
56	Dominican Republic	1998	Oats	3
56	Dominican Republic	1998	Offals, Edible	6
56	Dominican Republic	1998	Oilcrops Oil, Other	1
56	Dominican Republic	1998	Onions	8
56	Dominican Republic	1998	Oranges, Mandarines	21
56	Dominican Republic	1998	Palm Oil	9
56	Dominican Republic	1998	Pelagic Fish	8
56	Dominican Republic	1998	Pigmeat	22
56	Dominican Republic	1998	Pineapples	18
56	Dominican Republic	1998	Plantains	78
56	Dominican Republic	1998	Potatoes	5
56	Dominican Republic	1998	Poultry Meat	62
56	Dominican Republic	1998	Pulses, Other	4
56	Dominican Republic	1998	Rice (Milled Equivalent)	115
56	Dominican Republic	1998	Roots, Other	4
56	Dominican Republic	1998	Soyabean Oil	26
56	Dominican Republic	1998	Spices, Other	1
56	Dominican Republic	1998	Sugar, Refined Equivalent	88
56	Dominican Republic	1998	Sunflowerseed Oil	3
56	Dominican Republic	1998	Sweet Potatoes	10
56	Dominican Republic	1998	Sweeteners, Other	1
56	Dominican Republic	1998	Tomatoes	81
56	Dominican Republic	1998	Vegetables, Other	24
56	Dominican Republic	1998	Wheat	59
56	Dominican Republic	1998	Wine	2
56	Dominican Republic	1998	Yams	4
56	Dominican Republic	1999	Apples	2
56	Dominican Republic	1999	Bananas	105
56	Dominican Republic	1999	Beans	11
56	Dominican Republic	1999	Beer	100
56	Dominican Republic	1999	Beverages, Alcoholic	15
56	Dominican Republic	1999	Bovine Meat	21
56	Dominican Republic	1999	Butter, Ghee	1
56	Dominican Republic	1999	Cassava	37
56	Dominican Republic	1999	Cheese	2
56	Dominican Republic	1999	Coconut Oil	1
56	Dominican Republic	1999	Coconuts - Incl Copra	24
56	Dominican Republic	1999	Coffee	8
56	Dominican Republic	1999	Demersal Fish	9
56	Dominican Republic	1999	Eggs	13
56	Dominican Republic	1999	Fats, Animals, Raw	4
56	Dominican Republic	1999	Freshwater Fish	1
56	Dominican Republic	1999	Fruits, Other	90
56	Dominican Republic	1999	Grapefruit	2
56	Dominican Republic	1999	Grapes	1
56	Dominican Republic	1999	Lemons, Limes	2
56	Dominican Republic	1999	Maize	29
56	Dominican Republic	1999	Maize Germ Oil	1
56	Dominican Republic	1999	Marine Fish, Other	1
56	Dominican Republic	1999	Milk, Whole	107
56	Dominican Republic	1999	Molluscs, Other	1
56	Dominican Republic	1999	Oats	1
56	Dominican Republic	1999	Offals, Edible	6
56	Dominican Republic	1999	Oilcrops Oil, Other	1
56	Dominican Republic	1999	Onions	13
56	Dominican Republic	1999	Oranges, Mandarines	22
56	Dominican Republic	1999	Palm Oil	10
56	Dominican Republic	1999	Pelagic Fish	10
56	Dominican Republic	1999	Pigmeat	20
56	Dominican Republic	1999	Pineapples	20
56	Dominican Republic	1999	Plantains	56
56	Dominican Republic	1999	Potatoes	8
56	Dominican Republic	1999	Poultry Meat	61
56	Dominican Republic	1999	Pulses, Other	8
56	Dominican Republic	1999	Rice (Milled Equivalent)	117
56	Dominican Republic	1999	Roots, Other	11
56	Dominican Republic	1999	Soyabean Oil	29
56	Dominican Republic	1999	Spices, Other	1
56	Dominican Republic	1999	Sugar, Refined Equivalent	90
56	Dominican Republic	1999	Sunflowerseed Oil	3
56	Dominican Republic	1999	Sweet Potatoes	12
56	Dominican Republic	1999	Sweeteners, Other	1
56	Dominican Republic	1999	Tomatoes	81
56	Dominican Republic	1999	Vegetables, Other	31
56	Dominican Republic	1999	Wheat	33
56	Dominican Republic	1999	Wine	1
56	Dominican Republic	1999	Yams	3
56	Dominican Republic	2000	Apples	3
56	Dominican Republic	2000	Bananas	95
56	Dominican Republic	2000	Beans	10
56	Dominican Republic	2000	Beer	109
56	Dominican Republic	2000	Beverages, Alcoholic	18

FAO country code	Country	Reference year	Food item	Gross food supply (g *capita*$^{-1}$ d^{-1})
56	Dominican Republic	2000	Bovine Meat	22
56	Dominican Republic	2000	Butter, Ghee	1
56	Dominican Republic	2000	Cassava	36
56	Dominican Republic	2000	Cheese	2
56	Dominican Republic	2000	Coconut Oil	1
56	Dominican Republic	2000	Coconuts - Incl Copra	38
56	Dominican Republic	2000	Coffee	10
56	Dominican Republic	2000	Crustaceans	1
56	Dominican Republic	2000	Demersal Fish	12
56	Dominican Republic	2000	Eggs	14
56	Dominican Republic	2000	Fats, Animals, Raw	4
56	Dominican Republic	2000	Freshwater Fish	1
56	Dominican Republic	2000	Fruits, Other	115
56	Dominican Republic	2000	Grapefruit	2
56	Dominican Republic	2000	Grapes	2
56	Dominican Republic	2000	Lemons, Limes	2
56	Dominican Republic	2000	Maize	23
56	Dominican Republic	2000	Maize Germ Oil	1
56	Dominican Republic	2000	Marine Fish, Other	1
56	Dominican Republic	2000	Milk, Whole	110
56	Dominican Republic	2000	Molluscs, Other	1
56	Dominican Republic	2000	Oats	2
56	Dominican Republic	2000	Offals, Edible	5
56	Dominican Republic	2000	Oilcrops Oil, Other	1
56	Dominican Republic	2000	Onions	10
56	Dominican Republic	2000	Oranges, Mandarines	36
56	Dominican Republic	2000	Palm Oil	12
56	Dominican Republic	2000	Pelagic Fish	10
56	Dominican Republic	2000	Pigmeat	20
56	Dominican Republic	2000	Pineapples	17
56	Dominican Republic	2000	Plantains	50
56	Dominican Republic	2000	Potatoes	6
56	Dominican Republic	2000	Poultry Meat	67
56	Dominican Republic	2000	Pulses, Other	5
56	Dominican Republic	2000	Rice (Milled Equivalent)	122
56	Dominican Republic	2000	Roots, Other	7
56	Dominican Republic	2000	Soyabean Oil	28
56	Dominican Republic	2000	Spices, Other	1
56	Dominican Republic	2000	Sugar, Refined Equivalent	87
56	Dominican Republic	2000	Sunflowerseed Oil	3
56	Dominican Republic	2000	Sweet Potatoes	7
56	Dominican Republic	2000	Sweeteners, Other	2
56	Dominican Republic	2000	Tomatoes	81
56	Dominican Republic	2000	Vegetables, Other	26
56	Dominican Republic	2000	Wheat	63
56	Dominican Republic	2000	Wine	3
56	Dominican Republic	2000	Yams	4
56	Dominican Republic	2001	Apples	3
56	Dominican Republic	2001	Bananas	83
56	Dominican Republic	2001	Beans	13
56	Dominican Republic	2001	Beer	90
56	Dominican Republic	2001	Beverages, Alcoholic	15
56	Dominican Republic	2001	Bovine Meat	22
56	Dominican Republic	2001	Butter, Ghee	1
56	Dominican Republic	2001	Cassava	35
56	Dominican Republic	2001	Cheese	2
56	Dominican Republic	2001	Coconut Oil	1
56	Dominican Republic	2001	Coconuts - Incl Copra	30
56	Dominican Republic	2001	Coffee	9
56	Dominican Republic	2001	Crustaceans	1
56	Dominican Republic	2001	Demersal Fish	12
56	Dominican Republic	2001	Eggs	16
56	Dominican Republic	2001	Fats, Animals, Raw	4
56	Dominican Republic	2001	Freshwater Fish	1
56	Dominican Republic	2001	Fruits, Other	154
56	Dominican Republic	2001	Grapefruit	2
56	Dominican Republic	2001	Grapes	2
56	Dominican Republic	2001	Maize	19
56	Dominican Republic	2001	Maize Germ Oil	1
56	Dominican Republic	2001	Marine Fish, Other	2
56	Dominican Republic	2001	Milk, Whole	117
56	Dominican Republic	2001	Molluscs, Other	1
56	Dominican Republic	2001	Oats	2
56	Dominican Republic	2001	Offals, Edible	6
56	Dominican Republic	2001	Oilcrops Oil, Other	1
56	Dominican Republic	2001	Onions	12
56	Dominican Republic	2001	Oranges, Mandarines	17
56	Dominican Republic	2001	Palm Oil	11
56	Dominican Republic	2001	Pelagic Fish	9
56	Dominican Republic	2001	Pigmeat	20
56	Dominican Republic	2001	Pineapples	37
56	Dominican Republic	2001	Plantains	92
56	Dominican Republic	2001	Potatoes	17
56	Dominican Republic	2001	Poultry Meat	64
56	Dominican Republic	2001	Pulses, Other	9
56	Dominican Republic	2001	Rice (Milled Equivalent)	129
56	Dominican Republic	2001	Roots, Other	3
56	Dominican Republic	2001	Soyabean Oil	26
56	Dominican Republic	2001	Spices, Other	1
56	Dominican Republic	2001	Sugar, Refined Equivalent	101
56	Dominican Republic	2001	Sunflowerseed Oil	3
56	Dominican Republic	2001	Sweet Potatoes	6
56	Dominican Republic	2001	Sweeteners, Other	1
56	Dominican Republic	2001	Tomatoes	89
56	Dominican Republic	2001	Vegetables, Other	40
56	Dominican Republic	2001	Wheat	41
56	Dominican Republic	2001	Wine	3

FAO country code	Country	Reference year	Food item	Gross food supply (g *capita*$^{-1}$ d^{-1})
56	Dominican Republic	2001	Yams	5
56	Dominican Republic	2002	Apples	2
56	Dominican Republic	2002	Bananas	104
56	Dominican Republic	2002	Beans	12
56	Dominican Republic	2002	Beer	101
56	Dominican Republic	2002	Beverages, Alcoholic	16
56	Dominican Republic	2002	Bovine Meat	22
56	Dominican Republic	2002	Butter, Ghee	1
56	Dominican Republic	2002	Cassava	33
56	Dominican Republic	2002	Cheese	2
56	Dominican Republic	2002	Cocoa Beans	2
56	Dominican Republic	2002	Coconut Oil	4
56	Dominican Republic	2002	Coconuts - Incl Copra	34
56	Dominican Republic	2002	Coffee	9
56	Dominican Republic	2002	Crustaceans	1
56	Dominican Republic	2002	Demersal Fish	8
56	Dominican Republic	2002	Eggs	19
56	Dominican Republic	2002	Fats, Animals, Raw	3
56	Dominican Republic	2002	Freshwater Fish	2
56	Dominican Republic	2002	Fruits, Other	169
56	Dominican Republic	2002	Grapefruit	3
56	Dominican Republic	2002	Grapes	2
56	Dominican Republic	2002	Lemons, Limes	1
56	Dominican Republic	2002	Maize	19
56	Dominican Republic	2002	Maize Germ Oil	1
56	Dominican Republic	2002	Marine Fish, Other	1
56	Dominican Republic	2002	Milk, Whole	141
56	Dominican Republic	2002	Molluscs, Other	1
56	Dominican Republic	2002	Oats	1
56	Dominican Republic	2002	Offals, Edible	6
56	Dominican Republic	2002	Oilcrops Oil, Other	1
56	Dominican Republic	2002	Olives	1
56	Dominican Republic	2002	Onions	12
56	Dominican Republic	2002	Oranges, Mandarines	23
56	Dominican Republic	2002	Palm Oil	11
56	Dominican Republic	2002	Pelagic Fish	13
56	Dominican Republic	2002	Pigmeat	20
56	Dominican Republic	2002	Pineapples	30
56	Dominican Republic	2002	Plantains	89
56	Dominican Republic	2002	Potatoes	14
56	Dominican Republic	2002	Poultry Meat	57
56	Dominican Republic	2002	Pulses, Other	10
56	Dominican Republic	2002	Rice (Milled Equivalent)	115
56	Dominican Republic	2002	Roots, Other	11
56	Dominican Republic	2002	Soyabean Oil	21
56	Dominican Republic	2002	Spices, Other	1
56	Dominican Republic	2002	Sugar, Refined Equivalent	101
56	Dominican Republic	2002	Sunflowerseed Oil	3
56	Dominican Republic	2002	Sweet Potatoes	7
56	Dominican Republic	2002	Sweeteners, Other	1
56	Dominican Republic	2002	Tomatoes	69
56	Dominican Republic	2002	Vegetables, Other	44
56	Dominican Republic	2002	Wheat	62
56	Dominican Republic	2002	Wine	1
56	Dominican Republic	2002	Yams	6
56	Dominican Republic	2003	Apples	2
56	Dominican Republic	2003	Bananas	102
56	Dominican Republic	2003	Beans	15
56	Dominican Republic	2003	Beer	102
56	Dominican Republic	2003	Beverages, Alcoholic	16
56	Dominican Republic	2003	Bovine Meat	21
56	Dominican Republic	2003	Butter, Ghee	1
56	Dominican Republic	2003	Cassava	33
56	Dominican Republic	2003	Cereals, Other	1
56	Dominican Republic	2003	Cheese	2
56	Dominican Republic	2003	Cocoa Beans	3
56	Dominican Republic	2003	Coconut Oil	3
56	Dominican Republic	2003	Coconuts - Incl Copra	28
56	Dominican Republic	2003	Coffee	8
56	Dominican Republic	2003	Crustaceans	1
56	Dominican Republic	2003	Demersal Fish	6
56	Dominican Republic	2003	Eggs	16
56	Dominican Republic	2003	Fats, Animals, Raw	3
56	Dominican Republic	2003	Freshwater Fish	1
56	Dominican Republic	2003	Fruits, Other	179
56	Dominican Republic	2003	Grapefruit	3
56	Dominican Republic	2003	Grapes	2
56	Dominican Republic	2003	Honey	1
56	Dominican Republic	2003	Lemons, Limes	1
56	Dominican Republic	2003	Maize	16
56	Dominican Republic	2003	Maize Germ Oil	1
56	Dominican Republic	2003	Marine Fish, Other	2
56	Dominican Republic	2003	Milk, Whole	85
56	Dominican Republic	2003	Oats	1
56	Dominican Republic	2003	Offals, Edible	5
56	Dominican Republic	2003	Onions	13
56	Dominican Republic	2003	Oranges, Mandarines	42
56	Dominican Republic	2003	Palm Oil	10
56	Dominican Republic	2003	Pelagic Fish	9
56	Dominican Republic	2003	Pigmeat	20
56	Dominican Republic	2003	Pineapples	26
56	Dominican Republic	2003	Plantains	93
56	Dominican Republic	2003	Potatoes	14
56	Dominican Republic	2003	Poultry Meat	47
56	Dominican Republic	2003	Pulses, Other	10
56	Dominican Republic	2003	Rice (Milled Equivalent)	93
56	Dominican Republic	2003	Roots, Other	15

FAO country code	Country	Reference year	Food item	Gross food supply (g $capita^{-1}$ d^{-1})
56	Dominican Republic	2003	Soyabean Oil	25
56	Dominican Republic	2003	Spices, Other	1
56	Dominican Republic	2003	Sugar, Refined Equivalent	96
56	Dominican Republic	2003	Sunflowerseed Oil	3
56	Dominican Republic	2003	Sweet Potatoes	7
56	Dominican Republic	2003	Sweeteners, Other	1
56	Dominican Republic	2003	Tomatoes	78
56	Dominican Republic	2003	Vegetables, Other	49
56	Dominican Republic	2003	Wheat	51
56	Dominican Republic	2003	Wine	2
56	Dominican Republic	2003	Yams	7
56	Dominican Republic	2004	Apples	2
56	Dominican Republic	2004	Bananas	95
56	Dominican Republic	2004	Beans	13
56	Dominican Republic	2004	Beer	102
56	Dominican Republic	2004	Beverages, Alcoholic	16
56	Dominican Republic	2004	Bovine Meat	21
56	Dominican Republic	2004	Butter, Ghee	1
56	Dominican Republic	2004	Cassava	24
56	Dominican Republic	2004	Cereals, Other	1
56	Dominican Republic	2004	Cheese	2
56	Dominican Republic	2004	Cocoa Beans	1
56	Dominican Republic	2004	Coconut Oil	2
56	Dominican Republic	2004	Coconuts - Incl Copra	28
56	Dominican Republic	2004	Coffee	10
56	Dominican Republic	2004	Crustaceans	1
56	Dominican Republic	2004	Demersal Fish	5
56	Dominican Republic	2004	Eggs	14
56	Dominican Republic	2004	Fats, Animals, Raw	3
56	Dominican Republic	2004	Freshwater Fish	1
56	Dominican Republic	2004	Fruits, Other	142
56	Dominican Republic	2004	Grapefruit	3
56	Dominican Republic	2004	Grapes	1
56	Dominican Republic	2004	Honey	1
56	Dominican Republic	2004	Lemons, Limes	1
56	Dominican Republic	2004	Maize	15
56	Dominican Republic	2004	Maize Germ Oil	1
56	Dominican Republic	2004	Marine Fish, Other	2
56	Dominican Republic	2004	Milk, Whole	90
56	Dominican Republic	2004	Oats	1
56	Dominican Republic	2004	Offals, Edible	5
56	Dominican Republic	2004	Olive Oil	1
56	Dominican Republic	2004	Onions	11
56	Dominican Republic	2004	Oranges, Mandarines	36
56	Dominican Republic	2004	Palm Oil	12
56	Dominican Republic	2004	Pelagic Fish	12
56	Dominican Republic	2004	Pigmeat	16
56	Dominican Republic	2004	Pineapples	25
56	Dominican Republic	2004	Plantains	82
56	Dominican Republic	2004	Potatoes	11
56	Dominican Republic	2004	Poultry Meat	71
56	Dominican Republic	2004	Pulses, Other	9
56	Dominican Republic	2004	Rice (Milled Equivalent)	113
56	Dominican Republic	2004	Roots, Other	13
56	Dominican Republic	2004	Soyabean Oil	29
56	Dominican Republic	2004	Spices, Other	1
56	Dominican Republic	2004	Sugar, Refined Equivalent	96
56	Dominican Republic	2004	Sunflowerseed Oil	3
56	Dominican Republic	2004	Sweet Potatoes	6
56	Dominican Republic	2004	Sweeteners, Other	1
56	Dominican Republic	2004	Tomatoes	76
56	Dominican Republic	2004	Vegetables, Other	46
56	Dominican Republic	2004	Wheat	54
56	Dominican Republic	2004	Wine	2
56	Dominican Republic	2004	Yams	4
56	Dominican Republic	2005	Apples	4
56	Dominican Republic	2005	Bananas	96
56	Dominican Republic	2005	Beans	16
56	Dominican Republic	2005	Beer	127
56	Dominican Republic	2005	Beverages, Alcoholic	14
56	Dominican Republic	2005	Bovine Meat	22
56	Dominican Republic	2005	Butter, Ghee	1
56	Dominican Republic	2005	Cassava	26
56	Dominican Republic	2005	Cereals, Other	1
56	Dominican Republic	2005	Cheese	2
56	Dominican Republic	2005	Cocoa Beans	1
56	Dominican Republic	2005	Coconut Oil	2
56	Dominican Republic	2005	Coconuts - Incl Copra	25
56	Dominican Republic	2005	Coffee	11
56	Dominican Republic	2005	Crustaceans	1
56	Dominican Republic	2005	Demersal Fish	7
56	Dominican Republic	2005	Eggs	17
56	Dominican Republic	2005	Fats, Animals, Raw	4
56	Dominican Republic	2005	Freshwater Fish	1
56	Dominican Republic	2005	Fruits, Other	134
56	Dominican Republic	2005	Grapefruit	3
56	Dominican Republic	2005	Grapes	2
56	Dominican Republic	2005	Honey	1
56	Dominican Republic	2005	Lemons, Limes	1
56	Dominican Republic	2005	Maize	16
56	Dominican Republic	2005	Maize Germ Oil	1
56	Dominican Republic	2005	Marine Fish, Other	2
56	Dominican Republic	2005	Milk, Whole	110
56	Dominican Republic	2005	Mutton & Goat Meat	1
56	Dominican Republic	2005	Oats	1
56	Dominican Republic	2005	Offals, Edible	5
56	Dominican Republic	2005	Onions	16

FAO country code	Country	Reference year	Food item	Gross food supply (g *capita*$^{-1}$ d^{-1})
56	Dominican Republic	2005	Oranges, Mandarines	27
56	Dominican Republic	2005	Palm Oil	12
56	Dominican Republic	2005	Pelagic Fish	8
56	Dominican Republic	2005	Pigmeat	25
56	Dominican Republic	2005	Pineapples	17
56	Dominican Republic	2005	Plantains	108
56	Dominican Republic	2005	Potatoes	16
56	Dominican Republic	2005	Poultry Meat	87
56	Dominican Republic	2005	Pulses, Other	8
56	Dominican Republic	2005	Rice (Milled Equivalent)	129
56	Dominican Republic	2005	Roots, Other	5
56	Dominican Republic	2005	Soyabean Oil	26
56	Dominican Republic	2005	Spices, Other	1
56	Dominican Republic	2005	Sugar, Refined Equivalent	97
56	Dominican Republic	2005	Sunflowerseed Oil	3
56	Dominican Republic	2005	Sweet Potatoes	7
56	Dominican Republic	2005	Sweeteners, Other	1
56	Dominican Republic	2005	Tomatoes	77
56	Dominican Republic	2005	Vegetables, Other	55
56	Dominican Republic	2005	Wheat	56
56	Dominican Republic	2005	Wine	2
56	Dominican Republic	2005	Yams	4
56	Dominican Republic	2006	Apples	3
56	Dominican Republic	2006	Bananas	71
56	Dominican Republic	2006	Beans	14
56	Dominican Republic	2006	Beer	131
56	Dominican Republic	2006	Beverages, Alcoholic	13
56	Dominican Republic	2006	Bovine Meat	28
56	Dominican Republic	2006	Butter, Ghee	1
56	Dominican Republic	2006	Cassava	33
56	Dominican Republic	2006	Cheese	2
56	Dominican Republic	2006	Cocoa Beans	1
56	Dominican Republic	2006	Coconut Oil	2
56	Dominican Republic	2006	Coconuts - Incl Copra	7
56	Dominican Republic	2006	Coffee	13
56	Dominican Republic	2006	Crustaceans	1
56	Dominican Republic	2006	Demersal Fish	7
56	Dominican Republic	2006	Eggs	19
56	Dominican Republic	2006	Fats, Animals, Raw	4
56	Dominican Republic	2006	Freshwater Fish	1
56	Dominican Republic	2006	Fruits, Other	147
56	Dominican Republic	2006	Grapefruit	3
56	Dominican Republic	2006	Grapes	2
56	Dominican Republic	2006	Honey	1
56	Dominican Republic	2006	Lemons, Limes	1
56	Dominican Republic	2006	Maize	9
56	Dominican Republic	2006	Maize Germ Oil	2
56	Dominican Republic	2006	Marine Fish, Other	3
56	Dominican Republic	2006	Milk, Whole	121
56	Dominican Republic	2006	Mutton & Goat Meat	1
56	Dominican Republic	2006	Oats	1
56	Dominican Republic	2006	Offals, Edible	6
56	Dominican Republic	2006	Olive Oil	1
56	Dominican Republic	2006	Onions	12
56	Dominican Republic	2006	Oranges, Mandarines	30
56	Dominican Republic	2006	Palm Oil	12
56	Dominican Republic	2006	Pelagic Fish	10
56	Dominican Republic	2006	Pigmeat	30
56	Dominican Republic	2006	Pineapples	18
56	Dominican Republic	2006	Plantains	140
56	Dominican Republic	2006	Potatoes	13
56	Dominican Republic	2006	Poultry Meat	92
56	Dominican Republic	2006	Pulses, Other	10
56	Dominican Republic	2006	Rice (Milled Equivalent)	133
56	Dominican Republic	2006	Roots, Other	6
56	Dominican Republic	2006	Soyabean Oil	25
56	Dominican Republic	2006	Spices, Other	1
56	Dominican Republic	2006	Sugar, Refined Equivalent	92
56	Dominican Republic	2006	Sunflowerseed Oil	2
56	Dominican Republic	2006	Sweet Potatoes	5
56	Dominican Republic	2006	Sweeteners, Other	1
56	Dominican Republic	2006	Tomatoes	65
56	Dominican Republic	2006	Vegetables, Other	47
56	Dominican Republic	2006	Wheat	66
56	Dominican Republic	2006	Wine	2
56	Dominican Republic	2006	Yams	6
56	Dominican Republic	2007	Apples	3
56	Dominican Republic	2007	Bananas	73
56	Dominican Republic	2007	Beans	15
56	Dominican Republic	2007	Beer	110
56	Dominican Republic	2007	Beverages, Alcoholic	14
56	Dominican Republic	2007	Bovine Meat	29
56	Dominican Republic	2007	Butter, Ghee	1
56	Dominican Republic	2007	Cassava	33
56	Dominican Republic	2007	Cereals, Other	2
56	Dominican Republic	2007	Cheese	2
56	Dominican Republic	2007	Cocoa Beans	1
56	Dominican Republic	2007	Coconut Oil	2
56	Dominican Republic	2007	Coconuts - Incl Copra	7
56	Dominican Republic	2007	Coffee	13
56	Dominican Republic	2007	Crustaceans	1
56	Dominican Republic	2007	Demersal Fish	6
56	Dominican Republic	2007	Eggs	17
56	Dominican Republic	2007	Fats, Animals, Raw	4
56	Dominican Republic	2007	Freshwater Fish	1
56	Dominican Republic	2007	Fruits, Other	172
56	Dominican Republic	2007	Grapefruit	2

FAO country code	Country	Reference year	Food item	Gross food supply (g *capita*$^{-1}$ d^{-1})
56	Dominican Republic	2007	Grapes	2
56	Dominican Republic	2007	Honey	1
56	Dominican Republic	2007	Lemons, Limes	1
56	Dominican Republic	2007	Maize	12
56	Dominican Republic	2007	Maize Germ Oil	2
56	Dominican Republic	2007	Marine Fish, Other	3
56	Dominican Republic	2007	Milk, Whole	129
56	Dominican Republic	2007	Molluscs, Other	1
56	Dominican Republic	2007	Mutton & Goat Meat	1
56	Dominican Republic	2007	Oats	1
56	Dominican Republic	2007	Offals, Edible	6
56	Dominican Republic	2007	Onions	14
56	Dominican Republic	2007	Oranges, Mandarines	31
56	Dominican Republic	2007	Palm Oil	12
56	Dominican Republic	2007	Pelagic Fish	10
56	Dominican Republic	2007	Pigmeat	28
56	Dominican Republic	2007	Pineapples	24
56	Dominican Republic	2007	Plantains	128
56	Dominican Republic	2007	Potatoes	13
56	Dominican Republic	2007	Poultry Meat	95
56	Dominican Republic	2007	Pulses, Other	8
56	Dominican Republic	2007	Rice (Milled Equivalent)	126
56	Dominican Republic	2007	Roots, Other	7
56	Dominican Republic	2007	Soyabean Oil	28
56	Dominican Republic	2007	Spices, Other	1
56	Dominican Republic	2007	Sugar, Refined Equivalent	94
56	Dominican Republic	2007	Sunflowerseed Oil	2
56	Dominican Republic	2007	Sweet Potatoes	7
56	Dominican Republic	2007	Sweeteners, Other	2
56	Dominican Republic	2007	Tomatoes	54
56	Dominican Republic	2007	Vegetables, Other	45
56	Dominican Republic	2007	Wheat	70
56	Dominican Republic	2007	Wine	3
56	Dominican Republic	2007	Yams	7
56	Dominican Republic	2008	Apples	3
56	Dominican Republic	2008	Bananas	57
56	Dominican Republic	2008	Beans	14
56	Dominican Republic	2008	Beer	122
56	Dominican Republic	2008	Beverages, Alcoholic	13
56	Dominican Republic	2008	Bovine Meat	29
56	Dominican Republic	2008	Butter, Ghee	1
56	Dominican Republic	2008	Cassava	27
56	Dominican Republic	2008	Cheese	2
56	Dominican Republic	2008	Cocoa Beans	2
56	Dominican Republic	2008	Coconut Oil	2
56	Dominican Republic	2008	Coconuts - Incl Copra	8
56	Dominican Republic	2008	Coffee	12
56	Dominican Republic	2008	Crustaceans	1
56	Dominican Republic	2008	Demersal Fish	7
56	Dominican Republic	2008	Eggs	18
56	Dominican Republic	2008	Fats, Animals, Raw	4
56	Dominican Republic	2008	Freshwater Fish	1
56	Dominican Republic	2008	Fruits, Other	126
56	Dominican Republic	2008	Grapefruit	3
56	Dominican Republic	2008	Grapes	2
56	Dominican Republic	2008	Honey	1
56	Dominican Republic	2008	Lemons, Limes	1
56	Dominican Republic	2008	Maize	19
56	Dominican Republic	2008	Maize Germ Oil	3
56	Dominican Republic	2008	Marine Fish, Other	3
56	Dominican Republic	2008	Milk, Whole	141
56	Dominican Republic	2008	Molluscs, Other	1
56	Dominican Republic	2008	Mutton & Goat Meat	1
56	Dominican Republic	2008	Oats	1
56	Dominican Republic	2008	Offals, Edible	6
56	Dominican Republic	2008	Oilcrops Oil, Other	1
56	Dominican Republic	2008	Onions	15
56	Dominican Republic	2008	Oranges, Mandarines	27
56	Dominican Republic	2008	Palm Oil	13
56	Dominican Republic	2008	Pelagic Fish	11
56	Dominican Republic	2008	Pigmeat	28
56	Dominican Republic	2008	Pineapples	27
56	Dominican Republic	2008	Plantains	88
56	Dominican Republic	2008	Potatoes	11
56	Dominican Republic	2008	Poultry Meat	87
56	Dominican Republic	2008	Pulses, Other	7
56	Dominican Republic	2008	Rice (Milled Equivalent)	123
56	Dominican Republic	2008	Roots, Other	5
56	Dominican Republic	2008	Soyabean Oil	28
56	Dominican Republic	2008	Spices, Other	1
56	Dominican Republic	2008	Sugar, Refined Equivalent	92
56	Dominican Republic	2008	Sunflowerseed Oil	2
56	Dominican Republic	2008	Sweet Potatoes	4
56	Dominican Republic	2008	Sweeteners, Other	2
56	Dominican Republic	2008	Tomatoes	61
56	Dominican Republic	2008	Vegetables, Other	46
56	Dominican Republic	2008	Wheat	68
56	Dominican Republic	2008	Wine	2
56	Dominican Republic	2008	Yams	7
56	Dominican Republic	2009	Apples	3
56	Dominican Republic	2009	Bananas	69
56	Dominican Republic	2009	Beans	14
56	Dominican Republic	2009	Beer	106
56	Dominican Republic	2009	Beverages, Alcoholic	10
56	Dominican Republic	2009	Bovine Meat	29
56	Dominican Republic	2009	Butter, Ghee	1
56	Dominican Republic	2009	Cassava	41

FAO country code	Country	Reference year	Food item	Gross food supply (g *capita*$^{-1}$ d^{-1})
56	Dominican Republic	2009	Cheese	2
56	Dominican Republic	2009	Cocoa Beans	2
56	Dominican Republic	2009	Coconut Oil	2
56	Dominican Republic	2009	Coconuts - Incl Copra	8
56	Dominican Republic	2009	Coffee	9
56	Dominican Republic	2009	Crustaceans	1
56	Dominican Republic	2009	Demersal Fish	7
56	Dominican Republic	2009	Eggs	19
56	Dominican Republic	2009	Fats, Animals, Raw	4
56	Dominican Republic	2009	Freshwater Fish	1
56	Dominican Republic	2009	Fruits, Other	223
56	Dominican Republic	2009	Grapefruit	3
56	Dominican Republic	2009	Grapes	3
56	Dominican Republic	2009	Honey	1
56	Dominican Republic	2009	Lemons, Limes	2
56	Dominican Republic	2009	Maize	18
56	Dominican Republic	2009	Maize Germ Oil	1
56	Dominican Republic	2009	Marine Fish, Other	2
56	Dominican Republic	2009	Milk, Whole	137
56	Dominican Republic	2009	Molluscs, Other	1
56	Dominican Republic	2009	Mutton & Goat Meat	1
56	Dominican Republic	2009	Oats	2
56	Dominican Republic	2009	Offals, Edible	6
56	Dominican Republic	2009	Oilcrops Oil, Other	1
56	Dominican Republic	2009	Onions	15
56	Dominican Republic	2009	Oranges, Mandarines	32
56	Dominican Republic	2009	Palm Oil	14
56	Dominican Republic	2009	Pelagic Fish	13
56	Dominican Republic	2009	Pigmeat	28
56	Dominican Republic	2009	Pineapples	31
56	Dominican Republic	2009	Plantains	128
56	Dominican Republic	2009	Potatoes	11
56	Dominican Republic	2009	Poultry Meat	93
56	Dominican Republic	2009	Pulses, Other	10
56	Dominican Republic	2009	Rice (Milled Equivalent)	133
56	Dominican Republic	2009	Roots, Other	6
56	Dominican Republic	2009	Soyabean Oil	28
56	Dominican Republic	2009	Spices, Other	1
56	Dominican Republic	2009	Sugar, Refined Equivalent	85
56	Dominican Republic	2009	Sunflowerseed Oil	2
56	Dominican Republic	2009	Sweet Potatoes	9
56	Dominican Republic	2009	Sweeteners, Other	2
56	Dominican Republic	2009	Tomatoes	58
56	Dominican Republic	2009	Vegetables, Other	52
56	Dominican Republic	2009	Wheat	52
56	Dominican Republic	2009	Wine	2
56	Dominican Republic	2009	Yams	7
56	Dominican Republic	2010	Apples	4
56	Dominican Republic	2010	Bananas	88
56	Dominican Republic	2010	Beans	16
56	Dominican Republic	2010	Beer	130
56	Dominican Republic	2010	Beverages, Alcoholic	11
56	Dominican Republic	2010	Bovine Meat	32
56	Dominican Republic	2010	Butter, Ghee	1
56	Dominican Republic	2010	Cassava	52
56	Dominican Republic	2010	Cheese	3
56	Dominican Republic	2010	Cocoa Beans	2
56	Dominican Republic	2010	Coconut Oil	2
56	Dominican Republic	2010	Coconuts - Incl Copra	19
56	Dominican Republic	2010	Coffee	5
56	Dominican Republic	2010	Crustaceans	1
56	Dominican Republic	2010	Demersal Fish	7
56	Dominican Republic	2010	Eggs	22
56	Dominican Republic	2010	Fats, Animals, Raw	4
56	Dominican Republic	2010	Freshwater Fish	1
56	Dominican Republic	2010	Fruits, Other	304
56	Dominican Republic	2010	Grapefruit	5
56	Dominican Republic	2010	Grapes	3
56	Dominican Republic	2010	Honey	1
56	Dominican Republic	2010	Lemons, Limes	1
56	Dominican Republic	2010	Maize	17
56	Dominican Republic	2010	Maize Germ Oil	1
56	Dominican Republic	2010	Marine Fish, Other	2
56	Dominican Republic	2010	Milk, Whole	117
56	Dominican Republic	2010	Molluscs, Other	1
56	Dominican Republic	2010	Mutton & Goat Meat	1
56	Dominican Republic	2010	Oats	1
56	Dominican Republic	2010	Offals, Edible	7
56	Dominican Republic	2010	Onions	16
56	Dominican Republic	2010	Oranges, Mandarines	33
56	Dominican Republic	2010	Palm Oil	16
56	Dominican Republic	2010	Pelagic Fish	13
56	Dominican Republic	2010	Pigmeat	29
56	Dominican Republic	2010	Pineapples	38
56	Dominican Republic	2010	Plantains	119
56	Dominican Republic	2010	Potatoes	13
56	Dominican Republic	2010	Poultry Meat	96
56	Dominican Republic	2010	Pulses, Other	10
56	Dominican Republic	2010	Rice (Milled Equivalent)	135
56	Dominican Republic	2010	Roots, Other	7
56	Dominican Republic	2010	Soyabean Oil	26
56	Dominican Republic	2010	Spices, Other	1
56	Dominican Republic	2010	Sugar, Refined Equivalent	79
56	Dominican Republic	2010	Sunflowerseed Oil	1
56	Dominican Republic	2010	Sweet Potatoes	11
56	Dominican Republic	2010	Sweeteners, Other	3
56	Dominican Republic	2010	Tomatoes	58

FAO country code	Country	Reference year	Food item	Gross food supply (g $capita^{-1}$ d^{-1})
56	Dominican Republic	2010	Vegetables, Other	53
56	Dominican Republic	2010	Wheat	79
56	Dominican Republic	2010	Wine	2
56	Dominican Republic	2010	Yams	7
56	Dominican Republic	2011	Apples	4
56	Dominican Republic	2011	Bananas	120
56	Dominican Republic	2011	Beans	21
56	Dominican Republic	2011	Beer	130
56	Dominican Republic	2011	Beverages, Alcoholic	10
56	Dominican Republic	2011	Bovine Meat	28
56	Dominican Republic	2011	Butter, Ghee	1
56	Dominican Republic	2011	Cassava	45
56	Dominican Republic	2011	Cheese	3
56	Dominican Republic	2011	Cocoa Beans	2
56	Dominican Republic	2011	Coconut Oil	2
56	Dominican Republic	2011	Coconuts - Incl Copra	23
56	Dominican Republic	2011	Coffee	6
56	Dominican Republic	2011	Crustaceans	1
56	Dominican Republic	2011	Demersal Fish	7
56	Dominican Republic	2011	Eggs	19
56	Dominican Republic	2011	Fats, Animals, Raw	4
56	Dominican Republic	2011	Freshwater Fish	1
56	Dominican Republic	2011	Fruits, Other	293
56	Dominican Republic	2011	Grapefruit	3
56	Dominican Republic	2011	Grapes	2
56	Dominican Republic	2011	Honey	1
56	Dominican Republic	2011	Lemons, Limes	2
56	Dominican Republic	2011	Maize	17
56	Dominican Republic	2011	Maize Germ Oil	1
56	Dominican Republic	2011	Marine Fish, Other	2
56	Dominican Republic	2011	Milk, Whole	110
56	Dominican Republic	2011	Molluscs, Other	1
56	Dominican Republic	2011	Mutton & Goat Meat	1
56	Dominican Republic	2011	Oats	1
56	Dominican Republic	2011	Offals, Edible	6
56	Dominican Republic	2011	Olive Oil	1
56	Dominican Republic	2011	Onions	21
56	Dominican Republic	2011	Oranges, Mandarines	33
56	Dominican Republic	2011	Palm Oil	21
56	Dominican Republic	2011	Pelagic Fish	13
56	Dominican Republic	2011	Pigmeat	32
56	Dominican Republic	2011	Pineapples	52
56	Dominican Republic	2011	Plantains	120
56	Dominican Republic	2011	Potatoes	14
56	Dominican Republic	2011	Poultry Meat	92
56	Dominican Republic	2011	Pulses, Other	10
56	Dominican Republic	2011	Rice (Milled Equivalent)	135
56	Dominican Republic	2011	Roots, Other	8
56	Dominican Republic	2011	Soyabean Oil	26
56	Dominican Republic	2011	Spices, Other	1
56	Dominican Republic	2011	Sugar, Refined Equivalent	79
56	Dominican Republic	2011	Sunflowerseed Oil	1
56	Dominican Republic	2011	Sweet Potatoes	9
56	Dominican Republic	2011	Sweeteners, Other	1
56	Dominican Republic	2011	Tomatoes	69
56	Dominican Republic	2011	Vegetables, Other	57
56	Dominican Republic	2011	Wheat	83
56	Dominican Republic	2011	Wine	3
56	Dominican Republic	2011	Yams	7
58	Ecuador	1992	Apples	6
58	Ecuador	1992	Bananas	220
58	Ecuador	1992	Barley	7
58	Ecuador	1992	Beans	8
58	Ecuador	1992	Beer	47
58	Ecuador	1992	Beverages, Alcoholic	4
58	Ecuador	1992	Bovine Meat	29
58	Ecuador	1992	Butter, Ghee	2
58	Ecuador	1992	Cassava	15
58	Ecuador	1992	Cheese	2
58	Ecuador	1992	Citrus, Other	10
58	Ecuador	1992	Cocoa Beans	4
58	Ecuador	1992	Coconut Oil	1
58	Ecuador	1992	Coconuts - Incl Copra	2
58	Ecuador	1992	Coffee	11
58	Ecuador	1992	Cottonseed Oil	1
58	Ecuador	1992	Crustaceans	6
58	Ecuador	1992	Demersal Fish	1
58	Ecuador	1992	Eggs	12
58	Ecuador	1992	Fats, Animals, Raw	7
58	Ecuador	1992	Freshwater Fish	1
58	Ecuador	1992	Fruits, Other	35
58	Ecuador	1992	Grapes	1
58	Ecuador	1992	Groundnuts (Shelled Eq)	1
58	Ecuador	1992	Lemons, Limes	2
58	Ecuador	1992	Maize	15
58	Ecuador	1992	Marine Fish, Other	1
58	Ecuador	1992	Meat, Other	2
58	Ecuador	1992	Milk, Whole	223
58	Ecuador	1992	Mutton & Goat Meat	1
58	Ecuador	1992	Oats	6
58	Ecuador	1992	Offals, Edible	7
58	Ecuador	1992	Oranges, Mandarines	23
58	Ecuador	1992	Palm Oil	26
58	Ecuador	1992	Palmkernel Oil	1
58	Ecuador	1992	Peas	2
58	Ecuador	1992	Pelagic Fish	11
58	Ecuador	1992	Pigmeat	21

FAO country code	Country	Reference year	Food item	Gross food supply (g $capita^{-1}$ d^{-1})
58	Ecuador	1992	Pineapples	10
58	Ecuador	1992	Plantains	177
58	Ecuador	1992	Potatoes	97
58	Ecuador	1992	Poultry Meat	21
58	Ecuador	1992	Pulses, Other	4
58	Ecuador	1992	Rice (Milled Equivalent)	131
58	Ecuador	1992	Roots, Other	1
58	Ecuador	1992	Soyabean Oil	13
58	Ecuador	1992	Sugar, Refined Equivalent	66
58	Ecuador	1992	Sunflowerseed Oil	3
58	Ecuador	1992	Sweeteners, Other	6
58	Ecuador	1992	Tomatoes	25
58	Ecuador	1992	Vegetables, Other	57
58	Ecuador	1992	Wheat	95
58	Ecuador	1992	Wine	1
58	Ecuador	1993	Apples	8
58	Ecuador	1993	Bananas	345
58	Ecuador	1993	Barley	6
58	Ecuador	1993	Beans	8
58	Ecuador	1993	Beer	42
58	Ecuador	1993	Beverages, Alcoholic	4
58	Ecuador	1993	Bovine Meat	32
58	Ecuador	1993	Butter, Ghee	2
58	Ecuador	1993	Cassava	14
58	Ecuador	1993	Cheese	3
58	Ecuador	1993	Citrus, Other	6
58	Ecuador	1993	Cocoa Beans	4
58	Ecuador	1993	Coconut Oil	1
58	Ecuador	1993	Coconuts - Incl Copra	2
58	Ecuador	1993	Coffee	9
58	Ecuador	1993	Crustaceans	7
58	Ecuador	1993	Demersal Fish	1
58	Ecuador	1993	Eggs	11
58	Ecuador	1993	Fats, Animals, Raw	7
58	Ecuador	1993	Freshwater Fish	1
58	Ecuador	1993	Fruits, Other	21
58	Ecuador	1993	Grapefruit	1
58	Ecuador	1993	Grapes	1
58	Ecuador	1993	Groundnuts (Shelled Eq)	1
58	Ecuador	1993	Lemons, Limes	3
58	Ecuador	1993	Maize	13
58	Ecuador	1993	Marine Fish, Other	1
58	Ecuador	1993	Meat, Other	2
58	Ecuador	1993	Milk, Whole	228
58	Ecuador	1993	Mutton & Goat Meat	2
58	Ecuador	1993	Oats	5
58	Ecuador	1993	Offals, Edible	8
58	Ecuador	1993	Oranges, Mandarines	26
58	Ecuador	1993	Palm Oil	28
58	Ecuador	1993	Palmkernel Oil	1
58	Ecuador	1993	Peas	2
58	Ecuador	1993	Pelagic Fish	10
58	Ecuador	1993	Pigmeat	21
58	Ecuador	1993	Pineapples	11
58	Ecuador	1993	Plantains	145
58	Ecuador	1993	Potatoes	79
58	Ecuador	1993	Poultry Meat	20
58	Ecuador	1993	Pulses, Other	3
58	Ecuador	1993	Rice (Milled Equivalent)	159
58	Ecuador	1993	Soyabean Oil	9
58	Ecuador	1993	Sugar, Refined Equivalent	66
58	Ecuador	1993	Sweeteners, Other	1
58	Ecuador	1993	Tomatoes	21
58	Ecuador	1993	Vegetables, Other	58
58	Ecuador	1993	Wheat	88
58	Ecuador	1993	Wine	2
58	Ecuador	1994	Apples	10
58	Ecuador	1994	Bananas	377
58	Ecuador	1994	Barley	4
58	Ecuador	1994	Beans	6
58	Ecuador	1994	Beer	25
58	Ecuador	1994	Beverages, Alcoholic	5
58	Ecuador	1994	Bovine Meat	32
58	Ecuador	1994	Butter, Ghee	2
58	Ecuador	1994	Cassava	14
58	Ecuador	1994	Cheese	3
58	Ecuador	1994	Citrus, Other	5
58	Ecuador	1994	Cocoa Beans	2
58	Ecuador	1994	Coconut Oil	1
58	Ecuador	1994	Coconuts - Incl Copra	2
58	Ecuador	1994	Coffee	9
58	Ecuador	1994	Crustaceans	6
58	Ecuador	1994	Demersal Fish	3
58	Ecuador	1994	Eggs	11
58	Ecuador	1994	Fats, Animals, Raw	7
58	Ecuador	1994	Fruits, Other	29
58	Ecuador	1994	Grapefruit	1
58	Ecuador	1994	Grapes	2
58	Ecuador	1994	Groundnuts (Shelled Eq)	1
58	Ecuador	1994	Lemons, Limes	3
58	Ecuador	1994	Maize	11
58	Ecuador	1994	Meat, Other	2
58	Ecuador	1994	Milk, Whole	247
58	Ecuador	1994	Mutton & Goat Meat	2
58	Ecuador	1994	Oats	6
58	Ecuador	1994	Offals, Edible	8
58	Ecuador	1994	Oranges, Mandarines	26

FAO country code	Country	Reference year	Food item	Gross food supply (g *capita*$^{-1}$ d^{-1})
58	Ecuador	1994	Palm Oil	26
58	Ecuador	1994	Palmkernel Oil	1
58	Ecuador	1994	Peas	2
58	Ecuador	1994	Pelagic Fish	10
58	Ecuador	1994	Pigmeat	21
58	Ecuador	1994	Pineapples	13
58	Ecuador	1994	Plantains	159
58	Ecuador	1994	Potatoes	100
58	Ecuador	1994	Poultry Meat	26
58	Ecuador	1994	Pulses, Other	4
58	Ecuador	1994	Rice (Milled Equivalent)	170
58	Ecuador	1994	Roots, Other	1
58	Ecuador	1994	Soyabean Oil	8
58	Ecuador	1994	Sugar, Refined Equivalent	67
58	Ecuador	1994	Sweeteners, Other	2
58	Ecuador	1994	Tomatoes	18
58	Ecuador	1994	Vegetables, Other	52
58	Ecuador	1994	Wheat	89
58	Ecuador	1994	Wine	4
58	Ecuador	1995	Apples	8
58	Ecuador	1995	Bananas	272
58	Ecuador	1995	Barley	9
58	Ecuador	1995	Beans	6
58	Ecuador	1995	Beer	21
58	Ecuador	1995	Beverages, Alcoholic	7
58	Ecuador	1995	Bovine Meat	36
58	Ecuador	1995	Butter, Ghee	2
58	Ecuador	1995	Cassava	14
58	Ecuador	1995	Cheese	3
58	Ecuador	1995	Citrus, Other	5
58	Ecuador	1995	Cocoa Beans	4
58	Ecuador	1995	Coconut Oil	1
58	Ecuador	1995	Coconuts - Incl Copra	2
58	Ecuador	1995	Coffee	8
58	Ecuador	1995	Crustaceans	6
58	Ecuador	1995	Eggs	12
58	Ecuador	1995	Fats, Animals, Raw	8
58	Ecuador	1995	Fruits, Other	27
58	Ecuador	1995	Grapefruit	1
58	Ecuador	1995	Grapes	2
58	Ecuador	1995	Groundnuts (Shelled Eq)	1
58	Ecuador	1995	Lemons, Limes	3
58	Ecuador	1995	Maize	10
58	Ecuador	1995	Marine Fish, Other	4
58	Ecuador	1995	Meat, Other	2
58	Ecuador	1995	Milk, Whole	265
58	Ecuador	1995	Mutton & Goat Meat	2
58	Ecuador	1995	Oats	3
58	Ecuador	1995	Offals, Edible	9
58	Ecuador	1995	Oranges, Mandarines	25
58	Ecuador	1995	Palm Oil	27
58	Ecuador	1995	Peas	2
58	Ecuador	1995	Pelagic Fish	11
58	Ecuador	1995	Pigmeat	22
58	Ecuador	1995	Pineapples	10
58	Ecuador	1995	Plantains	107
58	Ecuador	1995	Potatoes	84
58	Ecuador	1995	Poultry Meat	26
58	Ecuador	1995	Pulses, Other	4
58	Ecuador	1995	Rice (Milled Equivalent)	152
58	Ecuador	1995	Roots, Other	1
58	Ecuador	1995	Soyabean Oil	15
58	Ecuador	1995	Sugar, Refined Equivalent	62
58	Ecuador	1995	Sweeteners, Other	1
58	Ecuador	1995	Tea	1
58	Ecuador	1995	Tomatoes	14
58	Ecuador	1995	Vegetables, Other	49
58	Ecuador	1995	Wheat	88
58	Ecuador	1995	Wine	2
58	Ecuador	1996	Apples	14
58	Ecuador	1996	Bananas	271
58	Ecuador	1996	Barley	10
58	Ecuador	1996	Beans	5
58	Ecuador	1996	Beer	23
58	Ecuador	1996	Beverages, Alcoholic	4
58	Ecuador	1996	Bovine Meat	36
58	Ecuador	1996	Butter, Ghee	2
58	Ecuador	1996	Cassava	14
58	Ecuador	1996	Cheese	4
58	Ecuador	1996	Citrus, Other	7
58	Ecuador	1996	Cocoa Beans	6
58	Ecuador	1996	Coconut Oil	1
58	Ecuador	1996	Coconuts - Incl Copra	2
58	Ecuador	1996	Coffee	10
58	Ecuador	1996	Crustaceans	6
58	Ecuador	1996	Eggs	10
58	Ecuador	1996	Fats, Animals, Raw	9
58	Ecuador	1996	Fruits, Other	38
58	Ecuador	1996	Grapefruit	1
58	Ecuador	1996	Grapes	2
58	Ecuador	1996	Groundnuts (Shelled Eq)	1
58	Ecuador	1996	Lemons, Limes	5
58	Ecuador	1996	Maize	11
58	Ecuador	1996	Marine Fish, Other	1
58	Ecuador	1996	Meat, Other	2
58	Ecuador	1996	Milk, Whole	302
58	Ecuador	1996	Mutton & Goat Meat	2

FAO country code	Country	Reference year	Food item	Gross food supply (g *capita*$^{-1}$ d^{-1})
58	Ecuador	1996	Oats	8
58	Ecuador	1996	Offals, Edible	9
58	Ecuador	1996	Oranges, Mandarines	25
58	Ecuador	1996	Palm Oil	25
58	Ecuador	1996	Peas	2
58	Ecuador	1996	Pelagic Fish	15
58	Ecuador	1996	Pigmeat	25
58	Ecuador	1996	Pineapples	10
58	Ecuador	1996	Plantains	141
58	Ecuador	1996	Potatoes	79
58	Ecuador	1996	Poultry Meat	36
58	Ecuador	1996	Pulses, Other	5
58	Ecuador	1996	Rice (Milled Equivalent)	151
58	Ecuador	1996	Roots, Other	1
58	Ecuador	1996	Soyabean Oil	13
58	Ecuador	1996	Sugar, Refined Equivalent	52
58	Ecuador	1996	Sweeteners, Other	1
58	Ecuador	1996	Tomatoes	15
58	Ecuador	1996	Vegetables, Other	50
58	Ecuador	1996	Wheat	91
58	Ecuador	1996	Wine	2
58	Ecuador	1997	Apples	11
58	Ecuador	1997	Bananas	414
58	Ecuador	1997	Barley	5
58	Ecuador	1997	Beans	4
58	Ecuador	1997	Beer	41
58	Ecuador	1997	Beverages, Alcoholic	1
58	Ecuador	1997	Bovine Meat	36
58	Ecuador	1997	Butter, Ghee	2
58	Ecuador	1997	Cassava	19
58	Ecuador	1997	Cheese	5
58	Ecuador	1997	Citrus, Other	16
58	Ecuador	1997	Cocoa Beans	5
58	Ecuador	1997	Coconut Oil	1
58	Ecuador	1997	Coconuts - Incl Copra	2
58	Ecuador	1997	Coffee	8
58	Ecuador	1997	Crustaceans	7
58	Ecuador	1997	Eggs	10
58	Ecuador	1997	Fats, Animals, Raw	9
58	Ecuador	1997	Fruits, Other	50
58	Ecuador	1997	Grapefruit	1
58	Ecuador	1997	Grapes	2
58	Ecuador	1997	Groundnuts (Shelled Eq)	2
58	Ecuador	1997	Lemons, Limes	3
58	Ecuador	1997	Maize	14
58	Ecuador	1997	Marine Fish, Other	1
58	Ecuador	1997	Meat, Other	2
58	Ecuador	1997	Milk, Whole	286
58	Ecuador	1997	Mutton & Goat Meat	2
58	Ecuador	1997	Oats	5
58	Ecuador	1997	Offals, Edible	9
58	Ecuador	1997	Oranges, Mandarines	49
58	Ecuador	1997	Palm Oil	26
58	Ecuador	1997	Palmkernel Oil	1
58	Ecuador	1997	Peas	3
58	Ecuador	1997	Pelagic Fish	16
58	Ecuador	1997	Pigmeat	25
58	Ecuador	1997	Pineapples	4
58	Ecuador	1997	Plantains	134
58	Ecuador	1997	Potatoes	110
58	Ecuador	1997	Poultry Meat	41
58	Ecuador	1997	Pulses, Other	5
58	Ecuador	1997	Rice (Milled Equivalent)	125
58	Ecuador	1997	Roots, Other	1
58	Ecuador	1997	Soyabean Oil	11
58	Ecuador	1997	Sugar, Refined Equivalent	50
58	Ecuador	1997	Sweeteners, Other	1
58	Ecuador	1997	Tomatoes	11
58	Ecuador	1997	Vegetables, Other	56
58	Ecuador	1997	Wheat	119
58	Ecuador	1997	Wine	1
58	Ecuador	1998	Apples	14
58	Ecuador	1998	Bananas	325
58	Ecuador	1998	Barley	5
58	Ecuador	1998	Beans	4
58	Ecuador	1998	Beer	49
58	Ecuador	1998	Beverages, Alcoholic	2
58	Ecuador	1998	Bovine Meat	36
58	Ecuador	1998	Butter, Ghee	2
58	Ecuador	1998	Cassava	14
58	Ecuador	1998	Cheese	6
58	Ecuador	1998	Citrus, Other	4
58	Ecuador	1998	Cocoa Beans	2
58	Ecuador	1998	Coconuts - Incl Copra	1
58	Ecuador	1998	Coffee	8
58	Ecuador	1998	Crustaceans	7
58	Ecuador	1998	Demersal Fish	1
58	Ecuador	1998	Eggs	11
58	Ecuador	1998	Fats, Animals, Raw	8
58	Ecuador	1998	Freshwater Fish	1
58	Ecuador	1998	Fruits, Other	57
58	Ecuador	1998	Grapefruit	1
58	Ecuador	1998	Grapes	3
58	Ecuador	1998	Lemons, Limes	3
58	Ecuador	1998	Maize	13
58	Ecuador	1998	Marine Fish, Other	1
58	Ecuador	1998	Meat, Other	2

FAO country code	Country	Reference year	Food item	Gross food supply (g *capita*$^{-1}$ d^{-1})
58	Ecuador	1998	Milk, Whole	300
58	Ecuador	1998	Mutton & Goat Meat	3
58	Ecuador	1998	Oats	4
58	Ecuador	1998	Offals, Edible	9
58	Ecuador	1998	Oranges, Mandarines	28
58	Ecuador	1998	Palm Oil	26
58	Ecuador	1998	Palmkernel Oil	2
58	Ecuador	1998	Peas	4
58	Ecuador	1998	Pelagic Fish	12
58	Ecuador	1998	Pigmeat	23
58	Ecuador	1998	Pineapples	15
58	Ecuador	1998	Plantains	70
58	Ecuador	1998	Potatoes	83
58	Ecuador	1998	Poultry Meat	25
58	Ecuador	1998	Pulses, Other	5
58	Ecuador	1998	Rice (Milled Equivalent)	128
58	Ecuador	1998	Roots, Other	1
58	Ecuador	1998	Soyabean Oil	11
58	Ecuador	1998	Sugar, Refined Equivalent	51
58	Ecuador	1998	Sweeteners, Other	1
58	Ecuador	1998	Tomatoes	15
58	Ecuador	1998	Vegetables, Other	56
58	Ecuador	1998	Wheat	119
58	Ecuador	1998	Wine	1
58	Ecuador	1999	Apples	7
58	Ecuador	1999	Bananas	352
58	Ecuador	1999	Barley	4
58	Ecuador	1999	Beans	4
58	Ecuador	1999	Beer	45
58	Ecuador	1999	Beverages, Alcoholic	1
58	Ecuador	1999	Bovine Meat	37
58	Ecuador	1999	Butter, Ghee	2
58	Ecuador	1999	Cassava	16
58	Ecuador	1999	Cheese	7
58	Ecuador	1999	Citrus, Other	3
58	Ecuador	1999	Cocoa Beans	3
58	Ecuador	1999	Coconuts - Incl Copra	1
58	Ecuador	1999	Coffee	10
58	Ecuador	1999	Crustaceans	6
58	Ecuador	1999	Demersal Fish	7
58	Ecuador	1999	Eggs	10
58	Ecuador	1999	Fats, Animals, Raw	8
58	Ecuador	1999	Freshwater Fish	1
58	Ecuador	1999	Fruits, Other	118
58	Ecuador	1999	Grapefruit	1
58	Ecuador	1999	Grapes	1
58	Ecuador	1999	Groundnuts (Shelled Eq)	2
58	Ecuador	1999	Lemons, Limes	6
58	Ecuador	1999	Maize	11
58	Ecuador	1999	Meat, Other	2
58	Ecuador	1999	Milk, Whole	264
58	Ecuador	1999	Mutton & Goat Meat	2
58	Ecuador	1999	Oats	7
58	Ecuador	1999	Offals, Edible	9
58	Ecuador	1999	Oranges, Mandarines	27
58	Ecuador	1999	Palm Oil	27
58	Ecuador	1999	Palmkernel Oil	1
58	Ecuador	1999	Peas	3
58	Ecuador	1999	Pelagic Fish	5
58	Ecuador	1999	Pepper	1
58	Ecuador	1999	Pigmeat	25
58	Ecuador	1999	Pineapples	12
58	Ecuador	1999	Plantains	91
58	Ecuador	1999	Potatoes	99
58	Ecuador	1999	Poultry Meat	33
58	Ecuador	1999	Pulses, Other	4
58	Ecuador	1999	Rice (Milled Equivalent)	140
58	Ecuador	1999	Roots, Other	1
58	Ecuador	1999	Soyabean Oil	12
58	Ecuador	1999	Sugar, Refined Equivalent	48
58	Ecuador	1999	Sweeteners, Other	1
58	Ecuador	1999	Tomatoes	14
58	Ecuador	1999	Vegetables, Other	40
58	Ecuador	1999	Wheat	106
58	Ecuador	2000	Apples	6
58	Ecuador	2000	Bananas	356
58	Ecuador	2000	Barley	4
58	Ecuador	2000	Beans	4
58	Ecuador	2000	Beer	36
58	Ecuador	2000	Beverages, Alcoholic	1
58	Ecuador	2000	Bovine Meat	37
58	Ecuador	2000	Butter, Ghee	2
58	Ecuador	2000	Cassava	15
58	Ecuador	2000	Cheese	7
58	Ecuador	2000	Citrus, Other	3
58	Ecuador	2000	Cocoa Beans	4
58	Ecuador	2000	Coconuts - Incl Copra	1
58	Ecuador	2000	Coffee	1
58	Ecuador	2000	Crustaceans	2
58	Ecuador	2000	Demersal Fish	10
58	Ecuador	2000	Eggs	12
58	Ecuador	2000	Fats, Animals, Raw	9
58	Ecuador	2000	Freshwater Fish	2
58	Ecuador	2000	Fruits, Other	66
58	Ecuador	2000	Grapefruit	1
58	Ecuador	2000	Grapes	2
58	Ecuador	2000	Groundnuts (Shelled Eq)	1

FAO country code	Country	Reference year	Food item	Gross food supply (g $capita^{-1}$ d^{-1})
58	Ecuador	2000	Lemons, Limes	7
58	Ecuador	2000	Maize	11
58	Ecuador	2000	Marine Fish, Other	1
58	Ecuador	2000	Meat, Other	2
58	Ecuador	2000	Milk, Whole	307
58	Ecuador	2000	Mutton & Goat Meat	2
58	Ecuador	2000	Oats	8
58	Ecuador	2000	Offals, Edible	10
58	Ecuador	2000	Oranges, Mandarines	33
58	Ecuador	2000	Palm Oil	28
58	Ecuador	2000	Palmkernel Oil	1
58	Ecuador	2000	Peas	3
58	Ecuador	2000	Pelagic Fish	4
58	Ecuador	2000	Pepper	1
58	Ecuador	2000	Pigmeat	25
58	Ecuador	2000	Pineapples	7
58	Ecuador	2000	Plantains	75
58	Ecuador	2000	Potatoes	29
58	Ecuador	2000	Poultry Meat	42
58	Ecuador	2000	Pulses, Other	4
58	Ecuador	2000	Rice (Milled Equivalent)	152
58	Ecuador	2000	Roots, Other	1
58	Ecuador	2000	Soyabean Oil	8
58	Ecuador	2000	Sugar, Refined Equivalent	46
58	Ecuador	2000	Sweeteners, Other	2
58	Ecuador	2000	Tomatoes	14
58	Ecuador	2000	Vegetables, Other	47
58	Ecuador	2000	Wheat	93
58	Ecuador	2001	Apples	11
58	Ecuador	2001	Bananas	281
58	Ecuador	2001	Barley	3
58	Ecuador	2001	Beans	1
58	Ecuador	2001	Beer	37
58	Ecuador	2001	Beverages, Alcoholic	1
58	Ecuador	2001	Bovine Meat	40
58	Ecuador	2001	Butter, Ghee	3
58	Ecuador	2001	Cassava	14
58	Ecuador	2001	Cheese	8
58	Ecuador	2001	Citrus, Other	4
58	Ecuador	2001	Cocoa Beans	2
58	Ecuador	2001	Coconut Oil	1
58	Ecuador	2001	Coconuts - Incl Copra	1
58	Ecuador	2001	Coffee	1
58	Ecuador	2001	Crustaceans	1
58	Ecuador	2001	Demersal Fish	2
58	Ecuador	2001	Eggs	12
58	Ecuador	2001	Fats, Animals, Raw	10
58	Ecuador	2001	Freshwater Fish	2
58	Ecuador	2001	Fruits, Other	73
58	Ecuador	2001	Grapefruit	1
58	Ecuador	2001	Grapes	2
58	Ecuador	2001	Groundnuts (Shelled Eq)	1
58	Ecuador	2001	Lemons, Limes	1
58	Ecuador	2001	Maize	12
58	Ecuador	2001	Marine Fish, Other	5
58	Ecuador	2001	Meat, Other	2
58	Ecuador	2001	Milk, Whole	328
58	Ecuador	2001	Mutton & Goat Meat	2
58	Ecuador	2001	Oats	5
58	Ecuador	2001	Offals, Edible	9
58	Ecuador	2001	Oranges, Mandarines	33
58	Ecuador	2001	Palm Oil	27
58	Ecuador	2001	Palmkernel Oil	2
58	Ecuador	2001	Peas	2
58	Ecuador	2001	Pelagic Fish	2
58	Ecuador	2001	Pepper	1
58	Ecuador	2001	Pigmeat	30
58	Ecuador	2001	Pineapples	5
58	Ecuador	2001	Plantains	103
58	Ecuador	2001	Potatoes	71
58	Ecuador	2001	Poultry Meat	44
58	Ecuador	2001	Pulses, Other	5
58	Ecuador	2001	Rice (Milled Equivalent)	119
58	Ecuador	2001	Roots, Other	1
58	Ecuador	2001	Soyabean Oil	7
58	Ecuador	2001	Sugar, Refined Equivalent	44
58	Ecuador	2001	Sweeteners, Other	2
58	Ecuador	2001	Tomatoes	8
58	Ecuador	2001	Vegetables, Other	42
58	Ecuador	2001	Wheat	119
58	Ecuador	2001	Wine	1
58	Ecuador	2002	Apples	10
58	Ecuador	2002	Bananas	243
58	Ecuador	2002	Barley	5
58	Ecuador	2002	Beer	40
58	Ecuador	2002	Beverages, Alcoholic	2
58	Ecuador	2002	Bovine Meat	43
58	Ecuador	2002	Butter, Ghee	3
58	Ecuador	2002	Cassava	14
58	Ecuador	2002	Cheese	10
58	Ecuador	2002	Citrus, Other	3
58	Ecuador	2002	Cocoa Beans	1
58	Ecuador	2002	Coconuts - Incl Copra	1
58	Ecuador	2002	Coffee	1
58	Ecuador	2002	Crustaceans	3
58	Ecuador	2002	Demersal Fish	2
58	Ecuador	2002	Eggs	12

FAO country code	Country	Reference year	Food item	Gross food supply (g *capita*$^{-1}$ d^{-1})
58	Ecuador	2002	Fats, Animals, Raw	10
58	Ecuador	2002	Freshwater Fish	3
58	Ecuador	2002	Fruits, Other	72
58	Ecuador	2002	Grapefruit	1
58	Ecuador	2002	Grapes	5
58	Ecuador	2002	Groundnuts (Shelled Eq)	1
58	Ecuador	2002	Lemons, Limes	2
58	Ecuador	2002	Maize	11
58	Ecuador	2002	Meat, Other	2
58	Ecuador	2002	Milk, Whole	344
58	Ecuador	2002	Mutton & Goat Meat	2
58	Ecuador	2002	Oats	8
58	Ecuador	2002	Offals, Edible	9
58	Ecuador	2002	Oranges, Mandarines	29
58	Ecuador	2002	Palm Oil	28
58	Ecuador	2002	Palmkernel Oil	2
58	Ecuador	2002	Peas	2
58	Ecuador	2002	Pelagic Fish	4
58	Ecuador	2002	Pigmeat	31
58	Ecuador	2002	Pineapples	8
58	Ecuador	2002	Plantains	116
58	Ecuador	2002	Potatoes	83
58	Ecuador	2002	Poultry Meat	44
58	Ecuador	2002	Pulses, Other	5
58	Ecuador	2002	Rice (Milled Equivalent)	127
58	Ecuador	2002	Roots, Other	1
58	Ecuador	2002	Soyabean Oil	14
58	Ecuador	2002	Sugar, Refined Equivalent	43
58	Ecuador	2002	Sweet Potatoes	1
58	Ecuador	2002	Sweeteners, Other	2
58	Ecuador	2002	Tomatoes	13
58	Ecuador	2002	Vegetables, Other	43
58	Ecuador	2002	Wheat	83
58	Ecuador	2002	Wine	1
58	Ecuador	2003	Apples	11
58	Ecuador	2003	Bananas	228
58	Ecuador	2003	Barley	4
58	Ecuador	2003	Beer	44
58	Ecuador	2003	Beverages, Alcoholic	2
58	Ecuador	2003	Bovine Meat	43
58	Ecuador	2003	Butter, Ghee	4
58	Ecuador	2003	Cassava	16
58	Ecuador	2003	Cheese	11
58	Ecuador	2003	Citrus, Other	4
58	Ecuador	2003	Cocoa Beans	2
58	Ecuador	2003	Coconuts - Incl Copra	1
58	Ecuador	2003	Coffee	1
58	Ecuador	2003	Crustaceans	5
58	Ecuador	2003	Demersal Fish	1
58	Ecuador	2003	Eggs	13
58	Ecuador	2003	Fats, Animals, Raw	10
58	Ecuador	2003	Freshwater Fish	4
58	Ecuador	2003	Fruits, Other	52
58	Ecuador	2003	Grapefruit	1
58	Ecuador	2003	Grapes	5
58	Ecuador	2003	Groundnuts (Shelled Eq)	1
58	Ecuador	2003	Lemons, Limes	1
58	Ecuador	2003	Maize	11
58	Ecuador	2003	Marine Fish, Other	3
58	Ecuador	2003	Meat, Other	2
58	Ecuador	2003	Milk, Whole	352
58	Ecuador	2003	Mutton & Goat Meat	3
58	Ecuador	2003	Oats	4
58	Ecuador	2003	Offals, Edible	9
58	Ecuador	2003	Oranges, Mandarines	20
58	Ecuador	2003	Palm Oil	24
58	Ecuador	2003	Palmkernel Oil	1
58	Ecuador	2003	Peas	2
58	Ecuador	2003	Pelagic Fish	4
58	Ecuador	2003	Pigmeat	32
58	Ecuador	2003	Pineapples	2
58	Ecuador	2003	Plantains	120
58	Ecuador	2003	Potatoes	65
58	Ecuador	2003	Poultry Meat	44
58	Ecuador	2003	Pulses, Other	4
58	Ecuador	2003	Rice (Milled Equivalent)	125
58	Ecuador	2003	Soyabean Oil	13
58	Ecuador	2003	Sugar, Refined Equivalent	44
58	Ecuador	2003	Sweeteners, Other	3
58	Ecuador	2003	Tomatoes	11
58	Ecuador	2003	Vegetables, Other	38
58	Ecuador	2003	Wheat	91
58	Ecuador	2003	Wine	1
58	Ecuador	2004	Apples	10
58	Ecuador	2004	Bananas	238
58	Ecuador	2004	Barley	1
58	Ecuador	2004	Beer	47
58	Ecuador	2004	Beverages, Alcoholic	3
58	Ecuador	2004	Bovine Meat	42
58	Ecuador	2004	Butter, Ghee	4
58	Ecuador	2004	Cassava	14
58	Ecuador	2004	Cheese	12
58	Ecuador	2004	Citrus, Other	3
58	Ecuador	2004	Cocoa Beans	2
58	Ecuador	2004	Coconuts - Incl Copra	1
58	Ecuador	2004	Crustaceans	4
58	Ecuador	2004	Demersal Fish	1

FAO countrycode	Country	Reference year	Food item	Gross food supply (g *capita*$^{-1}$ d^{-1})
58	Ecuador	2004	Eggs	13
58	Ecuador	2004	Fats, Animals, Raw	11
58	Ecuador	2004	Freshwater Fish	3
58	Ecuador	2004	Fruits, Other	53
58	Ecuador	2004	Grapefruit	1
58	Ecuador	2004	Grapes	4
58	Ecuador	2004	Groundnuts (Shelled Eq)	2
58	Ecuador	2004	Lemons, Limes	1
58	Ecuador	2004	Maize	11
58	Ecuador	2004	Marine Fish, Other	3
58	Ecuador	2004	Meat, Other	2
58	Ecuador	2004	Milk, Whole	366
58	Ecuador	2004	Mutton & Goat Meat	3
58	Ecuador	2004	Oats	9
58	Ecuador	2004	Offals, Edible	9
58	Ecuador	2004	Onions	1
58	Ecuador	2004	Oranges, Mandarines	29
58	Ecuador	2004	Palm Oil	25
58	Ecuador	2004	Palmkernel Oil	1
58	Ecuador	2004	Peas	1
58	Ecuador	2004	Pelagic Fish	5
58	Ecuador	2004	Pigmeat	33
58	Ecuador	2004	Pineapples	1
58	Ecuador	2004	Plantains	117
58	Ecuador	2004	Potatoes	79
58	Ecuador	2004	Poultry Meat	43
58	Ecuador	2004	Pulses, Other	4
58	Ecuador	2004	Rice (Milled Equivalent)	115
58	Ecuador	2004	Roots, Other	1
58	Ecuador	2004	Soyabean Oil	13
58	Ecuador	2004	Spices, Other	1
58	Ecuador	2004	Sugar, Refined Equivalent	42
58	Ecuador	2004	Sunflowerseed Oil	1
58	Ecuador	2004	Sweeteners, Other	4
58	Ecuador	2004	Tomatoes	18
58	Ecuador	2004	Vegetables, Other	43
58	Ecuador	2004	Wheat	94
58	Ecuador	2004	Wine	1
58	Ecuador	2005	Apples	12
58	Ecuador	2005	Bananas	207
58	Ecuador	2005	Barley	1
58	Ecuador	2005	Beans	1
58	Ecuador	2005	Beer	51
58	Ecuador	2005	Beverages, Alcoholic	3
58	Ecuador	2005	Bovine Meat	42
58	Ecuador	2005	Butter, Ghee	4
58	Ecuador	2005	Cassava	28
58	Ecuador	2005	Cheese	13
58	Ecuador	2005	Citrus, Other	4
58	Ecuador	2005	Cocoa Beans	1
58	Ecuador	2005	Coconuts - Incl Copra	1
58	Ecuador	2005	Crustaceans	5
58	Ecuador	2005	Eggs	13
58	Ecuador	2005	Fats, Animals, Raw	10
58	Ecuador	2005	Freshwater Fish	4
58	Ecuador	2005	Fruits, Other	59
58	Ecuador	2005	Grapefruit	1
58	Ecuador	2005	Grapes	3
58	Ecuador	2005	Groundnuts (Shelled Eq)	2
58	Ecuador	2005	Lemons, Limes	3
58	Ecuador	2005	Maize	12
58	Ecuador	2005	Marine Fish, Other	1
58	Ecuador	2005	Meat, Other	2
58	Ecuador	2005	Milk, Whole	375
58	Ecuador	2005	Mutton & Goat Meat	2
58	Ecuador	2005	Oats	4
58	Ecuador	2005	Offals, Edible	9
58	Ecuador	2005	Oranges, Mandarines	20
58	Ecuador	2005	Palm Oil	26
58	Ecuador	2005	Palmkernel Oil	1
58	Ecuador	2005	Peas	1
58	Ecuador	2005	Pelagic Fish	7
58	Ecuador	2005	Pigmeat	33
58	Ecuador	2005	Pineapples	2
58	Ecuador	2005	Plantains	95
58	Ecuador	2005	Potatoes	73
58	Ecuador	2005	Poultry Meat	42
58	Ecuador	2005	Pulses, Other	5
58	Ecuador	2005	Rice (Milled Equivalent)	125
58	Ecuador	2005	Soyabean Oil	14
58	Ecuador	2005	Spices, Other	1
58	Ecuador	2005	Sugar, Refined Equivalent	42
58	Ecuador	2005	Sunflowerseed Oil	1
58	Ecuador	2005	Sweet Potatoes	1
58	Ecuador	2005	Sweeteners, Other	4
58	Ecuador	2005	Tomatoes	15
58	Ecuador	2005	Vegetables, Other	48
58	Ecuador	2005	Wheat	99
58	Ecuador	2005	Wine	1
58	Ecuador	2006	Apples	12
58	Ecuador	2006	Bananas	181
58	Ecuador	2006	Barley	1
58	Ecuador	2006	Beans	1
58	Ecuador	2006	Beer	51
58	Ecuador	2006	Beverages, Alcoholic	3
58	Ecuador	2006	Bovine Meat	41
58	Ecuador	2006	Butter, Ghee	4

FAO country code	Country	Reference year	Food item	Gross food supply (g *capita*$^{-1}$ d^{-1})
58	Ecuador	2006	Cassava	11
58	Ecuador	2006	Cheese	14
58	Ecuador	2006	Citrus, Other	4
58	Ecuador	2006	Cocoa Beans	1
58	Ecuador	2006	Coconuts - Incl Copra	1
58	Ecuador	2006	Crustaceans	6
58	Ecuador	2006	Demersal Fish	2
58	Ecuador	2006	Eggs	14
58	Ecuador	2006	Fats, Animals, Raw	10
58	Ecuador	2006	Freshwater Fish	3
58	Ecuador	2006	Fruits, Other	60
58	Ecuador	2006	Grapefruit	1
58	Ecuador	2006	Grapes	3
58	Ecuador	2006	Groundnuts (Shelled Eq)	2
58	Ecuador	2006	Maize	12
58	Ecuador	2006	Marine Fish, Other	1
58	Ecuador	2006	Meat, Other	2
58	Ecuador	2006	Milk, Whole	383
58	Ecuador	2006	Mutton & Goat Meat	2
58	Ecuador	2006	Oats	7
58	Ecuador	2006	Offals, Edible	9
58	Ecuador	2006	Oranges, Mandarines	14
58	Ecuador	2006	Palm Oil	24
58	Ecuador	2006	Palmkernel Oil	1
58	Ecuador	2006	Peas	1
58	Ecuador	2006	Pelagic Fish	7
58	Ecuador	2006	Pigmeat	33
58	Ecuador	2006	Pineapples	1
58	Ecuador	2006	Plantains	95
58	Ecuador	2006	Potatoes	61
58	Ecuador	2006	Poultry Meat	59
58	Ecuador	2006	Pulses, Other	4
58	Ecuador	2006	Rice (Milled Equivalent)	122
58	Ecuador	2006	Soyabean Oil	12
58	Ecuador	2006	Spices, Other	1
58	Ecuador	2006	Sugar, Refined Equivalent	43
58	Ecuador	2006	Sunflowerseed Oil	1
58	Ecuador	2006	Sweeteners, Other	3
58	Ecuador	2006	Tomatoes	13
58	Ecuador	2006	Vegetables, Other	48
58	Ecuador	2006	Wheat	100
58	Ecuador	2006	Wine	1
58	Ecuador	2007	Apples	12
58	Ecuador	2007	Bananas	165
58	Ecuador	2007	Barley	1
58	Ecuador	2007	Beans	1
58	Ecuador	2007	Beer	67
58	Ecuador	2007	Beverages, Alcoholic	3
58	Ecuador	2007	Bovine Meat	45
58	Ecuador	2007	Butter, Ghee	4
58	Ecuador	2007	Cassava	2
58	Ecuador	2007	Cheese	15
58	Ecuador	2007	Citrus, Other	4
58	Ecuador	2007	Cocoa Beans	1
58	Ecuador	2007	Coconuts - Incl Copra	1
58	Ecuador	2007	Crustaceans	5
58	Ecuador	2007	Demersal Fish	1
58	Ecuador	2007	Eggs	14
58	Ecuador	2007	Fats, Animals, Raw	11
58	Ecuador	2007	Freshwater Fish	2
58	Ecuador	2007	Fruits, Other	59
58	Ecuador	2007	Grapefruit	1
58	Ecuador	2007	Grapes	4
58	Ecuador	2007	Groundnuts (Shelled Eq)	2
58	Ecuador	2007	Lemons, Limes	1
58	Ecuador	2007	Maize	11
58	Ecuador	2007	Marine Fish, Other	1
58	Ecuador	2007	Meat, Other	2
58	Ecuador	2007	Milk, Whole	388
58	Ecuador	2007	Mutton & Goat Meat	2
58	Ecuador	2007	Oats	9
58	Ecuador	2007	Offals, Edible	9
58	Ecuador	2007	Oranges, Mandarines	16
58	Ecuador	2007	Palm Oil	25
58	Ecuador	2007	Palmkernel Oil	2
58	Ecuador	2007	Peas	1
58	Ecuador	2007	Pelagic Fish	9
58	Ecuador	2007	Pigmeat	37
58	Ecuador	2007	Pineapples	1
58	Ecuador	2007	Plantains	87
58	Ecuador	2007	Potatoes	53
58	Ecuador	2007	Poultry Meat	65
58	Ecuador	2007	Pulses, Other	5
58	Ecuador	2007	Rice (Milled Equivalent)	120
58	Ecuador	2007	Soyabean Oil	12
58	Ecuador	2007	Spices, Other	1
58	Ecuador	2007	Sugar, Refined Equivalent	43
58	Ecuador	2007	Sunflowerseed Oil	2
58	Ecuador	2007	Sweet Potatoes	1
58	Ecuador	2007	Sweeteners, Other	4
58	Ecuador	2007	Tomatoes	14
58	Ecuador	2007	Vegetables, Other	47
58	Ecuador	2007	Wheat	97
58	Ecuador	2007	Wine	1
58	Ecuador	2008	Apples	10
58	Ecuador	2008	Bananas	181
58	Ecuador	2008	Barley	1

FAO countrycode	Country	Reference year	Food item	Gross food supply (g *capita*$^{-1}$ d^{-1})
58	Ecuador	2008	Beans	1
58	Ecuador	2008	Beer	69
58	Ecuador	2008	Beverages, Alcoholic	3
58	Ecuador	2008	Bovine Meat	47
58	Ecuador	2008	Butter, Ghee	4
58	Ecuador	2008	Cassava	15
58	Ecuador	2008	Cheese	16
58	Ecuador	2008	Citrus, Other	4
58	Ecuador	2008	Cocoa Beans	1
58	Ecuador	2008	Coconuts - Incl Copra	2
58	Ecuador	2008	Crustaceans	6
58	Ecuador	2008	Demersal Fish	1
58	Ecuador	2008	Eggs	15
58	Ecuador	2008	Fats, Animals, Raw	12
58	Ecuador	2008	Freshwater Fish	3
58	Ecuador	2008	Fruits, Other	71
58	Ecuador	2008	Grapefruit	1
58	Ecuador	2008	Grapes	4
58	Ecuador	2008	Groundnuts (Shelled Eq)	2
58	Ecuador	2008	Lemons, Limes	2
58	Ecuador	2008	Maize	11
58	Ecuador	2008	Marine Fish, Other	1
58	Ecuador	2008	Meat, Other	2
58	Ecuador	2008	Milk, Whole	392
58	Ecuador	2008	Mutton & Goat Meat	2
58	Ecuador	2008	Oats	4
58	Ecuador	2008	Offals, Edible	10
58	Ecuador	2008	Oranges, Mandarines	15
58	Ecuador	2008	Palm Oil	25
58	Ecuador	2008	Palmkernel Oil	2
58	Ecuador	2008	Peas	1
58	Ecuador	2008	Pelagic Fish	10
58	Ecuador	2008	Pigmeat	41
58	Ecuador	2008	Pineapples	4
58	Ecuador	2008	Plantains	76
58	Ecuador	2008	Potatoes	43
58	Ecuador	2008	Poultry Meat	63
58	Ecuador	2008	Pulses, Other	5
58	Ecuador	2008	Rice (Milled Equivalent)	120
58	Ecuador	2008	Soyabean Oil	14
58	Ecuador	2008	Spices, Other	1
58	Ecuador	2008	Sugar, Refined Equivalent	43
58	Ecuador	2008	Sunflowerseed Oil	2
58	Ecuador	2008	Sweet Potatoes	1
58	Ecuador	2008	Sweeteners, Other	4
58	Ecuador	2008	Tomatoes	11
58	Ecuador	2008	Vegetables, Other	53
58	Ecuador	2008	Wheat	101
58	Ecuador	2008	Whey	1
58	Ecuador	2008	Wine	1
58	Ecuador	2009	Apples	10
58	Ecuador	2009	Bananas	250
58	Ecuador	2009	Barley	1
58	Ecuador	2009	Beans	1
58	Ecuador	2009	Beer	57
58	Ecuador	2009	Beverages, Alcoholic	3
58	Ecuador	2009	Bovine Meat	49
58	Ecuador	2009	Butter, Ghee	4
58	Ecuador	2009	Cassava	9
58	Ecuador	2009	Cheese	16
58	Ecuador	2009	Citrus, Other	5
58	Ecuador	2009	Cocoa Beans	1
58	Ecuador	2009	Coconuts - Incl Copra	1
58	Ecuador	2009	Crustaceans	9
58	Ecuador	2009	Demersal Fish	2
58	Ecuador	2009	Eggs	15
58	Ecuador	2009	Fats, Animals, Raw	11
58	Ecuador	2009	Freshwater Fish	2
58	Ecuador	2009	Fruits, Other	71
58	Ecuador	2009	Grapefruit	1
58	Ecuador	2009	Grapes	4
58	Ecuador	2009	Groundnuts (Shelled Eq)	2
58	Ecuador	2009	Maize	11
58	Ecuador	2009	Meat, Other	2
58	Ecuador	2009	Milk, Whole	399
58	Ecuador	2009	Mutton & Goat Meat	2
58	Ecuador	2009	Oats	6
58	Ecuador	2009	Offals, Edible	10
58	Ecuador	2009	Onions	6
58	Ecuador	2009	Oranges, Mandarines	14
58	Ecuador	2009	Palm Oil	25
58	Ecuador	2009	Palmkernel Oil	1
58	Ecuador	2009	Peas	1
58	Ecuador	2009	Pelagic Fish	8
58	Ecuador	2009	Pigmeat	34
58	Ecuador	2009	Pineapples	2
58	Ecuador	2009	Plantains	87
58	Ecuador	2009	Potatoes	44
58	Ecuador	2009	Poultry Meat	58
58	Ecuador	2009	Pulses, Other	5
58	Ecuador	2009	Rice (Milled Equivalent)	121
58	Ecuador	2009	Soyabean Oil	14
58	Ecuador	2009	Sugar, Refined Equivalent	44
58	Ecuador	2009	Sunflowerseed Oil	1
58	Ecuador	2009	Sweet Potatoes	1
58	Ecuador	2009	Sweeteners, Other	4
58	Ecuador	2009	Tomatoes	10

FAO country code	Country	Reference year	Food item	Gross food supply (g *capita*$^{-1}$ d^{-1})
58	Ecuador	2009	Vegetables, Other	55
58	Ecuador	2009	Wheat	101
58	Ecuador	2009	Wine	1
58	Ecuador	2010	Apples	11
58	Ecuador	2010	Bananas	270
58	Ecuador	2010	Barley	1
58	Ecuador	2010	Beans	1
58	Ecuador	2010	Beer	63
58	Ecuador	2010	Beverages, Alcoholic	3
58	Ecuador	2010	Bovine Meat	48
58	Ecuador	2010	Butter, Ghee	4
58	Ecuador	2010	Cassava	8
58	Ecuador	2010	Cheese	17
58	Ecuador	2010	Citrus, Other	5
58	Ecuador	2010	Cocoa Beans	1
58	Ecuador	2010	Coconuts - Incl Copra	1
58	Ecuador	2010	Crustaceans	9
58	Ecuador	2010	Demersal Fish	2
58	Ecuador	2010	Eggs	18
58	Ecuador	2010	Fats, Animals, Raw	11
58	Ecuador	2010	Freshwater Fish	2
58	Ecuador	2010	Fruits, Other	82
58	Ecuador	2010	Grapefruit	1
58	Ecuador	2010	Grapes	5
58	Ecuador	2010	Groundnuts (Shelled Eq)	2
58	Ecuador	2010	Lemons, Limes	1
58	Ecuador	2010	Maize	15
58	Ecuador	2010	Meat, Other	2
58	Ecuador	2010	Milk, Whole	402
58	Ecuador	2010	Mutton & Goat Meat	1
58	Ecuador	2010	Oats	5
58	Ecuador	2010	Offals, Edible	10
58	Ecuador	2010	Onions	7
58	Ecuador	2010	Oranges, Mandarines	15
58	Ecuador	2010	Palm Oil	25
58	Ecuador	2010	Palmkernel Oil	1
58	Ecuador	2010	Peas	1
58	Ecuador	2010	Pelagic Fish	8
58	Ecuador	2010	Pigmeat	35
58	Ecuador	2010	Pineapples	3
58	Ecuador	2010	Plantains	88
58	Ecuador	2010	Potatoes	60
58	Ecuador	2010	Poultry Meat	63
58	Ecuador	2010	Pulses, Other	5
58	Ecuador	2010	Rice (Milled Equivalent)	122
58	Ecuador	2010	Soyabean Oil	14
58	Ecuador	2010	Sugar, Refined Equivalent	44
58	Ecuador	2010	Sunflowerseed Oil	2
58	Ecuador	2010	Sweet Potatoes	1
58	Ecuador	2010	Sweeteners, Other	4
58	Ecuador	2010	Tomatoes	11
58	Ecuador	2010	Vegetables, Other	59
58	Ecuador	2010	Wheat	107
58	Ecuador	2010	Wine	1
58	Ecuador	2011	Apples	11
58	Ecuador	2011	Bananas	278
58	Ecuador	2011	Barley	1
58	Ecuador	2011	Beans	1
58	Ecuador	2011	Beer	60
58	Ecuador	2011	Beverages, Alcoholic	3
58	Ecuador	2011	Bovine Meat	48
58	Ecuador	2011	Butter, Ghee	4
58	Ecuador	2011	Cassava	8
58	Ecuador	2011	Cheese	18
58	Ecuador	2011	Citrus, Other	4
58	Ecuador	2011	Cocoa Beans	1
58	Ecuador	2011	Coconuts - Incl Copra	1
58	Ecuador	2011	Crustaceans	9
58	Ecuador	2011	Demersal Fish	2
58	Ecuador	2011	Eggs	20
58	Ecuador	2011	Fats, Animals, Raw	11
58	Ecuador	2011	Freshwater Fish	2
58	Ecuador	2011	Fruits, Other	68
58	Ecuador	2011	Grapefruit	1
58	Ecuador	2011	Grapes	6
58	Ecuador	2011	Groundnuts (Shelled Eq)	2
58	Ecuador	2011	Lemons, Limes	1
58	Ecuador	2011	Maize	16
58	Ecuador	2011	Meat, Other	2
58	Ecuador	2011	Milk, Whole	408
58	Ecuador	2011	Mutton & Goat Meat	1
58	Ecuador	2011	Oats	7
58	Ecuador	2011	Offals, Edible	10
58	Ecuador	2011	Onions	3
58	Ecuador	2011	Oranges, Mandarines	8
58	Ecuador	2011	Palm Oil	25
58	Ecuador	2011	Palmkernel Oil	2
58	Ecuador	2011	Peas	1
58	Ecuador	2011	Pelagic Fish	7
58	Ecuador	2011	Pigmeat	38
58	Ecuador	2011	Pineapples	4
58	Ecuador	2011	Plantains	90
58	Ecuador	2011	Potatoes	52
58	Ecuador	2011	Poultry Meat	60
58	Ecuador	2011	Pulses, Other	5
58	Ecuador	2011	Rice (Milled Equivalent)	122
58	Ecuador	2011	Soyabean Oil	14

FAO country code	Country	Reference year	Food item	Gross food supply (g *capita*$^{-1}$ d^{-1})
58	Ecuador	2011	Spices, Other	1
58	Ecuador	2011	Sugar, Refined Equivalent	45
58	Ecuador	2011	Sunflowerseed Oil	3
58	Ecuador	2011	Sweet Potatoes	1
58	Ecuador	2011	Sweeteners, Other	3
58	Ecuador	2011	Tomatoes	9
58	Ecuador	2011	Vegetables, Other	59
58	Ecuador	2011	Wheat	108
58	Ecuador	2011	Wine	1
59	Egypt	1992	Apples	11
59	Egypt	1992	Bananas	17
59	Egypt	1992	Barley	1
59	Egypt	1992	Beans	1
59	Egypt	1992	Beer	2
59	Egypt	1992	Bovine Meat	22
59	Egypt	1992	Butter, Ghee	6
59	Egypt	1992	Cereals, Other	1
59	Egypt	1992	Cheese	14
59	Egypt	1992	Coconuts - Incl Copra	1
59	Egypt	1992	Cottonseed Oil	9
59	Egypt	1992	Dates	26
59	Egypt	1992	Demersal Fish	2
59	Egypt	1992	Eggs	5
59	Egypt	1992	Fats, Animals, Raw	2
59	Egypt	1992	Freshwater Fish	11
59	Egypt	1992	Fruits, Other	40
59	Egypt	1992	Grapes	28
59	Egypt	1992	Groundnuts (Shelled Eq)	1
59	Egypt	1992	Lemons, Limes	13
59	Egypt	1992	Maize	156
59	Egypt	1992	Marine Fish, Other	8
59	Egypt	1992	Meat, Other	5
59	Egypt	1992	Milk, Whole	23
59	Egypt	1992	Mutton & Goat Meat	4
59	Egypt	1992	Offals, Edible	4
59	Egypt	1992	Olives	4
59	Egypt	1992	Onions	23
59	Egypt	1992	Oranges, Mandarines	84
59	Egypt	1992	Pelagic Fish	2
59	Egypt	1992	Pimento	2
59	Egypt	1992	Potatoes	52
59	Egypt	1992	Poultry Meat	14
59	Egypt	1992	Pulses, Other	20
59	Egypt	1992	Rice (Milled Equivalent)	94
59	Egypt	1992	Roots, Other	5
59	Egypt	1992	Sesameseed	2
59	Egypt	1992	Sorghum	12
59	Egypt	1992	Soyabean Oil	1
59	Egypt	1992	Soyabeans	1
59	Egypt	1992	Sugar Cane	69
59	Egypt	1992	Sugar, Refined Equivalent	76
59	Egypt	1992	Sunflowerseed Oil	8
59	Egypt	1992	Sweet Potatoes	3
59	Egypt	1992	Sweeteners, Other	2
59	Egypt	1992	Tea	4
59	Egypt	1992	Tomatoes	198
59	Egypt	1992	Vegetables, Other	167
59	Egypt	1992	Wheat	399
59	Egypt	1993	Apples	15
59	Egypt	1993	Bananas	17
59	Egypt	1993	Barley	1
59	Egypt	1993	Beans	1
59	Egypt	1993	Beer	2
59	Egypt	1993	Bovine Meat	22
59	Egypt	1993	Butter, Ghee	6
59	Egypt	1993	Cereals, Other	1
59	Egypt	1993	Cheese	14
59	Egypt	1993	Coconuts - Incl Copra	1
59	Egypt	1993	Cottonseed Oil	9
59	Egypt	1993	Dates	26
59	Egypt	1993	Demersal Fish	2
59	Egypt	1993	Eggs	5
59	Egypt	1993	Fats, Animals, Raw	1
59	Egypt	1993	Freshwater Fish	11
59	Egypt	1993	Fruits, Other	42
59	Egypt	1993	Grapes	30
59	Egypt	1993	Groundnuts (Shelled Eq)	1
59	Egypt	1993	Lemons, Limes	13
59	Egypt	1993	Maize	162
59	Egypt	1993	Marine Fish, Other	5
59	Egypt	1993	Meat, Other	4
59	Egypt	1993	Milk, Whole	25
59	Egypt	1993	Mutton & Goat Meat	4
59	Egypt	1993	Offals, Edible	5
59	Egypt	1993	Olives	5
59	Egypt	1993	Onions	24
59	Egypt	1993	Oranges, Mandarines	61
59	Egypt	1993	Palmkernel Oil	1
59	Egypt	1993	Pelagic Fish	3
59	Egypt	1993	Pimento	2
59	Egypt	1993	Potatoes	52
59	Egypt	1993	Poultry Meat	14
59	Egypt	1993	Pulses, Other	20
59	Egypt	1993	Rice (Milled Equivalent)	100
59	Egypt	1993	Roots, Other	5
59	Egypt	1993	Sesameseed	2
59	Egypt	1993	Sorghum	12

FAO country code	Country	Reference year	Food item	Gross food supply (g *capita*$^{-1}$ d^{-1})
59	Egypt	1993	Soyabean Oil	1
59	Egypt	1993	Soyabeans	1
59	Egypt	1993	Sugar Cane	69
59	Egypt	1993	Sugar, Refined Equivalent	71
59	Egypt	1993	Sunflowerseed Oil	7
59	Egypt	1993	Sweet Potatoes	5
59	Egypt	1993	Sweeteners, Other	2
59	Egypt	1993	Tea	3
59	Egypt	1993	Tomatoes	197
59	Egypt	1993	Vegetables, Other	162
59	Egypt	1993	Wheat	394
59	Egypt	1994	Apples	14
59	Egypt	1994	Bananas	19
59	Egypt	1994	Barley	1
59	Egypt	1994	Beans	1
59	Egypt	1994	Beer	2
59	Egypt	1994	Bovine Meat	26
59	Egypt	1994	Butter, Ghee	6
59	Egypt	1994	Cheese	16
59	Egypt	1994	Coconuts - Incl Copra	2
59	Egypt	1994	Cottonseed Oil	7
59	Egypt	1994	Dates	27
59	Egypt	1994	Demersal Fish	3
59	Egypt	1994	Eggs	6
59	Egypt	1994	Fats, Animals, Raw	1
59	Egypt	1994	Freshwater Fish	11
59	Egypt	1994	Fruits, Other	45
59	Egypt	1994	Grapes	29
59	Egypt	1994	Groundnuts (Shelled Eq)	2
59	Egypt	1994	Lemons, Limes	12
59	Egypt	1994	Maize	158
59	Egypt	1994	Marine Fish, Other	3
59	Egypt	1994	Meat, Other	4
59	Egypt	1994	Milk, Whole	28
59	Egypt	1994	Mutton & Goat Meat	4
59	Egypt	1994	Offals, Edible	5
59	Egypt	1994	Olives	6
59	Egypt	1994	Onions	14
59	Egypt	1994	Oranges, Mandarines	71
59	Egypt	1994	Palmkernel Oil	1
59	Egypt	1994	Pelagic Fish	7
59	Egypt	1994	Pimento	2
59	Egypt	1994	Potatoes	44
59	Egypt	1994	Poultry Meat	15
59	Egypt	1994	Pulses, Other	20
59	Egypt	1994	Rice (Milled Equivalent)	106
59	Egypt	1994	Roots, Other	5
59	Egypt	1994	Sesameseed	3
59	Egypt	1994	Sorghum	13
59	Egypt	1994	Soyabean Oil	2
59	Egypt	1994	Soyabeans	1
59	Egypt	1994	Sugar Cane	73
59	Egypt	1994	Sugar, Refined Equivalent	70
59	Egypt	1994	Sunflowerseed Oil	7
59	Egypt	1994	Sweet Potatoes	6
59	Egypt	1994	Sweeteners, Other	2
59	Egypt	1994	Tea	3
59	Egypt	1994	Tomatoes	204
59	Egypt	1994	Vegetables, Other	170
59	Egypt	1994	Wheat	414
59	Egypt	1995	Apples	18
59	Egypt	1995	Bananas	20
59	Egypt	1995	Barley	1
59	Egypt	1995	Beans	1
59	Egypt	1995	Beer	2
59	Egypt	1995	Bovine Meat	26
59	Egypt	1995	Butter, Ghee	6
59	Egypt	1995	Cheese	16
59	Egypt	1995	Coconuts - Incl Copra	1
59	Egypt	1995	Cottonseed Oil	7
59	Egypt	1995	Crustaceans	1
59	Egypt	1995	Dates	27
59	Egypt	1995	Demersal Fish	3
59	Egypt	1995	Eggs	6
59	Egypt	1995	Fats, Animals, Raw	1
59	Egypt	1995	Freshwater Fish	12
59	Egypt	1995	Fruits, Other	51
59	Egypt	1995	Grapes	30
59	Egypt	1995	Groundnuts (Shelled Eq)	2
59	Egypt	1995	Lemons, Limes	12
59	Egypt	1995	Maize	154
59	Egypt	1995	Maize Germ Oil	1
59	Egypt	1995	Marine Fish, Other	2
59	Egypt	1995	Meat, Other	4
59	Egypt	1995	Milk, Whole	30
59	Egypt	1995	Mutton & Goat Meat	4
59	Egypt	1995	Offals, Edible	5
59	Egypt	1995	Olives	9
59	Egypt	1995	Onions	10
59	Egypt	1995	Oranges, Mandarines	77
59	Egypt	1995	Palmkernel Oil	1
59	Egypt	1995	Pelagic Fish	6
59	Egypt	1995	Pimento	2
59	Egypt	1995	Potatoes	57
59	Egypt	1995	Poultry Meat	17
59	Egypt	1995	Pulses, Other	20
59	Egypt	1995	Rice (Milled Equivalent)	113

FAO country code	Country	Reference year	Food item	Gross food supply (g *capita*$^{-1}$ d^{-1})
59	Egypt	1995	Roots, Other	5
59	Egypt	1995	Sesameseed	3
59	Egypt	1995	Sorghum	12
59	Egypt	1995	Soyabean Oil	3
59	Egypt	1995	Sugar Cane	74
59	Egypt	1995	Sugar, Refined Equivalent	70
59	Egypt	1995	Sunflowerseed Oil	6
59	Egypt	1995	Sweet Potatoes	6
59	Egypt	1995	Sweeteners, Other	3
59	Egypt	1995	Tea	3
59	Egypt	1995	Tomatoes	203
59	Egypt	1995	Vegetables, Other	195
59	Egypt	1995	Wheat	412
59	Egypt	1995	Whey	1
59	Egypt	1996	Apples	17
59	Egypt	1996	Bananas	23
59	Egypt	1996	Barley	1
59	Egypt	1996	Beans	1
59	Egypt	1996	Beer	2
59	Egypt	1996	Bovine Meat	25
59	Egypt	1996	Butter, Ghee	6
59	Egypt	1996	Cheese	17
59	Egypt	1996	Coconuts - Incl Copra	2
59	Egypt	1996	Cottonseed Oil	5
59	Egypt	1996	Dates	29
59	Egypt	1996	Demersal Fish	3
59	Egypt	1996	Eggs	6
59	Egypt	1996	Fats, Animals, Raw	1
59	Egypt	1996	Freshwater Fish	13
59	Egypt	1996	Fruits, Other	50
59	Egypt	1996	Grapes	38
59	Egypt	1996	Groundnuts (Shelled Eq)	2
59	Egypt	1996	Lemons, Limes	12
59	Egypt	1996	Maize	151
59	Egypt	1996	Marine Fish, Other	1
59	Egypt	1996	Meat, Other	4
59	Egypt	1996	Milk, Whole	33
59	Egypt	1996	Mutton & Goat Meat	4
59	Egypt	1996	Offals, Edible	5
59	Egypt	1996	Olives	9
59	Egypt	1996	Onions	13
59	Egypt	1996	Oranges, Mandarines	79
59	Egypt	1996	Palmkernel Oil	1
59	Egypt	1996	Pelagic Fish	11
59	Egypt	1996	Pimento	2
59	Egypt	1996	Potatoes	59
59	Egypt	1996	Poultry Meat	17
59	Egypt	1996	Pulses, Other	20
59	Egypt	1996	Rice (Milled Equivalent)	113
59	Egypt	1996	Roots, Other	6
59	Egypt	1996	Sesameseed	3
59	Egypt	1996	Sorghum	12
59	Egypt	1996	Soyabean Oil	4
59	Egypt	1996	Sugar Cane	70
59	Egypt	1996	Sugar, Refined Equivalent	71
59	Egypt	1996	Sunflowerseed Oil	6
59	Egypt	1996	Sweet Potatoes	5
59	Egypt	1996	Sweeteners, Other	3
59	Egypt	1996	Tea	3
59	Egypt	1996	Tomatoes	238
59	Egypt	1996	Vegetables, Other	211
59	Egypt	1996	Wheat	414
59	Egypt	1996	Whey	1
59	Egypt	1997	Apples	17
59	Egypt	1997	Bananas	25
59	Egypt	1997	Barley	1
59	Egypt	1997	Beans	1
59	Egypt	1997	Beer	2
59	Egypt	1997	Bovine Meat	27
59	Egypt	1997	Butter, Ghee	6
59	Egypt	1997	Cheese	18
59	Egypt	1997	Coconuts - Incl Copra	2
59	Egypt	1997	Cottonseed Oil	4
59	Egypt	1997	Dates	29
59	Egypt	1997	Demersal Fish	3
59	Egypt	1997	Eggs	6
59	Egypt	1997	Fats, Animals, Raw	1
59	Egypt	1997	Freshwater Fish	13
59	Egypt	1997	Fruits, Other	52
59	Egypt	1997	Grapes	34
59	Egypt	1997	Groundnut Oil	1
59	Egypt	1997	Groundnuts (Shelled Eq)	3
59	Egypt	1997	Lemons, Limes	10
59	Egypt	1997	Maize	159
59	Egypt	1997	Marine Fish, Other	2
59	Egypt	1997	Meat, Other	4
59	Egypt	1997	Milk, Whole	35
59	Egypt	1997	Mutton & Goat Meat	4
59	Egypt	1997	Offals, Edible	5
59	Egypt	1997	Oilcrops, Other	1
59	Egypt	1997	Olives	8
59	Egypt	1997	Onions	11
59	Egypt	1997	Oranges, Mandarines	75
59	Egypt	1997	Palmkernel Oil	1
59	Egypt	1997	Pelagic Fish	9
59	Egypt	1997	Pimento	2
59	Egypt	1997	Potatoes	52

FAO country code	Country	Reference year	Food item	Gross food supply (g *capita*$^{-1}$ d^{-1})
59	Egypt	1997	Poultry Meat	22
59	Egypt	1997	Pulses, Other	20
59	Egypt	1997	Rice (Milled Equivalent)	112
59	Egypt	1997	Roots, Other	4
59	Egypt	1997	Sesameseed	3
59	Egypt	1997	Sorghum	12
59	Egypt	1997	Soyabean Oil	3
59	Egypt	1997	Spices, Other	1
59	Egypt	1997	Sugar Cane	91
59	Egypt	1997	Sugar, Refined Equivalent	74
59	Egypt	1997	Sunflowerseed Oil	6
59	Egypt	1997	Sweet Potatoes	7
59	Egypt	1997	Sweeteners, Other	3
59	Egypt	1997	Tea	3
59	Egypt	1997	Tomatoes	229
59	Egypt	1997	Vegetables, Other	240
59	Egypt	1997	Wheat	414
59	Egypt	1997	Whey	1
59	Egypt	1998	Apples	16
59	Egypt	1998	Bananas	26
59	Egypt	1998	Barley	1
59	Egypt	1998	Beans	1
59	Egypt	1998	Beer	2
59	Egypt	1998	Bovine Meat	29
59	Egypt	1998	Butter, Ghee	6
59	Egypt	1998	Cheese	19
59	Egypt	1998	Coconuts - Incl Copra	2
59	Egypt	1998	Cottonseed Oil	3
59	Egypt	1998	Dates	32
59	Egypt	1998	Demersal Fish	4
59	Egypt	1998	Eggs	6
59	Egypt	1998	Fats, Animals, Raw	1
59	Egypt	1998	Freshwater Fish	15
59	Egypt	1998	Fruits, Other	53
59	Egypt	1998	Grapes	37
59	Egypt	1998	Groundnut Oil	1
59	Egypt	1998	Groundnuts (Shelled Eq)	2
59	Egypt	1998	Lemons, Limes	9
59	Egypt	1998	Maize	165
59	Egypt	1998	Maize Germ Oil	1
59	Egypt	1998	Marine Fish, Other	2
59	Egypt	1998	Meat, Other	4
59	Egypt	1998	Milk, Whole	37
59	Egypt	1998	Mutton & Goat Meat	4
59	Egypt	1998	Nuts	1
59	Egypt	1998	Offals, Edible	5
59	Egypt	1998	Olives	8
59	Egypt	1998	Onions	21
59	Egypt	1998	Oranges, Mandarines	62
59	Egypt	1998	Palmkernel Oil	1
59	Egypt	1998	Pelagic Fish	12
59	Egypt	1998	Pimento	2
59	Egypt	1998	Potatoes	56
59	Egypt	1998	Poultry Meat	21
59	Egypt	1998	Pulses, Other	20
59	Egypt	1998	Rice (Milled Equivalent)	108
59	Egypt	1998	Roots, Other	3
59	Egypt	1998	Sesameseed	3
59	Egypt	1998	Sorghum	13
59	Egypt	1998	Soyabean Oil	4
59	Egypt	1998	Spices, Other	1
59	Egypt	1998	Sugar Cane	113
59	Egypt	1998	Sugar, Refined Equivalent	70
59	Egypt	1998	Sunflowerseed Oil	6
59	Egypt	1998	Sweet Potatoes	8
59	Egypt	1998	Sweeteners, Other	3
59	Egypt	1998	Tea	3
59	Egypt	1998	Tomatoes	221
59	Egypt	1998	Vegetables, Other	230
59	Egypt	1998	Wheat	401
59	Egypt	1998	Whey	1
59	Egypt	1999	Apples	17
59	Egypt	1999	Bananas	28
59	Egypt	1999	Barley	1
59	Egypt	1999	Beans	1
59	Egypt	1999	Beer	2
59	Egypt	1999	Bovine Meat	29
59	Egypt	1999	Butter, Ghee	6
59	Egypt	1999	Cheese	20
59	Egypt	1999	Cocoa Beans	1
59	Egypt	1999	Coconuts - Incl Copra	1
59	Egypt	1999	Cottonseed Oil	2
59	Egypt	1999	Crustaceans	1
59	Egypt	1999	Dates	34
59	Egypt	1999	Demersal Fish	5
59	Egypt	1999	Eggs	5
59	Egypt	1999	Fats, Animals, Raw	1
59	Egypt	1999	Freshwater Fish	17
59	Egypt	1999	Fruits, Other	49
59	Egypt	1999	Grapes	38
59	Egypt	1999	Groundnut Oil	1
59	Egypt	1999	Groundnuts (Shelled Eq)	3
59	Egypt	1999	Lemons, Limes	10
59	Egypt	1999	Maize	171
59	Egypt	1999	Marine Fish, Other	4
59	Egypt	1999	Meat, Other	5
59	Egypt	1999	Milk, Whole	37

FAO country code	Country	Reference year	Food item	Gross food supply (g $capita^{-1}$ d^{-1})
59	Egypt	1999	Mutton & Goat Meat	3
59	Egypt	1999	Nuts	1
59	Egypt	1999	Offals, Edible	6
59	Egypt	1999	Olives	12
59	Egypt	1999	Onions	29
59	Egypt	1999	Oranges, Mandarines	79
59	Egypt	1999	Palmkernel Oil	1
59	Egypt	1999	Pelagic Fish	11
59	Egypt	1999	Pimento	2
59	Egypt	1999	Potatoes	51
59	Egypt	1999	Poultry Meat	23
59	Egypt	1999	Pulses, Other	19
59	Egypt	1999	Rice (Milled Equivalent)	111
59	Egypt	1999	Roots, Other	2
59	Egypt	1999	Sesameseed	3
59	Egypt	1999	Sorghum	18
59	Egypt	1999	Soyabean Oil	4
59	Egypt	1999	Spices, Other	1
59	Egypt	1999	Sugar Cane	126
59	Egypt	1999	Sugar, Refined Equivalent	71
59	Egypt	1999	Sunflowerseed Oil	6
59	Egypt	1999	Sweet Potatoes	9
59	Egypt	1999	Sweeteners, Other	3
59	Egypt	1999	Tea	3
59	Egypt	1999	Tomatoes	237
59	Egypt	1999	Vegetables, Other	256
59	Egypt	1999	Wheat	377
59	Egypt	1999	Whey	2
59	Egypt	2000	Apples	19
59	Egypt	2000	Bananas	29
59	Egypt	2000	Barley	1
59	Egypt	2000	Beans	1
59	Egypt	2000	Beer	2
59	Egypt	2000	Bovine Meat	30
59	Egypt	2000	Butter, Ghee	7
59	Egypt	2000	Cheese	20
59	Egypt	2000	Cocoa Beans	1
59	Egypt	2000	Coconuts - Incl Copra	2
59	Egypt	2000	Cottonseed Oil	2
59	Egypt	2000	Crustaceans	1
59	Egypt	2000	Dates	37
59	Egypt	2000	Demersal Fish	8
59	Egypt	2000	Eggs	6
59	Egypt	2000	Fats, Animals, Raw	1
59	Egypt	2000	Freshwater Fish	19
59	Egypt	2000	Fruits, Other	48
59	Egypt	2000	Grapes	40
59	Egypt	2000	Groundnut Oil	1
59	Egypt	2000	Groundnuts (Shelled Eq)	3
59	Egypt	2000	Lemons, Limes	10
59	Egypt	2000	Maize	167
59	Egypt	2000	Marine Fish, Other	3
59	Egypt	2000	Meat, Other	5
59	Egypt	2000	Milk, Whole	38
59	Egypt	2000	Mutton & Goat Meat	3
59	Egypt	2000	Nuts	2
59	Egypt	2000	Offals, Edible	6
59	Egypt	2000	Olives	11
59	Egypt	2000	Onions	22
59	Egypt	2000	Oranges, Mandarines	74
59	Egypt	2000	Palmkernel Oil	1
59	Egypt	2000	Pelagic Fish	10
59	Egypt	2000	Pimento	2
59	Egypt	2000	Potatoes	53
59	Egypt	2000	Poultry Meat	24
59	Egypt	2000	Pulses, Other	21
59	Egypt	2000	Rice (Milled Equivalent)	111
59	Egypt	2000	Roots, Other	2
59	Egypt	2000	Sesameseed	4
59	Egypt	2000	Sorghum	16
59	Egypt	2000	Soyabean Oil	4
59	Egypt	2000	Sugar Cane	147
59	Egypt	2000	Sugar, Refined Equivalent	73
59	Egypt	2000	Sunflowerseed Oil	6
59	Egypt	2000	Sweet Potatoes	9
59	Egypt	2000	Sweeteners, Other	2
59	Egypt	2000	Tea	3
59	Egypt	2000	Tomatoes	253
59	Egypt	2000	Vegetables, Other	279
59	Egypt	2000	Wheat	373
59	Egypt	2000	Whey	2
59	Egypt	2001	Apples	19
59	Egypt	2001	Bananas	31
59	Egypt	2001	Barley	1
59	Egypt	2001	Beans	1
59	Egypt	2001	Beer	6
59	Egypt	2001	Bovine Meat	22
59	Egypt	2001	Butter, Ghee	6
59	Egypt	2001	Cheese	19
59	Egypt	2001	Coconuts - Incl Copra	1
59	Egypt	2001	Cottonseed Oil	2
59	Egypt	2001	Crustaceans	1
59	Egypt	2001	Dates	41
59	Egypt	2001	Demersal Fish	8
59	Egypt	2001	Eggs	7
59	Egypt	2001	Fats, Animals, Raw	1
59	Egypt	2001	Freshwater Fish	20

FAO countrycode	Country	Reference year	Food item	Gross food supply (g $capita^{-1}$ d^{-1})
59	Egypt	2001	Fruits, Other	47
59	Egypt	2001	Grapes	41
59	Egypt	2001	Groundnut Oil	1
59	Egypt	2001	Groundnuts (Shelled Eq)	3
59	Egypt	2001	Lemons, Limes	10
59	Egypt	2001	Maize	162
59	Egypt	2001	Marine Fish, Other	3
59	Egypt	2001	Meat, Other	5
59	Egypt	2001	Milk, Whole	45
59	Egypt	2001	Mutton & Goat Meat	3
59	Egypt	2001	Nuts	2
59	Egypt	2001	Offals, Edible	4
59	Egypt	2001	Oilcrops, Other	1
59	Egypt	2001	Olives	12
59	Egypt	2001	Onions	16
59	Egypt	2001	Oranges, Mandarines	72
59	Egypt	2001	Palmkernel Oil	1
59	Egypt	2001	Pelagic Fish	10
59	Egypt	2001	Pimento	2
59	Egypt	2001	Potatoes	54
59	Egypt	2001	Poultry Meat	25
59	Egypt	2001	Pulses, Other	25
59	Egypt	2001	Rice (Milled Equivalent)	109
59	Egypt	2001	Roots, Other	2
59	Egypt	2001	Sesameseed	3
59	Egypt	2001	Sorghum	12
59	Egypt	2001	Soyabean Oil	4
59	Egypt	2001	Spices, Other	1
59	Egypt	2001	Sugar Cane	145
59	Egypt	2001	Sugar, Refined Equivalent	78
59	Egypt	2001	Sunflowerseed Oil	5
59	Egypt	2001	Sweet Potatoes	11
59	Egypt	2001	Sweeteners, Other	3
59	Egypt	2001	Tea	3
59	Egypt	2001	Tomatoes	231
59	Egypt	2001	Vegetables, Other	256
59	Egypt	2001	Wheat	395
59	Egypt	2001	Whey	3
59	Egypt	2002	Apples	20
59	Egypt	2002	Bananas	32
59	Egypt	2002	Barley	1
59	Egypt	2002	Beans	1
59	Egypt	2002	Beer	5
59	Egypt	2002	Bovine Meat	24
59	Egypt	2002	Butter, Ghee	6
59	Egypt	2002	Cheese	22
59	Egypt	2002	Coconuts - Incl Copra	2
59	Egypt	2002	Cottonseed Oil	3
59	Egypt	2002	Crustaceans	1
59	Egypt	2002	Dates	39
59	Egypt	2002	Demersal Fish	8
59	Egypt	2002	Eggs	10
59	Egypt	2002	Fats, Animals, Raw	1
59	Egypt	2002	Freshwater Fish	21
59	Egypt	2002	Fruits, Other	51
59	Egypt	2002	Grapes	39
59	Egypt	2002	Groundnut Oil	1
59	Egypt	2002	Groundnuts (Shelled Eq)	3
59	Egypt	2002	Lemons, Limes	11
59	Egypt	2002	Maize	167
59	Egypt	2002	Maize Germ Oil	1
59	Egypt	2002	Marine Fish, Other	2
59	Egypt	2002	Meat, Other	5
59	Egypt	2002	Milk, Whole	41
59	Egypt	2002	Mutton & Goat Meat	3
59	Egypt	2002	Nuts	2
59	Egypt	2002	Offals, Edible	5
59	Egypt	2002	Oilcrops, Other	1
59	Egypt	2002	Olive Oil	1
59	Egypt	2002	Olives	13
59	Egypt	2002	Onions	15
59	Egypt	2002	Oranges, Mandarines	82
59	Egypt	2002	Palmkernel Oil	1
59	Egypt	2002	Pelagic Fish	8
59	Egypt	2002	Pimento	2
59	Egypt	2002	Potatoes	55
59	Egypt	2002	Poultry Meat	25
59	Egypt	2002	Pulses, Other	24
59	Egypt	2002	Rice (Milled Equivalent)	123
59	Egypt	2002	Roots, Other	2
59	Egypt	2002	Sesameseed	3
59	Egypt	2002	Sorghum	13
59	Egypt	2002	Soyabean Oil	5
59	Egypt	2002	Spices, Other	1
59	Egypt	2002	Sugar Cane	150
59	Egypt	2002	Sugar, Refined Equivalent	74
59	Egypt	2002	Sunflowerseed Oil	5
59	Egypt	2002	Sweet Potatoes	8
59	Egypt	2002	Sweeteners, Other	3
59	Egypt	2002	Tea	3
59	Egypt	2002	Tomatoes	243
59	Egypt	2002	Vegetables, Other	253
59	Egypt	2002	Wheat	377
59	Egypt	2002	Whey	3
59	Egypt	2003	Apples	20
59	Egypt	2003	Bananas	31
59	Egypt	2003	Barley	1

FAO country code	Country	Reference year	Food item	Gross food supply (g *capita*$^{-1}$ d^{-1})
59	Egypt	2003	Beans	2
59	Egypt	2003	Beer	6
59	Egypt	2003	Bovine Meat	25
59	Egypt	2003	Butter, Ghee	6
59	Egypt	2003	Cheese	26
59	Egypt	2003	Coconuts - Incl Copra	2
59	Egypt	2003	Cottonseed Oil	2
59	Egypt	2003	Dates	40
59	Egypt	2003	Demersal Fish	9
59	Egypt	2003	Eggs	9
59	Egypt	2003	Fats, Animals, Raw	1
59	Egypt	2003	Freshwater Fish	23
59	Egypt	2003	Fruits, Other	44
59	Egypt	2003	Grapes	42
59	Egypt	2003	Groundnut Oil	1
59	Egypt	2003	Groundnuts (Shelled Eq)	3
59	Egypt	2003	Lemons, Limes	11
59	Egypt	2003	Maize	170
59	Egypt	2003	Marine Fish, Other	3
59	Egypt	2003	Meat, Other	5
59	Egypt	2003	Milk, Whole	50
59	Egypt	2003	Mutton & Goat Meat	3
59	Egypt	2003	Nuts	2
59	Egypt	2003	Offals, Edible	5
59	Egypt	2003	Oilcrops, Other	1
59	Egypt	2003	Olives	8
59	Egypt	2003	Onions	12
59	Egypt	2003	Oranges, Mandarines	78
59	Egypt	2003	Palmkernel Oil	1
59	Egypt	2003	Pelagic Fish	7
59	Egypt	2003	Pimento	2
59	Egypt	2003	Potatoes	53
59	Egypt	2003	Poultry Meat	25
59	Egypt	2003	Pulses, Other	22
59	Egypt	2003	Rice (Milled Equivalent)	119
59	Egypt	2003	Roots, Other	4
59	Egypt	2003	Sesameseed	3
59	Egypt	2003	Sorghum	12
59	Egypt	2003	Soyabean Oil	5
59	Egypt	2003	Spices, Other	1
59	Egypt	2003	Sugar Cane	136
59	Egypt	2003	Sugar, Refined Equivalent	67
59	Egypt	2003	Sunflowerseed Oil	5
59	Egypt	2003	Sweet Potatoes	10
59	Egypt	2003	Sweeteners, Other	3
59	Egypt	2003	Tea	3
59	Egypt	2003	Tomatoes	252
59	Egypt	2003	Vegetables, Other	278
59	Egypt	2003	Wheat	364
59	Egypt	2003	Whey	2
59	Egypt	2004	Apples	21
59	Egypt	2004	Bananas	31
59	Egypt	2004	Barley	1
59	Egypt	2004	Beans	1
59	Egypt	2004	Beer	10
59	Egypt	2004	Bovine Meat	28
59	Egypt	2004	Butter, Ghee	6
59	Egypt	2004	Cheese	24
59	Egypt	2004	Cocoa Beans	1
59	Egypt	2004	Coconuts - Incl Copra	2
59	Egypt	2004	Cottonseed Oil	2
59	Egypt	2004	Crustaceans	1
59	Egypt	2004	Dates	41
59	Egypt	2004	Demersal Fish	8
59	Egypt	2004	Eggs	8
59	Egypt	2004	Fats, Animals, Raw	1
59	Egypt	2004	Freshwater Fish	22
59	Egypt	2004	Fruits, Other	50
59	Egypt	2004	Grapes	44
59	Egypt	2004	Groundnut Oil	1
59	Egypt	2004	Groundnuts (Shelled Eq)	2
59	Egypt	2004	Lemons, Limes	10
59	Egypt	2004	Maize	163
59	Egypt	2004	Marine Fish, Other	4
59	Egypt	2004	Meat, Other	5
59	Egypt	2004	Milk, Whole	27
59	Egypt	2004	Mutton & Goat Meat	2
59	Egypt	2004	Nuts	1
59	Egypt	2004	Offals, Edible	4
59	Egypt	2004	Oilcrops, Other	1
59	Egypt	2004	Olives	12
59	Egypt	2004	Onions	18
59	Egypt	2004	Oranges, Mandarines	78
59	Egypt	2004	Palmkernel Oil	1
59	Egypt	2004	Pelagic Fish	8
59	Egypt	2004	Pimento	2
59	Egypt	2004	Potatoes	65
59	Egypt	2004	Poultry Meat	25
59	Egypt	2004	Pulses, Other	22
59	Egypt	2004	Rice (Milled Equivalent)	113
59	Egypt	2004	Roots, Other	4
59	Egypt	2004	Sesameseed	3
59	Egypt	2004	Sorghum	9
59	Egypt	2004	Soyabean Oil	4
59	Egypt	2004	Spices, Other	1
59	Egypt	2004	Sugar Cane	132
59	Egypt	2004	Sugar, Refined Equivalent	68

FAO country code	Country	Reference year	Food item	Gross food supply (g *capita*$^{-1}$ d^{-1})
59	Egypt	2004	Sunflowerseed Oil	5
59	Egypt	2004	Sweet Potatoes	8
59	Egypt	2004	Sweeteners, Other	2
59	Egypt	2004	Tomatoes	266
59	Egypt	2004	Vegetables, Other	261
59	Egypt	2004	Wheat	371
59	Egypt	2004	Whey	4
59	Egypt	2005	Apples	22
59	Egypt	2005	Bananas	31
59	Egypt	2005	Barley	1
59	Egypt	2005	Beans	1
59	Egypt	2005	Beer	10
59	Egypt	2005	Bovine Meat	31
59	Egypt	2005	Butter, Ghee	6
59	Egypt	2005	Cheese	23
59	Egypt	2005	Coconuts - Incl Copra	3
59	Egypt	2005	Cottonseed Oil	2
59	Egypt	2005	Crustaceans	1
59	Egypt	2005	Dates	40
59	Egypt	2005	Demersal Fish	9
59	Egypt	2005	Eggs	7
59	Egypt	2005	Fats, Animals, Raw	1
59	Egypt	2005	Freshwater Fish	22
59	Egypt	2005	Fruits, Other	46
59	Egypt	2005	Grapes	47
59	Egypt	2005	Groundnut Oil	1
59	Egypt	2005	Groundnuts (Shelled Eq)	2
59	Egypt	2005	Lemons, Limes	11
59	Egypt	2005	Maize	161
59	Egypt	2005	Maize Germ Oil	1
59	Egypt	2005	Marine Fish, Other	5
59	Egypt	2005	Meat, Other	5
59	Egypt	2005	Milk, Whole	39
59	Egypt	2005	Mutton & Goat Meat	3
59	Egypt	2005	Nuts	2
59	Egypt	2005	Offals, Edible	5
59	Egypt	2005	Olives	11
59	Egypt	2005	Onions	33
59	Egypt	2005	Oranges, Mandarines	83
59	Egypt	2005	Palmkernel Oil	2
59	Egypt	2005	Pelagic Fish	7
59	Egypt	2005	Pimento	2
59	Egypt	2005	Potatoes	86
59	Egypt	2005	Poultry Meat	25
59	Egypt	2005	Pulses, Other	24
59	Egypt	2005	Rice (Milled Equivalent)	101
59	Egypt	2005	Roots, Other	3
59	Egypt	2005	Sesameseed	2
59	Egypt	2005	Sorghum	8
59	Egypt	2005	Soyabean Oil	4
59	Egypt	2005	Spices, Other	1
59	Egypt	2005	Sugar Cane	122
59	Egypt	2005	Sugar, Refined Equivalent	66
59	Egypt	2005	Sunflowerseed Oil	4
59	Egypt	2005	Sweet Potatoes	11
59	Egypt	2005	Sweeteners, Other	3
59	Egypt	2005	Tomatoes	260
59	Egypt	2005	Vegetables, Other	267
59	Egypt	2005	Wheat	396
59	Egypt	2005	Whey	4
59	Egypt	2006	Apples	21
59	Egypt	2006	Bananas	29
59	Egypt	2006	Barley	1
59	Egypt	2006	Beans	1
59	Egypt	2006	Beer	7
59	Egypt	2006	Bovine Meat	36
59	Egypt	2006	Butter, Ghee	5
59	Egypt	2006	Cheese	23
59	Egypt	2006	Coconuts - Incl Copra	1
59	Egypt	2006	Cottonseed Oil	2
59	Egypt	2006	Crustaceans	1
59	Egypt	2006	Dates	45
59	Egypt	2006	Demersal Fish	12
59	Egypt	2006	Eggs	7
59	Egypt	2006	Fats, Animals, Raw	1
59	Egypt	2006	Freshwater Fish	21
59	Egypt	2006	Fruits, Other	62
59	Egypt	2006	Grapes	47
59	Egypt	2006	Groundnut Oil	1
59	Egypt	2006	Groundnuts (Shelled Eq)	2
59	Egypt	2006	Lemons, Limes	10
59	Egypt	2006	Maize	162
59	Egypt	2006	Maize Germ Oil	1
59	Egypt	2006	Marine Fish, Other	6
59	Egypt	2006	Meat, Other	5
59	Egypt	2006	Milk, Whole	51
59	Egypt	2006	Mutton & Goat Meat	4
59	Egypt	2006	Nuts	2
59	Egypt	2006	Offals, Edible	6
59	Egypt	2006	Olives	18
59	Egypt	2006	Onions	30
59	Egypt	2006	Oranges, Mandarines	86
59	Egypt	2006	Palmkernel Oil	1
59	Egypt	2006	Pelagic Fish	7
59	Egypt	2006	Pimento	2
59	Egypt	2006	Potatoes	57
59	Egypt	2006	Poultry Meat	27

FAO countrycode	Country	Reference year	Food item	Gross food supply (g *capita*$^{-1}$ d^{-1})
59	Egypt	2006	Pulses, Other	23
59	Egypt	2006	Rice (Milled Equivalent)	107
59	Egypt	2006	Roots, Other	4
59	Egypt	2006	Sesameseed	2
59	Egypt	2006	Sorghum	9
59	Egypt	2006	Soyabean Oil	4
59	Egypt	2006	Soyabeans	1
59	Egypt	2006	Spices, Other	1
59	Egypt	2006	Sugar Cane	120
59	Egypt	2006	Sugar, Refined Equivalent	63
59	Egypt	2006	Sunflowerseed Oil	4
59	Egypt	2006	Sweet Potatoes	11
59	Egypt	2006	Sweeteners, Other	3
59	Egypt	2006	Tomatoes	287
59	Egypt	2006	Vegetables, Other	284
59	Egypt	2006	Wheat	391
59	Egypt	2006	Whey	5
59	Egypt	2007	Apples	20
59	Egypt	2007	Bananas	31
59	Egypt	2007	Barley	1
59	Egypt	2007	Beer	10
59	Egypt	2007	Bovine Meat	39
59	Egypt	2007	Butter, Ghee	6
59	Egypt	2007	Cheese	23
59	Egypt	2007	Coconuts - Incl Copra	1
59	Egypt	2007	Cottonseed Oil	2
59	Egypt	2007	Crustaceans	1
59	Egypt	2007	Dates	44
59	Egypt	2007	Demersal Fish	13
59	Egypt	2007	Eggs	8
59	Egypt	2007	Fats, Animals, Raw	1
59	Egypt	2007	Freshwater Fish	21
59	Egypt	2007	Fruits, Other	59
59	Egypt	2007	Grapes	47
59	Egypt	2007	Groundnut Oil	1
59	Egypt	2007	Groundnuts (Shelled Eq)	3
59	Egypt	2007	Lemons, Limes	10
59	Egypt	2007	Maize	163
59	Egypt	2007	Maize Germ Oil	1
59	Egypt	2007	Marine Fish, Other	5
59	Egypt	2007	Meat, Other	4
59	Egypt	2007	Milk, Whole	69
59	Egypt	2007	Mutton & Goat Meat	5
59	Egypt	2007	Nuts	2
59	Egypt	2007	Offals, Edible	6
59	Egypt	2007	Olives	18
59	Egypt	2007	Onions	42
59	Egypt	2007	Oranges, Mandarines	83
59	Egypt	2007	Pelagic Fish	9
59	Egypt	2007	Pimento	2
59	Egypt	2007	Potatoes	71
59	Egypt	2007	Poultry Meat	30
59	Egypt	2007	Pulses, Other	19
59	Egypt	2007	Rice (Milled Equivalent)	109
59	Egypt	2007	Roots, Other	4
59	Egypt	2007	Sesameseed	2
59	Egypt	2007	Sorghum	8
59	Egypt	2007	Soyabean Oil	6
59	Egypt	2007	Soyabeans	2
59	Egypt	2007	Spices, Other	1
59	Egypt	2007	Sugar Cane	124
59	Egypt	2007	Sugar, Refined Equivalent	64
59	Egypt	2007	Sunflowerseed Oil	5
59	Egypt	2007	Sweet Potatoes	11
59	Egypt	2007	Sweeteners, Other	3
59	Egypt	2007	Tea	1
59	Egypt	2007	Tomatoes	286
59	Egypt	2007	Vegetables, Other	288
59	Egypt	2007	Wheat	389
59	Egypt	2007	Whey	6
59	Egypt	2008	Apples	20
59	Egypt	2008	Bananas	35
59	Egypt	2008	Barley	1
59	Egypt	2008	Beans	1
59	Egypt	2008	Beer	7
59	Egypt	2008	Bovine Meat	33
59	Egypt	2008	Butter, Ghee	6
59	Egypt	2008	Cheese	23
59	Egypt	2008	Cocoa Beans	1
59	Egypt	2008	Coconuts - Incl Copra	1
59	Egypt	2008	Coffee	1
59	Egypt	2008	Cottonseed Oil	1
59	Egypt	2008	Crustaceans	1
59	Egypt	2008	Dates	43
59	Egypt	2008	Demersal Fish	13
59	Egypt	2008	Eggs	10
59	Egypt	2008	Fats, Animals, Raw	1
59	Egypt	2008	Freshwater Fish	24
59	Egypt	2008	Fruits, Other	61
59	Egypt	2008	Grapes	48
59	Egypt	2008	Groundnut Oil	1
59	Egypt	2008	Groundnuts (Shelled Eq)	3
59	Egypt	2008	Lemons, Limes	11
59	Egypt	2008	Maize	164
59	Egypt	2008	Maize Germ Oil	1
59	Egypt	2008	Marine Fish, Other	3
59	Egypt	2008	Meat, Other	4

FAO country code	Country	Reference year	Food item	Gross food supply (g *capita*$^{-1}$ d^{-1})
59	Egypt	2008	Milk, Whole	69
59	Egypt	2008	Mutton & Goat Meat	6
59	Egypt	2008	Nuts	2
59	Egypt	2008	Offals, Edible	6
59	Egypt	2008	Oilcrops Oil, Other	1
59	Egypt	2008	Olives	16
59	Egypt	2008	Onions	60
59	Egypt	2008	Oranges, Mandarines	78
59	Egypt	2008	Palmkernel Oil	1
59	Egypt	2008	Pelagic Fish	8
59	Egypt	2008	Pimento	2
59	Egypt	2008	Potatoes	89
59	Egypt	2008	Poultry Meat	27
59	Egypt	2008	Pulses, Other	14
59	Egypt	2008	Rice (Milled Equivalent)	111
59	Egypt	2008	Roots, Other	4
59	Egypt	2008	Sesameseed	2
59	Egypt	2008	Sorghum	9
59	Egypt	2008	Soyabean Oil	7
59	Egypt	2008	Soyabeans	2
59	Egypt	2008	Spices, Other	1
59	Egypt	2008	Sugar Cane	118
59	Egypt	2008	Sugar, Refined Equivalent	65
59	Egypt	2008	Sunflowerseed Oil	4
59	Egypt	2008	Sweet Potatoes	8
59	Egypt	2008	Sweeteners, Other	4
59	Egypt	2008	Tea	4
59	Egypt	2008	Tomatoes	300
59	Egypt	2008	Vegetables, Other	273
59	Egypt	2008	Wheat	399
59	Egypt	2008	Whey	5
59	Egypt	2008	Wine	1
59	Egypt	2009	Apples	18
59	Egypt	2009	Bananas	35
59	Egypt	2009	Barley	1
59	Egypt	2009	Beans	1
59	Egypt	2009	Beer	7
59	Egypt	2009	Bovine Meat	35
59	Egypt	2009	Butter, Ghee	6
59	Egypt	2009	Cheese	19
59	Egypt	2009	Cocoa Beans	1
59	Egypt	2009	Coffee	1
59	Egypt	2009	Cottonseed Oil	1
59	Egypt	2009	Crustaceans	2
59	Egypt	2009	Dates	40
59	Egypt	2009	Demersal Fish	12
59	Egypt	2009	Eggs	7
59	Egypt	2009	Fats, Animals, Raw	1
59	Egypt	2009	Freshwater Fish	25
59	Egypt	2009	Fruits, Other	58
59	Egypt	2009	Grapes	39
59	Egypt	2009	Groundnut Oil	1
59	Egypt	2009	Groundnuts (Shelled Eq)	2
59	Egypt	2009	Lemons, Limes	25
59	Egypt	2009	Maize	172
59	Egypt	2009	Maize Germ Oil	1
59	Egypt	2009	Marine Fish, Other	4
59	Egypt	2009	Meat, Other	4
59	Egypt	2009	Milk, Whole	55
59	Egypt	2009	Mutton & Goat Meat	5
59	Egypt	2009	Nuts	2
59	Egypt	2009	Offals, Edible	4
59	Egypt	2009	Oilcrops Oil, Other	1
59	Egypt	2009	Olives	13
59	Egypt	2009	Onions	60
59	Egypt	2009	Oranges, Mandarines	73
59	Egypt	2009	Palmkernel Oil	1
59	Egypt	2009	Pelagic Fish	7
59	Egypt	2009	Pimento	1
59	Egypt	2009	Potatoes	94
59	Egypt	2009	Poultry Meat	29
59	Egypt	2009	Pulses, Other	15
59	Egypt	2009	Rice (Milled Equivalent)	107
59	Egypt	2009	Roots, Other	3
59	Egypt	2009	Sesameseed	2
59	Egypt	2009	Sorghum	8
59	Egypt	2009	Soyabean Oil	7
59	Egypt	2009	Soyabeans	3
59	Egypt	2009	Sugar Cane	109
59	Egypt	2009	Sugar, Refined Equivalent	60
59	Egypt	2009	Sunflowerseed Oil	4
59	Egypt	2009	Sweet Potatoes	11
59	Egypt	2009	Sweeteners, Other	3
59	Egypt	2009	Tea	3
59	Egypt	2009	Tomatoes	330
59	Egypt	2009	Vegetables, Other	282
59	Egypt	2009	Wheat	398
59	Egypt	2009	Whey	2
59	Egypt	2010	Apples	19
59	Egypt	2010	Bananas	33
59	Egypt	2010	Beans	1
59	Egypt	2010	Beer	7
59	Egypt	2010	Bovine Meat	37
59	Egypt	2010	Butter, Ghee	6
59	Egypt	2010	Cheese	22
59	Egypt	2010	Cocoa Beans	1
59	Egypt	2010	Coconuts - Incl Copra	1

FAO country code	Country	Reference year	Food item	Gross food supply (g *capita*$^{-1}$ d^{-1})
59	Egypt	2010	Coffee	1
59	Egypt	2010	Cottonseed Oil	1
59	Egypt	2010	Crustaceans	2
59	Egypt	2010	Dates	42
59	Egypt	2010	Demersal Fish	10
59	Egypt	2010	Eggs	8
59	Egypt	2010	Fats, Animals, Raw	1
59	Egypt	2010	Freshwater Fish	34
59	Egypt	2010	Fruits, Other	56
59	Egypt	2010	Grapes	42
59	Egypt	2010	Groundnut Oil	1
59	Egypt	2010	Groundnuts (Shelled Eq)	3
59	Egypt	2010	Lemons, Limes	9
59	Egypt	2010	Maize	171
59	Egypt	2010	Maize Germ Oil	1
59	Egypt	2010	Marine Fish, Other	2
59	Egypt	2010	Meat, Other	5
59	Egypt	2010	Milk, Whole	63
59	Egypt	2010	Mutton & Goat Meat	5
59	Egypt	2010	Nuts	3
59	Egypt	2010	Offals, Edible	6
59	Egypt	2010	Olives	13
59	Egypt	2010	Onions	55
59	Egypt	2010	Oranges, Mandarines	78
59	Egypt	2010	Pelagic Fish	12
59	Egypt	2010	Pimento	2
59	Egypt	2010	Potatoes	92
59	Egypt	2010	Poultry Meat	34
59	Egypt	2010	Pulses, Other	22
59	Egypt	2010	Rice (Milled Equivalent)	100
59	Egypt	2010	Roots, Other	3
59	Egypt	2010	Sesameseed	2
59	Egypt	2010	Sorghum	7
59	Egypt	2010	Soyabean Oil	7
59	Egypt	2010	Soyabeans	3
59	Egypt	2010	Sugar Cane	109
59	Egypt	2010	Sugar, Refined Equivalent	71
59	Egypt	2010	Sunflowerseed Oil	5
59	Egypt	2010	Sweet Potatoes	11
59	Egypt	2010	Sweeteners, Other	4
59	Egypt	2010	Tea	3
59	Egypt	2010	Tomatoes	270
59	Egypt	2010	Vegetables, Other	269
59	Egypt	2010	Wheat	399
59	Egypt	2010	Whey	7
59	Egypt	2011	Apples	19
59	Egypt	2011	Bananas	33
59	Egypt	2011	Barley	1
59	Egypt	2011	Beans	2
59	Egypt	2011	Beer	4
59	Egypt	2011	Bovine Meat	36
59	Egypt	2011	Butter, Ghee	8
59	Egypt	2011	Cheese	18
59	Egypt	2011	Cocoa Beans	1
59	Egypt	2011	Coffee	1
59	Egypt	2011	Cottonseed Oil	1
59	Egypt	2011	Crustaceans	2
59	Egypt	2011	Dates	42
59	Egypt	2011	Demersal Fish	12
59	Egypt	2011	Eggs	8
59	Egypt	2011	Fats, Animals, Raw	1
59	Egypt	2011	Freshwater Fish	36
59	Egypt	2011	Fruits, Other	50
59	Egypt	2011	Grapes	39
59	Egypt	2011	Groundnut Oil	1
59	Egypt	2011	Groundnuts (Shelled Eq)	3
59	Egypt	2011	Lemons, Limes	9
59	Egypt	2011	Maize	173
59	Egypt	2011	Maize Germ Oil	1
59	Egypt	2011	Marine Fish, Other	2
59	Egypt	2011	Meat, Other	4
59	Egypt	2011	Milk, Whole	73
59	Egypt	2011	Mutton & Goat Meat	4
59	Egypt	2011	Nuts	2
59	Egypt	2011	Offals, Edible	5
59	Egypt	2011	Oilcrops Oil, Other	1
59	Egypt	2011	Olives	13
59	Egypt	2011	Onions	55
59	Egypt	2011	Oranges, Mandarines	70
59	Egypt	2011	Pelagic Fish	8
59	Egypt	2011	Pimento	2
59	Egypt	2011	Potatoes	95
59	Egypt	2011	Poultry Meat	32
59	Egypt	2011	Pulses, Other	14
59	Egypt	2011	Rice (Milled Equivalent)	108
59	Egypt	2011	Roots, Other	3
59	Egypt	2011	Sesameseed	2
59	Egypt	2011	Sorghum	9
59	Egypt	2011	Soyabean Oil	8
59	Egypt	2011	Soyabeans	3
59	Egypt	2011	Spices, Other	1
59	Egypt	2011	Sugar Cane	107
59	Egypt	2011	Sugar, Refined Equivalent	72
59	Egypt	2011	Sunflowerseed Oil	5
59	Egypt	2011	Sweet Potatoes	8
59	Egypt	2011	Sweeteners, Other	4
59	Egypt	2011	Tea	3

FAO country code	Country	Reference year	Food item	Gross food supply (g *capita*$^{-1}$ d^{-1})
59	Egypt	2011	Tomatoes	250
59	Egypt	2011	Vegetables, Other	260
59	Egypt	2011	Wheat	400
59	Egypt	2011	Whey	6
60	El Salvador	1992	Apples	3
60	El Salvador	1992	Bananas	13
60	El Salvador	1992	Beans	30
60	El Salvador	1992	Beer	40
60	El Salvador	1992	Beverages, Alcoholic	4
60	El Salvador	1992	Bovine Meat	12
60	El Salvador	1992	Cassava	20
60	El Salvador	1992	Cereals, Other	1
60	El Salvador	1992	Cheese	2
60	El Salvador	1992	Cocoa Beans	1
60	El Salvador	1992	Coconut Oil	1
60	El Salvador	1992	Coconuts - Incl Copra	21
60	El Salvador	1992	Coffee	4
60	El Salvador	1992	Cottonseed Oil	9
60	El Salvador	1992	Demersal Fish	1
60	El Salvador	1992	Eggs	21
60	El Salvador	1992	Fats, Animals, Raw	8
60	El Salvador	1992	Freshwater Fish	3
60	El Salvador	1992	Fruits, Other	42
60	El Salvador	1992	Grapes	1
60	El Salvador	1992	Honey	1
60	El Salvador	1992	Lemons, Limes	12
60	El Salvador	1992	Maize	236
60	El Salvador	1992	Marine Fish, Other	3
60	El Salvador	1992	Milk, Whole	197
60	El Salvador	1992	Nuts	1
60	El Salvador	1992	Offals, Edible	2
60	El Salvador	1992	Oilcrops Oil, Other	1
60	El Salvador	1992	Onions	4
60	El Salvador	1992	Oranges, Mandarines	62
60	El Salvador	1992	Pigmeat	6
60	El Salvador	1992	Pineapples	7
60	El Salvador	1992	Plantains	38
60	El Salvador	1992	Potatoes	12
60	El Salvador	1992	Poultry Meat	20
60	El Salvador	1992	Rice (Milled Equivalent)	23
60	El Salvador	1992	Sesameseed	1
60	El Salvador	1992	Sorghum	61
60	El Salvador	1992	Soyabean Oil	1
60	El Salvador	1992	Soyabeans	1
60	El Salvador	1992	Sugar, Non-Centrifugal	7
60	El Salvador	1992	Sugar, Refined Equivalent	85
60	El Salvador	1992	Sweeteners, Other	1
60	El Salvador	1992	Tomatoes	13
60	El Salvador	1992	Vegetables, Other	64
60	El Salvador	1992	Wheat	91
60	El Salvador	1993	Apples	2
60	El Salvador	1993	Bananas	11
60	El Salvador	1993	Beans	29
60	El Salvador	1993	Beer	44
60	El Salvador	1993	Beverages, Alcoholic	4
60	El Salvador	1993	Bovine Meat	14
60	El Salvador	1993	Cassava	19
60	El Salvador	1993	Cheese	2
60	El Salvador	1993	Cocoa Beans	1
60	El Salvador	1993	Coconut Oil	1
60	El Salvador	1993	Coconuts - Incl Copra	20
60	El Salvador	1993	Coffee	4
60	El Salvador	1993	Cottonseed Oil	11
60	El Salvador	1993	Crustaceans	1
60	El Salvador	1993	Eggs	18
60	El Salvador	1993	Fats, Animals, Raw	7
60	El Salvador	1993	Freshwater Fish	2
60	El Salvador	1993	Fruits, Other	40
60	El Salvador	1993	Grapes	1
60	El Salvador	1993	Honey	1
60	El Salvador	1993	Lemons, Limes	11
60	El Salvador	1993	Maize	231
60	El Salvador	1993	Milk, Whole	186
60	El Salvador	1993	Nuts	1
60	El Salvador	1993	Offals, Edible	2
60	El Salvador	1993	Oilcrops Oil, Other	1
60	El Salvador	1993	Onions	3
60	El Salvador	1993	Oranges, Mandarines	62
60	El Salvador	1993	Pelagic Fish	2
60	El Salvador	1993	Pigmeat	6
60	El Salvador	1993	Pineapples	7
60	El Salvador	1993	Plantains	37
60	El Salvador	1993	Potatoes	6
60	El Salvador	1993	Poultry Meat	22
60	El Salvador	1993	Rice (Milled Equivalent)	26
60	El Salvador	1993	Sesameseed	1
60	El Salvador	1993	Sorghum	60
60	El Salvador	1993	Soyabean Oil	1
60	El Salvador	1993	Soyabeans	1
60	El Salvador	1993	Sugar, Non-Centrifugal	6
60	El Salvador	1993	Sugar, Refined Equivalent	87
60	El Salvador	1993	Sweeteners, Other	1
60	El Salvador	1993	Tomatoes	16
60	El Salvador	1993	Vegetables, Other	44
60	El Salvador	1993	Wheat	89
60	El Salvador	1994	Apples	4
60	El Salvador	1994	Bananas	10

FAO countrycode	Country	Reference year	Food item	Gross food supply (g *capita*$^{-1}$ d^{-1})
60	El Salvador	1994	Beans	28
60	El Salvador	1994	Beer	42
60	El Salvador	1994	Beverages, Alcoholic	5
60	El Salvador	1994	Bovine Meat	15
60	El Salvador	1994	Cassava	21
60	El Salvador	1994	Cheese	2
60	El Salvador	1994	Cocoa Beans	1
60	El Salvador	1994	Coconut Oil	1
60	El Salvador	1994	Coconuts - Incl Copra	17
60	El Salvador	1994	Coffee	3
60	El Salvador	1994	Cottonseed Oil	10
60	El Salvador	1994	Demersal Fish	1
60	El Salvador	1994	Eggs	19
60	El Salvador	1994	Fats, Animals, Raw	8
60	El Salvador	1994	Freshwater Fish	1
60	El Salvador	1994	Fruits, Other	45
60	El Salvador	1994	Grapes	1
60	El Salvador	1994	Honey	1
60	El Salvador	1994	Lemons, Limes	11
60	El Salvador	1994	Maize	231
60	El Salvador	1994	Marine Fish, Other	1
60	El Salvador	1994	Milk, Whole	179
60	El Salvador	1994	Nuts	1
60	El Salvador	1994	Offals, Edible	3
60	El Salvador	1994	Oilcrops Oil, Other	1
60	El Salvador	1994	Onions	3
60	El Salvador	1994	Oranges, Mandarines	59
60	El Salvador	1994	Pelagic Fish	2
60	El Salvador	1994	Pigmeat	7
60	El Salvador	1994	Pineapples	10
60	El Salvador	1994	Plantains	32
60	El Salvador	1994	Potatoes	7
60	El Salvador	1994	Poultry Meat	18
60	El Salvador	1994	Rice (Milled Equivalent)	26
60	El Salvador	1994	Roots, Other	26
60	El Salvador	1994	Sesameseed	1
60	El Salvador	1994	Sorghum	58
60	El Salvador	1994	Soyabean Oil	1
60	El Salvador	1994	Soyabeans	1
60	El Salvador	1994	Sugar, Non-Centrifugal	6
60	El Salvador	1994	Sugar, Refined Equivalent	78
60	El Salvador	1994	Sunflowerseed Oil	1
60	El Salvador	1994	Sweeteners, Other	1
60	El Salvador	1994	Tomatoes	11
60	El Salvador	1994	Vegetables, Other	46
60	El Salvador	1994	Wheat	90
60	El Salvador	1994	Wine	1
60	El Salvador	1995	Apples	3
60	El Salvador	1995	Bananas	7
60	El Salvador	1995	Beans	26
60	El Salvador	1995	Beer	47
60	El Salvador	1995	Beverages, Alcoholic	6
60	El Salvador	1995	Bovine Meat	18
60	El Salvador	1995	Cassava	14
60	El Salvador	1995	Cereals, Other	1
60	El Salvador	1995	Cheese	4
60	El Salvador	1995	Cocoa Beans	1
60	El Salvador	1995	Coconut Oil	2
60	El Salvador	1995	Coconuts - Incl Copra	17
60	El Salvador	1995	Coffee	4
60	El Salvador	1995	Cottonseed Oil	7
60	El Salvador	1995	Demersal Fish	1
60	El Salvador	1995	Eggs	17
60	El Salvador	1995	Fats, Animals, Raw	8
60	El Salvador	1995	Freshwater Fish	2
60	El Salvador	1995	Fruits, Other	46
60	El Salvador	1995	Grapes	2
60	El Salvador	1995	Groundnuts (Shelled Eq)	1
60	El Salvador	1995	Honey	1
60	El Salvador	1995	Lemons, Limes	10
60	El Salvador	1995	Maize	248
60	El Salvador	1995	Marine Fish, Other	1
60	El Salvador	1995	Milk, Whole	161
60	El Salvador	1995	Offals, Edible	3
60	El Salvador	1995	Oilcrops Oil, Other	1
60	El Salvador	1995	Onions	3
60	El Salvador	1995	Oranges, Mandarines	48
60	El Salvador	1995	Pelagic Fish	3
60	El Salvador	1995	Pigmeat	6
60	El Salvador	1995	Pineapples	7
60	El Salvador	1995	Plantains	37
60	El Salvador	1995	Potatoes	5
60	El Salvador	1995	Poultry Meat	19
60	El Salvador	1995	Rice (Milled Equivalent)	21
60	El Salvador	1995	Roots, Other	24
60	El Salvador	1995	Sesameseed	1
60	El Salvador	1995	Sorghum	61
60	El Salvador	1995	Soyabean Oil	1
60	El Salvador	1995	Soyabeans	1
60	El Salvador	1995	Sugar, Non-Centrifugal	6
60	El Salvador	1995	Sugar, Refined Equivalent	83
60	El Salvador	1995	Sunflowerseed Oil	1
60	El Salvador	1995	Sweeteners, Other	1
60	El Salvador	1995	Tomatoes	25
60	El Salvador	1995	Vegetables, Other	47
60	El Salvador	1995	Wheat	83
60	El Salvador	1995	Wine	1

FAO countrycode	Country	Reference year	Food item	Gross food supply (g *capita*$^{-1}$ d^{-1})
60	El Salvador	1996	Apples	5
60	El Salvador	1996	Bananas	8
60	El Salvador	1996	Beans	26
60	El Salvador	1996	Beer	45
60	El Salvador	1996	Beverages, Alcoholic	5
60	El Salvador	1996	Bovine Meat	19
60	El Salvador	1996	Cassava	13
60	El Salvador	1996	Cheese	6
60	El Salvador	1996	Cocoa Beans	1
60	El Salvador	1996	Coconut Oil	2
60	El Salvador	1996	Coconuts - Incl Copra	17
60	El Salvador	1996	Coffee	4
60	El Salvador	1996	Cottonseed Oil	6
60	El Salvador	1996	Eggs	16
60	El Salvador	1996	Fats, Animals, Raw	9
60	El Salvador	1996	Freshwater Fish	1
60	El Salvador	1996	Fruits, Other	46
60	El Salvador	1996	Grapefruit	1
60	El Salvador	1996	Grapes	2
60	El Salvador	1996	Groundnuts (Shelled Eq)	1
60	El Salvador	1996	Honey	1
60	El Salvador	1996	Lemons, Limes	10
60	El Salvador	1996	Maize	240
60	El Salvador	1996	Marine Fish, Other	1
60	El Salvador	1996	Milk, Whole	177
60	El Salvador	1996	Nuts	1
60	El Salvador	1996	Offals, Edible	3
60	El Salvador	1996	Oilcrops Oil, Other	1
60	El Salvador	1996	Onions	3
60	El Salvador	1996	Oranges, Mandarines	21
60	El Salvador	1996	Pelagic Fish	3
60	El Salvador	1996	Pigmeat	6
60	El Salvador	1996	Pineapples	4
60	El Salvador	1996	Plantains	22
60	El Salvador	1996	Potatoes	8
60	El Salvador	1996	Poultry Meat	19
60	El Salvador	1996	Rice (Milled Equivalent)	23
60	El Salvador	1996	Roots, Other	22
60	El Salvador	1996	Sesameseed	1
60	El Salvador	1996	Sorghum	59
60	El Salvador	1996	Soyabean Oil	1
60	El Salvador	1996	Soyabeans	1
60	El Salvador	1996	Sugar, Non-Centrifugal	6
60	El Salvador	1996	Sugar, Refined Equivalent	91
60	El Salvador	1996	Sunflowerseed Oil	1
60	El Salvador	1996	Tomatoes	18
60	El Salvador	1996	Vegetables, Other	46
60	El Salvador	1996	Wheat	90
60	El Salvador	1997	Apples	6
60	El Salvador	1997	Bananas	14
60	El Salvador	1997	Beans	26
60	El Salvador	1997	Beer	42
60	El Salvador	1997	Beverages, Alcoholic	7
60	El Salvador	1997	Bovine Meat	21
60	El Salvador	1997	Cassava	9
60	El Salvador	1997	Cheese	4
60	El Salvador	1997	Cocoa Beans	1
60	El Salvador	1997	Coconut Oil	2
60	El Salvador	1997	Coconuts - Incl Copra	17
60	El Salvador	1997	Coffee	4
60	El Salvador	1997	Cottonseed Oil	5
60	El Salvador	1997	Demersal Fish	1
60	El Salvador	1997	Eggs	18
60	El Salvador	1997	Fats, Animals, Raw	8
60	El Salvador	1997	Freshwater Fish	1
60	El Salvador	1997	Fruits, Other	53
60	El Salvador	1997	Grapefruit	1
60	El Salvador	1997	Grapes	2
60	El Salvador	1997	Groundnuts (Shelled Eq)	1
60	El Salvador	1997	Honey	1
60	El Salvador	1997	Lemons, Limes	13
60	El Salvador	1997	Maize	228
60	El Salvador	1997	Marine Fish, Other	1
60	El Salvador	1997	Milk, Whole	196
60	El Salvador	1997	Nuts	1
60	El Salvador	1997	Offals, Edible	3
60	El Salvador	1997	Oilcrops Oil, Other	1
60	El Salvador	1997	Onions	4
60	El Salvador	1997	Oranges, Mandarines	23
60	El Salvador	1997	Pelagic Fish	3
60	El Salvador	1997	Pigmeat	6
60	El Salvador	1997	Pineapples	4
60	El Salvador	1997	Plantains	16
60	El Salvador	1997	Potatoes	11
60	El Salvador	1997	Poultry Meat	20
60	El Salvador	1997	Rice (Milled Equivalent)	25
60	El Salvador	1997	Roots, Other	23
60	El Salvador	1997	Sesameseed	1
60	El Salvador	1997	Sorghum	63
60	El Salvador	1997	Soyabean Oil	1
60	El Salvador	1997	Soyabeans	1
60	El Salvador	1997	Sugar, Non-Centrifugal	6
60	El Salvador	1997	Sugar, Refined Equivalent	100
60	El Salvador	1997	Sunflowerseed Oil	1
60	El Salvador	1997	Tomatoes	19
60	El Salvador	1997	Vegetables, Other	54
60	El Salvador	1997	Wheat	89

FAO country code	Country	Reference year	Food item	Gross food supply (g $capita^{-1}$ d^{-1})
60	El Salvador	1998	Apples	5
60	El Salvador	1998	Bananas	17
60	El Salvador	1998	Beans	26
60	El Salvador	1998	Beer	40
60	El Salvador	1998	Beverages, Alcoholic	7
60	El Salvador	1998	Bovine Meat	18
60	El Salvador	1998	Cassava	3
60	El Salvador	1998	Cheese	3
60	El Salvador	1998	Cocoa Beans	1
60	El Salvador	1998	Coconut Oil	2
60	El Salvador	1998	Coconuts - Incl Copra	17
60	El Salvador	1998	Coffee	4
60	El Salvador	1998	Cottonseed Oil	4
60	El Salvador	1998	Eggs	17
60	El Salvador	1998	Fats, Animals, Raw	9
60	El Salvador	1998	Freshwater Fish	1
60	El Salvador	1998	Fruits, Other	46
60	El Salvador	1998	Grapefruit	1
60	El Salvador	1998	Grapes	2
60	El Salvador	1998	Groundnuts (Shelled Eq)	1
60	El Salvador	1998	Honey	1
60	El Salvador	1998	Lemons, Limes	9
60	El Salvador	1998	Maize	219
60	El Salvador	1998	Milk, Whole	189
60	El Salvador	1998	Offals, Edible	3
60	El Salvador	1998	Oilcrops Oil, Other	1
60	El Salvador	1998	Onions	2
60	El Salvador	1998	Oranges, Mandarines	16
60	El Salvador	1998	Pelagic Fish	2
60	El Salvador	1998	Pigmeat	7
60	El Salvador	1998	Pineapples	2
60	El Salvador	1998	Plantains	14
60	El Salvador	1998	Potatoes	7
60	El Salvador	1998	Poultry Meat	23
60	El Salvador	1998	Rice (Milled Equivalent)	27
60	El Salvador	1998	Roots, Other	22
60	El Salvador	1998	Sesameseed	2
60	El Salvador	1998	Sorghum	60
60	El Salvador	1998	Soyabean Oil	5
60	El Salvador	1998	Soyabeans	1
60	El Salvador	1998	Sugar, Non-Centrifugal	6
60	El Salvador	1998	Sugar, Refined Equivalent	102
60	El Salvador	1998	Sunflowerseed Oil	3
60	El Salvador	1998	Sweeteners, Other	1
60	El Salvador	1998	Tomatoes	24
60	El Salvador	1998	Vegetables, Other	50
60	El Salvador	1998	Wheat	90
60	El Salvador	1998	Wine	1
60	El Salvador	1999	Apples	7
60	El Salvador	1999	Bananas	13
60	El Salvador	1999	Beans	30
60	El Salvador	1999	Beer	46
60	El Salvador	1999	Beverages, Alcoholic	8
60	El Salvador	1999	Bovine Meat	18
60	El Salvador	1999	Cassava	9
60	El Salvador	1999	Cheese	4
60	El Salvador	1999	Cocoa Beans	1
60	El Salvador	1999	Coconut Oil	2
60	El Salvador	1999	Coconuts - Incl Copra	22
60	El Salvador	1999	Coffee	4
60	El Salvador	1999	Cottonseed Oil	3
60	El Salvador	1999	Eggs	19
60	El Salvador	1999	Fats, Animals, Raw	9
60	El Salvador	1999	Freshwater Fish	1
60	El Salvador	1999	Fruits, Other	51
60	El Salvador	1999	Grapes	3
60	El Salvador	1999	Groundnuts (Shelled Eq)	1
60	El Salvador	1999	Honey	1
60	El Salvador	1999	Lemons, Limes	8
60	El Salvador	1999	Maize	209
60	El Salvador	1999	Milk, Whole	209
60	El Salvador	1999	Molluscs, Other	1
60	El Salvador	1999	Offals, Edible	3
60	El Salvador	1999	Oilcrops Oil, Other	1
60	El Salvador	1999	Olive Oil	1
60	El Salvador	1999	Onions	5
60	El Salvador	1999	Oranges, Mandarines	21
60	El Salvador	1999	Pelagic Fish	3
60	El Salvador	1999	Pigmeat	8
60	El Salvador	1999	Pineapples	4
60	El Salvador	1999	Plantains	22
60	El Salvador	1999	Potatoes	14
60	El Salvador	1999	Poultry Meat	21
60	El Salvador	1999	Rice (Milled Equivalent)	24
60	El Salvador	1999	Roots, Other	24
60	El Salvador	1999	Sesameseed	2
60	El Salvador	1999	Sorghum	47
60	El Salvador	1999	Soyabean Oil	7
60	El Salvador	1999	Soyabeans	1
60	El Salvador	1999	Sugar, Non-Centrifugal	6
60	El Salvador	1999	Sugar, Refined Equivalent	100
60	El Salvador	1999	Sunflowerseed Oil	3
60	El Salvador	1999	Tomatoes	22
60	El Salvador	1999	Vegetables, Other	63
60	El Salvador	1999	Wheat	87
60	El Salvador	1999	Wine	1
60	El Salvador	2000	Apples	8

FAO country code	Country	Reference year	Food item	Gross food supply (g *capita*$^{-1}$ d^{-1})
60	El Salvador	2000	Bananas	22
60	El Salvador	2000	Beans	36
60	El Salvador	2000	Beer	44
60	El Salvador	2000	Beverages, Alcoholic	11
60	El Salvador	2000	Bovine Meat	22
60	El Salvador	2000	Butter, Ghee	1
60	El Salvador	2000	Cassava	8
60	El Salvador	2000	Cheese	5
60	El Salvador	2000	Cocoa Beans	1
60	El Salvador	2000	Coconut Oil	2
60	El Salvador	2000	Coconuts - Incl Copra	23
60	El Salvador	2000	Coffee	4
60	El Salvador	2000	Cottonseed Oil	3
60	El Salvador	2000	Demersal Fish	1
60	El Salvador	2000	Eggs	21
60	El Salvador	2000	Fats, Animals, Raw	8
60	El Salvador	2000	Freshwater Fish	1
60	El Salvador	2000	Fruits, Other	54
60	El Salvador	2000	Grapefruit	1
60	El Salvador	2000	Grapes	3
60	El Salvador	2000	Groundnuts (Shelled Eq)	1
60	El Salvador	2000	Lemons, Limes	7
60	El Salvador	2000	Maize	214
60	El Salvador	2000	Marine Fish, Other	1
60	El Salvador	2000	Milk, Whole	210
60	El Salvador	2000	Molluscs, Other	1
60	El Salvador	2000	Offals, Edible	3
60	El Salvador	2000	Oilcrops Oil, Other	1
60	El Salvador	2000	Onions	5
60	El Salvador	2000	Oranges, Mandarines	19
60	El Salvador	2000	Pelagic Fish	3
60	El Salvador	2000	Pigmeat	7
60	El Salvador	2000	Pineapples	2
60	El Salvador	2000	Plantains	40
60	El Salvador	2000	Potatoes	29
60	El Salvador	2000	Poultry Meat	29
60	El Salvador	2000	Rice (Milled Equivalent)	30
60	El Salvador	2000	Roots, Other	22
60	El Salvador	2000	Sesameseed	2
60	El Salvador	2000	Sorghum	47
60	El Salvador	2000	Soyabean Oil	8
60	El Salvador	2000	Soyabeans	1
60	El Salvador	2000	Sugar, Non-Centrifugal	6
60	El Salvador	2000	Sugar, Refined Equivalent	100
60	El Salvador	2000	Sunflowerseed Oil	2
60	El Salvador	2000	Tomatoes	22
60	El Salvador	2000	Vegetables, Other	105
60	El Salvador	2000	Wheat	91
60	El Salvador	2000	Wine	1
60	El Salvador	2001	Apples	5
60	El Salvador	2001	Bananas	23
60	El Salvador	2001	Beans	38
60	El Salvador	2001	Beer	41
60	El Salvador	2001	Beverages, Alcoholic	11
60	El Salvador	2001	Bovine Meat	21
60	El Salvador	2001	Butter, Ghee	1
60	El Salvador	2001	Cassava	8
60	El Salvador	2001	Cereals, Other	2
60	El Salvador	2001	Cheese	5
60	El Salvador	2001	Citrus, Other	3
60	El Salvador	2001	Cocoa Beans	1
60	El Salvador	2001	Coconut Oil	2
60	El Salvador	2001	Coconuts - Incl Copra	23
60	El Salvador	2001	Coffee	4
60	El Salvador	2001	Cottonseed Oil	3
60	El Salvador	2001	Demersal Fish	1
60	El Salvador	2001	Eggs	23
60	El Salvador	2001	Fats, Animals, Raw	9
60	El Salvador	2001	Freshwater Fish	1
60	El Salvador	2001	Fruits, Other	49
60	El Salvador	2001	Grapefruit	1
60	El Salvador	2001	Grapes	3
60	El Salvador	2001	Groundnuts (Shelled Eq)	1
60	El Salvador	2001	Lemons, Limes	9
60	El Salvador	2001	Maize	220
60	El Salvador	2001	Maize Germ Oil	2
60	El Salvador	2001	Marine Fish, Other	1
60	El Salvador	2001	Milk, Whole	211
60	El Salvador	2001	Molluscs, Other	1
60	El Salvador	2001	Nuts	1
60	El Salvador	2001	Offals, Edible	3
60	El Salvador	2001	Oilcrops Oil, Other	1
60	El Salvador	2001	Olive Oil	1
60	El Salvador	2001	Onions	6
60	El Salvador	2001	Oranges, Mandarines	22
60	El Salvador	2001	Pelagic Fish	8
60	El Salvador	2001	Pigmeat	6
60	El Salvador	2001	Pineapples	4
60	El Salvador	2001	Plantains	45
60	El Salvador	2001	Potatoes	41
60	El Salvador	2001	Poultry Meat	36
60	El Salvador	2001	Rice (Milled Equivalent)	29
60	El Salvador	2001	Roots, Other	23
60	El Salvador	2001	Sesameseed	2
60	El Salvador	2001	Sorghum	47
60	El Salvador	2001	Soyabean Oil	8
60	El Salvador	2001	Soyabeans	2

FAO country code	Country	Reference year	Food item	Gross food supply (g $capita^{-1}$ d^{-1})
60	El Salvador	2001	Sugar, Non-Centrifugal	6
60	El Salvador	2001	Sugar, Refined Equivalent	97
60	El Salvador	2001	Sunflowerseed Oil	2
60	El Salvador	2001	Tomatoes	28
60	El Salvador	2001	Vegetables, Other	103
60	El Salvador	2001	Wheat	93
60	El Salvador	2001	Wine	1
60	El Salvador	2002	Apples	21
60	El Salvador	2002	Bananas	22
60	El Salvador	2002	Beans	45
60	El Salvador	2002	Beer	41
60	El Salvador	2002	Beverages, Alcoholic	11
60	El Salvador	2002	Bovine Meat	25
60	El Salvador	2002	Butter, Ghee	1
60	El Salvador	2002	Cassava	8
60	El Salvador	2002	Cereals, Other	2
60	El Salvador	2002	Cheese	8
60	El Salvador	2002	Citrus, Other	4
60	El Salvador	2002	Cocoa Beans	1
60	El Salvador	2002	Coconut Oil	2
60	El Salvador	2002	Coconuts - Incl Copra	24
60	El Salvador	2002	Coffee	4
60	El Salvador	2002	Cottonseed Oil	3
60	El Salvador	2002	Crustaceans	4
60	El Salvador	2002	Demersal Fish	1
60	El Salvador	2002	Eggs	26
60	El Salvador	2002	Fats, Animals, Raw	8
60	El Salvador	2002	Freshwater Fish	2
60	El Salvador	2002	Fruits, Other	45
60	El Salvador	2002	Grapes	3
60	El Salvador	2002	Groundnuts (Shelled Eq)	2
60	El Salvador	2002	Lemons, Limes	8
60	El Salvador	2002	Maize	215
60	El Salvador	2002	Maize Germ Oil	1
60	El Salvador	2002	Marine Fish, Other	2
60	El Salvador	2002	Milk, Whole	211
60	El Salvador	2002	Molluscs, Other	1
60	El Salvador	2002	Nuts	1
60	El Salvador	2002	Offals, Edible	4
60	El Salvador	2002	Oilcrops Oil, Other	1
60	El Salvador	2002	Onions	6
60	El Salvador	2002	Oranges, Mandarines	25
60	El Salvador	2002	Pelagic Fish	7
60	El Salvador	2002	Pigmeat	6
60	El Salvador	2002	Pineapples	2
60	El Salvador	2002	Plantains	45
60	El Salvador	2002	Potatoes	46
60	El Salvador	2002	Poultry Meat	39
60	El Salvador	2002	Rice (Milled Equivalent)	31
60	El Salvador	2002	Roots, Other	24
60	El Salvador	2002	Sesameseed	2
60	El Salvador	2002	Sorghum	47
60	El Salvador	2002	Soyabean Oil	9
60	El Salvador	2002	Soyabeans	2
60	El Salvador	2002	Sugar, Non-Centrifugal	6
60	El Salvador	2002	Sugar, Refined Equivalent	91
60	El Salvador	2002	Sunflowerseed Oil	2
60	El Salvador	2002	Sweeteners, Other	4
60	El Salvador	2002	Tomatoes	33
60	El Salvador	2002	Vegetables, Other	110
60	El Salvador	2002	Wheat	93
60	El Salvador	2002	Wine	1
60	El Salvador	2003	Apples	20
60	El Salvador	2003	Bananas	21
60	El Salvador	2003	Beans	43
60	El Salvador	2003	Beer	48
60	El Salvador	2003	Beverages, Alcoholic	11
60	El Salvador	2003	Bovine Meat	21
60	El Salvador	2003	Butter, Ghee	1
60	El Salvador	2003	Cassava	7
60	El Salvador	2003	Cereals, Other	2
60	El Salvador	2003	Cheese	9
60	El Salvador	2003	Citrus, Other	1
60	El Salvador	2003	Cocoa Beans	1
60	El Salvador	2003	Coconut Oil	1
60	El Salvador	2003	Coconuts - Incl Copra	24
60	El Salvador	2003	Coffee	7
60	El Salvador	2003	Cottonseed Oil	1
60	El Salvador	2003	Crustaceans	6
60	El Salvador	2003	Demersal Fish	1
60	El Salvador	2003	Eggs	26
60	El Salvador	2003	Fats, Animals, Raw	9
60	El Salvador	2003	Freshwater Fish	2
60	El Salvador	2003	Fruits, Other	49
60	El Salvador	2003	Grapes	3
60	El Salvador	2003	Groundnuts (Shelled Eq)	1
60	El Salvador	2003	Lemons, Limes	6
60	El Salvador	2003	Maize	221
60	El Salvador	2003	Marine Fish, Other	2
60	El Salvador	2003	Milk, Whole	203
60	El Salvador	2003	Molluscs, Other	1
60	El Salvador	2003	Nuts	1
60	El Salvador	2003	Offals, Edible	4
60	El Salvador	2003	Oilcrops Oil, Other	1
60	El Salvador	2003	Olive Oil	1
60	El Salvador	2003	Onions	8
60	El Salvador	2003	Oranges, Mandarines	31

FAO country code	Country	Reference year	Food item	Gross food supply (g *capita*$^{-1}$ d^{-1})
60	El Salvador	2003	Pelagic Fish	4
60	El Salvador	2003	Pigmeat	6
60	El Salvador	2003	Pineapples	3
60	El Salvador	2003	Plantains	46
60	El Salvador	2003	Potatoes	41
60	El Salvador	2003	Poultry Meat	37
60	El Salvador	2003	Rice (Milled Equivalent)	31
60	El Salvador	2003	Roots, Other	20
60	El Salvador	2003	Sesameseed	1
60	El Salvador	2003	Sorghum	47
60	El Salvador	2003	Soyabean Oil	9
60	El Salvador	2003	Soyabeans	2
60	El Salvador	2003	Sugar, Non-Centrifugal	6
60	El Salvador	2003	Sugar, Refined Equivalent	87
60	El Salvador	2003	Sunflowerseed Oil	3
60	El Salvador	2003	Sweeteners, Other	4
60	El Salvador	2003	Tomatoes	37
60	El Salvador	2003	Vegetables, Other	100
60	El Salvador	2003	Wheat	81
60	El Salvador	2004	Apples	13
60	El Salvador	2004	Bananas	19
60	El Salvador	2004	Beans	40
60	El Salvador	2004	Beer	54
60	El Salvador	2004	Beverages, Alcoholic	12
60	El Salvador	2004	Bovine Meat	20
60	El Salvador	2004	Butter, Ghee	1
60	El Salvador	2004	Cassava	9
60	El Salvador	2004	Cereals, Other	1
60	El Salvador	2004	Cheese	9
60	El Salvador	2004	Citrus, Other	1
60	El Salvador	2004	Cocoa Beans	1
60	El Salvador	2004	Coconut Oil	1
60	El Salvador	2004	Coconuts - Incl Copra	25
60	El Salvador	2004	Coffee	7
60	El Salvador	2004	Cottonseed Oil	1
60	El Salvador	2004	Crustaceans	5
60	El Salvador	2004	Demersal Fish	1
60	El Salvador	2004	Eggs	27
60	El Salvador	2004	Fats, Animals, Raw	8
60	El Salvador	2004	Freshwater Fish	2
60	El Salvador	2004	Fruits, Other	54
60	El Salvador	2004	Grapes	3
60	El Salvador	2004	Groundnuts (Shelled Eq)	1
60	El Salvador	2004	Lemons, Limes	8
60	El Salvador	2004	Maize	235
60	El Salvador	2004	Maize Germ Oil	1
60	El Salvador	2004	Marine Fish, Other	2
60	El Salvador	2004	Milk, Whole	208
60	El Salvador	2004	Molluscs, Other	1
60	El Salvador	2004	Nuts	1
60	El Salvador	2004	Oats	1
60	El Salvador	2004	Offals, Edible	3
60	El Salvador	2004	Oilcrops Oil, Other	2
60	El Salvador	2004	Olive Oil	1
60	El Salvador	2004	Onions	10
60	El Salvador	2004	Oranges, Mandarines	35
60	El Salvador	2004	Pelagic Fish	6
60	El Salvador	2004	Pigmeat	7
60	El Salvador	2004	Pineapples	4
60	El Salvador	2004	Plantains	47
60	El Salvador	2004	Potatoes	33
60	El Salvador	2004	Poultry Meat	43
60	El Salvador	2004	Rice (Milled Equivalent)	26
60	El Salvador	2004	Roots, Other	13
60	El Salvador	2004	Sesameseed	1
60	El Salvador	2004	Sorghum	45
60	El Salvador	2004	Soyabean Oil	6
60	El Salvador	2004	Soyabeans	2
60	El Salvador	2004	Sugar, Refined Equivalent	90
60	El Salvador	2004	Sunflowerseed Oil	3
60	El Salvador	2004	Sweeteners, Other	5
60	El Salvador	2004	Tomatoes	41
60	El Salvador	2004	Vegetables, Other	104
60	El Salvador	2004	Wheat	79
60	El Salvador	2004	Wine	1
60	El Salvador	2005	Apples	14
60	El Salvador	2005	Bananas	19
60	El Salvador	2005	Beans	36
60	El Salvador	2005	Beer	59
60	El Salvador	2005	Beverages, Alcoholic	10
60	El Salvador	2005	Bovine Meat	21
60	El Salvador	2005	Cassava	11
60	El Salvador	2005	Cereals, Other	1
60	El Salvador	2005	Cheese	8
60	El Salvador	2005	Citrus, Other	1
60	El Salvador	2005	Cocoa Beans	1
60	El Salvador	2005	Coconut Oil	1
60	El Salvador	2005	Coconuts - Incl Copra	24
60	El Salvador	2005	Coffee	6
60	El Salvador	2005	Crustaceans	8
60	El Salvador	2005	Demersal Fish	2
60	El Salvador	2005	Eggs	26
60	El Salvador	2005	Fats, Animals, Raw	9
60	El Salvador	2005	Freshwater Fish	2
60	El Salvador	2005	Fruits, Other	57
60	El Salvador	2005	Grapes	3
60	El Salvador	2005	Groundnuts (Shelled Eq)	1

FAO country code	Country	Reference year	Food item	Gross food supply (g $capita^{-1}$ d^{-1})
60	El Salvador	2005	Honey	1
60	El Salvador	2005	Lemons, Limes	8
60	El Salvador	2005	Maize	221
60	El Salvador	2005	Marine Fish, Other	2
60	El Salvador	2005	Milk, Whole	239
60	El Salvador	2005	Nuts	1
60	El Salvador	2005	Oats	1
60	El Salvador	2005	Offals, Edible	3
60	El Salvador	2005	Oilcrops Oil, Other	4
60	El Salvador	2005	Olive Oil	1
60	El Salvador	2005	Onions	11
60	El Salvador	2005	Oranges, Mandarines	51
60	El Salvador	2005	Pelagic Fish	7
60	El Salvador	2005	Pigmeat	8
60	El Salvador	2005	Pineapples	4
60	El Salvador	2005	Plantains	48
60	El Salvador	2005	Potatoes	29
60	El Salvador	2005	Poultry Meat	46
60	El Salvador	2005	Rice (Milled Equivalent)	28
60	El Salvador	2005	Roots, Other	15
60	El Salvador	2005	Sesameseed	1
60	El Salvador	2005	Sorghum	42
60	El Salvador	2005	Soyabean Oil	7
60	El Salvador	2005	Soyabeans	2
60	El Salvador	2005	Sugar, Refined Equivalent	94
60	El Salvador	2005	Sunflowerseed Oil	3
60	El Salvador	2005	Sweeteners, Other	7
60	El Salvador	2005	Tomatoes	45
60	El Salvador	2005	Vegetables, Other	132
60	El Salvador	2005	Wheat	73
60	El Salvador	2005	Wine	1
60	El Salvador	2006	Apples	14
60	El Salvador	2006	Bananas	18
60	El Salvador	2006	Beans	48
60	El Salvador	2006	Beer	65
60	El Salvador	2006	Beverages, Alcoholic	9
60	El Salvador	2006	Bovine Meat	24
60	El Salvador	2006	Cassava	10
60	El Salvador	2006	Cereals, Other	1
60	El Salvador	2006	Cheese	9
60	El Salvador	2006	Cocoa Beans	1
60	El Salvador	2006	Coconut Oil	1
60	El Salvador	2006	Coconuts - Incl Copra	24
60	El Salvador	2006	Coffee	7
60	El Salvador	2006	Crustaceans	7
60	El Salvador	2006	Demersal Fish	2
60	El Salvador	2006	Eggs	27
60	El Salvador	2006	Fats, Animals, Raw	10
60	El Salvador	2006	Freshwater Fish	2
60	El Salvador	2006	Fruits, Other	54
60	El Salvador	2006	Grapes	3
60	El Salvador	2006	Groundnuts (Shelled Eq)	1
60	El Salvador	2006	Lemons, Limes	8
60	El Salvador	2006	Maize	190
60	El Salvador	2006	Maize Germ Oil	1
60	El Salvador	2006	Marine Fish, Other	3
60	El Salvador	2006	Milk, Whole	219
60	El Salvador	2006	Nuts	2
60	El Salvador	2006	Oats	1
60	El Salvador	2006	Offals, Edible	4
60	El Salvador	2006	Oilcrops Oil, Other	4
60	El Salvador	2006	Olive Oil	1
60	El Salvador	2006	Onions	13
60	El Salvador	2006	Oranges, Mandarines	51
60	El Salvador	2006	Pelagic Fish	3
60	El Salvador	2006	Pigmeat	10
60	El Salvador	2006	Pineapples	4
60	El Salvador	2006	Plantains	50
60	El Salvador	2006	Potatoes	35
60	El Salvador	2006	Poultry Meat	47
60	El Salvador	2006	Rice (Milled Equivalent)	29
60	El Salvador	2006	Roots, Other	13
60	El Salvador	2006	Sesameseed	1
60	El Salvador	2006	Sorghum	45
60	El Salvador	2006	Soyabean Oil	8
60	El Salvador	2006	Soyabeans	2
60	El Salvador	2006	Sugar, Refined Equivalent	100
60	El Salvador	2006	Sunflowerseed Oil	4
60	El Salvador	2006	Sweeteners, Other	7
60	El Salvador	2006	Tomatoes	52
60	El Salvador	2006	Vegetables, Other	149
60	El Salvador	2006	Wheat	74
60	El Salvador	2006	Wine	1
60	El Salvador	2007	Apples	14
60	El Salvador	2007	Bananas	19
60	El Salvador	2007	Beans	36
60	El Salvador	2007	Beer	65
60	El Salvador	2007	Beverages, Alcoholic	9
60	El Salvador	2007	Bovine Meat	25
60	El Salvador	2007	Cassava	13
60	El Salvador	2007	Cereals, Other	2
60	El Salvador	2007	Cheese	11
60	El Salvador	2007	Cocoa Beans	1
60	El Salvador	2007	Coconut Oil	1
60	El Salvador	2007	Coconuts - Incl Copra	27
60	El Salvador	2007	Coffee	8
60	El Salvador	2007	Crustaceans	7

FAO countrycode	Country	Reference year	Food item	Gross food supply (g *capita*$^{-1}$ d^{-1})
60	El Salvador	2007	Demersal Fish	2
60	El Salvador	2007	Eggs	26
60	El Salvador	2007	Fats, Animals, Raw	9
60	El Salvador	2007	Freshwater Fish	3
60	El Salvador	2007	Fruits, Other	55
60	El Salvador	2007	Grapes	3
60	El Salvador	2007	Groundnuts (Shelled Eq)	1
60	El Salvador	2007	Lemons, Limes	9
60	El Salvador	2007	Maize	193
60	El Salvador	2007	Marine Fish, Other	3
60	El Salvador	2007	Milk, Whole	226
60	El Salvador	2007	Molluscs, Other	1
60	El Salvador	2007	Nuts	1
60	El Salvador	2007	Oats	1
60	El Salvador	2007	Offals, Edible	3
60	El Salvador	2007	Oilcrops Oil, Other	3
60	El Salvador	2007	Olive Oil	1
60	El Salvador	2007	Onions	12
60	El Salvador	2007	Oranges, Mandarines	54
60	El Salvador	2007	Pelagic Fish	3
60	El Salvador	2007	Pigmeat	8
60	El Salvador	2007	Pineapples	5
60	El Salvador	2007	Plantains	55
60	El Salvador	2007	Potatoes	39
60	El Salvador	2007	Poultry Meat	50
60	El Salvador	2007	Rice (Milled Equivalent)	28
60	El Salvador	2007	Roots, Other	16
60	El Salvador	2007	Sesameseed	1
60	El Salvador	2007	Sorghum	45
60	El Salvador	2007	Soyabean Oil	9
60	El Salvador	2007	Soyabeans	2
60	El Salvador	2007	Sugar, Refined Equivalent	98
60	El Salvador	2007	Sunflowerseed Oil	4
60	El Salvador	2007	Sweeteners, Other	6
60	El Salvador	2007	Tomatoes	50
60	El Salvador	2007	Vegetables, Other	154
60	El Salvador	2007	Wheat	75
60	El Salvador	2007	Wine	1
60	El Salvador	2008	Apples	14
60	El Salvador	2008	Bananas	19
60	El Salvador	2008	Beans	47
60	El Salvador	2008	Beer	58
60	El Salvador	2008	Beverages, Alcoholic	8
60	El Salvador	2008	Bovine Meat	25
60	El Salvador	2008	Cassava	12
60	El Salvador	2008	Cereals, Other	3
60	El Salvador	2008	Cheese	11
60	El Salvador	2008	Cocoa Beans	1
60	El Salvador	2008	Coconut Oil	1
60	El Salvador	2008	Coconuts - Incl Copra	23
60	El Salvador	2008	Coffee	8
60	El Salvador	2008	Crustaceans	3
60	El Salvador	2008	Eggs	24
60	El Salvador	2008	Fats, Animals, Raw	6
60	El Salvador	2008	Freshwater Fish	3
60	El Salvador	2008	Fruits, Other	74
60	El Salvador	2008	Grapes	3
60	El Salvador	2008	Groundnuts (Shelled Eq)	1
60	El Salvador	2008	Honey	1
60	El Salvador	2008	Lemons, Limes	8
60	El Salvador	2008	Maize	195
60	El Salvador	2008	Marine Fish, Other	1
60	El Salvador	2008	Milk, Whole	228
60	El Salvador	2008	Nuts	2
60	El Salvador	2008	Oats	1
60	El Salvador	2008	Offals, Edible	4
60	El Salvador	2008	Oilcrops Oil, Other	3
60	El Salvador	2008	Olive Oil	1
60	El Salvador	2008	Onions	12
60	El Salvador	2008	Oranges, Mandarines	48
60	El Salvador	2008	Pelagic Fish	3
60	El Salvador	2008	Pigmeat	8
60	El Salvador	2008	Pineapples	6
60	El Salvador	2008	Plantains	56
60	El Salvador	2008	Potatoes	29
60	El Salvador	2008	Poultry Meat	44
60	El Salvador	2008	Rice (Milled Equivalent)	30
60	El Salvador	2008	Roots, Other	17
60	El Salvador	2008	Sesameseed	1
60	El Salvador	2008	Sorghum	45
60	El Salvador	2008	Soyabean Oil	8
60	El Salvador	2008	Soyabeans	2
60	El Salvador	2008	Sugar, Refined Equivalent	99
60	El Salvador	2008	Sunflowerseed Oil	3
60	El Salvador	2008	Sweeteners, Other	4
60	El Salvador	2008	Tomatoes	53
60	El Salvador	2008	Vegetables, Other	160
60	El Salvador	2008	Wheat	77
60	El Salvador	2008	Wine	1
60	El Salvador	2009	Apples	2
60	El Salvador	2009	Bananas	15
60	El Salvador	2009	Beans	42
60	El Salvador	2009	Beer	57
60	El Salvador	2009	Beverages, Alcoholic	9
60	El Salvador	2009	Bovine Meat	24
60	El Salvador	2009	Cassava	11
60	El Salvador	2009	Cereals, Other	2

FAO country code	Country	Reference year	Food item	Gross food supply (g *capita*$^{-1}$ d^{-1})
60	El Salvador	2009	Cheese	10
60	El Salvador	2009	Citrus, Other	1
60	El Salvador	2009	Cocoa Beans	1
60	El Salvador	2009	Coconut Oil	1
60	El Salvador	2009	Coconuts - Incl Copra	22
60	El Salvador	2009	Coffee	6
60	El Salvador	2009	Crustaceans	5
60	El Salvador	2009	Eggs	24
60	El Salvador	2009	Fats, Animals, Raw	9
60	El Salvador	2009	Freshwater Fish	3
60	El Salvador	2009	Fruits, Other	76
60	El Salvador	2009	Grapes	3
60	El Salvador	2009	Groundnuts (Shelled Eq)	1
60	El Salvador	2009	Lemons, Limes	13
60	El Salvador	2009	Maize	199
60	El Salvador	2009	Milk, Whole	249
60	El Salvador	2009	Nuts	2
60	El Salvador	2009	Oats	1
60	El Salvador	2009	Offals, Edible	3
60	El Salvador	2009	Oilcrops Oil, Other	3
60	El Salvador	2009	Olive Oil	1
60	El Salvador	2009	Onions	12
60	El Salvador	2009	Oranges, Mandarines	45
60	El Salvador	2009	Pelagic Fish	11
60	El Salvador	2009	Pigmeat	8
60	El Salvador	2009	Pineapples	4
60	El Salvador	2009	Plantains	53
60	El Salvador	2009	Potatoes	31
60	El Salvador	2009	Poultry Meat	45
60	El Salvador	2009	Rice (Milled Equivalent)	27
60	El Salvador	2009	Roots, Other	16
60	El Salvador	2009	Sorghum	46
60	El Salvador	2009	Soyabean Oil	8
60	El Salvador	2009	Soyabeans	2
60	El Salvador	2009	Sugar, Refined Equivalent	99
60	El Salvador	2009	Sunflowerseed Oil	3
60	El Salvador	2009	Sweeteners, Other	4
60	El Salvador	2009	Tomatoes	45
60	El Salvador	2009	Vegetables, Other	121
60	El Salvador	2009	Wheat	77
60	El Salvador	2009	Wine	1
60	El Salvador	2010	Apples	2
60	El Salvador	2010	Bananas	17
60	El Salvador	2010	Beans	37
60	El Salvador	2010	Beer	57
60	El Salvador	2010	Beverages, Alcoholic	7
60	El Salvador	2010	Bovine Meat	25
60	El Salvador	2010	Cassava	11
60	El Salvador	2010	Cheese	11
60	El Salvador	2010	Citrus, Other	1
60	El Salvador	2010	Cocoa Beans	1
60	El Salvador	2010	Coconut Oil	1
60	El Salvador	2010	Coconuts - Incl Copra	20
60	El Salvador	2010	Coffee	8
60	El Salvador	2010	Crustaceans	2
60	El Salvador	2010	Eggs	24
60	El Salvador	2010	Fats, Animals, Raw	8
60	El Salvador	2010	Freshwater Fish	3
60	El Salvador	2010	Fruits, Other	69
60	El Salvador	2010	Grapes	3
60	El Salvador	2010	Groundnuts (Shelled Eq)	1
60	El Salvador	2010	Lemons, Limes	10
60	El Salvador	2010	Maize	210
60	El Salvador	2010	Marine Fish, Other	7
60	El Salvador	2010	Milk, Whole	216
60	El Salvador	2010	Nuts	2
60	El Salvador	2010	Oats	1
60	El Salvador	2010	Offals, Edible	3
60	El Salvador	2010	Oilcrops Oil, Other	2
60	El Salvador	2010	Olive Oil	1
60	El Salvador	2010	Onions	11
60	El Salvador	2010	Oranges, Mandarines	42
60	El Salvador	2010	Pelagic Fish	6
60	El Salvador	2010	Pigmeat	8
60	El Salvador	2010	Pineapples	2
60	El Salvador	2010	Plantains	29
60	El Salvador	2010	Potatoes	29
60	El Salvador	2010	Poultry Meat	48
60	El Salvador	2010	Rice (Milled Equivalent)	28
60	El Salvador	2010	Roots, Other	16
60	El Salvador	2010	Sorghum	46
60	El Salvador	2010	Soyabean Oil	7
60	El Salvador	2010	Soyabeans	2
60	El Salvador	2010	Sugar, Refined Equivalent	98
60	El Salvador	2010	Sunflowerseed Oil	3
60	El Salvador	2010	Sweeteners, Other	2
60	El Salvador	2010	Tomatoes	37
60	El Salvador	2010	Vegetables, Other	118
60	El Salvador	2010	Wheat	78
60	El Salvador	2010	Wine	1
60	El Salvador	2011	Apples	2
60	El Salvador	2011	Bananas	16
60	El Salvador	2011	Beans	42
60	El Salvador	2011	Beer	52
60	El Salvador	2011	Beverages, Alcoholic	8
60	El Salvador	2011	Bovine Meat	20
60	El Salvador	2011	Cassava	15

FAO country code	Country	Reference year	Food item	Gross food supply (g $capita^{-1}$ d^{-1})
60	El Salvador	2011	Cereals, Other	1
60	El Salvador	2011	Cheese	11
60	El Salvador	2011	Cocoa Beans	1
60	El Salvador	2011	Coconut Oil	1
60	El Salvador	2011	Coconuts - Incl Copra	19
60	El Salvador	2011	Coffee	8
60	El Salvador	2011	Crustaceans	2
60	El Salvador	2011	Eggs	22
60	El Salvador	2011	Fats, Animals, Raw	8
60	El Salvador	2011	Freshwater Fish	3
60	El Salvador	2011	Fruits, Other	73
60	El Salvador	2011	Grapes	3
60	El Salvador	2011	Groundnuts (Shelled Eq)	1
60	El Salvador	2011	Lemons, Limes	9
60	El Salvador	2011	Maize	199
60	El Salvador	2011	Marine Fish, Other	7
60	El Salvador	2011	Milk, Whole	191
60	El Salvador	2011	Nuts	2
60	El Salvador	2011	Oats	1
60	El Salvador	2011	Offals, Edible	3
60	El Salvador	2011	Oilcrops Oil, Other	3
60	El Salvador	2011	Olive Oil	1
60	El Salvador	2011	Onions	12
60	El Salvador	2011	Oranges, Mandarines	46
60	El Salvador	2011	Pelagic Fish	6
60	El Salvador	2011	Pigmeat	7
60	El Salvador	2011	Pineapples	2
60	El Salvador	2011	Plantains	26
60	El Salvador	2011	Potatoes	35
60	El Salvador	2011	Poultry Meat	50
60	El Salvador	2011	Rice (Milled Equivalent)	28
60	El Salvador	2011	Roots, Other	17
60	El Salvador	2011	Sesameseed	1
60	El Salvador	2011	Sorghum	45
60	El Salvador	2011	Soyabean Oil	8
60	El Salvador	2011	Soyabeans	2
60	El Salvador	2011	Sugar, Refined Equivalent	100
60	El Salvador	2011	Sunflowerseed Oil	3
60	El Salvador	2011	Sweeteners, Other	3
60	El Salvador	2011	Tomatoes	45
60	El Salvador	2011	Vegetables, Other	118
60	El Salvador	2011	Wheat	77
60	El Salvador	2011	Wine	1
63	Estonia	1992	Apples	36
63	Estonia	1992	Bananas	3
63	Estonia	1992	Barley	17
63	Estonia	1992	Beer	80
63	Estonia	1992	Beverages, Alcoholic	30
63	Estonia	1992	Beverages, Fermented	1
63	Estonia	1992	Bovine Meat	68
63	Estonia	1992	Butter, Ghee	14
63	Estonia	1992	Cephalopods	10
63	Estonia	1992	Cereals, Other	1
63	Estonia	1992	Cheese	28
63	Estonia	1992	Coffee	2
63	Estonia	1992	Cream	6
63	Estonia	1992	Crustaceans	1
63	Estonia	1992	Demersal Fish	14
63	Estonia	1992	Eggs	42
63	Estonia	1992	Fats, Animals, Raw	4
63	Estonia	1992	Freshwater Fish	10
63	Estonia	1992	Fruits, Other	19
63	Estonia	1992	Grapes	1
63	Estonia	1992	Honey	1
63	Estonia	1992	Marine Fish, Other	4
63	Estonia	1992	Meat, Other	1
63	Estonia	1992	Milk, Whole	576
63	Estonia	1992	Mutton & Goat Meat	3
63	Estonia	1992	Oats	6
63	Estonia	1992	Offals, Edible	17
63	Estonia	1992	Oilcrops Oil, Other	1
63	Estonia	1992	Onions	4
63	Estonia	1992	Oranges, Mandarines	17
63	Estonia	1992	Peas	1
63	Estonia	1992	Pelagic Fish	31
63	Estonia	1992	Pigmeat	77
63	Estonia	1992	Potatoes	295
63	Estonia	1992	Poultry Meat	18
63	Estonia	1992	Rye	83
63	Estonia	1992	Sugar, Refined Equivalent	47
63	Estonia	1992	Sunflowerseed Oil	5
63	Estonia	1992	Sweeteners, Other	1
63	Estonia	1992	Tomatoes	14
63	Estonia	1992	Vegetables, Other	150
63	Estonia	1992	Wheat	193
63	Estonia	1992	Wine	1
63	Estonia	1993	Apples	72
63	Estonia	1993	Bananas	4
63	Estonia	1993	Barley	17
63	Estonia	1993	Beer	80
63	Estonia	1993	Beverages, Alcoholic	32
63	Estonia	1993	Beverages, Fermented	1
63	Estonia	1993	Bovine Meat	71
63	Estonia	1993	Butter, Ghee	15
63	Estonia	1993	Cephalopods	2
63	Estonia	1993	Cereals, Other	3
63	Estonia	1993	Cheese	27

FAO country code	Country	Reference year	Food item	Gross food supply (g $capita^{-1}$ d^{-1})
63	Estonia	1993	Citrus, Other	7
63	Estonia	1993	Cocoa Beans	1
63	Estonia	1993	Coffee	5
63	Estonia	1993	Cream	7
63	Estonia	1993	Crustaceans	2
63	Estonia	1993	Demersal Fish	13
63	Estonia	1993	Eggs	37
63	Estonia	1993	Fats, Animals, Raw	5
63	Estonia	1993	Freshwater Fish	7
63	Estonia	1993	Fruits, Other	19
63	Estonia	1993	Grapes	4
63	Estonia	1993	Honey	1
63	Estonia	1993	Lemons, Limes	1
63	Estonia	1993	Marine Fish, Other	4
63	Estonia	1993	Meat, Other	2
63	Estonia	1993	Milk, Whole	542
63	Estonia	1993	Mutton & Goat Meat	2
63	Estonia	1993	Nuts	1
63	Estonia	1993	Oats	18
63	Estonia	1993	Offals, Edible	13
63	Estonia	1993	Oilcrops Oil, Other	1
63	Estonia	1993	Onions	5
63	Estonia	1993	Oranges, Mandarines	20
63	Estonia	1993	Peas	2
63	Estonia	1993	Pelagic Fish	48
63	Estonia	1993	Pigmeat	55
63	Estonia	1993	Potatoes	316
63	Estonia	1993	Poultry Meat	10
63	Estonia	1993	Rice (Milled Equivalent)	3
63	Estonia	1993	Rye	68
63	Estonia	1993	Soyabean Oil	1
63	Estonia	1993	Sugar, Refined Equivalent	52
63	Estonia	1993	Sunflowerseed Oil	8
63	Estonia	1993	Tea	1
63	Estonia	1993	Tomatoes	15
63	Estonia	1993	Vegetables, Other	115
63	Estonia	1993	Wheat	199
63	Estonia	1993	Wine	4
63	Estonia	1994	Apples	28
63	Estonia	1994	Bananas	8
63	Estonia	1994	Barley	17
63	Estonia	1994	Beer	95
63	Estonia	1994	Beverages, Alcoholic	31
63	Estonia	1994	Beverages, Fermented	1
63	Estonia	1994	Bovine Meat	57
63	Estonia	1994	Butter, Ghee	15
63	Estonia	1994	Cereals, Other	3
63	Estonia	1994	Cheese	32
63	Estonia	1994	Citrus, Other	4
63	Estonia	1994	Cocoa Beans	1
63	Estonia	1994	Coffee	7
63	Estonia	1994	Cream	7
63	Estonia	1994	Crustaceans	3
63	Estonia	1994	Demersal Fish	19
63	Estonia	1994	Eggs	39
63	Estonia	1994	Fats, Animals, Raw	4
63	Estonia	1994	Freshwater Fish	4
63	Estonia	1994	Fruits, Other	18
63	Estonia	1994	Grapefruit	1
63	Estonia	1994	Grapes	6
63	Estonia	1994	Groundnuts (Shelled Eq)	1
63	Estonia	1994	Honey	1
63	Estonia	1994	Lemons, Limes	1
63	Estonia	1994	Marine Fish, Other	4
63	Estonia	1994	Meat, Other	2
63	Estonia	1994	Milk, Whole	614
63	Estonia	1994	Mutton & Goat Meat	2
63	Estonia	1994	Nuts	2
63	Estonia	1994	Oats	35
63	Estonia	1994	Offals, Edible	12
63	Estonia	1994	Oilcrops Oil, Other	3
63	Estonia	1994	Onions	8
63	Estonia	1994	Oranges, Mandarines	11
63	Estonia	1994	Peas	2
63	Estonia	1994	Pelagic Fish	18
63	Estonia	1994	Pigmeat	65
63	Estonia	1994	Potatoes	330
63	Estonia	1994	Poultry Meat	18
63	Estonia	1994	Rice (Milled Equivalent)	2
63	Estonia	1994	Rye	89
63	Estonia	1994	Soyabean Oil	1
63	Estonia	1994	Sugar, Refined Equivalent	69
63	Estonia	1994	Sunflowerseed Oil	14
63	Estonia	1994	Sweeteners, Other	2
63	Estonia	1994	Tea	1
63	Estonia	1994	Tomatoes	12
63	Estonia	1994	Vegetables, Other	152
63	Estonia	1994	Wheat	182
63	Estonia	1994	Wine	4
63	Estonia	1995	Apples	52
63	Estonia	1995	Bananas	12
63	Estonia	1995	Barley	41
63	Estonia	1995	Beer	100
63	Estonia	1995	Beverages, Alcoholic	29
63	Estonia	1995	Beverages, Fermented	1
63	Estonia	1995	Bovine Meat	46
63	Estonia	1995	Butter, Ghee	15

FAO country code	Country	Reference year	Food item	Gross food supply (g *capita*$^{-1}$ d^{-1})
63	Estonia	1995	Cereals, Other	9
63	Estonia	1995	Cheese	25
63	Estonia	1995	Citrus, Other	12
63	Estonia	1995	Cocoa Beans	4
63	Estonia	1995	Coffee	10
63	Estonia	1995	Cream	6
63	Estonia	1995	Crustaceans	7
63	Estonia	1995	Demersal Fish	32
63	Estonia	1995	Eggs	37
63	Estonia	1995	Fats, Animals, Raw	4
63	Estonia	1995	Freshwater Fish	7
63	Estonia	1995	Fruits, Other	40
63	Estonia	1995	Grapefruit	1
63	Estonia	1995	Grapes	9
63	Estonia	1995	Groundnuts (Shelled Eq)	1
63	Estonia	1995	Honey	1
63	Estonia	1995	Lemons, Limes	1
63	Estonia	1995	Maize	21
63	Estonia	1995	Meat, Other	1
63	Estonia	1995	Milk, Whole	555
63	Estonia	1995	Mutton & Goat Meat	2
63	Estonia	1995	Nuts	3
63	Estonia	1995	Oats	46
63	Estonia	1995	Offals, Edible	12
63	Estonia	1995	Oilcrops Oil, Other	2
63	Estonia	1995	Onions	10
63	Estonia	1995	Oranges, Mandarines	26
63	Estonia	1995	Peas	8
63	Estonia	1995	Pelagic Fish	11
63	Estonia	1995	Pigmeat	67
63	Estonia	1995	Pineapples	1
63	Estonia	1995	Potatoes	384
63	Estonia	1995	Poultry Meat	20
63	Estonia	1995	Pulses, Other	1
63	Estonia	1995	Rice (Milled Equivalent)	4
63	Estonia	1995	Rye	59
63	Estonia	1995	Soyabean Oil	2
63	Estonia	1995	Spices, Other	1
63	Estonia	1995	Sugar, Refined Equivalent	60
63	Estonia	1995	Sunflowerseed Oil	15
63	Estonia	1995	Sweeteners, Other	2
63	Estonia	1995	Tea	1
63	Estonia	1995	Tomatoes	18
63	Estonia	1995	Vegetables, Other	118
63	Estonia	1995	Wheat	211
63	Estonia	1995	Wine	9
63	Estonia	1996	Apples	47
63	Estonia	1996	Bananas	11
63	Estonia	1996	Barley	55
63	Estonia	1996	Beer	98
63	Estonia	1996	Beverages, Alcoholic	26
63	Estonia	1996	Beverages, Fermented	2
63	Estonia	1996	Bovine Meat	50
63	Estonia	1996	Butter, Ghee	14
63	Estonia	1996	Cephalopods	5
63	Estonia	1996	Cereals, Other	17
63	Estonia	1996	Cheese	20
63	Estonia	1996	Citrus, Other	8
63	Estonia	1996	Cocoa Beans	15
63	Estonia	1996	Coffee	10
63	Estonia	1996	Cream	4
63	Estonia	1996	Crustaceans	7
63	Estonia	1996	Demersal Fish	20
63	Estonia	1996	Eggs	33
63	Estonia	1996	Fats, Animals, Raw	5
63	Estonia	1996	Freshwater Fish	9
63	Estonia	1996	Fruits, Other	45
63	Estonia	1996	Grapefruit	1
63	Estonia	1996	Grapes	12
63	Estonia	1996	Groundnuts (Shelled Eq)	1
63	Estonia	1996	Honey	1
63	Estonia	1996	Lemons, Limes	1
63	Estonia	1996	Maize	9
63	Estonia	1996	Meat, Other	2
63	Estonia	1996	Milk, Whole	451
63	Estonia	1996	Molluscs, Other	1
63	Estonia	1996	Mutton & Goat Meat	1
63	Estonia	1996	Nuts	5
63	Estonia	1996	Oats	23
63	Estonia	1996	Offals, Edible	12
63	Estonia	1996	Oilcrops Oil, Other	6
63	Estonia	1996	Onions	11
63	Estonia	1996	Oranges, Mandarines	20
63	Estonia	1996	Peas	6
63	Estonia	1996	Pelagic Fish	8
63	Estonia	1996	Pigmeat	72
63	Estonia	1996	Pineapples	1
63	Estonia	1996	Potatoes	416
63	Estonia	1996	Poultry Meat	33
63	Estonia	1996	Rice (Milled Equivalent)	8
63	Estonia	1996	Rye	60
63	Estonia	1996	Soyabean Oil	2
63	Estonia	1996	Spices, Other	1
63	Estonia	1996	Sugar, Refined Equivalent	82
63	Estonia	1996	Sunflowerseed Oil	13
63	Estonia	1996	Sweeteners, Other	3
63	Estonia	1996	Tea	1

FAO country code	Country	Reference year	Food item	Gross food supply (g *capita*$^{-1}$ d^{-1})
63	Estonia	1996	Tomatoes	17
63	Estonia	1996	Vegetables, Other	141
63	Estonia	1996	Wheat	224
63	Estonia	1996	Wine	12
63	Estonia	1997	Apples	55
63	Estonia	1997	Bananas	21
63	Estonia	1997	Barley	48
63	Estonia	1997	Beer	120
63	Estonia	1997	Beverages, Alcoholic	25
63	Estonia	1997	Beverages, Fermented	3
63	Estonia	1997	Bovine Meat	40
63	Estonia	1997	Butter, Ghee	12
63	Estonia	1997	Cereals, Other	29
63	Estonia	1997	Cheese	16
63	Estonia	1997	Citrus, Other	8
63	Estonia	1997	Cocoa Beans	10
63	Estonia	1997	Coconuts - Incl Copra	1
63	Estonia	1997	Coffee	9
63	Estonia	1997	Cottonseed Oil	2
63	Estonia	1997	Cream	5
63	Estonia	1997	Crustaceans	10
63	Estonia	1997	Demersal Fish	27
63	Estonia	1997	Eggs	31
63	Estonia	1997	Fats, Animals, Raw	4
63	Estonia	1997	Freshwater Fish	9
63	Estonia	1997	Fruits, Other	51
63	Estonia	1997	Grapefruit	2
63	Estonia	1997	Grapes	13
63	Estonia	1997	Honey	1
63	Estonia	1997	Lemons, Limes	2
63	Estonia	1997	Maize	2
63	Estonia	1997	Meat, Other	2
63	Estonia	1997	Milk, Whole	407
63	Estonia	1997	Molluscs, Other	1
63	Estonia	1997	Mutton & Goat Meat	1
63	Estonia	1997	Nuts	5
63	Estonia	1997	Oats	21
63	Estonia	1997	Offals, Edible	10
63	Estonia	1997	Oilcrops Oil, Other	14
63	Estonia	1997	Onions	13
63	Estonia	1997	Oranges, Mandarines	33
63	Estonia	1997	Peas	8
63	Estonia	1997	Pelagic Fish	6
63	Estonia	1997	Pigmeat	60
63	Estonia	1997	Pineapples	1
63	Estonia	1997	Potatoes	344
63	Estonia	1997	Poultry Meat	46
63	Estonia	1997	Rice (Milled Equivalent)	6
63	Estonia	1997	Rye	62
63	Estonia	1997	Soyabean Oil	4
63	Estonia	1997	Sugar, Refined Equivalent	89
63	Estonia	1997	Sunflowerseed Oil	8
63	Estonia	1997	Sweeteners, Other	6
63	Estonia	1997	Tea	1
63	Estonia	1997	Tomatoes	20
63	Estonia	1997	Vegetables, Other	148
63	Estonia	1997	Wheat	215
63	Estonia	1997	Wine	16
63	Estonia	1998	Apples	38
63	Estonia	1998	Bananas	24
63	Estonia	1998	Barley	43
63	Estonia	1998	Beer	141
63	Estonia	1998	Beverages, Alcoholic	19
63	Estonia	1998	Beverages, Fermented	6
63	Estonia	1998	Bovine Meat	45
63	Estonia	1998	Butter, Ghee	16
63	Estonia	1998	Cereals, Other	27
63	Estonia	1998	Cheese	11
63	Estonia	1998	Citrus, Other	3
63	Estonia	1998	Cocoa Beans	16
63	Estonia	1998	Coffee	9
63	Estonia	1998	Cottonseed Oil	2
63	Estonia	1998	Cream	5
63	Estonia	1998	Crustaceans	10
63	Estonia	1998	Demersal Fish	22
63	Estonia	1998	Eggs	35
63	Estonia	1998	Fats, Animals, Raw	3
63	Estonia	1998	Freshwater Fish	11
63	Estonia	1998	Fruits, Other	52
63	Estonia	1998	Grapefruit	4
63	Estonia	1998	Grapes	18
63	Estonia	1998	Honey	1
63	Estonia	1998	Lemons, Limes	2
63	Estonia	1998	Maize	15
63	Estonia	1998	Marine Fish, Other	1
63	Estonia	1998	Meat, Other	1
63	Estonia	1998	Milk, Whole	395
63	Estonia	1998	Mutton & Goat Meat	1
63	Estonia	1998	Nuts	6
63	Estonia	1998	Oats	21
63	Estonia	1998	Offals, Edible	12
63	Estonia	1998	Oilcrops Oil, Other	15
63	Estonia	1998	Onions	14
63	Estonia	1998	Oranges, Mandarines	55
63	Estonia	1998	Peas	6
63	Estonia	1998	Pelagic Fish	2
63	Estonia	1998	Pigmeat	65

FAO country code	Country	Reference year	Food item	Gross food supply (g $capita^{-1}$ d^{-1})
63	Estonia	1998	Pineapples	2
63	Estonia	1998	Potatoes	255
63	Estonia	1998	Poultry Meat	57
63	Estonia	1998	Rice (Milled Equivalent)	6
63	Estonia	1998	Rye	76
63	Estonia	1998	Soyabean Oil	5
63	Estonia	1998	Sugar, Refined Equivalent	86
63	Estonia	1998	Sunflowerseed Oil	1
63	Estonia	1998	Sweeteners, Other	7
63	Estonia	1998	Tea	1
63	Estonia	1998	Tomatoes	24
63	Estonia	1998	Vegetables, Other	148
63	Estonia	1998	Wheat	202
63	Estonia	1998	Wine	16
63	Estonia	1999	Apples	62
63	Estonia	1999	Bananas	26
63	Estonia	1999	Barley	19
63	Estonia	1999	Beans	1
63	Estonia	1999	Beer	175
63	Estonia	1999	Beverages, Alcoholic	19
63	Estonia	1999	Beverages, Fermented	15
63	Estonia	1999	Bovine Meat	41
63	Estonia	1999	Butter, Ghee	16
63	Estonia	1999	Cereals, Other	20
63	Estonia	1999	Cheese	36
63	Estonia	1999	Citrus, Other	2
63	Estonia	1999	Cocoa Beans	17
63	Estonia	1999	Coconuts - Incl Copra	1
63	Estonia	1999	Coffee	12
63	Estonia	1999	Cottonseed Oil	2
63	Estonia	1999	Cream	4
63	Estonia	1999	Crustaceans	25
63	Estonia	1999	Demersal Fish	5
63	Estonia	1999	Eggs	31
63	Estonia	1999	Fats, Animals, Raw	4
63	Estonia	1999	Freshwater Fish	10
63	Estonia	1999	Fruits, Other	50
63	Estonia	1999	Grapefruit	2
63	Estonia	1999	Grapes	18
63	Estonia	1999	Honey	1
63	Estonia	1999	Lemons, Limes	2
63	Estonia	1999	Maize	6
63	Estonia	1999	Milk, Whole	434
63	Estonia	1999	Mutton & Goat Meat	1
63	Estonia	1999	Nuts	2
63	Estonia	1999	Oats	9
63	Estonia	1999	Offals, Edible	11
63	Estonia	1999	Oilcrops Oil, Other	13
63	Estonia	1999	Onions	16
63	Estonia	1999	Oranges, Mandarines	36
63	Estonia	1999	Peas	1
63	Estonia	1999	Pelagic Fish	4
63	Estonia	1999	Pigmeat	83
63	Estonia	1999	Pineapples	2
63	Estonia	1999	Potatoes	377
63	Estonia	1999	Poultry Meat	37
63	Estonia	1999	Rice (Milled Equivalent)	7
63	Estonia	1999	Rye	83
63	Estonia	1999	Soyabean Oil	3
63	Estonia	1999	Spices, Other	1
63	Estonia	1999	Sugar, Refined Equivalent	117
63	Estonia	1999	Sunflowerseed Oil	1
63	Estonia	1999	Sweeteners, Other	10
63	Estonia	1999	Tea	1
63	Estonia	1999	Tomatoes	29
63	Estonia	1999	Vegetables, Other	149
63	Estonia	1999	Wheat	178
63	Estonia	1999	Wine	15
63	Estonia	2000	Apples	75
63	Estonia	2000	Bananas	22
63	Estonia	2000	Barley	22
63	Estonia	2000	Beer	178
63	Estonia	2000	Beverages, Alcoholic	19
63	Estonia	2000	Beverages, Fermented	17
63	Estonia	2000	Bovine Meat	37
63	Estonia	2000	Butter, Ghee	10
63	Estonia	2000	Cereals, Other	14
63	Estonia	2000	Cheese	27
63	Estonia	2000	Citrus, Other	5
63	Estonia	2000	Cocoa Beans	11
63	Estonia	2000	Coffee	12
63	Estonia	2000	Cream	3
63	Estonia	2000	Crustaceans	7
63	Estonia	2000	Demersal Fish	16
63	Estonia	2000	Eggs	33
63	Estonia	2000	Fats, Animals, Raw	5
63	Estonia	2000	Freshwater Fish	10
63	Estonia	2000	Fruits, Other	57
63	Estonia	2000	Grapefruit	2
63	Estonia	2000	Grapes	19
63	Estonia	2000	Honey	1
63	Estonia	2000	Lemons, Limes	3
63	Estonia	2000	Maize	19
63	Estonia	2000	Milk, Whole	313
63	Estonia	2000	Mutton & Goat Meat	1
63	Estonia	2000	Nuts	3
63	Estonia	2000	Oats	7

FAO country code	Country	Reference year	Food item	Gross food supply (g $capita^{-1}$ d^{-1})
63	Estonia	2000	Offals, Edible	8
63	Estonia	2000	Oilcrops Oil, Other	13
63	Estonia	2000	Onions	15
63	Estonia	2000	Oranges, Mandarines	41
63	Estonia	2000	Peas	4
63	Estonia	2000	Pelagic Fish	16
63	Estonia	2000	Pigmeat	76
63	Estonia	2000	Pineapples	5
63	Estonia	2000	Potatoes	389
63	Estonia	2000	Poultry Meat	49
63	Estonia	2000	Rice (Milled Equivalent)	6
63	Estonia	2000	Rye	90
63	Estonia	2000	Soyabean Oil	5
63	Estonia	2000	Sugar, Refined Equivalent	133
63	Estonia	2000	Sunflowerseed Oil	1
63	Estonia	2000	Sweeteners, Other	5
63	Estonia	2000	Tea	2
63	Estonia	2000	Tomatoes	18
63	Estonia	2000	Vegetables, Other	155
63	Estonia	2000	Wheat	182
63	Estonia	2000	Wine	16
63	Estonia	2001	Apples	65
63	Estonia	2001	Bananas	20
63	Estonia	2001	Barley	20
63	Estonia	2001	Beans	2
63	Estonia	2001	Beer	190
63	Estonia	2001	Beverages, Alcoholic	24
63	Estonia	2001	Beverages, Fermented	16
63	Estonia	2001	Bovine Meat	34
63	Estonia	2001	Butter, Ghee	7
63	Estonia	2001	Cephalopods	4
63	Estonia	2001	Cereals, Other	11
63	Estonia	2001	Cheese	28
63	Estonia	2001	Citrus, Other	3
63	Estonia	2001	Cocoa Beans	14
63	Estonia	2001	Coffee	13
63	Estonia	2001	Cream	3
63	Estonia	2001	Crustaceans	21
63	Estonia	2001	Demersal Fish	9
63	Estonia	2001	Eggs	35
63	Estonia	2001	Fats, Animals, Raw	3
63	Estonia	2001	Freshwater Fish	10
63	Estonia	2001	Fruits, Other	63
63	Estonia	2001	Grapefruit	2
63	Estonia	2001	Grapes	18
63	Estonia	2001	Honey	1
63	Estonia	2001	Lemons, Limes	3
63	Estonia	2001	Maize	8
63	Estonia	2001	Milk, Whole	394
63	Estonia	2001	Mutton & Goat Meat	1
63	Estonia	2001	Nuts	3
63	Estonia	2001	Oats	6
63	Estonia	2001	Offals, Edible	8
63	Estonia	2001	Oilcrops Oil, Other	14
63	Estonia	2001	Onions	19
63	Estonia	2001	Oranges, Mandarines	40
63	Estonia	2001	Peas	2
63	Estonia	2001	Pelagic Fish	2
63	Estonia	2001	Pigmeat	78
63	Estonia	2001	Pineapples	5
63	Estonia	2001	Potatoes	361
63	Estonia	2001	Poultry Meat	54
63	Estonia	2001	Rice (Milled Equivalent)	7
63	Estonia	2001	Rye	90
63	Estonia	2001	Soyabean Oil	8
63	Estonia	2001	Sugar, Refined Equivalent	125
63	Estonia	2001	Sunflowerseed Oil	1
63	Estonia	2001	Sweeteners, Other	14
63	Estonia	2001	Tea	1
63	Estonia	2001	Tomatoes	34
63	Estonia	2001	Vegetables, Other	163
63	Estonia	2001	Wheat	192
63	Estonia	2001	Wine	14
63	Estonia	2002	Apples	64
63	Estonia	2002	Bananas	19
63	Estonia	2002	Barley	28
63	Estonia	2002	Beer	208
63	Estonia	2002	Beverages, Alcoholic	29
63	Estonia	2002	Beverages, Fermented	16
63	Estonia	2002	Bovine Meat	37
63	Estonia	2002	Butter, Ghee	10
63	Estonia	2002	Cephalopods	1
63	Estonia	2002	Cereals, Other	12
63	Estonia	2002	Cheese	46
63	Estonia	2002	Citrus, Other	1
63	Estonia	2002	Cocoa Beans	12
63	Estonia	2002	Coffee	15
63	Estonia	2002	Cream	4
63	Estonia	2002	Crustaceans	27
63	Estonia	2002	Demersal Fish	4
63	Estonia	2002	Eggs	31
63	Estonia	2002	Fats, Animals, Raw	3
63	Estonia	2002	Freshwater Fish	16
63	Estonia	2002	Fruits, Other	52
63	Estonia	2002	Grapefruit	1
63	Estonia	2002	Grapes	16
63	Estonia	2002	Honey	2

FAO country code	Country	Reference year	Food item	Gross food supply (g *capita*$^{-1}$ d^{-1})
63	Estonia	2002	Lemons, Limes	2
63	Estonia	2002	Maize	11
63	Estonia	2002	Milk, Whole	308
63	Estonia	2002	Molluscs, Other	1
63	Estonia	2002	Mutton & Goat Meat	1
63	Estonia	2002	Nuts	3
63	Estonia	2002	Oats	7
63	Estonia	2002	Offals, Edible	10
63	Estonia	2002	Oilcrops Oil, Other	12
63	Estonia	2002	Onions	17
63	Estonia	2002	Oranges, Mandarines	36
63	Estonia	2002	Peas	3
63	Estonia	2002	Pelagic Fish	1
63	Estonia	2002	Pigmeat	80
63	Estonia	2002	Pineapples	5
63	Estonia	2002	Potatoes	310
63	Estonia	2002	Poultry Meat	59
63	Estonia	2002	Rice (Milled Equivalent)	7
63	Estonia	2002	Rye	86
63	Estonia	2002	Soyabean Oil	9
63	Estonia	2002	Sugar, Refined Equivalent	99
63	Estonia	2002	Sunflowerseed Oil	1
63	Estonia	2002	Sweeteners, Other	9
63	Estonia	2002	Tea	1
63	Estonia	2002	Tomatoes	34
63	Estonia	2002	Vegetables, Other	163
63	Estonia	2002	Wheat	183
63	Estonia	2002	Wine	17
63	Estonia	2003	Apples	63
63	Estonia	2003	Bananas	25
63	Estonia	2003	Barley	28
63	Estonia	2003	Beans	1
63	Estonia	2003	Beer	202
63	Estonia	2003	Beverages, Alcoholic	34
63	Estonia	2003	Beverages, Fermented	11
63	Estonia	2003	Bovine Meat	29
63	Estonia	2003	Butter, Ghee	15
63	Estonia	2003	Cereals, Other	16
63	Estonia	2003	Cheese	40
63	Estonia	2003	Citrus, Other	1
63	Estonia	2003	Cocoa Beans	14
63	Estonia	2003	Coconuts - Incl Copra	1
63	Estonia	2003	Coffee	16
63	Estonia	2003	Cream	5
63	Estonia	2003	Crustaceans	25
63	Estonia	2003	Demersal Fish	3
63	Estonia	2003	Eggs	29
63	Estonia	2003	Fats, Animals, Raw	3
63	Estonia	2003	Freshwater Fish	11
63	Estonia	2003	Fruits, Other	42
63	Estonia	2003	Grapefruit	1
63	Estonia	2003	Grapes	22
63	Estonia	2003	Honey	1
63	Estonia	2003	Lemons, Limes	3
63	Estonia	2003	Maize	11
63	Estonia	2003	Milk, Whole	306
63	Estonia	2003	Mutton & Goat Meat	1
63	Estonia	2003	Nuts	5
63	Estonia	2003	Oats	7
63	Estonia	2003	Offals, Edible	10
63	Estonia	2003	Oilcrops Oil, Other	12
63	Estonia	2003	Olives	1
63	Estonia	2003	Onions	18
63	Estonia	2003	Oranges, Mandarines	36
63	Estonia	2003	Peas	2
63	Estonia	2003	Pelagic Fish	7
63	Estonia	2003	Pigmeat	80
63	Estonia	2003	Pineapples	3
63	Estonia	2003	Potatoes	327
63	Estonia	2003	Poultry Meat	58
63	Estonia	2003	Rice (Milled Equivalent)	8
63	Estonia	2003	Rye	80
63	Estonia	2003	Soyabean Oil	9
63	Estonia	2003	Spices, Other	1
63	Estonia	2003	Sugar, Refined Equivalent	101
63	Estonia	2003	Sunflowerseed Oil	2
63	Estonia	2003	Sweeteners, Other	9
63	Estonia	2003	Tea	1
63	Estonia	2003	Tomatoes	38
63	Estonia	2003	Vegetables, Other	215
63	Estonia	2003	Wheat	186
63	Estonia	2003	Wine	18
63	Estonia	2004	Apples	59
63	Estonia	2004	Bananas	28
63	Estonia	2004	Barley	22
63	Estonia	2004	Beer	255
63	Estonia	2004	Beverages, Alcoholic	42
63	Estonia	2004	Beverages, Fermented	15
63	Estonia	2004	Bovine Meat	34
63	Estonia	2004	Butter, Ghee	16
63	Estonia	2004	Cephalopods	1
63	Estonia	2004	Cereals, Other	10
63	Estonia	2004	Cheese	22
63	Estonia	2004	Citrus, Other	2
63	Estonia	2004	Cocoa Beans	23
63	Estonia	2004	Coffee	16
63	Estonia	2004	Cream	3

FAO countrycode	Country	Reference year	Food item	Gross food supply (g *capita*$^{-1}$ d^{-1})
63	Estonia	2004	Crustaceans	22
63	Estonia	2004	Demersal Fish	5
63	Estonia	2004	Eggs	28
63	Estonia	2004	Fats, Animals, Raw	4
63	Estonia	2004	Freshwater Fish	5
63	Estonia	2004	Fruits, Other	52
63	Estonia	2004	Grapefruit	2
63	Estonia	2004	Grapes	24
63	Estonia	2004	Groundnuts (Shelled Eq)	1
63	Estonia	2004	Honey	2
63	Estonia	2004	Lemons, Limes	3
63	Estonia	2004	Maize	17
63	Estonia	2004	Maize Germ Oil	1
63	Estonia	2004	Marine Fish, Other	1
63	Estonia	2004	Milk, Whole	314
63	Estonia	2004	Mutton & Goat Meat	1
63	Estonia	2004	Nuts	5
63	Estonia	2004	Oats	6
63	Estonia	2004	Offals, Edible	14
63	Estonia	2004	Oilcrops Oil, Other	12
63	Estonia	2004	Onions	17
63	Estonia	2004	Oranges, Mandarines	35
63	Estonia	2004	Peas	2
63	Estonia	2004	Pelagic Fish	4
63	Estonia	2004	Pigmeat	72
63	Estonia	2004	Pineapples	3
63	Estonia	2004	Potatoes	201
63	Estonia	2004	Poultry Meat	43
63	Estonia	2004	Pulses, Other	5
63	Estonia	2004	Rice (Milled Equivalent)	8
63	Estonia	2004	Rye	44
63	Estonia	2004	Soyabean Oil	9
63	Estonia	2004	Spices, Other	1
63	Estonia	2004	Sugar, Refined Equivalent	123
63	Estonia	2004	Sunflowerseed Oil	1
63	Estonia	2004	Sweeteners, Other	9
63	Estonia	2004	Tea	1
63	Estonia	2004	Tomatoes	41
63	Estonia	2004	Vegetables, Other	170
63	Estonia	2004	Wheat	216
63	Estonia	2004	Wine	22
63	Estonia	2005	Apples	59
63	Estonia	2005	Bananas	19
63	Estonia	2005	Barley	27
63	Estonia	2005	Beer	258
63	Estonia	2005	Beverages, Alcoholic	37
63	Estonia	2005	Beverages, Fermented	2
63	Estonia	2005	Bovine Meat	35
63	Estonia	2005	Butter, Ghee	10
63	Estonia	2005	Cephalopods	1
63	Estonia	2005	Cereals, Other	7
63	Estonia	2005	Cheese	20
63	Estonia	2005	Citrus, Other	1
63	Estonia	2005	Cocoa Beans	20
63	Estonia	2005	Coconuts - Incl Copra	1
63	Estonia	2005	Coffee	14
63	Estonia	2005	Cream	3
63	Estonia	2005	Crustaceans	22
63	Estonia	2005	Demersal Fish	6
63	Estonia	2005	Eggs	29
63	Estonia	2005	Fats, Animals, Raw	4
63	Estonia	2005	Freshwater Fish	7
63	Estonia	2005	Fruits, Other	52
63	Estonia	2005	Grapefruit	2
63	Estonia	2005	Grapes	15
63	Estonia	2005	Groundnuts (Shelled Eq)	1
63	Estonia	2005	Honey	2
63	Estonia	2005	Lemons, Limes	2
63	Estonia	2005	Maize	17
63	Estonia	2005	Maize Germ Oil	1
63	Estonia	2005	Meat, Other	13
63	Estonia	2005	Milk, Whole	326
63	Estonia	2005	Molluscs, Other	1
63	Estonia	2005	Mutton & Goat Meat	1
63	Estonia	2005	Nuts	5
63	Estonia	2005	Oats	7
63	Estonia	2005	Offals, Edible	12
63	Estonia	2005	Oilcrops Oil, Other	12
63	Estonia	2005	Onions	12
63	Estonia	2005	Oranges, Mandarines	39
63	Estonia	2005	Peas	1
63	Estonia	2005	Pelagic Fish	4
63	Estonia	2005	Pigmeat	72
63	Estonia	2005	Pineapples	5
63	Estonia	2005	Potatoes	328
63	Estonia	2005	Poultry Meat	45
63	Estonia	2005	Pulses, Other	5
63	Estonia	2005	Rice (Milled Equivalent)	5
63	Estonia	2005	Rye	52
63	Estonia	2005	Soyabean Oil	9
63	Estonia	2005	Spices, Other	1
63	Estonia	2005	Sugar, Refined Equivalent	122
63	Estonia	2005	Sunflowerseed Oil	1
63	Estonia	2005	Sweeteners, Other	10
63	Estonia	2005	Tea	1
63	Estonia	2005	Tomatoes	38
63	Estonia	2005	Vegetables, Other	179

FAO country code	Country	Reference year	Food item	Gross food supply (g $capita^{-1}$ d^{-1})
63	Estonia	2005	Wheat	232
63	Estonia	2005	Wine	24
63	Estonia	2006	Apples	24
63	Estonia	2006	Bananas	21
63	Estonia	2006	Barley	7
63	Estonia	2006	Beer	282
63	Estonia	2006	Beverages, Alcoholic	45
63	Estonia	2006	Beverages, Fermented	1
63	Estonia	2006	Bovine Meat	38
63	Estonia	2006	Butter, Ghee	10
63	Estonia	2006	Cephalopods	2
63	Estonia	2006	Cereals, Other	2
63	Estonia	2006	Cheese	24
63	Estonia	2006	Citrus, Other	1
63	Estonia	2006	Cocoa Beans	20
63	Estonia	2006	Coconuts - Incl Copra	1
63	Estonia	2006	Coffee	14
63	Estonia	2006	Cream	2
63	Estonia	2006	Crustaceans	9
63	Estonia	2006	Demersal Fish	6
63	Estonia	2006	Eggs	28
63	Estonia	2006	Fats, Animals, Raw	3
63	Estonia	2006	Freshwater Fish	8
63	Estonia	2006	Fruits, Other	50
63	Estonia	2006	Grapefruit	3
63	Estonia	2006	Grapes	22
63	Estonia	2006	Groundnuts (Shelled Eq)	1
63	Estonia	2006	Honey	3
63	Estonia	2006	Lemons, Limes	3
63	Estonia	2006	Maize	17
63	Estonia	2006	Maize Germ Oil	1
63	Estonia	2006	Meat, Other	2
63	Estonia	2006	Milk, Whole	325
63	Estonia	2006	Molluscs, Other	1
63	Estonia	2006	Mutton & Goat Meat	1
63	Estonia	2006	Nuts	5
63	Estonia	2006	Oats	4
63	Estonia	2006	Offals, Edible	12
63	Estonia	2006	Oilcrops Oil, Other	14
63	Estonia	2006	Onions	12
63	Estonia	2006	Oranges, Mandarines	49
63	Estonia	2006	Peas	3
63	Estonia	2006	Pelagic Fish	6
63	Estonia	2006	Pepper	1
63	Estonia	2006	Pigmeat	73
63	Estonia	2006	Pineapples	6
63	Estonia	2006	Potatoes	344
63	Estonia	2006	Poultry Meat	45
63	Estonia	2006	Pulses, Other	13
63	Estonia	2006	Rice (Milled Equivalent)	6
63	Estonia	2006	Rye	42
63	Estonia	2006	Soyabean Oil	8
63	Estonia	2006	Spices, Other	1
63	Estonia	2006	Sugar, Refined Equivalent	107
63	Estonia	2006	Sunflowerseed Oil	1
63	Estonia	2006	Sweeteners, Other	12
63	Estonia	2006	Tea	1
63	Estonia	2006	Tomatoes	42
63	Estonia	2006	Vegetables, Other	171
63	Estonia	2006	Wheat	268
63	Estonia	2006	Wine	21
63	Estonia	2007	Apples	41
63	Estonia	2007	Bananas	23
63	Estonia	2007	Barley	7
63	Estonia	2007	Beer	279
63	Estonia	2007	Beverages, Alcoholic	42
63	Estonia	2007	Beverages, Fermented	2
63	Estonia	2007	Bovine Meat	39
63	Estonia	2007	Butter, Ghee	5
63	Estonia	2007	Cephalopods	1
63	Estonia	2007	Cereals, Other	3
63	Estonia	2007	Cheese	19
63	Estonia	2007	Citrus, Other	1
63	Estonia	2007	Cocoa Beans	23
63	Estonia	2007	Coffee	17
63	Estonia	2007	Cream	2
63	Estonia	2007	Crustaceans	9
63	Estonia	2007	Demersal Fish	16
63	Estonia	2007	Eggs	30
63	Estonia	2007	Fats, Animals, Raw	4
63	Estonia	2007	Freshwater Fish	10
63	Estonia	2007	Fruits, Other	66
63	Estonia	2007	Grapefruit	4
63	Estonia	2007	Grapes	21
63	Estonia	2007	Groundnuts (Shelled Eq)	1
63	Estonia	2007	Honey	2
63	Estonia	2007	Lemons, Limes	3
63	Estonia	2007	Maize	17
63	Estonia	2007	Maize Germ Oil	1
63	Estonia	2007	Meat, Other	1
63	Estonia	2007	Milk, Whole	340
63	Estonia	2007	Mutton & Goat Meat	1
63	Estonia	2007	Nuts	8
63	Estonia	2007	Oats	7
63	Estonia	2007	Offals, Edible	14
63	Estonia	2007	Oilcrops Oil, Other	12
63	Estonia	2007	Olive Oil	1

FAO country code	Country	Reference year	Food item	Gross food supply (g $capita^{-1}$ d^{-1})
63	Estonia	2007	Onions	15
63	Estonia	2007	Oranges, Mandarines	52
63	Estonia	2007	Peas	4
63	Estonia	2007	Pelagic Fish	6
63	Estonia	2007	Pepper	1
63	Estonia	2007	Pigmeat	78
63	Estonia	2007	Pineapples	7
63	Estonia	2007	Plantains	2
63	Estonia	2007	Potatoes	318
63	Estonia	2007	Poultry Meat	48
63	Estonia	2007	Pulses, Other	16
63	Estonia	2007	Rice (Milled Equivalent)	6
63	Estonia	2007	Rye	52
63	Estonia	2007	Soyabean Oil	8
63	Estonia	2007	Spices, Other	1
63	Estonia	2007	Sugar, Refined Equivalent	110
63	Estonia	2007	Sweeteners, Other	10
63	Estonia	2007	Tea	1
63	Estonia	2007	Tomatoes	49
63	Estonia	2007	Vegetables, Other	209
63	Estonia	2007	Wheat	254
63	Estonia	2007	Wine	23
63	Estonia	2008	Apples	51
63	Estonia	2008	Bananas	28
63	Estonia	2008	Barley	9
63	Estonia	2008	Beer	258
63	Estonia	2008	Beverages, Alcoholic	54
63	Estonia	2008	Beverages, Fermented	3
63	Estonia	2008	Bovine Meat	39
63	Estonia	2008	Butter, Ghee	6
63	Estonia	2008	Cephalopods	2
63	Estonia	2008	Cheese	18
63	Estonia	2008	Citrus, Other	1
63	Estonia	2008	Cocoa Beans	20
63	Estonia	2008	Coconut Oil	1
63	Estonia	2008	Coconuts - Incl Copra	1
63	Estonia	2008	Coffee	19
63	Estonia	2008	Cream	10
63	Estonia	2008	Crustaceans	7
63	Estonia	2008	Demersal Fish	18
63	Estonia	2008	Eggs	29
63	Estonia	2008	Fats, Animals, Raw	4
63	Estonia	2008	Freshwater Fish	8
63	Estonia	2008	Fruits, Other	56
63	Estonia	2008	Grapefruit	6
63	Estonia	2008	Grapes	23
63	Estonia	2008	Groundnuts (Shelled Eq)	1
63	Estonia	2008	Honey	1
63	Estonia	2008	Lemons, Limes	4
63	Estonia	2008	Maize	17
63	Estonia	2008	Maize Germ Oil	2
63	Estonia	2008	Milk, Whole	496
63	Estonia	2008	Mutton & Goat Meat	2
63	Estonia	2008	Nuts	8
63	Estonia	2008	Oats	5
63	Estonia	2008	Offals, Edible	14
63	Estonia	2008	Oilcrops Oil, Other	12
63	Estonia	2008	Onions	16
63	Estonia	2008	Oranges, Mandarines	53
63	Estonia	2008	Peas	1
63	Estonia	2008	Pelagic Fish	9
63	Estonia	2008	Pepper	1
63	Estonia	2008	Pigmeat	75
63	Estonia	2008	Pimento	1
63	Estonia	2008	Pineapples	6
63	Estonia	2008	Potatoes	234
63	Estonia	2008	Poultry Meat	50
63	Estonia	2008	Pulses, Other	17
63	Estonia	2008	Rice (Milled Equivalent)	8
63	Estonia	2008	Rye	52
63	Estonia	2008	Soyabean Oil	7
63	Estonia	2008	Spices, Other	1
63	Estonia	2008	Sugar, Refined Equivalent	102
63	Estonia	2008	Sweeteners, Other	9
63	Estonia	2008	Tea	1
63	Estonia	2008	Tomatoes	49
63	Estonia	2008	Vegetables, Other	199
63	Estonia	2008	Wheat	259
63	Estonia	2008	Wine	30
63	Estonia	2009	Apples	58
63	Estonia	2009	Bananas	23
63	Estonia	2009	Barley	15
63	Estonia	2009	Beer	243
63	Estonia	2009	Beverages, Alcoholic	54
63	Estonia	2009	Beverages, Fermented	8
63	Estonia	2009	Bovine Meat	35
63	Estonia	2009	Butter, Ghee	7
63	Estonia	2009	Cephalopods	2
63	Estonia	2009	Cheese	20
63	Estonia	2009	Citrus, Other	1
63	Estonia	2009	Cocoa Beans	17
63	Estonia	2009	Coconut Oil	1
63	Estonia	2009	Coffee	18
63	Estonia	2009	Cream	13
63	Estonia	2009	Crustaceans	5
63	Estonia	2009	Demersal Fish	16
63	Estonia	2009	Eggs	31

FAO country code	Country	Reference year	Food item	Gross food supply (g *capita*$^{-1}$ d^{-1})
63	Estonia	2009	Fats, Animals, Raw	4
63	Estonia	2009	Freshwater Fish	7
63	Estonia	2009	Fruits, Other	60
63	Estonia	2009	Grapefruit	5
63	Estonia	2009	Grapes	18
63	Estonia	2009	Groundnuts (Shelled Eq)	1
63	Estonia	2009	Honey	2
63	Estonia	2009	Lemons, Limes	4
63	Estonia	2009	Maize	18
63	Estonia	2009	Maize Germ Oil	1
63	Estonia	2009	Milk, Whole	372
63	Estonia	2009	Mutton & Goat Meat	2
63	Estonia	2009	Nuts	8
63	Estonia	2009	Oats	7
63	Estonia	2009	Offals, Edible	15
63	Estonia	2009	Oilcrops Oil, Other	13
63	Estonia	2009	Olive Oil	1
63	Estonia	2009	Onions	20
63	Estonia	2009	Oranges, Mandarines	52
63	Estonia	2009	Peas	1
63	Estonia	2009	Pelagic Fish	10
63	Estonia	2009	Pepper	1
63	Estonia	2009	Pigmeat	73
63	Estonia	2009	Pimento	1
63	Estonia	2009	Pineapples	7
63	Estonia	2009	Potatoes	276
63	Estonia	2009	Poultry Meat	55
63	Estonia	2009	Rice (Milled Equivalent)	5
63	Estonia	2009	Rye	53
63	Estonia	2009	Soyabean Oil	7
63	Estonia	2009	Spices, Other	1
63	Estonia	2009	Sugar, Refined Equivalent	107
63	Estonia	2009	Sweeteners, Other	10
63	Estonia	2009	Tea	1
63	Estonia	2009	Tomatoes	48
63	Estonia	2009	Vegetables, Other	227
63	Estonia	2009	Wheat	263
63	Estonia	2009	Wine	28
63	Estonia	2010	Apples	39
63	Estonia	2010	Bananas	24
63	Estonia	2010	Barley	35
63	Estonia	2010	Beer	264
63	Estonia	2010	Beverages, Alcoholic	55
63	Estonia	2010	Beverages, Fermented	11
63	Estonia	2010	Bovine Meat	35
63	Estonia	2010	Butter, Ghee	6
63	Estonia	2010	Cephalopods	1
63	Estonia	2010	Cheese	22
63	Estonia	2010	Cocoa Beans	15
63	Estonia	2010	Coconut Oil	1
63	Estonia	2010	Coffee	15
63	Estonia	2010	Cream	9
63	Estonia	2010	Crustaceans	3
63	Estonia	2010	Demersal Fish	20
63	Estonia	2010	Eggs	32
63	Estonia	2010	Fats, Animals, Raw	4
63	Estonia	2010	Freshwater Fish	7
63	Estonia	2010	Fruits, Other	57
63	Estonia	2010	Grapefruit	7
63	Estonia	2010	Grapes	17
63	Estonia	2010	Honey	2
63	Estonia	2010	Lemons, Limes	4
63	Estonia	2010	Maize	20
63	Estonia	2010	Maize Germ Oil	1
63	Estonia	2010	Meat, Other	1
63	Estonia	2010	Milk, Whole	343
63	Estonia	2010	Molluscs, Other	1
63	Estonia	2010	Mutton & Goat Meat	2
63	Estonia	2010	Nuts	10
63	Estonia	2010	Oats	6
63	Estonia	2010	Offals, Edible	15
63	Estonia	2010	Oilcrops Oil, Other	12
63	Estonia	2010	Olive Oil	1
63	Estonia	2010	Olives	1
63	Estonia	2010	Onions	20
63	Estonia	2010	Oranges, Mandarines	49
63	Estonia	2010	Peas	14
63	Estonia	2010	Pelagic Fish	8
63	Estonia	2010	Pigmeat	73
63	Estonia	2010	Pimento	1
63	Estonia	2010	Pineapples	6
63	Estonia	2010	Potatoes	305
63	Estonia	2010	Poultry Meat	58
63	Estonia	2010	Rice (Milled Equivalent)	7
63	Estonia	2010	Rye	42
63	Estonia	2010	Soyabean Oil	4
63	Estonia	2010	Spices, Other	1
63	Estonia	2010	Sugar, Refined Equivalent	91
63	Estonia	2010	Sweeteners, Other	12
63	Estonia	2010	Tea	1
63	Estonia	2010	Tomatoes	47
63	Estonia	2010	Vegetables, Other	233
63	Estonia	2010	Wheat	238
63	Estonia	2010	Wine	32
63	Estonia	2011	Apples	34
63	Estonia	2011	Bananas	24
63	Estonia	2011	Barley	8

FAO country code	Country	Reference year	Food item	Gross food supply (g $capita^{-1}$ d^{-1})
63	Estonia	2011	Beer	270
63	Estonia	2011	Beverages, Alcoholic	55
63	Estonia	2011	Beverages, Fermented	19
63	Estonia	2011	Bovine Meat	37
63	Estonia	2011	Butter, Ghee	6
63	Estonia	2011	Cephalopods	1
63	Estonia	2011	Cheese	26
63	Estonia	2011	Citrus, Other	1
63	Estonia	2011	Cocoa Beans	20
63	Estonia	2011	Coconut Oil	1
63	Estonia	2011	Coffee	14
63	Estonia	2011	Cream	11
63	Estonia	2011	Crustaceans	3
63	Estonia	2011	Demersal Fish	20
63	Estonia	2011	Eggs	31
63	Estonia	2011	Fats, Animals, Raw	4
63	Estonia	2011	Freshwater Fish	7
63	Estonia	2011	Fruits, Other	68
63	Estonia	2011	Grapefruit	5
63	Estonia	2011	Grapes	16
63	Estonia	2011	Groundnuts (Shelled Eq)	1
63	Estonia	2011	Honey	2
63	Estonia	2011	Lemons, Limes	4
63	Estonia	2011	Maize	24
63	Estonia	2011	Maize Germ Oil	1
63	Estonia	2011	Milk, Whole	317
63	Estonia	2011	Molluscs, Other	1
63	Estonia	2011	Mutton & Goat Meat	1
63	Estonia	2011	Nuts	7
63	Estonia	2011	Oats	8
63	Estonia	2011	Offals, Edible	14
63	Estonia	2011	Oilcrops Oil, Other	10
63	Estonia	2011	Olive Oil	1
63	Estonia	2011	Olives	1
63	Estonia	2011	Onions	16
63	Estonia	2011	Oranges, Mandarines	41
63	Estonia	2011	Peas	12
63	Estonia	2011	Pelagic Fish	8
63	Estonia	2011	Pepper	1
63	Estonia	2011	Pigmeat	71
63	Estonia	2011	Pimento	1
63	Estonia	2011	Pineapples	5
63	Estonia	2011	Potatoes	312
63	Estonia	2011	Poultry Meat	53
63	Estonia	2011	Rice (Milled Equivalent)	7
63	Estonia	2011	Rye	47
63	Estonia	2011	Soyabean Oil	4
63	Estonia	2011	Spices, Other	1
63	Estonia	2011	Sugar, Refined Equivalent	86
63	Estonia	2011	Sweeteners, Other	12
63	Estonia	2011	Tea	1
63	Estonia	2011	Tomatoes	51
63	Estonia	2011	Vegetables, Other	267
63	Estonia	2011	Wheat	263
63	Estonia	2011	Wine	36
238	Ethiopia	1992	Bananas	3
238	Ethiopia	1992	Barley	34
238	Ethiopia	1992	Beans	4
238	Ethiopia	1992	Beer	16
238	Ethiopia	1992	Beverages, Fermented	6
238	Ethiopia	1992	Bovine Meat	12
238	Ethiopia	1992	Butter, Ghee	1
238	Ethiopia	1992	Cereals, Other	61
238	Ethiopia	1992	Coffee	3
238	Ethiopia	1992	Eggs	3
238	Ethiopia	1992	Fats, Animals, Raw	1
238	Ethiopia	1992	Fruits, Other	5
238	Ethiopia	1992	Groundnut Oil	1
238	Ethiopia	1992	Honey	1
238	Ethiopia	1992	Maize	72
238	Ethiopia	1992	Meat, Other	6
238	Ethiopia	1992	Milk, Whole	35
238	Ethiopia	1992	Millet	7
238	Ethiopia	1992	Mutton & Goat Meat	7
238	Ethiopia	1992	Nuts	3
238	Ethiopia	1992	Oats	2
238	Ethiopia	1992	Offals, Edible	5
238	Ethiopia	1992	Oilcrops Oil, Other	1
238	Ethiopia	1992	Oilcrops, Other	5
238	Ethiopia	1992	Onions	2
238	Ethiopia	1992	Oranges, Mandarines	1
238	Ethiopia	1992	Peas	7
238	Ethiopia	1992	Pimento	5
238	Ethiopia	1992	Potatoes	15
238	Ethiopia	1992	Poultry Meat	4
238	Ethiopia	1992	Pulses, Other	26
238	Ethiopia	1992	Rape and Mustard Oil	3
238	Ethiopia	1992	Rice (Milled Equivalent)	1
238	Ethiopia	1992	Roots, Other	111
238	Ethiopia	1992	Sesameseed	1
238	Ethiopia	1992	Sorghum	44
238	Ethiopia	1992	Soyabean Oil	1
238	Ethiopia	1992	Soyabeans	1
238	Ethiopia	1992	Sugar, Refined Equivalent	7
238	Ethiopia	1992	Sweet Potatoes	7
238	Ethiopia	1992	Tomatoes	2
238	Ethiopia	1992	Vegetables, Other	21

FAO country code	Country	Reference year	Food item	Gross food supply (g *capita*$^{-1}$ d^{-1})
238	Ethiopia	1992	Wheat	77
238	Ethiopia	1992	Yams	10
238	Ethiopia	1993	Bananas	3
238	Ethiopia	1993	Barley	31
238	Ethiopia	1993	Beans	1
238	Ethiopia	1993	Beer	6
238	Ethiopia	1993	Beverages, Fermented	5
238	Ethiopia	1993	Bovine Meat	12
238	Ethiopia	1993	Butter, Ghee	1
238	Ethiopia	1993	Cereals, Other	59
238	Ethiopia	1993	Coffee	4
238	Ethiopia	1993	Eggs	1
238	Ethiopia	1993	Fats, Animals, Raw	1
238	Ethiopia	1993	Fruits, Other	5
238	Ethiopia	1993	Honey	1
238	Ethiopia	1993	Maize	101
238	Ethiopia	1993	Meat, Other	3
238	Ethiopia	1993	Milk, Whole	29
238	Ethiopia	1993	Millet	5
238	Ethiopia	1993	Mutton & Goat Meat	3
238	Ethiopia	1993	Nuts	3
238	Ethiopia	1993	Oats	2
238	Ethiopia	1993	Offals, Edible	3
238	Ethiopia	1993	Oilcrops Oil, Other	1
238	Ethiopia	1993	Onions	4
238	Ethiopia	1993	Oranges, Mandarines	1
238	Ethiopia	1993	Palm Oil	1
238	Ethiopia	1993	Peas	5
238	Ethiopia	1993	Pimento	5
238	Ethiopia	1993	Potatoes	15
238	Ethiopia	1993	Poultry Meat	2
238	Ethiopia	1993	Pulses, Other	19
238	Ethiopia	1993	Rape and Mustard Oil	1
238	Ethiopia	1993	Rice (Milled Equivalent)	1
238	Ethiopia	1993	Roots, Other	129
238	Ethiopia	1993	Sorghum	45
238	Ethiopia	1993	Soyabean Oil	1
238	Ethiopia	1993	Sugar, Refined Equivalent	8
238	Ethiopia	1993	Sweet Potatoes	8
238	Ethiopia	1993	Tomatoes	2
238	Ethiopia	1993	Vegetables, Other	27
238	Ethiopia	1993	Wheat	60
238	Ethiopia	1993	Yams	8
238	Ethiopia	1994	Bananas	2
238	Ethiopia	1994	Barley	31
238	Ethiopia	1994	Beer	4
238	Ethiopia	1994	Beverages, Fermented	6
238	Ethiopia	1994	Bovine Meat	12
238	Ethiopia	1994	Butter, Ghee	1
238	Ethiopia	1994	Cereals, Other	59
238	Ethiopia	1994	Coffee	4
238	Ethiopia	1994	Eggs	1
238	Ethiopia	1994	Fats, Animals, Raw	1
238	Ethiopia	1994	Fruits, Other	5
238	Ethiopia	1994	Honey	1
238	Ethiopia	1994	Maize	100
238	Ethiopia	1994	Maize Germ Oil	1
238	Ethiopia	1994	Meat, Other	3
238	Ethiopia	1994	Milk, Whole	29
238	Ethiopia	1994	Millet	7
238	Ethiopia	1994	Mutton & Goat Meat	3
238	Ethiopia	1994	Nuts	3
238	Ethiopia	1994	Oats	2
238	Ethiopia	1994	Offals, Edible	3
238	Ethiopia	1994	Oilcrops Oil, Other	1
238	Ethiopia	1994	Onions	4
238	Ethiopia	1994	Oranges, Mandarines	1
238	Ethiopia	1994	Palm Oil	1
238	Ethiopia	1994	Peas	4
238	Ethiopia	1994	Pimento	6
238	Ethiopia	1994	Potatoes	14
238	Ethiopia	1994	Poultry Meat	2
238	Ethiopia	1994	Pulses, Other	19
238	Ethiopia	1994	Rape and Mustard Oil	1
238	Ethiopia	1994	Rice (Milled Equivalent)	1
238	Ethiopia	1994	Roots, Other	134
238	Ethiopia	1994	Sorghum	51
238	Ethiopia	1994	Soyabean Oil	1
238	Ethiopia	1994	Sugar, Refined Equivalent	7
238	Ethiopia	1994	Sweet Potatoes	8
238	Ethiopia	1994	Tomatoes	2
238	Ethiopia	1994	Vegetables, Other	27
238	Ethiopia	1994	Wheat	67
238	Ethiopia	1994	Yams	8
238	Ethiopia	1995	Bananas	2
238	Ethiopia	1995	Barley	35
238	Ethiopia	1995	Beans	1
238	Ethiopia	1995	Beer	5
238	Ethiopia	1995	Beverages, Fermented	5
238	Ethiopia	1995	Bovine Meat	11
238	Ethiopia	1995	Butter, Ghee	1
238	Ethiopia	1995	Cereals, Other	59
238	Ethiopia	1995	Coffee	4
238	Ethiopia	1995	Eggs	1
238	Ethiopia	1995	Fats, Animals, Raw	1
238	Ethiopia	1995	Fruits, Other	6
238	Ethiopia	1995	Honey	1

FAO country code	Country	Reference year	Food item	Gross food supply (g *capita*$^{-1}$ d^{-1})
238	Ethiopia	1995	Maize	99
238	Ethiopia	1995	Meat, Other	3
238	Ethiopia	1995	Milk, Whole	31
238	Ethiopia	1995	Millet	6
238	Ethiopia	1995	Mutton & Goat Meat	3
238	Ethiopia	1995	Nuts	3
238	Ethiopia	1995	Oats	2
238	Ethiopia	1995	Offals, Edible	3
238	Ethiopia	1995	Oilcrops Oil, Other	1
238	Ethiopia	1995	Onions	4
238	Ethiopia	1995	Oranges, Mandarines	1
238	Ethiopia	1995	Peas	4
238	Ethiopia	1995	Pimento	5
238	Ethiopia	1995	Potatoes	14
238	Ethiopia	1995	Poultry Meat	2
238	Ethiopia	1995	Pulses, Other	24
238	Ethiopia	1995	Roots, Other	138
238	Ethiopia	1995	Sorghum	55
238	Ethiopia	1995	Sugar, Refined Equivalent	7
238	Ethiopia	1995	Sweet Potatoes	8
238	Ethiopia	1995	Tomatoes	2
238	Ethiopia	1995	Vegetables, Other	27
238	Ethiopia	1995	Wheat	65
238	Ethiopia	1995	Yams	8
238	Ethiopia	1996	Bananas	2
238	Ethiopia	1996	Barley	33
238	Ethiopia	1996	Beans	3
238	Ethiopia	1996	Beer	6
238	Ethiopia	1996	Beverages, Fermented	8
238	Ethiopia	1996	Bovine Meat	12
238	Ethiopia	1996	Butter, Ghee	1
238	Ethiopia	1996	Cereals, Other	63
238	Ethiopia	1996	Coffee	3
238	Ethiopia	1996	Eggs	1
238	Ethiopia	1996	Fats, Animals, Raw	1
238	Ethiopia	1996	Fruits, Other	6
238	Ethiopia	1996	Honey	1
238	Ethiopia	1996	Maize	106
238	Ethiopia	1996	Meat, Other	3
238	Ethiopia	1996	Milk, Whole	35
238	Ethiopia	1996	Millet	9
238	Ethiopia	1996	Mutton & Goat Meat	3
238	Ethiopia	1996	Nuts	3
238	Ethiopia	1996	Oats	4
238	Ethiopia	1996	Offals, Edible	3
238	Ethiopia	1996	Oilcrops Oil, Other	1
238	Ethiopia	1996	Oilcrops, Other	1
238	Ethiopia	1996	Onions	4
238	Ethiopia	1996	Oranges, Mandarines	1
238	Ethiopia	1996	Peas	4
238	Ethiopia	1996	Pimento	5
238	Ethiopia	1996	Potatoes	14
238	Ethiopia	1996	Poultry Meat	2
238	Ethiopia	1996	Pulses, Other	24
238	Ethiopia	1996	Rice (Milled Equivalent)	1
238	Ethiopia	1996	Roots, Other	138
238	Ethiopia	1996	Sorghum	58
238	Ethiopia	1996	Soyabean Oil	1
238	Ethiopia	1996	Sugar, Refined Equivalent	8
238	Ethiopia	1996	Sweet Potatoes	7
238	Ethiopia	1996	Tomatoes	2
238	Ethiopia	1996	Vegetables, Other	27
238	Ethiopia	1996	Wheat	65
238	Ethiopia	1996	Yams	7
238	Ethiopia	1997	Bananas	3
238	Ethiopia	1997	Barley	34
238	Ethiopia	1997	Beans	3
238	Ethiopia	1997	Beer	6
238	Ethiopia	1997	Beverages, Fermented	9
238	Ethiopia	1997	Bovine Meat	12
238	Ethiopia	1997	Butter, Ghee	1
238	Ethiopia	1997	Cereals, Other	68
238	Ethiopia	1997	Coffee	4
238	Ethiopia	1997	Eggs	1
238	Ethiopia	1997	Fats, Animals, Raw	1
238	Ethiopia	1997	Fruits, Other	6
238	Ethiopia	1997	Honey	1
238	Ethiopia	1997	Maize	110
238	Ethiopia	1997	Meat, Other	3
238	Ethiopia	1997	Milk, Whole	35
238	Ethiopia	1997	Millet	11
238	Ethiopia	1997	Mutton & Goat Meat	3
238	Ethiopia	1997	Nuts	4
238	Ethiopia	1997	Oats	3
238	Ethiopia	1997	Offals, Edible	3
238	Ethiopia	1997	Oilcrops Oil, Other	1
238	Ethiopia	1997	Onions	4
238	Ethiopia	1997	Oranges, Mandarines	1
238	Ethiopia	1997	Palm Oil	1
238	Ethiopia	1997	Peas	5
238	Ethiopia	1997	Pimento	4
238	Ethiopia	1997	Potatoes	13
238	Ethiopia	1997	Poultry Meat	2
238	Ethiopia	1997	Pulses, Other	24
238	Ethiopia	1997	Rice (Milled Equivalent)	1
238	Ethiopia	1997	Roots, Other	138
238	Ethiopia	1997	Sorghum	58

FAO countrycode	Country	Reference year	Food item	Gross food supply (g $capita^{-1}$ d^{-1})
238	Ethiopia	1997	Sugar, Refined Equivalent	9
238	Ethiopia	1997	Sweet Potatoes	8
238	Ethiopia	1997	Tomatoes	2
238	Ethiopia	1997	Vegetables, Other	28
238	Ethiopia	1997	Wheat	65
238	Ethiopia	1997	Yams	8
238	Ethiopia	1998	Bananas	3
238	Ethiopia	1998	Barley	34
238	Ethiopia	1998	Beans	2
238	Ethiopia	1998	Beer	5
238	Ethiopia	1998	Beverages, Fermented	8
238	Ethiopia	1998	Bovine Meat	12
238	Ethiopia	1998	Butter, Ghee	1
238	Ethiopia	1998	Cereals, Other	68
238	Ethiopia	1998	Coffee	3
238	Ethiopia	1998	Eggs	1
238	Ethiopia	1998	Fats, Animals, Raw	1
238	Ethiopia	1998	Freshwater Fish	1
238	Ethiopia	1998	Fruits, Other	6
238	Ethiopia	1998	Honey	1
238	Ethiopia	1998	Maize	110
238	Ethiopia	1998	Meat, Other	3
238	Ethiopia	1998	Milk, Whole	34
238	Ethiopia	1998	Millet	9
238	Ethiopia	1998	Mutton & Goat Meat	3
238	Ethiopia	1998	Nuts	3
238	Ethiopia	1998	Oats	2
238	Ethiopia	1998	Offals, Edible	3
238	Ethiopia	1998	Oilcrops Oil, Other	1
238	Ethiopia	1998	Onions	4
238	Ethiopia	1998	Oranges, Mandarines	1
238	Ethiopia	1998	Palm Oil	1
238	Ethiopia	1998	Peas	5
238	Ethiopia	1998	Pimento	5
238	Ethiopia	1998	Potatoes	13
238	Ethiopia	1998	Poultry Meat	2
238	Ethiopia	1998	Pulses, Other	24
238	Ethiopia	1998	Rice (Milled Equivalent)	1
238	Ethiopia	1998	Roots, Other	140
238	Ethiopia	1998	Sorghum	61
238	Ethiopia	1998	Sugar, Refined Equivalent	9
238	Ethiopia	1998	Sweet Potatoes	9
238	Ethiopia	1998	Tomatoes	2
238	Ethiopia	1998	Vegetables, Other	26
238	Ethiopia	1998	Wheat	67
238	Ethiopia	1998	Yams	6
238	Ethiopia	1999	Bananas	3
238	Ethiopia	1999	Barley	34
238	Ethiopia	1999	Beans	3
238	Ethiopia	1999	Beer	6
238	Ethiopia	1999	Beverages, Fermented	11
238	Ethiopia	1999	Bovine Meat	12
238	Ethiopia	1999	Butter, Ghee	1
238	Ethiopia	1999	Cereals, Other	67
238	Ethiopia	1999	Coffee	3
238	Ethiopia	1999	Eggs	1
238	Ethiopia	1999	Fats, Animals, Raw	1
238	Ethiopia	1999	Freshwater Fish	1
238	Ethiopia	1999	Fruits, Other	6
238	Ethiopia	1999	Honey	1
238	Ethiopia	1999	Maize	114
238	Ethiopia	1999	Meat, Other	3
238	Ethiopia	1999	Milk, Whole	34
238	Ethiopia	1999	Millet	13
238	Ethiopia	1999	Mutton & Goat Meat	3
238	Ethiopia	1999	Nuts	3
238	Ethiopia	1999	Oats	2
238	Ethiopia	1999	Offals, Edible	3
238	Ethiopia	1999	Oilcrops Oil, Other	1
238	Ethiopia	1999	Onions	4
238	Ethiopia	1999	Oranges, Mandarines	1
238	Ethiopia	1999	Palm Oil	1
238	Ethiopia	1999	Peas	5
238	Ethiopia	1999	Pimento	5
238	Ethiopia	1999	Potatoes	14
238	Ethiopia	1999	Poultry Meat	2
238	Ethiopia	1999	Pulses, Other	23
238	Ethiopia	1999	Rice (Milled Equivalent)	1
238	Ethiopia	1999	Roots, Other	141
238	Ethiopia	1999	Sorghum	61
238	Ethiopia	1999	Sugar, Refined Equivalent	9
238	Ethiopia	1999	Sweet Potatoes	9
238	Ethiopia	1999	Tomatoes	2
238	Ethiopia	1999	Vegetables, Other	27
238	Ethiopia	1999	Wheat	68
238	Ethiopia	1999	Yams	7
238	Ethiopia	2000	Bananas	4
238	Ethiopia	2000	Barley	32
238	Ethiopia	2000	Beans	4
238	Ethiopia	2000	Beer	5
238	Ethiopia	2000	Beverages, Fermented	9
238	Ethiopia	2000	Bovine Meat	12
238	Ethiopia	2000	Butter, Ghee	1
238	Ethiopia	2000	Cereals, Other	68
238	Ethiopia	2000	Coffee	3
238	Ethiopia	2000	Eggs	1
238	Ethiopia	2000	Fats, Animals, Raw	1

FAO countrycode	Country	Reference year	Food item	Gross food supply (g *capita*$^{-1}$ d^{-1})
238	Ethiopia	2000	Freshwater Fish	1
238	Ethiopia	2000	Fruits, Other	10
238	Ethiopia	2000	Honey	1
238	Ethiopia	2000	Maize	115
238	Ethiopia	2000	Meat, Other	3
238	Ethiopia	2000	Milk, Whole	27
238	Ethiopia	2000	Millet	10
238	Ethiopia	2000	Mutton & Goat Meat	3
238	Ethiopia	2000	Nuts	3
238	Ethiopia	2000	Oats	2
238	Ethiopia	2000	Offals, Edible	3
238	Ethiopia	2000	Oilcrops Oil, Other	1
238	Ethiopia	2000	Onions	4
238	Ethiopia	2000	Oranges, Mandarines	1
238	Ethiopia	2000	Peas	6
238	Ethiopia	2000	Pimento	5
238	Ethiopia	2000	Potatoes	13
238	Ethiopia	2000	Poultry Meat	2
238	Ethiopia	2000	Pulses, Other	26
238	Ethiopia	2000	Rice (Milled Equivalent)	1
238	Ethiopia	2000	Roots, Other	143
238	Ethiopia	2000	Sorghum	61
238	Ethiopia	2000	Soyabean Oil	1
238	Ethiopia	2000	Sugar, Refined Equivalent	9
238	Ethiopia	2000	Sweet Potatoes	12
238	Ethiopia	2000	Tomatoes	2
238	Ethiopia	2000	Vegetables, Other	26
238	Ethiopia	2000	Wheat	83
238	Ethiopia	2000	Yams	7
238	Ethiopia	2001	Bananas	4
238	Ethiopia	2001	Barley	34
238	Ethiopia	2001	Beans	7
238	Ethiopia	2001	Beer	6
238	Ethiopia	2001	Beverages, Fermented	9
238	Ethiopia	2001	Bovine Meat	12
238	Ethiopia	2001	Butter, Ghee	1
238	Ethiopia	2001	Cereals, Other	66
238	Ethiopia	2001	Coffee	4
238	Ethiopia	2001	Eggs	1
238	Ethiopia	2001	Fats, Animals, Raw	1
238	Ethiopia	2001	Freshwater Fish	1
238	Ethiopia	2001	Fruits, Other	7
238	Ethiopia	2001	Honey	1
238	Ethiopia	2001	Maize	111
238	Ethiopia	2001	Meat, Other	3
238	Ethiopia	2001	Milk, Whole	29
238	Ethiopia	2001	Millet	10
238	Ethiopia	2001	Mutton & Goat Meat	3
238	Ethiopia	2001	Nuts	3
238	Ethiopia	2001	Oats	2
238	Ethiopia	2001	Offals, Edible	3
238	Ethiopia	2001	Oilcrops Oil, Other	1
238	Ethiopia	2001	Onions	5
238	Ethiopia	2001	Oranges, Mandarines	1
238	Ethiopia	2001	Palm Oil	1
238	Ethiopia	2001	Peas	6
238	Ethiopia	2001	Pimento	5
238	Ethiopia	2001	Potatoes	15
238	Ethiopia	2001	Poultry Meat	2
238	Ethiopia	2001	Pulses, Other	25
238	Ethiopia	2001	Rice (Milled Equivalent)	1
238	Ethiopia	2001	Roots, Other	144
238	Ethiopia	2001	Sorghum	63
238	Ethiopia	2001	Soyabean Oil	1
238	Ethiopia	2001	Sugar, Refined Equivalent	9
238	Ethiopia	2001	Sweet Potatoes	15
238	Ethiopia	2001	Tomatoes	1
238	Ethiopia	2001	Vegetables, Other	27
238	Ethiopia	2001	Wheat	92
238	Ethiopia	2001	Yams	7
238	Ethiopia	2002	Bananas	6
238	Ethiopia	2002	Barley	36
238	Ethiopia	2002	Beans	1
238	Ethiopia	2002	Beer	6
238	Ethiopia	2002	Beverages, Fermented	8
238	Ethiopia	2002	Bovine Meat	14
238	Ethiopia	2002	Butter, Ghee	1
238	Ethiopia	2002	Cereals, Other	67
238	Ethiopia	2002	Coffee	1
238	Ethiopia	2002	Eggs	1
238	Ethiopia	2002	Fats, Animals, Raw	1
238	Ethiopia	2002	Fruits, Other	7
238	Ethiopia	2002	Honey	2
238	Ethiopia	2002	Maize	109
238	Ethiopia	2002	Meat, Other	3
238	Ethiopia	2002	Milk, Whole	86
238	Ethiopia	2002	Millet	9
238	Ethiopia	2002	Mutton & Goat Meat	3
238	Ethiopia	2002	Nuts	3
238	Ethiopia	2002	Oats	1
238	Ethiopia	2002	Offals, Edible	4
238	Ethiopia	2002	Oilcrops Oil, Other	1
238	Ethiopia	2002	Onions	5
238	Ethiopia	2002	Oranges, Mandarines	1
238	Ethiopia	2002	Palm Oil	1
238	Ethiopia	2002	Peas	6
238	Ethiopia	2002	Pimento	4

FAO country code	Country	Reference year	Food item	Gross food supply (g $capita^{-1}$ d^{-1})
238	Ethiopia	2002	Potatoes	12
238	Ethiopia	2002	Poultry Meat	2
238	Ethiopia	2002	Pulses, Other	26
238	Ethiopia	2002	Rice (Milled Equivalent)	1
238	Ethiopia	2002	Roots, Other	146
238	Ethiopia	2002	Sorghum	62
238	Ethiopia	2002	Sugar, Refined Equivalent	9
238	Ethiopia	2002	Sweet Potatoes	13
238	Ethiopia	2002	Tomatoes	1
238	Ethiopia	2002	Vegetables, Other	29
238	Ethiopia	2002	Wheat	92
238	Ethiopia	2002	Yams	7
238	Ethiopia	2003	Bananas	6
238	Ethiopia	2003	Barley	35
238	Ethiopia	2003	Beans	2
238	Ethiopia	2003	Beer	6
238	Ethiopia	2003	Beverages, Fermented	8
238	Ethiopia	2003	Bovine Meat	13
238	Ethiopia	2003	Butter, Ghee	1
238	Ethiopia	2003	Cereals, Other	68
238	Ethiopia	2003	Coffee	1
238	Ethiopia	2003	Eggs	1
238	Ethiopia	2003	Fats, Animals, Raw	1
238	Ethiopia	2003	Fruits, Other	8
238	Ethiopia	2003	Groundnuts (Shelled Eq)	1
238	Ethiopia	2003	Honey	1
238	Ethiopia	2003	Maize	106
238	Ethiopia	2003	Meat, Other	3
238	Ethiopia	2003	Milk, Whole	86
238	Ethiopia	2003	Millet	9
238	Ethiopia	2003	Mutton & Goat Meat	3
238	Ethiopia	2003	Nuts	3
238	Ethiopia	2003	Oats	1
238	Ethiopia	2003	Offals, Edible	4
238	Ethiopia	2003	Oilcrops Oil, Other	2
238	Ethiopia	2003	Onions	6
238	Ethiopia	2003	Oranges, Mandarines	1
238	Ethiopia	2003	Palm Oil	1
238	Ethiopia	2003	Peas	7
238	Ethiopia	2003	Pimento	4
238	Ethiopia	2003	Potatoes	17
238	Ethiopia	2003	Poultry Meat	2
238	Ethiopia	2003	Pulses, Other	25
238	Ethiopia	2003	Rice (Milled Equivalent)	1
238	Ethiopia	2003	Roots, Other	147
238	Ethiopia	2003	Sorghum	64
238	Ethiopia	2003	Sugar, Refined Equivalent	9
238	Ethiopia	2003	Sweet Potatoes	19
238	Ethiopia	2003	Tomatoes	2
238	Ethiopia	2003	Vegetables, Other	27
238	Ethiopia	2003	Wheat	97
238	Ethiopia	2003	Yams	7
238	Ethiopia	2004	Bananas	6
238	Ethiopia	2004	Barley	40
238	Ethiopia	2004	Beans	5
238	Ethiopia	2004	Beer	6
238	Ethiopia	2004	Beverages, Fermented	9
238	Ethiopia	2004	Bovine Meat	12
238	Ethiopia	2004	Butter, Ghee	1
238	Ethiopia	2004	Cereals, Other	69
238	Ethiopia	2004	Coffee	1
238	Ethiopia	2004	Eggs	1
238	Ethiopia	2004	Fats, Animals, Raw	1
238	Ethiopia	2004	Fruits, Other	8
238	Ethiopia	2004	Honey	2
238	Ethiopia	2004	Maize	100
238	Ethiopia	2004	Meat, Other	3
238	Ethiopia	2004	Milk, Whole	83
238	Ethiopia	2004	Millet	10
238	Ethiopia	2004	Mutton & Goat Meat	4
238	Ethiopia	2004	Nuts	2
238	Ethiopia	2004	Oats	1
238	Ethiopia	2004	Offals, Edible	4
238	Ethiopia	2004	Oilcrops Oil, Other	2
238	Ethiopia	2004	Onions	7
238	Ethiopia	2004	Oranges, Mandarines	1
238	Ethiopia	2004	Palm Oil	1
238	Ethiopia	2004	Peas	7
238	Ethiopia	2004	Pimento	4
238	Ethiopia	2004	Potatoes	16
238	Ethiopia	2004	Poultry Meat	2
238	Ethiopia	2004	Pulses, Other	25
238	Ethiopia	2004	Rice (Milled Equivalent)	1
238	Ethiopia	2004	Roots, Other	150
238	Ethiopia	2004	Sorghum	62
238	Ethiopia	2004	Soyabean Oil	1
238	Ethiopia	2004	Sugar, Refined Equivalent	13
238	Ethiopia	2004	Sweet Potatoes	17
238	Ethiopia	2004	Tomatoes	1
238	Ethiopia	2004	Vegetables, Other	33
238	Ethiopia	2004	Wheat	91
238	Ethiopia	2004	Yams	6
238	Ethiopia	2005	Bananas	7
238	Ethiopia	2005	Barley	41
238	Ethiopia	2005	Beans	7
238	Ethiopia	2005	Beer	7
238	Ethiopia	2005	Beverages, Fermented	10

FAO country code	Country	Reference year	Food item	Gross food supply (g *capita*$^{-1}$ d^{-1})
238	Ethiopia	2005	Bovine Meat	13
238	Ethiopia	2005	Butter, Ghee	1
238	Ethiopia	2005	Cereals, Other	74
238	Ethiopia	2005	Eggs	1
238	Ethiopia	2005	Fats, Animals, Raw	1
238	Ethiopia	2005	Fruits, Other	12
238	Ethiopia	2005	Honey	1
238	Ethiopia	2005	Maize	111
238	Ethiopia	2005	Meat, Other	3
238	Ethiopia	2005	Milk, Whole	67
238	Ethiopia	2005	Millet	11
238	Ethiopia	2005	Mutton & Goat Meat	4
238	Ethiopia	2005	Nuts	3
238	Ethiopia	2005	Oats	2
238	Ethiopia	2005	Offals, Edible	4
238	Ethiopia	2005	Oilcrops Oil, Other	3
238	Ethiopia	2005	Oilcrops, Other	1
238	Ethiopia	2005	Onions	7
238	Ethiopia	2005	Oranges, Mandarines	2
238	Ethiopia	2005	Palm Oil	1
238	Ethiopia	2005	Peas	7
238	Ethiopia	2005	Pimento	4
238	Ethiopia	2005	Potatoes	13
238	Ethiopia	2005	Poultry Meat	2
238	Ethiopia	2005	Pulses, Other	28
238	Ethiopia	2005	Rice (Milled Equivalent)	1
238	Ethiopia	2005	Roots, Other	152
238	Ethiopia	2005	Sorghum	57
238	Ethiopia	2005	Soyabean Oil	1
238	Ethiopia	2005	Sugar, Refined Equivalent	12
238	Ethiopia	2005	Sweet Potatoes	15
238	Ethiopia	2005	Tomatoes	1
238	Ethiopia	2005	Vegetables, Other	36
238	Ethiopia	2005	Wheat	99
238	Ethiopia	2005	Yams	5
238	Ethiopia	2006	Bananas	7
238	Ethiopia	2006	Barley	38
238	Ethiopia	2006	Beans	4
238	Ethiopia	2006	Beer	10
238	Ethiopia	2006	Beverages, Fermented	12
238	Ethiopia	2006	Bovine Meat	13
238	Ethiopia	2006	Butter, Ghee	1
238	Ethiopia	2006	Cereals, Other	73
238	Ethiopia	2006	Eggs	1
238	Ethiopia	2006	Fats, Animals, Raw	1
238	Ethiopia	2006	Fruits, Other	13
238	Ethiopia	2006	Groundnuts (Shelled Eq)	1
238	Ethiopia	2006	Honey	2
238	Ethiopia	2006	Maize	113
238	Ethiopia	2006	Meat, Other	3
238	Ethiopia	2006	Milk, Whole	72
238	Ethiopia	2006	Millet	14
238	Ethiopia	2006	Mutton & Goat Meat	4
238	Ethiopia	2006	Nuts	2
238	Ethiopia	2006	Oats	1
238	Ethiopia	2006	Offals, Edible	4
238	Ethiopia	2006	Oilcrops Oil, Other	2
238	Ethiopia	2006	Oilcrops, Other	1
238	Ethiopia	2006	Onions	5
238	Ethiopia	2006	Oranges, Mandarines	2
238	Ethiopia	2006	Palm Oil	1
238	Ethiopia	2006	Peas	6
238	Ethiopia	2006	Pimento	3
238	Ethiopia	2006	Potatoes	13
238	Ethiopia	2006	Poultry Meat	2
238	Ethiopia	2006	Pulses, Other	31
238	Ethiopia	2006	Rice (Milled Equivalent)	1
238	Ethiopia	2006	Roots, Other	154
238	Ethiopia	2006	Sorghum	66
238	Ethiopia	2006	Soyabean Oil	1
238	Ethiopia	2006	Sugar, Refined Equivalent	13
238	Ethiopia	2006	Sweet Potatoes	14
238	Ethiopia	2006	Tomatoes	1
238	Ethiopia	2006	Vegetables, Other	33
238	Ethiopia	2006	Wheat	88
238	Ethiopia	2006	Yams	6
238	Ethiopia	2007	Bananas	8
238	Ethiopia	2007	Barley	36
238	Ethiopia	2007	Beans	4
238	Ethiopia	2007	Beer	11
238	Ethiopia	2007	Beverages, Fermented	9
238	Ethiopia	2007	Bovine Meat	12
238	Ethiopia	2007	Butter, Ghee	1
238	Ethiopia	2007	Cereals, Other	74
238	Ethiopia	2007	Coffee	1
238	Ethiopia	2007	Eggs	1
238	Ethiopia	2007	Fats, Animals, Raw	1
238	Ethiopia	2007	Fruits, Other	13
238	Ethiopia	2007	Groundnuts (Shelled Eq)	1
238	Ethiopia	2007	Honey	1
238	Ethiopia	2007	Maize	109
238	Ethiopia	2007	Meat, Other	3
238	Ethiopia	2007	Milk, Whole	81
238	Ethiopia	2007	Millet	11
238	Ethiopia	2007	Mutton & Goat Meat	5
238	Ethiopia	2007	Nuts	2
238	Ethiopia	2007	Oats	1

FAO country code	Country	Reference year	Food item	Gross food supply (g $capita^{-1}$ d^{-1})
238	Ethiopia	2007	Offals, Edible	4
238	Ethiopia	2007	Oilcrops Oil, Other	2
238	Ethiopia	2007	Onions	6
238	Ethiopia	2007	Oranges, Mandarines	2
238	Ethiopia	2007	Palm Oil	2
238	Ethiopia	2007	Peas	7
238	Ethiopia	2007	Pimento	3
238	Ethiopia	2007	Potatoes	15
238	Ethiopia	2007	Poultry Meat	2
238	Ethiopia	2007	Pulses, Other	32
238	Ethiopia	2007	Rice (Milled Equivalent)	2
238	Ethiopia	2007	Roots, Other	152
238	Ethiopia	2007	Sorghum	62
238	Ethiopia	2007	Soyabean Oil	1
238	Ethiopia	2007	Spices, Other	1
238	Ethiopia	2007	Sugar, Refined Equivalent	13
238	Ethiopia	2007	Sweet Potatoes	18
238	Ethiopia	2007	Tomatoes	1
238	Ethiopia	2007	Vegetables, Other	28
238	Ethiopia	2007	Wheat	87
238	Ethiopia	2007	Yams	8
238	Ethiopia	2008	Bananas	6
238	Ethiopia	2008	Barley	36
238	Ethiopia	2008	Beans	5
238	Ethiopia	2008	Beer	10
238	Ethiopia	2008	Beverages, Fermented	11
238	Ethiopia	2008	Bovine Meat	13
238	Ethiopia	2008	Butter, Ghee	1
238	Ethiopia	2008	Cereals, Other	70
238	Ethiopia	2008	Coffee	2
238	Ethiopia	2008	Eggs	1
238	Ethiopia	2008	Fats, Animals, Raw	1
238	Ethiopia	2008	Freshwater Fish	1
238	Ethiopia	2008	Fruits, Other	10
238	Ethiopia	2008	Groundnuts (Shelled Eq)	1
238	Ethiopia	2008	Honey	1
238	Ethiopia	2008	Maize	115
238	Ethiopia	2008	Meat, Other	4
238	Ethiopia	2008	Milk, Whole	102
238	Ethiopia	2008	Millet	13
238	Ethiopia	2008	Mutton & Goat Meat	5
238	Ethiopia	2008	Nuts	2
238	Ethiopia	2008	Oats	1
238	Ethiopia	2008	Offals, Edible	4
238	Ethiopia	2008	Oilcrops Oil, Other	2
238	Ethiopia	2008	Oilcrops, Other	1
238	Ethiopia	2008	Onions	5
238	Ethiopia	2008	Oranges, Mandarines	1
238	Ethiopia	2008	Palm Oil	5
238	Ethiopia	2008	Peas	8
238	Ethiopia	2008	Pimento	4
238	Ethiopia	2008	Potatoes	10
238	Ethiopia	2008	Poultry Meat	2
238	Ethiopia	2008	Pulses, Other	34
238	Ethiopia	2008	Rice (Milled Equivalent)	2
238	Ethiopia	2008	Roots, Other	142
238	Ethiopia	2008	Sorghum	64
238	Ethiopia	2008	Spices, Other	1
238	Ethiopia	2008	Sugar, Refined Equivalent	13
238	Ethiopia	2008	Sweet Potatoes	9
238	Ethiopia	2008	Sweeteners, Other	1
238	Ethiopia	2008	Tomatoes	1
238	Ethiopia	2008	Vegetables, Other	31
238	Ethiopia	2008	Wheat	91
238	Ethiopia	2008	Yams	6
238	Ethiopia	2009	Bananas	6
238	Ethiopia	2009	Barley	43
238	Ethiopia	2009	Beans	6
238	Ethiopia	2009	Beer	10
238	Ethiopia	2009	Beverages, Fermented	12
238	Ethiopia	2009	Bovine Meat	11
238	Ethiopia	2009	Butter, Ghee	1
238	Ethiopia	2009	Cereals, Other	69
238	Ethiopia	2009	Coffee	2
238	Ethiopia	2009	Eggs	1
238	Ethiopia	2009	Fats, Animals, Raw	1
238	Ethiopia	2009	Freshwater Fish	1
238	Ethiopia	2009	Fruits, Other	12
238	Ethiopia	2009	Groundnuts (Shelled Eq)	1
238	Ethiopia	2009	Honey	1
238	Ethiopia	2009	Maize	113
238	Ethiopia	2009	Meat, Other	4
238	Ethiopia	2009	Milk, Whole	88
238	Ethiopia	2009	Millet	13
238	Ethiopia	2009	Mutton & Goat Meat	5
238	Ethiopia	2009	Nuts	2
238	Ethiopia	2009	Oats	1
238	Ethiopia	2009	Offals, Edible	4
238	Ethiopia	2009	Oilcrops Oil, Other	1
238	Ethiopia	2009	Oilcrops, Other	1
238	Ethiopia	2009	Onions	5
238	Ethiopia	2009	Oranges, Mandarines	2
238	Ethiopia	2009	Palm Oil	5
238	Ethiopia	2009	Peas	8
238	Ethiopia	2009	Pimento	3
238	Ethiopia	2009	Potatoes	16
238	Ethiopia	2009	Poultry Meat	2

FAO countrycode	Country	Reference year	Food item	Gross food supply (g *capita*$^{-1}$ d^{-1})
238	Ethiopia	2009	Pulses, Other	34
238	Ethiopia	2009	Rice (Milled Equivalent)	3
238	Ethiopia	2009	Roots, Other	133
238	Ethiopia	2009	Sorghum	71
238	Ethiopia	2009	Spices, Other	1
238	Ethiopia	2009	Sugar, Refined Equivalent	12
238	Ethiopia	2009	Sweet Potatoes	15
238	Ethiopia	2009	Tomatoes	1
238	Ethiopia	2009	Vegetables, Other	36
238	Ethiopia	2009	Wheat	99
238	Ethiopia	2009	Yams	10
238	Ethiopia	2010	Bananas	8
238	Ethiopia	2010	Barley	40
238	Ethiopia	2010	Beans	7
238	Ethiopia	2010	Beer	11
238	Ethiopia	2010	Beverages, Fermented	14
238	Ethiopia	2010	Bovine Meat	13
238	Ethiopia	2010	Butter, Ghee	1
238	Ethiopia	2010	Cereals, Other	74
238	Ethiopia	2010	Coffee	3
238	Ethiopia	2010	Eggs	1
238	Ethiopia	2010	Fats, Animals, Raw	1
238	Ethiopia	2010	Freshwater Fish	1
238	Ethiopia	2010	Fruits, Other	10
238	Ethiopia	2010	Groundnuts (Shelled Eq)	1
238	Ethiopia	2010	Honey	2
238	Ethiopia	2010	Maize	116
238	Ethiopia	2010	Meat, Other	4
238	Ethiopia	2010	Milk, Whole	123
238	Ethiopia	2010	Millet	16
238	Ethiopia	2010	Mutton & Goat Meat	4
238	Ethiopia	2010	Nuts	2
238	Ethiopia	2010	Oats	1
238	Ethiopia	2010	Offals, Edible	4
238	Ethiopia	2010	Oilcrops Oil, Other	1
238	Ethiopia	2010	Onions	7
238	Ethiopia	2010	Oranges, Mandarines	2
238	Ethiopia	2010	Palm Oil	6
238	Ethiopia	2010	Peas	8
238	Ethiopia	2010	Pimento	2
238	Ethiopia	2010	Potatoes	11
238	Ethiopia	2010	Poultry Meat	2
238	Ethiopia	2010	Pulses, Other	34
238	Ethiopia	2010	Rice (Milled Equivalent)	3
238	Ethiopia	2010	Roots, Other	133
238	Ethiopia	2010	Sorghum	70
238	Ethiopia	2010	Spices, Other	1
238	Ethiopia	2010	Sugar, Refined Equivalent	13
238	Ethiopia	2010	Sweet Potatoes	23
238	Ethiopia	2010	Tomatoes	1
238	Ethiopia	2010	Vegetables, Other	43
238	Ethiopia	2010	Wheat	88
238	Ethiopia	2010	Yams	9
238	Ethiopia	2011	Bananas	8
238	Ethiopia	2011	Barley	39
238	Ethiopia	2011	Beans	6
238	Ethiopia	2011	Beer	11
238	Ethiopia	2011	Beverages, Fermented	14
238	Ethiopia	2011	Bovine Meat	12
238	Ethiopia	2011	Butter, Ghee	1
238	Ethiopia	2011	Cereals, Other	75
238	Ethiopia	2011	Coffee	2
238	Ethiopia	2011	Eggs	1
238	Ethiopia	2011	Fats, Animals, Raw	1
238	Ethiopia	2011	Freshwater Fish	1
238	Ethiopia	2011	Fruits, Other	11
238	Ethiopia	2011	Groundnuts (Shelled Eq)	2
238	Ethiopia	2011	Honey	1
238	Ethiopia	2011	Maize	118
238	Ethiopia	2011	Meat, Other	3
238	Ethiopia	2011	Milk, Whole	95
238	Ethiopia	2011	Millet	16
238	Ethiopia	2011	Mutton & Goat Meat	4
238	Ethiopia	2011	Nuts	2
238	Ethiopia	2011	Oats	1
238	Ethiopia	2011	Offals, Edible	4
238	Ethiopia	2011	Oilcrops Oil, Other	2
238	Ethiopia	2011	Onions	9
238	Ethiopia	2011	Oranges, Mandarines	2
238	Ethiopia	2011	Palm Oil	5
238	Ethiopia	2011	Peas	8
238	Ethiopia	2011	Pimento	3
238	Ethiopia	2011	Potatoes	10
238	Ethiopia	2011	Poultry Meat	2
238	Ethiopia	2011	Pulses, Other	38
238	Ethiopia	2011	Rape and Mustard Oil	1
238	Ethiopia	2011	Rice (Milled Equivalent)	4
238	Ethiopia	2011	Roots, Other	141
238	Ethiopia	2011	Sorghum	69
238	Ethiopia	2011	Soyabeans	1
238	Ethiopia	2011	Spices, Other	1
238	Ethiopia	2011	Sugar, Refined Equivalent	15
238	Ethiopia	2011	Sweet Potatoes	12
238	Ethiopia	2011	Tomatoes	2
238	Ethiopia	2011	Vegetables, Other	38
238	Ethiopia	2011	Wheat	87
238	Ethiopia	2011	Yams	8

FAO country code	Country	Reference year	Food item	Gross food supply (g $capita^{-1}$ d^{-1})
67	Finland	1992	Apples	40
67	Finland	1992	Bananas	42
67	Finland	1992	Barley	7
67	Finland	1992	Beer	254
67	Finland	1992	Beverages, Alcoholic	20
67	Finland	1992	Beverages, Fermented	1
67	Finland	1992	Bovine Meat	54
67	Finland	1992	Butter, Ghee	22
67	Finland	1992	Cereals, Other	1
67	Finland	1992	Cheese	36
67	Finland	1992	Cocoa Beans	1
67	Finland	1992	Coconuts - Incl Copra	1
67	Finland	1992	Coffee	33
67	Finland	1992	Cream	18
67	Finland	1992	Crustaceans	4
67	Finland	1992	Demersal Fish	9
67	Finland	1992	Eggs	29
67	Finland	1992	Fats, Animals, Raw	2
67	Finland	1992	Freshwater Fish	22
67	Finland	1992	Fruits, Other	42
67	Finland	1992	Grapefruit	2
67	Finland	1992	Grapes	15
67	Finland	1992	Groundnuts (Shelled Eq)	1
67	Finland	1992	Honey	1
67	Finland	1992	Lemons, Limes	2
67	Finland	1992	Marine Fish, Other	5
67	Finland	1992	Meat, Other	2
67	Finland	1992	Milk, Whole	397
67	Finland	1992	Molluscs, Other	1
67	Finland	1992	Mutton & Goat Meat	1
67	Finland	1992	Nuts	3
67	Finland	1992	Oats	15
67	Finland	1992	Offals, Edible	8
67	Finland	1992	Oilcrops Oil, Other	2
67	Finland	1992	Onions	12
67	Finland	1992	Oranges, Mandarines	67
67	Finland	1992	Peas	4
67	Finland	1992	Pelagic Fish	46
67	Finland	1992	Pigmeat	88
67	Finland	1992	Pineapples	8
67	Finland	1992	Potatoes	182
67	Finland	1992	Poultry Meat	21
67	Finland	1992	Rape and Mustard Oil	19
67	Finland	1992	Rice (Milled Equivalent)	13
67	Finland	1992	Rye	48
67	Finland	1992	Soyabean Oil	3
67	Finland	1992	Sugar, Refined Equivalent	101
67	Finland	1992	Sunflowerseed Oil	4
67	Finland	1992	Sweeteners, Other	8
67	Finland	1992	Tea	1
67	Finland	1992	Tomatoes	36
67	Finland	1992	Vegetables, Other	114
67	Finland	1992	Wheat	193
67	Finland	1992	Wine	15
67	Finland	1993	Apples	47
67	Finland	1993	Bananas	36
67	Finland	1993	Barley	8
67	Finland	1993	Beer	244
67	Finland	1993	Beverages, Alcoholic	16
67	Finland	1993	Beverages, Fermented	1
67	Finland	1993	Bovine Meat	52
67	Finland	1993	Butter, Ghee	21
67	Finland	1993	Cereals, Other	1
67	Finland	1993	Cheese	33
67	Finland	1993	Coconut Oil	3
67	Finland	1993	Coconuts - Incl Copra	1
67	Finland	1993	Coffee	36
67	Finland	1993	Cream	16
67	Finland	1993	Crustaceans	5
67	Finland	1993	Demersal Fish	9
67	Finland	1993	Eggs	28
67	Finland	1993	Fats, Animals, Raw	2
67	Finland	1993	Freshwater Fish	25
67	Finland	1993	Fruits, Other	35
67	Finland	1993	Grapefruit	2
67	Finland	1993	Grapes	16
67	Finland	1993	Groundnuts (Shelled Eq)	1
67	Finland	1993	Honey	1
67	Finland	1993	Lemons, Limes	2
67	Finland	1993	Marine Fish, Other	5
67	Finland	1993	Meat, Other	2
67	Finland	1993	Milk, Whole	395
67	Finland	1993	Molluscs, Other	1
67	Finland	1993	Mutton & Goat Meat	1
67	Finland	1993	Nuts	4
67	Finland	1993	Oats	15
67	Finland	1993	Offals, Edible	8
67	Finland	1993	Oilcrops Oil, Other	3
67	Finland	1993	Onions	11
67	Finland	1993	Oranges, Mandarines	61
67	Finland	1993	Peas	4
67	Finland	1993	Pelagic Fish	42
67	Finland	1993	Pigmeat	81
67	Finland	1993	Pineapples	6
67	Finland	1993	Potatoes	182
67	Finland	1993	Poultry Meat	20
67	Finland	1993	Rape and Mustard Oil	19

FAO country code	Country	Reference year	Food item	Gross food supply (g *capita*$^{-1}$ d^{-1})
67	Finland	1993	Rice (Milled Equivalent)	13
67	Finland	1993	Rye	50
67	Finland	1993	Sugar, Refined Equivalent	103
67	Finland	1993	Sunflowerseed Oil	3
67	Finland	1993	Sweeteners, Other	7
67	Finland	1993	Tea	1
67	Finland	1993	Tomatoes	39
67	Finland	1993	Vegetables, Other	114
67	Finland	1993	Wheat	161
67	Finland	1993	Wine	15
67	Finland	1994	Apples	49
67	Finland	1994	Bananas	38
67	Finland	1994	Barley	9
67	Finland	1994	Beer	237
67	Finland	1994	Beverages, Alcoholic	12
67	Finland	1994	Bovine Meat	54
67	Finland	1994	Butter, Ghee	16
67	Finland	1994	Cereals, Other	1
67	Finland	1994	Cheese	34
67	Finland	1994	Coconut Oil	2
67	Finland	1994	Coconuts - Incl Copra	1
67	Finland	1994	Coffee	37
67	Finland	1994	Cream	19
67	Finland	1994	Crustaceans	5
67	Finland	1994	Demersal Fish	11
67	Finland	1994	Eggs	27
67	Finland	1994	Fats, Animals, Raw	2
67	Finland	1994	Freshwater Fish	24
67	Finland	1994	Fruits, Other	42
67	Finland	1994	Grapefruit	2
67	Finland	1994	Grapes	17
67	Finland	1994	Groundnuts (Shelled Eq)	2
67	Finland	1994	Honey	2
67	Finland	1994	Lemons, Limes	2
67	Finland	1994	Marine Fish, Other	6
67	Finland	1994	Meat, Other	2
67	Finland	1994	Milk, Whole	426
67	Finland	1994	Molluscs, Other	1
67	Finland	1994	Mutton & Goat Meat	1
67	Finland	1994	Nuts	4
67	Finland	1994	Oats	13
67	Finland	1994	Offals, Edible	7
67	Finland	1994	Oilcrops Oil, Other	2
67	Finland	1994	Onions	9
67	Finland	1994	Oranges, Mandarines	53
67	Finland	1994	Peas	4
67	Finland	1994	Pelagic Fish	42
67	Finland	1994	Pigmeat	80
67	Finland	1994	Pineapples	9
67	Finland	1994	Potatoes	184
67	Finland	1994	Poultry Meat	23
67	Finland	1994	Rape and Mustard Oil	19
67	Finland	1994	Rice (Milled Equivalent)	18
67	Finland	1994	Rye	47
67	Finland	1994	Sugar, Refined Equivalent	95
67	Finland	1994	Sunflowerseed Oil	3
67	Finland	1994	Sweeteners, Other	6
67	Finland	1994	Tea	1
67	Finland	1994	Tomatoes	46
67	Finland	1994	Vegetables, Other	139
67	Finland	1994	Wheat	155
67	Finland	1994	Wine	15
67	Finland	1995	Apples	33
67	Finland	1995	Bananas	32
67	Finland	1995	Barley	22
67	Finland	1995	Beer	242
67	Finland	1995	Beverages, Alcoholic	11
67	Finland	1995	Beverages, Fermented	1
67	Finland	1995	Bovine Meat	53
67	Finland	1995	Butter, Ghee	18
67	Finland	1995	Cereals, Other	1
67	Finland	1995	Cheese	36
67	Finland	1995	Cocoa Beans	1
67	Finland	1995	Coconut Oil	2
67	Finland	1995	Coffee	21
67	Finland	1995	Cream	17
67	Finland	1995	Crustaceans	3
67	Finland	1995	Demersal Fish	10
67	Finland	1995	Eggs	31
67	Finland	1995	Fats, Animals, Raw	3
67	Finland	1995	Freshwater Fish	29
67	Finland	1995	Fruits, Other	30
67	Finland	1995	Grapefruit	2
67	Finland	1995	Grapes	10
67	Finland	1995	Groundnuts (Shelled Eq)	1
67	Finland	1995	Honey	1
67	Finland	1995	Lemons, Limes	1
67	Finland	1995	Marine Fish, Other	1
67	Finland	1995	Meat, Other	2
67	Finland	1995	Milk, Whole	384
67	Finland	1995	Molluscs, Other	1
67	Finland	1995	Mutton & Goat Meat	1
67	Finland	1995	Nuts	4
67	Finland	1995	Oats	16
67	Finland	1995	Offals, Edible	7
67	Finland	1995	Oilcrops Oil, Other	3
67	Finland	1995	Olives	1

FAO country code	Country	Reference year	Food item	Gross food supply (g *capita*$^{-1}$ d^{-1})
67	Finland	1995	Onions	11
67	Finland	1995	Oranges, Mandarines	31
67	Finland	1995	Peas	4
67	Finland	1995	Pelagic Fish	46
67	Finland	1995	Pigmeat	89
67	Finland	1995	Pineapples	4
67	Finland	1995	Potatoes	183
67	Finland	1995	Poultry Meat	26
67	Finland	1995	Rape and Mustard Oil	12
67	Finland	1995	Rice (Milled Equivalent)	6
67	Finland	1995	Rye	43
67	Finland	1995	Soyabean Oil	9
67	Finland	1995	Sugar, Refined Equivalent	94
67	Finland	1995	Sunflowerseed Oil	2
67	Finland	1995	Sweeteners, Other	6
67	Finland	1995	Tomatoes	35
67	Finland	1995	Vegetables, Other	131
67	Finland	1995	Wheat	170
67	Finland	1995	Wine	15
67	Finland	1996	Apples	41
67	Finland	1996	Bananas	29
67	Finland	1996	Barley	32
67	Finland	1996	Beer	225
67	Finland	1996	Beverages, Alcoholic	13
67	Finland	1996	Beverages, Fermented	3
67	Finland	1996	Bovine Meat	52
67	Finland	1996	Butter, Ghee	18
67	Finland	1996	Cereals, Other	1
67	Finland	1996	Cheese	39
67	Finland	1996	Coffee	30
67	Finland	1996	Cream	18
67	Finland	1996	Crustaceans	4
67	Finland	1996	Demersal Fish	12
67	Finland	1996	Eggs	28
67	Finland	1996	Fats, Animals, Raw	2
67	Finland	1996	Freshwater Fish	27
67	Finland	1996	Fruits, Other	39
67	Finland	1996	Grapefruit	2
67	Finland	1996	Grapes	13
67	Finland	1996	Groundnuts (Shelled Eq)	2
67	Finland	1996	Honey	1
67	Finland	1996	Lemons, Limes	2
67	Finland	1996	Marine Fish, Other	3
67	Finland	1996	Meat, Other	2
67	Finland	1996	Milk, Whole	359
67	Finland	1996	Molluscs, Other	1
67	Finland	1996	Mutton & Goat Meat	1
67	Finland	1996	Nuts	4
67	Finland	1996	Oats	16
67	Finland	1996	Offals, Edible	7
67	Finland	1996	Oilcrops Oil, Other	3
67	Finland	1996	Olives	1
67	Finland	1996	Onions	12
67	Finland	1996	Oranges, Mandarines	56
67	Finland	1996	Palm Oil	1
67	Finland	1996	Peas	4
67	Finland	1996	Pelagic Fish	46
67	Finland	1996	Pigmeat	89
67	Finland	1996	Pineapples	7
67	Finland	1996	Potatoes	188
67	Finland	1996	Poultry Meat	30
67	Finland	1996	Rape and Mustard Oil	11
67	Finland	1996	Rice (Milled Equivalent)	11
67	Finland	1996	Rye	43
67	Finland	1996	Soyabean Oil	8
67	Finland	1996	Sugar, Refined Equivalent	90
67	Finland	1996	Sunflowerseed Oil	1
67	Finland	1996	Sweeteners, Other	6
67	Finland	1996	Tea	1
67	Finland	1996	Tomatoes	42
67	Finland	1996	Vegetables, Other	139
67	Finland	1996	Wheat	160
67	Finland	1996	Wine	14
67	Finland	1997	Apples	40
67	Finland	1997	Bananas	30
67	Finland	1997	Barley	25
67	Finland	1997	Beer	229
67	Finland	1997	Beverages, Alcoholic	9
67	Finland	1997	Beverages, Fermented	7
67	Finland	1997	Bovine Meat	53
67	Finland	1997	Butter, Ghee	15
67	Finland	1997	Cheese	37
67	Finland	1997	Cocoa Beans	1
67	Finland	1997	Coconut Oil	1
67	Finland	1997	Coconuts - Incl Copra	1
67	Finland	1997	Coffee	30
67	Finland	1997	Cream	18
67	Finland	1997	Crustaceans	4
67	Finland	1997	Demersal Fish	12
67	Finland	1997	Eggs	27
67	Finland	1997	Fats, Animals, Raw	4
67	Finland	1997	Freshwater Fish	30
67	Finland	1997	Fruits, Other	39
67	Finland	1997	Grapefruit	3
67	Finland	1997	Grapes	12
67	Finland	1997	Groundnuts (Shelled Eq)	1
67	Finland	1997	Honey	1

FAO country code	Country	Reference year	Food item	Gross food supply (g *capita*$^{-1}$ d^{-1})
67	Finland	1997	Lemons, Limes	2
67	Finland	1997	Marine Fish, Other	2
67	Finland	1997	Meat, Other	1
67	Finland	1997	Milk, Whole	395
67	Finland	1997	Molluscs, Other	1
67	Finland	1997	Mutton & Goat Meat	1
67	Finland	1997	Nuts	4
67	Finland	1997	Oats	14
67	Finland	1997	Offals, Edible	5
67	Finland	1997	Oilcrops Oil, Other	4
67	Finland	1997	Olives	1
67	Finland	1997	Onions	9
67	Finland	1997	Oranges, Mandarines	59
67	Finland	1997	Peas	4
67	Finland	1997	Pelagic Fish	41
67	Finland	1997	Pigmeat	90
67	Finland	1997	Pineapples	6
67	Finland	1997	Potatoes	189
67	Finland	1997	Poultry Meat	30
67	Finland	1997	Rape and Mustard Oil	22
67	Finland	1997	Rice (Milled Equivalent)	12
67	Finland	1997	Rye	43
67	Finland	1997	Sugar, Refined Equivalent	90
67	Finland	1997	Sunflowerseed Oil	2
67	Finland	1997	Sweeteners, Other	6
67	Finland	1997	Tea	1
67	Finland	1997	Tomatoes	38
67	Finland	1997	Vegetables, Other	155
67	Finland	1997	Wheat	191
67	Finland	1997	Wine	17
67	Finland	1998	Apples	43
67	Finland	1998	Bananas	29
67	Finland	1998	Barley	38
67	Finland	1998	Beer	224
67	Finland	1998	Beverages, Alcoholic	13
67	Finland	1998	Beverages, Fermented	7
67	Finland	1998	Bovine Meat	54
67	Finland	1998	Butter, Ghee	16
67	Finland	1998	Cheese	41
67	Finland	1998	Cocoa Beans	2
67	Finland	1998	Coffee	31
67	Finland	1998	Cream	18
67	Finland	1998	Crustaceans	4
67	Finland	1998	Demersal Fish	11
67	Finland	1998	Eggs	26
67	Finland	1998	Fats, Animals, Raw	4
67	Finland	1998	Freshwater Fish	27
67	Finland	1998	Fruits, Other	36
67	Finland	1998	Grapefruit	3
67	Finland	1998	Grapes	13
67	Finland	1998	Groundnuts (Shelled Eq)	2
67	Finland	1998	Honey	1
67	Finland	1998	Lemons, Limes	2
67	Finland	1998	Marine Fish, Other	2
67	Finland	1998	Meat, Other	2
67	Finland	1998	Milk, Whole	395
67	Finland	1998	Molluscs, Other	1
67	Finland	1998	Mutton & Goat Meat	1
67	Finland	1998	Nuts	3
67	Finland	1998	Oats	11
67	Finland	1998	Offals, Edible	5
67	Finland	1998	Oilcrops Oil, Other	4
67	Finland	1998	Olives	1
67	Finland	1998	Onions	13
67	Finland	1998	Oranges, Mandarines	64
67	Finland	1998	Peas	3
67	Finland	1998	Pelagic Fish	44
67	Finland	1998	Pigmeat	94
67	Finland	1998	Pineapples	5
67	Finland	1998	Potatoes	188
67	Finland	1998	Poultry Meat	33
67	Finland	1998	Rape and Mustard Oil	23
67	Finland	1998	Rice (Milled Equivalent)	11
67	Finland	1998	Rye	44
67	Finland	1998	Sugar, Refined Equivalent	87
67	Finland	1998	Sunflowerseed Oil	1
67	Finland	1998	Sweeteners, Other	5
67	Finland	1998	Tea	1
67	Finland	1998	Tomatoes	35
67	Finland	1998	Vegetables, Other	128
67	Finland	1998	Wheat	180
67	Finland	1998	Wine	19
67	Finland	1999	Apples	64
67	Finland	1999	Bananas	32
67	Finland	1999	Barley	11
67	Finland	1999	Beer	239
67	Finland	1999	Beverages, Alcoholic	15
67	Finland	1999	Beverages, Fermented	7
67	Finland	1999	Bovine Meat	52
67	Finland	1999	Butter, Ghee	16
67	Finland	1999	Cheese	44
67	Finland	1999	Cocoa Beans	2
67	Finland	1999	Coconut Oil	1
67	Finland	1999	Coffee	33
67	Finland	1999	Cream	17
67	Finland	1999	Crustaceans	4
67	Finland	1999	Demersal Fish	11

FAO country code	Country	Reference year	Food item	Gross food supply (g *capita*$^{-1}$ d^{-1})
67	Finland	1999	Eggs	25
67	Finland	1999	Fats, Animals, Raw	2
67	Finland	1999	Freshwater Fish	32
67	Finland	1999	Fruits, Other	42
67	Finland	1999	Grapefruit	4
67	Finland	1999	Grapes	13
67	Finland	1999	Groundnuts (Shelled Eq)	2
67	Finland	1999	Honey	2
67	Finland	1999	Lemons, Limes	2
67	Finland	1999	Marine Fish, Other	2
67	Finland	1999	Meat, Other	2
67	Finland	1999	Milk, Whole	348
67	Finland	1999	Molluscs, Other	1
67	Finland	1999	Mutton & Goat Meat	1
67	Finland	1999	Nuts	3
67	Finland	1999	Oats	14
67	Finland	1999	Offals, Edible	5
67	Finland	1999	Oilcrops Oil, Other	4
67	Finland	1999	Olives	1
67	Finland	1999	Onions	11
67	Finland	1999	Oranges, Mandarines	68
67	Finland	1999	Peas	3
67	Finland	1999	Pelagic Fish	36
67	Finland	1999	Pigmeat	93
67	Finland	1999	Pineapples	7
67	Finland	1999	Potatoes	192
67	Finland	1999	Poultry Meat	35
67	Finland	1999	Rape and Mustard Oil	20
67	Finland	1999	Rice (Milled Equivalent)	11
67	Finland	1999	Rye	44
67	Finland	1999	Sugar, Refined Equivalent	86
67	Finland	1999	Sunflowerseed Oil	3
67	Finland	1999	Sweeteners, Other	5
67	Finland	1999	Tomatoes	42
67	Finland	1999	Vegetables, Other	148
67	Finland	1999	Wheat	217
67	Finland	1999	Wine	19
67	Finland	2000	Apples	62
67	Finland	2000	Bananas	30
67	Finland	2000	Barley	16
67	Finland	2000	Beer	230
67	Finland	2000	Beverages, Alcoholic	18
67	Finland	2000	Beverages, Fermented	3
67	Finland	2000	Bovine Meat	49
67	Finland	2000	Butter, Ghee	14
67	Finland	2000	Cheese	39
67	Finland	2000	Cocoa Beans	3
67	Finland	2000	Coffee	29
67	Finland	2000	Cream	17
67	Finland	2000	Crustaceans	4
67	Finland	2000	Demersal Fish	11
67	Finland	2000	Eggs	25
67	Finland	2000	Fats, Animals, Raw	2
67	Finland	2000	Freshwater Fish	20
67	Finland	2000	Fruits, Other	40
67	Finland	2000	Grapefruit	3
67	Finland	2000	Grapes	11
67	Finland	2000	Groundnuts (Shelled Eq)	2
67	Finland	2000	Honey	1
67	Finland	2000	Lemons, Limes	2
67	Finland	2000	Marine Fish, Other	2
67	Finland	2000	Meat, Other	2
67	Finland	2000	Milk, Whole	374
67	Finland	2000	Molluscs, Other	1
67	Finland	2000	Mutton & Goat Meat	1
67	Finland	2000	Nuts	3
67	Finland	2000	Oats	9
67	Finland	2000	Offals, Edible	4
67	Finland	2000	Oilcrops Oil, Other	2
67	Finland	2000	Olives	1
67	Finland	2000	Onions	14
67	Finland	2000	Oranges, Mandarines	70
67	Finland	2000	Peas	3
67	Finland	2000	Pelagic Fish	47
67	Finland	2000	Pigmeat	90
67	Finland	2000	Pineapples	6
67	Finland	2000	Potatoes	190
67	Finland	2000	Poultry Meat	37
67	Finland	2000	Rape and Mustard Oil	16
67	Finland	2000	Rice (Milled Equivalent)	11
67	Finland	2000	Rye	43
67	Finland	2000	Soyabean Oil	5
67	Finland	2000	Sugar, Refined Equivalent	91
67	Finland	2000	Sunflowerseed Oil	3
67	Finland	2000	Sweeteners, Other	5
67	Finland	2000	Tomatoes	42
67	Finland	2000	Vegetables, Other	139
67	Finland	2000	Wheat	213
67	Finland	2000	Wine	21
67	Finland	2001	Apples	77
67	Finland	2001	Bananas	30
67	Finland	2001	Barley	16
67	Finland	2001	Beer	243
67	Finland	2001	Beverages, Alcoholic	19
67	Finland	2001	Beverages, Fermented	3
67	Finland	2001	Bovine Meat	50
67	Finland	2001	Butter, Ghee	13

FAO countrycode	Country	Reference year	Food item	Gross food supply (g *capita*$^{-1}$ d^{-1})
67	Finland	2001	Cereals, Other	1
67	Finland	2001	Cheese	43
67	Finland	2001	Cocoa Beans	3
67	Finland	2001	Coffee	30
67	Finland	2001	Cream	18
67	Finland	2001	Crustaceans	5
67	Finland	2001	Demersal Fish	12
67	Finland	2001	Eggs	24
67	Finland	2001	Fats, Animals, Raw	3
67	Finland	2001	Freshwater Fish	26
67	Finland	2001	Fruits, Other	44
67	Finland	2001	Grapefruit	3
67	Finland	2001	Grapes	12
67	Finland	2001	Groundnuts (Shelled Eq)	2
67	Finland	2001	Honey	1
67	Finland	2001	Lemons, Limes	2
67	Finland	2001	Marine Fish, Other	2
67	Finland	2001	Meat, Other	2
67	Finland	2001	Milk, Whole	350
67	Finland	2001	Molluscs, Other	1
67	Finland	2001	Mutton & Goat Meat	1
67	Finland	2001	Nuts	3
67	Finland	2001	Oats	8
67	Finland	2001	Offals, Edible	4
67	Finland	2001	Oilcrops Oil, Other	2
67	Finland	2001	Olives	1
67	Finland	2001	Onions	11
67	Finland	2001	Oranges, Mandarines	72
67	Finland	2001	Peas	3
67	Finland	2001	Pelagic Fish	41
67	Finland	2001	Pigmeat	87
67	Finland	2001	Pineapples	7
67	Finland	2001	Potatoes	194
67	Finland	2001	Poultry Meat	43
67	Finland	2001	Rape and Mustard Oil	11
67	Finland	2001	Rice (Milled Equivalent)	12
67	Finland	2001	Rye	42
67	Finland	2001	Soyabean Oil	13
67	Finland	2001	Sugar, Refined Equivalent	82
67	Finland	2001	Sunflowerseed Oil	3
67	Finland	2001	Sweeteners, Other	5
67	Finland	2001	Tea	1
67	Finland	2001	Tomatoes	44
67	Finland	2001	Vegetables, Other	138
67	Finland	2001	Wheat	218
67	Finland	2001	Wine	23
67	Finland	2002	Apples	61
67	Finland	2002	Bananas	31
67	Finland	2002	Barley	12
67	Finland	2002	Beer	243
67	Finland	2002	Beverages, Alcoholic	19
67	Finland	2002	Beverages, Fermented	3
67	Finland	2002	Bovine Meat	49
67	Finland	2002	Butter, Ghee	13
67	Finland	2002	Cheese	43
67	Finland	2002	Cocoa Beans	3
67	Finland	2002	Coconut Oil	1
67	Finland	2002	Coffee	30
67	Finland	2002	Cream	18
67	Finland	2002	Crustaceans	4
67	Finland	2002	Demersal Fish	10
67	Finland	2002	Eggs	24
67	Finland	2002	Fats, Animals, Raw	3
67	Finland	2002	Freshwater Fish	27
67	Finland	2002	Fruits, Other	44
67	Finland	2002	Grapefruit	3
67	Finland	2002	Grapes	12
67	Finland	2002	Groundnuts (Shelled Eq)	2
67	Finland	2002	Honey	1
67	Finland	2002	Lemons, Limes	2
67	Finland	2002	Marine Fish, Other	3
67	Finland	2002	Meat, Other	2
67	Finland	2002	Milk, Whole	351
67	Finland	2002	Molluscs, Other	1
67	Finland	2002	Mutton & Goat Meat	1
67	Finland	2002	Nuts	3
67	Finland	2002	Oats	11
67	Finland	2002	Offals, Edible	5
67	Finland	2002	Oilcrops Oil, Other	2
67	Finland	2002	Olive Oil	1
67	Finland	2002	Olives	1
67	Finland	2002	Onions	13
67	Finland	2002	Oranges, Mandarines	67
67	Finland	2002	Peas	3
67	Finland	2002	Pelagic Fish	43
67	Finland	2002	Pigmeat	88
67	Finland	2002	Pineapples	9
67	Finland	2002	Potatoes	191
67	Finland	2002	Poultry Meat	46
67	Finland	2002	Rape and Mustard Oil	19
67	Finland	2002	Rice (Milled Equivalent)	14
67	Finland	2002	Rye	41
67	Finland	2002	Soyabean Oil	3
67	Finland	2002	Sugar, Refined Equivalent	79
67	Finland	2002	Sunflowerseed Oil	4
67	Finland	2002	Sweeteners, Other	8
67	Finland	2002	Tomatoes	45

FAO country code	Country	Reference year	Food item	Gross food supply (g *capita*$^{-1}$ d^{-1})
67	Finland	2002	Vegetables, Other	140
67	Finland	2002	Wheat	213
67	Finland	2002	Wine	24
67	Finland	2003	Apples	70
67	Finland	2003	Bananas	32
67	Finland	2003	Barley	13
67	Finland	2003	Beer	241
67	Finland	2003	Beverages, Alcoholic	19
67	Finland	2003	Beverages, Fermented	3
67	Finland	2003	Bovine Meat	50
67	Finland	2003	Butter, Ghee	11
67	Finland	2003	Cheese	44
67	Finland	2003	Citrus, Other	1
67	Finland	2003	Cocoa Beans	4
67	Finland	2003	Coconut Oil	2
67	Finland	2003	Coffee	31
67	Finland	2003	Cream	18
67	Finland	2003	Crustaceans	4
67	Finland	2003	Demersal Fish	10
67	Finland	2003	Eggs	23
67	Finland	2003	Fats, Animals, Raw	2
67	Finland	2003	Freshwater Fish	32
67	Finland	2003	Fruits, Other	44
67	Finland	2003	Grapefruit	3
67	Finland	2003	Grapes	12
67	Finland	2003	Groundnuts (Shelled Eq)	2
67	Finland	2003	Honey	1
67	Finland	2003	Lemons, Limes	2
67	Finland	2003	Marine Fish, Other	4
67	Finland	2003	Meat, Other	3
67	Finland	2003	Milk, Whole	344
67	Finland	2003	Molluscs, Other	1
67	Finland	2003	Mutton & Goat Meat	1
67	Finland	2003	Nuts	3
67	Finland	2003	Oats	11
67	Finland	2003	Offals, Edible	4
67	Finland	2003	Oilcrops Oil, Other	3
67	Finland	2003	Olive Oil	1
67	Finland	2003	Olives	1
67	Finland	2003	Onions	11
67	Finland	2003	Oranges, Mandarines	75
67	Finland	2003	Peas	3
67	Finland	2003	Pelagic Fish	39
67	Finland	2003	Pigmeat	88
67	Finland	2003	Pineapples	12
67	Finland	2003	Potatoes	197
67	Finland	2003	Poultry Meat	49
67	Finland	2003	Rape and Mustard Oil	21
67	Finland	2003	Rice (Milled Equivalent)	12
67	Finland	2003	Rye	40
67	Finland	2003	Sugar, Refined Equivalent	79
67	Finland	2003	Sunflowerseed Oil	3
67	Finland	2003	Sweeteners, Other	7
67	Finland	2003	Tea	1
67	Finland	2003	Tomatoes	44
67	Finland	2003	Vegetables, Other	144
67	Finland	2003	Wheat	215
67	Finland	2003	Wine	27
67	Finland	2004	Apples	70
67	Finland	2004	Bananas	34
67	Finland	2004	Barley	12
67	Finland	2004	Beer	263
67	Finland	2004	Beverages, Alcoholic	18
67	Finland	2004	Beverages, Fermented	2
67	Finland	2004	Bovine Meat	52
67	Finland	2004	Butter, Ghee	11
67	Finland	2004	Cereals, Other	1
67	Finland	2004	Cheese	39
67	Finland	2004	Citrus, Other	1
67	Finland	2004	Cocoa Beans	4
67	Finland	2004	Coffee	32
67	Finland	2004	Cream	18
67	Finland	2004	Crustaceans	5
67	Finland	2004	Demersal Fish	9
67	Finland	2004	Eggs	23
67	Finland	2004	Fats, Animals, Raw	1
67	Finland	2004	Freshwater Fish	39
67	Finland	2004	Fruits, Other	45
67	Finland	2004	Grapefruit	3
67	Finland	2004	Grapes	14
67	Finland	2004	Groundnuts (Shelled Eq)	2
67	Finland	2004	Honey	1
67	Finland	2004	Lemons, Limes	2
67	Finland	2004	Marine Fish, Other	4
67	Finland	2004	Meat, Other	3
67	Finland	2004	Milk, Whole	353
67	Finland	2004	Molluscs, Other	1
67	Finland	2004	Mutton & Goat Meat	1
67	Finland	2004	Nuts	2
67	Finland	2004	Oats	10
67	Finland	2004	Offals, Edible	5
67	Finland	2004	Oilcrops Oil, Other	2
67	Finland	2004	Olive Oil	1
67	Finland	2004	Olives	1
67	Finland	2004	Onions	15
67	Finland	2004	Oranges, Mandarines	82
67	Finland	2004	Peas	3

FAO countrycode	Country	Reference year	Food item	Gross food supply (g *capita*$^{-1}$ d^{-1})
67	Finland	2004	Pelagic Fish	37
67	Finland	2004	Pigmeat	90
67	Finland	2004	Pineapples	12
67	Finland	2004	Potatoes	200
67	Finland	2004	Poultry Meat	49
67	Finland	2004	Rape and Mustard Oil	21
67	Finland	2004	Rice (Milled Equivalent)	10
67	Finland	2004	Rye	42
67	Finland	2004	Soyabean Oil	3
67	Finland	2004	Sugar, Refined Equivalent	79
67	Finland	2004	Sunflowerseed Oil	2
67	Finland	2004	Sweeteners, Other	9
67	Finland	2004	Tea	1
67	Finland	2004	Tomatoes	45
67	Finland	2004	Vegetables, Other	146
67	Finland	2004	Wheat	220
67	Finland	2004	Wine	27
67	Finland	2005	Apples	66
67	Finland	2005	Bananas	32
67	Finland	2005	Barley	12
67	Finland	2005	Beer	244
67	Finland	2005	Beverages, Alcoholic	18
67	Finland	2005	Beverages, Fermented	4
67	Finland	2005	Bovine Meat	52
67	Finland	2005	Butter, Ghee	11
67	Finland	2005	Cereals, Other	1
67	Finland	2005	Cheese	40
67	Finland	2005	Cocoa Beans	4
67	Finland	2005	Coconut Oil	1
67	Finland	2005	Coffee	33
67	Finland	2005	Cream	18
67	Finland	2005	Crustaceans	5
67	Finland	2005	Demersal Fish	7
67	Finland	2005	Eggs	23
67	Finland	2005	Fats, Animals, Raw	2
67	Finland	2005	Freshwater Fish	32
67	Finland	2005	Fruits, Other	50
67	Finland	2005	Grapefruit	2
67	Finland	2005	Grapes	13
67	Finland	2005	Groundnuts (Shelled Eq)	2
67	Finland	2005	Honey	2
67	Finland	2005	Lemons, Limes	2
67	Finland	2005	Marine Fish, Other	3
67	Finland	2005	Meat, Other	3
67	Finland	2005	Milk, Whole	351
67	Finland	2005	Molluscs, Other	1
67	Finland	2005	Mutton & Goat Meat	1
67	Finland	2005	Nuts	3
67	Finland	2005	Oats	19
67	Finland	2005	Offals, Edible	5
67	Finland	2005	Oilcrops Oil, Other	2
67	Finland	2005	Olive Oil	1
67	Finland	2005	Olives	1
67	Finland	2005	Onions	13
67	Finland	2005	Oranges, Mandarines	82
67	Finland	2005	Peas	3
67	Finland	2005	Pelagic Fish	40
67	Finland	2005	Pigmeat	92
67	Finland	2005	Pineapples	10
67	Finland	2005	Potatoes	197
67	Finland	2005	Poultry Meat	49
67	Finland	2005	Rape and Mustard Oil	24
67	Finland	2005	Rice (Milled Equivalent)	13
67	Finland	2005	Rye	42
67	Finland	2005	Sugar, Refined Equivalent	75
67	Finland	2005	Sunflowerseed Oil	2
67	Finland	2005	Sweeteners, Other	8
67	Finland	2005	Tea	1
67	Finland	2005	Tomatoes	44
67	Finland	2005	Vegetables, Other	159
67	Finland	2005	Wheat	215
67	Finland	2005	Wine	29
67	Finland	2006	Apples	67
67	Finland	2006	Bananas	31
67	Finland	2006	Barley	12
67	Finland	2006	Beer	229
67	Finland	2006	Beverages, Alcoholic	17
67	Finland	2006	Beverages, Fermented	5
67	Finland	2006	Bovine Meat	52
67	Finland	2006	Butter, Ghee	12
67	Finland	2006	Cereals, Other	1
67	Finland	2006	Cheese	40
67	Finland	2006	Citrus, Other	2
67	Finland	2006	Cocoa Beans	5
67	Finland	2006	Coffee	32
67	Finland	2006	Cream	18
67	Finland	2006	Crustaceans	5
67	Finland	2006	Demersal Fish	8
67	Finland	2006	Eggs	23
67	Finland	2006	Fats, Animals, Raw	2
67	Finland	2006	Freshwater Fish	33
67	Finland	2006	Fruits, Other	47
67	Finland	2006	Grapefruit	2
67	Finland	2006	Grapes	13
67	Finland	2006	Groundnuts (Shelled Eq)	3
67	Finland	2006	Honey	2
67	Finland	2006	Lemons, Limes	2

FAO country code	Country	Reference year	Food item	Gross food supply (g *capita*$^{-1}$ d^{-1})
67	Finland	2006	Marine Fish, Other	3
67	Finland	2006	Meat, Other	3
67	Finland	2006	Milk, Whole	357
67	Finland	2006	Molluscs, Other	1
67	Finland	2006	Mutton & Goat Meat	1
67	Finland	2006	Nuts	3
67	Finland	2006	Oats	20
67	Finland	2006	Offals, Edible	5
67	Finland	2006	Oilcrops Oil, Other	3
67	Finland	2006	Olive Oil	1
67	Finland	2006	Olives	1
67	Finland	2006	Onions	12
67	Finland	2006	Oranges, Mandarines	69
67	Finland	2006	Palm Oil	5
67	Finland	2006	Peas	3
67	Finland	2006	Pelagic Fish	40
67	Finland	2006	Pigmeat	91
67	Finland	2006	Pineapples	11
67	Finland	2006	Potatoes	196
67	Finland	2006	Poultry Meat	47
67	Finland	2006	Rape and Mustard Oil	19
67	Finland	2006	Rice (Milled Equivalent)	12
67	Finland	2006	Rye	45
67	Finland	2006	Sugar, Refined Equivalent	73
67	Finland	2006	Sunflowerseed Oil	3
67	Finland	2006	Sweeteners, Other	9
67	Finland	2006	Tea	1
67	Finland	2006	Tomatoes	43
67	Finland	2006	Vegetables, Other	150
67	Finland	2006	Wheat	226
67	Finland	2006	Wine	29
67	Finland	2007	Apples	71
67	Finland	2007	Bananas	35
67	Finland	2007	Barley	12
67	Finland	2007	Beer	237
67	Finland	2007	Beverages, Alcoholic	14
67	Finland	2007	Beverages, Fermented	6
67	Finland	2007	Bovine Meat	51
67	Finland	2007	Butter, Ghee	10
67	Finland	2007	Cereals, Other	1
67	Finland	2007	Cheese	44
67	Finland	2007	Citrus, Other	1
67	Finland	2007	Cocoa Beans	6
67	Finland	2007	Coconut Oil	1
67	Finland	2007	Coffee	33
67	Finland	2007	Cream	19
67	Finland	2007	Crustaceans	5
67	Finland	2007	Demersal Fish	8
67	Finland	2007	Eggs	23
67	Finland	2007	Fats, Animals, Raw	2
67	Finland	2007	Freshwater Fish	41
67	Finland	2007	Fruits, Other	51
67	Finland	2007	Grapefruit	2
67	Finland	2007	Grapes	15
67	Finland	2007	Groundnuts (Shelled Eq)	2
67	Finland	2007	Honey	1
67	Finland	2007	Lemons, Limes	2
67	Finland	2007	Marine Fish, Other	4
67	Finland	2007	Meat, Other	5
67	Finland	2007	Milk, Whole	349
67	Finland	2007	Molluscs, Other	1
67	Finland	2007	Mutton & Goat Meat	1
67	Finland	2007	Nuts	4
67	Finland	2007	Oats	14
67	Finland	2007	Offals, Edible	4
67	Finland	2007	Oilcrops Oil, Other	3
67	Finland	2007	Olive Oil	1
67	Finland	2007	Olives	2
67	Finland	2007	Onions	14
67	Finland	2007	Oranges, Mandarines	67
67	Finland	2007	Peas	3
67	Finland	2007	Pelagic Fish	40
67	Finland	2007	Pigmeat	96
67	Finland	2007	Pineapples	12
67	Finland	2007	Plantains	1
67	Finland	2007	Potatoes	188
67	Finland	2007	Poultry Meat	51
67	Finland	2007	Rape and Mustard Oil	21
67	Finland	2007	Rice (Milled Equivalent)	13
67	Finland	2007	Rye	46
67	Finland	2007	Soyabean Oil	2
67	Finland	2007	Sugar, Refined Equivalent	76
67	Finland	2007	Sunflowerseed Oil	2
67	Finland	2007	Sweeteners, Other	9
67	Finland	2007	Tea	1
67	Finland	2007	Tomatoes	44
67	Finland	2007	Vegetables, Other	158
67	Finland	2007	Wheat	221
67	Finland	2007	Wine	32
67	Finland	2008	Apples	63
67	Finland	2008	Bananas	28
67	Finland	2008	Barley	13
67	Finland	2008	Beer	233
67	Finland	2008	Beverages, Alcoholic	11
67	Finland	2008	Beverages, Fermented	2
67	Finland	2008	Bovine Meat	50
67	Finland	2008	Butter, Ghee	12

FAO countrycode	Country	Reference year	Food item	Gross food supply (g *capita*$^{-1}$ d^{-1})
67	Finland	2008	Cereals, Other	3
67	Finland	2008	Cheese	49
67	Finland	2008	Citrus, Other	1
67	Finland	2008	Cocoa Beans	6
67	Finland	2008	Coconut Oil	1
67	Finland	2008	Coffee	35
67	Finland	2008	Cream	19
67	Finland	2008	Crustaceans	5
67	Finland	2008	Demersal Fish	8
67	Finland	2008	Eggs	24
67	Finland	2008	Fats, Animals, Raw	2
67	Finland	2008	Freshwater Fish	40
67	Finland	2008	Fruits, Other	51
67	Finland	2008	Grapefruit	2
67	Finland	2008	Grapes	16
67	Finland	2008	Groundnuts (Shelled Eq)	2
67	Finland	2008	Honey	1
67	Finland	2008	Lemons, Limes	2
67	Finland	2008	Marine Fish, Other	4
67	Finland	2008	Meat, Other	5
67	Finland	2008	Milk, Whole	353
67	Finland	2008	Molluscs, Other	1
67	Finland	2008	Mutton & Goat Meat	1
67	Finland	2008	Nuts	4
67	Finland	2008	Oats	18
67	Finland	2008	Offals, Edible	4
67	Finland	2008	Oilcrops Oil, Other	2
67	Finland	2008	Olive Oil	1
67	Finland	2008	Olives	2
67	Finland	2008	Onions	13
67	Finland	2008	Oranges, Mandarines	63
67	Finland	2008	Peas	3
67	Finland	2008	Pelagic Fish	37
67	Finland	2008	Pigmeat	93
67	Finland	2008	Pineapples	13
67	Finland	2008	Potatoes	188
67	Finland	2008	Poultry Meat	54
67	Finland	2008	Rape and Mustard Oil	19
67	Finland	2008	Rice (Milled Equivalent)	13
67	Finland	2008	Rye	44
67	Finland	2008	Soyabean Oil	3
67	Finland	2008	Spices, Other	1
67	Finland	2008	Sugar, Refined Equivalent	64
67	Finland	2008	Sunflowerseed Oil	2
67	Finland	2008	Sweet Potatoes	1
67	Finland	2008	Sweeteners, Other	9
67	Finland	2008	Tea	1
67	Finland	2008	Tomatoes	47
67	Finland	2008	Vegetables, Other	154
67	Finland	2008	Wheat	225
67	Finland	2008	Wine	33
67	Finland	2009	Apples	61
67	Finland	2009	Bananas	26
67	Finland	2009	Barley	13
67	Finland	2009	Beer	229
67	Finland	2009	Beverages, Alcoholic	11
67	Finland	2009	Beverages, Fermented	3
67	Finland	2009	Bovine Meat	50
67	Finland	2009	Butter, Ghee	10
67	Finland	2009	Cereals, Other	5
67	Finland	2009	Cheese	48
67	Finland	2009	Citrus, Other	1
67	Finland	2009	Cocoa Beans	6
67	Finland	2009	Coconut Oil	1
67	Finland	2009	Coffee	33
67	Finland	2009	Cream	19
67	Finland	2009	Crustaceans	5
67	Finland	2009	Demersal Fish	7
67	Finland	2009	Eggs	23
67	Finland	2009	Fats, Animals, Raw	2
67	Finland	2009	Freshwater Fish	37
67	Finland	2009	Fruits, Other	58
67	Finland	2009	Grapefruit	2
67	Finland	2009	Grapes	17
67	Finland	2009	Groundnuts (Shelled Eq)	3
67	Finland	2009	Honey	1
67	Finland	2009	Lemons, Limes	2
67	Finland	2009	Marine Fish, Other	4
67	Finland	2009	Meat, Other	5
67	Finland	2009	Milk, Whole	351
67	Finland	2009	Molluscs, Other	1
67	Finland	2009	Mutton & Goat Meat	1
67	Finland	2009	Nuts	5
67	Finland	2009	Oats	16
67	Finland	2009	Offals, Edible	5
67	Finland	2009	Oilcrops Oil, Other	2
67	Finland	2009	Olive Oil	1
67	Finland	2009	Olives	2
67	Finland	2009	Onions	14
67	Finland	2009	Oranges, Mandarines	68
67	Finland	2009	Peas	3
67	Finland	2009	Pelagic Fish	38
67	Finland	2009	Pigmeat	93
67	Finland	2009	Pineapples	11
67	Finland	2009	Potatoes	183
67	Finland	2009	Poultry Meat	50
67	Finland	2009	Rape and Mustard Oil	22

FAO country code	Country	Reference year	Food item	Gross food supply (g $capita^{-1}$ d^{-1})
67	Finland	2009	Rice (Milled Equivalent)	12
67	Finland	2009	Rye	43
67	Finland	2009	Spices, Other	1
67	Finland	2009	Sugar, Refined Equivalent	75
67	Finland	2009	Sunflowerseed Oil	3
67	Finland	2009	Sweet Potatoes	1
67	Finland	2009	Sweeteners, Other	8
67	Finland	2009	Tea	1
67	Finland	2009	Tomatoes	48
67	Finland	2009	Vegetables, Other	170
67	Finland	2009	Wheat	214
67	Finland	2009	Wine	33
67	Finland	2010	Apples	63
67	Finland	2010	Bananas	27
67	Finland	2010	Barley	13
67	Finland	2010	Beer	227
67	Finland	2010	Beverages, Alcoholic	11
67	Finland	2010	Beverages, Fermented	2
67	Finland	2010	Bovine Meat	51
67	Finland	2010	Butter, Ghee	13
67	Finland	2010	Cereals, Other	7
67	Finland	2010	Cheese	48
67	Finland	2010	Citrus, Other	2
67	Finland	2010	Cocoa Beans	8
67	Finland	2010	Coffee	34
67	Finland	2010	Cream	17
67	Finland	2010	Crustaceans	6
67	Finland	2010	Demersal Fish	8
67	Finland	2010	Eggs	24
67	Finland	2010	Fats, Animals, Raw	2
67	Finland	2010	Freshwater Fish	37
67	Finland	2010	Fruits, Other	51
67	Finland	2010	Grapefruit	2
67	Finland	2010	Grapes	16
67	Finland	2010	Groundnuts (Shelled Eq)	3
67	Finland	2010	Honey	2
67	Finland	2010	Lemons, Limes	2
67	Finland	2010	Marine Fish, Other	4
67	Finland	2010	Meat, Other	5
67	Finland	2010	Milk, Whole	350
67	Finland	2010	Molluscs, Other	1
67	Finland	2010	Mutton & Goat Meat	1
67	Finland	2010	Nuts	5
67	Finland	2010	Oats	17
67	Finland	2010	Offals, Edible	4
67	Finland	2010	Oilcrops Oil, Other	2
67	Finland	2010	Olive Oil	1
67	Finland	2010	Olives	2
67	Finland	2010	Onions	13
67	Finland	2010	Oranges, Mandarines	70
67	Finland	2010	Palm Oil	3
67	Finland	2010	Peas	3
67	Finland	2010	Pelagic Fish	41
67	Finland	2010	Pigmeat	100
67	Finland	2010	Pineapples	12
67	Finland	2010	Potatoes	184
67	Finland	2010	Poultry Meat	48
67	Finland	2010	Rape and Mustard Oil	20
67	Finland	2010	Rice (Milled Equivalent)	12
67	Finland	2010	Rye	45
67	Finland	2010	Sugar, Refined Equivalent	67
67	Finland	2010	Sunflowerseed Oil	2
67	Finland	2010	Sweet Potatoes	1
67	Finland	2010	Sweeteners, Other	8
67	Finland	2010	Tea	1
67	Finland	2010	Tomatoes	42
67	Finland	2010	Vegetables, Other	165
67	Finland	2010	Wheat	223
67	Finland	2010	Wine	33
67	Finland	2011	Apples	63
67	Finland	2011	Bananas	34
67	Finland	2011	Barley	13
67	Finland	2011	Beer	235
67	Finland	2011	Beverages, Alcoholic	11
67	Finland	2011	Beverages, Fermented	2
67	Finland	2011	Bovine Meat	52
67	Finland	2011	Butter, Ghee	15
67	Finland	2011	Cereals, Other	3
67	Finland	2011	Cheese	53
67	Finland	2011	Citrus, Other	1
67	Finland	2011	Cocoa Beans	5
67	Finland	2011	Coffee	33
67	Finland	2011	Cream	18
67	Finland	2011	Crustaceans	6
67	Finland	2011	Demersal Fish	8
67	Finland	2011	Eggs	24
67	Finland	2011	Fats, Animals, Raw	2
67	Finland	2011	Freshwater Fish	37
67	Finland	2011	Fruits, Other	56
67	Finland	2011	Grapefruit	2
67	Finland	2011	Grapes	14
67	Finland	2011	Groundnuts (Shelled Eq)	3
67	Finland	2011	Honey	1
67	Finland	2011	Lemons, Limes	4
67	Finland	2011	Marine Fish, Other	4
67	Finland	2011	Meat, Other	6
67	Finland	2011	Milk, Whole	339

FAO country code	Country	Reference year	Food item	Gross food supply (g $capita^{-1}$ d^{-1})
67	Finland	2011	Molluscs, Other	1
67	Finland	2011	Mutton & Goat Meat	2
67	Finland	2011	Nuts	6
67	Finland	2011	Oats	18
67	Finland	2011	Offals, Edible	4
67	Finland	2011	Oilcrops Oil, Other	2
67	Finland	2011	Olive Oil	1
67	Finland	2011	Olives	2
67	Finland	2011	Onions	16
67	Finland	2011	Oranges, Mandarines	70
67	Finland	2011	Palm Oil	3
67	Finland	2011	Peas	3
67	Finland	2011	Pelagic Fish	41
67	Finland	2011	Pigmeat	95
67	Finland	2011	Pineapples	13
67	Finland	2011	Plantains	2
67	Finland	2011	Potatoes	183
67	Finland	2011	Poultry Meat	50
67	Finland	2011	Rape and Mustard Oil	18
67	Finland	2011	Rice (Milled Equivalent)	12
67	Finland	2011	Rye	45
67	Finland	2011	Spices, Other	1
67	Finland	2011	Sugar, Refined Equivalent	68
67	Finland	2011	Sunflowerseed Oil	3
67	Finland	2011	Sweet Potatoes	1
67	Finland	2011	Sweeteners, Other	9
67	Finland	2011	Tea	1
67	Finland	2011	Tomatoes	47
67	Finland	2011	Vegetables, Other	178
67	Finland	2011	Wheat	214
67	Finland	2011	Wine	33
68	France	1992	Apples	35
68	France	1992	Bananas	22
68	France	1992	Barley	1
68	France	1992	Beans	3
68	France	1992	Beer	95
68	France	1992	Beverages, Alcoholic	7
68	France	1992	Beverages, Fermented	41
68	France	1992	Bovine Meat	83
68	France	1992	Butter, Ghee	23
68	France	1992	Cephalopods	1
68	France	1992	Cheese	59
68	France	1992	Cocoa Beans	8
68	France	1992	Coconut Oil	1
68	France	1992	Coconuts - Incl Copra	2
68	France	1992	Coffee	16
68	France	1992	Cream	12
68	France	1992	Crustaceans	10
68	France	1992	Demersal Fish	28
68	France	1992	Eggs	40
68	France	1992	Fats, Animals, Raw	12
68	France	1992	Fish, Body Oil	1
68	France	1992	Freshwater Fish	8
68	France	1992	Fruits, Other	84
68	France	1992	Grapefruit	12
68	France	1992	Grapes	6
68	France	1992	Groundnut Oil	4
68	France	1992	Groundnuts (Shelled Eq)	3
68	France	1992	Honey	1
68	France	1992	Lemons, Limes	7
68	France	1992	Maize	35
68	France	1992	Maize Germ Oil	1
68	France	1992	Marine Fish, Other	2
68	France	1992	Meat, Other	18
68	France	1992	Milk, Whole	271
68	France	1992	Molluscs, Other	18
68	France	1992	Mutton & Goat Meat	12
68	France	1992	Nuts	10
68	France	1992	Offals, Edible	27
68	France	1992	Oilcrops Oil, Other	5
68	France	1992	Olive Oil	2
68	France	1992	Olives	2
68	France	1992	Onions	13
68	France	1992	Oranges, Mandarines	74
68	France	1992	Palm Oil	4
68	France	1992	Palmkernel Oil	1
68	France	1992	Peas	2
68	France	1992	Pelagic Fish	16
68	France	1992	Pigmeat	96
68	France	1992	Pineapples	6
68	France	1992	Potatoes	206
68	France	1992	Poultry Meat	60
68	France	1992	Pulses, Other	1
68	France	1992	Rape and Mustard Oil	8
68	France	1992	Rice (Milled Equivalent)	10
68	France	1992	Rye	2
68	France	1992	Soyabean Oil	1
68	France	1992	Sugar, Refined Equivalent	93
68	France	1992	Sunflowerseed Oil	20
68	France	1992	Sweeteners, Other	2
68	France	1992	Tea	1
68	France	1992	Tomatoes	63
68	France	1992	Vegetables, Other	251
68	France	1992	Wheat	240
68	France	1992	Wine	179
68	France	1993	Apples	33
68	France	1993	Bananas	22

FAO country code	Country	Reference year	Food item	Gross food supply (g *capita*$^{-1}$ d^{-1})
68	France	1993	Barley	1
68	France	1993	Beans	3
68	France	1993	Beer	94
68	France	1993	Beverages, Alcoholic	7
68	France	1993	Beverages, Fermented	41
68	France	1993	Bovine Meat	72
68	France	1993	Butter, Ghee	22
68	France	1993	Cephalopods	1
68	France	1993	Cheese	60
68	France	1993	Cocoa Beans	8
68	France	1993	Coconut Oil	1
68	France	1993	Coconuts - Incl Copra	1
68	France	1993	Coffee	17
68	France	1993	Cream	12
68	France	1993	Crustaceans	10
68	France	1993	Demersal Fish	28
68	France	1993	Eggs	40
68	France	1993	Fats, Animals, Raw	12
68	France	1993	Freshwater Fish	8
68	France	1993	Fruits, Other	72
68	France	1993	Grapefruit	11
68	France	1993	Grapes	7
68	France	1993	Groundnut Oil	4
68	France	1993	Groundnuts (Shelled Eq)	3
68	France	1993	Honey	1
68	France	1993	Lemons, Limes	5
68	France	1993	Maize	35
68	France	1993	Maize Germ Oil	1
68	France	1993	Marine Fish, Other	2
68	France	1993	Meat, Other	18
68	France	1993	Milk, Whole	247
68	France	1993	Molluscs, Other	18
68	France	1993	Mutton & Goat Meat	12
68	France	1993	Nuts	10
68	France	1993	Offals, Edible	27
68	France	1993	Oilcrops Oil, Other	6
68	France	1993	Olive Oil	2
68	France	1993	Olives	2
68	France	1993	Onions	12
68	France	1993	Oranges, Mandarines	75
68	France	1993	Palm Oil	2
68	France	1993	Peas	1
68	France	1993	Pelagic Fish	16
68	France	1993	Pigmeat	98
68	France	1993	Pineapples	5
68	France	1993	Potatoes	203
68	France	1993	Poultry Meat	58
68	France	1993	Pulses, Other	2
68	France	1993	Rape and Mustard Oil	7
68	France	1993	Rice (Milled Equivalent)	9
68	France	1993	Rye	1
68	France	1993	Soyabean Oil	4
68	France	1993	Sugar, Refined Equivalent	90
68	France	1993	Sunflowerseed Oil	20
68	France	1993	Sweeteners, Other	2
68	France	1993	Tea	1
68	France	1993	Tomatoes	58
68	France	1993	Vegetables, Other	269
68	France	1993	Wheat	256
68	France	1993	Wine	175
68	France	1994	Apples	32
68	France	1994	Bananas	18
68	France	1994	Barley	1
68	France	1994	Beans	3
68	France	1994	Beer	93
68	France	1994	Beverages, Alcoholic	7
68	France	1994	Beverages, Fermented	38
68	France	1994	Bovine Meat	71
68	France	1994	Butter, Ghee	23
68	France	1994	Cephalopods	1
68	France	1994	Cheese	60
68	France	1994	Cocoa Beans	8
68	France	1994	Coconuts - Incl Copra	2
68	France	1994	Coffee	16
68	France	1994	Cream	13
68	France	1994	Crustaceans	9
68	France	1994	Demersal Fish	29
68	France	1994	Eggs	41
68	France	1994	Fats, Animals, Raw	12
68	France	1994	Fish, Body Oil	1
68	France	1994	Freshwater Fish	8
68	France	1994	Fruits, Other	81
68	France	1994	Grapefruit	13
68	France	1994	Grapes	10
68	France	1994	Groundnut Oil	4
68	France	1994	Groundnuts (Shelled Eq)	2
68	France	1994	Honey	1
68	France	1994	Lemons, Limes	6
68	France	1994	Maize	35
68	France	1994	Maize Germ Oil	1
68	France	1994	Marine Fish, Other	2
68	France	1994	Meat, Other	17
68	France	1994	Milk, Whole	232
68	France	1994	Molluscs, Other	18
68	France	1994	Mutton & Goat Meat	12
68	France	1994	Nuts	9
68	France	1994	Oats	1

FAO country code	Country	Reference year	Food item	Gross food supply (g *capita*$^{-1}$ d^{-1})
68	France	1994	Offals, Edible	26
68	France	1994	Oilcrops Oil, Other	7
68	France	1994	Olive Oil	2
68	France	1994	Olives	2
68	France	1994	Onions	13
68	France	1994	Oranges, Mandarines	84
68	France	1994	Palm Oil	1
68	France	1994	Palmkernel Oil	1
68	France	1994	Peas	1
68	France	1994	Pelagic Fish	16
68	France	1994	Pigmeat	98
68	France	1994	Pineapples	5
68	France	1994	Potatoes	187
68	France	1994	Poultry Meat	63
68	France	1994	Pulses, Other	2
68	France	1994	Rape and Mustard Oil	8
68	France	1994	Rice (Milled Equivalent)	10
68	France	1994	Rye	2
68	France	1994	Soyabean Oil	3
68	France	1994	Sugar, Refined Equivalent	88
68	France	1994	Sunflowerseed Oil	20
68	France	1994	Sweeteners, Other	4
68	France	1994	Tea	1
68	France	1994	Tomatoes	63
68	France	1994	Vegetables, Other	197
68	France	1994	Wheat	256
68	France	1994	Wine	171
68	France	1995	Apples	28
68	France	1995	Bananas	25
68	France	1995	Barley	1
68	France	1995	Beans	3
68	France	1995	Beer	94
68	France	1995	Beverages, Alcoholic	7
68	France	1995	Beverages, Fermented	37
68	France	1995	Bovine Meat	76
68	France	1995	Butter, Ghee	24
68	France	1995	Cephalopods	2
68	France	1995	Cheese	60
68	France	1995	Cocoa Beans	8
68	France	1995	Coconuts - Incl Copra	1
68	France	1995	Coffee	14
68	France	1995	Cream	11
68	France	1995	Crustaceans	10
68	France	1995	Demersal Fish	29
68	France	1995	Eggs	43
68	France	1995	Fats, Animals, Raw	13
68	France	1995	Fish, Body Oil	1
68	France	1995	Freshwater Fish	8
68	France	1995	Fruits, Other	82
68	France	1995	Grapefruit	14
68	France	1995	Grapes	10
68	France	1995	Groundnut Oil	4
68	France	1995	Groundnuts (Shelled Eq)	3
68	France	1995	Honey	1
68	France	1995	Lemons, Limes	6
68	France	1995	Maize	35
68	France	1995	Maize Germ Oil	1
68	France	1995	Marine Fish, Other	2
68	France	1995	Meat, Other	17
68	France	1995	Milk, Whole	220
68	France	1995	Molluscs, Other	18
68	France	1995	Mutton & Goat Meat	12
68	France	1995	Nuts	10
68	France	1995	Oats	1
68	France	1995	Offals, Edible	26
68	France	1995	Oilcrops Oil, Other	6
68	France	1995	Olive Oil	2
68	France	1995	Olives	2
68	France	1995	Onions	14
68	France	1995	Oranges, Mandarines	88
68	France	1995	Palm Oil	1
68	France	1995	Palmkernel Oil	1
68	France	1995	Peas	1
68	France	1995	Pelagic Fish	16
68	France	1995	Pigmeat	98
68	France	1995	Pineapples	5
68	France	1995	Potatoes	194
68	France	1995	Poultry Meat	65
68	France	1995	Pulses, Other	2
68	France	1995	Rape and Mustard Oil	8
68	France	1995	Rice (Milled Equivalent)	9
68	France	1995	Rye	2
68	France	1995	Soyabean Oil	4
68	France	1995	Sugar, Refined Equivalent	88
68	France	1995	Sunflowerseed Oil	20
68	France	1995	Sweeteners, Other	6
68	France	1995	Tea	1
68	France	1995	Tomatoes	65
68	France	1995	Vegetables, Other	214
68	France	1995	Wheat	257
68	France	1995	Wine	161
68	France	1996	Apples	23
68	France	1996	Bananas	19
68	France	1996	Barley	1
68	France	1996	Beans	2
68	France	1996	Beer	91
68	France	1996	Beverages, Alcoholic	7

FAO countrycode	Country	Reference year	Food item	Gross food supply (g *capita*$^{-1}$ d^{-1})
68	France	1996	Beverages, Fermented	34
68	France	1996	Bovine Meat	76
68	France	1996	Butter, Ghee	24
68	France	1996	Cephalopods	1
68	France	1996	Cheese	60
68	France	1996	Cocoa Beans	9
68	France	1996	Coconuts - Incl Copra	1
68	France	1996	Coffee	15
68	France	1996	Cream	12
68	France	1996	Crustaceans	9
68	France	1996	Demersal Fish	30
68	France	1996	Eggs	43
68	France	1996	Fats, Animals, Raw	14
68	France	1996	Fish, Body Oil	1
68	France	1996	Freshwater Fish	9
68	France	1996	Fruits, Other	83
68	France	1996	Grapefruit	13
68	France	1996	Grapes	10
68	France	1996	Groundnut Oil	3
68	France	1996	Groundnuts (Shelled Eq)	2
68	France	1996	Honey	1
68	France	1996	Lemons, Limes	6
68	France	1996	Maize	35
68	France	1996	Maize Germ Oil	1
68	France	1996	Marine Fish, Other	2
68	France	1996	Meat, Other	17
68	France	1996	Milk, Whole	178
68	France	1996	Molluscs, Other	19
68	France	1996	Mutton & Goat Meat	12
68	France	1996	Nuts	10
68	France	1996	Oats	1
68	France	1996	Offals, Edible	25
68	France	1996	Oilcrops Oil, Other	6
68	France	1996	Olive Oil	2
68	France	1996	Olives	2
68	France	1996	Onions	13
68	France	1996	Oranges, Mandarines	83
68	France	1996	Palm Oil	1
68	France	1996	Peas	2
68	France	1996	Pelagic Fish	16
68	France	1996	Pigmeat	97
68	France	1996	Pineapples	4
68	France	1996	Potatoes	194
68	France	1996	Poultry Meat	70
68	France	1996	Pulses, Other	1
68	France	1996	Rape and Mustard Oil	7
68	France	1996	Rice (Milled Equivalent)	9
68	France	1996	Rye	2
68	France	1996	Soyabean Oil	3
68	France	1996	Sugar, Refined Equivalent	90
68	France	1996	Sunflowerseed Oil	19
68	France	1996	Sweeteners, Other	7
68	France	1996	Tea	1
68	France	1996	Tomatoes	64
68	France	1996	Vegetables, Other	212
68	France	1996	Wheat	258
68	France	1996	Wine	165
68	France	1997	Apples	26
68	France	1997	Bananas	3
68	France	1997	Barley	1
68	France	1997	Beans	3
68	France	1997	Beer	82
68	France	1997	Beverages, Alcoholic	7
68	France	1997	Beverages, Fermented	31
68	France	1997	Bovine Meat	76
68	France	1997	Butter, Ghee	25
68	France	1997	Cephalopods	1
68	France	1997	Cheese	61
68	France	1997	Cocoa Beans	9
68	France	1997	Coconuts - Incl Copra	1
68	France	1997	Coffee	16
68	France	1997	Cream	14
68	France	1997	Crustaceans	8
68	France	1997	Demersal Fish	30
68	France	1997	Eggs	43
68	France	1997	Fats, Animals, Raw	14
68	France	1997	Fish, Body Oil	1
68	France	1997	Freshwater Fish	9
68	France	1997	Fruits, Other	75
68	France	1997	Grapefruit	15
68	France	1997	Grapes	8
68	France	1997	Groundnut Oil	3
68	France	1997	Groundnuts (Shelled Eq)	3
68	France	1997	Honey	1
68	France	1997	Lemons, Limes	6
68	France	1997	Maize	35
68	France	1997	Maize Germ Oil	1
68	France	1997	Marine Fish, Other	2
68	France	1997	Meat, Other	16
68	France	1997	Milk, Whole	178
68	France	1997	Molluscs, Other	20
68	France	1997	Mutton & Goat Meat	12
68	France	1997	Nuts	10
68	France	1997	Oats	1
68	France	1997	Offals, Edible	26
68	France	1997	Oilcrops Oil, Other	7
68	France	1997	Olive Oil	3

FAO country code	Country	Reference year	Food item	Gross food supply (g $capita^{-1}$ d^{-1})
68	France	1997	Olives	3
68	France	1997	Onions	14
68	France	1997	Oranges, Mandarines	91
68	France	1997	Palm Oil	1
68	France	1997	Peas	2
68	France	1997	Pelagic Fish	14
68	France	1997	Pigmeat	98
68	France	1997	Pineapples	4
68	France	1997	Potatoes	192
68	France	1997	Poultry Meat	72
68	France	1997	Pulses, Other	1
68	France	1997	Rape and Mustard Oil	6
68	France	1997	Rice (Milled Equivalent)	12
68	France	1997	Rye	1
68	France	1997	Soyabean Oil	4
68	France	1997	Sugar, Refined Equivalent	93
68	France	1997	Sunflowerseed Oil	20
68	France	1997	Sweeteners, Other	5
68	France	1997	Tea	1
68	France	1997	Tomatoes	65
68	France	1997	Vegetables, Other	204
68	France	1997	Wheat	258
68	France	1997	Wine	158
68	France	1998	Apples	27
68	France	1998	Bananas	2
68	France	1998	Barley	1
68	France	1998	Beans	3
68	France	1998	Beer	89
68	France	1998	Beverages, Alcoholic	7
68	France	1998	Beverages, Fermented	28
68	France	1998	Bovine Meat	76
68	France	1998	Butter, Ghee	25
68	France	1998	Cephalopods	1
68	France	1998	Cheese	63
68	France	1998	Cocoa Beans	9
68	France	1998	Coconuts - Incl Copra	2
68	France	1998	Coffee	15
68	France	1998	Cream	14
68	France	1998	Crustaceans	9
68	France	1998	Dates	1
68	France	1998	Demersal Fish	30
68	France	1998	Eggs	43
68	France	1998	Fats, Animals, Raw	13
68	France	1998	Fish, Body Oil	1
68	France	1998	Freshwater Fish	9
68	France	1998	Fruits, Other	73
68	France	1998	Grapefruit	15
68	France	1998	Grapes	5
68	France	1998	Groundnut Oil	2
68	France	1998	Groundnuts (Shelled Eq)	2
68	France	1998	Honey	1
68	France	1998	Lemons, Limes	6
68	France	1998	Maize	35
68	France	1998	Maize Germ Oil	1
68	France	1998	Marine Fish, Other	1
68	France	1998	Meat, Other	16
68	France	1998	Milk, Whole	171
68	France	1998	Molluscs, Other	19
68	France	1998	Mutton & Goat Meat	12
68	France	1998	Nuts	11
68	France	1998	Oats	1
68	France	1998	Offals, Edible	27
68	France	1998	Oilcrops Oil, Other	6
68	France	1998	Olive Oil	4
68	France	1998	Olives	3
68	France	1998	Onions	14
68	France	1998	Oranges, Mandarines	100
68	France	1998	Palm Oil	1
68	France	1998	Peas	2
68	France	1998	Pelagic Fish	18
68	France	1998	Pigmeat	102
68	France	1998	Pineapples	4
68	France	1998	Potatoes	186
68	France	1998	Poultry Meat	73
68	France	1998	Pulses, Other	1
68	France	1998	Rape and Mustard Oil	8
68	France	1998	Rice (Milled Equivalent)	13
68	France	1998	Rye	1
68	France	1998	Soyabean Oil	3
68	France	1998	Sugar, Refined Equivalent	92
68	France	1998	Sunflowerseed Oil	20
68	France	1998	Sweeteners, Other	10
68	France	1998	Tea	1
68	France	1998	Tomatoes	68
68	France	1998	Vegetables, Other	215
68	France	1998	Wheat	262
68	France	1998	Wine	160
68	France	1999	Apples	26
68	France	1999	Bananas	3
68	France	1999	Barley	1
68	France	1999	Beans	3
68	France	1999	Beer	89
68	France	1999	Beverages, Alcoholic	7
68	France	1999	Beverages, Fermented	28
68	France	1999	Bovine Meat	72
68	France	1999	Butter, Ghee	24
68	France	1999	Cephalopods	1

FAO countrycode	Country	Reference year	Food item	Gross food supply(g *capita*$^{-1}$ d^{-1})
68	France	1999	Cheese	64
68	France	1999	Cocoa Beans	10
68	France	1999	Coconuts - Incl Copra	2
68	France	1999	Coffee	15
68	France	1999	Cream	15
68	France	1999	Crustaceans	9
68	France	1999	Demersal Fish	30
68	France	1999	Eggs	44
68	France	1999	Fats, Animals, Raw	12
68	France	1999	Fish, Body Oil	1
68	France	1999	Freshwater Fish	10
68	France	1999	Fruits, Other	81
68	France	1999	Grapefruit	17
68	France	1999	Grapes	6
68	France	1999	Groundnut Oil	3
68	France	1999	Groundnuts (Shelled Eq)	3
68	France	1999	Honey	1
68	France	1999	Lemons, Limes	6
68	France	1999	Maize	35
68	France	1999	Maize Germ Oil	1
68	France	1999	Marine Fish, Other	1
68	France	1999	Meat, Other	16
68	France	1999	Milk, Whole	170
68	France	1999	Molluscs, Other	20
68	France	1999	Mutton & Goat Meat	12
68	France	1999	Nuts	11
68	France	1999	Oats	1
68	France	1999	Offals, Edible	27
68	France	1999	Oilcrops Oil, Other	5
68	France	1999	Olive Oil	4
68	France	1999	Olives	3
68	France	1999	Onions	14
68	France	1999	Oranges, Mandarines	103
68	France	1999	Palm Oil	2
68	France	1999	Palmkernel Oil	1
68	France	1999	Peas	2
68	France	1999	Pelagic Fish	16
68	France	1999	Pigmeat	102
68	France	1999	Pineapples	4
68	France	1999	Potatoes	187
68	France	1999	Poultry Meat	69
68	France	1999	Pulses, Other	1
68	France	1999	Rape and Mustard Oil	8
68	France	1999	Rice (Milled Equivalent)	13
68	France	1999	Rye	1
68	France	1999	Soyabean Oil	3
68	France	1999	Sugar, Refined Equivalent	92
68	France	1999	Sunflowerseed Oil	20
68	France	1999	Sweeteners, Other	11
68	France	1999	Tea	1
68	France	1999	Tomatoes	72
68	France	1999	Vegetables, Other	213
68	France	1999	Wheat	263
68	France	1999	Wine	153
68	France	2000	Apples	23
68	France	2000	Bananas	4
68	France	2000	Barley	1
68	France	2000	Beans	3
68	France	2000	Beer	79
68	France	2000	Beverages, Alcoholic	7
68	France	2000	Beverages, Fermented	25
68	France	2000	Bovine Meat	71
68	France	2000	Butter, Ghee	24
68	France	2000	Cephalopods	2
68	France	2000	Cheese	65
68	France	2000	Cocoa Beans	11
68	France	2000	Coconuts - Incl Copra	2
68	France	2000	Coffee	15
68	France	2000	Cream	14
68	France	2000	Crustaceans	8
68	France	2000	Demersal Fish	30
68	France	2000	Eggs	44
68	France	2000	Fats, Animals, Raw	12
68	France	2000	Fish, Body Oil	1
68	France	2000	Freshwater Fish	9
68	France	2000	Fruits, Other	85
68	France	2000	Grapefruit	16
68	France	2000	Grapes	17
68	France	2000	Groundnut Oil	3
68	France	2000	Groundnuts (Shelled Eq)	3
68	France	2000	Honey	1
68	France	2000	Lemons, Limes	6
68	France	2000	Maize	37
68	France	2000	Maize Germ Oil	1
68	France	2000	Marine Fish, Other	1
68	France	2000	Meat, Other	16
68	France	2000	Milk, Whole	168
68	France	2000	Molluscs, Other	19
68	France	2000	Mutton & Goat Meat	12
68	France	2000	Nuts	11
68	France	2000	Oats	1
68	France	2000	Offals, Edible	25
68	France	2000	Oilcrops Oil, Other	6
68	France	2000	Olive Oil	4
68	France	2000	Olives	3
68	France	2000	Onions	19
68	France	2000	Oranges, Mandarines	112

FAO countrycode	Country	Reference year	Food item	Gross food supply (g *capita*$^{-1}$ d^{-1})
68	France	2000	Palm Oil	1
68	France	2000	Palmkernel Oil	1
68	France	2000	Peas	2
68	France	2000	Pelagic Fish	16
68	France	2000	Pigmeat	103
68	France	2000	Pineapples	4
68	France	2000	Potatoes	185
68	France	2000	Poultry Meat	73
68	France	2000	Pulses, Other	2
68	France	2000	Rape and Mustard Oil	9
68	France	2000	Rice (Milled Equivalent)	13
68	France	2000	Rye	1
68	France	2000	Soyabean Oil	3
68	France	2000	Sugar, Refined Equivalent	90
68	France	2000	Sunflowerseed Oil	20
68	France	2000	Sweeteners, Other	9
68	France	2000	Tea	1
68	France	2000	Tomatoes	70
68	France	2000	Vegetables, Other	210
68	France	2000	Wheat	263
68	France	2000	Wine	140
68	France	2001	Apples	39
68	France	2001	Bananas	7
68	France	2001	Barley	1
68	France	2001	Beans	3
68	France	2001	Beer	82
68	France	2001	Beverages, Alcoholic	7
68	France	2001	Beverages, Fermented	25
68	France	2001	Bovine Meat	75
68	France	2001	Butter, Ghee	23
68	France	2001	Cephalopods	1
68	France	2001	Cheese	67
68	France	2001	Cocoa Beans	10
68	France	2001	Coconuts - Incl Copra	2
68	France	2001	Coffee	15
68	France	2001	Cream	15
68	France	2001	Crustaceans	9
68	France	2001	Demersal Fish	32
68	France	2001	Eggs	43
68	France	2001	Fats, Animals, Raw	11
68	France	2001	Fish, Body Oil	1
68	France	2001	Freshwater Fish	10
68	France	2001	Fruits, Other	86
68	France	2001	Grapefruit	13
68	France	2001	Grapes	10
68	France	2001	Groundnut Oil	2
68	France	2001	Groundnuts (Shelled Eq)	2
68	France	2001	Honey	1
68	France	2001	Lemons, Limes	5
68	France	2001	Maize	35
68	France	2001	Maize Germ Oil	1
68	France	2001	Marine Fish, Other	1
68	France	2001	Meat, Other	16
68	France	2001	Milk, Whole	177
68	France	2001	Molluscs, Other	20
68	France	2001	Mutton & Goat Meat	9
68	France	2001	Nuts	11
68	France	2001	Oats	1
68	France	2001	Offals, Edible	25
68	France	2001	Oilcrops Oil, Other	7
68	France	2001	Olive Oil	4
68	France	2001	Olives	3
68	France	2001	Onions	18
68	France	2001	Oranges, Mandarines	98
68	France	2001	Palm Oil	1
68	France	2001	Peas	1
68	France	2001	Pelagic Fish	19
68	France	2001	Pigmeat	102
68	France	2001	Pineapples	5
68	France	2001	Potatoes	183
68	France	2001	Poultry Meat	76
68	France	2001	Pulses, Other	1
68	France	2001	Rape and Mustard Oil	8
68	France	2001	Rice (Milled Equivalent)	14
68	France	2001	Rye	1
68	France	2001	Soyabean Oil	5
68	France	2001	Sugar, Refined Equivalent	91
68	France	2001	Sunflowerseed Oil	20
68	France	2001	Sweeteners, Other	11
68	France	2001	Tea	1
68	France	2001	Tomatoes	70
68	France	2001	Vegetables, Other	207
68	France	2001	Wheat	269
68	France	2001	Wine	139
68	France	2002	Apples	37
68	France	2002	Bananas	6
68	France	2002	Barley	1
68	France	2002	Beans	3
68	France	2002	Beer	80
68	France	2002	Beverages, Alcoholic	7
68	France	2002	Beverages, Fermented	25
68	France	2002	Bovine Meat	77
68	France	2002	Butter, Ghee	23
68	France	2002	Cephalopods	1
68	France	2002	Cereals, Other	1
68	France	2002	Cheese	67
68	France	2002	Cocoa Beans	10

FAO countrycode	Country	Reference year	Food item	Gross food supply (g *capita*$^{-1}$ d^{-1})
68	France	2002	Coconuts - Incl Copra	2
68	France	2002	Coffee	15
68	France	2002	Cream	15
68	France	2002	Crustaceans	10
68	France	2002	Dates	1
68	France	2002	Demersal Fish	29
68	France	2002	Eggs	42
68	France	2002	Fats, Animals, Raw	12
68	France	2002	Fish, Body Oil	1
68	France	2002	Freshwater Fish	9
68	France	2002	Fruits, Other	86
68	France	2002	Grapefruit	11
68	France	2002	Grapes	14
68	France	2002	Groundnut Oil	2
68	France	2002	Groundnuts (Shelled Eq)	3
68	France	2002	Honey	1
68	France	2002	Lemons, Limes	7
68	France	2002	Maize	35
68	France	2002	Maize Germ Oil	2
68	France	2002	Marine Fish, Other	1
68	France	2002	Meat, Other	14
68	France	2002	Milk, Whole	183
68	France	2002	Molluscs, Other	19
68	France	2002	Mutton & Goat Meat	9
68	France	2002	Nuts	11
68	France	2002	Oats	1
68	France	2002	Offals, Edible	25
68	France	2002	Oilcrops Oil, Other	8
68	France	2002	Olive Oil	4
68	France	2002	Olives	3
68	France	2002	Onions	20
68	France	2002	Oranges, Mandarines	110
68	France	2002	Palm Oil	1
68	France	2002	Peas	1
68	France	2002	Pelagic Fish	24
68	France	2002	Pigmeat	98
68	France	2002	Pineapples	5
68	France	2002	Potatoes	181
68	France	2002	Poultry Meat	70
68	France	2002	Pulses, Other	1
68	France	2002	Rape and Mustard Oil	9
68	France	2002	Rice (Milled Equivalent)	14
68	France	2002	Rye	1
68	France	2002	Soyabean Oil	4
68	France	2002	Sugar, Refined Equivalent	93
68	France	2002	Sunflowerseed Oil	20
68	France	2002	Sweeteners, Other	11
68	France	2002	Tea	1
68	France	2002	Tomatoes	65
68	France	2002	Vegetables, Other	214
68	France	2002	Wheat	270
68	France	2002	Wine	138
68	France	2003	Apples	30
68	France	2003	Bananas	7
68	France	2003	Barley	1
68	France	2003	Beans	3
68	France	2003	Beer	82
68	France	2003	Beverages, Alcoholic	7
68	France	2003	Beverages, Fermented	22
68	France	2003	Bovine Meat	72
68	France	2003	Butter, Ghee	22
68	France	2003	Cephalopods	1
68	France	2003	Cereals, Other	1
68	France	2003	Cheese	66
68	France	2003	Cocoa Beans	11
68	France	2003	Coconuts - Incl Copra	2
68	France	2003	Coffee	14
68	France	2003	Cream	12
68	France	2003	Crustaceans	10
68	France	2003	Dates	1
68	France	2003	Demersal Fish	30
68	France	2003	Eggs	42
68	France	2003	Fats, Animals, Raw	12
68	France	2003	Fish, Body Oil	1
68	France	2003	Freshwater Fish	10
68	France	2003	Fruits, Other	80
68	France	2003	Grapefruit	15
68	France	2003	Grapes	7
68	France	2003	Groundnut Oil	3
68	France	2003	Groundnuts (Shelled Eq)	2
68	France	2003	Honey	1
68	France	2003	Lemons, Limes	5
68	France	2003	Maize	35
68	France	2003	Maize Germ Oil	1
68	France	2003	Marine Fish, Other	1
68	France	2003	Meat, Other	12
68	France	2003	Milk, Whole	181
68	France	2003	Molluscs, Other	20
68	France	2003	Mutton & Goat Meat	9
68	France	2003	Nuts	10
68	France	2003	Oats	1
68	France	2003	Offals, Edible	24
68	France	2003	Oilcrops Oil, Other	7
68	France	2003	Olive Oil	4
68	France	2003	Olives	3
68	France	2003	Onions	16
68	France	2003	Oranges, Mandarines	112

FAO country code	Country	Reference year	Food item	Gross food supply (g $capita^{-1}$ d^{-1})
68	France	2003	Palm Oil	1
68	France	2003	Peas	1
68	France	2003	Pelagic Fish	20
68	France	2003	Pigmeat	103
68	France	2003	Pineapples	5
68	France	2003	Potatoes	176
68	France	2003	Poultry Meat	67
68	France	2003	Pulses, Other	2
68	France	2003	Rape and Mustard Oil	9
68	France	2003	Rice (Milled Equivalent)	14
68	France	2003	Rye	1
68	France	2003	Soyabean Oil	5
68	France	2003	Sugar, Refined Equivalent	92
68	France	2003	Sunflowerseed Oil	18
68	France	2003	Sweeteners, Other	10
68	France	2003	Tea	1
68	France	2003	Tomatoes	70
68	France	2003	Vegetables, Other	206
68	France	2003	Wheat	266
68	France	2003	Wine	138
68	France	2004	Apples	38
68	France	2004	Bananas	8
68	France	2004	Barley	1
68	France	2004	Beans	2
68	France	2004	Beer	76
68	France	2004	Beverages, Alcoholic	7
68	France	2004	Beverages, Fermented	24
68	France	2004	Bovine Meat	72
68	France	2004	Butter, Ghee	22
68	France	2004	Cephalopods	1
68	France	2004	Cheese	65
68	France	2004	Cocoa Beans	11
68	France	2004	Coconuts - Incl Copra	2
68	France	2004	Coffee	13
68	France	2004	Cream	12
68	France	2004	Crustaceans	10
68	France	2004	Dates	1
68	France	2004	Demersal Fish	31
68	France	2004	Eggs	43
68	France	2004	Fats, Animals, Raw	11
68	France	2004	Fish, Body Oil	1
68	France	2004	Freshwater Fish	10
68	France	2004	Fruits, Other	90
68	France	2004	Grapefruit	16
68	France	2004	Grapes	6
68	France	2004	Groundnut Oil	1
68	France	2004	Groundnuts (Shelled Eq)	2
68	France	2004	Honey	1
68	France	2004	Lemons, Limes	5
68	France	2004	Maize	36
68	France	2004	Maize Germ Oil	1
68	France	2004	Meat, Other	10
68	France	2004	Milk, Whole	165
68	France	2004	Molluscs, Other	21
68	France	2004	Mutton & Goat Meat	9
68	France	2004	Nuts	10
68	France	2004	Oats	1
68	France	2004	Offals, Edible	22
68	France	2004	Oilcrops Oil, Other	7
68	France	2004	Olive Oil	4
68	France	2004	Olives	3
68	France	2004	Onions	19
68	France	2004	Oranges, Mandarines	122
68	France	2004	Palm Oil	1
68	France	2004	Palmkernel Oil	1
68	France	2004	Peas	1
68	France	2004	Pelagic Fish	16
68	France	2004	Pigmeat	92
68	France	2004	Pineapples	7
68	France	2004	Plantains	1
68	France	2004	Potatoes	182
68	France	2004	Poultry Meat	66
68	France	2004	Pulses, Other	1
68	France	2004	Rape and Mustard Oil	8
68	France	2004	Rice (Milled Equivalent)	13
68	France	2004	Rye	1
68	France	2004	Soyabean Oil	4
68	France	2004	Sugar, Refined Equivalent	96
68	France	2004	Sunflowerseed Oil	19
68	France	2004	Sweeteners, Other	7
68	France	2004	Tea	1
68	France	2004	Tomatoes	73
68	France	2004	Vegetables, Other	214
68	France	2004	Wheat	272
68	France	2004	Wine	146
68	France	2005	Apples	44
68	France	2005	Bananas	9
68	France	2005	Beans	2
68	France	2005	Beer	74
68	France	2005	Beverages, Alcoholic	6
68	France	2005	Beverages, Fermented	24
68	France	2005	Bovine Meat	73
68	France	2005	Butter, Ghee	22
68	France	2005	Cephalopods	1
68	France	2005	Cheese	65
68	France	2005	Cocoa Beans	11
68	France	2005	Coconuts - Incl Copra	2

FAO country code	Country	Reference year	Food item	Gross food supply (g $capita^{-1}$ d^{-1})
68	France	2005	Coffee	13
68	France	2005	Cream	13
68	France	2005	Crustaceans	10
68	France	2005	Dates	1
68	France	2005	Demersal Fish	28
68	France	2005	Eggs	39
68	France	2005	Fats, Animals, Raw	9
68	France	2005	Fish, Body Oil	1
68	France	2005	Freshwater Fish	10
68	France	2005	Fruits, Other	95
68	France	2005	Grapefruit	16
68	France	2005	Grapes	7
68	France	2005	Groundnut Oil	2
68	France	2005	Groundnuts (Shelled Eq)	2
68	France	2005	Honey	1
68	France	2005	Lemons, Limes	5
68	France	2005	Maize	36
68	France	2005	Maize Germ Oil	1
68	France	2005	Meat, Other	9
68	France	2005	Milk, Whole	144
68	France	2005	Molluscs, Other	22
68	France	2005	Mutton & Goat Meat	9
68	France	2005	Nuts	9
68	France	2005	Oats	1
68	France	2005	Offals, Edible	21
68	France	2005	Oilcrops Oil, Other	7
68	France	2005	Olive Oil	4
68	France	2005	Olives	3
68	France	2005	Onions	15
68	France	2005	Oranges, Mandarines	124
68	France	2005	Palm Oil	1
68	France	2005	Palmkernel Oil	3
68	France	2005	Peas	1
68	France	2005	Pelagic Fish	21
68	France	2005	Pigmeat	94
68	France	2005	Pineapples	6
68	France	2005	Potatoes	144
68	France	2005	Poultry Meat	57
68	France	2005	Pulses, Other	2
68	France	2005	Rape and Mustard Oil	8
68	France	2005	Rice (Milled Equivalent)	14
68	France	2005	Rye	1
68	France	2005	Soyabean Oil	6
68	France	2005	Sugar, Refined Equivalent	97
68	France	2005	Sunflowerseed Oil	20
68	France	2005	Sweeteners, Other	7
68	France	2005	Tea	1
68	France	2005	Tomatoes	68
68	France	2005	Vegetables, Other	202
68	France	2005	Wheat	274
68	France	2005	Wine	147
68	France	2006	Apples	34
68	France	2006	Bananas	10
68	France	2006	Beans	2
68	France	2006	Beer	77
68	France	2006	Beverages, Alcoholic	6
68	France	2006	Beverages, Fermented	24
68	France	2006	Bovine Meat	71
68	France	2006	Butter, Ghee	22
68	France	2006	Cephalopods	1
68	France	2006	Cheese	66
[truncated: 566,093 more chars]
